# Tropane and related alkaloid skeletons via a radical [3+3]-annulation process

**DOI:** 10.1038/s42004-022-00671-x

**Published:** 2022-04-28

**Authors:** Eloïse Colson, Julie Andrez, Ali Dabbous, Fabrice Dénès, Vincent Maurel, Jean-Marie Mouesca, Philippe Renaud

**Affiliations:** 1 Department of Chemistry, Biochemistry and Pharmaceutical Sciences (DCBP), University of Bern, Freiestrasse 3, CH-3012 Bern, Switzerland; 2 Univ. Grenoble Alpes, CEA, CNRS, IRIG, SyMMES, F-38000 Grenoble, France

**Keywords:** Synthetic chemistry methodology, Reaction mechanisms, Photocatalysis

## Abstract

Tropanes and related bicyclic alkaloids are highly attractive compounds possessing a broad biological activity. Here we report a mild and simple protocol for the synthesis of *N*-arylated 8-azabicyclo[3.2.1]octane and 9-azabicyclo[3.3.1]nonane derivatives. It provides these valuable bicyclic alkaloid skeletons in good yields and high levels of diastereoselectivity from simple and readily available starting materials using visible-light photoredox catalysis. These bicyclic aniline derivatives are hardly accessible via the classical Robinson tropane synthesis and represent a particularly attractive scaffold for medicinal chemistry. This unprecedented annulation process takes advantage of the unique reactivity of ethyl 2-(acetoxymethyl)acrylate as a 1,3-bis radical acceptor and of cyclic *N,N*-dialkylanilines as radical 1,3-bis radical donors. The success of this process relies on efficient electron transfer processes and highly selective deprotonation of aminium radical cations leading to the key α-amino radical intermediates.

## Abstract (web summary)

*N*-arylated bicyclic alkaloid skeletons are attractive scaffolds for medicinal chemistry, but aren’t conveniently accessible via the classical Robinson tropane synthesis. Here, a mild and simple visible-light catalyzed radical [3 + 3]-annulation process is designed, affording *N*-arylated 8-azabicyclo[3.2.1]octane and 9-azabicyclo[3.3.1]nonane derivatives with good yields and high diastereoselectivity from readily available starting materials.

## Introduction

**Fig. 1:**
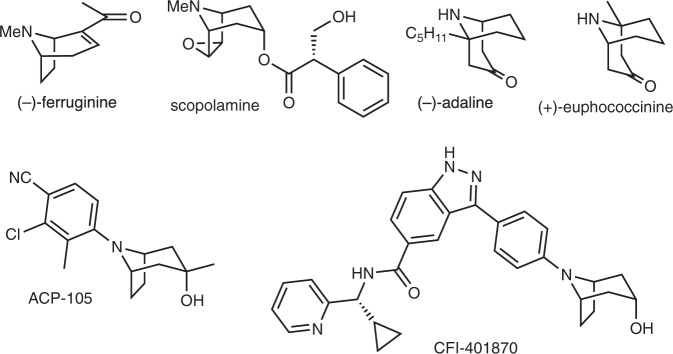
Tropanes and homotropanes. Selected examples of tropane and homotropane alkaloids as well as *N*-arylated tropane derivatives of biological interest.

Nitrogen-containing moieties are omnipresent in natural products, biologically active compounds and agricultural chemicals. In particular, the 8-azabicyclo[3.2.1]octane and 9-azabicyclo[3.3.1]nonane skeletons constitute the core of many natural tropane^1^ and homotropane alkaloids^2^, respectively, and analogues presenting a wide range of biological activities. For instance, tropinone, cocaine and scopolamine (Fig. 1) are amongst the most popular representative examples of natural alkaloids presenting a 8-azabicyclo[3.2.1]octane (tropane) skeleton as their base structure. Unlike its natural enantiomer, unnatural (−)-ferruginine (Fig. 1), is a good agonist for nicotinic acetylcholine receptor. The 9-azabicyclo[3.3.1]nonane (homotropane) skeleton can be found in the structure of (–)-adaline, and (+)-euphococcinine (Fig. 1)^2^, two important defensive alkaloids from, respectively, the European ladybug *Adalia bipunctata* and the Australian ladybug *Cryptolaemis montrouzieri*. Moreover, *N*-arylated tropanes and related bicyclic aniline derivatives^3^ represent a particularly attractive scaffold for medicinal chemistry as illustrated by ACP 105^4,5^, an orally available and potent selective androgen receptor modulator, and CFI-401870^6^, a single-digit nanomolar tyrosine threonine kinase (TTK) inhibitor (Fig. 1).

**Fig. 2:**
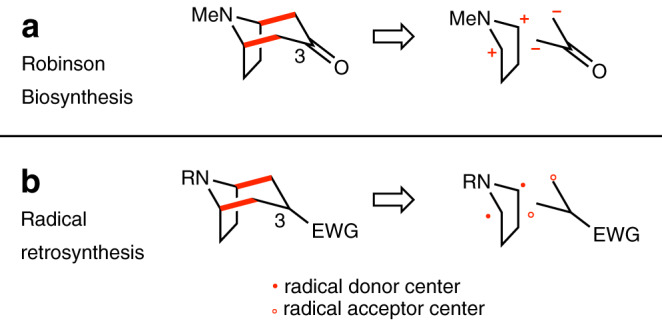
Retrosynthetic analysis. Retrosynthetic analysis of tropane alkaloids according to their biosynthesis and to Robinson synthesis (**a**) as a source of inspiration for a radical retrosynthetic analysis (**b**). New C–C bonds are indicated in red, radical donor as red dot and radical acceptor as red circle.

Since the report by Robinson of the multicomponent synthesis of tropinone based on a cascade Mannich reaction^7,8^, access to tropanes and related alkaloids has been the object of intense activity^9^. Strategies based on ionic reactions, cycloadditions and transition metal mediated processes have been reported. The preparation of *N*-arylated tropane derivatives via Robinson synthesis is a knotty process^10^ with limited scope^11–13^ and they are mostly prepared via *N*-demethylation followed by a dubious *N*-arylation step^14^. Despite their attractiveness to build cycles, radical processes have only been scarcely used in tropane synthesis^15^. The development of a general and flexible access to tropane related heterocycles using a mild radical-based approach is expected to complement nicely the existing methods by allowing a direct preparation of *N*-arylated derivatives and by modifying the substitution pattern at position 3 of the tropane skeleton. To gain in efficiency, a cascade processes involving consecutive formation of C–C bonds is particularly sought. The biosynthesis of tropane alkaloids^16^ as well as Robinson biomimetic synthesis^7,8^ are both based on an ionic [3 + 3]-annulation processes involving 1,3-bis-iminium ions and 1,3-bis-enolates synthons (Fig. 2a). Inspired by this observation, we hypothesized that a radical version might be possible by using 1,3-bis-α-amino radical donor and 1,3-bis radical acceptor (Fig. 2b), radical donor and acceptor being defined and represented according to Curran’s proposal in his seminal article on retrosynthetic planning of radical reactions^17^. To the best of our knowledge, this type of radical [3 + 3]-annulation has not yet been reported^18–24^.

**Fig. 3:**
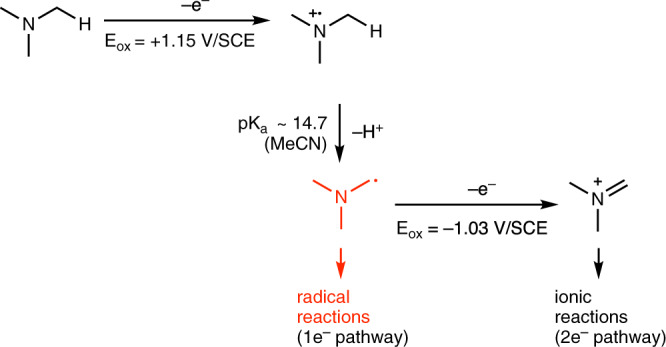
Oxidation of trimethylamine. One- vs. two-electron pathways. Desired radical process indicated in red.

The α-functionalization of tertiary amines has attracted much interest in the past and methods involving one- and two-electron oxidation pathway have been reported (Fig. 3). One-electron oxidation of the amine affords a nitrogen-centered radical cation. The oxidation leads to a massive increase of the acidity of the α-C–H bond relative to the starting amine^25^. Rapid deprotonation of the radical cation gives the α-aminoalkyl radical that can be used for a variety of radical processes, such as for instance addition to alkenes leading to C–C bond formation (one-electron pathway)^26^. On the other hand, the α-aminoalkyl radical is more easily oxidized than the starting amine and therefore further oxidation to the corresponding iminium ion can take place (two-electron pathway)^27^. The iminium intermediate can be used for a variety of useful ionic transformations^28,29^. As a consequence, the use of stoichiometric oxidants to perform radical reaction via the one-electron pathway is very challenging due to rapid overoxidation of the intermediate α-aminoalkyl radical to the corresponding iminium ion^30^.

**Fig. 4:**
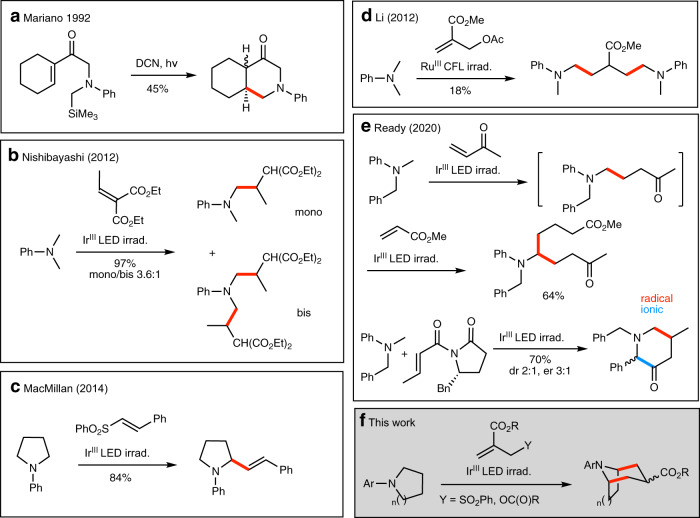
Photoredox catalyzed α-functionalization of tertiary amines. Selected leading contributions in the field (**a**–**e**) and proposed annulation strategy (**f**). New C–C bonds are indicated in red (radical process) and in blue (ionic process).

In this context, the single electron oxidation of amines *via* photoinduced electron transfer has been shown to be a suitable method to suppress this overoxidation. Mariano and co-workers reported in 1992 the photosensitized generation of α-aminoalkyl radicals from easily oxidized α-silyl amines and their subsequent intra- and intermolecular additions to electron-poor olefins. Interestingly, they reported a 6-*endo*-trig cyclization process (Fig. 4a)^31^. Pandey took also advantage of the oxidation of a bis-α-silylamine to generate an azomethine ylide that can be used in 1,3-dipolar cycloaddition^32^. Pandey, Reiser et al.^33^ and Nishibayashi^26,34^ independently extended this chemistry to photoredox-mediated protocols not only on α-silylated amines but also respectively on *N*-aryl-tetrahydroisoquinolines^33^ and on cyclic and acyclic amines. Nishibayashi et al. reported the formation of bis-alkylated product starting from symmetrical starting materials, demonstrating that the intermediate mono-alkylated product has a similar reactivity as the starting amine under specific reaction conditions (Fig. 4b)^34^. In 2014, MacMillan et al. reported the α-vinylation of aniline derivatives and *N*-Boc-protected amino acids using vinyl sulfones as coupling partners (Fig. 4c)^35^. In this case, no bis-vinylation was reported presumably due to the excess of the starting amine used under their conditions. The same year, Li and co-workers reported the first α-allylation of *N*-aryl tertiary amines using an allylic radical trap^36^. Double addition to the radical trap was observed in one case albeit in very moderate yield (Fig. 4d). In 2020, Ready et al. reported a method for the regioselective α-functionalization of tertiary amines. Interestingly, the initially formed α-alkylation product could be further alkylated in a one-pot sequence (Fig. 4e)^37^. The second alkylation takes place in a regioselective manner at the aliphatic position over the benzylic position. In one example, they observed the formation of a remarkable [3 + 3] adduct (Fig. 4e). In this reaction, the initial radical adduct is presumably abstracting a hydrogen from the benzylic position. Reduction of the benzylic radical followed by nucleophilic displacement of the Evans auxiliary account for product formation. All these results incited us to examine annulation reactions based on the double activation of tertiary amines. We described here a tandem process for the synthesis of tropane and homotropane alkaloid skeletons (Fig. 4f). The reaction is based on the activation of cyclic tertiary *N*-arylamines to generate 1,3-bis radical donors that react with ester activated allylic radical traps acting as 1,3-bis radical acceptors. This formal [3 + 3] annulation reaction relies on the regioselective radical formation at the α and α′ positions of the amine, on a rapid 6-*endo*-cyclization that outcompetes intermolecular processes, and on the fact that the final azabicycles are not undergoing further oxidation.

## Results and discussion

### Proof of concept and optimization

To prove the validity of the annulation approach, *N*-phenylpyrrolidine **1a** was reacted in 1,2-dichloroethane (DCE) with a series of allyl radical traps possessing different leaving groups Y in the presence of different catalysts and base additives. The results are collected in Table 1 (more entries are available in the supporting information). Three side products were identified during this optimization phase: the mono-allylated product **2a** (an intermediate in the reaction leading to **3a**), the α,α′-bis-allylated product **4a** and the bis-addition product **5a**. Since all these side products are difficult to separate from the desired tropane **3a**, conditions were sought to minimize their formation. We started the optimization by using ethyl 2-((phenylsulfonyl)methyl)acrylate (Y = SO_2_Ph, 1.2 equivalents) as a trap under conditions similar to the one reported in the literature for reactions with vinyl sulfones (see Scheme 3 C)^35^, i.e., by using [Ir{dF(CF_3_)ppy}_2_(dtbpy)]PF_6_ (Ir-A) as a catalyst, CsOAc (3 equivalents) as a base and irradiating with 390 nm LEDs in 1,2-DCE (Table 1, entry 1). Gratifyingly, the reaction afforded the desired tropane **3a** in 45% yield (all yields determined by ^1^H-NMR analysis using an internal standard). Decreasing the base loading to 1.2 equivalents (Table 1, entry 2) and slightly increasing the amount of the allylsulfone to 1.4 equivalents proved to be beneficial and the product was formed in 65% yield (Table 1, entry 3). However, under these conditions, the product was contaminated with **4a** and **5a** leading to difficult isolation (isolated yield of 43%). The readily available catalyst [Ir(dtbbpy)(ppy)_2_]PF_6_ (Ir-B) proved to be a suitable catalyst for the reaction, yielding the cyclized product in 47% yield (Table 1, entry 4). A screening of bases revealed that CsOAc was the optimal base. The use of NaOAc and Cs_2_CO_3_ gave low yields of mono-allylated pyrrolidine **2a (**Table 1 Entries 5 and 6). The use of [Ir(dFppy)_2_(dtbbpy)]PF_6_ (Ir-C) afforded the cyclic product **3a** in 50% yield (Table 1, entry 7). Other catalysts presenting suitable redox properties were also tested (for redox properties of the tested catalysts, see supporting information). Ru(bpy)_3_(PF_6_)_2_ (Ru-A) and 2,4,5,6-tetrakis(9*H*-carbazol-9-yl) isophthalonitrile (4CzIPN) afforded the bicycle **3a**, together with significant amounts of mono-allylated intermediate **2a** (Table 1, entries 8–9). Eosin Y provide only the mono-allylated product **2a** (51% yield, Table 1, entry 10). The influence of the radical trap was investigated next. Pleasingly, ethyl 2-(acetoxymethyl)acrylate^36^ (Y = OAc) gave the desired product in shorter reaction time (Table 1, entry 11) and similar yield than that with the sulfone trap (Table 1, entry 3). The corresponding sulfide trap (Y = S-*tert*-docedyl) provided also the desired cyclized product in satisfactory yield (Table 1, Entry 12). However, product isolation was more difficult and the release of one equivalent of a smelly thiol makes the reaction less attractive. Finally, the allyl bromide (Y = Br) was tested and only intermediate **2a** was obtained, with no further conversion to the tropane skeleton **3a** (Table 1, Entry 13). Other catalysts (organic photocatalysts), bases (Na_2_CO_3_, K_2_CO_3_) and solvents (DMF, DME, EtOAc, DCM or MeCN) were tested but were all detrimental to the formation of **3a** (see supporting information). Due to shorter reaction time and cleaner product formation, the reaction was further optimized using the allyl acetate trap and catalyst Ir-A. The formation of the bis-allylated product **4a** and the bis-addition product **5a** could be minimized by working under higher dilution (0.05 M) and a much shorter reaction time of 20 min (Table 1, entry 14). Reducing the catalyst loading to 1 mol% proved beneficial to the reaction (Table 1, entry 15) and using a smaller excess of the allyl acetate radical trap (1.1 equivalents) did not affect the reaction yield (Table 1, entry 16). Finally, the optimized conditions were used with the corresponding pivalate (Y = OPiv) and trifluoroacetate (Y = OTFA) radical traps (Table 1, entries 17 and 18) but the yields were lower, particularly with the trifluoroacetate.

**Table 1:** Optimization of the reaction conditions.


Entry	Cat (mol%)	Base (equiv)	Y (equiv)	Time	[1a]	Yield^a^	Side product
1	Ir-A (2)	CsOAc (3.0)	SO_2_Ph (1.2)	16 h	0.1 M	45%^b^	4a, 5a
2	Ir-A (2)	CsOAc (1.2	SO_2_Ph (1.2)	16 h	0.1 M	59%^b^	4a, 5a
3	Ir-A (2)	CsOAc (1.2)	SO_2_Ph (1.4)	16 h	0.1 M	65%^b^ 43%^c,d^	4a, 5a
4	Ir-B (2)	CsOAc (1.2)	SO_2_Ph (1.4)	16 h	0.1 M	47%^b^	4a, 5a
5	Ir-B (2)	NaOAc (1.2)	SO_2_Ph (1.4)	16 h	0.1 M	–	2a (26%)^b^
6	Ir-B (2)	Cs_2_CO_3_ (1.2)	SO_2_Ph (1.4)	16 h	0.1 M	–	2a (31%)^b^
7	Ir-C (2)	CsOAc (1.2)	SO_2_Ph (1.4)	16 h	0.1 M	50%^b^	4a, 5a
8	Ru-A (2)	CsOAc (1.2)	SO_2_Ph (1.4)	16 h	0.1 M	30%^b^	2a (30%)^b^
9	4CzIPN (2)	CsOAc (1.2)	SO_2_Ph (1.4)	16 h	0.1 M	21%^b^	2a (31%)^b^
10	Eosin Y^e^ (2)	CsOAc (1.2)	SO_2_Ph (1.4)	16 h	0.1 M	–	2a (51%)^b^
11	Ir-A (2)	CsOAc (1.2)	OAc (1.4)	8 h	0.1 M	46%^b^	None
12	Ir-A (2)	CsOAc (1.2)	S*t*-Do (1.4)	8 h	0.1 M	51%^b^	None
13	Ir-A (2)	CsOAc (1.2)	Br (1.4)	8 h	0.1 M	–	2a (66%)^b^
14	Ir-A (2)	CsOAc (1.2)	OAc (1.4)	20 min	0.05	52%^d^	4a
15	Ir-A (1)	CsOAc (1.2)	OAc (1.4)	20 min	0.05	58%^c,d^	4a
16	Ir-A (1)	CsOAc (1.2)	OAc (1.1)	20 min	0.05	57%^d^	–
17	Ir-A (1)	CsOAc (1.2)	OPiv (1.1)	20 min	0.05	45%^d^	–
18	Ir-A (1)	CsOAc (1.2)	OTFA (1.1)	2 h	0.05	27%^d^	–

New C–C bonds are indicated in red.

^a^Reactions run on 0.2 mmol scale.

^b^Yield for the major diastereomer determined on the crude product by ^1^H-NMR analysis using ethylene carbonate as a standard. Levels of diastereoselectivity ranging from 5:1 to 7:1.

^c^Average of three runs.

^d^Isolated yield.

^e^Irradiation at 325 nm in DMF as a solvent.

**Fig. 5:**
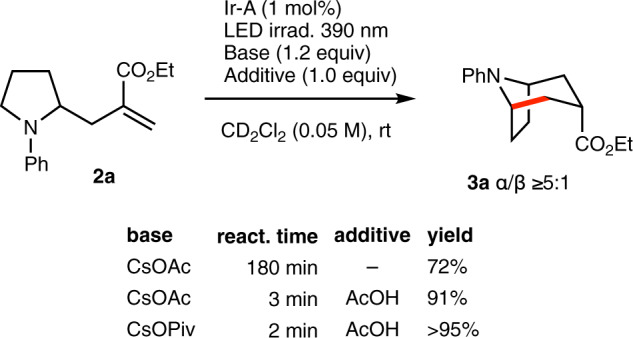
Cyclization of the mono-allylated pyrrolidine 2a. Influence of acid additives on the cyclization step. New C–C bonds is indicated in red.

To gain more understanding on the reaction efficiency, cyclization of the pure mono-allylated **2a** product was examined (Fig. 5). The reaction was run in deuterated dichloromethane under identical conditions (catalyst and base) than the annulation process and was monitored by ^1^H-NMR. Full conversion of **2a** was achieved after 3 h of irradiation and the bicyclic product **3a** resulting from a 6-*endo* cyclization was obtained in 72% yield. The long reaction time necessary to reach full conversion was puzzling since the overall one-pot annulation process was complete within less than 20 minutes. Since acetic acid (one equivalent) is generated during the allylation step leading to **2a**, the reaction was repeated in the presence of one equivalent of acetic acid. Remarkably, the cyclization was finished in 3 min and an improved yield of 91% was obtained. Running the reaction with CsOPiv (1.2 equiv) and acetic acid (1.0 equiv) afforded the cyclized product in nearly quantitative yield within 2 min. This is in accordance with findings reported by Yoon et al. where their photocatalytic addition of α‑aminoalkyl radicals on Michael acceptors was improved by the addition of a Brønsted acids such as TFA^38^.

Adding acetic acid (1 equiv) to the one-pot annulation reaction either at the beginning or during the reaction did not improve the yield indicating that the in situ generated acetic acid was sufficient for the whole process to occur. Running the reaction with CsOPiv (1.2 equivalents) as a base gave results very similar to CsOAc. Overall, these findings show that the optimized conditions for the annulation reaction (Table 1, entry 16) do not need to be modified.

### Reaction scope

**Fig. 6:**
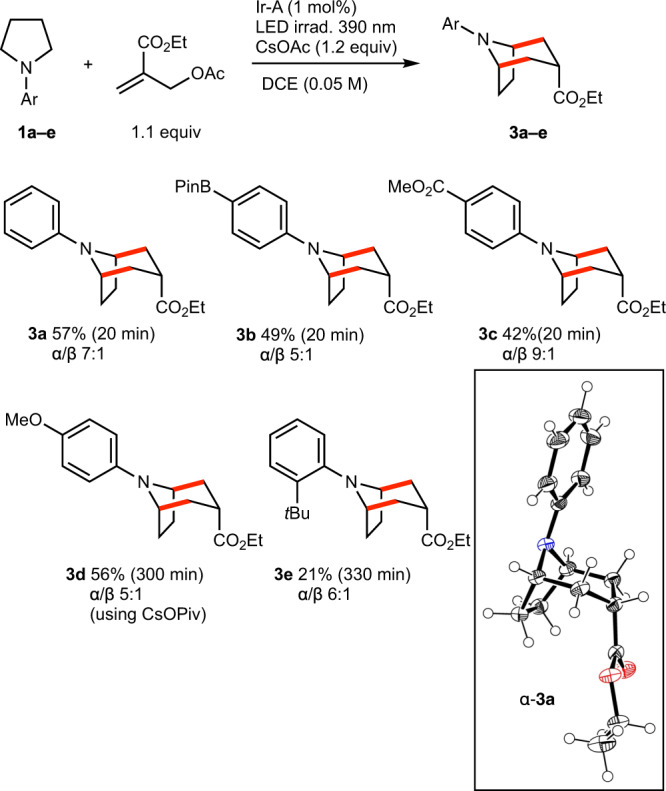
Tropane skeletons via radical annulation. Scope of the *N*-aryl moiety. New C–C bonds are indicated in red. X-ray crystal structure of **3a** (ellipsoids drawn at 50% probability).

The scope of the reaction was examined using the optimized reaction conditions. At first, different *N*-arylpyrrolidines **1a**–**e** were examined (Fig. 6). The reaction works well with p-substituted *N*-arylpyrrolidines leading to **3a**–**3d** in 42–57% yield and good levels of diastereoselectivity in favor of the α isomer (α/β ≥ 5:1). The relative configuration of the **3a** was determined by single crystal X-ray diffraction crystallography. Interestingly, the electron rich *N*-*p*-methoxyphenylpyrrolidine required longer reaction time despite the fact that this compound is more easily oxidizable, indicating that the critical step of the reaction is probably the deprotonation of the radical cations leading to the α-aminoalkyl radicals as discussed by Mariano in his early work (see a discussion of the mechanism, *vide infra*)^39,40^. In this particular case, the use of the slightly more basic CsOPiv instead of CsOAc provided **3d** in a good 56% yield. The presence of *ortho*-substituent proved to be more problematic as shown by the long reaction time and low yield (20%) observed for the formation of **3e**.

**Fig. 7:**
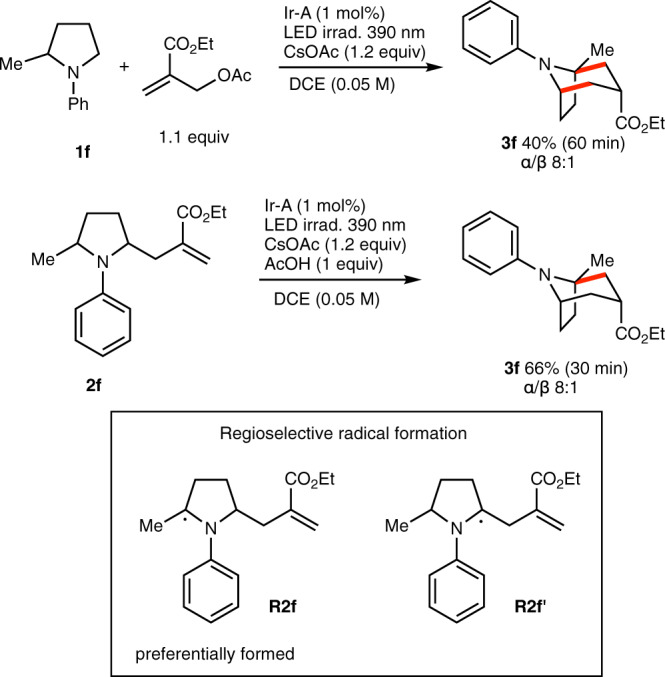
Annulation reaction of *N*-phenyl-2-methylpyrrolidine. New C–C are indicated in red. The reaction involves the regioselective activation of 2,5-disubstituted pyrrolidine to form preferentially **R2f** over **R2f’**.

Next, the use of a 2-substituted pyrrolidine was examined. For this purpose, the 2-methyl substituted *N*-phenylpyrrolidine **1f** was prepared and submitted to our standard reaction conditions (Fig. 7). It afforded the desired bicyclic **3f** containing a quaternary carbon at the bridgehead position in 40% yield. This result was surpassing our expectation since a difficult regioselectivity control was expected for the second step of the process, i.e., the conversion of **2f** to **3f** via 6-*endo*-trig cyclization. A study of this cyclization showed that **3f** could be obtained from **2f** in 66% isolated yield indicating that the desired α-amino-α-methyl radical **R2f** was preferentially formed over **R2f’**. This cyclization yield compared well with the non-methylated substrate **2a** that afforded **3a** in 77% isolated yield.

**Fig. 8:**
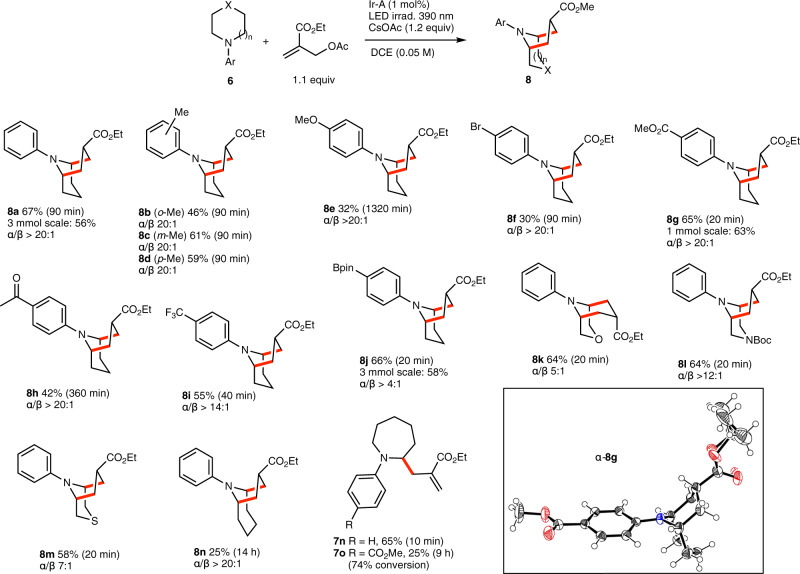
Homotropane [3.3.1] and extended tropane [4.3.1] skeletons. New C–C bonds are indicated in red. Products are drawn in their major conformations attributed from ^1^H-NMR spectra analysis. X-ray crystal structures of α-**8g**, ellipsoids drawn at 50% probability (oxygen and nitrogen atom are represented in red and blue, respectively).

Encouraged by these results, we then turned our attention to the preparation of the homotropane 9-azabicyclo[3.3.1]nonane skeleton as well as higher [4.3.1]-analogues (Fig. 8). Piperidine derivatives proved in general to react slightly better than pyrrolidine derivatives. *N*-Phenylpiperidine **6a** gave the cyclized product **8a** in 67% yield (56% on 3 mmol scale) with an excellent level of diastereoselectivity (α/β > 20:1). The *N*-toluyl derivatives **8b**–**8d** were obtained in similar yields and levels of diastereoselectivity. The electron rich *N*-*p*-anisyl derivatives in the presence of CsOPiv as a base required longer reaction time and afforded **8e** in lower yield (32%). Aromatic rings substituted by electron withdrawing groups afforded the homotropanes **8f**–**8j** in good yields and shorter reaction times in most cases. The *p*-ester substituted homotropane **8g** was prepared 63% yield on 1 mmol scale and the *p*-pinacolboryl derivative **8j** in 58% yield on 3 mmol scale. Gratifyingly, in all the scale-up experiments for **8a**, **8g** and **8j**, the catalyst loading could be decreased to 0.5 mol%. The relative α-configuration of **8g** was confirmed by single crystal X-ray diffraction crystallography. Annulations starting from *N*-phenylmorpholine, *N*-phenylpiperazine and *N*-phenylthiomorpholine derivatives **6k**–**m** provided the bicyclic amines **8k**–**m** in good yields. Remarkably, no products arising from the ring-opening of the piperazine^41^ and thiomorpholine^42,43^ were observed. Finally, the reactivity of azepane derivatives were investigated. The *N*-phenyl derivative **6n** gave the bicylic amine **8n** in a modest 25% yield after 14 h. When the reaction was stopped after 10 min, the allylated product **7n** was obtained in 65% yield together with some cyclized product indicating that the 6-*endo* cyclization was probably the yield limiting step. This was confirmed by an independent cyclization attempt starting from **7n** that afforded **8n** in 24% yield after two hours, along with 9% unreacted **7n** and some unidentified side products. The *N*-(*p*-methoxycarbonylphenyl) derivative **6o** was also examined. After 9 h of reaction, only the allylated product **7o** was formed in a low 25% yield and incomplete conversion.

**Fig. 9:**
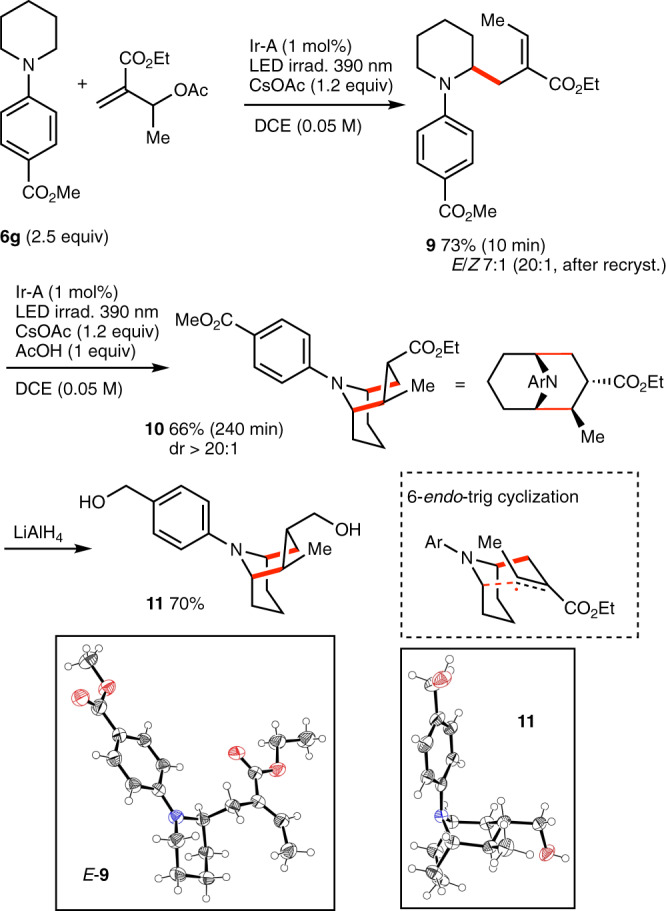
2,3-Disubsituted [4.4.1]-homotropane. Diastereoselective preparation of a 2,3-disubsituted [4.4.1]-homotropane. New C–C are indicated in red. X-Ray crystal structure of *E*-**9** and **1**, ellipsoids drawn at 50% probability.

2-Alkyl-3-ethoxycarbonyl substituted tropanes and homotropanes skeletons are expected to be accessible by using easily available acetylated Baylis–Hillman adducts. In order to test this hypothesis, ethyl 2-(1-acetoxyethyl)acrylate was prepared from the ethyl acrylate and acetaldehyde and reacted with *N*-(*p*-methoxycarbonylphenyl)piperidine **6g** (Fig. 9). When 1.1 equivalent of the trap was employed under our standard reaction conditions, prolongated irradiation afforded a mixture of the desired tropane skeleton **10** together with mono-allylated piperidine **9** and the bis-allylated product. Using a 2.5 fold excess of the amine allowed for the formation of the bis-allylated product to be suppressed and, after 10 min of irradiation, the intermediate **9** was obtained in 73% yield and with a good *E*-stereoselectivity (*E/Z* 7:1). Recrystallization of **9** afforded a virtually diastereomerically pure product (*E*/*Z* > 20:1). Gratifyingly, under our optimized cyclization conditions the major *E*-**9** afforded **10** in 66% yield and as a single diastereomer (dr > 20:1). However, despite the good results obtained for both steps separately, the one-pot process provided a mixture of **9** and **10**. This mixture can be treated under the cyclization conditions to ultimately give the cyclized product **10** in 71% yield contaminated with a by-product, presumably a diastereomer. The diester **10** was converted to corresponding diol **11** whose relative configuration could be determined by single crystal X-ray diffraction crystallography (Fig. 9). The formation of the major diastereomers results presumably from a chair-like transition state as depicted in Fig. 9.

**Fig. 10:**
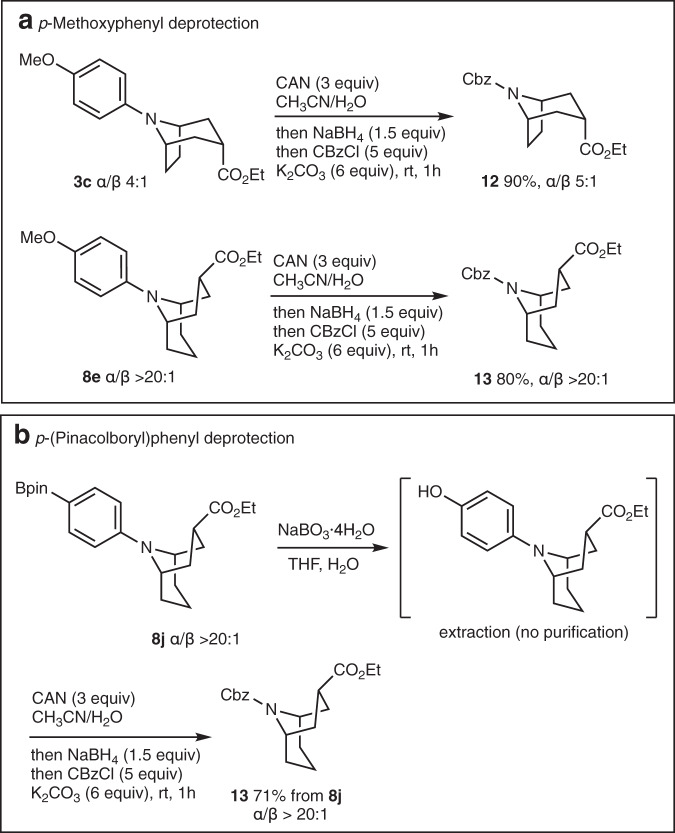
N-Dearylation. Dearylation of (**a**) the *N*-MeOPh derivative **3c** and **8e**, and (**b**) the *N*-*p*-pinBPh derivative **8j**.

Since most of the bicyclic alkaloids are either secondary amines (nortropanes, homotropanes) or *N*-methylated tertiary amines (tropanes), it is important to achieve the deprotection of the *N*-aryl moiety. Methoxyphenyl substituents can be removed under oxidative conditions using chemical oxidants such as cerium ammonium nitrate (CAN) or electrochemical methods^44^. The cleavage of the *N*-*p*-MeOPh group of **3c** and **8e** was investigated first and proved to be problematic when an aqueous CAN solution was used^45^ as the quinone liberated during the process reacted with the liberated secondary amines. Gratifyingly, the deprotections were successfully achieved using CAN in CH_3_CN/water followed by subsequent treatment of the reaction mixture with sodium borohydride to reduce the quinone, then with benzyl chloroformate to prepare their *N*-Cbz-protected form^46^. Under these conditions, the desired Cbz-protected nortropane **12** and homotropane **13** were obtained in 90% and 80% yields, respectively, without erosion of the diastereomeric ratio (Fig. 10a). The Cbz-protecting group was introduced to facilitate product purification but also for both its facile deprotection and conversion to the corresponding *N*-methyl derivative^47^. Since annulation reactions with the *N*-*p*-MeOPh substituted cyclic amines were slow and moderately efficient, we also investigated the deprotection of the *N*-(*p*-pinacolboryl)phenyl derivative **8j** that was formed in good yield. The dearylation of **8j** to **13** (Fig. 10b) was conveniently performed in 71% yield by sodium perborate treatment, affording after simple extraction the crude phenol, followed by CAN and CbzCl treatment according to the optimized procedure developed for the *para*-methoxy derivatives **3c** and **8e**.

**Fig. 11:**
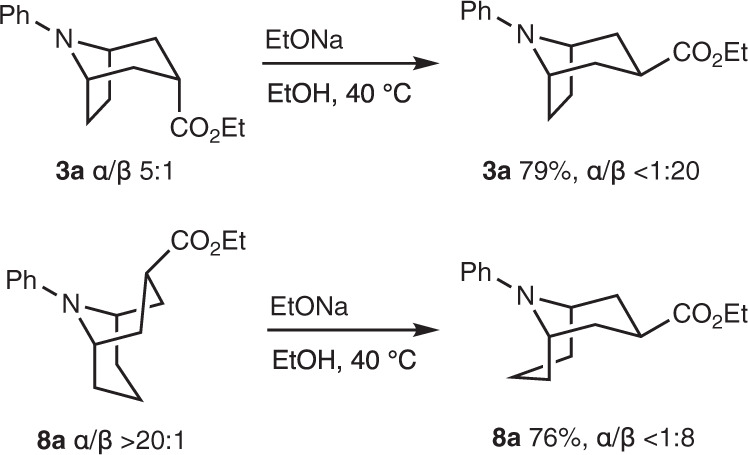
Epimerization. Base promoted thermodynamic epimerization of tropane and homotropane derivatives.

Finally, we hypothesized that the major α-ethoxycarbonyl substituted tropanes and homotropanes were formed by kinetic protonation of the intermediate enolate from the less hindered *exo*-face (see mechanism, *vide infra*). Epimerization of α-stereoisomers to the more stable β-products was therefore examined. Heating the ester **3a** and **8a** at 40 °C in ethanol in the presence of sodium ethoxide afforded the β isomers in good to excellent levels of diastereoselectivity (Fig. 11). This strategy is expected to be useful for the stereocontrolled synthesis of tropanes and homotropane alkaloids.

### Mechanism

**Fig. 12:**
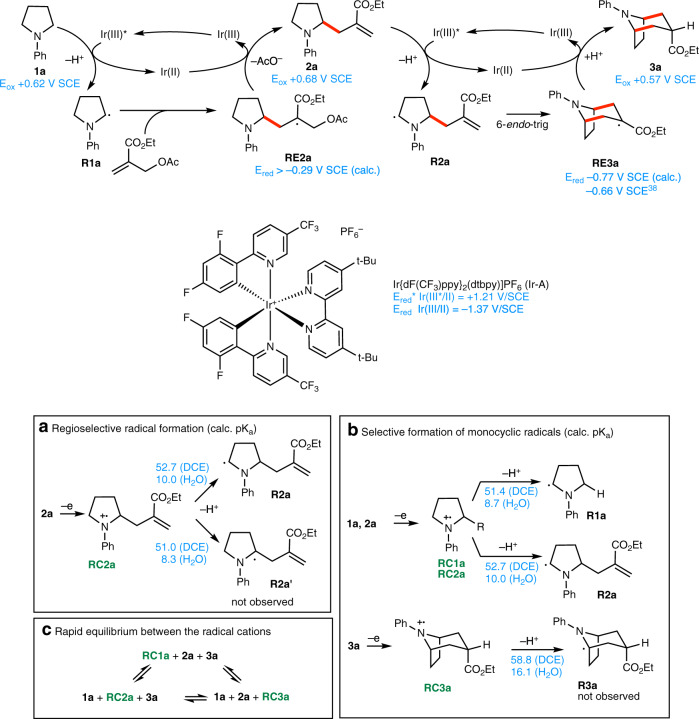
Mechanism. Possible mechanism for the [3 + 3] radical annulation and analysis of (**a**) the regioselectivity of radical formation, (**b**) the selective formation of bicyclic radicals, and (**c**) the equilibrium between the different radical cations formed during the reaction. New C–C bonds are indicated in red. Redox potentials and pK_a_s are indicated in blue. Radical cations are labeled in green.

As mentioned in the introduction, the photocatalytic generation of α-aminoalkyl radicals from cyclic anilines and their subsequent intermolecular addition to Michael acceptors are well-established processes. However, the selectivity of the reaction sequence responsible for the annulation process is quite unique and deserves to be discussed (Fig. 12). Upon blue light irradiation the catalyst Ir-A reaches its excited state Ir(III)*(E_ox*_ Ir (III*/II) = +1.21 V SCE in MeCN)^48^ and is able to oxidize *N*-phenylpyrrolidine **1a** to its aminium radical cation **RC1a** (E_red_ = +0.62 V/SCE in MeCN), which generates the α-aminoalkyl radical **R1a** upon deprotonation. This nucleophilic radical adds to the electron-poor double bond of the allylic trap, leading to an intermediate α-ester radical **RE2a**. The reduction potential of the tertiary radical **RE2a** is estimated to be E_red_ > –0.29 V SCE (see supporting information for details). Such a radical is expected to be readily reduced by the Ir(II) species (E_red_ Ir (III/II) = –1.37 V/SCE)^48^, thus closing the first catalytic cycle and providing a transient enolate intermediate that produces the mono-allylated pyrrolidine **2a** upon β-fragmentation. The intermediate **2a** is now able to enter the second catalytic cycle. After oxidation of **2a** and subsequent deprotonation, the α-aminoalkyl radical **R2a** is formed selectively. The latter undergoes a 6-*endo*-trig cyclization, leading to the bicyclic α-ester radical **RE3a** whose calculated reduction potential (Er_ed_ = –0.77 V SCE, see supporting information for details) is in good agreement with the experimental value of E_red_ = –0.66 V SCE reported for the secondary α-ester radical CH_3_CH(•)CO_2_Me^49^. This allows for its facile reduction by the Ir(II) species, thus closing the second catalytic cycle. *exo*-Selective protonation of the transient enolate ultimately delivers the desired bicyclic product **3a**.

The success of this approach relies on three key features. Firstly, the radical cation **RC2a** needs to be regioselectively deprotonated to form the radical **R2a**. No product results resulting from **R2a**′ has been identified but we cannot exclude that such products are formed in small amount (Fig. 12a). The calculated pK_a_ values for the C(2)–H and C(3)–H bond (see scheme 11 A) are very close and cannot be used to rationalize this result. Nevertheless, this type of selectivity for the less substituted position is well-established and can be rationalized by both steric and stereoelectronic effects^25,50–54^. Secondly, in principle all three amines **1a**, **2a** and **3a** present in solution can be easily oxidized by the Ir(III)* catalyst excited state. However, only **RC1a** and **RC2a** (calculated pK_a_ 51.4 and 52.7 in DCE) are undergoing deprotonation to generate α-aminoalkyl radicals. The deprotonation of the bicyclic radical **RC3a** (pK_a_ 58.4) is much less favorable and no product resulting from the bicyclic radical **R3a** has been observed (Fig. 12b). A separate attempt to achieve allylation of pure **3a** under our standard reaction conditions led only to partial decomposition of the starting material without formation of an allylated product. Since the oxidation potentials of all three amines **1a**, **2a** and **3a** are close and well below the oxidation potential of the excited Ir(III)* catalyst, we believe that the efficacy of the whole process is due to the fact that oxidation of **3a**, which is becoming increasingly important as the reaction proceeds, is not leading to its decomposition as long as **1a** and **2a** are present in solution due to rapid electron transfers between the different amines in solution (Fig. 12c). Such rapid electron transfers involving radical cations are well-documented in the literature^55–57^ and have led to the use of triarylaminium radical cation as organic mediator in electro-organic synthesis^58–61^.

The proposed mechanism suggests that the efficiency of the annulation process is dictated by the formation of a final product that cannot deliver efficiently an α-aminoakyl radical due to an unfavorable (or less favorable) deprotonation step. This may explain the difficulties and low yields observed in the annulation of azepanes leading to [4.3.1] bicycles (see formation of **8n** in Fig. 8), for which the larger bicyclic systems are expected to favor deprotonation due to an increase of conformational flexibility leading to product and catalyst degradation. This assumption is supported by calculations, the oxidation potential of **8n** (E_ox_ + 0.66 V/SCE) lying within the range of our catalyst and the pK_a_ of the corresponding radical cation **RC8n** (pK_a_ 49.5 (DCE), 6.8 (H_2_O)) being far below the ones of the less flexible **RC3a** (pK_a_ 58.8 (DCE), 16.1 (H_2_O)) and **RC8a** (pK_a_ 57.1 (DCE), 14.4 (H_2_O)) and similar to the one of the monocyclic amines.

**Fig. 13:**
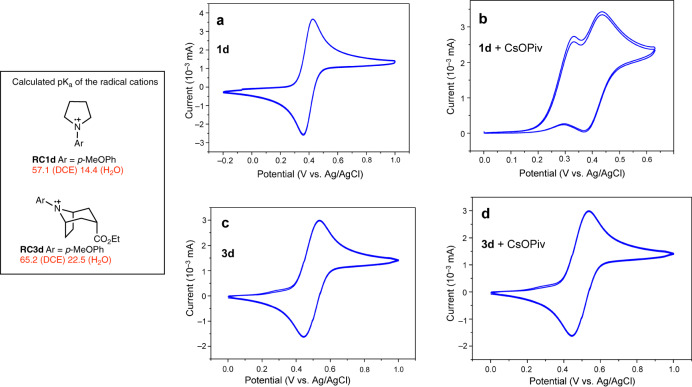
Electrochemical study. Cyclic voltammograms of *N*-*p*-methoxyphenylamines **1d** and **3d** (1 mM) in the absence (**a** and **c**) and in the presence of cesium pivalate (1.2 mM) (**b** and **d**). Voltammograms were recorded at 100 mV/s in acetonitrile containing [Bu_4_N][PF_6_] (0.1 M) as a supporting electrolyte. pK_a_s are indicated in red. Voltammogram traces are depicted in blue.

Counter-intuitively, the reactions with electron-enriched aniline systems such as *N*-(para-methoxyphenyl)pyrrolidine **1d** (to form **3d**, see Fig. 6) and *N*-(para-methoxyphenyl)piperidine **6e** (to form **8e**, Fig. 8) were more difficult than those with electron-poorer systems. They required much longer reaction times and, in the case of **1d**, the use of a slightly stronger base. This indicates that the critical step of the process is probably not the amine oxidation but rather the deprotonation of the radical cation. Calculations showed that the more stable *para*-methoxyphenyl substituted radical cation **RC1d** is also much less acidic (pK_a_ = +57.1 (DCE)/+14.4 (H_2_O)) than **RC1a** (pK_a_ = +51.4 (DCE)/+8.7 (H_2_O)). The electrochemical investigation of **1d** by cyclic voltammetry provided a quasi-reversible oxidation wave in the absence of added base (Fig. 13a) whilst the simple *N*-phenylpyrrolidine **1a** produced irreversible oxidation event (see supporting information)^62,63^. The addition of cesium pivalate drastically changed the cyclic voltammogram of **1d** and two oxidation waves were observed (Fig. 13b). A first irreversible oxidation wave appeared at a slightly lower oxidation potential than the oxidation event of the amine alone. This shift of potential relative to the cyclic voltammogram in the absence of base is attributed to an oxidation event of the amine interacting with cesium pivalate, possibly a proton-coupled electron transfer process^64,65^. The second wave, whose potential corresponds to the oxidation event observed in the absence of base, becomes less reversible in presence of cesium pivalate. The voltammogram of the tropane **3d** (Fig. 13c) indicates as anticipated a reversible oxidation process. Interestingly, it is almost not affected by the presence of cesium pivalate indicating that the bicyclic cation radical of **3d** is not deprotonated by the base during the measurement (Fig. 13d). This observation supports the proposed mechanism and the fact that the photoredox annulation process stops at the bicyclic stage.

**Fig. 14:**
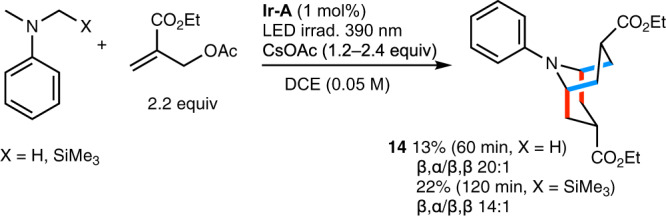
Double annulation process. Synthesis of a homotropane skeleton via a double [3 + 3] annulation reaction. C–C Bonds formed during the first annulation process are indicated in red, C–C Bonds formed during the second annulation process are indicated in blue.

The hypothesis that the reactions stop at the bicyclic stage due to inefficient deprotonation of the bicyclic radical cation offers the opportunity to run a more complex reaction process involving a bis-annulation process starting from a tertiary acyclic aniline derivative and ending with an oxidation-resistant bicyclic system. This assumption was tested with *N,N*-dimethyl aniline. In the presence of 2.2 equivalents of the allylic acetate radical trap added in two portions (at the beginning of the reaction and after 15 min), the product of bis-annulation product **14** was isolated in 13% yield with good stereocontrol. By starting from *N*-methyl-*N*-trimethylsilylmethylaniline, the yield for the formation of **14** could be improved to 22%. In this process, the product of the first annulation (Fig. 14, blue bond formation) leads to a piperidine derivative that can further react via a second annulation process (Fig. 14, red bond formation) to produce **14**. All attempts to stop at the piperidine stage gave complex inseparable mixture of products.

## Conclusion

In summary, we have developed a new annulation strategy for the synthesis of bicyclic alkaloid skeletons. The reactions take place under mild conditions and afford *N*-arylated tropane and homotropane frameworks in good yields and good to excellent levels of diastereoselectivity starting from simple and readily available starting materials using visible-light photoredox catalysis. This annulation process takes advantage of the unique reactivity of ethyl 2-(acetoxymethyl)acrylate as a 1,3-bis radical acceptor and of cyclic *N,N*-dialkylanilines as radical 1,3-bis radical donor. This method complements nicely the classical Robinson synthesis by allowing to prepare directly the biologically relevant *N*-arylated skeletons and by introducing an ester group at position 3 suitable for further derivatization toward application in medicinal chemistry. Preliminary results with differently 2-substituted allyl acetates indicate that the ester group can be substituted by other electron withdrawing groups such as a nitrile, a sulfone and a boronic ester. Details will be reported in due time together with the synthesis of optically pure biologically relevant compounds.

## Methods

### General procedure

In an oven-dried 10 mL vial were successively added ethyl 2-(acetoxymethyl)prop-2-enoate (0.22 mmol, 1.1 equiv), *N*-arylpyrrolidine or *N*-arylpiperidine (0.20 mmol, 1 equiv.), [Ir{dF(CF_3_)ppy}_2_(dtbpy)]PF_6_ (2.2 mg, 0.02 mmol, 1 mol%) and cesium acetate (46 mg, 0.24 mmol, 1.2 equiv.). The vial was closed with a rubber septum and evacuated/filled with N_2_ (3×). Finally, dry and degassed 1,2-DCE (4.00 mL) was added. The resulting yellow mixture was placed 5 cm away from a 390 nm blue LED and stirred until completion of the reaction. Reactions usually turned orange and a fine white precipitate was observed. The reaction mixture was diluted with sat. NaHCO_3_ (3 mL) and the aq. phase was extracted with CH_2_Cl_2_ (3 × 5 mL) and the combined organic phases were dried over Na_2_SO_4_, filtered, and concentrated under reduced pressure to give crude product as an orange oil. Purification by flash column chromatography on neutral Alox^®^ or SiO_2_ afforded the bicyclic product. Details including experimental procedures and product characterizations are avaiable in the Supplementary Methods (Supplementary Tables 1–6). Copies of relevant NMR data are available in the Supplementary Note 1.

Calculations. All DFT calculations were performed with the ADF (Amsterdam Density Functional) code developed by E. J. Baerends et al.^66^ using triple-zeta basis sets (no frozen core). Geometry optimizations were performed *in vacuo* relying on the Generalized Gradient Approximation VBP exchange-correlation (XC) potential (VWN and BP by Vosko et al.^67^, corrective terms by Becke^68^ for the exchange and Perdew^69^ for the correlation) with ADF grid precision 6 throughout. Details are available in the Supplementary Methods (Supplementary Figs. 1,2 and Supplementary Tables 7–10).

### Cyclic voltammetry

Cyclic Voltammetry experiments were performed in acetonitrile at room temperature in an argon-filled glovebox with 0.1 M of [Bu_4_N][PF_6_] as a supporting electrolyte. Data were collected using a BioLogic SP-300 potentiostat connected to a three-electrodes system, including a glassy carbon disk (d = 1 mm) working electrode, a platinum wire counter electrode, and an Ag/AgCl reference electrode. The voltammograms of each compound were recorded at different scan rates (from 20 mV/s to 20 V/s). The linearity of the oxidation current with the square root of the scan rates was checked for the four compounds in the different experimental cases. Potential calibration was performed at the end of each data collection cycle using the ferrocene/ferrocenium couple as an internal standard (E° = 0.380 V/SCE). More details are available in the Supplementary Methods (Supplementary Figs. 3,4 and Supplementary Tables 11).

## Supplementary information

Supplemental Material

Description of Additional Supplementary Files

Supplementary Data 1

Supplementary Data 2

Supplementary Data 3

Supplementary Data 4

Supplementary Data 5

## Data Availability

The X-ray crystallographic coordinates for structures reported in this Article have been deposited at the Cambridge Crystallographic Data Centre (CCDC), under deposition number CCDC 2111680 (**3a**), CCDC 2111681 (α-**8g**), CCDC 2111682 (*E*-**9**), and CCDC 2111684 (**11**). These data can be obtained free of charge from The Cambridge Crystallographic Data Centre via www.ccdc.cam.ac.uk/data_request/cif. The cif files for **3a**, α-**8g**, **9** and **11** are also available as Supplementary Data 1–4. DFT geometry-optimized coordinates are available in the Supplementary Data 5. Any other relevant data are available from the authors upon reasonable request.
