# Supplementary material for: Tropane and related alkaloid skeletons via a radical [3+3]-annulation process
Source: Commun Chem. 2022 Apr 28;5:57. doi: 10.1038/s42004-022-00671-x (PMC9814087; doi:10.1038/s42004-022-00671-x)
Supplement: Supplementary file 1 — Supplemental Material [file 42004_2022_671_MOESM1_ESM.pdf]

Supporting Information

Tropane and Related Alkaloid Skeletons via a Radical [3+3]-Annulation Process

Eloïse Colson, Julie Andrez, Ali Dabbous[b] Fabrice Dénès, Vincent Maurel,* Jean-Marie Mouesca,* and Philippe Renaud*

a) University of Bern, Department of Chemistry, Biochemistry and Pharmaceutical Sciences (DCBP), Freiestrasse 3, CH-3012 Bern, Switzerland

b) Univ. Grenoble Alpes, CEA, CNRS, IRIG , SyMMES, F-38000 Grenoble, France

vincent.maurel@cea.fr, jean-marie.mouesca@cea.fr, philippe.renaud@unibe.ch

Table of contents

SUPPLEMENTARY METHODS 6

Synthesis 6

General information 6

Instrumentation 6

Materials 7

Preparation of amine precursors 7

General procedure A 7

1-Phenylpyrrolidine (1a) 7

1-(4-Bromophenyl)pyrrolidine 8

1-[4-(3,3,4,4-Tetramethyl-1lambda3,2,5-bromadioxolan-1-yl)phenyl]pyrrolidine (1b) 8

Methyl 4-pyrrolidin-1-ylbenzoate (1c) 9

1-(4-Methoxyphenyl)pyrrolidine (1d) 9

1-(2-*tert*-Butylphenyl)pyrrolidine (1e) 9

2-Methyl-1-phenyl-pyrrolidine (1f) 9

1-Phenylpiperidine (6a) 10

1-(*o*-Tolyl)piperidine (6b) 10

1-(*m*-Tolyl)piperidine (6c) 10

1-(*p*-Tolyl)piperidine (6d) 11

1-(4-Methoxyphenyl)piperidine (6e) 11

1-(4-Bromophenyl)piperidine (6f) 11

Methyl 4-(1-piperidyl)benzoate (6g) 11

1-[4-(1-Piperidyl)phenyl]ethanone) (6h) 12

1-[4-(trifluoromethyl)phenyl]piperidine (6i) 12

1-[4-(3,3,4,4-Tetramethyl-1lambda3,2,5-bromadioxolan-1-yl)phenyl]piperidine (6j) 12

tert-Butyl 4-phenylpiperazine-1-carboxylate (6l) 13

4-Phenylthiomorpholine (6m) 13

1-Phenylazepane (6n) 14

Methyl 4-(azepan-1-yl)benzoate (6o) 14

Preparation of radical traps 14

Ethyl 2-(benzenesulfonylmethyl)prop-2-enoate 14

Ethyl 2-(hydroxymethyl)prop-2-enoate 15

Ethyl 3-hydroxy-2-methylene-butanoate 15

Ethyl 2-[(2,2,2-trifluoroacetyl)oxymethyl]prop-2-enoate 15

Ethyl 2-(bromomethyl)prop-2-enoate 16

Ethyl 2-(1,1-dimethyldecylsulfanylmethyl)prop-2-enoate 16

General Procedure B: acetylation of allylester-alcohols 17

Ethyl 2-(acetoxymethyl)prop-2-enoate ... 17
2-Ethoxycarbonylallyl 2,2-dimethylpropanoate ... 17
Ethyl 3-acetoxy-2-methylene-butanoate ... 17
Optimization of the one-pot procedure ... 18
Supplementary Table 1. ... 19
Supplementary Table 2 ... 20
Supplementary Table 3 ... 22
Supplementary Table 4 ... 23
One-pot synthesis of the bicyclic products ... 25
General Procedure C (0.2 mmol scale) ... 25
Ethyl 8-phenyl-8-azabicyclo[3.2.1]octane-3-carboxylate (3a) ... 25
Ethyl 8-[4-(4,4,5,5-tetramethyl-1,3,2-dioxaborolan-2-yl)phenyl]-8-azabicyclo[3.2.1]octane-3-carboxylate (3b) ... 26
8-(4-Methoxycarbonylphenyl)-8-azabicyclo[3.2.1]octane-3-carboxylate (3c) ... 27
Ethyl 8-(4-methoxyphenyl)-8-azabicyclo[3.2.1]octane-3-carboxylate (3d) ... 27
Ethyl 8-(2-tert-butylphenyl)-8-azabicyclo[3.2.1]octane-3-carboxylate (3e) ... 28
Ethyl 1-methyl-8-phenyl-8-azabicyclo[3.2.1]octane-3-carboxylate (3f) ... 29
Conformation of homotropanes ... 29
Ethyl 9-phenyl-9-azabicyclo[3.3.1]nonane-3-carboxylate (8a) ... 30
Ethyl 9-(o-tolyl)-9-azabicyclo[3.3.1]nonane-3-carboxylate (8b) ... 31
Ethyl 9-(m-tolyl)-9-azabicyclo[3.3.1]nonane-3-carboxylate (8c) ... 31
Ethyl 9-(p-tolyl)-9-azabicyclo[3.3.1]nonane-3-carboxylate (8d) ... 32
Ethyl 9-(4-methoxyphenyl)-9-azabicyclo[3.3.1]nonane-3-carboxylate (8e) ... 32
Ethyl 9-(4-bromophenyl)-9-azabicyclo[3.3.1]nonane-3-carboxylate (8f) ... 33
Ethyl 9-(4-methoxycarbonylphenyl)-9-azabicyclo[3.3.1]nonane-3-carboxylate (8g). ... 33
Ethyl 9-(4-acetylphenyl)-9-azabicyclo[3.3.1]nonane-3-carboxylate (8h) ... 34
Ethyl 9-[4-(trifluoromethyl)phenyl]-9-azabicyclo[3.3.1]nonane-3-carboxylate (8i) ... 34
Ethyl 9-[4-(4,4,5,5-tetramethyl-1,3,2-dioxaborolan-2-yl)phenyl]-9-azabicyclo[3.3.1]nonane-3-carboxylate (8j) ... 35
Ethyl 9-phenyl-3-oxa-9-azabicyclo[3.3.1]nonane-7-carboxylate (8k) ... 36
O3-tert-Butyl O7-ethyl 9-phenyl-3,9-diazabicyclo[3.3.1]nonane-3,7-dicarboxylate (8l) ... 36
Ethyl 9-phenyl-3-thia-9-azabicyclo[3.3.1]nonane-7-carboxylate (8m) ... 37
Ethyl 10-phenyl-10-azabicyclo[4.3.1]decane-8-carboxylate (8n) ... 37
Diethyl 9-phenyl-9-azabicyclo[3.3.1]nonane-3,7-dicarboxylate (14) ... 38
Unsuccessful substrates ... 39
Characterization of the mono-allylated intermediates ... 40
Ethyl 2-[(1-phenylpyrrolidin-2-yl)methyl]prop-2-enoate (2a) ... 40
Ethyl 2-[(5-methyl-1-phenyl-pyrrolidin-2-yl)methyl]prop-2-enoate (2f) ... 40
Ethyl 2-[(1-phenylazepan-2-yl)methyl]prop-2-enoate (7n) ... 42

Methyl 4-[2-(2-ethoxycarbonylallyl)azepan-1-yl]benzoate (7o) 43
Methyl 4-[2-[(E)-2-ethoxycarbonylbut-2-enyl]-1-piperidyl]benzoate (9) 43
Study of the cyclization step 44
General Procedure D 44
Supplementary Table 5 45
Supplementary Table 6 46
Synthesis of bicyclic compounds from monoallylated cyclic amines 46
Modified General Procedure D 46
Ethyl 8-phenyl-8-azabicyclo[3.2.1]octane-3-carboxylate (3a) 47
Ethyl 1-methyl-8-phenyl-8-azabicyclo[3.2.1]octane-3-carboxylate (3f) 47
Ethyl 10-phenyl-10-azabicyclo[4.3.1]decane-8-carboxylate (8n) 47
Ethyl 9-(4-methoxycarbonylphenyl)-2-methyl-9-azabicyclo[3.3.1]nonane-3-carboxylate (10) 47
Derivatization of 10 for X-ray crystallography 48
[4-[3-(hydroxymethyl)-2-methyl-9-azabicyclo[3.3.1]nonan-9-yl]phenyl]methanol (11) 48
Oxidation of the BPin derivative 49
Ethyl 9-(4-hydroxyphenyl)-9-azabicyclo[3.3.1]nonane-3-carboxylate 49
Ethyl 9-(4-methoxyphenyl)-9-azabicyclo[3.3.1]nonane-3-carboxylate (8e) 50
Epimerization (from the α to the β diastereoisomer) 50
Ethyl 8-phenyl-8-azabicyclo[3.2.1]octane-3-carboxylate (3aβ, *exo* product): 50
Ethyl 9-phenyl-9-azabicyclo[3.3.1]nonane-3-carboxylate (8aβ, exo product) 51
Deprotection of the aryl moiety 51
General procedure E: deprotection of the aryl moiety[21] 51
O8-Benzyl O3-ethyl 8-azabicyclo[3.2.1]octane-3,8-dicarboxylate (12) 52
O9-Benzyl O3-ethyl 9-azabicyclo[3.3.1]nonane-3,9-dicarboxylate (13, from 8e) 54
O9-Benzyl O3-ethyl 9-azabicyclo[3.3.1]nonane-3,9-dicarboxylate (13, from 8j) 54
Characterization of side-products 55
Ethyl 2-[[5-(2-ethoxycarbonylallyl)-1-phenyl-pyrrolidin-2-yl]methyl]prop-2-enoate (4a) 55
Ethyl 1-(2-ethoxycarbonylallyl)-8-phenyl-8-azabicyclo[3.2.1]octane-3-carboxylate 56
Ethyl 3-(1-phenylpyrrolidin-2-yl)-2-[(1-phenylpyrrolidin-2-yl)methyl]propanoate (5a) 56
Methyl 4-[2,6-bis[(Z)-2-ethoxycarbonylbut-2-enyl]-1-piperidyl]benzoate 57

Calculations 58
DFT methodology 58
Supplementary Figure 1 58
Redox potentials 58
pKa constants 59
Supplementary Table 7 60
Supplementary Table 8 61
Supplementary Table 9 62

Supplementary Table 10 62
Conformational study 63
Supplementary Figure 2 64

Electrochemistry 64
General 64
Cyclic Voltammetry setup 64
Results 64
Supplementary Table 11 65
Supplementary Figure 3 66
Supplementary Figure 4 67

SUPPLEMENTARY REFERENCES 68

SUPPLEMENTARY NOTE 1 69

Figures

Supplementary Figure 1 58
Supplementary Figure 2 64
Supplementary Figure 3 66
Supplementary Figure 4 67

Tables

Supplementary Table 1. 19
Supplementary Table 2 20
Supplementary Table 3 22
Supplementary Table 4 23
Supplementary Table 5 45
Supplementary Table 6 46
Supplementary Table 7 60
Supplementary Table 8 61
Supplementary Table 9 62
Supplementary Table 10 62
Supplementary Table 11 65

Supplementary Methods

Synthesis

General information

All the glassware was oven-dried at 160 °C or flame-dried under vacuum, assembled hot and allowed to cool under a positive pressure of nitrogen. Unless otherwise stated, all reactions were performed under nitrogen atmosphere. Non-aqueous reagents were transferred under nitrogen *via* syringe or cannula. Silica gel 60 Å (230–400 mesh particle size, SiliCycle) and aluminum oxide neutral (40–160 µm) were used for flash column chromatography. Thin layer chromatography (TLC) was performed on 0.25 mm silica gel 60 with fluorescent indicator UV 254; visualization under UV light (254 nm) or by staining with a solution of potassium permanganate [$KMnO_4$ (3 g), K_2CO_3 (20 g) and NaOH 5% (3 mL) in H_2O (300 mL)] and subsequent heating.

Instrumentation

^{1}H, ^{13}C, ^{11}B and ^{19}F NMR spectra were recorded on a 300 MHz spectrometer (^{1}H: 300 MHz, ^{13}C: 75 MHz, ^{11}B: 96 MHz, ^{19}F: 282 MHz) and on a 400 MHz spectrometer (^{1}H: 400 MHz, ^{13}C: 101 MHz) operating at 22 °C, unless otherwise stated. Chemical shifts (δ) were reported in parts per million with the residual solvent peak used as an internal standard ($CHCl_3$: δ = 7.26 ppm and C_6H_6: δ = 7.16 ppm for ^{1}H NMR spectra and $CDCl_3$: δ = 77.0 ppm and C_6D_6: δ = 128.0 ppm for ^{13}C NMR spectra). The following abbreviations were used to explain the multiplicities: s (singlet), d (doublet), t (triplet), q (quadruplet), p (pentuplet), hx (hexuplet), hp (heptuplet), m (multiplet), br (broad). The prefix app (apparent) was added when different coupling constants appeared accidentally equal. Coupling constants *J* are reported in Hz and with an accuracy of one unit of the last digit. HRMS analyses were recorded on a hybrid quadrupole time-of-flight mass spectrometer using positive electrospray and on a double-focusing magnetic sector mass spectrometer using electron impact (70 eV). Mass spectra were measured in electron impact (EI) mode at 70 eV, with solid probe inlet, source temperature of 200 °C, acceleration voltage of 5 kV, and resolution of 2’500. The instrument was scanned between m/z 50 and 1000 at scan rate of 2 s / decade in the magnetic scan mode. Perfluorokerosene served for calibration. Infrared spectra were recorded neat on an FT-IR spectrometer equipped with a Golden Gate Single Reflection Diamond ATR System and are reported in wave numbers (cm^{-1}). Kessil lamps were used for photoredox reactions and were run at maximum power (100 W) 5 cm away from reaction vessel with a cooling fan.

Materials

Unless otherwise stated, all reagents were obtained from commercial sources and used without further purification. Aniline and *N*,*N*-dimethylaniline were redistilled prior to use. Solvents for reactions (THF, Et_2O, CH_2Cl_2, *n*-hexane) were first distilled then filtered over two columns of dried alumina under a positive pressure of argon. Commercial benzene and toluene were filtered over two columns of dried alumina under a positive pressure of argon. Commercial 1,2-dichloroethane was dried over a short column of activated neutral alumina under a positive pressure of nitrogen, stored on activated 3Å molecular sieve and used within less than a month; it was degassed by bubbling nitrogen for 15 minutes prior to reaction. Solvents for extraction and flash column chromatography were of technical grade and were distilled prior to use. Commercial photocatalysts (Strem or Aldrich) were used without purification.

Preparation of amine precursors

General procedure A

In a two-neck, oven-dried flask equipped with a magnetic stirring bar and a reflux condenser, was introduced K_2CO_3 (27.5 mmol, 1.1 equiv.), N,N-dimethylformamide (25 mL) and the appropriate aniline (25 mmol, 1 equiv.). The resulting mixture was degassed by bubbling N_2 for 15 min, and then the dibromoalkane (27.5 mmol, 1.1 equiv.) was added to the suspension *via* a syringe. The reaction mixture was heated to 80 °C and stirred overnight at the same temperature. Upon completion (TLC monitoring), the reaction mixture was diluted with Et_2O (100 mL). Water (100 mL) was added and the phases were separated. The organic phase was extracted with 1 M HCl (3 x 50 mL). The pH of the aqueous phase was adjusted to pH = 8 with 1 M NaOH and extracted with Et_2O (3 x 100 mL). The combined organic phases were dried with Na_2SO_4, filtered and concentrated under reduced pressure to give crude product. Purification by flash column chromatography on silica gel afforded the desired products.

The purity of the reagents was taken into account in most cases, hence some discrepancy can be observed between the indicated mass and the number of mmols. The purity of the reagent was only indicated if lower than 97%.

1-Phenylpyrrolidine (1a)

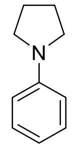

1a
$C_{10}H_{13}N$
MW: 147,2210

Following **General Procedure A** with aniline (4.6 mL, 50.0 mmol) and 1,4-dibromobutane (6.6 mL, 55.0 mmol). Purification by flash column chromatography on silica gel (heptanes/EtOAc 1:30 to 1:10) afforded 1-phenylpyrrolidine as a pale-yellow oil (6.27 g, 42.6 mmol, 85%). The product was stored at –18 °C, protected from light and under N_2.

^{1}H NMR (300 MHz, $CDCl_3$): δ 7.24 (m, 2H), 6.67 (m, 1H), 6.58 (m, 2H), 3.37–3.22 (m, 4H), 2.02 (m, 4H).

The physical and spectral data are in accordance with the reported literature data.[1]

1-(4-Bromophenyl)pyrrolidine

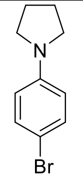

$C_{10}H_{12}BrN$
MW: 226,1170

Following **general procedure A** with 4-bromoaniline (869 mg, 5.00 mmol) and 1,4-dibromobutane (0.66 mL, 5.50 mmol). Purification by silica gel column chromatography (heptanes/EtOAc 1:9) afforded 1-(4-bromophenyl)pyrrolidine as an off-white solid (148 mg, **13%**).

^1H NMR (300 MHz, $CDCl_3$): δ 7.28 (m, 2H), 6.46 – 6.38 (m, 2H), 3.33 – 3.18 (m, 4H), 2.08 – 1.91 (m, 4H).

The physical and spectral data are in accordance with the reported literature data.[2]

1-[4-(3,3,4,4-Tetramethyl-1lambda3,2,5-bromadioxolan-1-yl)phenyl]pyrrolidine (1b)

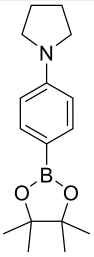

1b
$C_{16}H_{24}BNO_2$
MW: 273,1830

Synthesized following a reported procedure.[3]

In a two-neck round-bottomed flask equipped with a reflux condenser was added 1-(4-bromophenyl)pyrrolidine (150 mg, 0.663 mmol) and degassed 1,4-dioxane (5 mL). To the solution was then added Bis(pinacolato)diboron (B_2Pin_2) (253 mg, 0.995 mmol), KOAc (195 mg, 1.99 mmol) and $PdCl_2$(dppf)-CH_2Cl_2 (12.0 mg, 0.0164 mmol). The reaction was stirred at 90 °C for 16 h. The reaction mixture was allowed to cool down to rt and was filtered through a pad of celite (washing with EtOAc). Volatile organic solvents were evaporated *in vacuo* to give crude product. Purification by flash column chromatography on silica gel (heptanes/EtOAc 98:2) afforded the title compound as an off-white solid (87 mg, 0.25 mmol, 38%).

Alternative procedure:

In a two-neck flask equipped with a magnetic stirring bar and a reflux condenser were introduced 4-(4,4,5,5-tetramethyl-1,3,2-dioxaborolan-2-yl)aniline (1.11 g, 5.00 mmol), N,N-dimethylformamide (5 mL), diisopropylethylamine (DIPEA) (2.56 mL, 15.0 mmol), and 1,4-dibromobutane (0.67 mL, 5.50 mmol). The reaction mixture was heated to 80 °C and stirred overnight at the same temperature. Upon completion (TLC monitoring), the reaction mixture was diluted with Et_2O (20 mL). Water (10 mL) was added and the phases were separated. The organic phase was washed with water (3 x 10 mL). The organic phase was dried with Na_2SO_4, filtered and concentrated under reduced pressure to give crude product. Purification by flash column chromatography on silica gel (pentane:Et_2O 90:10) afforded the desired product as a white crystalline solid (1.15 g, 4.2 mmol, 84%).

^1H NMR (300 MHz, C_6D_6): δ 8.34–8.23 (m, 2H), 6.58–6.48 (m, 2H), 2.86–2.74 (m, 4H), 1.41–1.27 (m, 4H), 1.20 (s, 12H). ^{11}B NMR (96 MHz, C_6D_6): δ 31.7.

The physical and spectral data are in accordance with the reported literature data.[4]

Methyl 4-pyrrolidin-1-ylbenzoate (1c)

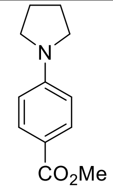

1c
$C_{12}H_{15}NO_2$
MW: 205,2570

Following **General Procedure A** with methyl 4-aminobenzoate (2.29 g, 15.0 mmol) and 1,4-dibromobutane (2.0 mL, 16.5 mmol). Purification by flash column chromatography on silica gel (heptanes/EtOAc 1:10) afforded methyl 4-pyrrolidin-1-ylbenzoate as a white solid (334 mg, 1.65 mmol, 11%).

^{1}H NMR (300 MHz, $CDCl_3$): *δ* 7.94–7.86 (m, 2H), 6.56–6.47 (m, 2H), 3.85 (s, 3H), 3.40–3.31 (m, 4H), 2.10–1.96 (m, 4H).

The physical and spectral data are in accordance with the reported literature data.[5]

1-(4-Methoxyphenyl)pyrrolidine (1d)

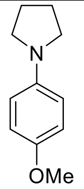

1d
$C_{11}H_{15}NO$
MW: 177,2470

Following **General Procedure A** with 4-methoxyaniline (6.16 g, 50.0 mmol) and 1,4-dibromobutane (6.6 mL, 55.0 mmol). Purification by flash column chromatography on silica gel (pentane/Et_2O 90:10) afforded 1-(4-methoxyphenyl)pyrrolidine as a white solid (7.36 g, 41.5 mmol, 83%). The product was stored at –18 °C, protected from light and under N_2.

^{1}H NMR (300 MHz, $CDCl_3$): *δ* 6.89–6.81 (m, 2H), 6.58–6.50 (m, 2H), 3.76 (s, 3H), 3.28–3.18 (m, 4H), 2.03–1.95 (m, 4H).

The physical and spectral data are in accordance with the reported literature data.[5]

1-(2-*tert*-Butylphenyl)pyrrolidine (1e)

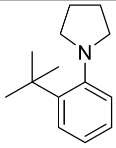

1e
$C_{14}H_{21}N$
MW: 203,3290

Following **General Procedure A** with 2-*tert*-butylaniline (1.56 mL, 10.0 mmol) and 1,4-dibromobutane (1.3 mL, 11.0 mmol). Purification by flash column chromatography on silica gel (heptanes/EtOAc 20:1) afforded 1-(2-*tert*-butylphenyl)pyrrolidine as a colorless oil (879 mg, 4.3 mmol, 43%).

^{1}H NMR (300 MHz, $CDCl_3$): *δ* 7.39–7.35 (m, 2H), 7.23 (td, *J* = 7.6, 1.7 Hz, 1H), 7.12 (td, *J* = 7.8, 1.6 Hz, 1H), 3.07–2.90 (m, 4H), 2.00–1.85 (m, 4H), 1.43 (s, 9H). ^{13}C NMR (75 MHz, $CDCl_3$): *δ* 151.8 (Cq), 149.3 (Cq), 127.1 (CH_{Ar}), 126.8 (CH_{Ar}), 126.7 (CH_{Ar}), 125.6 (CH_{Ar}), 56.3 (2xCH_2), 35.6 (Cq), 31.2 (3xCH_3), 24.8 (2xCH_2). FT-IR (neat, cm^{-1}): 2949, 2907, 2872, 2805, 1485, 1440, 1121, 1088, 1054, 753, 532. HRMS (ESI) m/z: $[M+H]^+$ Calcd for $C_{14}H_{22}N$ 204.1747. Found 204.1738. R_f = 0.75 (heptanes/EtOAc 40:1).

2-Methyl-1-phenyl-pyrrolidine (1f)

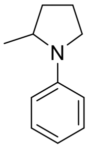

1f
$C_{11}H_{15}N$
MW: 161,2480

In a two-neck oven-dried flask equipped with a magnetic stirring bar and a reflux condenser, was introduced K_2CO_3 (1.54 g, 11.0 mmol), N,N-dimethylformamide (10 mL) and aniline (1.0 mL, 11.0 mmol). The resulting mixture was degassed by bubbling N_2 for 15 min, and then 1,4-dibromopentane (1.4 mL, 10.0 mmol) was added to the suspension *via* a syringe. The reaction mixture was heated to 80 °C and stirred overnight at the same

temperature. Upon completion (TLC monitoring), the mixture was diluted with EtOAc (40 mL). Water (40 mL) was added and the phases were separated. The organic phase was extracted with 1 M HCl (3 x 25 mL). The water phase was adjusted to pH = 8 with 1 M NaOH and extracted with EtOAc (3 x 25 mL). The combined organic phases were dried with Na_2SO_4, filtered and concentrated to give crude product. Purification by flash column chromatography on silica gel (heptanes/EtOAc 20:1 to 10:1) afforded 2-methyl-1-phenyl-pyrrolidine as a yellow oil (1.07 g, 6.7 mmol, 67%).

^{1}H NMR (300 MHz, $CDCl_3$): δ 7.29–7.14 (m, 2H), 6.63 (m, 3H), 3.89 (m, 1H), 3.48–3.38 (m, 1H), 3.17 (m, 1H), 2.17–1.92 (m, 3H), 1.78–1.66 (m, 1H), 1.19 (d, *J* = 6.2 Hz, 3H).

The physical and spectral data are in accordance with the reported literature data.[1]

1-Phenylpiperidine (6a)

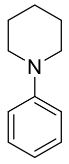

6a
$C_{11}H_{15}N$
MW: 161,2480

Following **General Procedure A** with aniline (4.6 mL, 50.0 mmol) and 1,5-dibromopentane (7.5 mL, 55.0 mmol). Purification by flash column chromatography on silica gel (pentane/Et_2O 20:1) afforded 1-phenylpiperidine as a colorless oil (6.12 g, 38 mmol, 76%).

^{1}H NMR (300 MHz, $CDCl_3$): δ 7.30–7.19 (m, 2H), 6.99–6.89 (m, 2H), 6.86–6.78 (m, 1H), 3.20–3.10 (m, 4H), 1.77–1.65 (m, 4H), 1.63–1.52 (m, 2H).

The physical and spectral data are in accordance with the reported literature data.[6]

1-(*o*-Tolyl)piperidine (6b)

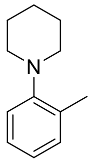

6b
$C_{12}H_{17}N$
MW: 175,2750

Following **General Procedure A** with 2-methylaniline (1.06 mL, 10.0 mmol) and 1,5-dibromopentane (1.5 mL, 11.0 mmol). Purification by flash column chromatography on silica gel (heptanes/EtOAc 20:1) afforded 1-(*o*-tolyl)piperidine as a light yellow oil (1.32 g, 7.5 mmol, 75%).

^{1}H NMR (300 MHz, $CDCl_3$): δ 7.21–710 (m, 2H), 7.01 (d, *J* = 7.5 Hz, 1H), 6.95 (td, *J* = 7.3, 1.4 Hz, 1H), 2.89–2.80 (m, 4H), 2.31 (s, 3H), 1.78–1.65 (m, 4H), 1.64–1.51 (m, 2H).

The physical and spectral data are in accordance with the reported literature data.[7]

1-(*m*-Tolyl)piperidine (6c)

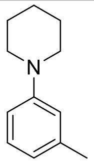

6c
$C_{12}H_{17}N$
MW: 175,2750

Following **General Procedure A** with 3-methylaniline (1.1 mL, 10.0 mmol) and 1,5-dibromopentane (1.5 mL, 11.0 mmol). Purification by flash column chromatography on silica gel (heptanes/EtOAc 20:1 to 15:1) afforded 1-(*m*-tolyl)piperidine as a light yellow oil (941 mg, 5.4 mmol, 54%).

^{1}H NMR (300 MHz, $CDCl_3$): δ 7.20–7.08 (m, 1H), 6.83–6.71 (m, 2H), 6.65 (d, *J* = 7.4 Hz, 1H), 3.18–3.10 (m, 4H), 2.31 (s, 3H), 1.78–1.64 (m, 4H), 1.64–1.50 (m, 2H).

The physical and spectral data are in accordance with the reported literature data.[8]

1-(*p*-Tolyl)piperidine (6d)

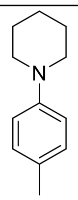

6d
$C_{12}H_{17}N$
MW: 175,2750

Following **General Procedure A** with 4-methylaniline (2.71 g, 25.0 mmol) and 1,5-dibromopentane (3.8 mL, 27.5 mmol). Purification by flash column chromatography on silica gel (heptanes/EtOAc 20:1 to 15:1) afforded 1-(*p*-tolyl)piperidine as a pale-yellow oil (3.48 g, 19.25 mmol, 77%).

^{1}H NMR (300 MHz, $CDCl_3$): δ 7.06 (d, J = 8.3 Hz, 2H), 6.87 (d, J = 8.4 Hz, 2H), 3.14–3.05 (m, 4H), 2.27 (s, 3H), 1.79–1.65 (m, 4H), 1.63–1.49 (m, 2H).

The physical and spectral data are in accordance with the reported literature data.[9]

1-(4-Methoxyphenyl)piperidine (6e)

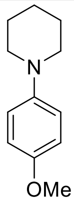

6e
$C_{12}H_{17}NO$
MW: 191,2740

Following **General Procedure A** with 4-methoxyaniline (6.28 g, 50.0 mmol) and 1,5-dibromopentane (7.6 mL, 55.0 mmol). Purification by flash column chromatography on silica gel (heptanes/EtOAc 85:15 to 80:20) afforded 1-phenylpiperidine as a pale-yellow oil (8.58 g, 45 mmol, 90%).

^{1}H NMR (300 MHz, $CDCl_3$): δ 6.95–6.88 (m, 2H), 6.86–6.79 (m, 2H), 3.77 (s, 3H), 3.02 (t, J = 5.5, 4H), 1.72 (p, J = 5.7 Hz, 4H), 1.60–1.49 (m, 2H).

The physical and spectral data are in accordance with the reported literature data.[10]

1-(4-Bromophenyl)piperidine (6f)

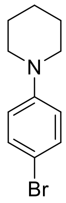

6f
$C_{11}H_{14}BrN$
MW: 240,1440

Following **General Procedure A** with 4-bromoaniline (8.6 g, 50.0 mmol) and 1,5-dibromopentane (7.6 mL, 55.0 mmol). Purification by flash column chromatography on silica gel (pentane/Et_2O 20:1) afforded 1-(4-bromophenyl)piperidine as a crystalline white solid (6.68 g, 28 mmol, 56%).

^{1}H NMR (300 MHz, $CDCl_3$): δ 7.35–7.28 (m, 2H), 6.83–6.75 (m, 2H), 3.17–3.07 (m, 4H), 1.74–1.64 (m, 4H), 1.65–1.50 (m, 2H).

The physical and spectral data are in accordance with the reported literature data.[11]

Methyl 4-(1-piperidyl)benzoate (6g)

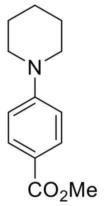

6g
$C_{13}H_{17}NO_2$
MW: 219,2840

Following **General Procedure A** with methyl 4-aminobenzoate (3.86 g, 25.0 mmol) and 1,5-dibromopentane (3.8 mL, 27.5 mmol). Purification by flash column chromatography on silica gel (heptanes/EtOAc 95:5) afforded methyl 4-(1-piperidyl)benzoate as a shiny white solid (1.20 g, 9.5 mmol, 38%).

^{1}H NMR (300 MHz, $CDCl_3$): δ 7.95–7.86 (m, 2H), 6.88 (m, 2H), 3.86 (s, 3H), 3.33 (t, J = 5.2 Hz, 4H), 1.77–1.55 (m, 6H).

The physical and spectral data are in accordance with the reported literature data.[9]

1-[4-(1-Piperidyl)phenyl]ethanone) (6h)

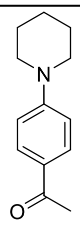

6h
$C_{13}H_{17}NO$
MW: 203,2850

Following **General Procedure A** with 1-(4-aminophenyl)ethanone (3.4 g, 25.0 mmol) and 1,5-dibromopentane (3.8 mL, 27.5 mmol). Purification by flash column chromatography on silica gel (heptanes/EtOAc 90:10 to 80:20) followed by trituration in pentane afforded 1-[4-(1-piperidyl)phenyl]ethenone) as a crystalline pale-yellow solid (1.51 g, 7.5 mmol, 30%).

^{1}H NMR (300 MHz, $CDCl_3$): δ 7.91–7.80 (m, 2H), 6.91–6.79 (m, 2H), 3.36 (m, 4H), 2.51 (s, 3H), 1.74–1.61 (m, 6H).

The physical and spectral data are in accordance with the reported literature data.[9]

1-[4-(trifluoromethyl)phenyl]piperidine (6i)

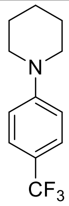

6i
$C_{12}H_{14}F_3N$
MW: 229,2462

Following **General Procedure A** with 4-(trifluoromethyl)aniline (3.2 mL, 25.0 mmol) and 1,5-dibromopentane (3.8 mL, 27.5 mmol). Purification by flash column chromatography on silica gel (pentane/Et_2O 20:1) afforded 1-[4-(trifluoromethyl)phenyl]piperidine as a colorless oil (602 mg, 2.63 mmol, 11%).

^{1}H NMR (300 MHz, $CDCl_3$): δ 7.45 (d, J = 8.8 Hz, 2H), 6.91 (d, J = 8.7 Hz, 2H), 3.27 (t, J = 5.6 Hz, 4H), 1.76–1.54 (m, 6H). ^{19}F NMR (282 MHz, $CDCl_3$): δ –61.2.

The physical and spectral data are in accordance with the reported literature data.[9]

1-[4-(3,3,4,4-Tetramethyl-1lambda3,2,5-bromadioxolan-1-yl)phenyl]piperidine (6j)

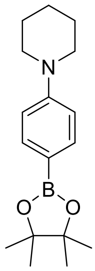

6j
$C_{17}H_{26}BNO_2$
MW: 287,2100

Synthesized following a reported procedure.[3]

In a two-neck round-bottomed flask equipped with a reflux condenser was added 1-(4-bromophenyl)piperidine **6f** (3.60 g, 15.0 mmol) and degassed 1,4-dioxane (120 mL). Bis(pinacolato)diboron (B_2Pin_2) (5.71 g, 22.5 mmol), KOAc (4.42 g, 45 mmol) and $PdCl_2$(dppf)-CH_2Cl_2 (549 mg, 5 mol%, 0.75 mmol) were added to the solution and the reaction mixture was stirred at 90 °C for 14 h. The reaction mixture was allowed to cool down to rt and was filtered through a pad of celite (washing with EtOAc). Volatile organic solvents were evaporated *in vacuo* to give crude product. Purification by flash column chromatography on silica gel (pentane/Et_2O 20:1) afforded title compound as a white crystalline solid (1.82 g, 6.3 mmol, 42%).

Alternative procedure:

In a two-neck flask equipped with a magnetic stirring bar and a reflux condenser were introduced 4-(4,4,5,5-tetramethyl-1,3,2-dioxaborolan-2-yl)aniline (1.11 g, 5.00 mmol), N,N-dimethylformamide (5 mL), diisopropylethylamine (DIPEA) (2.56 mL, 15.0 mmol), and 1,4-dibromopentane (0.77 mL, 5.50 mmol). The reaction mixture was heated to 80 °C and stirred overnight at the same temperature. Upon completion (TLC monitoring), the reaction mixture was diluted with Et_2O (20 mL). Water (10 mL) was added and the phases were separated. The organic phase was washed with water (3 x 10 mL). The

organic phase was dried with Na_2SO_4, filtered and concentrated under reduced pressure to give crude product. Purification by flash column chromatography on silica gel (pentane:Et_2O 90:10) afforded the desired product as a white crystalline solid (1.09 g, 3.8 mmol, 76%).

^{1}H NMR (300 MHz, $CDCl_3$): δ 7.73–7.64 (m, 2H), 6.94–6.83 (m, 2H), 3.30–3.20 (m, 4H), 1.74–1.56 (m, 6H), 1.32 (s, 12H). ^{11}B NMR (96 MHz, $CDCl_3$): δ 30.6. ^{13}C NMR (75 MHz, $CDCl_3$): δ 154.1 (Cq), 136.2 (2xCH_{Ar}), 114.7 (2xCH_{Ar}), 83.4 (2xCq), 49.6 (2xCH_2), 25.7 (2xCH_2), 25.0 (4xCH_3), 24.6 (CH_2). Due to coupling to the quadrupolar ^{11}B and ^{10}B nuclei, the aromatic carbon atom bearing the BPin was not detected. FT-IR (neat, cm^{-1}): 2976, 2934, 2846, 1604, 1360, 1238, 1141, 1127, 1090, 860, 819, 654. HRMS (ESI) m/z: [M+H]$^+$ Calcd for $C_{17}H_{27}O_2NB$ 288.2129; found 288.2125. mp 96.2–97.1 °C. R_f = 0.43 (pentane/Et_2O 99:1).

tert-Butyl 4-phenylpiperazine-1-carboxylate (6l)

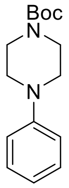

6l
$C_{15}H_{22}N_2O_2$
MW: 262,3530

To a solution of Boc_2O (1.6 mL, 7.00 mmol) and DMAP (122 mg, 1.00 mmol) in DCM (15 mL) were added dropwise 1-phenylpiperazine (0.8 mL, 5.00 mmol) and triethylamine (0.73 mL, 5.25 mmol). The resulting reaction mixture was stirred at rt for 2 h. Water (15 mL) was added. The phases were separated and the aqueous layer was extracted with DCM (3 x 20 mL). The combined organic layers were dried with Na_2SO_4 and concentrated under reduced pressure to give crude product as a yellow solid. The residue was purified by flash column chromatography on silica gel (heptanes/EtOAc 90:10) to give product as a white solid (1.17 g, 4.45 mmol, 89%).

^{1}H NMR (300 MHz, $CDCl_3$): δ 7.32–7.23 (m, 2H), 6.90 (m, 3H), 3.58 (t, *J* = 5.0 Hz, 4H), 3.13 (t, *J* = 5.3 Hz, 4H), 1.48 (s, 9H).

The physical and spectral data are in accordance with reported the literature data.[12]

4-Phenylthiomorpholine (6m)

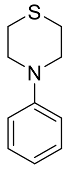

6f
$C_{10}H_{13}NS$
MW: 179,2810

In a flame-dried two-neck flask equipped with a reflux condenser was added K_2CO_3 (2.76 g, 20.0 mmol), CuI (190 mg, 1.00 mmol), L-proline (230 mg, 2.00 mmol) and dry DMSO (22 mL). Then, iodobenzene (1.12 mL, 10.0 mmol) and thiomorpholine (1.56 mL, 15.0 mmol) were added *via* a syringe. The reaction mixture was vigorously stirred at 100 °C for 5 hours, before being allowed to cool down to rt. Water (60 mL) and EtOAc (60 mL) were added. The phases were separated and aqueous phase was extracted with EtOAc (2 x 30 mL). The combined organic phases were washed with brine (1 x 50 mL), dried over Na_2SO_4, and concentrated under reduced pressure to give crude product as a brown oil. Purification by flash column chromatography on silica gel (pentane/Et_2O 95:5) afforded the title compound as a pale-yellow oil that became a white solid upon storage at 4 °C (912 mg, 5.1 mmol, 51%).

^{1}H NMR (300 MHz, $CDCl_3$): δ 7.34–7.21 (m, 2H), 6.92–6.84 (m, 3H), 3.59–3.50 (m, 4H), 2.81–2.70 (m, 4H).

The physical and spectral data are in accordance with the reported literature data.[13]

1-Phenylazepane (6n)

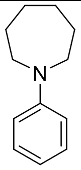

6n
$C_{12}H_{17}N$
MW: 175,2750

Following **General Procedure A** with aniline (2.3 mL, 25.0 mmol) and 1,6-dibromohexane (4.3 mL, 27.5 mmol). Purification by flash column chromatography on silica gel (heptanes/EtOAc 97:3 to 95:5) afforded 1-phenylazepane as a colorless oil (608 mg, 3.25 mmol, 13%). The product is suspected to decompose upon prolonged reaction time.

^{1}H NMR (300 MHz, $CDCl_3$): *δ* 7.25–7.15 (m, 2H), 6.69 (d, *J* = 8.2 Hz, 2H), 6.63 (t, *J* = 7.3 Hz, 1H), 3.48 (t, *J* = 5.9 Hz, 4H), 1.94–1.73 (m, 4H), 1.67–1.46 (m, 4H).

The physical and spectral data are in accordance with the reported literature data.[5]

Methyl 4-(azepan-1-yl)benzoate (6o)

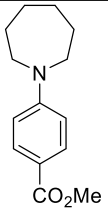

6o
$C_{14}H_{19}NO_2$
MW: 233,3110

Following **General Procedure A** with methyl 4-aminobenzoate (1.53 g, 10.0 mmol) and 1,6-dibromohexane (1.7 mL, 11.0 mmol) for 2h45 (Caution: the product decomposes upon prolonged reaction time and reaction must be stopped before complete conversion of starting aniline). Purification by flash column chromatography on silica gel (heptanes/EtOAc 90:10 to 70:30) afforded 1-phenylazepane as a shiny white solid (587 mg, 2.4 mmol, 24%).

^{1}H NMR (300 MHz, $CDCl_3$) *δ* 7.90–7.81 (m, 2H), 6.62–6.51 (m, 2H), 3.85 (s, 3H), 3.41 (t, *J* = 6.7 Hz, 2H), 3.17 (t, *J* = 7.1 Hz, 2H), 1.95–1.80 (m, 2H), 1.71–1.60 (m, 2H), 1.57–1.36 (m, 4H).

The physical and spectral data are in accordance with the reported literature data.[14]

Preparation of radical traps

Ethyl 2-(benzenesulfonylmethyl)prop-2-enoate

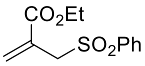

$C_{12}H_{14}O_4S$
MW: 254,3000

Synthesized following a known literature procedure.[15]

Ethyl 2-methylprop-2-enoate (5.02 mL, 40.0 mmol) and absolute ethanol (80 mL) were added to a 250-mL, three-necked, round-bottomed flask equipped with a thermometer, a nitrogen inlet, and a septum. The mixture was cooled to 0 °C and I_2 (12.0 g, 47.3 mmol) and benzenesulfinic acid sodium salt (13.8 g, 84.1 mmol) were then added. The reaction mixture was stirred at 0 °C for 5 h, then allowed to warm-up to room temperature. The reaction mixture was partitioned between DCM (120 mL) and water (100 mL). The phases were separated (use a flashlight to see the phase separation) and the organic phase was washed with water (2 x 100 mL). The combined aqueous layers were extracted with DCM (2 x 25 mL). The combined organic phases were washed with saturated $NaHCO_3$ (2 x 100 mL) and with 5% $Na_2S_2O_3$ (2 x 100 mL) then dried over Na_2SO_4, filtered and concentrated to give crude intermediate as a thick orange oil. The crude intermediate was diluted with DCM (50 mL). Freshly distilled triethylamine (11.0 mL, 78.9 mmol) was added dropwise over 10 minutes and the reaction mixture was stirred overnight at rt. The reaction mixture was concentrated *in vacuo*

until half volume, then directly loaded on the column including precipitated solids. Elution with heptanes/TBME (80:20 to 40:60) afforded the title compound as a pale-yellow thick oil (8.00 g, 31.6 mmol, 79%).

^{1}H NMR (300 MHz, $CDCl_3$): δ 7.90–7.80 (m, 2H), 7.64 (m, 1H), 7.59–7.47 (m, 2H), 6.50 (d, J = 0.6 Hz, 1H), 5.90 (q, J = 0.8, 0.6 Hz, 1H), 4.15 (d, J = 0.8 Hz, 2H), 4.00 (q, J = 7.1 Hz, 2H), 1.16 (t, J = 7.1 Hz, 3H).

The physical and spectral data are in accordance with the reported literature data.[15]

Ethyl 2-(hydroxymethyl)prop-2-enoate

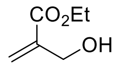

$C_6H_{10}O_3$
MW: 130,1430

To a solution of paraformaldehyde (95.0 %, 4.1 g, 130 mmol) and ethyl prop-2-enoate (11.0 mL, 100 mmol) in 80 mL dioxane-water (1:1, v/v) was added DABCO (1.49 g, 130 mmol) and the reaction was stirred at room temperature overnight. The reaction mixture was partitioned between EtOAc (400 mL) and water (200 mL). The organic layer was separated and washed with brine (1 x 200 mL), dried over anhydrous Na_2SO_4 and concentrated under reduced pressure to give crude product. Purification by flash column chromatography on silica gel (heptanes/EtOAc 70:30 to 50:50) afforded the title compound as a pale-yellow oil (6.1 g, 47 mmol, 47%). The compound can be stored in the fridge at +4 °C for several months.

^{1}H NMR (300 MHz, $CDCl_3$): δ 6.23 (q, J = 1.0 Hz, 1H), 5.81 (q, J = 1.4 Hz, 1H), 4.30 (m, 2H), 4.22 (q, J = 7.1 Hz, 2H), 2.56 (bs, 1H), 1.29 (t, J = 7.1 Hz, 3H). The physical and spectral data are in accordance with the reported literature data.[16]

Ethyl 3-hydroxy-2-methylene-butanoate

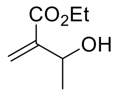

$C_7H_{12}O_3$
MW: 144,1700

In a seal tube were successively added DABCO (3.37 g, 30 mmol), ethyl prop-2-enoate (16.0 mL, 150 mmol) and acetaldehyde (4.2 mL, 75 mmol). The neat reaction mixture was stirred at room temperature for 4 days and was then diluted with Et_2O (300 mL). The organic phase was washed with water (2x 150 mL), brine (1 x 100 mL), dried over anhydrous Na_2SO_4 and concentrated under reduced pressure to give crude product as a yellow liquid (8.43 g, 58.5 mmol, 78%) which was sufficiently pure for the acetylation step.

^{1}H NMR (300 MHz, $CDCl_3$): δ 6.24–6.18 (m, 1H), 5.84–5.76 (m, 1H), 4.67–4.56 (m, 1H), 4.25 (q, J = 7.1 Hz, 2H), 2.70 (bs, 1H, hydroxyl proton), 1.39 (d, J = 6.4 Hz, 3H), 1.32 (t, J = 7.2 Hz, 3H).

The physical and spectral data are in accordance with the reported literature data.[17]

Ethyl 2-[(2,2,2-trifluoroacetyl)oxymethyl]prop-2-enoate

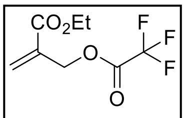

$C_8H_9F_3O_4$
MW: 226,1512

To a stirred solution of ethyl 2-(hydroxymethyl)prop-2-enoate (1.32 g, 10.0 mmol) and 2,6-lutidine (1.28 mL, 11.0 mmol) at 0 °C and under N_2 was added dropwise trifluoroacetic anhydride (1.39 mL, 10.0 mmol). Upon completion of the reaction (TLC monitoring), the reaction mixture was directly subjected to a distillation which

afforded unclean product. Further purification by flash column chromatography on silica gel (heptanes/EtOAc 90:10) afforded clean title compound as a colorless oil (966 mg, 4.7 mmol, 47%).

^{1}H NMR (300 MHz, $CDCl_3$): δ 6.48 (d, J = 0.8 Hz, 1H, C=$\underline{CH_2}$), 5.93 (q, J = 1.2 Hz, 1H, C=$\underline{CH_2}$), 5.06 (m, 2H, $\underline{CH_2}OCOCF_3$), 4.26 (q, J = 7.1 Hz, 2H, $CO_2\underline{CH_2}CH_3$), 1.31 (t, J = 7.1 Hz, 3H, $CO_2CH_2\underline{CH_3}$). ^{13}C NMR (75 MHz, $CDCl_3$): δ 164.6 ($\underline{C}O_2Et$), 157.1 (q, $^2J_{C\text{–}F}$ = 42.7 Hz, F_3C-$\underline{C=O}$), 133.8 ($\underline{C}=CH_2$), 129.5 (C=$\underline{C}H_2$), 114.6 (q, $^1J_{C\text{–}F}$ = 285.8 Hz, $F_3\underline{C}$), 65.7 ($\underline{C}H_2OCOCF_3$), 61.5 ($CO_2\underline{C}H_2CH_3$), 14.2 ($CO_2CH_2\underline{C}H_3$). ^{19}F NMR (282 MHz, $CDCl_3$): δ –75.0. FT-IR (cm^{-1}, neat): 1787, 1718, 1220, 1132, 1025, 774, 732, 522. HRMS (ESI) m/z: $[M+Na]^+$ Calcd for $C_8H_8O_4F_3Na$ 249.0345; Found 249.0346. R_f = 0.37 (heptanes/EtOAc 90:10).

Ethyl 2-(bromomethyl)prop-2-enoate

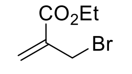

$C_6H_9BrO_2$
MW: 193,0400

Phosphorus tribromide (0.72 mL, 7.53 mmol) was added dropwise to a solution of ethyl 2-(hydroxymethyl)prop-2-enoate (2.80 g, 21.5 mmol) in dry Et_2O (20 mL) at –10°C and under N_2,. The reaction mixture was allowed to warm up to rt and stirred at this temperature for 1h30. Water (10 mL) was added and the mixture was extracted with Et_2O (3 x 50 mL). The organic phase was washed with brine (1 x 50 mL), dried over Na_2SO_4 and concentrated under reduced pressure to give crude product. Purification by flash column chromatography on silica gel (heptanes/EtOAc 90:10) afforded the title compound as a pale-yellow oil (3.44 g, 17.85 mmol, 83%).

^{1}H NMR (300 MHz, $CDCl_3$): δ 6.33 (d, J = 0.8 Hz, 1H), 5.94 (q, J = 0.9 Hz, 1H), 4.27 (q, J = 7.1 Hz, 2H), 4.18 (d, J = 0.9 Hz, 2H), 1.33 (t, J = 7.1 Hz, 3H).

The physical and spectral data are in accordance with the reported literature data.[16]

Ethyl 2-(1,1-dimethyldecylsulfanylmethyl)prop-2-enoate

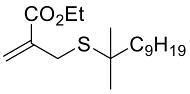

$C_{18}H_{34}O_2S$
M W: 314,5280

In a 50-mL oven-dried flask under N_2 was added sodium methoxide (341 mg, 6.00 mmol) and dry methanol (25 mL). 2-Methylundecane-2-thiol (mixture of isomers, 1.19 mL, 5.00 mmol) was then added dropwise and the mixture was stirred at rt for 1h. Then, ethyl 2-(bromomethyl)prop-2-enoate (1.06 g, 5.50 mmol) was added and the mixture was stirred 6 hour at rt. At this point a second more polar spot appeared and the reaction was stopped before completion. Water (25 mL) was added and the mixture was extracted with Et_2O (3 x 50 mL) The combined organic phases were dried over anhydrous Na_2SO_4, filtered and concentrated to give crude product. Purification by flash column chromatography on silica gel (heptanes/EtOAc 98:2) afforded the title compound as a pale-yellow oil (mixture of isomers, 0.55 g, 1.75 mmol, 35%).

^{1}H NMR (300 MHz, $CDCl_3$): δ 6.18 (m, 1H), 5.76 (m, 1H), 4.19 (q, J = 7.1 Hz, 2H), 3.42–3.21 (m, 2H), 1.58–1.02 (m, 17H), 0.94–0.76 (m, 12H). ^{13}C NMR (75 MHz, $CDCl_3$): δ 166.4, 138.1, 126.6, 61.0, 32.6, 29.5, 28.8, 22.8, 14.3, 8.8. FT-IR (neat, cm^{-1}): 2957, 2929, 2871, 1716, 1632, 1462, 1323, 1182, 1126, 1026, 942, 810. HRMS (ESI) m/z: $[M+H]^+$ Calcd for $C_{18}H_{35}O_2S$ 315.2352; Found 315.2347. R_f = 0.32 (heptanes/EtOAc 94:6).

General Procedure B: acetylation of allylester-alcohols

To a solution of allylester-alcohol (10 mmol, 1 equiv.) in dry DCM (40 mL) cooled to 0 °C was added pyridine (1.6 mL, 20 mmol, 2 equiv.) and then slowly the acyl chloride derivative (20 mmol, 2 equiv.). The reaction mixture was stirred at rt until completion (TLC monitoring). The reaction mixture was taken up in Et_2O (200 mL) and successively washed with 2M HCl (2 x 100 mL), sat. $NaHCO_3$ (2 x 100 mL) and brine (1 x 100 mL). The organic layer was dried over anhydrous Na_2SO_4 and concentrated under reduced pressure to give crude product as a yellow liquid essentially analytically clean. A short purification by flash column chromatography on silica gel afforded pure product.

Ethyl 2-(acetoxymethyl)prop-2-enoate

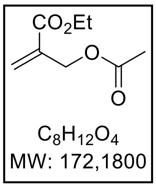

Synthesized following **General Procedure B** with ethyl 2-(hydroxymethyl)prop-2-enoate (6.10 g, 46.9 mmol) and acetyl chloride (6.80 mL, 93.7 mmol) reacting for 1h30. Purification by flash column chromatography on silica gel (heptanes/EtOAc 80:20) afforded the title compound as a colorless liquid (7.03 g, 40.8 mmol, 87%).

^{1}H NMR (300 MHz, $CDCl_3$): δ 6.35 (q, J = 1.0 Hz, 1H), 5.83 (q, J = 1.4 Hz, 1H), 4.81 (m, 2H), 4.24 (q, J = 7.1 Hz, 2H), 2.10 (s, 3H), 1.30 (t, J = 7.1 Hz, 3H).

The physical and spectral data are in accordance with the reported literature data.[18]

2-Ethoxycarbonylallyl 2,2-dimethylpropanoate

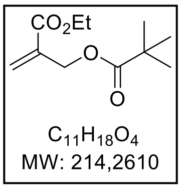

Synthesized following **General Procedure B** with ethyl 2-(hydroxymethyl)prop-2-enoate (0.65 g, 5.00 mmol) and pivaloyl chloride (0.73 mL, 10.0 mmol) reacting for 16 h. Purification by flash column chromatography on silica gel (pentane/Et_2O 90:10) afforded the title compound as a colorless liquid (912 mg, 4.25 mmol, 85%).

^{1}H NMR (300 MHz, $CDCl_3$): δ 6.34 (q, J = 1.1 Hz, 1H), 5.79 (q, J = 1.5 Hz, 1H), 4.79 (dd, J = 1.6, 1.1 Hz, 2H), 4.23 (q, J = 7.1 Hz, 2H), 1.30 (t, J = 7.1 Hz, 3H), 1.22 (s, 9H). ^{13}C NMR (75 MHz, $CDCl_3$): δ 178.0 (C=O), 165.4 (C=O), 136.0 (Cq), 126.6 (CH_2), 62.5 (CH_2), 61.1 (CH_2), 39.0 (Cq), 27.3 (3xCH_3), 14.3 (CH_3). FT-IR (neat, cm^{-1}): 2976, 2874, 1725, 1304, 1270, 1132, 1031. HRMS (ESI) m/z: $[M+H]^+$ Calcd for $C_{11}H_{19}O_4$ 215.1278; Found 215.1276. R_f = 0.63 (pentane/Et_2O 90:10).

Ethyl 3-acetoxy-2-methylene-butanoate

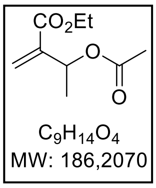

Synthesized following **General Procedure B** with ethyl 3-hydroxy-2-methylene-butanoate (1.84 g, 12.8 mmol) and acetyl chloride (1.1 mL, 15.3 mmol; Comment: 1.2 eq were used instead of the usual 2.0 eq) reacting for 60 minutes. Purification by flash column chromatography on silica gel (heptanes/EtOAc 90:10) afforded the title compound as a colorless oil (1.89 g, 10.2 mmol, 80%).

^{1}H NMR (300 MHz, $CDCl_3$): δ 6.28–6.25 (br s, 1H), 5.79 (t, J = 1.2 Hz, 1H), 5.70 (br q, J = 6.6 Hz, 1H), 4.22 (q, J = 7.1 Hz, 2H), 2.06 (s, 3H), 1.39 (d, J = 6.5 Hz, 3H), 1.29 (t, J = 7.1 Hz, 3H). ^{13}C NMR (75 MHz, $CDCl_3$):

δ 169.9 (C=O), 165.4 (C=O), 141.5 (Cq), 124.5 (CH_2), 68.3 (CH), 61.0 (CH_2), 21.3 (CH_3), 20.3 (CH_3), 14.3 (CH_3). FT-IR (neat, cm^{-1}): 2984, 1715, 1635, 1369, 1232, 1174, 1079, 1040, 952, 814. HRMS (ESI) m/z: $[M+Na]^+$ Calcd for $C_9H_{14}O_4Na$ 208.0784; Found 209.0781. R_f = 0.33 (heptanes/EtOAc 90:10).

Optimization of the one-pot procedure

All reactions were performed on 0.2 mmol scale. NMR yields were measured using 0.5 equivalent of ethylene carbonate as internal standard. 2,5-Bis-allylated product **4a** and dimer **5a** were identified as the major undesired products of the reaction and it was sought to suppress their formation.

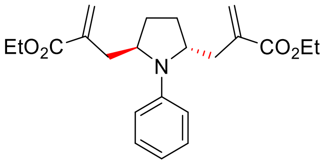

4a

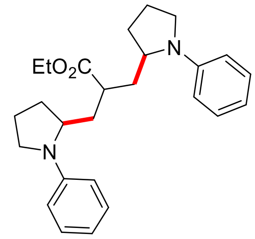

5a

Supplementary Table 1.
Allyl sulfone trap and [Ir{dF(CF$_3$)ppy}$_2$(dtbpy)]PF$_6$: screening of equivalents and preliminary solvent screening

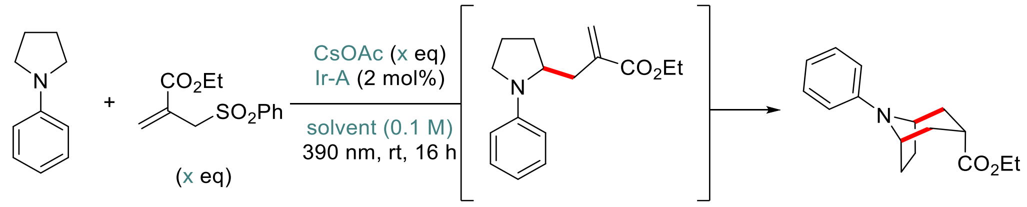

1a **2a** **3a**

Ir-A = [Ir{dF(CF$_3$)ppy}$_2$(dtbpy)]PF$_6$

Entry	Solvent	Eq. CsOAc	Eq. trap	Cat. loading	NMR yield	Isolated yield
1	DCE	3.0	1.2	2 mol%	45%	
2	MeCN	3.0	1.2	2 mol%	15%	
3	DCE	2.0	1.2	2 mol%	55%	
4	DCE	2.0	3.0	2 mol%	Traces[a]	
5	DCE	1.2	1.2	2 mol%	59%	51%
6	Acetone	1.2	1.2	2 mol%	33%	
7	MeCN	1.2	1.2	2 mol%	29%	
8	EtOAc	1.2	1.2	2 mol%	41%	
9	DCE	1.2	1.4	2 mol%	75%	40%
10	DCE	1.2	1.4	2 mol%	61%	42%
11	DCE	1.2	1.4	2 mol%	59%	47%
12	DCE	1.2 (wet)	1.4	2 mol%	46%	
13	DCE	1.2	1.4	2 mol%	51%	
14	DCE	1.2	1.4	2 mol%	54%	
15	DCE	1.0	1.0	2 mol%	31%	
16	DCE	1.0	1.0	1 mol%	n.d.	

[a] 30% of **4a** was observed

Supplementary Table 2
Catalyst screening

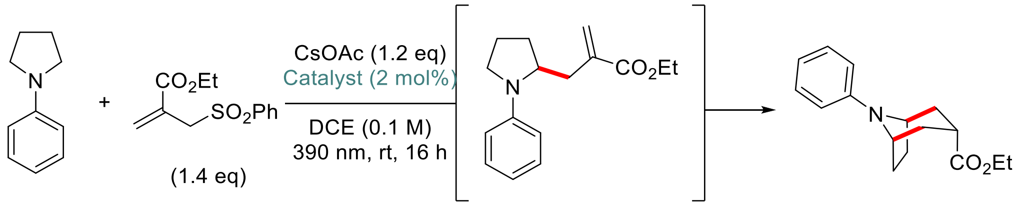

1a **2a** **3a**

Entry	Catalyst (CAS)	Ir(III*/II) (V vs SCE in MeCN)	Ir(III/II) (V vs SCE in MeCN)	NMR yield of **2a**	NMR yield of **3a**
1	870987-63-6 (Ir-A)	+1.21	–1.37	–	65%
2	1072067-44-7 (**Ir-C**)	+1.10	–1.74	–	50%
3	676525-77-2 (**Ir-B**)	+0.66	–1.51	–	47%
4	1092775-62-6	+0.97	–1.23	–	43%
5	1335047-34-1	+0.97	–1.43	–	42%
6	60804-74-2 (**Ru-A**)	Ru(II*/I) = +0.77	Ru(II/I) = –1.33	30%	30%
7	1416881-51-1	+1.35	–1.21	31%	21%
8	387859-70-3	+0.36	–1.87	45%	–
9[a]	Eosin Y	+0.83	–1.06	15%[b]	–
10[a]	Fluorescein	+0.78	–1.27	6%	–

[a] Reactions performed under 525 nm Kessil lamps. [b] 51% for the reaction carried out in DMF

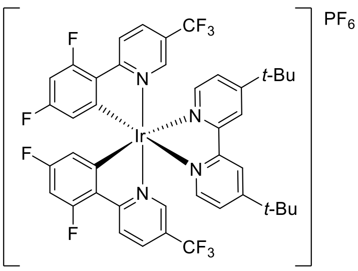

Ir-A
Ir{dF(CF_3)ppy}$_2$(dtbpy)]PF_6

CAS: 870987-63-6

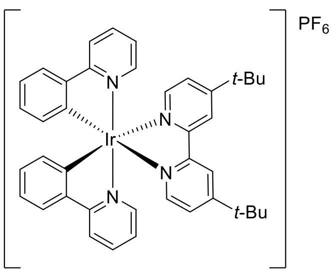

Ir-B
[Ir(dtbbpy)(ppy)$_2$]PF_6

CAS: 676525-77-2

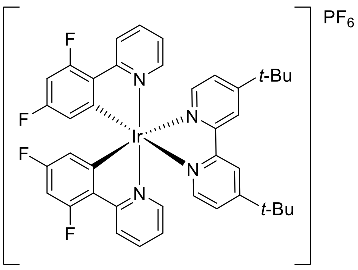

Ir-C
[Ir(dFppy)$_2$(dtbbpy)]PF_6

CAS: 1072067-44-7

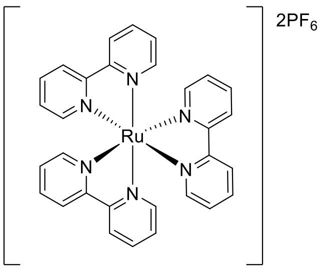

Ru-A
Ru(bpy)$_3$(PF_6)$_2$

CAS: 60804-74-2

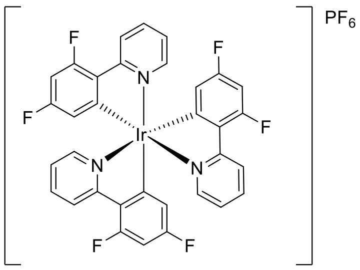

Ir(dFppy)$_3$

CAS: 387859-70-3

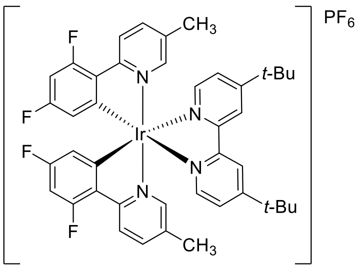

[Ir(dF(Me)ppy)$_2$(dtbbpy)]PF_6

CAS: 1335047-34-1

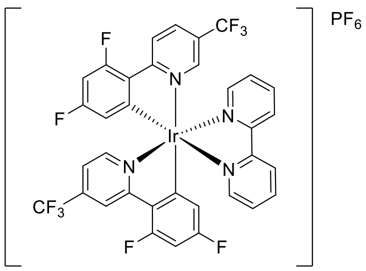

[Ir{dFCF_3ppy}$_2$(bpy)]PF_6

CAS: 1092775-62-6

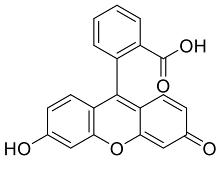

Fluorescein
CAS: 2321:07-5

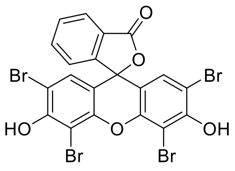

Eosin Y
CAS: 15086-94-9

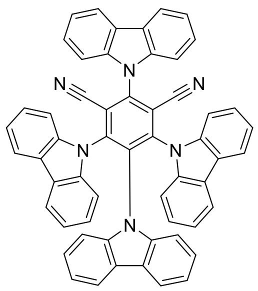

4CzIPN
1,2,3,5-Tetrakis(carbazol-9-yl)-4,6-dicyanobenzene
CAS: 1416881-51-1

Supplementary Table 3
Allyl sulfone trap and [Ir(dtbbpy)(ppy)2]PF6. Base and solvent screening

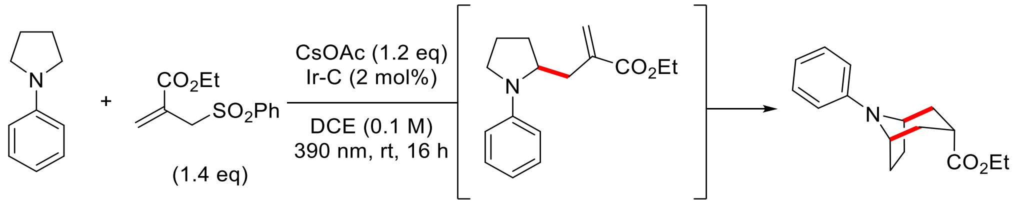

1a **2a** **3a**

Entry	Deviation to standard conditions	NMR yield of 3a	other
**1**	None	47±3%[a]	
**2**	1h45	–	37% of **2a**[b]
**3**	CsOAc (2.0 equiv)	47%	
**4**	DCE passed over SiO_2	51%	
**5**	DCE not dried	49%	
**6**	0.05 M	47%	
**7**	0.5 M	38%	
**8**	NaOAc (1.2 equiv)	-	15–26% of **2a**
**11**	K_2CO_3 (1.2 equiv)	30±15%[c]	
**12**	Na_2CO_3 (1.2 equiv)	12%[b]	
**14**	Cs_2CO_3 (1.2 equiv)	-	31% of **2a**
**15**	CsOAc (0.5 equiv)	39%	
**16**	DMSO	-	-
**18**	DMF	-	14% of **5a**[c]
**19**	DMA	-	-
**20**	DCM	50%	
**21**	$CHCl_3$	-	-
**22**	**1a** (2.0 equiv), Allylsulfone (1.0 equiv)	14% (isolated)	28% (isolated yield) of **5a**

[a] Average of 3 runs. [b] Isolated yield. [c] Isolated yield. Not reproducible.

Supplementary Table 4

Allyl acetate trap and [Ir{dF(CF$_3$)ppy}$_2$(dtbpy)]PF$_6$: Final optimization

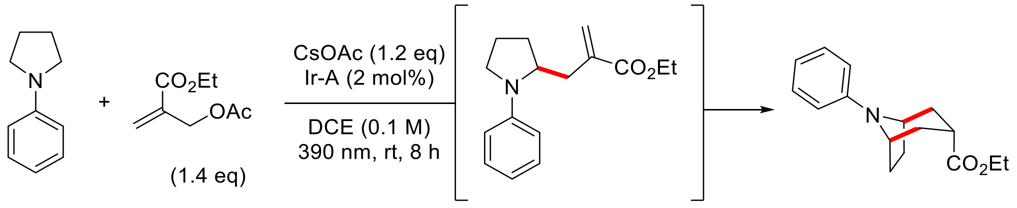

1a **2a** **3a**

Ir-A = [Ir{dF(CF$_3$)ppy}$_2$(dtbpy)]PF$_6$

Entry	Deviation to standard conditions	yield 3a NMR	yield 3a isolated	Other remarks
**1**	Allylsulfone trap	65%		
**2**	None	-	46±2%, dr 7:1[b]	**4a** not detected[a]
**3**	Allylbromide trap	-		66% NMR yield of **2a**
**4**	Allylsulfide	77%	~51% isolated, dr 5:1	Difficult separation
**5**	Eosin Y (5 mol%, 525 nm)	-		Traces of **2a**
**6**	2 h		41%, dr 7:1	
**7**	20 min	44%		Full conv. within 20 min
**8**	5 min	-	-	50% NMR yield of **2a**
**9**	20 min, Ir cat. 1 mol%	49%	37%, dr 7:1	**4a** (10%, isolated yield)[a]
**10**	15 min, 1.0 eq trap	44%	36%, dr 8:1	
**11**	10 min	52%	43%, dr 7:1	9% (isolated yield) of **4a**[a]
**In further experiments, reaction time was lowered to 20 min for all reactions**				
**12**	1.0 eq trap, 0.5 eq base		14%	4% of **5a**
**13**	0.5 eq base	51%	39%	5% of **5a**
**14**	0.05 M	67%	52%, dr 9:1	
**15**	0.025 M	64%	58%, dr 8:1	
**16**	0.05 M, 1 mol% cat	-	58±4 %, dr 7:1[b]	
**17**	0.05 M, 0.5 mol% cat	-	55%, dr 8:1	

Entry	Deviation to standard conditions	NMR yield of 3a	Isolated yield of 3a	Other remarks
**In further experiments, reaction molarity was set to 0.05 M and catalyst loading to 1 mol%**				
**18**	DCM	-	59%, dr 10:1	
**19**	CF_3-C_6H_5	-	50%, dr 7:1	
**20**	AcOH 1.0 eq	-	51%, dr 9:1	
**21**	AcOH 0.5 eq at t0	-	52%, dr 7:1	
**22**	AcOH 0. 5eq at t+5min	-	52%, dr 7:1	
**23**	Trap 1.0 eq, AcOH 1.0 eq	-	41%, dr 7:1	
**24**	CsPiv	-	51%, dr 5:1	
**25**	AcOH 1.0 eq at t0, then 0.5 eq over 10 min	56%	49%, dr 10:1	
**26**	AcOH 1.0 eq, trap 3.0 eq	-	31%	18% of **4a**
**27**	Trap 1.1 eq	-	57%, dr 7:1	No **4a** detected
**28**	Trap 1.2 eq	-	56%, dr 7:1	No **4a** detected
**29**	Trap 1.3 eq	-	52%, dr 7:1	**4a** detected
**30**	Trap 1.5 eq	-	-	Messy crude
**31**	Trap 1.1 eq, 0.025 M	-	56%, dr 7:1	
**32**	Trap 1.1 eq, 0.01 M	-	54%, dr 7:1	
**33**	Trap 1.1 eq, 2.0 eq. CsOAc	-	53%, dr 7:1	
**34**	Trap 1.1 eq, 3.0 eq. CsOAc	-	49%, dr 7:1	
**35**	Trap 1.1 eq., Y = OTFA	-	27%, dr 7:1	2h20 reaction time
**36**	Trap 1.1 eq., Y = OPiv	-	45%, dr 5:1	
**Control experiments**				
**37**	No catalyst	–	–	20 min reaction time
**38**	No catalyst	–	–	6 h reaction time. Partial decomposition of SM and trap
**39**	No light	–	–	
**40**	No base	–	–	

[a] It was showed that under the reaction conditions, undesired product **4a** is able to cyclize. This explains why **4a** was observed after 20 minutes, but not after 8 hours of reaction. [b]average of three runs.

One-pot synthesis of the bicyclic products

General Procedure C (0.2 mmol scale)

In an oven-dried 10 mL-vial were successively added the radical trap (0.22 mmol, 1.1 equiv.), the aniline derivative, if solid or oily (0.20 mmol, 1 equiv.), [Ir{dF(CF_3)ppy}$_2$(dtbpy)]PF_6 (2.2 mg, 0.02 mmol, 1 mol%) and cesium acetate (46 mg, 0.24 mmol, 1.2 equiv.). The vial was closed with a rubber septum and evacuated/refilled with N_2 (x 3). If liquid, the aniline starting material (0.20 mmol, 1 equiv.) was then added *via* a Hamilton syringe. Finally, dry and degassed 1,2-dichloroethane (4.00 mL, 0.05 M) was added. The resulting yellow mixture was placed in front of a 390 nm blue LED (5 cm distance) and stirred until completion of the reaction (TLC monitoring). Reactions usually turned orange and a fine white precipitate was observed. The reaction mixture was diluted with sat. $NaHCO_3$ (3 mL). The aqueous phase was extracted with DCM (3 x 5 mL) and the combined organic phases were dried over Na_2SO_4, filtered, and concentrated under reduced pressure to give crude product as an orange oil. Purification by flash column chromatography on neutral Alox or SiO_2 afforded the bicyclic product.

Modified general procedure C for the synthesis of electron-rich derivatives (0.2 mmol):

Cesium acetate was replaced by the slightly more basic cesium pivalate (56.2 mg, 0.24 mmol, 1.2 equiv.). All other parameters unchanged.

Note: for most characterized compounds, the major (α) diastereoisomer is slightly more apolar than the minor (β) diastereoisomer. It is usually possible, with great care, to partially or fully separate them; this was done only if characterization of the mix was difficult. Unless otherwise stated, FT-IR and HRMS were measured on the mixture of diastereoisomers.

Ethyl 8-phenyl-8-azabicyclo[3.2.1]octane-3-carboxylate (3a)

Following **General Procedure C** with *N*-phenylpyrrolidine **1a** (0.029 mL, 0.200 mmol) and ethyl 2-(acetoxymethyl)prop-2-enoate (37.9 mg, 0.220 mmol), reacting for 20 minutes. Purification by flash column chromatography (SiO_2, heptanes/EtOAc 99:1 or pentane/Et_2O 96:4) afforded the major diastereoisomer as a white crystalline solid, closely followed by the minor diastereoisomer as a white solid (29.6 mg, 0.114 mmol, 57%, α/β 7:1).

Characterization of the α diastereoisomer:

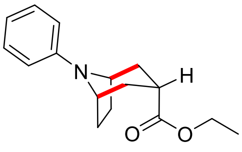

3a
$C_{16}H_{21}NO_2$
MW: 259,3490

^1H NMR (400 MHz, $CDCl_3$): δ 7.29–7.20 (m, 2H), 6.78 (d, J = 8.1 Hz, 2H), 6.71 (t, J = 7.2 Hz, 1H), 4.21 (q, J = 7.2 Hz, 2H), 4.17–4.15 (m, 2H), 2.48 (tt, J = 7.7, 1.8 Hz, 1H, -CH-CO_2Et), 2.28–2.17 (m, 2H), 2.17–2.09 (m, 2H), 2.04–1.96 (m, 2H), 1.88–1.78 (m, 2H), 1.31 (t, J = 7.1 Hz, 3H). ^{13}C NMR (101 MHz, $CDCl_3$): δ 176.1 (C=O), 146.4 (Cq), 129.7 (2xCH_{Ar}), 116.9 (CH_{Ar}), 115.2 (2CH_{Ar}), 61.0 (CH_2), 53.2 (2xCH), 34.6 (CH-CO_2Et), 27.1 (2xCH_2), 26.4 (2xCH_2), 14.4 (CH_3). FT-IR (cm^{-1}, neat): 2926, 1721, 1596,

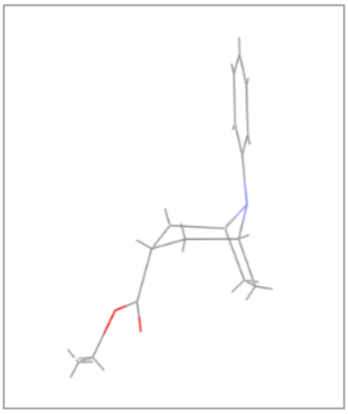

1494, 1368, 1330, 1292, 1173, 1047, 748, 693. HRMS (ESI) m/z: $[M+H]^+$ Calcd for $C_{16}H_{22}NO_2$ 260.1645; Found 260.1639. Mp 83.0–84.2 °C. R_f = 0.34 (pentane/Et_2O 96:4).

Coupling constant compatible with an equatorial proton (CH-CO_2Et) on a chair conformation (J_{ax-eq} = 7.7 Hz, J_{eq-eq} = 1.8 Hz). Structure confirmed by single-crystal X-ray diffraction.

Crystallization from DCM upon slow evaporation affords colorless crystals suitable for X-Ray analysis.

Characterization of the β diastereoisomer:

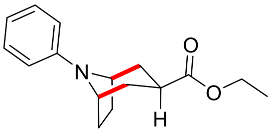

3aβ
$C_{16}H_{21}NO_2$
MW: 259,3490

^{1}H NMR (300 MHz, $CDCl_3$): δ 7.25–7.19 (m, 2H), 6.77 (d, J = 8.1 Hz, 2H), 6.70 (tt, J = 7.3, 1.1 Hz, 1H), 4.32–4.25 (m, 2H), 4.05 (q, J = 7.1 Hz, 2H), 2.89 (tt, J = 12.3, 5.3 Hz, 1H, -CH-CO_2Et), 2.16–2.07 (m, 2H), 2.07–1.97 (m, 2H), 1.85–1.75 (m, 2H), 1.64 (ddd, J = 13.8, 6.0, 2.9 Hz, 2H), 1.20 (t, J = 7.2 Hz, 3H).

Coupling constants of CH-CO_2Et compatible with an axial proton on a chair conformation (J_{ax-ax} = 12.3 Hz, J_{ax-eq} = 5.3 Hz).

^{13}C NMR (75 MHz, $CDCl_3$): δ 175.1 (C=O), 146.2 (Cq), 129.7 (2xCH$_{Ar}$), 117.2 (CH$_{Ar}$), 115.2 (2xCH$_{Ar}$), 60.5 (CH_2), 53.7 (2xCH), 35.5 (CH-CO_2Et), 29.3 (2xCH_2), 28.3 (2xCH_2), 14.3 (CH_3). FT-IR (cm^{-1}, neat): 2957, 1722, 1597, 1501, 1365, 1179, 1045, 937, 754, 694, 583. HRMS (ESI) m/z: $[M+H]^+$ Calcd for $C_{16}H_{22}NO_2$ 260.1645; Found 260.1642. Mp 71.5–73.1 °C. R_f = 0.22 (pentane/Et_2O 96:4).

Ethyl 8-[4-(4,4,5,5-tetramethyl-1,3,2-dioxaborolan-2-yl)phenyl]-8-azabicyclo[3.2.1]octane-3-carboxylate (3b)

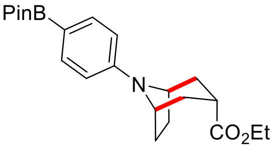

3b
$C_{22}H_{32}BNO_4$
MW: 385,3110

Following **General Procedure C** with 1-[4-(4,4,5,5-tetramethyl-1,3,2-dioxaborolan-2-yl)phenyl]pyrrolidine **1b** (54.6 mg, 0.20 mmol) and ethyl 2-(acetoxymethyl)prop-2-enoate (37.9 mg, 0.220 mmol) reacting for 20 minutes. Purification by flash column chromatography (SiO_2, heptanes/EtOAc 95:5) afforded the major diastereoisomer as a crystalline white solid (30.3 mg), followed by a mixture of both diastereoisomers as a white solid (7.5 mg, α/β 0.59/1) (m_{tot} = 37.8 mg, 0.10 mmol, 49%, α/β 5:1 (from crude NMR)).

Characterization of the α diastereoisomer:

^{1}H NMR (300 MHz, $CDCl_3$): δ 7.74–7.64 (m, 2H), 6.78–6.69 (m, 2H), 4.20 (m, 4H, overlapping -N-CH and -$CO_2CH_2CH_3$), 2.46 (tt, J_{ax-eq} = 7.0, J_{eq-eq} = 2.5 Hz, 1H, -CH-CO_2Et), 2.26–2.07 (m, 4H), 2.06–1.89 (m, 2H), 1.84 (m, 2H), 1.32 (s, 12H), 1.30 (t, J = 7.2 Hz, 3H). ^{13}C NMR (101 MHz, $CDCl_3$): δ 176.1 (C=O), 148.7 (Cq), 136.7 (2xCH$_{Ar}$), 114.11 (2xCH$_{Ar}$), 83.3 (2xCq), 61.0 (CH_2), 53.0 (2xCH), 34.6 (CH), 27.0 (2xCH_2), 26.7 (2xCH_2), 25.0 (4xCH_3), 14.4 (CH_3). Due to coupling to the quadrupolar ^{11}B and ^{10}B nuclei, the aromatic carbon atom bearing the BPin was not detected. ^{11}B NMR (96 MHz, $CDCl_3$): δ 30.9. FT-IR (cm^{-1}, neat):

2982, 2956, 2927, 1716, 1596, 1353, 1295, 1140, 1090, 1041, 825, 654. HRMS (ESI) m/z: $[M+H]^+$ Calcd for $C_{22}H_{33}O_4NB$ 386.2497; Found 386.2492. Mp 152.9–153.9 °C. R_f = 0.19 (heptanes/EtOAc 90:10)

Characteristic peaks of the β diastereoisomer extracted from the mixture:

^1H NMR (300 MHz, $CDCl_3$): δ 4.37–4.29 (m, 2H, 2xN-$\underline{CH}$), 4.03 (q, J = 7.1 Hz, 2H, -$CO_2\underline{CH_2}CH_3$), 2.89 (tt, $J_{ax\text{-}ax}$ = 11.7, $J_{ax\text{-}eq}$ = 5.3 Hz, 1H, -$\underline{CH}$-CO_2Et), 1.18 (t, J = 7.1 Hz, 3H, -$CO_2CH_2\underline{CH_3}$). ^{13}C NMR (101 MHz, $CDCl_3$): δ 174.9 (C=O), 148.6 (Cq), 114.09 (2xCH_{Ar}), 60.5 (CH_2), 53.5 (2xCH), 35.4 (CH), 29.7 (2xCH_2), 28.2 (2xCH_2), 14.3 (CH_3). ^{11}B NMR (96 MHz, $CDCl_3$): δ 30.9.

8-(4-Methoxycarbonylphenyl)-8-azabicyclo[3.2.1]octane-3-carboxylate (3c)

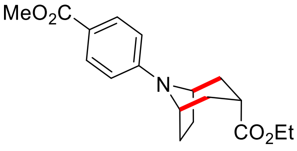

3c

$C_{18}H_{23}NO_4$

MW: 317,3850

Following **General Procedure C** with methyl 4-pyrrolidin-1-ylbenzoate (41.1 mg, 0.20 mmol) and ethyl 2-(acetoxymethyl)prop-2-enoate **1c** (37.9 mg, 0.220 mmol) reacting for 40 minutes. Purification by flash column chromatography (SiO_2, heptanes/EtOAc 95:5) afforded the product as a white solid (26.6 mg, 0.084 mmol, 42%, α/β 9:1).

Characterization of the α diastereoisomer:

^1H NMR (400 MHz, $CDCl_3$): δ 7.95–7.87 (m, 2H), 6.72 (d, J = 8.3 Hz, 2H), 4.28–4.21 (m, 2H), 4.21 (q, J = 7.1 Hz, 2H), 3.85 (s, 3H), 2.51 (tt, $J_{ax\text{-}eq}$ = 7.2, $J_{eq\text{-}eq}$ = 2.0 Hz, 1H, -$\underline{CH}$-CO_2Et), 2.28–2.10 (m, 4H), 2.06–1.96 (m, 2H), 1.90–1.82 (m, 2H), 1.31 (t, J = 7.1 Hz, 3H). ^{13}C NMR (101 MHz, $CDCl_3$): δ 175.8 (C=O), 167.4 (C=O), 149.6 (Cq), 131.9 (2xCH_{Ar}), 117.9 (Cq), 113.8 (2xCH_{Ar}), 61.1 (CH_2), 53.5 (2xCH), 51.7 (O-$\underline{C}H_3$), 34.5 (CH), 27.3 (2xCH_2), 27.0 (2xCH_2), 14.3 (CH_3). FT-IR (cm^{-1}, neat): 2949, 1717, 1695, 1603, 1516, 1434, 1382, 1279, 1176, 1110, 1044, 769. HRMS (ESI) m/z: $[M+H]^+$ Calcd for $C_{18}H_{24}NO_4$ 318.1700; Found 318.1688. Mp 104–106 °C. R_f = 0.25 (heptanes/EtOAc 80:20).

Characteristic peaks of the β diastereoisomer extracted from a mixed fraction:

^1H NMR (400 MHz, $CDCl_3$): δ 4.35 (dq, J = 5.3, 2.8 Hz, 2H), 4.05 (q, J = 7.1 Hz, 2H), 2.92 (tt, $J_{ax\text{-}ax}$ = 12.2, $J_{ax\text{-}eq}$ = 5.4 Hz, 1H, -$\underline{CH}$-CO_2Et), 1.94 (dd, J = 12.7, 2.7 Hz, 2H), 1.70 (ddd, J = 13.8, 5.6, 2.5 Hz, 2H), 1.19 (t, J = 7.1 Hz, 3H). ^{13}C NMR (101 MHz, $CDCl_3$): δ 174.7 (C=O), 149.7 (Cq), 131.3 (2xCH_{Ar}), 117.7 (Cq), 60.7 (CH_2), 53.7 (2xCH), 35.4 (CH), 29.9 (CH_2), 28.2 (CH_2), 14.3 (CH_3).

Ethyl 8-(4-methoxyphenyl)-8-azabicyclo[3.2.1]octane-3-carboxylate (3d)

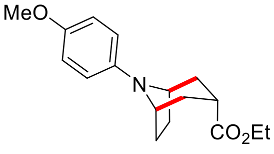

3d

$C_{17}H_{23}NO_3$

MW: 289,3750

Following **modified General Procedure C** with 1-(4-methoxyphenyl)pyrrolidine **1d** (35.4 mg, 0.20 mmol) and ethyl 2-(acetoxymethyl)prop-2-enoate (37.9 mg, 0.22 mmol), reacting for 5h30. Purification by flash column chromatography (SiO_2, heptanes/EtOAc 92:8) afforded the product as a white solid (32.1 mg, 0.11 mmol, 56%, α/β 5:1). Under the same purification conditions, the diastereoisomers can be partially separated to give a pure sample of the major diastereoisomer.

Characterization of the α diastereoisomer:

^{1}H NMR (300 MHz, $CDCl_3$): δ 6.87–6.80 (m, 2H), 6.76–6.69 (m, 2H), 4.20 (q, J = 7.1 Hz, 2H), 4.11–4.04 (m, 2H), 3.76 (s, 3H), 2.48 (tt, $J_{ax\text{-}eq}$ = 7.8, $J_{eq\text{-}eq}$ = 1.6 Hz, 1H, -CH-CO_2Et), 2.21 (ddd, J = 14.4, 7.8, 3.2 Hz, 2H), 2.21 (ddd, J = 14.5, 3.2, 1.5 Hz, 2H), 2.00–1.95 (m, 2H), 1.85–1.75 (m, 2H), 1.30 (t, J = 7.1 Hz, 3H). ^{13}C NMR (101 MHz, $CDCl_3$): δ 176.2 (C=O), 151.6 (Cq), 141.0 (Cq), 116.4 (2xCH_{Ar}), 115.2 (2xCH_{Ar}), 60.9 (CH_2), 55.8 (O-CH_3), 53.8 (2xCH), 34.6 (CH), 27.1 (2xCH_2), 26.3 (2xCH_2), 14.4 (CH_3). Mp 74.8–75.9 °C. $R_{f\ \text{major dia}}$: 0.43 (heptanes/EtOAc 80:20)

Characteristic peaks of the β diastereoisomer extracted from the mixed fraction:

^{1}H NMR (300 MHz, $CDCl_3$): δ 4.05 (q, J = 7.0 Hz, 2H), 3.76 (s, 3H), 2.86 (tt, $J_{\text{ax-ax}}$ = 12.3, $J_{\text{ax-eq}}$ = 5.2 Hz, 1H, -CH-CO_2Et), 2.07–1.97 (m, 2H), 1.64–1.58 (m, 2H), 1.20 (t, J = 7.1 Hz, 3H). ^{13}C NMR (101 MHz, $CDCl_3$): δ 175.2 (C=O), 151.8 (Cq), 140.7 (Cq), 116.43 (2xCH_{Ar}), 60.5 (CH_2), 55.85 (O-CH_3), 54.4 (2xCH), 35.4 (CH), 29.2 (2xCH_2), 28.3 (2xCH_2), 14.32 (CH_3). $R_{f\ \text{minor dia}}$: 0.34 (heptanes/EtOAc 80:20).

FT-IR (cm^{-1}, neat): 2985, 2952, 1719, 1510, 1446, 1372, 1244, 1176, 1163, 1079, 1048, 1032, 820. HRMS (ESI) m/z: $[M-H]^-$ Calcd for $C_{17}H_{22}NO_3$ 288.1594; Found 288.1586.

Ethyl 8-(2-tert-butylphenyl)-8-azabicyclo[3.2.1]octane-3-carboxylate (3e)

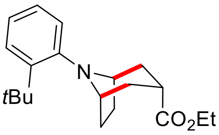

3e
$C_{20}H_{29}NO_2$
MW: 315,4570

Following **General Procedure C** with 1-(2-*tert*-butylphenyl)pyrrolidine **1e** (40.7 mg, 0.20 mmol) and ethyl 2-(acetoxymethyl)prop-2-enoate (37.9 mg, 0.22 mmol) reacting for 6 hours. Purification by flash column chromatography (SiO_2, heptanes/EtOAc 99.25:0.75) afforded unclean product (contaminated with traces mono-allylated intermediate having the same Rf), from which a dr of 6:1 could be determined. Second purification afforded clean product as a colorless oil (13.0 mg, 0.042 mmol, 21%). Only the major diastereoisomer was characterized.

Characterization of the α diastereoisomer:

^{1}H NMR (300 MHz, $CDCl_3$): δ 7.40 (dd, J = 7.4, 2.3 Hz, 1H), 7.12 (td, J = 7.6, 2.1 Hz, 1H), 7.07 (td, J = 7.4, 1.8 Hz, 1H), 6.99 (dd, J = 7.4, 2.1 Hz, 1H), 4.21 (q, J = 7.1 Hz, 2H), 3.43 (pent, J = 3.0 Hz, 2H), 2.72 (tt, $J_{\text{ax-eq}}$ = 8.4, $J_{\text{eq-eq}}$ = 2.4 Hz, 1H, -CH-CO_2Et), 2.41 (ddd, J = 13.8, 8.6, 4.1 Hz, 2H), 2.25 (dt, J = 14.0, 2.4 Hz, 2H), 2.12 (dd, J = 8.9, 4.5 Hz, 2H), 1.86–1.71 (m, 2H), 1.52 (s, 9H), 1.31 (t, J = 7.1 Hz, 3H). ^{13}C NMR (75 MHz, $CDCl_3$): δ 176.6 (C=O), 152.2 (Cq), 147.7 (Cq), 127.1 (CH_{Ar}), 126.5 (CH_{Ar}), 126.3 (CH_{Ar}), 124.8 (CH_{Ar}), 61.5 (2xCH), 60.7 (CH_2), 35.7 (Cq), 33.8 (CH), 33.2 (2xCH_2), 31.6 (3xCH_3), 28.7 (2xCH_2), 14.2 (CH_3). FT-IR (cm^{-1}, neat): 2949, 1717, 1695, 1603, 1516, 1434, 1382, 1279, 1176, 1110, 1044, 769. HRMS (ESI) m/z: $[M+H]^+$ Calcd for $C_{20}H_{30}NO_2$ 316.2271; Found 318.2266. Mp 104–106 °C. R_f = 0.41 (heptanes/EtOAc 90:10).

Ethyl 1-methyl-8-phenyl-8-azabicyclo[3.2.1]octane-3-carboxylate (3f)

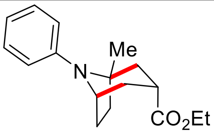

3f
$C_{17}H_{23}NO_2$
MW: 273,3760

Following **General Procedure C** with 2-methyl-1-phenyl-pyrrolidine **1f** (32.2 mg, 0.20 mmol) and ethyl 2-(acetoxymethyl)prop-2-enoate (37.9 mg, 0.220 mmol) reacting for 60 minutes. Purification by flash column chromatography (SiO_2, pentane/Et_2O 95:5) afforded the product as a colorless oil (22.0 mg, 0.08 mmol, 40%, α/β 8:1).

Characterization of the α diastereoisomer:

^1H NMR (400 MHz, $CDCl_3$): δ 7.25–7.17 (m, 2H), 7.06–6.99 (m, 2H), 6.80 (tt, J = 7.3, 1.1 Hz, 1H), 4.28–4.14 (m, 3H), 2.65–2.57 (m, 1H, -<u>CH</u>-CO_2Et), 2.30 (ddd, J = 14.2, 8.0, 1.8 Hz, 1H), 2.22 (dd, J = 14.1, 1.8 Hz, 1H), 2.14–2.06 (m, 2H), 2.06–1.90 (m, 2H), 1.74–1.60 (m, 2H), 1.48 (s, 3H), 1.31 (t, J = 7.1 Hz, 3H). ^{13}C NMR (101 MHz, $CDCl_3$): δ 176.2 (C=O), 147.5 (Cq), 129.0 (2xCH_{Ar}), 119.5 (2xCH_{Ar}), 118.9 (CH_{Ar}), 60.9 (Cq + CH_2), 59.6 (CH), 38.1 (CH_2), 35.7 (CH), 33.6 (CH_2), 27.7 (CH_2), 27.4 (CH_3), 25.7 (CH_2), 14.2 (CH_3). $R_{f\ major\ dia}$= 0.31 (heptanes/EtOAc 90:10).

Characteristic peaks of the β diastereoisomer:

^1H NMR (400 MHz, $CDCl_3$): δ 4.33–4.29 (m, 1H), 4.08 (q, J = 7.2 Hz, 2H), 2.89 (tt, J_{ax-ax} = 12.3, J_{ax-eq} = 5.4 Hz, 1H, -<u>CH</u>-CO_2Et), 1.89–1.82 (m, 2H), 1.49 (s, 3H), 1.22 (t, J = 7.1 Hz, 3H). ^{13}C NMR (101 MHz, $CDCl_3$): δ 175.2 (C=O), 147.2 (Cq), 129.0 (2xCH_{Ar}), 119.9 (2xCH_{Ar}), 119.2 (CH_{Ar}), 60.5 (CH_2), 60.2 (CH), 39.2 (CH_2), 36.6 (<u>CH</u>-CO_2Et), 36.0 (CH_2), 30.1 (CH_2), 26.8 (CH_3), 26.7 (CH_2), 14.2 (CH_3). $R_{f\ minor\ dia}$= 0.22 (heptanes/EtOAc 90:10).

FT-IR (cm^{-1}, neat): 2927, 1723, 1596, 1494, 1368, 1184, 1106, 1028, 861, 751, 692. HRMS (ESI) m/z: $[M+H]^+$ Calcd for $C_{17}H_{24}NO_2$ 274.1802; Found 274.1793.

Conformation of homotropanes

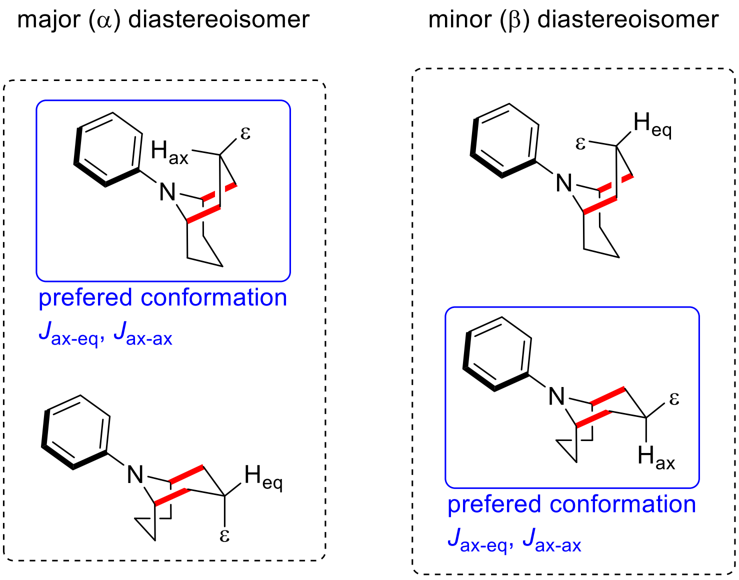

The configuration of the major diastereoisomer was confirmed by single crystal X-ray crystallography of **8g**. In the solid state, the ring containing the ester moiety adopts a boat conformation with the ester group lying in the bowsprit position, which is in agreement with the ^3J coupling constants observed in the ^1H NMR spectrum. As similar coupling constants were observed for the proton alpha to the ester group in the two diastereoisomers, it can be concluded that the minor diastereoisomer adopts in solution a chair

conformation for the 6-membered ring containing the ester group, with the latter adopting an equatorial position as well. In agreement with these finding, the CH-CO_2Et proton of the major diastereoisomer is systematically observed at higher field (shielding effect of the aromatic ring) compared to the minor diastereoisomer, as it was the case for the pyrrolidine derivatives.

Ethyl 9-phenyl-9-azabicyclo[3.3.1]nonane-3-carboxylate (8a)

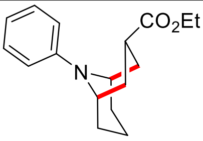

8a
$C_{17}H_{23}NO_2$
MW: 273,3760

0.2 mmol scale synthesis:

Following **General Procedure C** with freshly purified 1-phenylpiperidine **6a** (32.2 mg, 0.20 mmol) and ethyl 2-(acetoxymethyl)prop-2-enoate (37.9 mg, 0.22 mmol) reacting for 1h30. Purification by flash column chromatography (SiO_2, heptanes/EtOAc 90:10) afforded the product as a colorless oil (37.3 mg, 0.13 mmol, 67%, α/β >20:1 on this scale). Erosion of the yield of up to 20% was observed when using not fresh 1-phenylpiperidine.

3 mmol scale synthesis:

Into a flame-dried Schlenck tube under N_2 were added ethyl 2-(acetoxymethyl)prop-2-enoate (568 mg, 3.30 mmol), freshly purified 1-phenylpiperidine **6a** (484 mg, 3.00 mmol), [Ir{dF(CF_3)ppy}$_2$(dtbpy)]PF_6 (16.8 mg, **0.5 mol%**) and cesium acetate (692 mg, 3.61 mmol). The vial was evacuated and filled with nitrogen (x 3). Dry 1,2-dichloroethane (60 mL) was added. The resulting yellow solution was was degassed by freeze-pump-thaw cycles (x 3). The mixture was placed between two 390 nm LEDs and stirred for 1h30. The reaction mixture was diluted with sat. $NaHCO_3$ (40 mL). The aqueous phase was extracted with DCM (3 x 60 mL) and the combined organic phases were dried over Na_2SO_4, filtered, and concentrated under reduced pressure to give crude product as an orange oil. Purification by flash column chromatography (SiO_2, heptanes/EtOAc 95:5) afforded the product as a colorless oil (462 mg, 1.68 mmol, 56%, α/β 92:8 on this scale). The diastereoisomers can be separated by flash column chromatography (SiO_2, pentane/Et_2O 95:5).

Characterization of the α diastereoisomer:

^{1}H NMR (300 MHz, $CDCl_3$): δ 7.25–7.17 (m, 2H), 6.88–6.82 (m, 2H), 6.68 (tt, J = 7.3, 1.0 Hz, 1H), 4.29–4.18 (m, 2H), 4.11 (q, J = 7.1 Hz, 2H), 2.48–2.31 (m, 3H), 2.02 (qt, J = 13.3, 4.5 Hz, 1H), 1.78 (tt, J = 13.2, 4.3 Hz, 2H), 1.73–1.63 (m, 2H), 1.63–1.50 (m, 1H), 1.50–1.43 (m, 2H), 1.23 (t, J = 7.1 Hz, 3H). ^{13}C NMR (101 MHz, $CDCl_3$): δ 176.0 (C=O), 150.7 (Cq), 129.6 (2xCH_{Ar}), 117.1 (CH_{Ar}), 114.3 (2xCH_{Ar}), 60.4 (CH_2), 47.2 (2xCH), 34.9 (CH), 30.5 (2xCH_2), 28.5 (2xCH_2), 14.4 (CH_3), 14.3 (CH_2). FT-IR (cm^{-1}, neat): 2928, 1726, 1594, 1498, 1280, 1244, 1174, 1033, 909, 747, 688. HRMS (ESI) m/z: [M+H]$^+$ Calcd for $C_{17}H_{24}NO_2$ 274.1802; Found 274.1803. R_f = 0.40 (heptanes/EtOAc 90:10).

Characterization of the β diastereoisomer:

^{1}H NMR (300 MHz, $CDCl_3$): δ 7.29–7.17 (m, 2H), 6.84 (d, J = 8.3 Hz, 2H), 6.68 (t, J = 7.2 Hz, 1H), 4.18–4.03 (m, 4H), 3.32 (tt, $J_{ax\text{-}ax}$ = 12.1, $J_{ax\text{-}eq}$ = 5.7 Hz, 1H, -CH-CO_2Et), 2.20–1.98 (m, 5H), 1.93 (ddd, J = 13.9,

5.7, 2.0 Hz, 2H), 1.76–1.62 (m, 3H), 1.23 (t, *J* = 7.1 Hz, 3H). ^{13}C NMR (75 MHz, $CDCl_3$): *δ* 175.5 (C=O), 148.7 (Cq), 129.6 (2xCH_{Ar}), 116.9 (CH_{Ar}), 114.0 (2xCH_{Ar}), 60.5 (CH_2), 48.0 (2xCH), 37.8 (CH-CO_2Et), 31.0 (2xCH_2), 27.9 (2xCH_2), 20.4 (CH_2), 14.4 (CH_3). FT-IR (cm^{-1}, neat): 2935, 1725, 1592, 1500, 1189, 1111, 1036, 908, 746, 689. HRMS (ESI) m/z: $[M+H]^+$ Calcd for $C_{17}H_{24}NO_2$ 274.1802; Found 274.1800. Mp 74.4–75.0 °C. R_f = 0.25 (pentane/Et_2O 94:6).

Ethyl 9-(o-tolyl)-9-azabicyclo[3.3.1]nonane-3-carboxylate (8b)

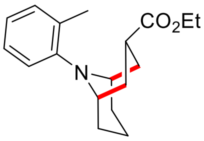

8b
$C_{18}H_{25}NO_2$
MW: 287,4030

Following **General Procedure C** with 1-(o-tolyl)piperidine **6b** (35.1 mg, 0.20 mmol) and ethyl 2-(acetoxymethyl)prop-2-enoate (37.9 mg, 0.22 mmol), reacting for 1h30. Purification by flash column chromatography (SiO_2, pentane/Et_2O 98:2) afforded the product as a colorless oil (26.2 mg, 0.09 mmol, 46%, α/β 20:1).

Characterization of the α diastereoisomer:

^{1}H NMR (400 MHz, $CDCl_3$): *δ* 7.16–7.07 (m, 2H), 6.95 (dd, *J* = 8.5, 1.2 Hz, 1H), 6.82 (td, *J* = 7.4, 1.2 Hz, 1H), 4.13 (q, *J* = 7.2 Hz, 2H), 3.84–3.74 (m, 2H), 2.73 (tt, $J_{ax\text{-}ax}$ = 12.8, $J_{ax\text{-}eq}$ = 6.0 Hz, 1H, -CH-CO_2Et), 2.41–2.30 (m, 2H), 2.27 (s, 3H), 2.06 (qt, *J* = 13.2, 4.4 Hz, 1H), 1.89 (tt, *J* = 13.1, 4.2 Hz, 2H), 1.69 (td, *J* = 13.6, 3.5 Hz, 2H), 1.64–1.53 (m, 1H), 1.51–1.41 (m, 2H), 1.25 (t, *J* = 7.2 Hz, 3H). ^{13}C NMR (101 MHz, $CDCl_3$): *δ* 176.2 (C=O), 151.8 (Cq), 131.9 (CH_{Ar}), 129.9 (Cq), 126.7 (CH_{Ar}), 120.4 (CH_{Ar}), 118.7 (CH_{Ar}), 60.4 (CH_2), 49.4 (2xCH), 34.9 (CH), 31.67 (2xCH_2), 29.1 (2xCH_2), 19.9 (CH_3), 14.4 (CH_3), 14.1 (CH_2). FT-IR (cm^{-1}, neat): 2927, 1727, 1595, 1488, 1277, 1241, 1173, 1049, 751. HRMS (ESI) m/z: $[M+H]^+$ Calcd for $C_{18}H_{26}NO_2$ 288.1958; Found 288.1970. R_f = 0.21 (pentane/Et_2O 95:5)

Characteristic peaks of the β diastereoisomer:

^{1}H NMR (400 MHz, $CDCl_3$): *δ* 3.62–3.55 (m, 2H, 2xN-CH), 3.39–3.29 (m, 1H, -CH-CO_2Et). ^{13}C NMR (101 MHz, $CDCl_3$): *δ* 176.0 (C=O), 131.8 (CH_{Ar}), 126.5 (CH_{Ar}), 120.8 (CH_{Ar}), 119.4 (CH_{Ar}), 60.5 (CH_2), 50.6 (2xCH), 37.5 (CH), 31.72 (2xCH_2), 28.6 (2xCH_2).

Ethyl 9-(m-tolyl)-9-azabicyclo[3.3.1]nonane-3-carboxylate (8c)

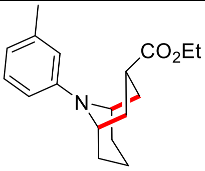

8c
$C_{18}H_{25}NO_2$
MW: 287,4030

Following **modified General Procedure C** with 1-(m-tolyl)piperidine **6c** (35.1 mg, 0.20 mmol) and ethyl 2-(acetoxymethyl)prop-2-enoate (37.9 mg, 0.22 mmol), reacting for 1h30. Purification by flash column chromatography (SiO_2, pentane/Et_2O 97:3) afforded the product as a colorless oil (35.2 mg, 0.12 mmol, 61%, α/β > 20:1).

^{1}H NMR (300 MHz, $CDCl_3$): *δ* 7.10 (t, *J* = 7.7 Hz, 1H), 6.71–6.61 (m, 2H), 6.51 (d, *J* = 7.4 Hz, 1H), 4.28–4.19 (m, 2H), 4.10 (q, *J* = 7.1 Hz, 2H), 2.49–2.31 (m, 3H), 2.29 (s, 3H), 2.01 (qt, *J* = 13.3, 4.4 Hz, 1H), 1.78 (tt, *J* = 13.1, 4.3 Hz, 2H), 1.71–1.63 (m, 2H), 1.60–1.50 (m, 1H), 1.50–1.40 (m, 2H), 1.23 (t, *J* = 7.2 Hz, 3H). ^{13}C NMR (75 MHz, $CDCl_3$): *δ* 176.0 (C=O), 150.7 (Cq), 139.3 (Cq), 129.5 (CH_{Ar}), 118.1 (CH_{Ar}), 115.1 (CH_{Ar}), 111.3 (CH_{Ar}), 60.4 (CH_2), 47.2 (2xCH), 34.9 (CH), 30.5 (2xCH_2), 28.5 (2xCH_2), 22.1 (CH_3), 14.4 (CH_3), 14.3 (CH_2). FT-IR (cm^{-1}, neat): 2925, 1726, 1597, 1492, 1282, 1245, 1174, 764, 729,

690. HRMS (ESI) m/z: [M+H]+ Calcd for $C_{18}H_{26}NO_2$ 288.1958; Found 288.1967. R_f = 0.28 (pentane/Et_2O 95:5).

Ethyl 9-(p-tolyl)-9-azabicyclo[3.3.1]nonane-3-carboxylate (8d)

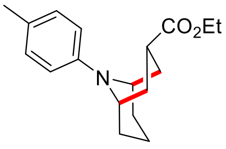

8d
$C_{18}H_{25}NO_2$
MW: 287,4030

Following **modified General Procedure C** with 1-(p-tolyl)piperidine **6d** (35.1 mg, 0.20 mmol) and ethyl 2-(acetoxymethyl)prop-2-enoate (37.9 mg, 0.22 mmol), reacting for 1h30. Purification by flash column chromatography (SiO_2, heptanes/EtOAc 96:4) afforded the product as a colorless oil (33.9 mg, 0.12 mmol, 59%, α/β 20:1).

Characterization of the α diastereoisomer:

^{1}H NMR (400 MHz, $CDCl_3$): δ 7.07–6.98 (m, 2H), 6.82–6.73 (m, 2H), 4.23–4.15 (m, 2H), 4.10 (q, J = 7.1 Hz, 2H), 2.50–2.30 (m, 3H, overlapping signals containing -CH-CO_2Et), 2.24 (s, 3H), 2.01 (qt, J = 13.4, 4.6 Hz, 1H), 1.78 (tt, J = 13.2, 4.3 Hz, 2H), 1.66 (ddd, J = 13.8, 12.1, 3.5 Hz, 2H), 1.58–1.49 (m, 1H), 1.49–1.40 (m, 2H), 1.23 (t, J = 7.1 Hz, 3H). ^{13}C NMR (101 MHz, $CDCl_3$): δ 176.0 (C=O), 148.6 (Cq), 130.1 (2xCH_{Ar}), 126.3 (Cq), 114.4 (2xCH_{Ar}), 60.4 (CH_2), 47.4 (2xCH), 34.8 (CH), 30.4 (2xCH_2), 28.4 (2xCH_2), 20.4 (CH_3), 14.4 (CH_3), 14.3 (CH_2). FT-IR (cm^{-1}, neat): 2923, 1726, 1615, 1511, 1279, 1246, 1173, 801. HRMS (ESI) m/z: [M+H]+ Calcd for $C_{18}H_{26}NO_2$ 288.1958; Found 288.1968. R_f = 0.29 (heptanes/EtOAc 90:10).

Characteristic peaks of the β diastereoisomer:

^{1}H NMR (400 MHz, $CDCl_3$): δ 3.31 (tt, $J_{ax\text{-}ax}$ = 12.2, $J_{ax\text{-}eq}$ = 5.7 Hz, 1H, CH-CO_2Et). ^{13}C NMR (101 MHz, $CDCl_3$): δ 48.1 (2xCH), 37.8 (CH_3), 30.9 (2xCH_2), 27.8 (2xCH_2).

Ethyl 9-(4-methoxyphenyl)-9-azabicyclo[3.3.1]nonane-3-carboxylate (8e)

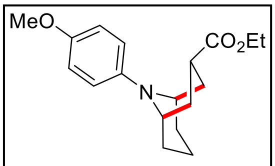

8e
$C_{18}H_{25}NO_3$
MW: 303,4020

Following **modified General Procedure C** with 1-(4-methoxyphenyl)piperidine **6e** (38.3 mg, 0.20 mmol) and ethyl 2-(acetoxymethyl)prop-2-enoate (37.9 mg, 0.22 mmol), reacting for 22h. Purification by flash column chromatography (SiO_2, pentane/Et_2O 96:4) afforded the product as a colorless oil that crystallizes slowly to a white solid upon storage at 4 °C (19.2 mg, 0.064 mmol, 32%, α/β >20:1).

Characterization of the α diastereoisomer:

^{1}H NMR (300 MHz, $CDCl_3$): δ 6.80 (s, 4H), 4.17–4.10 (m, 2H), 4.10 (q, J = 7.1 Hz, 2H), 3.75 (s, 3H), 2.47 (tt, J = 12.4, 5.9 Hz, 1H), 2.42–2.26 (m, 2H), 2.00 (qt, J = 13.3, 4.4 Hz, 1H), 1.79 (tt, J = 13.2, 4.3 Hz, 2H), 1.65 (ddd, J = 14.0, 12.3, 3.5 Hz, 2H), 1.58–1.49 (m, 1H), 1.49–1.39 (m, 2H), 1.23 (t, J = 7.1 Hz, 3H). ^{13}C NMR (101 MHz, $CDCl_3$): δ 176.1 (C=O), 151.6 (Cq), 145.3 (Cq), 115.7 (2xCH_{Ar}), 115.1 (2xCH_{Ar}), 60.4 (CH_2), 55.9 (CH_3), 47.8 (2xCH), 34.7 (CH), 30.6 (2xCH_2), 28.4 (2xCH_2), 14.4 (CH_3), 14.3 (CH_2). FT-IR (cm^{-1}, neat): 2870, 1726, 1507, 1241, 1177, 1039, 910, 812, 731, 616. HRMS (ESI) m/z: [M+H]+ Calcd for $C_{18}H_{26}NO_3$ 304.1907; Found 304.1899. Mp 42.5–43.7 °C. R_f = 0.31 (pentane/Et_2O 90:10).

Ethyl 9-(4-bromophenyl)-9-azabicyclo[3.3.1]nonane-3-carboxylate (8f)

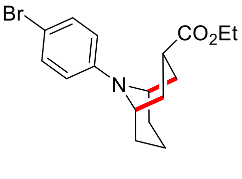

8f
$C_{17}H_{22}BrNO_2$
MW: 352,2720

Following **General Procedure C** with 1-(4-bromophenyl)piperidine **6f** (49.0 mg, 0.20 mmol) and ethyl 2-(acetoxymethyl)prop-2-enoate (37.9 mg, 0.22 mmol) reacting for 1h30. Purification by flash column chromatography (SiO_2, heptanes/EtOAc 97:3) afforded the product as a colorless oil (21.2 mg, 0.06 mmol, 30%, α/β >20:1).

Characterization of the α diastereoisomer:

^{1}H NMR (300 MHz, $CDCl_3$): δ 7.32–7.21 (m, 2H), 6.76–6.67 (m, 2H), 4.22–4.12 (m, 2H), 4.11 (q, J = 7.1 Hz, 2H), 2.45–2.27 (m, 3H), 2.01 (qt, J = 13.4, 4.1 Hz, 1H), 1.82–1.64 (m, 4H), 1.64–1.50 (m, 1H), 1.50–1.38 (m, 2H), 1.23 (t, J = 7.1 Hz, 3H). ^{13}C NMR (75 MHz, $CDCl_3$): δ 175.7 (C=O), 149.7 (Cq), 132.3 (2xCH_{Ar}), 115.9 (2xCH_{Ar}), 108.6 (Cq), 60.5 (CH_2), 47.4 (2xCH), 34.9 (CH), 30.2 (2xCH_2), 28.4 (2xCH_2), 14.4 (CH_3), 14.2 (CH_2). FT-IR (cm^{-1}, neat): 2927, 2868, 1724, 1586, 1490, 1282, 1246, 1174, 1043, 908, 804, 732. HRMS (ESI) m/z: $[M+H]^+$ Calcd for $C_{17}H_{23}BrNO_2$ 352.0907; Found 352.0914. R_f = 0.30 (heptanes/EtOAc 90:10).

Ethyl 9-(4-methoxycarbonylphenyl)-9-azabicyclo[3.3.1]nonane-3-carboxylate (8g).

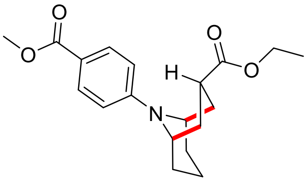

8g
$C_{19}H_{25}NO_4$
MW: 331,4120

0.2 mmol synthesis

Following **General Procedure C** with methyl 4-(1-piperidyl)benzoate **6g** (43.9 mg, 0.20 mmol) and ethyl 2-(acetoxymethyl)prop-2-enoate (37.9 mg, 0.22 mmol) reacting for 20 minutes. Purification by flash column chromatography (SiO_2, heptanes/EtOAc 90:10) afforded the product as a white solid (43.1 mg, 0.13 mmol, 65%, α/β >20:1). Recrystallization from Et_2O/hexane at rt afforded single-crystals suitable for X-Ray diffraction analysis.

1 mmol synthesis

Into an oven-dried seal tube were added under N_2 ethyl 2-(acetoxymethyl)prop-2-enoate (189 mg, 1.1 mmol), methyl 4-(1-piperidyl)benzoate (219 mg, 1.0 mmol), $[Ir\{dF(CF_3)ppy\}_2(dtbpy)]PF_6$ (5.6 mg, **0.5 mol%**) and cesium acetate (231 mg, 1.2 mmol). The vial was evacuated and filled with nitrogen (x 3). Dry and degassed 1,2-dichloroethane (20 mL) was added. The resulting yellow solution was placed in front of a 390 nm LED and stirred for 1h. Purification by flash column chromatography (SiO_2, heptanes/EtOAc 90:10) afforded the product a white solid (209 mg, 0.63 mmol, 63%, α/β >20:1).

Characterization of the α diastereoisomer:

^{1}H NMR (300 MHz, $CDCl_3$): δ 7.91–7.82 (m, 2H), 6.83–6.74 (m, 2H), 4.38–4.28 (m, 2H), 4.10 (q, J = 7.1 Hz, 2H), 3.84 (s, 3H), 2.45–2.23 (m, 3H, including -<u>CH</u>-CO_2Et), 2.14–1.89 (m, 1H), 1.82–1.63 (m, 4H), 1.63–1.42 (m, 3H), 1.22 (t, J = 7.2 Hz, 3H). ^{13}C NMR (75 MHz, $CDCl_3$): δ 175.5 (C=O), 167.2 (C=O), 153.7 (Cq), 131.8 (2xCH_{Ar}), 117.7 (Cq), 112.5 (2xCH_{Ar}), 60.6 (CH_2), 51.6 (CH_3), 47.2 (2xCH), 35.1 (CH), 30.2 (2xCH_2), 28.5 (2xCH_2), 14.3 (CH_3), 14.1 (CH_2). FT-IR (cm^{-1}, neat): 2929, 1729, 1694, 1599, 1517, 1282,

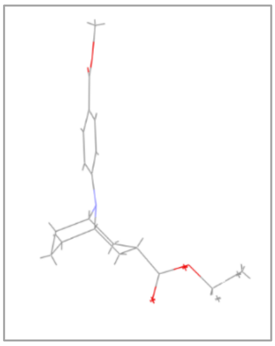

1247, 1170, 1107, 836, 768. HRMS (ESI) m/z: $[M+H]^+$ Calcd for $C_{19}H_{26}NO_4$ 332.1856; Found 332.1862. Mp 85.9–86.5 °C. R_f = 0.31 (heptanes/EtOAc 80:20).

Characteristic peaks of the β diastereoisomer:

^{1}H NMR (400 MHz, $CDCl_3$): δ 3.31 (tt, $J_{ax\text{-}ax}$ = 11.8, $J_{ax\text{-}eq}$ = 5.7 Hz, 1H, -CH-CO_2Et). X-Ray diffraction analysis confirmed the configuration of the major diastereoisomer.

Ethyl 9-(4-acetylphenyl)-9-azabicyclo[3.3.1]nonane-3-carboxylate (8h)

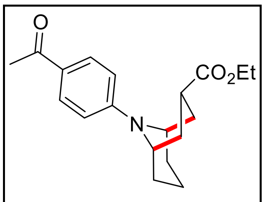

8h
$C_{19}H_{25}NO_3$
MW: 315,4130

Following **General Procedure C** with 1-[4-(1-piperidyl)phenyl]ethanone **6h** (40.7 mg, 0.20 mmol) and ethyl 2-(acetoxymethyl)prop-2-enoate (37.9 mg, 0.22 mmol) reacting for 3h. Purification by flash column chromatography (SiO_2, heptanes/EtOAc 95:5 to 88:12) afforded the product as a colorless oil (27.1 mg, 0.08 mmol, 42%, α/β >20:1) along with mono-allylated intermediate (3.7 mg, 6%; R_f = 0.24 (heptanes/EtOAc 80:20)).

Characterization of the α diastereoisomer:

^{1}H NMR (400 MHz, $CDCl_3$): δ 7.87–7.81 (m, 2H), 6.83–6.76 (m, 2H), 4.40–4.32 (m, 2H), 4.11 (q, J = 7.2 Hz, 2H), 2.49 (s, 3H), 2.45–2.25 (m, 3H), 2.04 (qt, J = 12.5, 4.2 Hz, 1H), 1.80–1.67 (m, 4H), 1.62–1.46 (m, 3H), 1.23 (t, J = 7.2 Hz, 3H).^{13}C NMR (75 MHz, $CDCl_3$): δ 196.2 (C=O), 175.4 (C=O), 153.8 (Cq), 131.1 (2xCH_{Ar}), 126.1 (Cq), 112.3 (2xCH_{Ar}), 60.6 (CH_2), 47.2 (2xCH), 35.1 (CH), 30.3 (2xCH_2), 28.6 (2xCH_2), 26.1 (CH_3), 14.3 (CH_3), 14.2 (CH_2). FT-IR (cm^{-1}, neat): 2927, 2870, 1725, 1661, 1591, 1278, 1253, 1188, 1044, 908, 813, 727, 588. HRMS (ESI) m/z: $[M+H]^+$ Calcd for $C_{19}H_{26}NO_3$ 316.1907; Found 316.1880. R_f = 0.21 (heptanes/EtOAc 80:20).

Characteristic peak of the β diastereoisomer:

^{1}H NMR (400 MHz, $CDCl_3$): δ 3.32 (tt, $J_{ax\text{-}ax}$ = 12.0, $J_{ax\text{-}eq}$ = 5.8 Hz, 1H, -CH-CO_2Et).

Ethyl 9-[4-(trifluoromethyl)phenyl]-9-azabicyclo[3.3.1]nonane-3-carboxylate (8i)

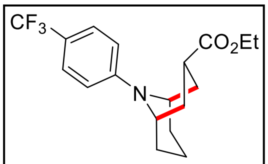

8i
$C_{18}H_{22}F_3NO_2$
MW: 341,3742

Following **General Procedure C** with 1-[4-(trifluoromethyl)phenyl]piperidine **6i** (45.8 mg, 0.20 mmol) and ethyl 2-(acetoxymethyl)prop-2-enoate (37.9 mg, 0.22 mmol) reacting for 40 minutes. Purification by flash column chromatography (SiO_2, pentane/Et_2O 95:5) afforded the product as a colorless oil (37.5 mg, 0.11 mmol, 55%, α/β >14:1).

Characterization of the α diastereoisomer:

^{1}H NMR (400 MHz, $CDCl_3$): δ 7.42 (d, J = 8.9 Hz, 2H), 6.84 (d, J = 8.8 Hz, 2H), 4.33–4.24 (m, 2H), 4.12 (q, J = 7.1 Hz, 2H), 2.46–2.24 (m, 3H), 2.04 (qt, J = 13.3, 4.3 Hz, 1H), 1.82–1.64 (m, 4H), 1.60–1.53 (m, 1H), 1.53–1.42 (m, 2H), 1.24 (t, J = 7.1 Hz, 3H). ^{13}C NMR (101 MHz, $CDCl_3$): δ 175.4 (C=O), 152.5 (Cq), 126.8 (q, $^3J_{C\text{–}F}$ = 3.7 Hz, 2xCH_{Ar}), 124.9 (q, $^1J_{C\text{–}F}$ = 270.1 Hz, CF_3), 118.1 (q, $^2J_{C\text{–}F}$ = 32.8 Hz, Cq), 113.0 (2xCH_{Ar}),

60.4 (CH_2), 47.1 (2xCH), 34.9 (CH), 30.1 (2xCH_2), 28.4 (2xCH_2), 14.2 (CH_3), 14.0 (CH_2). ^{19}F NMR (282 MHz, $CDCl_3$): δ –61.1. FT-IR (cm^{-1}, neat): 2931, 1726, 1610, 1521, 1401, 1320, 1302, 1286, 1257, 1103, 1067, 813, 588. HRMS (ESI) m/z: $[M+H]^+$ Calcd for $C_{18}H_{23}F_3NO_2$ 342.1675; Found 342.1674. R_f = 0.28 (pentane/Et_2O 90:10).

Characteristic peaks of the β diastereoisomer:

^{1}H NMR (400 MHz, $CDCl_3$): δ 4.19 (br m, 2H), 3.32 (tt, $J_{ax\text{-}ax}$ = 12.0, $J_{ax\text{-}eq}$ = 5.7 Hz, 1H, -CH-CO_2Et), 1.23 (t, 3H, J = 7.1 Hz). ^{13}C NMR (101 MHz, $CDCl_3$): δ 175.0 (C=O), 112.8 (2xCH_{Ar}), 60.5 (CH_2), 47.9 (2xCH), 37.5 (CH), 31.0 (2xCH_2), 28.0 (2xCH_2).

Ethyl 9-[4-(4,4,5,5-tetramethyl-1,3,2-dioxaborolan-2-yl)phenyl]-9-azabicyclo[3.3.1]nonane-3-carboxylate (8j)

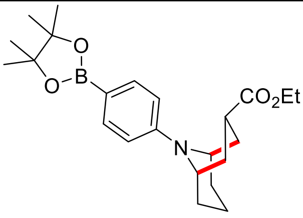

8j
$C_{23}H_{34}BNO_4$
MW: 399,3380

0.2 mmol scale synthesis:

Following **General Procedure C** with 1-[4-(4,4,5,5-tetramethyl-1,3,2-dioxaborolan-2-yl)phenyl]piperidine **6j** (58.6 mg, 0.20 mmol) and ethyl 2-(acetoxymethyl)prop-2-enoate (37.9 mg, 0.22 mmol) reacting for 40 minutes. Purification by flash column chromatography (SiO_2, heptanes/EtOAc 90:10) afforded the product as a colorless oil (52.4 mg, 0.13 mmol, 66%, α/β >20:1). Upon evaporation from pentane the oil became a crystalline solid.

3 mmol scale synthesis:

Into a flame-dried Schlenck tube were added under nitrogen ethyl 2-(acetoxymethyl)prop-2-enoate (568 mg, 3.30 mmol), 1-[4-(4,4,5,5-tetramethyl-1,3,2-dioxaborolan-2-yl)phenyl]piperidine **6j** (862 mg, 3.00 mmol), [Ir{dF(CF_3)ppy}$_2$(dtbpy)]PF_6 (16.8 mg, **0.5 mol%**) and cesium acetate (692 mg, 3.61 mmol). The vial was evacuated and filled with nitrogen (x 3). Dry 1,2-dichloroethane (60 mL) was canuled into the vial, a yellow solution was obtained that was degassed by 3 freeze-and-thaw cycles. The mixture was placed between two 390 nm LED and stirred for 1h. Purification by flash column chromatography (SiO_2, heptanes/EtOAc 90:10) afforded the product as a white solid (694 mg, 1.74 mmol, 58%, α/β >20:1).

Characterization of the α diastereoisomer:

^{1}H NMR (300 MHz, $CDCl_3$): δ 7.72–7.61 (m, 2H), 6.85–6.77 (m, 2H), 4.37–4.25 (m, 2H), 4.09 (q, J = 7.1 Hz, 2H), 2.45–2.25 (m, 3H), 2.02 (qt, J = 11.7, 3.6 Hz, 1H), 1.82–1.59 (m, 4H), 1.59–1.40 (m, 3H), 1.32 (s, 12H), 1.22 (t, J = 7.1 Hz, 3H). ^{13}C NMR (101 MHz, $CDCl_3$): δ 175.8 (C=O), 152.8 (Cq), 136.8 (2xCH_{Ar}), 113.0 (2xCH_{Ar}), 83.4 (2xCq), 60.5 (CH_2), 47.0 (2xCH), 35.0 (CH), 30.3 (2xCH_2), 28.5 (2xCH_2), 25.0 (4xCH_3), 14.4 (CH_3), 14.3 (CH_2). Due to coupling to the quadrupolar ^{11}B and ^{10}B nuclei, the aromatic carbon atom bearing the BPin was not detected. ^{11}B NMR (96 MHz, $CDCl_3$): δ +31.6. FT-IR (cm^{-1}, neat): 2975, 2928,

1726, 1600, 1399, 1359, 1141, 1091, 961, 908, 860, 731, 653. HRMS (ESI) m/z: $[M+H]^+$ Calcd for $C_{23}H_{34}BNO_2$ 400.2654; Found 400.2663. Mp 86.7–88.0 °C. R_f = 0.31 (heptanes/EtOAc 90:10).

Ethyl 9-phenyl-3-oxa-9-azabicyclo[3.3.1]nonane-7-carboxylate (8k)

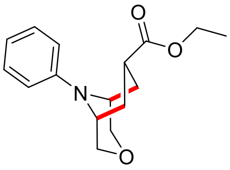

8k
$C_{16}H_{21}NO_3$
MW: 275,3480

Following **General Procedure C** with 4-phenylmorpholine (32.6 mg, 0.20 mmol) and ethyl 2-(acetoxymethyl)prop-2-enoate (37.9 mg, 0.22 mmol) reacting for 20 minutes. Purification by flash column chromatography (SiO_2, heptanes/EtOAc 92:8) afforded an inseparable mixture of diastereoisomers as a colorless oil (35.4 mg, 0.13 mmol, 64%, α/β 5:1). The oil became a white solid after a few days at 4 °C.

Characterization of the α diastereoisomer:

1H NMR (400 MHz, $CDCl_3$) δ 7.28–7.25 (m, 2H), 6.82–6.80 (m, 2H), 6.77–7.73 (m, 1H), 4.18 (q, J = 7.2 Hz, 2H), 3.92 (br d, J = 11.3, 2H), 3.83 (dt, J = 11.0, 2.3 Hz, 2H), 3.77–3.73 (m, 2H), 2.60 (tt, J = 7.5, 1.9 Hz, 1H), 2.42 (dq, J = 14.2, 1.8 Hz, 2H), 2.17 (dddd, J = 14.7, 7.3, 5.2, 2.0 Hz, 2H), 1.29 (t, J = 7.1, 7.1 Hz, 3H). ^{13}C NMR (101 MHz, $CDCl_3$): δ 173.7 (C=O), 147.7 (Cq), 129.8 ($2xCH_{Ar}$), 117.9 (CH_{Ar}), 113.66 ($2xCH_{Ar}$), 70.8 ($2xCH_2$), 60.5 (CH_2), 48.9 (2xCH), 37.0 (CH), 25.3 ($2xCH_2$), 14.3 (CH_3). R_f = 0.35 (heptanes/EtOAc 80:20).

Characteristic peaks of the β diastereoisomer:

^1H NMR (400 MHz, $CDCl_3$): δ 7.28–7.25 (m, 2H), 6.82–6.80 (m, 2H), 6.77–7.73 (m, 1H), 4.07 (q, J = 7.1 Hz, 2H), 4.02–3.95 (m, 4H), 3.87–3.84 (m, 3H), 2.08 (ddt, J = 14.0, 12.3, 3.9 Hz, 2H), 1.96 (ddd, J = 14.0, 5.6, 2.7 Hz, 2H), 1.21 (t, J = 7.1 Hz, 3H). ^{13}C NMR (101 MHz, $CDCl_3$): δ 175.3 (C=O), 147.4 (Cq), 129.7 ($2xCH_{Ar}$), 118.0 (CH_{Ar}), 113.71 ($2xCH_{Ar}$), 71.4 ($2xCH_2$), 60.4 (CH_2), 49.5 (2xCH), 37.6 ($\underline{CH}$-CO_2Et), 28.1 ($2xCH_2$), 14.3 (CH_3).

FT-IR (cm^{-1}, neat): 2958, 2851, 1721, 1595, 1497, 1258, 1112, 1187, 1033, 920, 748, 688. HRMS (ESI) m/z: $[M+H]^+$ Calcd for $C_{16}H_{22}NO_3$ 275.1516; Found 275.1512. Mp 54.5–55.6 °C (measured on the mixture).

O3-tert-Butyl O7-ethyl 9-phenyl-3,9-diazabicyclo[3.3.1]nonane-3,7-dicarboxylate (8l)

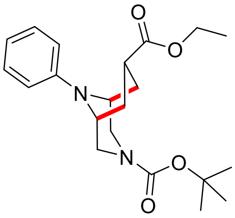

8l
$C_{21}H_{30}N_2O_4$
MW: 374,4810

Following **General Procedure C** with *tert*-butyl 4-phenylpiperazine-1-carboxylate **6l** (52.5 mg, 0.20 mmol) and ethyl 2-(acetoxymethyl)prop-2-enoate (37.9 mg, 0.22 mmol) reacting for 20 minutes. Purification by flash column chromatography (SiO_2, heptanes/EtOAc 90:10) afforded the product as a colorless oil (48.2 mg, 0.13 mmol, 64%, α/β >12:1).

Characterization of the α diastereoisomer:

^1H NMR (400 MHz, $CDCl_3$): δ 7.29–7.21 (m, 2H), 6.89–6.80 (m, 2H), 6.76 (tt, J = 7.2, 1.1 Hz, 1H), 4.16–4.00 (m, 2H), 4.10 (q, J = 7.1 Hz, 2H), 4.00–3.75 (br s, 2H), 3.09 (br d, J = 12.4 Hz, 2H), 2.46 (pent, J = 6.7 Hz, 1H, -$\underline{CH}$-CO_2Et), 2.27 (pent, J = 7.4, 2H), 2.09 (ddd, J = 14.2, 6.5, 2.7 Hz, 2H),

1.49 (s, 9H), 1.24 (t, J = 7.2 Hz, 3H). ^{13}C NMR (101 MHz, $CDCl_3$): δ 175.0 (C=O), 156.2 (C=O), 148.6 (Cq), 129.79 (2xCH_{Ar}), 118.3 (CH_{Ar}), 114.4 (2xCH_{Ar}), 80.1 (Cq), 60.56 (CH_2), 48.4 (CH_2), 47.8 (2xCH), 47.4 (CH_2), 35.4 (C̲H-CO_2Et), 28.5 (3xCH_3), 27.1 (2xCH_2), 14.25 (CH_3). FT-IR (cm^{-1}, neat): 2976, 2928, 2859, 1723, 1693, 1596, 1500, 1388, 1365, 1277, 1172, 1123, 1075, 1035, 918, 749, 729, 689. HRMS (ESI) m/z: $[M+H]^+$ Calcd for $C_{21}H_{31}N_2O_4$ 375.2278; Found 375.2269. R_f = 0.34 (heptanes/EtOAc 80:20).

Characteristic peaks of the β diastereoisomer:

^1H NMR (400 MHz, $CDCl_3$): δ 3.38–3.20 (m, 2H), 1.48 (s, 9H), 1.20 (t, J = 7.2 Hz, 3H). ^{13}C NMR (101 MHz, $CDCl_3$): δ 174.0 (C=O), 155.1 (C=O), 147.5 (Cq), 129.75 (2xCH_{Ar}), 80.3 (Cq), 60.5 (CH_2), 36.1 (C̲H-CO_2Et), 28.6 (3xCH_3), 14.27 (CH_3).

Ethyl 9-phenyl-3-thia-9-azabicyclo[3.3.1]nonane-7-carboxylate (8m)

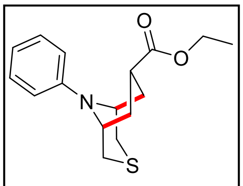

8m
$C_{16}H_{21}NO_2S$
MW: 291,4090

Following **General Procedure C** with 4-phenylthiomorpholine **6m** (35.9 mg, 0.20 mmol) and ethyl 2-(acetoxymethyl)prop-2-enoate (37.9 mg, 0.22 mmol) reacting for 20 minutes. Purification by flash column chromatography (SiO_2, pentane/Et_2O 90:10) afforded the product as a colorless oil (33.7 mg, 0.12 mmol, 58%, α/β 7:1).

Characterization of the α diastereoisomer:

^1H NMR (400 MHz, $CDCl_3$): δ 7.24 (dd, J = 8.9, 7.3 Hz, 2H), 6.84 (d, J = 8.3 Hz, 2H), 6.74 (t, J = 7.2 Hz, 1H), 4.43–4.33 (m, 2H), 4.15 (q, J = 7.1 Hz, 2H), 3.20 (dd, J = 13.2, 3.0 Hz, 2H), 2.49 (tt, J_{ax-ax} = 11.4, J_{ax-eq} = 5.4 Hz, 1H, -CH̲-CO_2Et), 2.44–2.32 (m, 2H), 2.32–2.15 (m, 2H), 2.12–2.02 (m, 2H), 1.26 (t, J = 7.2 Hz, 3H). ^{13}C NMR (101 MHz, $CDCl_3$): δ 175.2 (C=O), 150.0 (Cq), 129.7 (2xCH_{Ar}), 118.0 (CH_{Ar}), 115.0 (2xCH_{Ar}), 60.5 (CH_2), 47.4 (2xCH), 35.5 (CH), 31.0 (2xCH_2), 28.2 (2xCH_2), 14.4 (CH_3). FT-IR (cm^{-1}, neat): 2924, 2866, 1723, 1594, 1497, 1392, 1272, 1173, 1034, 748, 689. HRMS (ESI) m/z: $[M+H]^+$ Calcd for $C_{16}H_{22}NO_2S$ 292.1366; Found 292.1363. R_f = 0.25 (pentane/Et_2O 90:10).

Characteristic peaks of the β diastereoisomer:

^1H NMR (400 MHz, $CDCl_3$): δ 4.57 (tt, J_{ax-ax} = 11.3, J_{ax-eq} = 5.9 Hz, 1H, -C̲H̲-CO_2Et), 4.33–4.27 (m, 2H), 4.10 (q, J = 7.0 Hz, 2H), 3.40 (ddd, J = 13.5, 3.8, 1.5 Hz, 2H), 2.32–2.15 (m, 2H), 1.21 (t, J = 7.2 Hz, 3H). ^{13}C NMR (101 MHz, $CDCl_3$): δ 175.2 (C=O), 148.1 (Cq), 117.9 (CH_{Ar}), 114.6 (2xCH_{Ar}), 60.4 (CH_2), 47.2 (2xCH), 36.6 (C̲H-CO_2Et), 28.3 (2xCH_2), 14.4 (CH_3).

Ethyl 10-phenyl-10-azabicyclo[4.3.1]decane-8-carboxylate (8n)

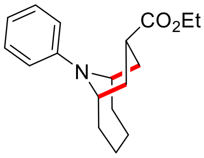

8n
$C_{18}H_{25}NO_2$
MW: 287,4030

Following **General Procedure C** with 1-phenylazepane **6n** (35.1 mg, 0.20 mmol) and ethyl 2-(acetoxymethyl)prop-2-enoate (37.9 mg, 0.22 mmol) reacting for 14 hours. Purification by flash column chromatography (SiO_2, heptanes/AcOEt 98:2) afforded the product as a pale-yellow oil (14.6 mg, 0.05 mmol, 25%, α/β >20:1). The yield could not be increased by using a two-step procedure. The chemical shifts

(^{1}H NMR) and coupling constants are compatible with depicted configuration and conformation.

Characterization of the α diastereoisomer:

^{1}H NMR (300 MHz, $CDCl_3$): δ 7.19 (dd, J = 8.7, 7.1 Hz, 2H), 6.78 (d, J = 8.4 Hz, 2H), 6.62 (t, J = 7.2 Hz, 1H), 4.27–4.18 (m, 2H), 4.13 (q, J = 7.1 Hz, 2H), 2.49 (tt, $J_{ax\text{-}ax}$ = 11.9, $J_{ax\text{-}eq}$ = 5.7 Hz, 1H, -CH-CO_2Et), 2.29–2.15 (m, 4H), 1.82 (td, J = 12.8, 6.0 Hz, 2H), 1.63–1.47 (m, 6H), 1.25 (t, J = 7.1 Hz, 3H). ^{13}C NMR (75 MHz, $CDCl_3$): δ 176.2 (C=O), 149.9 (Cq), 129.5 (2xCH_{Ar}), 115.7 (CH_{Ar}), 111.9 (2xCH_{Ar}), 60.6 (CH_2), 50.5 (2xCH), 36.9 (CH, CH-CO_2Et), 34.9 (2xCH_2), 29.7 (2xCH_2), 25.9 (2xCH_2), 14.4 (CH_3). FT-IR (cm^{-1}, neat): 2924, 2858, 1728, 1594, 1501, 1178, 1155, 1039, 744, 690. HRMS (ESI) m/z: [M+H]$^+$ Calcd for $C_{18}H_{26}NO_2$ 288.1958; Found 288.1966. R_f = 0.50 (heptanes/EtOAc 90:10)

Diethyl 9-phenyl-9-azabicyclo[3.3.1]nonane-3,7-dicarboxylate (14)

Protocol 1 from *N*,*N*-dimethyl aniline (0.5 mmol scale)

In an oven-dried vial were added under N_2 ethyl 2-(acetoxymethyl)prop-2-enoate (94.7 mg, 0.55 mmol), [Ir{dF(CF_3)ppy}$_2$(dtbpy)]PF_6 (5.6 mg, 0.005 mmol), and cesium acetate (230 mg, 1.20 mmol, 2.4 equiv.). The vial was evacuated and filled with nitrogen (x 3). *N*,*N*-dimethylaniline (63 µL, 0.50 mmol) and degassed 1,2-dichloroethane (10.0 mL) were added. The resulting yellow solution was placed in front of a 390 nm LED and stirred for 15 min. At this point a solution of ethyl 2-(acetoxymethyl)prop-2-enoate (94.7 mg, 0.55 mmol) in degassed DCE (0.5 mL) was added and the reaction mixture was stirred for one more hour. Upon completion of reaction the reaction mixture was diluted with sat. $NaHCO_3$ (15 mL). The aqueous phase was extracted with DCM (3 x 15 mL). The combined organic phases were washed once with brine, dried over Na_2SO_4, filtered and concentrated. Flash column chromatography (Si_2O, pentane/Et_2O 90:10 to 80:20) afforded the desired product as a colorless oil (22.5 mg, 0.065 mmol, 13%, β,α/β,β 20:1).

Protocol 2 from N-methyl-N-(trimethylsilylmethyl)aniline (0.5 mmol scale)

Identical as protocol 1 except for the base: cesium acetate (115 mg, 0.60 mmol, 1.2 equiv.) is used. The desired product was obtained as a colorless oil (38.1 mg, 0.11 mmol, 22 %, β,α/β,β 14:1).

Characterization of the β,α diastereoisomer:

^{1}H NMR (400 MHz, $CDCl_3$): δ 7.22 (dd, J = 8.9, 7.2 Hz, 2H), 6.85 (d, J = 7.9 Hz, 2H), 6.71 (tt, J = 7.2, 1.1 Hz, 1H), 4.37–4.27 (m, 2H), 4.13 (q, J = 7.1 Hz, 2H), 4.10 (q, J = 7.2 Hz, 2H), 3.03 (tt, $J_{ax\text{-}ax}$ = 12.5, $J_{ax\text{-}eq}$ = 4.6 Hz, 1H, 1x -CH-CO_2Et), 2.51–2.37 (m, 3H), 1.89 (td, J = 12.9, 4.0 Hz, 2H), 1.83–1.74 (m, 2H), 1.71–1.56 (m, 2H), 1.25 (t, J = 7.1 Hz, 3H), 1.23 (t, J = 7.1 Hz, 3H). ^{13}C NMR (101 MHz, $CDCl_3$): δ 175.6 (C=O), 175.5 (C=O), 150.0 (Cq), 129.7 (2xCH_{Ar}), 117.8 (CH_{Ar}), 114.5 (2xCH_{Ar}), 60.64 (CH_2), 60.59 (CH_2), 46.7 (2xCH), 34.6 (CH), 33.0 (2xCH_2), 32.6 (CH), 28.7 (2xCH_2), 14.4 (2xCH_3). FT-IR (neat, cm^{-1}): 2978, 2950, 1724, 1594, 1498, 1256, 1176, 1032, 749, 731, 690. HRMS (ESI) m/z: [M+H]$^+$ Calcd for $C_{20}H_{28}NO_4$ 346.2013; Found 346.2006. [M+Na]$^+$ Calcd for $C_{20}H_{27}NO_4Na$ 368.1832; Found 368.1826. R_f = 0.29 (pentane/Et_2O 84:16).

Characteristic peaks of the β,β diastereoisomer:

^{1}H NMR (400 MHz, $CDCl_3$): *δ* 4.25–4.19 (m, 4H), 3.16 (tt, $J_{ax\text{-}ax}$ = 12.2, $J_{ax\text{-}eq}$ = 5.8 Hz, 2H, 2x -<u>CH</u>-CO_2Et), 2.14 (td, *J* = 13.0, 5.4 Hz, 4H). ^{13}C NMR (101 MHz, $CDCl_3$): *δ* 175.0 (C=O), 148.0 (Cq), 129.6 (CH_{Ar}), 117.5 (2xCH_{Ar}), 114.2 (2xCH_{Ar}), 47.6 (CH), 37.5 (CH), 30.4 (CH_2).

Unsuccessful substrates

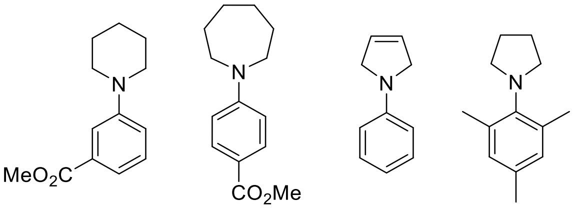

Characterization of the mono-allylated intermediates

Ethyl 2-[(1-phenylpyrrolidin-2-yl)methyl]prop-2-enoate (2a)

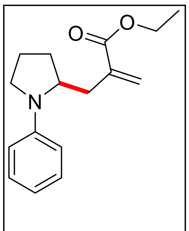

2a
$C_{16}H_{21}NO_2$
MW: 259,3490

In a 10 mL oven-dried vial were added under N_2 ethyl 2-(acetoxymethyl)prop-2-enoate (72.4 mg, 0.42 mmol), [Ir{dF(CF_3)ppy}$_2$(dtbpy)]PF_6 (3.4 mg, 0.030 mmol), and cesium acetate (69 mg, 0.36 mmol). The vial was evacuated and filled with N_2 (x 3), and 1-phenylpyrrolidine **1a** (0.043 mL, 0.30 mmol) was added *via* a Hamilton syringe. Degassed 1,2-dichloroethane (6.00 mL) was added. The resulting yellow solution was placed in front of a 390 nm LED and stirred for 15 minutes. The reaction mixture was diluted with sat. $NaHCO_3$ (5 mL) and the aqueous phase was extracted with DCM (3 x 7 mL). The combined organic phases were dried over Na_2SO_4, filtered, and concentrated under reduced pressure to give crude product as an orange oil. Purification by flash column chromatography (SiO_2, heptanes/EtOAc 99.5:0.5) afforded the mono-allylated product as a colorless oil (33.0 mg, 0.13 mmol, 42%).

^{1}H NMR (300 MHz, $CDCl_3$): δ 7.25 (t, J = 7.8 Hz, 2H), 6.78 (d, J = 8.1 Hz, 2H), 6.68 (t, J = 7.3 Hz, 1H), 6.27 (d, J = 1.5 Hz, 1H), 5.60 (br s, 1H), 4.33–4.21 (m, 2H), 4.01–3.91 (m, 1H), 3.47–3.32 (m, 1H), 3.19 (td, J = 9.2, 7.2 Hz, 1H), 2.93 (dd, J = 13.7, 2.3 Hz, 1H), 2.12 (m, J = 13.7, 10.4 Hz, 1H), 2.08–1.94 (m, 2H), 1.91–1.81 (m, 2H), 1.37 (t, J = 7.1 Hz, 3H). ^{13}C NMR (101 MHz, $CDCl_3$): δ 167.3 (C=O), 147.2 (Cq), 138.7 (Cq), 129.4 (2xCH_{Ar}), 127.7 (=CH_2), 115.6 (CH_{Ar}), 112.1 (2xCH_{Ar}), 61.1 (CH_2), 57.4 (CH), 48.4 (CH_2), 35.5 (CH_2), 29.1 (CH_2), 23.2 (CH_2), 14.4 (CH_3). FT-IR (cm^{-1}, neat): 2967, 1709, 1596, 1504, 1363, 1177, 1159, 1139, 991, 743, 692, 512. HRMS (ESI) m/z: [M+H]$^+$ Calcd for $C_{16}H_{22}O_2N$ 260.1643; Found 260.1645. R_f = 0.23 (heptanes/EtOAc 95:5).

Ethyl 2-[(5-methyl-1-phenyl-pyrrolidin-2-yl)methyl]prop-2-enoate (2f)

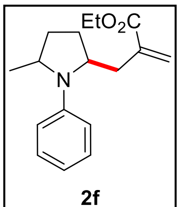

2f
$C_{17}H_{23}NO_2$
MW: 273,3760

In an oven-dried vial were added under N_2 2-methyl-1-phenyl-pyrrolidine **1f** (161 mg, 1.00 mmol), ethyl 2-(acetoxymethyl)prop-2-enoate (207 mg, 1.20 mmol), [Ir{dF(CF_3)ppy}$_2$(dtbpy)]PF_6 (5.6 mg, 0.005 mmol) and cesium acetate (231 mg, 1.20 mmol). The vial was evacuated and filled with nitrogen (x 3). Degassed 1,2-dichloroethane (20 mL) was added. The mixture was placed in front of a 390 nm LED and stirred for 10 minutes. The reaction mixture was diluted with sat. $NaHCO_3$ (15 mL) and the aqueous phase was extracted with DCM (3 x 20 mL). The combined organic phases were dried over Na_2SO_4, filtered, and concentrated under reduced pressure to give crude product as an orange oil. Purification by flash column chromatography (SiO_2, pentane/Et_2O 40:1) afforded the mono-allylated product as a colorless oil and inseparable mixture of diastereoisomers (130 mg, 0.48 mmol, 48%, *trans*/*cis* = 2.8:1).

R_f = 0.33 (pentane/Et_2O 40:1)

The diastereoisomers were separated on a reverse phase preparative system (acidic conditions).
The mixture of diastereoisomers (130 mg) was dissolved in a mix of solution D [100% MeCN + 1% TFA] and solution A [100% H_2O + 1% TFA]. The 15-mL solution was centrifugated and loaded on a Waters PrepLC System (15% D in A to 65% D in A over 50 minutes at a flow rate of 20 mL/min). The minor diastereoisomer was recovered with 28% D in A. The major diastereoisomer was recovered with 47% D in A. The solvent was evaporated on a lyophilisation system overnight and the TFA-salt was taken in EtOAc (10 mL) and aq. sat. $NaHCO_3$ (10 mL). The phases were separated and the aqueous phase extracted with EtOAc (2 x 10 mL). A short filtration on silica gel afforded the clean fractions.
Description of the trans diastereoisomer:
Appearance: white solid
^{1}H NMR (400 MHz, $CDCl_3$): δ 7.24 (td, *J* = 7.8, 7.3, 1.5 Hz, 2H), 6.78 (d, *J* = 7.9 Hz, 2H), 6.63 (tt, *J* = 7.3, 1.1 Hz, 1H), 6.27 (d, *J* = 1.5 Hz, 1H), 5.58 (br s, 1H), 4.33–4.21 (m, 2H), 4.11–4.01 (m, 2H, including N-C*H*Me), 2.97 (br dd, *J* = 13.8, 3.2 Hz, 1H), 2.20 (tt, *J* = 11.9, 7.2 Hz, 1H), 2.11–2.00 (m, 1H), 1.98 (dd, *J* = 13.8, 10.5 Hz, 1H), 1.82 (dd, *J* = 12.4, 6.7 Hz, 1H), 1.64 (dd, *J* = 12.0, 6.6 Hz, 1H), 1.38 (t, *J* = 7.2 Hz, 3H), 1.12 (d, *J* = 6.1 Hz, 3H, N-CH*Me*). ^{13}C NMR (101 MHz, $CDCl_3$): δ 167.3 (C=O), 145.1 (Cq), 138.9 (Cq), 129.3 (2xCH_{Ar}), 127.6 (=CH_2), 115.1 (CH_{Ar}), 113.7 (2xCH_{Ar}), 61.0 (CH_2), 56.5 (CH), 53.0 (CH), 33.9 (CH_2), 30.2 (CH_2), 26.3 (CH_2), 18.2 (CH_3), 14.4 (CH_3). FT-IR (cm^{-1}, neat): 2960, 2927, 1709, 1596, 1502, 1359, 1181, 1147, 744, 694. HRMS (ESI) m/z: $[M+H]^+$ Calcd for $C_{17}H_{24}O_2N$ 274.1802; Found 274.1794. Mp 36.9–37.7 °C. R_f = 0.33 (pentane/Et_2O 40:1)
Description of the cis diastereoisomer:
Appearance: colorless oil
^{1}H NMR (400 MHz, $CDCl_3$): δ 7.29–7.19 (dd, *J* = 8.9, 7.2 Hz, 2H), 6.80 (d, *J* = 7.6 Hz, 2H), 6.67 (tt, *J* = 7.3, 1.1 Hz, 1H), 6.26 (d, *J* = 1.5 Hz, 1H), 5.62 (br s, 1H), 4.35–4.19 (m, 2H), 3.90 (ddt, *J* = 10.8, 7.6, 4.0 Hz, 1H), 3.80 (hx, *J* = 6.2 Hz, 1H, N-C*H*Me), 3.05 (ddd, *J* = 13.7, 3.5, 1.4 Hz, 1H), 2.16 (dd, *J* = 13.7, 10.3 Hz, 1H), 2.07 (dtd, *J* = 12.0, 7.2, 6.0 Hz, 1H), 1.92–1.75 (m, 2H), 1.70 (ddt, *J* = 12.3, 8.2, Hz, 1H), 1.37 (t, *J* = 7.2 Hz, 3H), 1.29 (d, *J* = 6.1 Hz, 3H, N-CH-*Me*). ^{13}C NMR (101 MHz, $CDCl_3$): δ 167.3 (C=O), 147.5 (Cq), 138.6 (Cq), 129.3 (2xCH_{Ar}), 127.6 (=CH_2), 115.8 (CH_{Ar}), 112.3 (2xCH_{Ar}), 61.1 (CH_2), 59.8 (CH), 55.9 (CH), 38.11 (CH_2), 32.4 (CH_2), 28.8 (CH_2), 21.9 (CH_3), 14.4 (CH_3). FT-IR (cm^{-1}, neat): 2962, 1709, 1595, 1501, 1354, 1147, 744, 694. HRMS (ESI) m/z: $[M+H]^+$ Calcd for $C_{17}H_{24}O_2N$ 274.1802; Found 274.1793. R_f = 0.33 (pentane/Et_2O 40:1).
Attribution of the relative configuration:
Literature reports: with respect to the *cis* isomer, the ring proton α to the nitrogen and the methyl group in the *trans* product will appear at lower field (higher ppm), and the methyl group will appear at higher field (lower ppm). No exceptions to this rule are known.[19,20]

	trans-1-Phenyl-2,5-dimethylpyrrolidine	cis-1-Phenyl-2,5-dimethylpyrrolidine	Product Major dia	Product Minor dia
N-C*H*Me	m, 3.98 (lower field)	m, 3.75	m, 4.06 (lower field)	m, 3.79
N-CH*Me*	d, 1.08 (higher field)	d, 1.27	d, 1.12 (higher field)	d, 1.29
	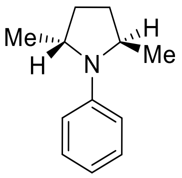	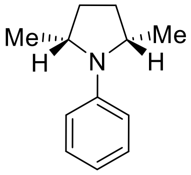	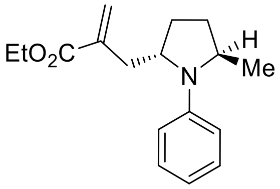	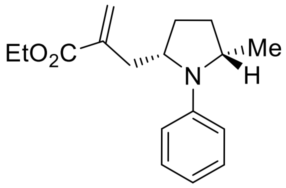

Hence, it can be concluded that, in our case, the major diastereoisomer is the *trans* product and the minor diastereoisomer the *cis* product.

Ethyl 2-[(1-phenylazepan-2-yl)methyl]prop-2-enoate (7n)

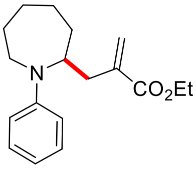

7n
$C_{18}H_{25}NO_2$
MW: 287,4030

In an oven-dry vial were added under N_2 1-phenylazepane **6n** (87.6 mg, 0.50 mmol), ethyl 2-(acetoxymethyl)prop-2-enoate (94.7 mg, 0.55 mmol), [Ir{dF(CF_3)ppy}$_2$(dtbpy)]PF_6 (5.6 mg, 0.005 mmol), and cesium acetate (115 mg, 0.6 mmol). The vial was evacuated and filled with nitrogen (x 3). Degassed 1,2-dichloroethane (10 mL) was added. The resulting yellow solution was placed in front of a 390 nm LED and stirred for 10 min. The reaction mixture was diluted with sat. $NaHCO_3$ (10 mL) and the aqueous phase was extracted with DCM (3 x 10 mL); combined organic phases were dried over Na_2SO_4, filtered, and concentrated under reduced pressure to give crude product as an orange oil. Purification by flash column chromatography (SiO_2, pentane/Et_2O 99:1 to 98:2) afforded the mono-allylated product as a colorless oil (93.1 mg, 0.33 mmol, 65%). Some cyclized product was also recovered at this point (2.6 mg, 2%), hence the necessity to control very closely the reaction.

^{1}H NMR (300 MHz, $CDCl_3$): δ 7.29–7.16 (m, 2H), 6.83 (d, J = 8.1 Hz, 2H), 6.62 (tt, J = 7.2, 1.0 Hz, 1H), 6.19 (d, J = 1.6 Hz, 1H), 5.60 (br s, 1H), 4.31–4.19 (m, 2H), 3.95 (dq, J = 15.1, 5.2 Hz, 1H), 3.54–3.41 (m, 1H), 3.31–3.12 (m, 1H), 2.77 (ddd, J = 13.5, 5.0, 1.4 Hz, 1H), 2.29 (dd, J = 13.4, 9.3 Hz, 1H), 2.07 (ddd, J = 14.4, 8.3, 6.2 Hz, 1H), 1.92–1.79 (m, 1H), 1.79–1.71 (m, 1H), 1.71–1.60 (m, 2H), 1.42–1.15 (m, 3H), 1.35 (t, J = 7.1 Hz, 3H). ^{13}C NMR (75 MHz, $CDCl_3$): δ 167.4 (C=O), 148.5 (Cq), 138.2 (Cq), 129.4 (2xCH_{Ar}), 127.3 (=CH_2), 114.8 (CH_{Ar}), 110.6 (2xCH_{Ar}), 61.0 (CH_2), 55.6 (CH), 43.0 (CH_2), 36.2 (CH_2), 34.8 (CH_2), 30.2 (CH_2), 27.2 (CH_2), 25.2 (CH_2), 14.3 (CH_3). FT-IR (cm^{-1}, neat): 2925, 2851, 1708, 1594, 1400, 1164, 1135, 908, 729, 692. HRMS (ESI) m/z: $[M+H]^+$ Calcd for $C_{18}H_{26}O_2N$ 288.1958. Found 288.1956. $[M+Na]^+$ Calcd for $C_{18}H_{25}O_2NNa$ 310.1778; Found 310.1775. R_f = 0.34 (heptanes/EtOAc 95:5).

Methyl 4-[2-(2-ethoxycarbonylallyl)azepan-1-yl]benzoate (7o)

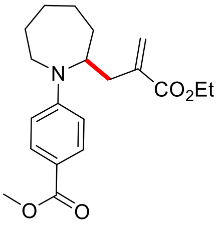

7o
$C_{20}H_{27}NO_4$
MW: 345,4390

In an oven-dry vial were added under N_2 methyl 4-(azepan-1-yl)benzoate **6o** (93.3 mg, 0.40 mmol), ethyl 2-(acetoxymethyl)prop-2-enoate (75.8 mg, 0.44 mmol), [Ir{dF(CF_3)ppy}$_2$(dtbpy)]PF_6 (4.5 mg, 0.04 mmol), and cesium acetate (92.3 mg, 0.48 mmol). The vial was evacuated and filled with nitrogen (x 3). Degassed 1,2-dichloroethane (8.00 mL) was added. The resulting yellow solution was placed in front of a 390 nm LED and stirred for 9 h. The reaction mixture was diluted with sat. $NaHCO_3$ (10 mL) and the aqueous phase was extracted with DCM (3 x 8 mL). The combined organic phases were dried over Na_2SO_4, filtered, and concentrated under reduced pressure to give crude product as an orange oil. Purification by flash column chromatography (SiO_2, pentane/Et_2O 80:20) afforded the mono-allylated product as a colorless oil (34.4 mg, 0.10 mmol, 25%) along with 26% unreacted starting material.

^{1}H NMR (300 MHz, $CDCl_3$): δ 7.85–7.80 (m, 2H), 6.57–6.51 (m, 2H), 6.20 (d, J = 1.4 Hz, 1H), 5.58 (q, J = 1.2 Hz, 1H), 4.24–4.17 (m, 2H), 3.84 (s, 3H), 3.71–3.61 (m, 1H), 3.38 (t, J = 6.7 Hz, 2H), 2.60 (ddd, J = 13.9, 7.3, 1.0 Hz, 1H), 2.45 (ddd, J = 13.8, 6.1, 0.9 Hz, 1H), 1.89–1.78 (m, 2H), 1.68–1.56 (m, 1H), 1.53–1.33 (m, 5H), 1.29 (t, J = 7.2 Hz, 3H). ^{13}C NMR (101 MHz, $CDCl_3$): δ 167.5 (C=O), 151.7 (Cq), 137.8 (Cq), 131.8 (2xCH_{Ar}), 127.8 (=CH_2), 118.1 (Cq), 111.7 (2xCH_{Ar}), 61.2 (CH_2), 52.4 (CH), 51.6 (CH_3), 37.8 (CH_2), 34.8 (CH_2), 33.9 (CH_2), 32.7 (CH_2), 28.2 (CH_2), 25.3 (CH_2), 14.3 (CH_3). FT-IR (cm^{-1}, neat): 3367, 2935, 1703, 1600, 1525, 1432, 1271, 1171, 1106, 769. HRMS (ESI) m/z: $[M+H]^+$ Calcd for $C_{20}H_{28}O_4N$ 346.2013; Found 346.2014. R_f = 0.36 (pentane/Et_2O 60:40).

Methyl 4-[2-[(E)-2-ethoxycarbonylbut-2-enyl]-1-piperidyl]benzoate (9)

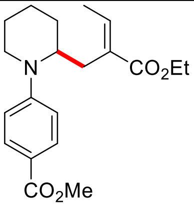

$C_{20}H_{27}NO_4$
MW: 345,4390

In an oven-dry vial were added under N_2 methyl 4-(1-piperidyl)benzoate **6g** (110 mg, 0.50 mmol), ethyl 3-acetoxy-2-methylene-butanoate (37.2 mg, 0.20 mmol), [Ir{dF(CF_3)ppy}$_2$(dtbpy)]PF_6 (2.2 mg, 0.002 mmol), and CsOAc (46.1 mg, 0.24 mmol). The vial was evacuated and filled with nitrogen (x 3). Degassed 1,2-dichloroethane (4.0 mL) was added. The resulting yellow solution was placed in front of a 390 nm LED and stirred for **10 min**. The reaction mixture was diluted with sat. $NaHCO_3$ (5 mL) and the aqueous phase was extracted with DCM (3 x 5 mL). The combined organic phases were dried over Na_2SO_4, filtered, and concentrated under reduced pressure to give crude product as an orange oil. Purification by flash column chromatography (SiO_2, pentane/Et_2O 90:10) afforded the mono-allylated product as a colorless oil (49.9 mg, 0.14 mmol, 73%, *E*/*Z* = 7:1). Crystallization from pentane at 4 °C afforded the product as a white solid, enriched in the *E* isomer (*E*/*Z* = 20/1).

^1H NMR (300 MHz, $CDCl_3$): δ 7.90–7.82 (m, 2H), 6.90–6.72 (m, 3H), 4.35–4.27 (m, 1H), 4.18 (q, J = 7.1 Hz, 2H), 3.84 (s, 3H), 3.65–3.56 (m, 1H), 3.11 (td, J = 12.6, 3.1 Hz, 1H), 2.67 (dd, J = 13.6, 9.0 Hz, 1H), 2.52 (dd, J = 13.5, 5.9 Hz, 1H), 1.79 (d, J = 7.2 Hz, 3H), 1.78–1.48 (m, 6H), 1.28 (t, J = 7.1 Hz, 3H). ^{13}C NMR (101 MHz, $CDCl_3$): δ 167.8 (C=O), 167.4 (C=O), 154.0 (Cq), 139.3 (=CH), 131.8 (2xCH_{Ar}), 130.9 (Cq), 118.1 (Cq), 113.5 (2xCH_{Ar}), 60.7 (CH_2), 53.8 (CH), 51.6 (CH_3), 42.1 (CH_2), 27.7 (CH_2), 25.5 (CH_2), 25.3 (CH_2), 19.3 (CH_2), 14.8 (CH_3), 14.4 (CH_3). FT-IR (cm^{-1}, neat): 2932, 2850, 1700, 1599, 1515, 1432, 1247, 1185, 1163, 1106, 768. HRMS (ESI) m/z: $[M+H]^+$ Calcd for $C_{20}H_{28}O_4N$ 346.2013. Found 346.2016. Calcd for $C_{20}H_{27}O_4NNa$ $[M+Na]^+$ 368.1832; Found 368.1835. Mp 52.3–54.1 °C. R_f = 0.33 (pentane/Et_2O 80:20).

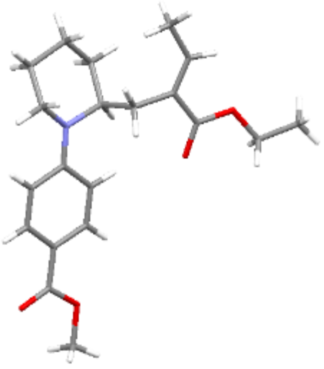

X-Ray: a sample was dissolved in the minimum amount of pentane with gentle heating, and the solution was then placed at 4 °C overnight to afford single crystals suitable for X-Ray diffraction analysis.

Study of the cyclization step

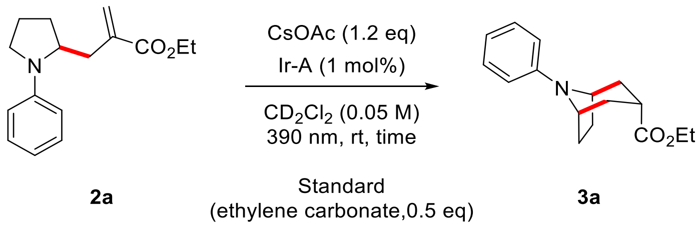

General Procedure D

In a dry NMR tube were added ethyl 2-[(1-phenylpyrrolidin-2-yl)methyl]prop-2-enoate **2a** (6.5 mg, 0.025 mmol), $[Ir\{dF(CF_3)ppy\}_2(dtbpy)]PF_6$ (0.3 mg, 0.27 x 10^{-3} mmol), 1,3-dioxolan-2-one (1.1 mg, 0.0125 mmol, standard) and cesium acetate (5.8 mg, 0.03 mmol). The tube was evacuated and filled with N_2 (x 3), and dry CD_2Cl_2 (0.5 mL) was added. The resulting yellow solution was degassed by freeze-pump-thaw cycles (x 3) and a first NMR measurement was made before irradiation to determine the exact standard-to-starting material ratio. The tube was then placed in front of a 390 nm LED and measurements were taken regularly (see table).

Supplementary Table 5
Cyclization study

	% conversion of **2a**	% yield of **3a**
t1: 3min	15	7
t2: 10min	24	18
t3: 20 min	41	26
t4: 40min	56	41
t5: 60min	65	48
t6: 90min	80	53
t7: 120min	87	58
t8: 150 min	94	66
t9: 180 min	100	72

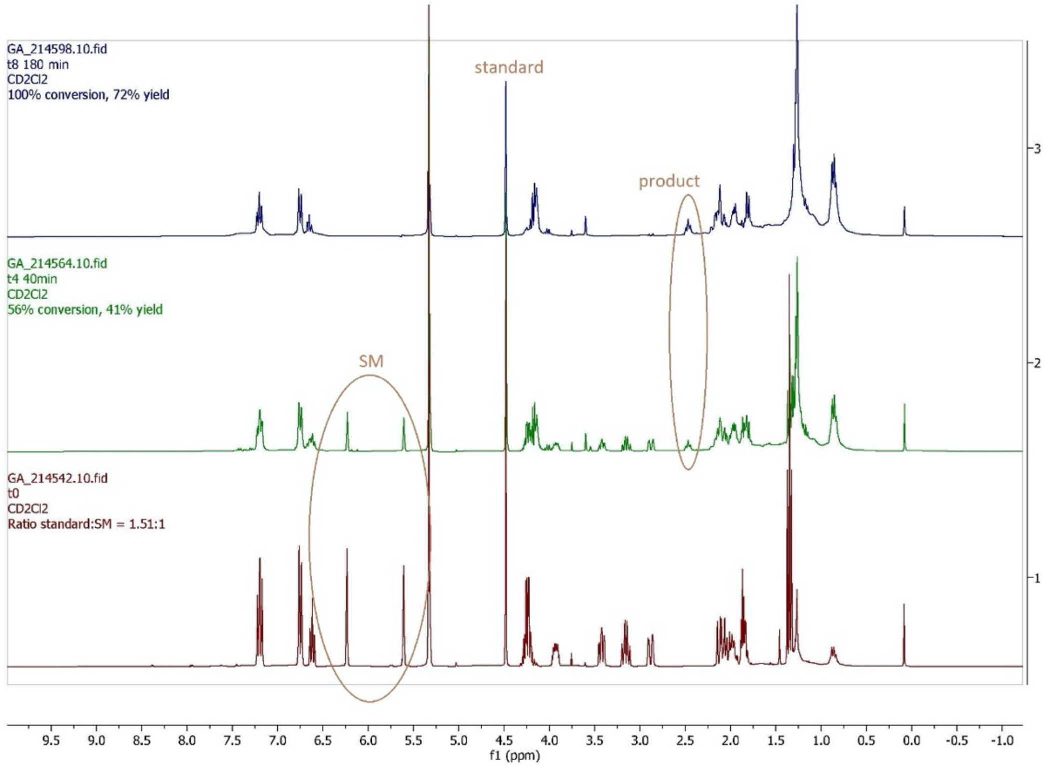

The ratio conversion/yield was not satisfying, and the reaction was very slow compared to the one-pot process, in which acetic acid is generated from the OAc-trap. The tested reaction conditions are summarized in Supplementary Table 6.

Supplementary Table 6
Cyclization study, role of additive

Entry	Deviation to general procedure D	NMR yield	Time	remarks
**1**	None	72%	3 h	Much slower than the one-pot process[a]
**2**	No catalyst	--	24 h	No product visible, degradation
**4**	AcOH (1.0 eq)	91%	3 min	dr 9:1[b]
**5**	CsPiv instead of CsOAc	Quant.	25 min	dr 5:1
**6**	CsPiv, AcOH (1.0 eq)	Quant.	< 2 min	dr 5:1

[a] Major diastereoisomer only. [b] The Stability of product under the reaction conditions was studied (stable, not reacting).

Synthesis of bicyclic compounds from monoallylated cyclic amines

The previously optimized conditions (see above) were applied on a substituted pyrrolidine derivative to study the influence of an alpha-substituent on the cyclization reaction.

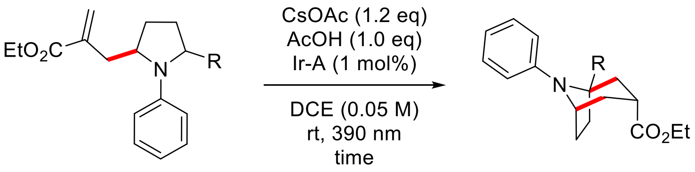

R = H, 5 min, **77%**, α/β 88:12
R = Me, 30 min, **66%**, α/β 82:18

Modified General Procedure D

In an oven-dry vial were added under N_2 the mono-allylated starting material (0.20 mmol, 1.0 equiv.), [Ir{dF(CF_3)ppy}$_2$(dtbpy)]PF_6 (2.2 mg, 0.02 mmol, 1 mol%), and cesium acetate (46 mg, 0.24 mmol, 1.2 equiv.). The vial was evacuated and filled with N_2 (x 3), then CH_3CO_2H (11.4 µL, 0.20 mmol, 1.0 equiv.) and degassed 1,2-dichloroethane (4 mL, 0.05 M) were successively added. The resulting yellow solution was placed in front of a 390 nm LED and stirred until consumption of the starting material (TLC or NMR monitoring). The reaction mixture was diluted with sat. $NaHCO_3$ (3 mL). The aqueous phase was extracted with DCM (3 x 5 mL) and the combined organic phases were dried over Na_2SO_4, filtered, and concentrated under reduced pressure to give crude product as an orange oil. Purification by flash column chromatography on silica gel afforded the bicyclic product.

Ethyl 8-phenyl-8-azabicyclo[3.2.1]octane-3-carboxylate (3a)

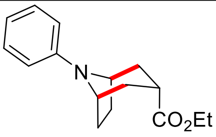

3a

Following **modified General Procedure D** with ethyl 2-[(1-phenylpyrrolidin-2-yl)methyl]prop-2-enoate **2a** (19.2 mg, 0.07 mmol), reacting for 5 minutes. Purification by flash column chromatography (SiO_2, heptanes/EtOAc 98:2) afforded the title compound as a white solid (14.0 mg, 0.054 mmol, 77%, α/β 88:12). Spectra in accordance with previously described product.

Ethyl 1-methyl-8-phenyl-8-azabicyclo[3.2.1]octane-3-carboxylate (3f)

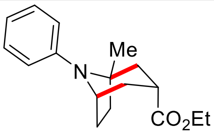

3f

Following **modified General Procedure D** with ethyl 2-[(5-methyl-1-phenyl-pyrrolidin-2-yl)methyl]prop-2-enoate **2f** (54.7 mg, 0.20 mmol, dr 3:1), reacting for 30 minutes. Purification by flash column chromatography (SiO_2, pentane/Et_2O 95:5) afforded the title compound as a white solid (35.8 mg, 0.13 mmol, 66%, α/β 82:18). Spectra in accordance with previously described product.

Ethyl 10-phenyl-10-azabicyclo[4.3.1]decane-8-carboxylate (8n)

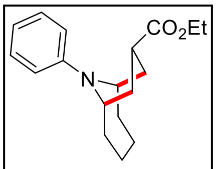

8n

Following **modified General Procedure D** with ethyl 2-[(1-phenylazepan-2-yl)methyl]prop-2-enoate **7n** (57.5 mg, 0.20 mmol), reacting for 30 minutes. Purification by flash column chromatography (SiO_2, pentane/Et_2O 99:1 to 98:2) afforded the product as a colorless oil (13.8 mg, 0.048 mmol, 24%, α/β >20:1) along with recovered starting material (5.2 mg, 9% recovered SM). Spectra in accordance with previously described product.

Ethyl 9-(4-methoxycarbonylphenyl)-2-methyl-9-azabicyclo[3.3.1]nonane-3-carboxylate (10)

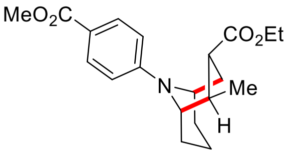

10
$C_{20}H_{27}NO_4$
MW: 345,4390

Following **modified General Procedure D** with methyl 4-[2-[(E)-2-ethoxycarbonylbut-2-enyl]-1-piperidyl]benzoate **9** (69.1 mg, 0.20 mmol), reacting for 4 hours (NMR follow-up). Purification by flash column chromatography (SiO_2, pentane/Et_2O 80:20) afforded the product as a colorless oil (45.4 mg, 0.13 mmol, 66%, dr >20:1).

^{1}H NMR (400 MHz, $CDCl_3$): δ 7.90–7.83 (m, 2H), 6.81–6.75 (m, 2H), 4.36–4.25 (m, 1H), 4.17–4.09 (m, 2H), 3.86–3.83 (m, 1H), 3.85 (s, 3H), 2.35 (ddd, J = 13.3, 10.9, 4.3 Hz, 1H), 2.14–1.95 (m, 3H), 1.77–1.64 (m, 3H), 1.64–1.49 (m, 2H), 1.45 (ddd, J = 13.9, 4.6, 2.1 Hz, 1H), 1.24 (t, J = 7.1 Hz, 3H), 1.07 (d, J = 6.1 Hz, 3H). ^{13}C NMR (75 MHz, $CDCl_3$): δ 175.8 (C=O), 167.3 (C=O), 153.5 (Cq), 131.9 (2xCH_{Ar}), 117.5 (Cq), 112.4 (2xCH_{Ar}), 60.5 (CH_2), 56.5 (CH), 51.6 (CH_3), 46.5 (CH), 43.4 (CH), 35.9 (CH), 29.8 (CH_2), 29.4 (CH_2), 28.9 (CH_2), 21.8 (CH_3), 14.8 (CH_2), 14.4 (CH_3). FT-IR (cm^{-1}, neat): 2932, 1705, 1599, 1514, 1433, 1279, 1253, 1182, 1107, 1060, 768, 729, 697. HRMS (ESI) m/z: Calcd for $C_{19}H_{28}NO_4$ 346.2013; Found 346.2004. R_f = 0.33 (pentane/Et_2O 80:20).

Derivatization of 10 for X-ray crystallography

[4-[3-(hydroxymethyl)-2-methyl-9-azabicyclo[3.3.1]nonan-9-yl]phenyl]methanol (11)

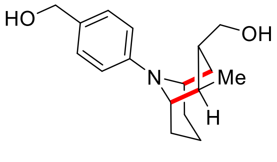

$C_{17}H_{25}NO_2$
MW: 275,3920

In a 25 mL two-neck round-bottom flask equipped with a reflux condenser and a rubber septum were added THF (3 mL) and $LiAlH_4$ (58 mg, 1.5 mmol). A solution of **10** (0.5 mmol, 173 mg, dr > 95:5) in THF (2 mL) was added dropwise at room temperature and the reaction mixture was stirred for 1 h. Upon completion of the reaction (TLC monitoring), the reaction mixture was cooled to 0 °C and 2 M aqueous NaOH was added to the mixture dropwise until a thick white precipitate was obtained (around 1 mL was necessary). The mixture was refluxed for 1 h, allowed to cool down to room temperature and filtered, rinsing with Et_2O. The mother liquor was dried over Na_2SO_4, filtered and concentrated under reduced pressure. The crude residue was purified by flash column chromatography (SiO_2, pentane/Et_2O 20:80) to afford the product as a thick colorless oil (97.0 mg, 0.35 mmol, 70%). The product was crystallized by slow evaporation from a heptanes/EtOAc or pentane/Et_2O mixture to give a white crystalline solid suitable for X-Ray analysis. Note that the product is not stable in $CDCl_3$.

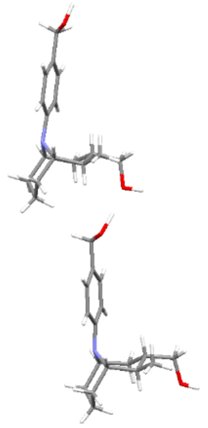

^1H NMR (400 MHz, C_6D_6): 7.19 (d, J = 8.7 Hz, 2H), 6.75 (d, J = 8.7 Hz, 2H), 4.43 (s, 2H), 4.02–3.92 (m, 1H), 3.59–3.54 (m, 1H), 3.32–3.22 (m, 2H), 1.98 (ddd, J = 12.2, 10.8, 4.3 Hz, 1H), 1.78 (qt, J = 13.1, 4.3 Hz, 1H), 1.64–1.56 (m, 2H), 1.45–1.08 (m, 6H), 0.90 (d, J = 6.8 Hz, 3H). ^{13}C NMR (101 MHz, C_6D_6): δ 150.8 (Cq), 129.8 (Cq), 129.1 (2xCH_{Ar}), 114.3 (2xCH_{Ar}), 65.3 (CH_2), 64.6 (CH_2), 57.8 (CH), 47.2 (CH), 38.4 (CH), 34.9 (CH), 30.28 (CH_2), 29.4 (CH_2), 29.3 (CH_2), 21.6 (CH_3), 15.2 (CH_2). FT-IR (cm^{-1}, neat): 3319, 2916, 2865, 1611, 1512, 1398, 1287, 1256, 1187, 999, 793. HRMS (ESI) m/z: $[M+H]^+$ Calcd for $C_{17}H_{26}NO_2$ 276.1958. Found 276.1932. Mp 101.5–103.2 °C. R_f = 0.29 (heptanes/EtOAc 55:45).

Oxidation of the BPin derivative

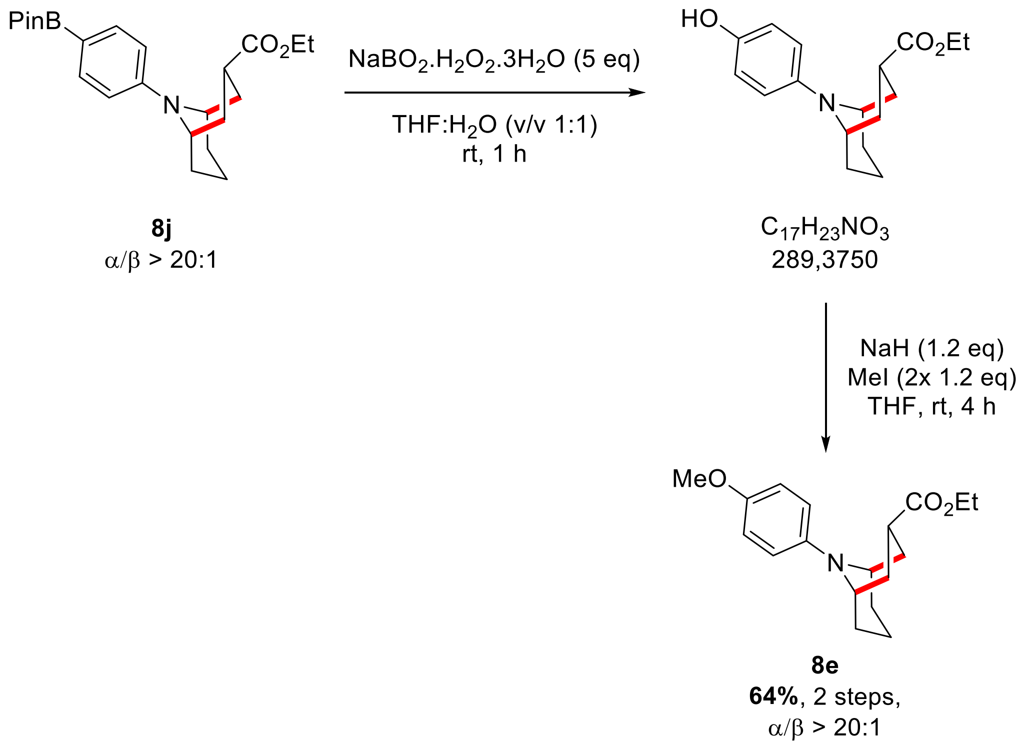

Ethyl 9-(4-hydroxyphenyl)-9-azabicyclo[3.3.1]nonane-3-carboxylate

To a suspension of ethyl 9-[4-(4,4,5,5-tetramethyl-1,3,2-dioxaborolan-2-yl)phenyl]-9-azabicyclo[3.3.1]nonane-3-carboxylate **8j** (120 mg, 0.30 mmol) in THF/H_2O (2.5 mL, v/v 1:1) was added sodium perborate (231 mg, 1.50 mmol) in one portion. The reaction mixture gradually turned bright pink. Upon completion (TLC monitoring, approx. 1h), the reaction mixture was quenched by addition of saturated aq. NH_4Cl (10 mL). The aqueous layer was extracted with Et_2O (3 x 10 mL) and the combined organic extracts were dried over Na_2SO_4, filtered and concentrated in vacuo. The crude residue was purified by flash column chromatography (SiO_2, pentane/Et_2O 70:30) to afford the ethyl 9-(4-hydroxyphenyl)-9-azabicyclo[3.3.1]nonane-3-carboxylate intermediate as a pink sticky oil, from which the residual solvents proved to be difficult to remove. Additionally, extreme broadening of the signals was observed in the NMR spectra (^{1}H and ^{13}C), hence it could not be directly characterized, and solvent could not be quantified.

Partial characterization of ethyl 9-(4-hydroxyphenyl)-9-azabicyclo[3.3.1]nonane-3-carboxylate:

FT-IR (neat, cm^{-1}): 3388, 2927, 1725, 1701, 1507, 1246, 1178, 908, 811, 729. HRMS (ESI) m/z: [M+H]$^+$ Calcd for $C_{17}H_{24}O_3N$ 290.1751; Found 290.1746.

The structure of this compound was assessed after methylation to form known compound **8e** according to the following procedure:

Ethyl 9-(4-methoxyphenyl)-9-azabicyclo[3.3.1]nonane-3-carboxylate (8e)

In an oven-dried seal tube was added THF (4 mL) followed by NaH (12.9 mg, 0.34 mmol, 55–60 % in mineral oil). Then, a solution of ethyl 9-(4-hydroxyphenyl)-9-azabicyclo[3.3.1]nonane-3-carboxylate previously obtained (estimated quantitative yield, 0.3 mmol engaged) in THF (4 mL, pink solution) was added dropwise. The mixture became dark green/blue and was stirred at rt for 20 minutes before addition of iodomethane (21 µL, 0.34 mmol). The reaction mixture was stirred at rt for 3 hours without reaching completion, hence another portion of iodomethane (21 µL, 0.34 mmol) was added. Approximately 2 hours after the second addition, the starting material was fully converted. The reaction mixture was quenched with water (5 mL) and extracted with EtOAc (2 x 10 mL). The organic extract was washed with brine (10 mL), dried over Na_2SO_4, and concentrated in vacuo. Purification by flash column chromatography (SiO_2, pentane/Et_2O 90:10) afforded the product as a crystalline white solid (57.9 mg, 0.19 mmol, 64% over two steps. α/β >20:1). Spectral data are in accordance with previously described product. ^{1}H NMR (300 MHz, $CDCl_3$): δ 6.80 (s, 4H), 4.17–4.10 (m, 2H), 4.10 (q, J = 7.1 Hz, 2H), 3.75 (s, 3H), 2.47 (tt, J = 12.4, 5.9 Hz, 1H), 2.42–2.26 (m, 2H), 2.00 (qt, J = 13.3, 4.4 Hz, 1H), 1.79 (tt, J = 13.2, 4.3 Hz, 2H), 1.65 (ddd, J = 14.0, 12.3, 3.5 Hz, 2H), 1.58–1.49 (m, 1H), 1.49–1.39 (m, 2H), 1.23 (t, J = 7.1 Hz, 3H). ^{13}C NMR (101 MHz, $CDCl_3$): δ 176.1 (C=O), 151.6 (Cq), 145.3 (Cq), 115.7 (2xCH_{Ar}), 115.1 (2xCH_{Ar}), 60.4 (CH_2), 55.9 (CH_3), 47.8 (2xCH), 34.7 (CH), 30.6 (2xCH_2), 28.4 (2xCH_2), 14.4 (CH_3), 14.3 (CH_2). FT-IR (cm^{-1}, neat): 2870, 1726, 1507, 1241, 1177, 1039, 910, 812, 731, 616. HRMS (ESI) m/z: $[M+H]^+$ Calcd for $C_{18}H_{26}NO_3$ 304.1907; Found 304.1899. Mp 42.5–43.7 °C. R_f = 0.31 (pentane/Et_2O 90:10).

Epimerization (from the α to the β diastereoisomer)

Ethyl 8-phenyl-8-azabicyclo[3.2.1]octane-3-carboxylate (3aβ, *exo* product):

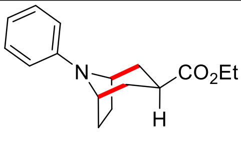

3a

To a solution of ethyl 8-phenyl-8-azabicyclo[3.2.1]octane-3-carboxylate **3aα** (α/β 5:1, 130 mg, 0.50 mmol) in absolute ethanol (4 mL) was added dropwise a solution of NaOEt in EtOH (2.7 M, 0.20 mL, 0.54 mmol).The reaction mixture was heated at 40 °C for 3 hours. TLC indicated complete epimerization and volatiles were evaporated under reduced pressure. The crude residue was purified by flash column chromatography (SiO_2, heptanes/EtOAc 90:10) to afford the desired product as a light yellow crystalline solid (61.7 mg, 0.40 mmol, 79%, α/β 1:20).

^{1}H NMR (300 MHz, $CDCl_3$): δ 7.25–7.19 (m, 2H), 6.77 (d, J = 8.1 Hz, 2H), 6.70 (tt, J = 7.3, 1.1 Hz, 1H), 4.32–4.25 (m, 2H), 4.05 (q, J = 7.1 Hz, 2H), 2.89 (tt, J = 12.3, 5.3 Hz, 1H, -CH-CO_2Et), 2.16–2.07 (m, 2H), 2.07–1.97 (m, 2H), 1.85–1.75 (m, 2H), 1.64 (ddd, J = 13.8, 6.0, 2.9 Hz, 2H), 1.20 (t, J = 7.2 Hz, 3H).

Coupling constants of CH-CO_2Et compatible with an axial proton on a chair conformation ($J_{ax\text{-}ax}$ = 12.3 Hz, $J_{ax\text{-}eq}$ = 5.3 Hz).

^{13}C NMR (75 MHz, $CDCl_3$): *δ* 175.1 (C=O), 146.2 (Cq), 129.7 (2xCH_{Ar}), 117.2 (CH_{Ar}), 115.2 (2xCH_{Ar}), 60.5 (CH_2), 53.7 (2xCH), 35.5 (C̲H-CO_2Et), 29.3 (2xCH_2), 28.3 (2xCH_2), 14.3 (CH_3). FT-IR (cm^{-1}, neat): 2957, 1722, 1597, 1501, 1365, 1179, 1045, 937, 754, 694, 583. HRMS (ESI) m/z: $[M+H]^+$ Calcd for $C_{16}H_{22}NO_2$ 260.1645; Found 260.1642. Mp 71.5–73.1 °C. R_f = 0.22 (pentane/Et_2O 96:4).

Ethyl 9-phenyl-9-azabicyclo[3.3.1]nonane-3-carboxylate (8aβ, exo product)

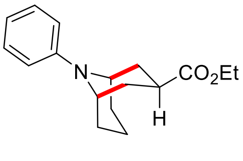

8a
$C_{17}H_{23}NO_2$
MW: 273,3760

To a solution of ethyl 9-phenyl-9-azabicyclo[3.3.1]nonane-3-carboxylate **8aα** (α/β >20:1, 122 mg, 0.45 mmol) in absolute ethanol (4 mL) was added dropwise a solution of NaOEt in EtOH (2.7 M, 0.17 mL, 0.46 mmol). The reaction mixture was stirred at rt for 1 hour and, in absence of epimerization, for 3 hours at 40 °C. TLC indicated complete epimerization and volatiles were evaporated under reduced pressure. The crude residue was purified by flash column chromatography (SiO_2, pentane/Et_2O 94:6) to afford the desired product as a white crystalline solid (92.7 mg, 0.34 mmol, 76%, α/β 1:8). For easier characterization of the β product, the diastereoisomers were separated by flash column chromatography (SiO_2, pentane/Et_2O 95:5).

Characterization of the β diastereoisomer:

^1H NMR (300 MHz, $CDCl_3$): *δ* 7.29–7.17 (m, 2H), 6.84 (d, *J* = 8.3 Hz, 2H), 6.68 (t, *J* = 7.2 Hz, 1H), 4.18–4.03 (m, 4H), 3.32 (tt, $J_{ax\text{-}ax}$ = 12.1, $J_{ax\text{-}eq}$ = 5.7 Hz, 1H, -C̲H̲-CO_2Et), 2.20–1.98 (m, 5H), 1.93 (ddd, *J* = 13.9, 5.7, 2.0 Hz, 2H), 1.76–1.62 (m, 3H), 1.23 (t, *J* = 7.1 Hz, 3H). ^{13}C NMR (75 MHz, $CDCl_3$): *δ* 175.5 (C=O), 148.7 (Cq), 129.6 (2xCH_{Ar}), 116.9 (CH_{Ar}), 114.0 (2xCH_{Ar}), 60.5 (CH_2), 48.0 (2xCH), 37.8 (C̲H-CO_2Et), 31.0 (2xCH_2), 27.9 (2xCH_2), 20.4 (CH_2), 14.4 (CH_3). FT-IR (cm^{-1}, neat): 2935, 1725, 1592, 1500, 1189, 1111, 1036, 908, 746, 689. HRMS (ESI) m/z: $[M+H]^+$ Calcd for $C_{17}H_{24}NO_2$ 274.1802; Found 274.1800. Mp 74.4–75.0 °C. R_f = 0.25 (pentane/Et_2O 94:6).

Deprotection of the aryl moiety

General procedure E: deprotection of the aryl moiety[21]

To a solution of *N*-aryl precursor (0.20 mmol) in MeCN/H_2O (12 mL, v/v 3:1) was added cerium ammonium nitrate (329 mg, 0.60 mmol) at 0 °C. The resulting clear yellow solution was stirred at rt overnight. Upon completion of the reaction, $NaBH_4$ (11.3 mg, 0.30 mmol) was added in one portion at 0 °C. *This should be done carefully as $NaBH_4$ can react violently under the aqueous acidic conditions of the reaction mixture*. After addition, the reaction mixture was stirred at rt for 30 min and TLC indicated full reduction of the quinone by-product. K_2CO_3 (powder, ~800 mg, 6.00 mmol) was added to the reaction mixture until pH = 11. At this point the reaction mixture was a dark red solution with a white precipitate on the bottom of the flask, however stirring was not prevented. An excess of benzyl chloroformate (Cbz-Cl) (0.14 mL, 1.0 mmol) was then added and the reaction mixture was stirred at rt for 2 h. The reaction mixture was transferred to a separatory funnel filtrating the white precipitate

over cotton and rinsing with Et_2O. Water (15 mL) was added and the mixture extracted with Et_2O (3 x 15 mL). The combined organic phases were washed with aq. sat. Na_2CO_3 (1 x 15 mL) and brine (1 x 15 mL), dried over anhydrous Na_2SO_4 and the solvent was removed in vacuo to give a dark red liquid. Flash column chromatography on silica gel afforded the desired products.

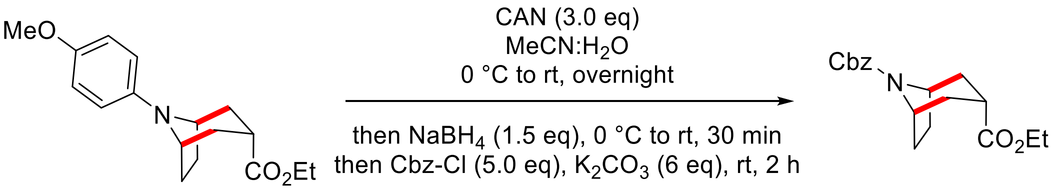

3d
α/β 4:1

12
90%, α/β 5:1

$C_{18}H_{23}NO_4$
MW: 317,3850

O8-Benzyl O3-ethyl 8-azabicyclo[3.2.1]octane-3,8-dicarboxylate (12)

The title product was prepared following **General Procedure E** with ethyl 8-(4-methoxyphenyl)-8-azabicyclo[3.2.1]octane-3-carboxylate **3d** (57.9 mg, 0.20 mmol, α/β 4:1). Flash column chromatography (SiO_2, pentane/Et_2O 90:10 to 80:20) afforded **12** as a pale-yellow oil (57.0 mg, 0.18 mmol, 90%, α/β 5:1).

Characterization of the α diastereoisomer at 25 °C:

^1H NMR (300 MHz, $CDCl_3$, 25 °C): δ 7.43–7.27 (m, 5H), 5.14 (s, 2H), 4.26 (br m, 2H), 4.19 (q, J = 7.1 Hz, 2H), 2.61 (tt, J_{ax-eq} = 8.1, J_{eq-eq} = 1.4 Hz, 1H, -CH-CO_2Et), 2.29 (dq, J = 14.6, 1.4 Hz, 2H), 2.21–1.58 (m, 6H, br signals), 1.28 (t, J = 7.1 Hz, 3H). ^{13}C NMR (75 MHz, $CDCl_3$, **25 °C**): δ 175.7 (C=O), 153.5 (C=O), 137.1 (Cq), 128.6 (2xCH_{Ar}), 128.03 (CH_{Ar}), 127.94 (2xCH_{Ar}), 66.7 (CH_2), 61.1 (CH_2), 53.0 (2xCH), 34.2 (CH), 31.3 (CH_2, br signal), 30.7 (CH_2, br signal), 27.4 (CH_2, br signal), 26.6 (CH_2, br signal), 14.3 (CH_3).

Characteristic peaks of the β diastereoisomer at 25 °C:

^1H NMR (300 MHz, $CDCl_3$, 25 °C): δ 7.43–7.27 (m, 5H), 5.14 (s, 2H), 4.37 (br m, 2H), 4.11 (q, J = 7.2 Hz, 2H), 2.81 (tt, J_{ax-ax} = 11.8, J_{ax-eq} = 5.6 Hz, 1H, -CH-CO_2Et), 1.22 (t, J = 7.1 Hz, 3H). ^{13}C NMR (75 MHz, $CDCl_3$, 25 °C): δ 174.8 (C=O), 137.0 (Cq), 66.9 (CH_2), 60.7 (CH_2), 53.2 (CH), 34.9 (CH).

FT-IR (neat, cm^{-1}): 2961, 1723, 1695, 1414, 1300, 1183, 1095, 1068, 1044, 729, 696. HRMS (ESI) m/z: $[M+Na]^+$ Calcd for $C_{18}H_{23}O_4NNa$ 340.1519; Found 340.1514. R_f = 0.33 (pentane/Et_2O 3:1)

Note: an NMR measured in C_6D_6 at 70 °C showed that free rotation around the C-N bond was still not fully achieved, hence extreme broadening of the signals was observed.

In order to obtain well defined signals, several measurements were made from 25 °C to –45 °C in $CDCl_3$. At –45 °C the rotation around the C-N bond was fully impaired and well-defined NMR spectra could be recorded (^1H, ^{13}C, HSQC, COSY):

Characterization of the α diastereoisomer at –45 °C:

^{1}H NMR (400 MHz, $CDCl_3$, –45 °C): δ 7.48–7.28 (m, 5H), 5.27–5.02 (m, 2H), 4.28–4.24 (m, 1H), 4.24–4.20 (m, 1H), 4.17 (q, J = 7.2 Hz, 2H), 2.63 (t, J_{ax-eq} = 7.8 Hz, 1H, -CH-CO_2Et), 2.30 (ddd, J = 14.1, 7.0, 2.3 Hz, 2H), 2.07 (ddd, J = 14.3, 8.0, 3.3 Hz, 1H), 2.03–1.53 (m, 5H), 1.28 (t, J = 7.1 Hz, 3H). ^{13}C NMR (101 MHz, $CDCl_3$, –45 °C): δ 175.7 (C=O), 153.1 (C=O), 136.5 (Cq), 128.5 (2xCH_{Ar}), 128.0 (CH_{Ar}), 127.9 (2xCH_{Ar}), 66.6 (CH_2), 61.2 (CH_2), 52.6 (CH), 52.5 (CH), 33.8 (CH), 30.8 (CH_2), 30.3 (CH_2), 27.0 (CH_2), 26.2 (CH_2), 14.20 (CH_3).

Characteristic peaks of the β diastereoisomer at –45 °C:

^{1}H NMR (400 MHz, $CDCl_3$, –45 °C): δ 7.48–7.28 (m, 5H), 4.39–4.35 (m, 1H), 4.34–4.29 (m, 1H), 4.08 (q, J = 7.2 Hz, 2H), 2.81 (tt, J_{ax-ax} = 11.9, J_{ax-eq} = 5.5 Hz, 1H, -CH-CO_2Et), 1.23 (t, J = 7.2 Hz, 3H). ^{13}C NMR (101 MHz, $CDCl_3$, –45 °C): δ 175.1 (C=O), 136.4, 127.94, 66.7 (CH_2), 60.8 (CH_2), 52.8 (CH), 52.7 (CH), 34.5 (CH), 33.4 (CH_2), 32.9 (CH_2), 28.2 (CH_2), 27.4 (CH_2), 14.18 (CH_3).

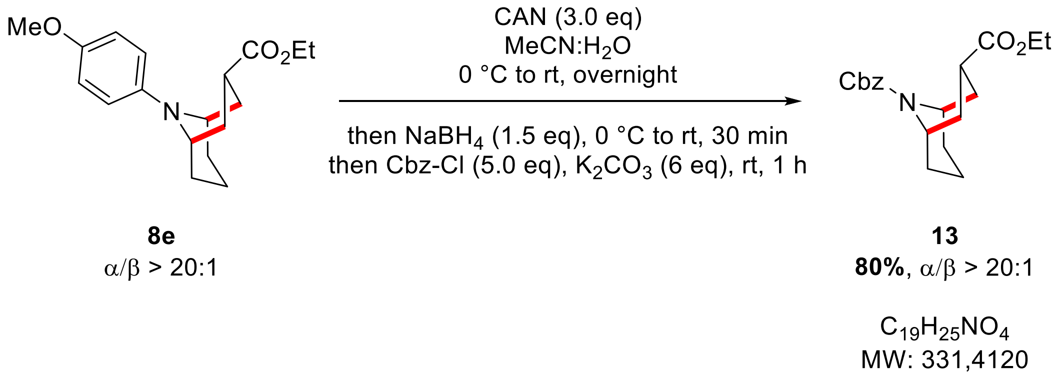

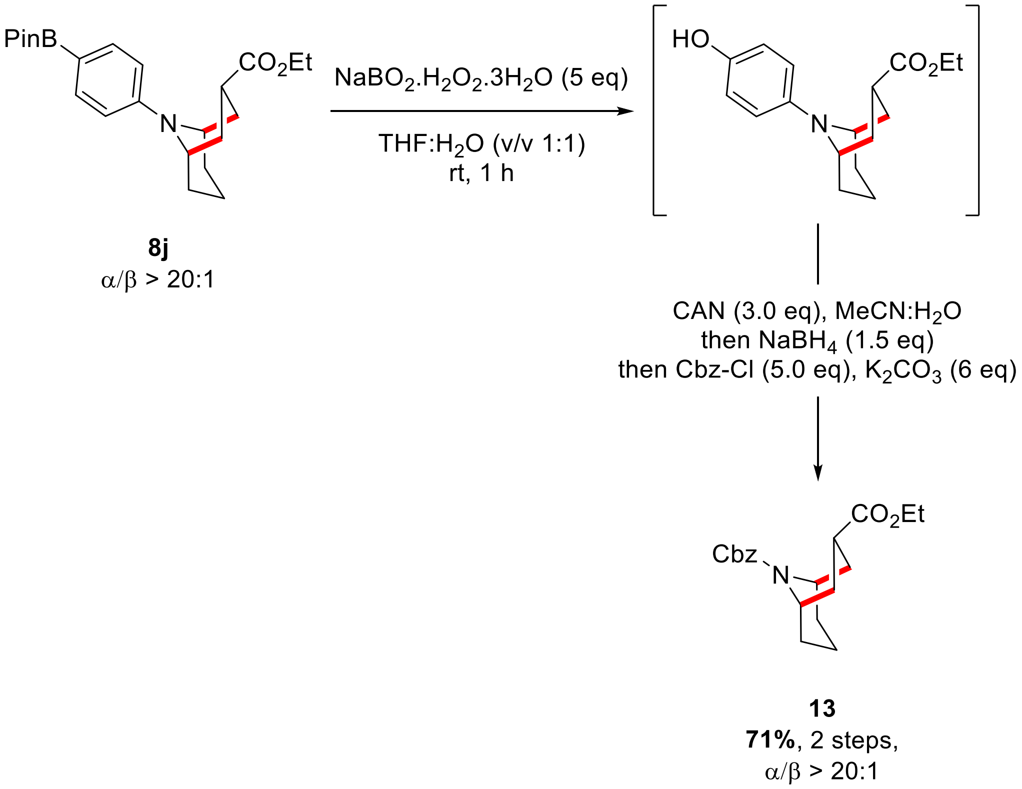

O9-Benzyl O3-ethyl 9-azabicyclo[3.3.1]nonane-3,9-dicarboxylate (13, from 8e)

From the PMP-derivative **8e**. The title compound was prepared following **General Procedure E** with ethyl 9-(4-methoxyphenyl)-9-azabicyclo[3.3.1]nonane-3-carboxylate **8e** (60.7 mg, 0.20 mmol, α/β >20:1). Flash column chromatography (SiO_2, pentane/Et_2O 90:10 to 80:20) afforded the desired product as a pale-yellow oil (53.1 mg, 0.16 mmol, 80%, α/β >20:1).

O9-Benzyl O3-ethyl 9-azabicyclo[3.3.1]nonane-3,9-dicarboxylate (13, from 8j)

To a suspension of ethyl-9-[4-(4,4,5,5-tetramethyl-1,3,2-dioxaborolan-2-yl)phenyl]-9-azabicyclo[3.3.1]nonane-3-carboxylate **8j** (79.9 mg, 0.20 mmol, α/β >20:1) in THF/H_2O (2 mL, v/v 1:1) was added sodium perborate (154 mg, 1.0 mmol) in 1 portion. The reaction mixture gradually turned bright pink. Upon completion (TLC monitoring, ca. 1h), the mixture was quenched by addition of sat. aq. NH_4Cl (10 mL). The aqueous layer was extracted with Et_2O (3 x 10 mL) and the combined organic extracts were dried over Na_2SO_4, filtered and concentrated in vacuo. The crude residue was engaged in the deprotection sequence following **General Procedure E**. Flash column chromatography (SiO_2, pentane/Et_2O 90:10 to 80:20) afforded the desired product as a pale-yellow oil (47.0 mg, 0.14 mmol, 71%, α/β >20:1).

Characterization of the α diastereoisomer (fully impaired rotation at 25 °C):

^{1}H NMR (400 MHz, $CDCl_3$): δ 7.42–7.27 (m, 5H), 5.19–5.07 (m, 2H), 4.59–4.51 (m, 1H), 4.51–4.44 (m, 1H), 4.12 (q, J = 7.1 Hz, 2H), 2.35–2.19 (m, 3H), 1.92 (qt, J = 13.2, 4.4 Hz, 1H), 1.73–1.52 (m, 5H), 1.52–1.38 (m, 2H), 1.24 (t, J = 7.1 Hz, 3H). ^{13}C NMR (75 MHz, $CDCl_3$): δ 175.3 (C=O), 155.3 (C=O), 137.1 (Cq), 128.6 (2xCH_{Ar}), 128.1 (CH_{Ar}), 127.8 (2xCH_{Ar}), 67.1 (CH_2), 60.6 (CH_2), 45.2 (CH), 44.7 (CH), 35.6 (CH), 30.9 (CH_2), 30.5 (CH_2), 28.5 (CH_2), 28.3 (CH_2), 14.3 (CH_3), 13.9 (CH_2). FT-IR (neat, cm^{-1}): 2931, 1728, 1689, 1423, 1323, 1300, 1248, 1176, 1087, 1048, 696. HRMS (ESI) m/z: $[M+H]^+$ Calcd for $C_{19}H_{26}NO_4$ 332.1856; Found 332.1854. R_f = 0.30 (heptanes/EtOAc 80:20).

Characteristic peak of the β diastereoisomer:

^{1}H NMR (400 MHz, $CDCl_3$): δ 3.23 (tt, $J_{ax\text{-}ax}$ = 11.8, $J_{ax\text{-}eq}$ = 6.4 Hz, 1H, -<u>CH</u>-CO_2Et).

Note: an NMR measured in C_6D_6 at 70 °C showed that free rotation around the C-N bond was still not fully achieved and peak broadening of the signals was observed. Hence the product was characterized at room temperature in $CDCl_3$.

Characterization of side-products

Note that these procedures are not optimized procedures. The goal of these experiments was to characterize possible non-desired products for a better optimization of the desired pathway for the model reaction.

Ethyl 2-[[5-(2-ethoxycarbonylallyl)-1-phenyl-pyrrolidin-2-yl]methyl]prop-2-enoate (4a)

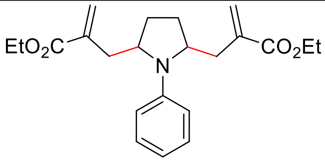

4a
$C_{22}H_{29}NO_4$
MW: 371,4770

In an oven-dried vial were added under N_2 ethyl 2-(benzenesulfonylmethyl)prop-2-enoate (153 mg, 0.60 mmol. 3.0 eq.), [Ir{dF(CF_3)ppy}$_2$(dtbpy)]PF_6 (4.5 mg, 0.04 mmol), and cesium acetate (76.8 mg, 0.400 mmol). The vial was evacuated and refilled with nitrogen (x 3), and 1-phenylpyrrolidine **1a** (0.029 mL, 0.20 mmol) was added *via* a Hamilton syringe. Degassed 1,2-dichloroethane (2.00 mL) was added and the mixture was placed in front of a 390 nm LED and stirred for 2h30. The reaction mixture was diluted with sat. $NaHCO_3$ (3 mL). The aqueous phase was extracted with DCM (3 x 5 mL) and the combined organic phases were dried over Na_2SO_4, filtered, and concentrated under reduced pressure to give crude product as an orange oil. Purification by flash column chromatography (SiO_2, heptanes/EtOAc 95:5) afforded the title compound as a colorless oil (22.4 mg, 0.06 mmol, 30%, dr 4:1, *cis*/*trans* relationship not assigned).

^{1}H NMR (300 MHz, $CDCl_3$): δ 7.32–7.21 (m, 2H, major+minor), 7.02–6.92 (m, 2H, major), 6.80–6.62 (m, 1H, major+minor, and 2H, minor), 6.29–6.23 (m, 2H, major+minor), 5.66–5.62 (m, 2H, minor), 5.56 (q, *J* = 1.1 Hz, 2H, major), 4.32–4.20 (m, 4H, major+minor), 4.09 (ddt, *J* = 10.0, 6.8, 3.6 Hz, 2H, major), 3.97–3.87 (m, 2H, minor), 3.08 (ddd, *J* = 13.3, 3.4, 1.1 Hz, 2H, minor), 2.98 (d, *J* = 13.7 Hz, 2H, major), 2.15 (dd, *J* = 13.6, 10.3 Hz, 2H, minor), 2.06–1.75 (m, 6H, major+minor), 1.375 (t, *J* = 7.2 Hz, 6H, major), 1.370 (t, *J* = 7.2 Hz, 6H, minor). ^{13}C NMR (75 MHz, $CDCl_3$): δ 167.3 (major+minor), 147.5 (minor), 144.8 (major), 138.8 (major), 138.5 (minor), 129.4 (major), 129.3 (minor), 127.7 (major), 127.6 (minor), 116.2 (minor), 115.6 (major), 114.0 (major), 112.5 (minor), 61.08 (minor), 61.05 (major), 59.4 (minor), 56.7 (major), 38.1 (minor), 33.9 (major), 28.3 (minor), 26.0 (major), 14.4 (major+minor). FT-IR (cm^{-1}, neat): 2972, 2900, 1708, 1596, 1503, 1360, 1179, 1151, 745, 698. HRMS (ESI) m/z: $[M+H]^+$ Calcd for $C_{22}H_{30}NO_4$ 372.2169; Found 372.2159. R_f = 0.37 (heptanes/EtOAc 80:20).

Ethyl 1-(2-ethoxycarbonylallyl)-8-phenyl-8-azabicyclo[3.2.1]octane-3-carboxylate

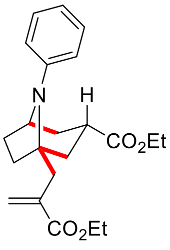

$C_{22}H_{29}NO_4$
MW: 371,4770

Into an oven-dry vial were added under N_2 ethyl 2-[[5-(2-ethoxycarbonylallyl)-1-phenyl-pyrrolidin-2-yl]methyl]prop-2-enoate **4a** (23 mg, 0.06 mmol), [Ir{dF(CF_3)ppy}$_2$(dtbpy)]PF_6 (1.4 mg, 2 mol%), and cesium acetate (23 mg, 0.12 mmol). The vial was evacuated and filled with nitrogen (x 3). Degassed 1,2-dichloroethane (1.00 mL) was added. The resulting yellow solution was placed in front of a 390 nm LED and stirred for 40 min. The reaction mixture was diluted with sat. $NaHCO_3$ (3 mL). The aqueous phase was extracted with DCM (3 x 3 mL) and the combined organic phases were dried over Na_2SO_4, filtered, and concentrated under reduced pressure to give crude product as an orange oil. Purification by flash column chromatography (neutral Alox, 100% heptanes to heptanes/EtOAc 99:1–97:3) afforded a first fraction containing the major diastereoisomer (3.0 mg) and a second fraction containing both diastereoisomers (5.4 mg, α/β 4:1), both as colorless oils (m_{tot} = 8.4 mg, 0.022 mmol, 37%, α/β 85:15). Only the major diastereoisomer was characterized.

^{1}H NMR (300 MHz, $CDCl_3$): δ 7.22 (t, J = 7.8 Hz, 2H), 7.07 (d, J = 8.1 Hz, 2H), 6.82 (t, J = 7.2 Hz, 1H), 6.22 (d, J = 1.6 Hz, 1H), 5.72 (d, J = 1.6 Hz, 1H), 4.26–4.12 (m, 5H), 3.13 (d, J = 15.1 Hz, 1H), 2.70–2.57 (m, 2H), 2.39 (dd, J = 14.2, 8.4 Hz, 1H), 2.20 (d, J = 14.0 Hz, 2H), 2.09–2.01 (m, 2H), 2.00–1.84 (m, 1H), 1.84–1.73 (m, 1H), 1.71–1.60 (m, 1H), 1.30 (t, J = 7.1 Hz, 3H), 1.28 (t, J = 7.1 Hz, 3H). ^{13}C NMR (101 MHz, $CDCl_3$): δ 176.2 (C=O), 168.0 (C=O), 147.3 (Cq), 137.1 (Cq), 129.1 (2xCH_{Ar}), 127.8 (=CH_2), 120.6 (2xCH_{Ar}), 119.5 (CH_{Ar}), 62.6 (Cq), 61.0 (CH_2), 60.6 (CH), 38.9 (CH_2), 35.4 (CH), 34.9 (CH_2), 31.7 (CH_2), 29.9 (CH_2), 27.3 (CH_2), 25.7 (CH_2), 14.36 (CH_3), 14.32 (CH_3). FT-IR (cm^{-1}, neat): 2978, 1715, 1596, 1493, 1370, 1238, 1180, 1087, 1046, 1027, 753, 696. HRMS (ESI) m/z: $[M+H]^+$ Calcd for $C_{22}H_{30}NO_4$ 372.2169; Found 372.2156. R_f = 0.34 (SiO_2, heptanes/EtOAc 85:15).

Ethyl 3-(1-phenylpyrrolidin-2-yl)-2-[(1-phenylpyrrolidin-2-yl)methyl]propanoate (5a)

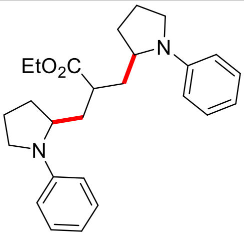

5a
$C_{26}H_{34}N_2O_2$
MW: 406,5700

In an oven-dried vial were added under N_2 ethyl 2-(benzenesulfonylmethyl)prop-2-enoate (50.9 mg, 0.20 mmol), Ir(dtbbpy)(ppy)$_2$]PF_6 (3.7 mg, 0.004 mmol), and cesium acetate (46.1 mg, 0.24 mmol). The vial was evacuated and filled with nitrogen (x 3), and 1-phenylpyrrolidine (0.057 mL, 0.40 mmol) was added via a syringe. 1,2-dichloroethane (2.00 mL) was added. The resulting yellow solution was placed in front of a 390 nm LED and stirred for 16 h. The reaction mixture was diluted with sat. $NaHCO_3$ (5 mL) and the phase were separated. The aqueous phase was extracted with DCM (3 x 5 mL) and the combined organic phases were dried over Na_2SO_4, filtered, and concentrated under reduced pressure to give the crude product as an orange oil. Purification by flash column

chromatography (neutral Alox, heptanes/EtOAc 100:0 to 99:1) afforded the title compound as a colorless oil (22.9 mg, 0.056 mmol, 28%, dr n.d.).

^{1}H NMR (400 MHz, $CDCl_3$): δ 7.25–7.20 (m, 4H, 3 diastereoisomers), 6.69–6.63 (m, 6H, 3 diastereoisomers), 4.33 (q, J = 7.1 Hz, 2H, 1 diastereoisomer), 4.20–4.11 (m, 1H, 1 diastereoisomer), 4.05 (dq, J = 10.4, 6.9 Hz, 1H, 1 diastereoisomer), 3.85 (q, J = 7.1 Hz, 2H, 1 diastereoisomer), 3.82–3.77 (m, 1H, 3 diastereoisomers), 3.65–3.58 (m, 1H, 3 diastereoisomers), 3.46–3.37 (m, 2H, 3 diastereoisomers), 3.19–3.10 (m, 2H, 3 diastereoisomers), 2.44–2.52 (m, 1H, 3 diastereoisomers), 2.26–1.26 (m, 12H, 3 diastereoisomers), 1.41 (t, J = 7.1 Hz, 3H, 1 diastereoisomers), 1.24 (t, J = 7.2 Hz, 3H, 1 diastereoisomers), 1.04 (t, J = 7.2 Hz, 3H, 1 diastereoisomers).

^{13}C NMR (101 MHz, $CDCl_3$): δ 175.4 (C=O, 3 diasteroisomers), 147.3 (Cq, 3 diasteroisomers), 129.4, 115.90, 115.83, 112.14, 112.03, 111.99, 60.9 (CH_2, 1 diasteroisomer), 60.8 (CH_2, 1 diasteroisomer), 60.6 (CH_2, 1 diasteroisomer), 56.8 (3x2CH, 3 diasteroisomers), 48.8 ($2CH_2$, 1 diasteroisomer), 48.54 ($2CH_2$, 1 diasteroisomer), 48.47 ($2CH_2$, 1 diasteroisomer), 41.44 (CH, 1 diasteroisomer), 41.32 (CH, 1 diasteroisomer), 41.28 (CH, 1 diasteroisomer), 36.7, 36.6, 36.2, 35.3, 30.8, 30.7, 30.3, 30.2, 29.8, 23.75, 23.69, 23.5, 14.6 (CH_3, 1 diasteroisomer), 14.3 (CH_3, 1 diasteroisomer), 14.0 (CH_3, 1 diasteroisomer).

FT-IR (cm^{-1}, neat): 2959, 1725, 1596, 1504, 1363, 1344, 1157, 991, 745, 692. HRMS (ESI) m/z: $[M+H]^+$ Calcd for $C_{26}H_{35}N_2O_2$ 407.2693. Found 407.2683. R_f = 0.28 (pentane/Et_2O 96:4).

Methyl 4-[2,6-bis[(Z)-2-ethoxycarbonylbut-2-enyl]-1-piperidyl]benzoate

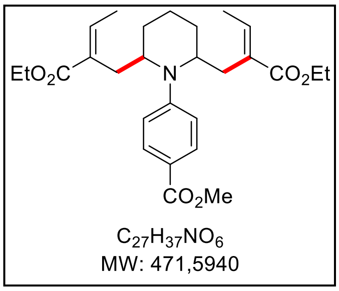

Following **General Procedure C** with ethyl 3-acetoxy-2-methylene-butanoate (41.0 mg, 0.22 mmol) and methyl 4-(1-piperidyl)benzoate (43.9 mg, 0.20 mmol) reacting for 3 hours. Purification by flash column chromatography (SiO_2, heptanes/EtOAc 80:20) followed by trituration in pentane afforded the product as a white solid (14.4 mg, 0.03 mmol, 15%, one diastereoisomer isolated, *cis*/*trans* relationship not assigned).

^{1}H NMR (300 MHz, $CDCl_3$): δ 7.97 (d, J = 8.8 Hz, 2H), 7.32 (d, J = 8.8 Hz, 2H), 7.02 (q, J = 7.1 Hz, 2H), 4.26 (q, J = 7.1 Hz, 4H), 4.19–4.10 (m, 2H), 3.86 (s, 3H), 2.73 (t, J = 13.1 Hz, 2H), 2.56 (dd, J = 13.4, 3.7 Hz, 2H), 1.90 (d, J = 7.2 Hz, 6H), 1.88–1.76 (m, 1H), 1.69–1.44 (m, 5H), 1.36 (t, J = 7.1 Hz, 6H). ^{13}C NMR (101 MHz, $CDCl_3$): δ 167.8 (2xC=O), 167.6 (C=O), 152.3 (Cq), 139.9 (2xCH=), 131.5 (2xCH_{Ar}), 131.1 (2xCq), 118.3 (Cq), 113.7 (2xCH_{Ar}), 60.9 (2xCH_2), 52.0 (2xCH), 51.6 (CH_3), 29.4 (2xCH_2), 26.9 (2xCH_2), 16.2 (CH_2), 15.0 (2xCH_3), 14.5 (2xCH_3). FT-IR (cm^{-1}, neat): 3029, 1691, 1599, 1260, 1192, 1103, 737. HRMS (ESI) m/z: $[M+H]^+$ Calcd for $C_{27}H_{38}O_6N$ 472.2694. Found 472.2677. Mp 126.3–127.5 °C. R_f = 0.14 (pentane/Et_2O 80:20).

Calculations

DFT methodology.

All DFT calculations were performed with the ADF (Amsterdam Density Functional) code developed by E. J. Baerends and co-workers[22] using triple-zeta basis sets (no frozen core). Geometry optimizations were performed *in vacuo* relying on the Generalized Gradient Approximation (GGA) VBP exchange-correlation (XC) potential (VWN + BP: Vosko, Wilk & Nusair[23] + corrective terms by Becke[24] for the exchange, and Perdew[25] for the correlation) with ADF grid precision 6 throughout.

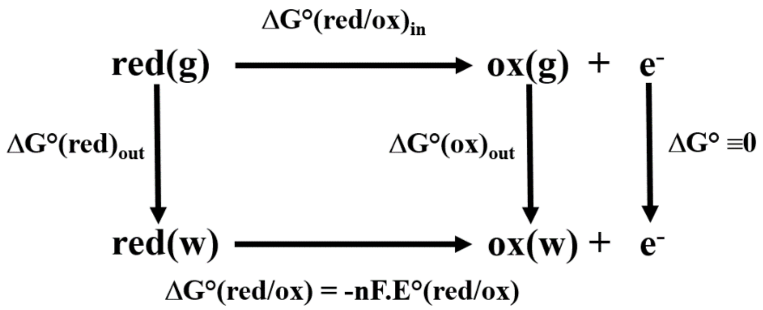

Supplementary Figure 1
Standard Born-Haber cycle for a redox reaction (cf. Eq. S1), with (g) and (w) corresponding to a gas phase and solvent environment, respectively.

Redox potentials

To compute redox potentials $E°_{DFT}$(red/ox) relative to the Standard Hydrogen Electrode (SHE), we relied on the standard Born-Haber cycle (Supplementary Figure 1) for which the standard Gibbs free energy of a redox half reaction consists of the free energy change in the gas phase (ΔG_{in}) and the solvation free energies (ΔG_{out}) of the oxidized (ox) and reduced (red) species of a given redox couple.[26,27]

$-nFE°_{DFT}(red/ox)_{SHE} = \Delta G_{in} + \Delta G_{out} + \Delta G_{SHE}$ (Eq. S1)

ΔG_{in} contains in principle all contributions *intrinsic* to the molecular system itself. These are:

i) the ionization energy of the reduced species, IE(red), which is computed as the enthalpic bonding energy (i.e. electronic internal energy) difference between the two redox partners, either bonding energies $\Delta E_B = E_B(ox)-E_B(red)$ or from total energies $\Delta E_T = E_T(ox)-E_T(red)$ with the VBP XC potential, following Noodleman *et al.*[28] (for a more general treatment, see Batista *et al.*[29,30]). Between bonding and total energies as defined in the ADF code, only the reference changes and disappears in the difference. We will report below total energies E_T.

ii) the internal nuclear energy E_{int} (computed by ADF; keyword "analyticalfreq") which is the sum of the zero-point energy, 3 kT (i.e. (3/2) kT for translation and (3/2) kT for rotation, therefore (1/2) kT for each degree of freedom), and a small correction term due to the vibrational partition function;

iii) the entropic term –TΔS (T set at 298 K) also computed by ADF (keyword "analyticalfreq").

We will verify below that both contributions to redox potentials coming from internal nuclear energies E_{int} and entropic terms $-T\Delta S$ turn out to be minor, as expected: structural changes upon oxidation being minor, they result for these two terms into changes of the order of a few hundredths of Volt) (see for example the work of Volbeda *et al*).[31]

ΔG_{out} contains contributions from the interaction of the complex with its polarizable environment. In the present case, these contributions have been limited to a dichloroethane (DCE, and anecdotically acetonitrile MeCN) solvent outside the complex. Solvation energies E_{env} have been computed with the COSMO (COnductor-like Screening MOdel)[32–34] ADF module, representing the solvent as a dielectric continuum. COSMO solvation energies behave according to a Born-like model, that is, they are proportional to Q^2 (the total cluster charge squared) multiplied by a factor of (1-1/[illegible]). We used the ADF option: "solv name" setting all required solvent parameters to mimick an average reaction field response of the environment.

Next, in Eq. S1, ΔG_{SHE} = -4.43 eV is defined at pH=0[35] (other authors recommend a value of -4.28 eV for theoretical DFT calculations).[36–38] The shift between SHE and Calomel is set at 0.24 V (i.e. $E°_{SCE}$ = $E°_{SHE}$ -0.24 V).

Finally, F is the Faraday constant (here: 1 elementary electric charge) and n=1 for a one-electron oxydation.

pKa constants

pKa values are computed for the following reaction: $AH^+ \rightarrow A + H^+$. As a consequence, there is no ion-pair contribution between A (neutral) and H^+. In addition to the quantities ΔG_{in} and ΔG_{out} defined in Eq.S1, but for free energies computed for AH^+ and A, we only need proton solvation energies $\Delta G(H^+)_{solv}$:

$pKa(AH^+/A) = (\Delta G_{in} + \Delta G_{out} + \Delta G(H^+)_{solv})/(0.0592)$ (Eq. S2)

In DCE, $\Delta G(H^+)_{DCE}$ = -8.91 eV (the calculated standard Gibbs solvation energy of the proton in pure DCE is at **860 kJ.mol^{-1}** and a saturated (0.131m) solution of water in DCE is more basic by 205 kJ.mol^{-1} with $D_{solv}Go(H^+)$ of **1065 kJ.mol^{-1}**).[39] We will use therefore the standard Gibbs solvation energy of the proton in pure DCE, that is -205.6 kcal/mol corresponding to -8.91 eV). Absolute pKa scales in water and DCE are shifted by 42.7 pKa units.[40,41] This shift corresponds to the difference between proton solvation energies in water (-11.44 eV)[39] and in DCE (-8.91 eV) converted into pKa units (i.e. (11.44-8.91)/0.0592 = 42.7). This allows to translate pKa's in DCE into (more familiar) equivalent pKa's in water.

Supplementary Table 7

Total energies ET (1 a.u. = 27.21 eV), solvation energies Esolv (eV), internal (Eint) and entropic (-TS) terms (in eV). In red are the corresponding differences for a given redox couple. Redox potential calculations in DCE (and MeCN) solvent are computer.

Redox couples	E_T (a.u.)	E_{solv} (eV)	E_{int} eV)	-TS (eV)	$E°_{SCE}$ (V)
**1a** (0)	-443.82495	-0.26 (DCE)<br>-0.46 (MeCN)	+5.81	-1.24	**+0.62** (DCE)<br>+0.57 (MeCN) [d]
**RC1a** (+1)°	-443.57324	-1.84 (DCE)<br>-2.09 (MeCN)	+5.82	-1.23	
**1a/ RC1a**	**+6.85**	**-1.58** (DCE)<br>**-1.63** (MeCN)	**+0.01**	**+0.01**	
**1d** (0)	-558.40454	-0.34 (DCE)<br>-0.58 (MeCN)	+6.70	-1.37	+0.29 (DCE)<br>+0.19 (MeCN)
**RC1d** (+1)°	-558.17167	-1.74 (DCE)<br>-2.08 (MeCN)	+6.76	-1.41	
**1d/ RC1d**	**+6.34**	**-1.40** (DCE)<br>**-1.50** (MeCN)	**+0.06**	**-0.04**	
**2a** (0)	-788.57020	-0.41 (DCE)	+8.78	-1.75	**+0.68** (DCE)
**RC2a** (+1)°	-788.31648	-1.92 (DCE)	+8.78	-1.79	
**2a/ RC2a**	**+6.90**	**-1.51** (DCE)	**+0.00**	**-0.04**	
**2d** (0)	-903.14568	-0.52 (DCE)	+9.67	-1.87	+0.34 (DCE)
**RC2d** (+1)°	-902.91215	-1.90 (DCE)	+9.70	-1.86	
**2d/ RC2d**	**+6.35**	**-1.38** (DCE)	**+0.03**	**+0.01**	
**3a** (0) [a]	-788.59035	-0.48 (DCE)	+8.92	-1.67	**+0.57** (DCE)
**RC3a** (+1)°	-788.34146	-1.91 (DCE)	+8.84	-1.69	
**3a/ RC3a**	**+6.77**	**-1.43** (DCE)	**-0.08**	**-0.02**	
**3d** (0)	-903.16588	-0.50 (DCE)	+9.71	-1.76	+0.29 (DCE)
**RC3d** (+1)°	-902.93738	-1.79 (DCE)	+9.75	-1.77	
**3d/ RC3d**	**+6.22**	**-1.29** (DCE)	**+0.04**	**-0.01**	
**8a** (0) [b]	-827.90811	-0.43 (DCE)	+9.62	-1.74	**+0.51** (DCE)
**RC8a** (+1)°	-827.66473	-1.88 (DCE)	+9.64	-1.75	
**8a/ RC8a**	**+6.62**	**-1.45** (DCE)	**+0.02**	**-0.01**	
**8n** (0) [c]	-867.23379	-0.43 (DCE)	+10.42	-1.81	**+0.66** (DCE)
**RC8n** (+1)°	-866.98528	-1.85 (DCE)	+10.43	-1.83	
**8n/ RC8n**	**+6.76**	**-1.42** (DCE)	**+0.01**	**-0.02**	

Supplementary Table 8

Total energies E_T (1 a.u. = 27.21 eV), solvation energies E_{solv} (eV), internal (E_{int}) and entropic (-TS) terms (in eV). a) ΔG values before including the proton solvation energy in DCE ($\Delta G(H^+)_{DCE}$ = -8.91 eV). Adding $\Delta G(H^+)_{DCE}$ yields the first/top number (in eV) of the last column. b) pKa values in DCE are given in parentheses (red) (cf. Eq.S2). An equivalent pKa in water is also provided (black values in parentheses). Notice that the regioselective radical formation (cf. Scheme 11 of main article) can be estimated from a comparison between ΔG values computed for R2a (0)° and R2a' (0)° (see corresponding lines).

Reactions	E_T (a.u.)	E_{solv} (eV)	E_{int} (eV)	-TS (eV)	ΔG (eV)[a]	**pKa**(DCE)[b]
**RC1a** (+1)°	-443.57324	-1.84	+5.82	-1.23		
**R1a** (0)°	-443.17837	-0.26	+5.45	-1.23		+3.04 eV
**RC1a → R1a + H⁺**	**+10.74 eV**	**+1.58**	**-0.37**	**0.00**	**+11.95**	(**51.4**/8.7)
**RC2a** (+1)°	-788.31648	-1.93	+8.78	-1.79		
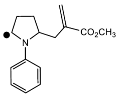 **R2a** (0)°	-787.91835	-0.42	+8.39	-1.71		+3.12 eV
**RC2a → R2a + H⁺**	**+10.83 eV**	**+1.51**	**-0.39**	**+0.08**	**+12.03**	(**52.7**/10.0)
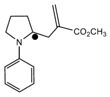 **R2a'** (0)°	-787.92278	-0.41	+8.39	-1.70		+3.02 eV
**RC2a → R2a' + H⁺**	**+10.71 eV**	**+1.52**	**-0.39**	**+0.09**	**+11.93**	(**51.0**/8.3)
**RC3a** (+1)°	-788.34146	-1.91	+8.84	-1.69		
**R3a** (0)°	-787.92776	-0.44	+8.47	-1.66		+3.48 eV
**RC3a → R3a + H⁺**	**+11.26 eV**	**+1.47**	**-0.37**	**+0.03**	**+12.39**	(**58.8**/16.1)
**RC8a** (+1)°	-827.66473	-1.88	+9.64	-1.75		
**R8a** (0)°	-827.25781	-0.41	+9.25	-1.73		+3.26 eV
**RC8a → R8a + H⁺**	**+11.07**	**+1.47**	**-0.39**	**+0.02**	**+12.17**	(**57.1**/14.4)
**RC8n** (+1)°	-866.98528	-1.85	+10.43	-1.83		
**R8n** (0)°	-866.59030	-0.40	+10.03	-1.79		+2.93 eV
**RC8n → R8n + H⁺**	**+10.75**	**+1.45**	**-0.40**	**+0.04**	**+11.84**	(**49.5**/6.8)

Supplementary Table 9

Same as Supplementary Table 8 but for molecules with OMe groups in the para position of their phenyl ring (cf. final "d" letter). Total energies E_T (1 a.u. = 27.21 eV), solvation energies E_{solv} (eV), internal (E_{int}) and entropic (-TS) terms (in eV). a) ΔG values before including the proton solvation energy in DCE ($\Delta G(H^+)_{DCE}$ = -8.91 eV). Adding $\Delta G(H^+)_{DCE}$ yields the first/top number (in eV) of the last column. b) pKa values in DCE are given in parentheses (red) (cf. Eq.S2). An equivalent pKa in water is also provided (black values in parentheses).

Reactions (**OMe**)	E_T (a.u.)	E_{solv} (eV)	E_{int} (eV)	-TS (eV)	□G (eV)[a]	**pKa**(DCE)[b]
**RC1d** (+1)°	-558.17167168	-1.74	+6.76	-1.41		
**R1d** (0)°	-557.75700626	-0.35	+6.37	-1.40		+3.77 eV
**RC1d → R1d + H⁺**	**+11.28 eV**	**+1.39**	**-0.39**	**+0.01**	**+12.29**	(**57.1**/14.4)
**RC2d** (+1)°	-902.91214540	-1.90	+9.70	-1.86		
**R2d** (0)°	-902.49602400	-0.51	+9.39	-1.86		+3.49 eV
**RC2d → R2d + H⁺**	**+11.32 eV**	**+1.39**	**-0.31**	**+0.00**	**+12.40**	(**59.0**/16.3)
**R2d'** (0)°	-902.49771926	-0.51	+9.26	-1.78		+3.40 eV
**RC2d → R2d' + H⁺**	**+11.28 eV**	**+1.39**	**-0.44**	**+0.08**	**+12.31**	(**57.4**/14.7)
**RC3d** (+1)°	-902.93738492	-1.79	+9.75	-1.77		
**R3d** (0)°	-902.50297706	-0.48	+9.36	-1.76		+3.86 eV
**RC3d → R3d + H⁺**	**+11.84 eV**	**+1.31**	**-0.39**	**+0.01**	**+12.77**	(**65.2**/22.5)

Supplementary Table 10

Total energies E_T (1 a.u. = 27.21 eV), solvation energies E_{solv} (eV), internal (E_{int}) and entropic (-TS) terms (in eV). In red are the corresponding differences for a given redox couple. Redox potential calculations in DCE solvent are computed according to Eq.S1 and referred to the calomel reference electrode. (a) Spontaneous dissociation (i.e. ejection of the acetate: >C=CH_2 + O_2C-CH_3) occurs upon reduction. The redox potential is thus evaluated upon preventing dissociation of the reduced species through constrained geometry-optimization (C-O distance kept at 1.47 Å of the oxidized species).

Redox couples	E_T (a.u.)	E_{solv} (eV)	E_{int} eV)	-TS (eV)	$E°_{SCE}$ (V)
**RE2a** (0)°	-1056.43223	-0.67 (DCE)	+11.06	-2.22	
**RE2a** (-1)	-1056.52333	-2.63 (DCE)	+10.93	-2.03	**> -0.29** (DCE)[a]
**RE2a (ox/red)**	**+2.48**	**+1.96** (DCE)	**+0.13**	**-0.19**	
**RE3a** (0)°	-827.24774	-0.47 (DCE)	+9.24	-1.75	
**RE3a** (-1)	-827.30833	-2.67 (DCE)	+9.13	-1.69	**-0.77** (DCE)
**RE3a (ox/red)**	**+1.65**	**+2.20** (DCE)	**+0.11**	**-0.06**	

Conformational study

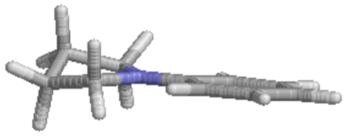	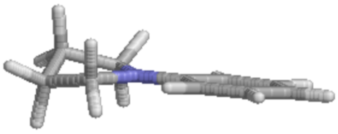	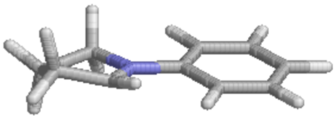
**1a**	**RC1a**	**R1a**
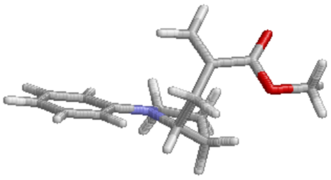	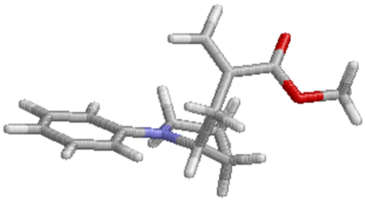	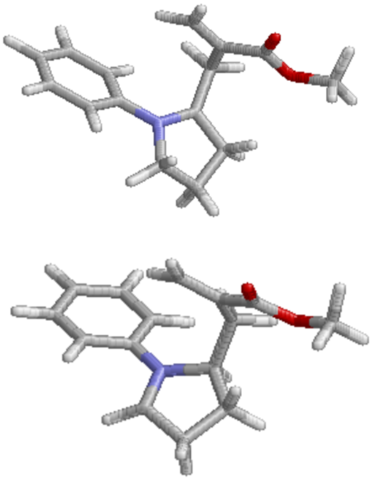
**2a**	**RC2a**	**R2a**<br>**Top : -(H_2)C-N-C°<**<br>**Bottom : -(H)C°-N-C(H)<**
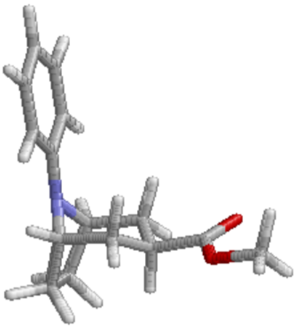	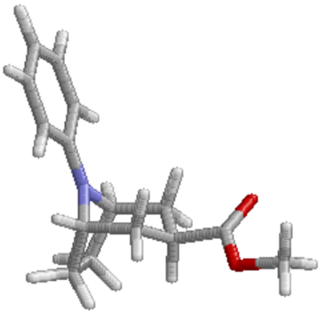	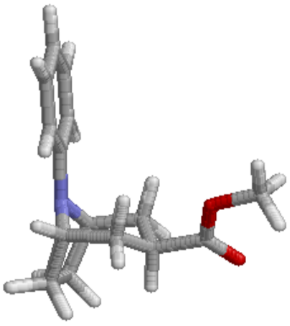
**3a**	**RC3a**	**R3a**
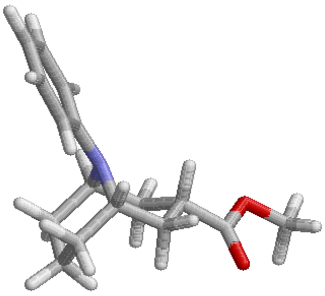	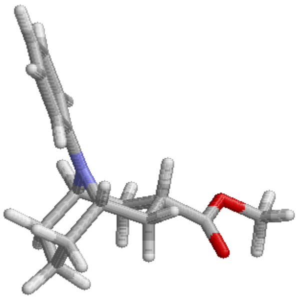	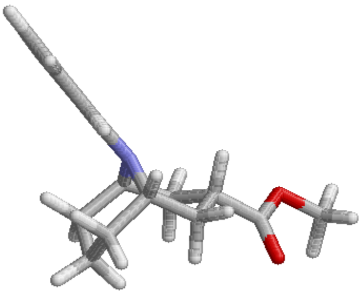
**8a**	**RC8a**	**R8a**

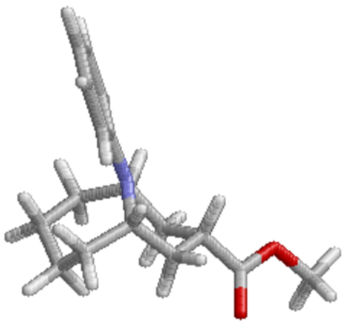	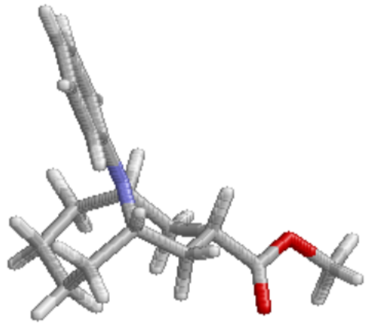	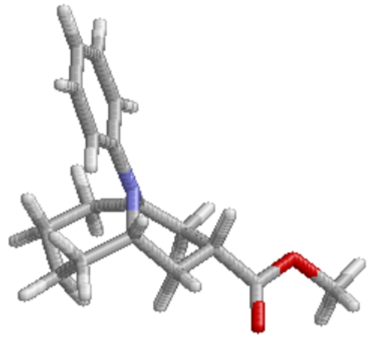
**8n**	**RC8n**	**R8n**

Supplementary Figure 2
Optimized geometries (GGA level: cf. DFT Methodology section above)

For geometry-optimized coordinates: see Supplementary Data 1

Electrochemistry

General

Cyclic Voltammetry for 1mM of 1-phenylpyrrolidine **(1a)**, methyl 4-(pyrrolidin-1-yl)benzoate **(1c)**, 1-(4-methoxyphenyl)pyrrolidine **(1d)**, and ethyl (1R,3R,5S)-8-(4-methoxyphenyl)-8-azabicyclo[3.2.1]octane-3-carboxylate **(3d)** were recorded with 0.1M of $[Bu_4N][PF_6]$ supporting electrolyte in acetonitrile solution.

Cyclic Voltammetry setup

Cyclic Voltammetry experiments were performed at room temperature in an argon-filled glovebox. Data were collected using a BioLogic SP-300 potentiostat connected to a three-electrodes system, including a glassy carbon disk (d=1mm) working electrode, a platinum wire counter electrode, and an Ag/AgCl reference electrode. The voltammograms of each compound were recorded at different scan rates (from 20 mV/s to 20 V/s). The linearity of the oxidation current with the square root of the scan rates was checked for the four compounds in the different experimental cases (inserts of figure SXI). Potential calibration was performed at the end of each data collection cycle using the ferrocene/ferrocenium couple as an internal standard (E° = 0.380 V/SCE).

Results

The electrochemical characteristics of the four studied compounds are summarized in the Table SX. The recorded voltammograms showed that **1a** possesses an irreversible electrochemical behavior with a main oxidation wave close to 0.62 V/SCE (Figure SX-A). This wave is attributed to the one-electron oxidation of **1a** into its cation radical. Additionally, the voltammogram of **1a** exhibits two small oxidation waves at 0.3 V and 0.5 V with their corresponding reduction events. Those waves are

attributed to products of the dimerization of the **1a** cation radical into benzidine compounds. Such dimerization is made possible by the lack of substituent in the 4- position of the aromatic ring.[42,43]

The cyclic voltammogram of **1c** exhibits a quasi-reversible oxidation event at $E_{1/2}$ = 0.91V/SCE (Figure SX-B). Similarly, **1d** and its corresponding final bicyclic product **3d** exhibit quasi-reversible oxidation waves at $E_{1/2}$ = 0.41 and 0.43 V/SCE respectively (Figure SX-C&D).

Then, the effect of the addition of a base was tested by recording the cyclic voltammograms of the studied compounds in presence of 1.2 equivalent of cesium pivalate (similar conditions as the optimized conditions for the photoredox reaction). The cyclic voltammogram of **1c** was very sensitive to the presence of the base. The addition of cesium pivalate leads to a new irreversible oxidation wave that appears at a slightly lower oxidation potential and with higher oxidation current than the oxidation event of the amine alone. This new wave is attributed to an oxidation event of the amine when it is in interaction with cesium pivalate. Moreover, the reversibility of the oxidation event observed in the absence of base disappears in the presence of cesium pivalate (Figure SXI-A1&2). The voltammograms of **1d** were also sensitive to the presence of the base, but less than in the case of **1c** as the newly formed event possesses a lower oxidative current than the initial event of **1d**, which still shows a small reduction event. (Figure SXI-B1&2). This is in agreement with the fact that the photoredox reaction kinetics is faster with **1c** than with **1d**.

On the opposite, the voltammograms of the **3d** final product are almost not affected by the presence of the cesium pivalate base (Figure SXI-C1&2). This indicates that the cation radical of **3d** cannot be deprotonated by the cesium pivalate. This observation supports the proposed mechanism and the fact that the photoredox reaction stops at the bicyclic stage.

Supplementary Table 11

Electrochemical characteristics of the studied amines based. 1) Epa is the potential at the maximum anodic current.

**Amine**	**E_{pa} vs. SCE in MeCN[1]**	**$E_{1/2}$ vs. SCE in MeCN**	**Reversibility in absence of base**	**Reversibility in Presence of base**
**1a**	0.62	-	Irreversible	Irreversible
**1c**	0.96	0.91	Quasi-reversible	Irreversible
**1d**	0.43	0.4	Quasi-reversible	Less reversible
**3d**	0.45	0.43	Quasi-reversible	Quasi-reversible

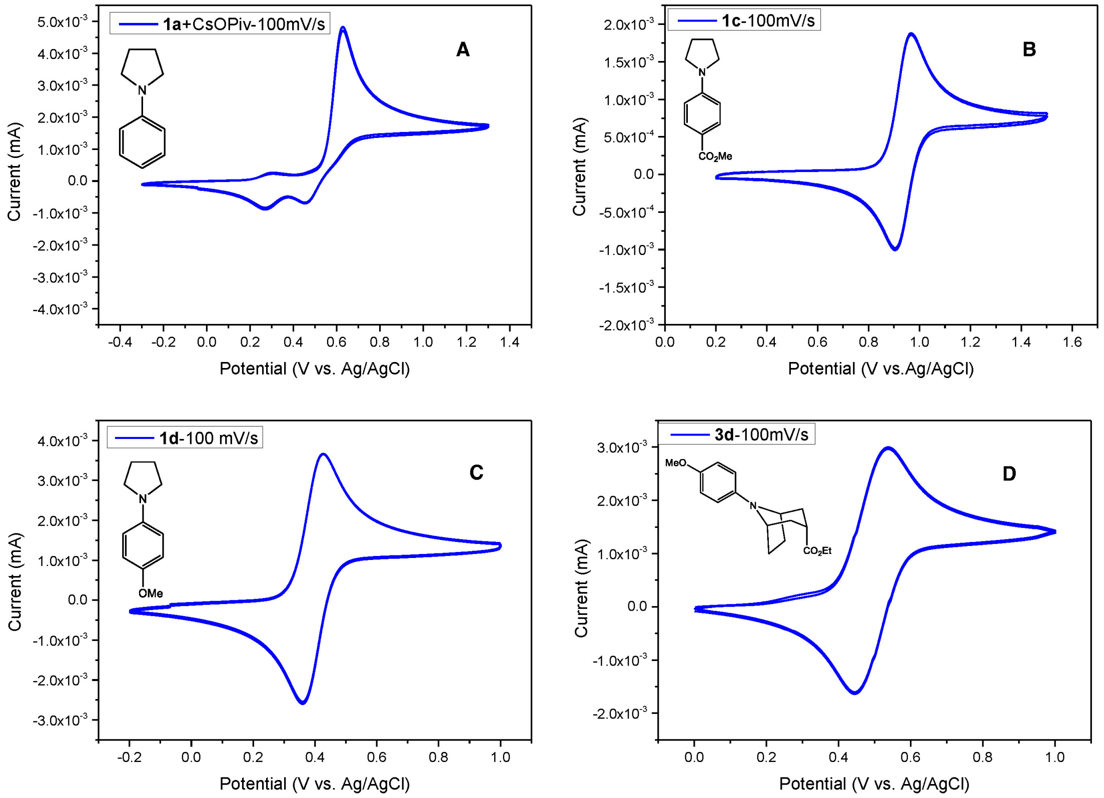

Supplementary Figure 3

(A) Cyclic Voltammograms of 1mM of 1a, (B) 1c, (C) 1d, and (D) 3d, all recorded in 0.1 M [Bu4N][PF6] in acetonitrile at 100mV/s scan rate.

In absence of Cesium Pivalate | In presence of Cesium Pivalate

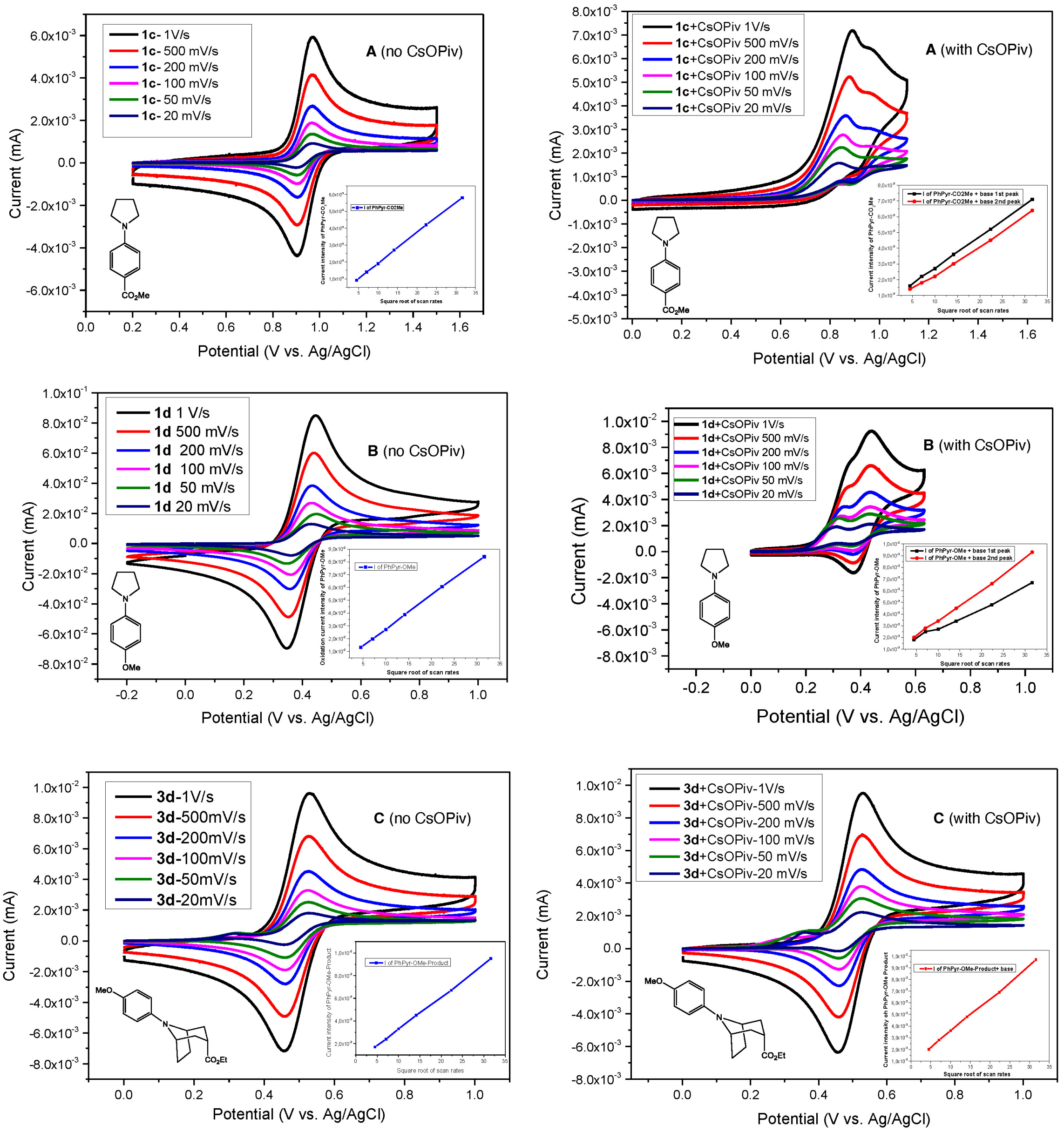

Supplementary Figure 4

(A) Cyclic Voltammograms of 1mM of 1c, (B) 1d, (C) 3d in presence or absence of 1.2mM of Cesium Pivalate base and recorded in 0.1 M [Bu_4N][PF_6] in acetonitrile at different scan rates

Supplementary References

[1] C. L. Joe, A. G. Doyle, *Angew. Chem. Int. Ed.* **2016**, *55*, 4040–4043.
[2] Q. Su, L. A. Dakin, J. S. Panek, *J. Org. Chem.* **2007**, *72*, 2–24.
[3] G. Patel, C. E. Karver, R. Behera, P. J. Guyett, C. Sullenberger, P. Edwards, N. E. Roncal, K. Mensa-Wilmot, M. P. Pollastri, *J. Med. Chem.* **2013**, *56*, 3820–3832.
[4] Q. Yin, H. F. T. Klare, M. Oestreich, *Angew. Chem. Int. Ed.* **2017**, *56*, 3712–3717.
[5] A. Noble, D. W. C. MacMillan, *J. Am. Chem. Soc.* **2014**, *136*, 11602–11605.
[6] N. Takasu, K. Oisaki, M. Kanai, *Org. Lett.* **2013**, *15*, 1918–1921.
[7] Z.-M. Zhang, Y.-T. Xu, L.-X. Shao, *J. Organomet. Chem.* **2021**, *940*, 121683.
[8] S. A. Girard, X. Hu, T. Knauber, F. Zhou, M.-O. Simon, G.-J. Deng, C.-J. Li, *Org. Lett.* **2012**, *14*, 5606–5609.
[9] R. Sun, Y. Qin, D. G. Nocera, *Angew. Chem. Int. Ed.* **2020**, *59*, 9527–9533.
[10] M. J. Cawley, F. G. N. Cloke, R. J. Fitzmaurice, S. E. Pearson, J. S. Scott, S. Caddick, *Org. Biomol. Chem.* **2008**, *6*, 2820–2825.
[11] Q. Zou, C. Wang, J. Smith, D. Xue, J. Xiao, *Chem. – Eur. J.* **2015**, *21*, 9656–9661.
[12] D. Chamorro-Arenas, U. Osorio-Nieto, L. Quintero, L. Hernández-García, F. Sartillo-Piscil, *J. Org. Chem.* **2018**, *83*, 15333–15346.
[13] M. Quadri, C. Stokes, A. Gulsevin, A. C. J. Felts, K. A. Abboud, R. L. Papke, N. A. Horenstein, *J. Med. Chem.* **2017**, *60*, 7928–7934.
[14] A. H. Sandtorv, D. R. Stuart, *Angew. Chem. Int. Ed.* **2016**, *55*, 15812–15815.
[15] *Org. Synth.* **2006**, *83*, 24.
[16] J. Zhang, Y. Li, R. Xu, Y. Chen, *Angew. Chem. Int. Ed.* **2017**, *56*, 12619–12623.
[17] B. M. Trost, M. R. Machacek, H. C. Tsui, *J. Am. Chem. Soc.* **2005**, *127*, 7014–7024.
[18] P. V. Ramachandran, T. E. Burghardt, L. Bland-Berry, *J. Org. Chem.* **2005**, *70*, 7911–7918.
[19] E. Breuer, D. Melumad, *J. Org. Chem.* **1973**, *38*, 1601–1602.
[20] C. Boga, F. Manescalchi, D. Savoia, *Tetrahedron* **1994**, *50*, 4709–4722.
[21] D. Taniyama, M. Hasegawa, K. Tomioka, *Tetrahedron Lett.* **2000**, *41*, 5533–5536.
[22] G. te Velde, E. J. Baerends, *J. Comput. Phys.* **1992**, *99*, 84–98.
[23] S. H. Vosko, L. Wilk, M. Nusair, *Can. J. Phys.* **1980**, *58*, 1200–1211.
[24] A. D. Becke, *Phys. Rev. A* **1988**, *38*, 3098–3100.
[25] J. P. Perdew, *Phys. Rev. B* **1986**, *33*, 8822–8824.
[26] J.-M. Mouesca, J. L. Chen, L. Noodleman, D. Bashford, D. A. Case, *J. Am. Chem. Soc.* **1994**, *116*, 11898–11914.
[27] B. S. Perrin Jr., T. Ichiye, *Proteins Struct. Funct. Bioinforma.* **2010**, *78*, 2798–2808.
[28] R. A. Torres, T. Lovell, L. Noodleman, D. A. Case, *J. Am. Chem. Soc.* **2003**, *125*, 1923–1936.
[29] L. E. Roy, E. R. Batista, P. J. Hay, *Inorg. Chem.* **2008**, *47*, 9228–9237.
[30] L. E. Roy, E. V. Jakubikova, E. R. Batista, "Accurate Calculation of Redox Potentials Using Density Functional Methods," can be found under http://www.researchgate.net/file.PostFileLoader.html?id=56673ec760614bd8848b4568&assetKey=AS%3A304487376326658%401449606855955, **n.d.**
[31] A. Volbeda, J. M. Mouesca, C. Darnault, M. M. Roessler, A. Parkin, F. A. Armstrong, J. C. Fontecilla-Camps, *Chem. Commun.* **2018**, *54*, 7175–7178.
[32] A. Klamt, G. Schüürmann, *J. Chem. Soc. Perkin Trans. 2* **1993**, *0*, 799–805.
[33] A. Klamt, *J. Phys. Chem.* **1995**, *99*, 2224–2235.
[34] A. Klamt, V. Jonas, *J. Chem. Phys.* **1996**, *105*, 9972–9981.
[35] H. Reiss, A. Heller, *J. Phys. Chem.* **1985**, *89*, 4207–4213.
[36] C. P. Kelly, C. J. Cramer, D. G. Truhlar, *J. Phys. Chem. B* **2006**, *110*, 16066–16081.
[37] C. P. Kelly, C. J. Cramer, D. G. Truhlar, *J. Phys. Chem. B* **2007**, *111*, 408–422.
[38] A. A. Isse, A. Gennaro, *J. Phys. Chem. B* **2010**, *114*, 7894–7899.
[39] D. Himmel, S. K. Goll, I. Leito, I. Krossing, *Chem. - Eur. J.* **2011**, *17*, 5808–5826.

[40] E. Paenurk, K. Kaupmees, D. Himmel, A. Kütt, I. Kaljurand, I. A. Koppel, I. Krossing, I. Leito, *Chem. Sci.* **2017**, *8*, 6964–6973.
[41] D. Himmel, S. K. Goll, I. Leito, I. Krossing, *Angew. Chem. Int. Ed.* **2010**, *49*, 6885–6888.
[42] E. T. Seo, R. F. Nelson, J. M. Fritsch, L. S. Marcoux, D. W. Leedy, R. N. Adams, *J. Am. Chem. Soc.* **1966**, *88*, 3498–3503.
[43] R. N. Adams, *Acc. Chem. Res.* **1969**, *2*, 175–180.

Supplementary Note 1

Starting materials – new compounds

1-(2-tert-butylphenyl)pyrrolidine (**1e**)

^{1}H-NMR (300 MHz, $CDCl_3$)

GA_208039.10.fid
ECO-2-150 F1
Proton_ns8_d1=10s CDCl3 /opt service 48

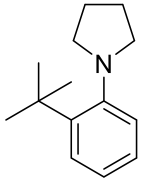

1e

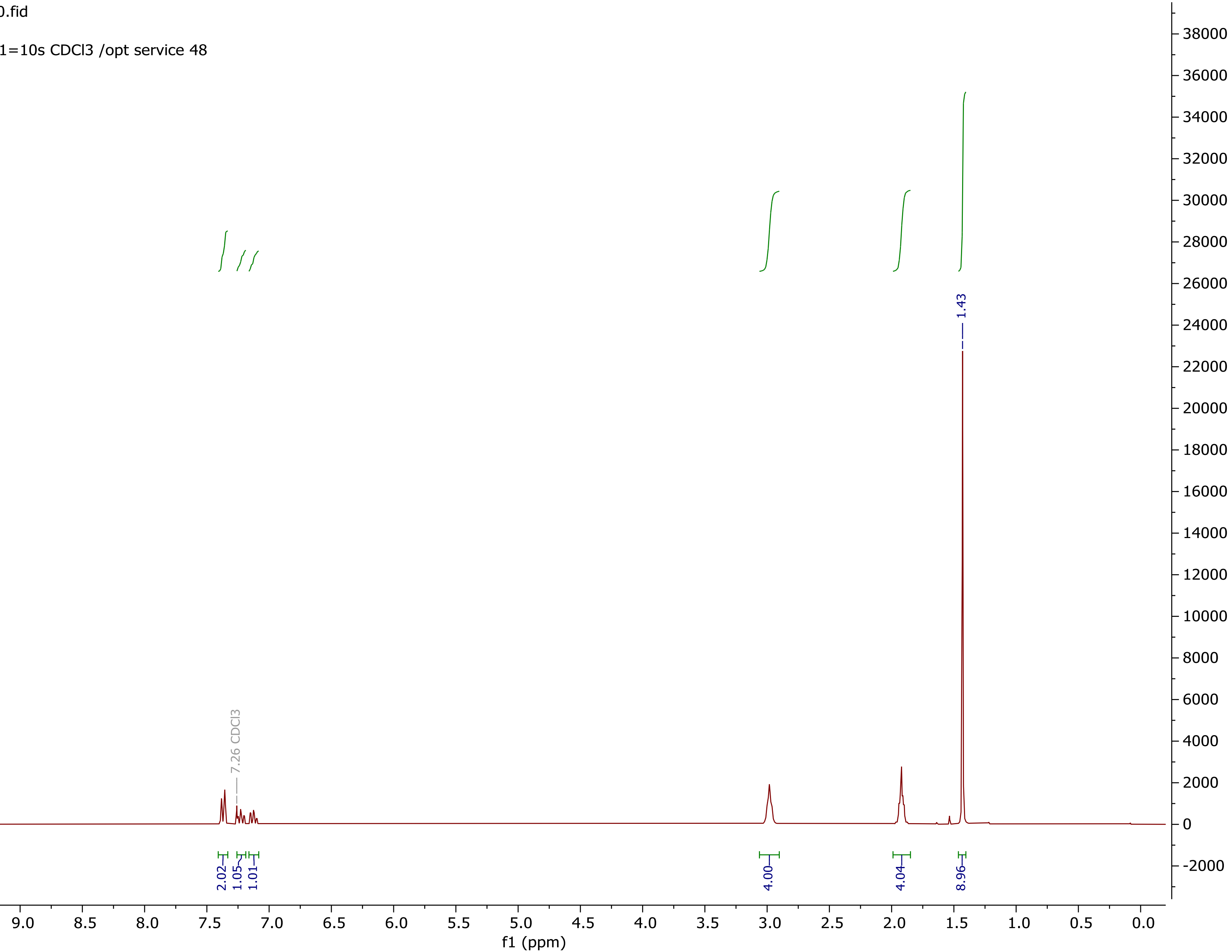

1-(2-tert-butylphenyl)pyrrolidine (**1e**)

^{1}H-NMR (300 MHz, $CDCl_3$)

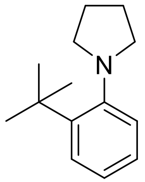

1e

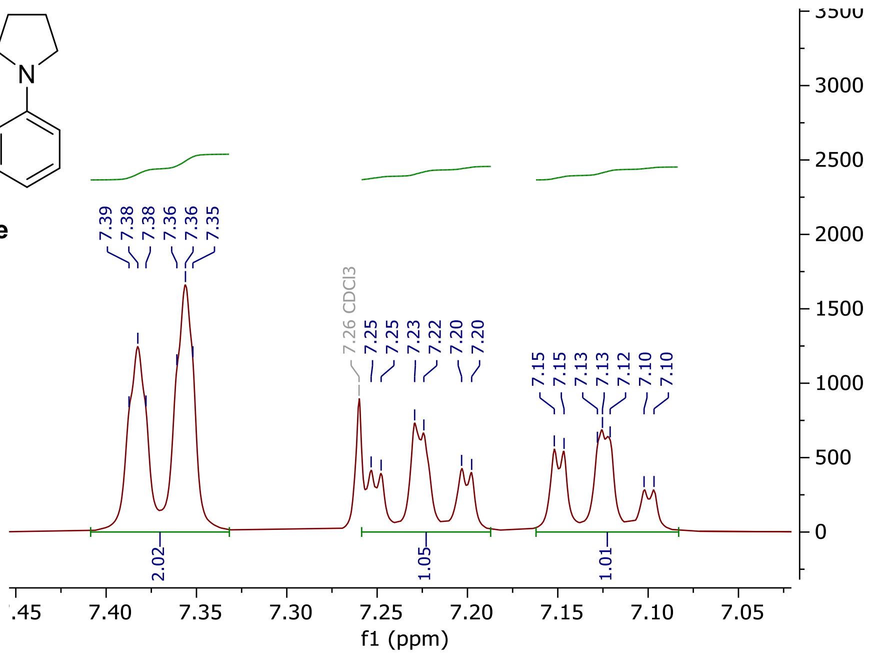

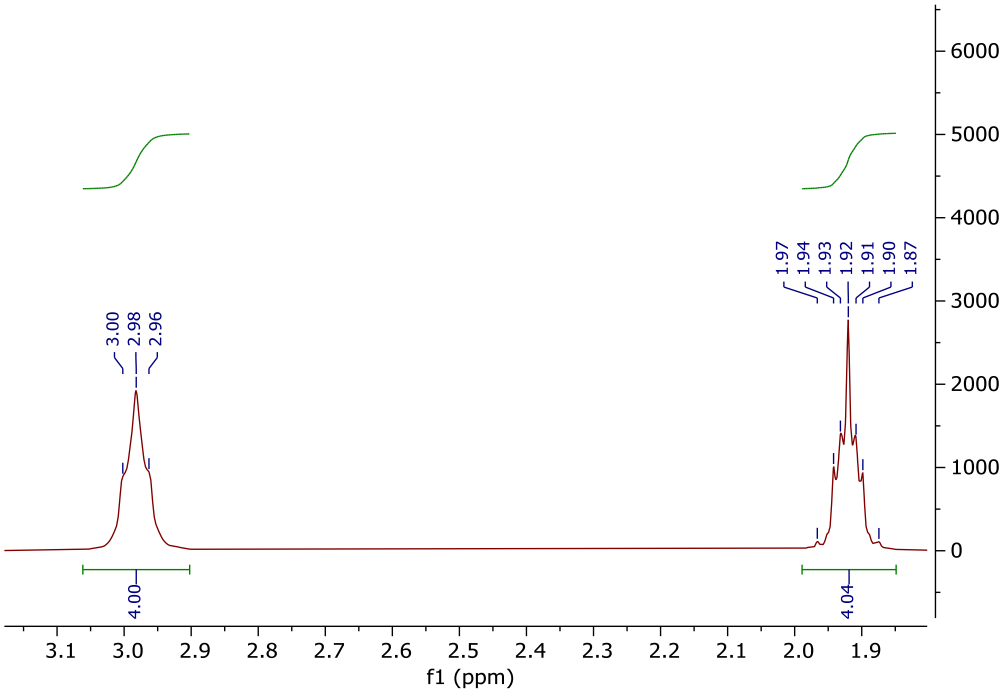

1-(2-tert-butylphenyl)pyrrolidine (**1e**)

^{13}C-NMR (75 MHz, $CDCl_3$)

GA_208039.11.fid
ECO-2-150 F1
Carbon_ns512 CDCl3 /opt service 48

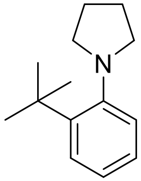

1e

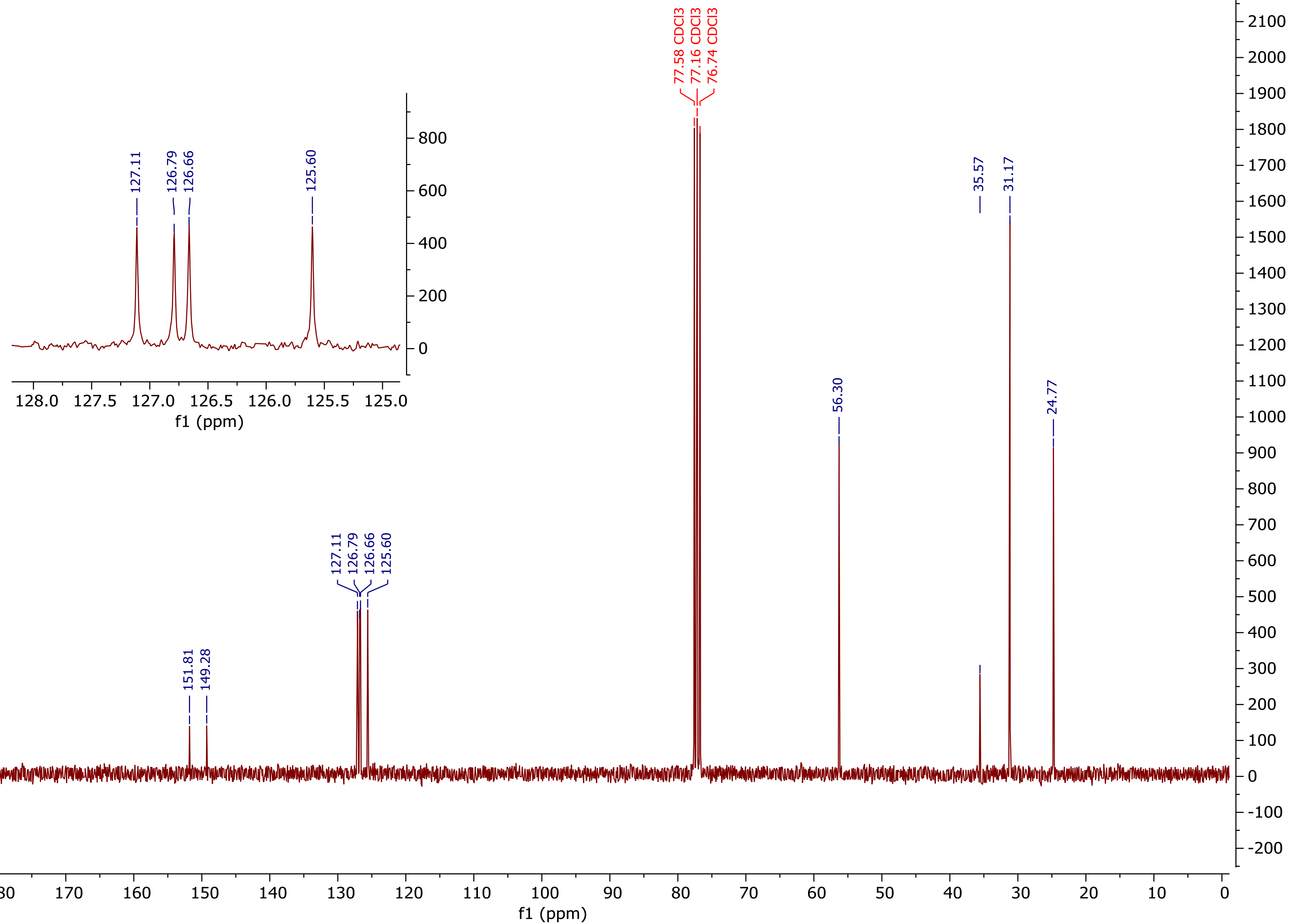

1-(2-tert-butylphenyl)pyrrolidine (**1e**)

^{13}C-NMR (75 MHz, $CDCl_3$)

GA_208039.12.fid
ECO-2-150 F1
Dept135_ns512 CDCl3 /opt service 48

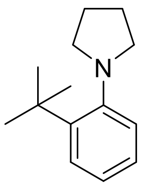

1e

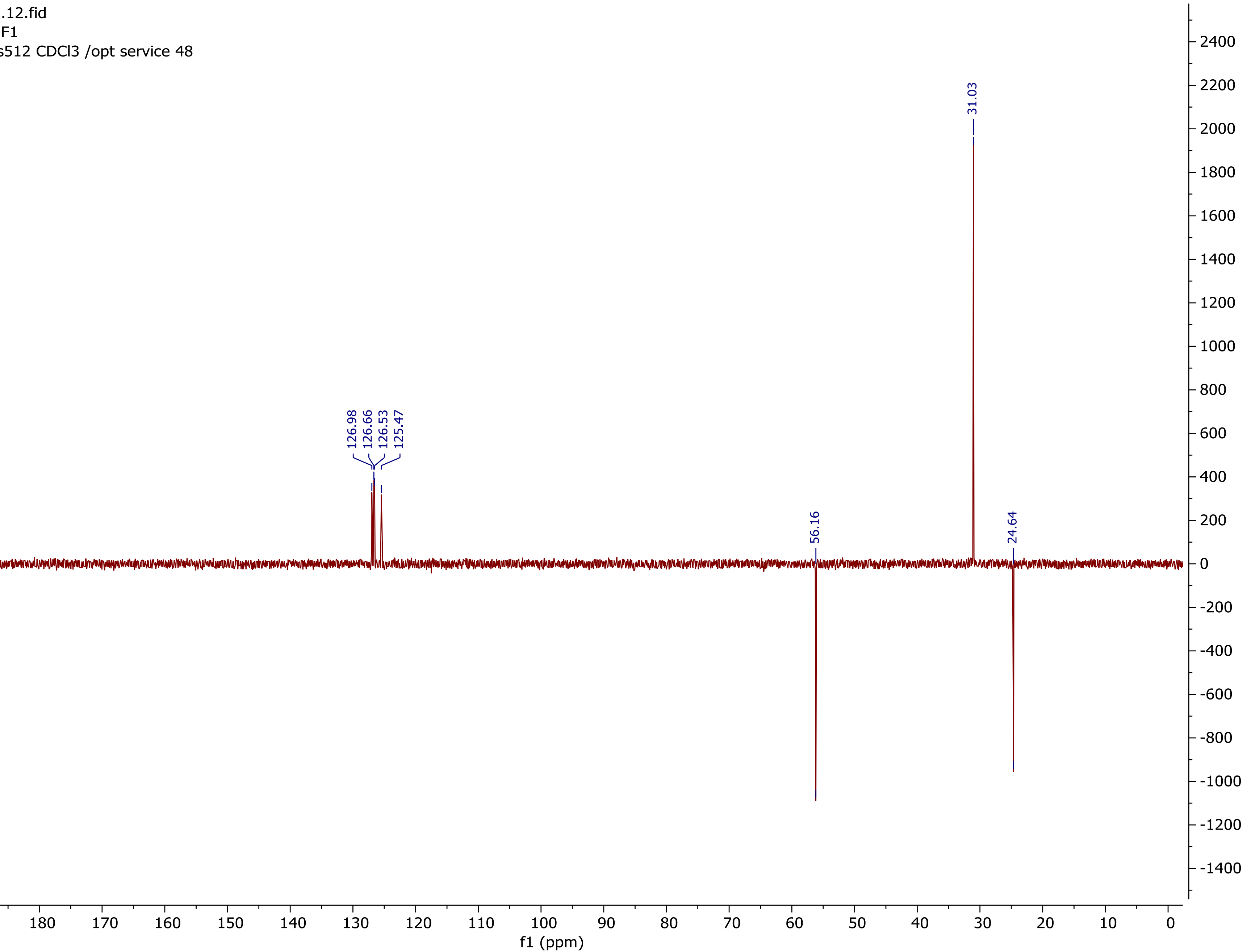

1-(2-tert-butylphenyl)pyrrolidine (**1e**)

FT-IR, ATR-diamond

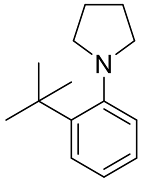

1e

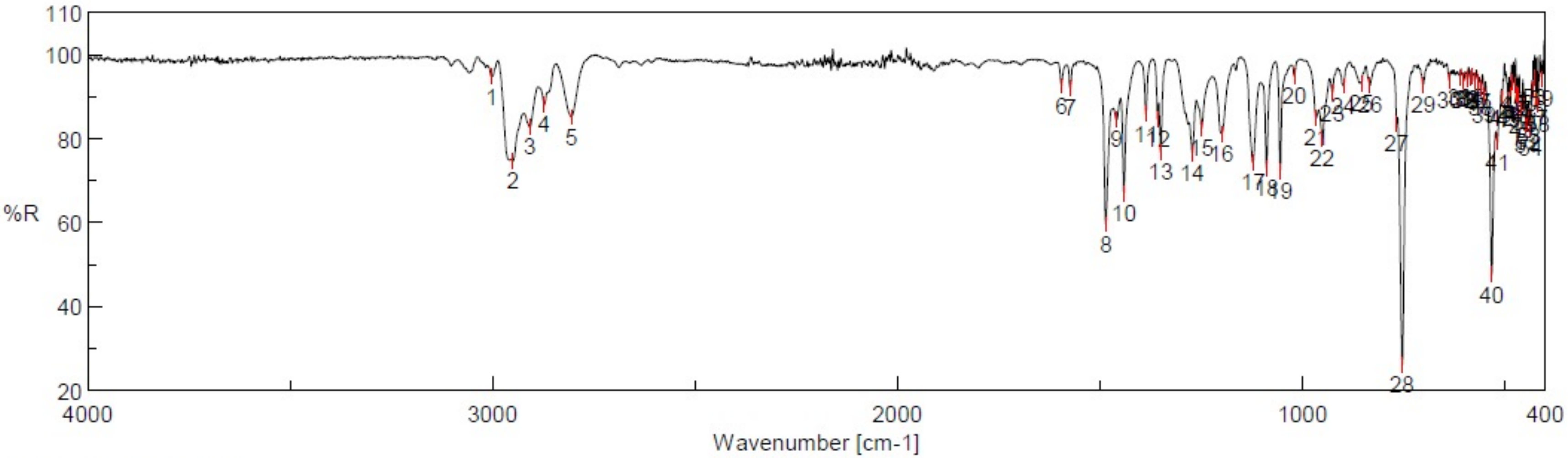

[Result of Peak Picking]

No.	Position	Intensity	No.	Position	Intensity	No.	Position	Intensity
1	3002.62	94.7337	2	2949.59	74.585	3	2907.65	82.666
4	2872.45	87.9866	5	2805.44	85.1259	6	1594.84	92.5492
7	1573.15	91.9947	8	1485.4	59.5101	9	1459.37	84.5572
10	1440.56	66.8887	11	1386.09	85.8078	12	1357.16	84.643
13	1349.44	76.6612	14	1271.82	76.519	15	1248.68	82.5096
16	1199.51	81.1614	17	1121.89	74.2052	18	1088.62	72.8872
19	1054.39	72.2895	20	1019.68	94.8546	21	965.68	84.9323
22	950.734	80.0852	23	925.664	90.7805	24	898.183	92.5194
25	854.311	93.112	26	832.616	92.7086	27	767.048	83.6262
28	753.066	25.8964	29	701.48	92.8159	30	636.876	93.7853
31	610.842	94.6671	32	600.717	93.7755	33	593.004	94.0357
34	585.772	94.3054	35	580.951	94.7133	36	574.201	93.6912
37	564.559	93.6255	38	559.738	91.9787	39	551.542	90.3323
40	532.257	47.5316	41	517.793	79.0746	42	506.705	89.6201
43	492.241	89.3316	44	486.456	90.3283	45	480.67	93.2675
46	475.849	92.8105	47	471.028	90.4686	48	466.689	90.286
49	458.493	86.8604	50	452.225	88.5322	51	446.922	83.193

1-[4-(3,3,4,4-tetramethyl-1lambda3,2,5-bromadioxolan-1-yl)phenyl]piperidine (**6j**)

^{1}H-NMR (300 MHz, $CDCl_3$)

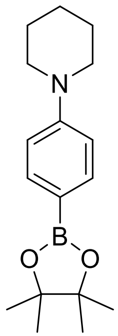

6j

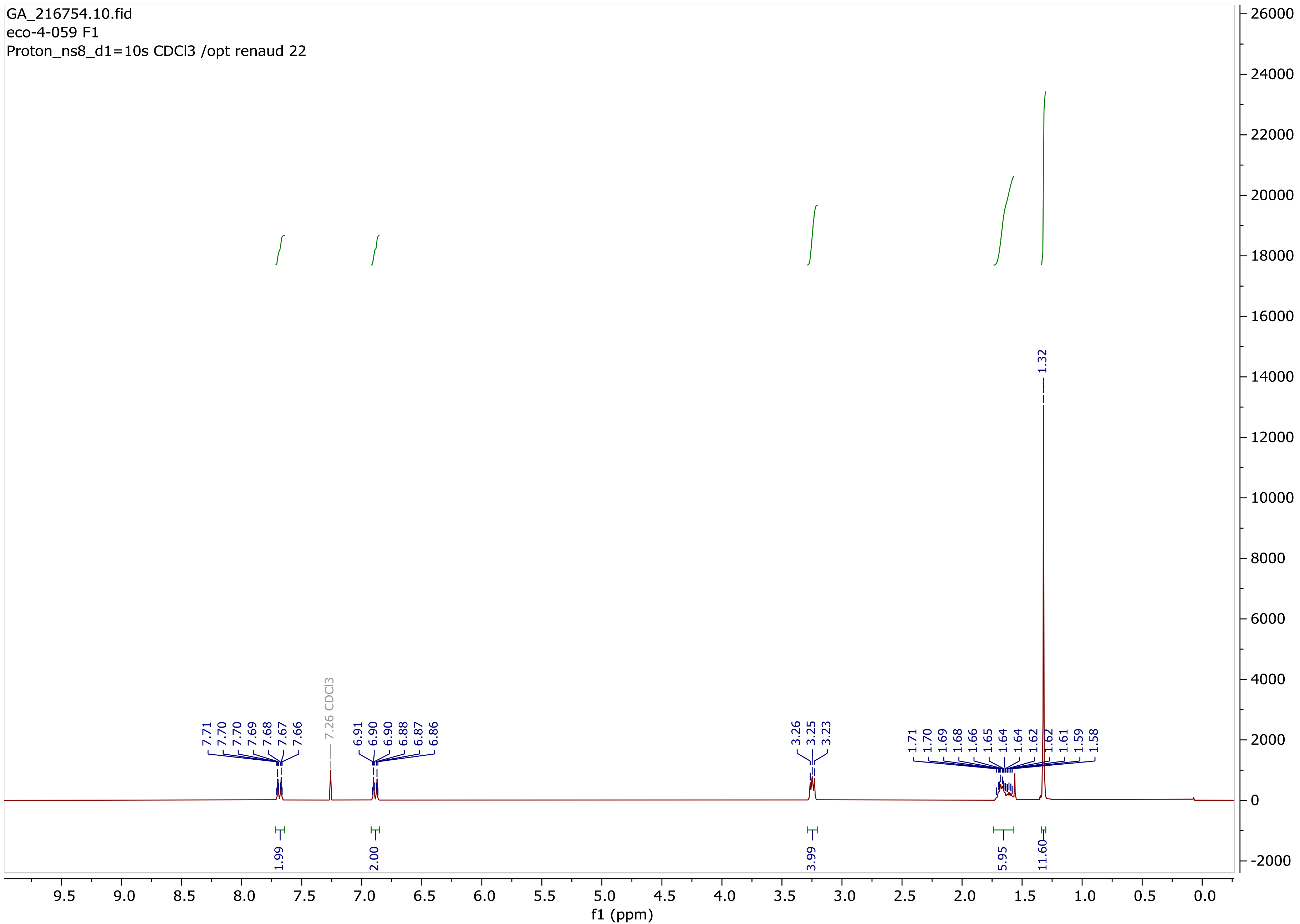

1-[4-(3,3,4,4-tetramethyl-1lambda3,2,5-bromadioxolan-1-yl)phenyl]piperidine (**6j**)

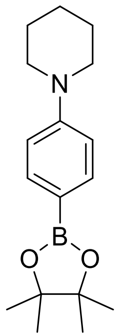

6j

^{11}B-NMR (96 MHz, $CDCl_3$)

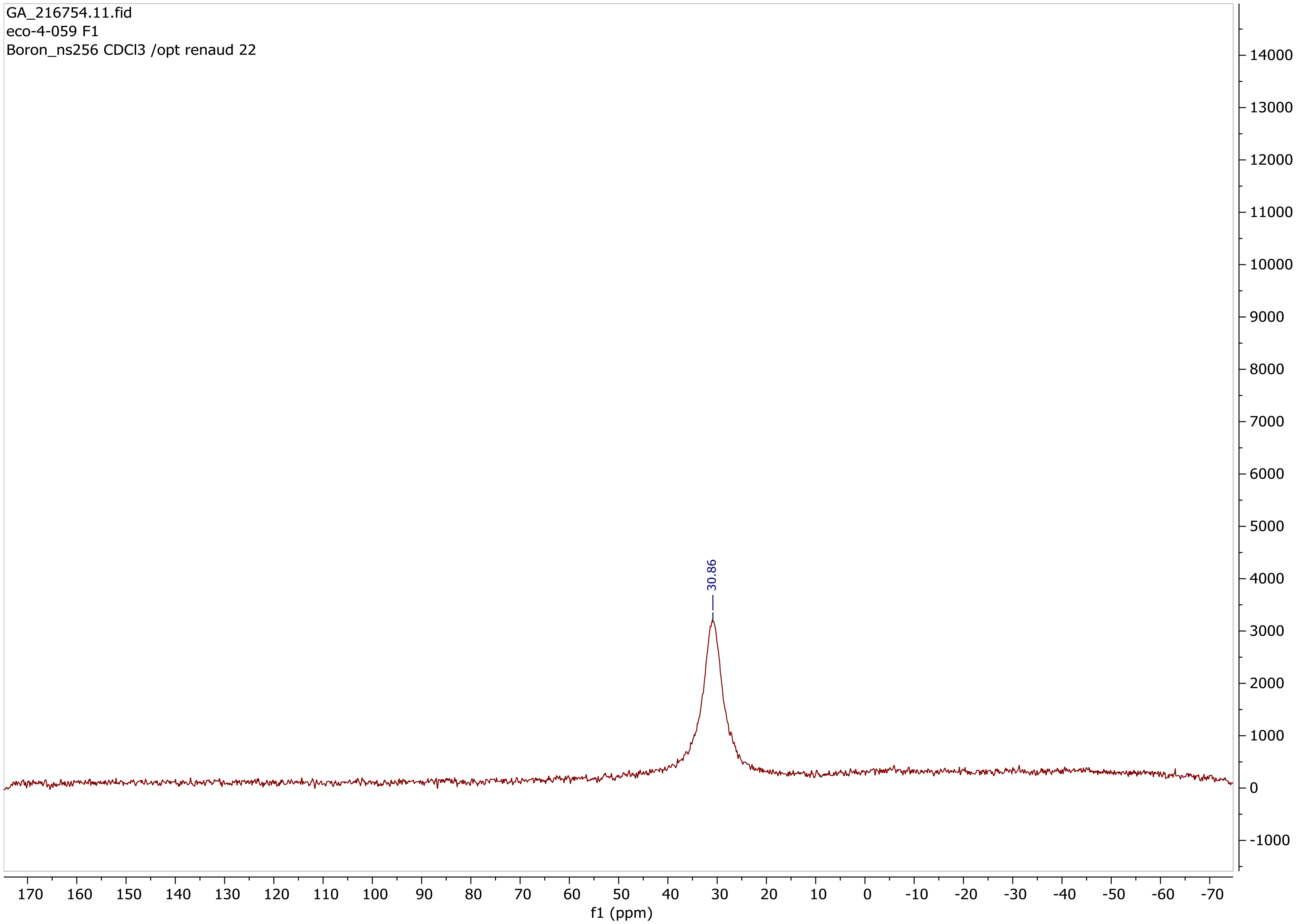

1-[4-(3,3,4,4-tetramethyl-1lambda3,2,5-bromadioxolan-1-yl)phenyl]piperidine (**6j**)

^{13}C-NMR (75 MHz, $CDCl_3$)

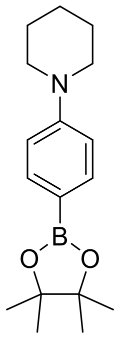

6j

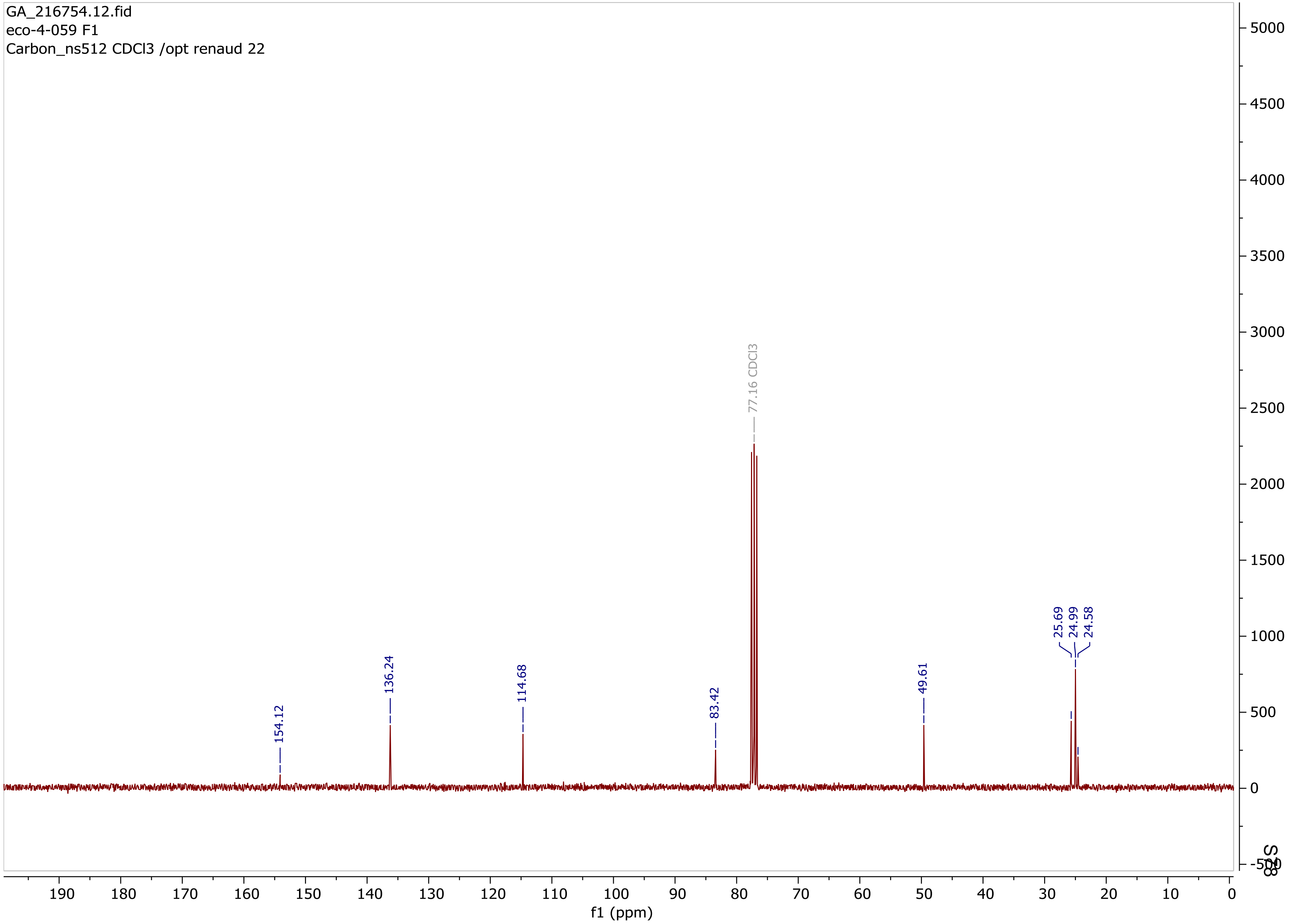

1-[4-(3,3,4,4-tetramethyl-1lambda3,2,5-bromadioxolan-1-yl)phenyl]piperidine (**6j**)

^{13}C-NMR (75 MHz, $CDCl_3$)

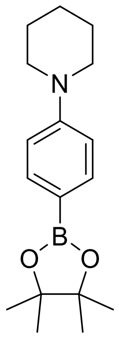

6j

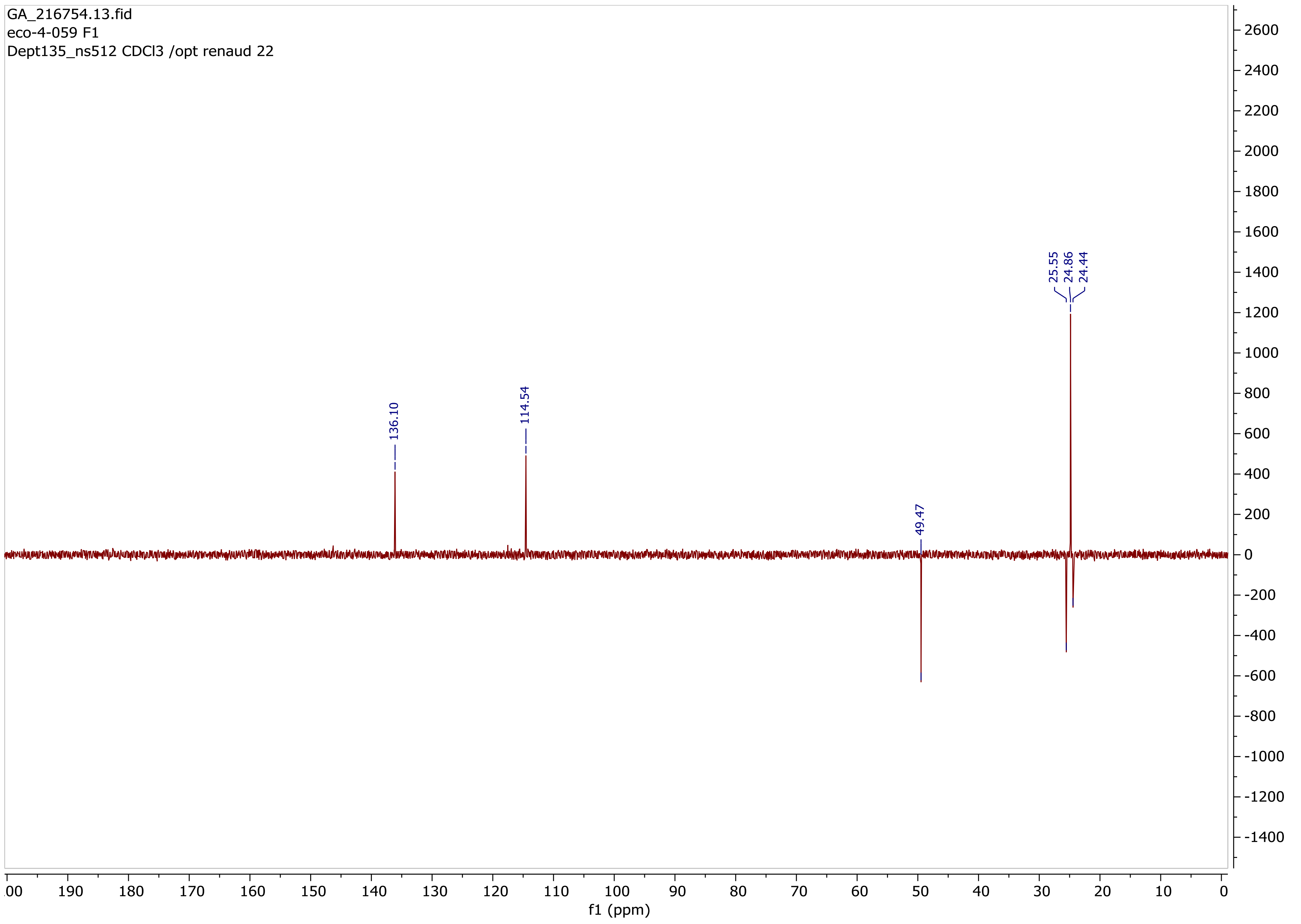

1-[4-(3,3,4,4-tetramethyl-1lambda3,2,5-bromadioxolan-1-yl)phenyl]piperidine (**6j**)

FT-IR, ATR-diamond

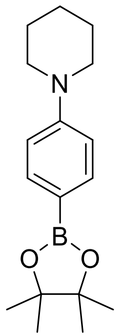

6j

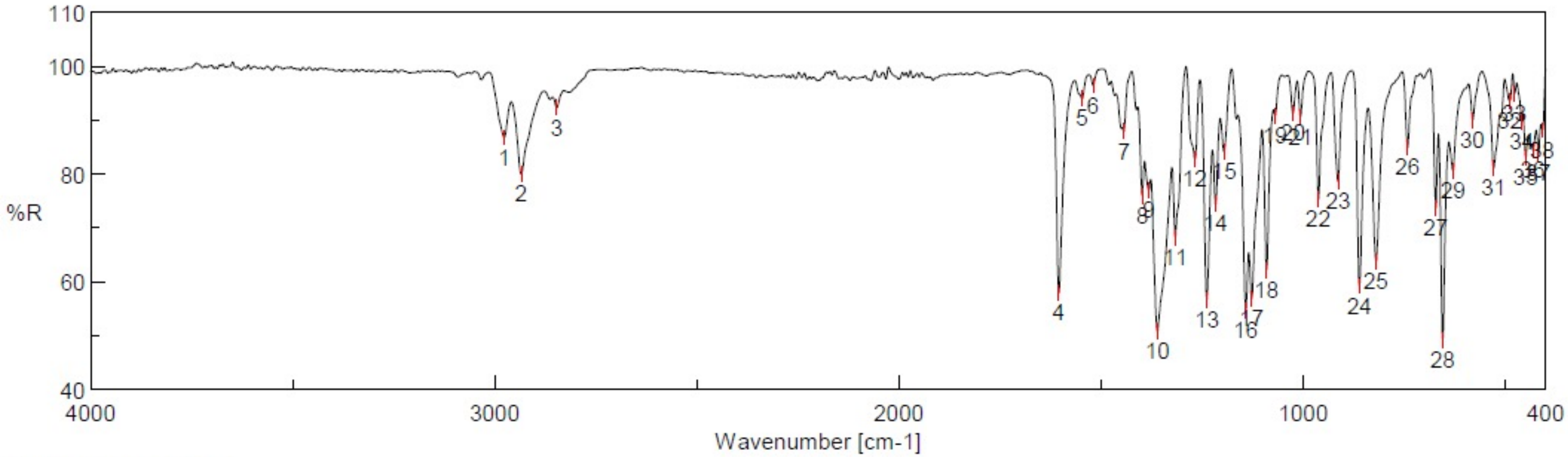

[Result of Peak Picking]

No.	Position	Intensity	No.	Position	Intensity	No.	Position	Intensity
1	2976.59	86.7752	2	2934.16	79.9735	3	2846.42	92.3045
4	1604.48	57.8274	5	1546.63	93.9703	6	1519.63	96.4306
7	1443.46	87.8751	8	1398.14	75.9626	9	1381.75	76.9318
10	1360.53	50.7087	11	1316.18	68.1985	12	1267.97	82.7235
13	1238.08	56.6708	14	1216.86	74.4324	15	1195.65	84.1091
16	1141.65	54.6081	17	1127.19	56.8815	18	1090.55	62.1477
19	1069.33	90.8961	20	1024.98	91.2387	21	1006.66	90.6378
22	962.305	75.266	23	913.129	78.6332	24	860.096	59.187
25	819.598	63.7668	26	741.496	84.9128	27	672.071	73.7246
28	654.715	49.1181	29	628.68	80.5184	30	580.469	89.9914
31	528.4	81.0018	32	488.866	93.6151	33	476.331	94.9341
34	458.975	89.6909	35	449.333	83.1435	36	431.012	84.355
37	417.513	83.9447	38	407.871	88.135			

Ethyl 2-[(2,2,2-trifluoroacetyl)oxymethyl]prop-2-enoate

^{1}H-NMR (400 MHz, $CDCl_3$)

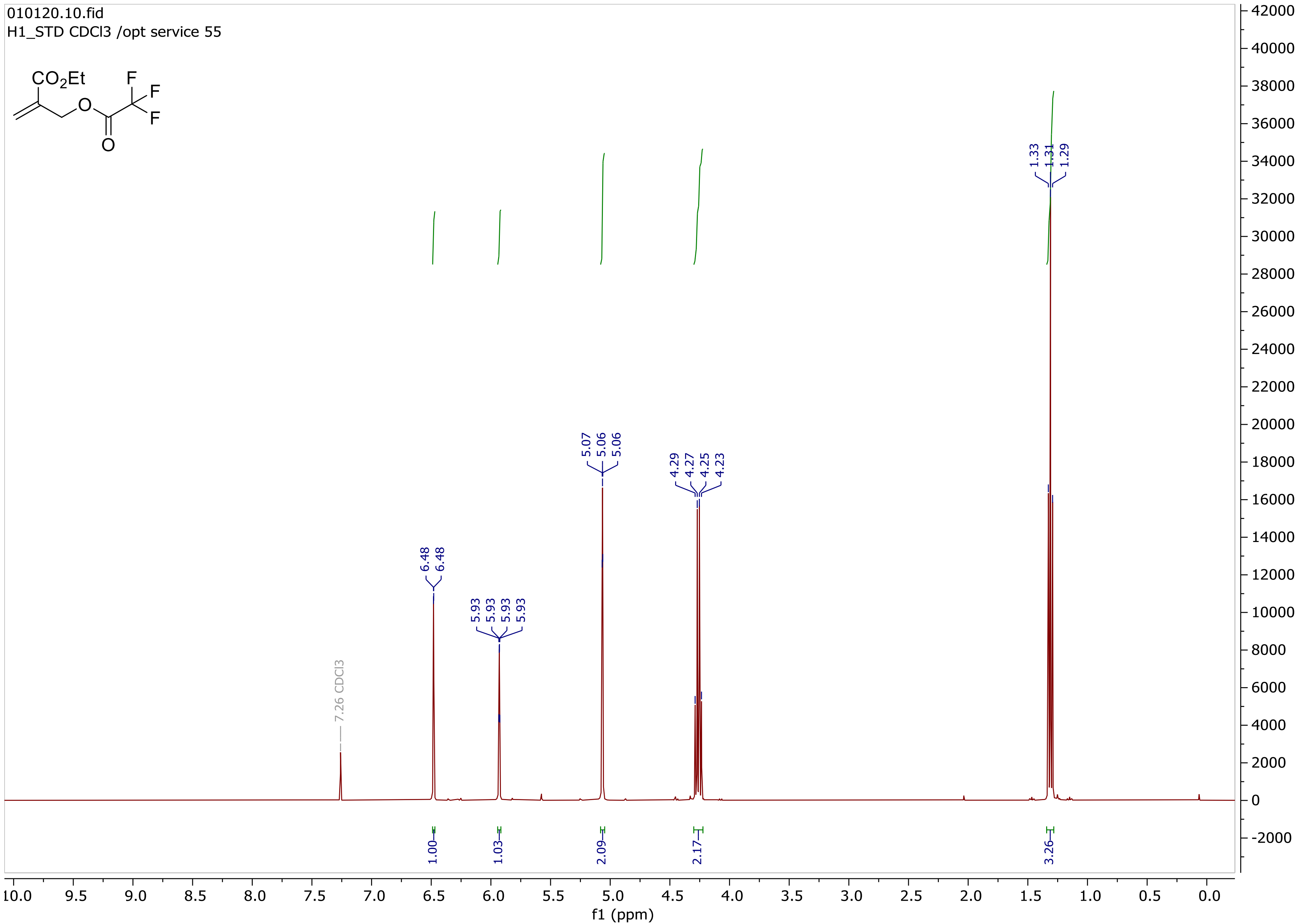

Ethyl 2-[(2,2,2-trifluoroacetyl)oxymethyl]prop-2-enoate

^{13}C-NMR (101 MHz, $CDCl_3$)

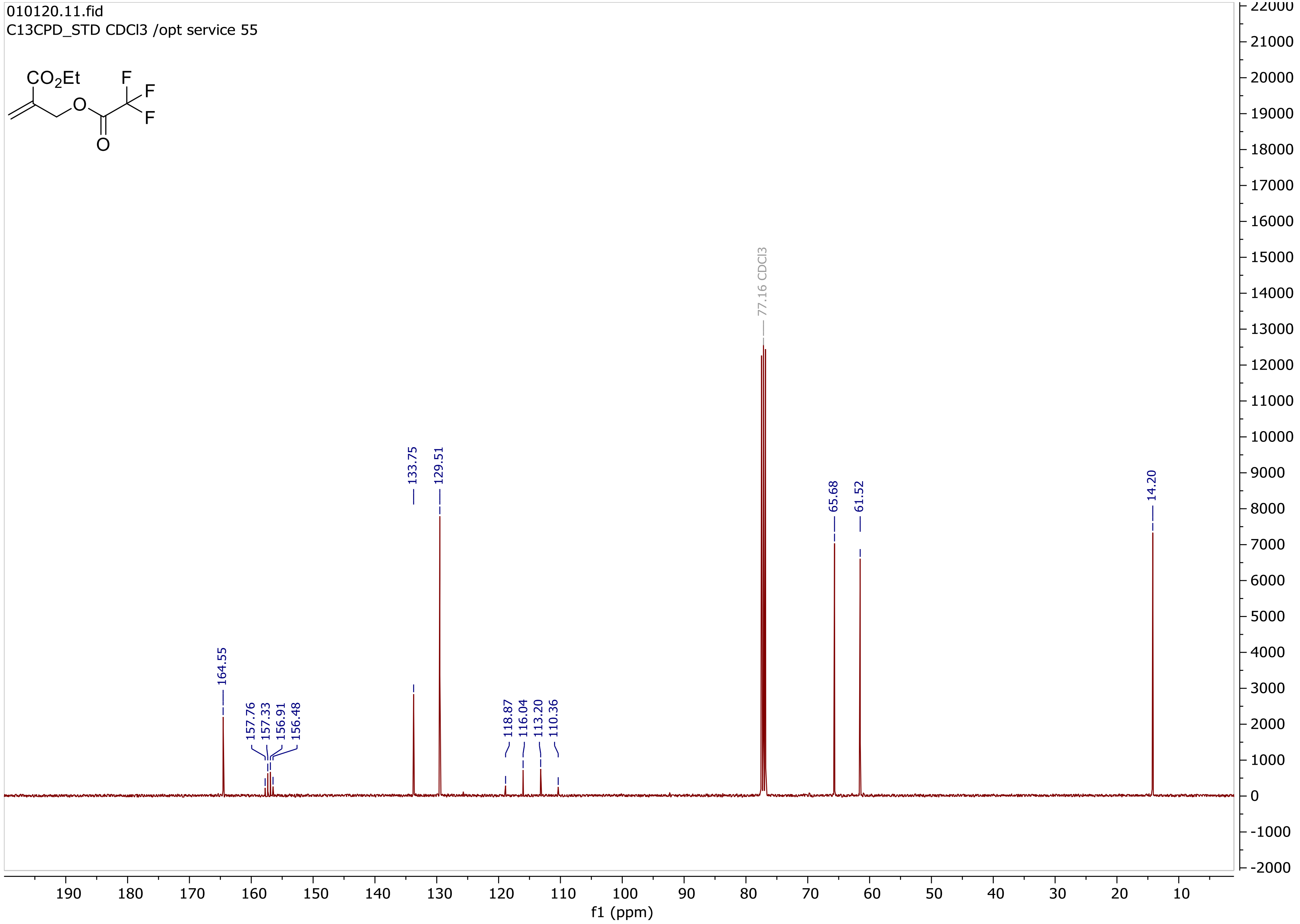

Ethyl 2-[(2,2,2-trifluoroacetyl)oxymethyl]prop-2-enoate

^{13}C-NMR (101 MHz, $CDCl_3$)

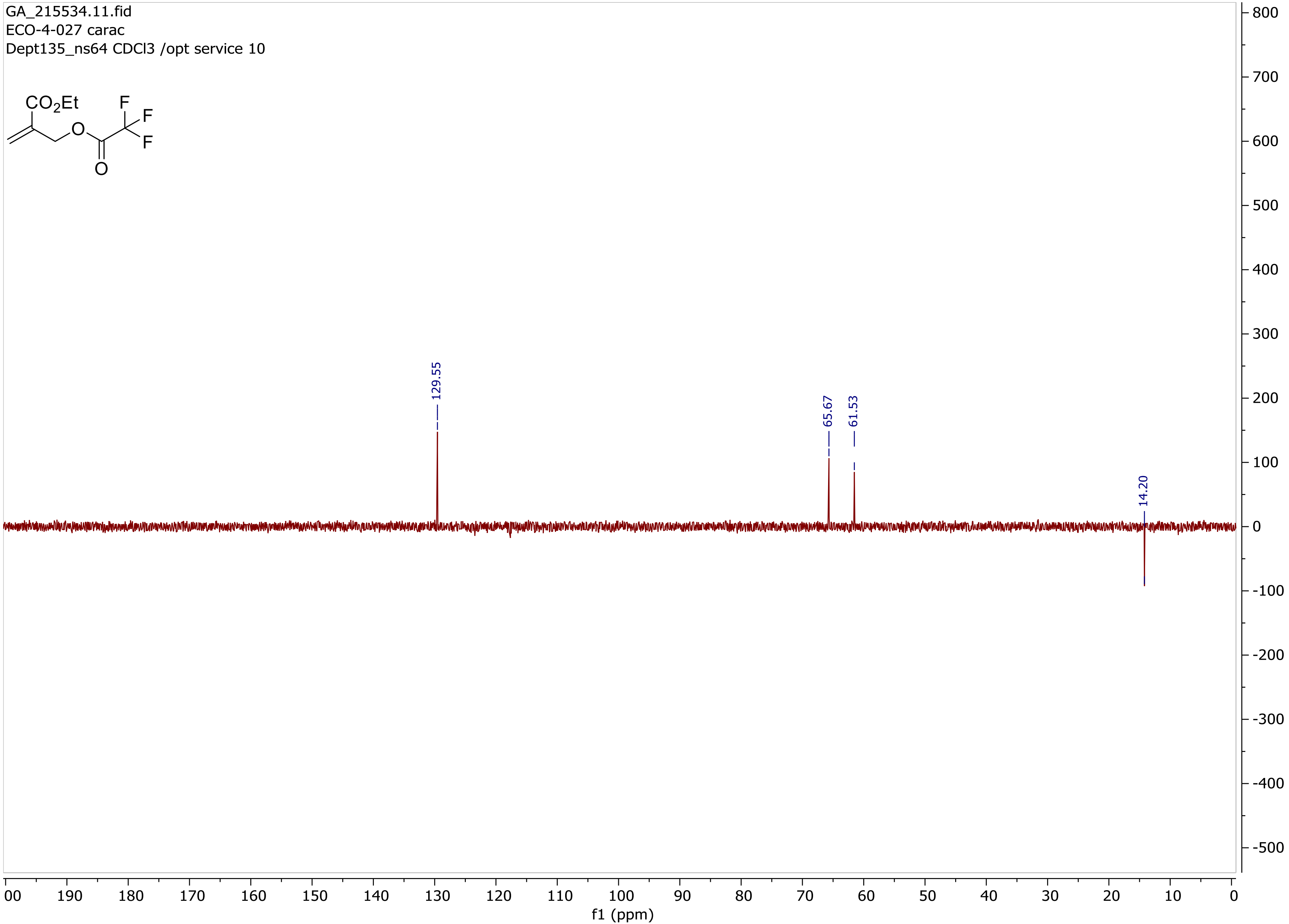

Ethyl 2-[(2,2,2-trifluoroacetyl)oxymethyl]prop-2-enoate

^{19}F NMR (282 MHz, $CDCl_3$)

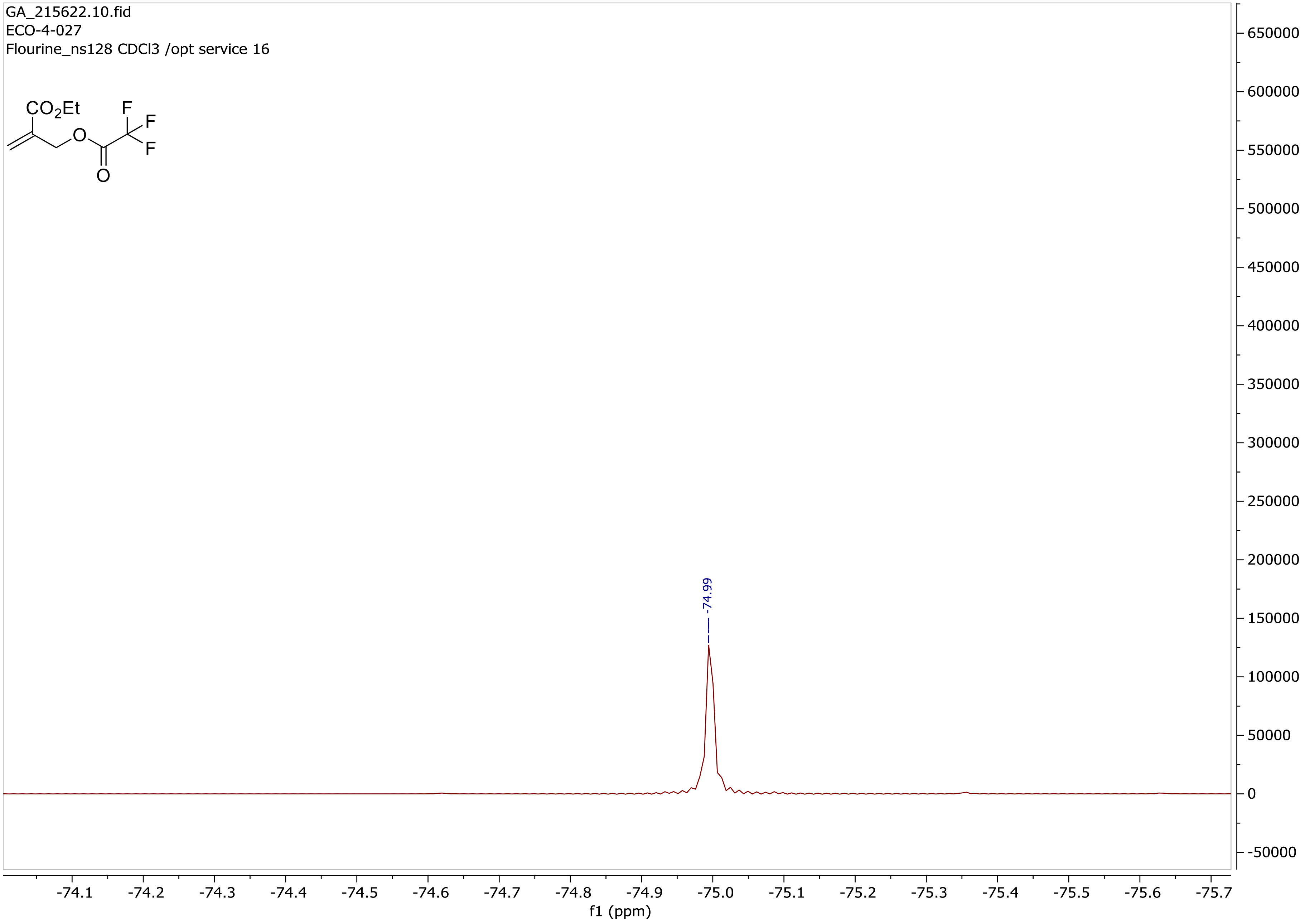

Ethyl 2-[(2,2,2-trifluoroacetyl)oxymethyl]prop-2-enoate

FT-IR, ATR-diamond

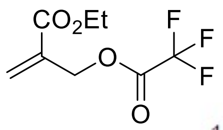

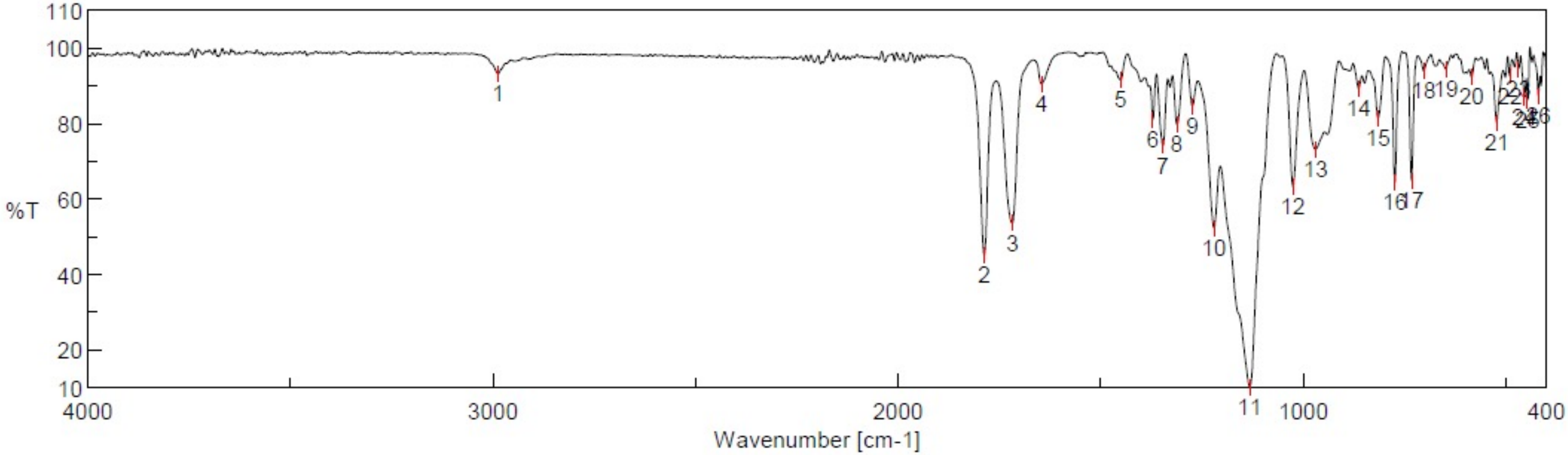

[Result of Peak Picking]

No.	Position	Intensity	No.	Position	Intensity	No.	Position	Intensity
1	2987.2	93	2	1787.69	45	3	1718.26	54
4	1645.95	90	5	1451.17	92	6	1371.14	81
7	1347.03	74	8	1312.32	80	9	1273.75	85
10	1220.72	52	11	1132.01	10	12	1025.94	63
13	970.019	73	14	862.989	89	15	815.742	81
16	774.279	64	17	732.817	65	18	702.926	94
19	647.965	94	20	584.325	92	21	522.615	80
22	490.795	93	23	468.617	95	24	454.154	87
25	446.44	86	26	419.442	87			

Ethyl 2-(1,1-dimethyldecylsulfanylmethyl)prop-2-enoate

^{1}H-NMR (300 MHz, $CDCl_3$)

GA_211109.10.fid
ECO-3-061 carac
Proton_ns8_d1=10s CDCl3 /opt service 44

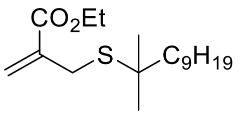

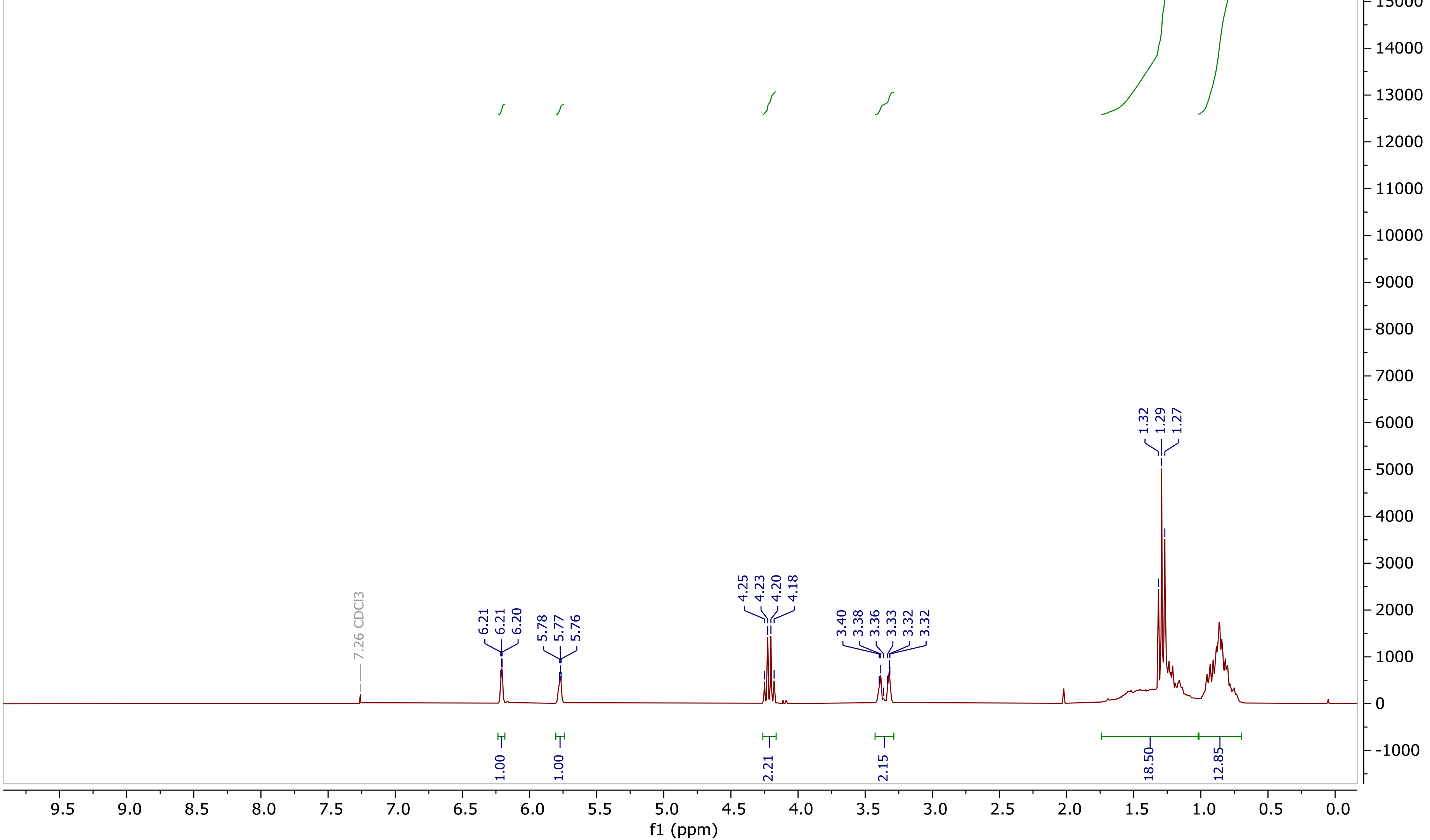

Ethyl 2-(1,1-dimethyldecylsulfanylmethyl)prop-2-enoate

^{1}H-NMR (300 MHz, $CDCl_3$)

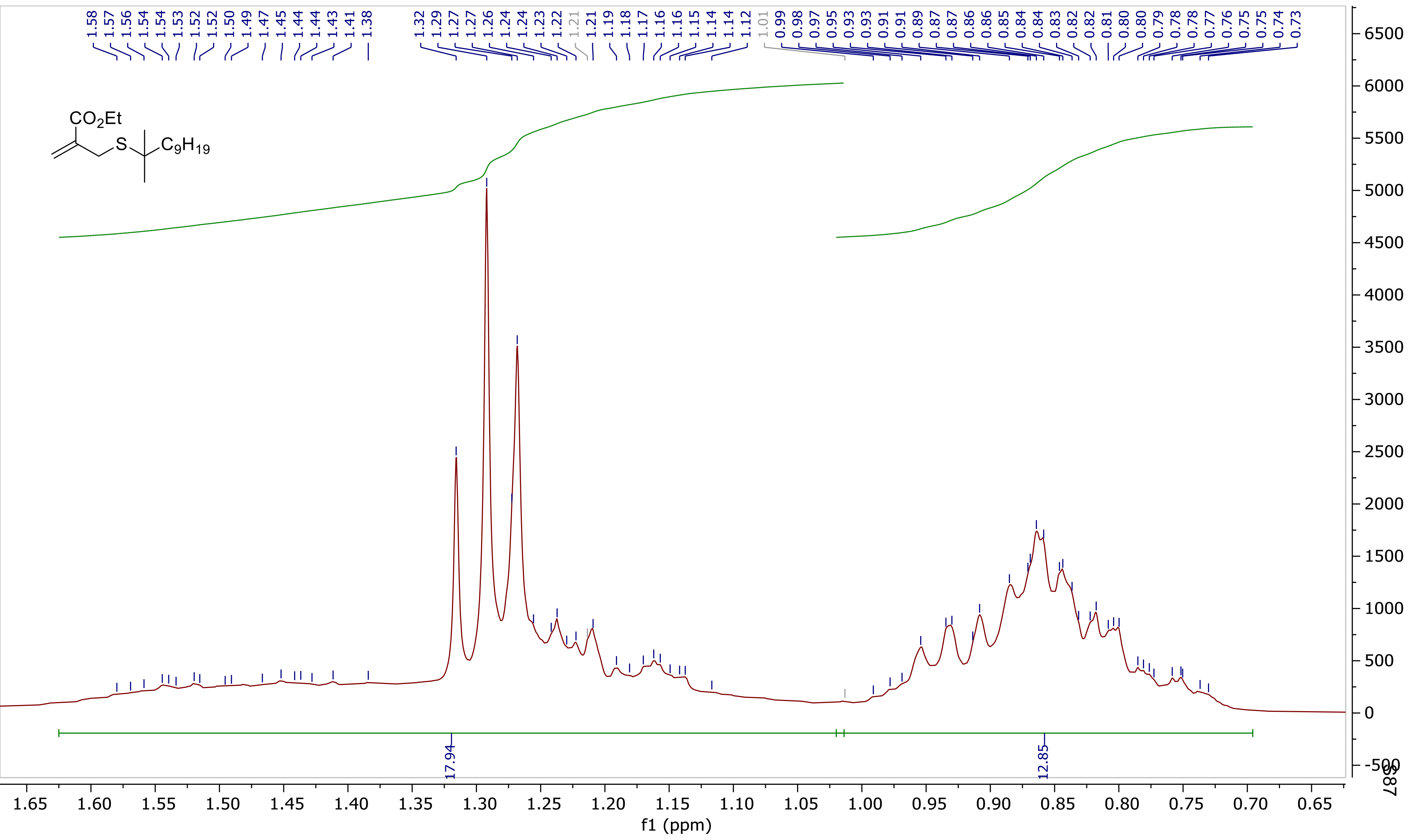

Ethyl 2-(1,1-dimethyldecylsulfanylmethyl)prop-2-enoate

^{13}C-NMR (75 MHz, $CDCl_3$)

GA_211109.11.fid
ECO-3-061 carac
Carbon_ns512 CDCl3 /opt service 44

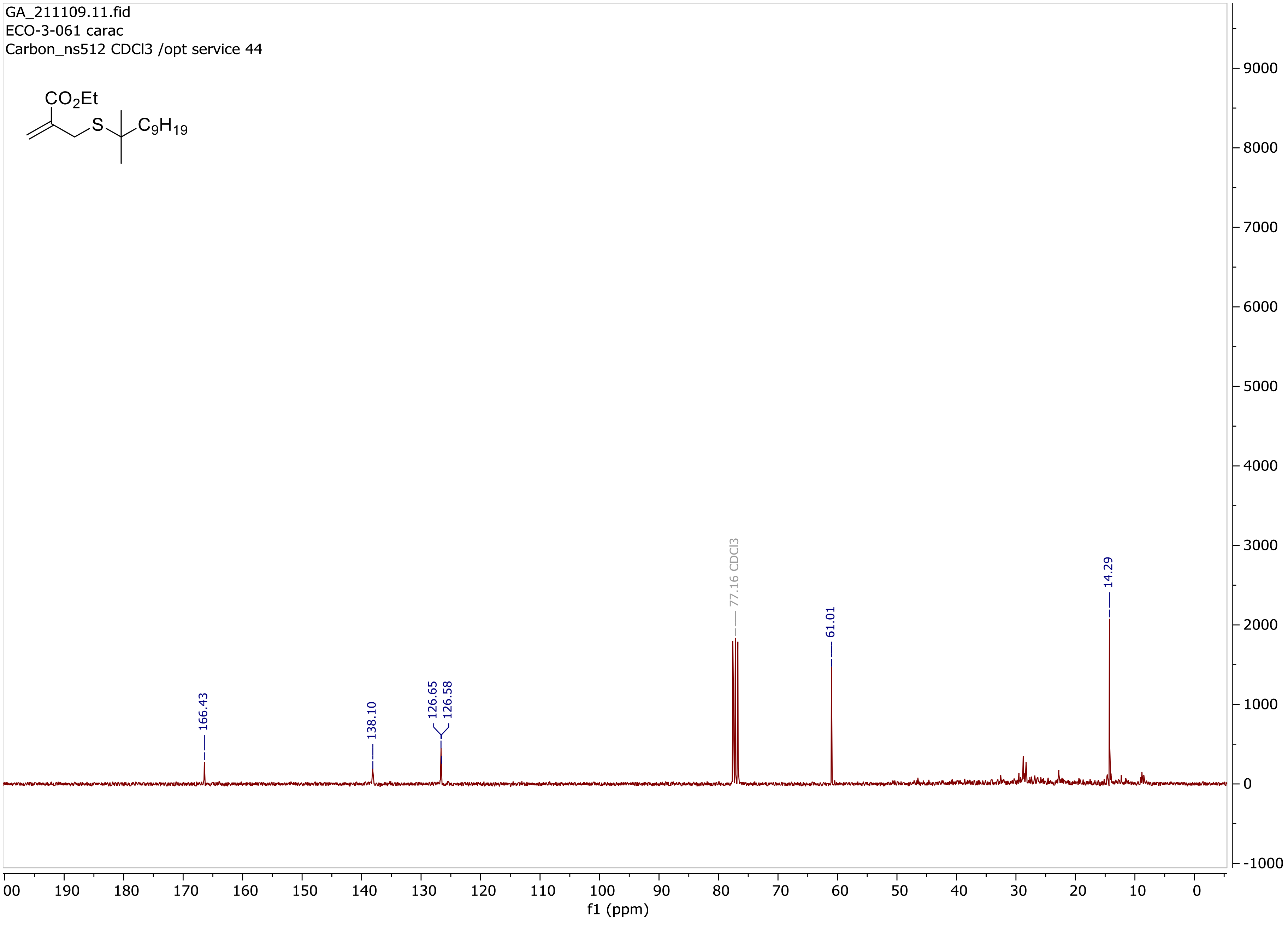

Ethyl 2-(1,1-dimethyldecylsulfanylmethyl)prop-2-enoate

^{13}C-NMR (75 MHz, $CDCl_3$)

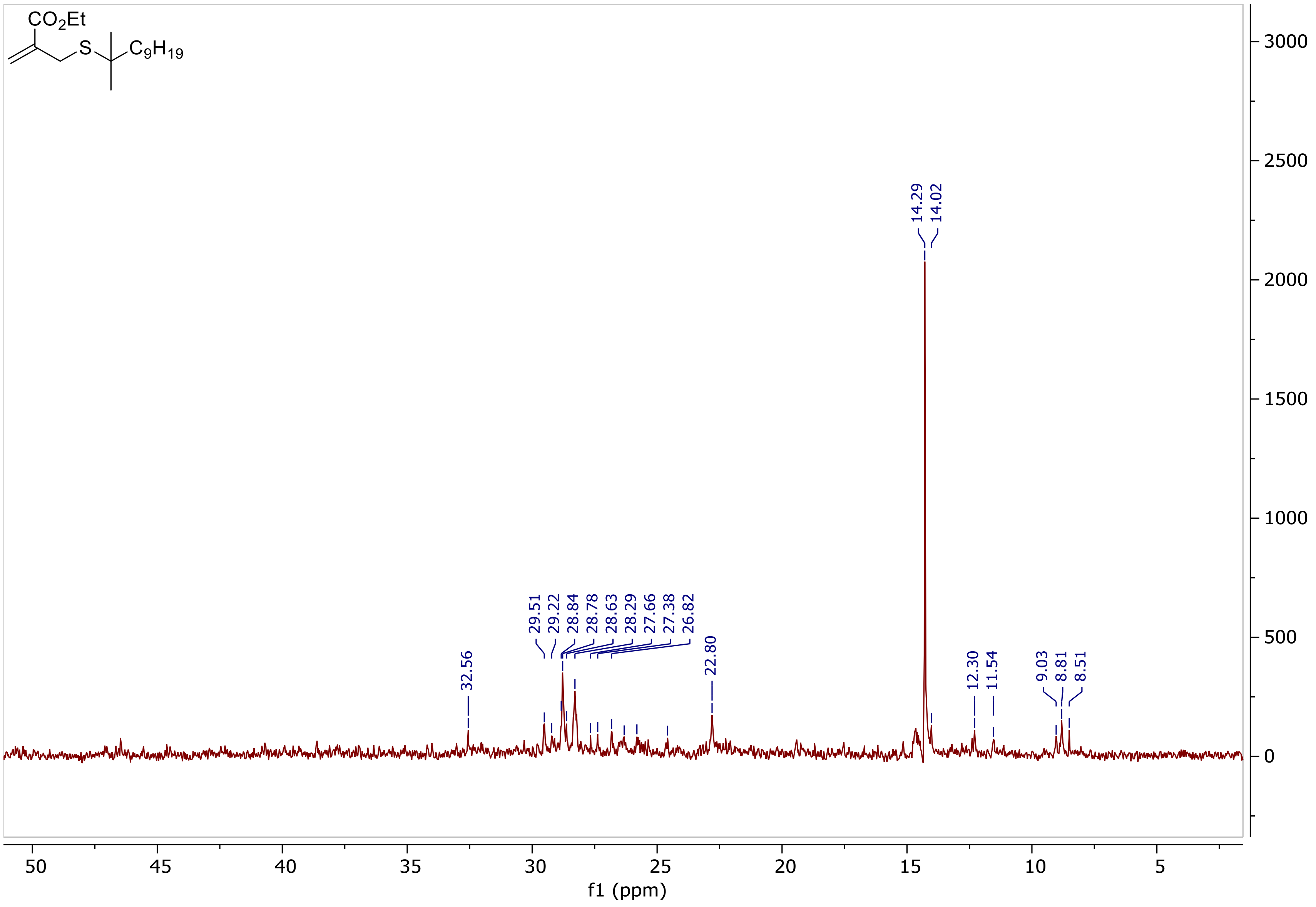

Ethyl 2-(1,1-dimethyldecylsulfanylmethyl)prop-2-enoate

^{13}C-NMR (75 MHz, $CDCl_3$)

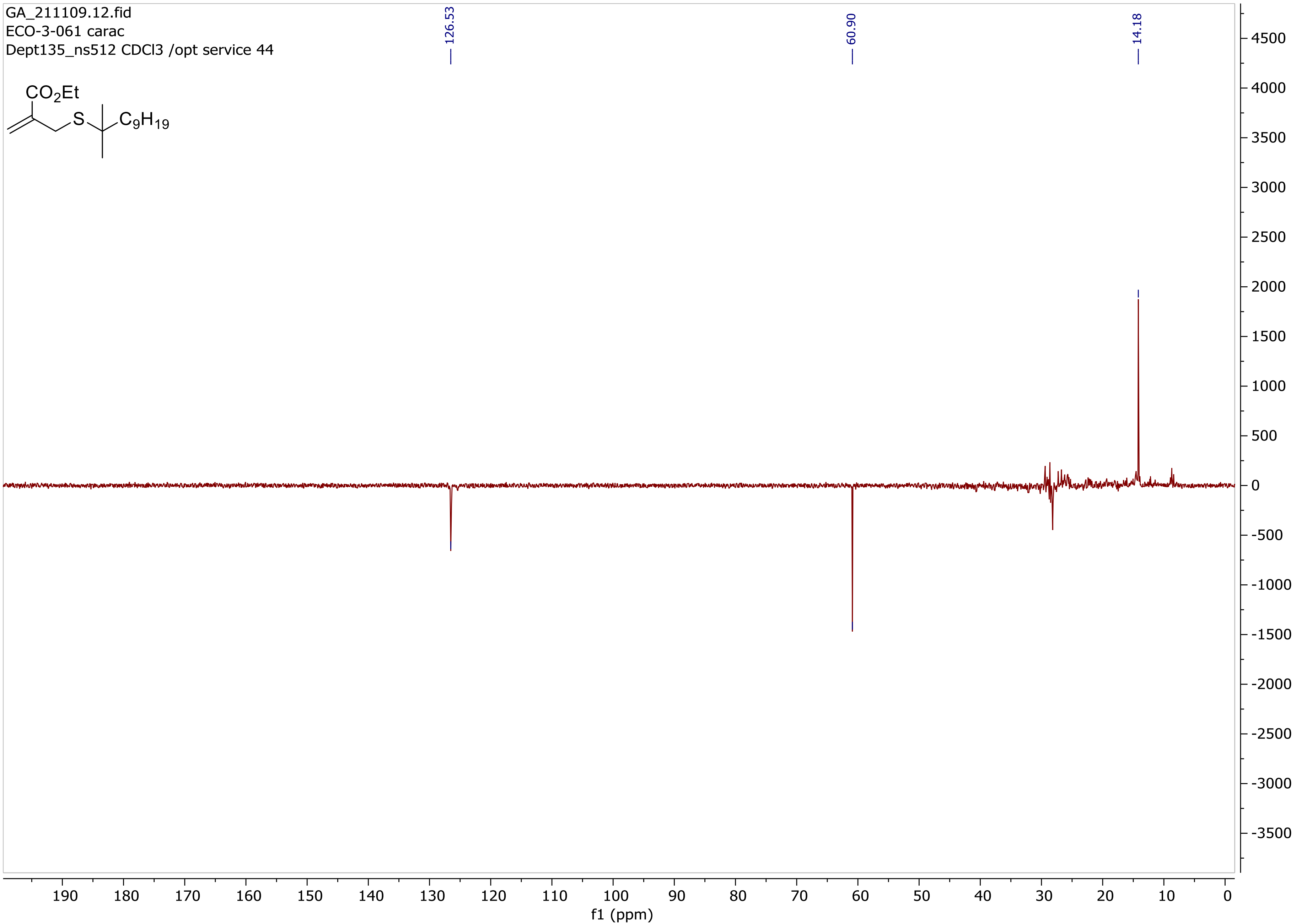

Ethyl 2-(1,1-dimethyldecylsulfanylmethyl)prop-2-enoate

FT-IR, ATR-diamond

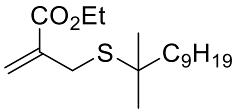

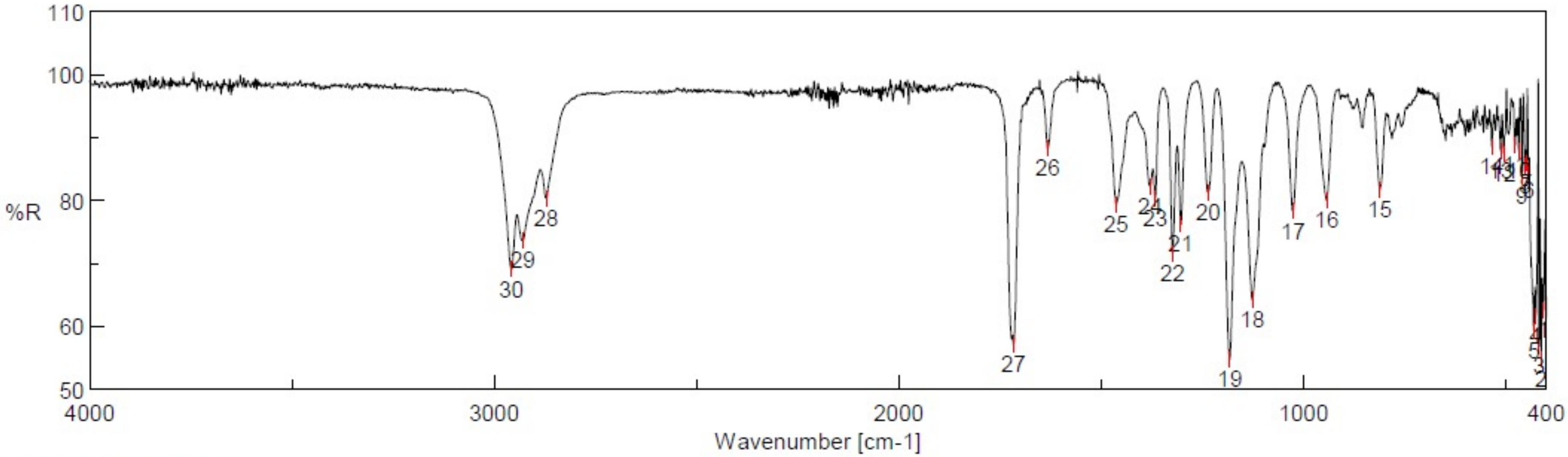

[Result of Peak Picking]

No.	Position	Intensity	No.	Position	Intensity	No.	Position	Intensity
1	406.907	62.5341	2	412.21	54.7823	3	417.513	56.8147
4	425.709	61.6499	5	430.53	59.3372	6	443.547	84.7056
7	449.333	85.9575	8	452.225	85.5099	9	459.939	83.8227
10	467.653	87.8551	11	476.813	88.8413	12	505.258	87.4684
13	512.49	87.9054	14	534.185	88.4645	15	810.92	81.7778
16	942.538	79.9937	17	1026.43	78.2643	18	1126.22	64.2497
19	1182.15	54.7329	20	1235.67	81.2639	21	1303.16	76.1814
22	1323.41	71.5493	23	1366.32	80.3491	24	1378.85	82.2067
25	1462.74	79.3291	26	1631.97	88.2679	27	1716.82	57.0244
28	2871.49	80.3648	29	2929.34	73.6198	30	2957.79	69.0602

2-ethoxycarbonylallyl 2,2-dimethylpropanoate

^{1}H-NMR (300 MHz, $CDCl_3$)

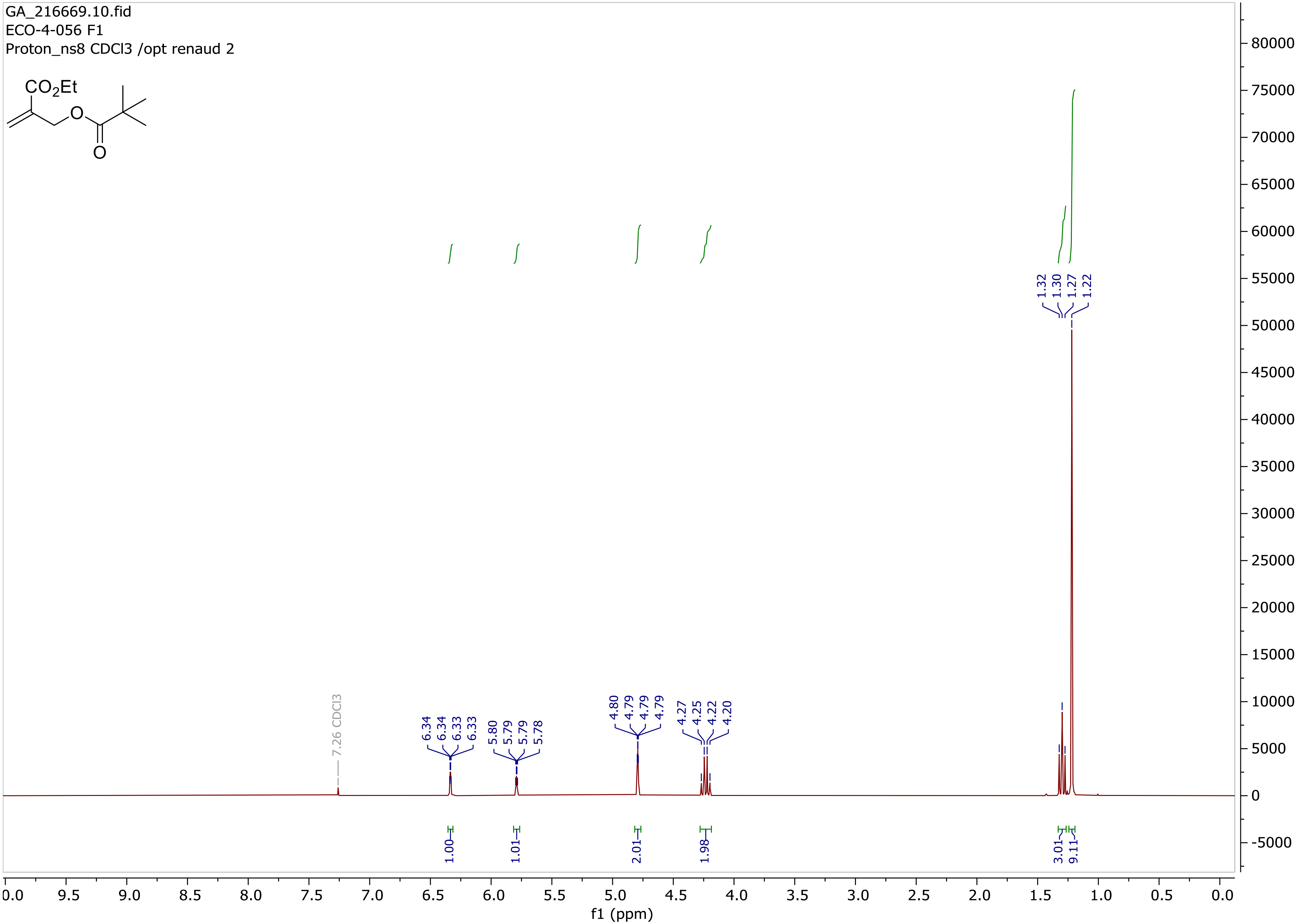

2-ethoxycarbonylallyl 2,2-dimethylpropanoate

^{1}H-NMR (300 MHz, $CDCl_3$)

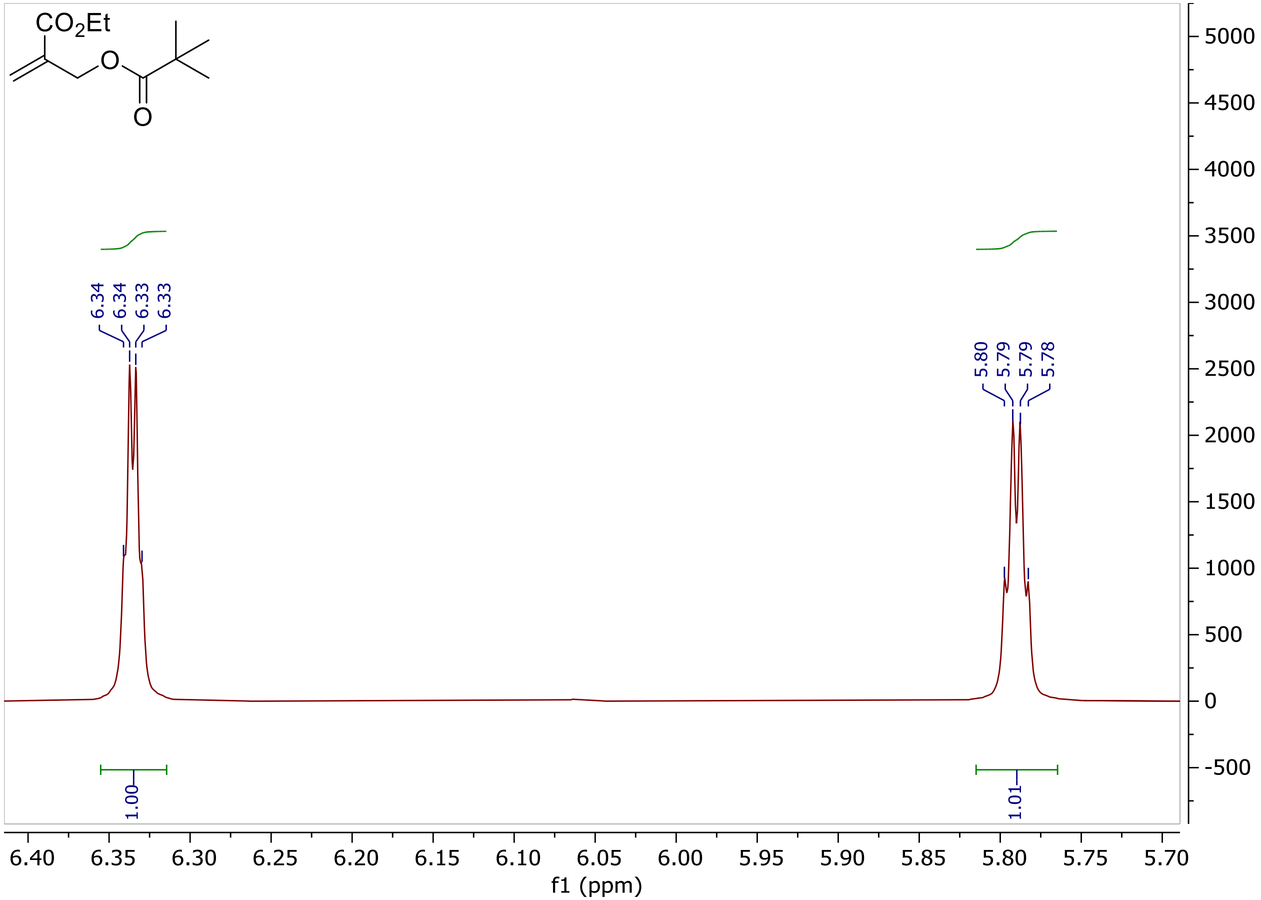

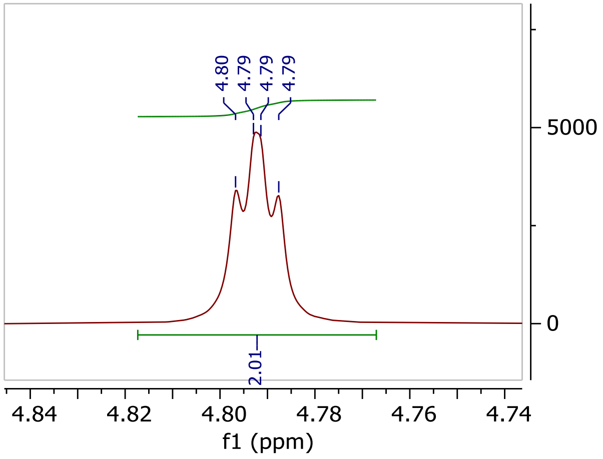

2-ethoxycarbonylallyl 2,2-dimethylpropanoate

^{13}C-NMR (75 MHz, $CDCl_3$)

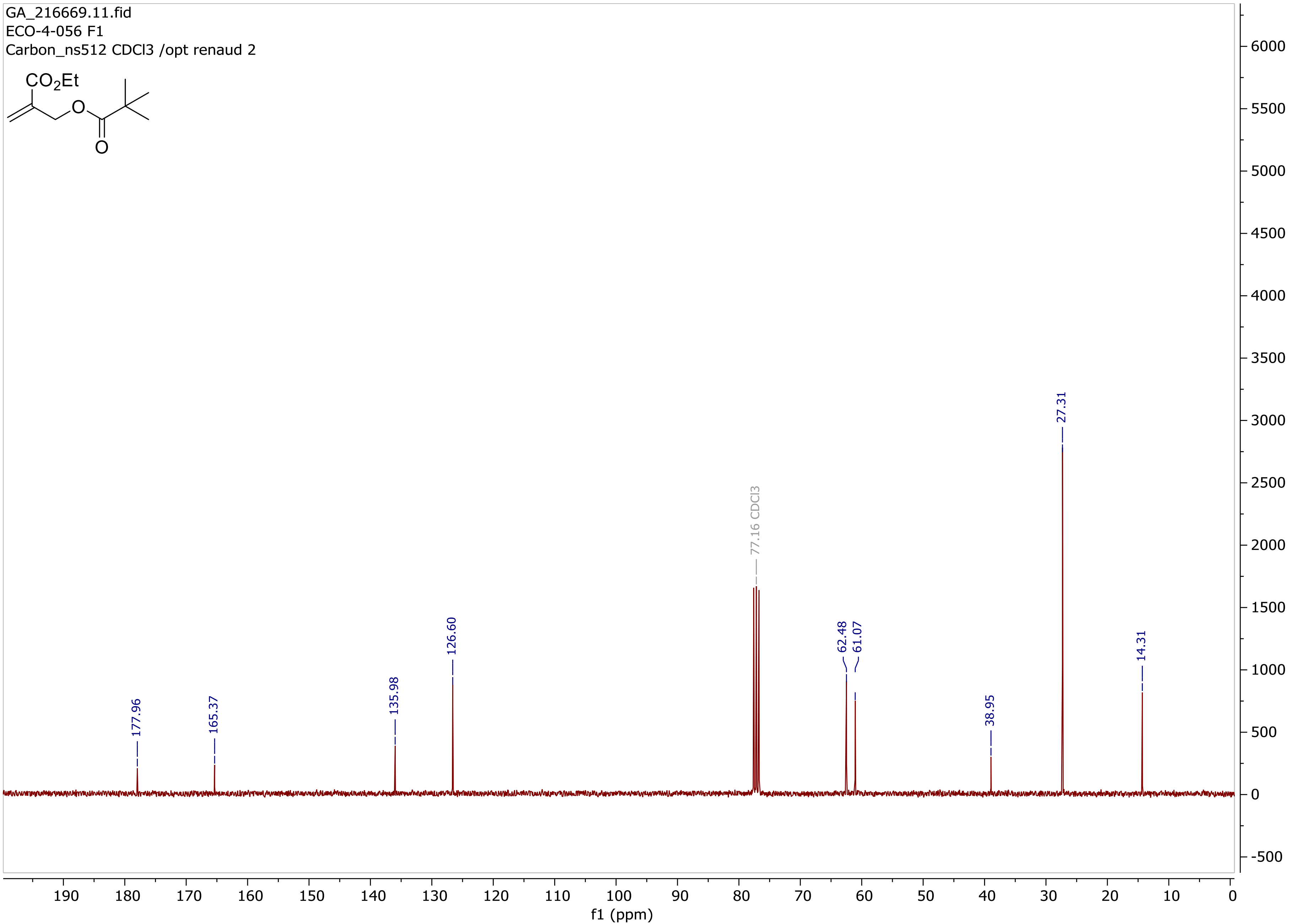

2-ethoxycarbonylallyl 2,2-dimethylpropanoate

^{13}C-NMR (75 MHz, $CDCl_3$)

GA_216669.12.fid
ECO-4-056 F1
Dept135_ns512 CDCl3 /opt renaud 2

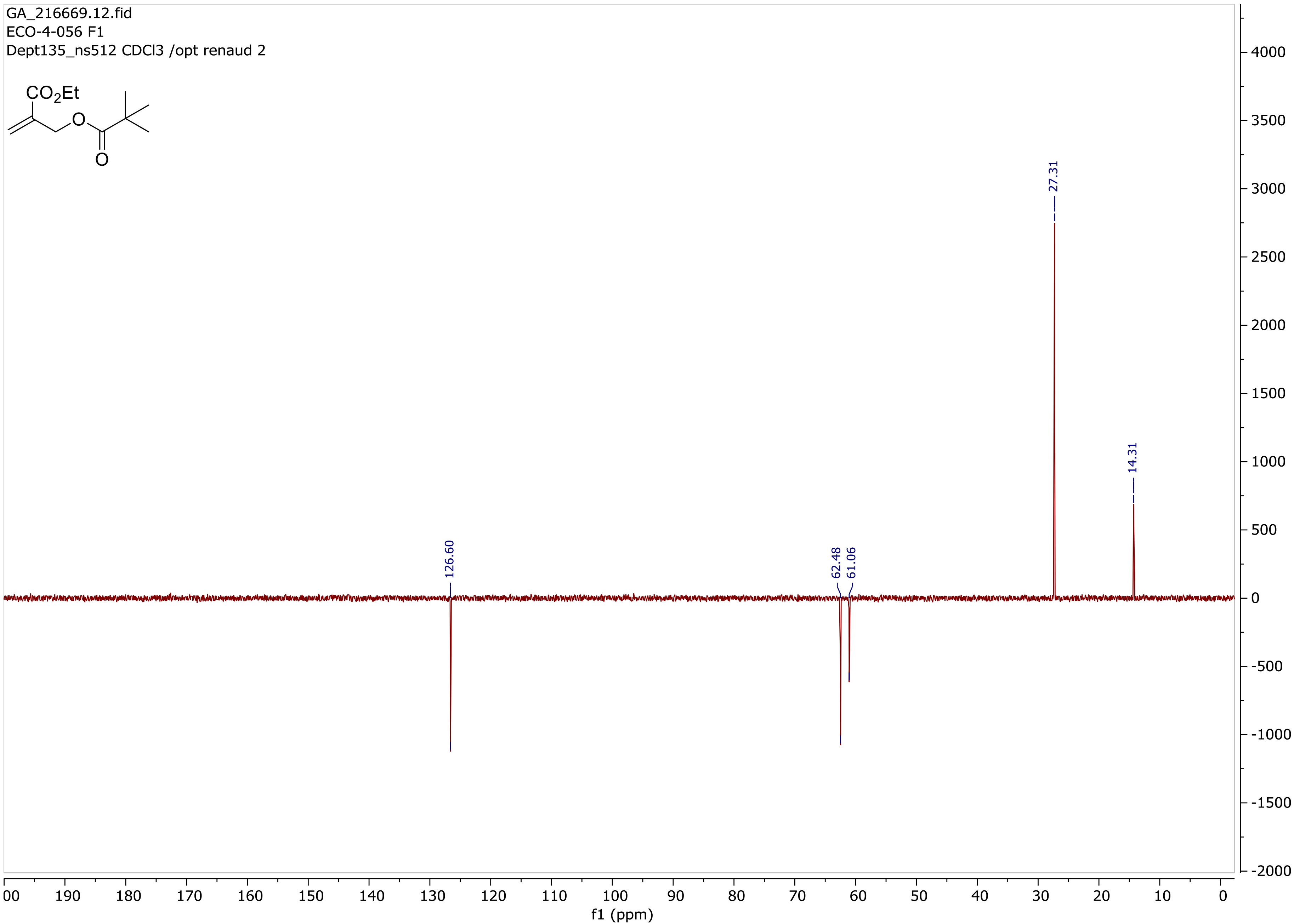

2-ethoxycarbonylallyl 2,2-dimethylpropanoate

FT-IR, ATR-diamond

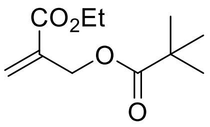

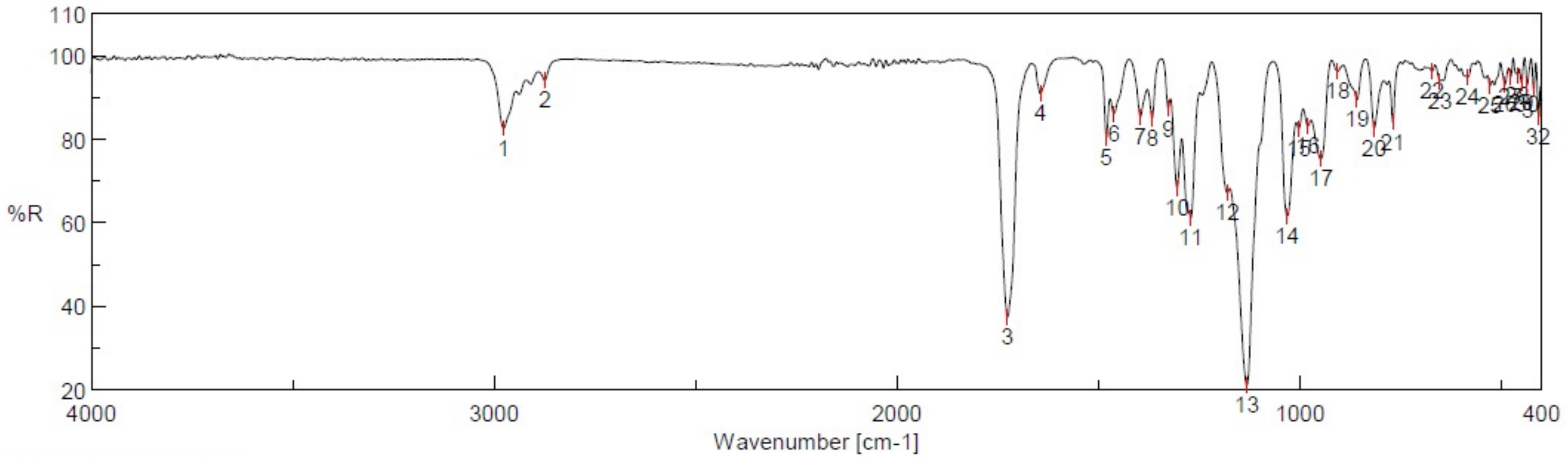

[Result of Peak Picking]

No.	Position	Intensity	No.	Position	Intensity	No.	Position	Intensity
1	2976.59	82.5862	2	2874.38	94.0438	3	1725.98	37.418
4	1644.02	90.8588	5	1480.1	80.2647	6	1461.78	86.0586
7	1396.21	85.3014	8	1366.32	85.0106	9	1326.79	87.2778
10	1304.61	68.3903	11	1270.86	61.0886	12	1180.22	67.0584
13	1132.01	21.0429	14	1031.73	61.6171	15	1002.8	82.4804
16	980.625	83.1581	17	947.842	75.3019	18	906.379	96.2743
19	858.168	89.494	20	815.742	82.5744	21	769.458	84.0797
22	672.071	96.2393	23	652.786	93.9354	24	583.361	94.8403
25	528.4	92.8208	26	491.759	92.9178	27	476.331	94.8961
28	459.939	95.3341	29	450.297	93.5449	30	435.834	93.0189
31	419.442	91.8013	32	407.871	85.2828			

2-ethoxycarbonylallyl 2,2-dimethylpropanoate

^{1}H-NMR (300 MHz, $CDCl_3$)

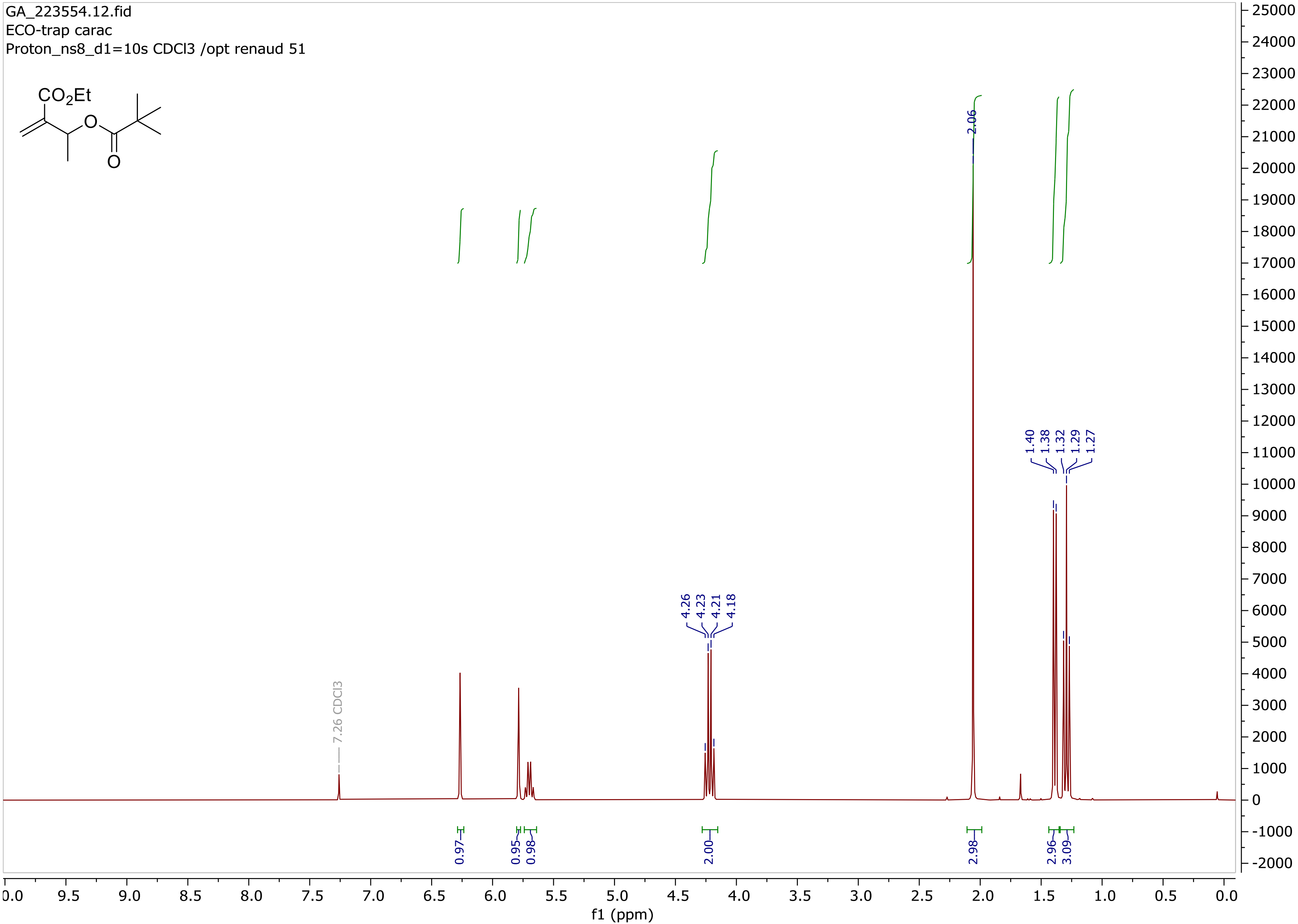

2-ethoxycarbonylallyl 2,2-dimethylpropanoate

^{1}H-NMR (300 MHz, $CDCl_3$)

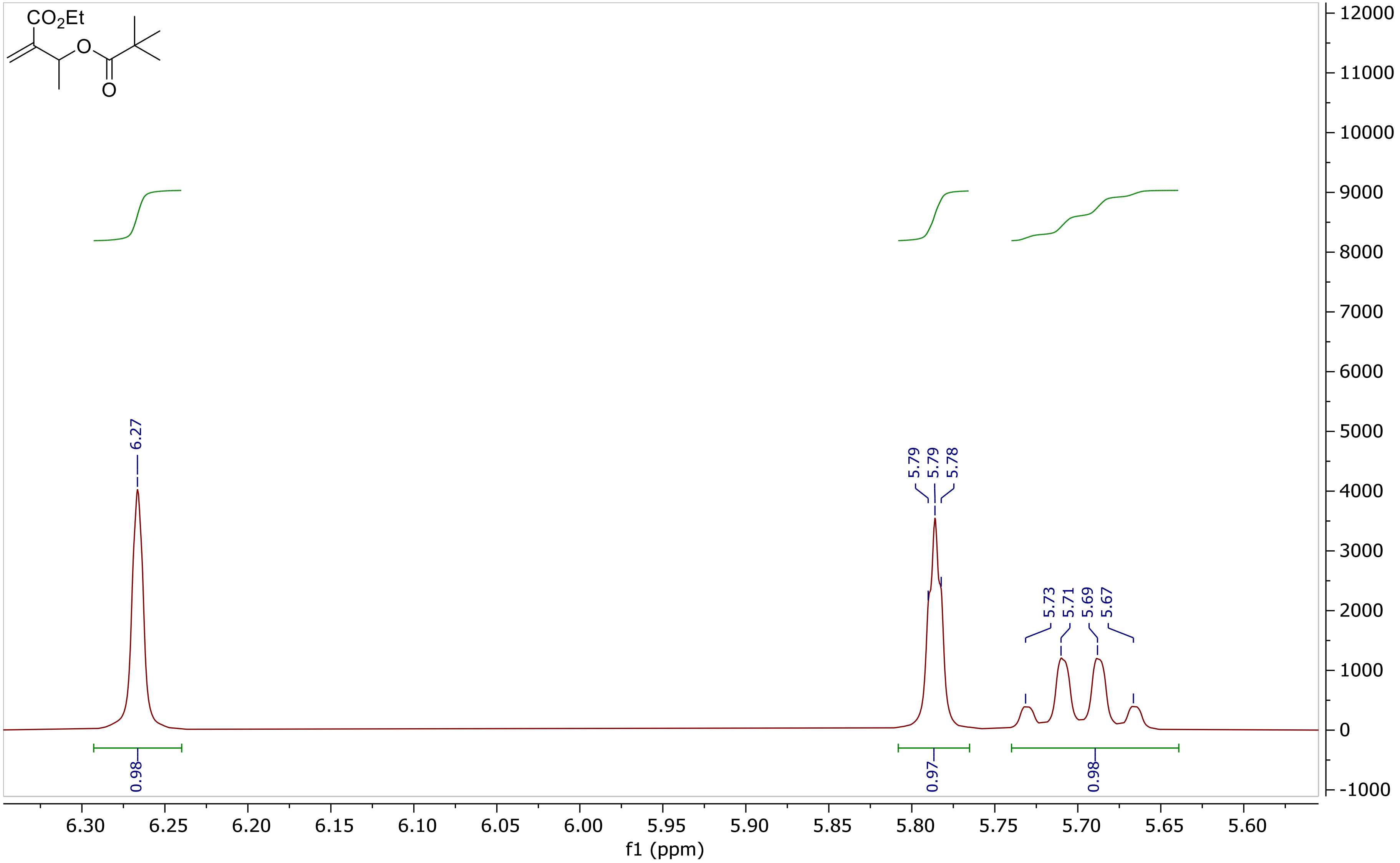

2-ethoxycarbonylallyl 2,2-dimethylpropanoate

^{13}C-NMR (75 MHz, $CDCl_3$)

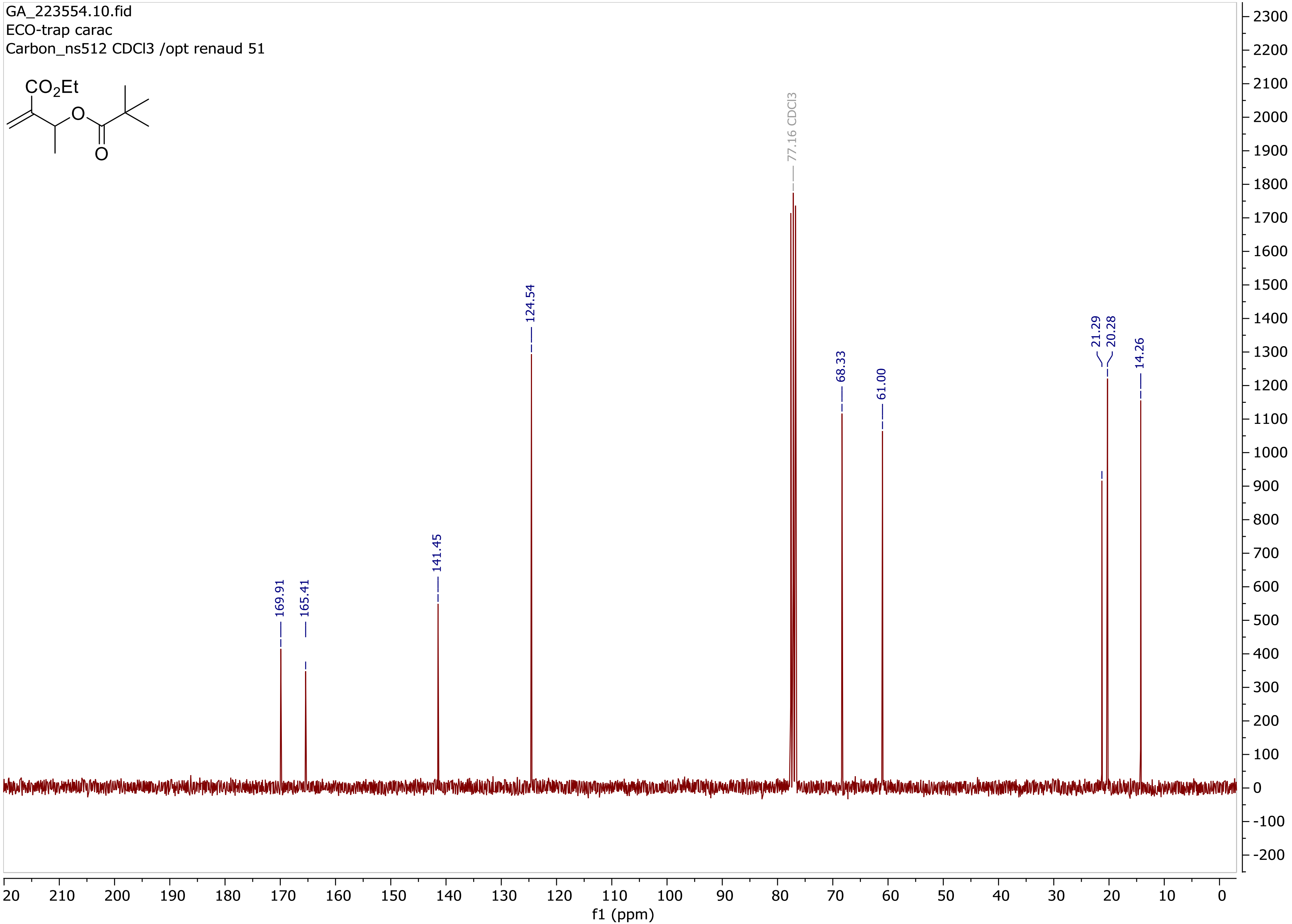

2-ethoxycarbonylallyl 2,2-dimethylpropanoate

^{13}C-NMR (75 MHz, $CDCl_3$)

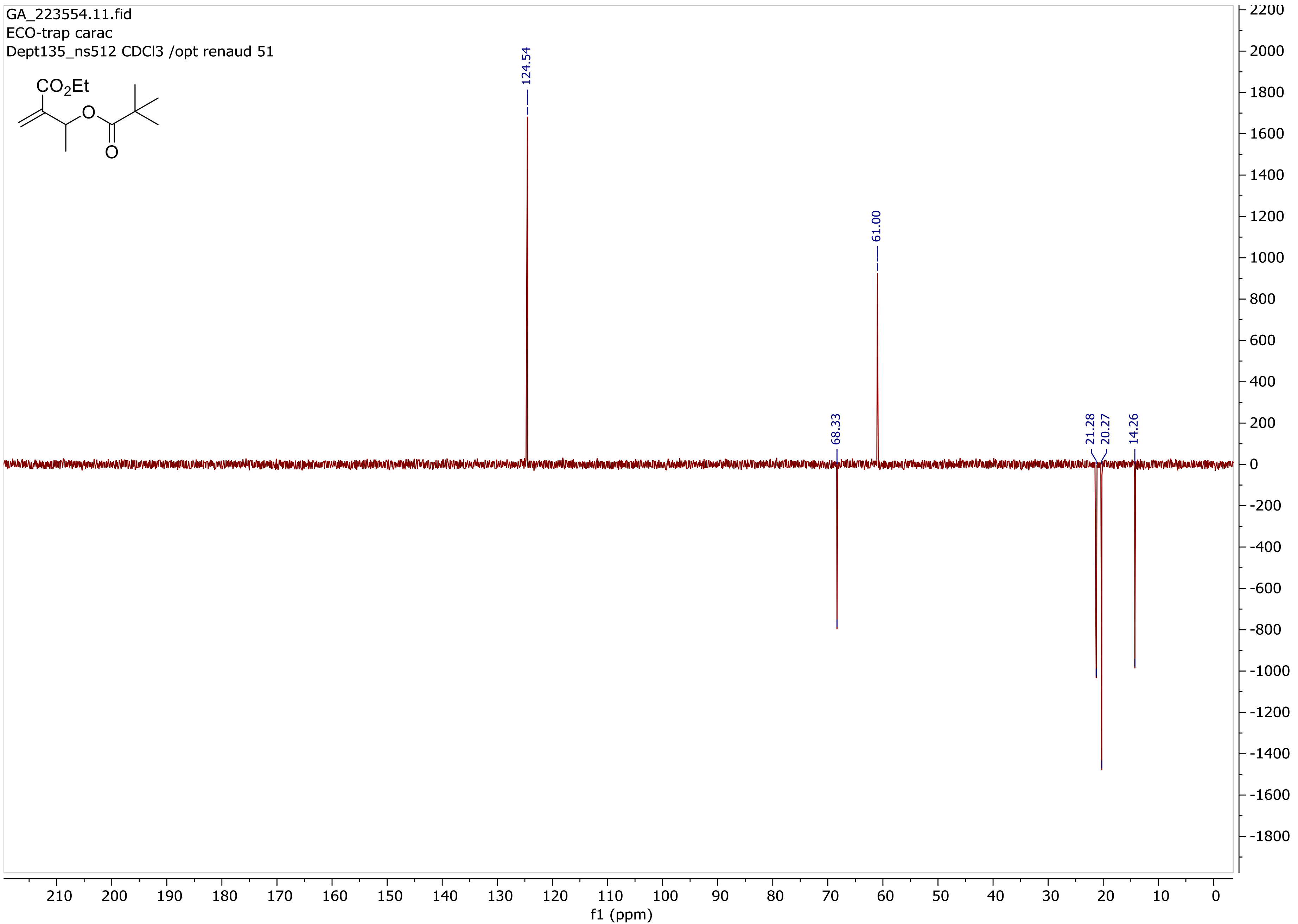

2-ethoxycarbonylallyl 2,2-dimethylpropanoate

FT-IR, ATR-diamond

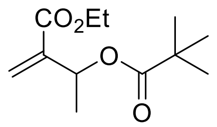

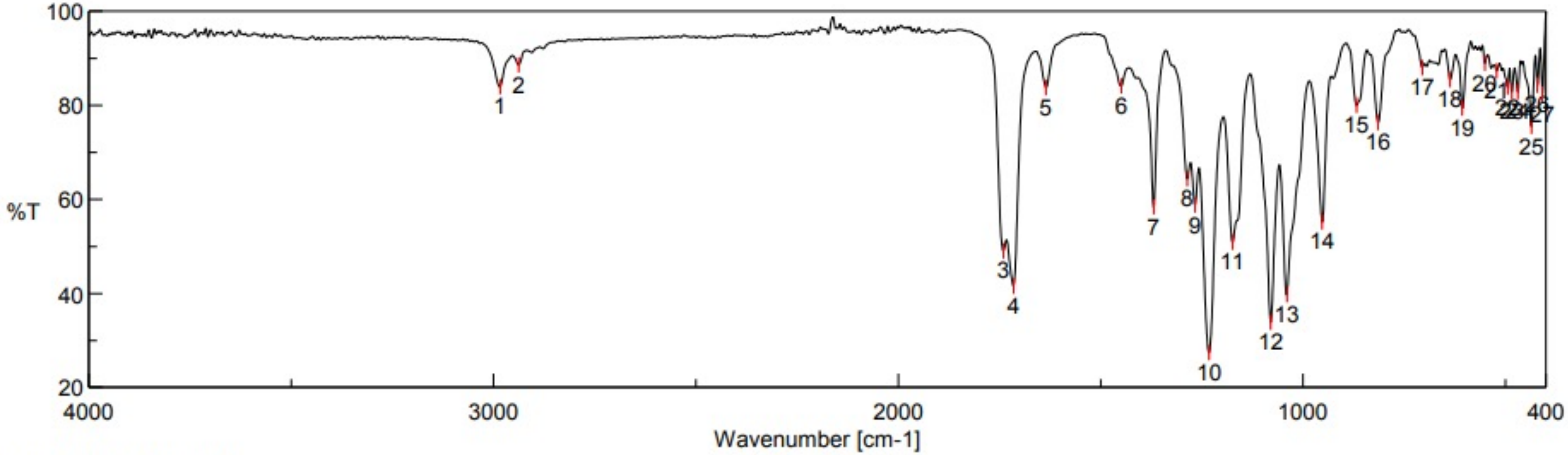

[Result of Peak Picking]

No.	Position	Intensity	No.	Position	Intensity	No.	Position	Intensity
1	2984.3	84	2	2938.02	89	3	1740.44	49
4	1715.37	41	5	1635.34	84	6	1449.24	84
7	1369.21	58	8	1286.29	64	9	1267	59
10	1232.29	27	11	1174.44	51	12	1079.94	34
13	1040.41	40	14	952.663	55	15	867.81	80
16	814.777	76	17	705.819	88	18	636.394	85
19	606.503	79	20	550.577	89	21	521.65	87
22	494.652	84	23	484.045	83	24	469.582	83
25	435.834	75	26	421.37	84	27	408.835	82

Bicyclic compounds

Ethyl 8-phenyl-8-azabicyclo[3.2.1]octane-3-carboxylate (**3aα**)

^{1}H-NMR (400 MHz, $CDCl_3$)

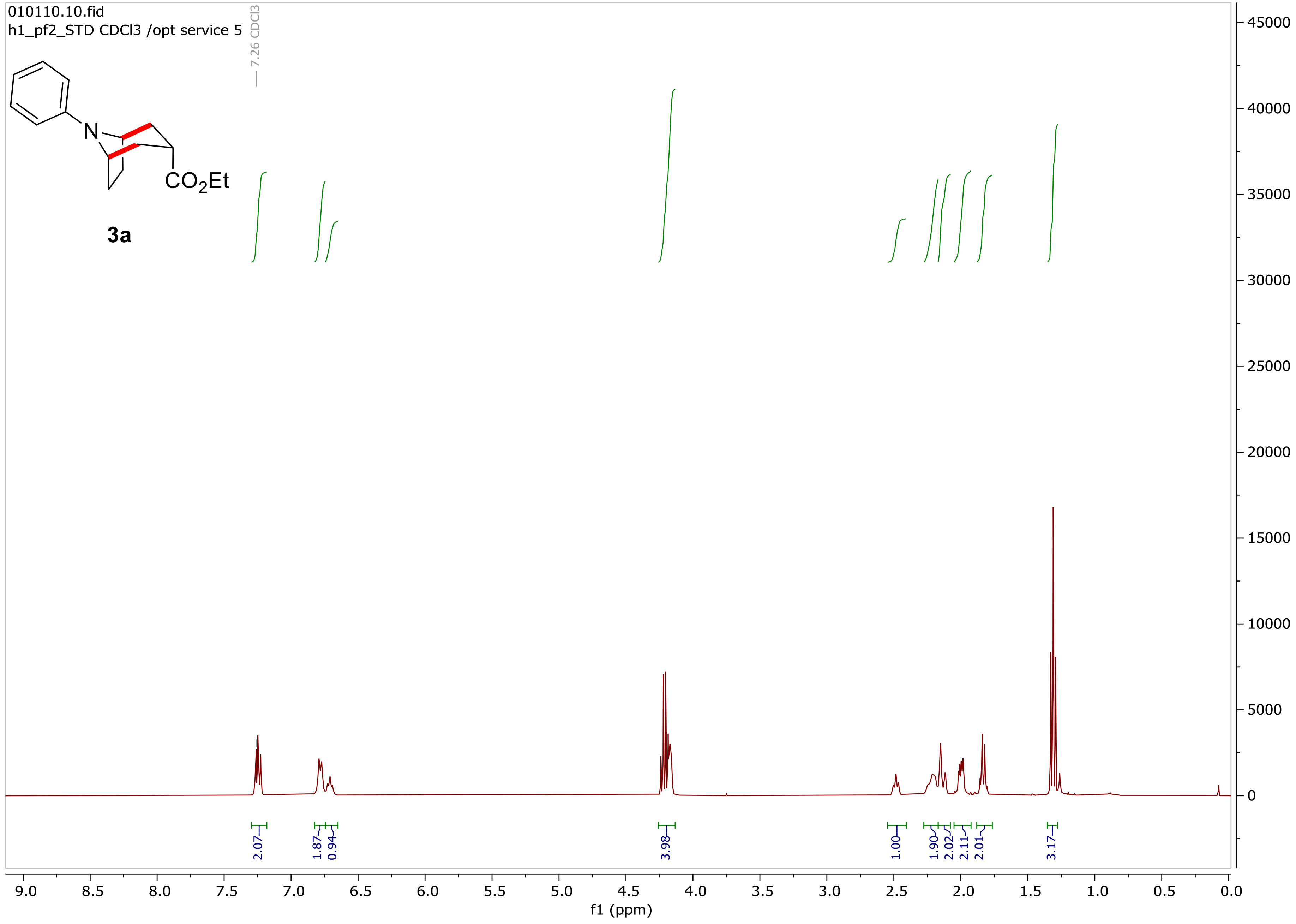

Ethyl 8-phenyl-8-azabicyclo[3.2.1]octane-3-carboxylate (**3aα**)

^{1}H-NMR (400 MHz, $CDCl_3$)

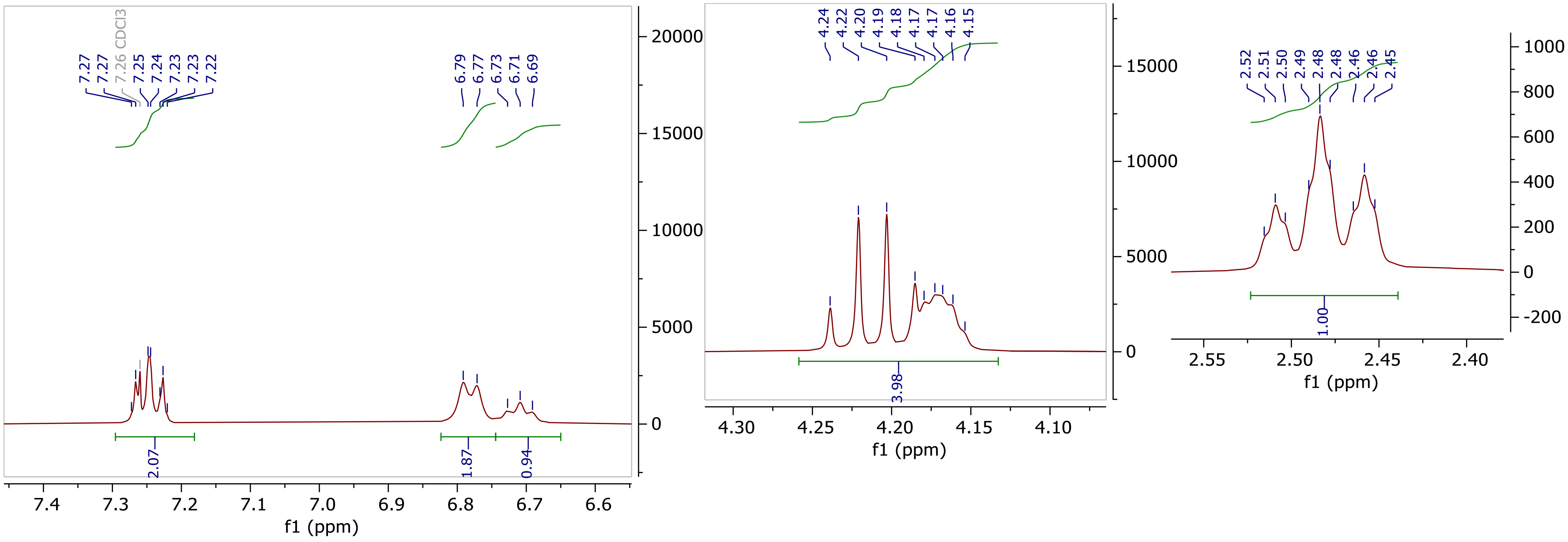

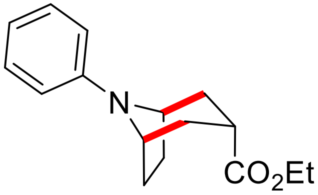

3a

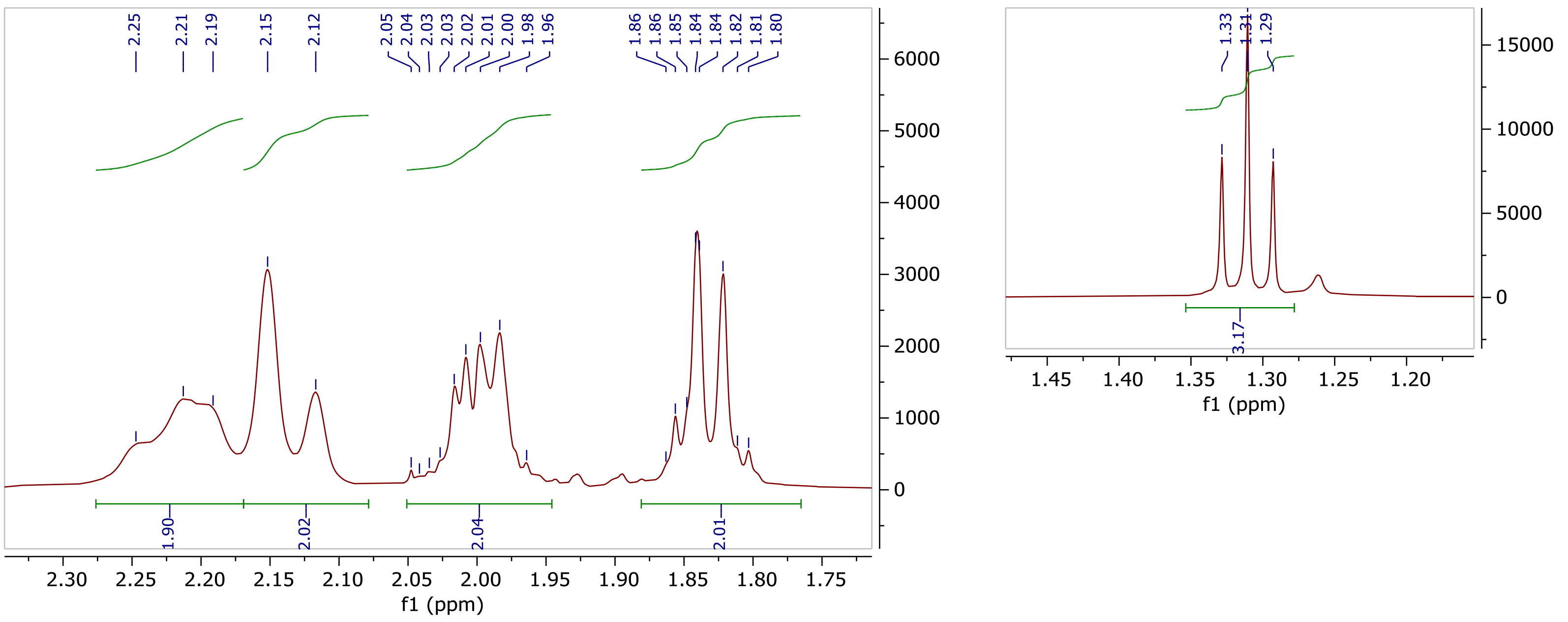

Ethyl 8-phenyl-8-azabicyclo[3.2.1]octane-3-carboxylate (**3aα**)

^{13}C-NMR (101 MHz, $CDCl_3$)

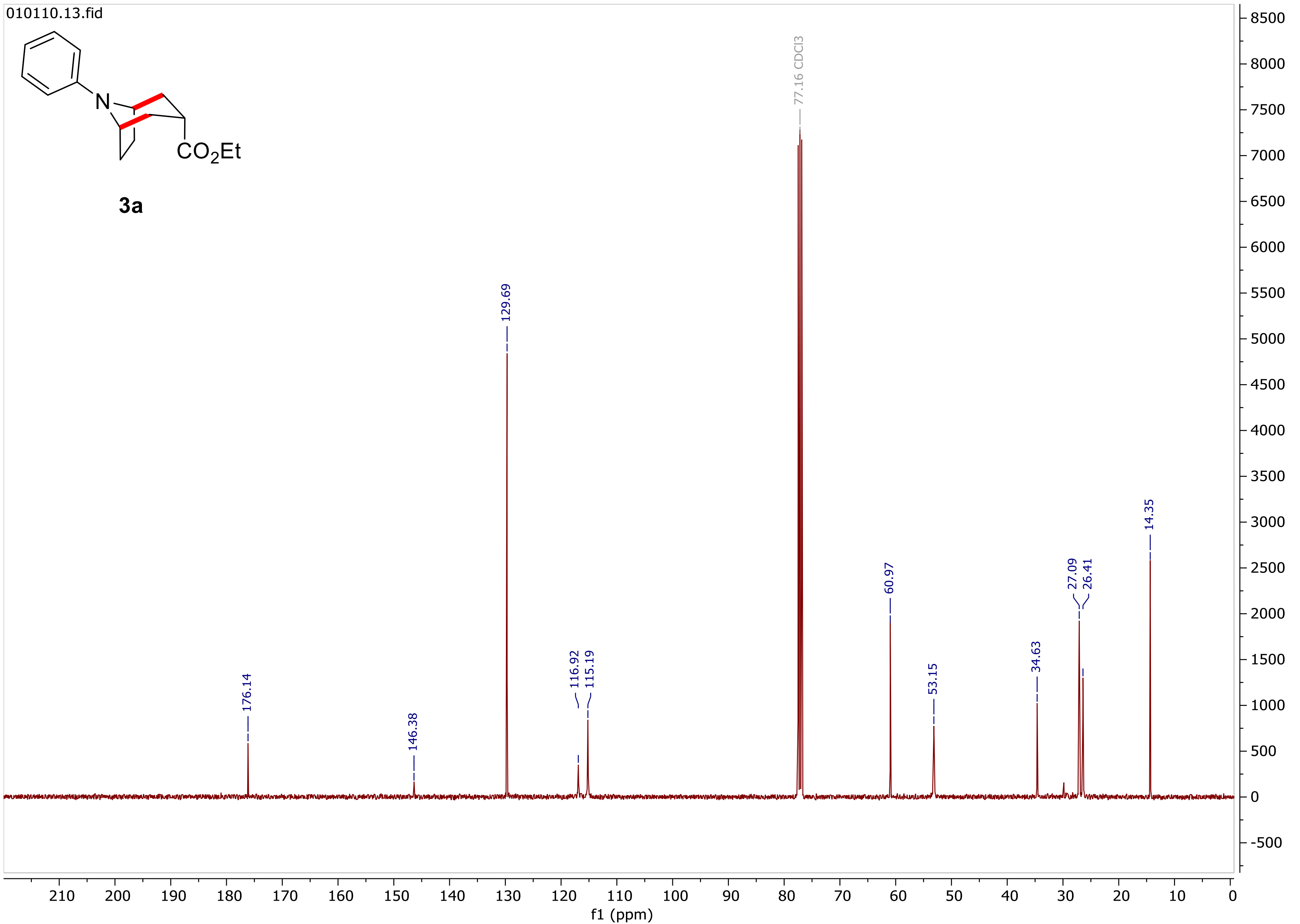

Ethyl 8-phenyl-8-azabicyclo[3.2.1]octane-3-carboxylate (**3aα**)

^{13}C-NMR (101 MHz, $CDCl_3$)

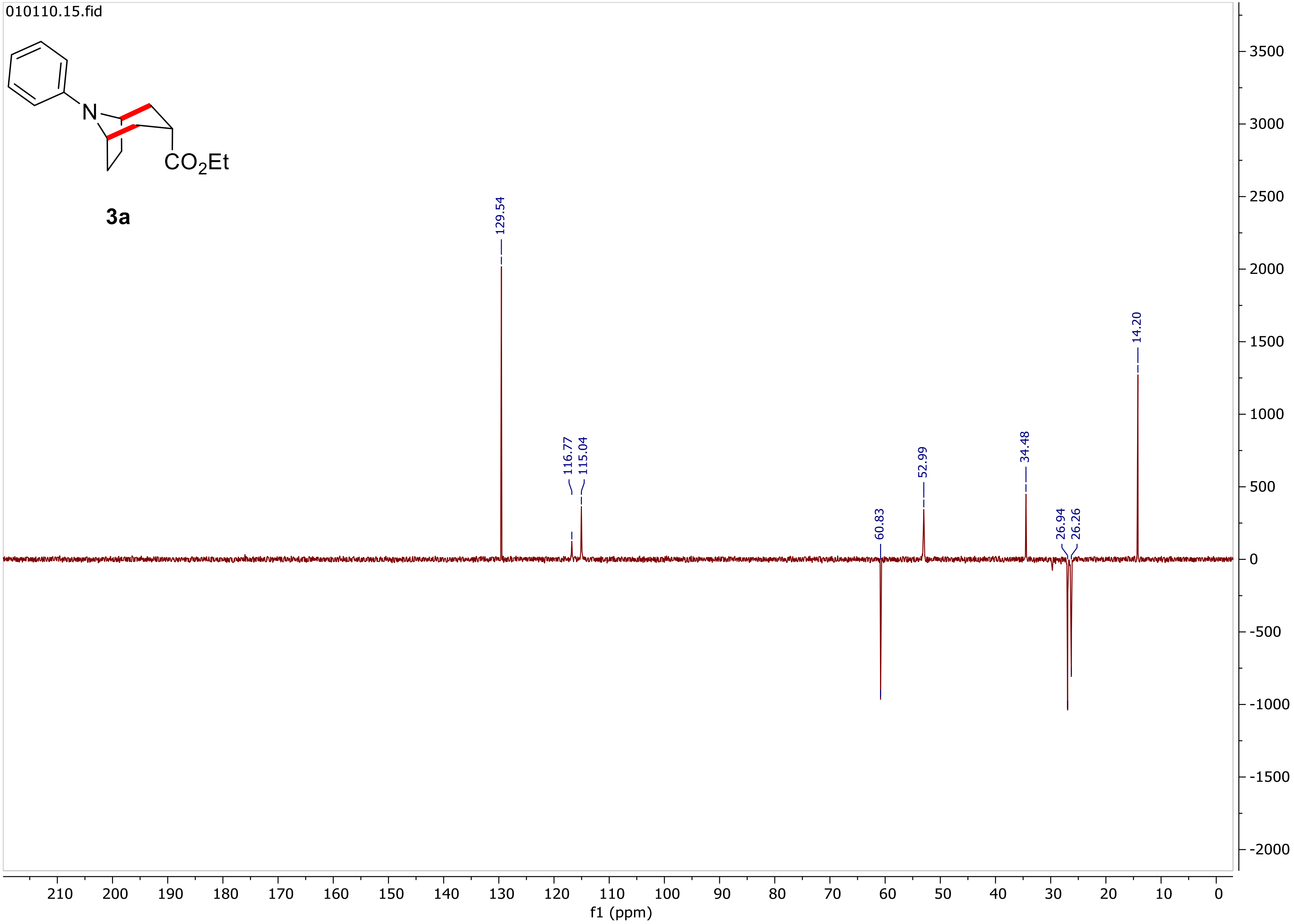

Ethyl 8-phenyl-8-azabicyclo[3.2.1]octane-3-carboxylate (**3aα**)

^1H, ^1H-COSY NMR (400 MHz, $CDCl_3$)

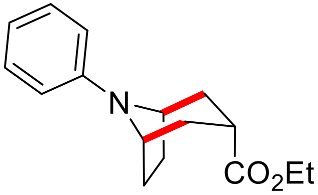

3a

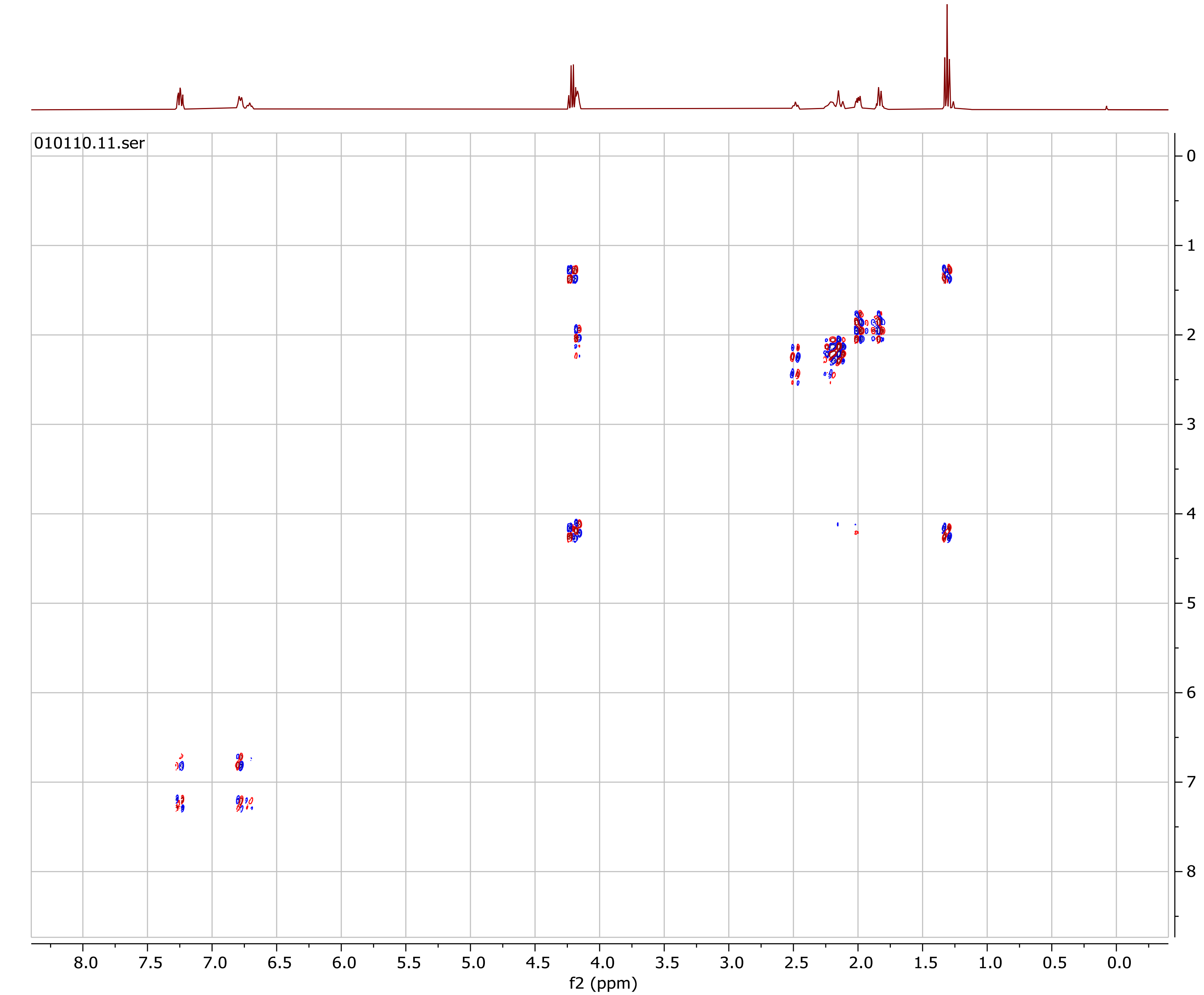

Ethyl 8-phenyl-8-azabicyclo[3.2.1]octane-3-carboxylate (**3aα**)

^{1}H, ^{13}C-HSQC NMR (400 MHz, $CDCl_3$)

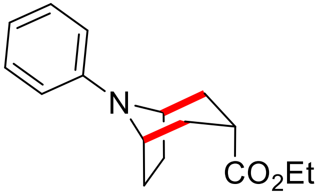

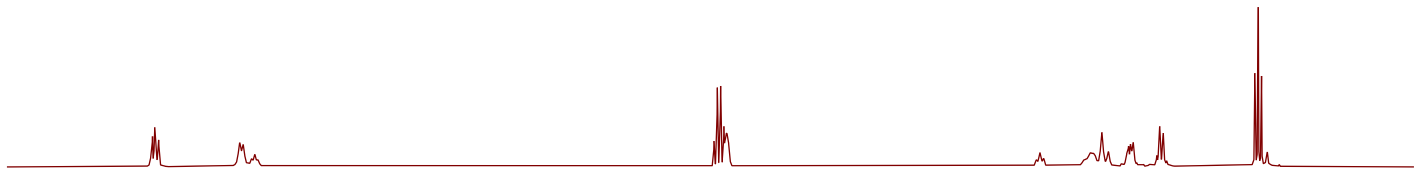

3a

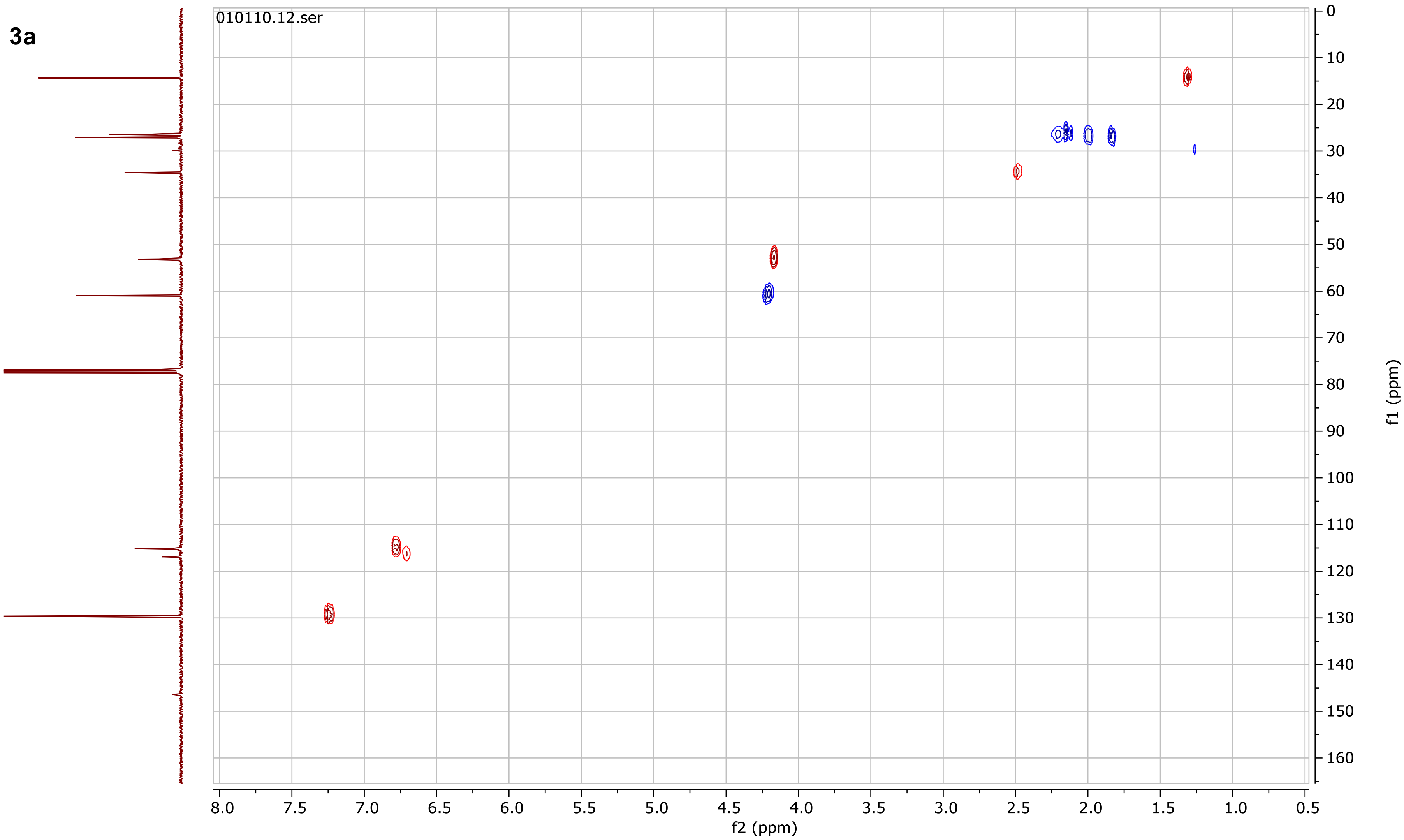

Ethyl 8-phenyl-8-azabicyclo[3.2.1]octane-3-carboxylate (**3aα**)

^{1}H, ^{13}C-HMBC NMR (400 MHz, $CDCl_3$)

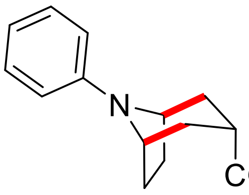

3a

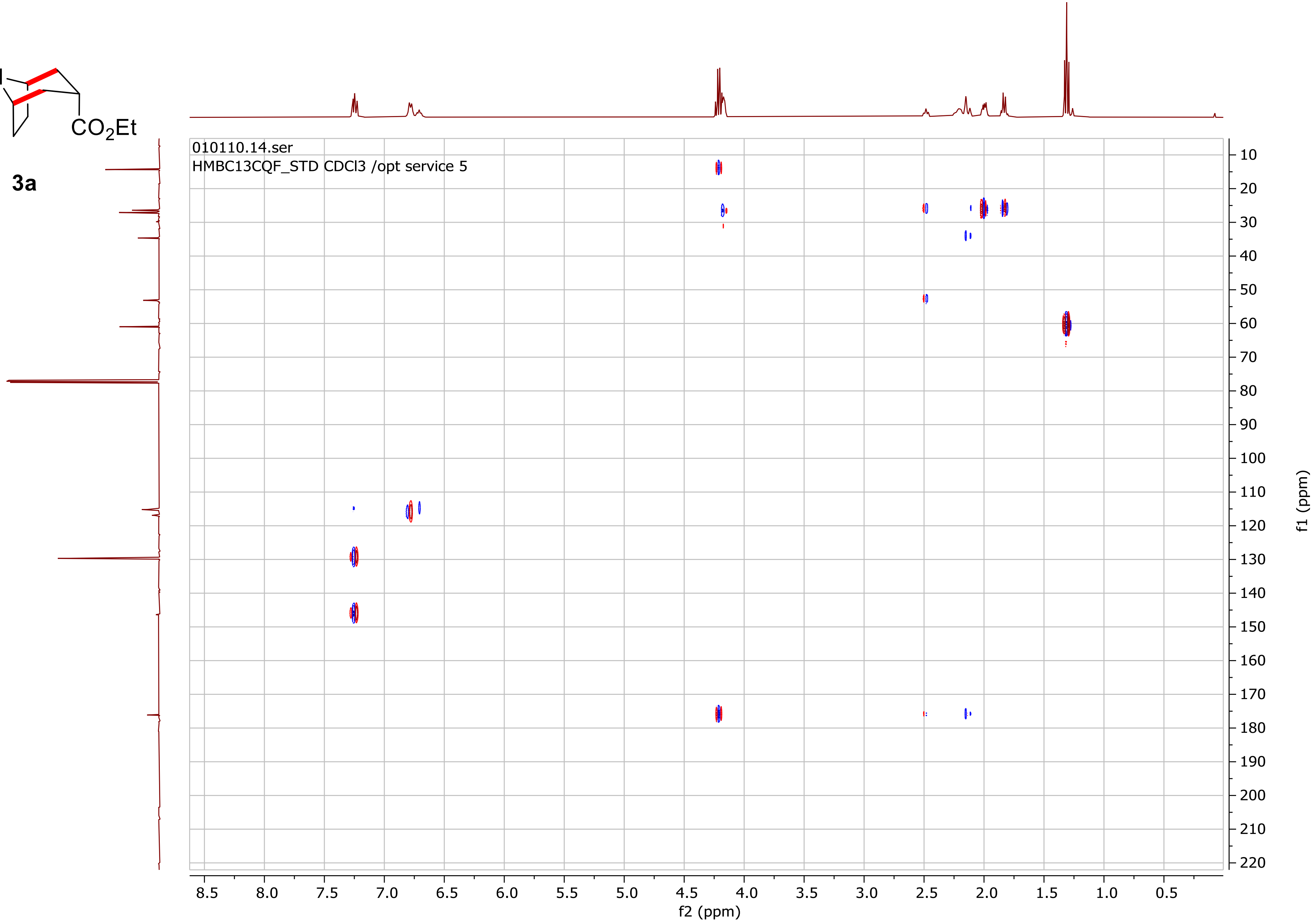

Ethyl 8-phenyl-8-azabicyclo[3.2.1]octane-3-carboxylate (**3aα**)

FT-IR, ATR-diamond

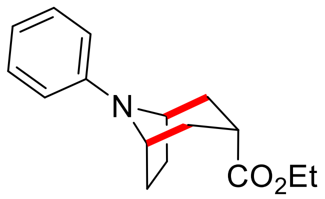

3a

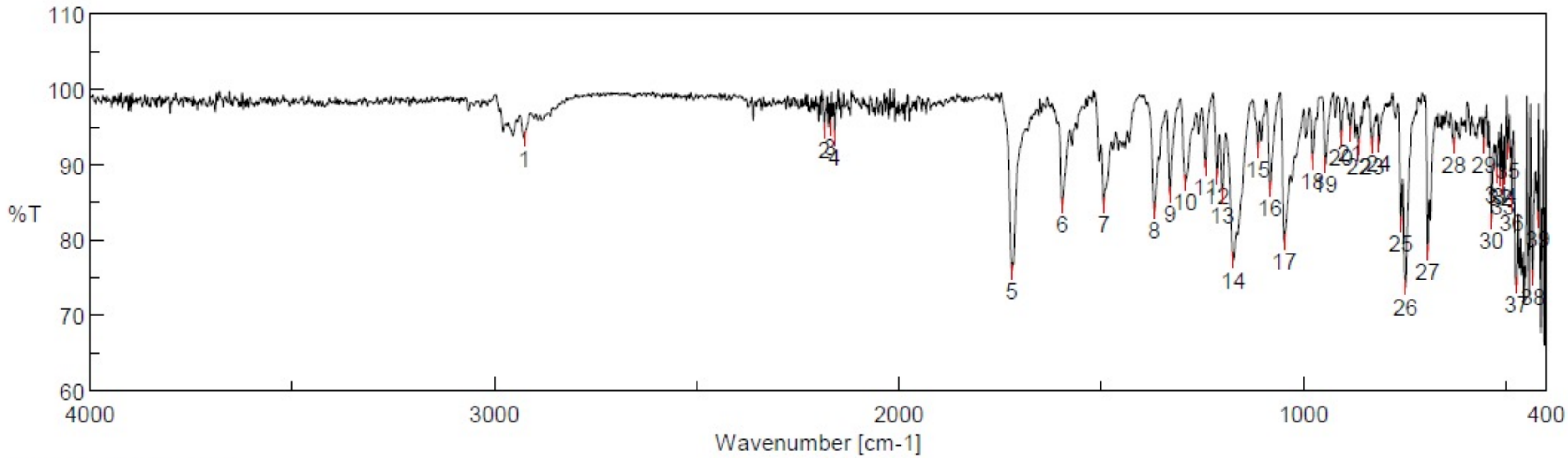

[Result of Peak Picking]

No.	Position	Intensity	No.	Position	Intensity	No.	Position	Intensity
1	2926.45	93	2	2184.47	95	3	2170.01	95
4	2158.92	94	5	1721.16	76	6	1596.77	85
7	1494.56	85	8	1368.73	84	9	1330.16	86
10	1292.07	88	11	1242.41	90	12	1213.97	88
13	1200.95	86	14	1173.47	77	15	1112.73	92
16	1083.32	87	17	1047.64	80	18	977.733	90
19	945.913	90	20	907.344	94	21	884.684	94
22	863.953	92	23	830.687	92	24	813.331	93
25	759.334	82	26	748.245	74	27	693.284	78
28	628.68	93	29	554.434	93	30	535.632	82
31	522.132	89	32	513.936	88	33	507.669	87
34	501.884	88	35	495.134	92	36	485.492	85
37	472.957	74	38	433.423	75	39	420.406	83

Ethyl 8-phenyl-8-azabicyclo[3.2.1]octane-3-carboxylate (**3aα**)

Xray analysis

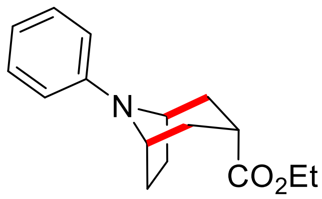

3a

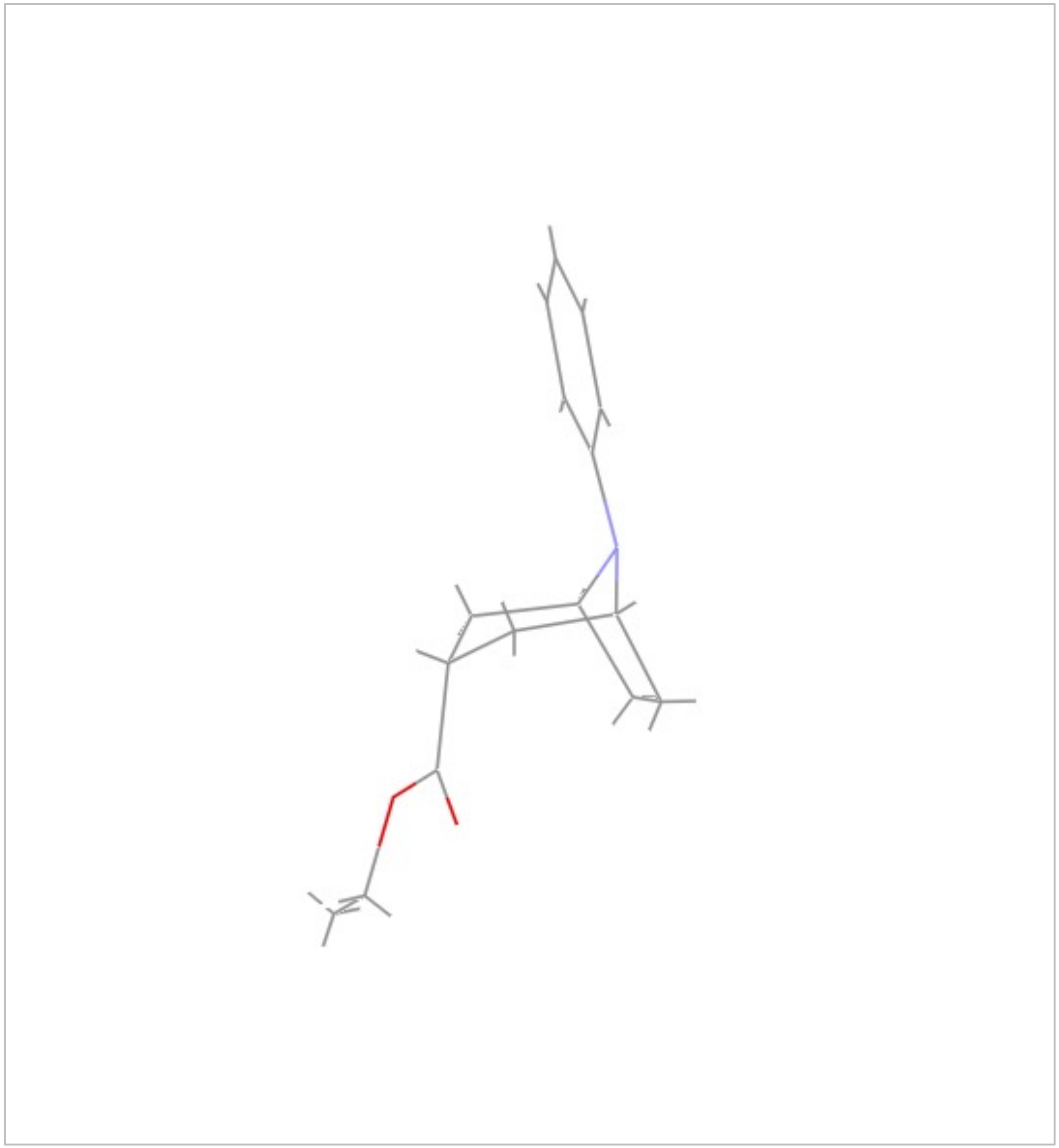

Ethyl 8-phenyl-8-azabicyclo[3.2.1]octane-3-carboxylate (**3aβ**)

^{1}H-NMR (300 MHz, $CDCl_3$)

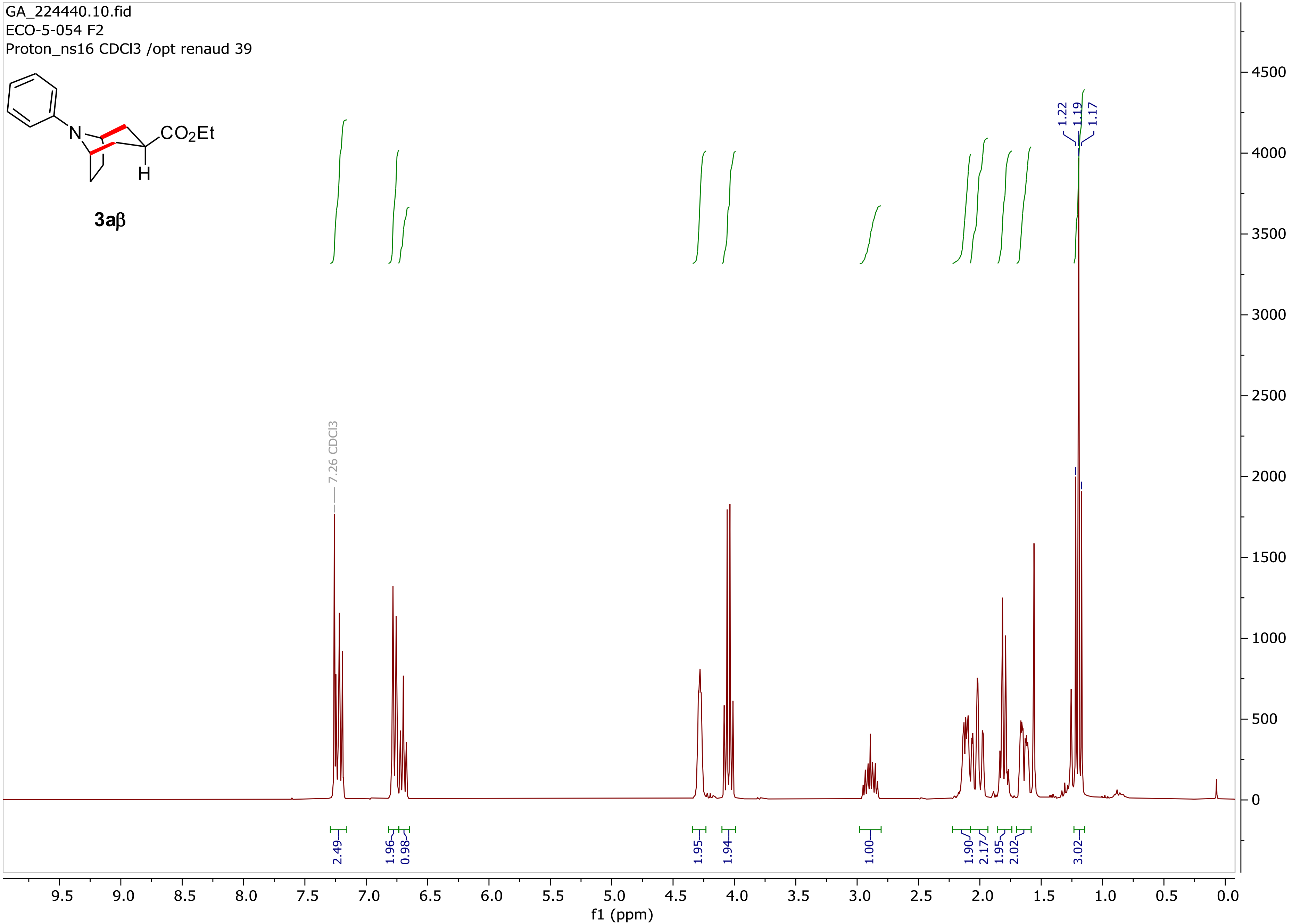

Ethyl 8-phenyl-8-azabicyclo[3.2.1]octane-3-carboxylate (**3aβ**)

^{1}H-NMR (300 MHz, $CDCl_3$)

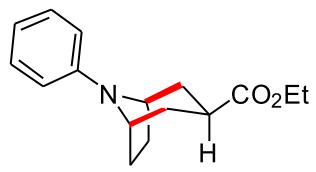

3aβ

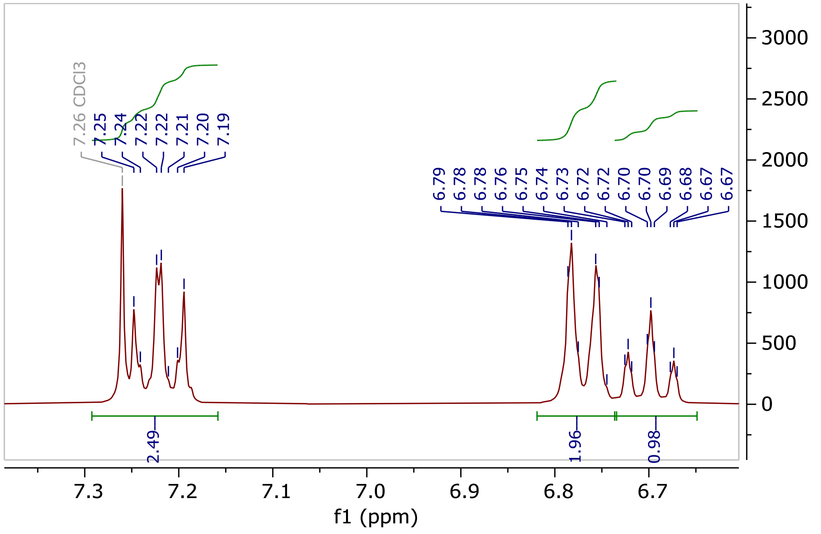

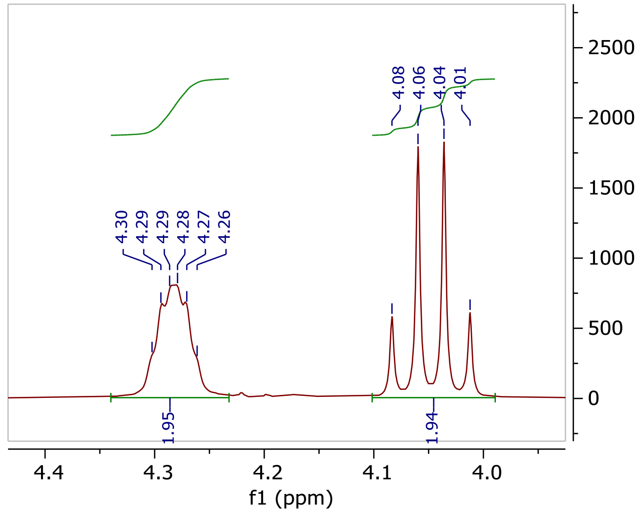

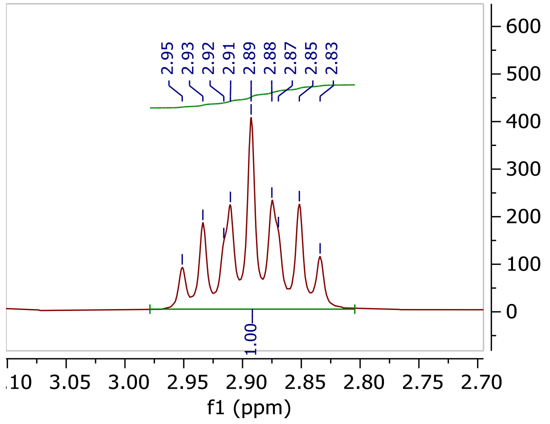

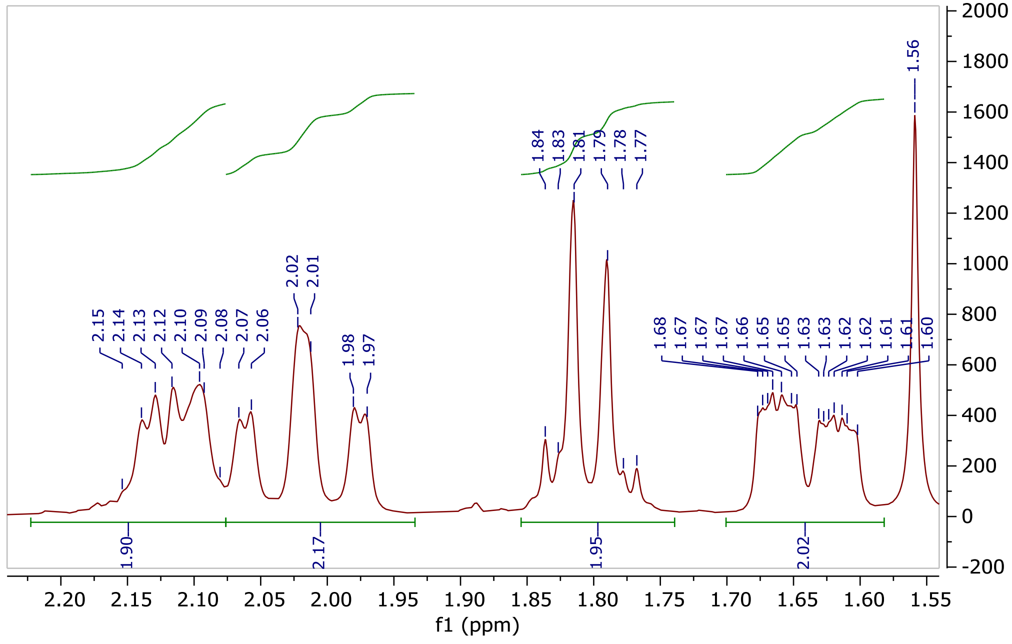

Ethyl 8-phenyl-8-azabicyclo[3.2.1]octane-3-carboxylate (**3aβ**)

^{13}C-NMR (75 MHz, $CDCl_3$)

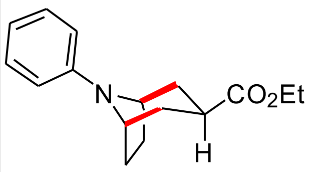

3aβ

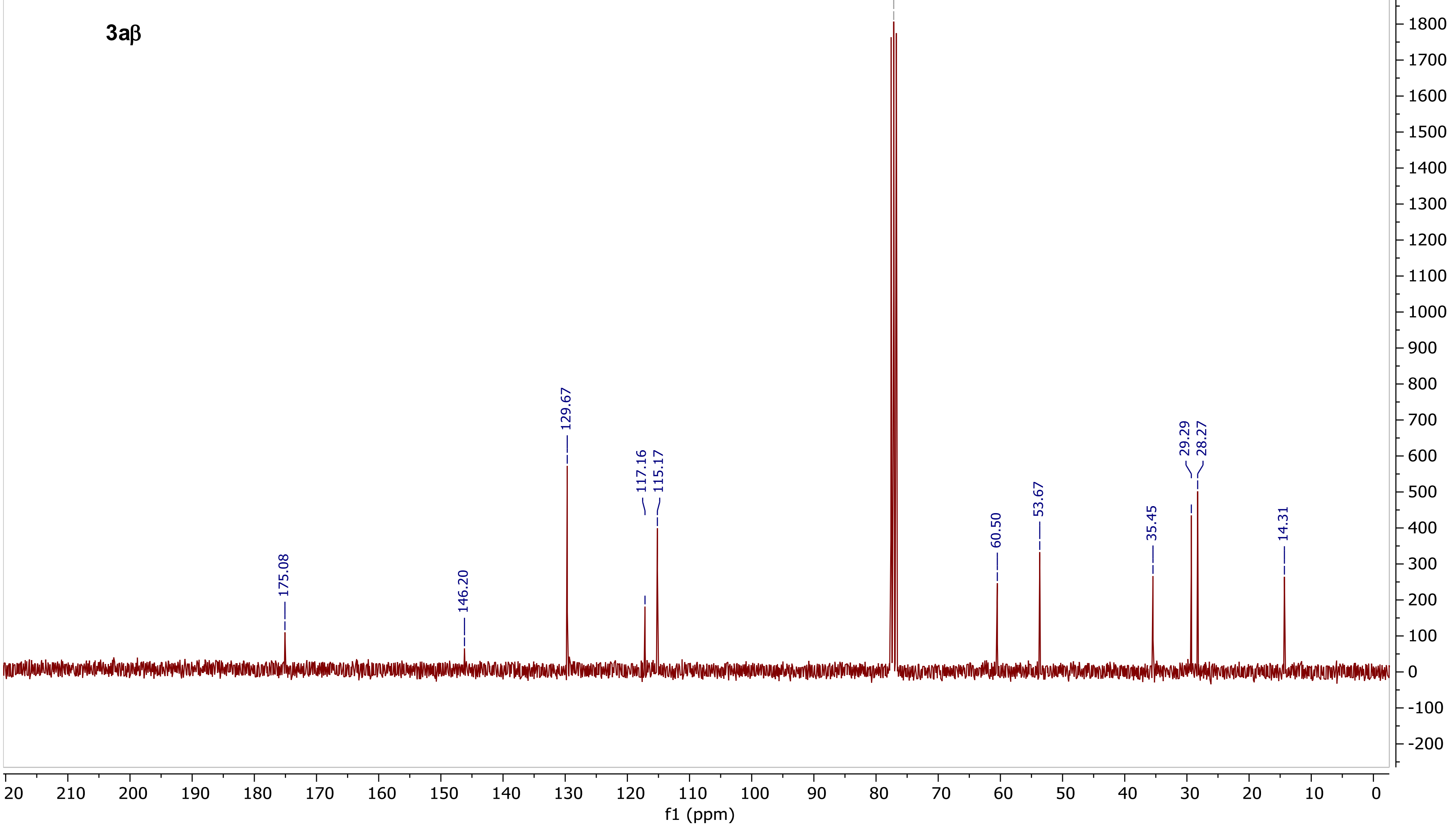

Ethyl 8-phenyl-8-azabicyclo[3.2.1]octane-3-carboxylate (**3aβ**)

^{13}C-NMR (75 MHz, $CDCl_3$)

GA_209381.12.fid
ECO-2-mix F2
Dept135_ns512 CDCl3 /opt service 14

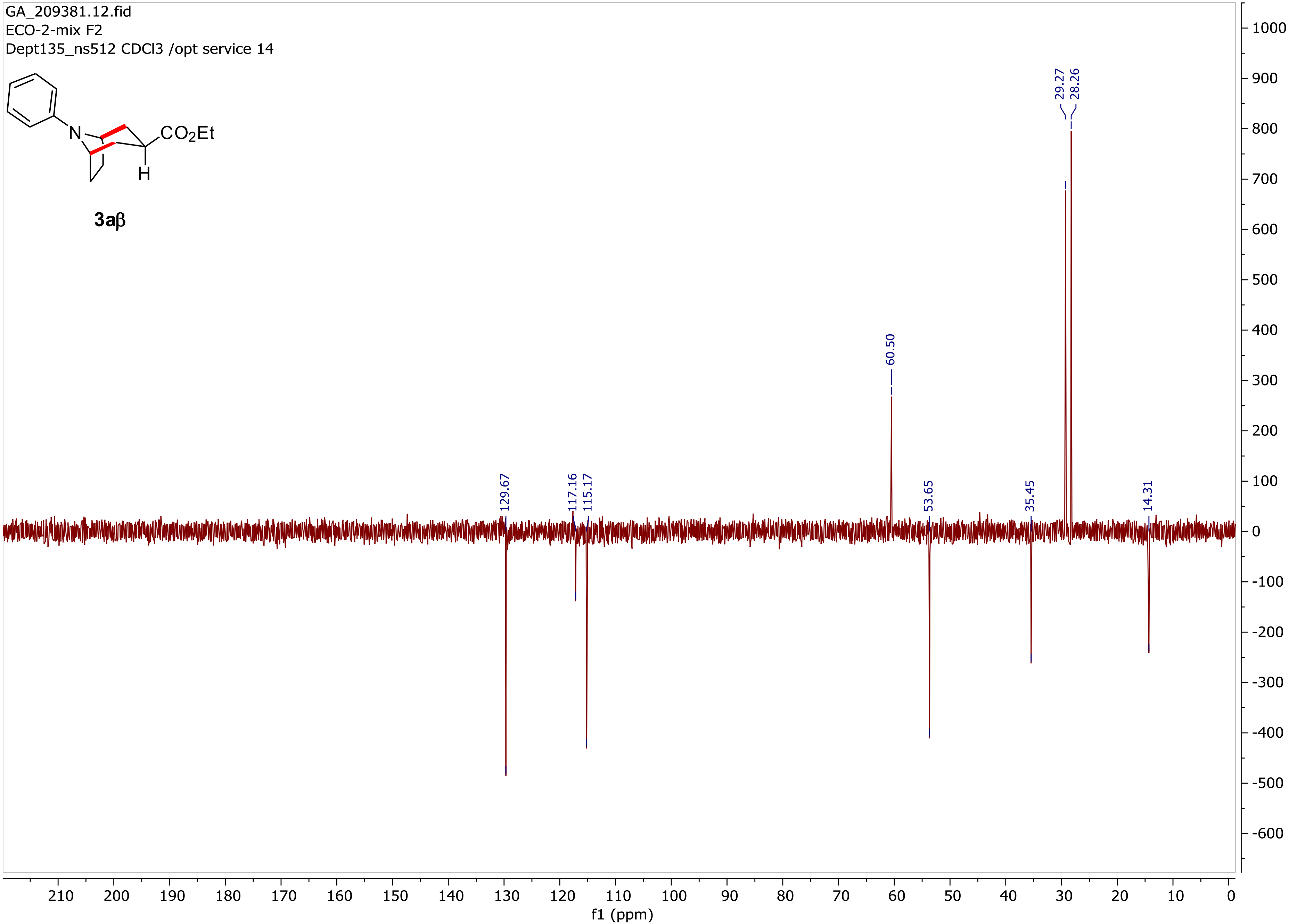

3aβ

Ethyl 8-phenyl-8-azabicyclo[3.2.1]octane-3-carboxylate (**3aβ**)

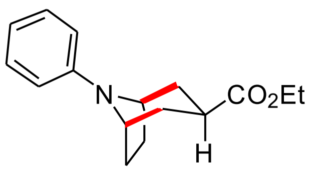

3aβ

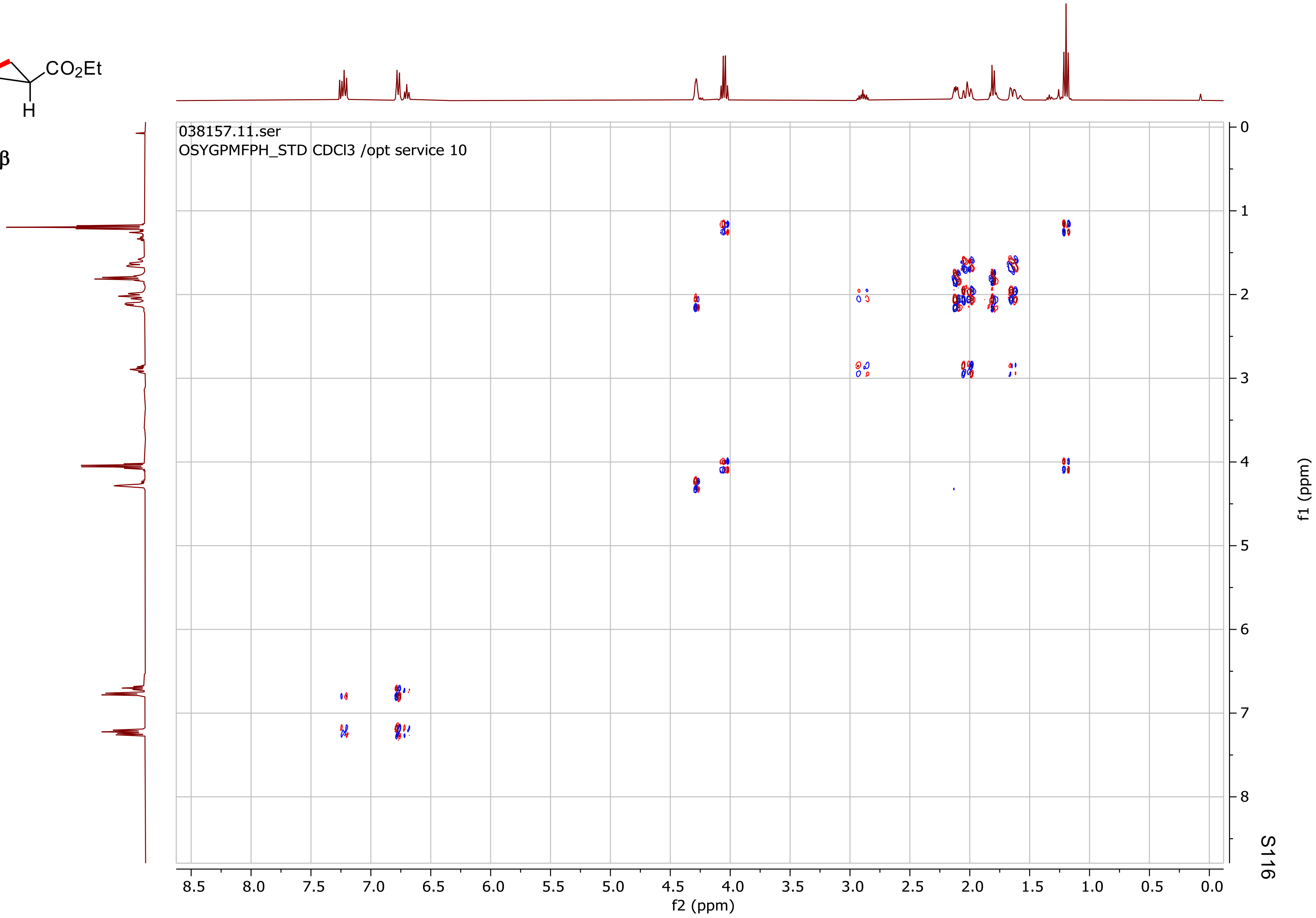

Ethyl 8-phenyl-8-azabicyclo[3.2.1]octane-3-carboxylate (**3aβ**)

^{1}H, ^{13}C-HSQC NMR (400 MHz, $CDCl_3$)

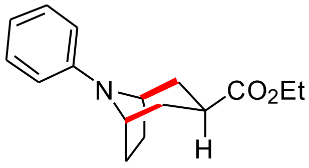

3aβ

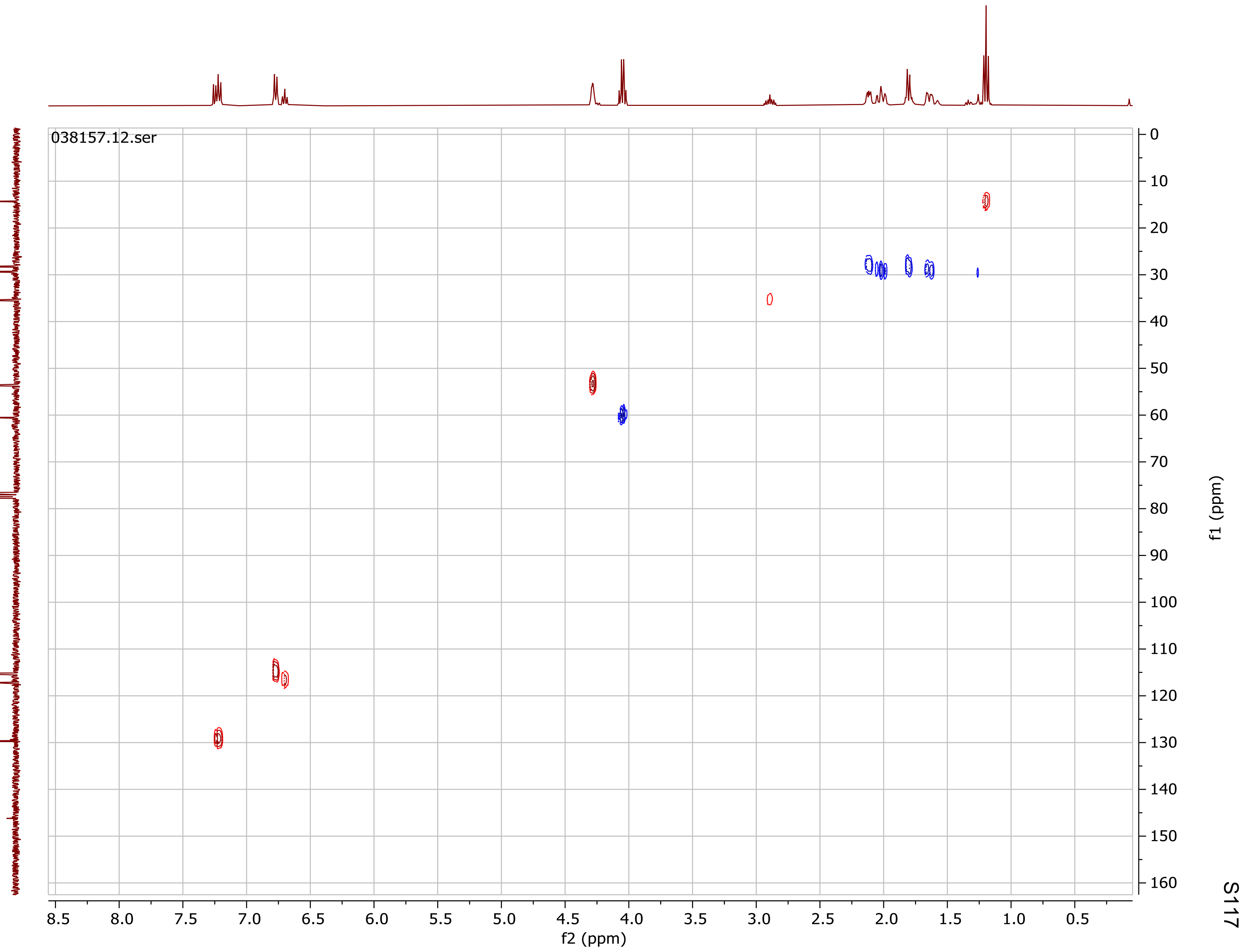

Ethyl 8-phenyl-8-azabicyclo[3.2.1]octane-3-carboxylate (**3aβ**)

^{1}H, ^{13}C-HMBC NMR (300 MHz, $CDCl_3$)

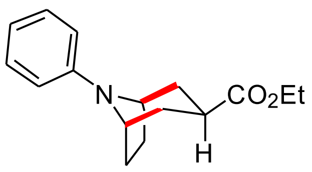

3aβ

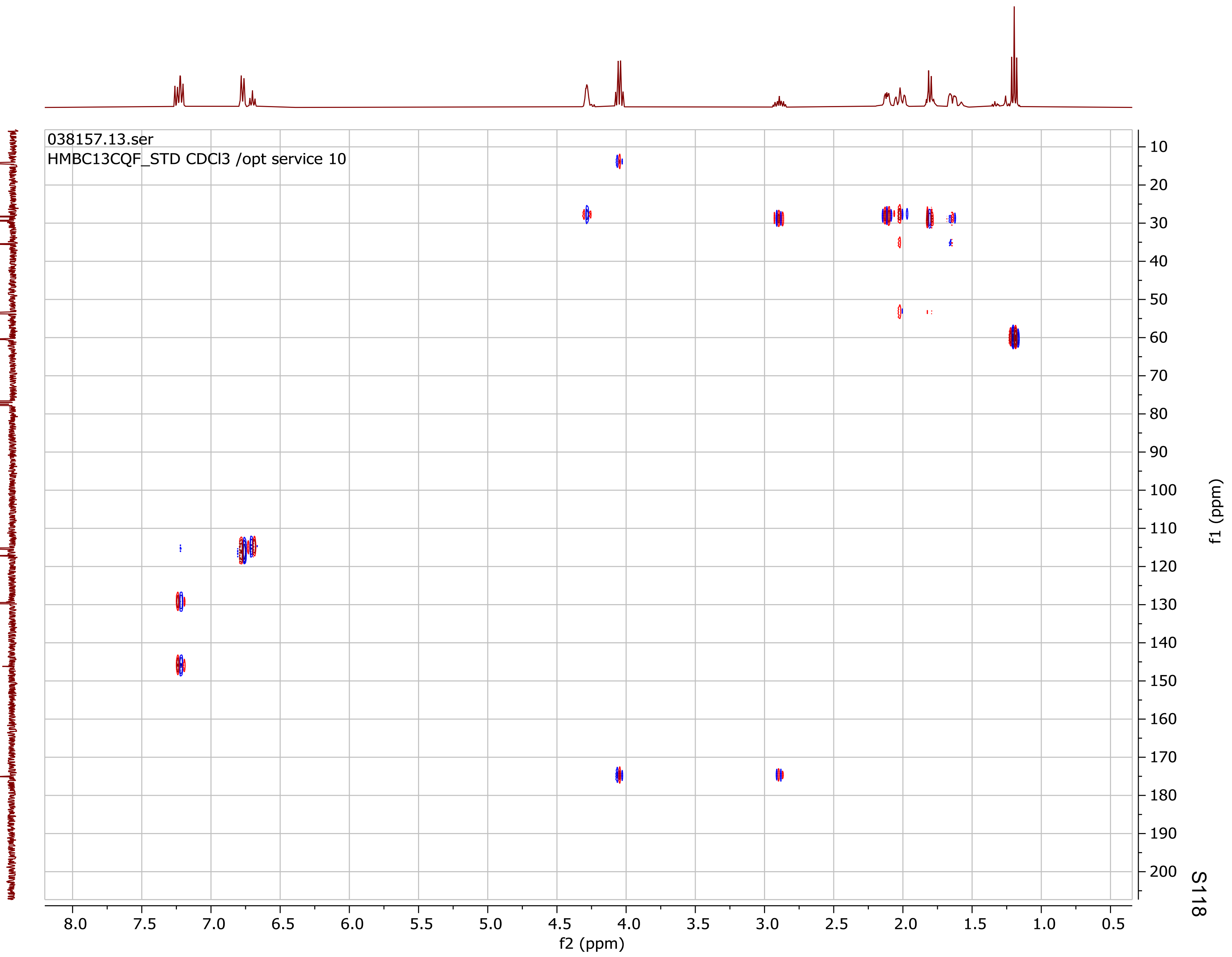

Ethyl 8-phenyl-8-azabicyclo[3.2.1]octane-3-carboxylate (**3aβ**)

FT-IR, ATR-diamond

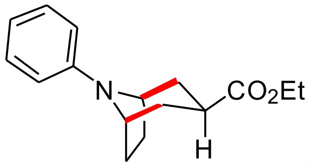

3aβ

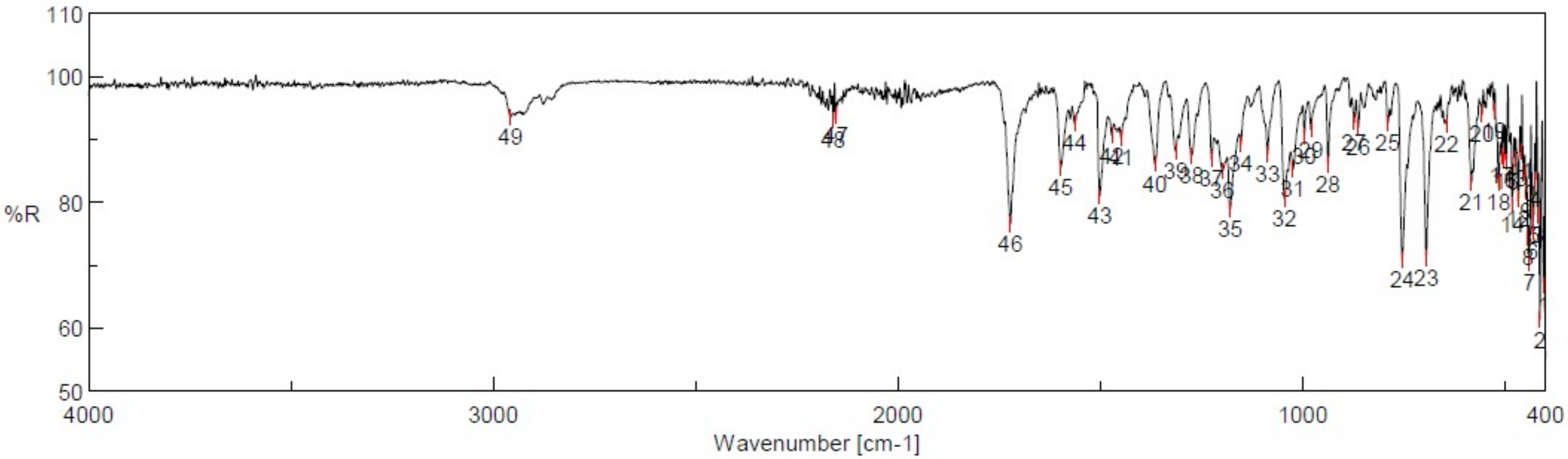

[Result of Peak Picking]

No.	Position	Intensity	No.	Position	Intensity	No.	Position	Intensity
1	404.978	66.8707	2	414.138	61.2278	3	418.959	77.9267
4	424.745	83.5973	5	428.602	78.0276	6	431.977	75.3118
7	440.173	70.4368	8	443.547	74.4544	9	447.886	81.7031
10	454.154	84.5445	11	460.421	88.0002	12	466.689	80.5916
13	475.367	86.937	14	482.117	79.4986	15	496.098	86.5019
16	501.884	86.6583	17	508.633	87.38	18	515.865	83.1016
19	527.436	94.4505	20	556.845	93.9786	21	583.361	83.1183
22	645.072	92.4079	23	694.73	71.1443	24	754.031	70.9355
25	789.707	92.5645	26	862.989	91.8364	27	873.113	92.6845
28	937.235	85.8978	29	979.661	91.5464	30	996.053	90.4401
31	1024.02	85.2205	32	1045.23	80.5661	33	1086.69	87.6988
34	1152.74	89.1915	35	1179.26	78.8215	36	1199.02	84.9645
37	1225.06	86.8776	38	1274.72	87.3383	39	1313.77	88.102
40	1365.35	86.0998	41	1448.76	90.2475	42	1471.9	90.5515
43	1501.31	81.0099	44	1563.5	92.4644	45	1597.73	85.5255
46	1722.12	76.5408	47	2153.62	93.7998	48	2159.88	93.0992
49	2957.79	93.4288						

Ethyl 8-phenyl-8-azabicyclo[3.2.1]octane-3-carboxylate (**3a**) **α/β 7:1 determined by NMR**

^{1}H-NMR (300 MHz, $CDCl_3$)

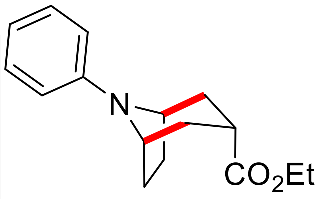

3a **7:1**

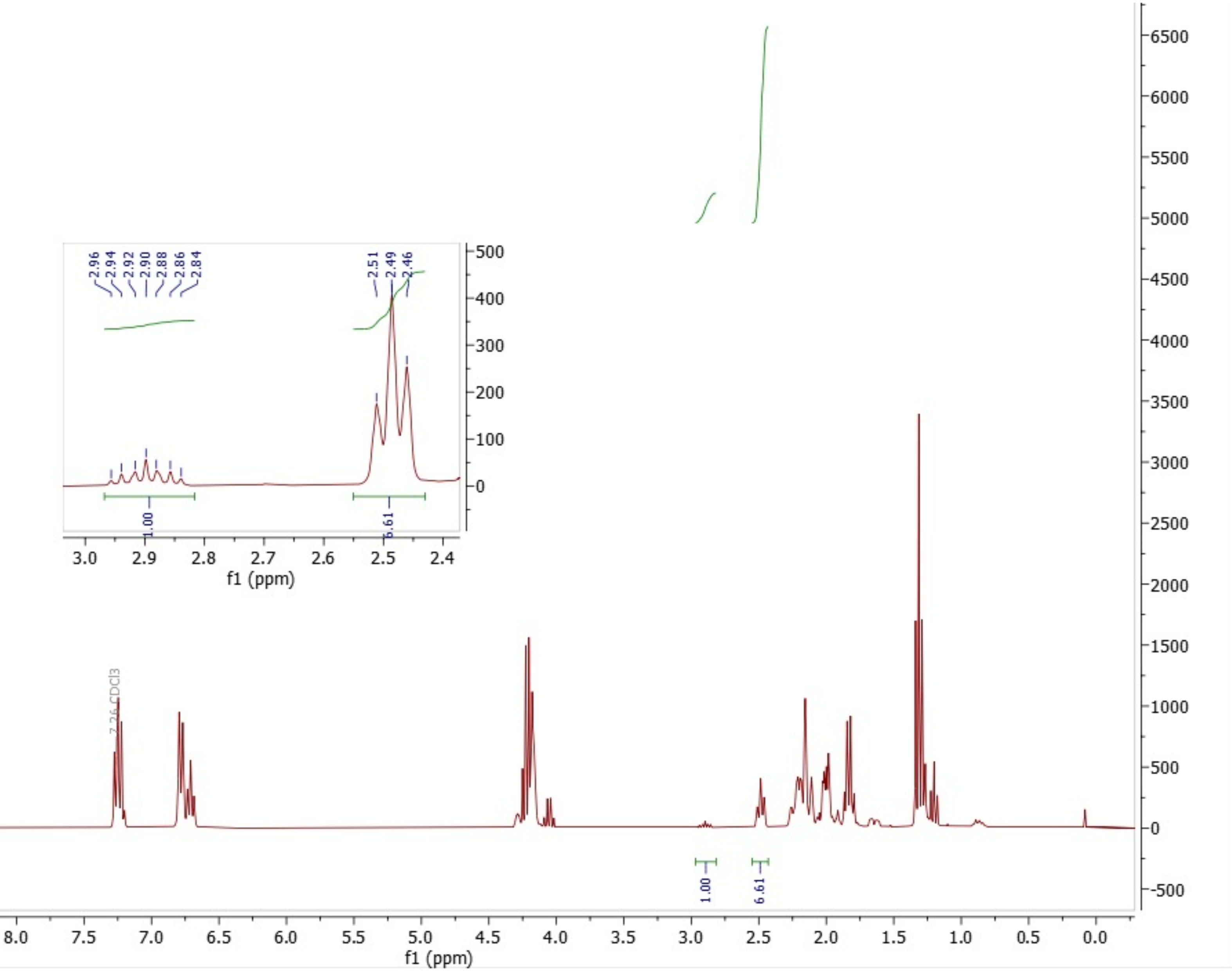

Ethyl 8-[4-(4,4,5,5-tetramethyl-1,3,2-dioxaborolan-2-yl)phenyl]-8-azabicyclo[3.2.1]octane-3-carboxylate (**3b**) **α/β 5:1 from crude NMR**

^{1}H-NMR (300 MHz, $CDCl_3$)

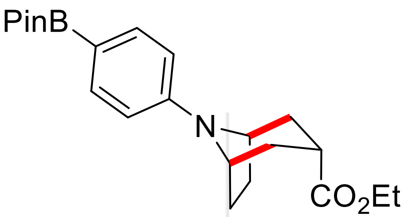

3b, a/ß 5:1

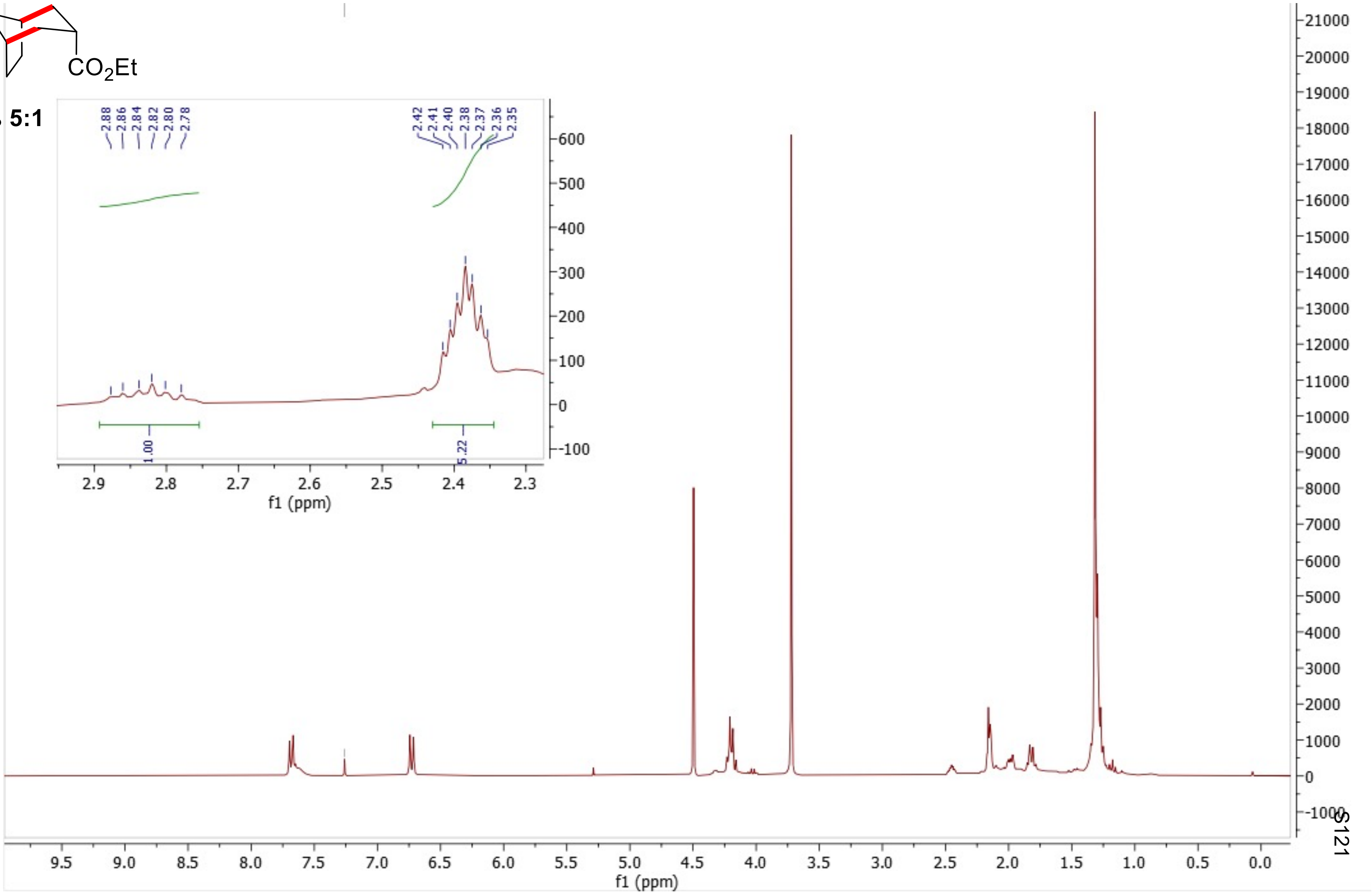

Ethyl 8-[4-(4,4,5,5-tetramethyl-1,3,2-dioxaborolan-2-yl)phenyl]-8-azabicyclo[3.2.1]octane-3-carboxylate **(3bα)**

^{1}H-NMR (400 MHz, $CDCl_3$)

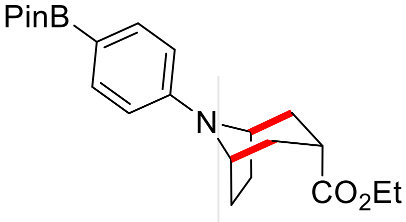

3b

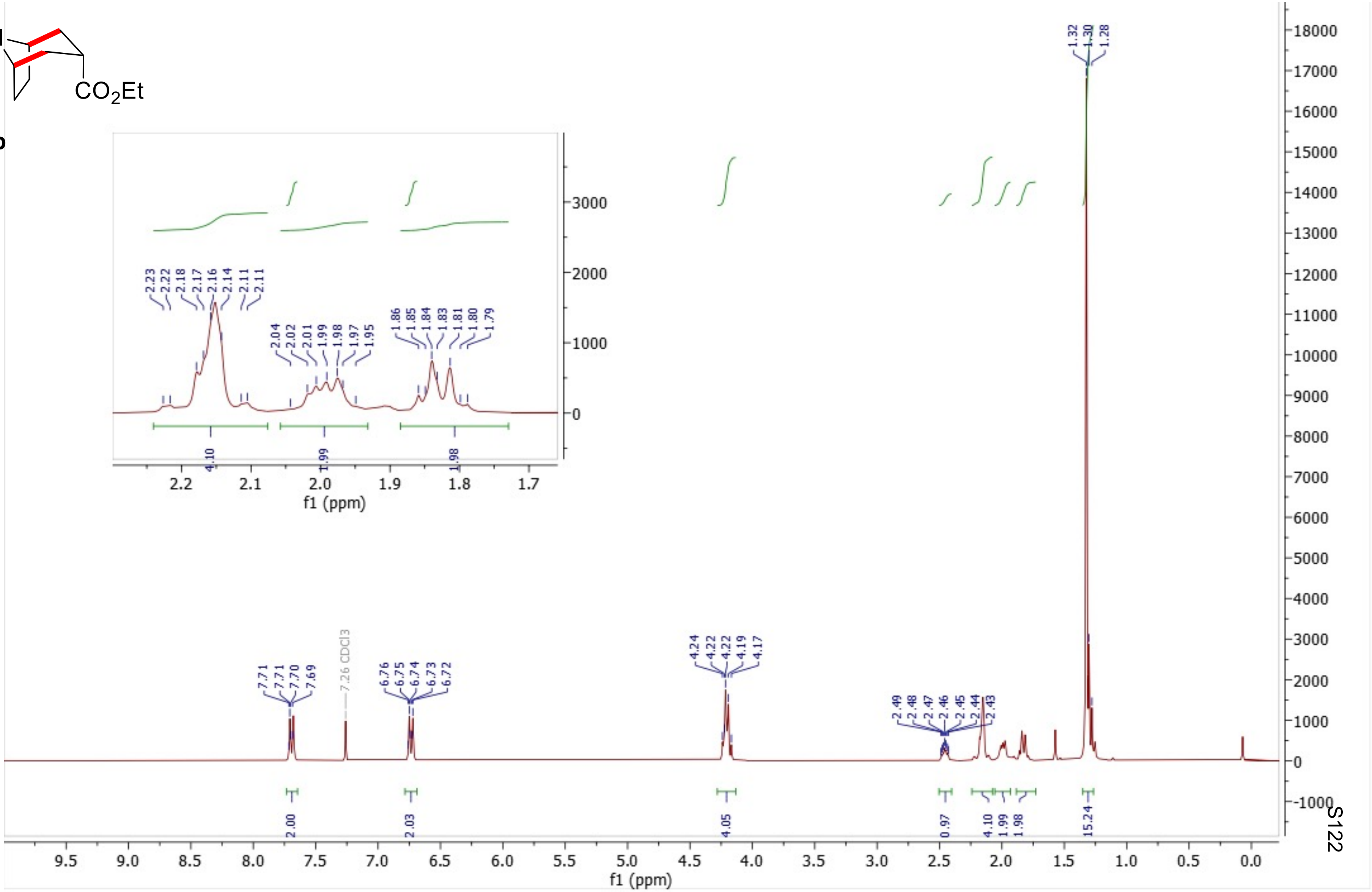

Ethyl 8-[4-(4,4,5,5-tetramethyl-1,3,2-dioxaborolan-2-yl)phenyl]-8-azabicyclo[3.2.1]octane-3-carboxylate **(3bα)**

^{11}B-NMR (96 MHz, $CDCl_3$)

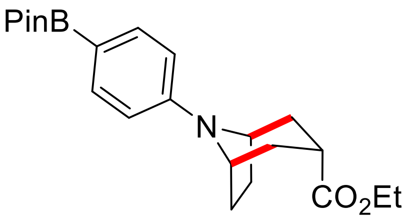

3b

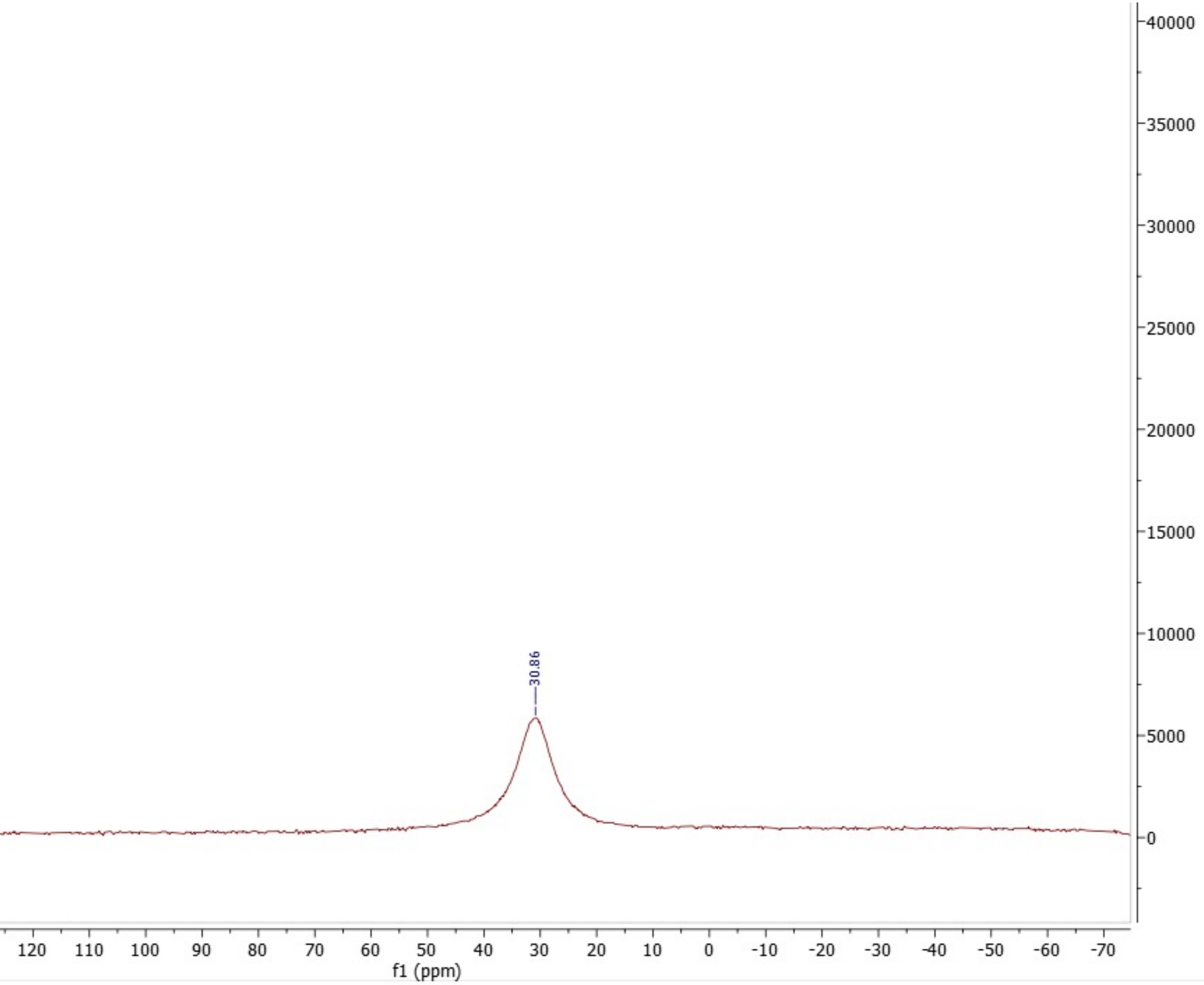

Ethyl 8-[4-(4,4,5,5-tetramethyl-1,3,2-dioxaborolan-2-yl)phenyl]-8-azabicyclo[3.2.1]octane-3-carboxylate **(3bα)**

^{13}C-NMR (101 MHz, $CDCl_3$)

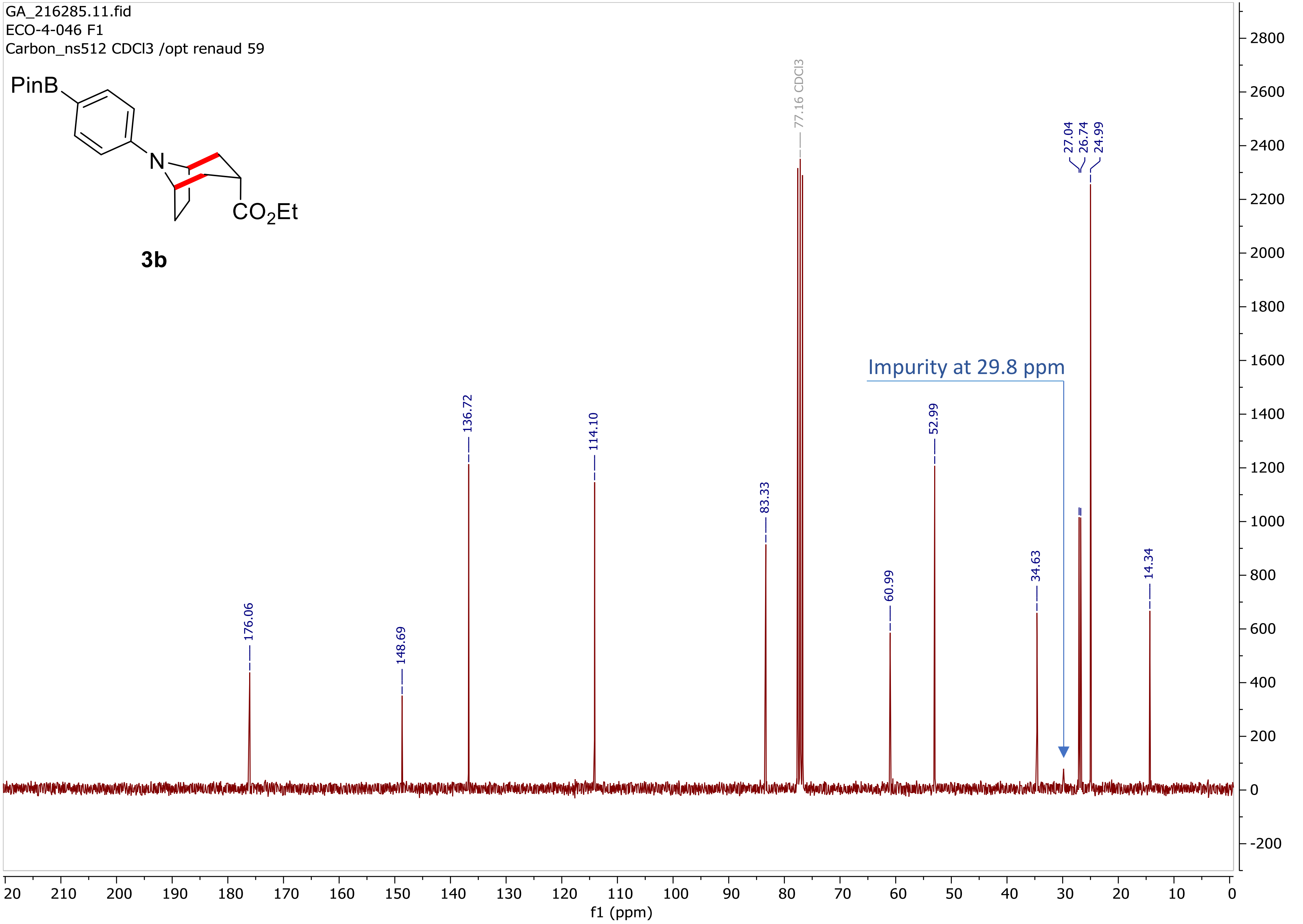

Ethyl 8-[4-(4,4,5,5-tetramethyl-1,3,2-dioxaborolan-2-yl)phenyl]-8-azabicyclo[3.2.1]octane-3-carboxylate **(3bα)**

^{13}C-NMR (101 MHz, $CDCl_3$)

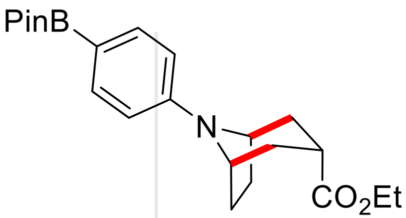

3b

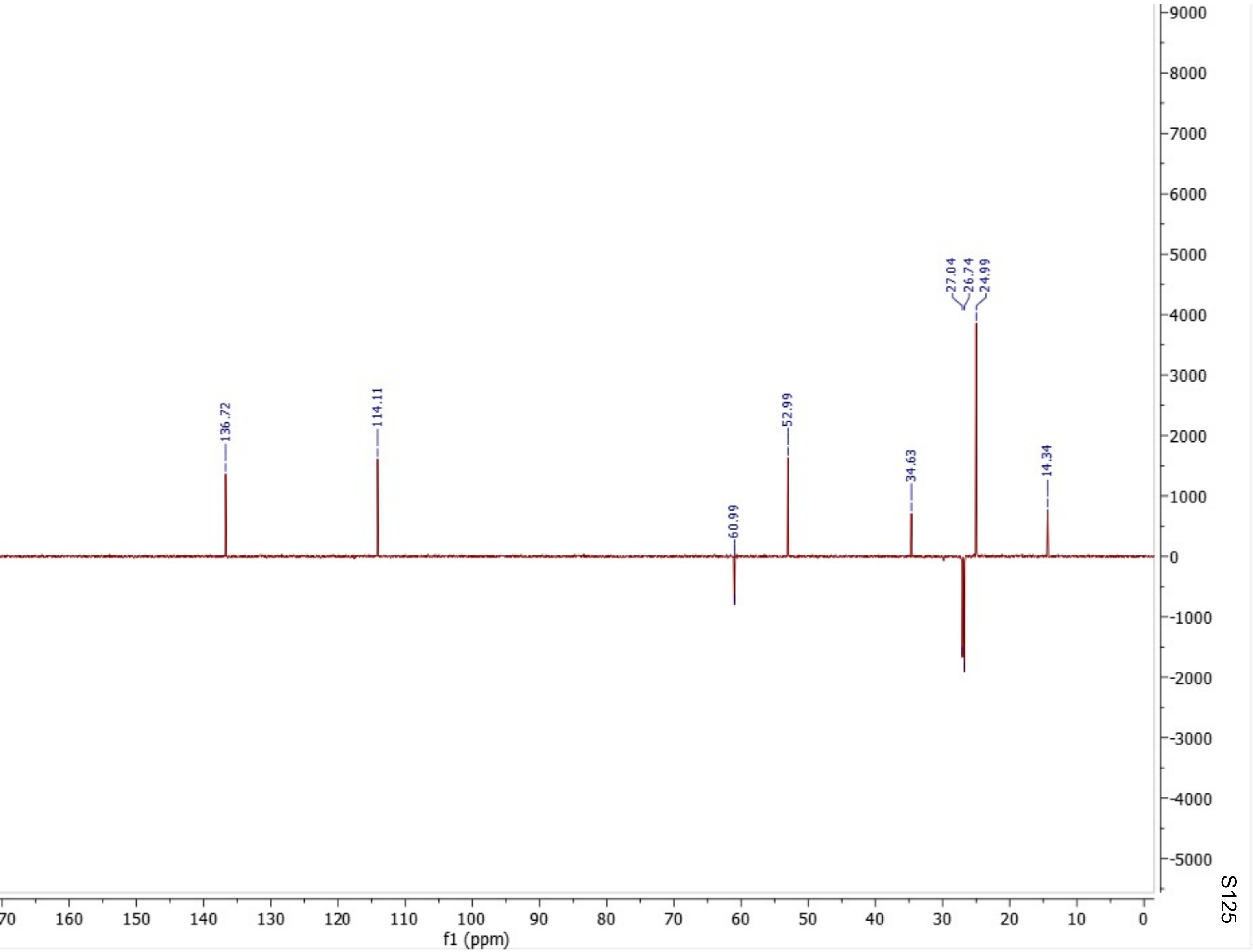

Ethyl 8-[4-(4,4,5,5-tetramethyl-1,3,2-dioxaborolan-2-yl)phenyl]-8-azabicyclo[3.2.1]octane-3-carboxylate **(3bα)**

^{1}H, ^{1}H-COSY NMR (400 MHz, $CDCl_3$)

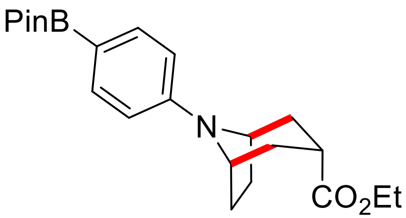

3b

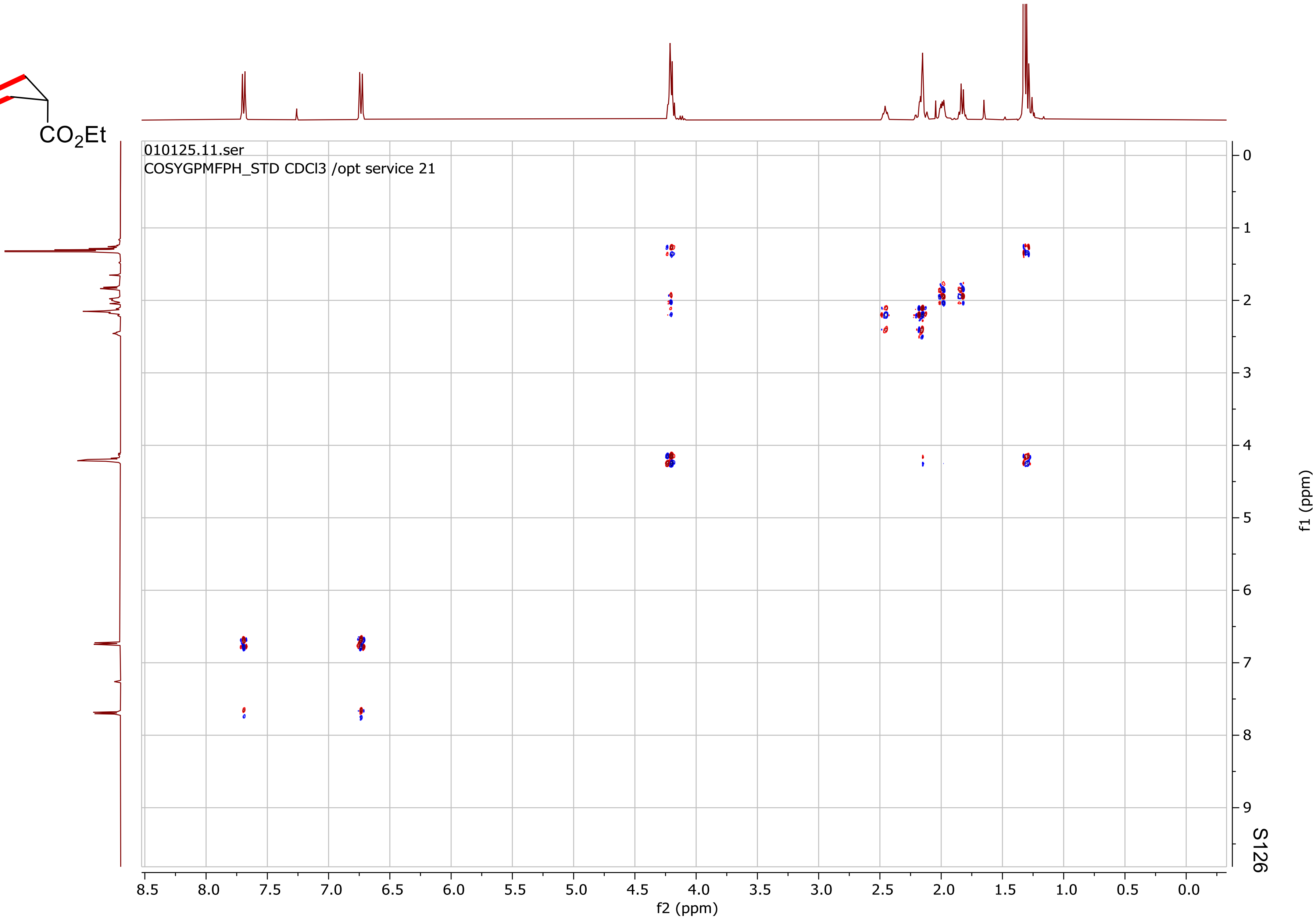

Ethyl 8-[4-(4,4,5,5-tetramethyl-1,3,2-dioxaborolan-2-yl)phenyl]-8-azabicyclo[3.2.1]octane-3-carboxylate **(3bα)**

^1H, ^{13}C-HSQC NMR (400 MHz, $CDCl_3$)

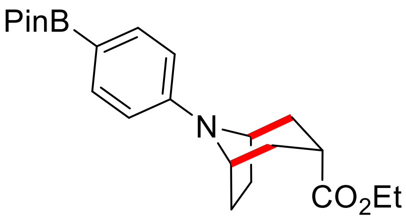

3b

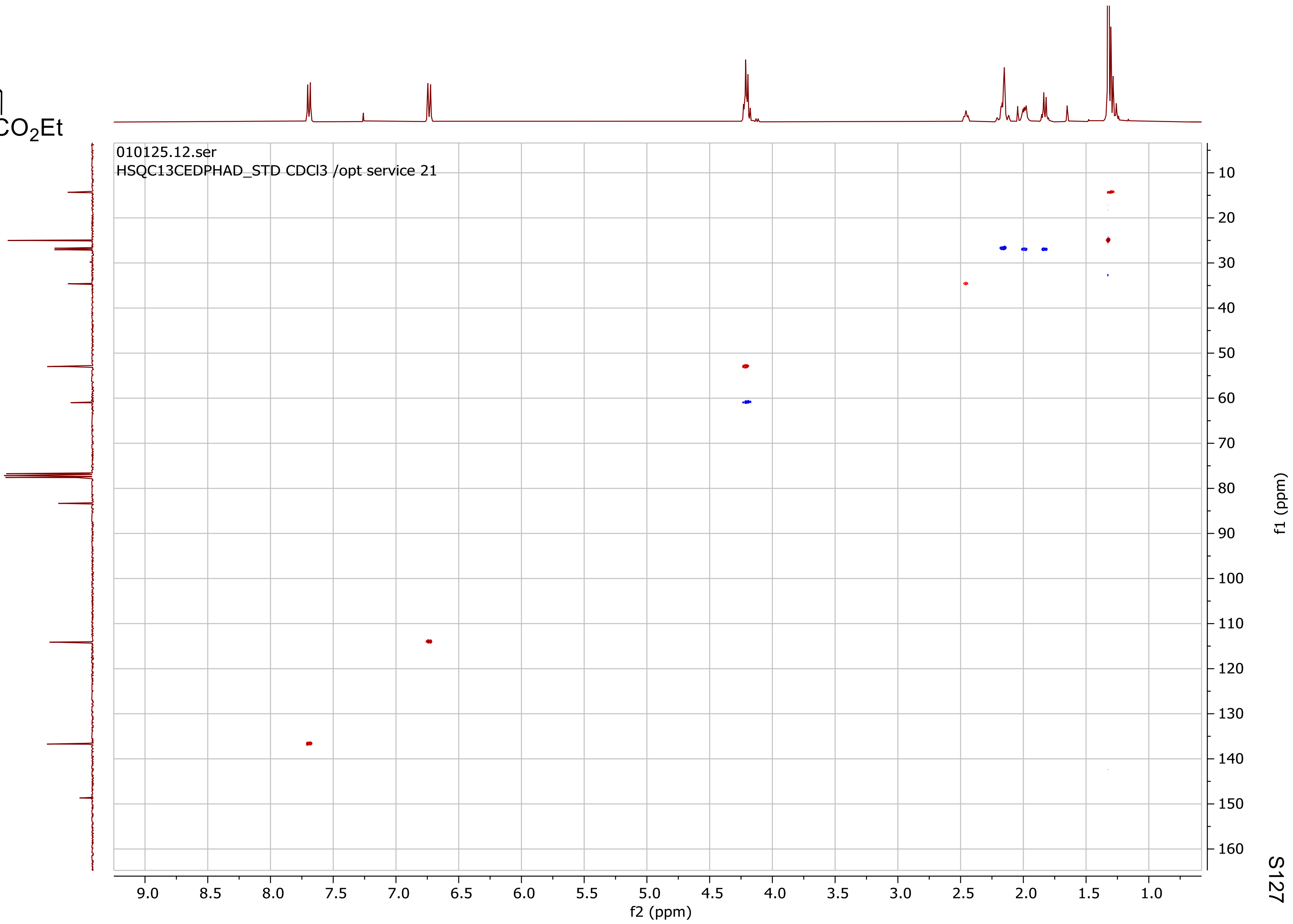

Ethyl 8-[4-(4,4,5,5-tetramethyl-1,3,2-dioxaborolan-2-yl)phenyl]-8-azabicyclo[3.2.1]octane-3-carboxylate **(3bα)**

^{1}H, ^{13}C-HMBC NMR (400 MHz, $CDCl_3$)

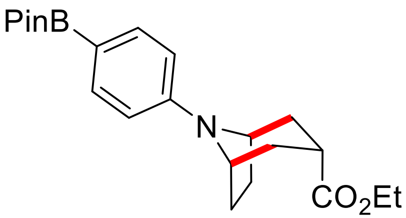

3b

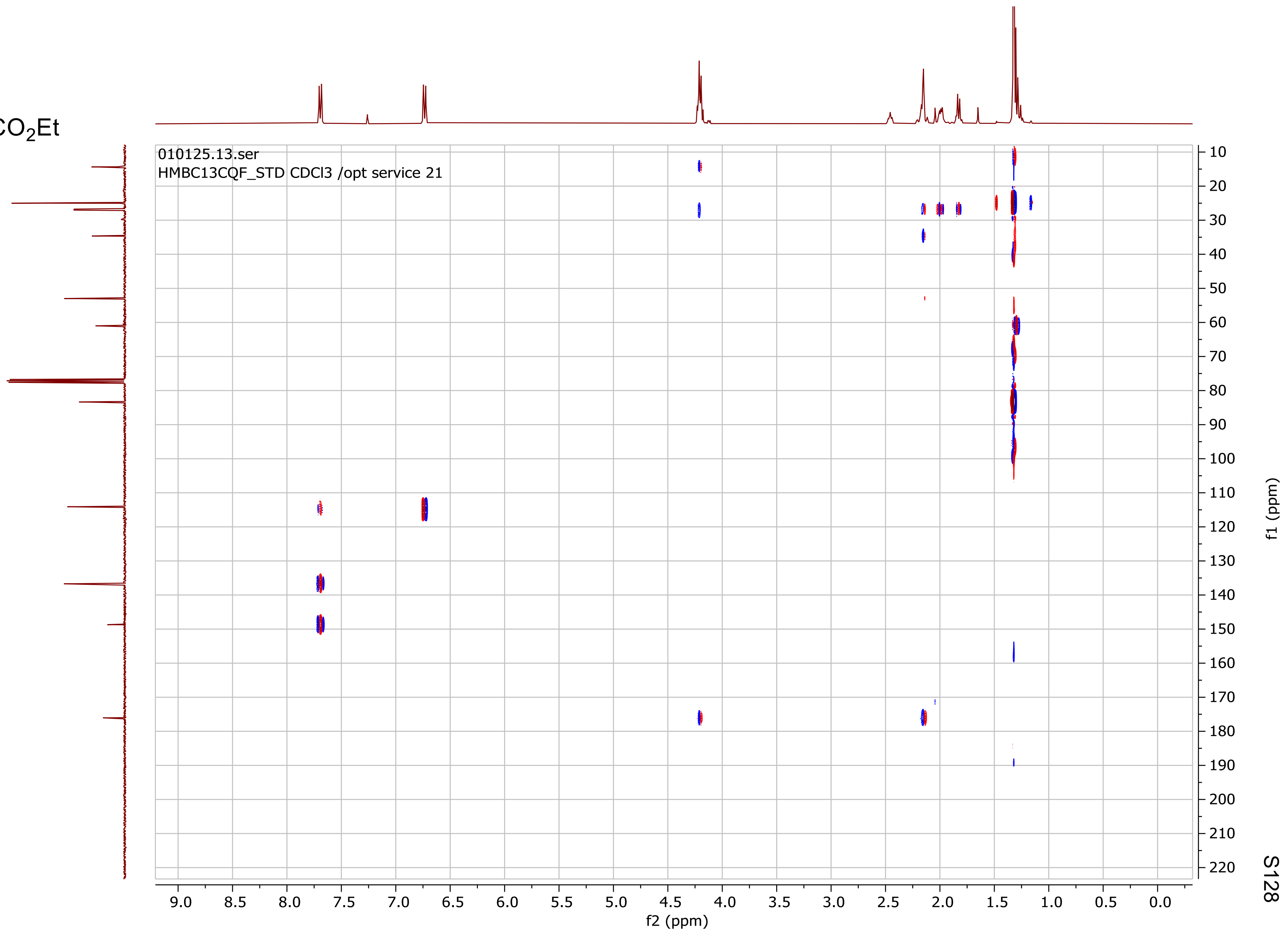

Ethyl 8-[4-(4,4,5,5-tetramethyl-1,3,2-dioxaborolan-2-yl)phenyl]-8-azabicyclo[3.2.1]octane-3-carboxylate (**3bα**)

FT-IR, ATR-diamond

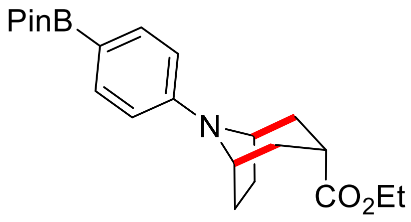

3b

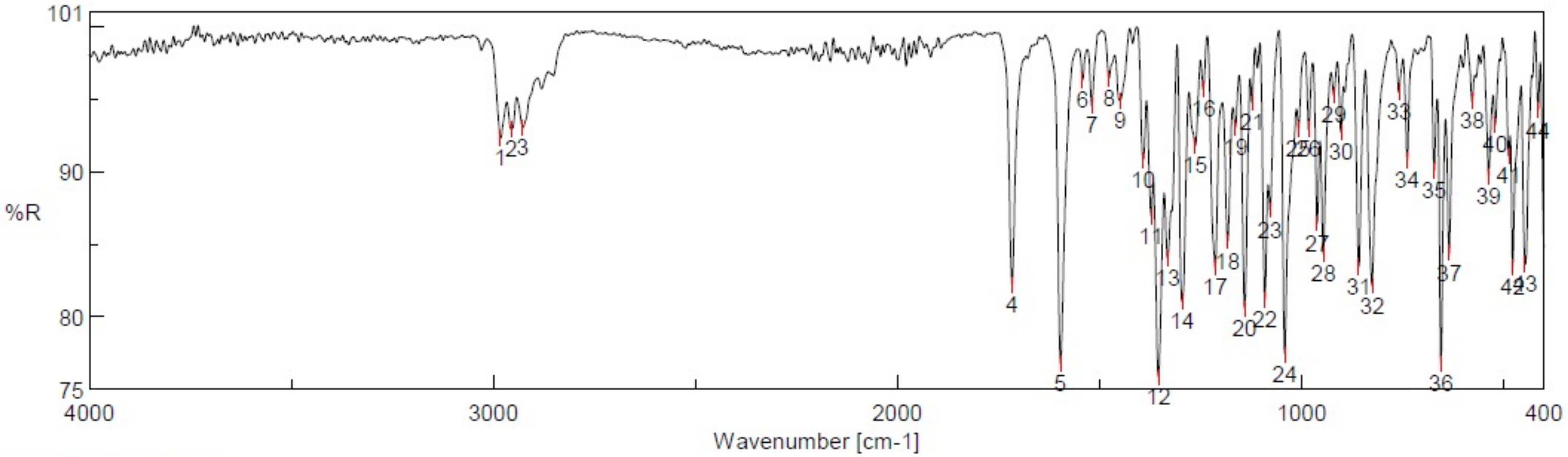

[Result of Peak Picking]

No.	Position	Intensity	No.	Position	Intensity	No.	Position	Intensity
1	2982.37	92.3167	2	2956.34	92.9491	3	2927.41	93.056
4	1716.34	82.1849	5	1596.77	76.7841	6	1541.81	96.3397
7	1518.67	94.5339	8	1476.24	96.4431	9	1449.24	94.9083
10	1391.39	90.8313	11	1372.1	86.9376	12	1353.78	75.8424
13	1331.61	84.028	14	1294.97	81.0175	15	1263.15	91.7596
16	1242.9	95.6732	17	1213.97	83.3555	18	1183.11	85.1996
19	1163.83	93.077	20	1140.69	80.5247	21	1122.37	94.7455
22	1090.55	81.1765	23	1078.01	87.3464	24	1041.37	77.4029
25	1007.62	92.9518	26	982.554	92.927	27	961.341	86.4149
28	945.913	84.3105	29	919.879	95.2561	30	902.523	92.7367
31	858.168	83.4161	32	825.384	82.1277	33	758.852	95.484
34	738.603	90.7875	35	672.071	90.0226	36	654.715	76.7628
37	634.466	84.3839	38	577.576	94.8473	39	537.078	89.6421
40	520.686	93.2093	41	486.938	91.067	42	477.296	83.4267
43	446.44	83.6053	44	414.62	94.2275			

Ethyl 8-[4-(4,4,5,5-tetramethyl-1,3,2-dioxaborolan-2-yl)phenyl]-8-azabicyclo[3.2.1]octane-3-carboxylate (**3b**) **α/β 1:1.7**

^{1}H-NMR (300 MHz, $CDCl_3$)

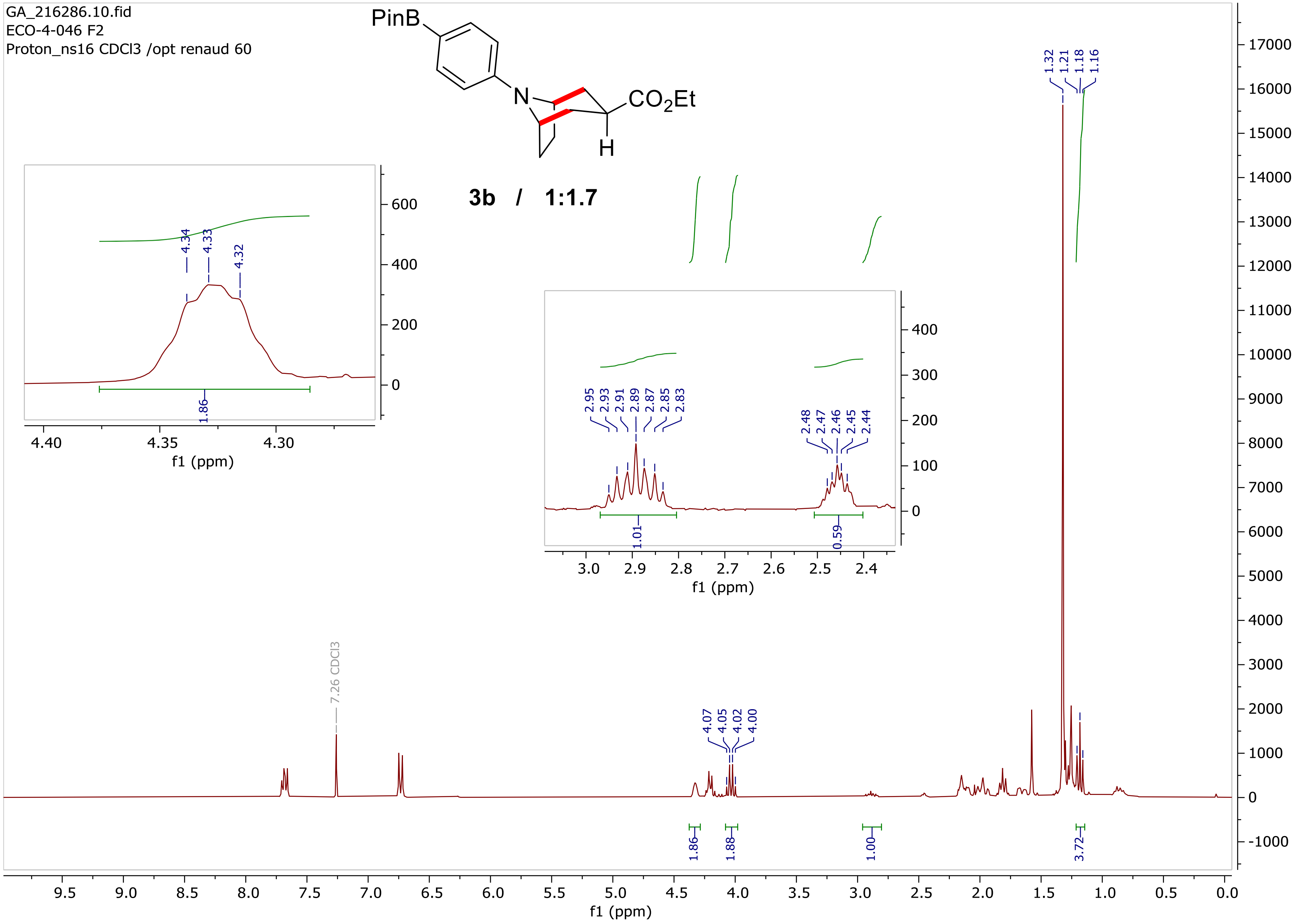

Ethyl 8-[4-(4,4,5,5-tetramethyl-1,3,2-dioxaborolan-2-yl)phenyl]-8-azabicyclo[3.2.1]octane-3-carboxylate (**3b**) **α/β 1:1.7**

^{11}B-NMR (96 MHz, $CDCl_3$)

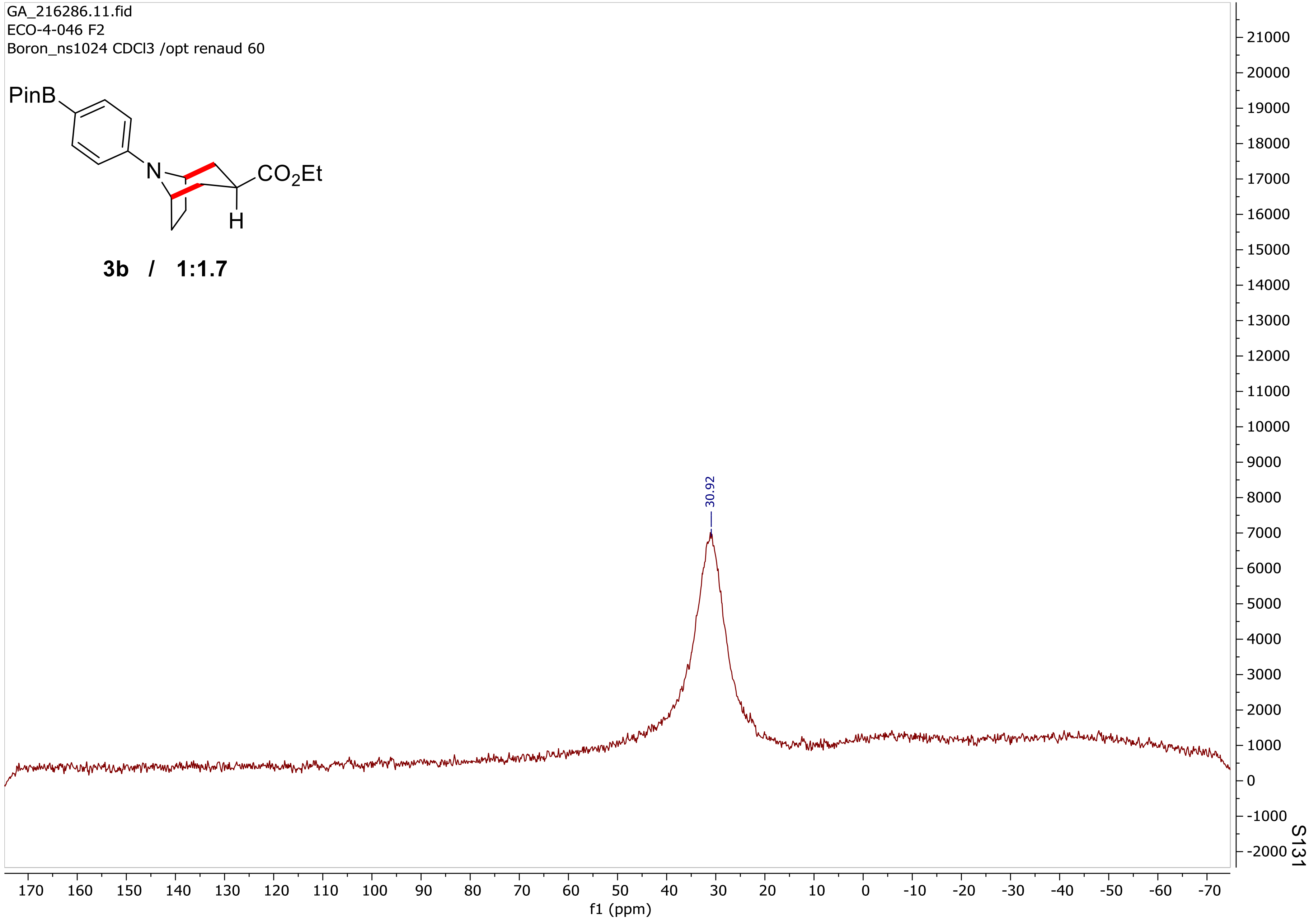

Ethyl 8-[4-(4,4,5,5-tetramethyl-1,3,2-dioxaborolan-2-yl)phenyl]-8-azabicyclo[3.2.1]octane-3-carboxylate (**3b**) **α/β 1:1.7**

^{13}C-NMR (101 MHz, $CDCl_3$)

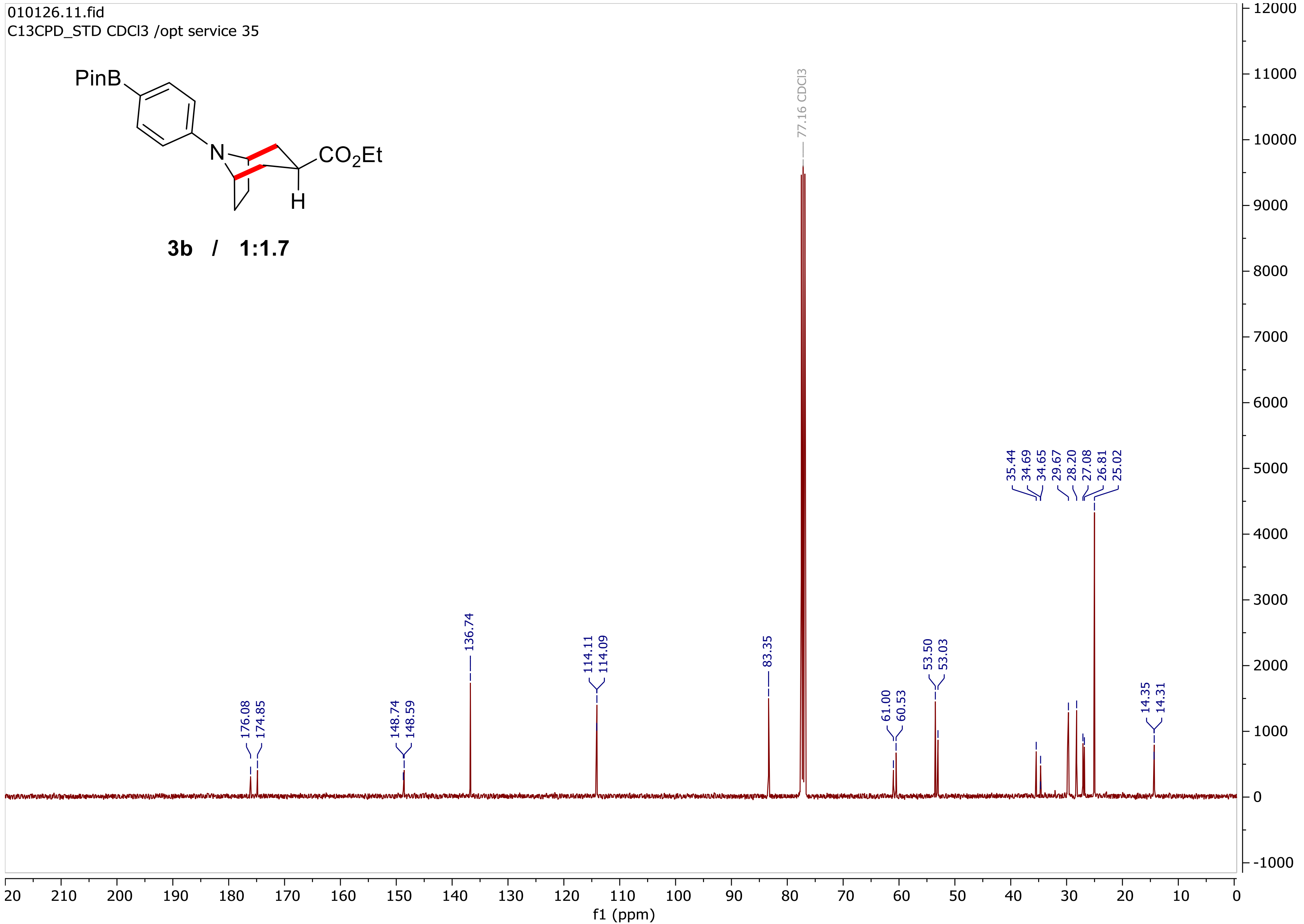

Ethyl 8-[4-(4,4,5,5-tetramethyl-1,3,2-dioxaborolan-2-yl)phenyl]-8-azabicyclo[3.2.1]octane-3-carboxylate (**3b**) **α/β 1:1.7**

^{13}C-NMR (101 MHz, $CDCl_3$)

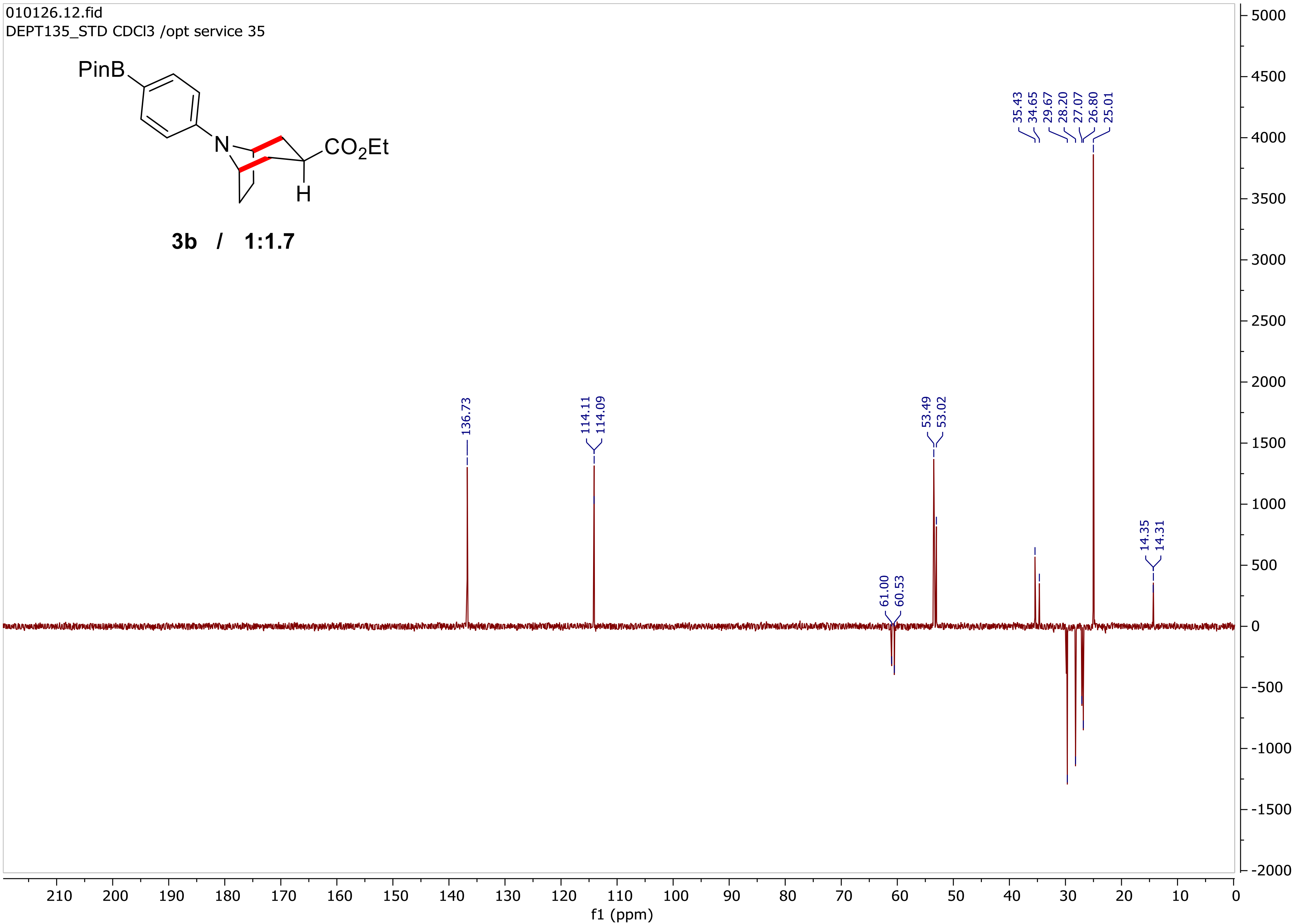

8-(4-methoxycarbonylphenyl)-8-azabicyclo[3.2.1]octane-3-carboxylate (**3c**) **α/β 9:1**

^{1}H-NMR (300 MHz, $CDCl_3$)

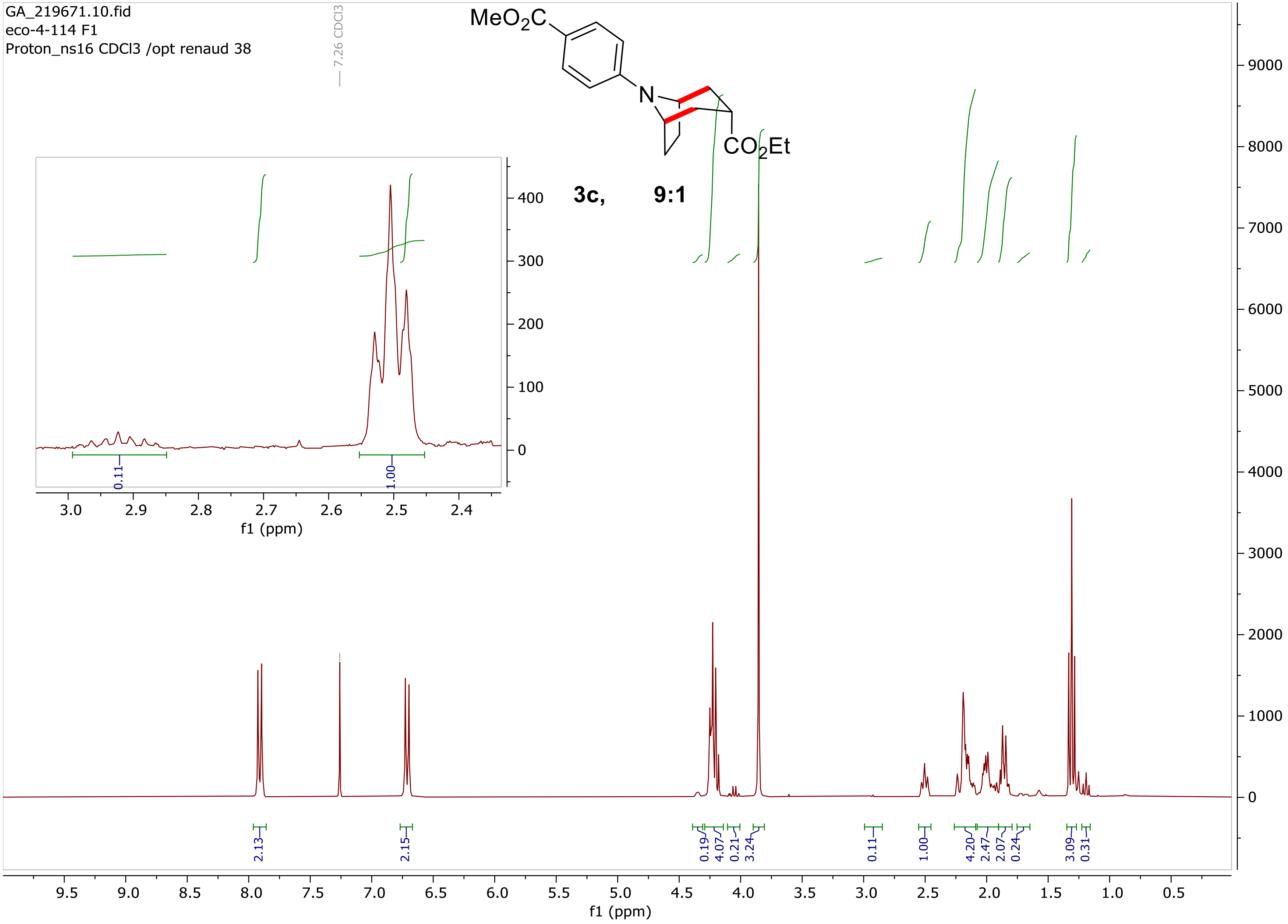

8-(4-methoxycarbonylphenyl)-8-azabicyclo[3.2.1]octane-3-carboxylate (**3cα**)

^{1}H-NMR (400 MHz, $CDCl_3$)

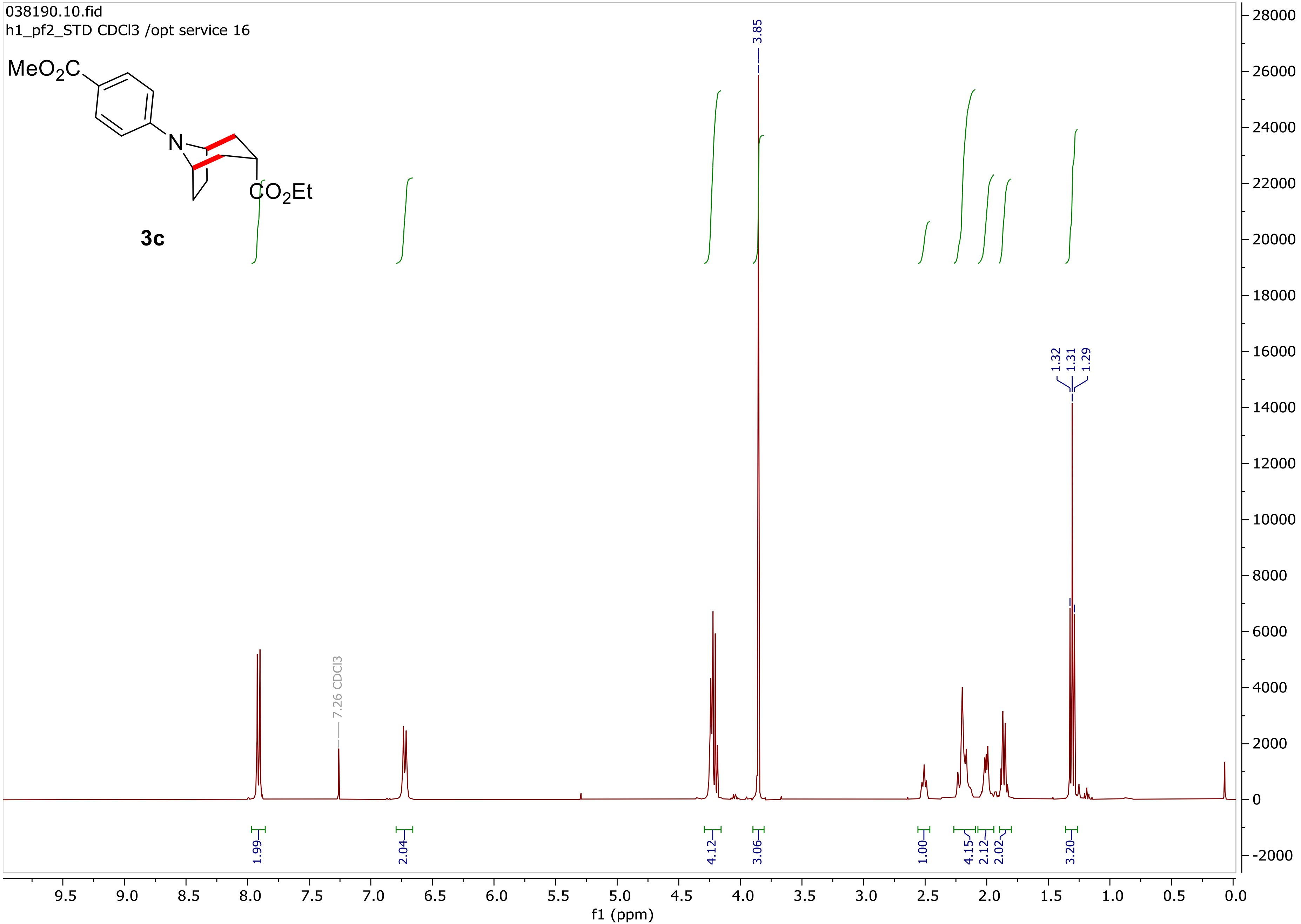

8-(4-methoxycarbonylphenyl)-8-azabicyclo[3.2.1]octane-3-carboxylate (**3cα**)

^{1}H-NMR (400 MHz, $CDCl_3$)

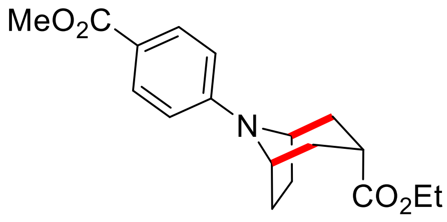

3c

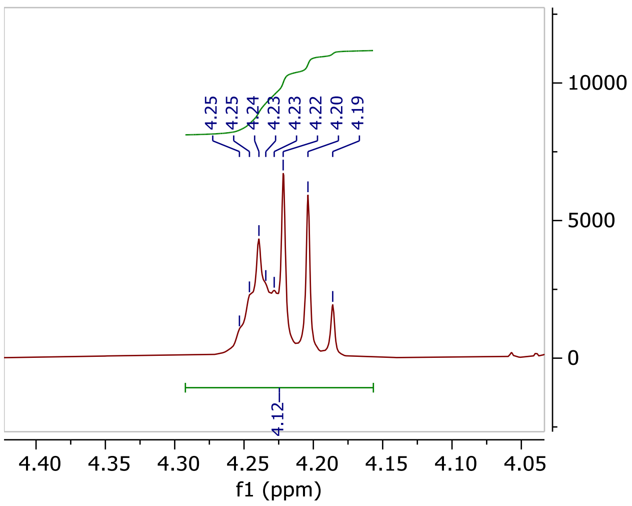

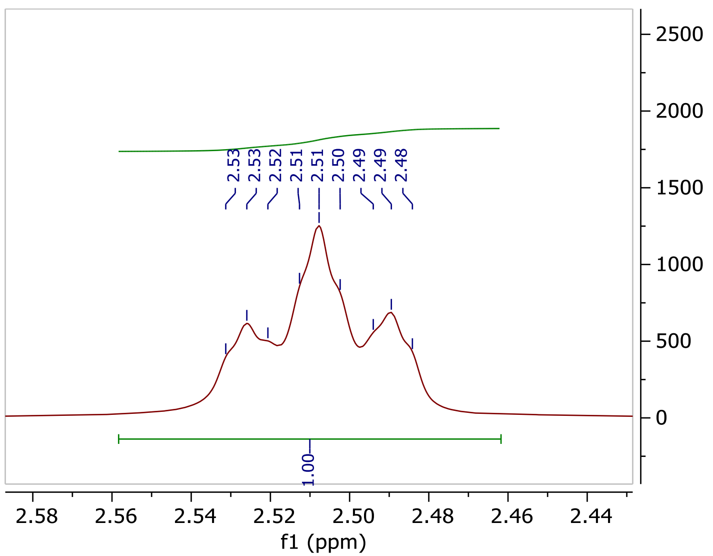

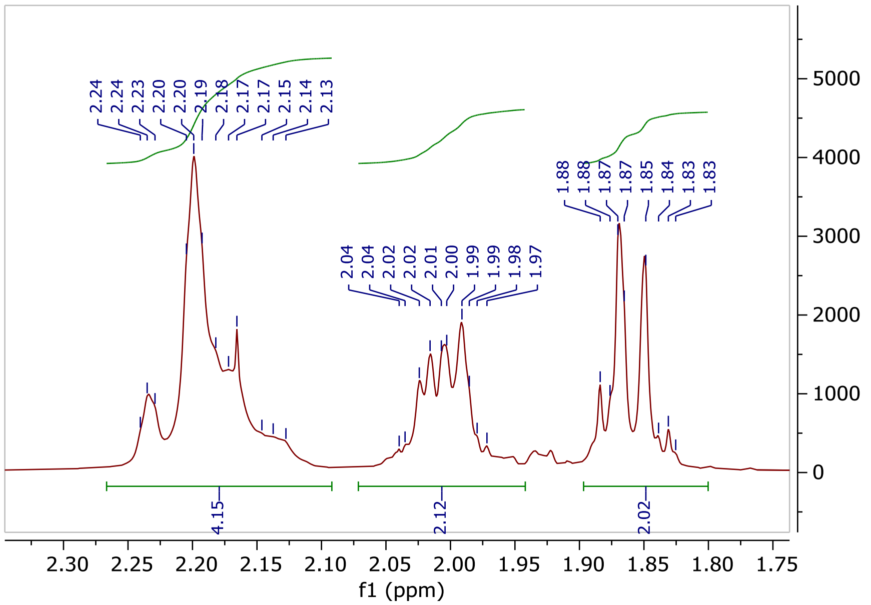

8-(4-methoxycarbonylphenyl)-8-azabicyclo[3.2.1]octane-3-carboxylate (**3cα**)

^{13}C-NMR (101 MHz, $CDCl_3$)

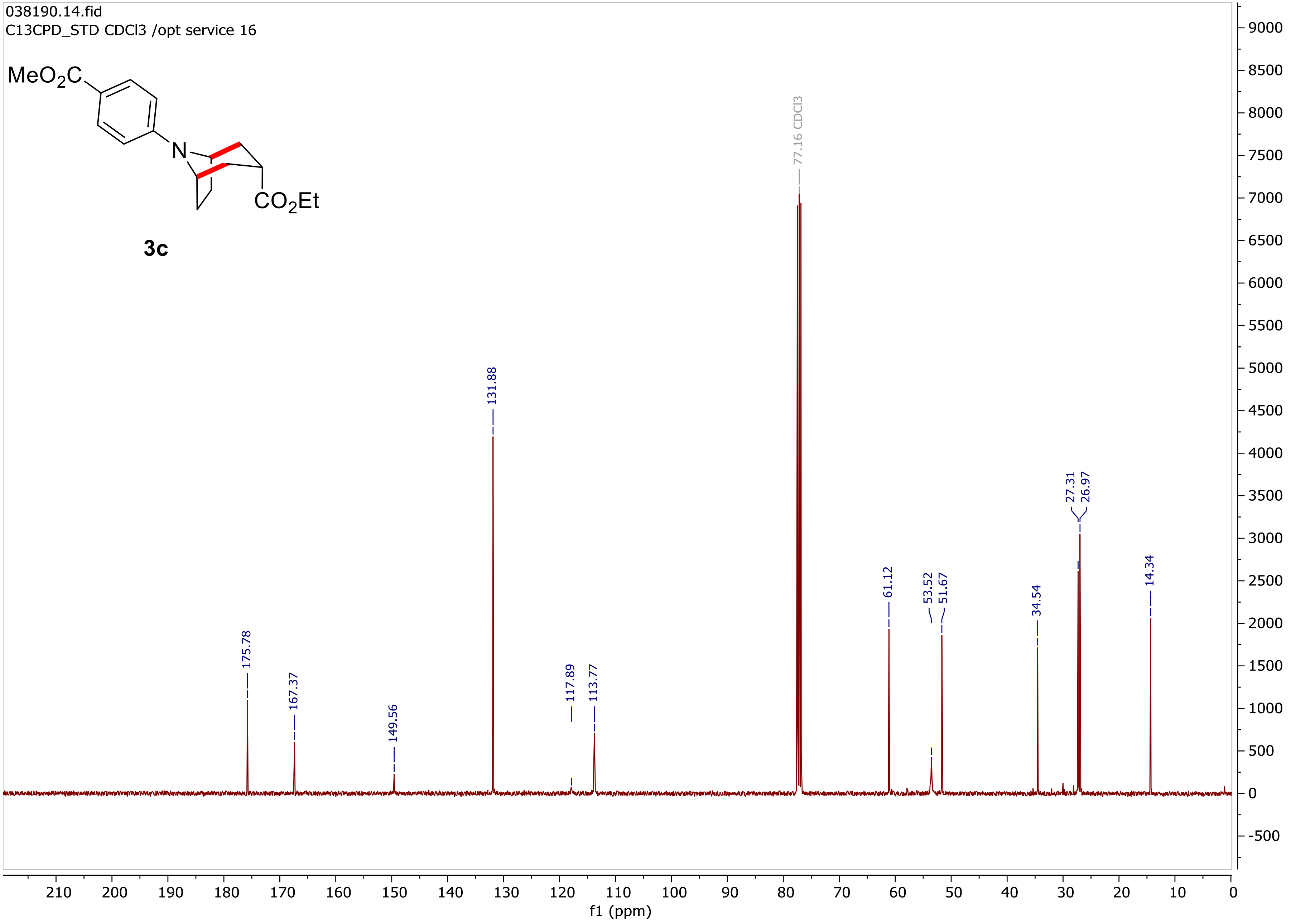

8-(4-methoxycarbonylphenyl)-8-azabicyclo[3.2.1]octane-3-carboxylate (**3cα**)

^{13}C-NMR (75 MHz, $CDCl_3$)

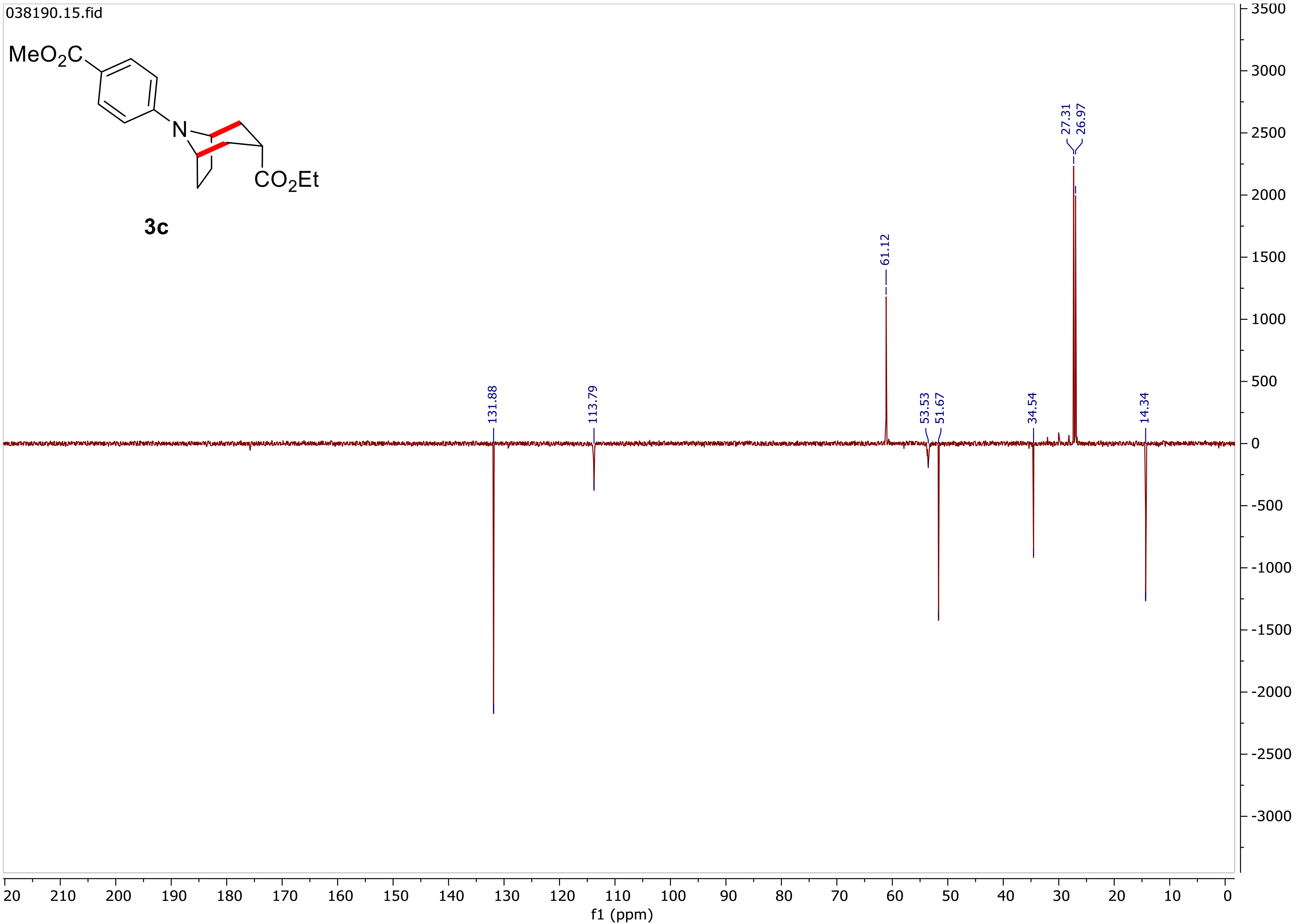

8-(4-methoxycarbonylphenyl)-8-azabicyclo[3.2.1]octane-3-carboxylate (**3cα**)

^{1}H, ^{1}H-COSY NMR (300 MHz, $CDCl_3$)

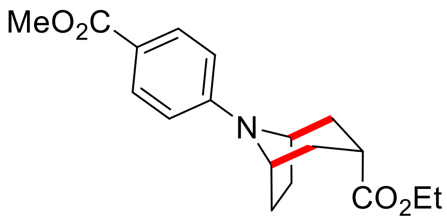

3c

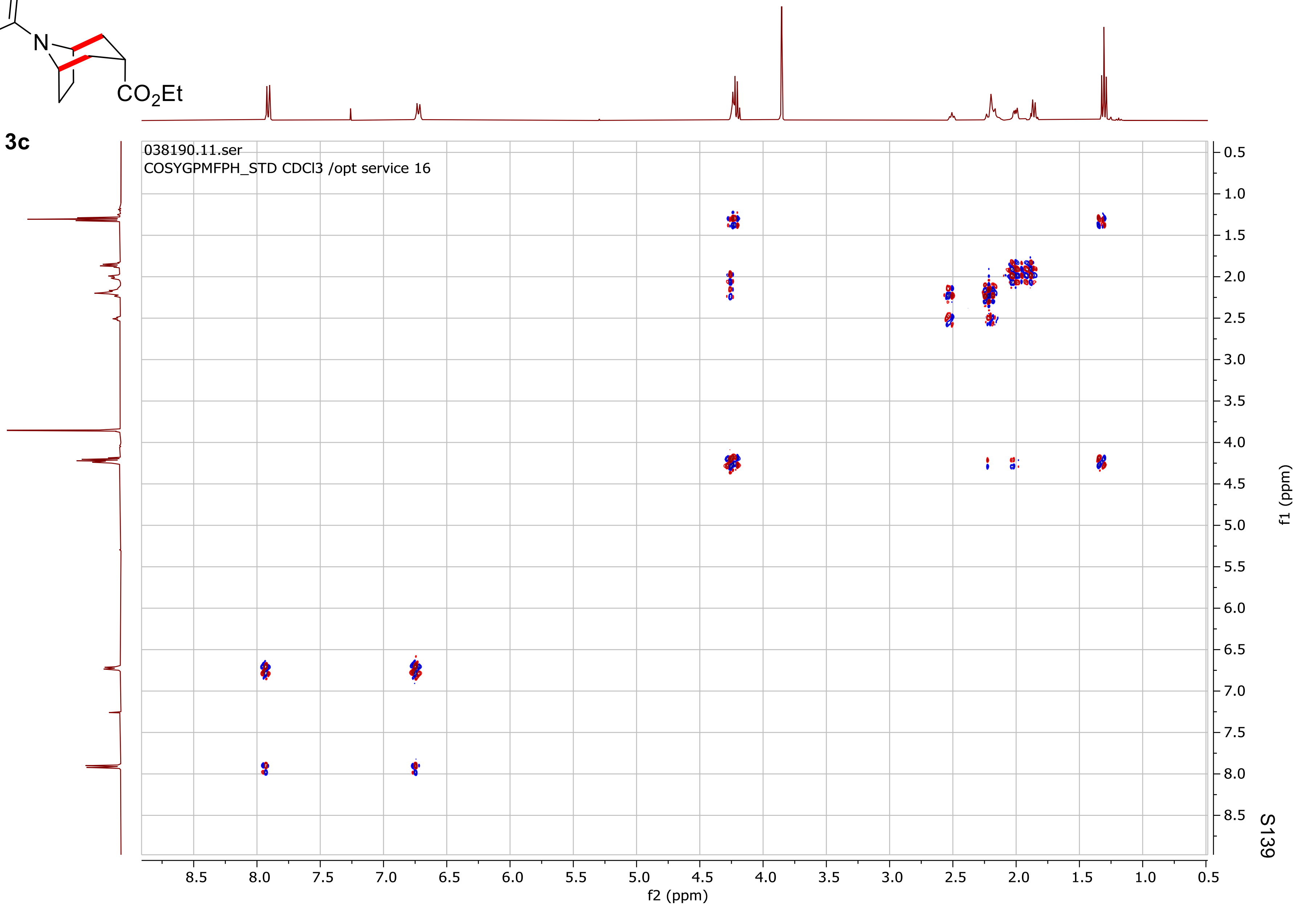

8-(4-methoxycarbonylphenyl)-8-azabicyclo[3.2.1]octane-3-carboxylate (**3cα**)

^{1}H, ^{13}C-HSQC NMR (400 MHz, $CDCl_3$)

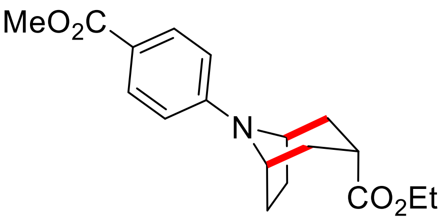

3c

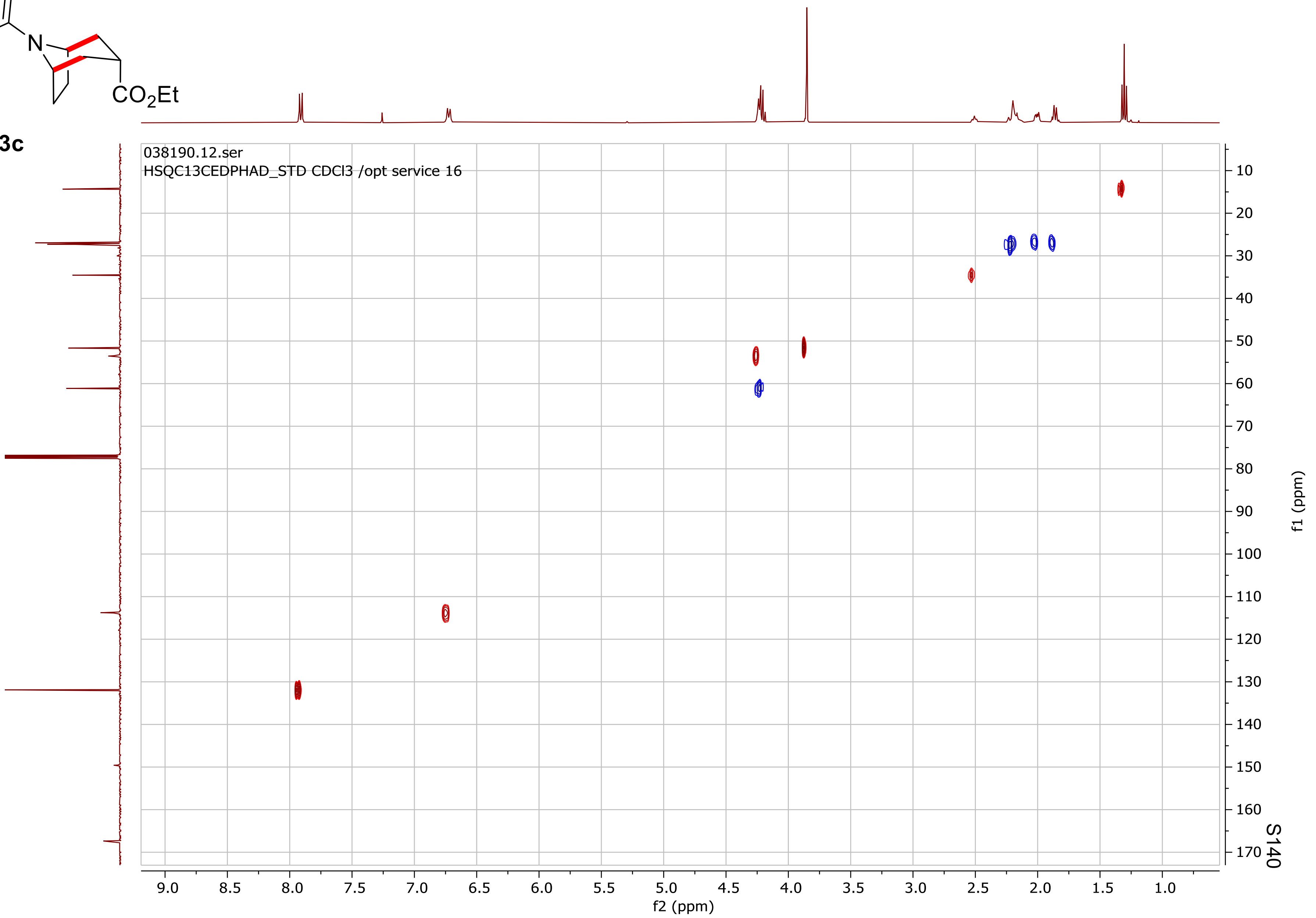

8-(4-methoxycarbonylphenyl)-8-azabicyclo[3.2.1]octane-3-carboxylate (**3cα**)

^{1}H, ^{13}C-HMBC NMR (400 MHz, $CDCl_3$)

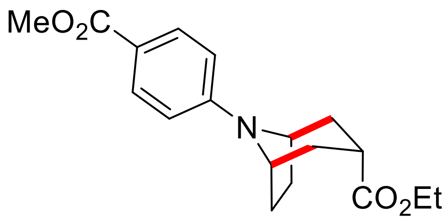

3c

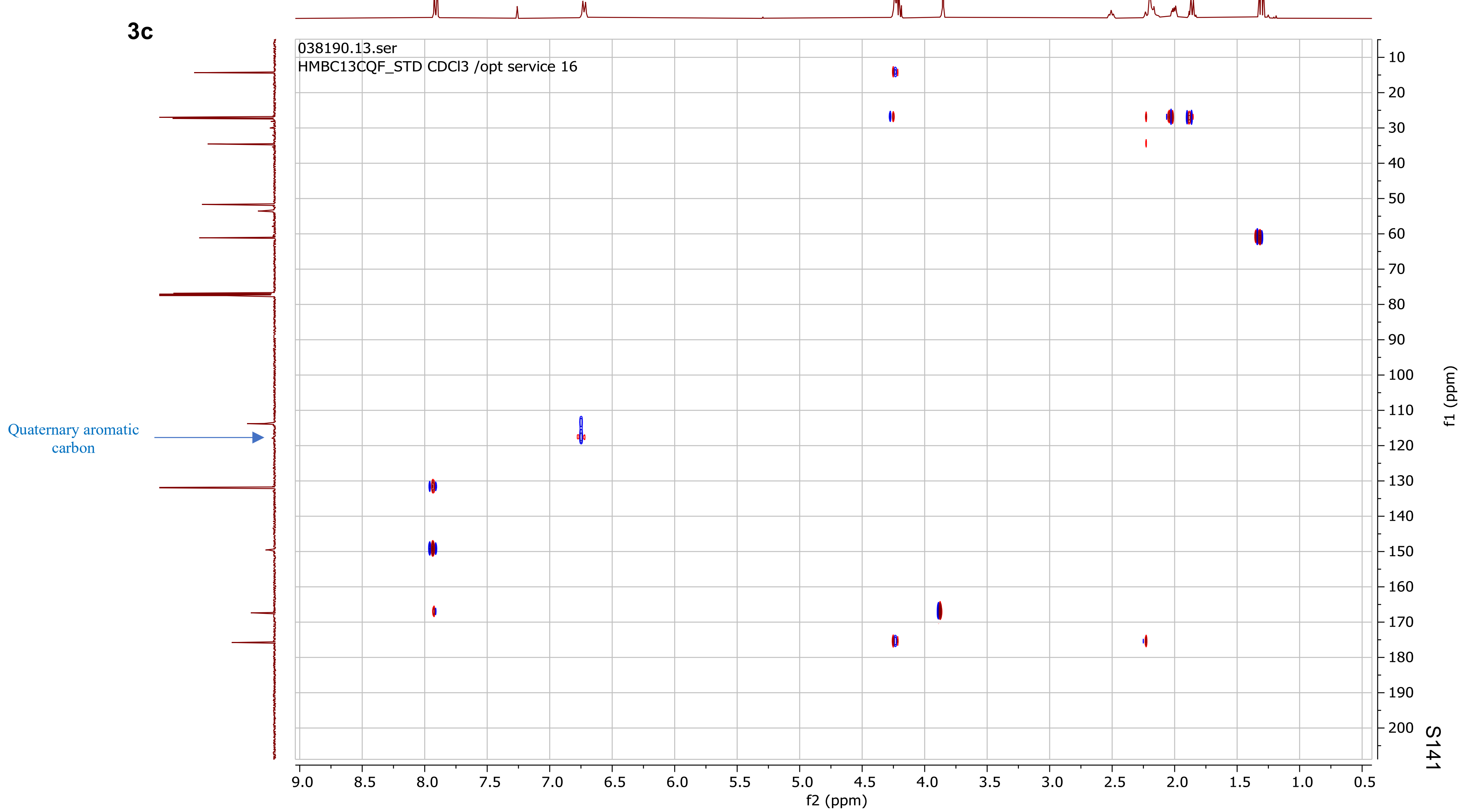

8-(4-methoxycarbonylphenyl)-8-azabicyclo[3.2.1]octane-3-carboxylate (**3cα**)

FT-IR, ATR-diamond

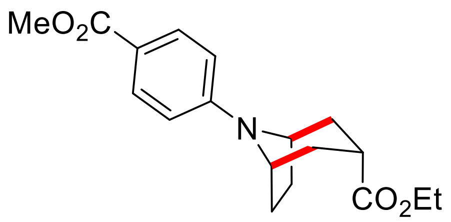

3c

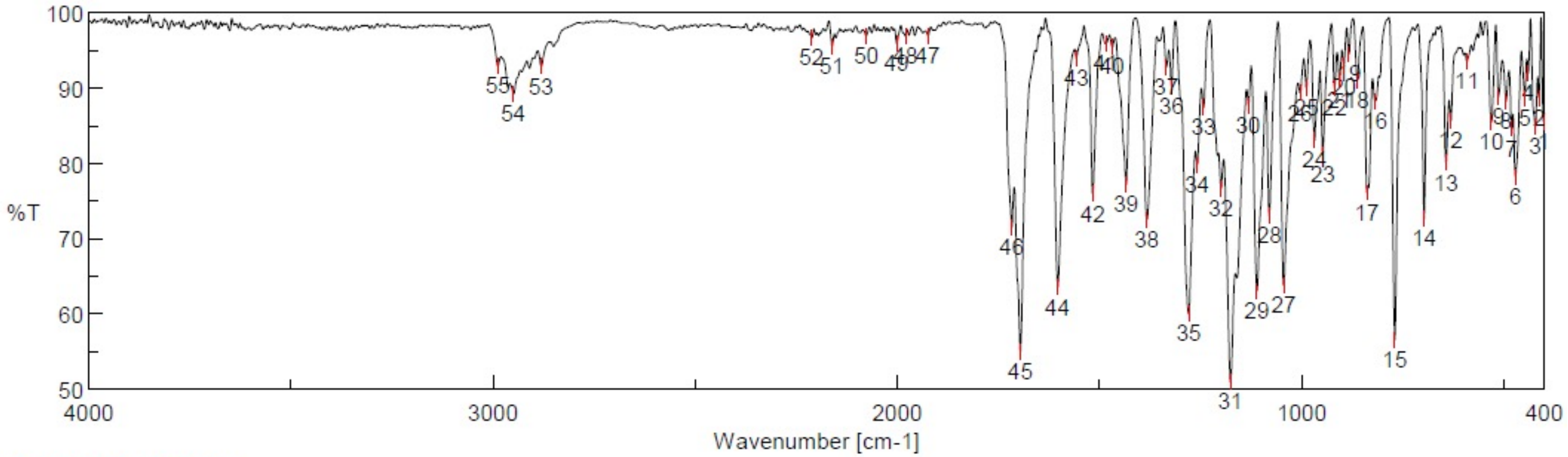

[Result of Peak Picking]

No.	Position	Intensity	No.	Position	Intensity	No.	Position	Intensity
1	402.085	86	2	412.692	89	3	422.334	85
4	439.69	92	5	448.369	89	6	471.51	78
7	481.153	85	8	494.652	88	9	512.972	89
10	531.293	85	11	591.075	94	12	631.573	86
13	643.144	80	14	697.141	73	15	769.458	57
16	818.634	88	17	838.883	76	18	862.989	91
19	883.238	95	20	895.773	93	21	906.379	91
22	918.914	90	23	947.842	81	24	968.09	83
25	988.339	90	26	1003.77	89	27	1044.26	64
28	1079.94	73	29	1110.8	63	30	1132.01	88
31	1176.36	51	32	1199.51	77	33	1242.9	87
34	1258.32	80	35	1279.54	60	36	1321.96	90
37	1334.5	93	38	1382.71	73	39	1434.78	77
40	1468.53	95	41	1484.92	96	42	1516.74	76
43	1557.24	94	44	1603.52	63	45	1696.09	55
46	1717.3	71	47	1924.61	97	48	1980.54	97
49	2001.75	96	50	2076.96	97	51	2161.81	96

8-(4-methoxycarbonylphenyl)-8-azabicyclo[3.2.1]octane-3-carboxylate (**3c, mix of dia**)

Characteristic peaks of the β product

^{1}H-NMR (400 MHz, $CDCl_3$)

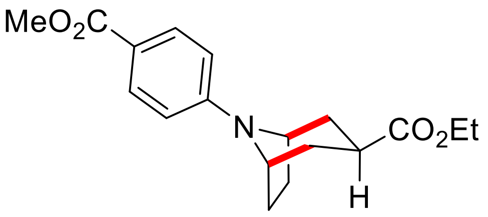

3c

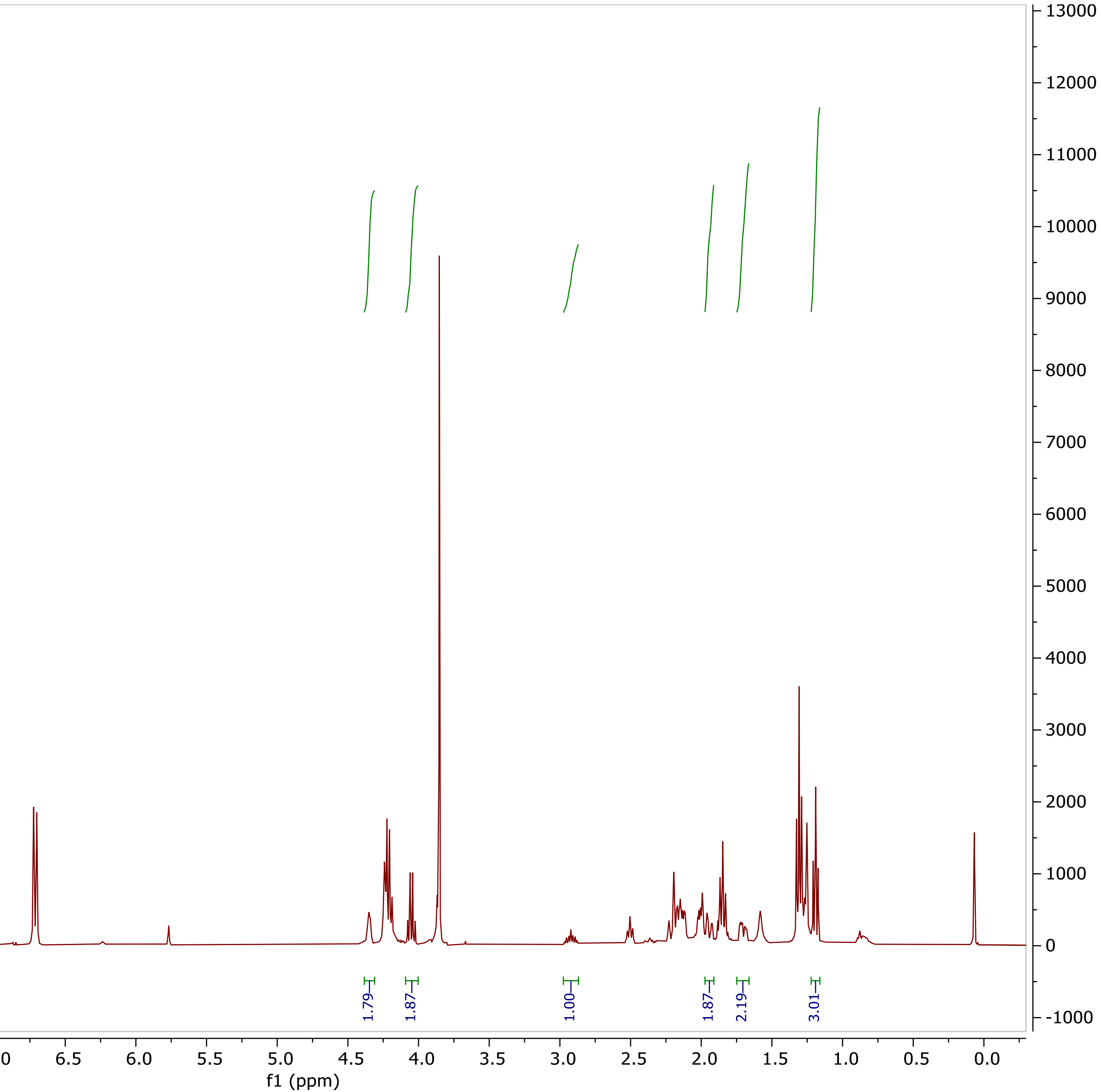

8-(4-methoxycarbonylphenyl)-8-azabicyclo[3.2.1]octane-3-carboxylate (**3c, mix of dia**)

Characteristic peaks of the β product

^{1}H-NMR (400 MHz, $CDCl_3$)

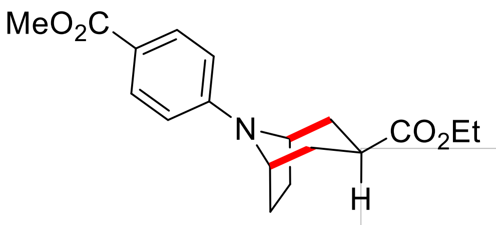

3c

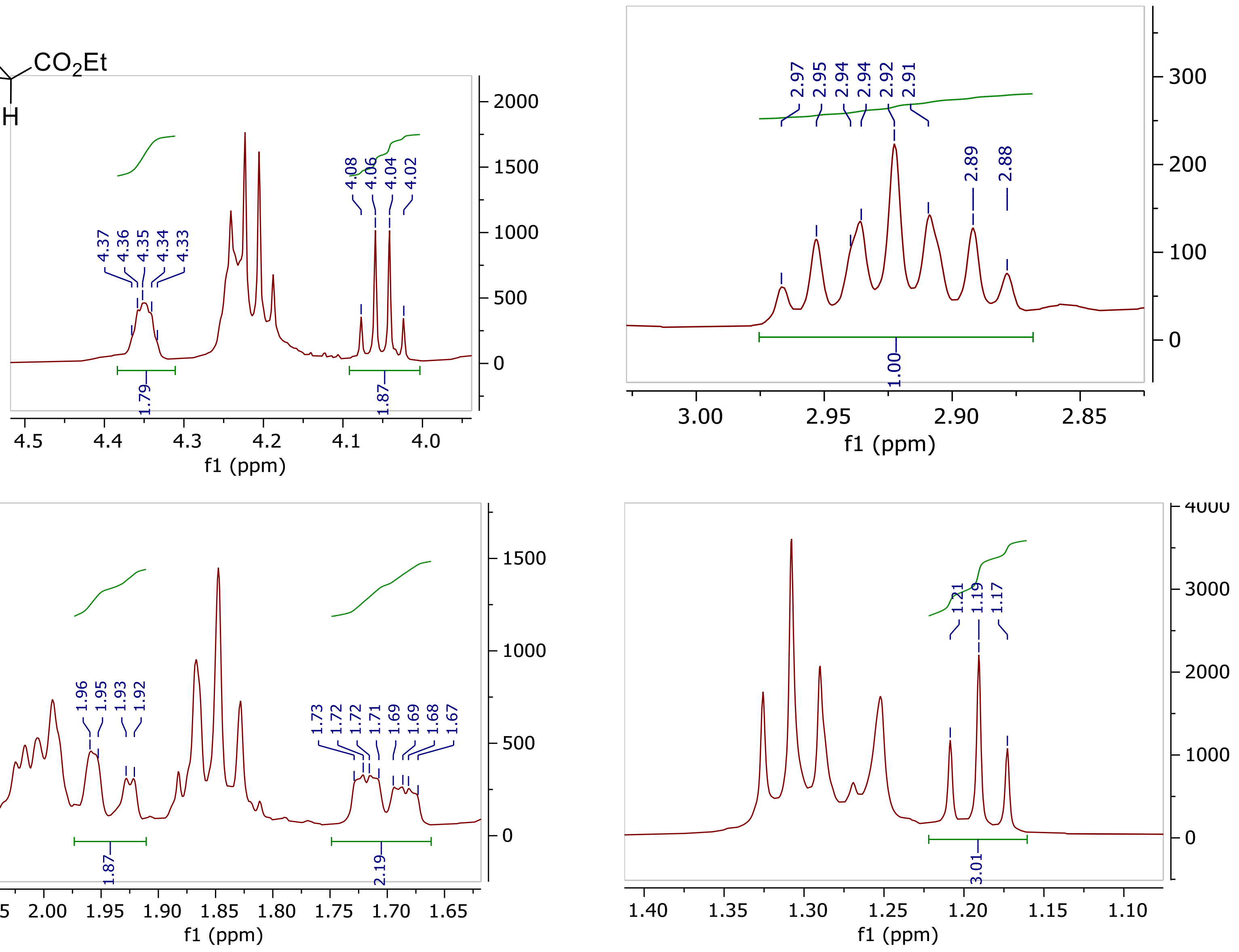

8-(4-methoxycarbonylphenyl)-8-azabicyclo[3.2.1]octane-3-carboxylate (**3c, mix of dia**)

Characteristic peaks of the β product

^{13}C-NMR (101 MHz, $CDCl_3$)

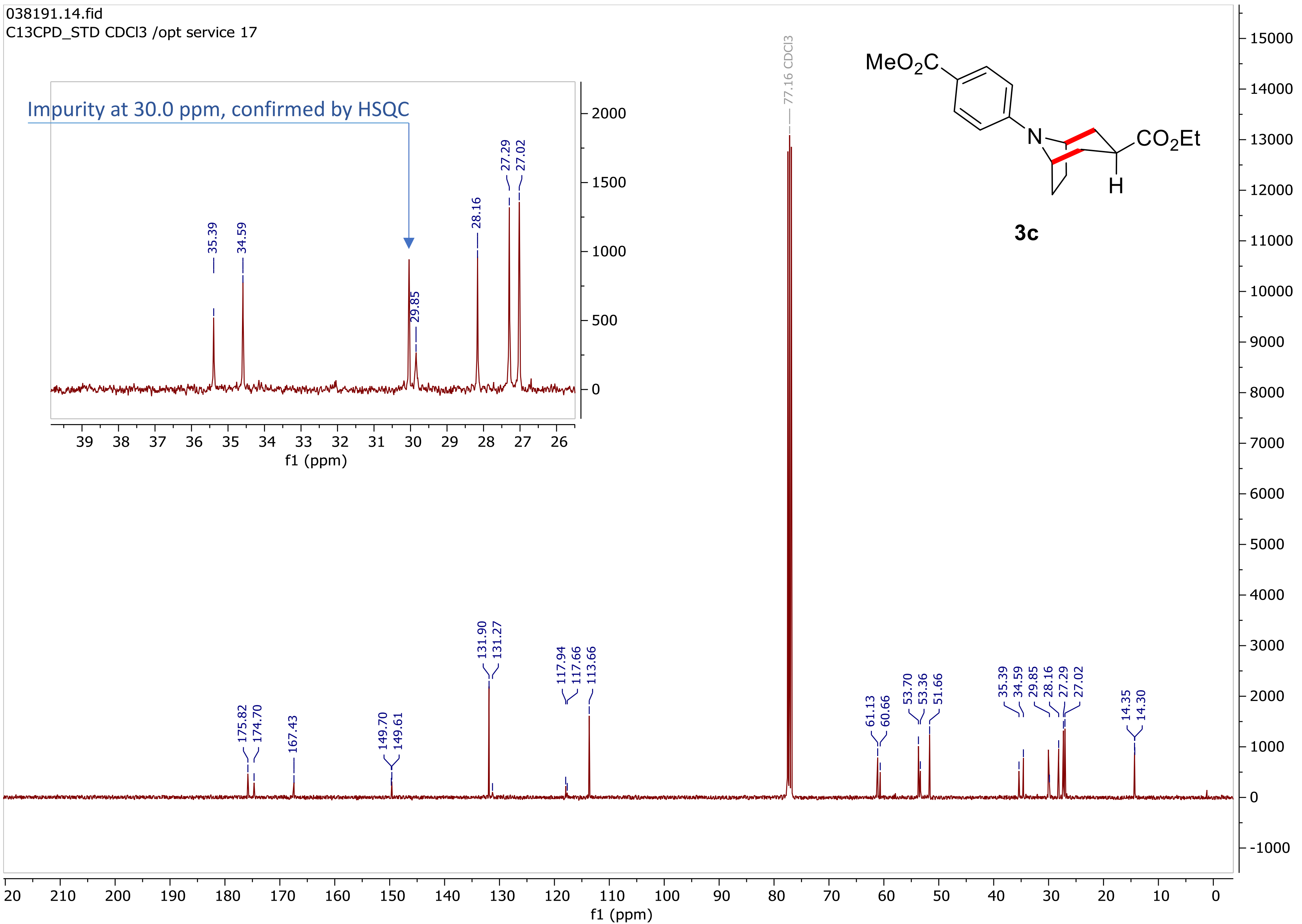

8-(4-methoxycarbonylphenyl)-8-azabicyclo[3.2.1]octane-3-carboxylate (**3c, mix of dia**)
Characteristic peaks of the β product

^{13}C-NMR (101 MHz, $CDCl_3$)

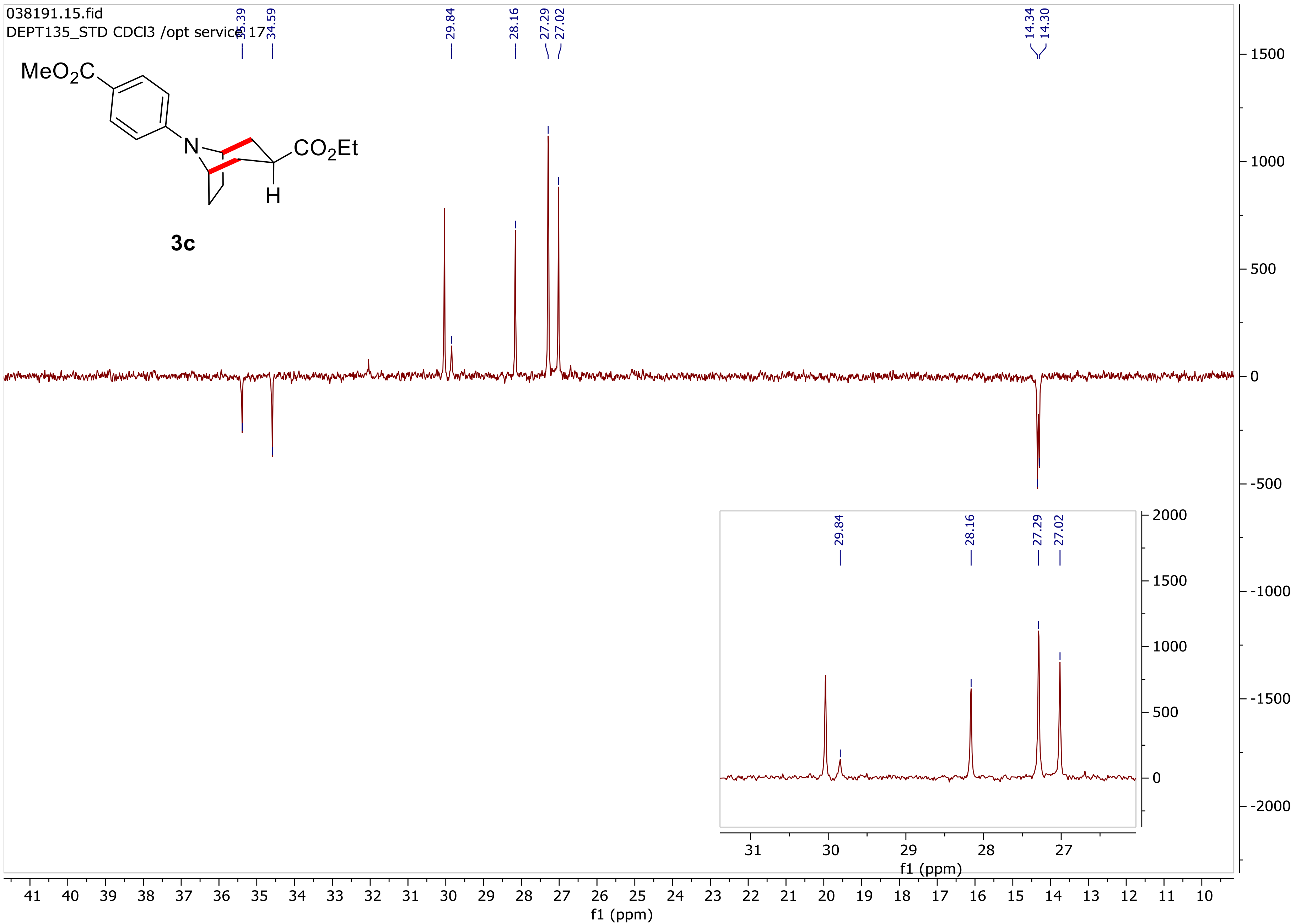

Ethyl 8-(4-methoxyphenyl)-8-azabicyclo[3.2.1]octane-3-carboxylate (**3dα**)

^{1}H-NMR (300 MHz, $CDCl_3$)

GA_206916.10.fid
eco-2-126 F1
Proton_ns16 CDCl3 /opt service 54

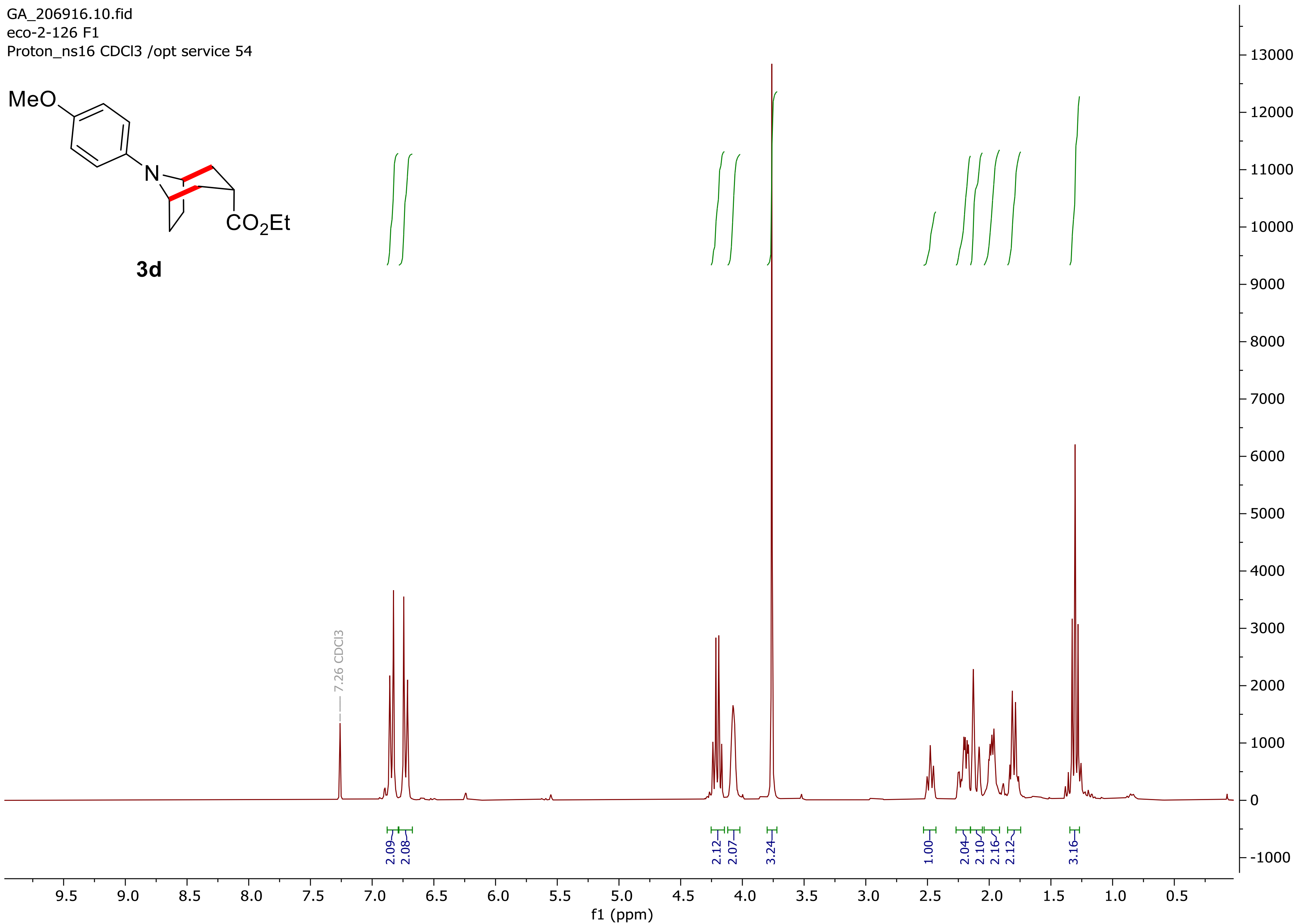

Ethyl 8-(4-methoxyphenyl)-8-azabicyclo[3.2.1]octane-3-carboxylate (**3dα**)

^{13}C-NMR (75 MHz, $CDCl_3$)

GA_206916.11.fid
eco-2-126 F1
Carbon_ns512 CDCl3 /opt service 54

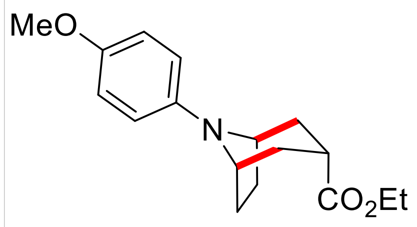

3d

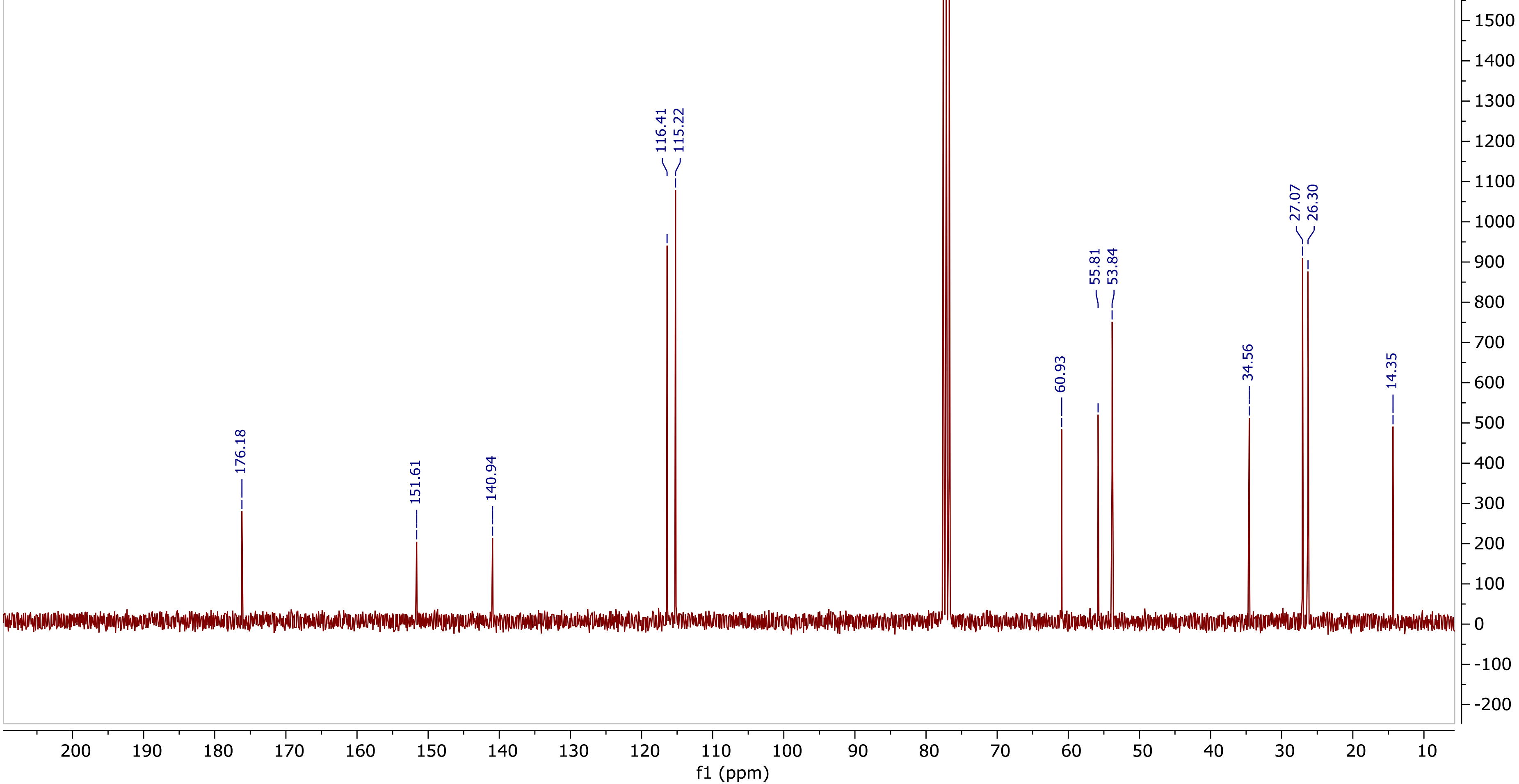

Ethyl 8-(4-methoxyphenyl)-8-azabicyclo[3.2.1]octane-3-carboxylate (**3dα**)

^{13}C-NMR (75 MHz, $CDCl_3$)

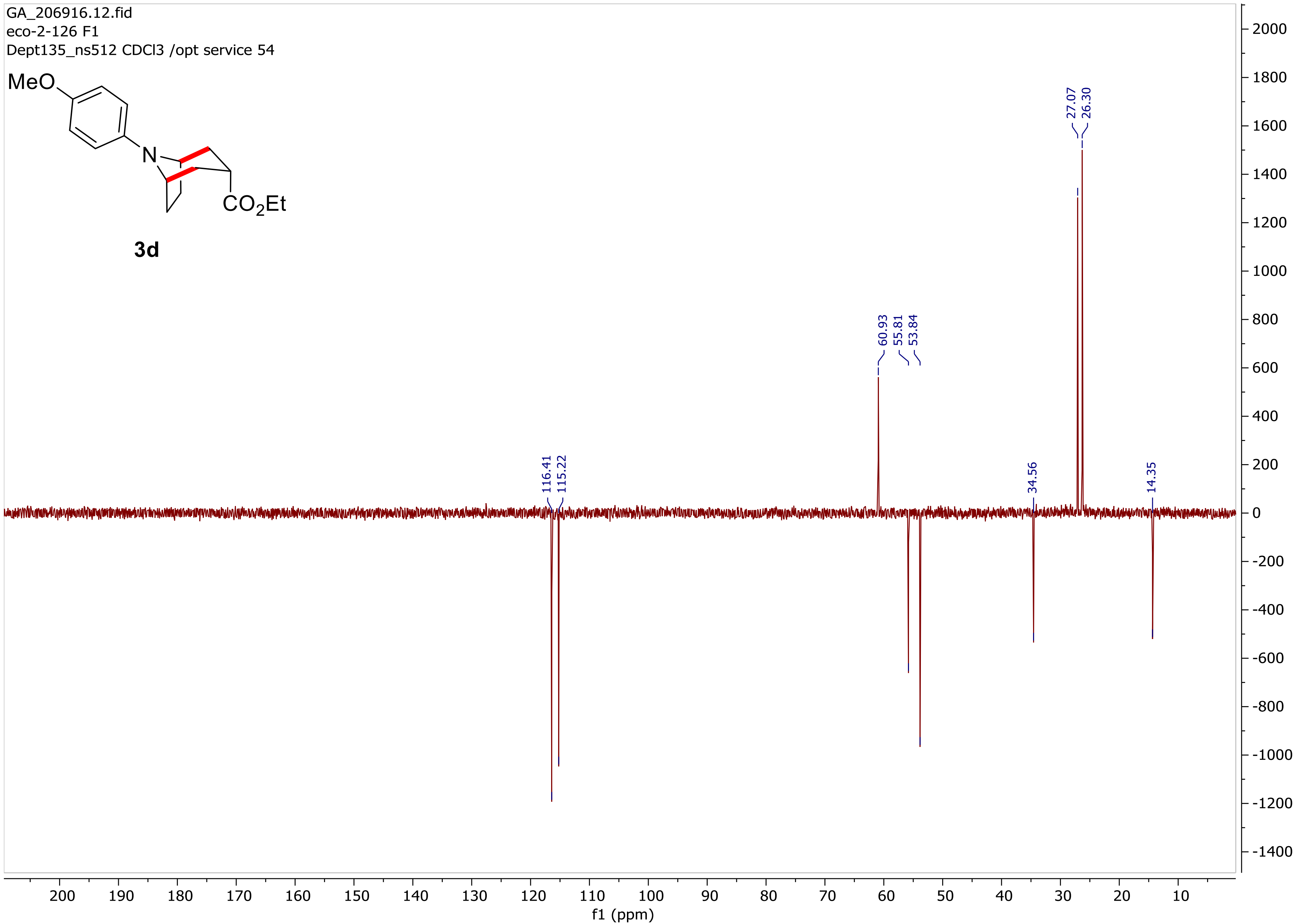

Ethyl 8-(4-methoxyphenyl)-8-azabicyclo[3.2.1]octane-3-carboxylate (**3d**) **α/β 5:1**

^{1}H-NMR (300 MHz, $CDCl_3$)

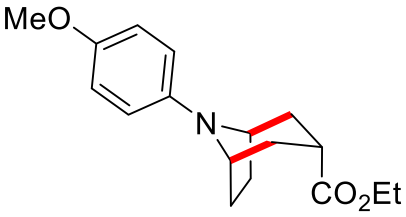

3d, a/ß 5:1

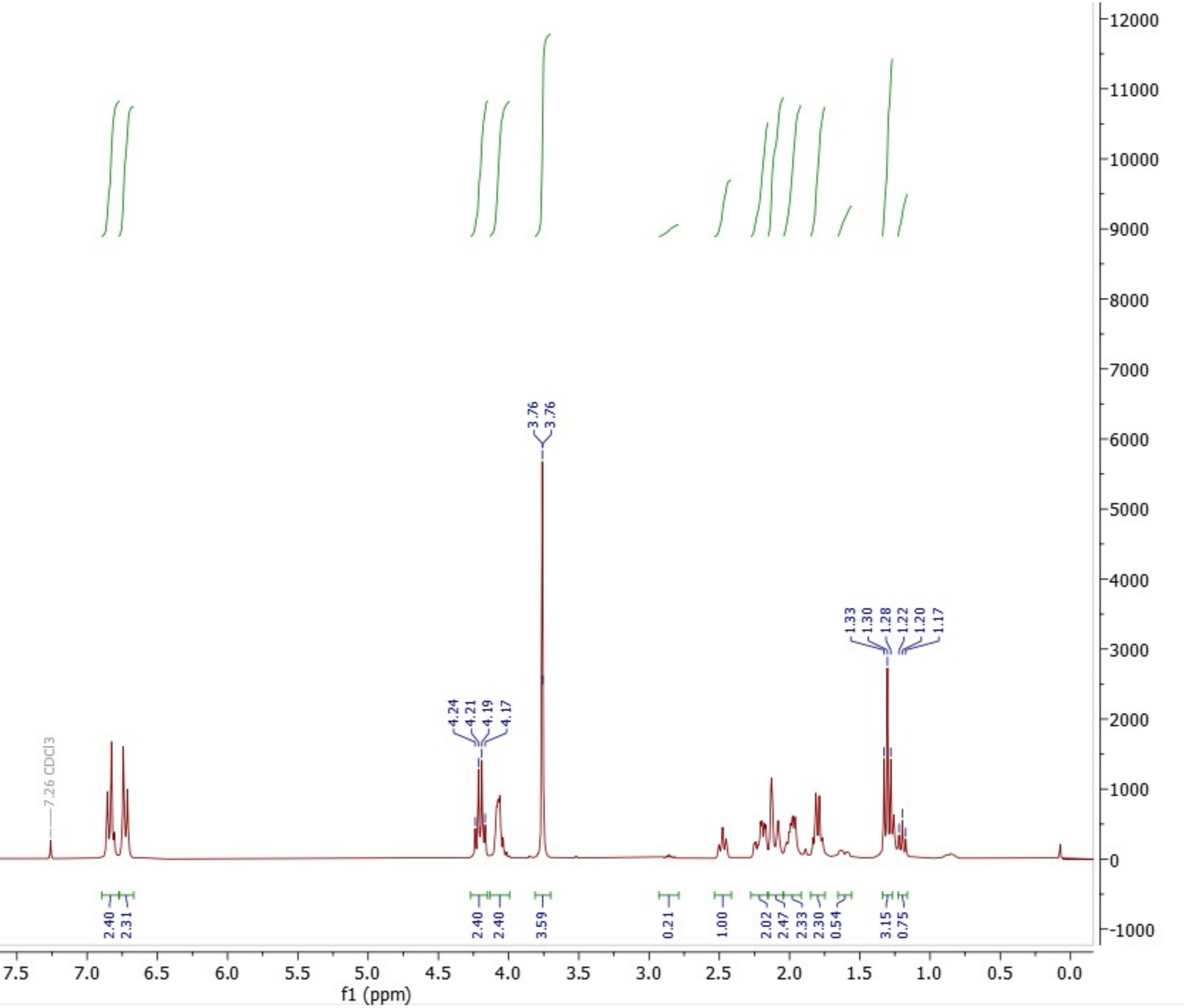

Ethyl 8-(4-methoxyphenyl)-8-azabicyclo[3.2.1]octane-3-carboxylate (**3d**) **α/β 5:1**

^{1}H-NMR (300 MHz, $CDCl_3$)

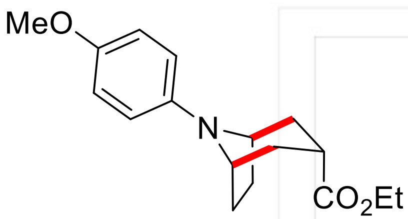

3d, a/ß 5:1

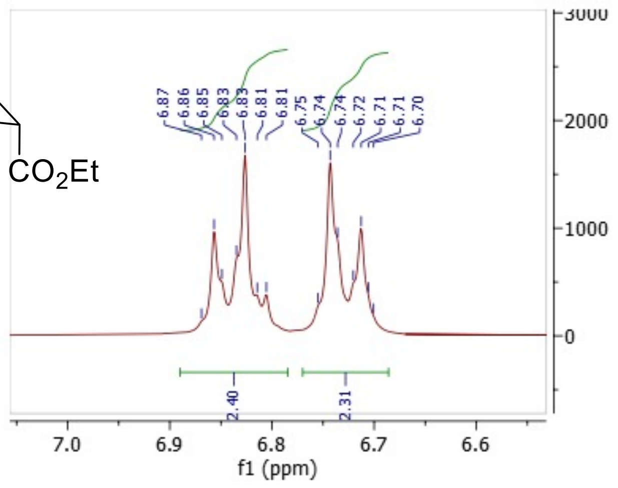

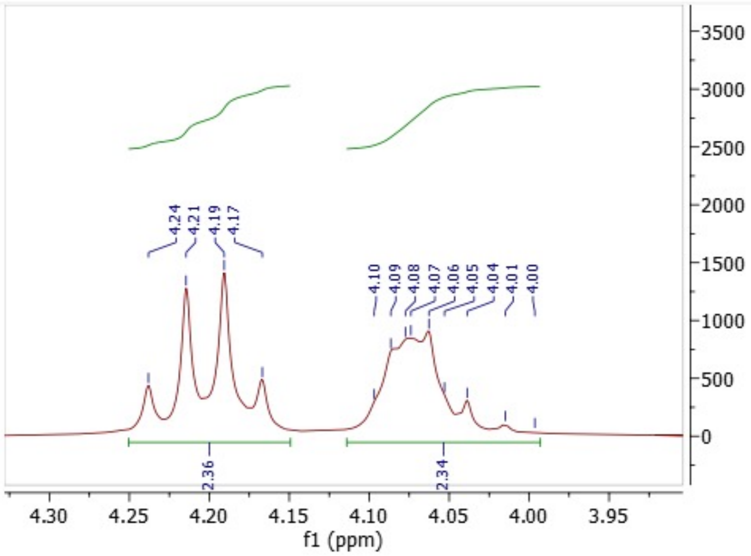

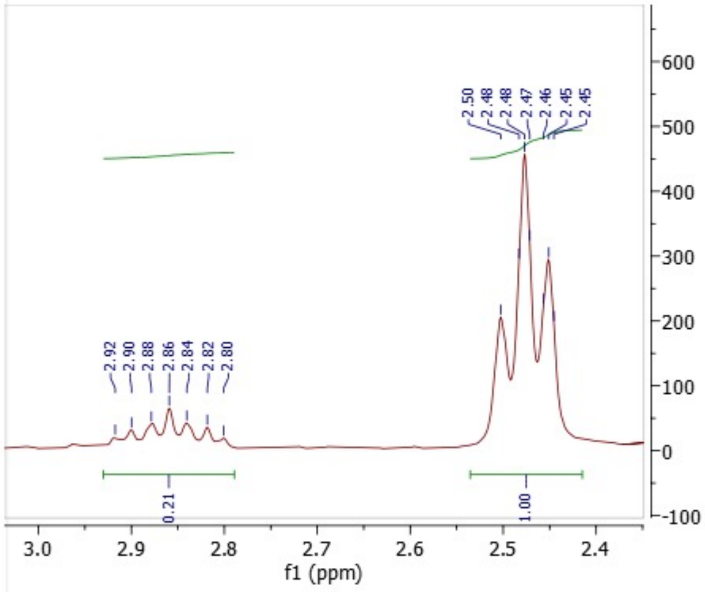

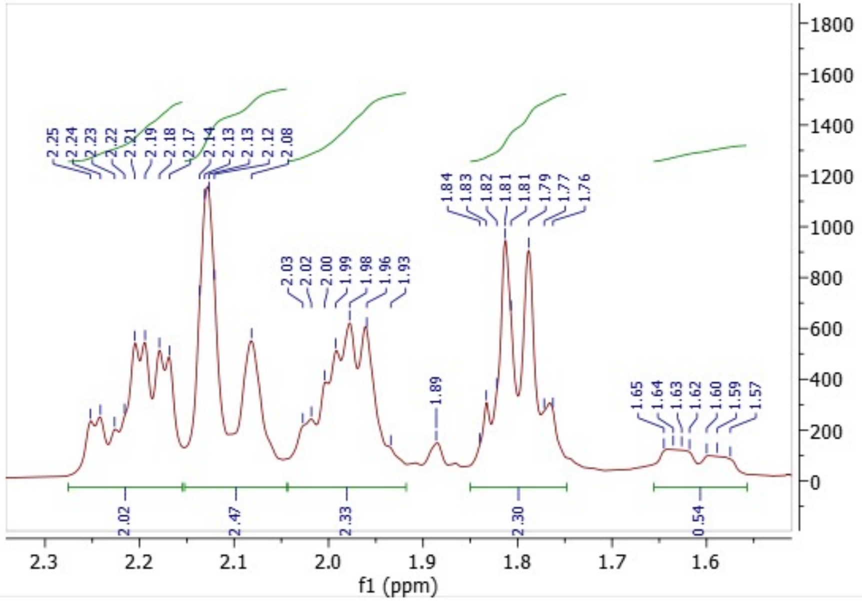

Ethyl 8-(4-methoxyphenyl)-8-azabicyclo[3.2.1]octane-3-carboxylate (**3d**) **α/β 5:1**

^{13}C-NMR (75 MHz, $CDCl_3$)

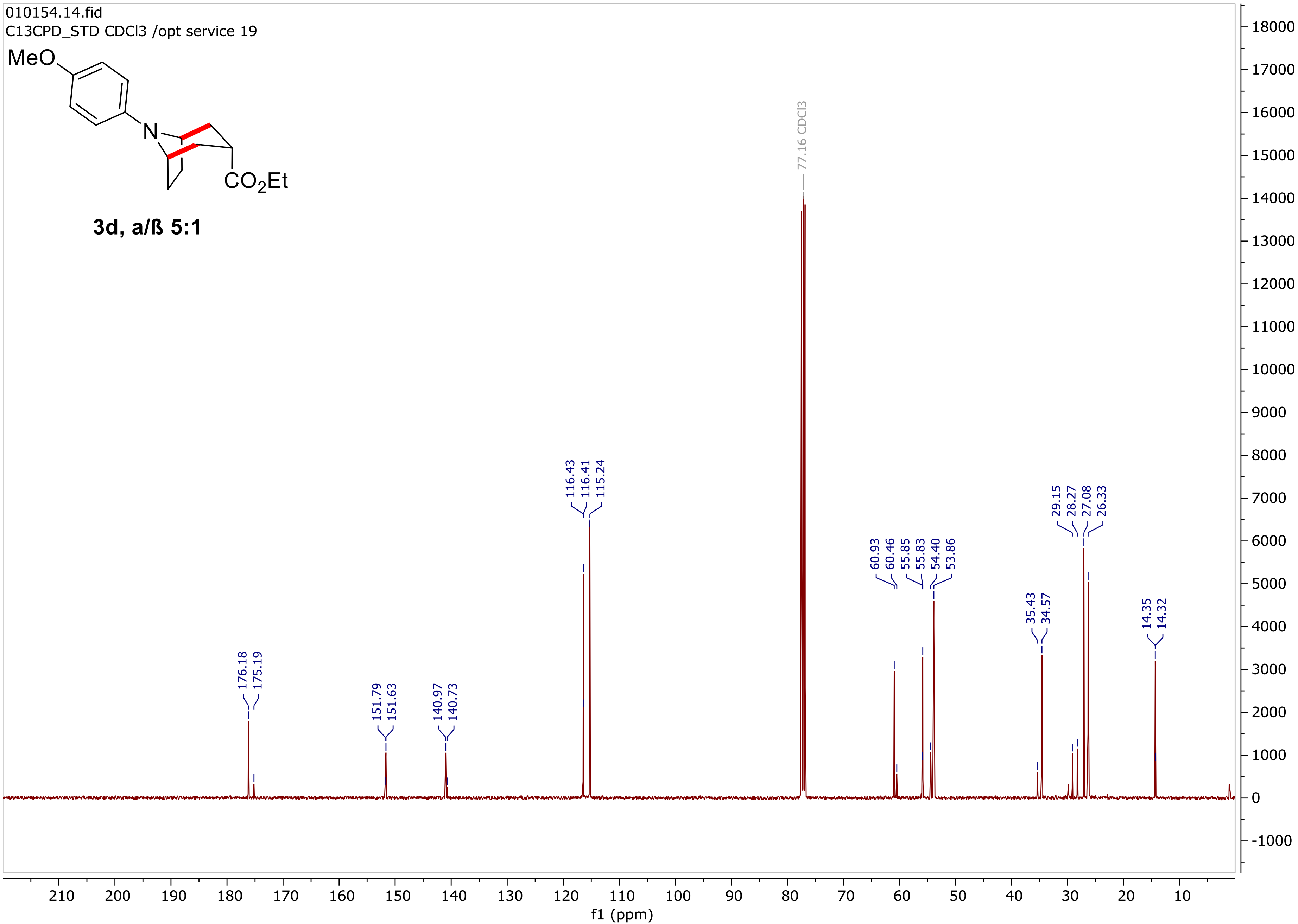

Ethyl 8-(4-methoxyphenyl)-8-azabicyclo[3.2.1]octane-3-carboxylate (**3d**) **α/β 5:1**

^{13}C-NMR (75 MHz, $CDCl_3$)

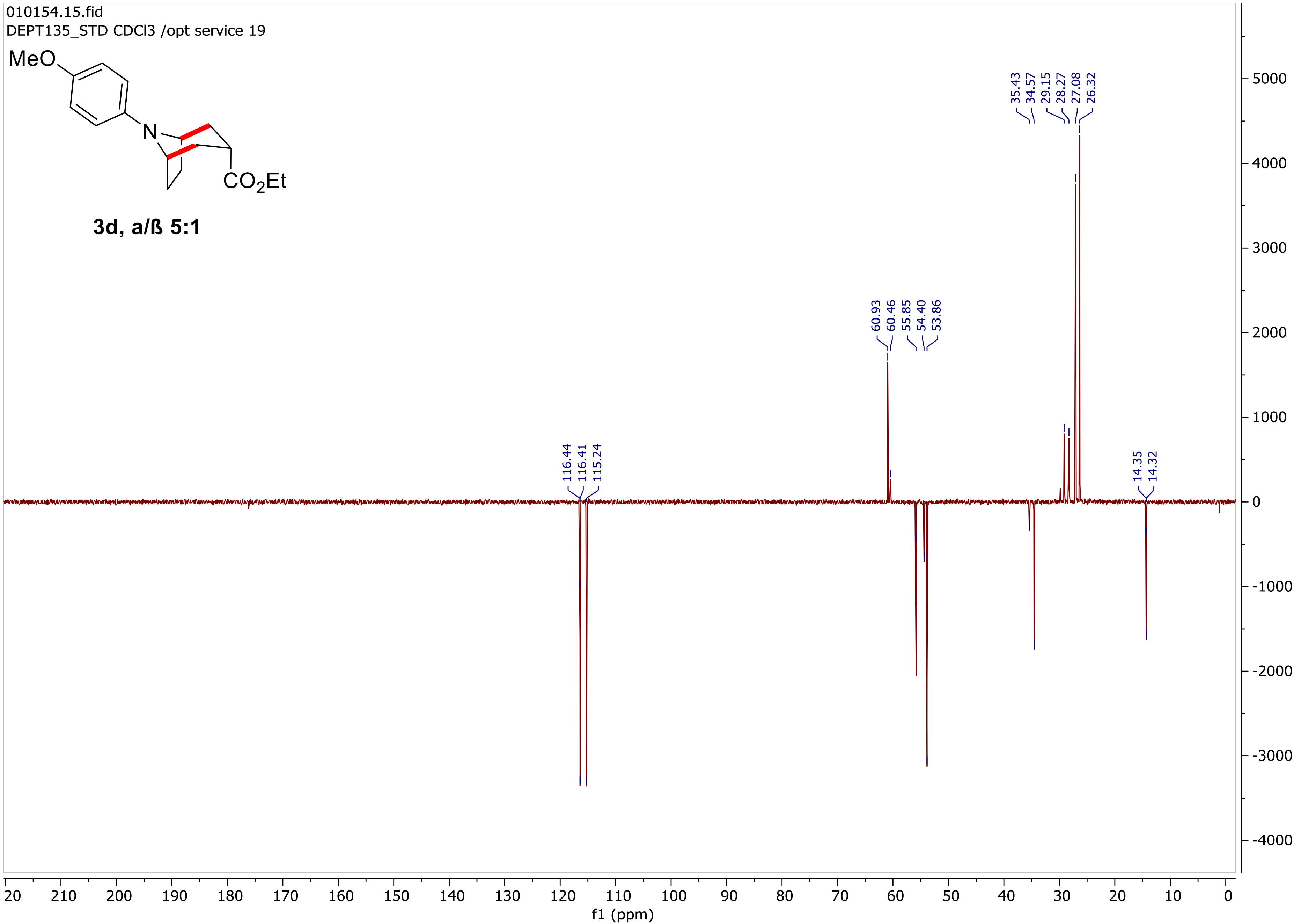

Ethyl 8-(4-methoxyphenyl)-8-azabicyclo[3.2.1]octane-3-carboxylate (**3d**) **α/β 5:1**

^{1}H, ^{1}H-COSY NMR (400 MHz, $CDCl_3$)

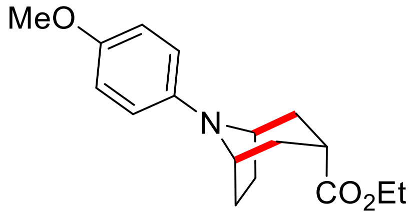

3d, a/ß 5:1

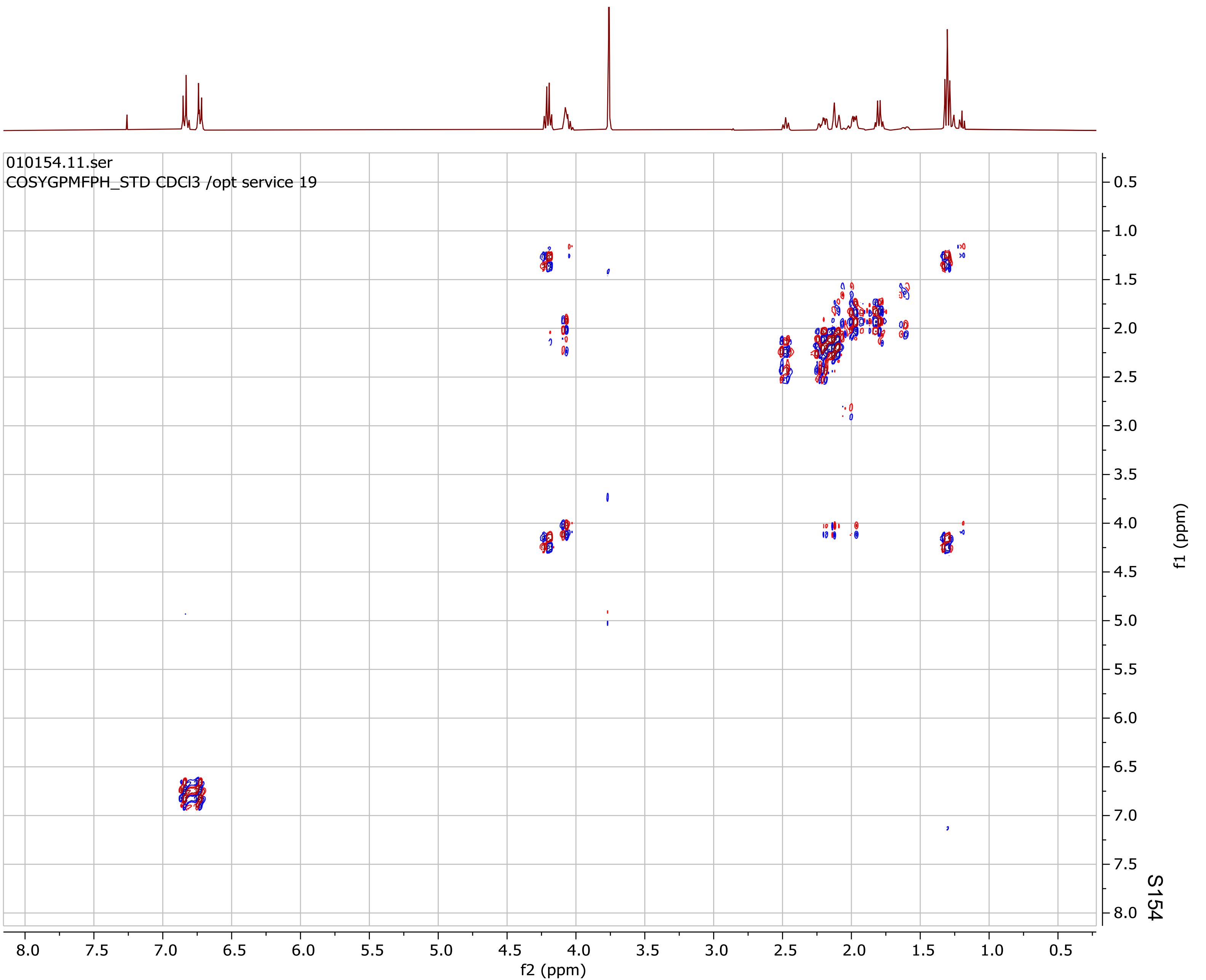

Ethyl 8-(4-methoxyphenyl)-8-azabicyclo[3.2.1]octane-3-carboxylate (**3d**) **α/β 5:1**

^{1}H, ^{13}C-HSQC NMR (400 MHz, $CDCl_3$)

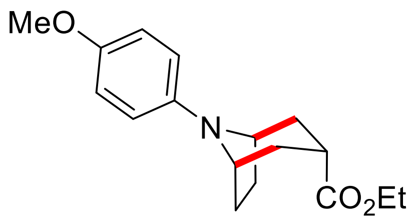

3d, a/ß 5:1

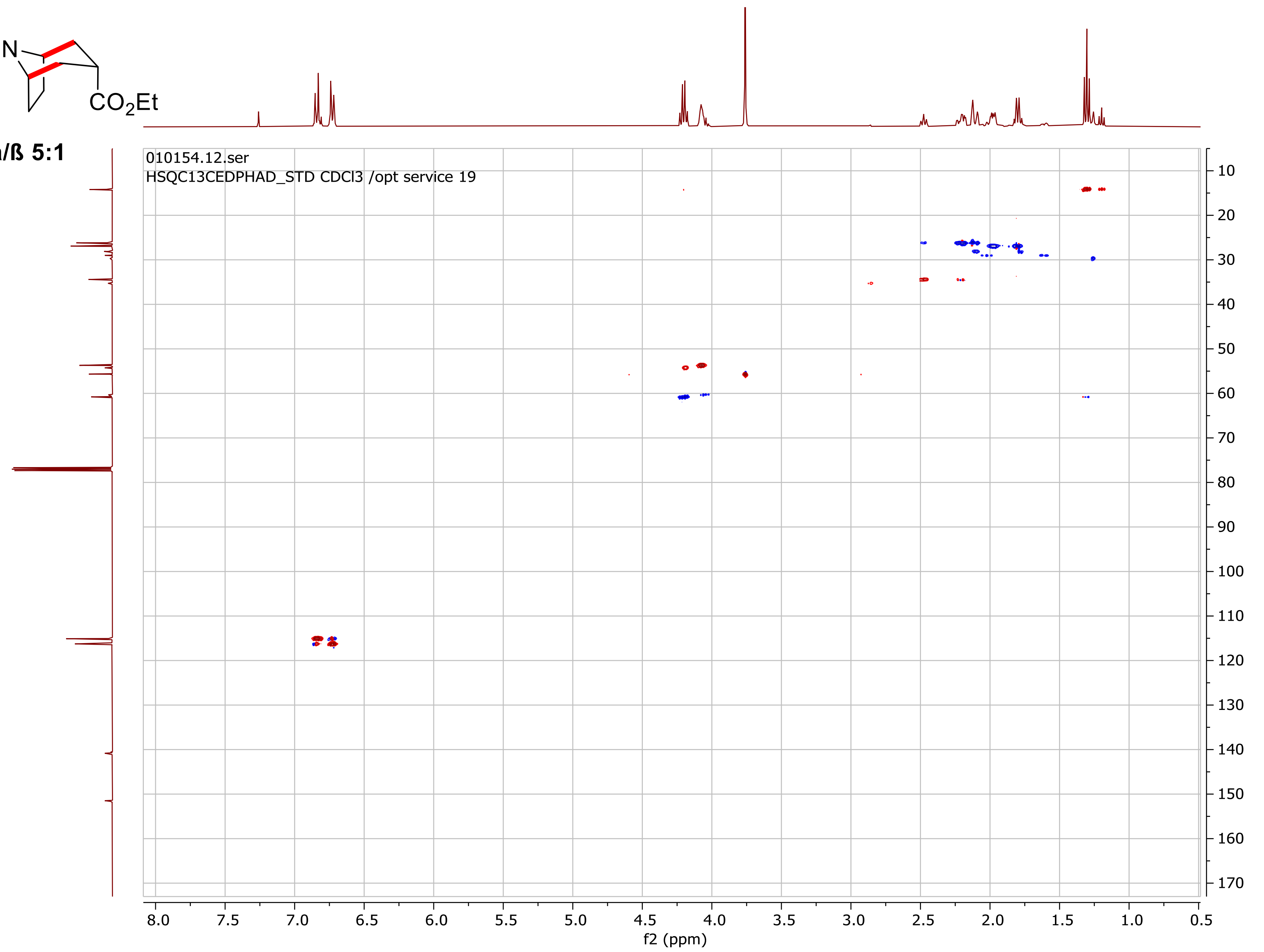

Ethyl 8-(4-methoxyphenyl)-8-azabicyclo[3.2.1]octane-3-carboxylate (**3d**) **α/β 5:1**

^1H, ^{13}C-HSQC NMR (300 MHz, $CDCl_3$)

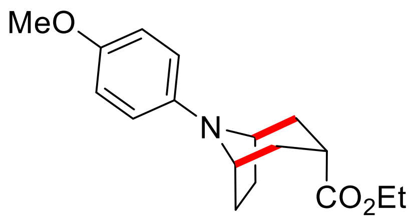

3d, a/ß 5:1

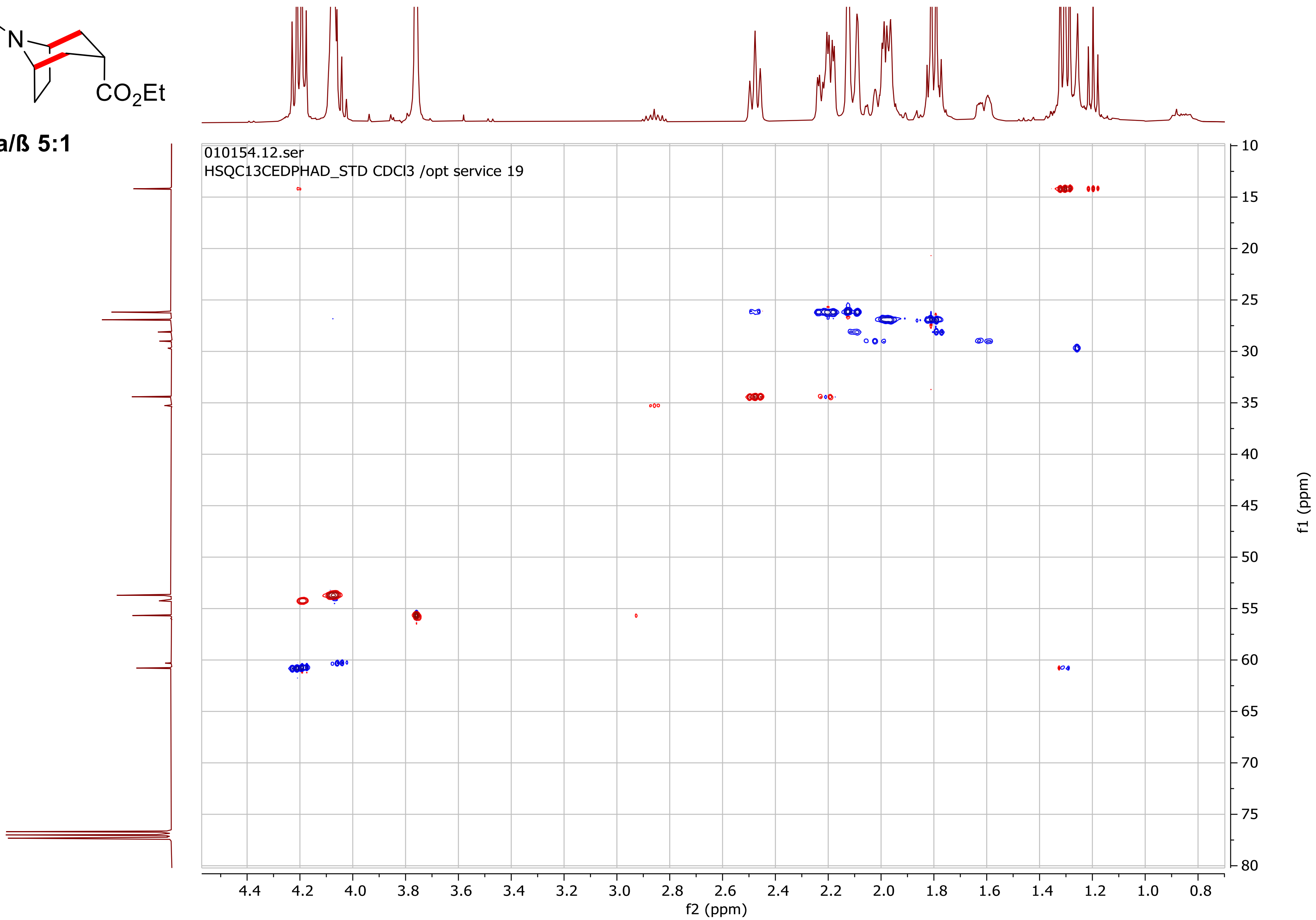

Ethyl 8-(4-methoxyphenyl)-8-azabicyclo[3.2.1]octane-3-carboxylate (**3d**) **α/β 5:1**

^1H, ^{13}C-HMBC NMR (300 MHz, $CDCl_3$)

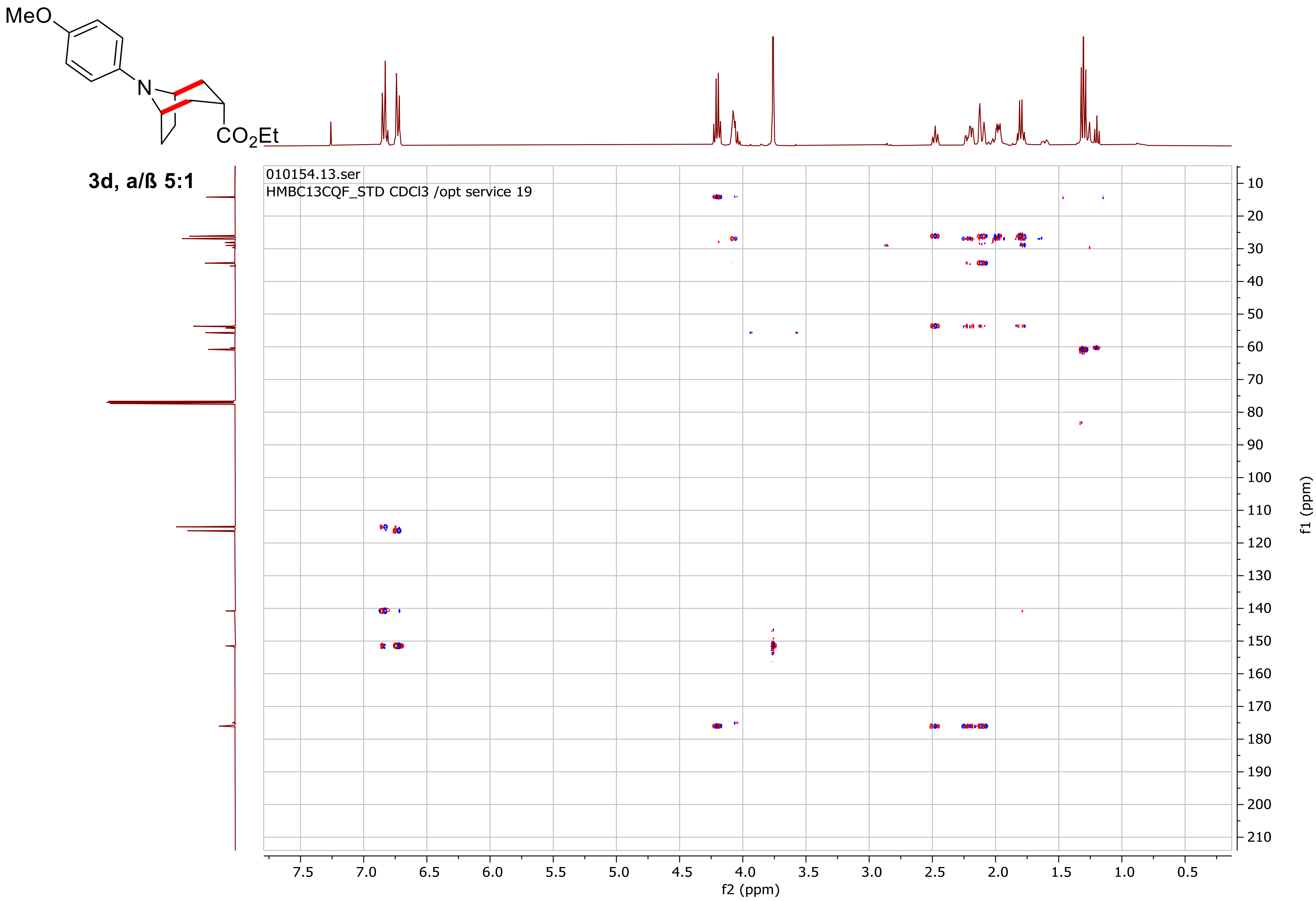

Ethyl 8-(4-methoxyphenyl)-8-azabicyclo[3.2.1]octane-3-carboxylate (**3d**) **α/β 5:1**

FT-IR, ATR-diamond

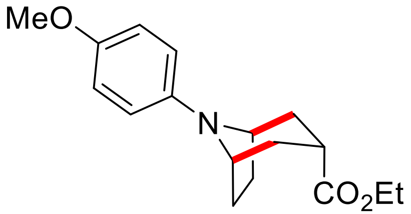

3d, a/ß 5:1

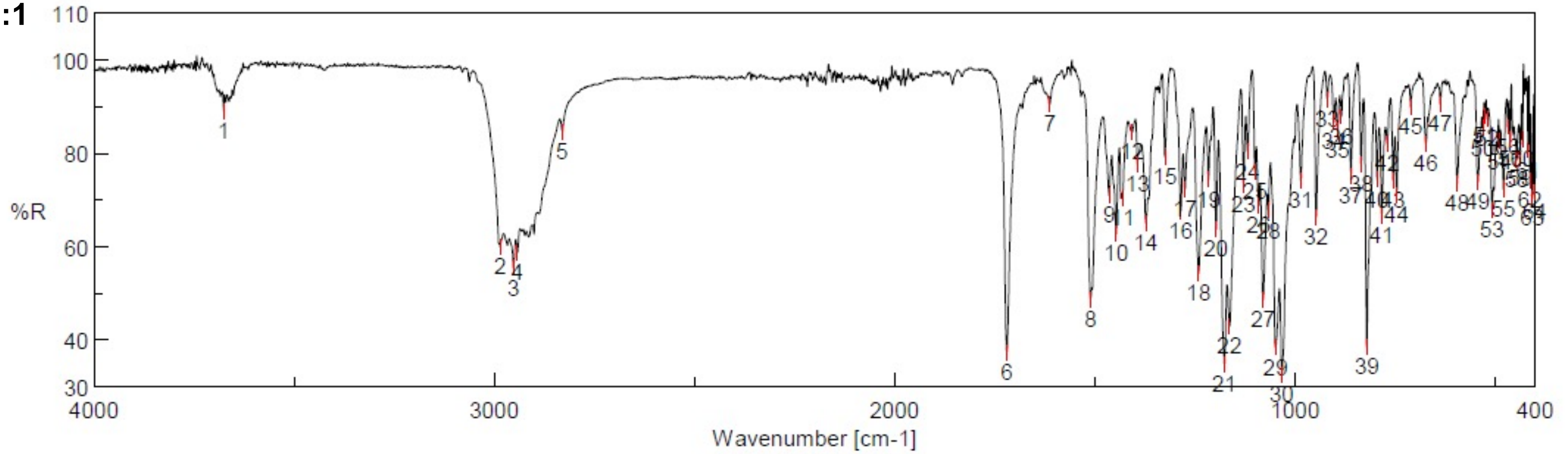

[Result of Peak Picking]

No.	Position	Intensity	No.	Position	Intensity	No.	Position	Intensity
1	3675.66	89.0562	2	2985.27	60.0397	3	2952	55.3798
4	2941.88	58.7635	5	2830.03	84.6499	6	1719.71	37.2626
7	1612.68	90.4472	8	1510.47	48.692	9	1464.19	70.9779
10	1446.83	62.7223	11	1430.44	70.2985	12	1406.33	84.5555
13	1393.32	77.6565	14	1372.1	65.0323	15	1324.86	79.2332
16	1285.32	67.509	17	1274.72	72.2347	18	1240.97	54.308
19	1215.9	74.3419	20	1197.1	63.6711	21	1176.36	34.8462
22	1163.35	42.8756	23	1128.64	73.2428	24	1118.03	80.3202
25	1099.71	76.1276	26	1090.55	68.818	27	1079.94	48.5501
28	1066.44	67.7128	29	1048.6	38.3581	30	1032.69	32.5384
31	985.447	74.1801	32	947.359	66.2803	33	919.397	91.5596
34	903.005	86.6709	35	893.362	84.7633	36	883.72	87.8701
37	860.096	75.1851	38	834.544	77.7785	39	820.563	38.4065
40	795.01	74.3486	41	782.958	66.5519	42	769.458	81.9663
43	754.995	74.1081	44	745.835	71.0029	45	710.64	89.925
46	673.035	82.0502	47	636.876	90.4691	48	594.932	73.5266
49	543.346	73.8567	50	530.811	85.3552	51	525.507	88.0835

Ethyl 8-(4-methoxyphenyl)-8-azabicyclo[3.2.1]octane-3-carboxylate (**3e**) **α/β 6:1**

^{1}H-NMR (300 MHz, $CDCl_3$)

GA_216550.10.fid
ECO-4-053 F1
Proton_ns16 CDCl3 /opt renaud 46

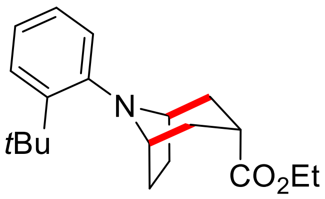

3e, a/ß 6:1

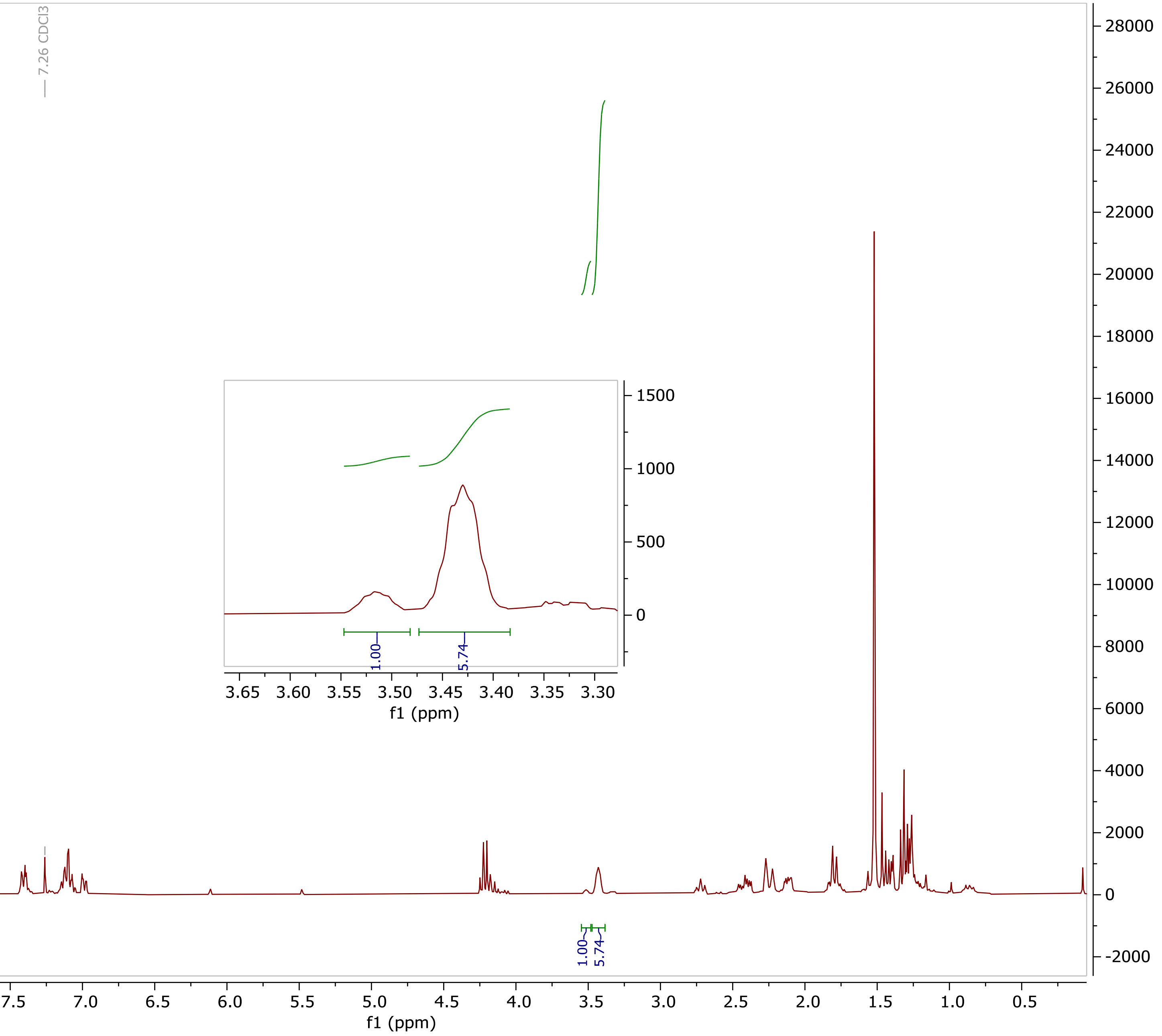

Ethyl 8-(4-methoxyphenyl)-8-azabicyclo[3.2.1]octane-3-carboxylate (**3eα**)

^{1}H-NMR (400 MHz, $CDCl_3$)

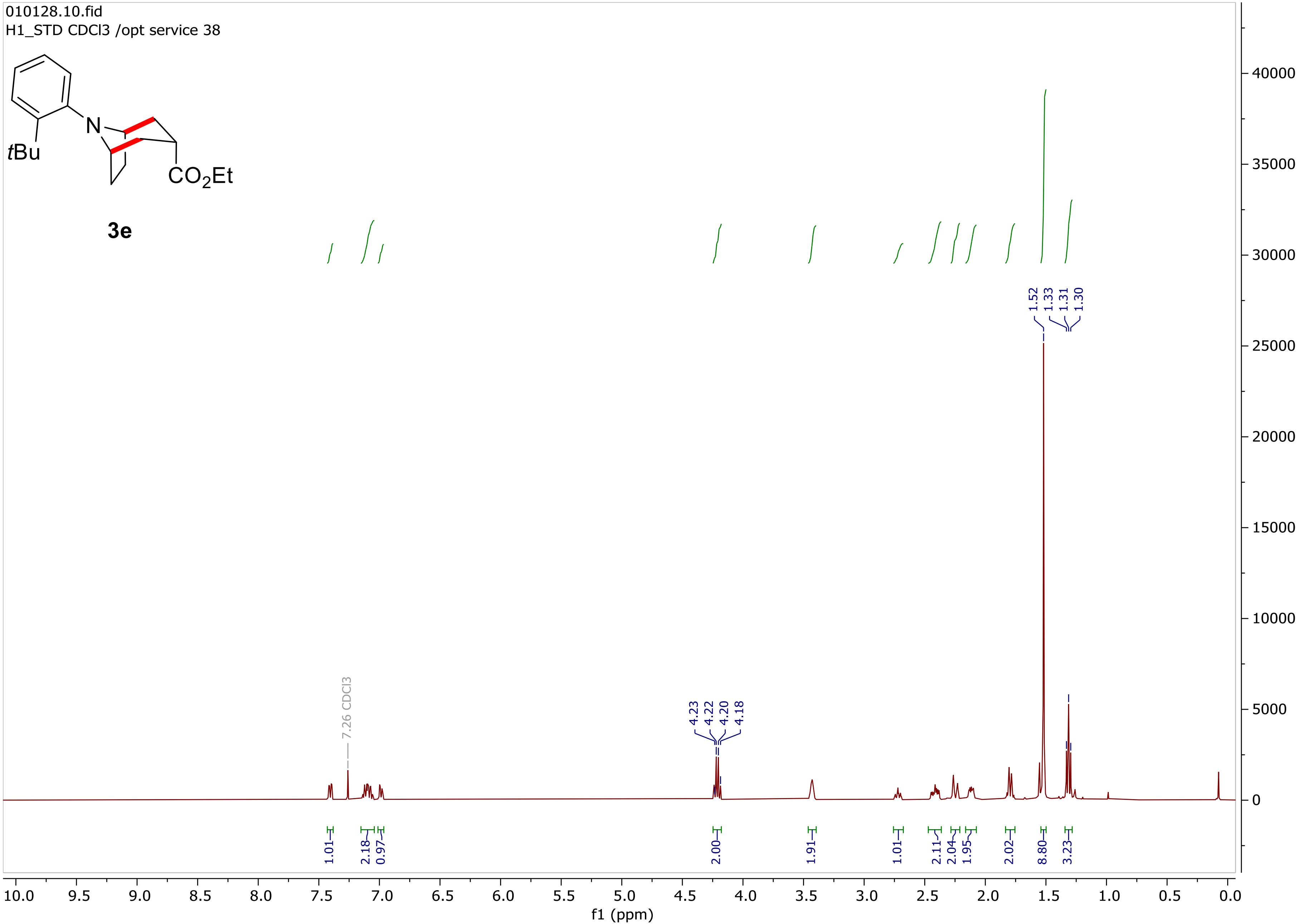

Ethyl 8-(4-methoxyphenyl)-8-azabicyclo[3.2.1]octane-3-carboxylate (**3eα**)

^{1}H-NMR (400 MHz, $CDCl_3$)

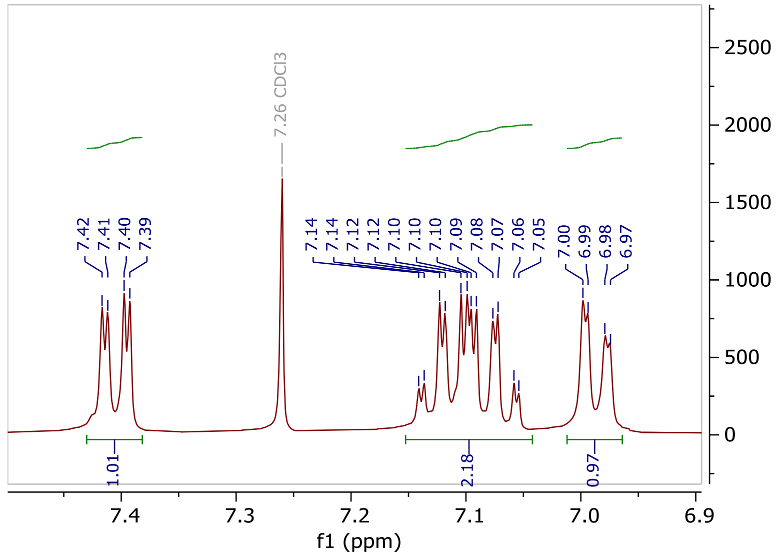

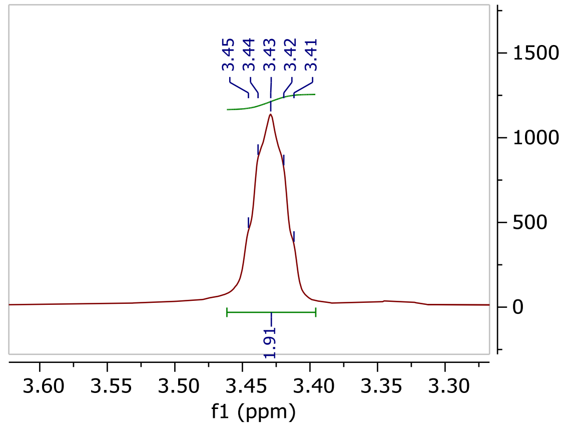

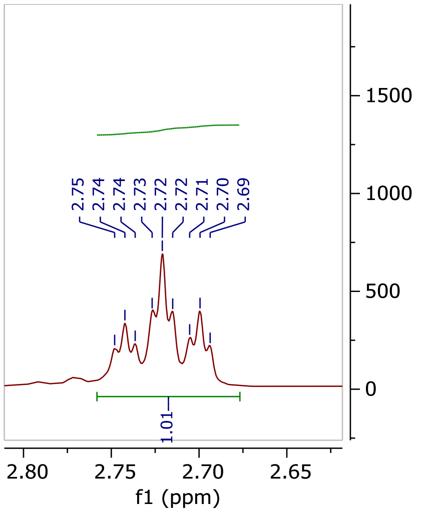

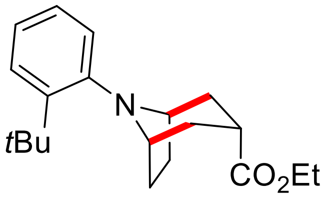

3e

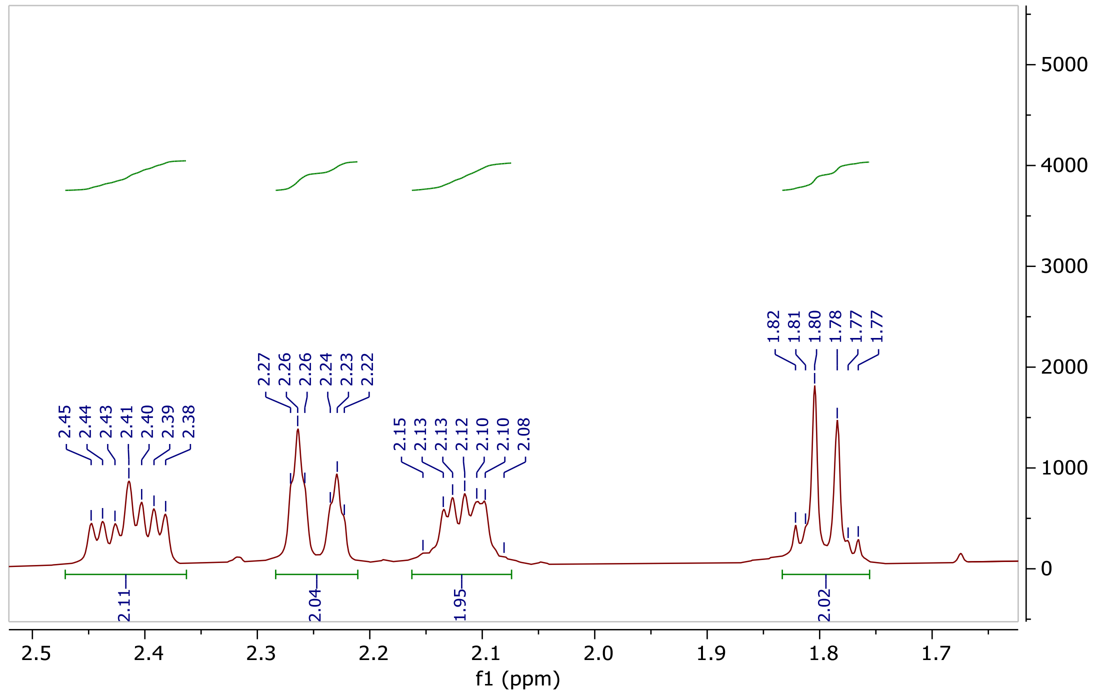

Ethyl 8-(4-methoxyphenyl)-8-azabicyclo[3.2.1]octane-3-carboxylate (**3eα**)

^{13}C-NMR (101 MHz, $CDCl_3$)

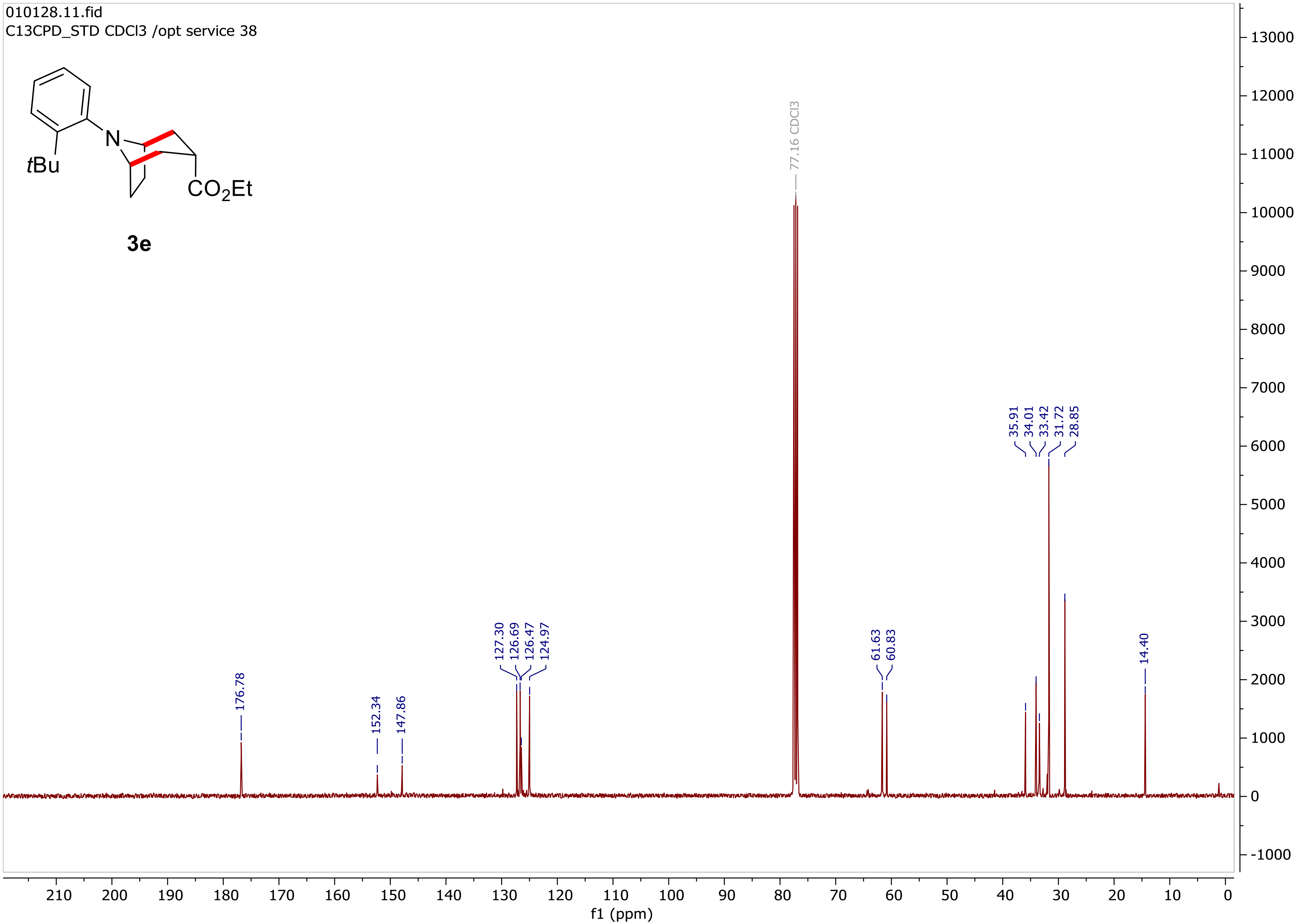

Ethyl 8-(4-methoxyphenyl)-8-azabicyclo[3.2.1]octane-3-carboxylate (**3eα**)

^{13}C-NMR (101 MHz, $CDCl_3$)

010128.12.fid
DEPT135_STD CDCl3 /opt service 38

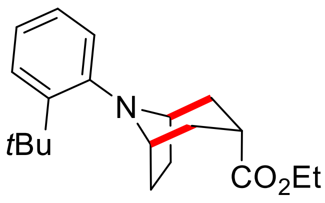

3e

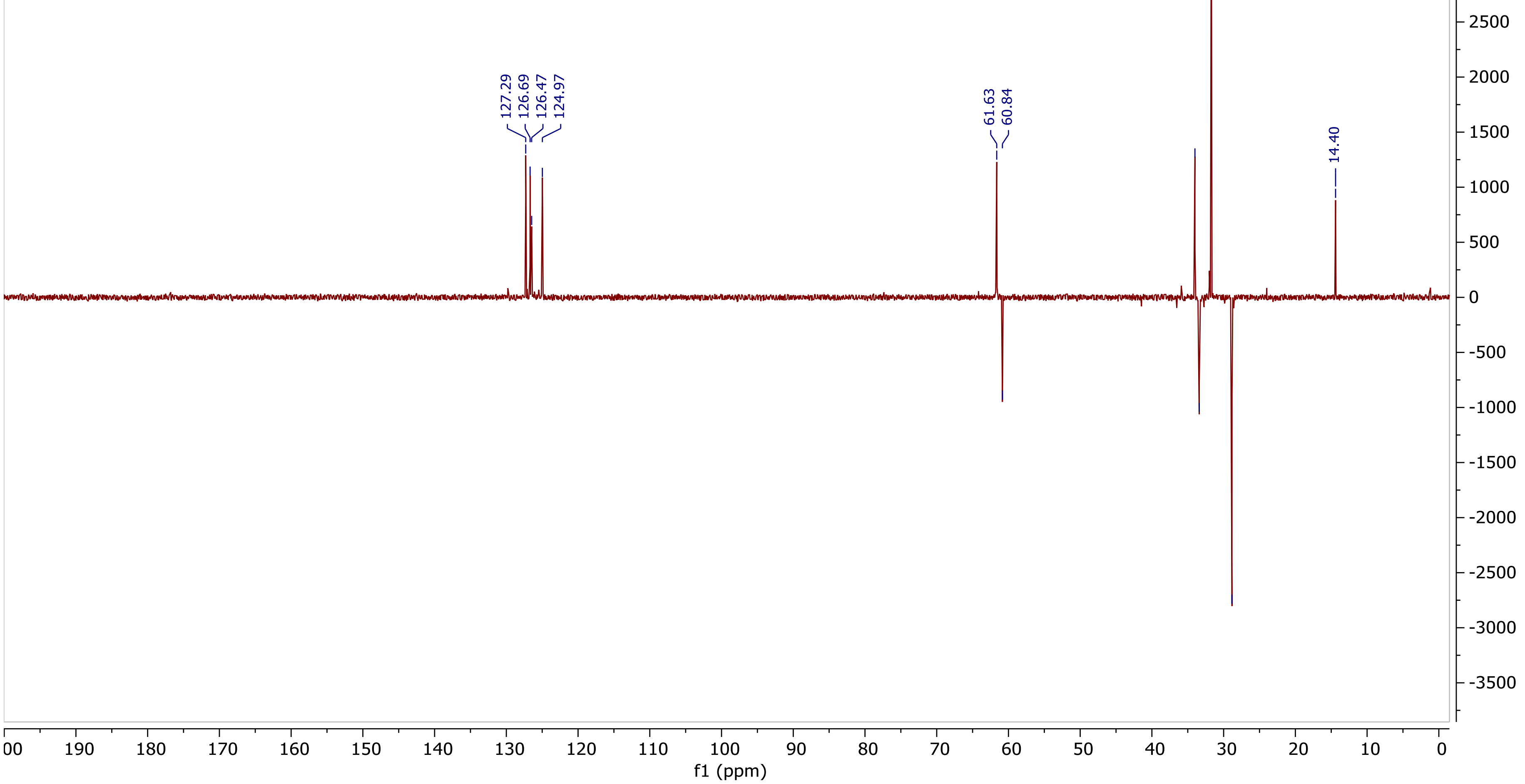

Ethyl 8-(4-methoxyphenyl)-8-azabicyclo[3.2.1]octane-3-carboxylate (**3eα**)

^{1}H, ^{1}H-COSY NMR (400 MHz, $CDCl_3$)

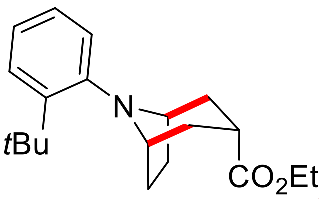

3e

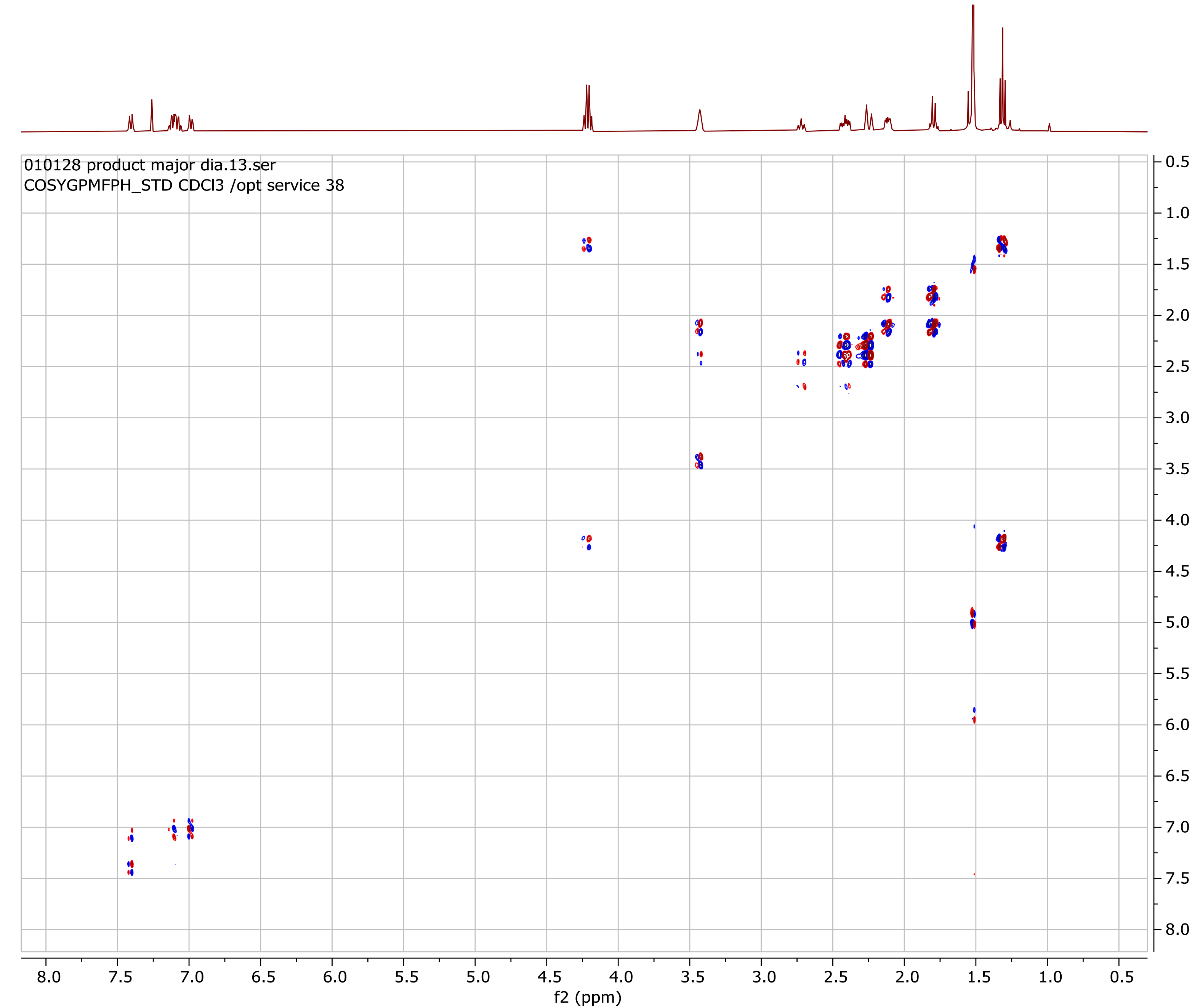

Ethyl 8-(4-methoxyphenyl)-8-azabicyclo[3.2.1]octane-3-carboxylate (**3eα**)

^{1}H, ^{13}C-HSQC NMR (400 MHz, $CDCl_3$)

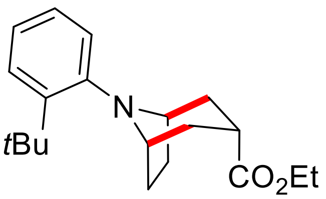

3e

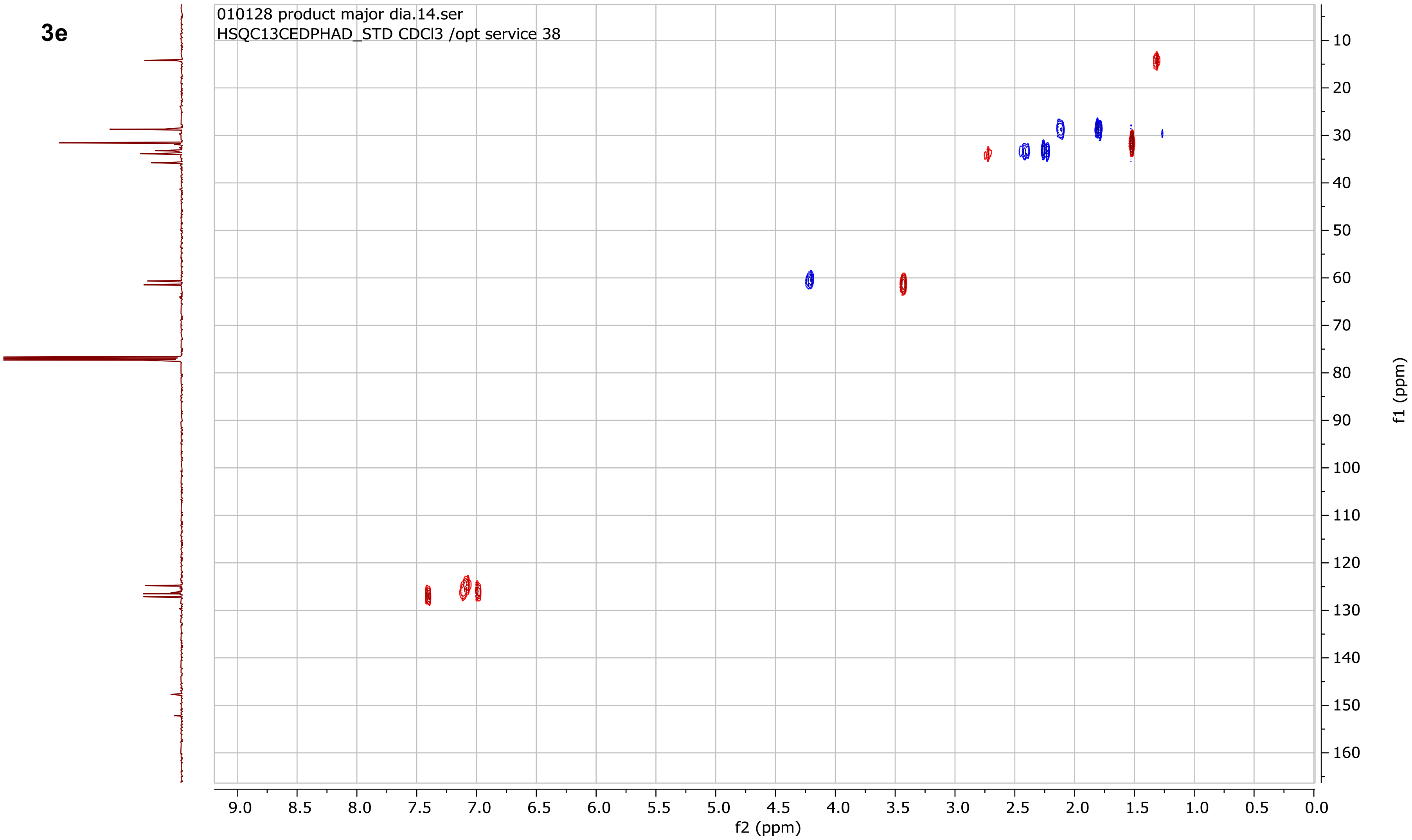

Ethyl 8-(4-methoxyphenyl)-8-azabicyclo[3.2.1]octane-3-carboxylate (**3eα**)

^{1}H, ^{13}C-HMBC NMR (300 MHz, $CDCl_3$)

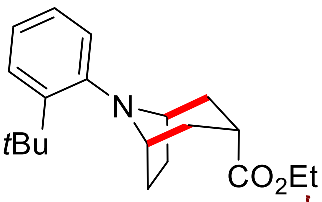

3e

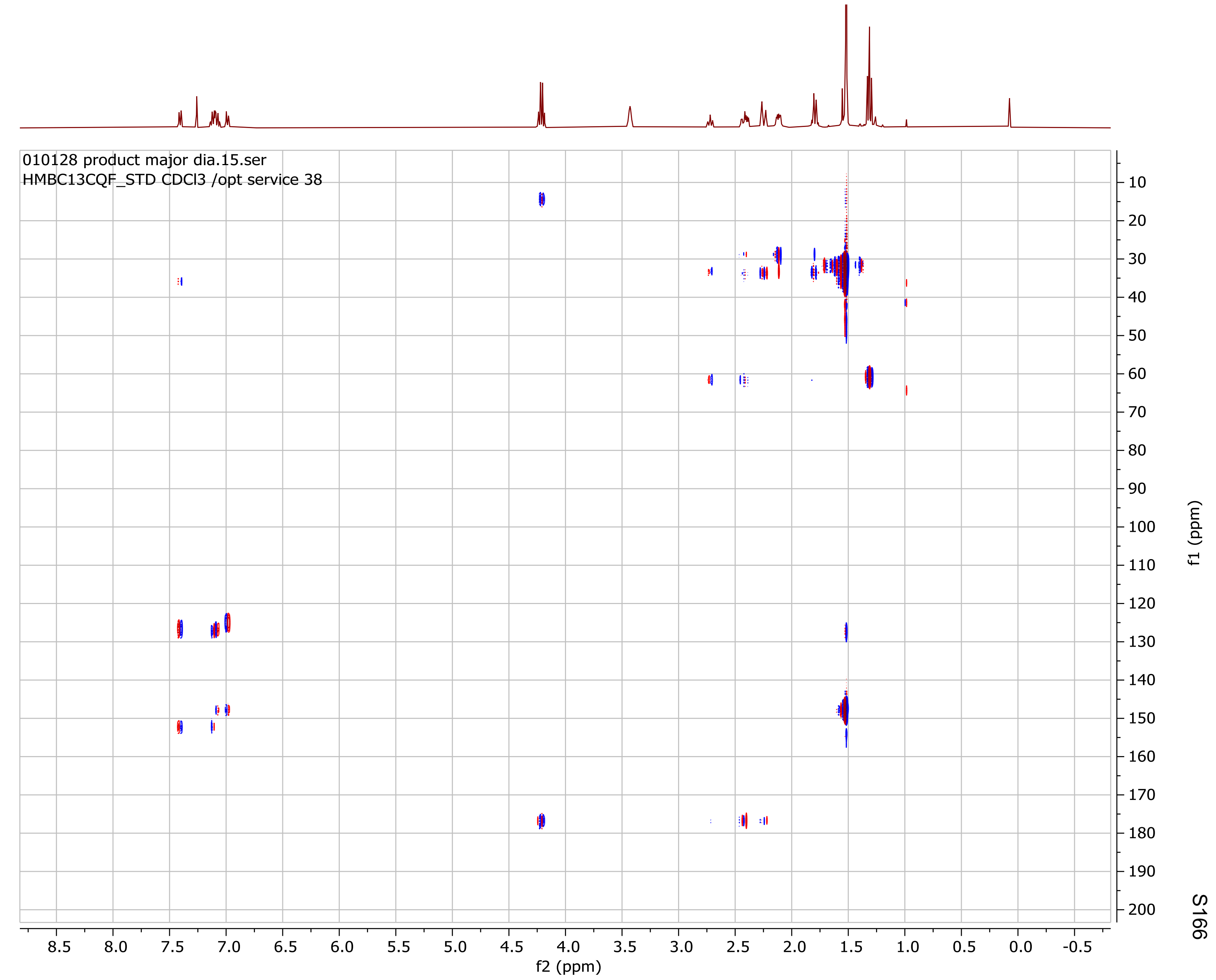

Ethyl 8-(4-methoxyphenyl)-8-azabicyclo[3.2.1]octane-3-carboxylate (**3eα**)

FT-IR, ATR-diamond

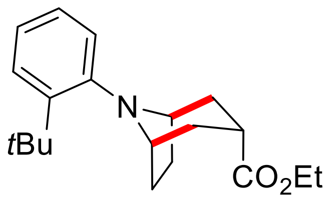

3e

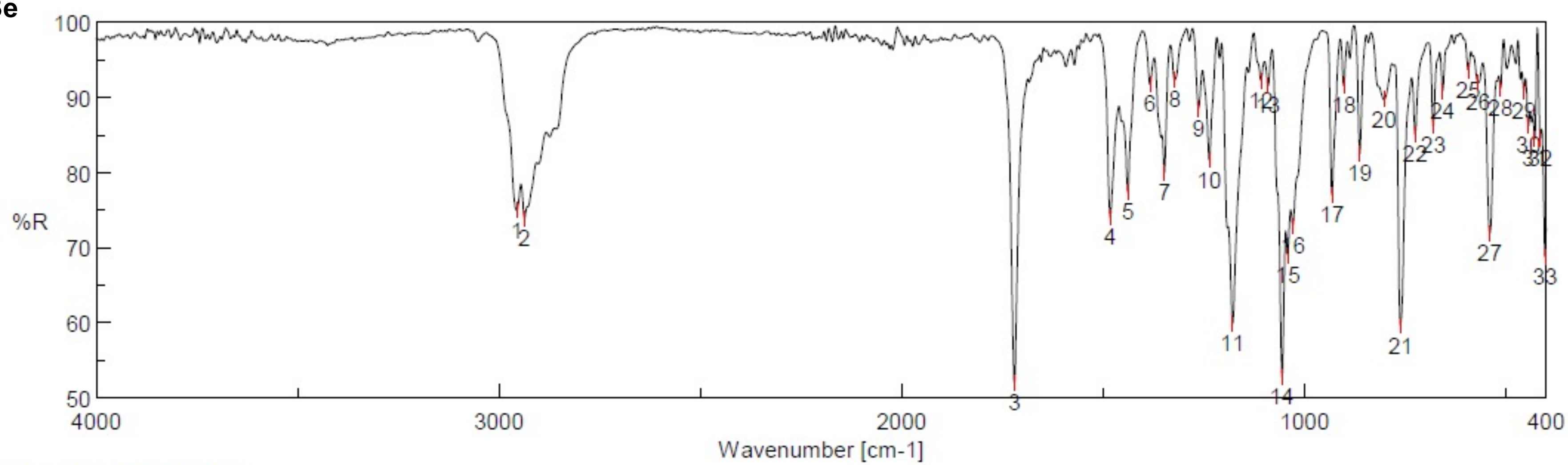

[Result of Peak Picking]

No.	Position	Intensity	No.	Position	Intensity	No.	Position	Intensity
1	2956.34	74.9479	2	2937.06	73.8853	3	1720.19	51.9227
4	1482.03	73.9532	5	1438.64	77.3709	6	1383.68	91.7249
7	1348	80.0039	8	1321.96	92.3093	9	1263.15	88.505
10	1236.15	81.6368	11	1178.29	59.8623	12	1107.9	92.261
13	1090.55	91.5461	14	1055.84	52.7669	15	1041.37	69.0082
16	1028.84	72.9374	17	931.45	76.9677	18	902.523	91.5387
19	862.989	82.5131	20	802.242	89.7133	21	761.744	59.564
22	725.104	85.0277	23	679.785	86.2427	24	656.643	90.8829
25	593.004	93.6207	26	568.898	91.9266	27	539.971	71.8974
28	512.972	91.2349	29	455.118	90.8773	30	443.547	86.284
31	428.12	84.4685	32	414.62	84.4741	33	402.085	68.7432

Ethyl 1-methyl-8-phenyl-8-azabicyclo[3.2.1]octane-3-carboxylate (**3f**) **α/β 8:1**

^{1}H-NMR (400 MHz, $CDCl_3$)

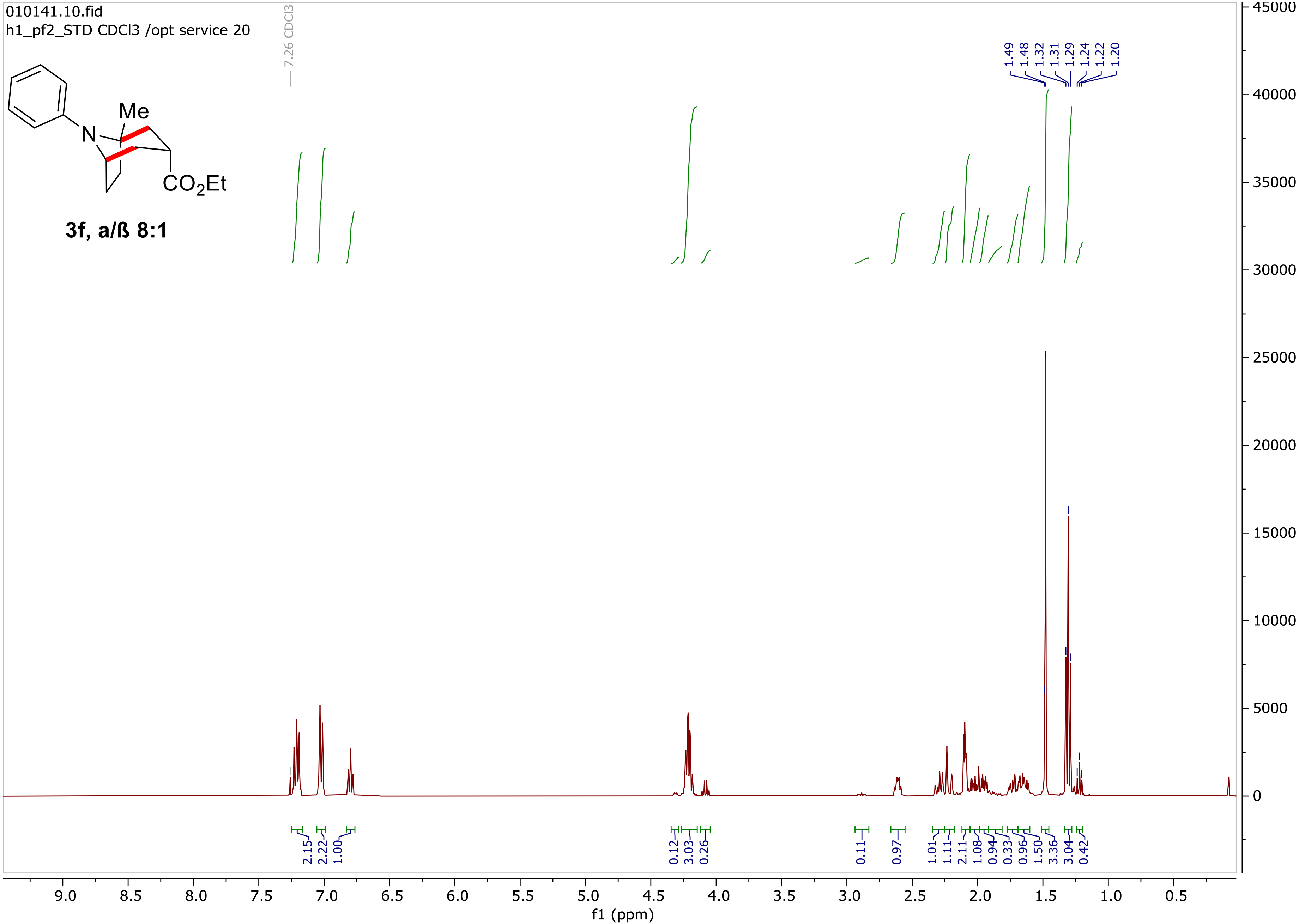

Ethyl 1-methyl-8-phenyl-8-azabicyclo[3.2.1]octane-3-carboxylate (**3f**) **α/β 8:1**

^{1}H-NMR (400 MHz, $CDCl_3$)

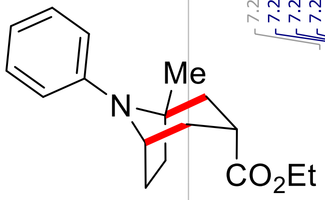

3f, a/ß 8:1

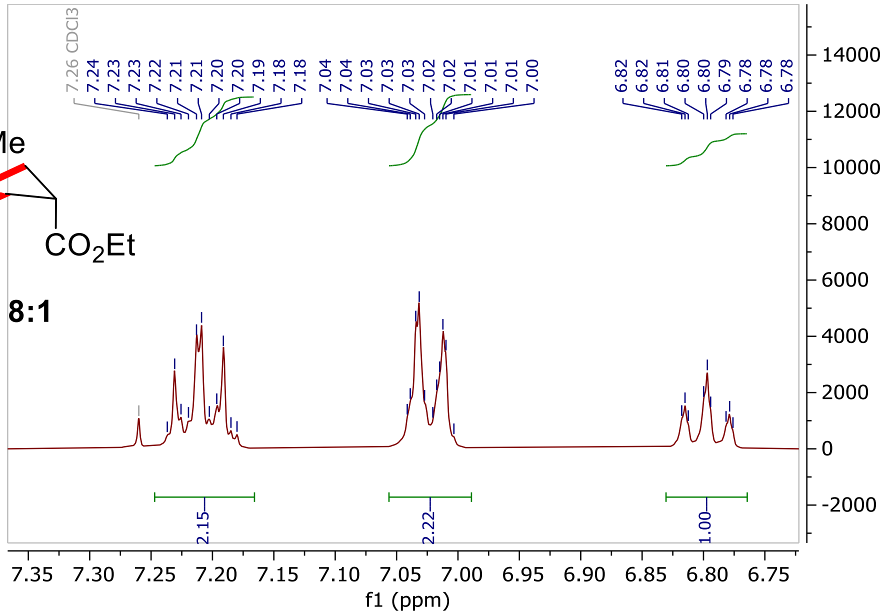

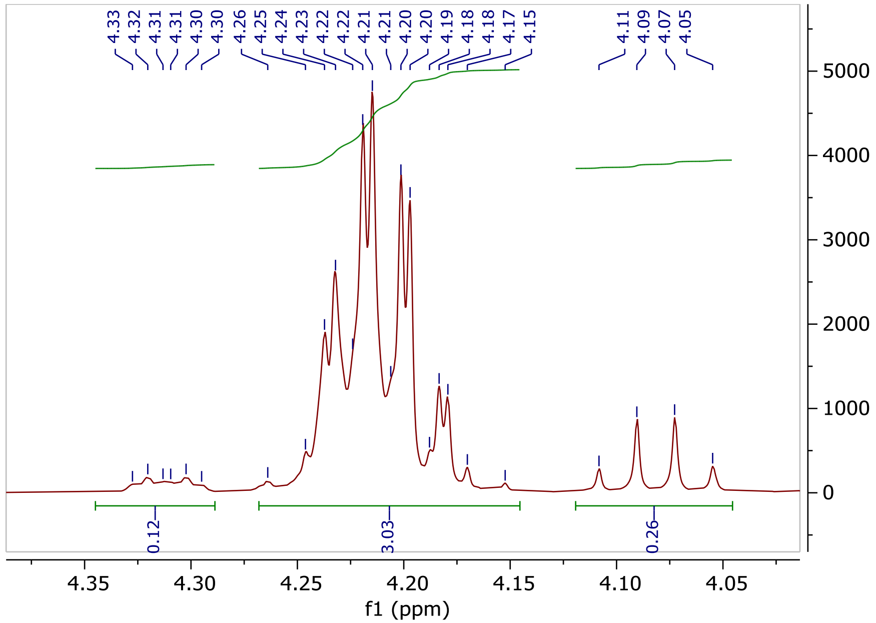

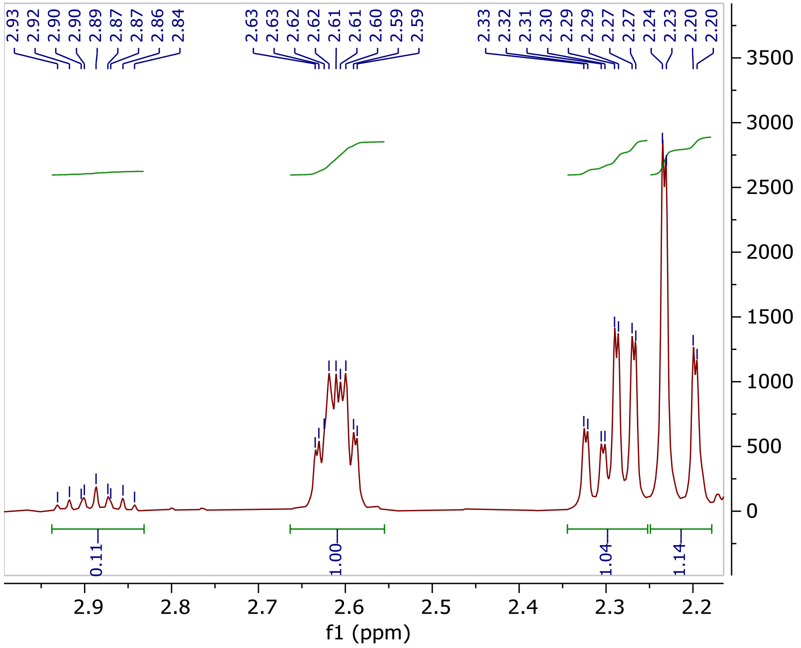

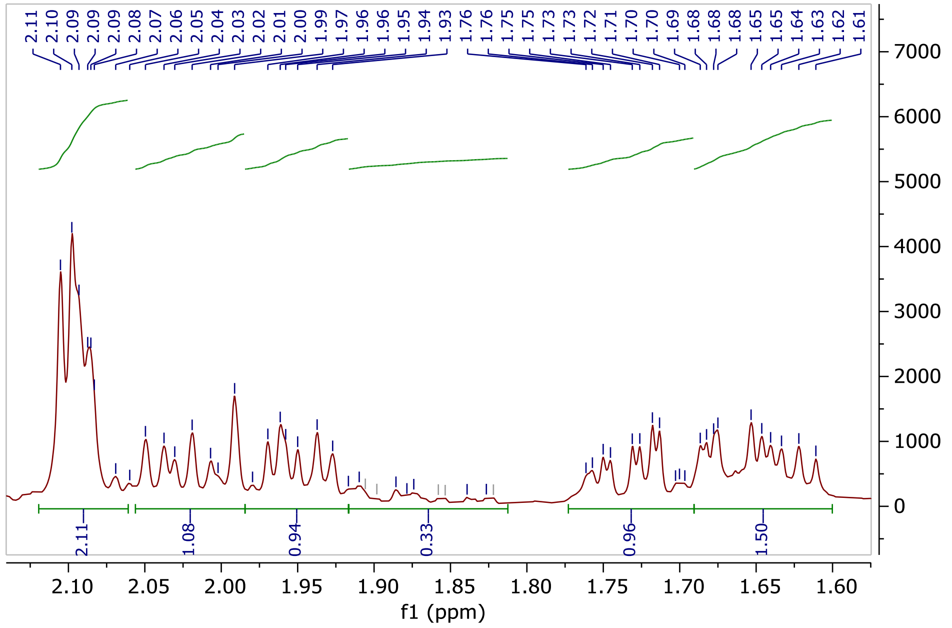

Ethyl 1-methyl-8-phenyl-8-azabicyclo[3.2.1]octane-3-carboxylate (**3f**) **α/β 8:1**

^{13C}-NMR (101 MHz, $CDCl_3$)

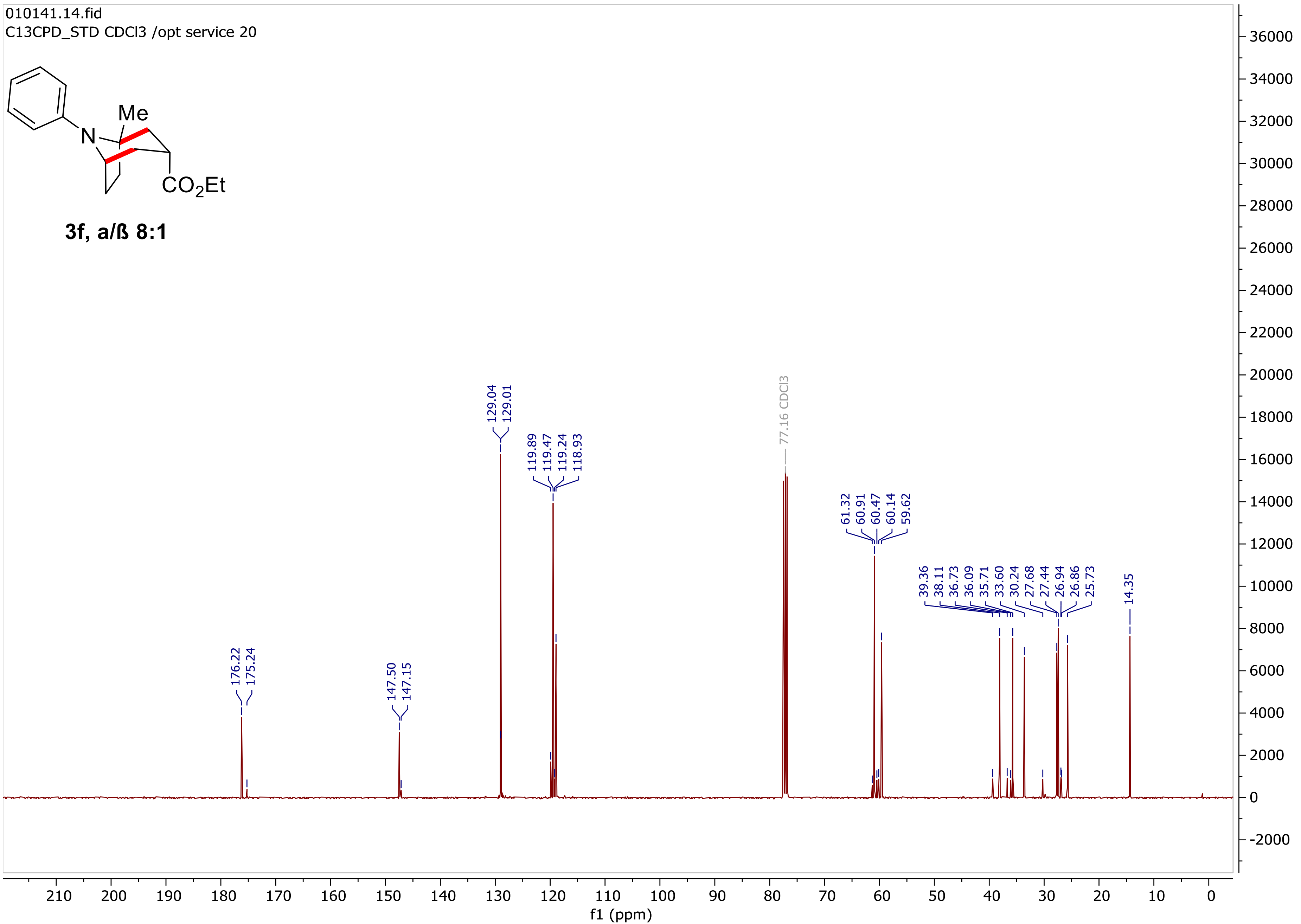

Ethyl 1-methyl-8-phenyl-8-azabicyclo[3.2.1]octane-3-carboxylate (**3f**) **α/β 8:1**

^{13}C-NMR (101 MHz, $CDCl_3$)

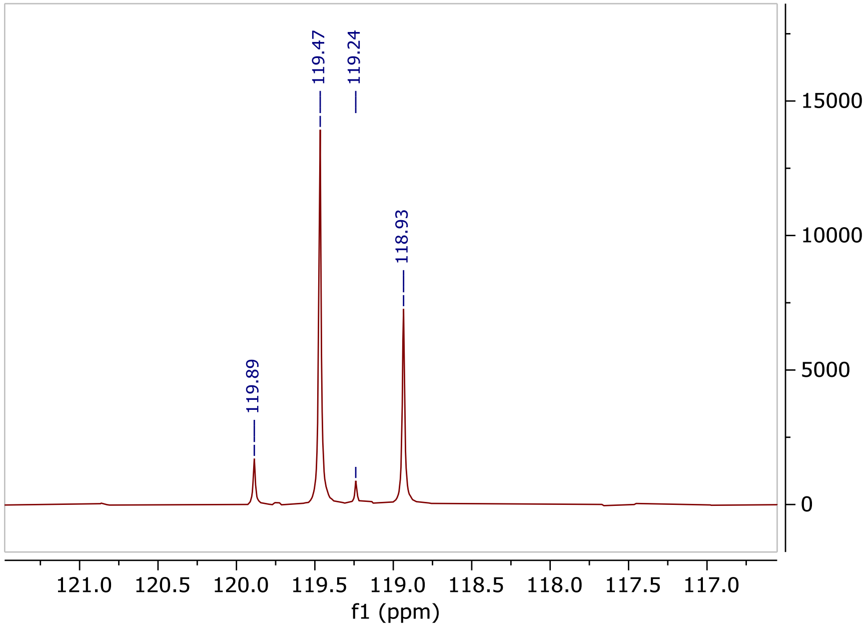

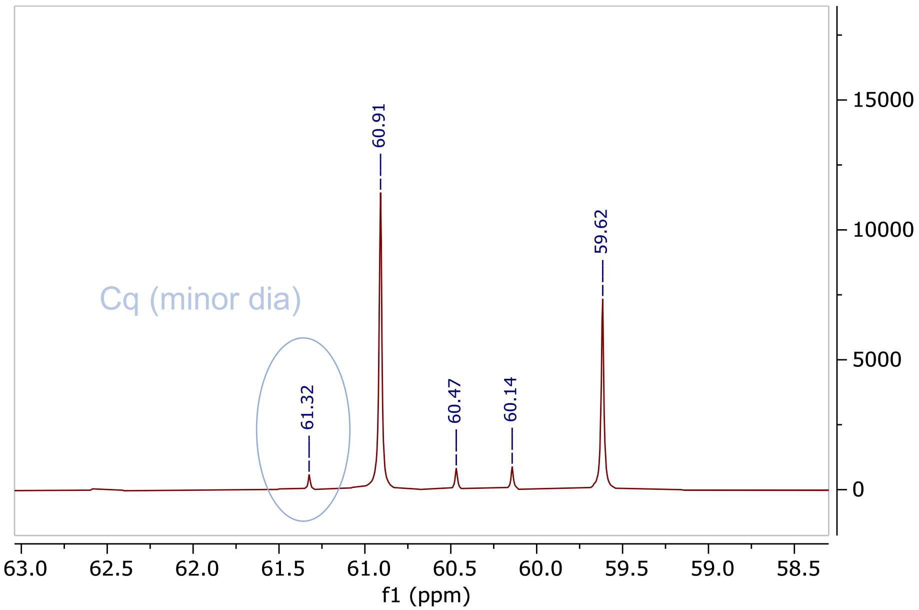

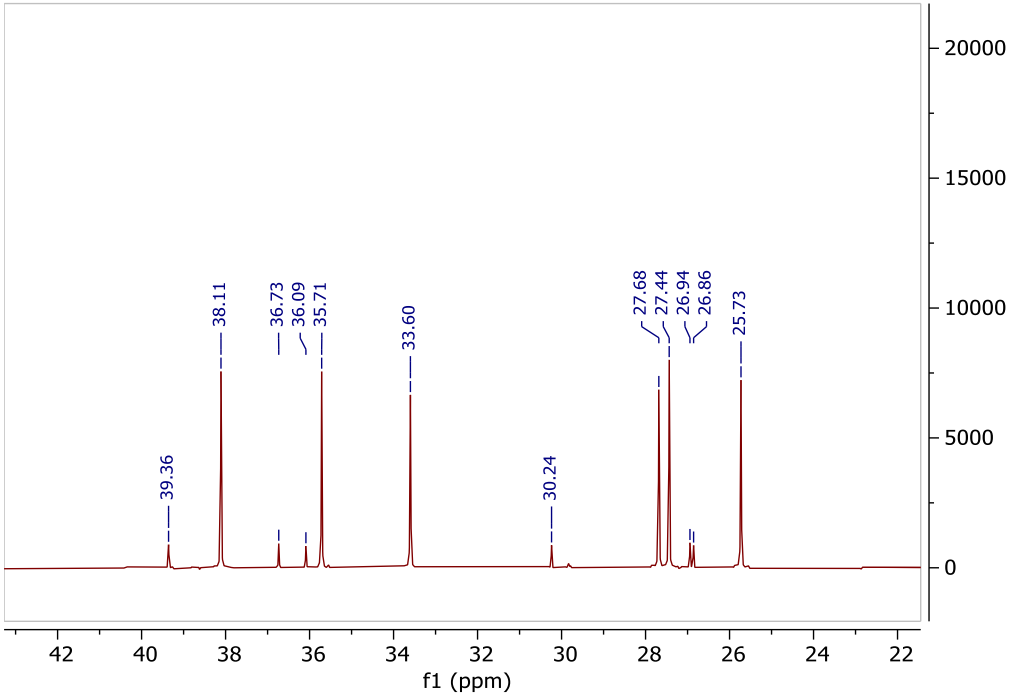

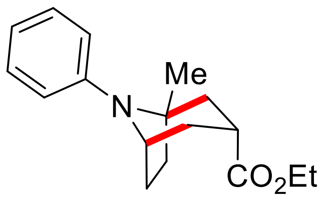

3f, a/ß 8:1

Ethyl 1-methyl-8-phenyl-8-azabicyclo[3.2.1]octane-3-carboxylate (**3f**) **α/β 8:1**

^{13}C-NMR (101 MHz, $CDCl_3$)

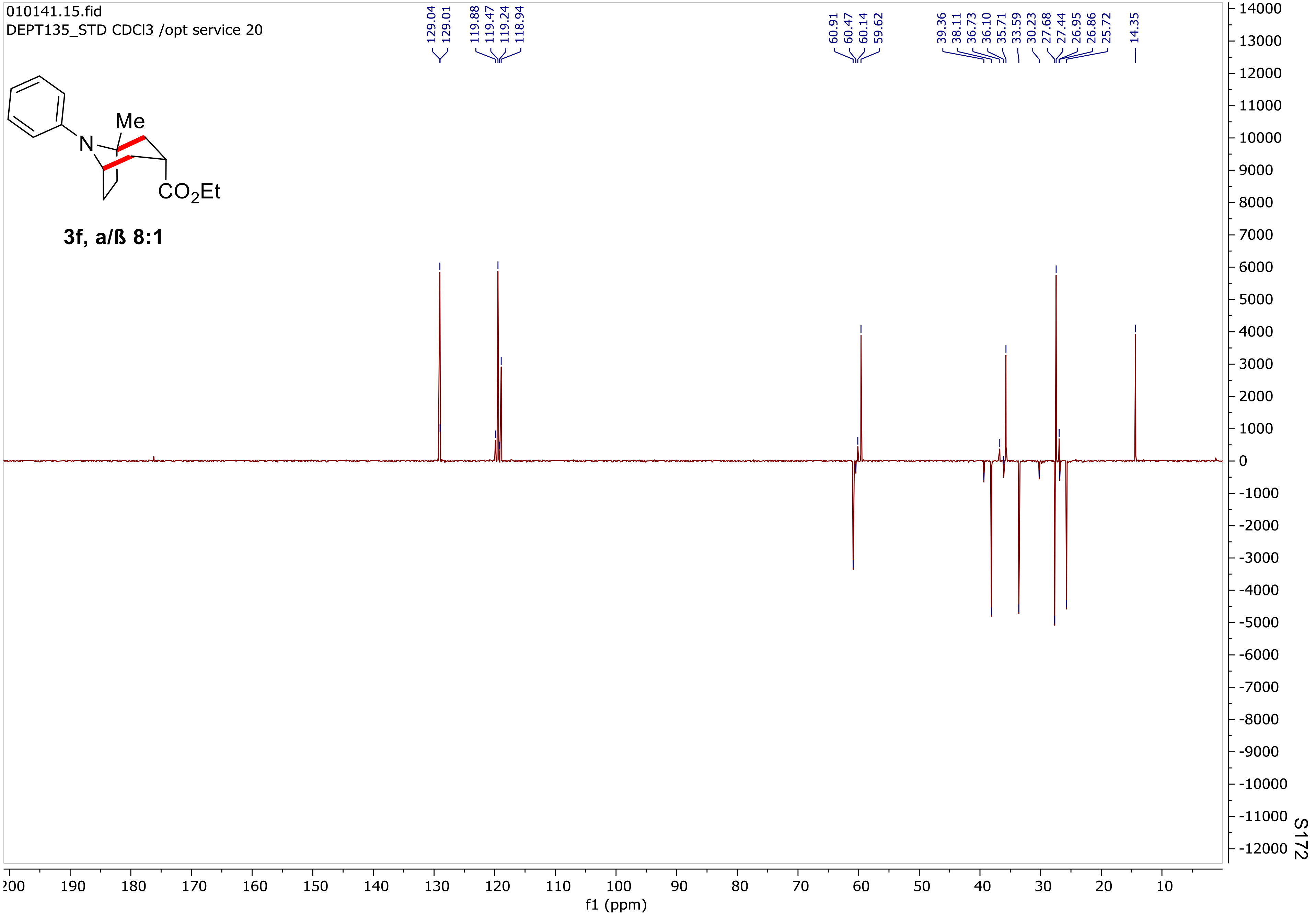

3f, a/ß 8:1

Ethyl 1-methyl-8-phenyl-8-azabicyclo[3.2.1]octane-3-carboxylate (**3f**) **α/β 8:1**

^{13}C-NMR (101 MHz, $CDCl_3$)

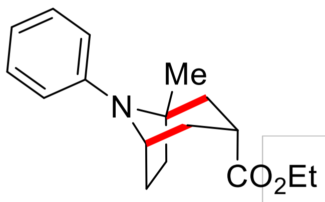

3f, a/ß 8:1

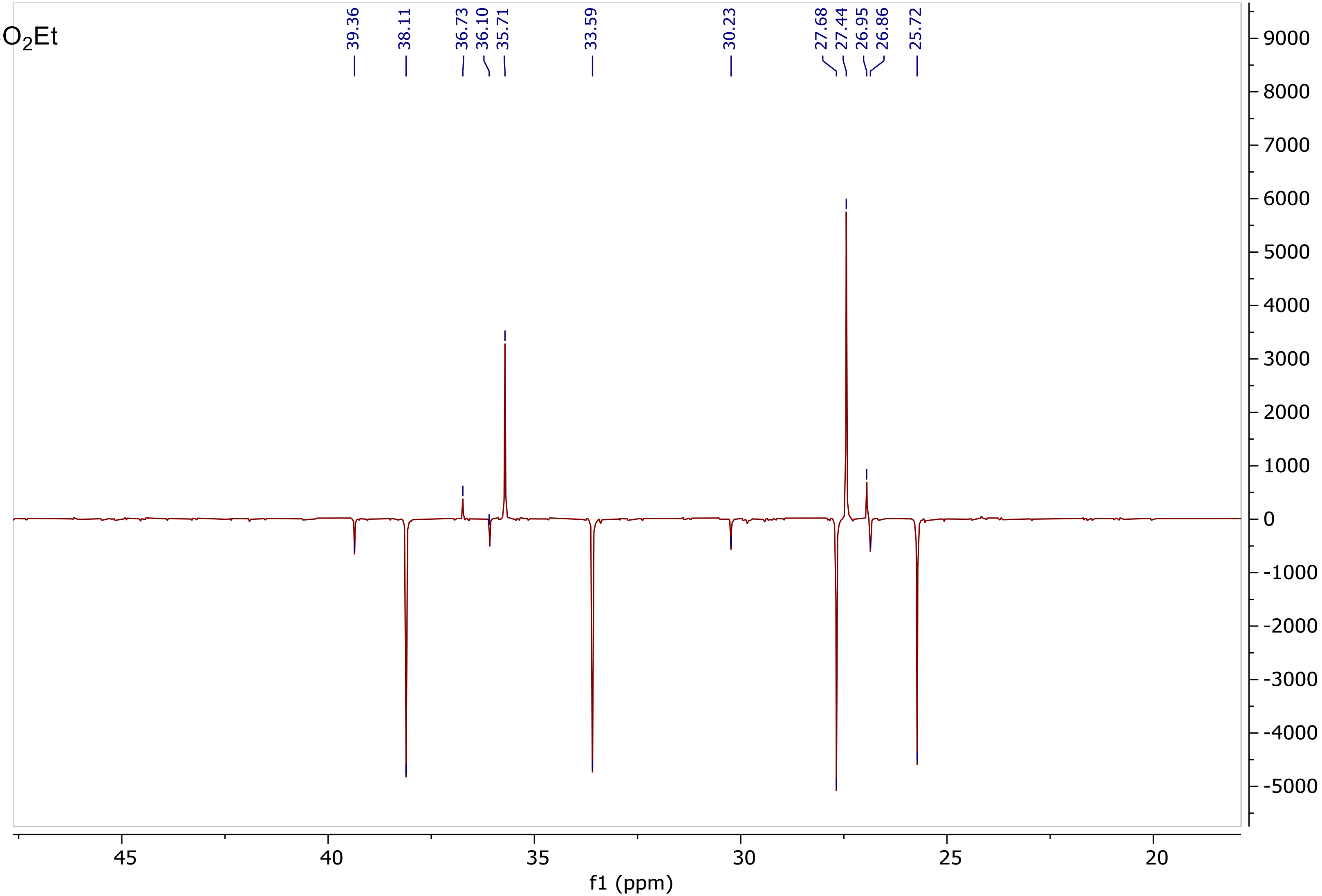

Ethyl 1-methyl-8-phenyl-8-azabicyclo[3.2.1]octane-3-carboxylate (**3f**) **α/β 8:1**

^{1}H, ^{1}H-COSY NMR (400 MHz, $CDCl_3$)

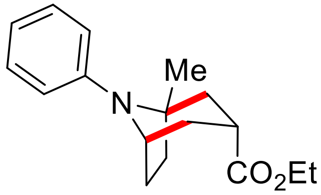

3f, a/ß 8:1

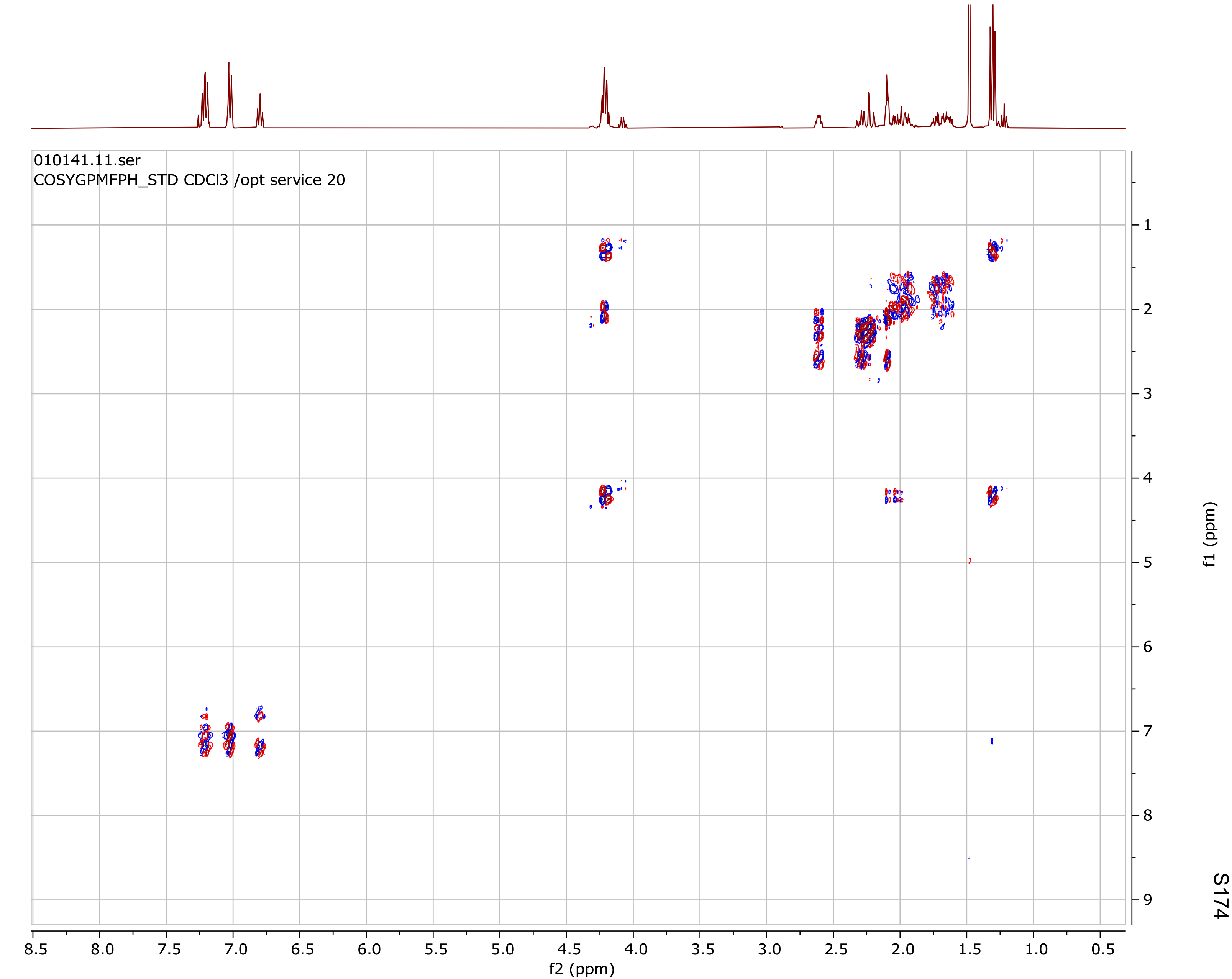

Ethyl 1-methyl-8-phenyl-8-azabicyclo[3.2.1]octane-3-carboxylate (**3f**) **α/β 8:1**

^{1}H, ^{13}C-HSQC NMR (300 MHz, $CDCl_3$)

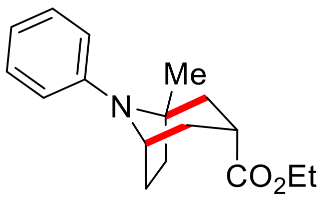

3f, a/ß 8:1

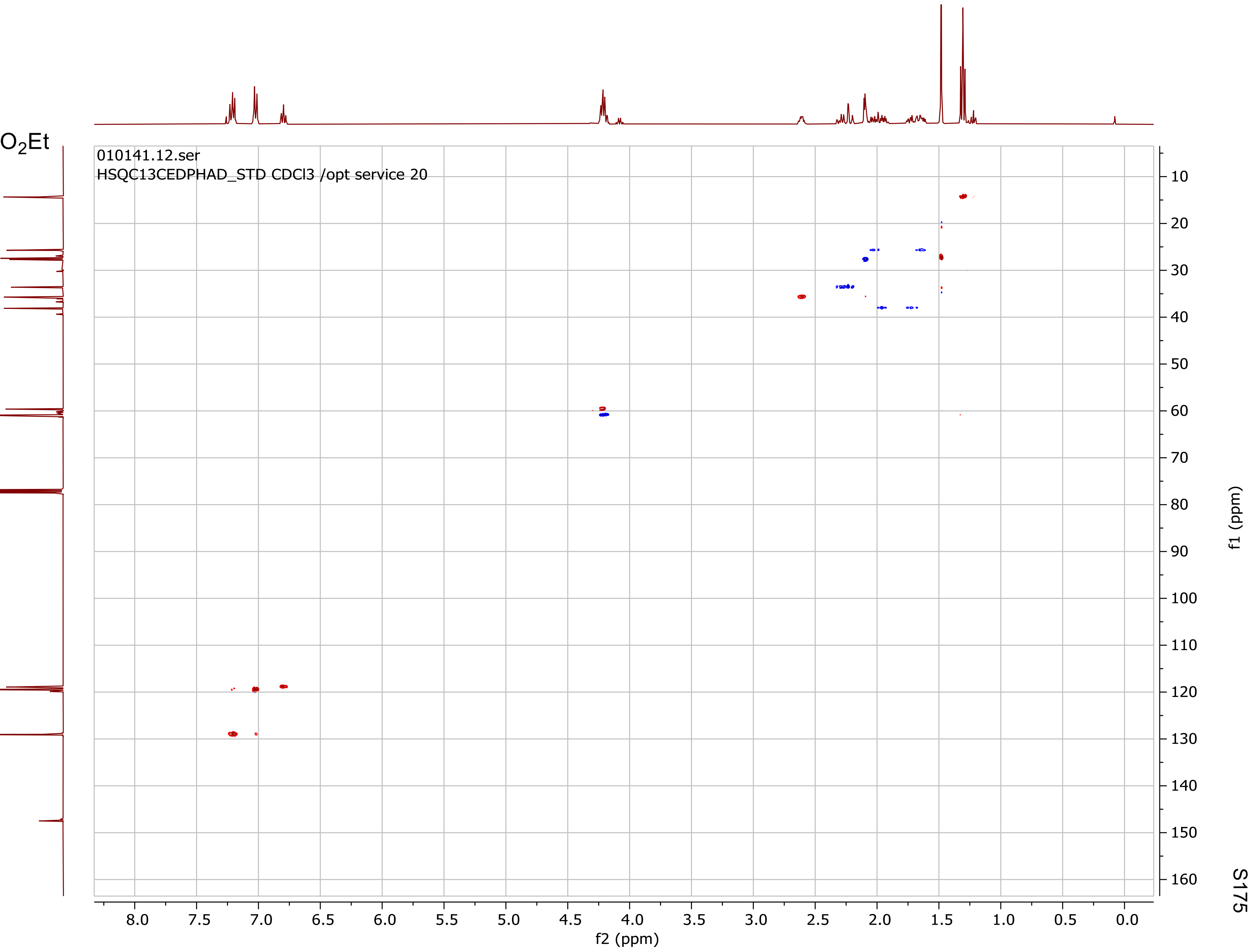

Ethyl 1-methyl-8-phenyl-8-azabicyclo[3.2.1]octane-3-carboxylate (**3f**) **α/β 8:1**

^{1}H, ^{13}C-HSQC NMR (300 MHz, $CDCl_3$)

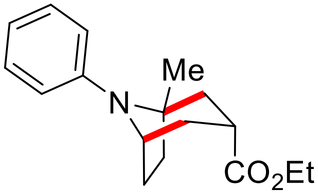

3f, a/ß 8:1

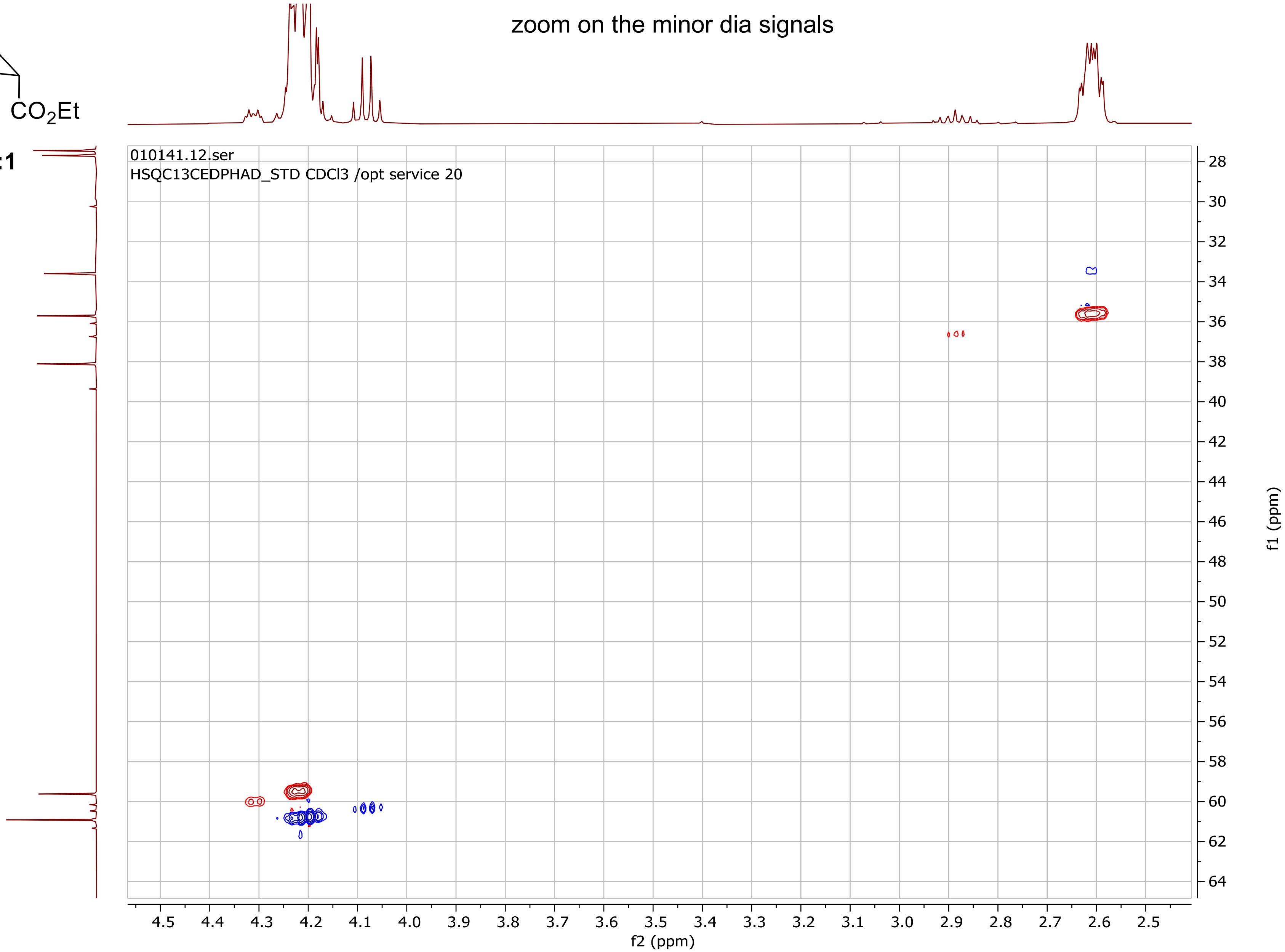

Ethyl 1-methyl-8-phenyl-8-azabicyclo[3.2.1]octane-3-carboxylate (**3f**) **α/β 8:1**

^1H, ^{13}C-HMBC NMR (300 MHz, $CDCl_3$)

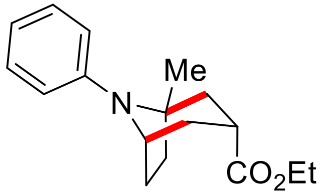

3f, a/ß 8:1

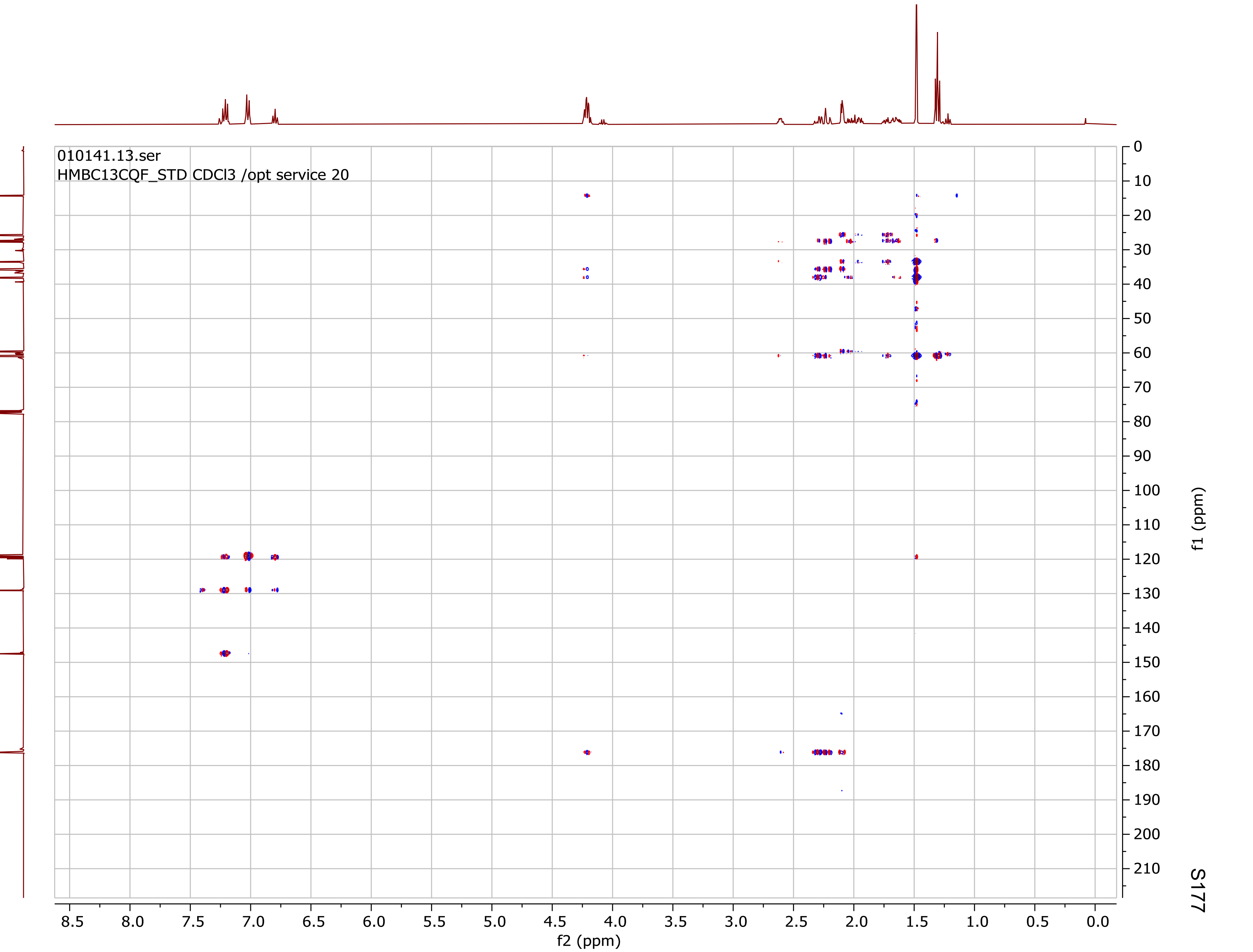

Ethyl 1-methyl-8-phenyl-8-azabicyclo[3.2.1]octane-3-carboxylate (**3f**) **α/β 8:1**

^{1}H, ^{13}C-HMBC NMR (300 MHz, $CDCl_3$)

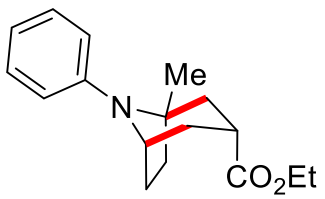

3f, a/ß 8:1

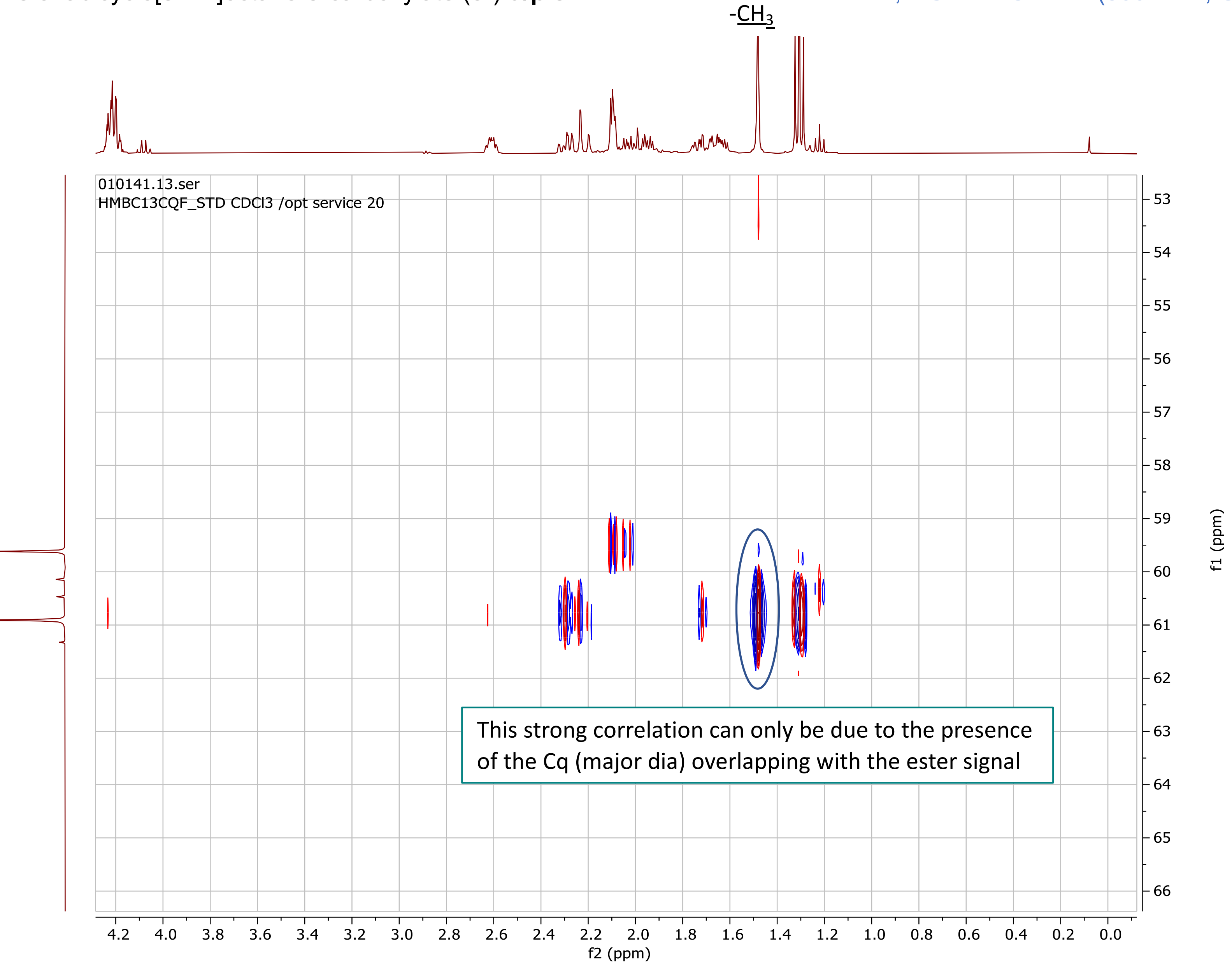

Ethyl 1-methyl-8-phenyl-8-azabicyclo[3.2.1]octane-3-carboxylate (**3f**) **α/β 8:1**

FT-IR, ATR-diamond

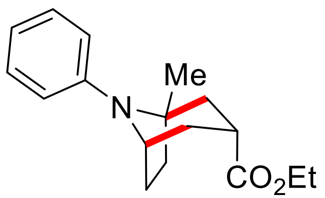

3f, a/ß 8:1

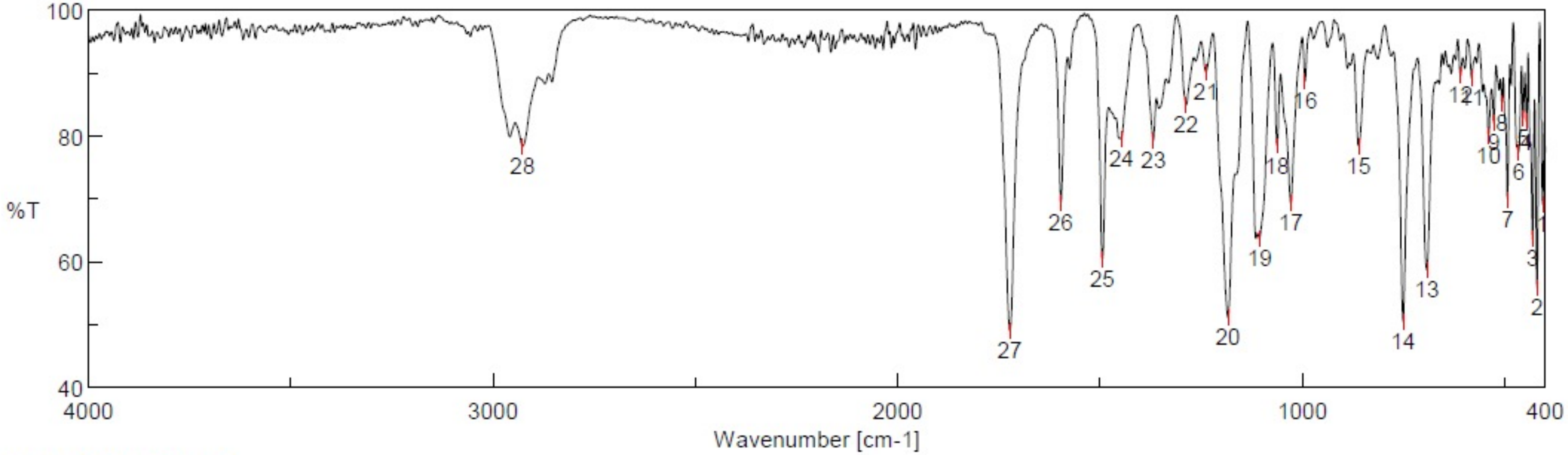

[Result of Peak Picking]

No.	Position	Intensity	No.	Position	Intensity	No.	Position	Intensity
1	405.942	69	2	420.406	56	3	431.012	64
4	445.476	82	5	454.154	83	6	465.725	77
7	492.723	70	8	507.187	85	9	526.471	82
10	539.971	80	11	581.433	89	12	609.396	90
13	692.32	59	14	751.138	50	15	861.06	78
16	993.16	89	17	1028.84	69	18	1062.59	78
19	1105.98	64	20	1184.08	51	21	1238.08	90
22	1287.25	85	23	1368.25	79	24	1447.31	80
25	1494.56	60	26	1596.77	69	27	1723.09	49
28	2927.41	78						

Ethyl 9-phenyl-9-azabicyclo[3.3.1]nonane-3-carboxylate (**8a**) **α/β >20:1** on 0.2 mmol scale

^{1}H-NMR (400 MHz, $CDCl_3$)

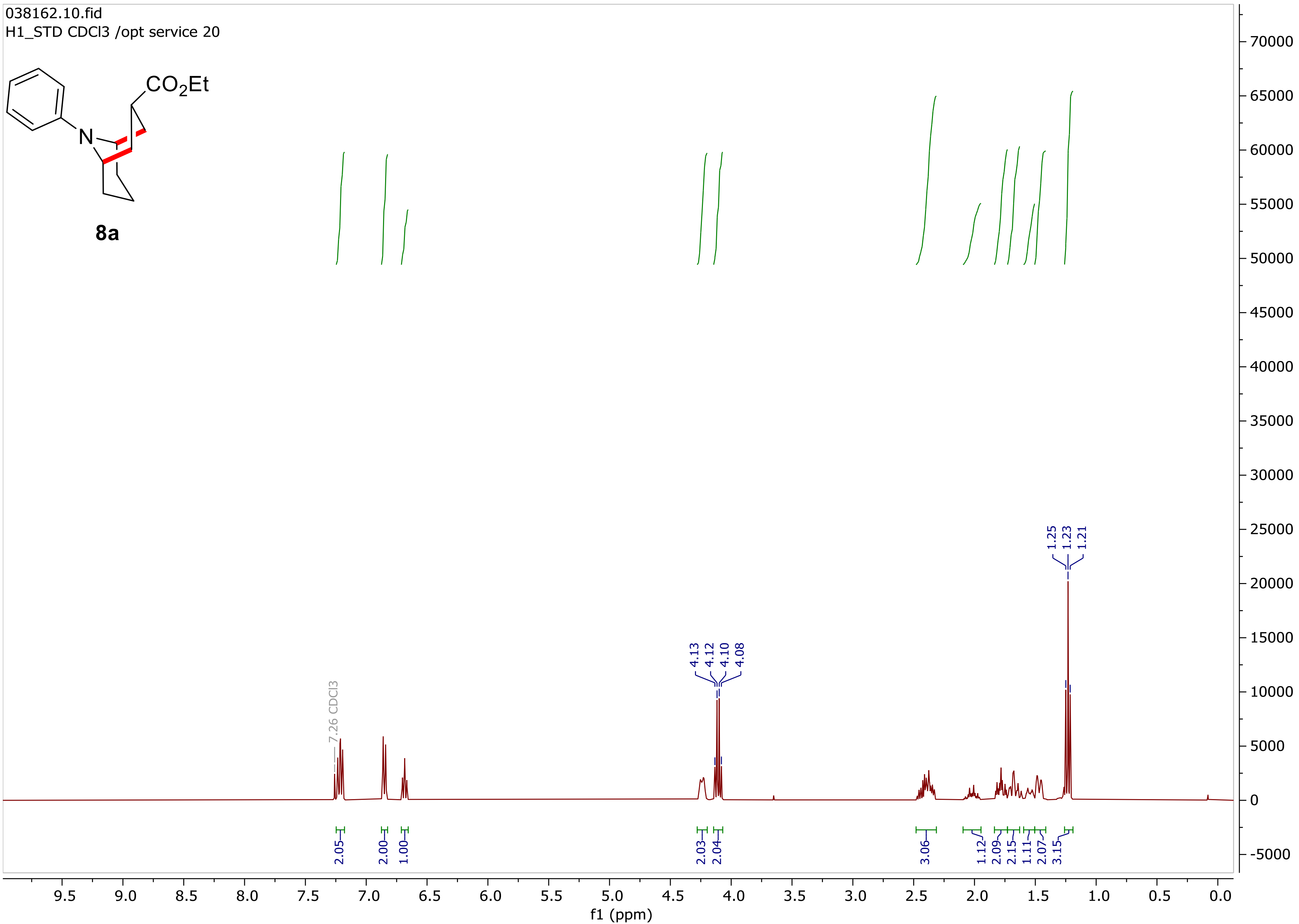

Ethyl 9-phenyl-9-azabicyclo[3.3.1]nonane-3-carboxylate (**8a**) **α/β 92:8** on 3 mmol scale

^{1}H-NMR (300 MHz, $CDCl_3$)

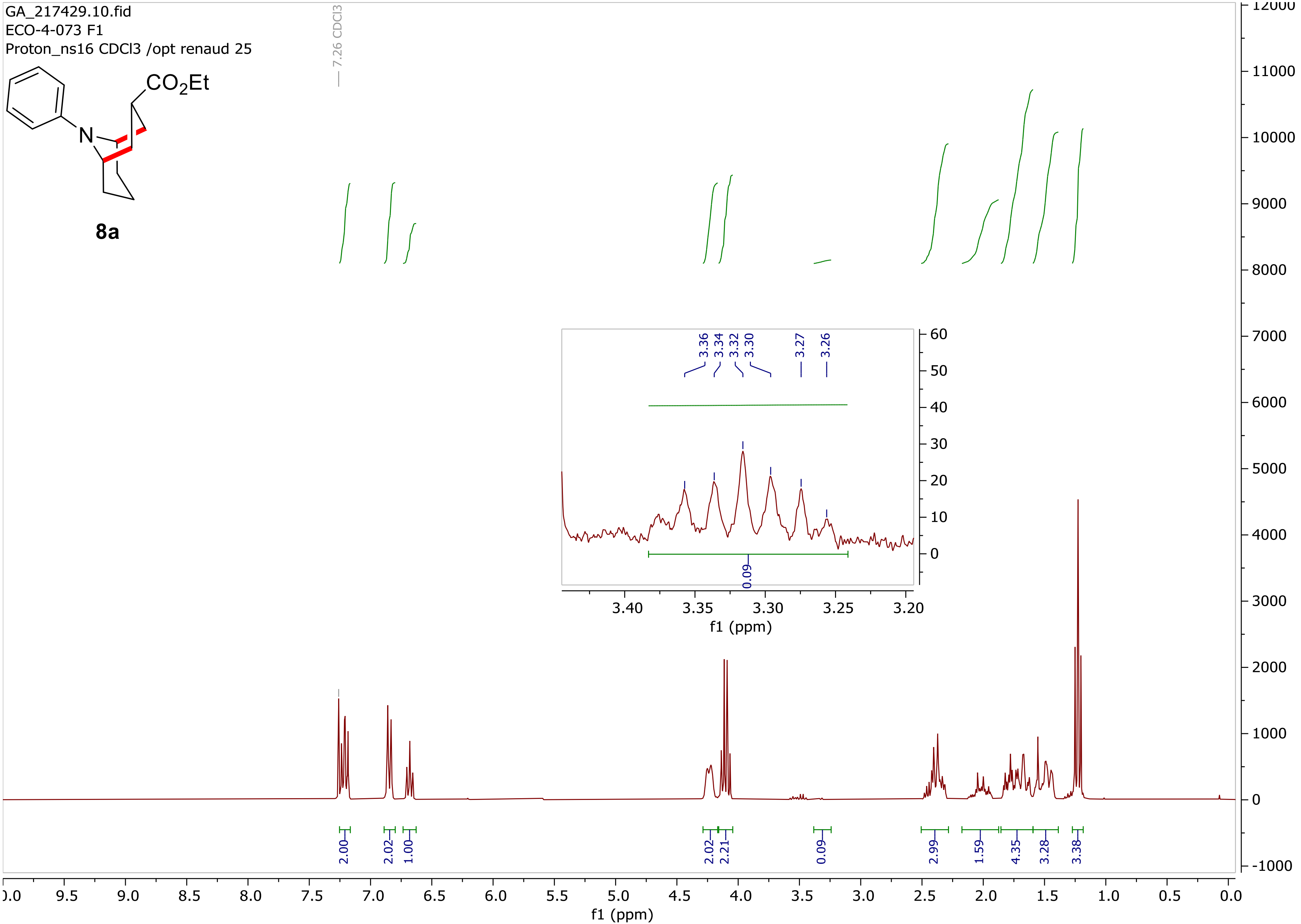

Ethyl 9-phenyl-9-azabicyclo[3.3.1]nonane-3-carboxylate (**8aα**)

^{1}H-NMR (400 MHz, $CDCl_3$)

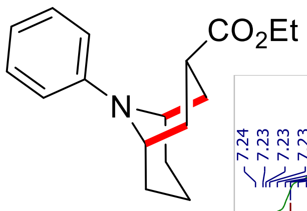

8a

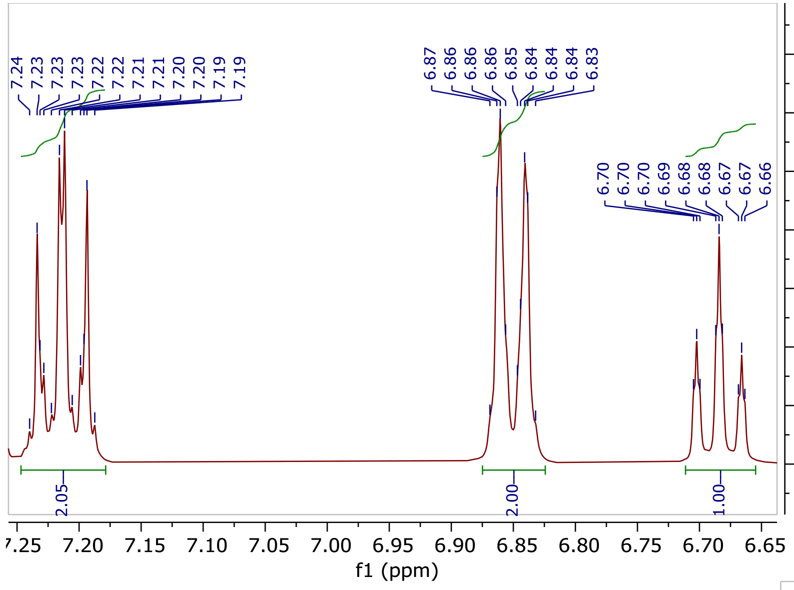

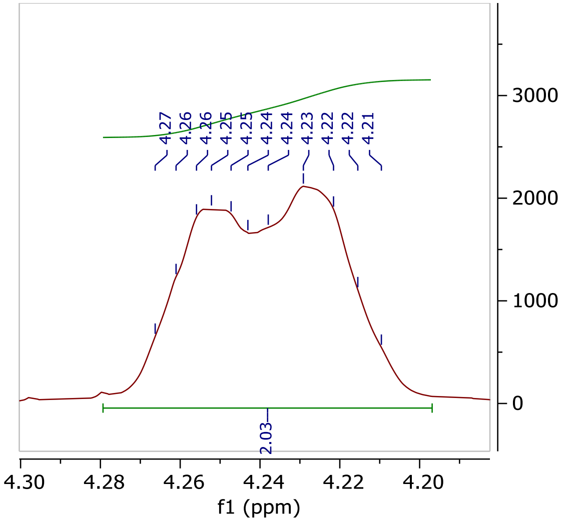

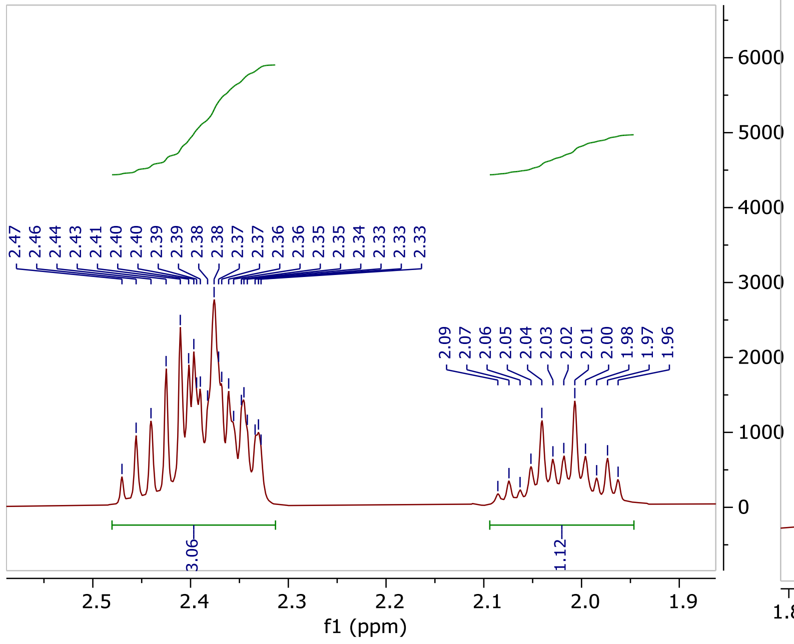

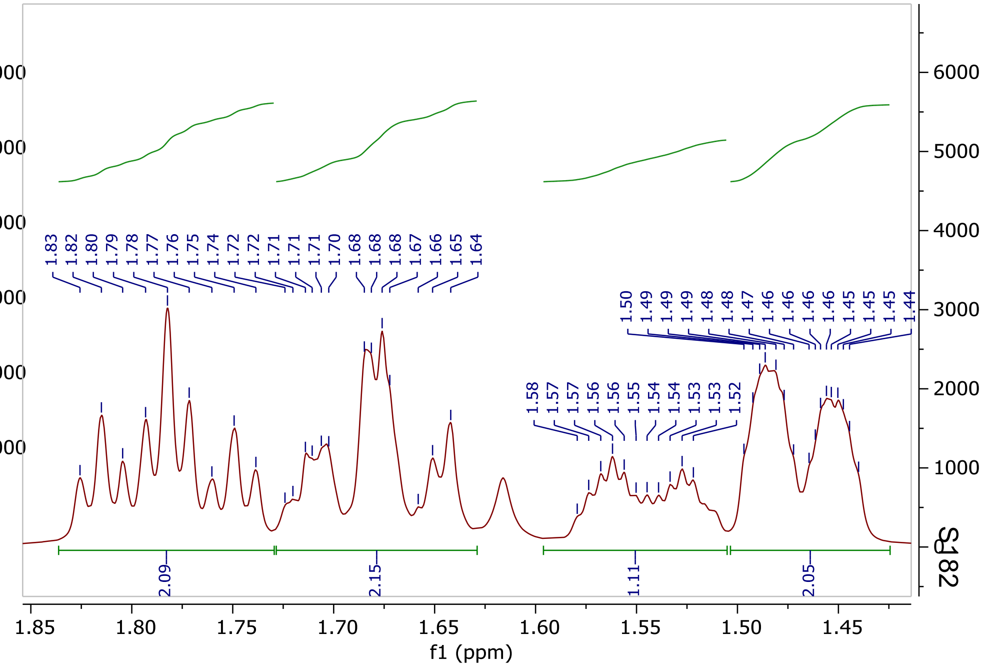

Ethyl 9-phenyl-9-azabicyclo[3.3.1]nonane-3-carboxylate (**8aα**)

^{13}C-NMR (75 MHz, $CDCl_3$)

GA_210780.10.fid
ECO-3-043 carac
Carbon_ns512 CDCl3 /opt service 22

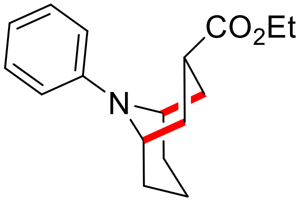

8a

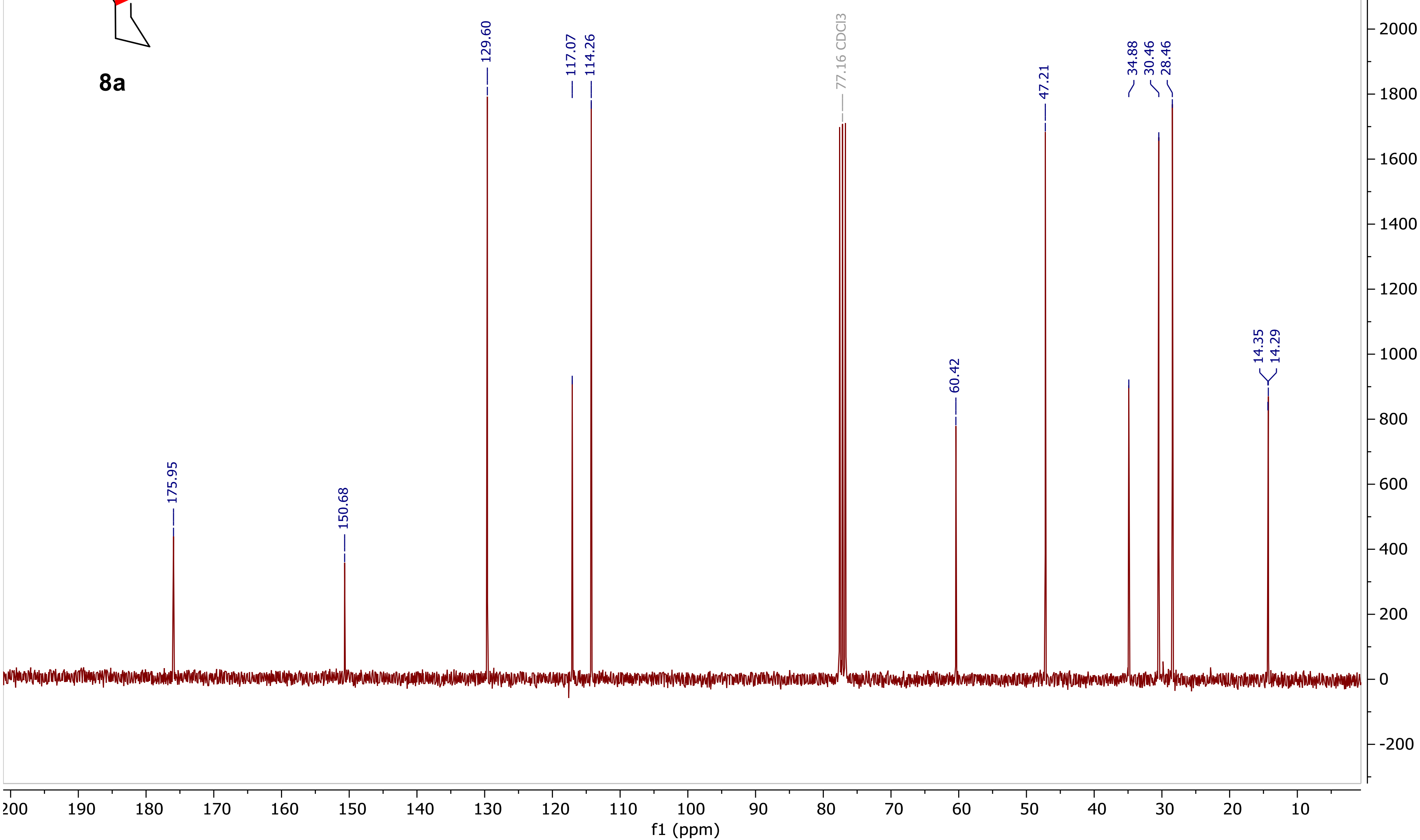

Ethyl 9-phenyl-9-azabicyclo[3.3.1]nonane-3-carboxylate (**8aα**)

^{13}C-NMR (75 MHz, $CDCl_3$)

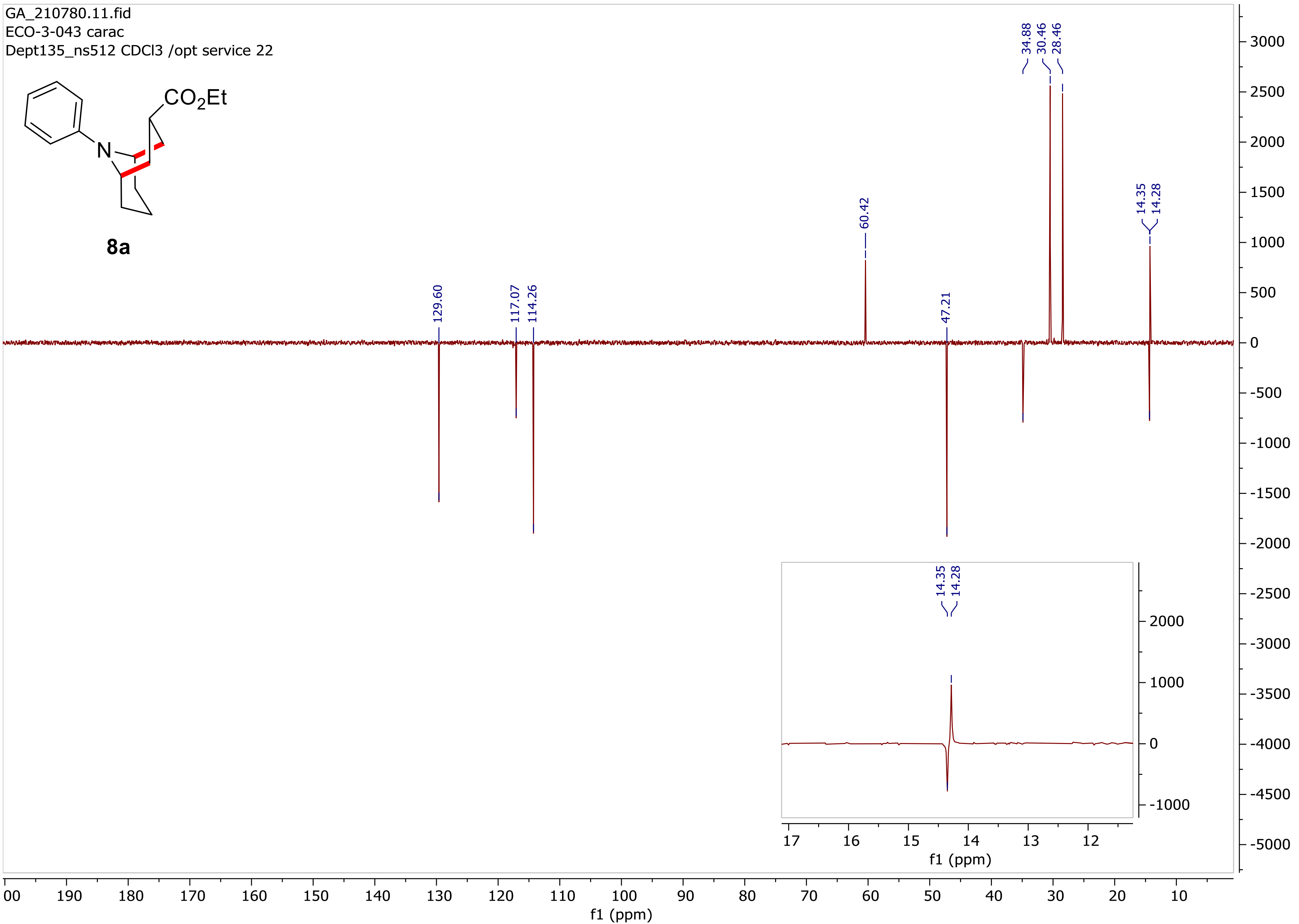

Ethyl 9-phenyl-9-azabicyclo[3.3.1]nonane-3-carboxylate (**8aα**) ^{1}H, ^{1}H-COSY NMR (400 MHz, $CDCl_3$)

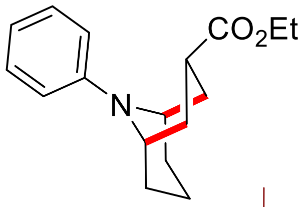

8a

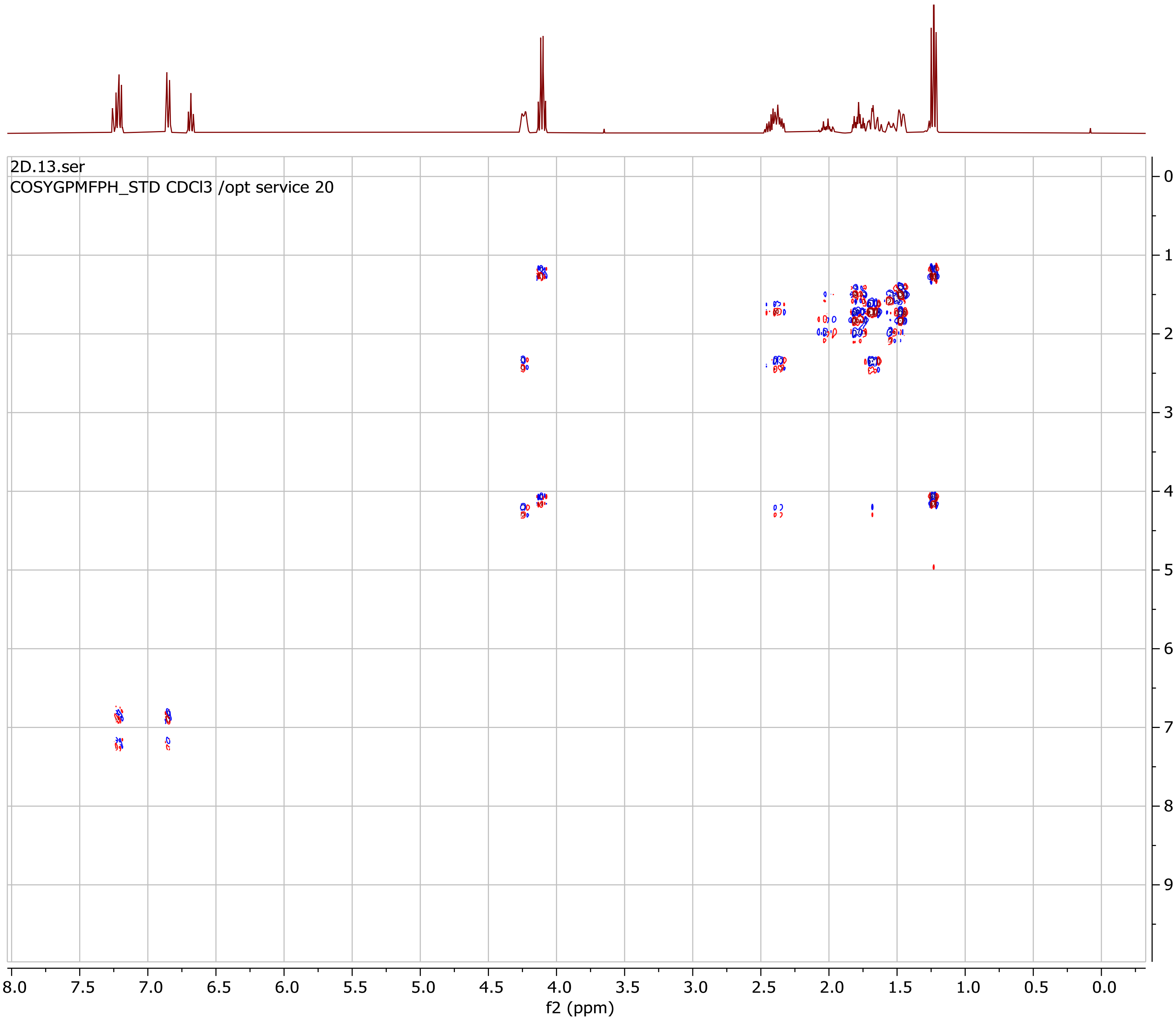

Ethyl 9-phenyl-9-azabicyclo[3.3.1]nonane-3-carboxylate (**8aα**)

^{1}H, ^{13}C-HSQC NMR (400 MHz, $CDCl_3$)

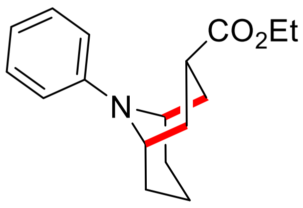

8a

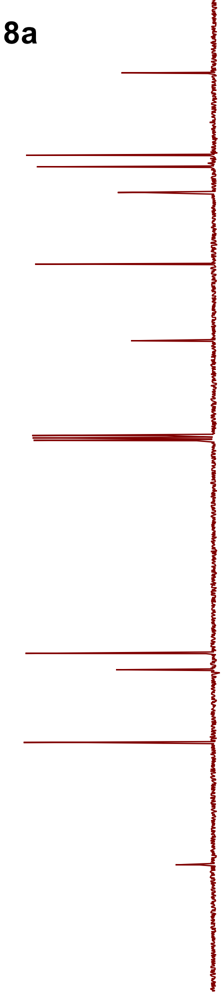

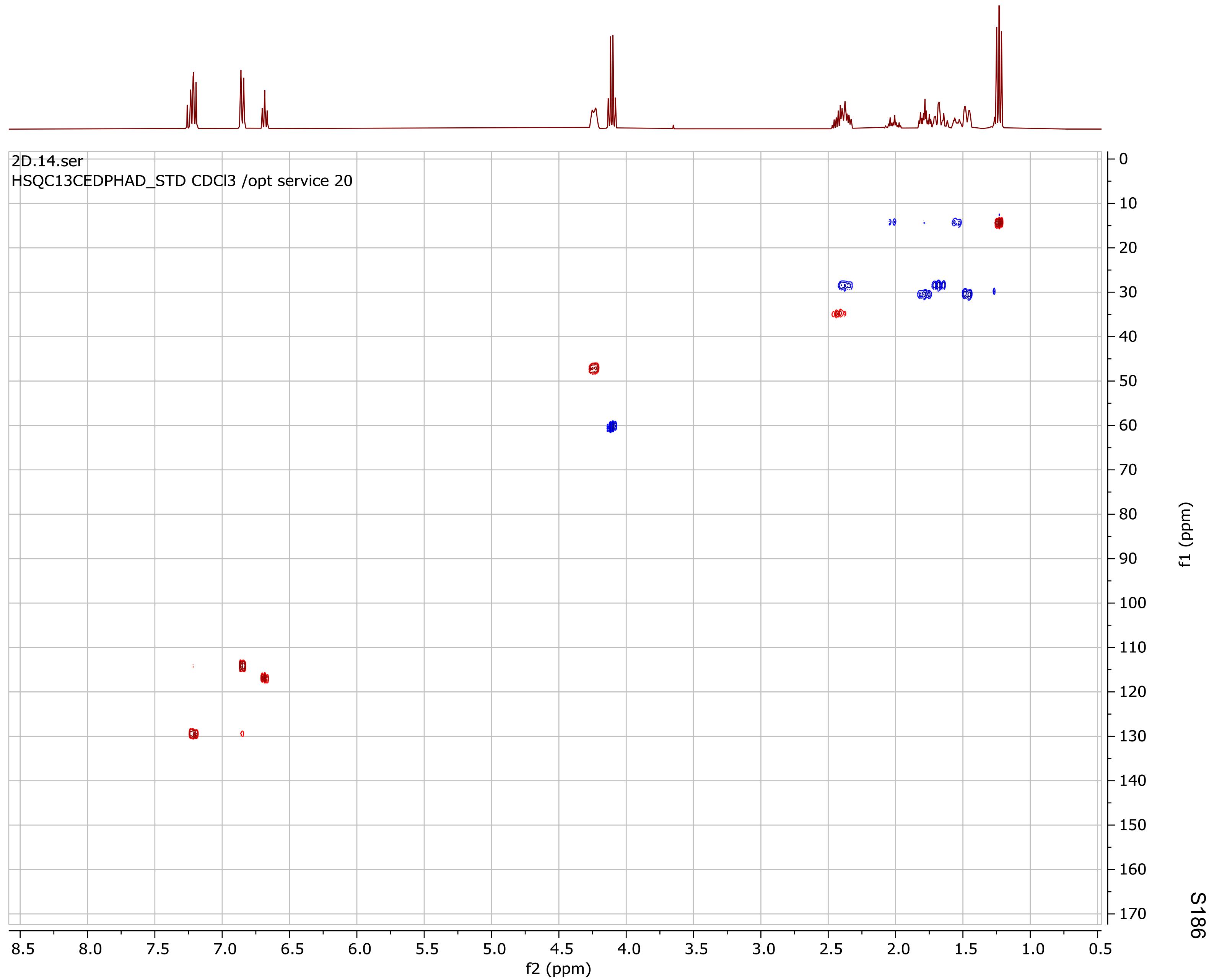

Ethyl 9-phenyl-9-azabicyclo[3.3.1]nonane-3-carboxylate (**8aα**)

^{1}H, ^{13}C-HMBC NMR (400 MHz, $CDCl_3$)

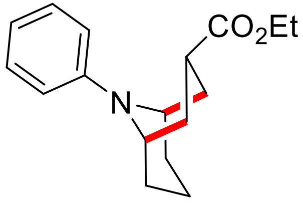

8a

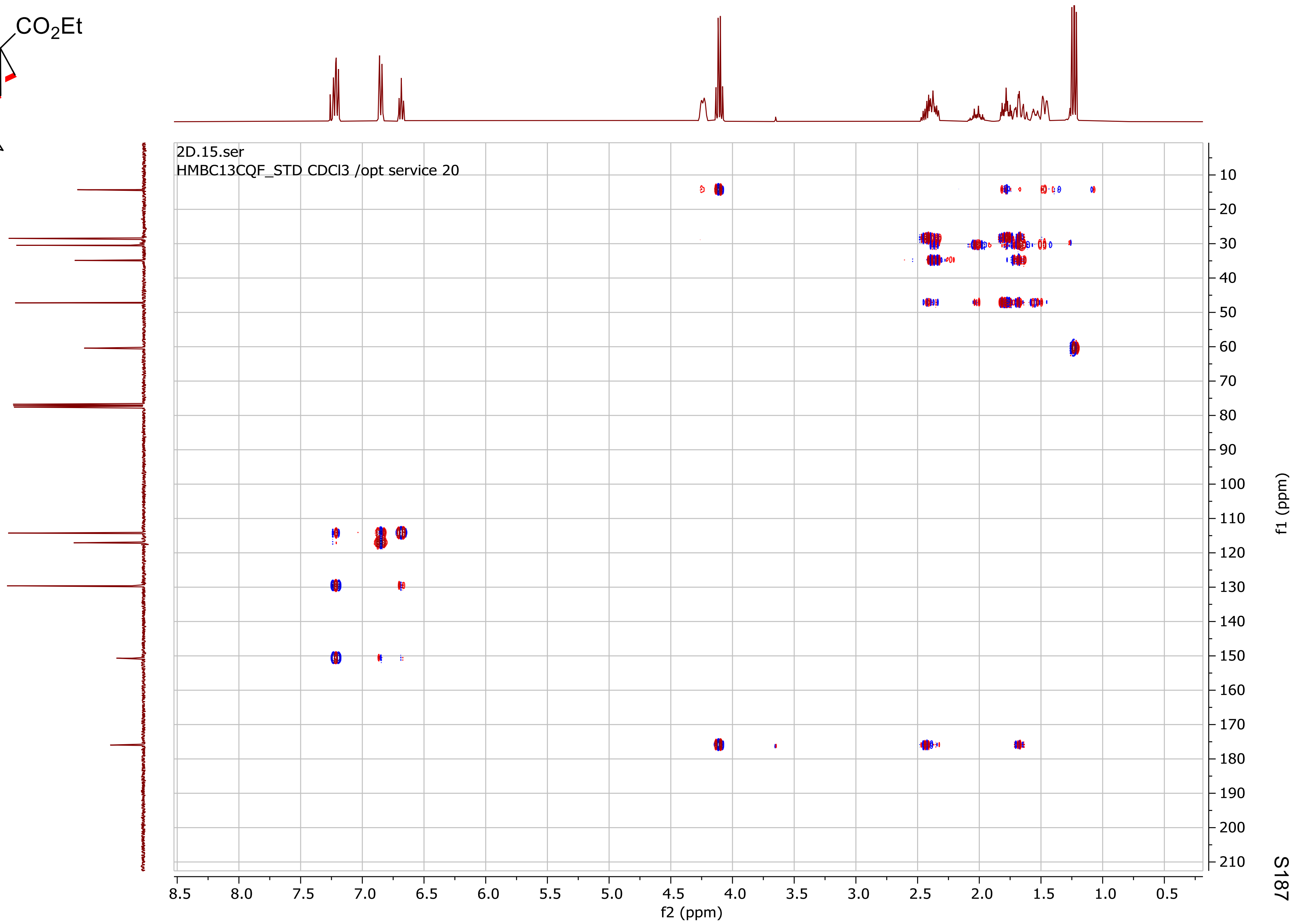

Ethyl 9-phenyl-9-azabicyclo[3.3.1]nonane-3-carboxylate (**8aα**)

FT-IR, ATR-diamond

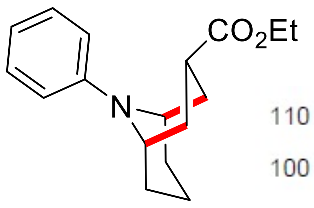

8a

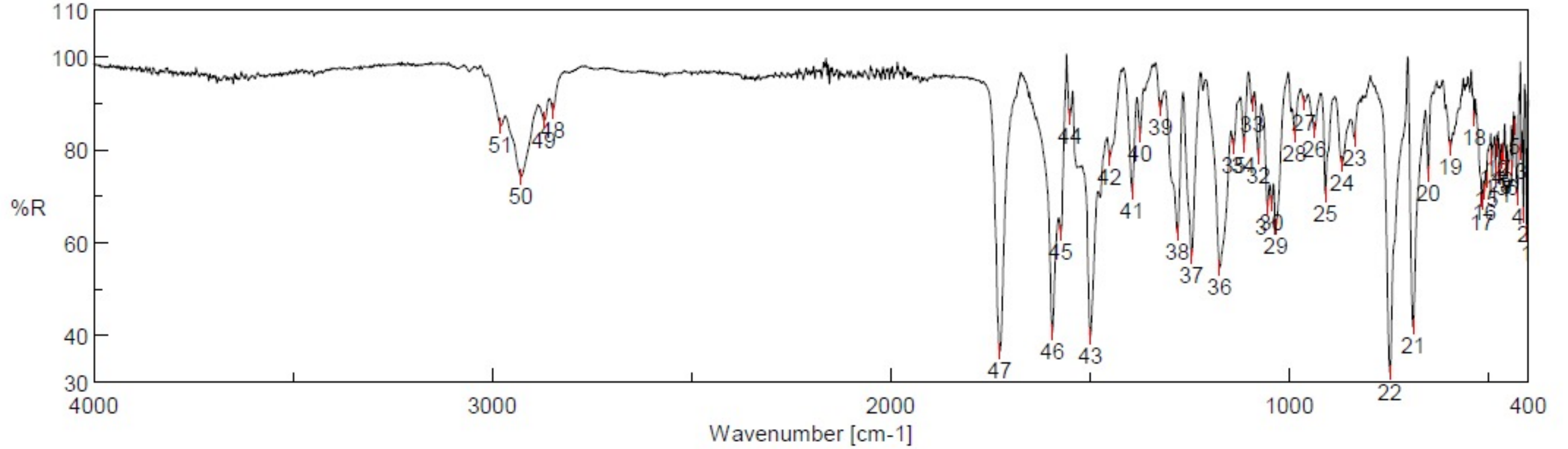

[Result of Peak Picking]

No.	Position	Intensity	No.	Position	Intensity	No.	Position	Intensity
1	404.014	62.0053	2	412.21	65.9467	3	417.513	79.3941
4	426.673	69.778	5	434.869	84.8131	6	439.69	76.0844
7	450.779	75.6865	8	455.6	76.4927	9	463.314	79.4275
10	467.653	77.7513	11	472.474	74.5768	12	477.778	80.1504
13	482.117	76.0162	14	491.277	78.2081	15	504.776	73.4229
16	510.08	70.8622	17	516.347	68.635	18	535.15	86.7998
19	595.414	80.5633	20	650.858	74.8062	21	688.945	41.8495
22	747.281	32.0363	23	835.99	82.3295	24	867.328	77.0792
25	909.272	70.4363	26	936.271	84.1991	27	962.305	90.0802
28	985.929	83.2978	29	1033.66	63.3579	30	1044.26	68.3345
31	1054.39	67.67	32	1077.53	78.6973	33	1092.48	89.7565
34	1113.69	80.9696	35	1139.24	80.902	36	1174.44	54.6578
37	1244.83	57.1933	38	1280.5	62.1092	39	1322.93	89.0015
40	1374.03	83.406	41	1394.28	71.036	42	1450.69	78.3069
43	1498.9	39.7487	44	1550.01	87.1773	45	1573.15	62.2225
46	1594.36	40.618	47	1725.98	36.5609	48	2847.86	88.4647
49	2870.04	86.3051	50	2928.38	74.0808	51	2979.48	85.1491

Ethyl 9-(o-tolyl)-9-azabicyclo[3.3.1]nonane-3-carboxylate (**8b**) **α/β 20:1**

^{1}H-NMR (400 MHz, $CDCl_3$)

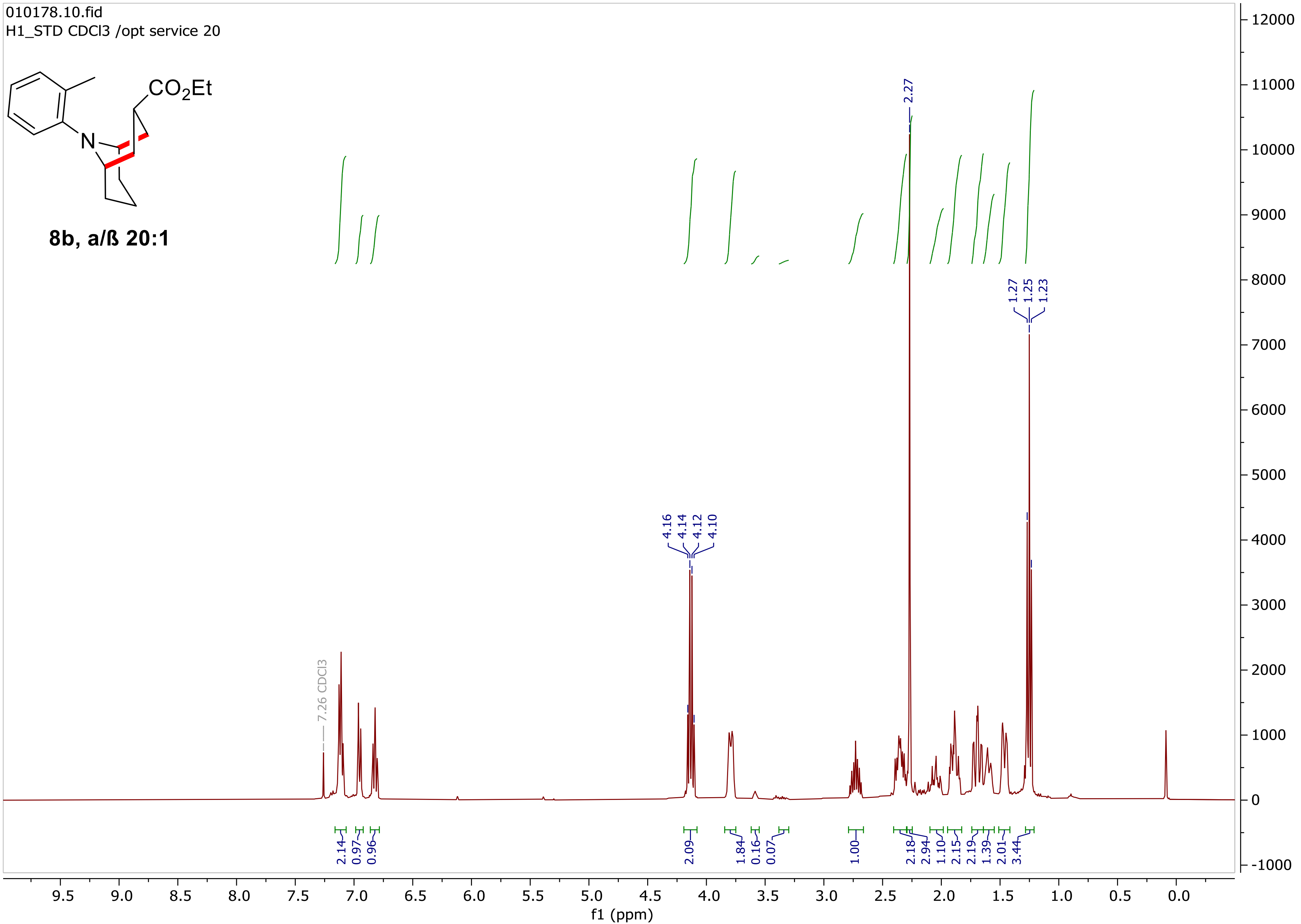

Ethyl 9-(o-tolyl)-9-azabicyclo[3.3.1]nonane-3-carboxylate (**8b**) **α/β 20:1**

^{1}H-NMR (400 MHz, $CDCl_3$)

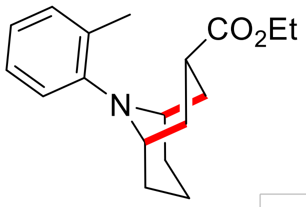

8b, a/ß 20:1

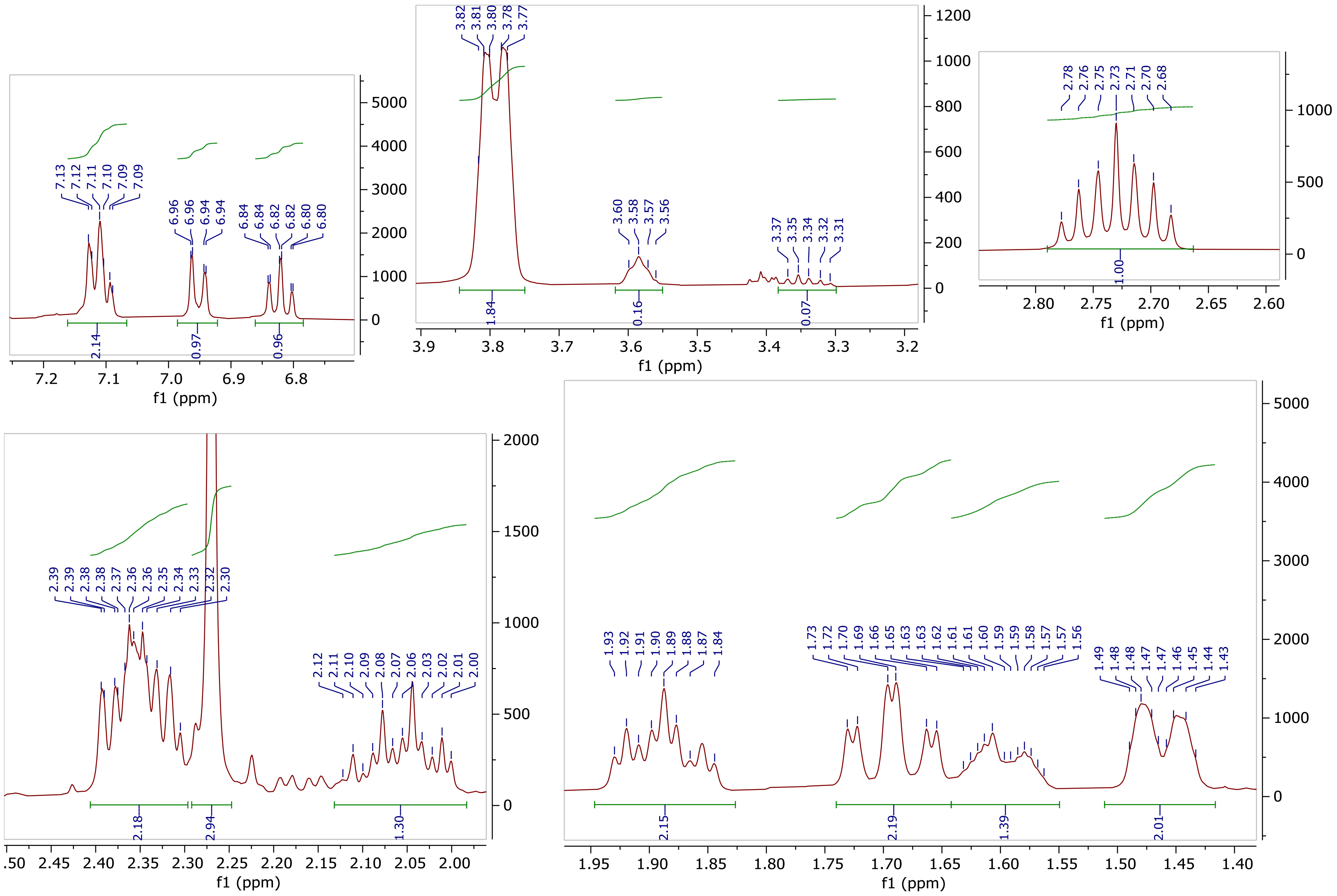

Ethyl 9-(o-tolyl)-9-azabicyclo[3.3.1]nonane-3-carboxylate (**8b**) **α/β 20:1**

^{13}C-NMR (101 MHz, $CDCl_3$)

010178.11.fid
C13CPD_STD CDCl3 /opt service 20

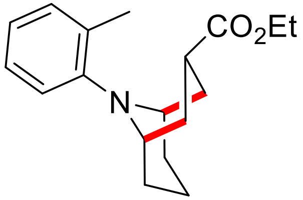

8b, a/ß 20:1

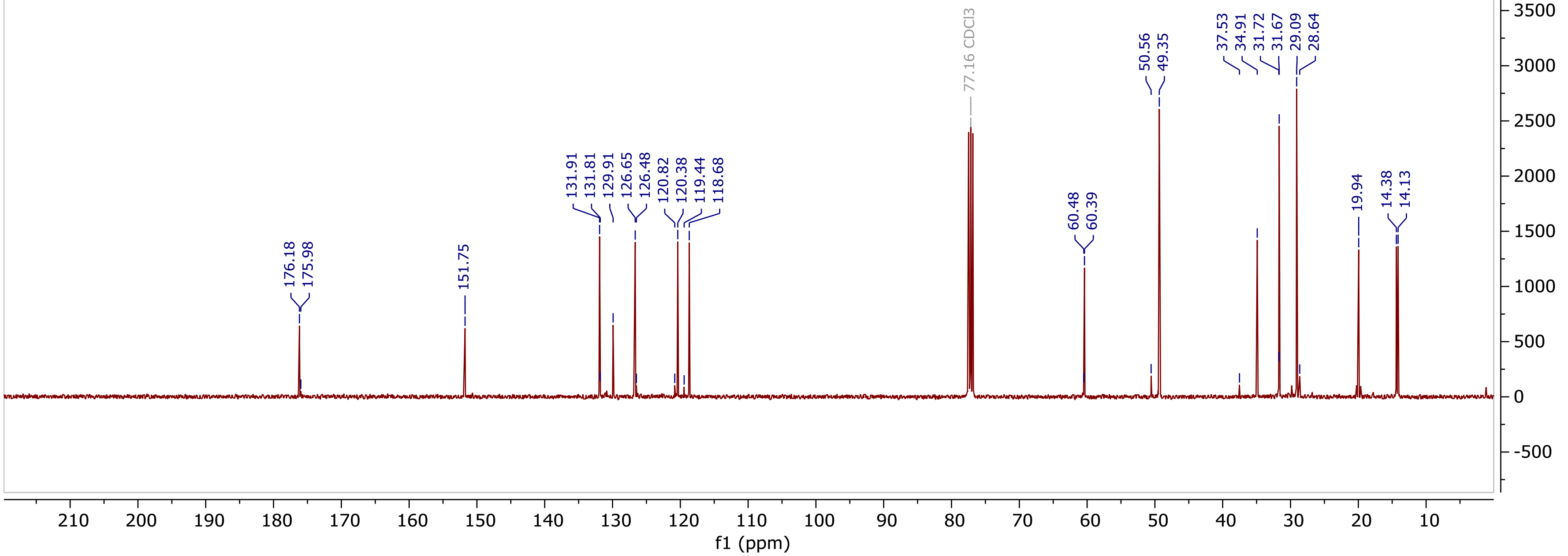

Ethyl 9-(o-tolyl)-9-azabicyclo[3.3.1]nonane-3-carboxylate (**8b**) **α/β 20:1**

^{13}C-NMR (101 MHz, $CDCl_3$)

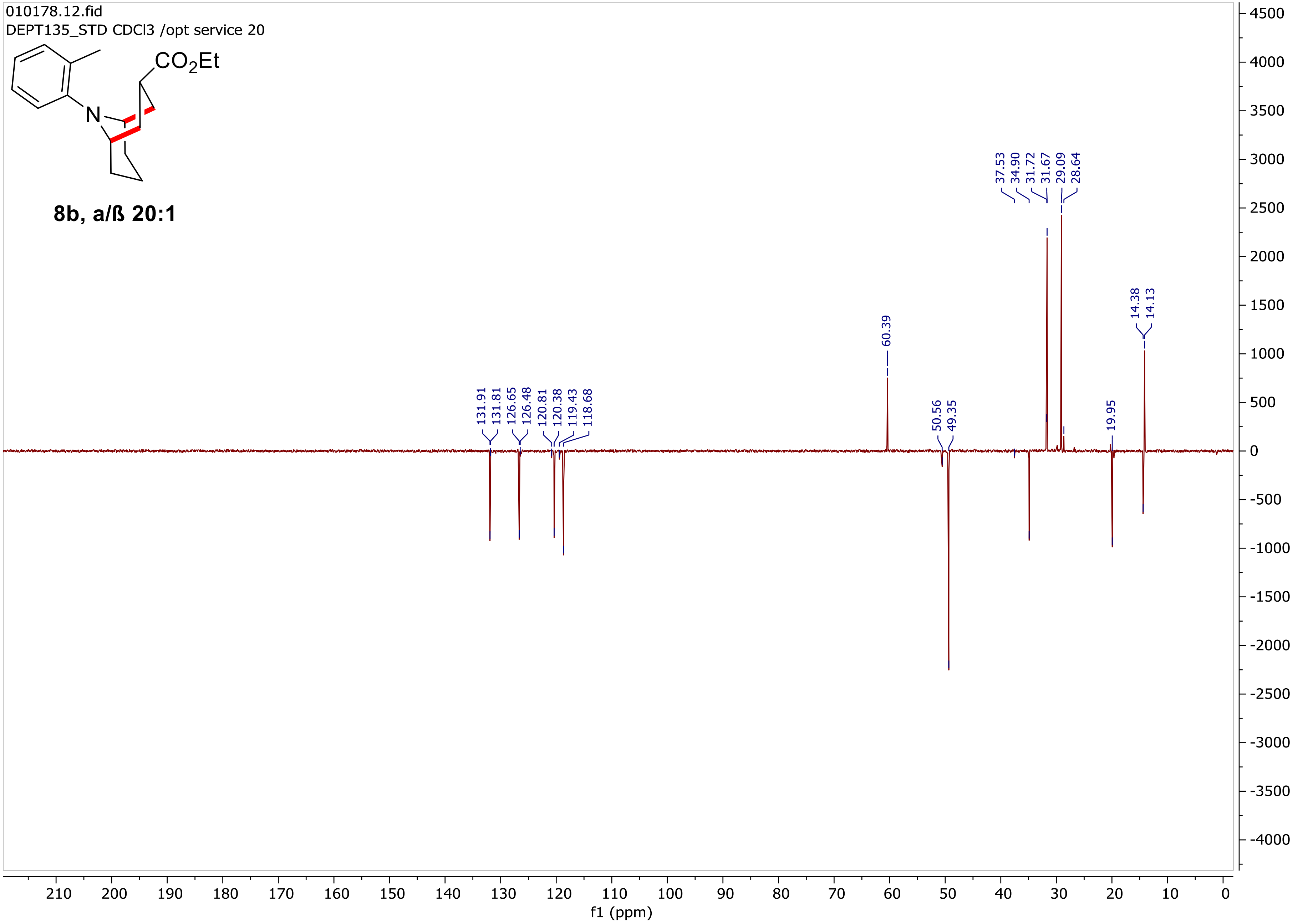

Ethyl 9-(o-tolyl)-9-azabicyclo[3.3.1]nonane-3-carboxylate (**8b**) **α/β 20:1**

^{1}H, ^{1}H-COSY NMR (400 MHz, $CDCl_3$)

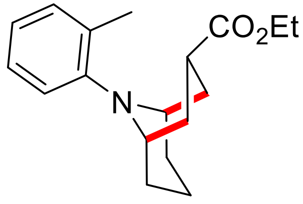

8b, a/ß 20:1

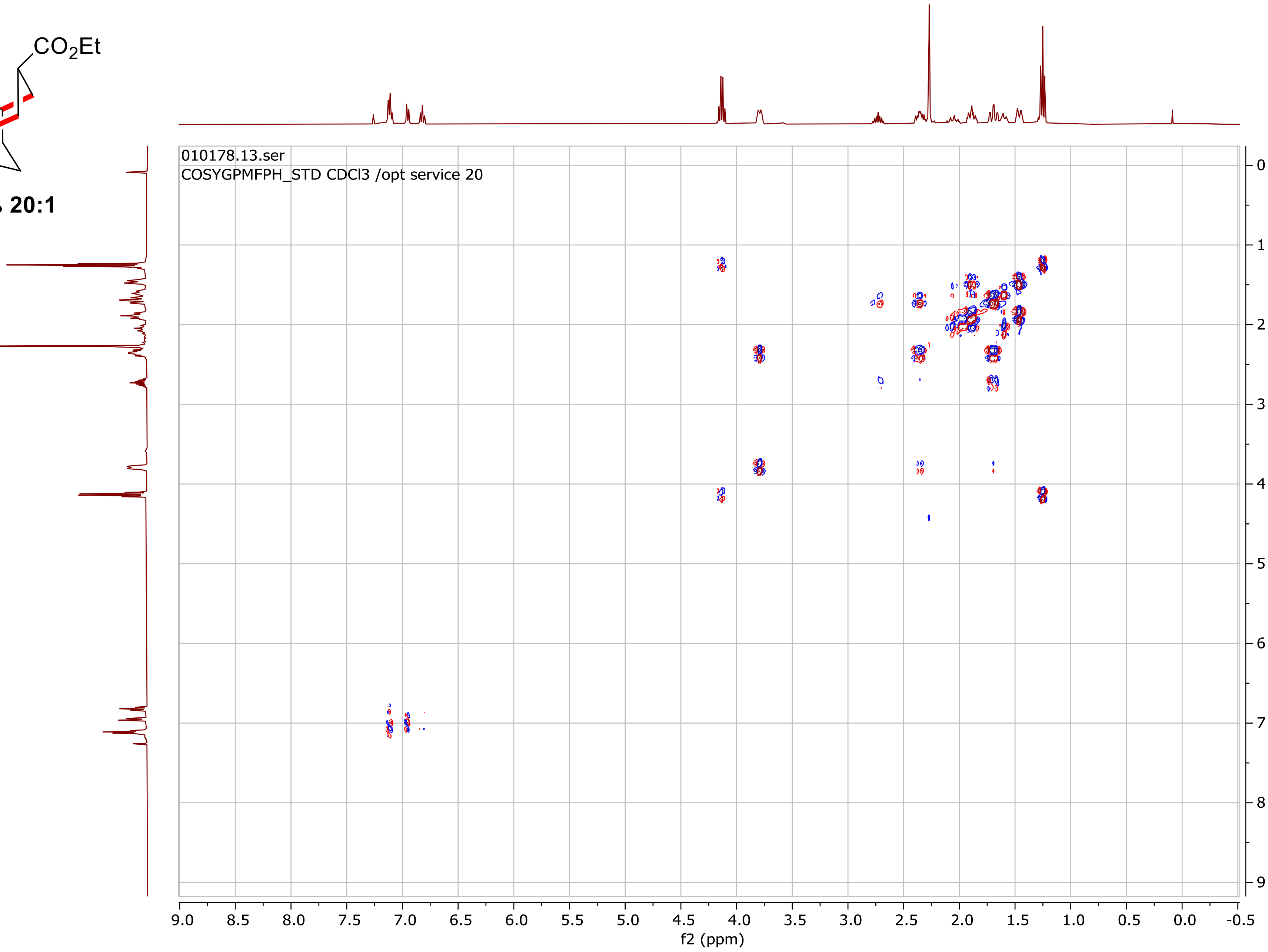

Ethyl 9-(o-tolyl)-9-azabicyclo[3.3.1]nonane-3-carboxylate (**8b**) **α/β 20:1**

^1H, ^{13}C-HSQC NMR (400 MHz, $CDCl_3$)

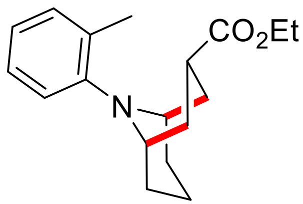

8b, a/ß 20:1

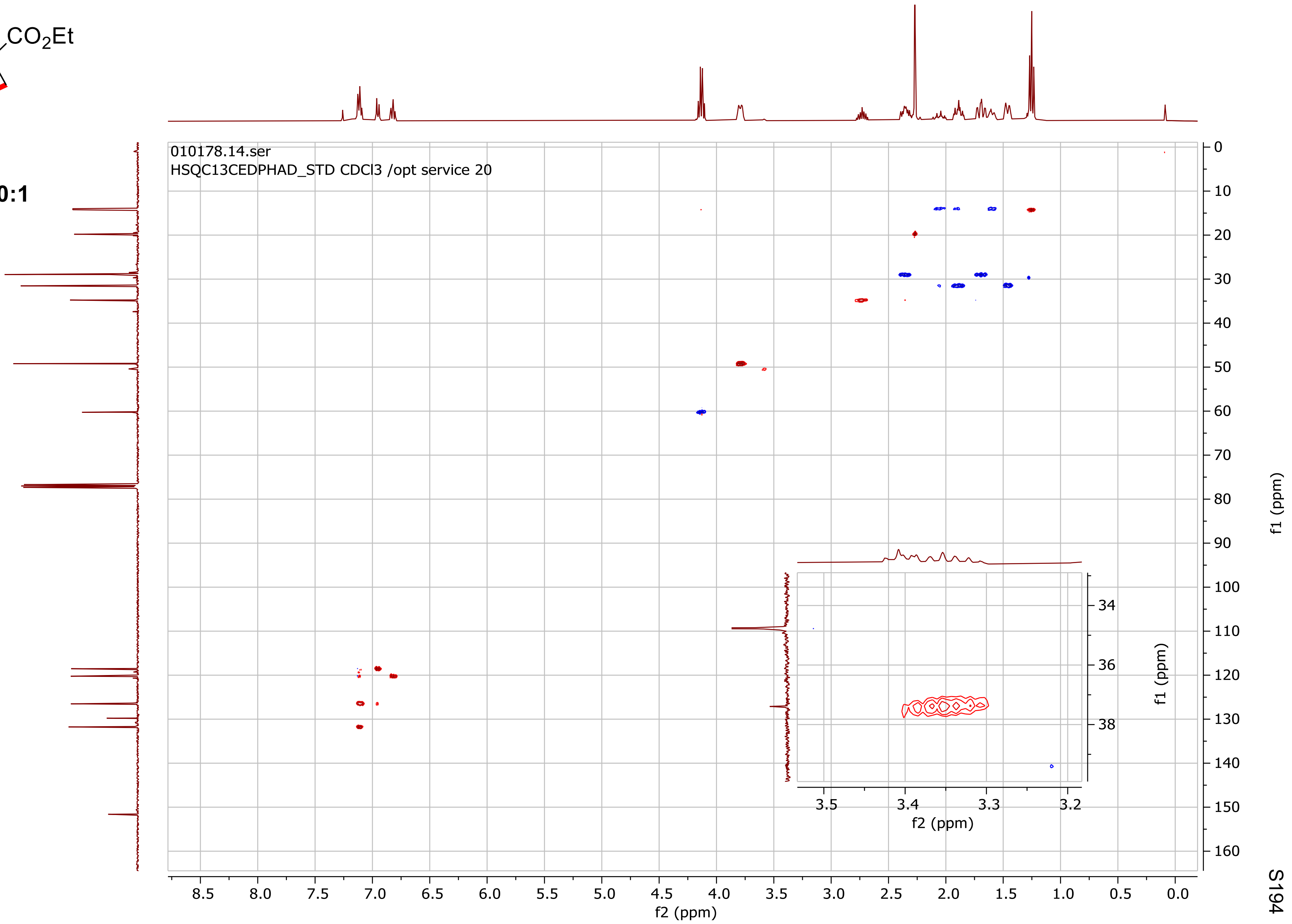

Ethyl 9-(o-tolyl)-9-azabicyclo[3.3.1]nonane-3-carboxylate (**8b**) **α/β 20:1**

^{1}H, ^{13}C-HMBC NMR (400 MHz, $CDCl_3$)

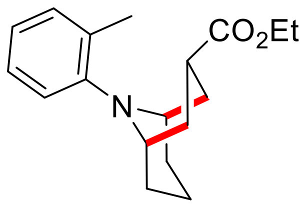

8b, a/ß 20:1

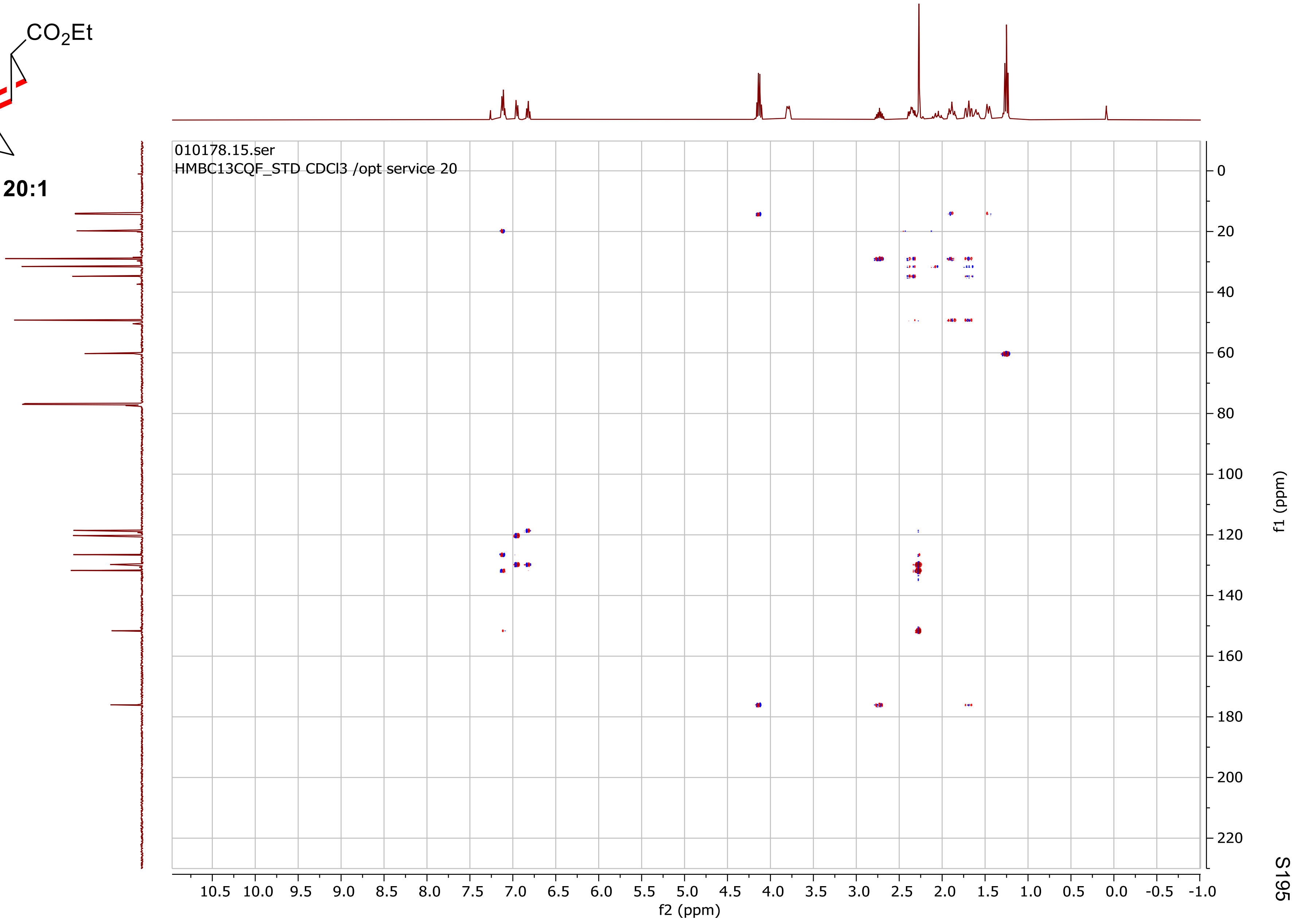

Ethyl 9-(o-tolyl)-9-azabicyclo[3.3.1]nonane-3-carboxylate (**8b**) **α/β 20:1**

FT-IR, ATR-diamond

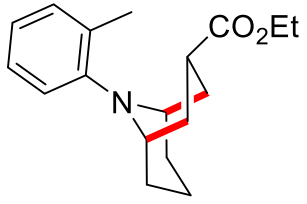

8b, a/ß 20:1

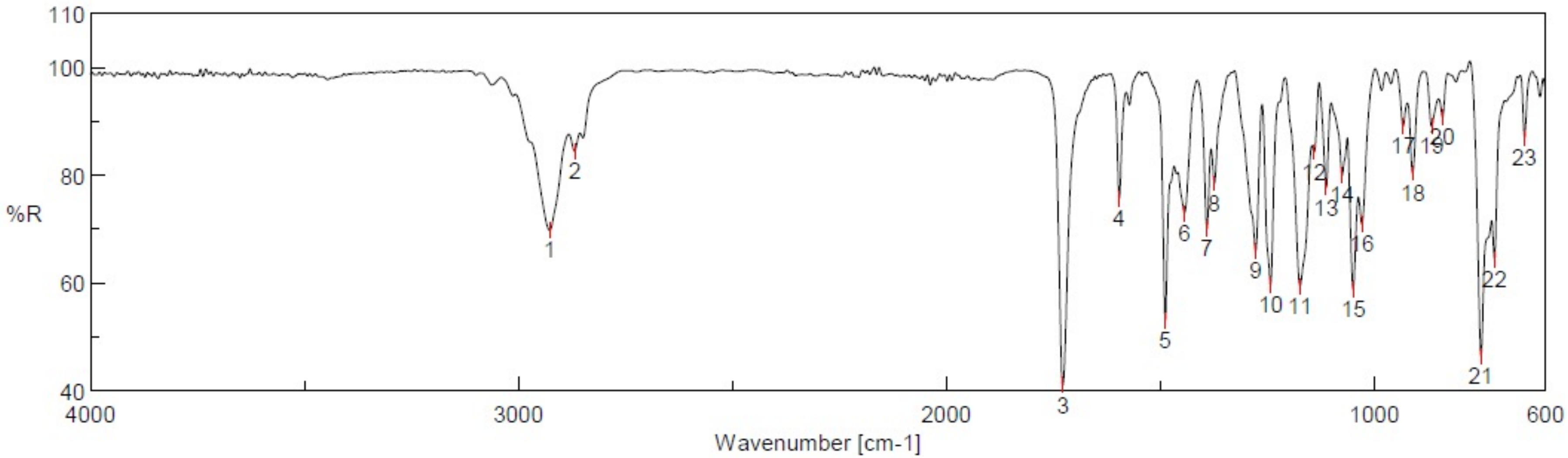

[Result of Peak Picking]

No.	Position	Intensity	No.	Position	Intensity	No.	Position	Intensity
1	2927.41	69.7635	2	2868.59	84.456	3	1727.91	40.9429
4	1595.81	75.6963	5	1488.78	53.0118	6	1443.46	72.9644
7	1391.39	70.0061	8	1374.03	78.4973	9	1277.61	65.8157
10	1241.93	59.6269	11	1173.47	59.4046	12	1140.69	84.2908
13	1112.73	77.6516	14	1075.12	79.9064	15	1049.09	58.7751
16	1028.84	70.8479	17	932.414	89.0887	18	910.236	80.3994
19	865.882	89.0197	20	840.812	90.7587	21	751.138	46.4454
22	719.318	64.3748	23	648.929	86.8473			

Ethyl 9-(m-tolyl)-9-azabicyclo[3.3.1]nonane-3-carboxylate (**8c**) **α/β >20:1**

^{1}H-NMR (400 MHz, $CDCl_3$)

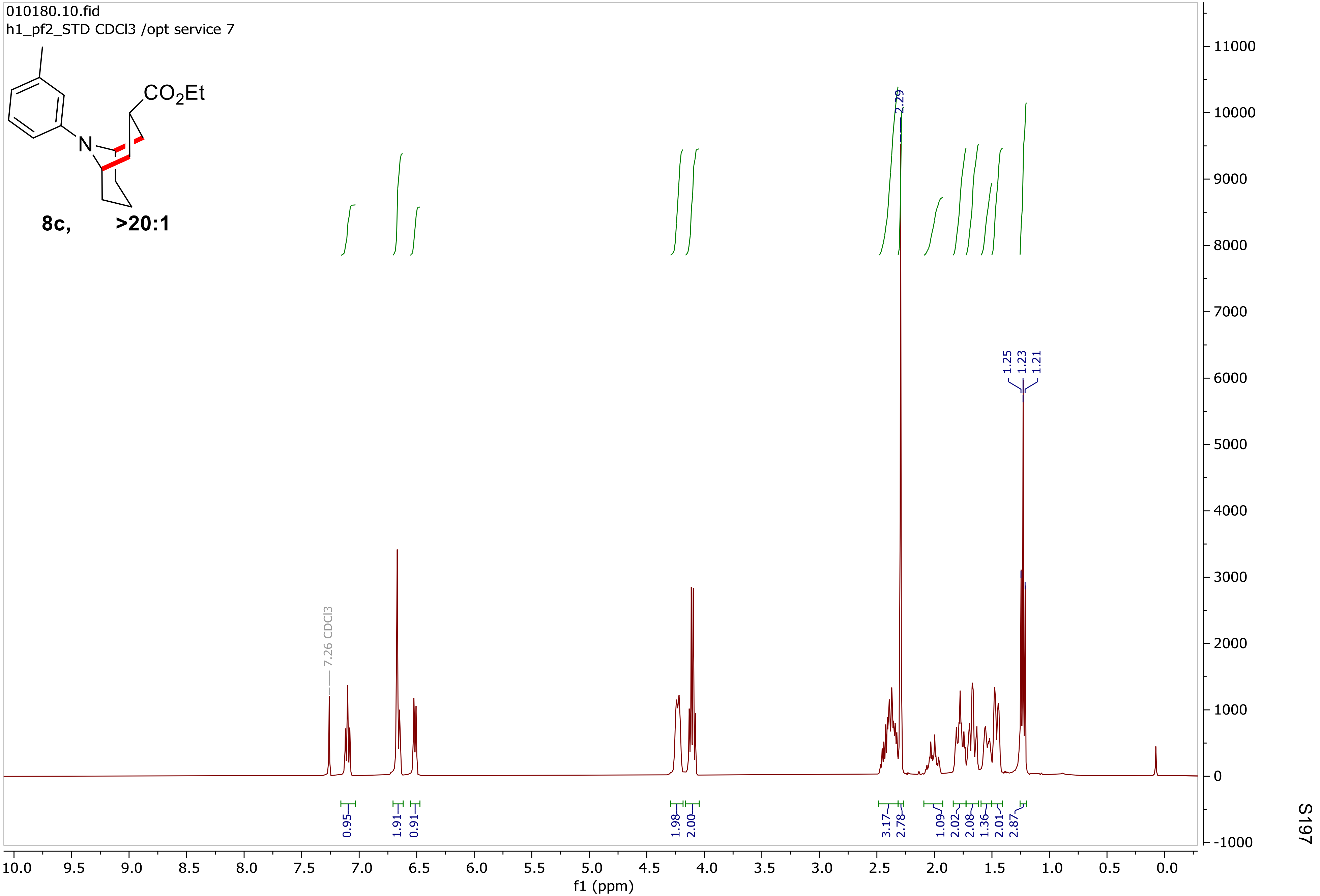

Ethyl 9-(m-tolyl)-9-azabicyclo[3.3.1]nonane-3-carboxylate (**8cα**)

^{1}H-NMR (400 MHz, $CDCl_3$)

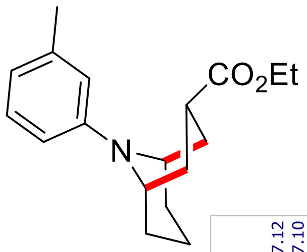

8c

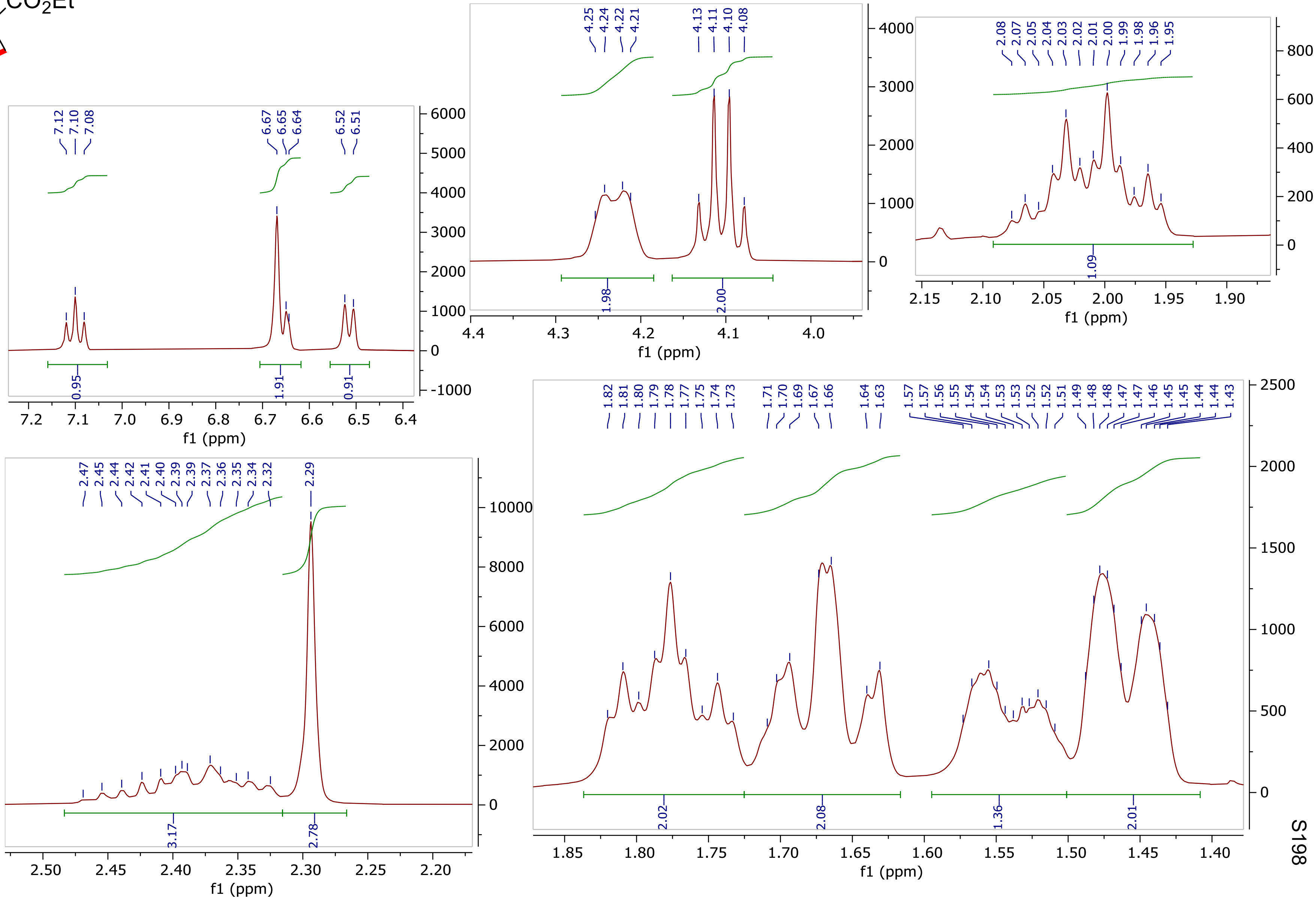

Ethyl 9-(m-tolyl)-9-azabicyclo[3.3.1]nonane-3-carboxylate (**8cα**)

^{13}C-NMR (75 MHz, $CDCl_3$)

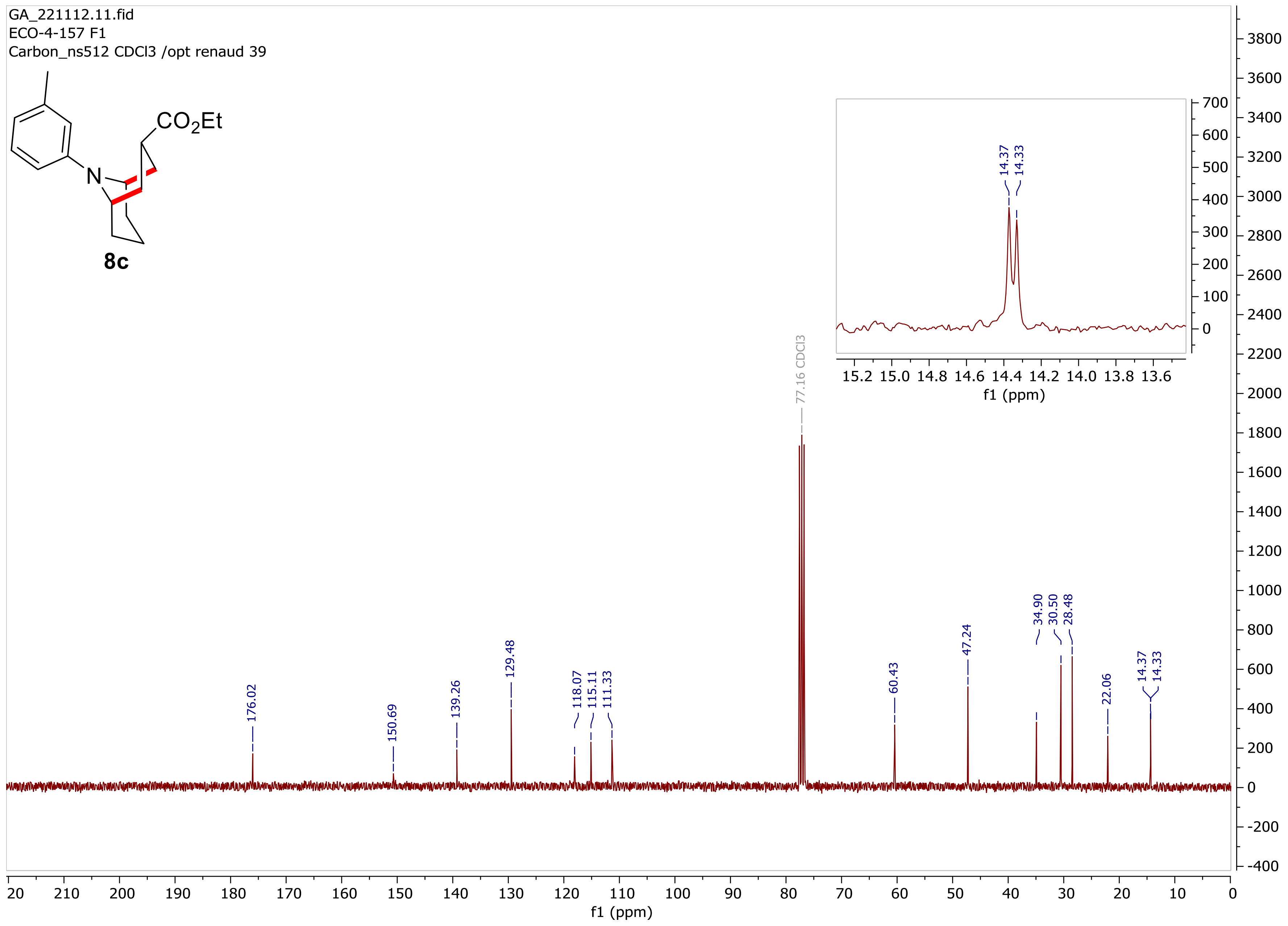

Ethyl 9-(m-tolyl)-9-azabicyclo[3.3.1]nonane-3-carboxylate (**8cα**)

^{13}C-NMR (75 MHz, $CDCl_3$)

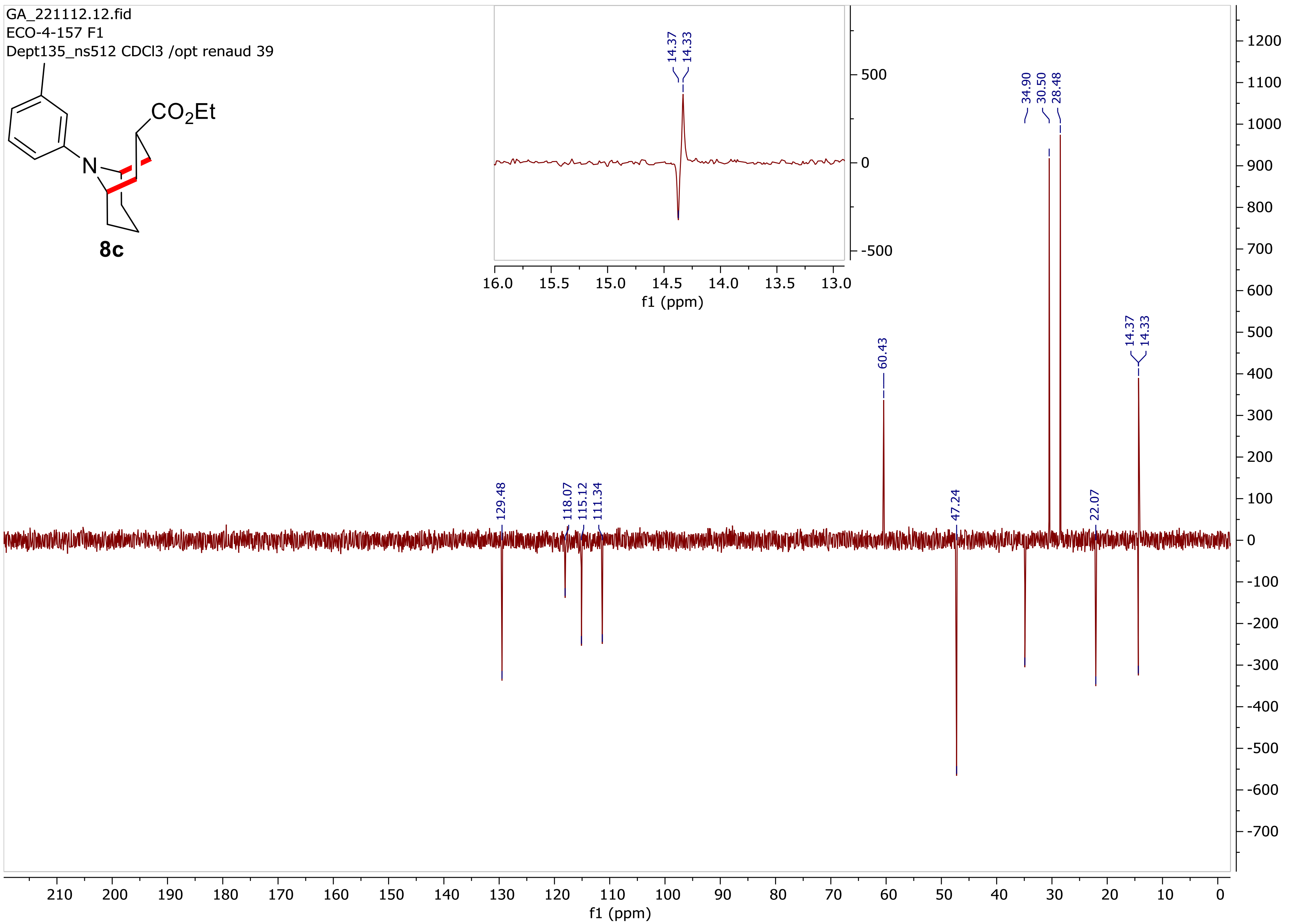

Ethyl 9-(m-tolyl)-9-azabicyclo[3.3.1]nonane-3-carboxylate (**8cα**)

^{1}H, ^{1}H-COSY NMR (400 MHz, $CDCl_3$)

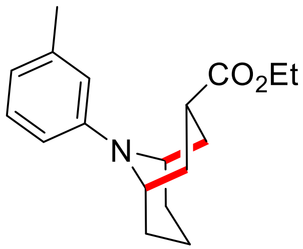

8c

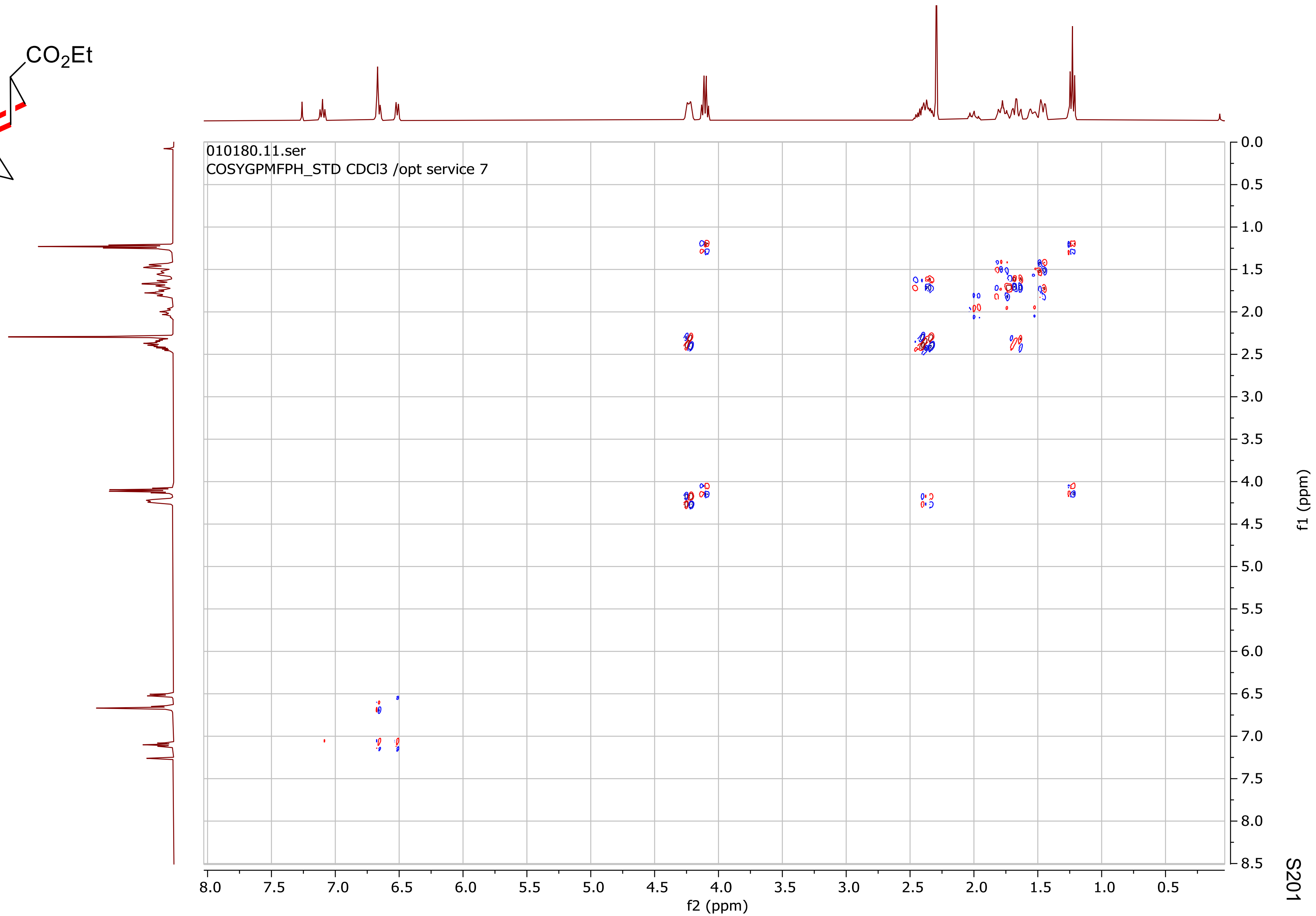

Ethyl 9-(m-tolyl)-9-azabicyclo[3.3.1]nonane-3-carboxylate (**8cα**)

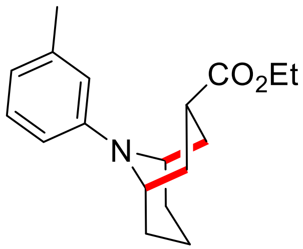

8c

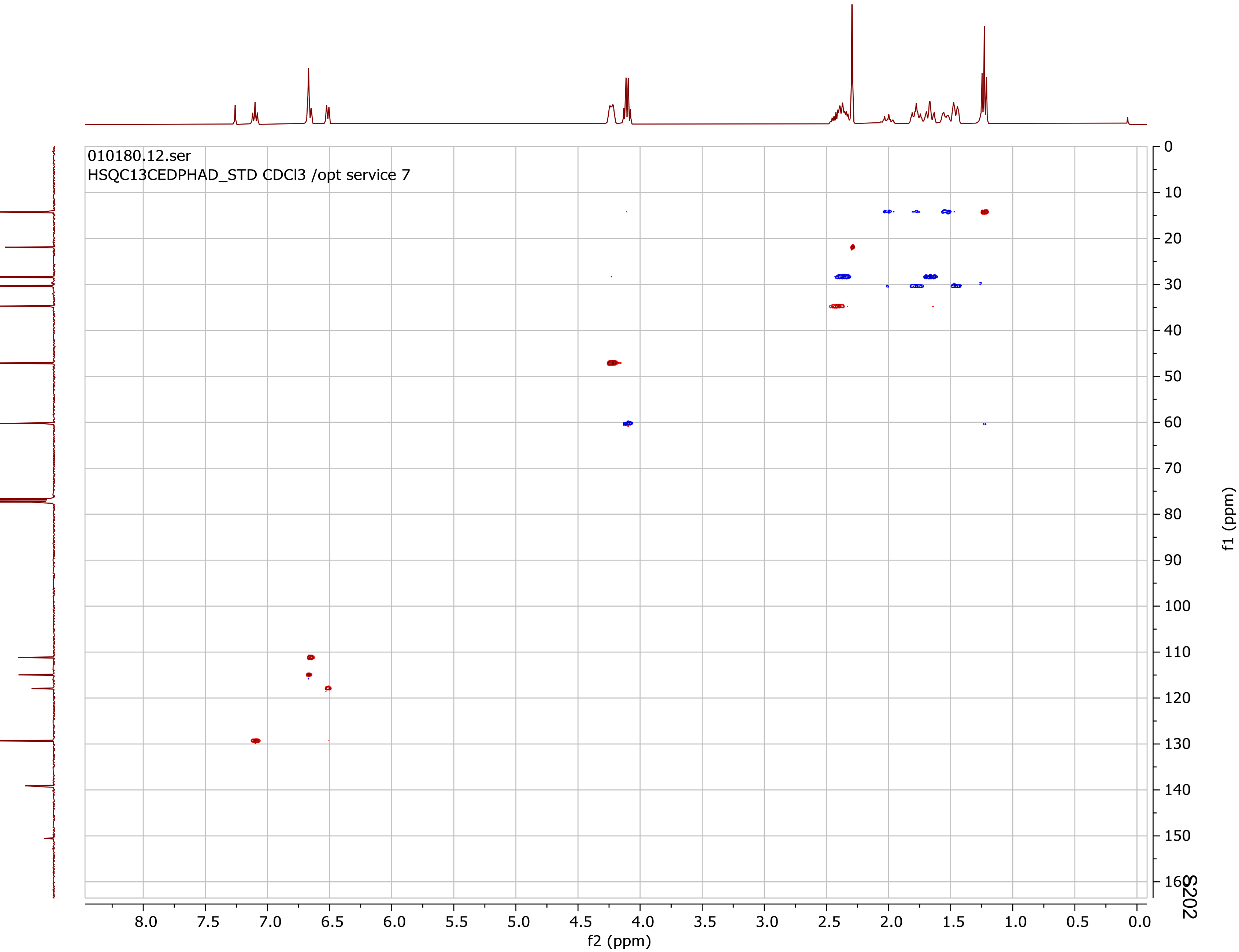

Ethyl 9-(m-tolyl)-9-azabicyclo[3.3.1]nonane-3-carboxylate (**8cα**)

^{1}H, ^{13}C-HMBC NMR (400 MHz, $CDCl_3$)

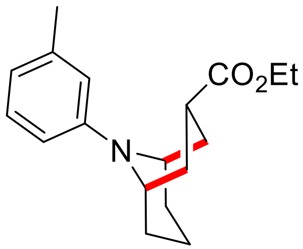

8c

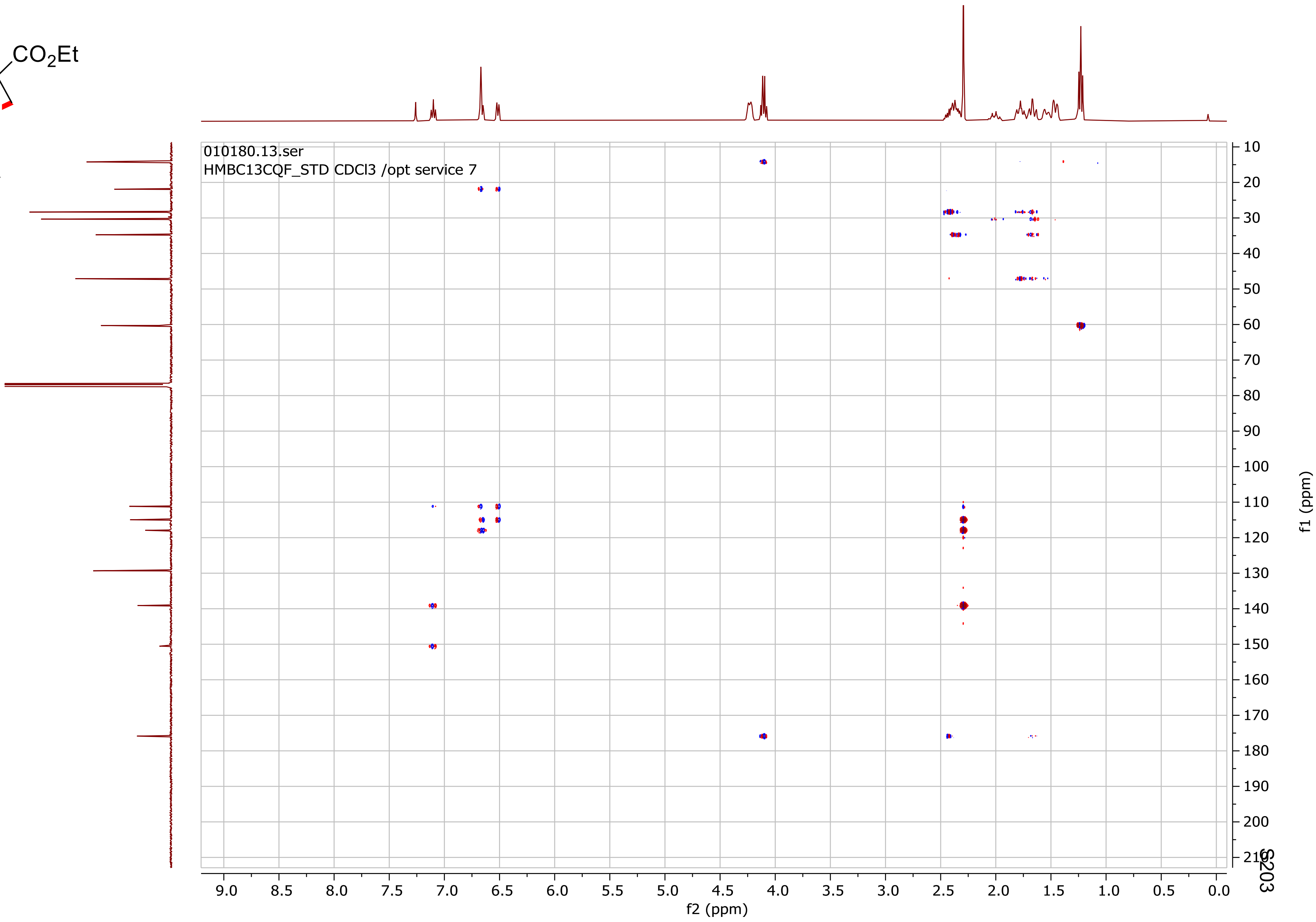

Ethyl 9-(m-tolyl)-9-azabicyclo[3.3.1]nonane-3-carboxylate (**8cα**)

FT-IR, ATR-diamond

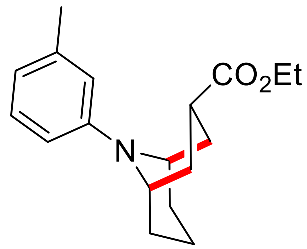

8c

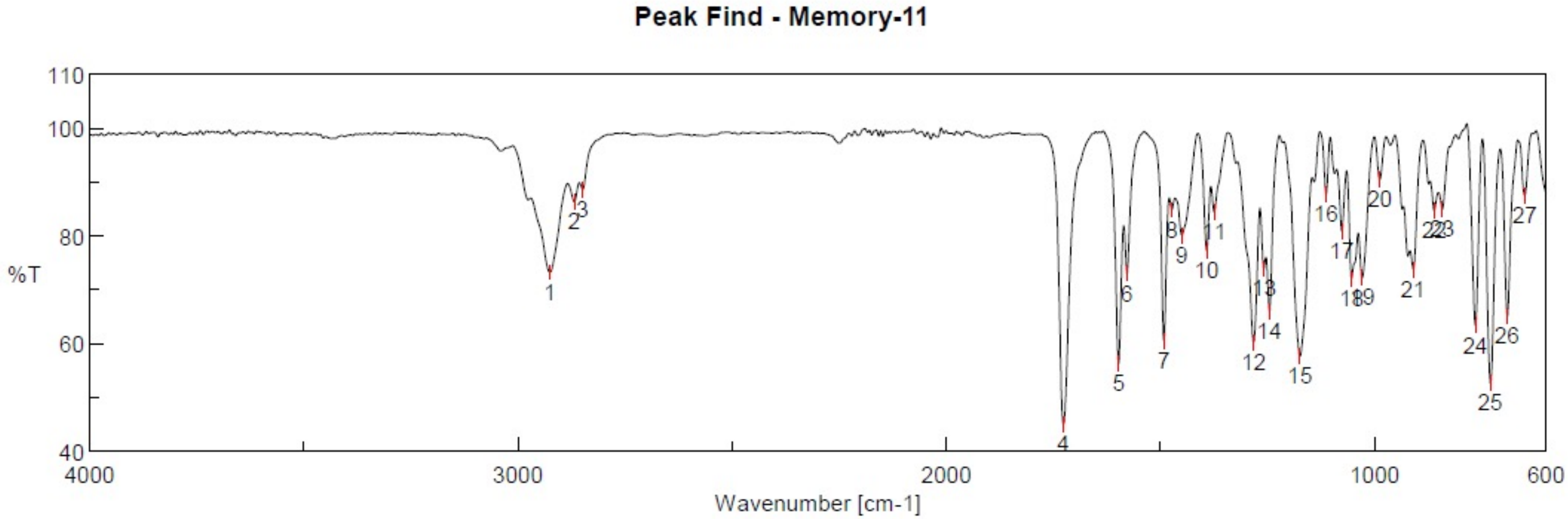

[Result of Peak Picking]

No.	Position	Intensity	No.	Position	Intensity	No.	Position	Intensity
1	2925.48	73	2	2868.59	86	3	2849.31	89
4	1726.94	45	5	1597.73	56	6	1578.45	73
7	1492.63	61	8	1473.35	85	9	1450.21	80
10	1392.35	77	11	1375	84	12	1282.43	60
13	1258.32	74	14	1245.79	66	15	1174.44	58
16	1113.69	88	17	1077.05	81	18	1053.91	72
19	1028.84	72	20	988.339	90	21	910.236	74
22	861.06	85	23	841.776	85	24	764.637	63
25	729.925	53	26	690.391	65	27	649.893	88

Ethyl 9-(p-tolyl)-9-azabicyclo[3.3.1]nonane-3-carboxylate (**8d**) **α/β 20:1**

^{1}H-NMR (400 MHz, $CDCl_3$)

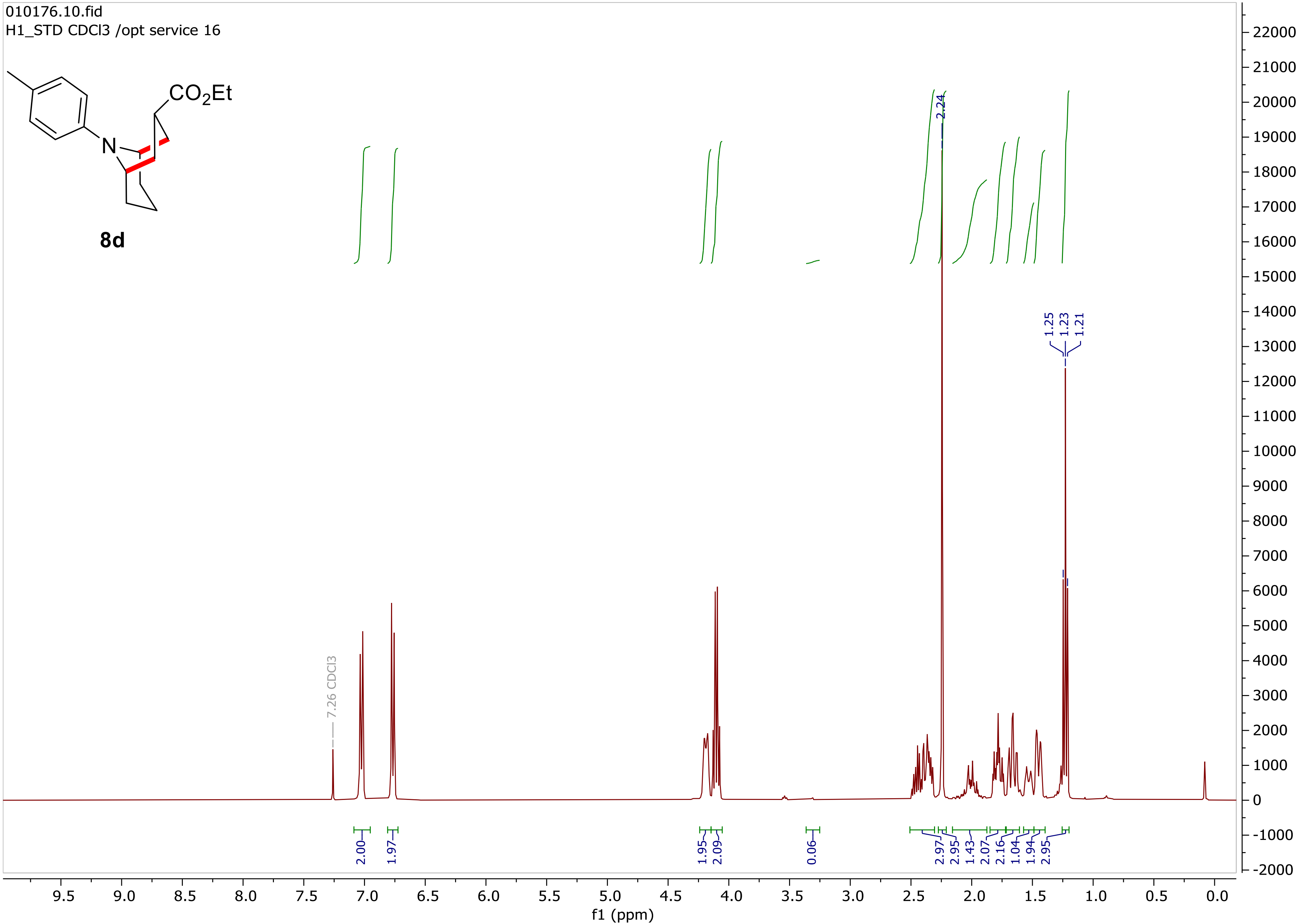

Ethyl 9-(p-tolyl)-9-azabicyclo[3.3.1]nonane-3-carboxylate (**8dα**)

^{1}H-NMR (400 MHz, $CDCl_3$)

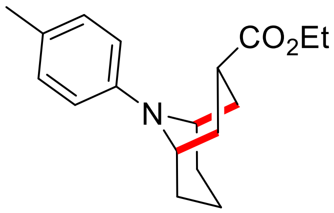

8d

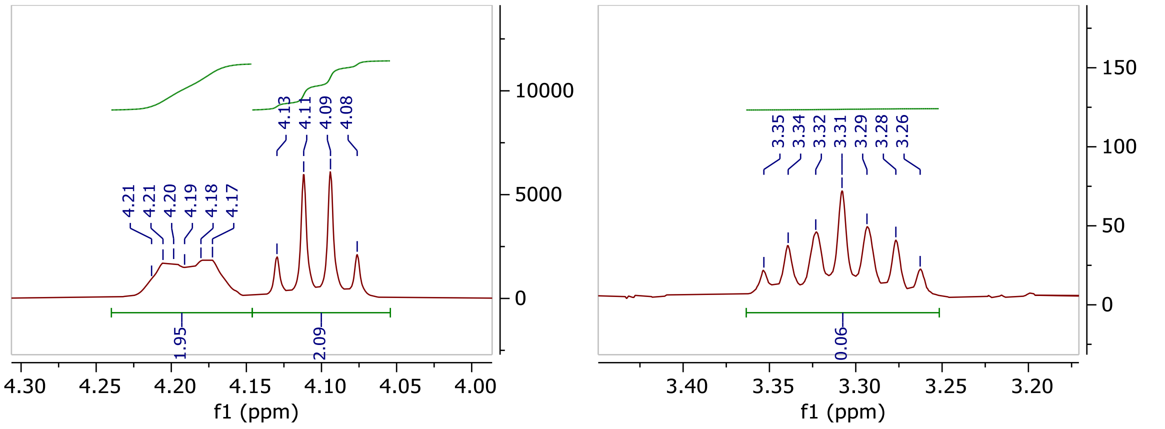

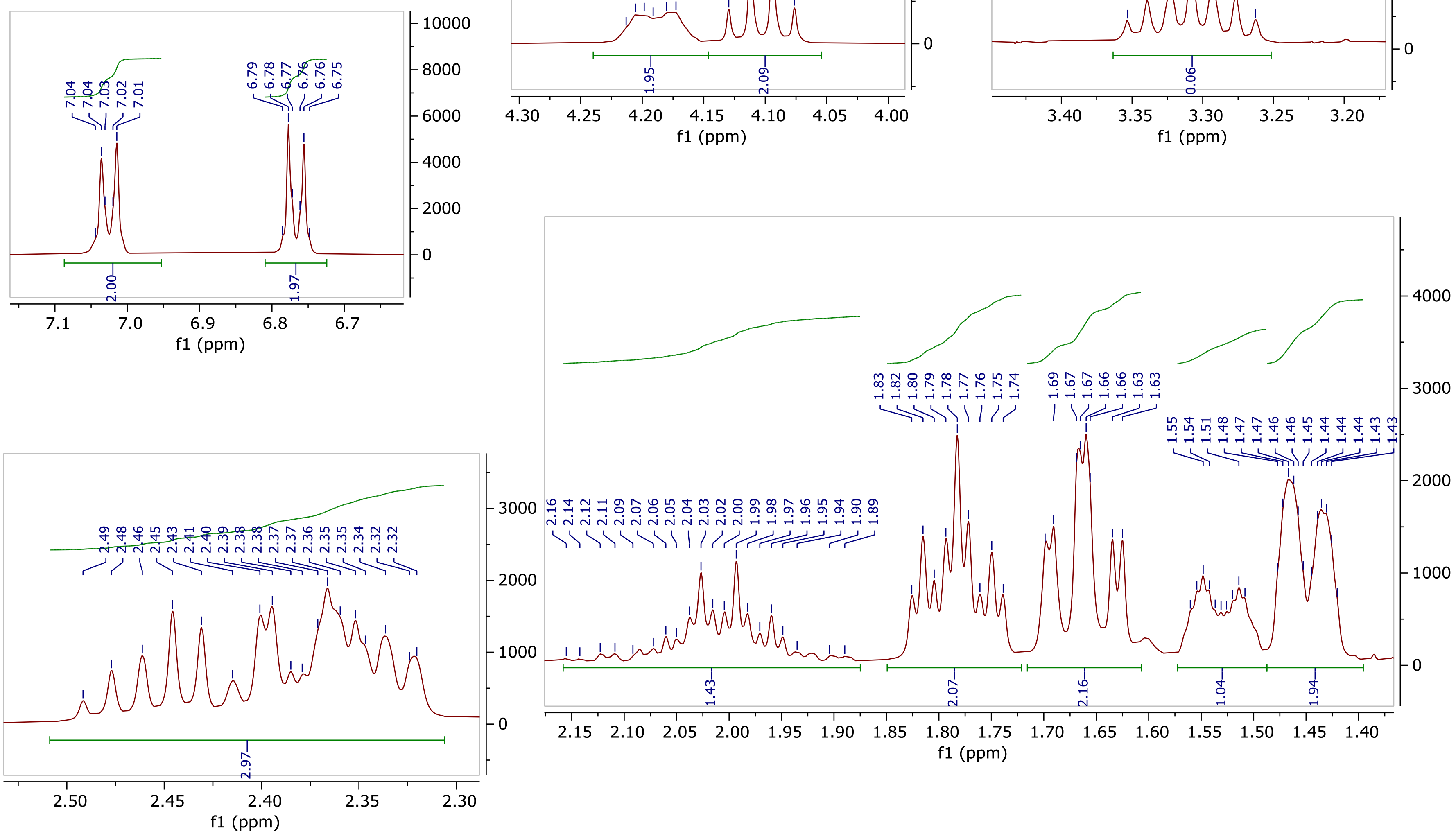

Ethyl 9-(p-tolyl)-9-azabicyclo[3.3.1]nonane-3-carboxylate (**8dα**)

^{13}C-NMR (101 MHz, $CDCl_3$)

010176.16.fid
C13CPD_STD CDCl3 /opt service 16

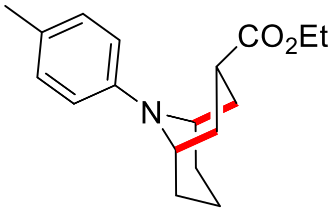

8d

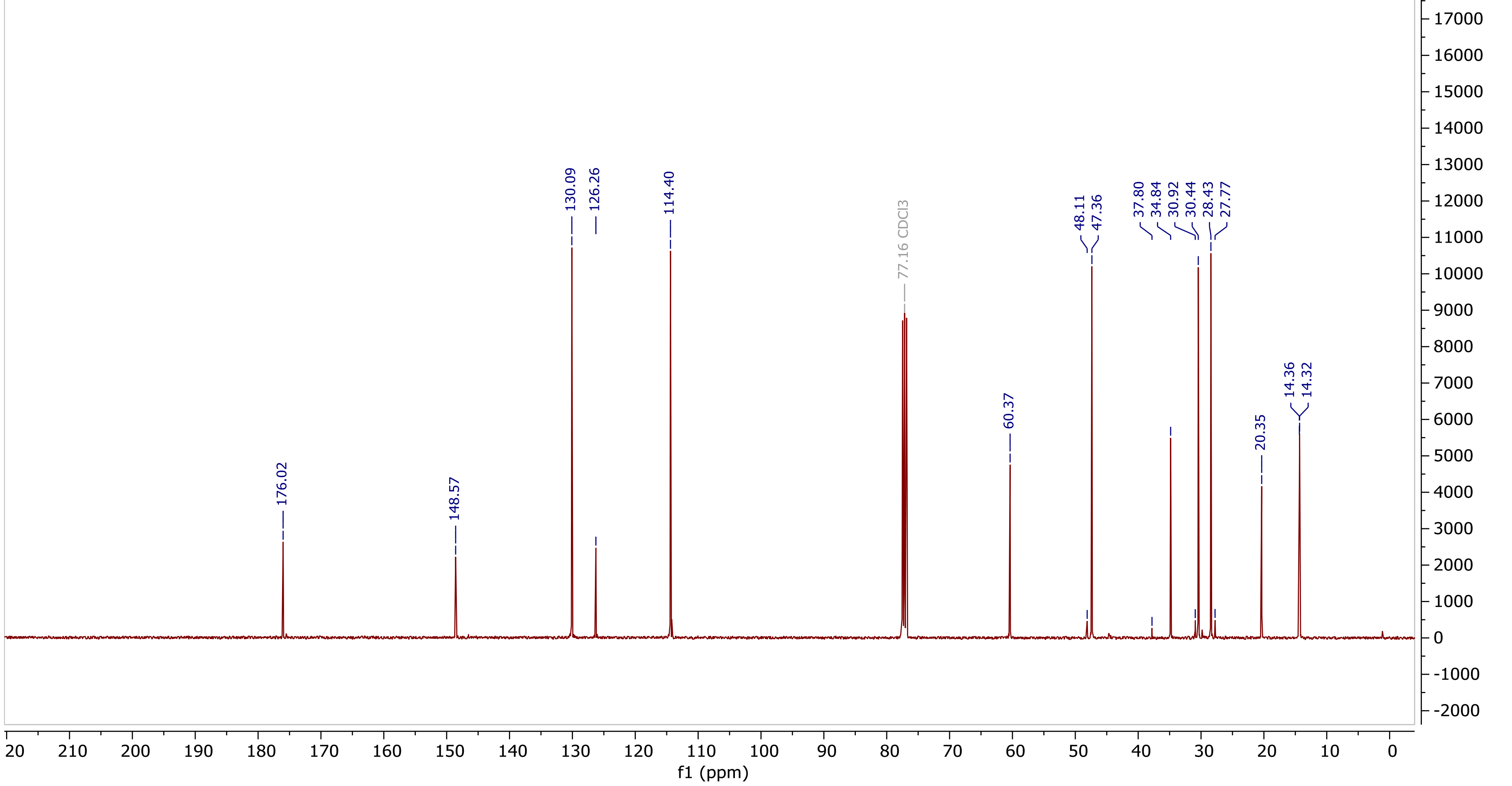

Ethyl 9-(p-tolyl)-9-azabicyclo[3.3.1]nonane-3-carboxylate (**8dα**)

^{13}C-NMR (101 MHz, $CDCl_3$)

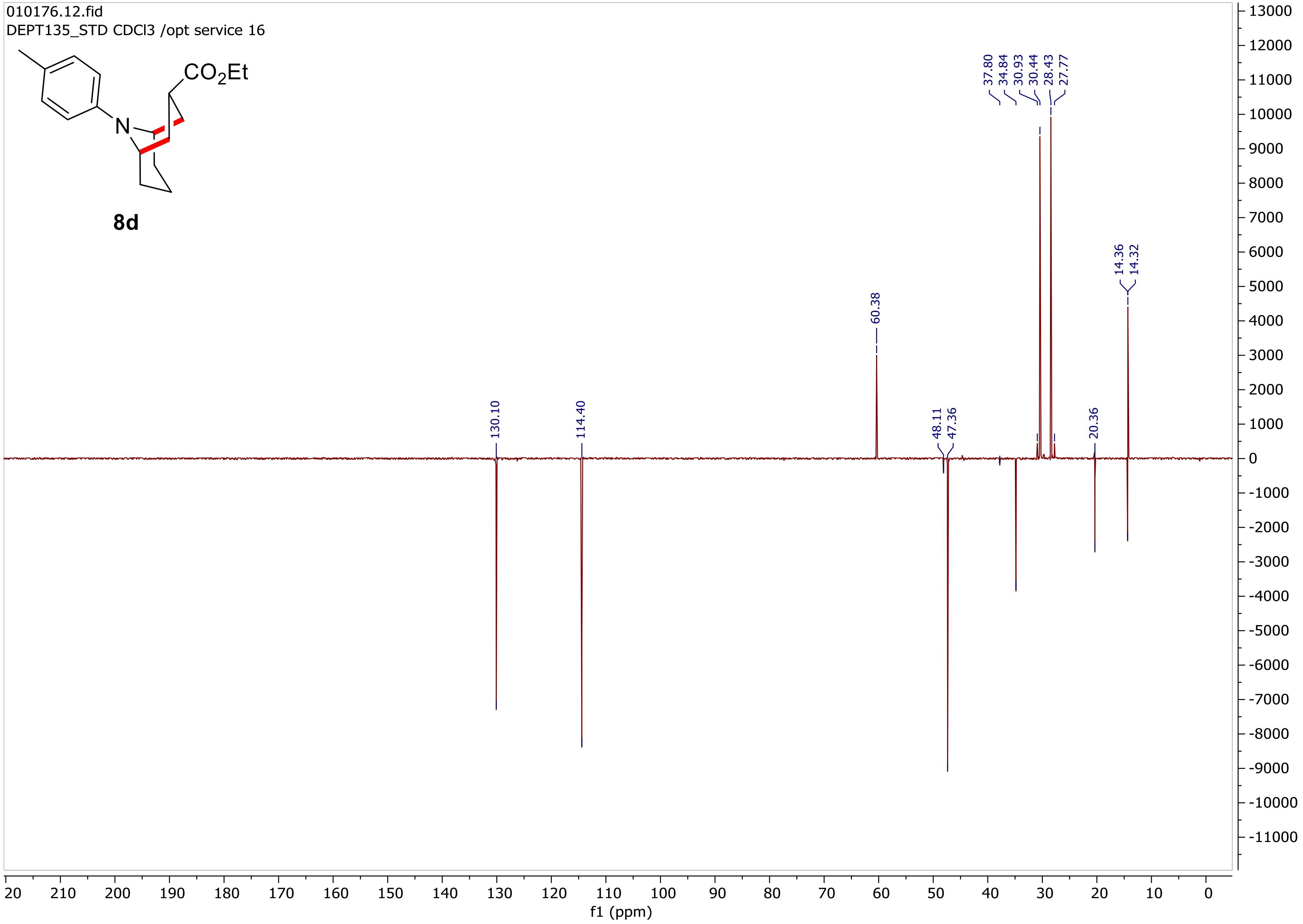

Ethyl 9-(p-tolyl)-9-azabicyclo[3.3.1]nonane-3-carboxylate (**8dα**)

^1H, ^1H-COSY NMR (400 MHz, $CDCl_3$)

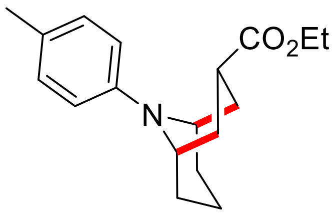

8d

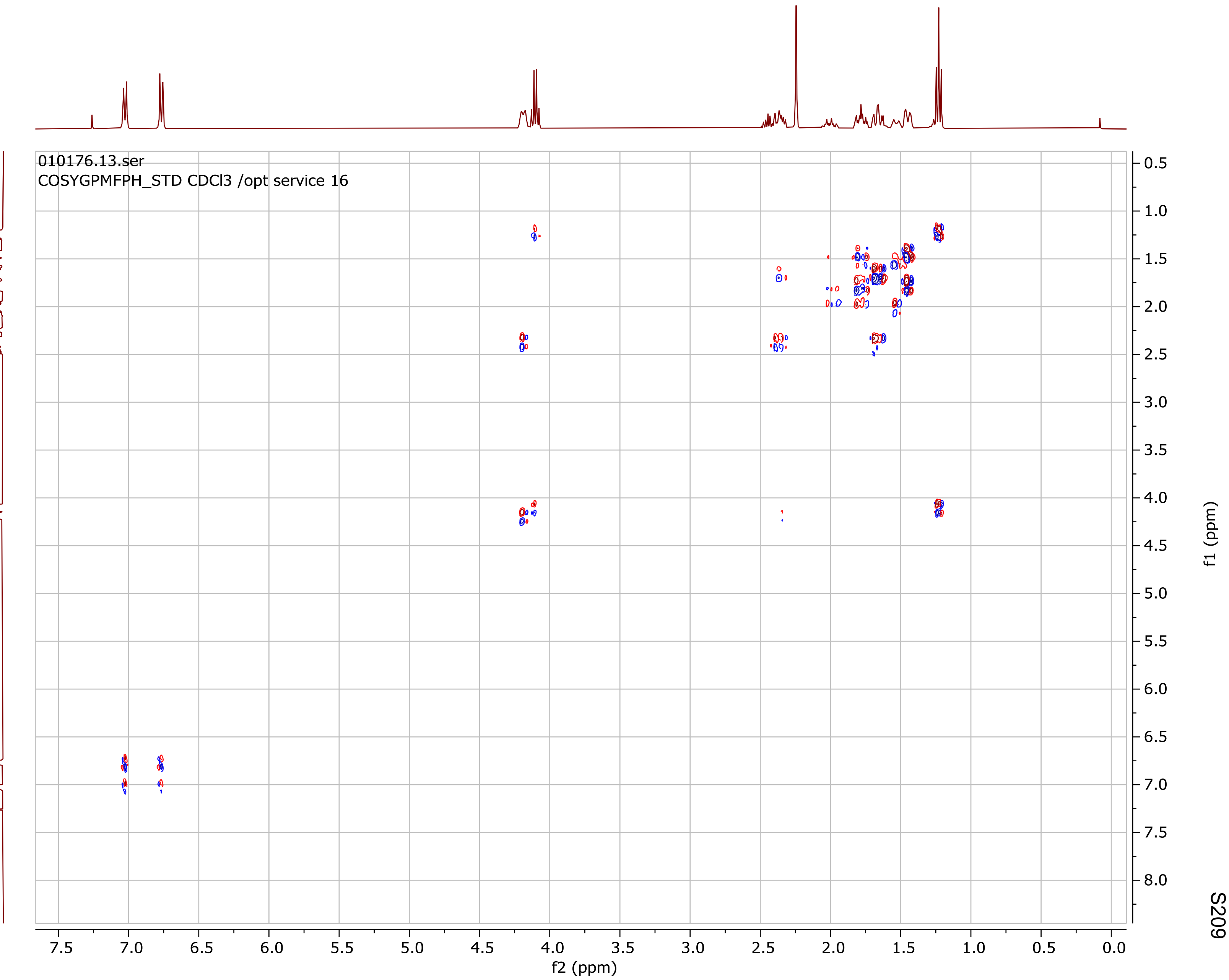

Ethyl 9-(p-tolyl)-9-azabicyclo[3.3.1]nonane-3-carboxylate (**8dα**)

^{1}H, ^{13}C-HSQC NMR (400 MHz, $CDCl_3$)

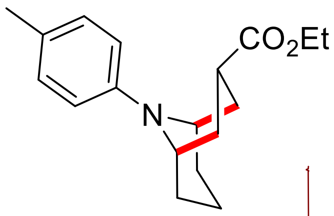

8d

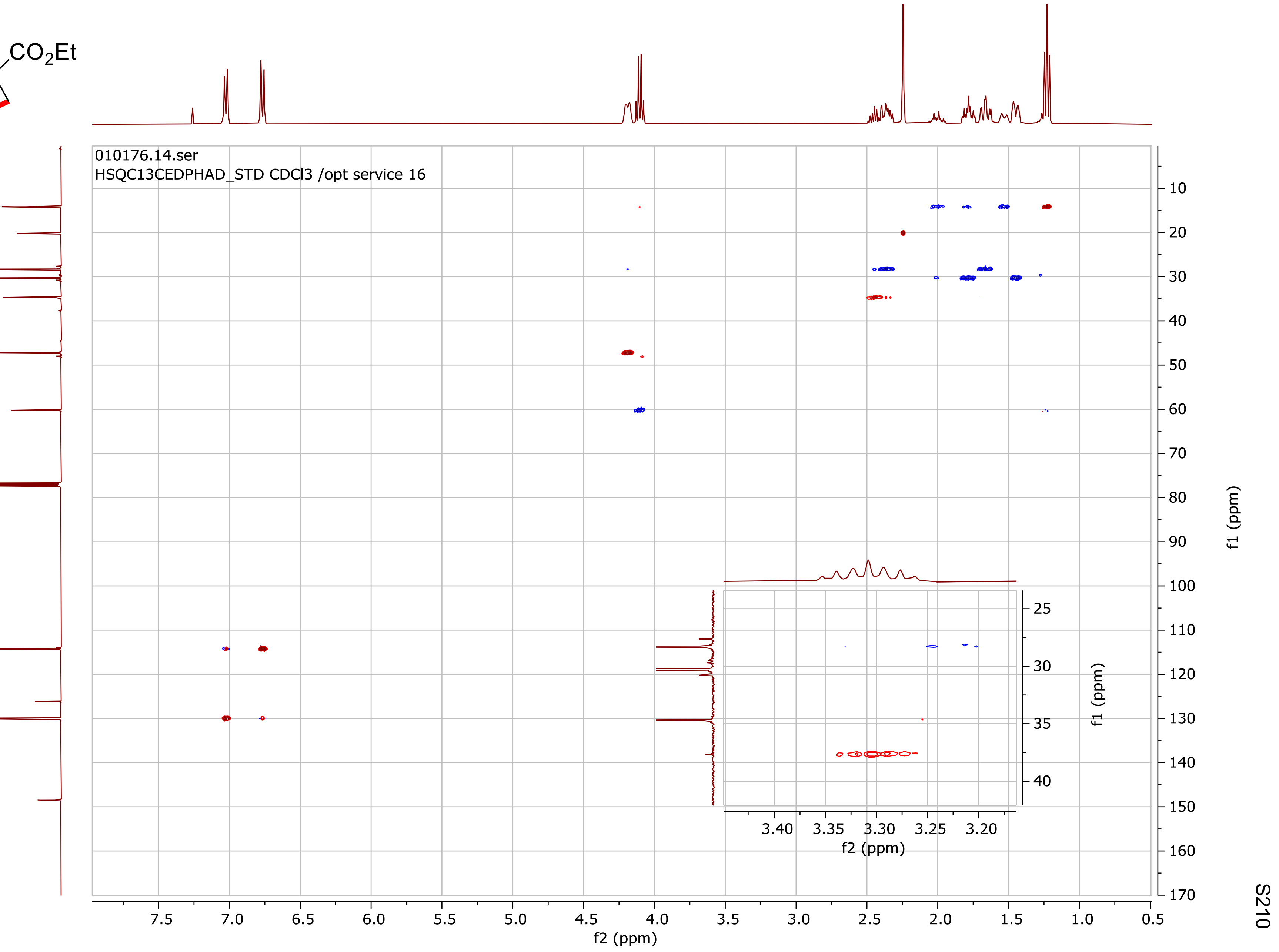

Ethyl 9-(p-tolyl)-9-azabicyclo[3.3.1]nonane-3-carboxylate (**8d**)

^{1}H, ^{13}C-HMBC NMR (400 MHz, $CDCl_3$)

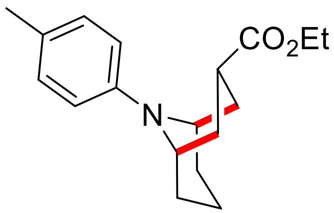

8d

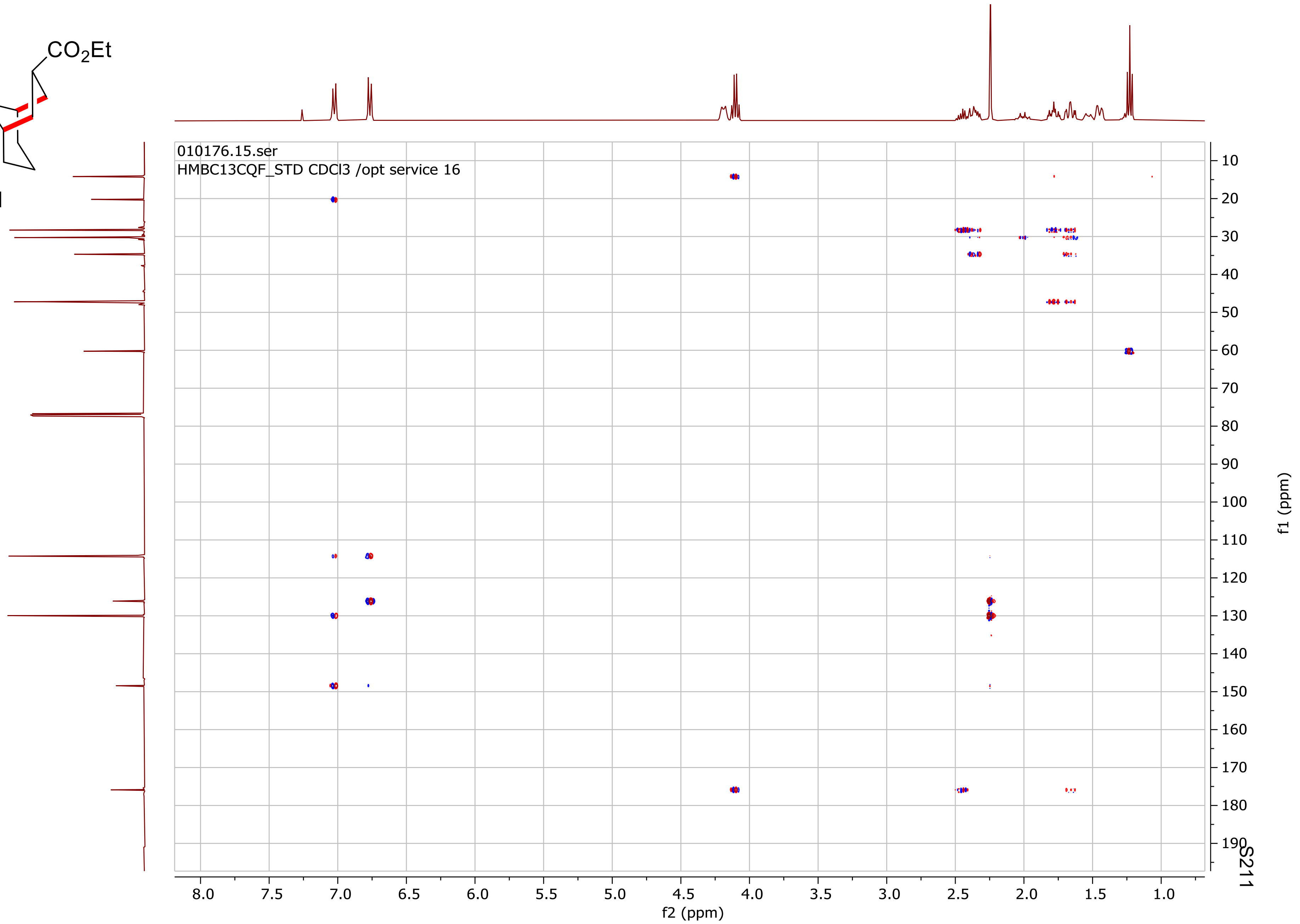

Ethyl 9-(p-tolyl)-9-azabicyclo[3.3.1]nonane-3-carboxylate (**8dα**)

FT-IR, ATR-diamond

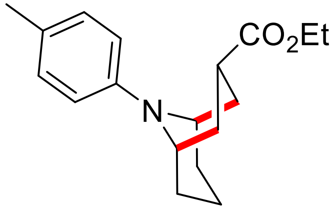

8d

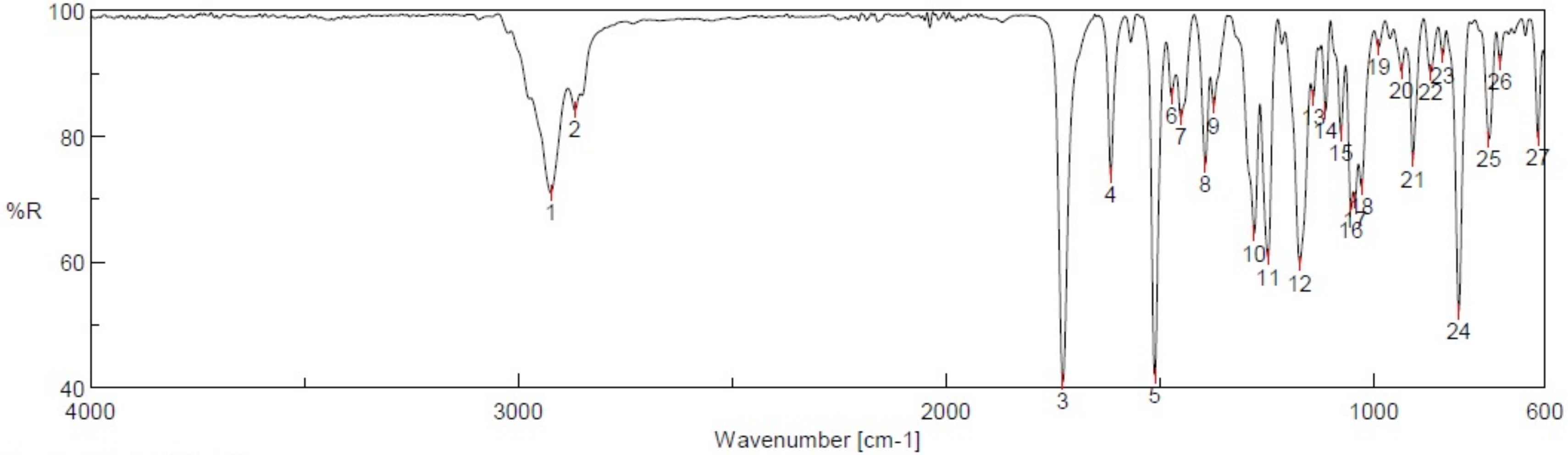

[Result of Peak Picking]

No.	Position	Intensity	No.	Position	Intensity	No.	Position	Intensity
1	2923.56	70.9805	2	2867.63	84.1265	3	1726.94	40.8735
4	1615.09	73.8606	5	1511.92	41.9441	6	1472.38	86.3378
7	1451.17	82.9867	8	1394.28	75.495	9	1374.03	84.8459
10	1279.54	64.5047	11	1246.75	60.703	12	1173.47	59.756
13	1141.65	86.2091	14	1112.73	84.0182	15	1077.05	80.3811
16	1053.91	68.2717	17	1044.26	69.7823	18	1028.84	71.9668
19	989.304	94.134	20	935.306	90.3184	21	908.308	76.3096
22	866.846	90.1498	23	838.883	92.8614	24	801.278	52.0821
25	730.889	79.5515	26	704.855	91.8059	27	616.145	79.6434

Ethyl 9-(4-methoxyphenyl)-9-azabicyclo[3.3.1]nonane-3-carboxylate (**8e**) **α/β >20:1**

^{1}H-NMR (300 MHz, $CDCl_3$)

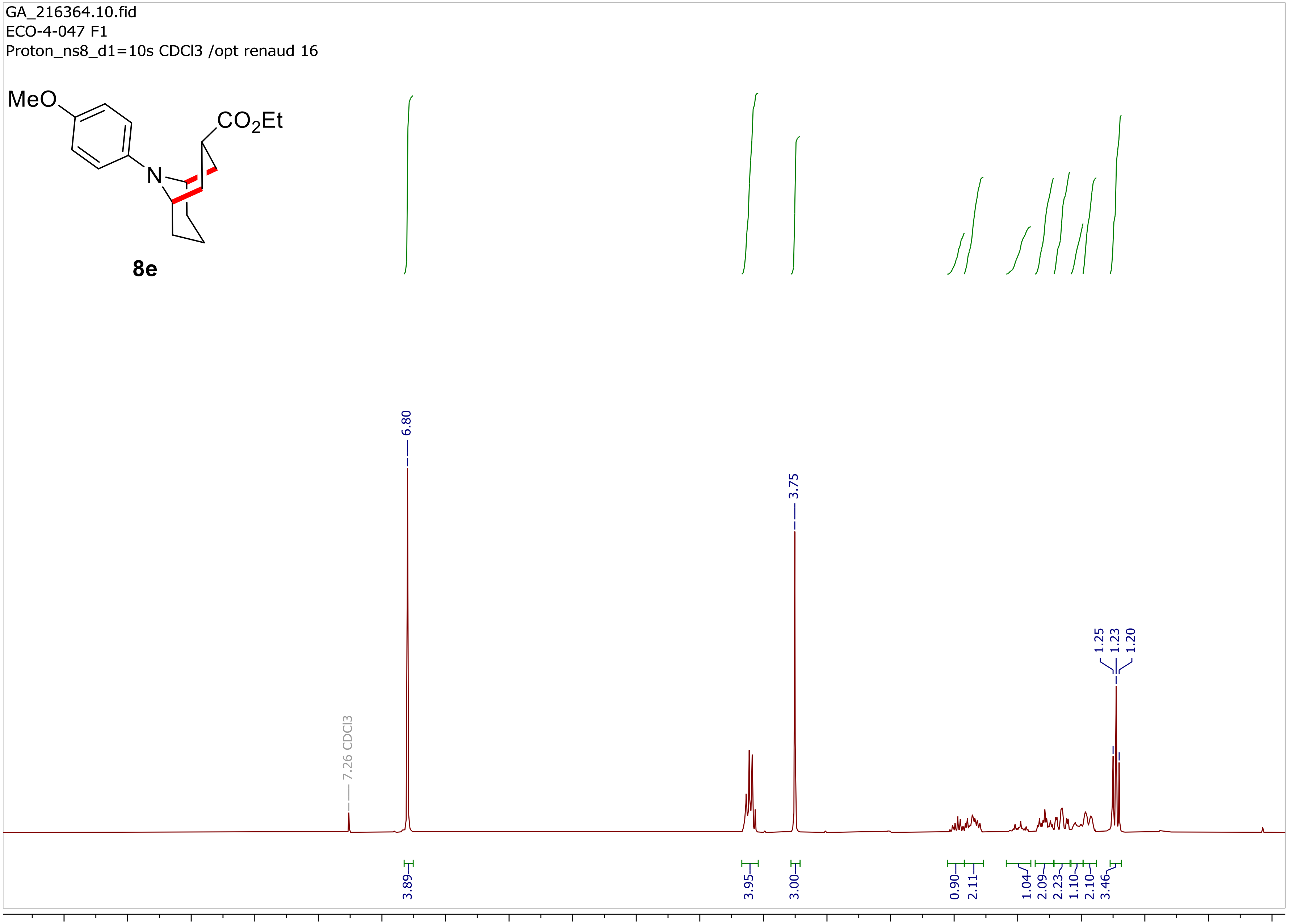

Ethyl 9-(4-methoxyphenyl)-9-azabicyclo[3.3.1]nonane-3-carboxylate (**8eα**)

^{1}H-NMR (300 MHz, $CDCl_3$)

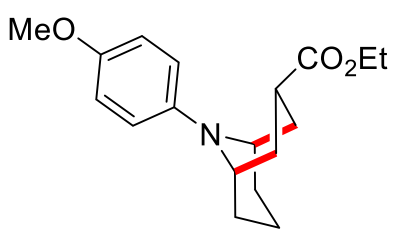

8e

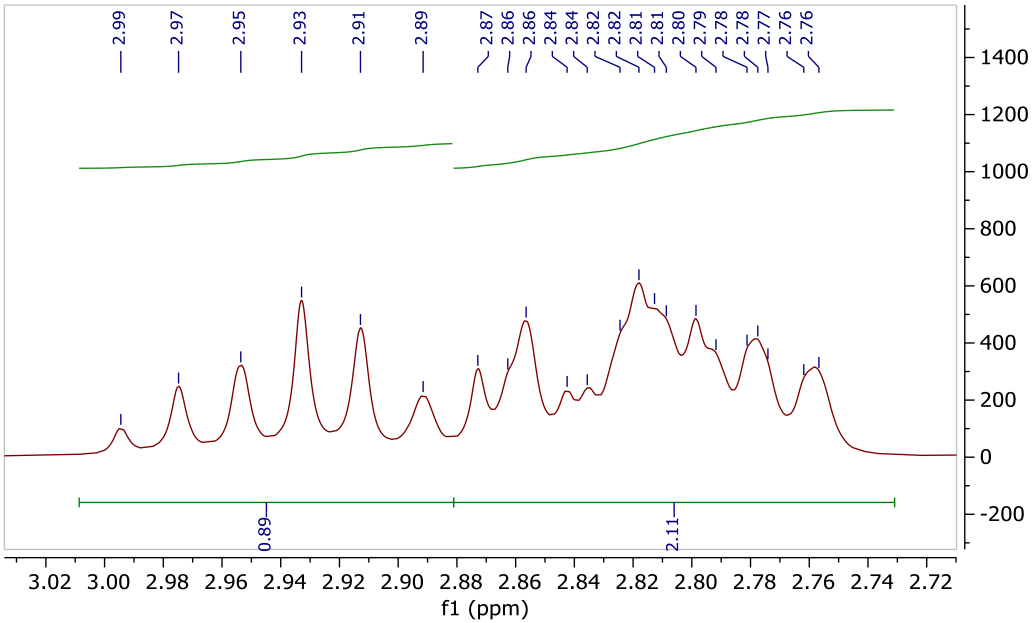

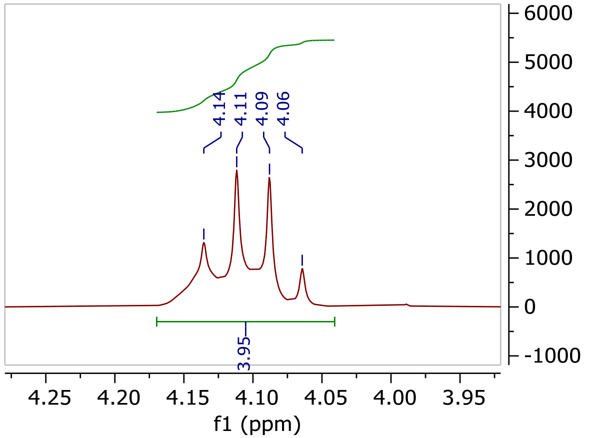

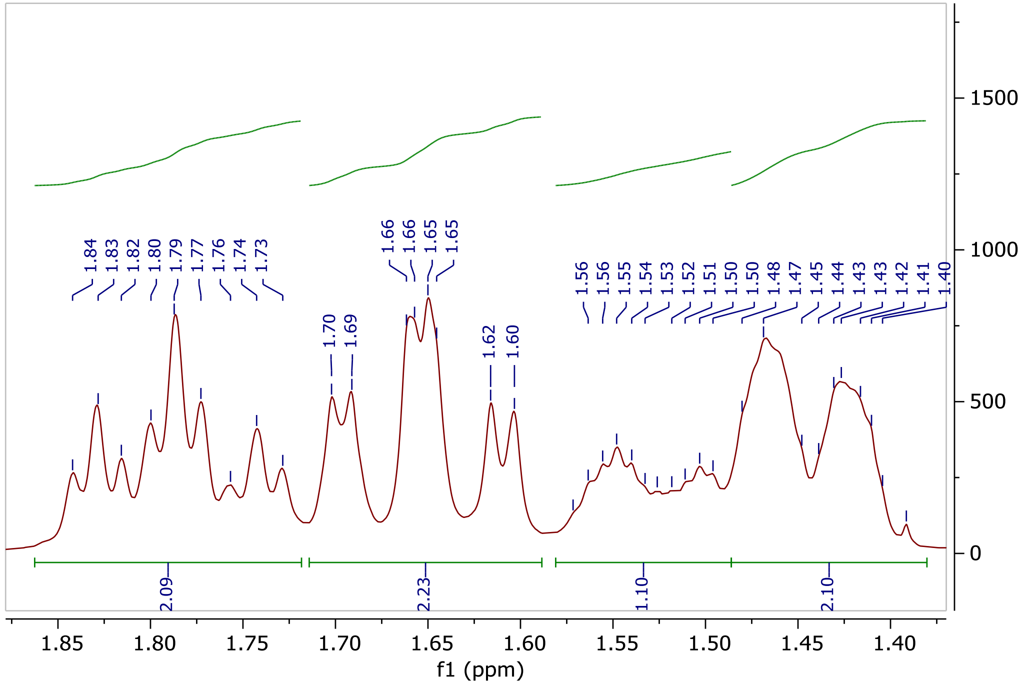

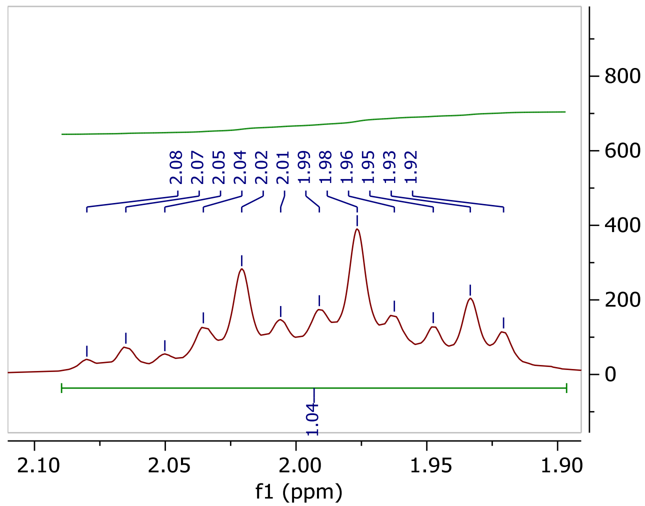

Ethyl 9-(4-methoxyphenyl)-9-azabicyclo[3.3.1]nonane-3-carboxylate (**8eα**)

^{13}C-NMR (101 MHz, $CDCl_3$)

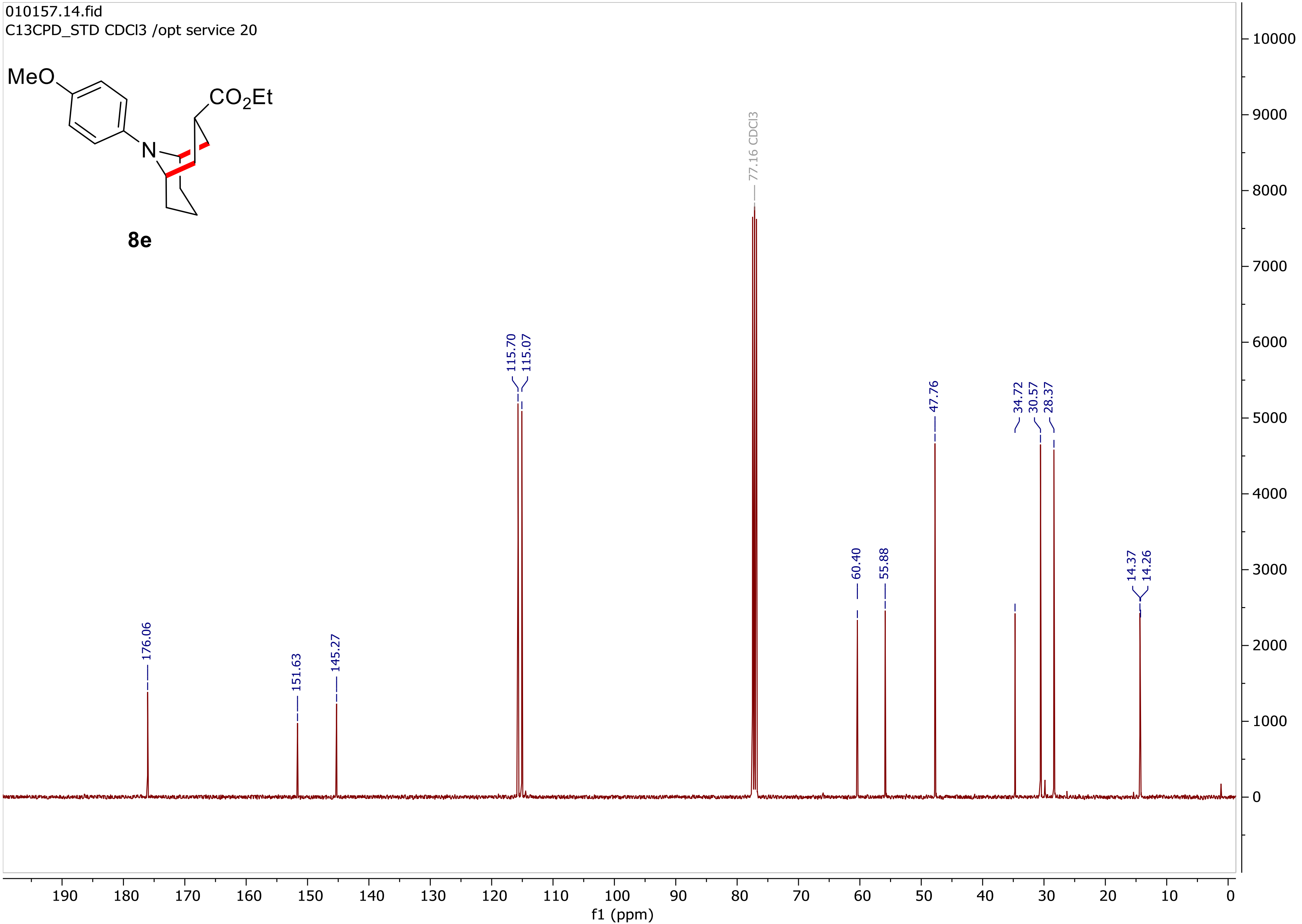

Ethyl 9-(4-methoxyphenyl)-9-azabicyclo[3.3.1]nonane-3-carboxylate (**8eα**)

^{13}C-NMR (101 MHz, $CDCl_3$)

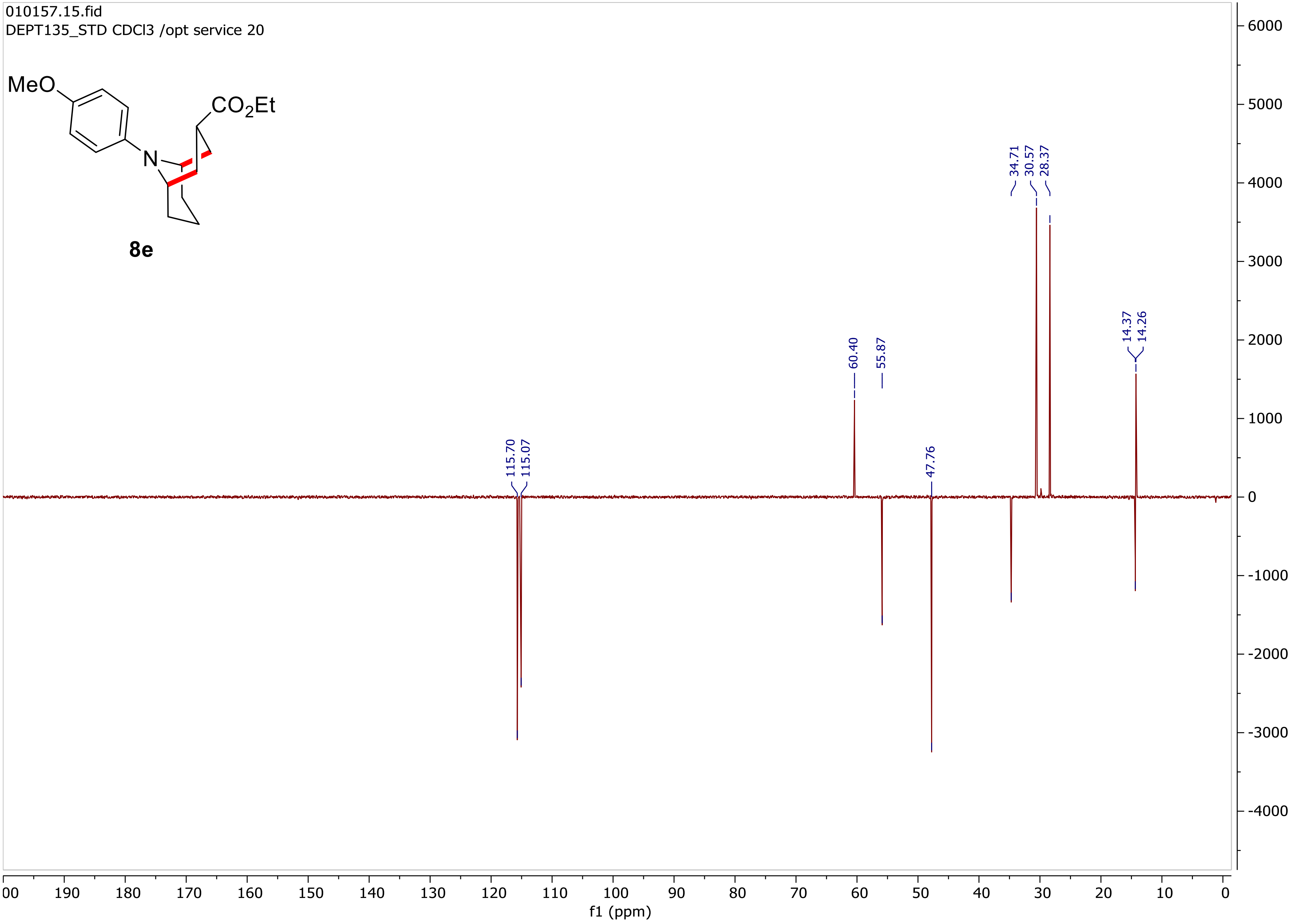

Ethyl 9-(4-methoxyphenyl)-9-azabicyclo[3.3.1]nonane-3-carboxylate (**8eα**)

^{1}H, ^{1}H-COSY NMR (400 MHz, $CDCl_3$)

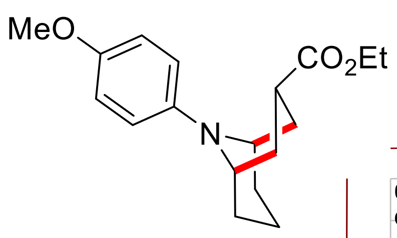

8e

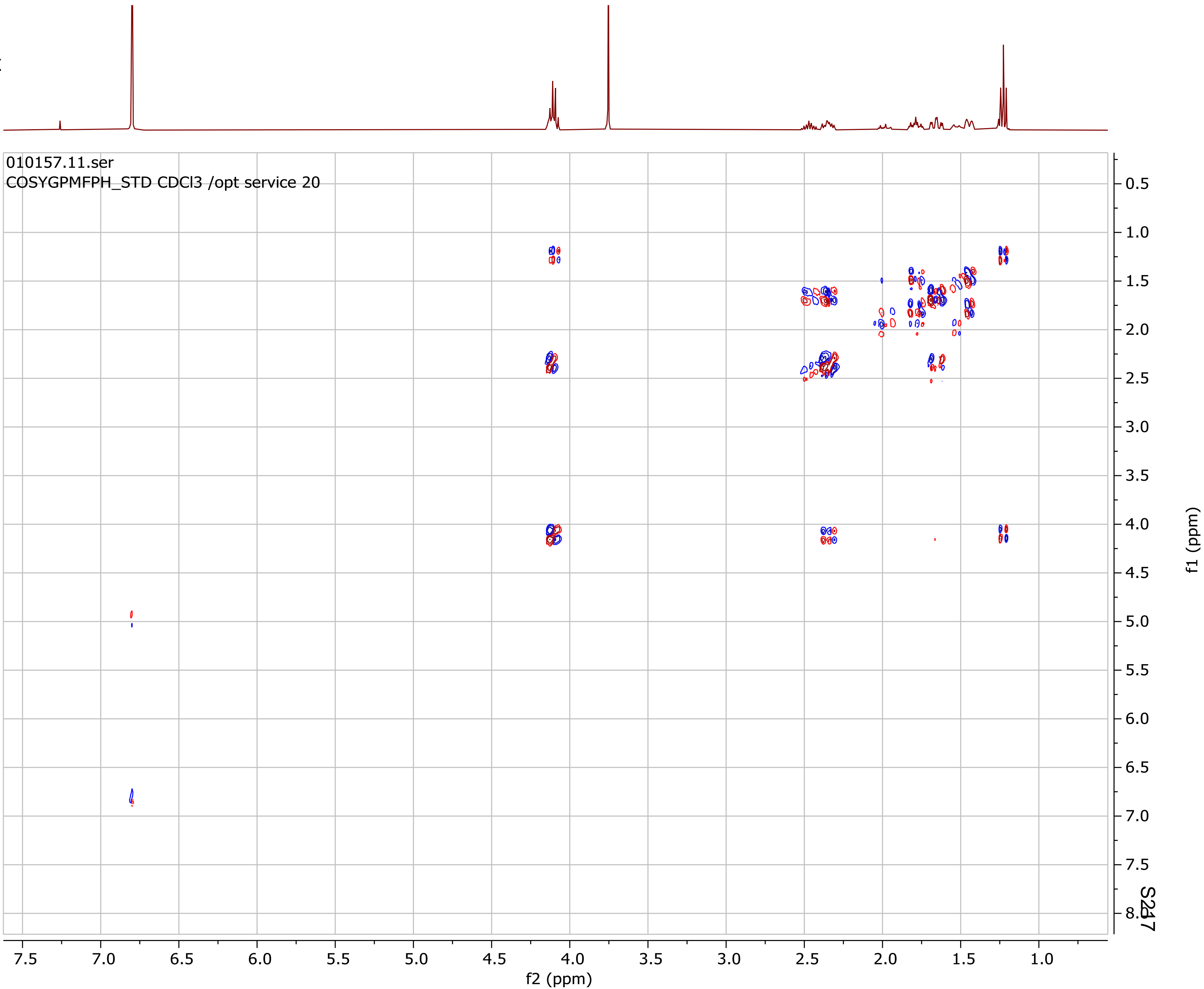

Ethyl 9-(4-methoxyphenyl)-9-azabicyclo[3.3.1]nonane-3-carboxylate (**8eα**)

^{1}H, ^{13}C-HSQC NMR (400 MHz, $CDCl_3$)

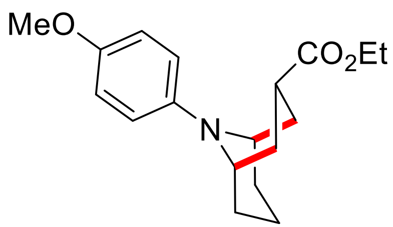

8e

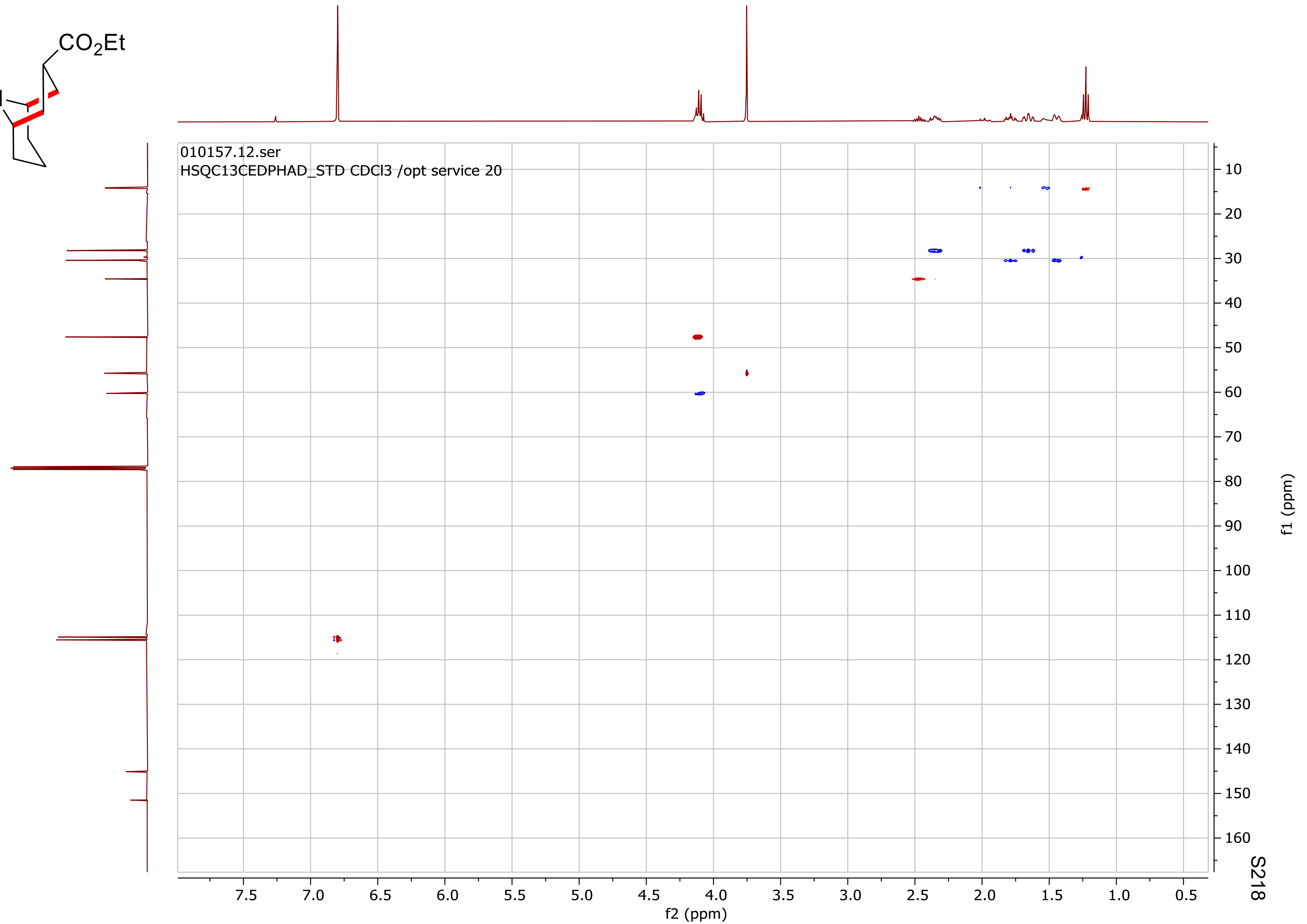

Ethyl 9-(4-methoxyphenyl)-9-azabicyclo[3.3.1]nonane-3-carboxylate (**8eα**)

^{1}H, ^{13}C-HMBC NMR (400 MHz, $CDCl_3$)

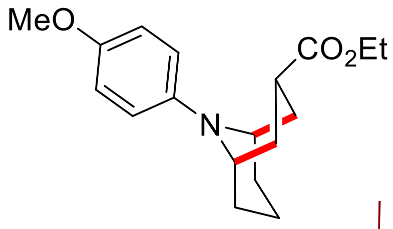

8e

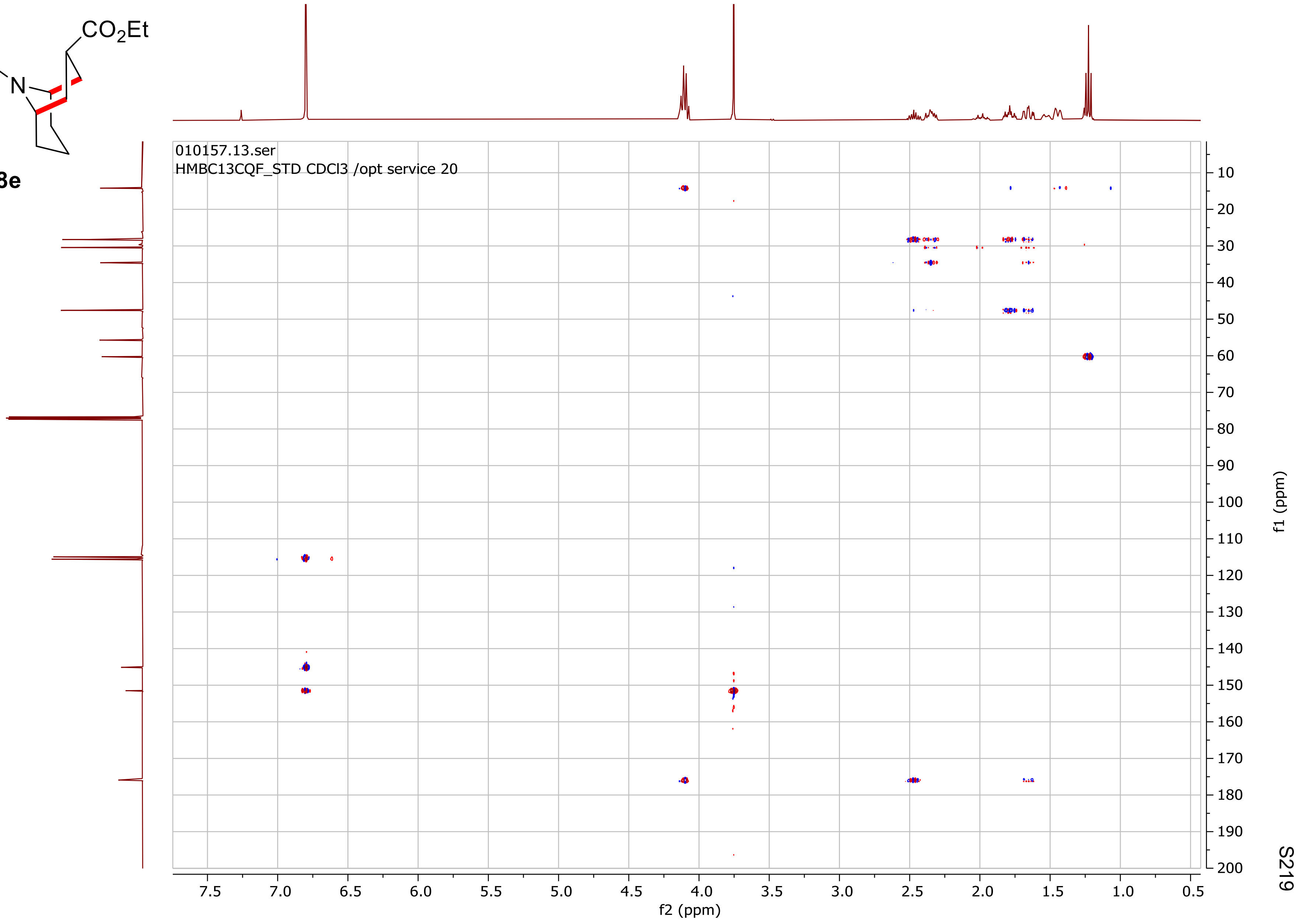

Ethyl 9-(4-methoxyphenyl)-9-azabicyclo[3.3.1]nonane-3-carboxylate (**8eα**)

FT-IR, ATR-diamond

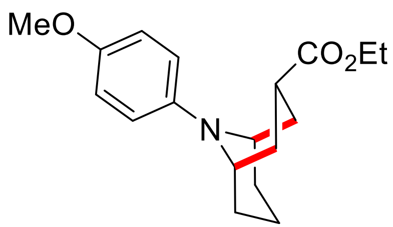

8e

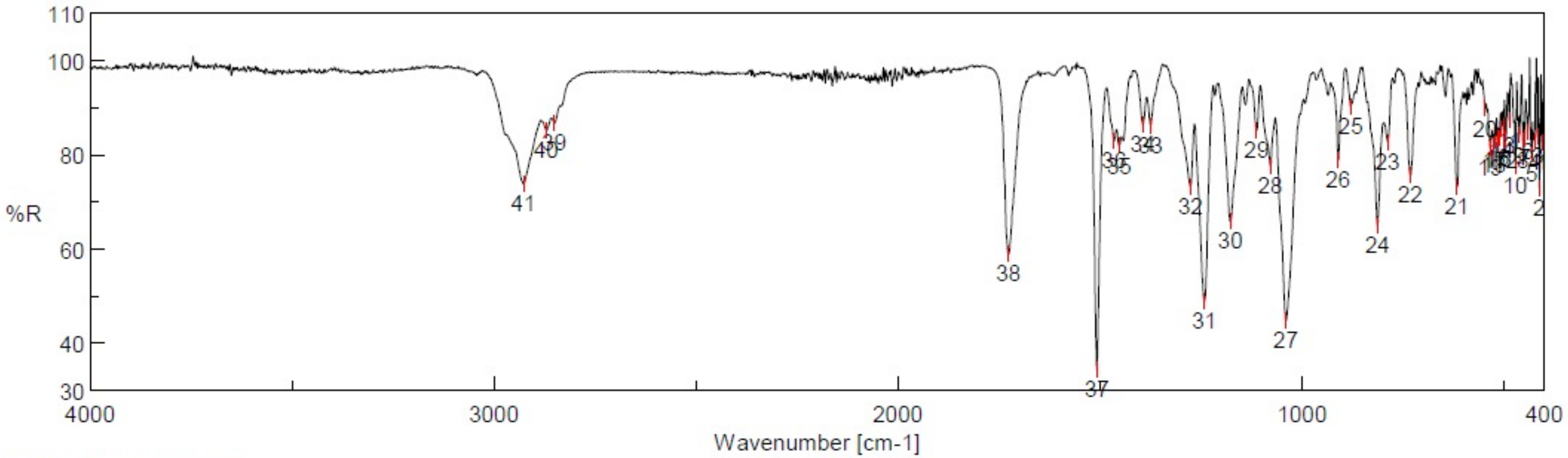

[Result of Peak Picking]

No.	Position	Intensity	No.	Position	Intensity	No.	Position	Intensity
1	404.978	82.6494	2	412.21	72.8732	3	417.031	83.7879
4	424.263	82.7849	5	431.012	80.1616	6	441.137	84.9468
7	448.851	83.6182	8	452.708	82.859	9	461.386	84.0746
10	471.51	77.524	11	486.456	87.2343	12	495.134	83.1342
13	501.401	86.1942	14	505.74	85.3827	15	511.526	83.4662
16	515.865	83.7578	17	521.65	83.0959	18	525.507	82.0142
19	534.185	81.4103	20	546.72	89.7613	21	616.627	73.1135
22	731.371	75.6542	23	787.779	82.7656	24	812.849	65.038
25	879.381	90.2278	26	910.236	78.8728	27	1039.44	44.7472
28	1078.01	77.6018	29	1112.24	85.1548	30	1177.33	65.851
31	1241.45	48.9282	32	1276.16	73.257	33	1374.51	86.2666
34	1393.8	86.4034	35	1452.14	81.8764	36	1465.15	82.8791
37	1507.1	34.3257	38	1725.98	58.9351	39	2850.27	86.7569
40	2870.04	85.1461	41	2926.93	73.7861			

Ethyl 9-(4-bromophenyl)-9-azabicyclo[3.3.1]nonane-3-carboxylate (**8f**) **α/β >20:1**

^{1}H-NMR (300 MHz, $CDCl_3$)

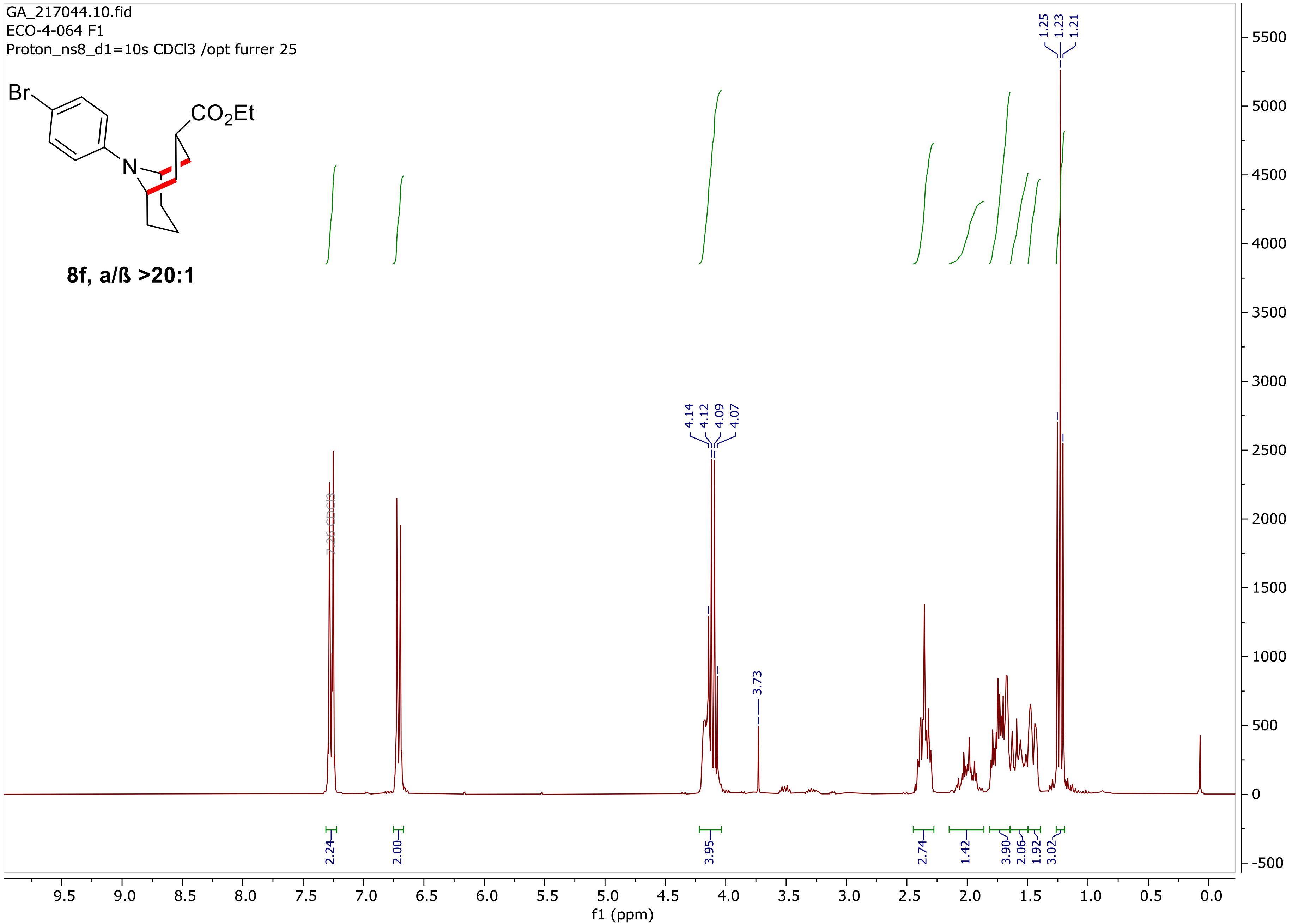

Ethyl 9-(4-bromophenyl)-9-azabicyclo[3.3.1]nonane-3-carboxylate (**8fα**)

^{1}H-NMR (300 MHz, $CDCl_3$)

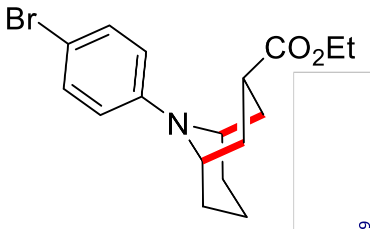

8f

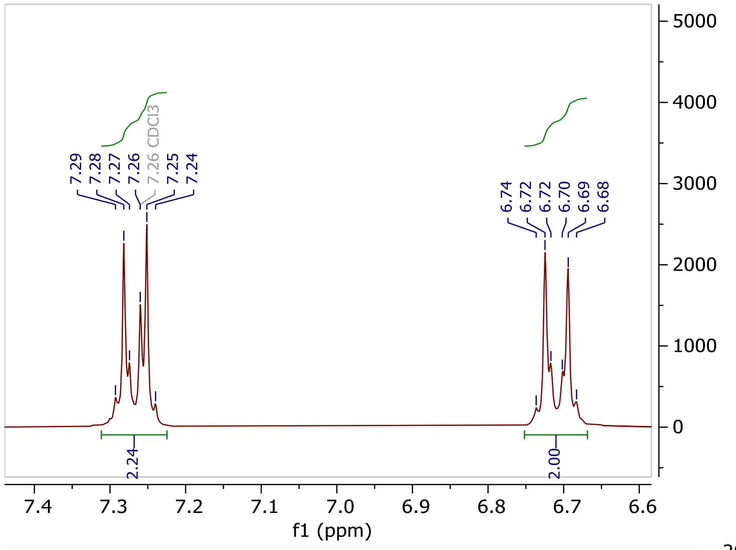

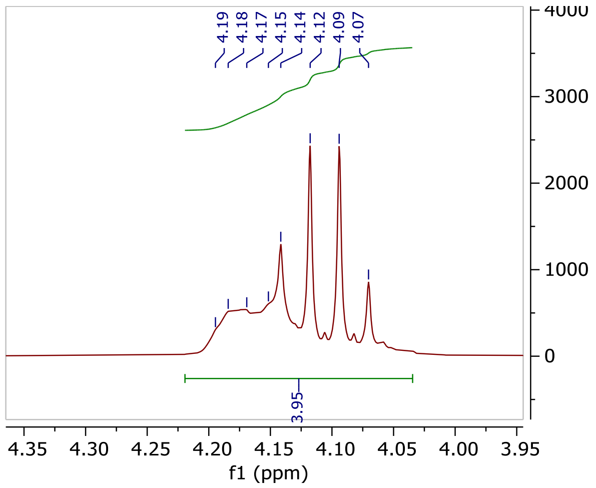

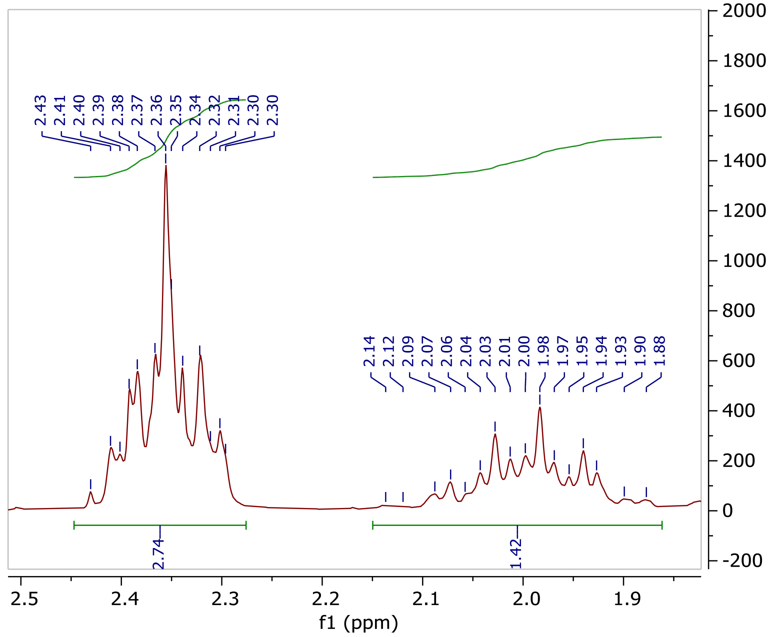

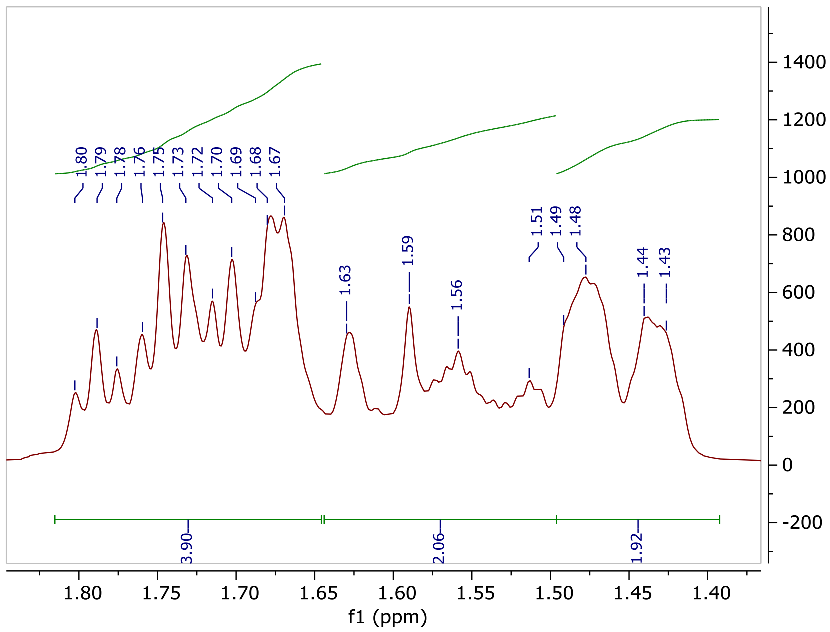

Ethyl 9-(4-bromophenyl)-9-azabicyclo[3.3.1]nonane-3-carboxylate (**8fα**)

^{13}C-NMR (75 MHz, $CDCl_3$)

GA_217044.11.fid
Carbon_ns512 CDCl3 /opt renaud 25

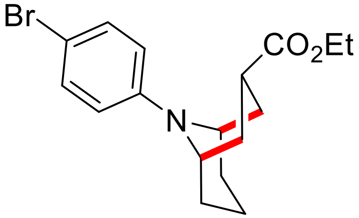

8f

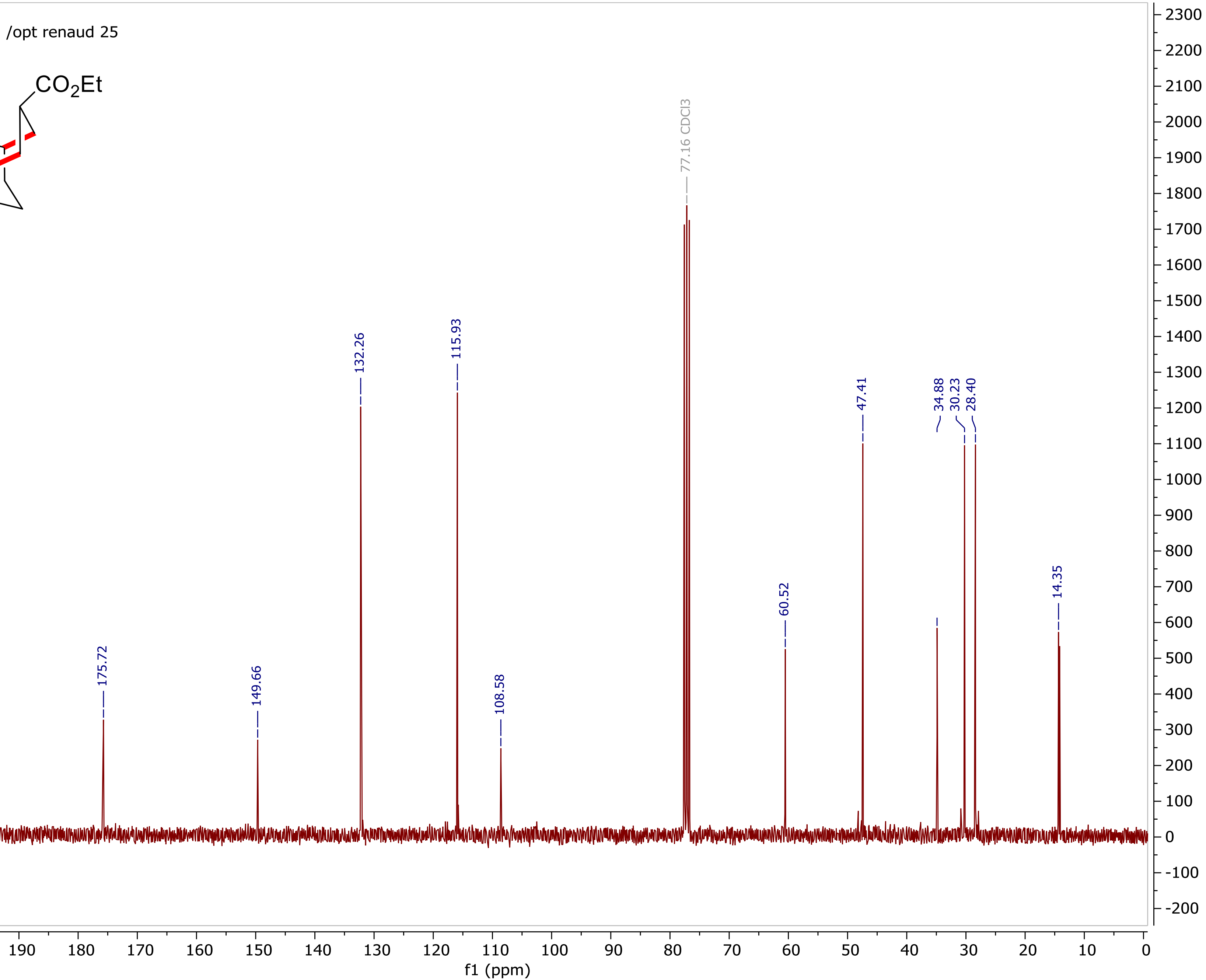

Ethyl 9-(4-bromophenyl)-9-azabicyclo[3.3.1]nonane-3-carboxylate (**8fα**)

^{13}C-NMR (75 MHz, $CDCl_3$)

GA_217044.12.fid
Dept135_ns512 CDCl3 /opt renaud 25

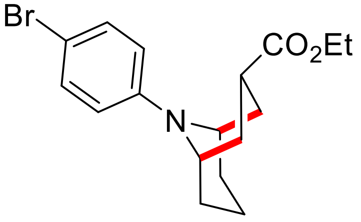

8f

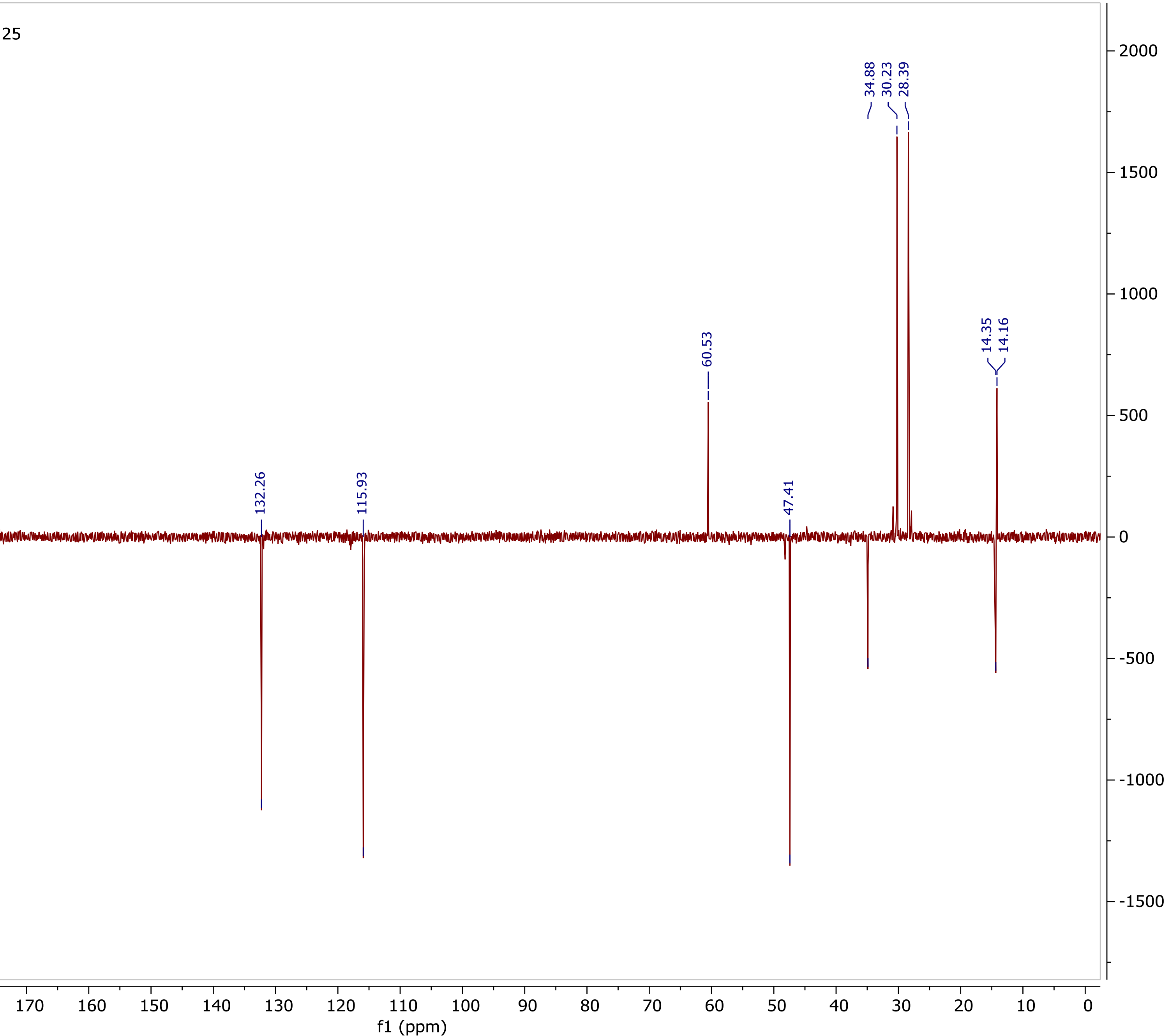

Ethyl 9-(4-bromophenyl)-9-azabicyclo[3.3.1]nonane-3-carboxylate (**8fα**)

^1H, ^1H-COSY NMR (400 MHz, $CDCl_3$)

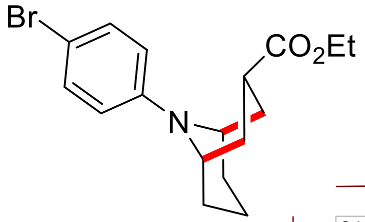

8f

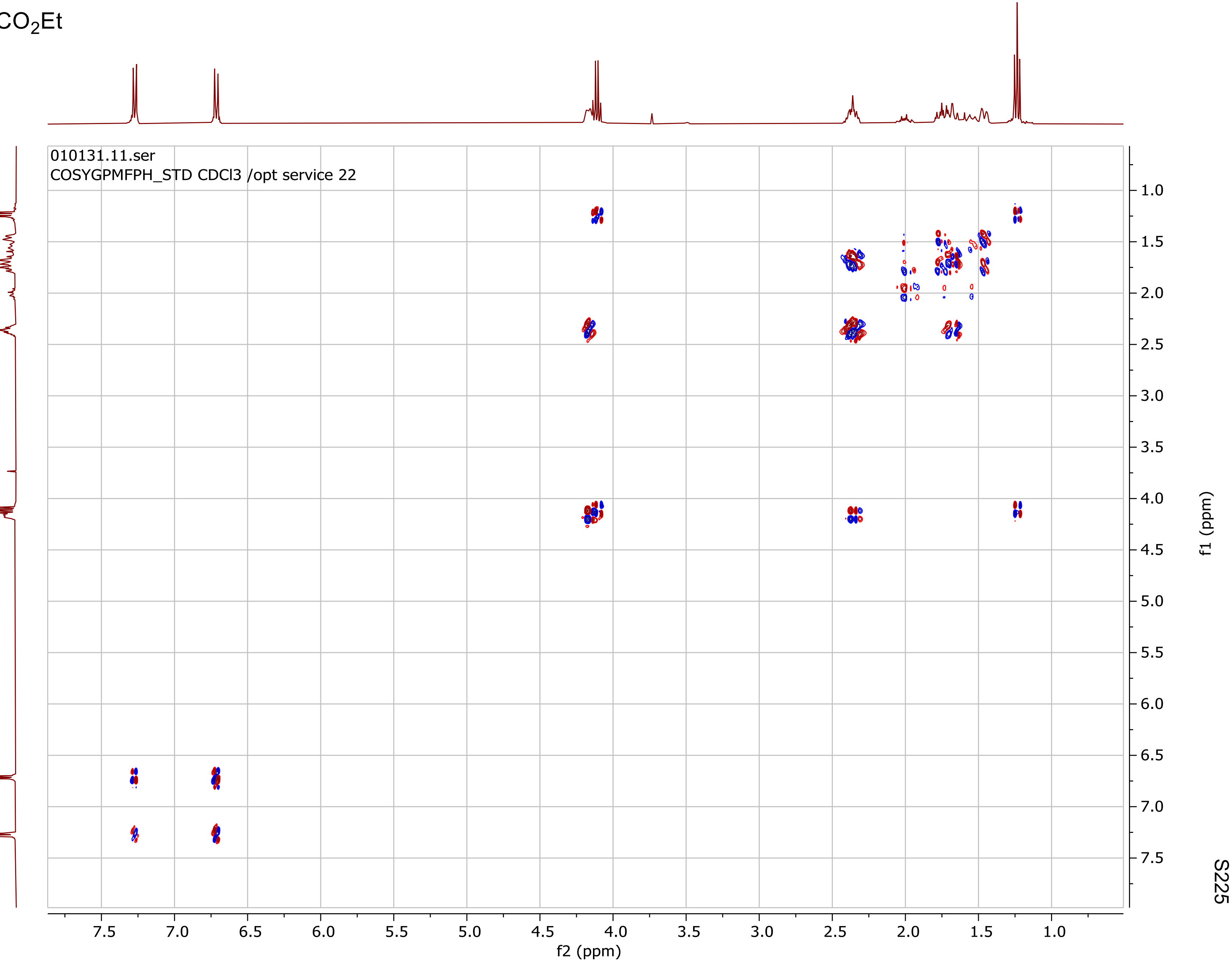

Ethyl 9-(4-bromophenyl)-9-azabicyclo[3.3.1]nonane-3-carboxylate (**8fα**)

^{1}H, ^{13}C-HSQC NMR (400 MHz, $CDCl_3$)

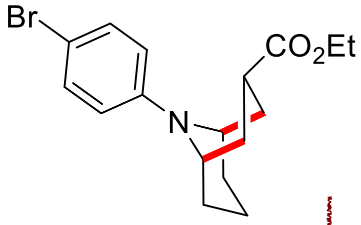

8f

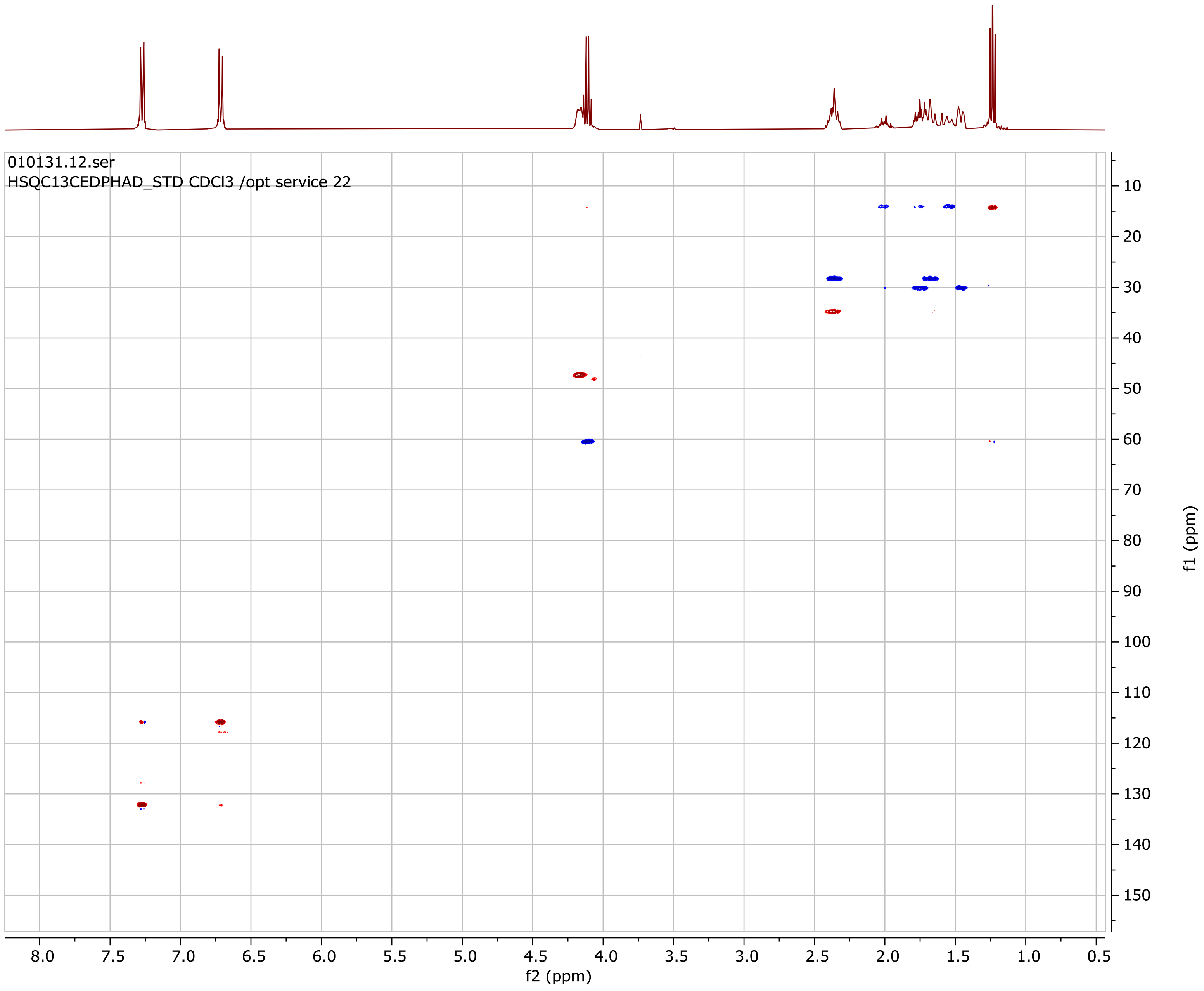

Ethyl 9-(4-bromophenyl)-9-azabicyclo[3.3.1]nonane-3-carboxylate (**8fα**)

^{1}H, ^{13}C-HMBC NMR (400 MHz, $CDCl_3$)

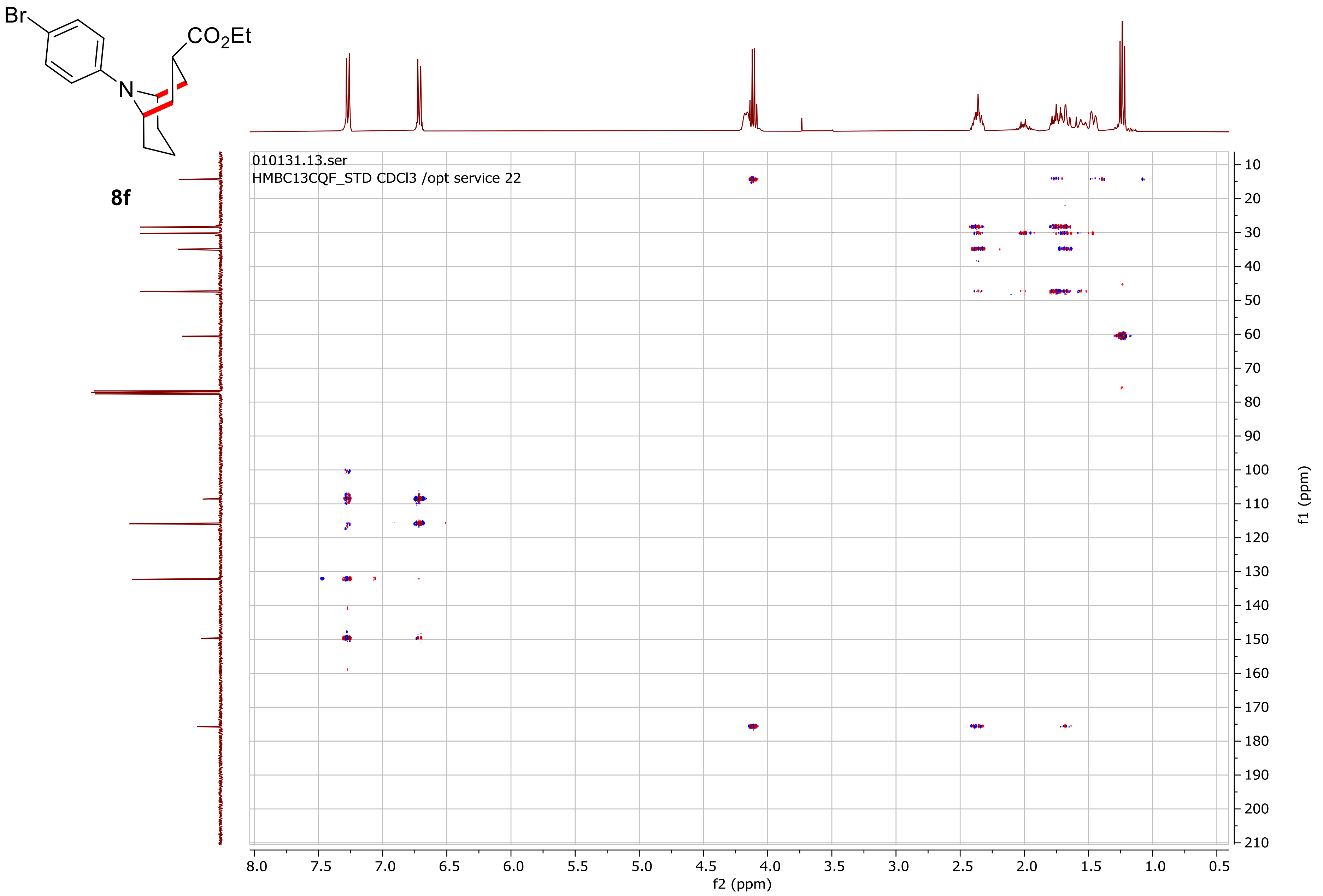

Ethyl 9-(4-bromophenyl)-9-azabicyclo[3.3.1]nonane-3-carboxylate (**8fα**)

FT-IR, ATR-diamond

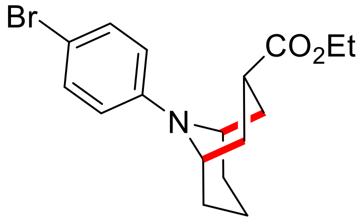

8f

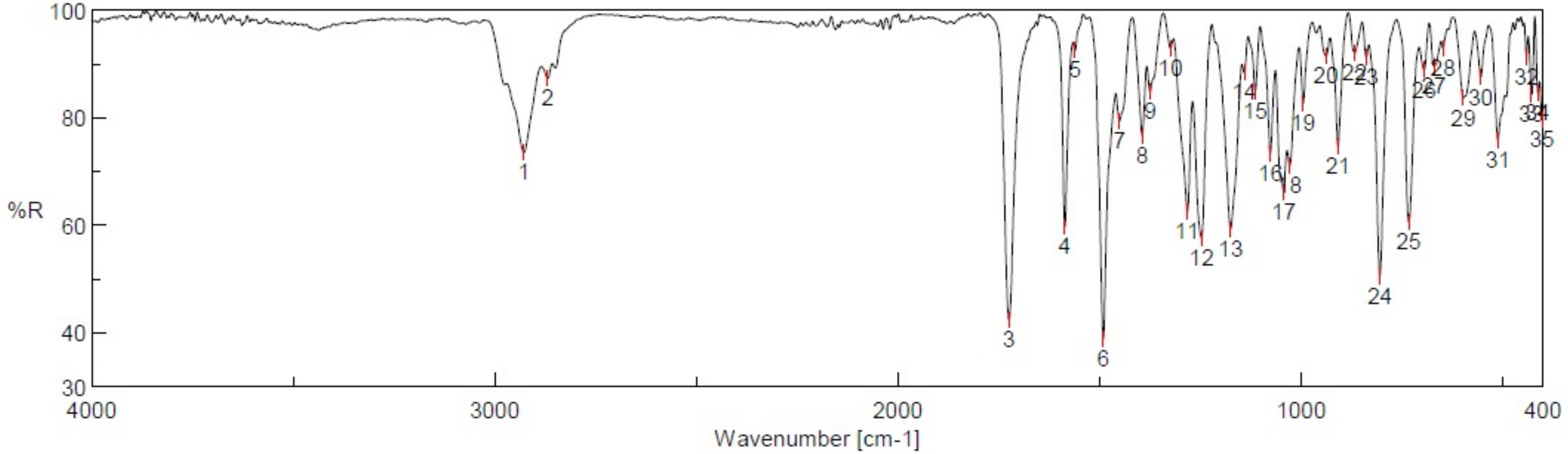

[Result of Peak Picking]

No.	Position	Intensity	No.	Position	Intensity	No.	Position	Intensity
1	2927.41	73.5286	2	2868.59	87.3571	3	1724.05	42.325
4	1586.16	59.8108	5	1560.13	92.549	6	1490.7	38.8652
7	1450.21	79.4177	8	1394.28	76.7312	9	1375	84.831
10	1323.89	92.9503	11	1282.43	62.5026	12	1246.75	57.603
13	1174.44	59.2677	14	1140.69	88.389	15	1113.69	85.0179
16	1076.08	73.3118	17	1043.3	66.2541	18	1027.87	71.0993
19	995.089	82.664	20	938.199	91.4425	21	908.308	74.6289
22	866.846	92.1217	23	838.883	91.3081	24	804.171	50.5513
25	732.817	60.6482	26	695.212	88.9143	27	671.106	89.6374
28	647.965	92.9559	29	597.825	83.6847	30	554.434	87.5573
31	512.008	75.6822	32	440.655	91.1946	33	428.12	84.3584
34	412.692	84.5218	35	402.085	79.6525			

Ethyl 9-(4-methoxycarbonylphenyl)-9-azabicyclo[3.3.1]nonane-3-carboxylate (**8g**) **α/β >20:1**

^{1}H-NMR (300 MHz, $CDCl_3$)

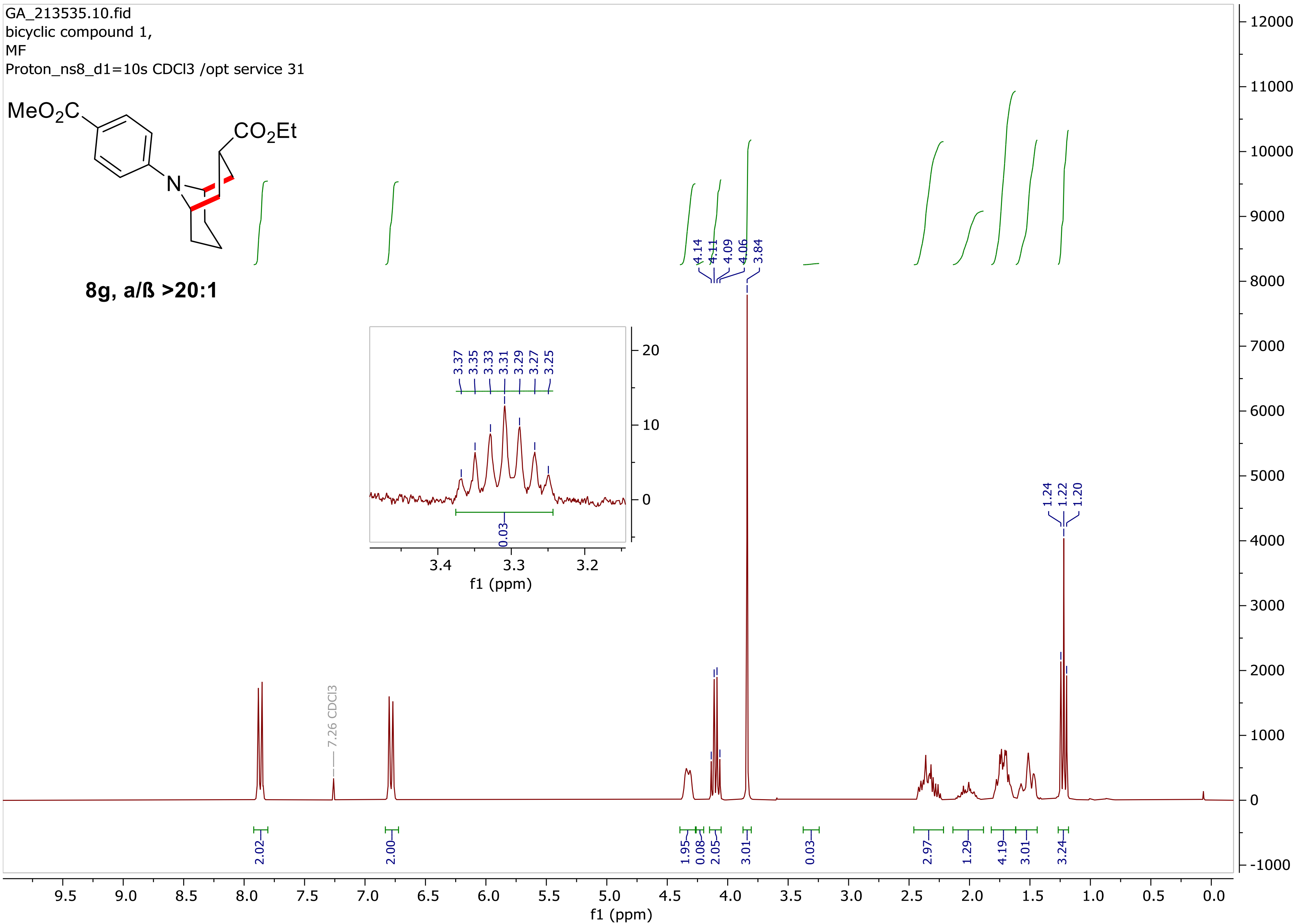

Ethyl 9-(4-methoxycarbonylphenyl)-9-azabicyclo[3.3.1]nonane-3-carboxylate (**8g**) **α/β >20:1**

^{1}H-NMR (300 MHz, $CDCl_3$)

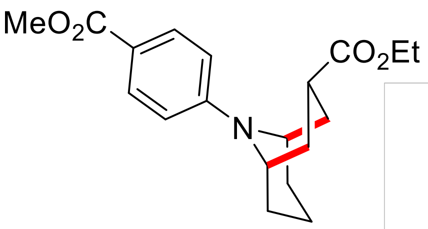

8g, a/ß >20:1

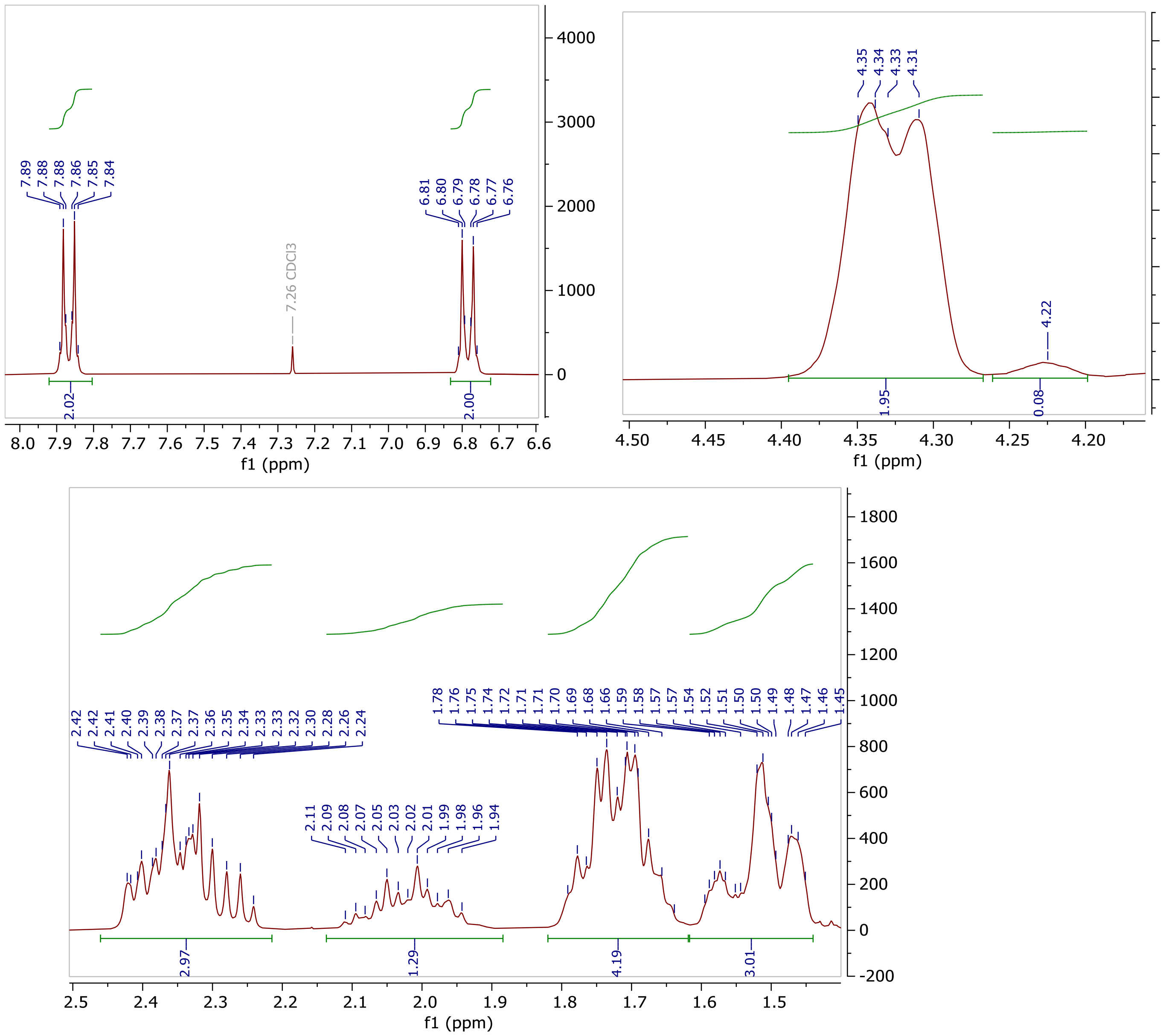

Ethyl 9-(4-methoxycarbonylphenyl)-9-azabicyclo[3.3.1]nonane-3-carboxylate (**8gα**)

^{13}C-NMR (75 MHz, $CDCl_3$)

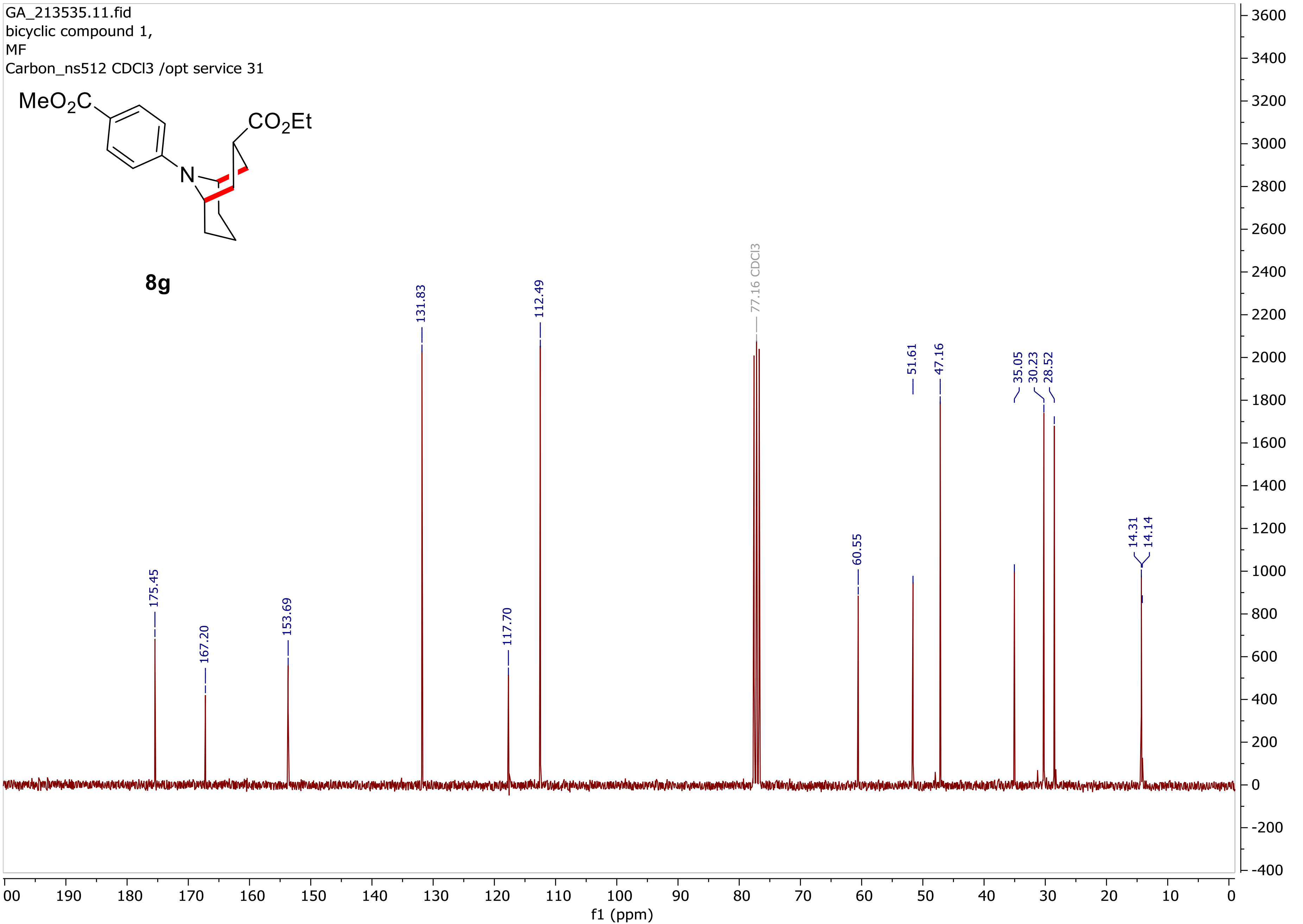

Ethyl 9-(4-methoxycarbonylphenyl)-9-azabicyclo[3.3.1]nonane-3-carboxylate (**8gα**)

^{13}C-NMR (75 MHz, $CDCl_3$)

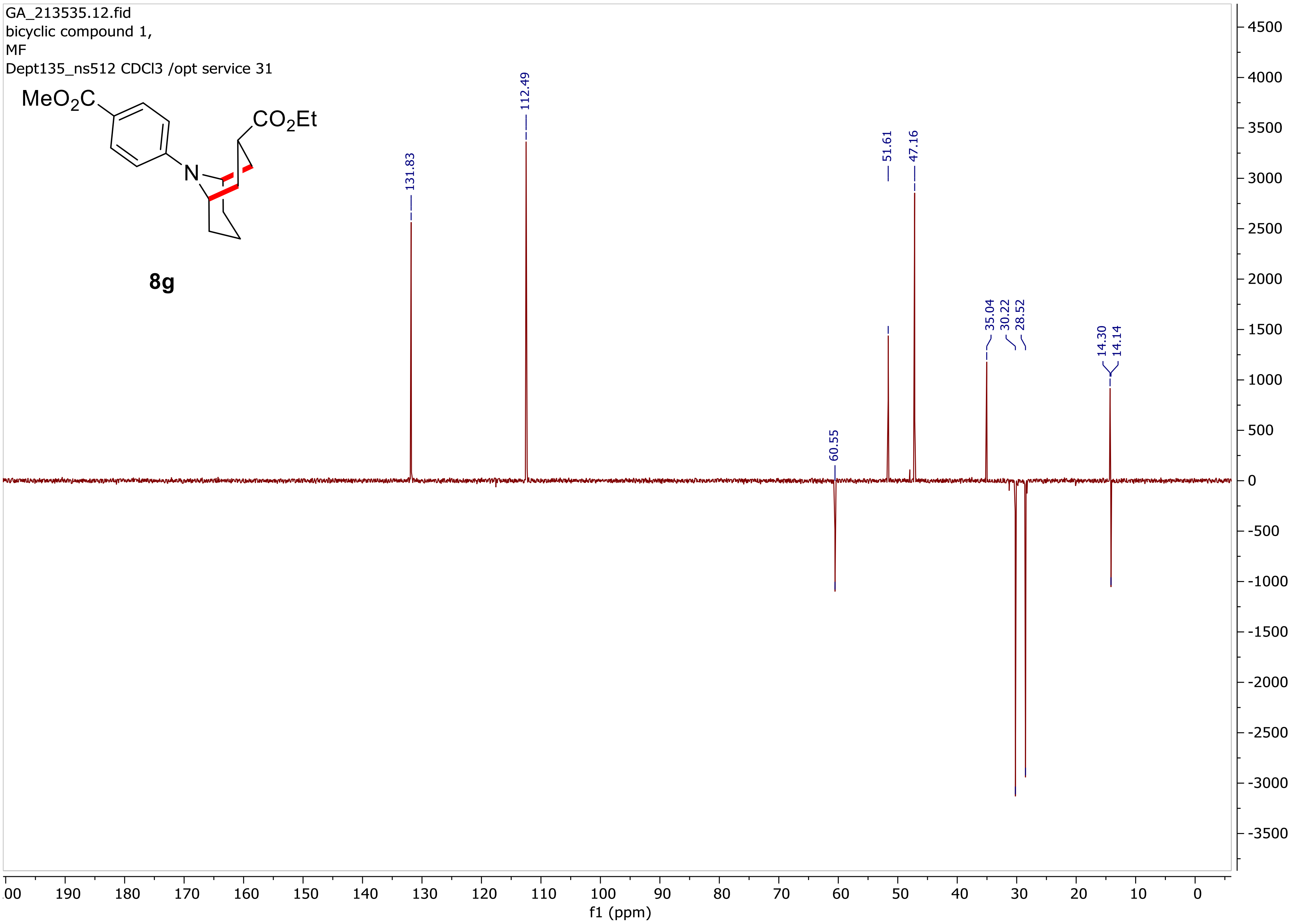

Ethyl 9-(4-methoxycarbonylphenyl)-9-azabicyclo[3.3.1]nonane-3-carboxylate (**8gα**)

^{1}H, ^{1}H-COSY NMR (400 MHz, $CDCl_3$)

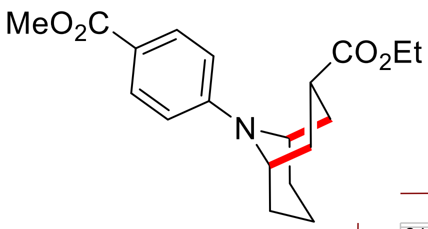

8g

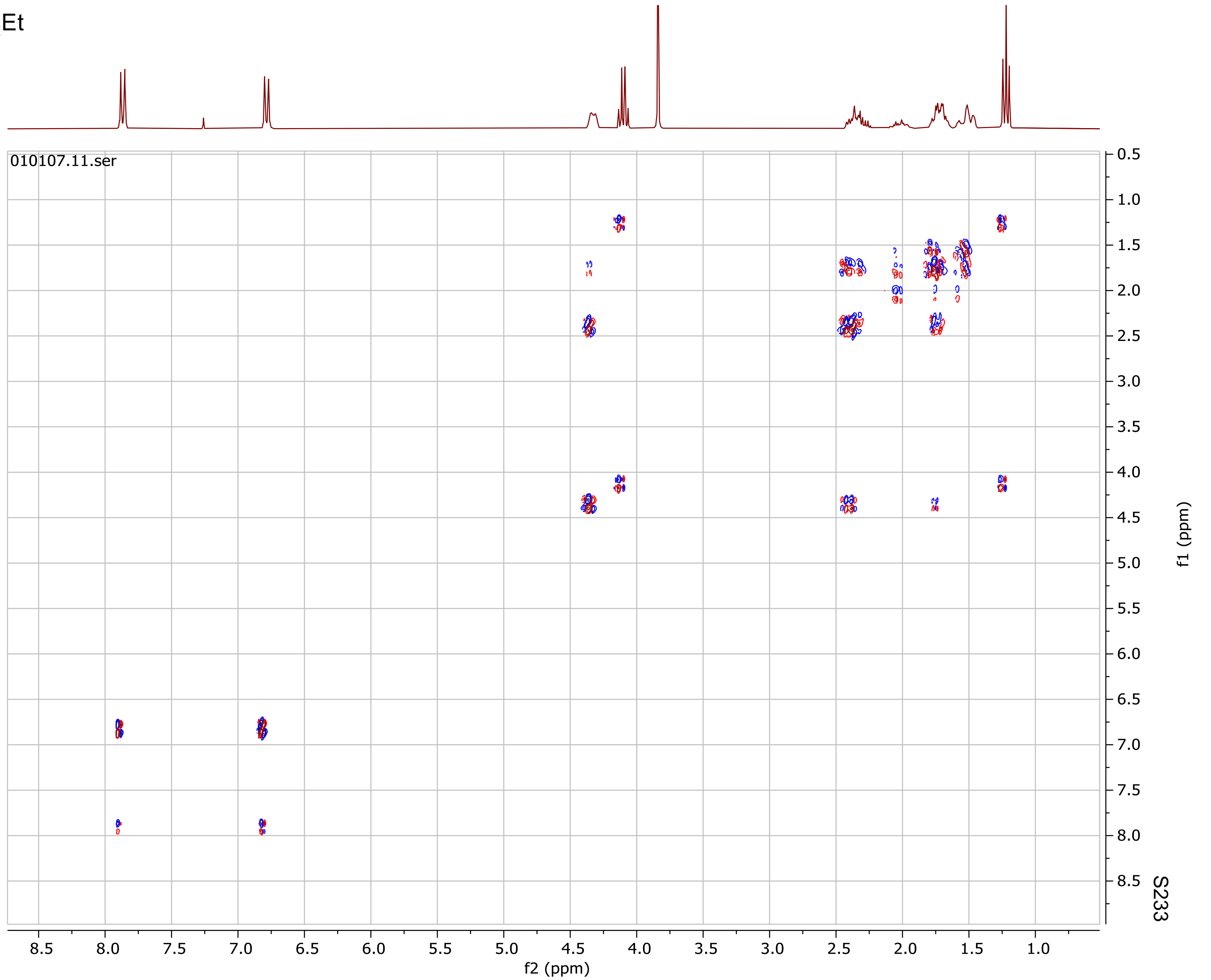

Ethyl 9-(4-methoxycarbonylphenyl)-9-azabicyclo[3.3.1]nonane-3-carboxylate (**8gα**)

^1H, ^{13}C-HSQC NMR (400 MHz, $CDCl_3$)

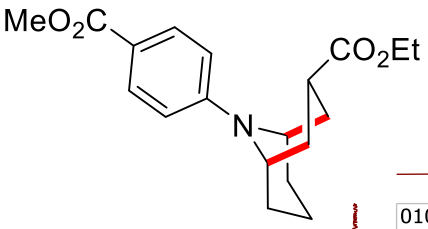

8g

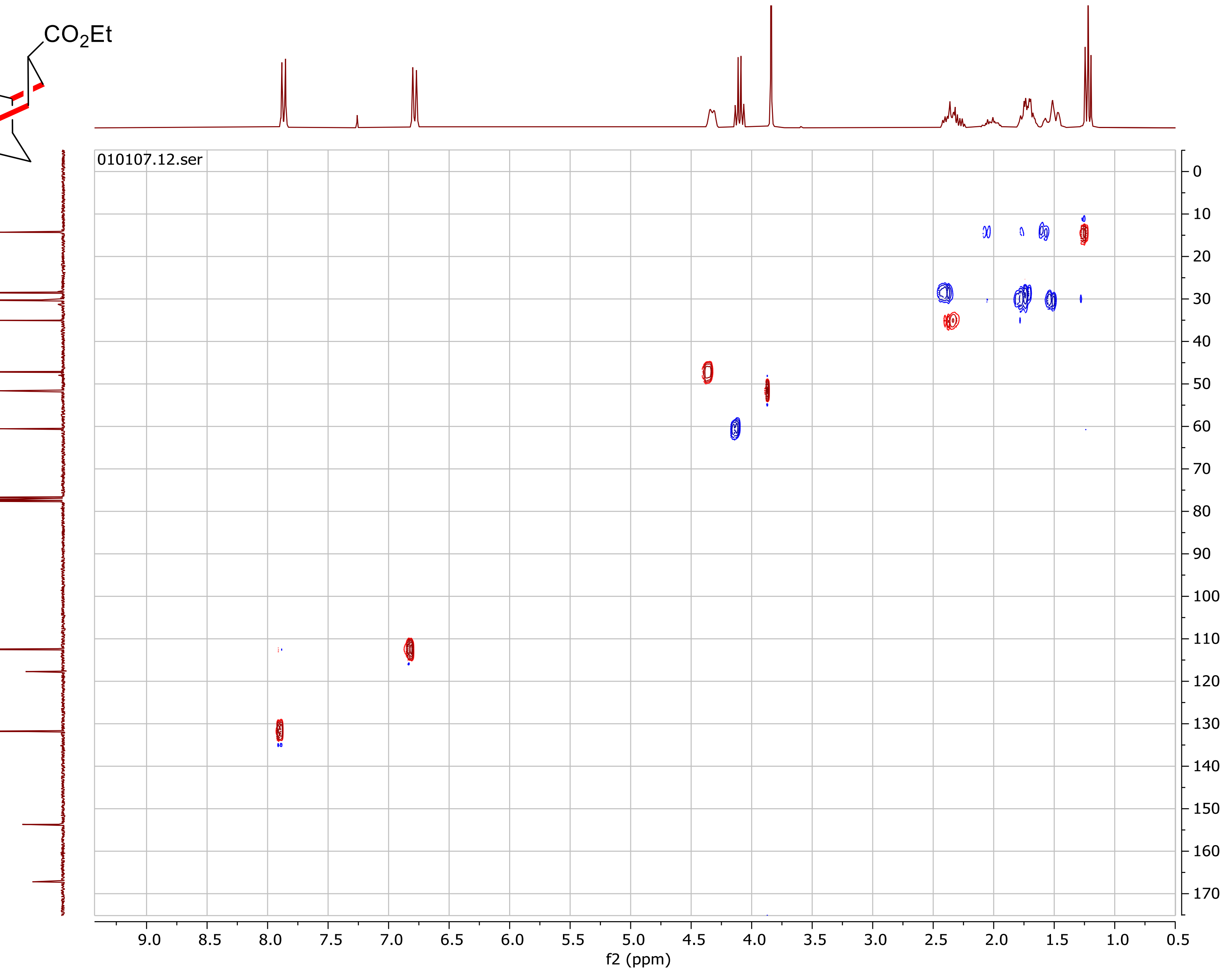

Ethyl 9-(4-methoxycarbonylphenyl)-9-azabicyclo[3.3.1]nonane-3-carboxylate (**8gα**)

^{1}H, ^{13}C-HMBC NMR (400 MHz, $CDCl_3$)

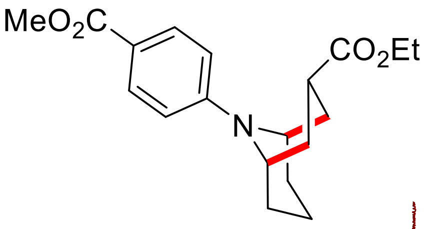

8g

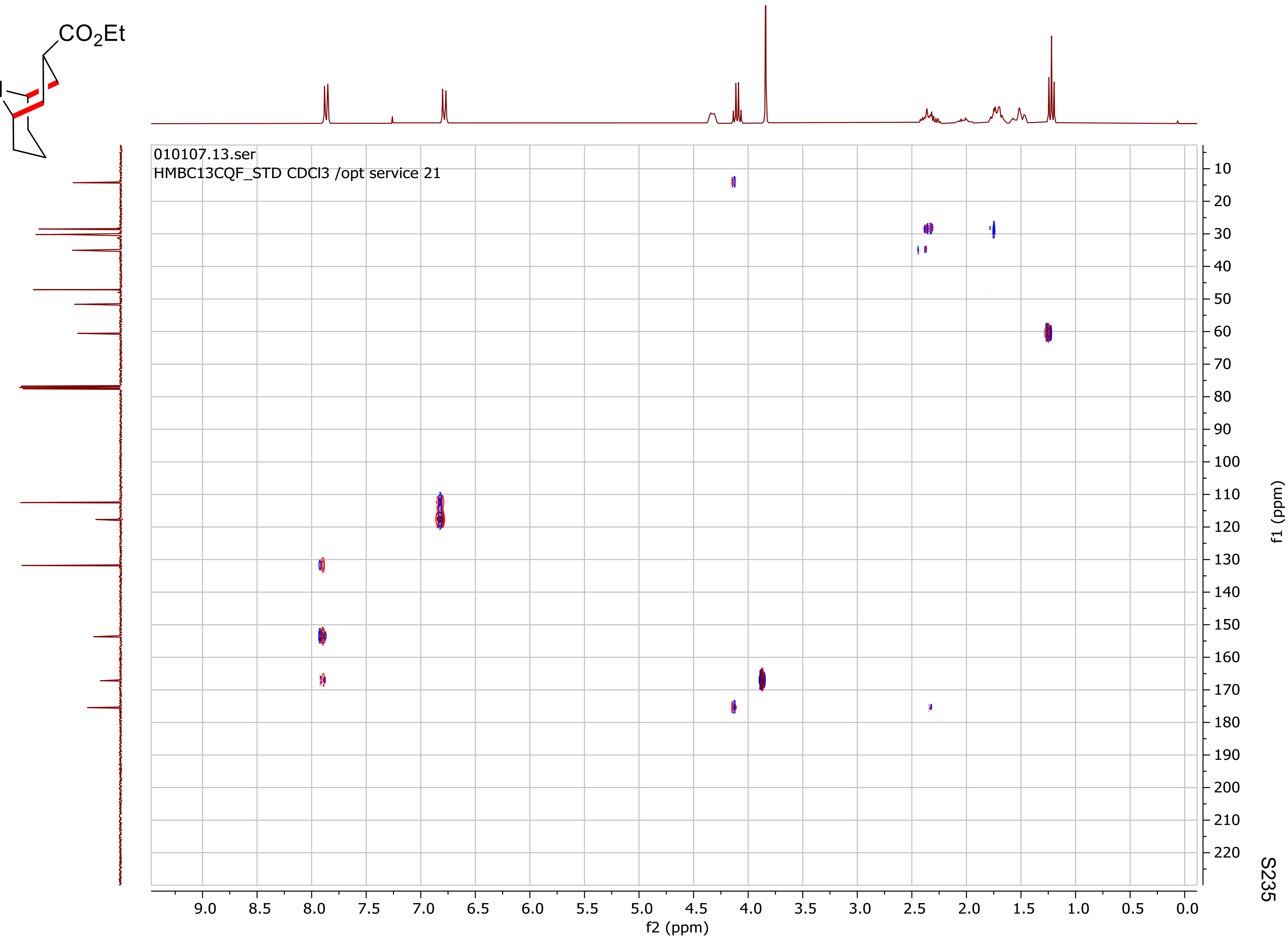

Ethyl 9-(4-methoxycarbonylphenyl)-9-azabicyclo[3.3.1]nonane-3-carboxylate (**8gα**)

FT-IR, ATR-diamond

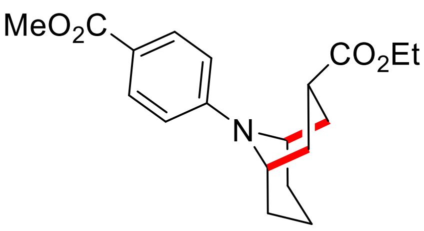

8g

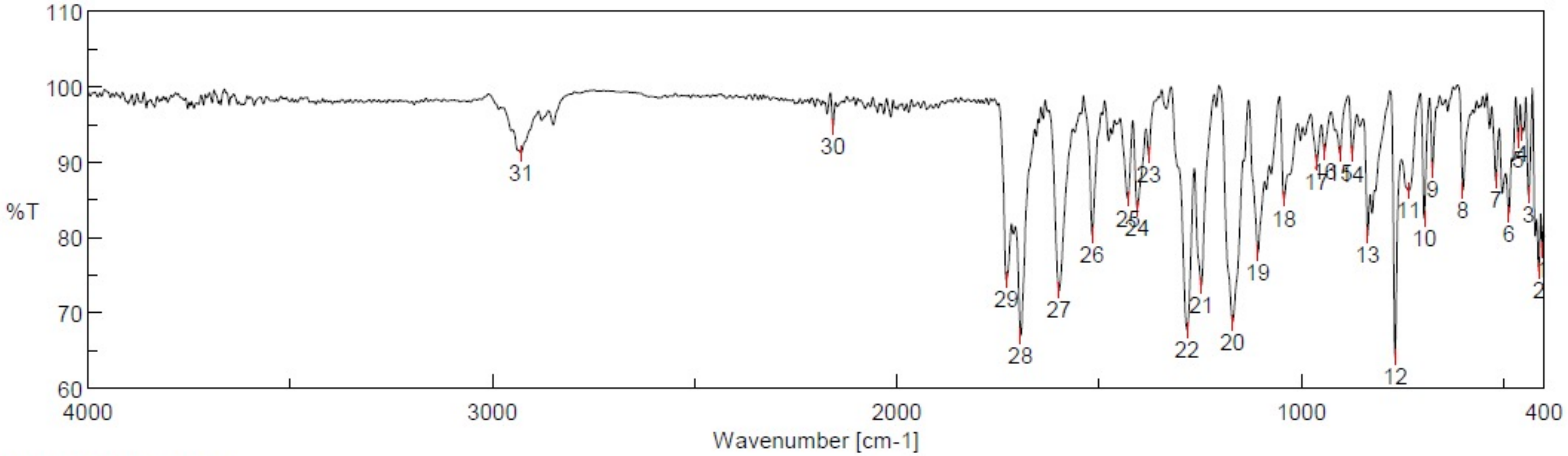

[Result of Peak Picking]

No.	Position	Intensity	No.	Position	Intensity	No.	Position	Intensity
1	404.014	78	2	412.692	76	3	438.726	86
4	454.154	94	5	463.796	93	6	486.938	83
7	518.758	88	8	600.717	86	9	675.928	89
10	696.177	83	11	734.746	86	12	768.494	64
13	835.99	80	14	874.56	91	15	904.451	91
16	943.02	91	17	962.305	90	18	1043.3	85
19	1107.9	78	20	1170.58	69	21	1247.72	74
22	1282.43	68	23	1376.93	91	24	1405.85	84
25	1428.99	85	26	1517.7	80	27	1599.66	73
28	1694.16	67	29	1729.83	74	30	2157.95	95
31	2929.34	91						

Ethyl 9-(4-methoxycarbonylphenyl)-9-azabicyclo[3.3.1]nonane-3-carboxylate (**8gα**) XRay

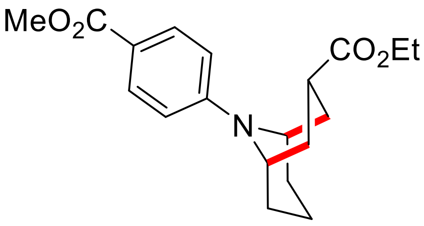

8g

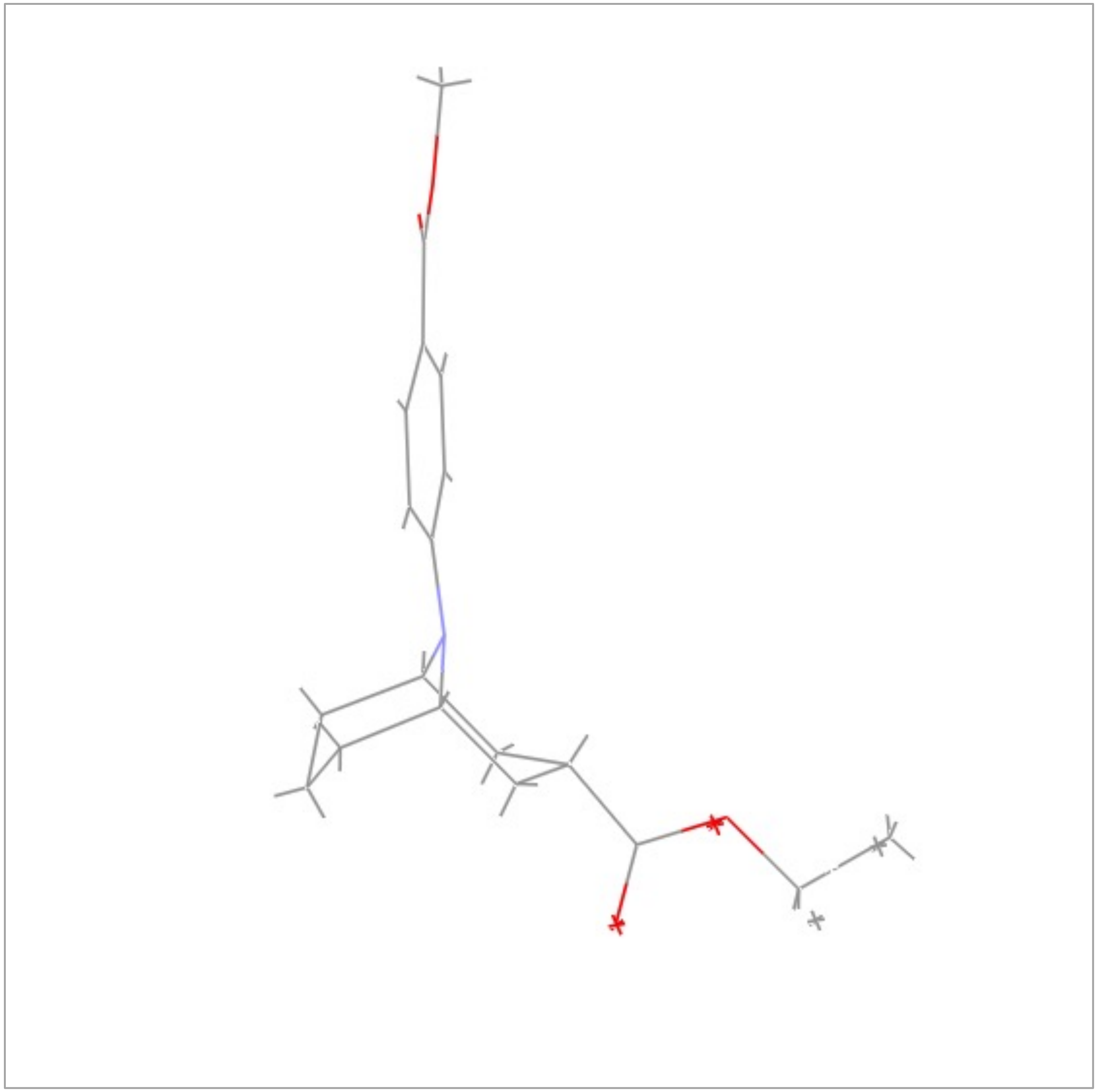

Ethyl 9-(4-acetylphenyl)-9-azabicyclo[3.3.1]nonane-3-carboxylate (**8h**) **α/β >20:1**

^{1}H-NMR (400 MHz, $CDCl_3$)

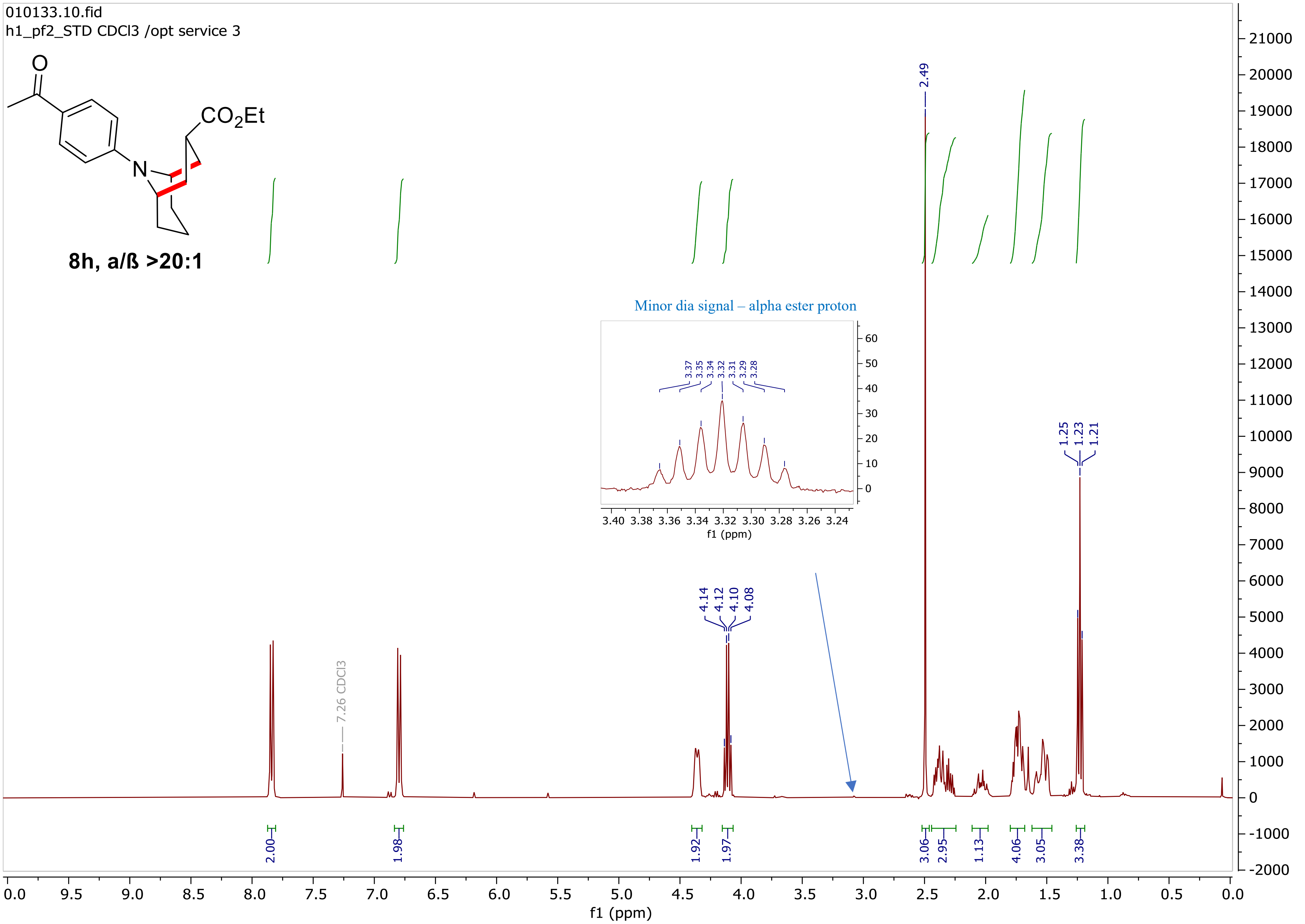

Ethyl 9-(4-acetylphenyl)-9-azabicyclo[3.3.1]nonane-3-carboxylate (**8h**) **α/β >20:1**

^{1}H-NMR (400 MHz, $CDCl_3$)

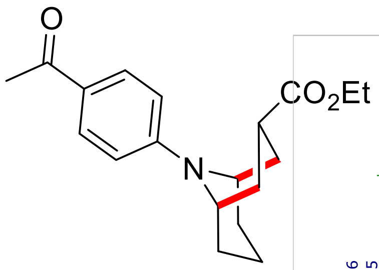

8h, a/ß >20:1

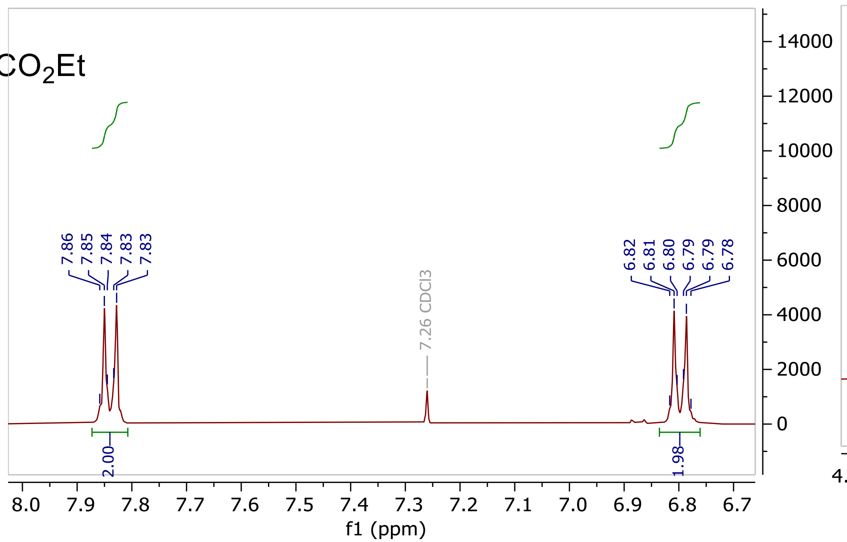

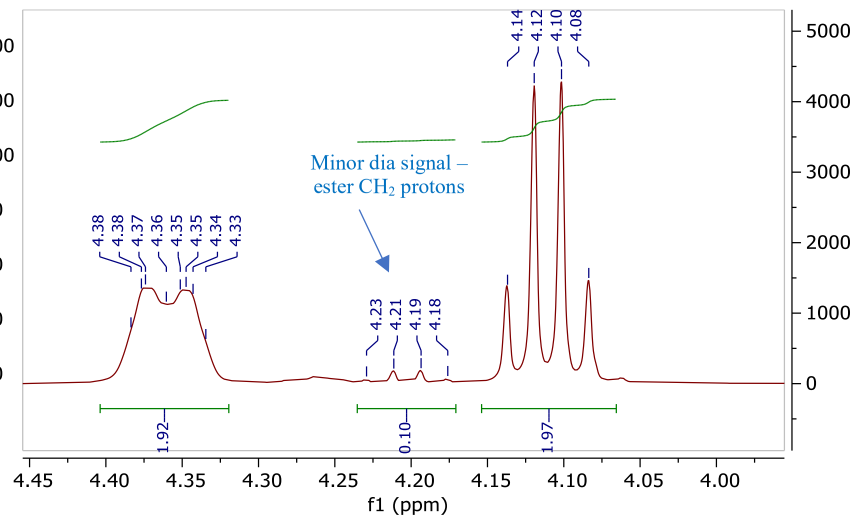

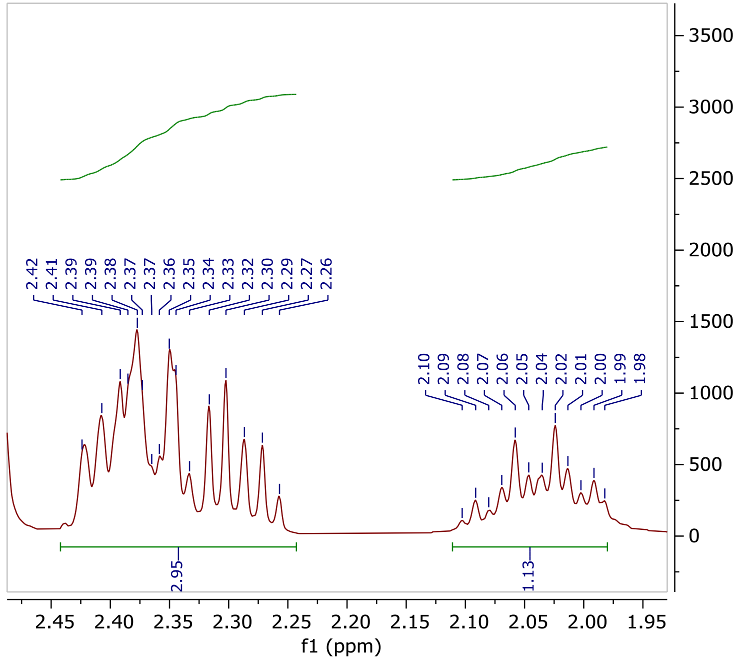

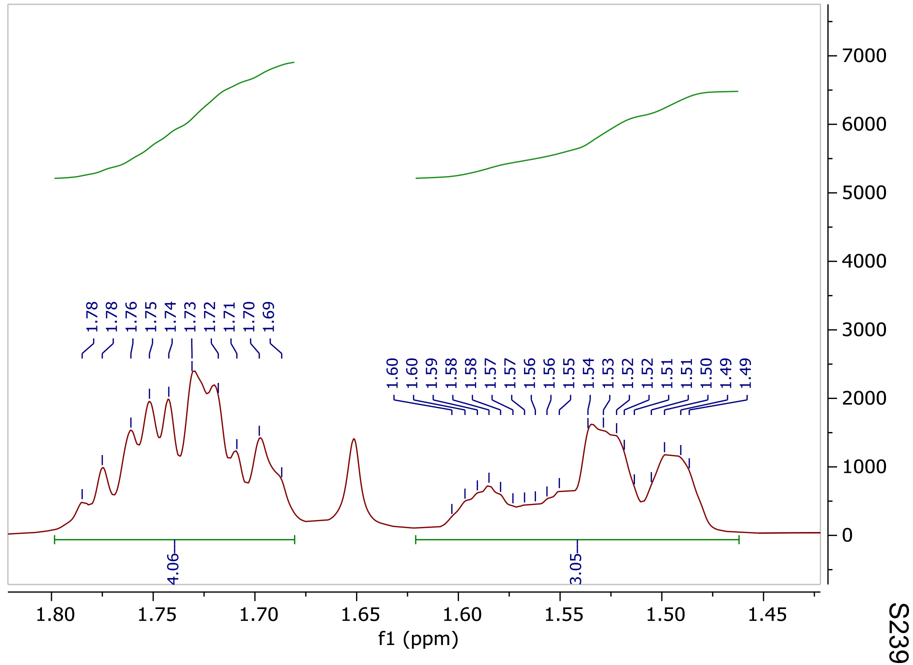

Ethyl 9-(4-acetylphenyl)-9-azabicyclo[3.3.1]nonane-3-carboxylate (**8hα**)

^{13}C-NMR (75 MHz, $CDCl_3$)

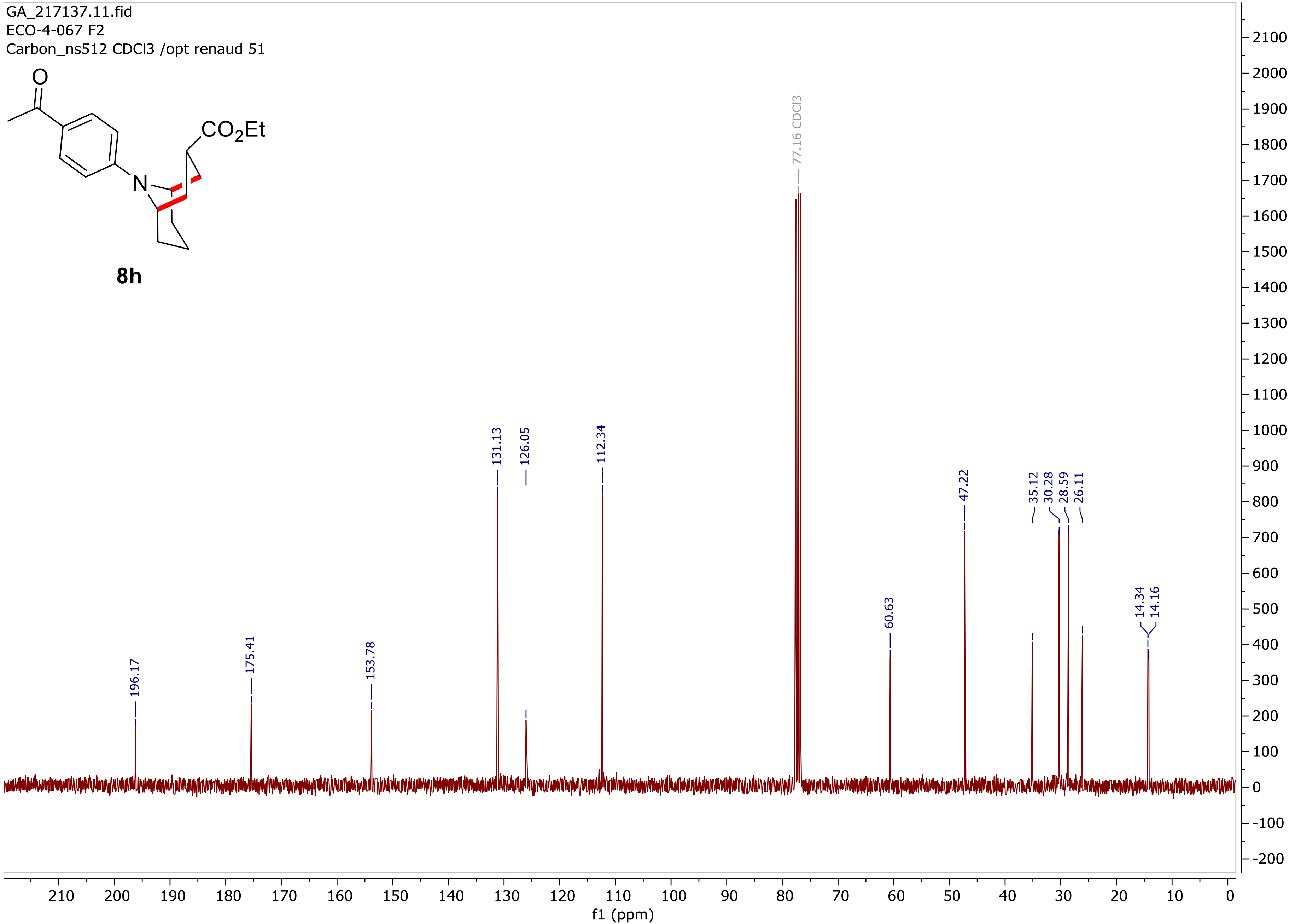

Ethyl 9-(4-acetylphenyl)-9-azabicyclo[3.3.1]nonane-3-carboxylate (**8hα**)

^{13}C-NMR (75 MHz, $CDCl_3$)

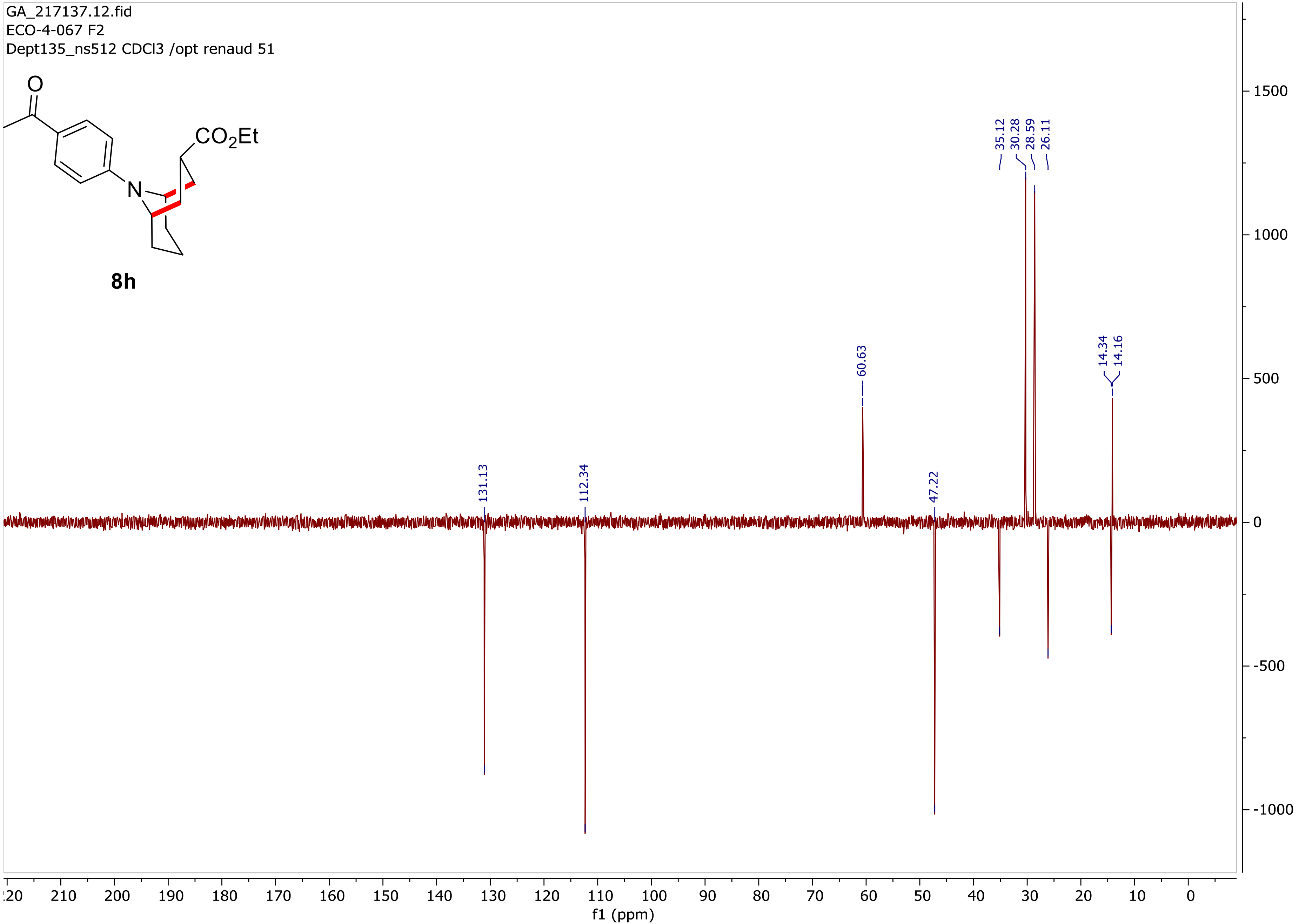

Ethyl 9-(4-acetylphenyl)-9-azabicyclo[3.3.1]nonane-3-carboxylate (**8hα**)

^{1}H, ^{1}H-COSY NMR (400 MHz, $CDCl_3$)

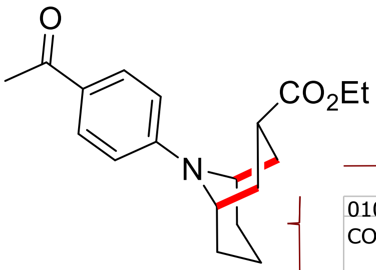

8h

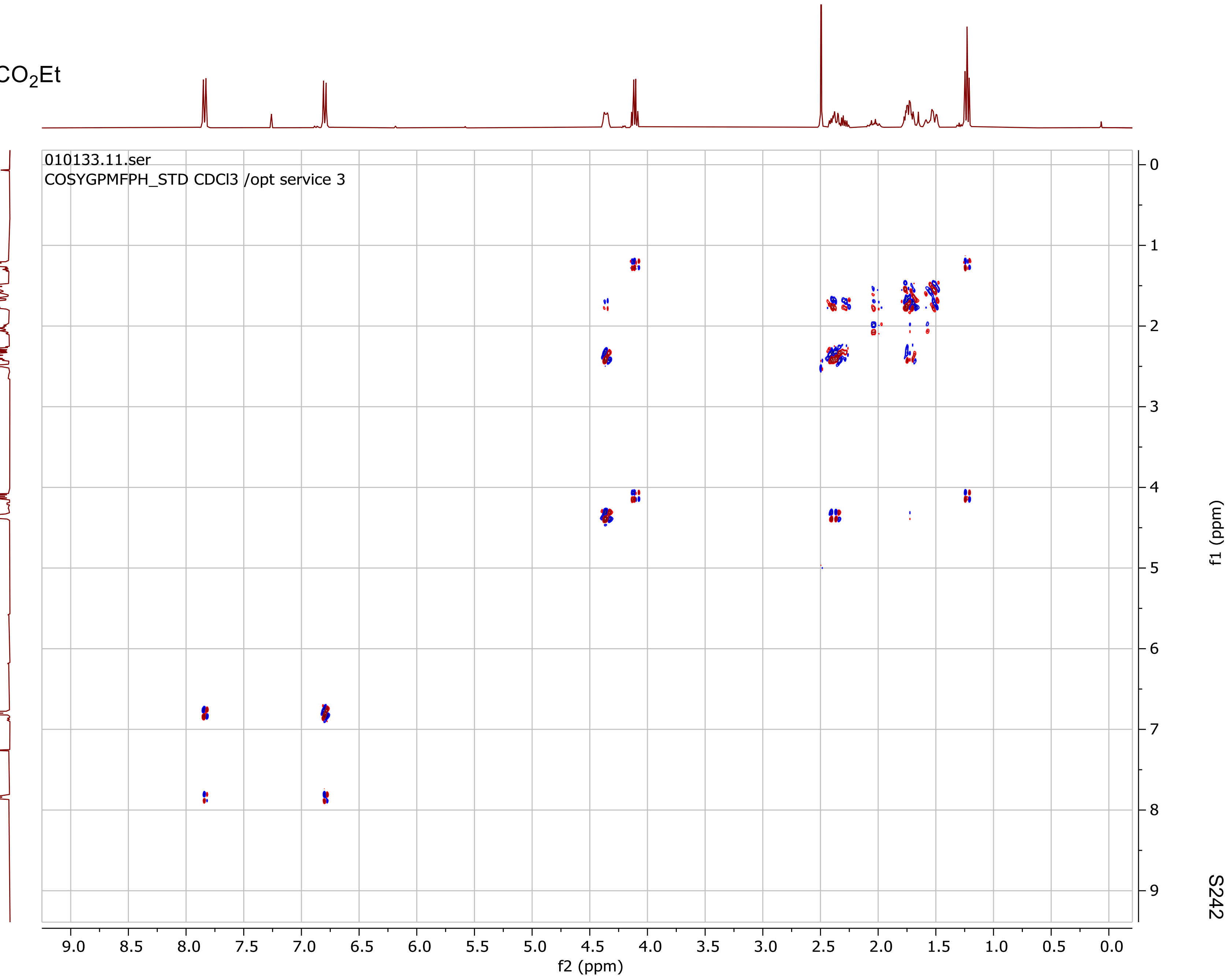

Ethyl 9-(4-acetylphenyl)-9-azabicyclo[3.3.1]nonane-3-carboxylate (**8hα**)

^{1}H, ^{13}C-HSQC NMR (400 MHz, $CDCl_3$)

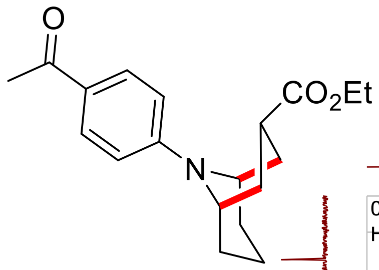

8h

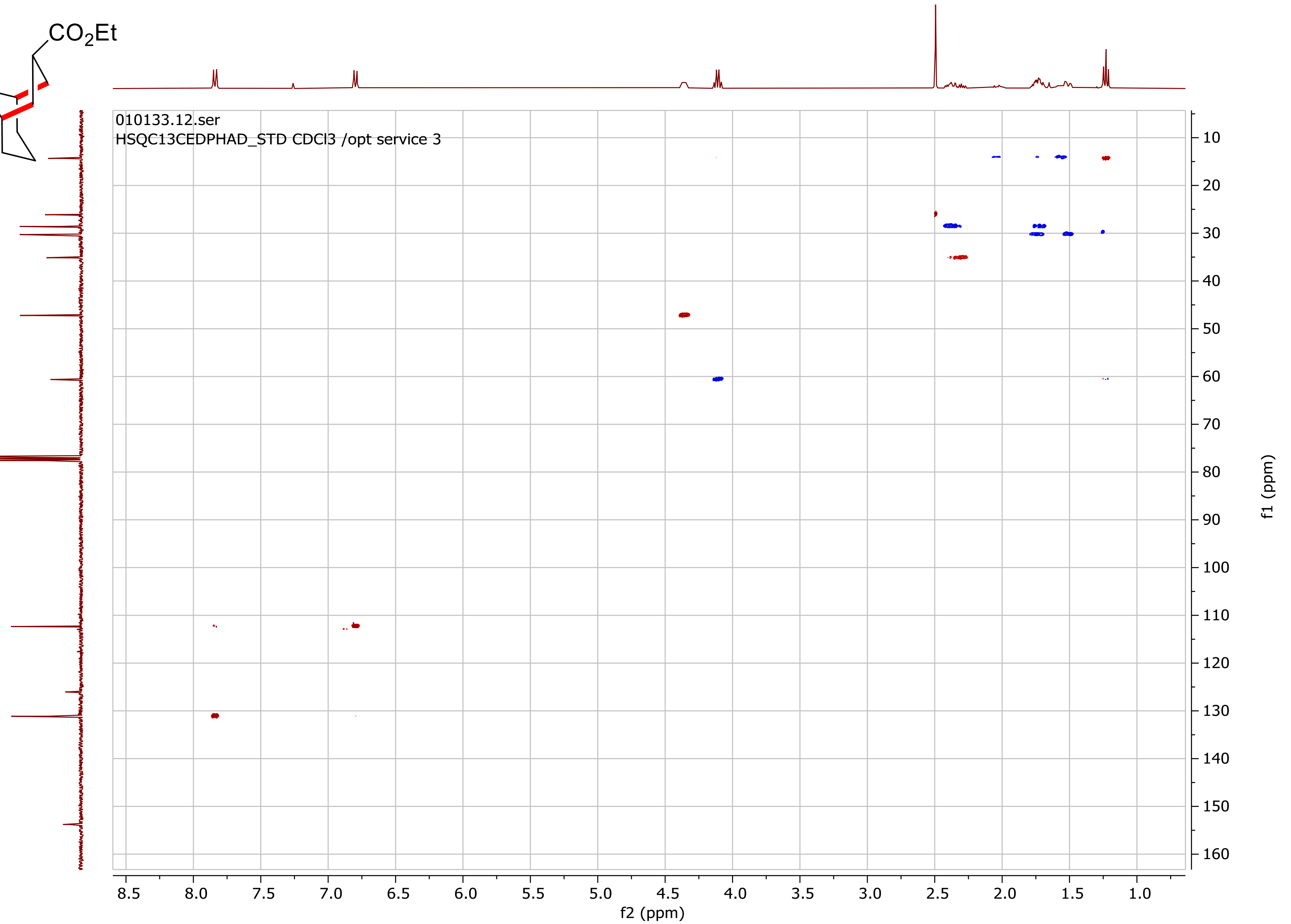

Ethyl 9-(4-acetylphenyl)-9-azabicyclo[3.3.1]nonane-3-carboxylate (**8hα**)

^{1}H, ^{13}C-HSQC NMR (400 MHz, $CDCl_3$)

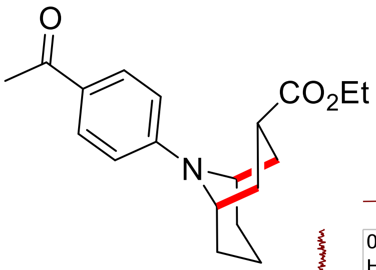

8h

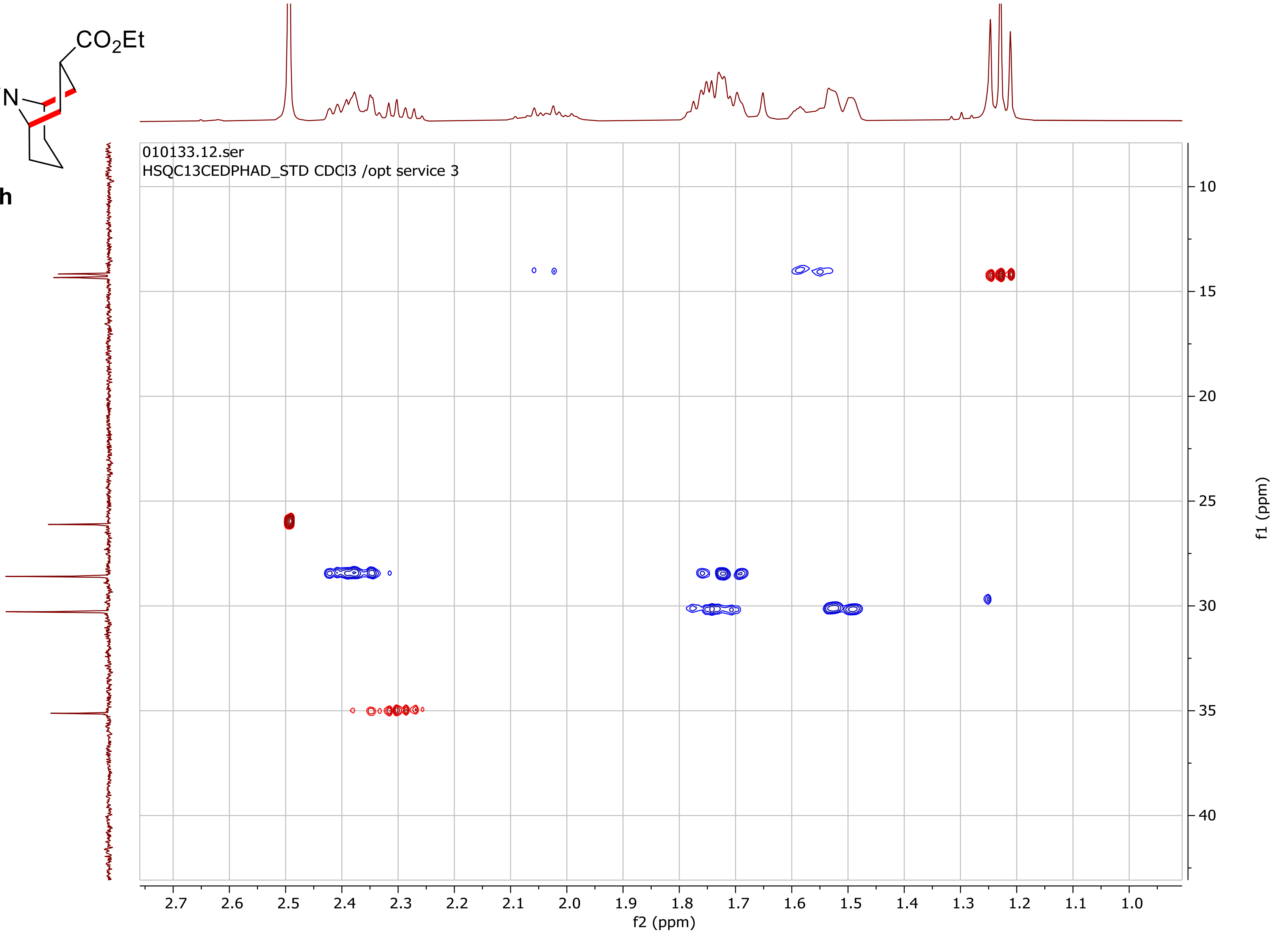

Ethyl 9-(4-acetylphenyl)-9-azabicyclo[3.3.1]nonane-3-carboxylate (**8hα**)

^1H, ^{13}C-HMBC NMR (400 MHz, $CDCl_3$)

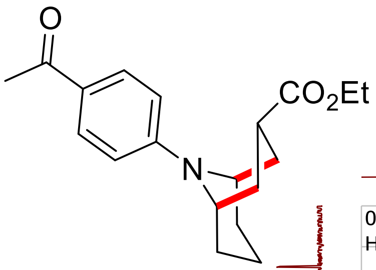

8h

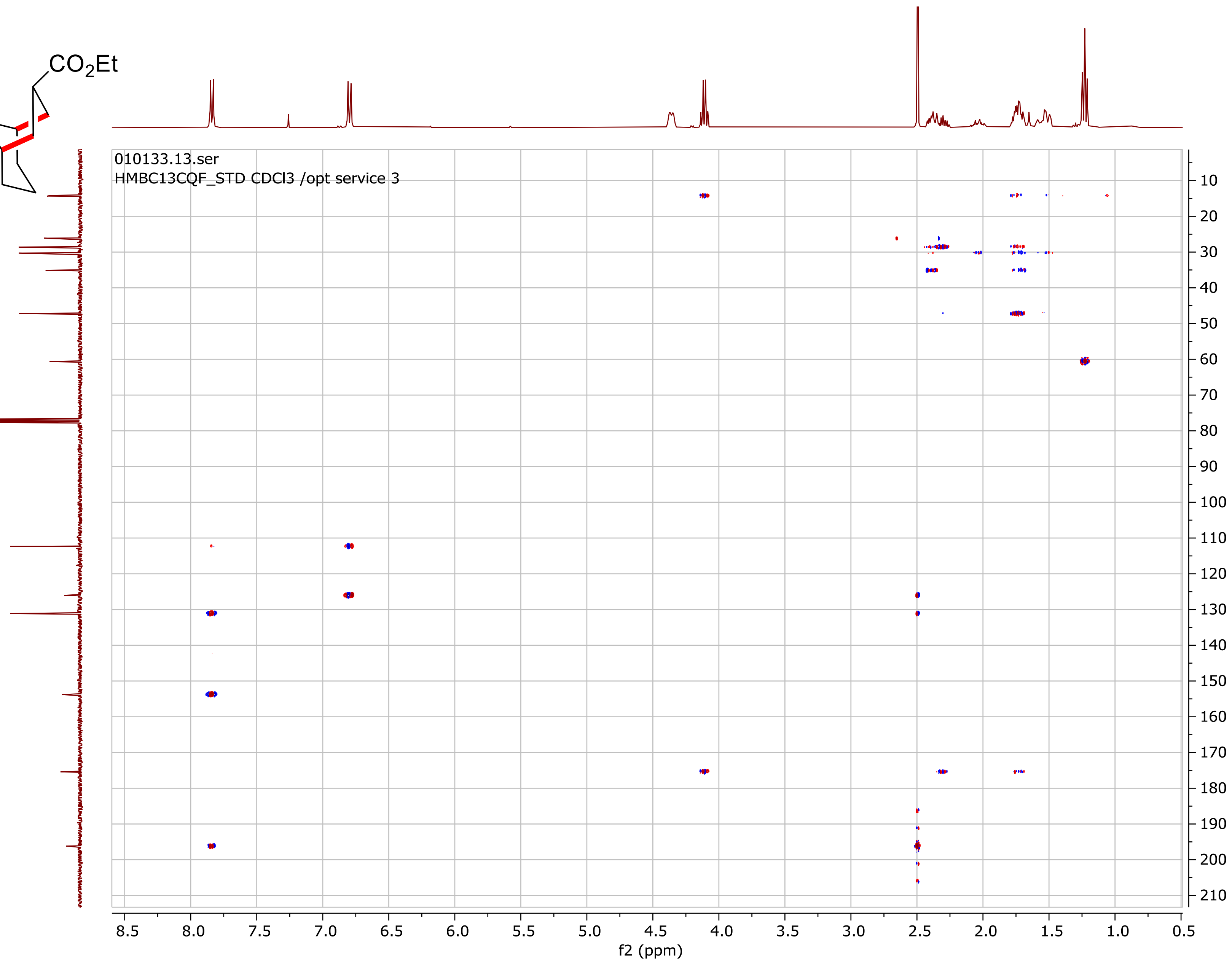

Ethyl 9-(4-acetylphenyl)-9-azabicyclo[3.3.1]nonane-3-carboxylate (**8hα**)

FT-IR, ATR-diamond

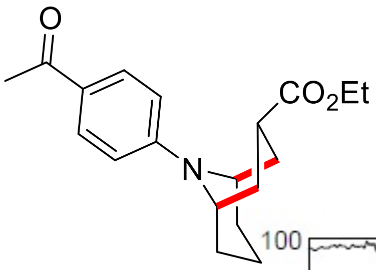

8h

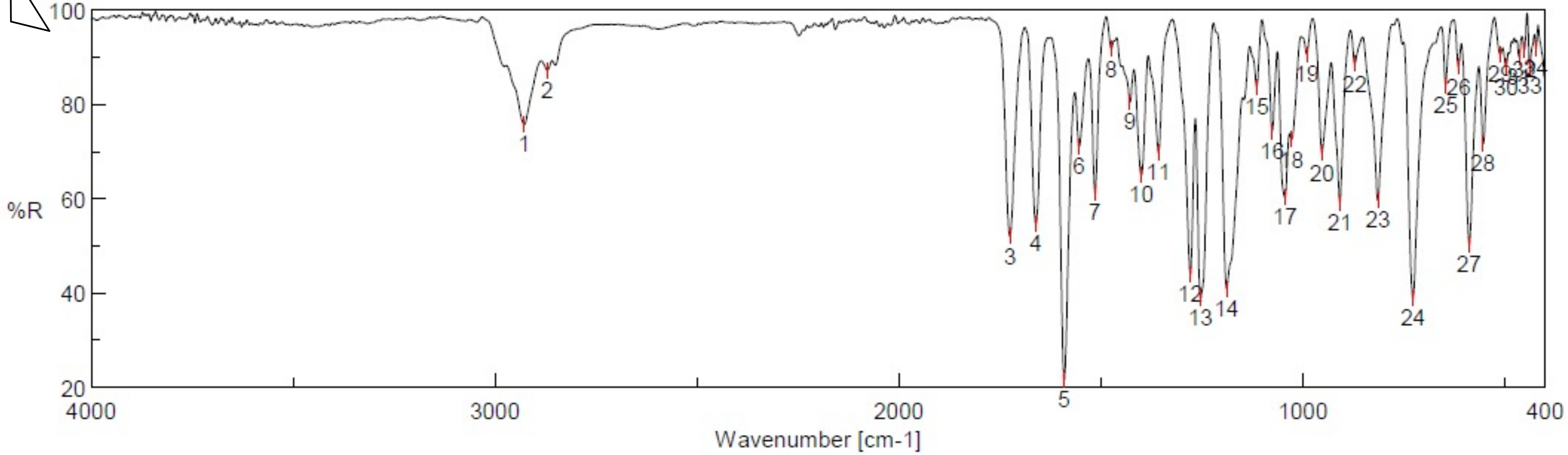

[Result of Peak Picking]

No.	Position	Intensity	No.	Position	Intensity	No.	Position	Intensity
1	2927.41	75.7148	2	2870.52	87.248	3	1725.01	51.9861
4	1661.37	54.5469	5	1590.99	21.5134	6	1554.34	71.0763
7	1514.81	61.3127	8	1474.31	91.7457	9	1428.03	80.4884
10	1400.07	65.0293	11	1357.64	69.8463	12	1278.57	43.9076
13	1253.5	38.8401	14	1188.9	40.8729	15	1113.69	83.7889
16	1076.08	74.3426	17	1044.26	60.2702	18	1027.87	72.5325
19	990.268	90.6413	20	952.663	69.7011	21	908.308	59.1514
22	871.667	88.64	23	813.813	59.6003	24	727.032	38.972
25	646.036	84.0832	26	614.217	87.9553	27	588.182	50.1469
28	553.47	71.6271	29	511.044	90.5387	30	496.58	87.9719
31	464.761	90.0831	32	451.261	91.5065	33	437.762	88.0154
34	422.334	91.8451						

Ethyl 9-[4-(trifluoromethyl)phenyl]-9-azabicyclo[3.3.1]nonane-3-carboxylate (**8i**) **α/β 14:1**

^{1}H-NMR (400 MHz, $CDCl_3$)

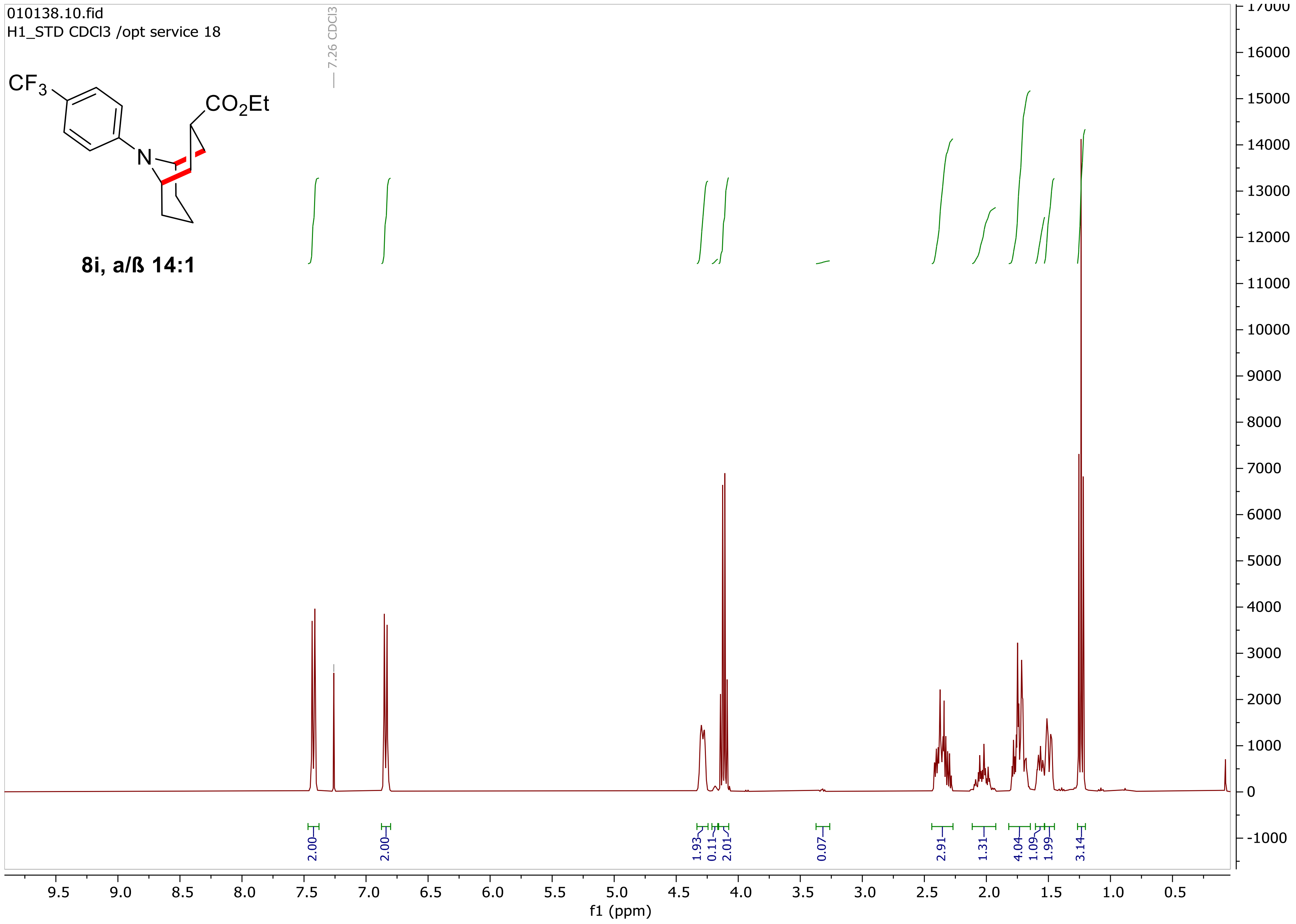

Ethyl 9-[4-(trifluoromethyl)phenyl]-9-azabicyclo[3.3.1]nonane-3-carboxylate (**8i**) **α/β 14:1**

^{1}H-NMR (400 MHz, $CDCl_3$)

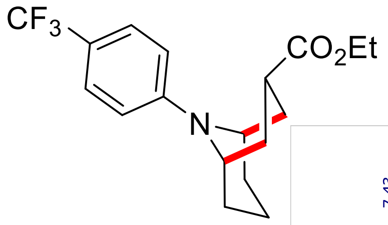

8i, a/ß 14:1

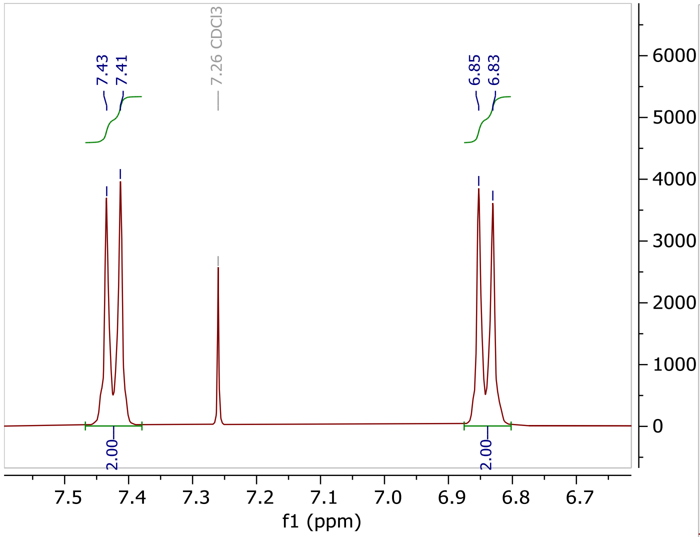

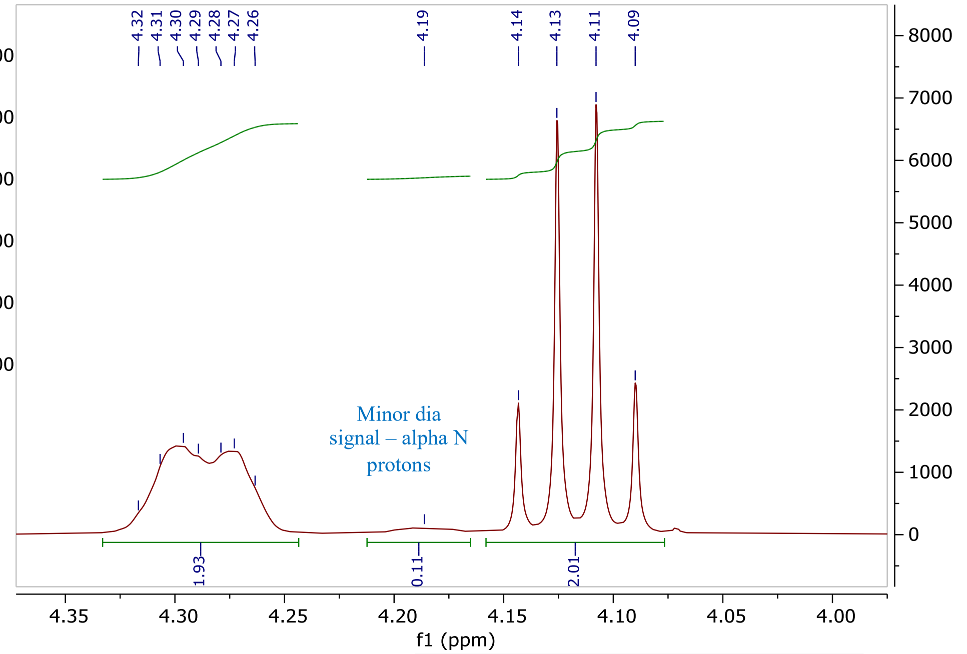

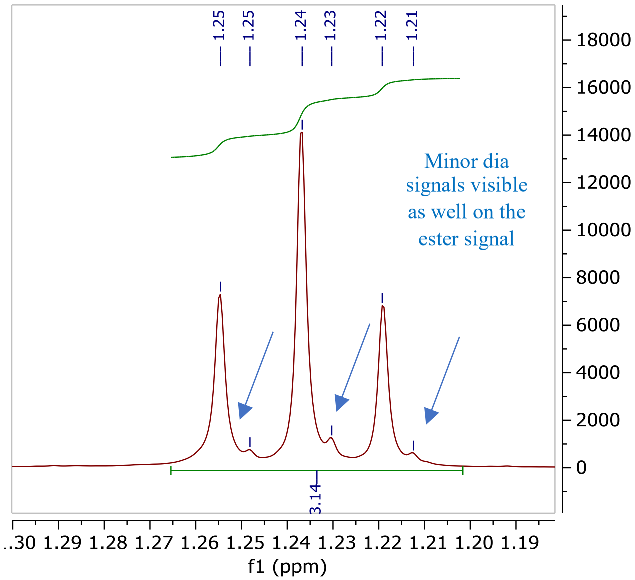

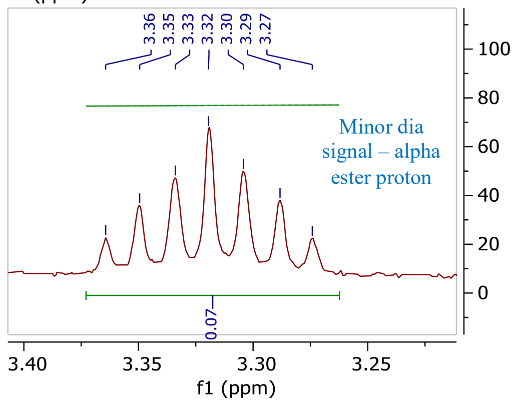

Ethyl 9-[4-(trifluoromethyl)phenyl]-9-azabicyclo[3.3.1]nonane-3-carboxylate (**8i**) **α/β 14:1**

^{1}H-NMR (400 MHz, $CDCl_3$)

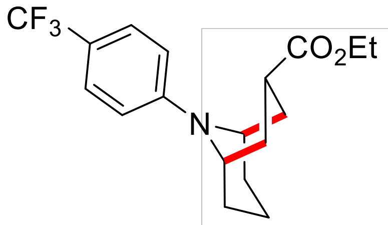

8i, a/ß 14:1

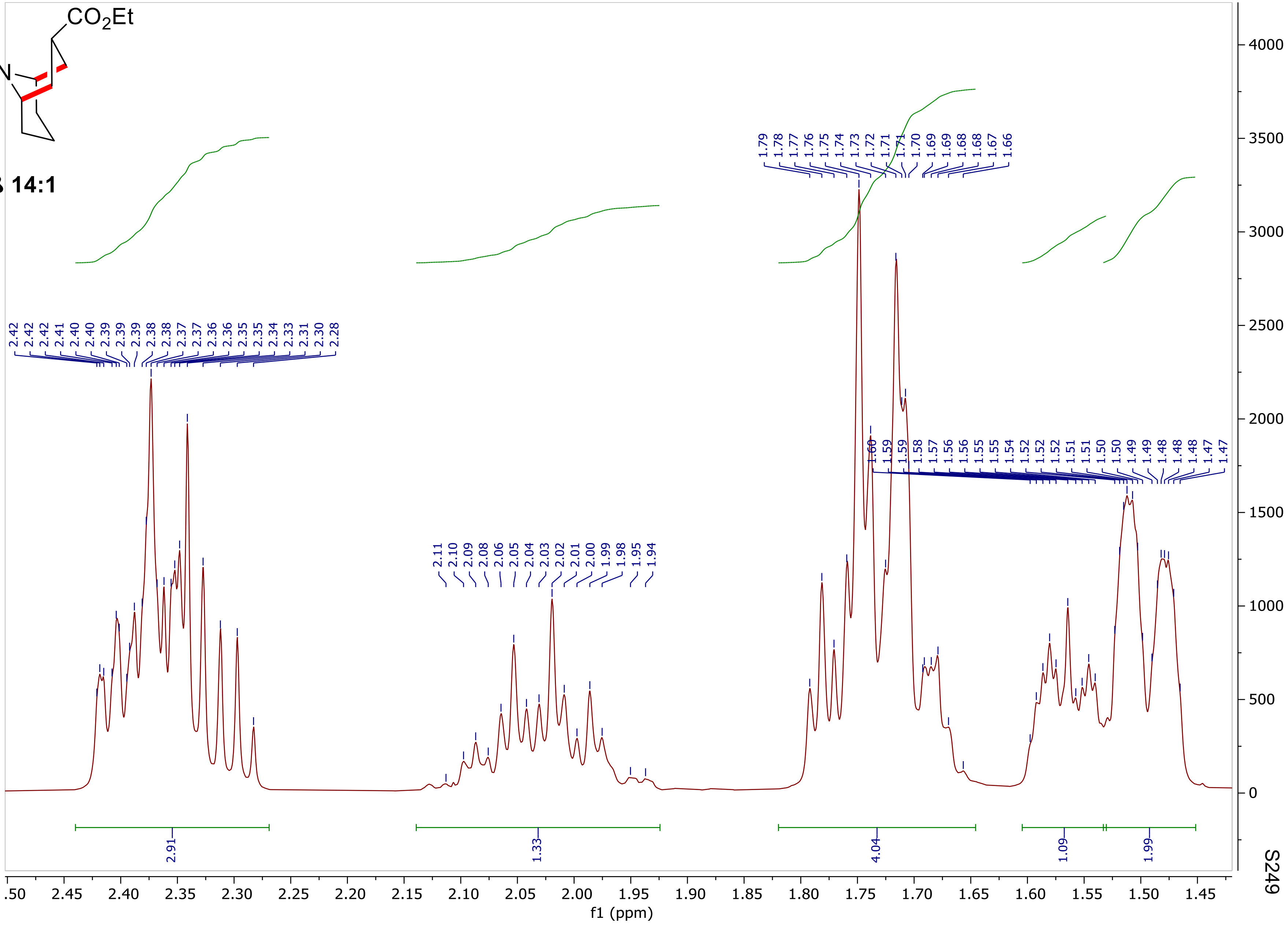

Ethyl 9-[4-(trifluoromethyl)phenyl]-9-azabicyclo[3.3.1]nonane-3-carboxylate (**8i**) **α/β 14:1**

^{13}C-NMR (101 MHz, $CDCl_3$)

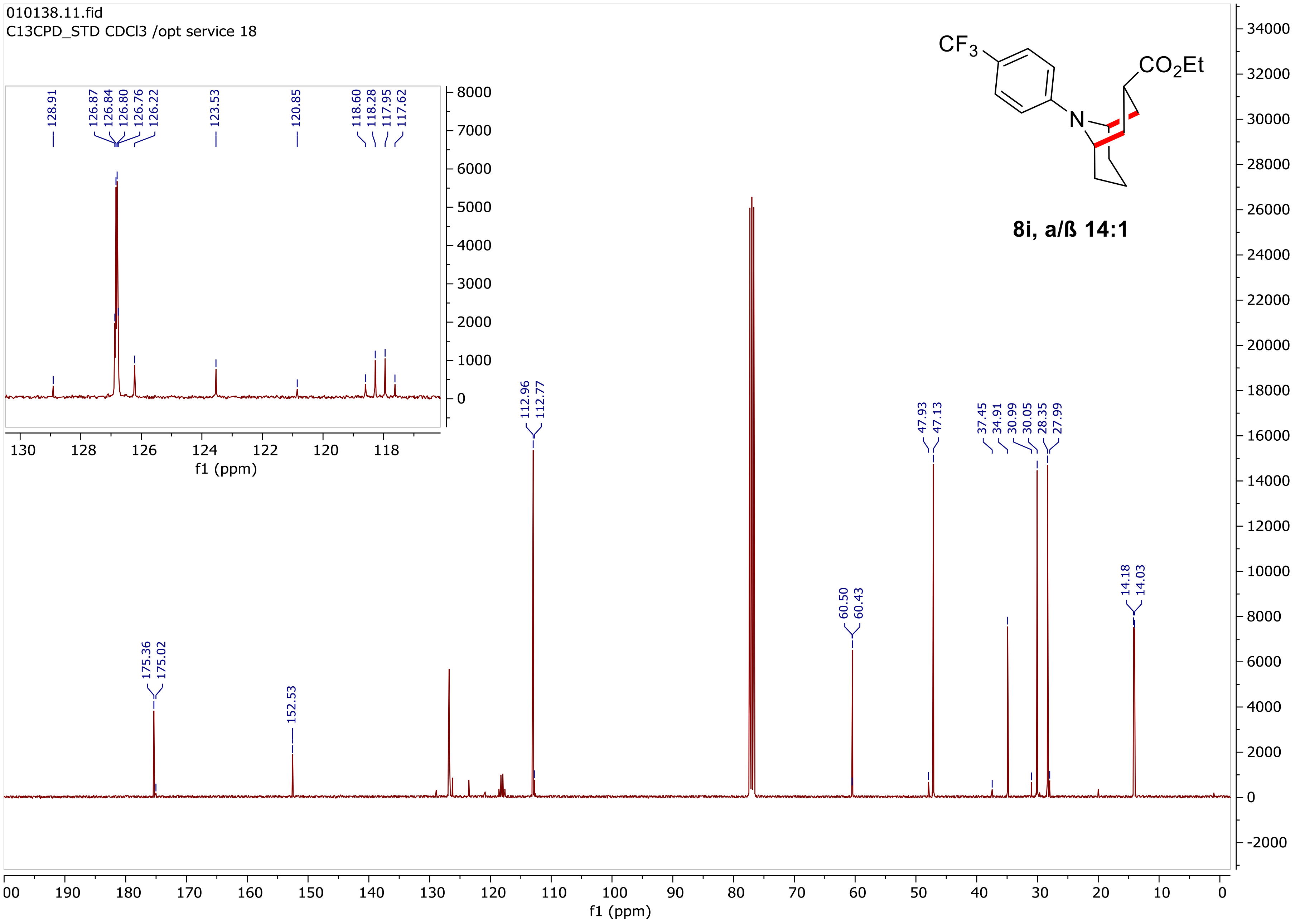

Ethyl 9-[4-(trifluoromethyl)phenyl]-9-azabicyclo[3.3.1]nonane-3-carboxylate (**8i**) **α/β 14:1**

^{13}C-NMR (101 MHz, $CDCl_3$)

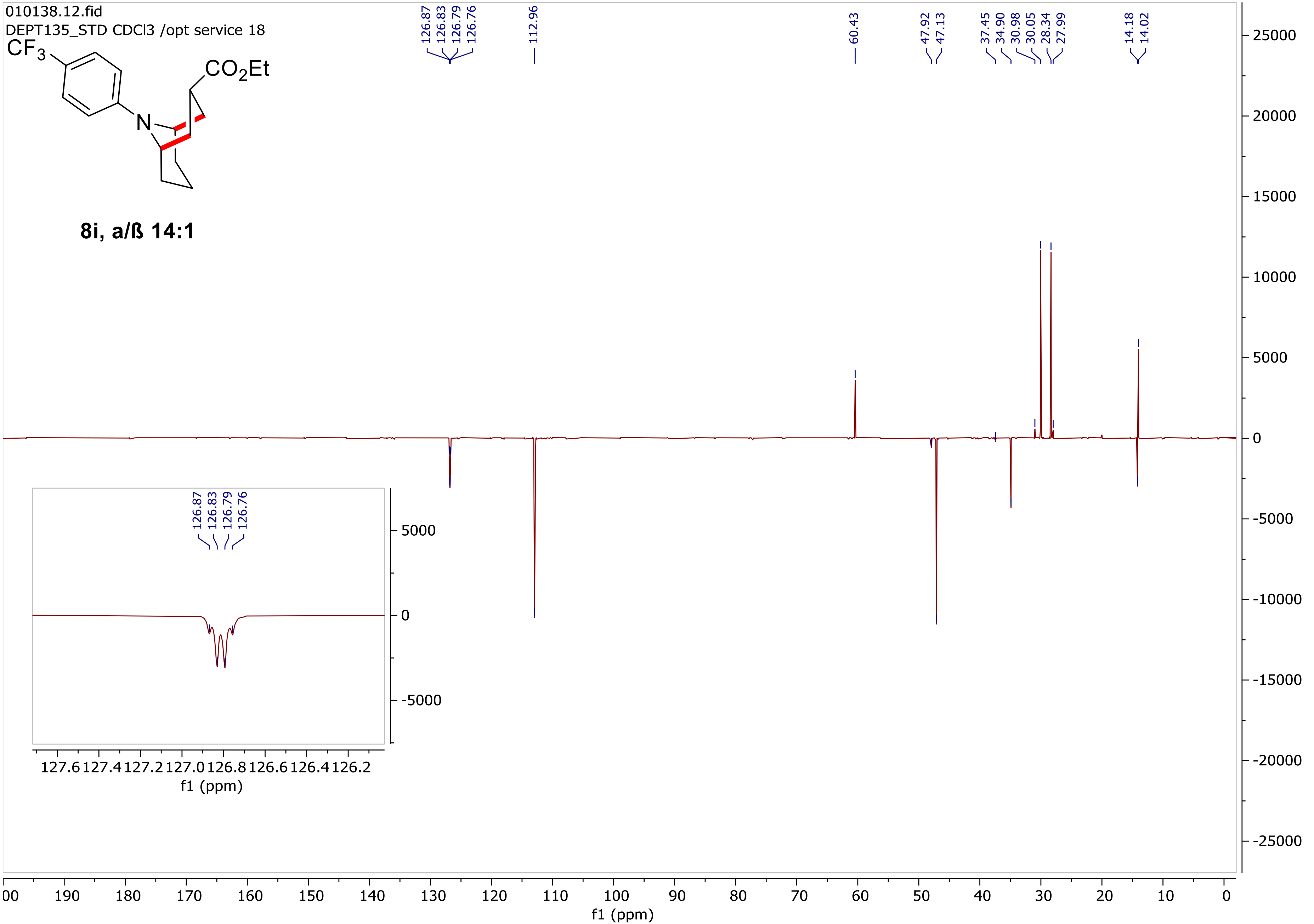

Ethyl 9-[4-(trifluoromethyl)phenyl]-9-azabicyclo[3.3.1]nonane-3-carboxylate (**8i**) **α/β 14:1**

^{1}H, ^{1}H-COSY NMR (400 MHz, $CDCl_3$)

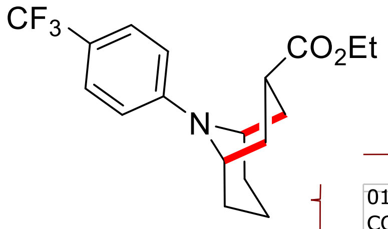

8i, a/ß 14:1

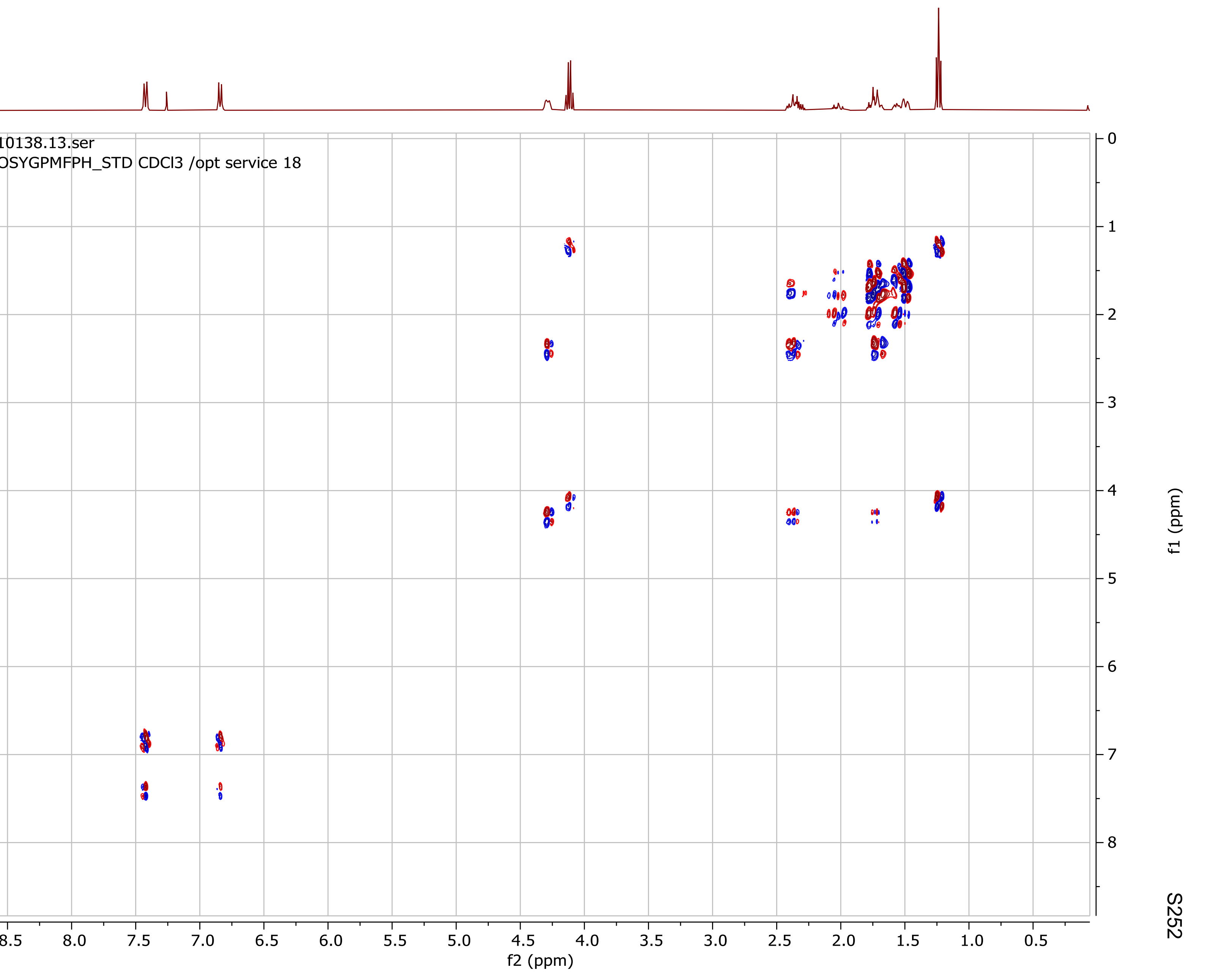

Ethyl 9-[4-(trifluoromethyl)phenyl]-9-azabicyclo[3.3.1]nonane-3-carboxylate (**8i**) **α/β 14:1**

^{1}H, ^{13}C-HSQC NMR (400 MHz, $CDCl_3$)

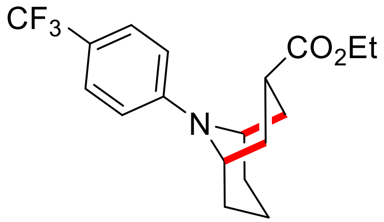

8i, a/ß 14:1

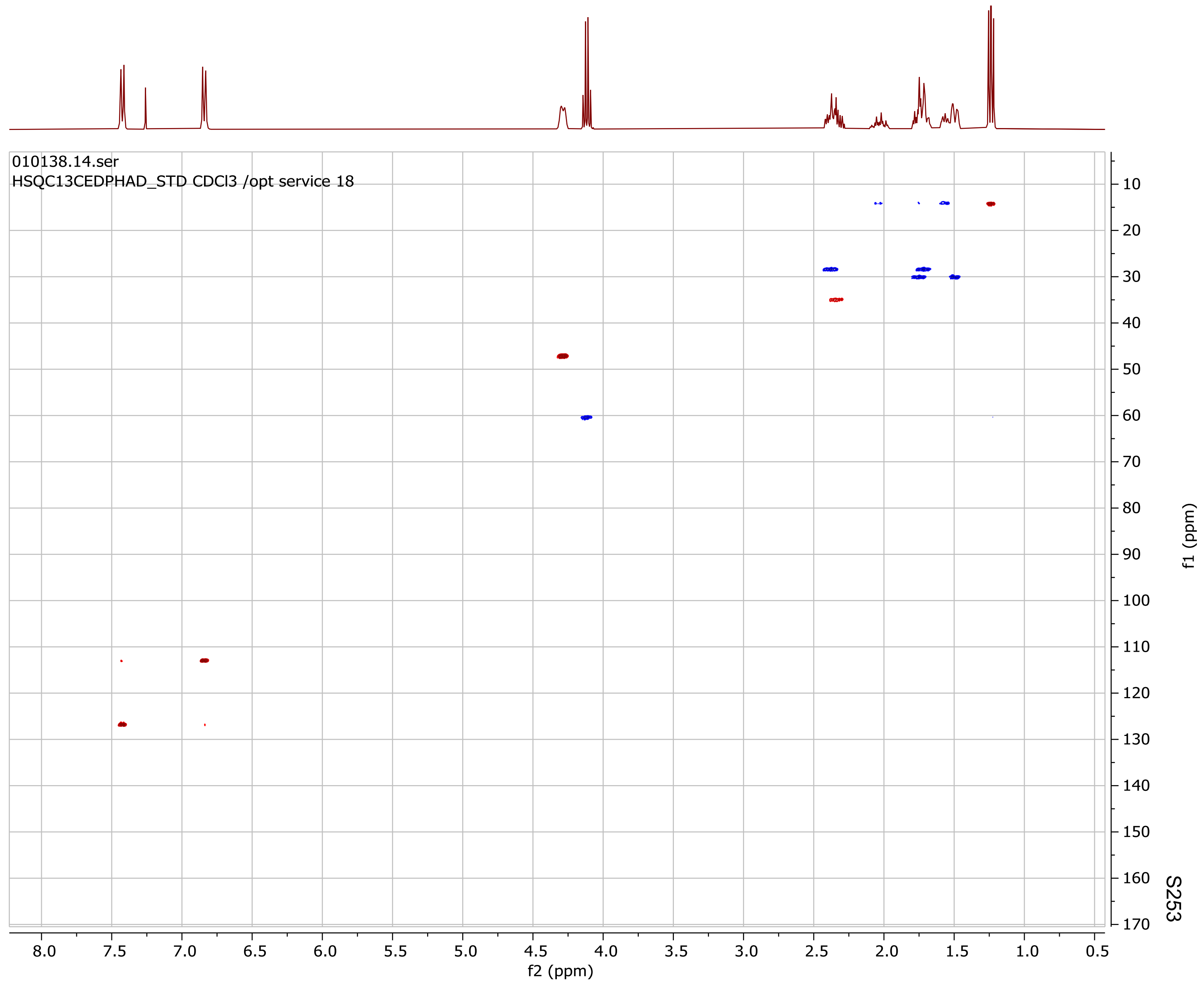

Ethyl 9-[4-(trifluoromethyl)phenyl]-9-azabicyclo[3.3.1]nonane-3-carboxylate (**8i**) **α/β 14:1**

^{1}H, ^{13}C-HSQC NMR (400 MHz, $CDCl_3$)

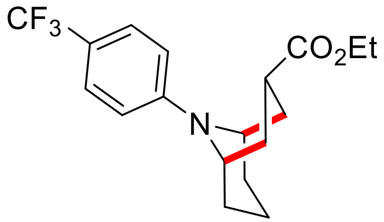

8i, a/ß 14:1

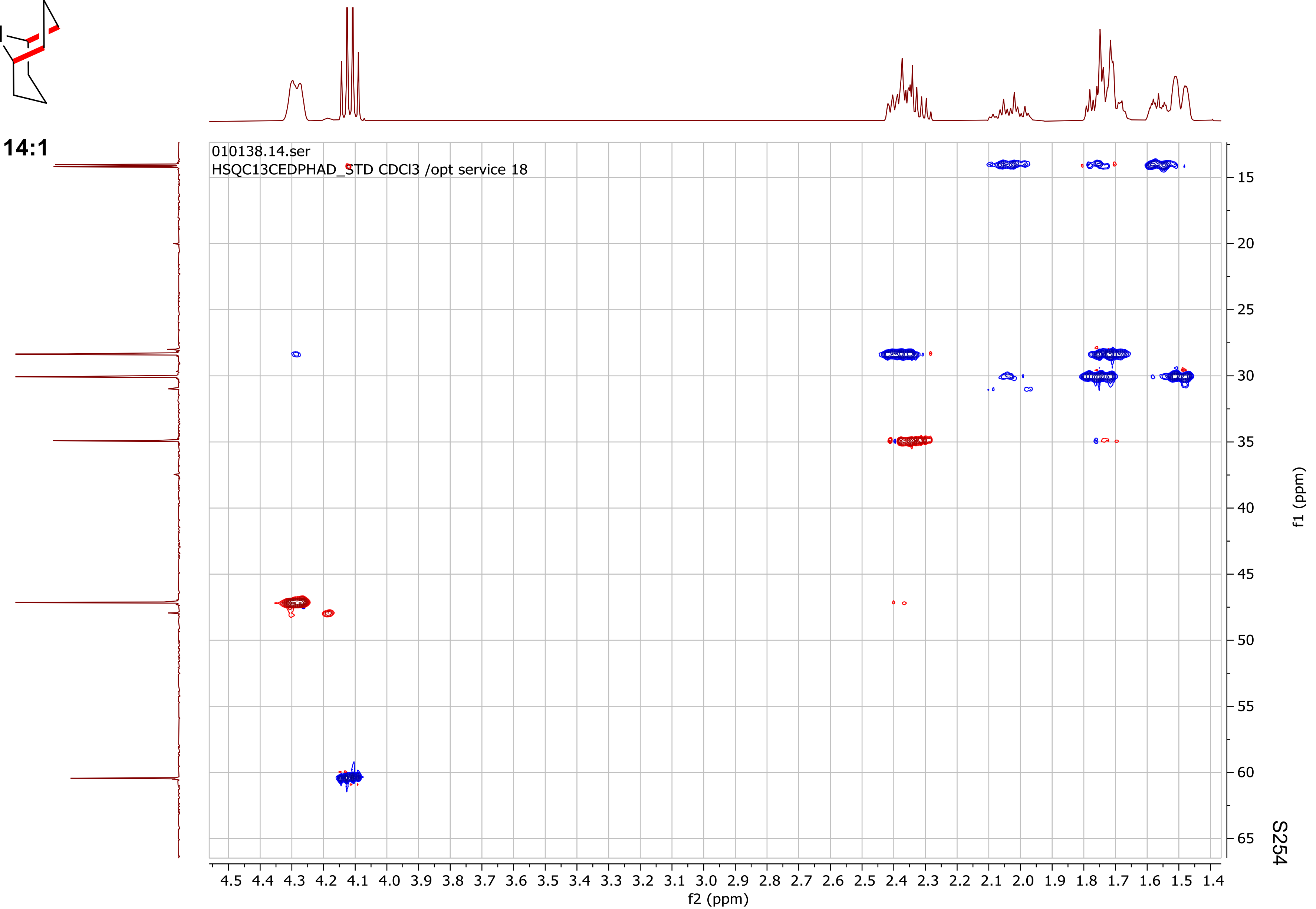

Ethyl 9-[4-(trifluoromethyl)phenyl]-9-azabicyclo[3.3.1]nonane-3-carboxylate (**8i**) **α/β 14:1**

^1H, ^{13}C-HMBC NMR (400 MHz, $CDCl_3$)

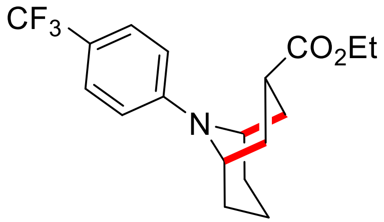

8i, a/ß 14:1

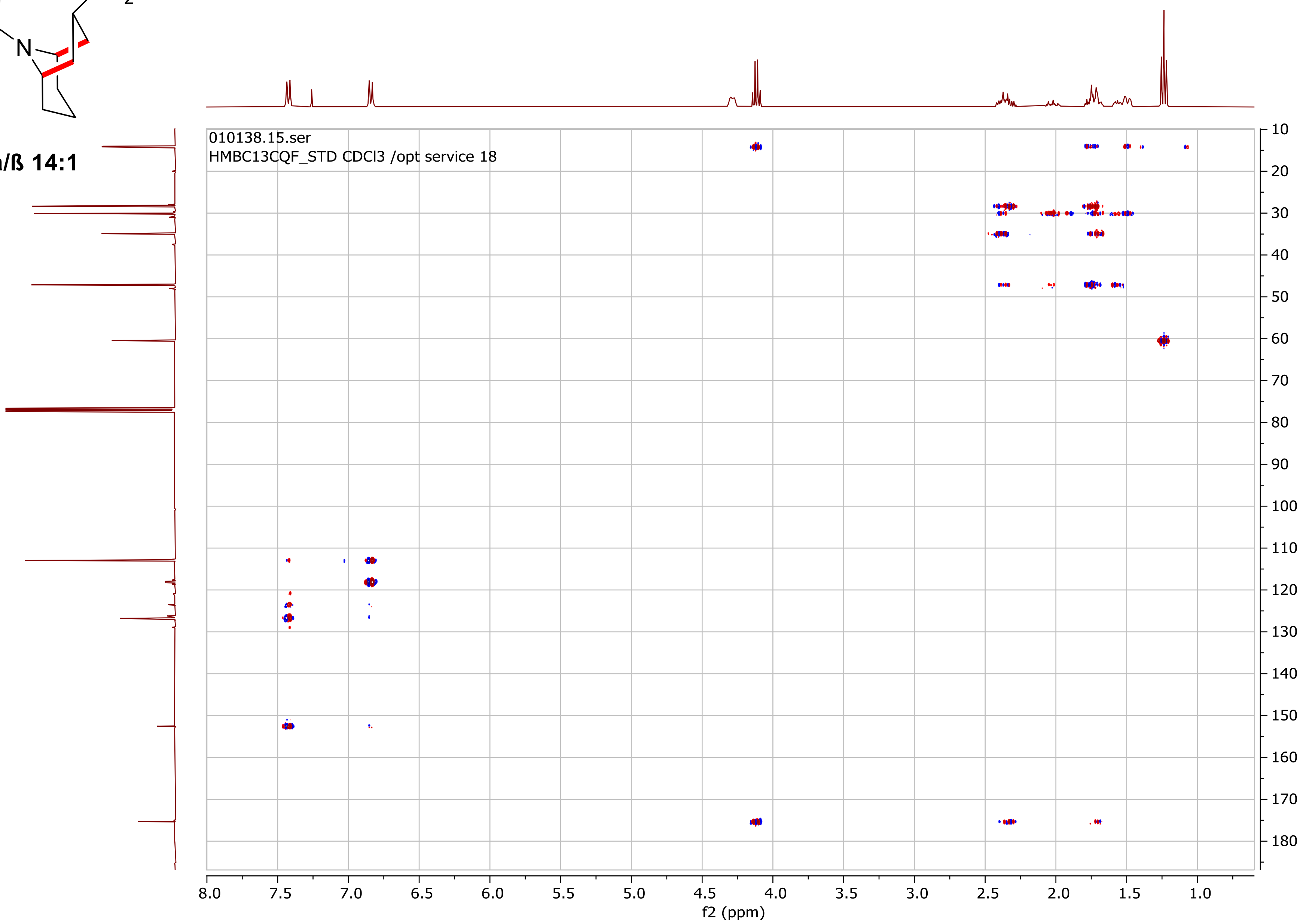

Ethyl 9-[4-(trifluoromethyl)phenyl]-9-azabicyclo[3.3.1]nonane-3-carboxylate (**8i**) **α/β 14:1**

FT-IR, ATR-diamond

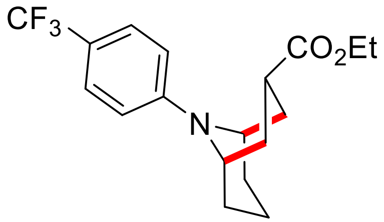

8i, a/ß 14:1

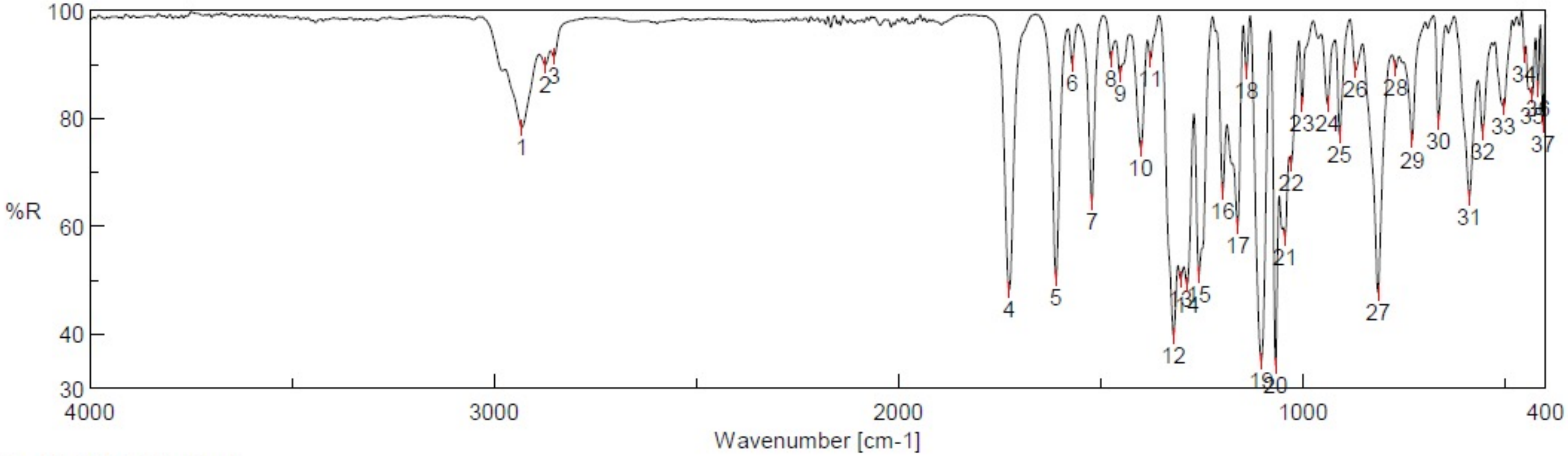

[Result of Peak Picking]

No.	Position	Intensity	No.	Position	Intensity	No.	Position	Intensity
1	2931.27	78.3519	2	2873.42	89.922	3	2852.2	91.458
4	1725.98	48.1808	5	1610.27	50.3762	6	1570.74	90.1332
7	1521.56	64.5891	8	1475.28	91.0578	9	1452.14	88.3035
10	1401.03	74.5497	11	1376.93	90.9722	12	1320.04	39.7232
13	1302.68	50.188	14	1286.29	49.1499	15	1257.36	51.0294
16	1198.54	66.3776	17	1160.94	60.0935	18	1139.72	88.831
19	1103.08	34.9199	20	1067.41	34.2417	21	1044.26	57.9829
22	1028.84	71.5877	23	1001.84	82.6092	24	939.163	82.6438
25	909.272	76.8691	26	869.739	88.9494	27	813.813	47.762
28	770.423	89.2398	29	729.925	75.9529	30	664.357	79.5068
31	588.182	65.4505	32	555.398	77.5655	33	503.33	82.1703
34	452.225	91.8666	35	432.941	84.2529	36	418.477	85.4968
37	405.942	78.8362						

Ethyl 9-[4-(4,4,5,5-tetramethyl-1,3,2-dioxaborolan-2-yl)phenyl]-9-azabicyclo[3.3.1]nonane-3-carboxylate (**8j**)
α/β >20:1

^{1}H-NMR (300 MHz, $CDCl_3$)

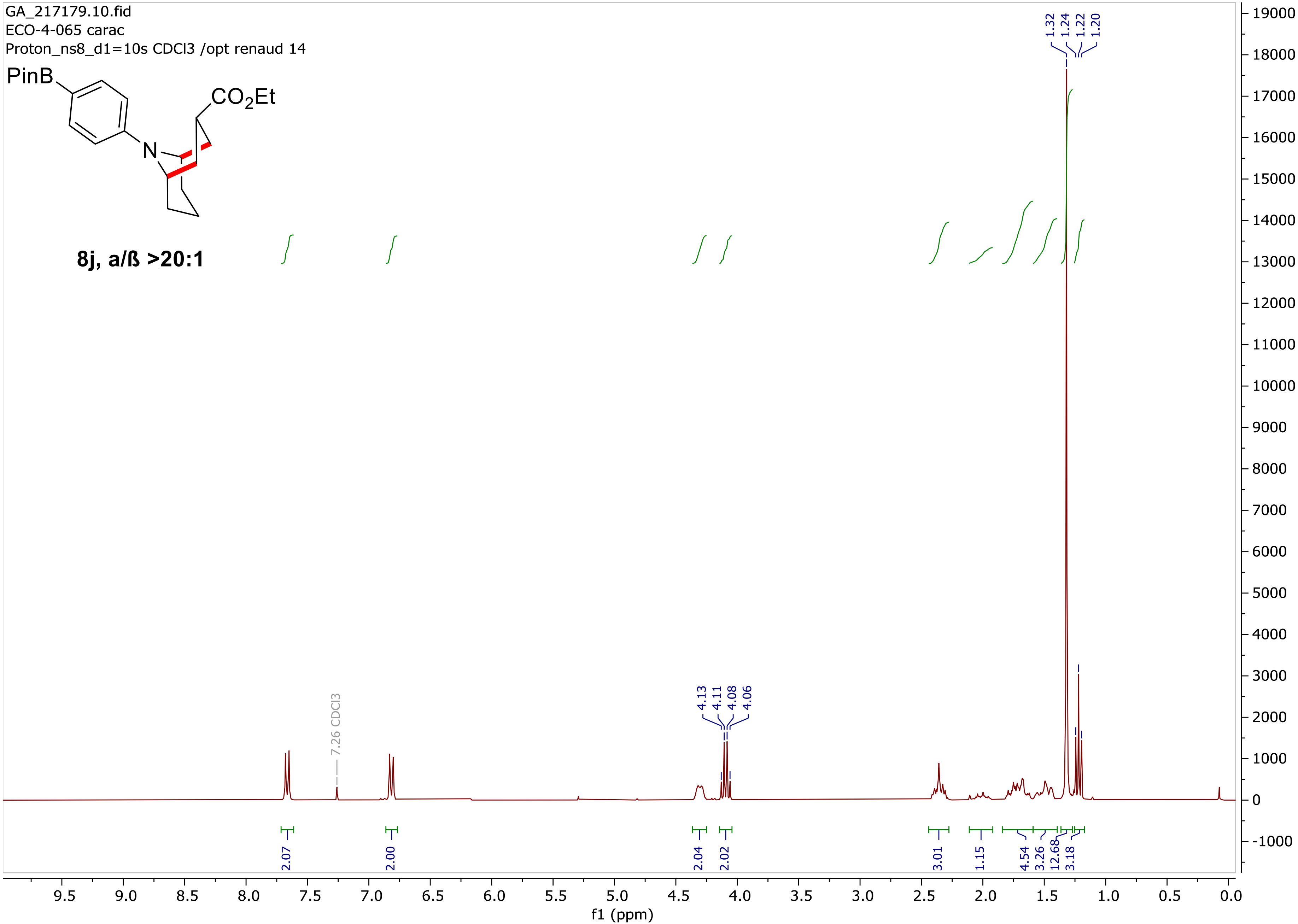

Ethyl 9-[4-(4,4,5,5-tetramethyl-1,3,2-dioxaborolan-2-yl)phenyl]-9-azabicyclo[3.3.1]nonane-3-carboxylate (**8jα**) ^{1}H-NMR (300 MHz, $CDCl_3$)

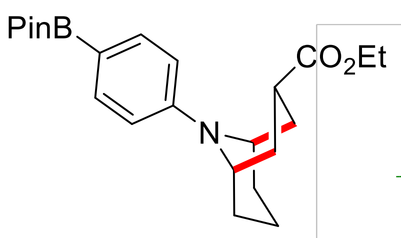

8j

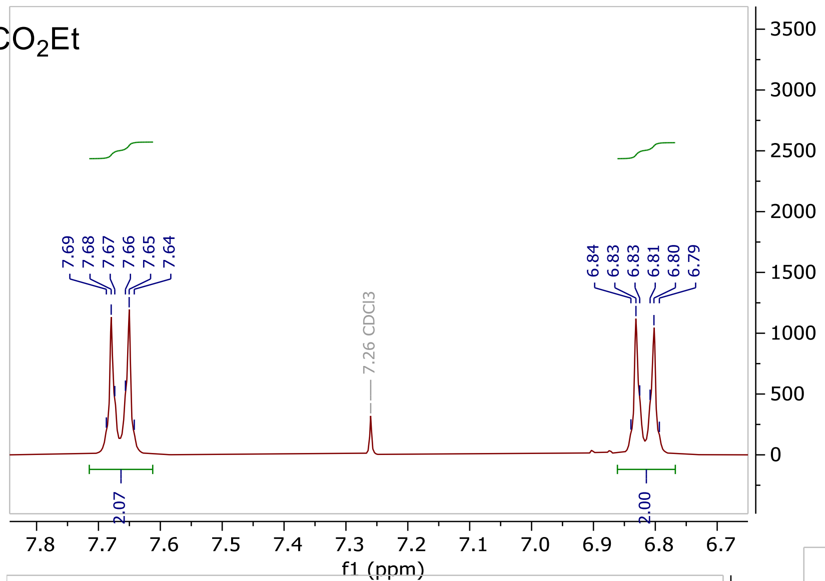

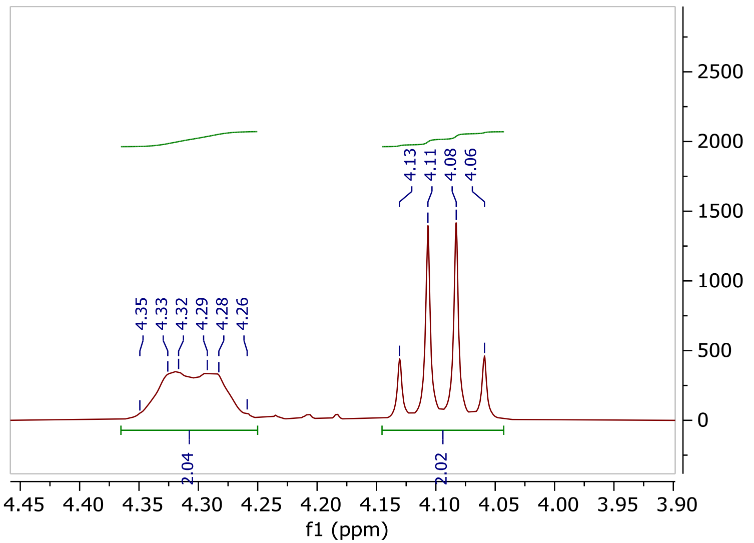

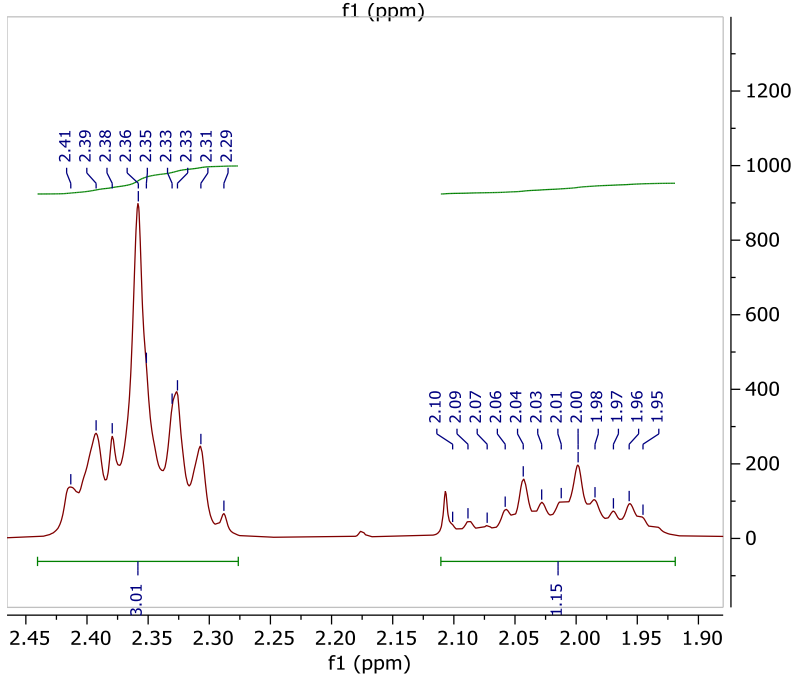

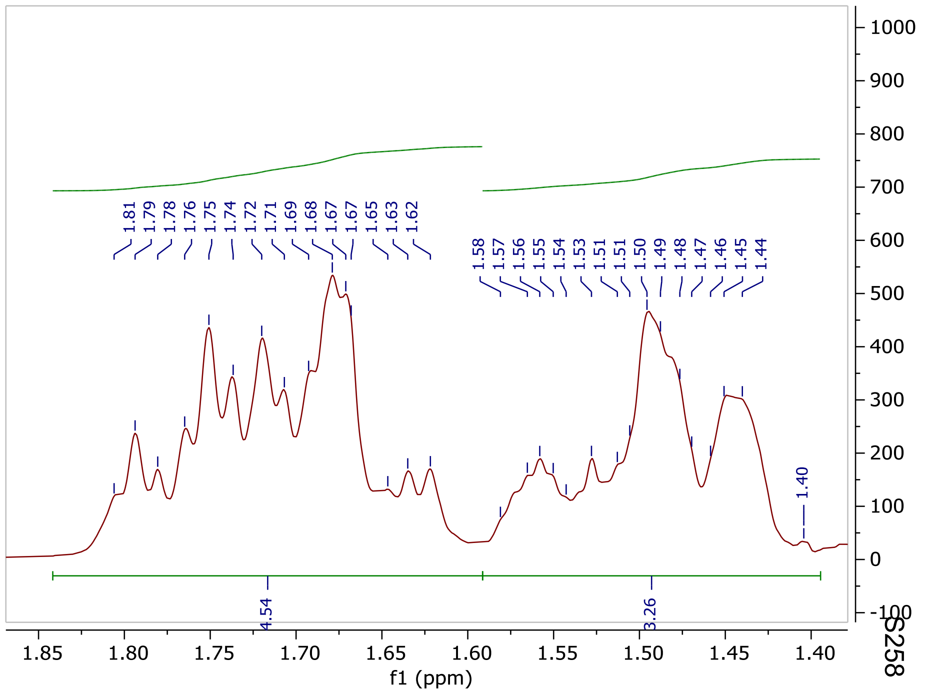

Ethyl 9-[4-(4,4,5,5-tetramethyl-1,3,2-dioxaborolan-2-yl)phenyl]-9-azabicyclo[3.3.1]nonane-3-carboxylate (**8jα**) ^{13}C-NMR (101 MHz, $CDCl_3$)

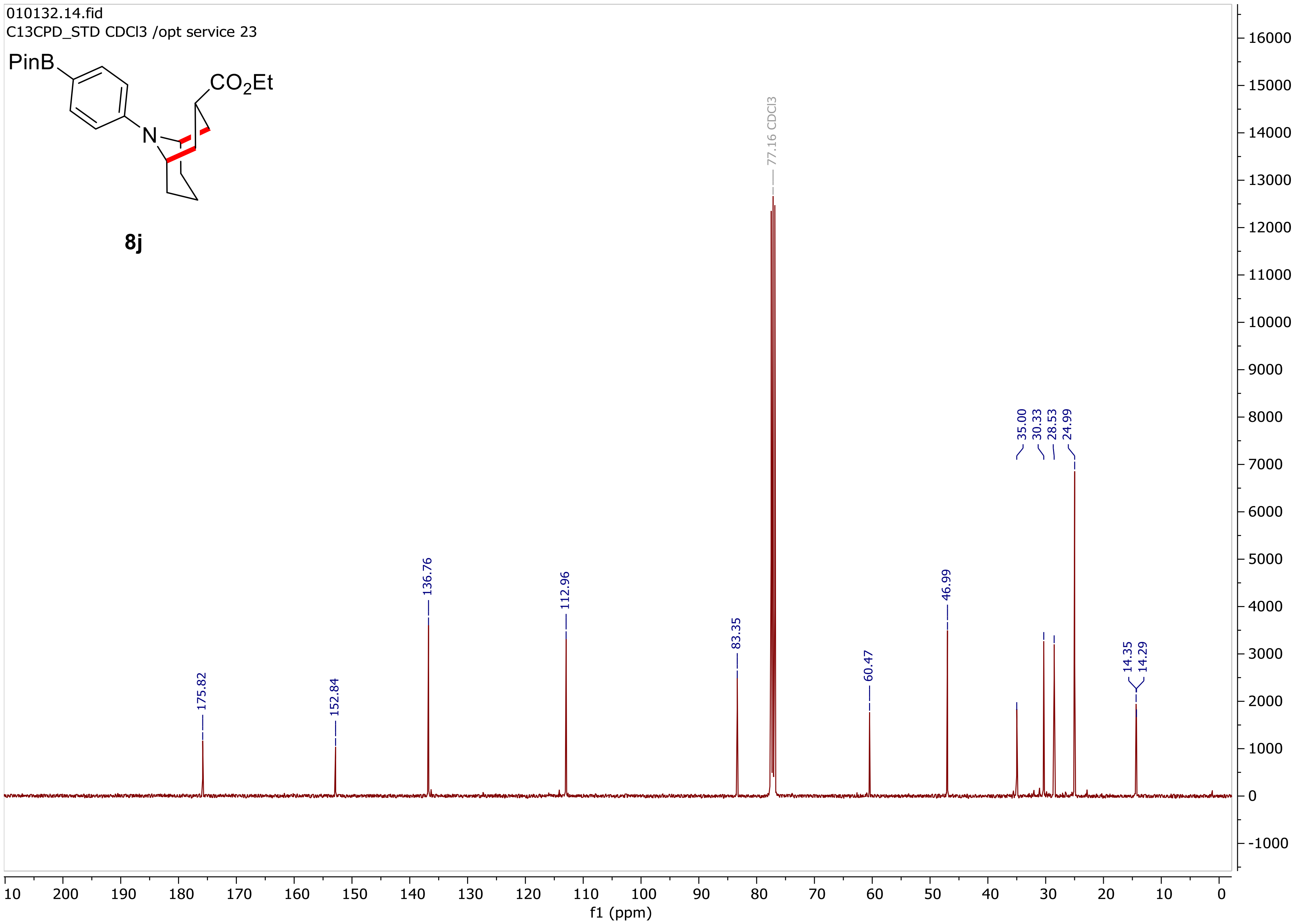

Ethyl 9-[4-(4,4,5,5-tetramethyl-1,3,2-dioxaborolan-2-yl)phenyl]-9-azabicyclo[3.3.1]nonane-3-carboxylate (**8jα**) ^{13}C-NMR (101 MHz, $CDCl_3$)

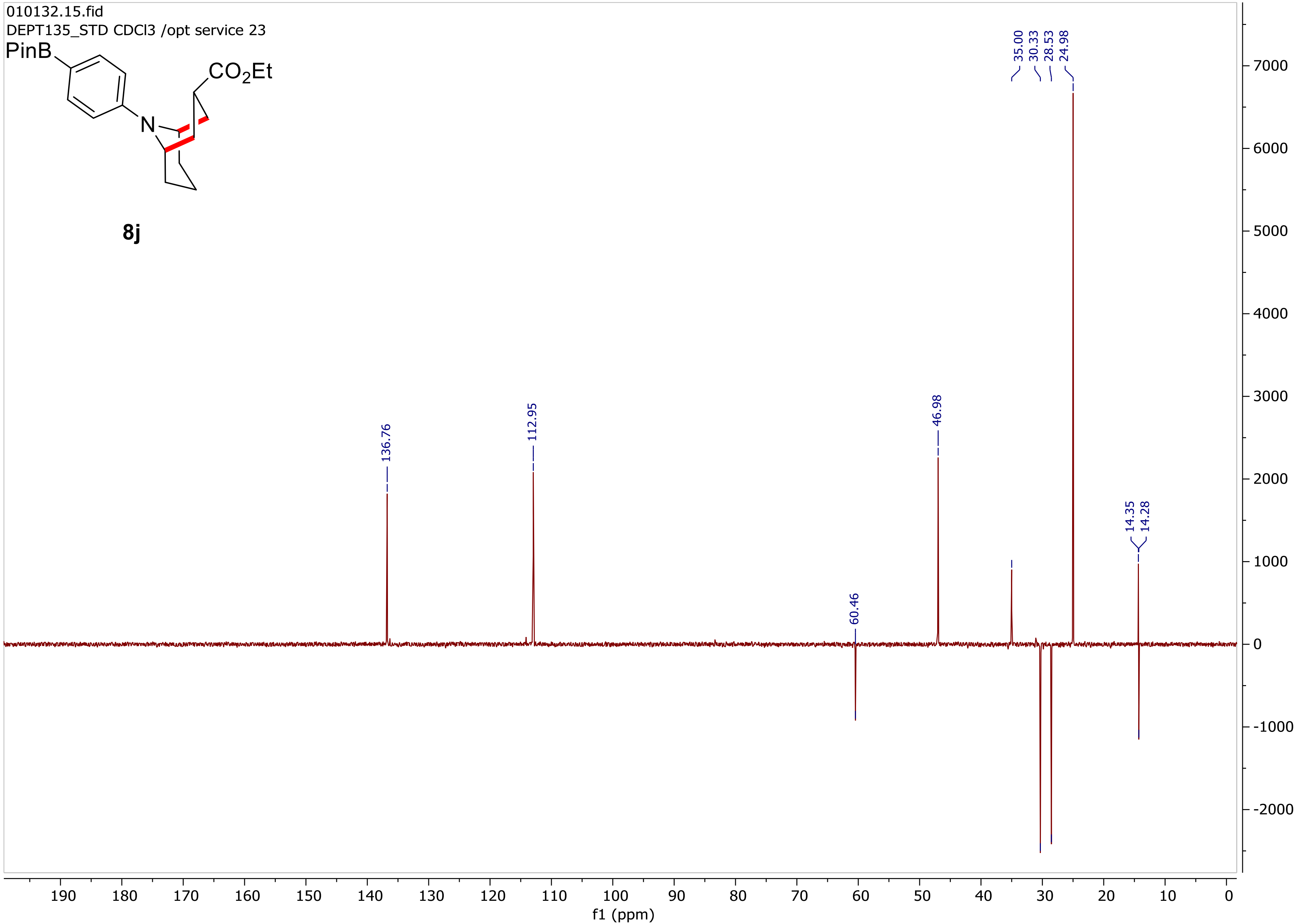

Ethyl 9-[4-(4,4,5,5-tetramethyl-1,3,2-dioxaborolan-2-yl)phenyl]-9-azabicyclo[3.3.1]nonane-3-carboxylate (**8jα**) ^1H, ^1H-COSY NMR (400 MHz, $CDCl_3$)

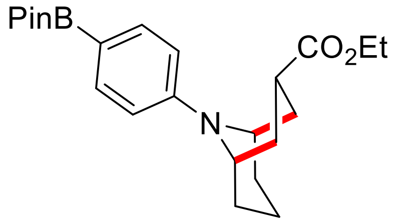

8j

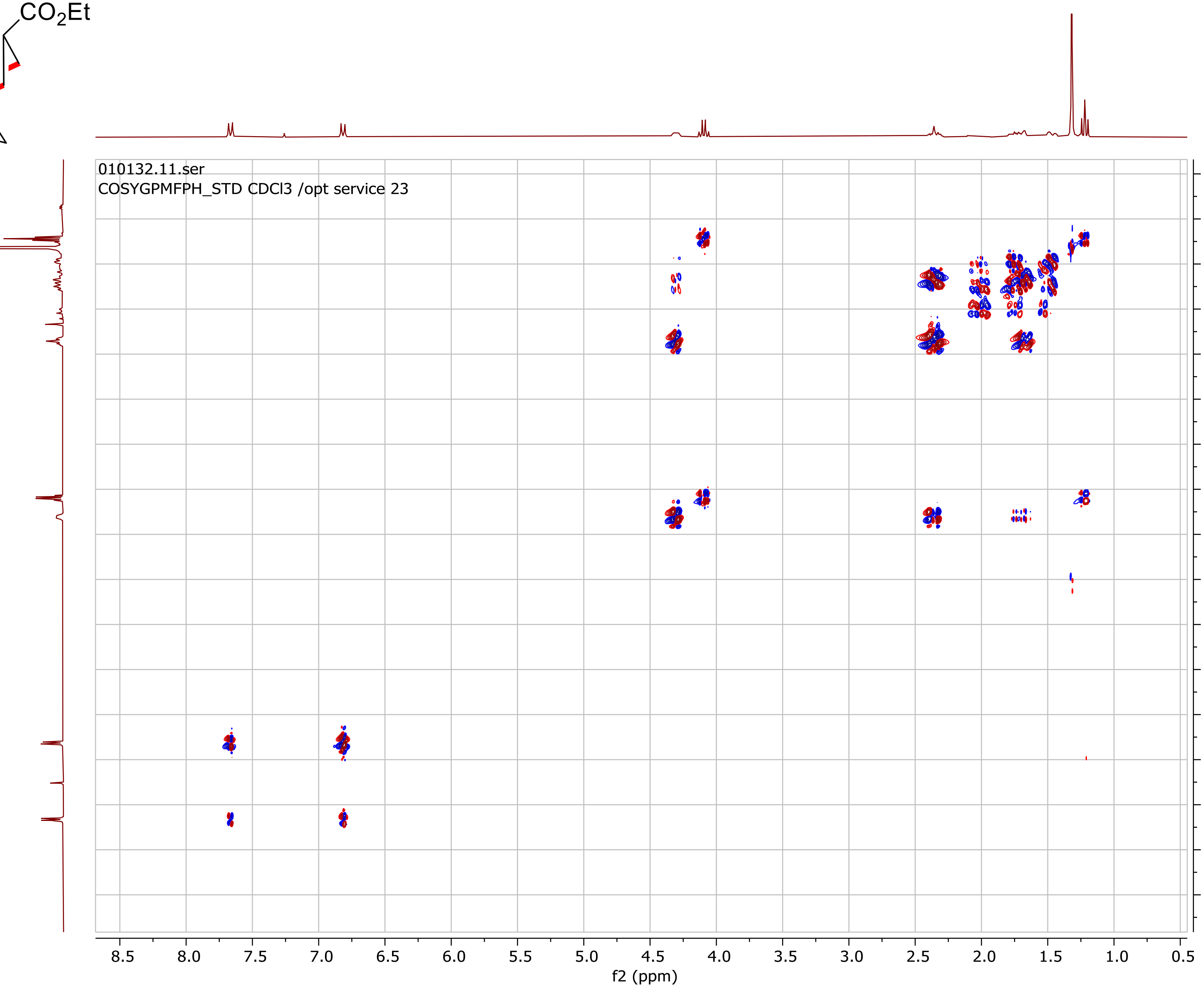

Ethyl 9-[4-(4,4,5,5-tetramethyl-1,3,2-dioxaborolan-2-yl)phenyl]-9-azabicyclo[3.3.1]nonane-3-carboxylate (**8jα**) ^{1}H, ^{13}C-HSQC NMR (400 MHz, $CDCl_3$)

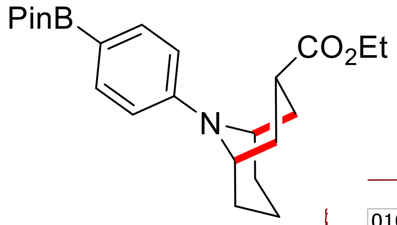

8j

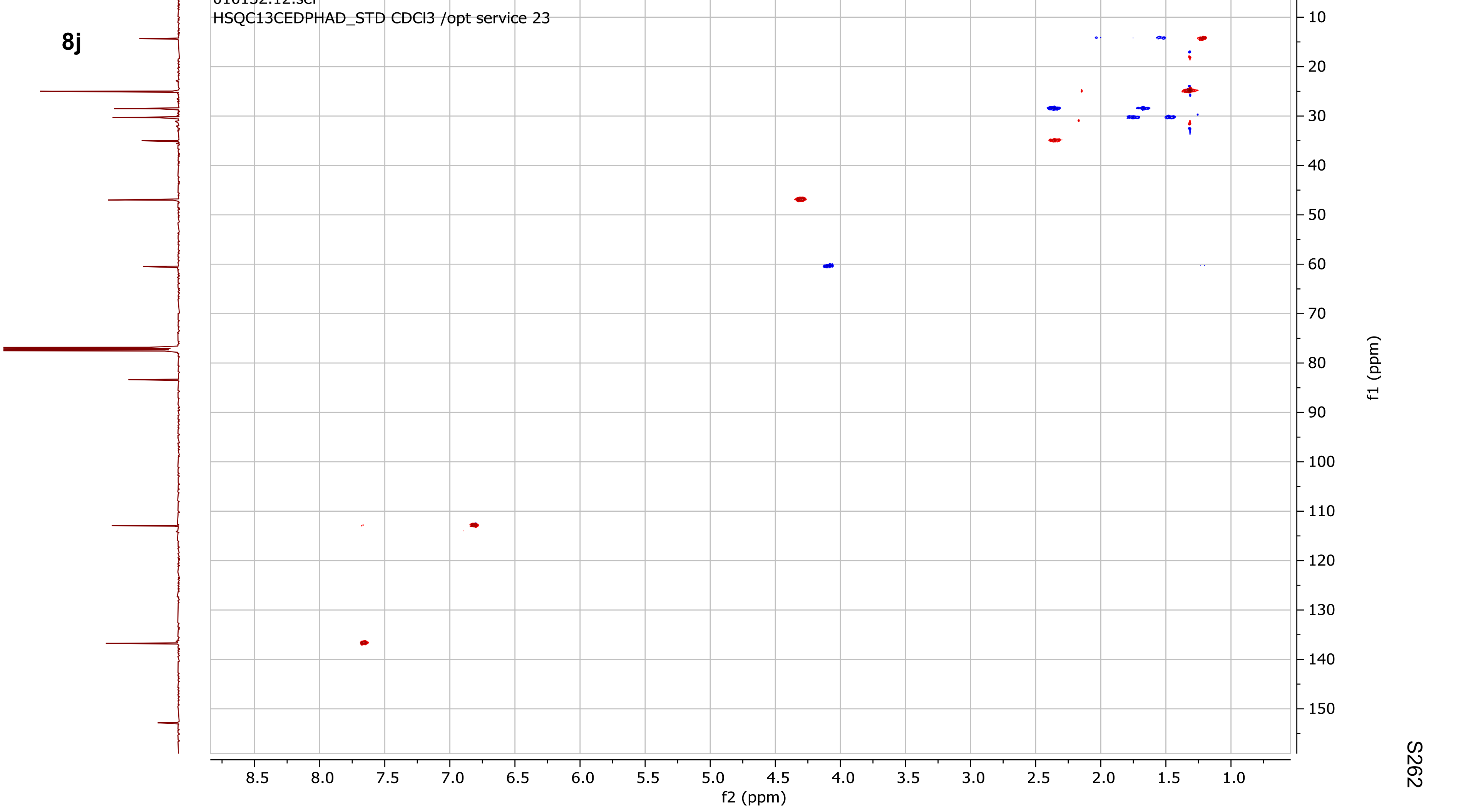

Ethyl 9-[4-(4,4,5,5-tetramethyl-1,3,2-dioxaborolan-2-yl)phenyl]-9-azabicyclo[3.3.1]nonane-3-carboxylate (**8jα**) ^{1}H, ^{13}C-HMBC NMR (400 MHz, $CDCl_3$)

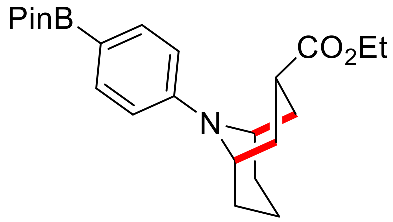

8j

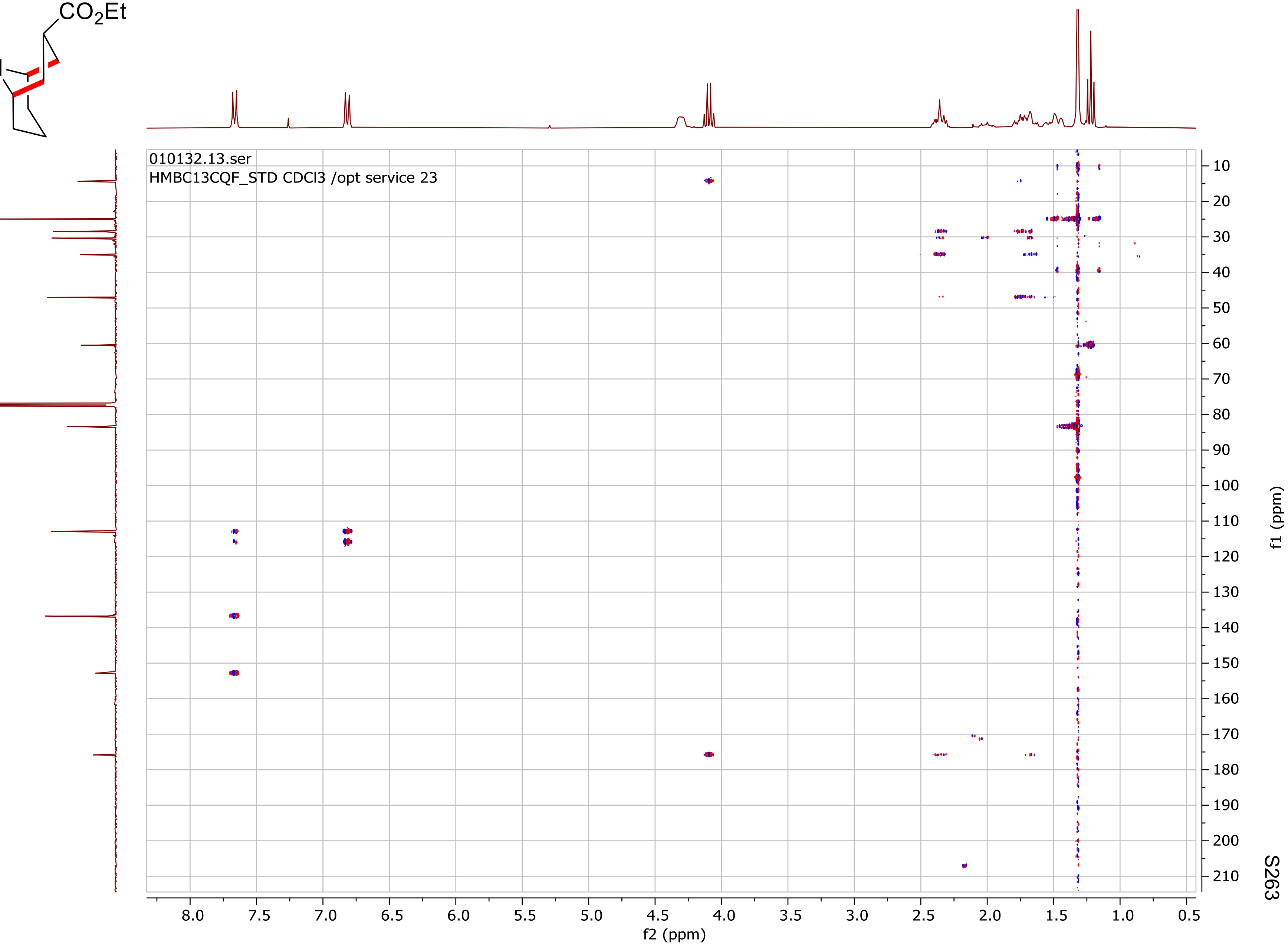

Ethyl 9-[4-(4,4,5,5-tetramethyl-1,3,2-dioxaborolan-2-yl)phenyl]-9-azabicyclo[3.3.1]nonane-3-carboxylate (**8jα**) FT-IR, ATR-diamond

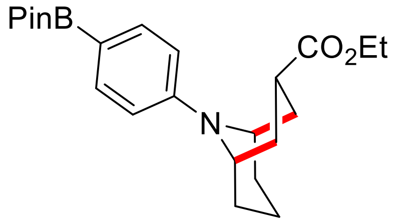

8j

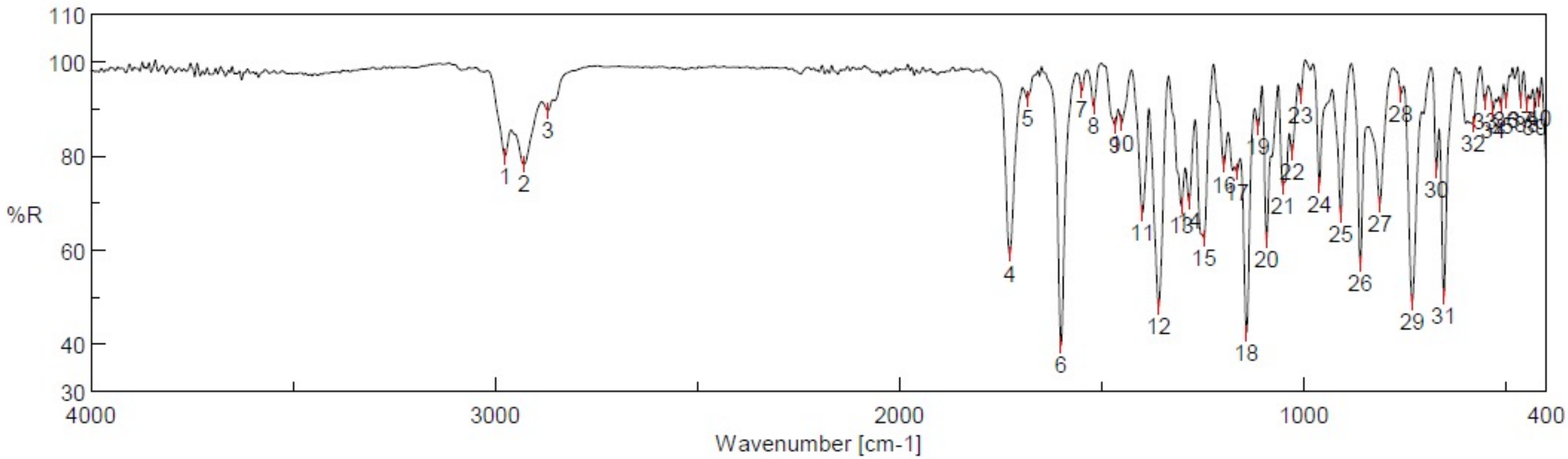

[Result of Peak Picking]

No.	Position	Intensity	No.	Position	Intensity	No.	Position	Intensity
1	2975.62	79.9583	2	2928.38	78.2126	3	2869.56	89.6229
4	1726.94	59.1708	5	1683.55	92.0904	6	1600.63	39.7493
7	1549.52	93.9113	8	1519.63	90.4881	9	1466.6	86.4664
10	1450.21	86.9241	11	1399.1	67.9219	12	1359.57	47.9912
13	1302.68	69.3993	14	1283.39	70.3173	15	1246.75	62.5036
16	1197.58	78.1178	17	1165.76	76.5517	18	1141.65	42.4274
19	1113.69	86.2635	20	1091.51	62.1624	21	1051.98	73.6252
22	1028.84	80.7406	23	1007.62	92.7556	24	961.341	73.8503
25	908.308	67.5861	26	860.096	57.0156	27	811.885	69.8498
28	759.816	93.1418	29	731.853	48.9163	30	672.071	77.0449
31	653.75	50.1423	32	581.433	86.6312	33	551.542	91.5535
34	531.293	89.7891	35	512.008	90.4974	36	499.473	91.8423
37	463.796	91.7799	38	448.369	90.9116	39	428.12	90.181
40	417.513	92.2077						

Ethyl 9-phenyl-3-oxa-9-azabicyclo[3.3.1]nonane-7-carboxylate (**8k**) **α/β 5:1**

^{1}H-NMR (400 MHz, $CDCl_3$)

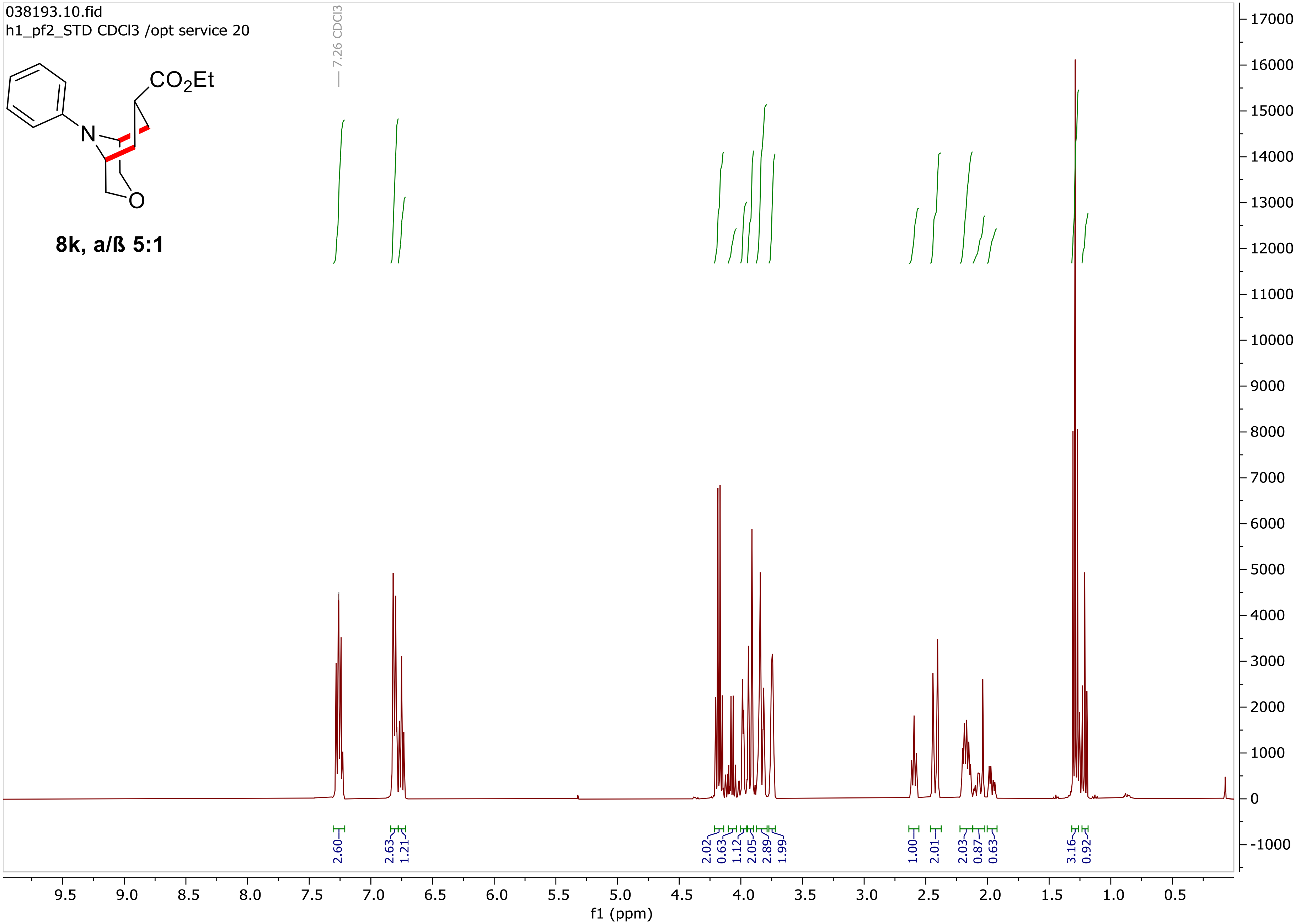

Ethyl 9-phenyl-3-oxa-9-azabicyclo[3.3.1]nonane-7-carboxylate (**8k**) **α/β 5:1**

^{1}H-NMR (400 MHz, $CDCl_3$)

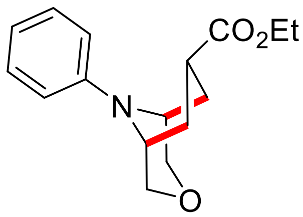

8k, a/ß 5:1

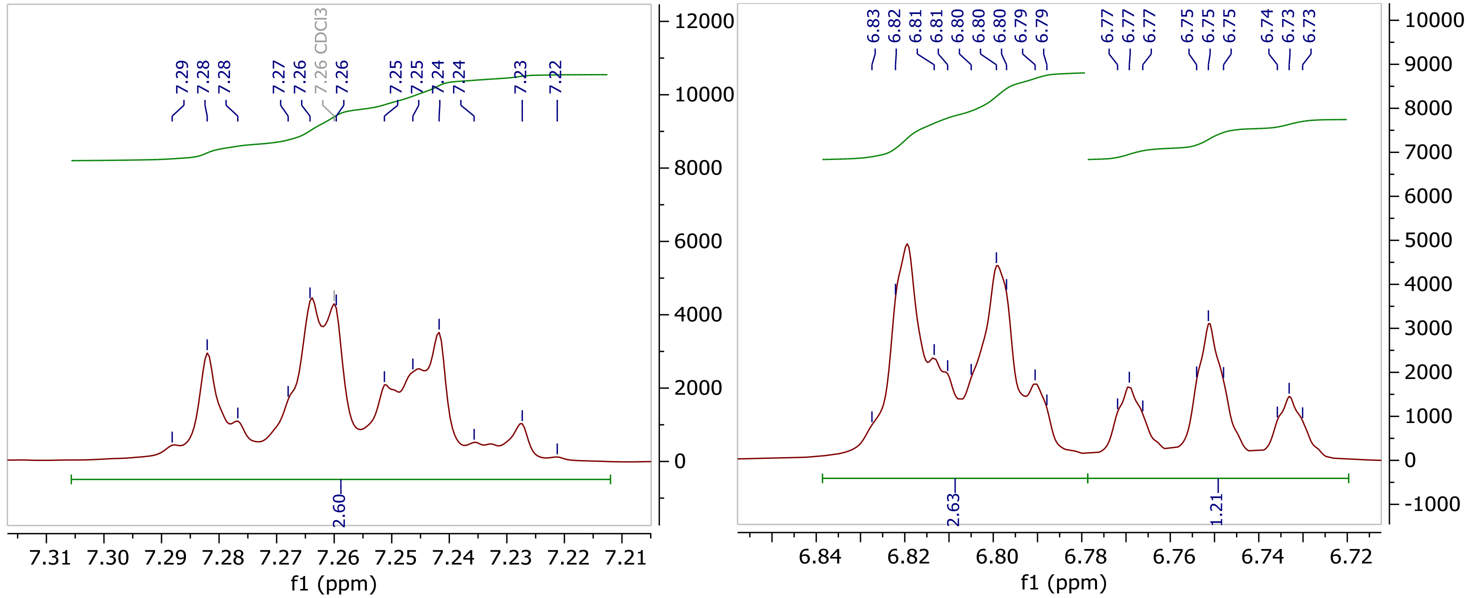

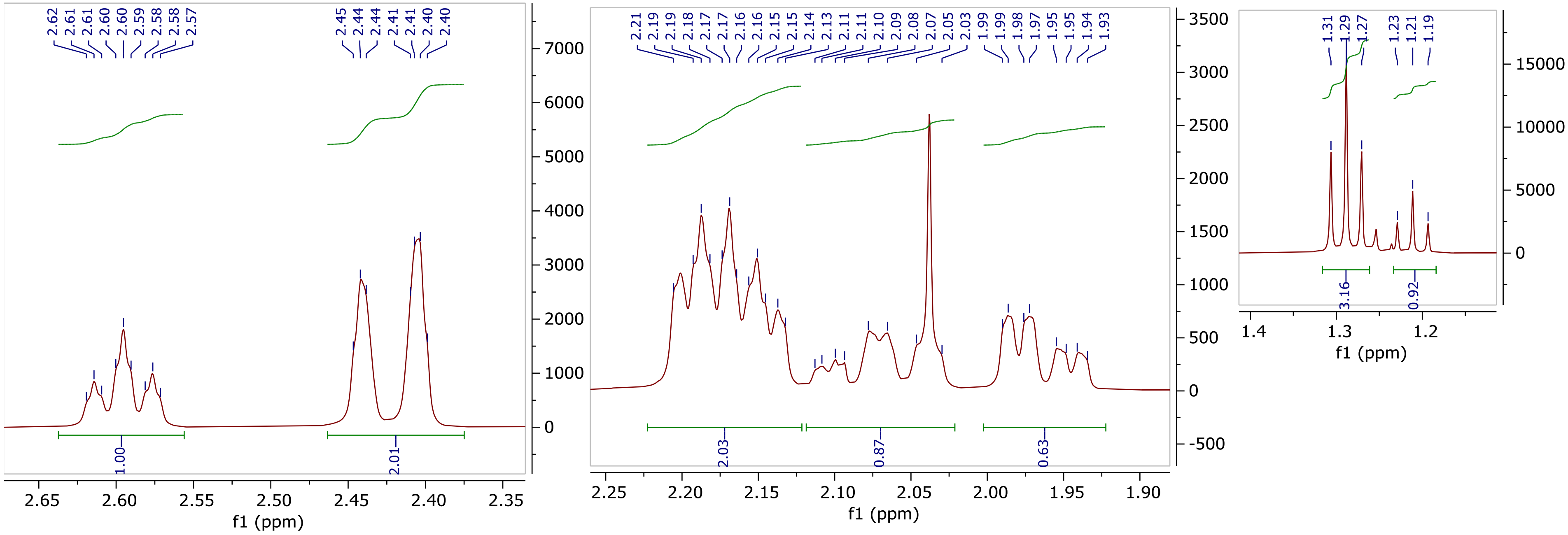

Ethyl 9-phenyl-3-oxa-9-azabicyclo[3.3.1]nonane-7-carboxylate (**8k**) **α/β 5:1**

^{1}H-NMR (400 MHz, $CDCl_3$)

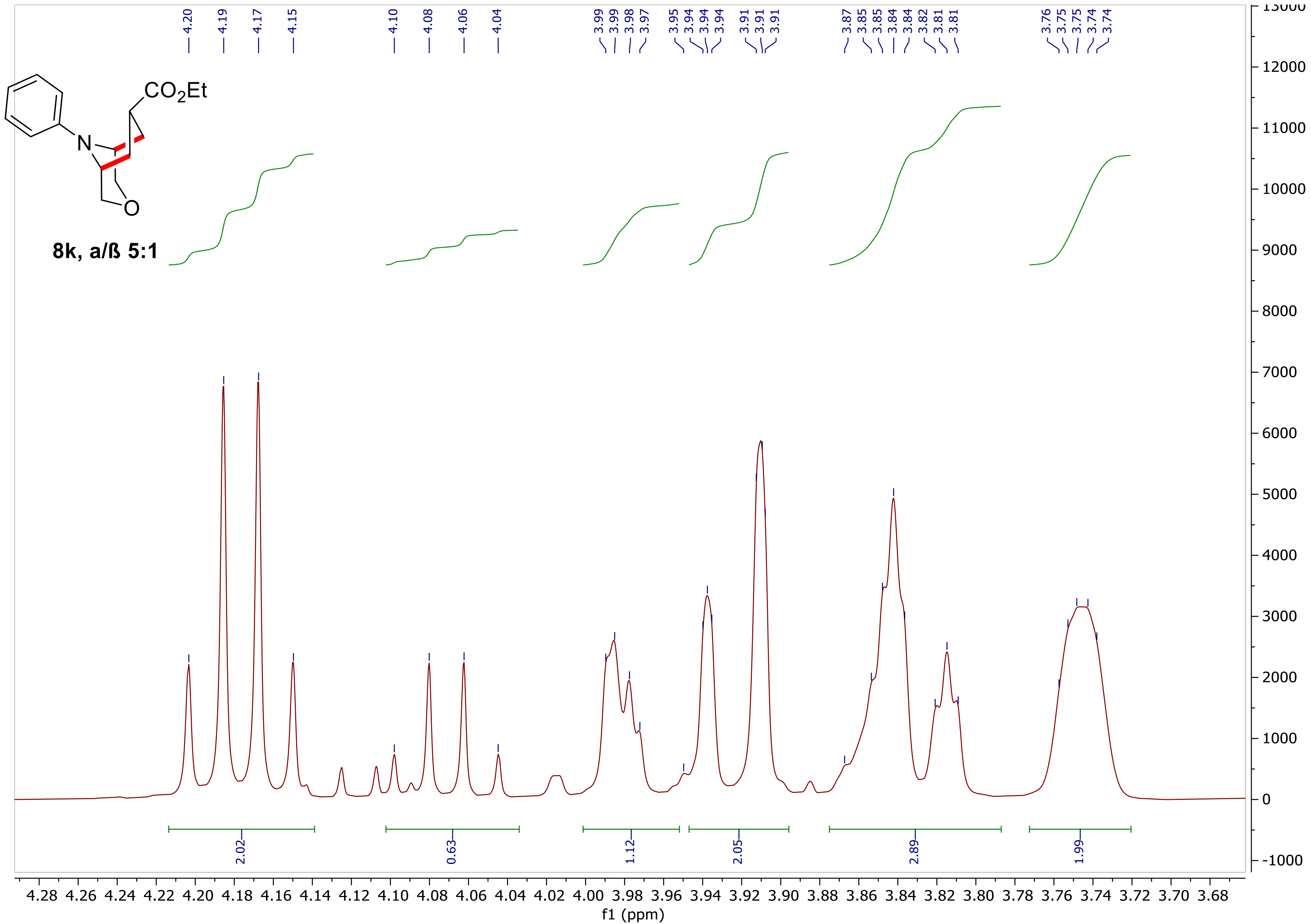

Ethyl 9-phenyl-3-oxa-9-azabicyclo[3.3.1]nonane-7-carboxylate (**8k**) **α/β 5:1**

^{13}C-NMR (101 MHz, $CDCl_3$)

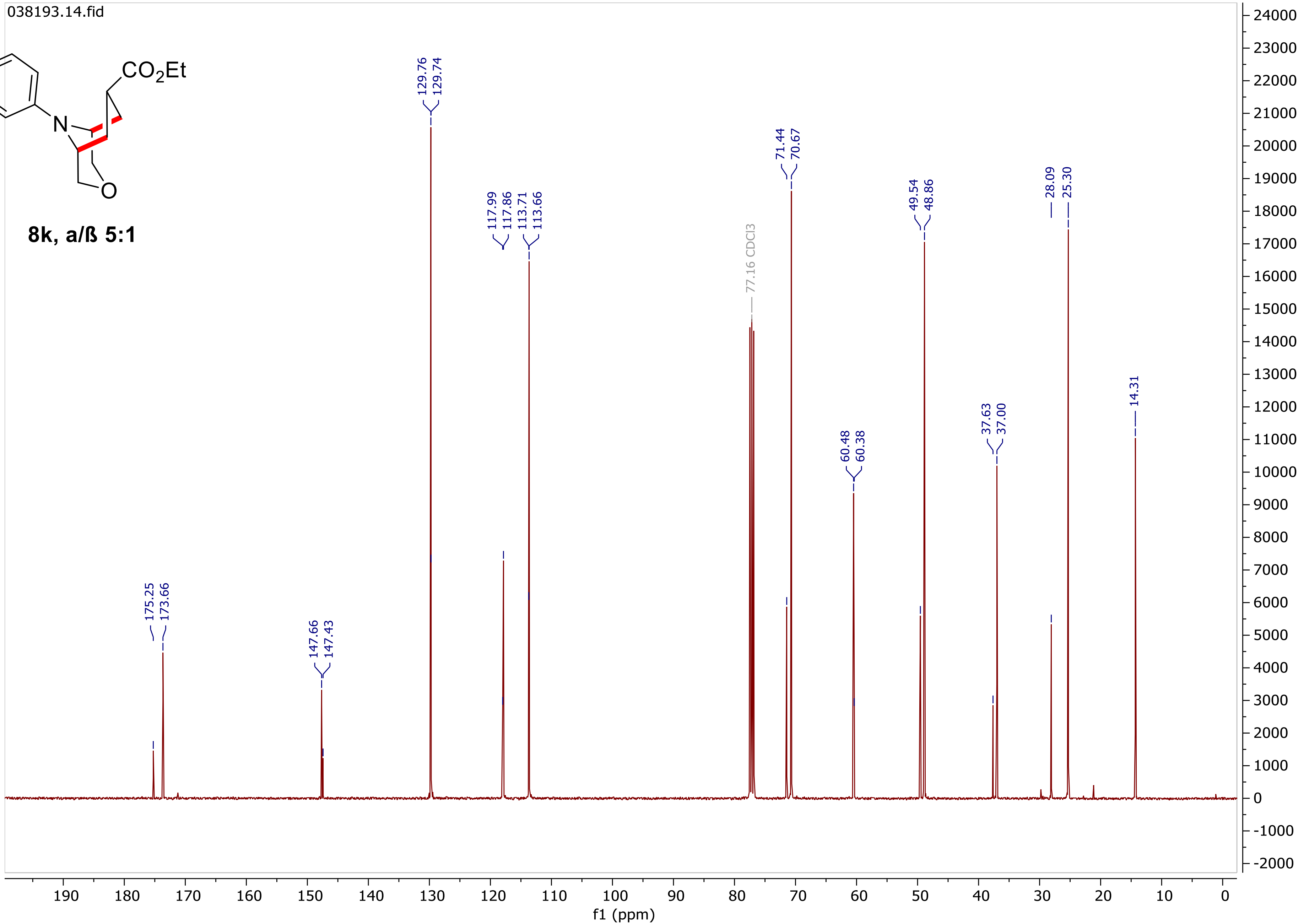

Ethyl 9-phenyl-3-oxa-9-azabicyclo[3.3.1]nonane-7-carboxylate (**8k**) **α/β 5:1**

^{13}C-NMR (101 MHz, $CDCl_3$)

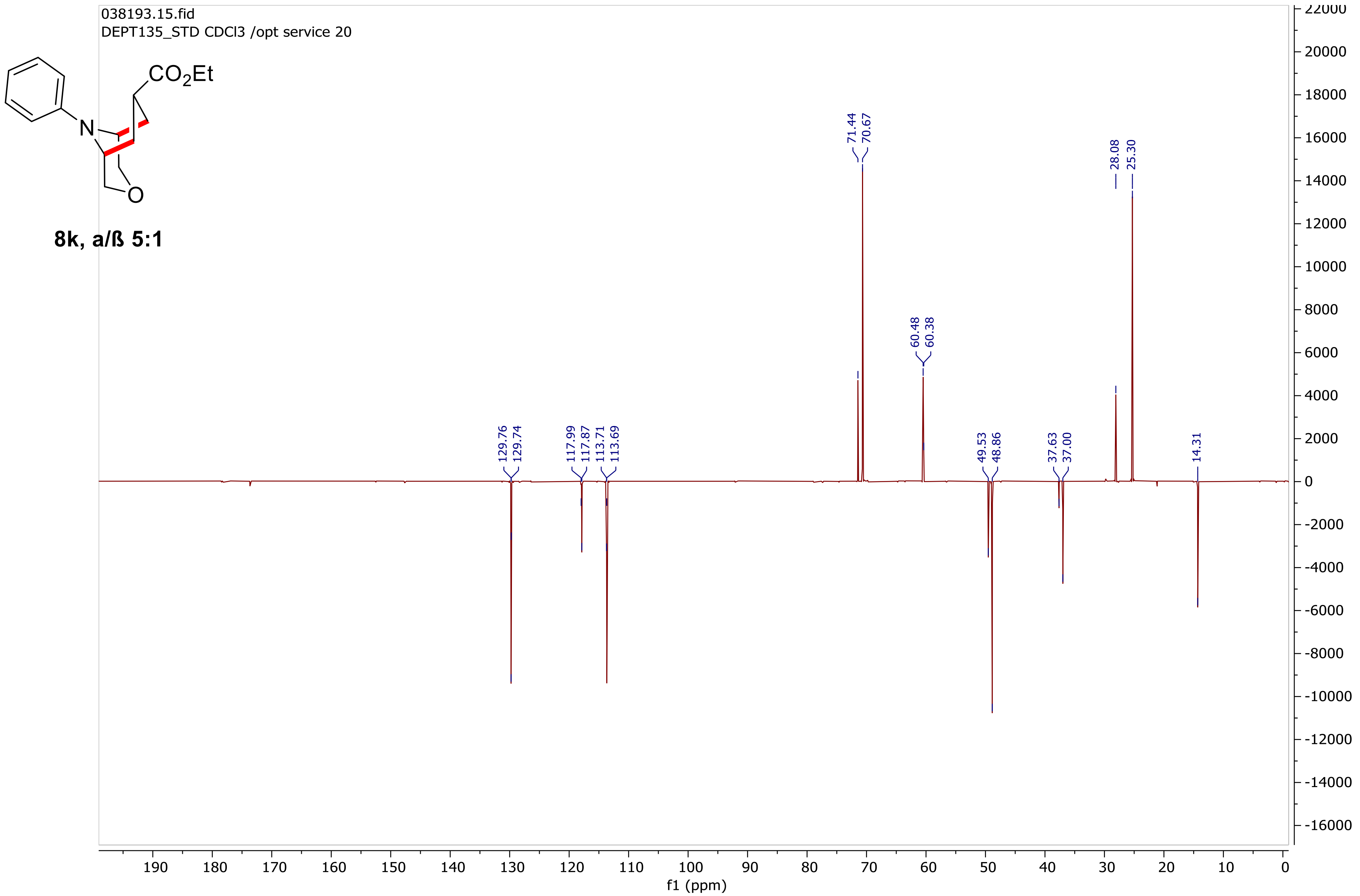

Ethyl 9-phenyl-3-oxa-9-azabicyclo[3.3.1]nonane-7-carboxylate (**8k**) **α/β 5:1**

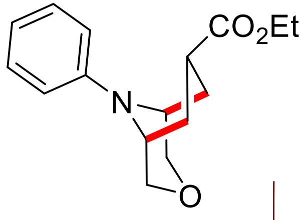

8k, a/ß 5:1

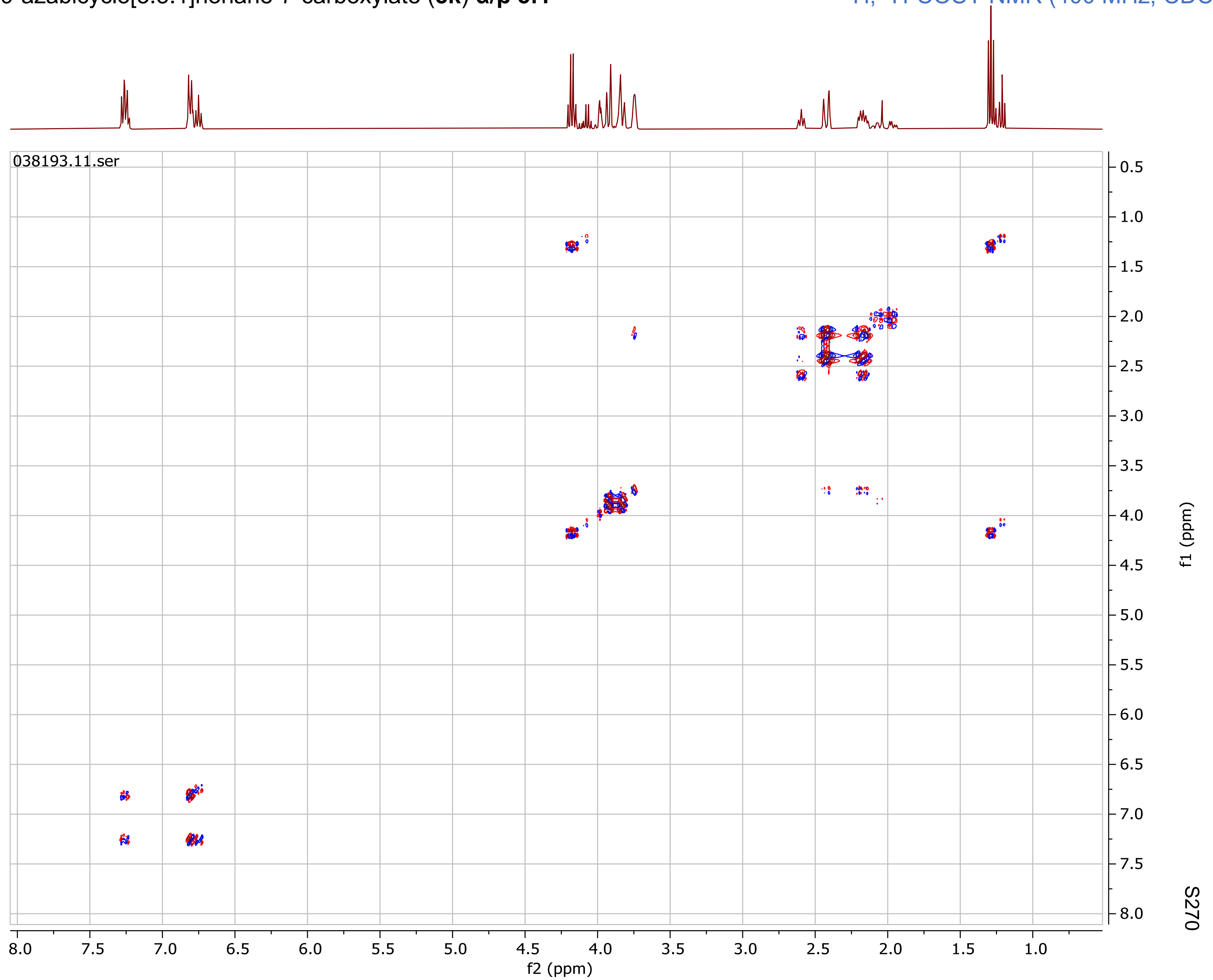

Ethyl 9-phenyl-3-oxa-9-azabicyclo[3.3.1]nonane-7-carboxylate (**8k**) **α/β 5:1**

^1H, ^{13}C-HSQC NMR (400 MHz, $CDCl_3$)

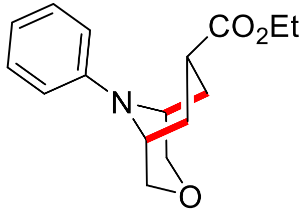

8k, a/ß 5:1

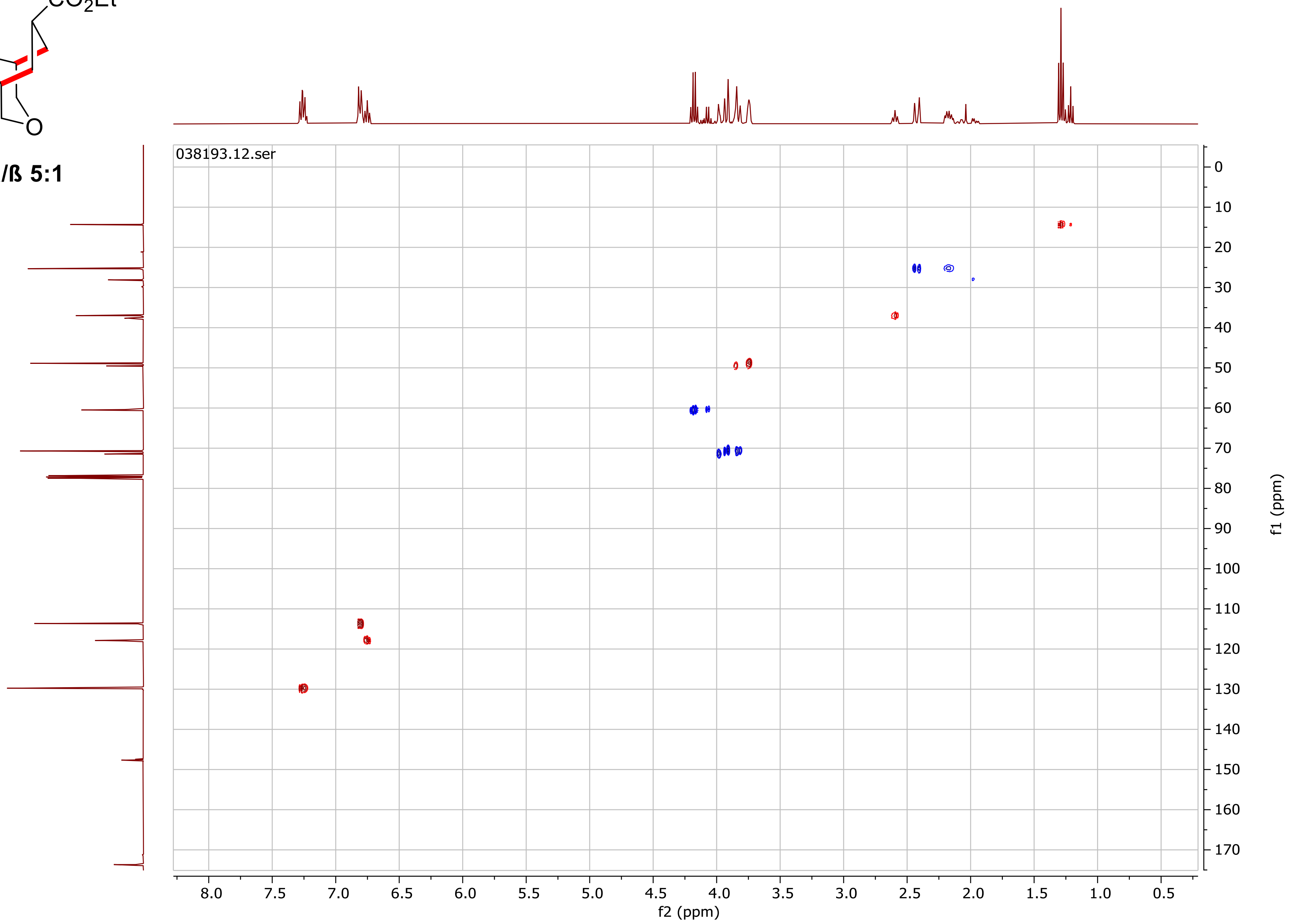

Ethyl 9-phenyl-3-oxa-9-azabicyclo[3.3.1]nonane-7-carboxylate (**8k**) **α/β 5:1**

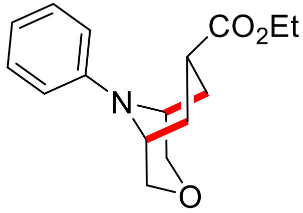

8k, a/ß 5:1

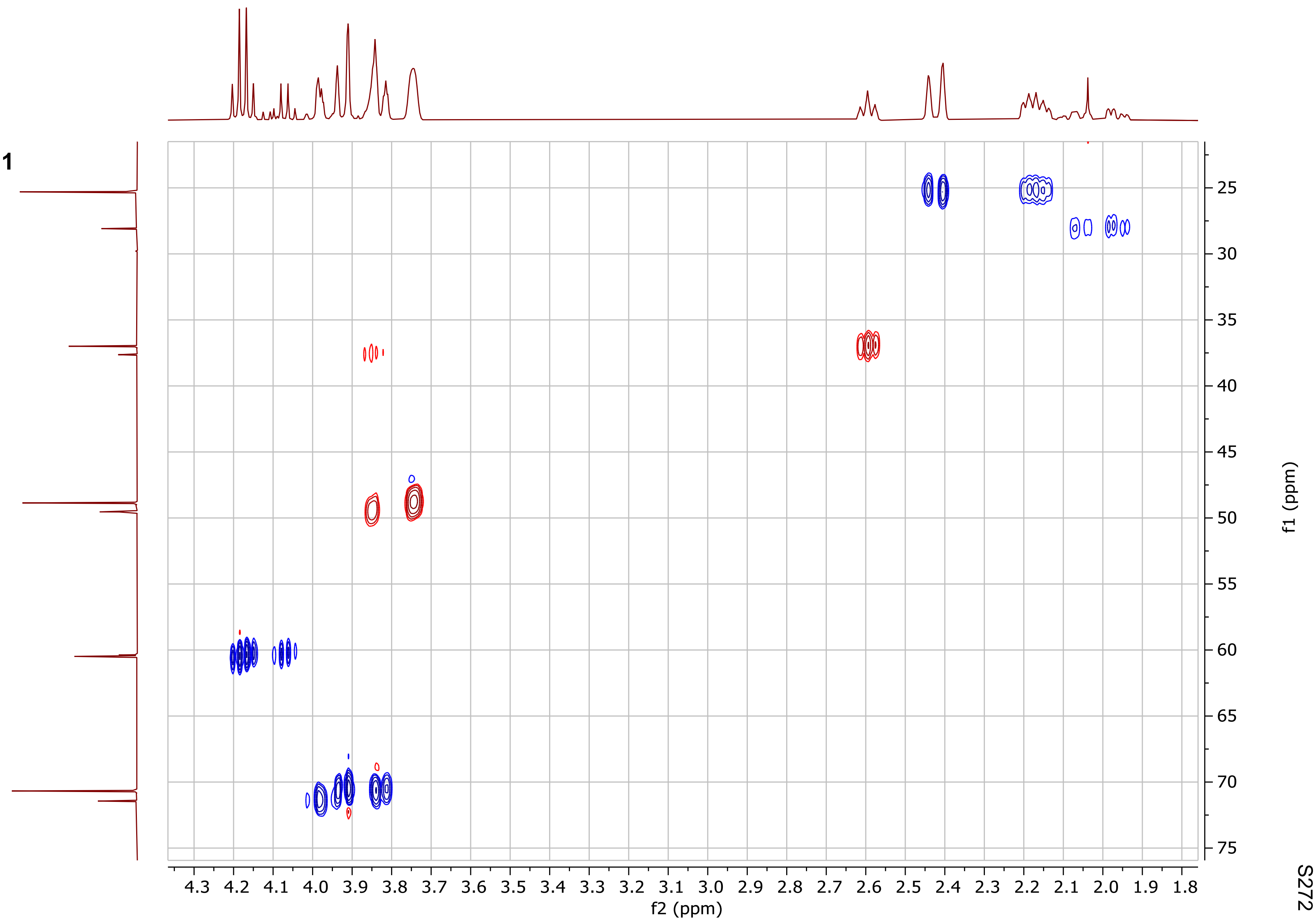

Ethyl 9-phenyl-3-oxa-9-azabicyclo[3.3.1]nonane-7-carboxylate (**8k**) **α/β 5:1**

^{1}H, ^{13}C-HMBC NMR (400 MHz, $CDCl_3$)

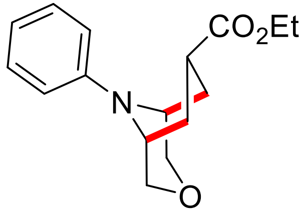

8k, a/ß 5:1

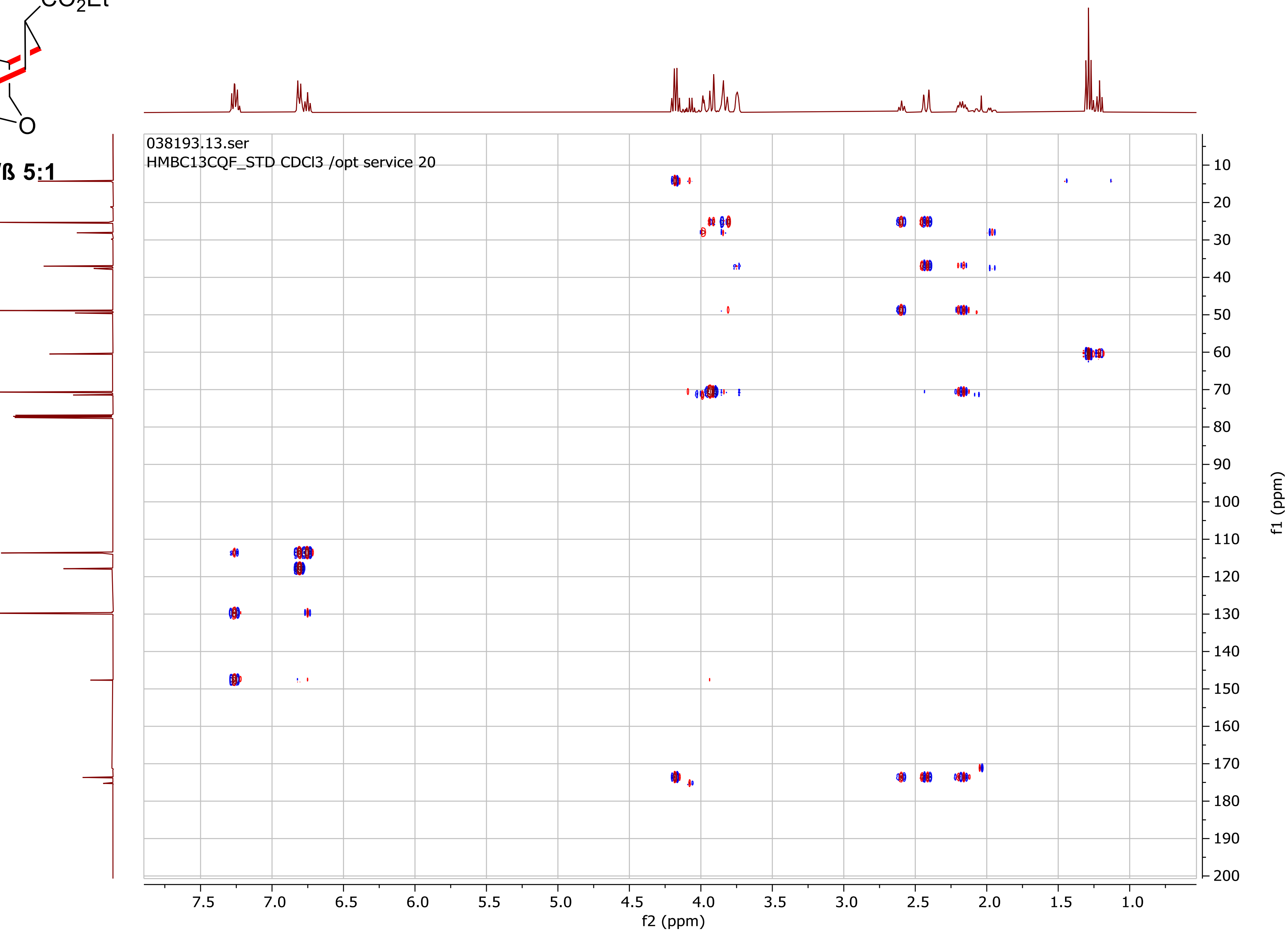

Ethyl 9-phenyl-3-oxa-9-azabicyclo[3.3.1]nonane-7-carboxylate (**8k**) **α/β 5:1**

FT-IR, ATR-diamond

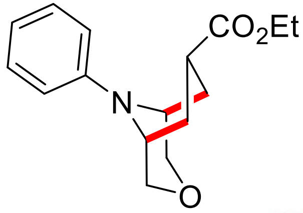

8k, a/ß 5:1

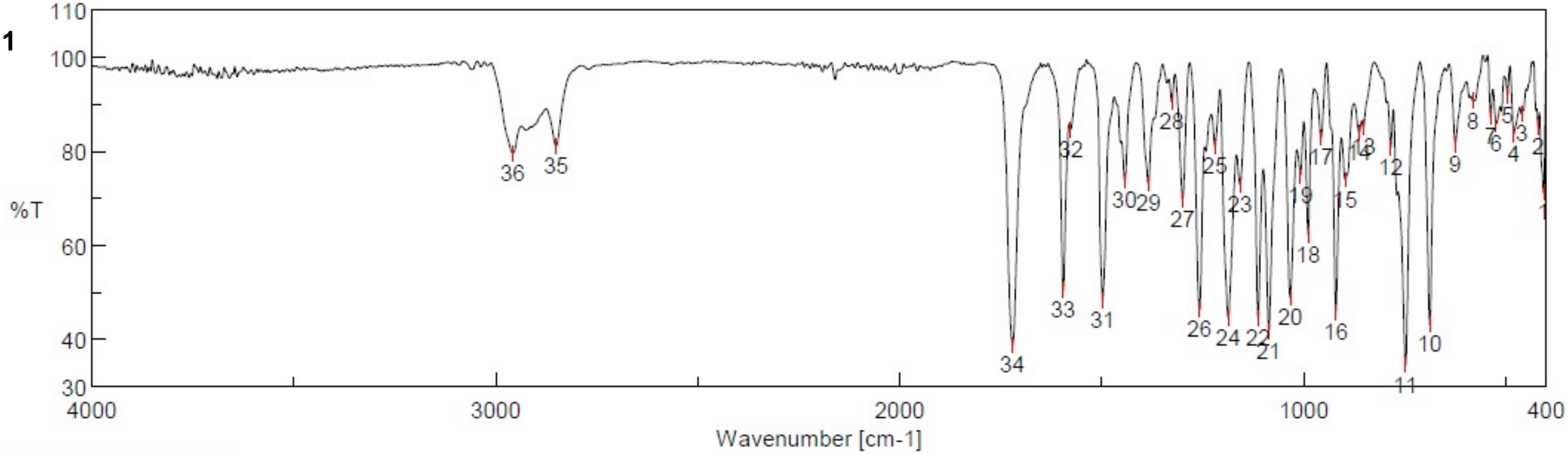

[Result of Peak Picking]

No.	Position	Intensity	No.	Position	Intensity	No.	Position	Intensity
1	405.942	71	2	419.442	85	3	460.904	88
4	480.188	84	5	495.616	92	6	523.579	86
7	537.078	87	8	581.433	91	9	624.823	82
10	688.463	43	11	748.245	35	12	784.886	81
13	852.382	85	14	862.989	84	15	897.701	74
16	920.843	46	17	957.484	83	18	989.304	62
19	1008.59	75	20	1033.66	49	21	1086.69	42
22	1112.73	44	23	1158.04	73	24	1186.97	45
25	1219.76	81	26	1258.32	46	27	1299.79	70
28	1326.79	90	29	1384.64	73	30	1443.46	74
31	1497.45	48	32	1576.52	85	33	1595.81	50
34	1721.16	39	35	2851.24	81	36	2958.27	80

O3-tert-butyl O7-ethyl 9-phenyl-3,9-diazabicyclo[3.3.1]nonane-3,7-dicarboxylate (**8l**) **α/β >12:1**

^{1}H-NMR (400 MHz, $CDCl_3$)

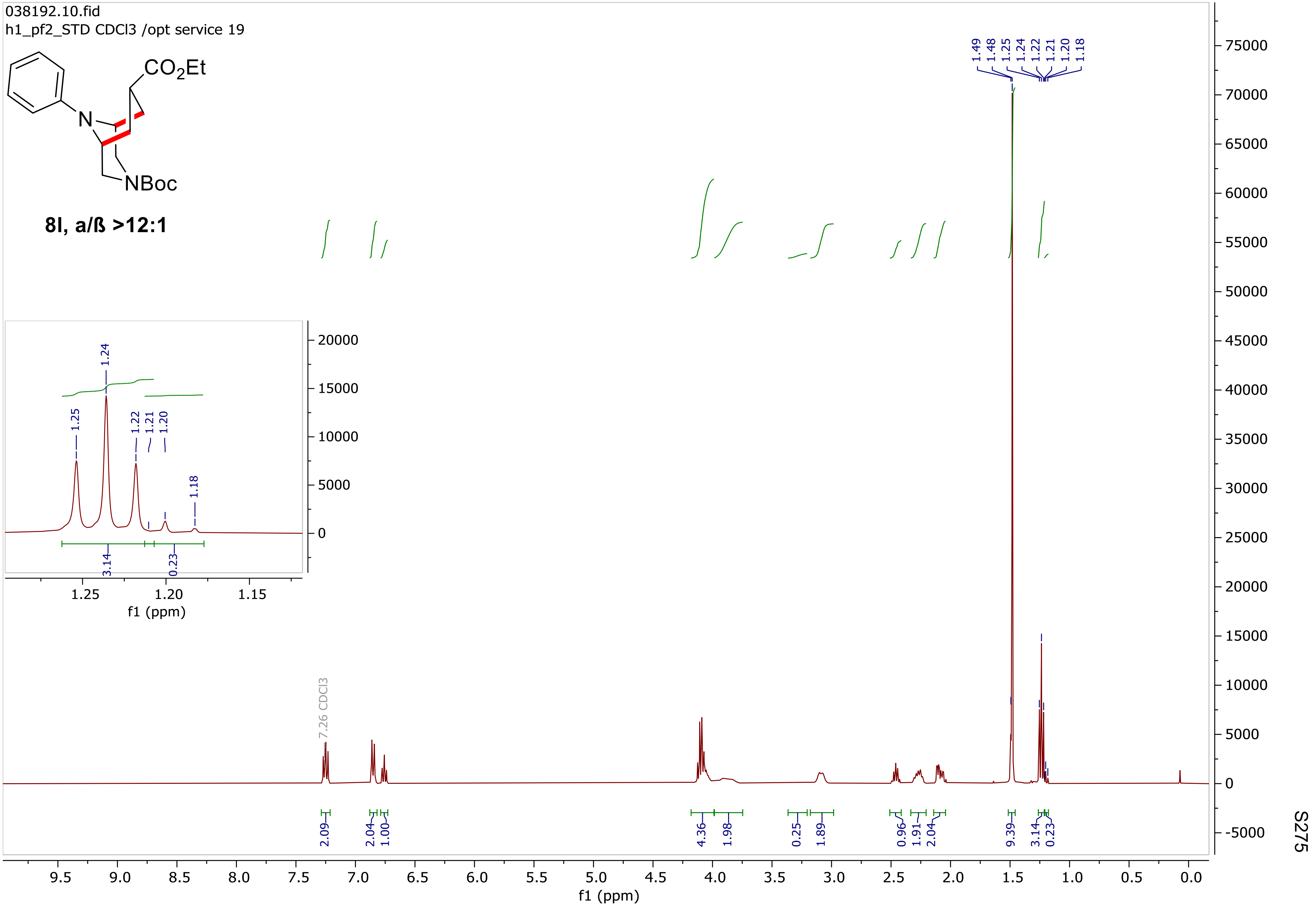

O3-tert-butyl O7-ethyl 9-phenyl-3,9-diazabicyclo[3.3.1]nonane-3,7-dicarboxylate (**8l**) **α/β >12:1**

^{1}H-NMR (400 MHz, $CDCl_3$)

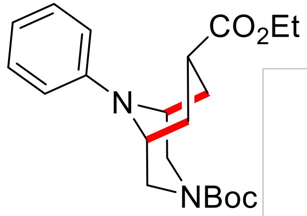

8l, a/ß >12:1

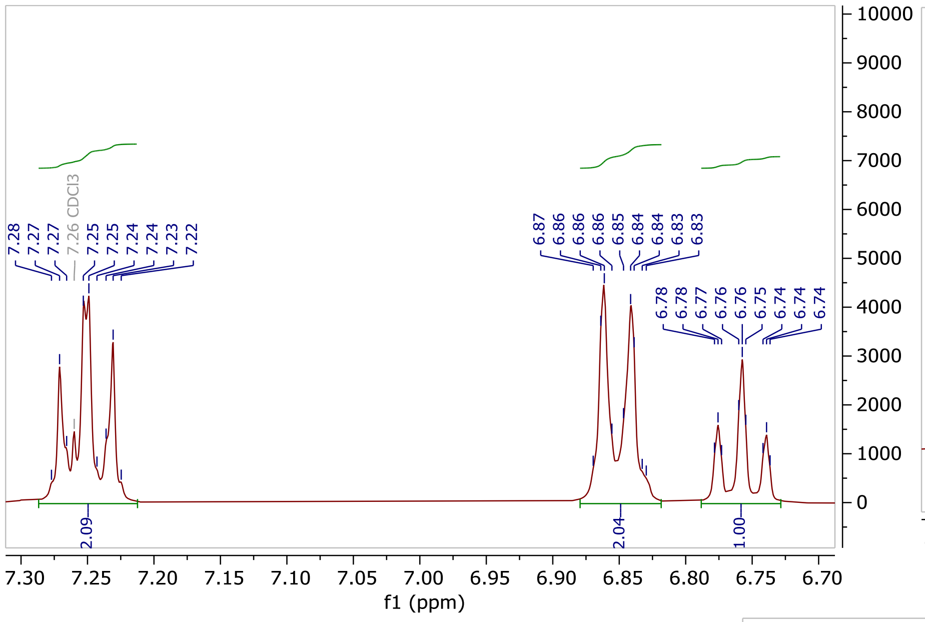

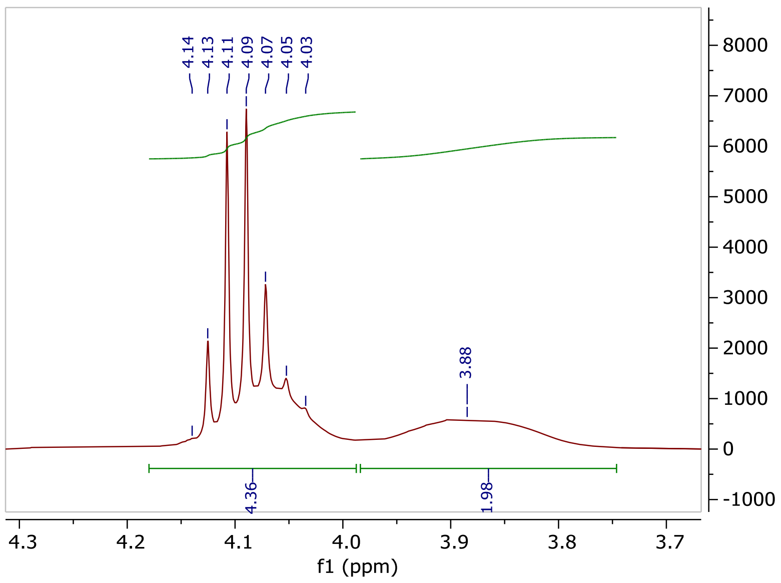

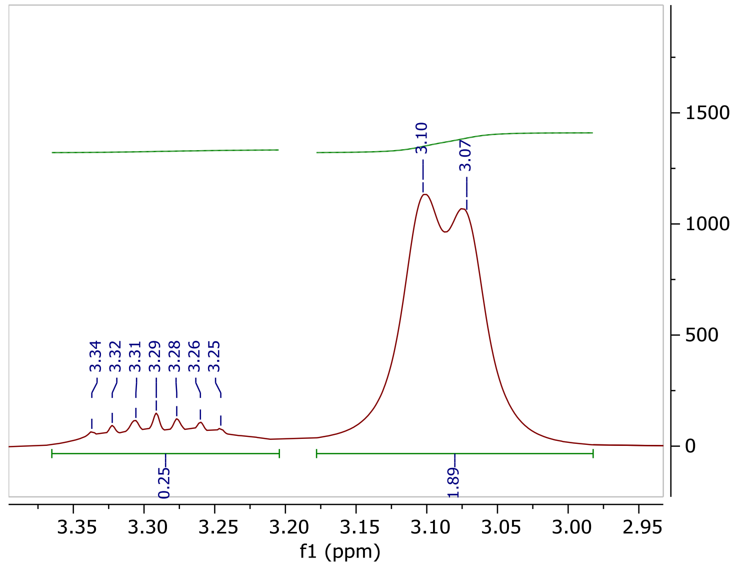

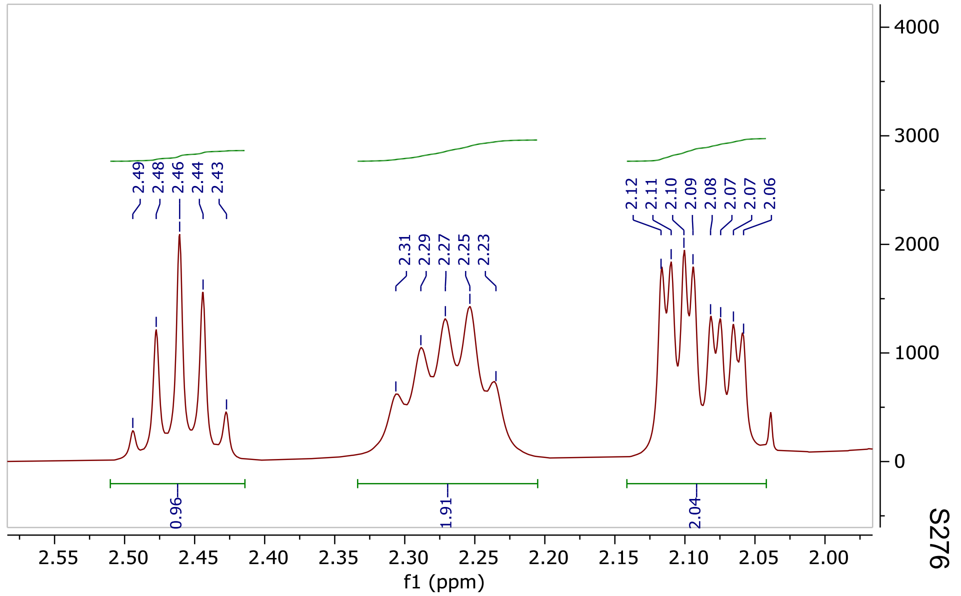

O3-tert-butyl O7-ethyl 9-phenyl-3,9-diazabicyclo[3.3.1]nonane-3,7-dicarboxylate (**8l**) **α/β >12:1**

^{13}C-NMR (101 MHz, $CDCl_3$)

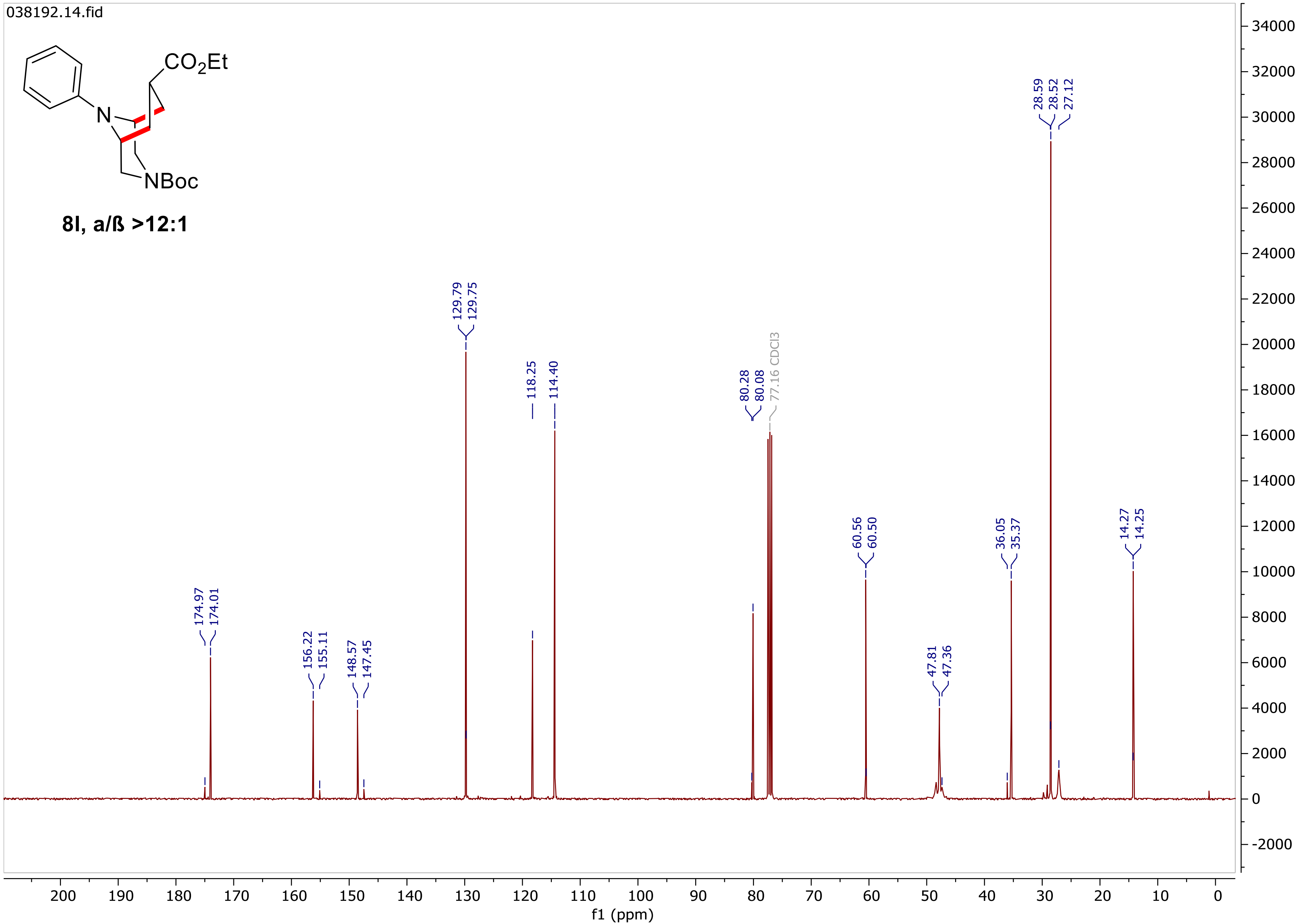

O3-tert-butyl O7-ethyl 9-phenyl-3,9-diazabicyclo[3.3.1]nonane-3,7-dicarboxylate (**8l**) **α/β >12:1**

^{13}C-NMR (101 MHz, $CDCl_3$)

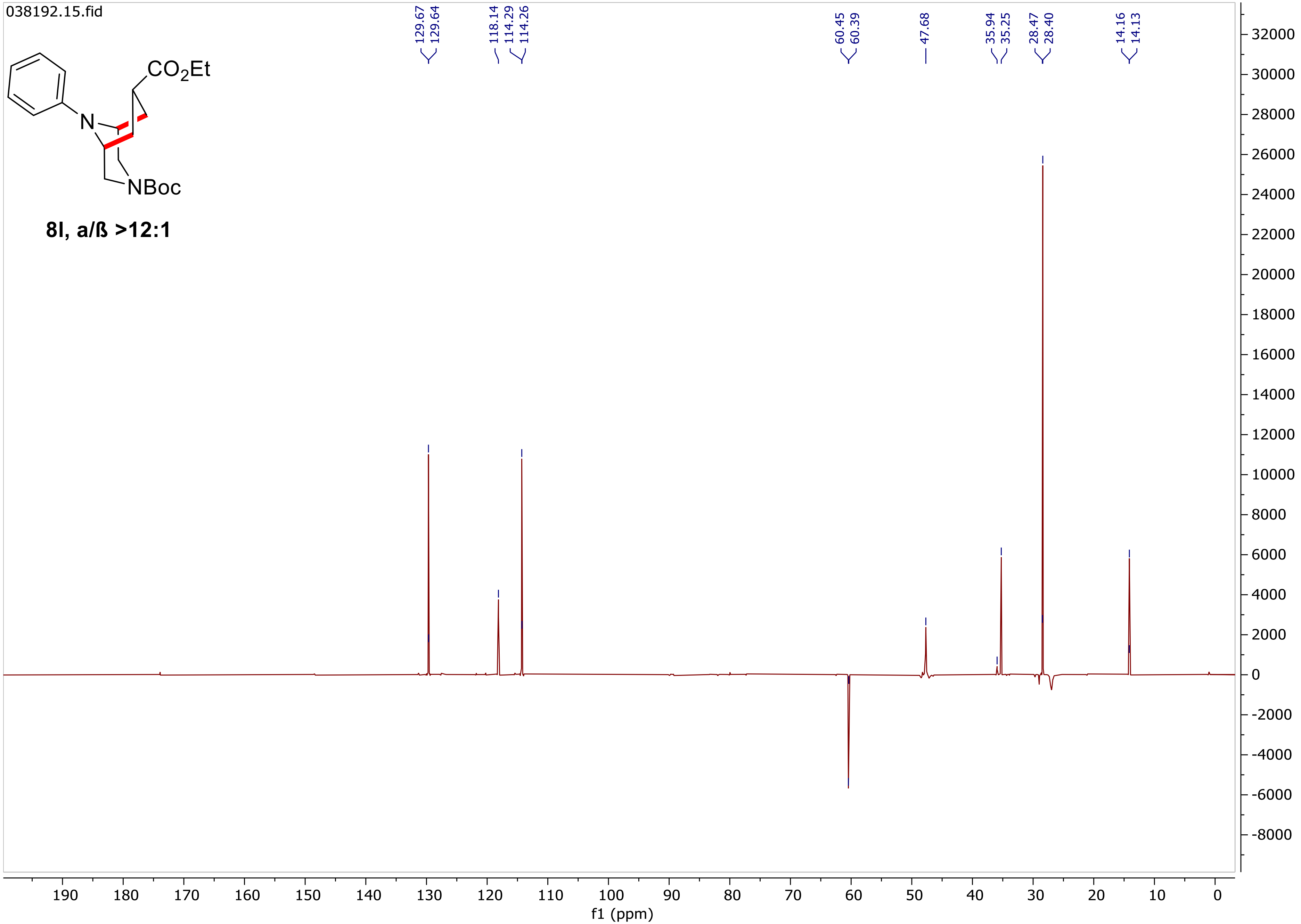

O3-tert-butyl O7-ethyl 9-phenyl-3,9-diazabicyclo[3.3.1]nonane-3,7-dicarboxylate (**8l**) **α/β >12:1**

^{1}H, ^{1}H-COSY NMR (400 MHz, $CDCl_3$)

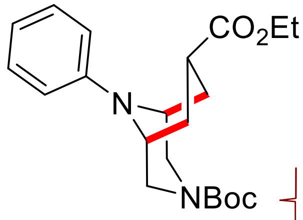

8l, a/ß >12:1

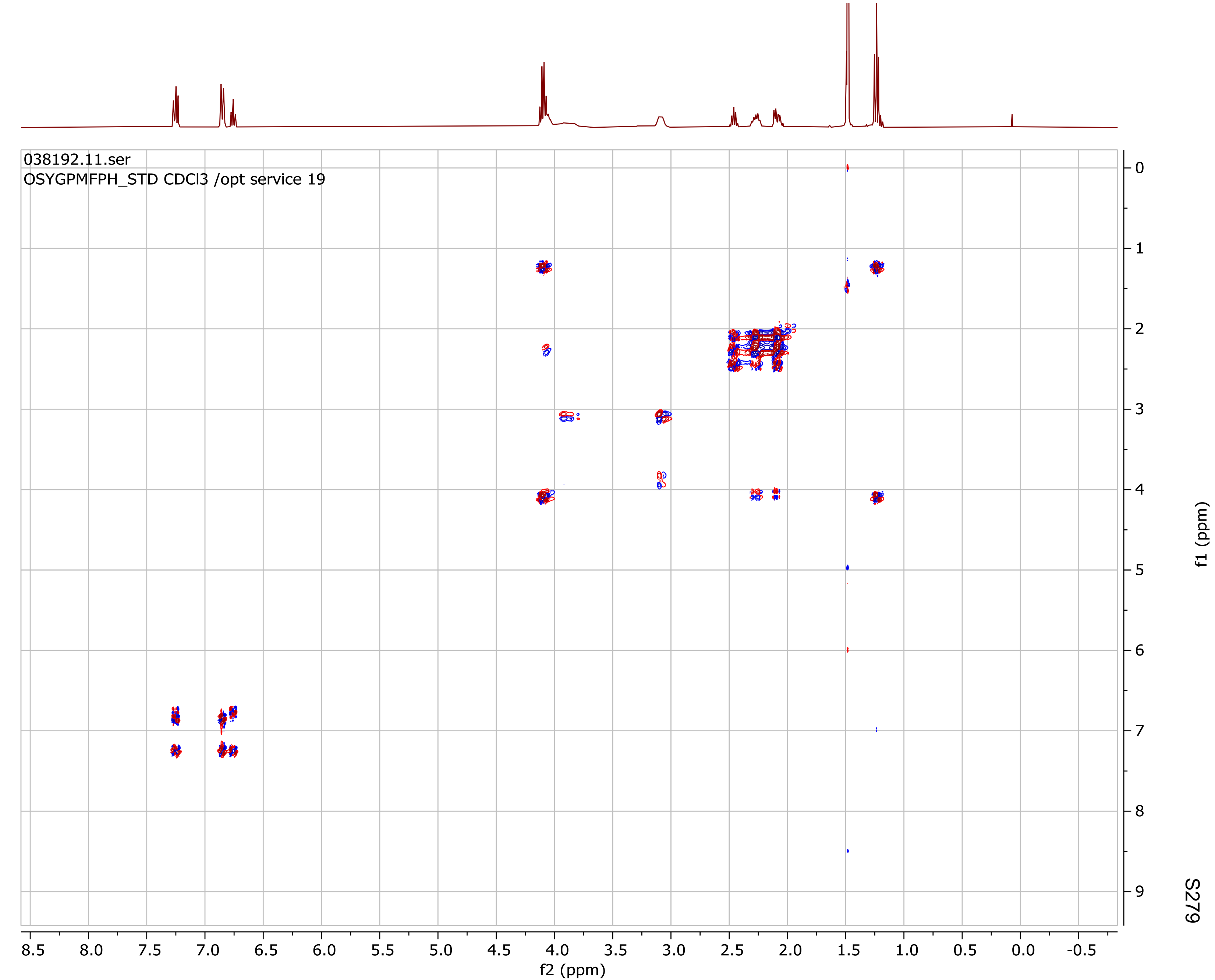

O3-tert-butyl O7-ethyl 9-phenyl-3,9-diazabicyclo[3.3.1]nonane-3,7-dicarboxylate (**8l**) **α/β >12:1**

^1H, ^1H-COSY NMR (400 MHz, $CDCl_3$)

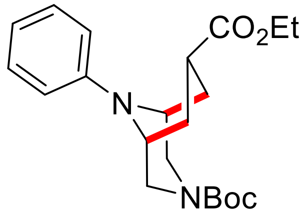

8l, a/ß >12:1

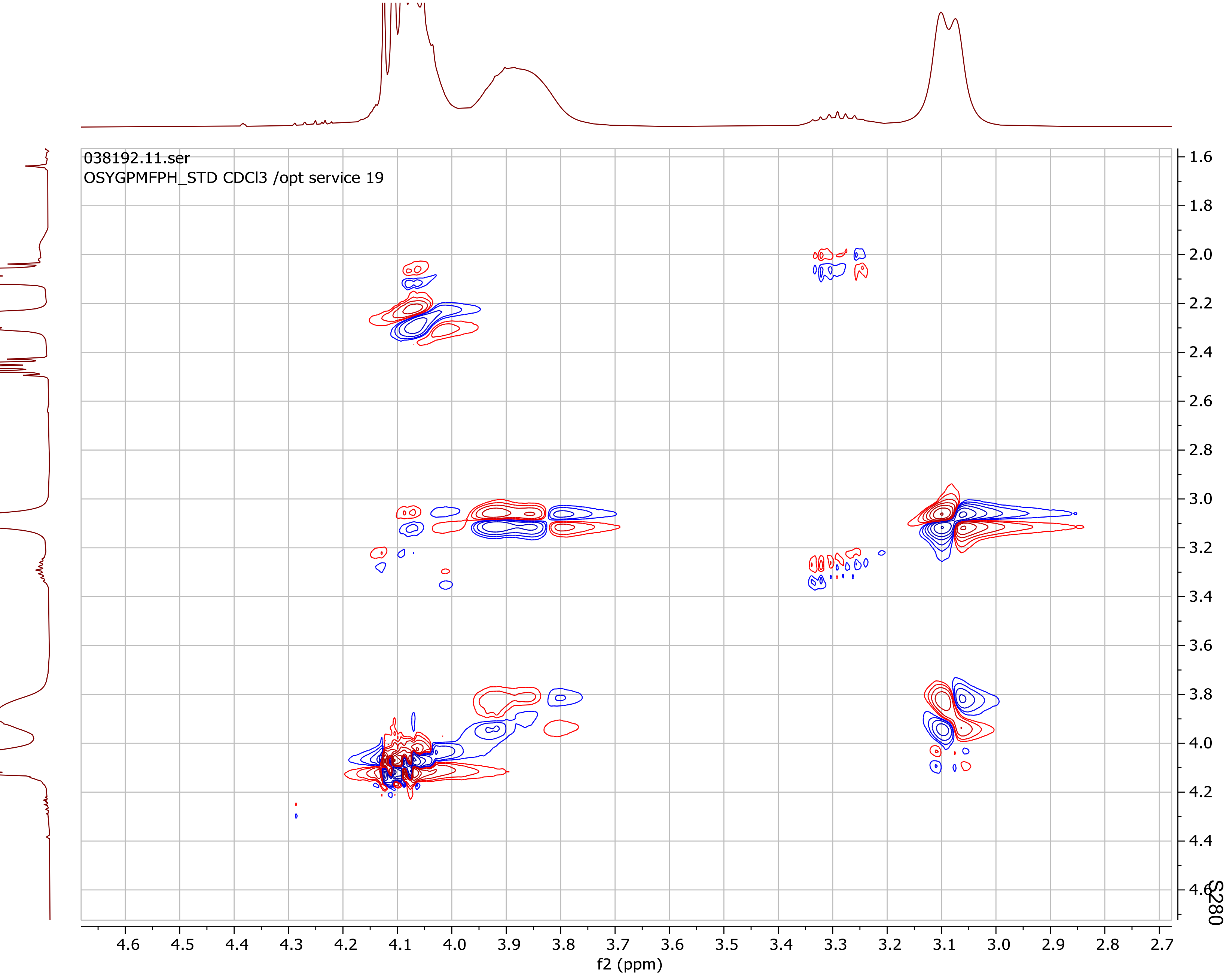

O3-tert-butyl O7-ethyl 9-phenyl-3,9-diazabicyclo[3.3.1]nonane-3,7-dicarboxylate (**8l**) **α/β >12:1**

^{1}H, ^{13}C-HSQC NMR (400 MHz, $CDCl_3$)

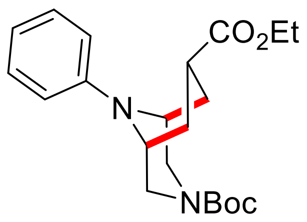

8l, a/ß >12:1

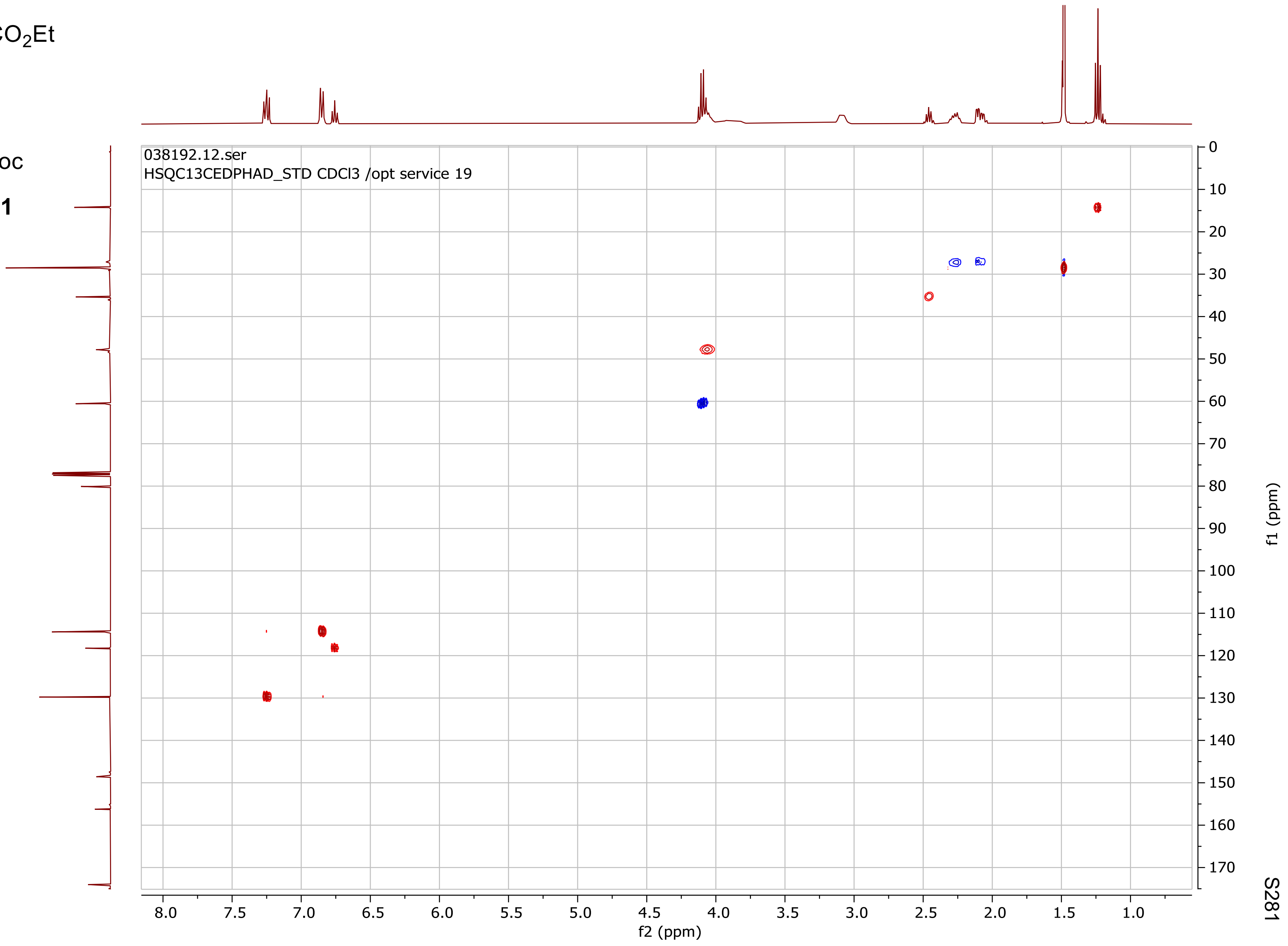

O3-tert-butyl O7-ethyl 9-phenyl-3,9-diazabicyclo[3.3.1]nonane-3,7-dicarboxylate (**8l**) **α/β >12:1**

^{1}H, ^{13}C-HSQC NMR (400 MHz, $CDCl_3$)

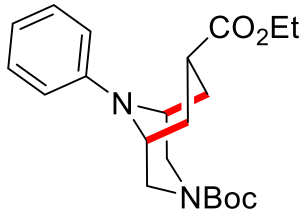

8l, a/ß >12:1

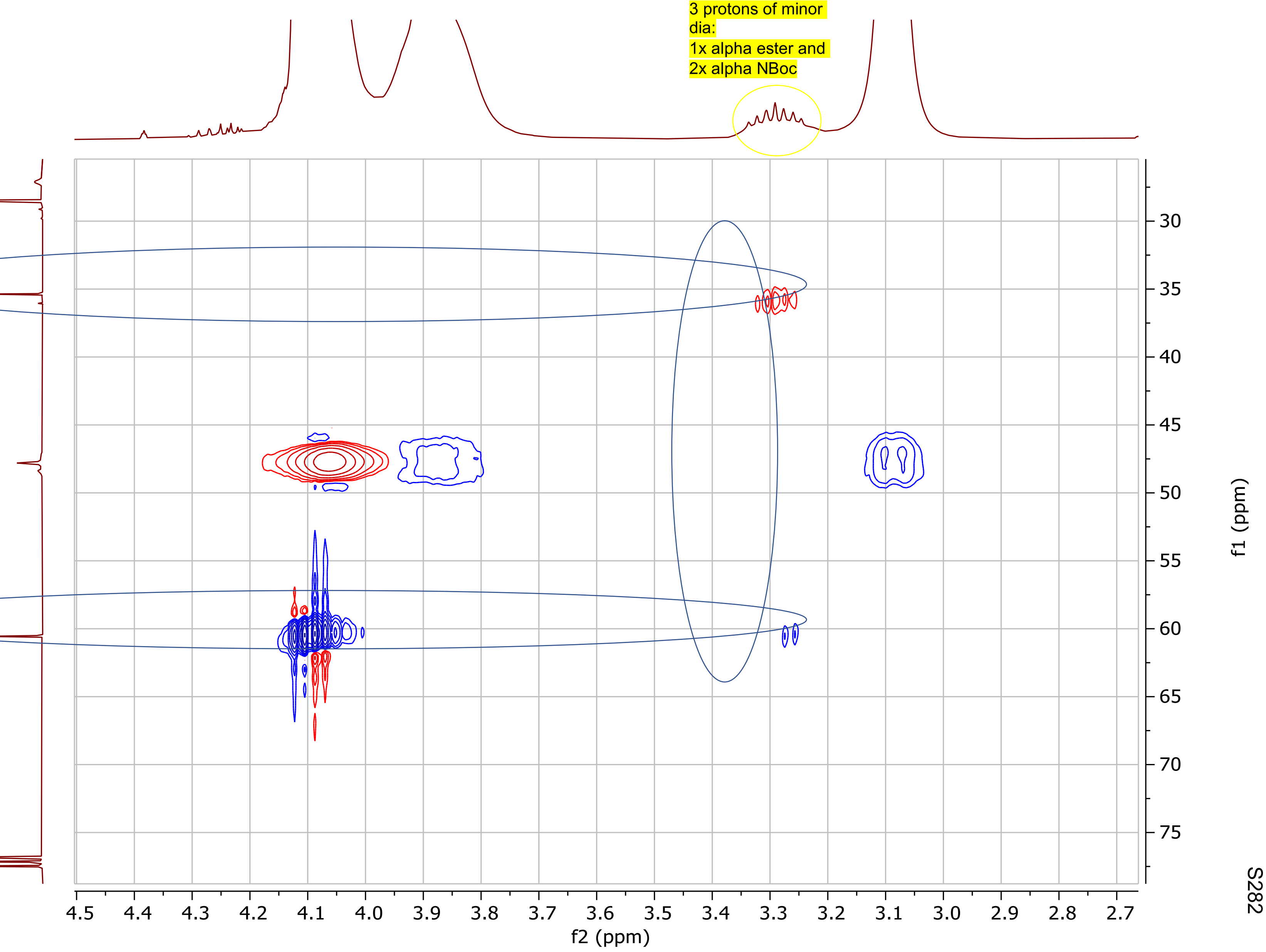

O3-tert-butyl O7-ethyl 9-phenyl-3,9-diazabicyclo[3.3.1]nonane-3,7-dicarboxylate (**8l**) **α/β >12:1**

^{1}H, ^{13}C-HMBC NMR (400 MHz, $CDCl_3$)

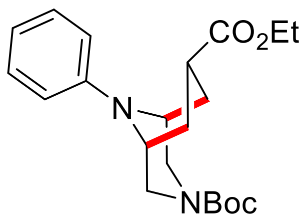

8l, a/ß >12:1

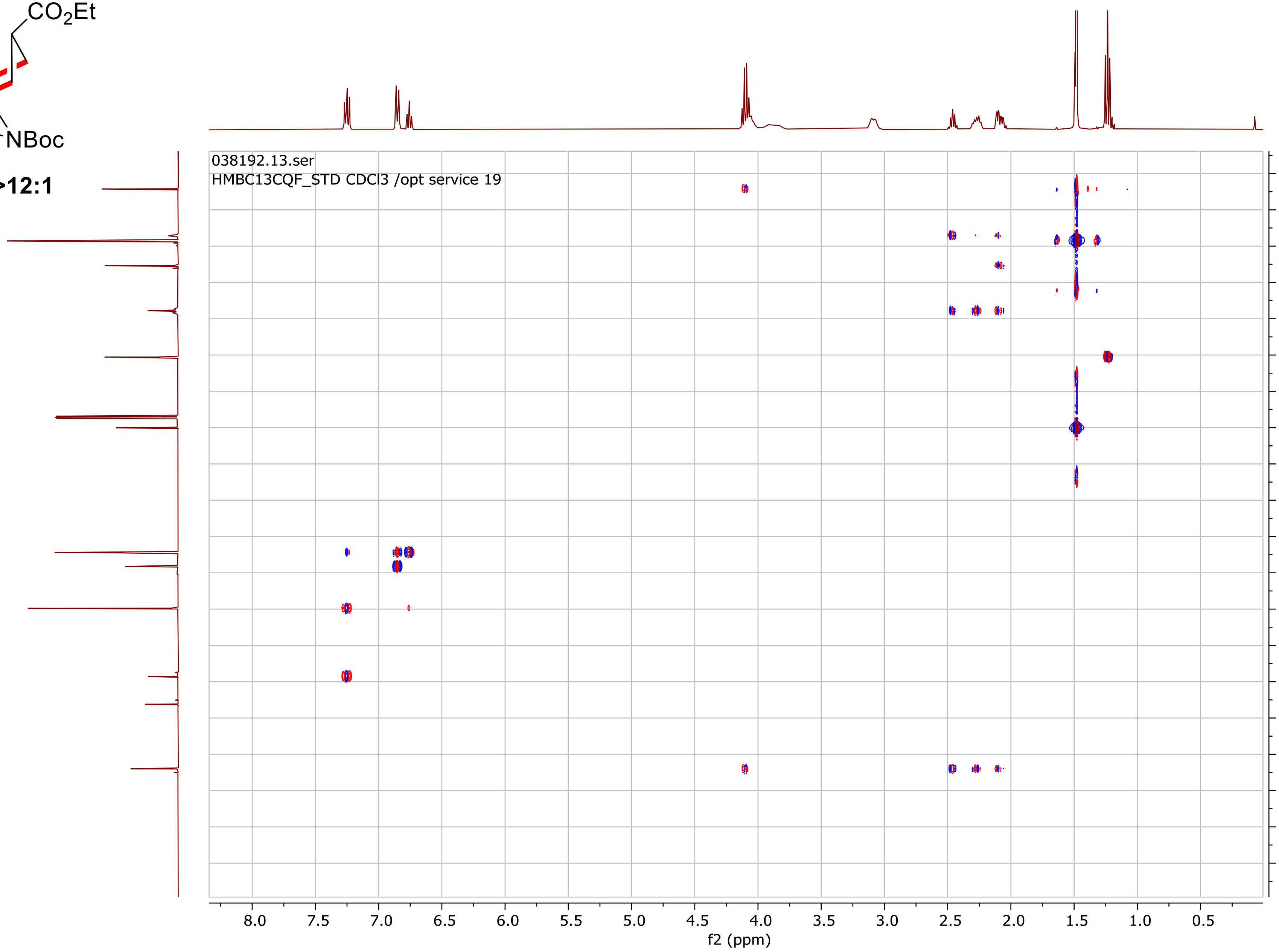

O3-tert-butyl O7-ethyl 9-phenyl-3,9-diazabicyclo[3.3.1]nonane-3,7-dicarboxylate (**8l**) **α/β >12:1** FT-IR, ATR-diamond

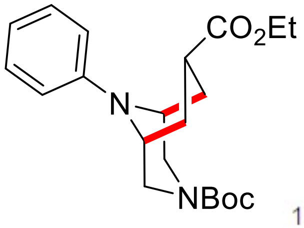

8l, a/ß >12:1

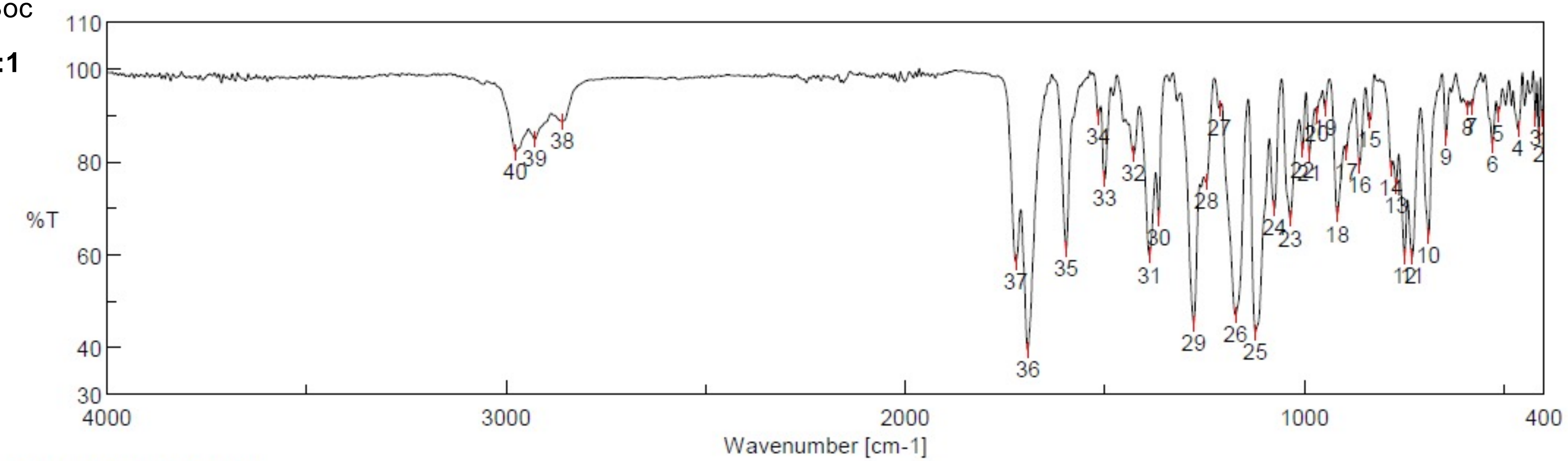

[Result of Peak Picking]

No.	Position	Intensity	No.	Position	Intensity	No.	Position	Intensity
1	404.014	89	2	412.692	86	3	421.37	89
4	464.761	87	5	514.901	90	6	529.364	84
7	581.433	92	8	591.075	92	9	645.072	85
10	689.427	64	11	729.925	60	12	749.209	60
13	770.423	75	14	782.958	79	15	837.919	89
16	862.025	79	17	895.773	82	18	917.95	69
19	948.806	91	20	969.055	90	21	987.375	82
22	1004.73	83	23	1035.59	68	24	1075.12	70
25	1123.33	43	26	1172.51	47	27	1213.01	91
28	1245.79	76	29	1277.61	45	30	1365.35	68
31	1388.5	60	32	1428.03	82	33	1500.35	76
34	1516.74	90	35	1596.77	61	36	1693.19	39
37	1723.09	58	38	2859.92	89	39	2928.38	85
40	2976.59	82						

Ethyl 9-phenyl-3-thia-9-azabicyclo[3.3.1]nonane-7-carboxylate (**8m**) **α/β 7:1**

^{1}H-NMR (400 MHz, $CDCl_3$)

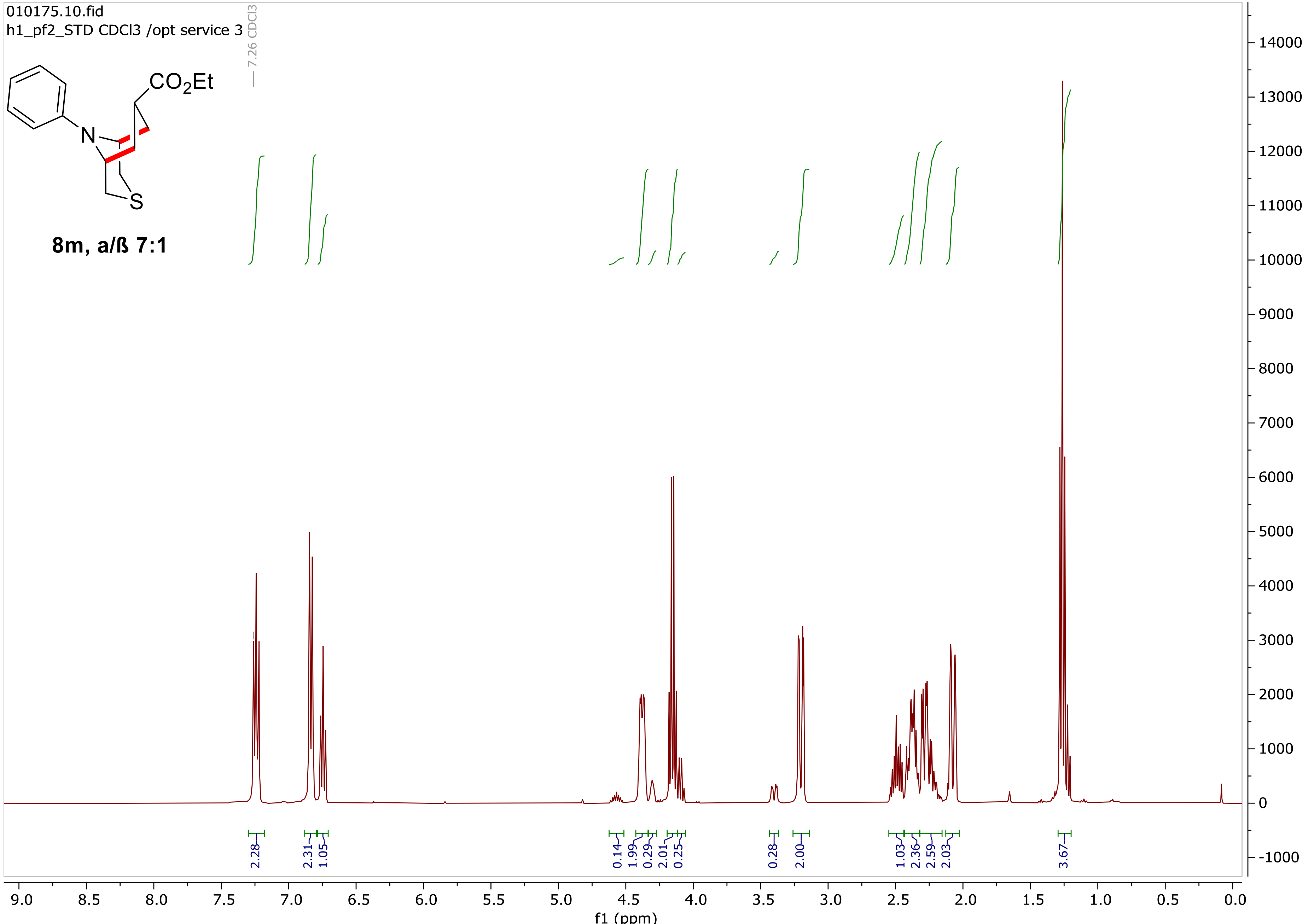

Ethyl 9-phenyl-3-thia-9-azabicyclo[3.3.1]nonane-7-carboxylate (**8m**) **α/β 7:1**

^{1}H-NMR (400 MHz, $CDCl_3$)

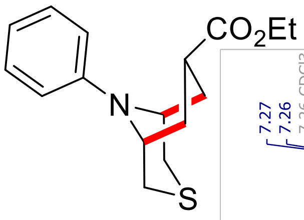

8m, a/ß 7:1

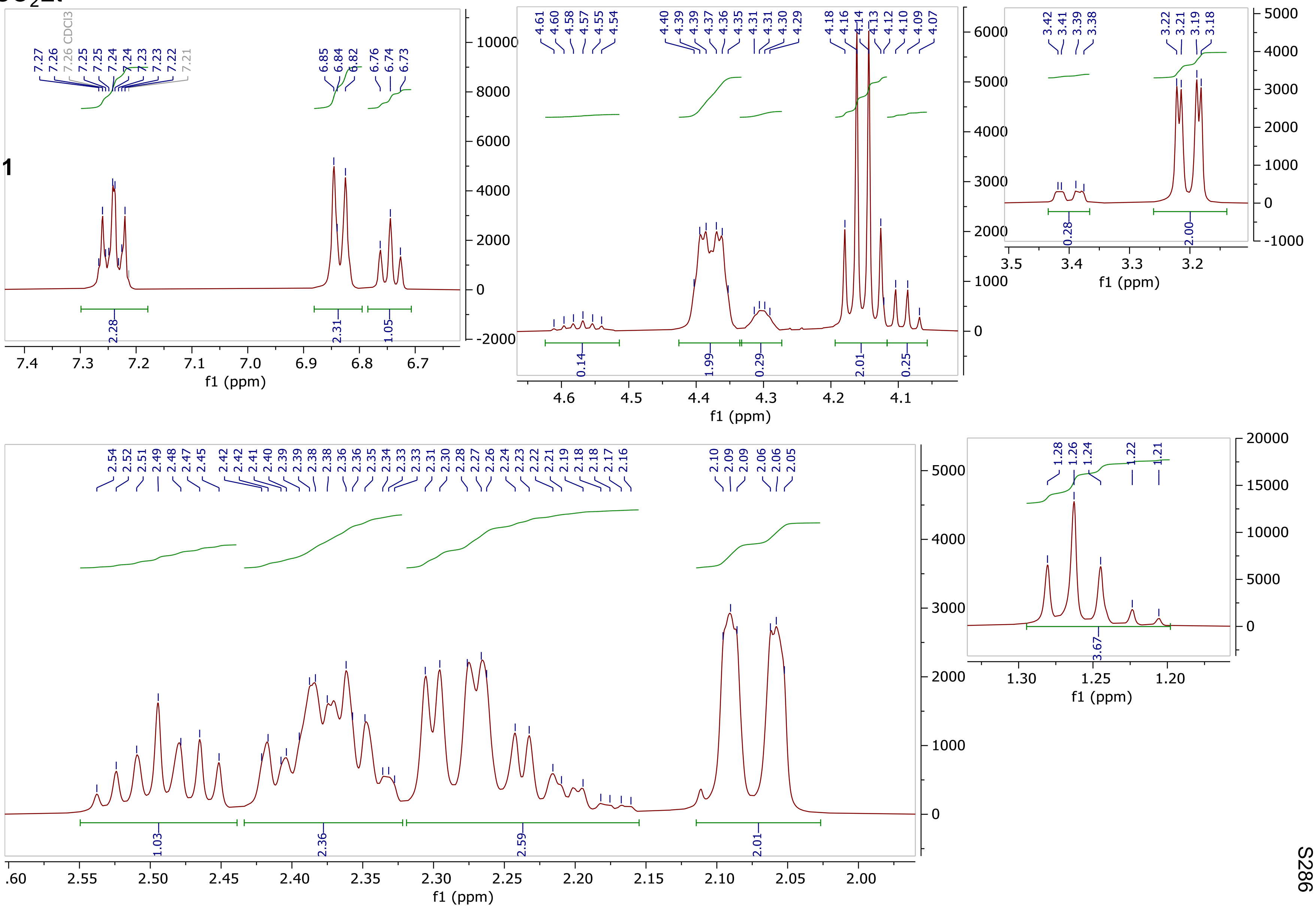

Ethyl 9-phenyl-3-thia-9-azabicyclo[3.3.1]nonane-7-carboxylate (**8m**) **α/β 7:1**

^{13}C-NMR (101 MHz, $CDCl_3$)

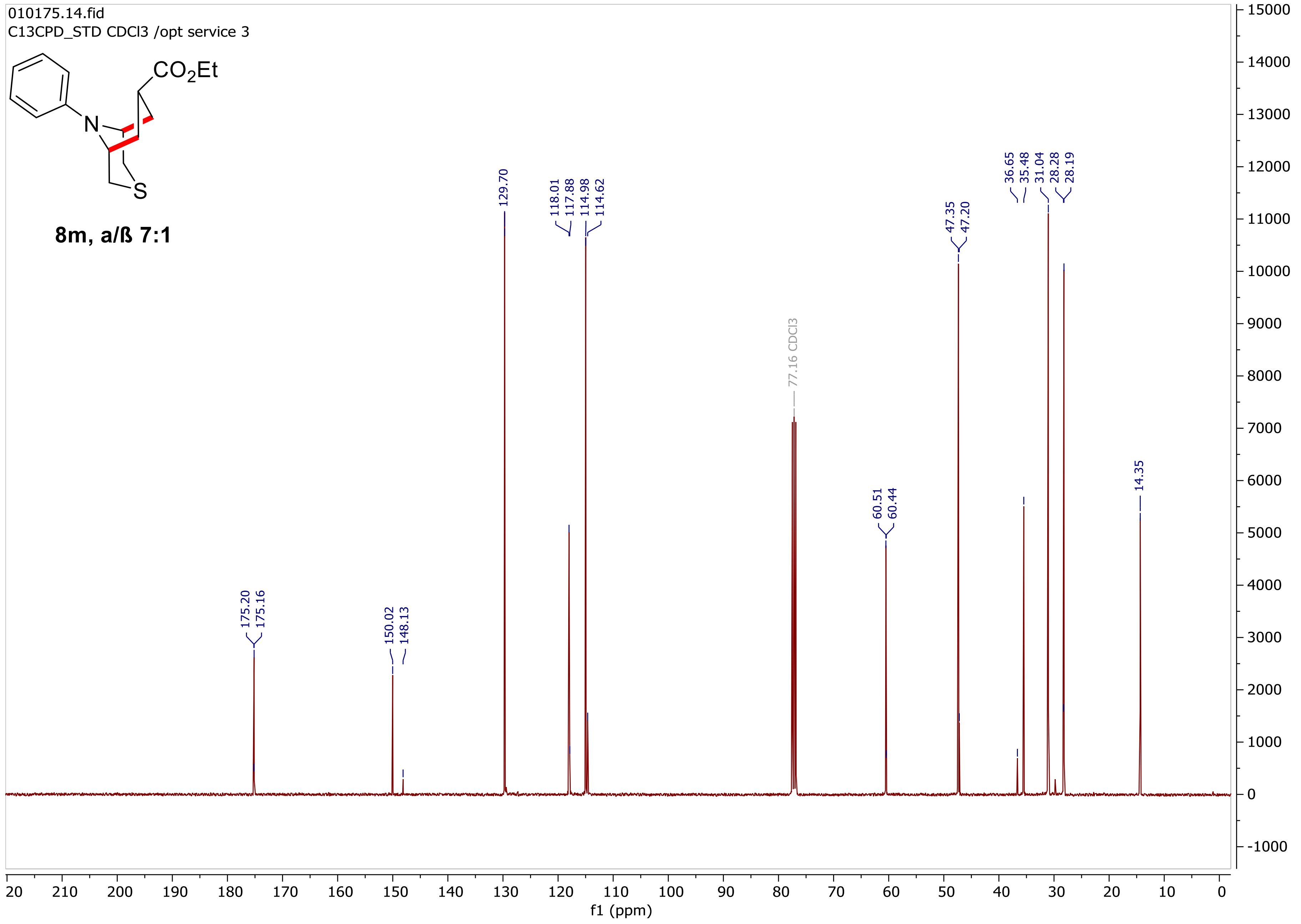

Ethyl 9-phenyl-3-thia-9-azabicyclo[3.3.1]nonane-7-carboxylate (**8m**) **α/β 7:1**

^{13}C-NMR (101 MHz, $CDCl_3$)

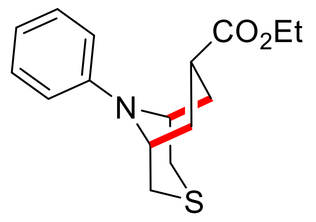

8m, a/ß 7:1

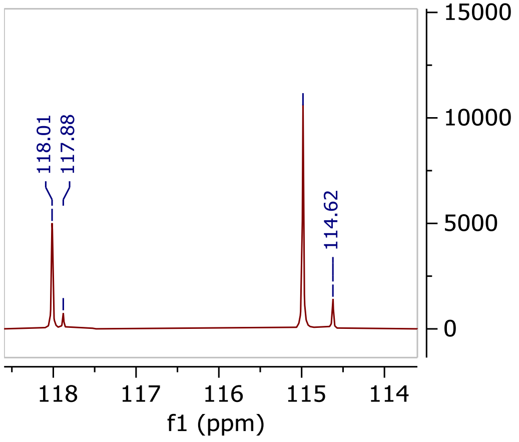

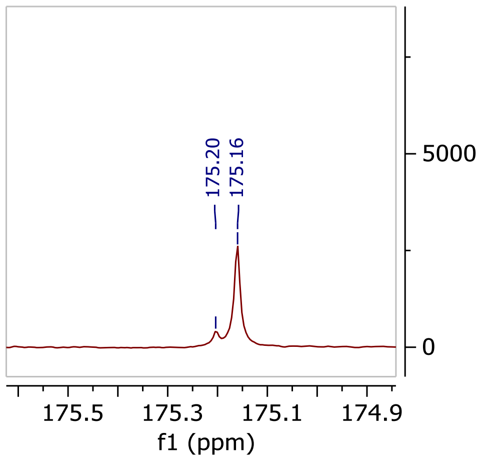

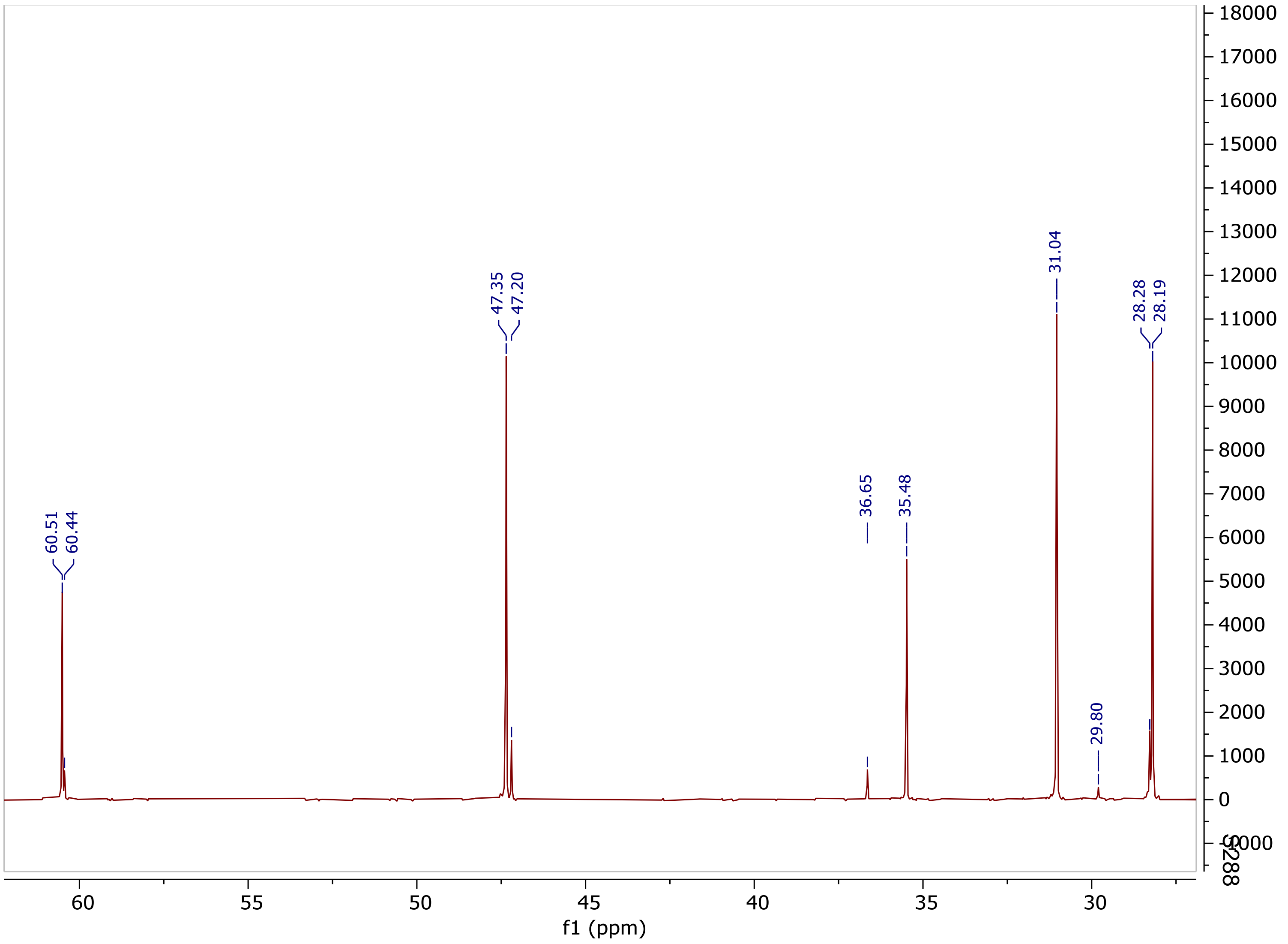

Ethyl 9-phenyl-3-thia-9-azabicyclo[3.3.1]nonane-7-carboxylate (**8m**) **α/β 7:1**

^{13}C-NMR (101 MHz, $CDCl_3$)

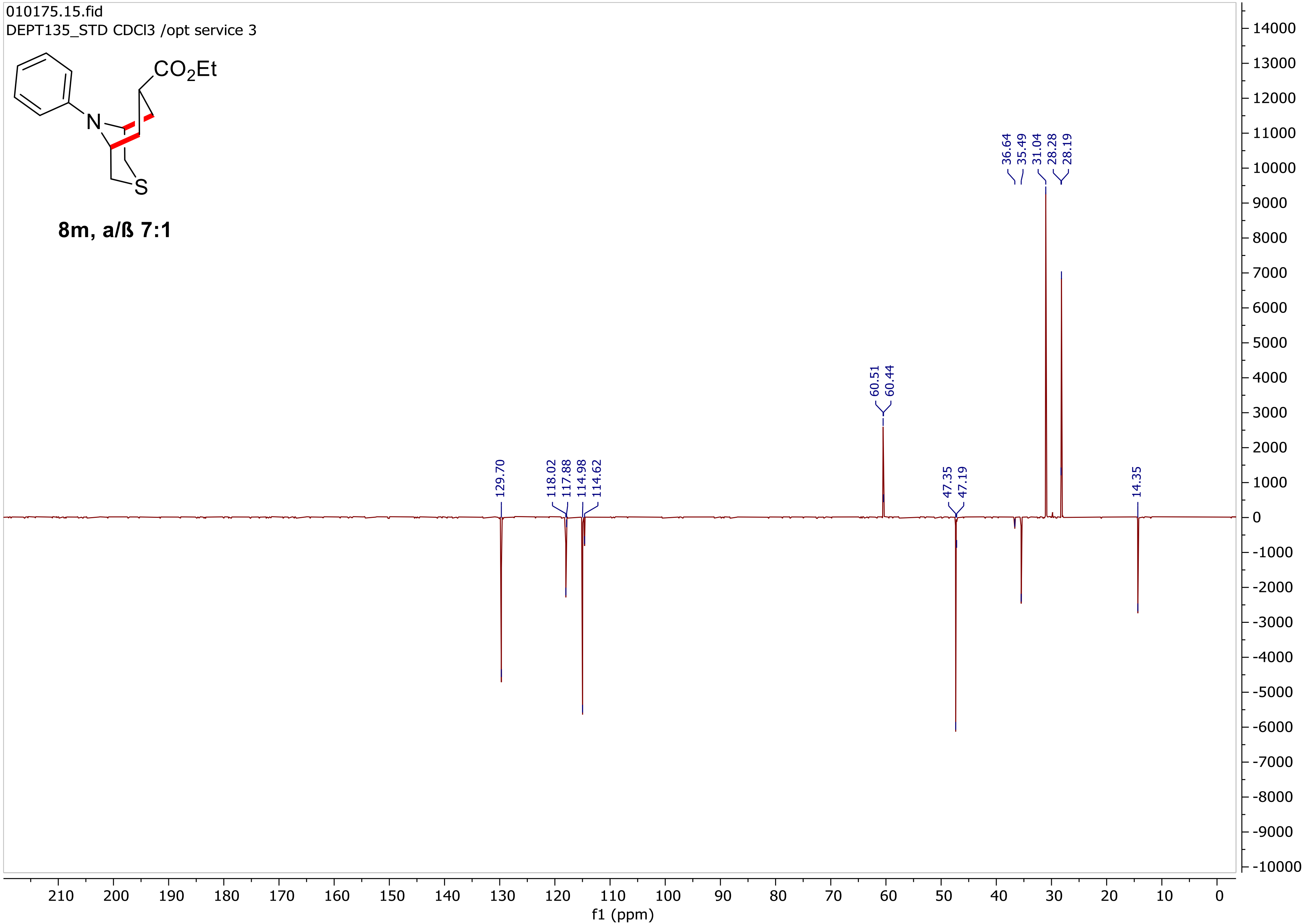

Ethyl 9-phenyl-3-thia-9-azabicyclo[3.3.1]nonane-7-carboxylate (**8m**) **α/β 7:1**

^1H, ^1H-COSY NMR (400 MHz, $CDCl_3$)

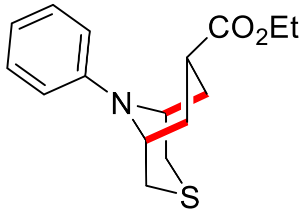

8m, a/ß 7:1

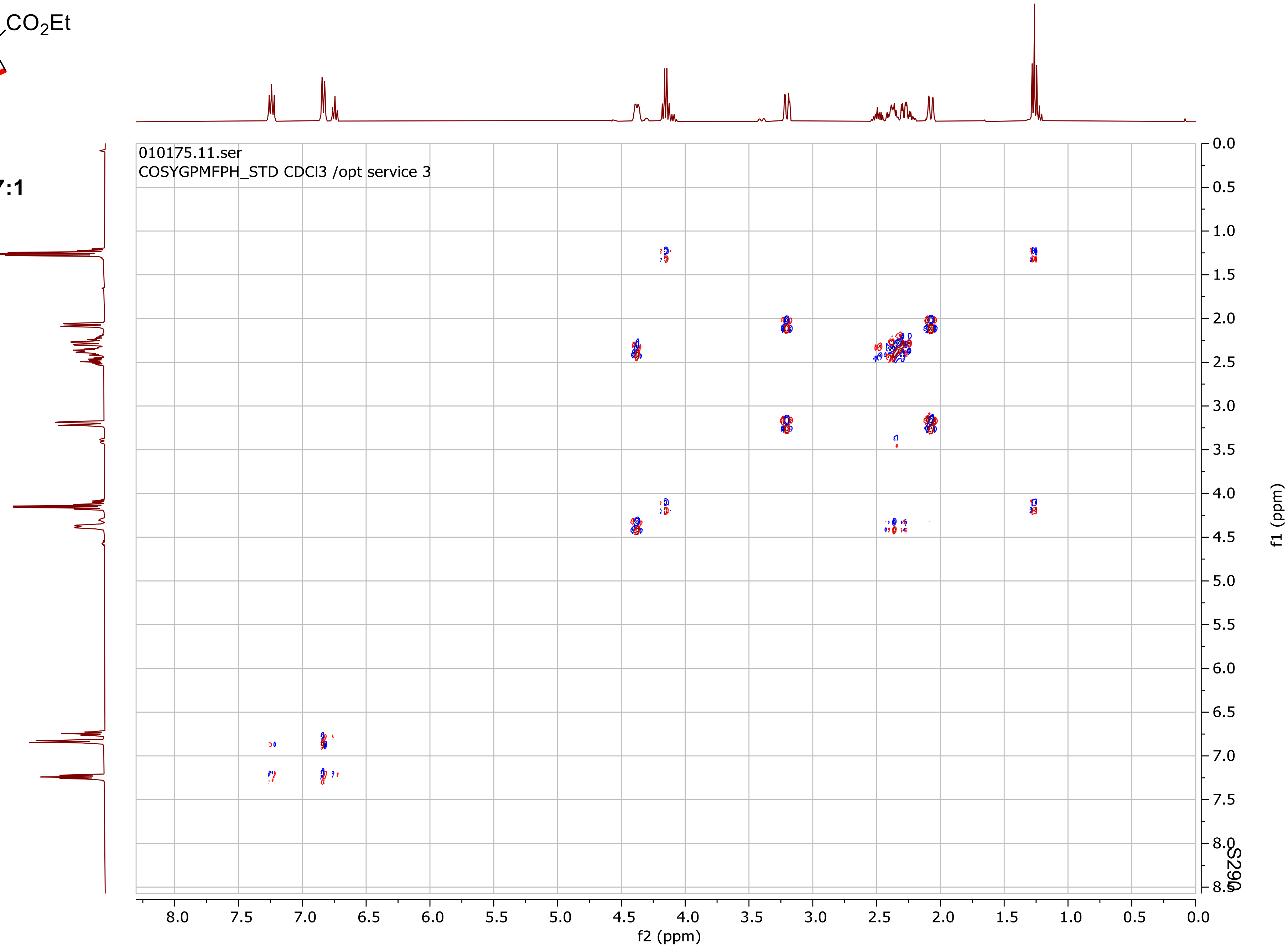

Ethyl 9-phenyl-3-thia-9-azabicyclo[3.3.1]nonane-7-carboxylate (**8m**) **α/β 7:1**

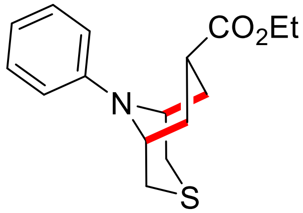

8m, a/ß 7:1

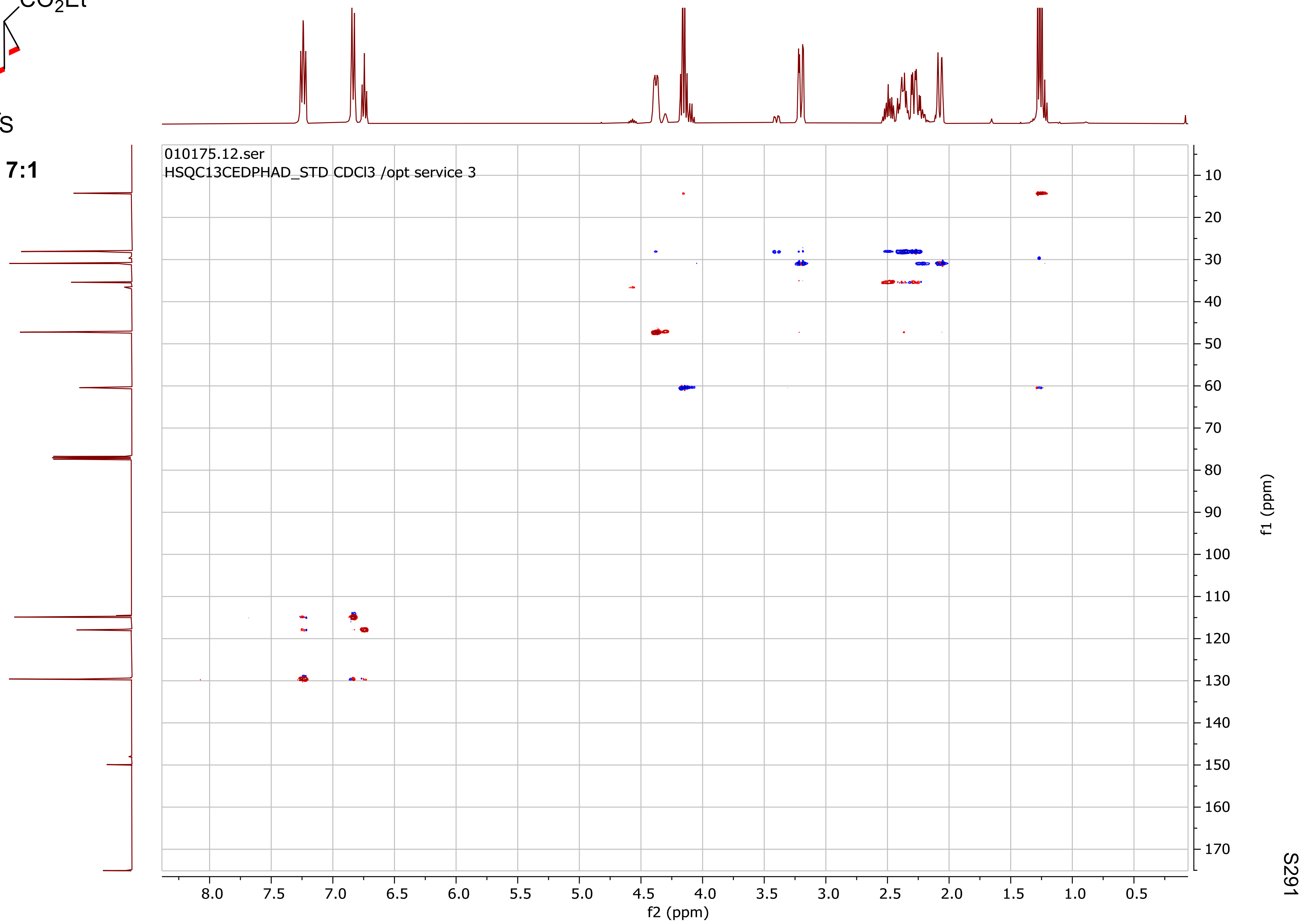

Ethyl 9-phenyl-3-thia-9-azabicyclo[3.3.1]nonane-7-carboxylate (**8m**) **α/β 7:1**

^{1}H, ^{13}C-HMBC NMR (400 MHz, $CDCl_3$)

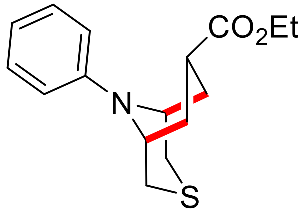

8m, a/ß 7:1

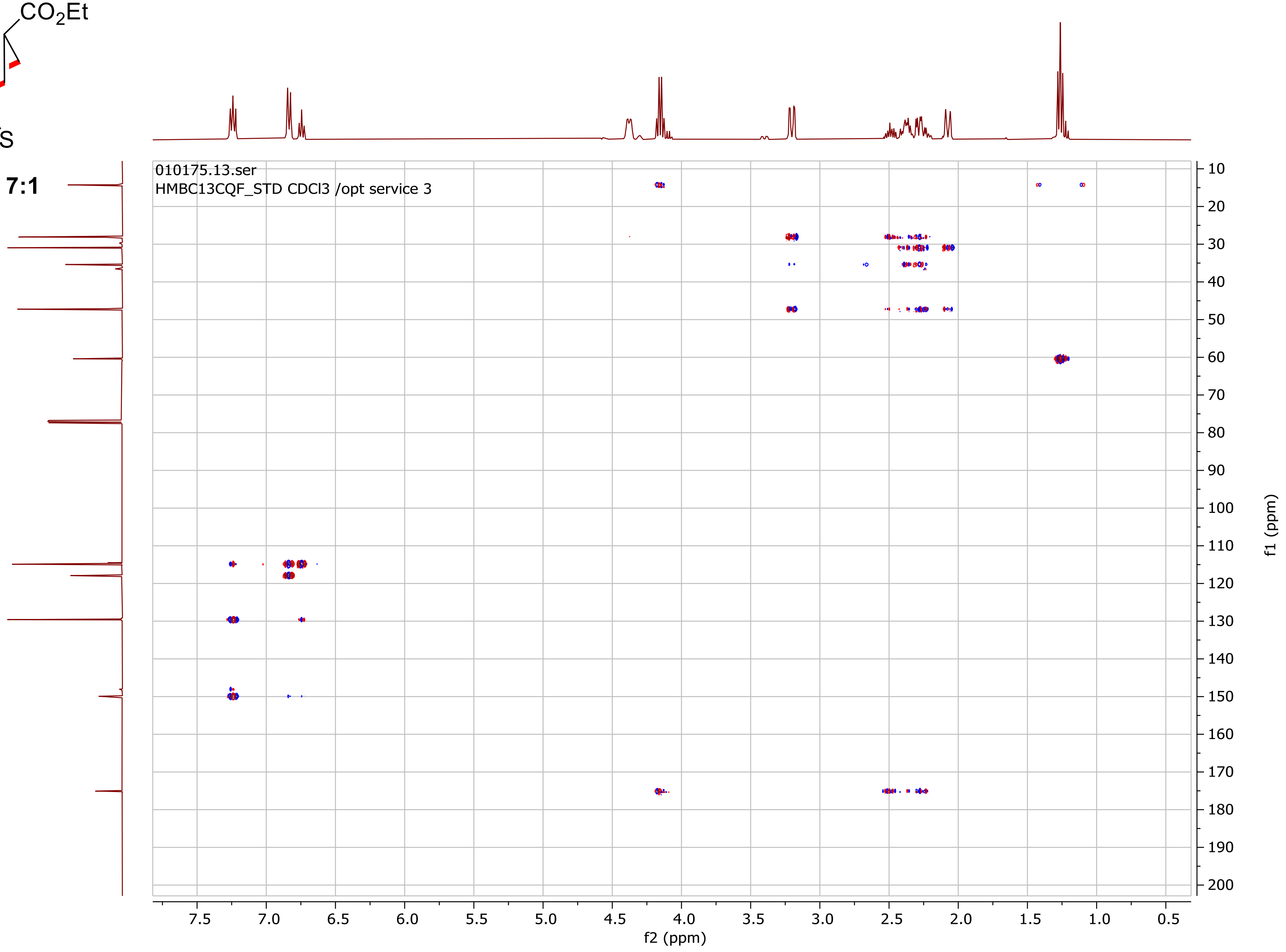

Ethyl 9-phenyl-3-thia-9-azabicyclo[3.3.1]nonane-7-carboxylate (**8m**) **α/β 7:1**

FT-IR, ATR-diamond

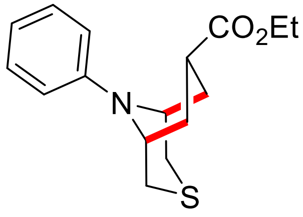

8m, a/ß 7:1

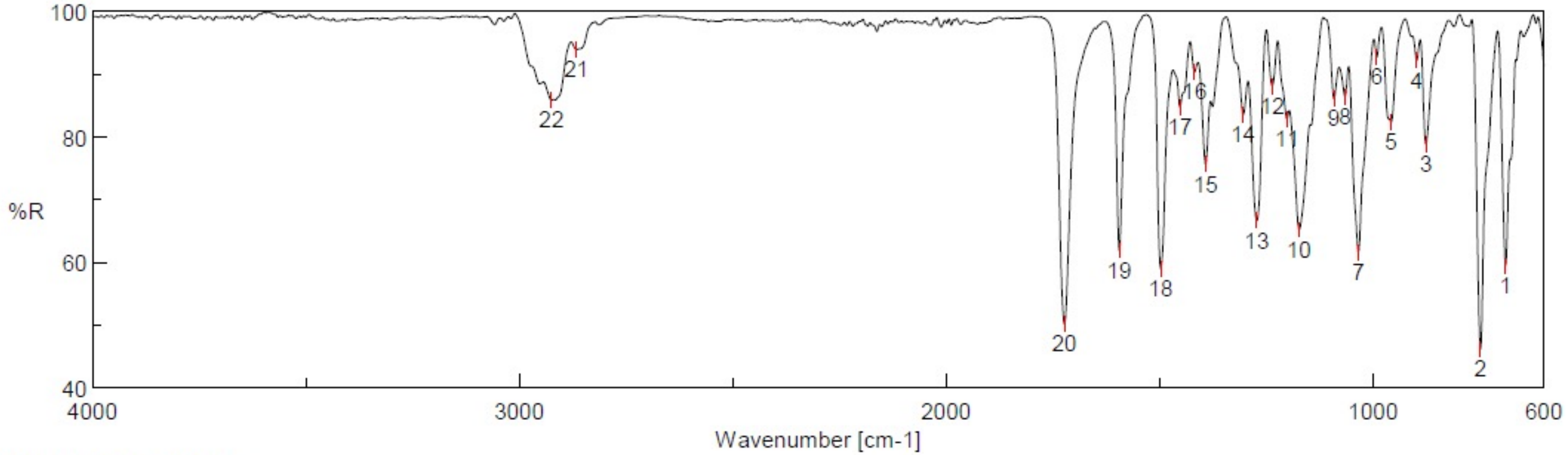

[Result of Peak Picking]

No.	Position	Intensity	No.	Position	Intensity	No.	Position	Intensity
1	689.427	59.479	2	748.245	46.1435	3	876.488	78.8304
4	897.701	92.1608	5	957.484	82.3346	6	991.232	92.7248
7	1034.62	61.5656	8	1065.48	86.4365	9	1091.51	86.0569
10	1173.47	65.1875	11	1201.43	82.895	12	1236.15	88.1142
13	1272.79	66.6104	14	1303.64	83.5607	15	1392.35	75.6035
16	1418.39	90.3471	17	1452.14	84.6992	18	1497.45	59.0196
19	1594.84	61.8829	20	1723.09	50.0923	21	2866.67	93.9088
22	2924.52	85.8312						

Ethyl 10-phenyl-10-azabicyclo[4.3.1]decane-8-carboxylate (**8n**) **α/β >20:1**

^{1}H-NMR (300 MHz, $CDCl_3$)

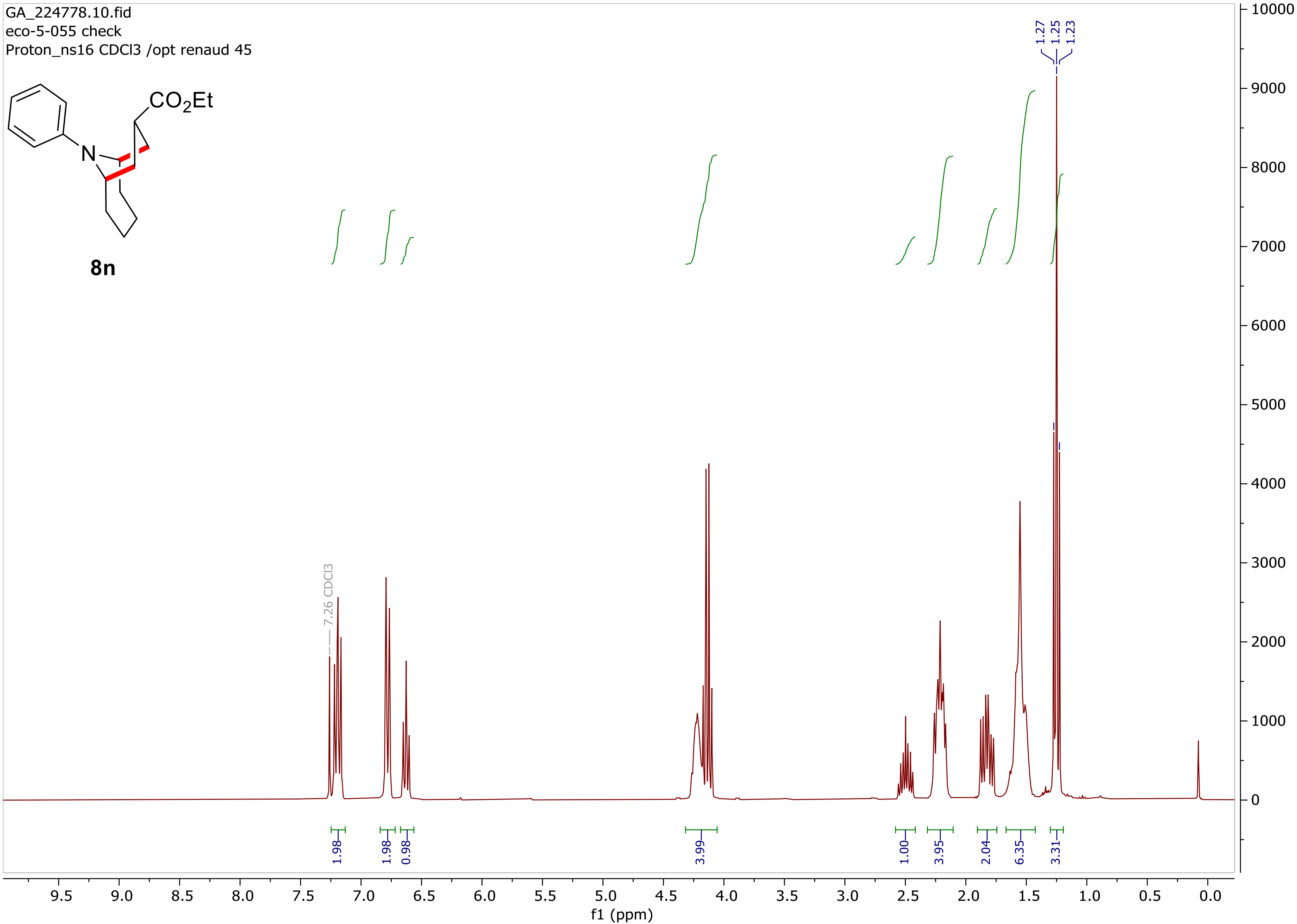

Ethyl 10-phenyl-10-azabicyclo[4.3.1]decane-8-carboxylate (**8nα**)

^{1}H-NMR (300 MHz, $CDCl_3$)

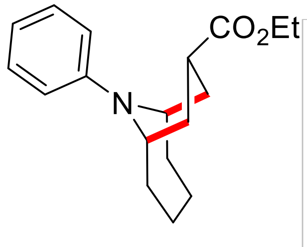

8n

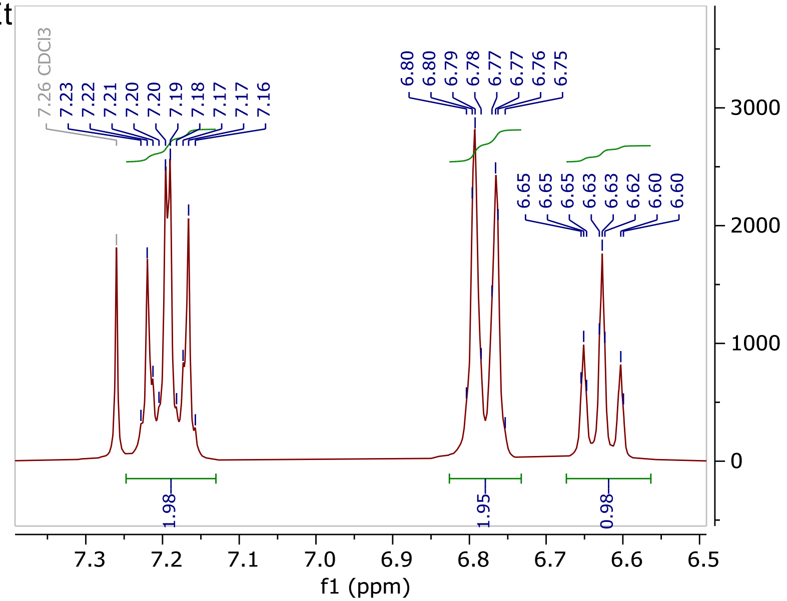

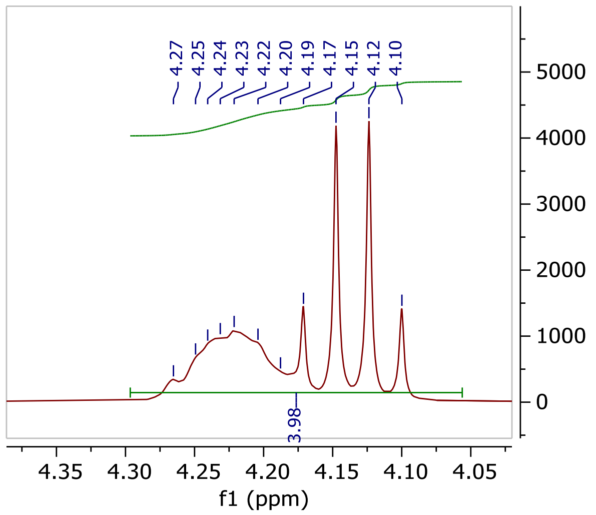

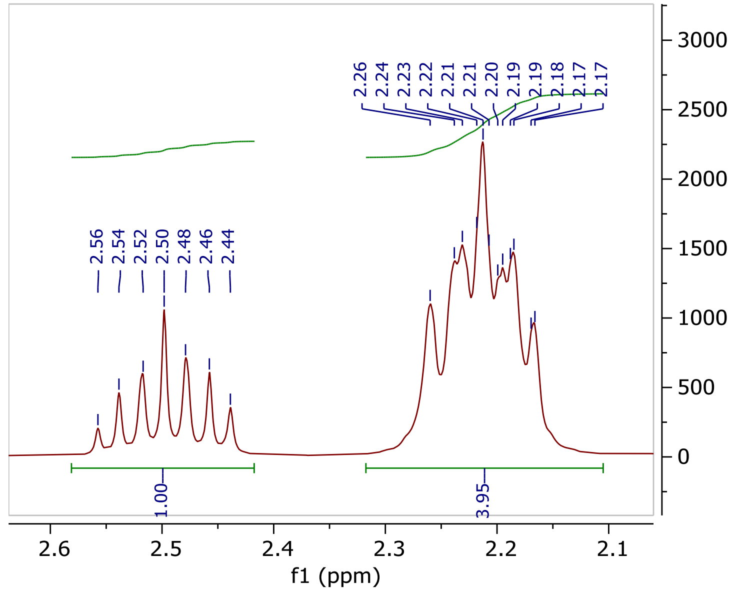

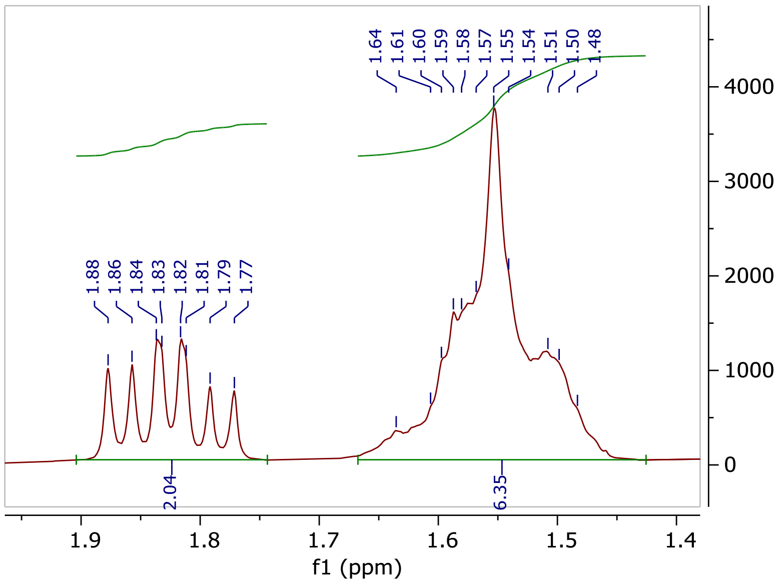

Ethyl 10-phenyl-10-azabicyclo[4.3.1]decane-8-carboxylate (**8nα**)

^{13}C-NMR (101 MHz, $CDCl_3$)

GA_224518.10.fid
ECO-5-055 F2
Carbon_ns512 CDCl3 /opt renaud 28

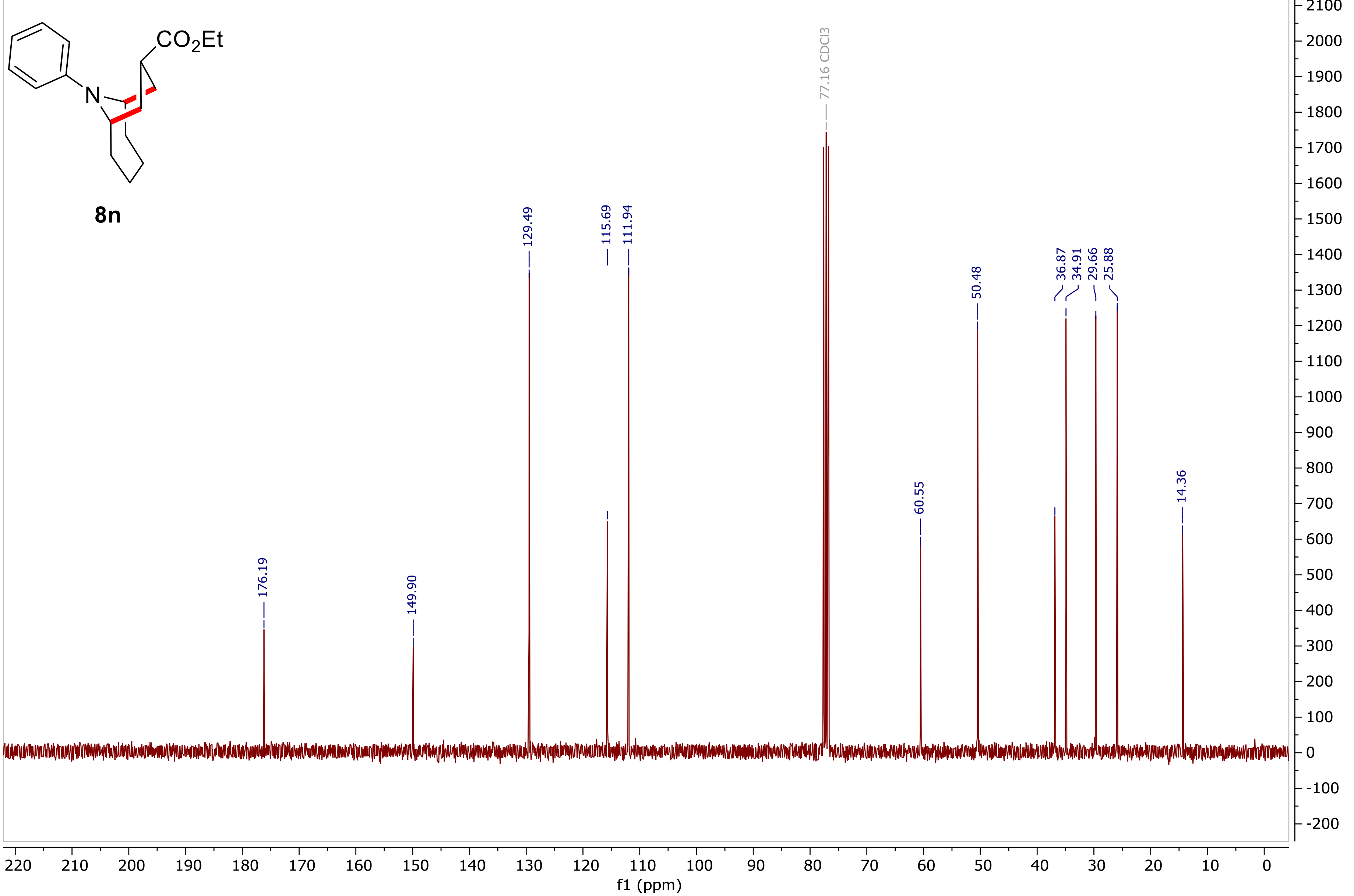

8n

Ethyl 10-phenyl-10-azabicyclo[4.3.1]decane-8-carboxylate (**8nα**)

^{13}C-NMR (101 MHz, $CDCl_3$)

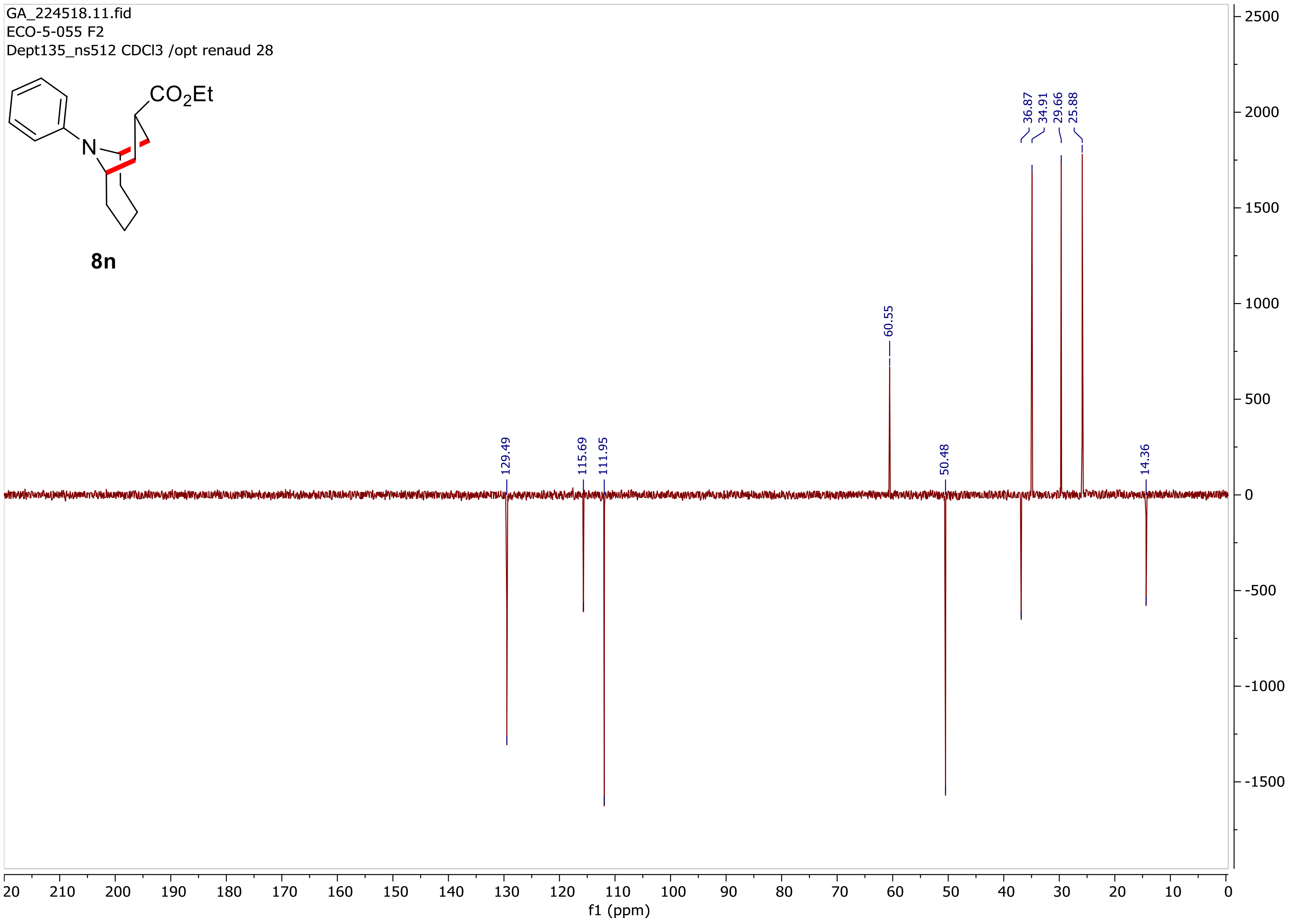

Ethyl 10-phenyl-10-azabicyclo[4.3.1]decane-8-carboxylate (**8nα**)

^1H, ^1H-COSY NMR (300 MHz, $CDCl_3$)

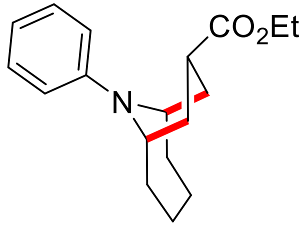

8n

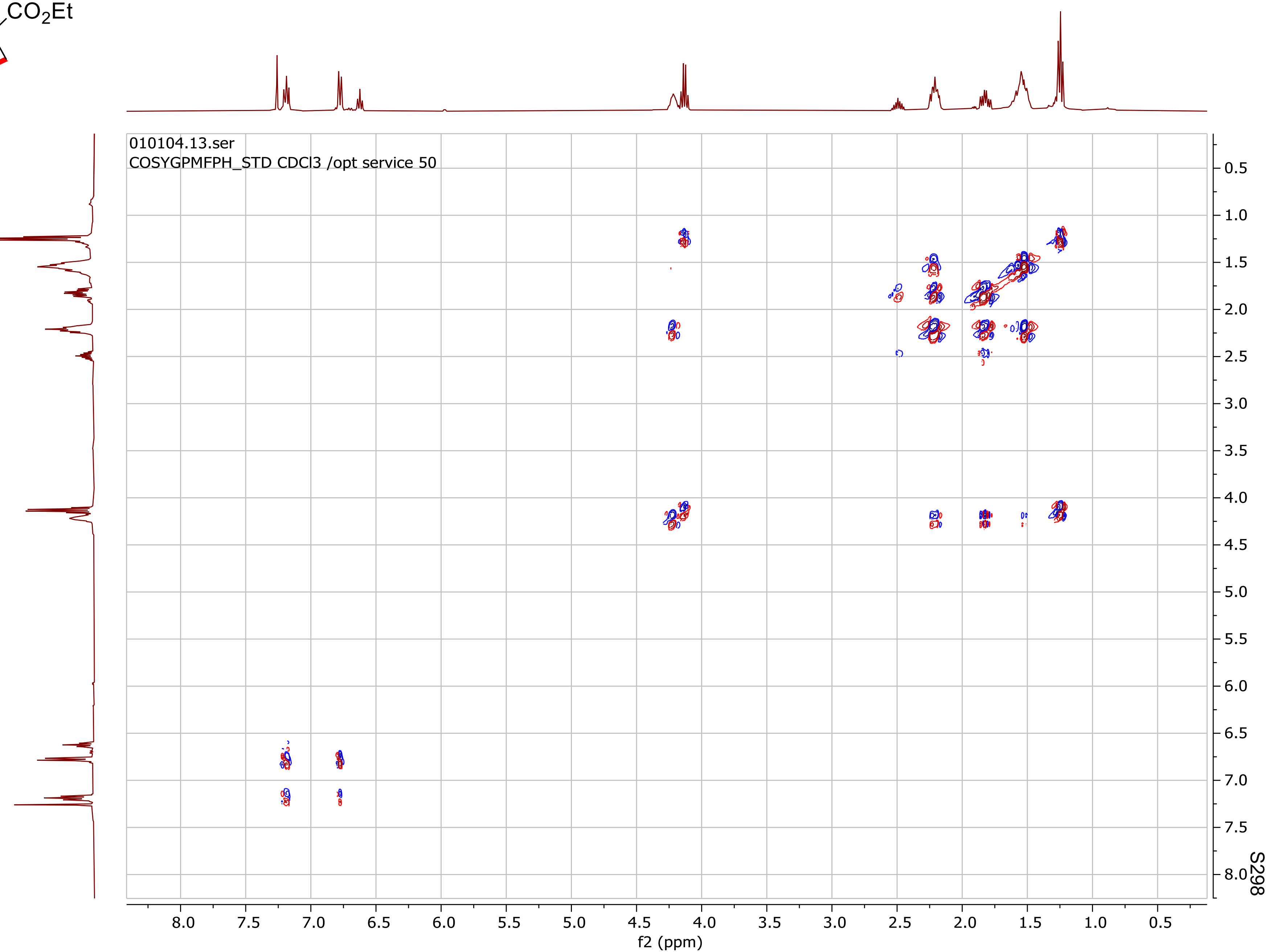

Ethyl 10-phenyl-10-azabicyclo[4.3.1]decane-8-carboxylate (**8nα**)

^{1}H, ^{13}C-HSQC NMR (300 MHz, $CDCl_3$)

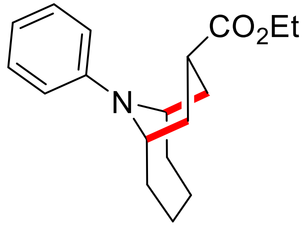

8n

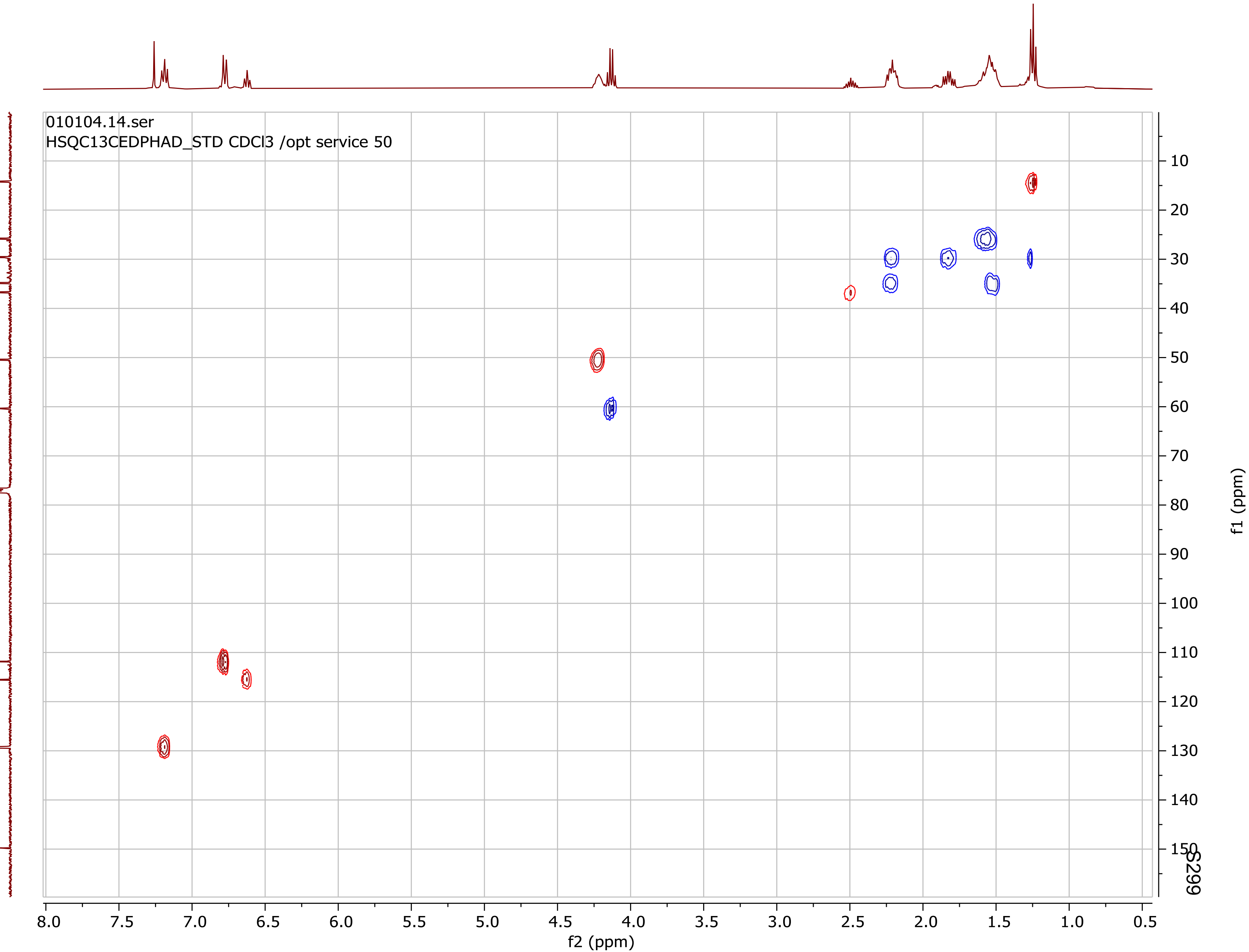

Ethyl 10-phenyl-10-azabicyclo[4.3.1]decane-8-carboxylate (**8nα**)

^{1}H, ^{13}C-HMBC NMR (300 MHz, $CDCl_3$)

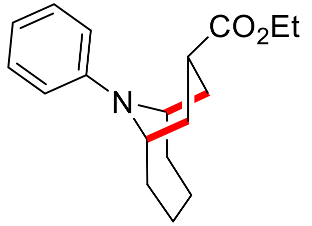

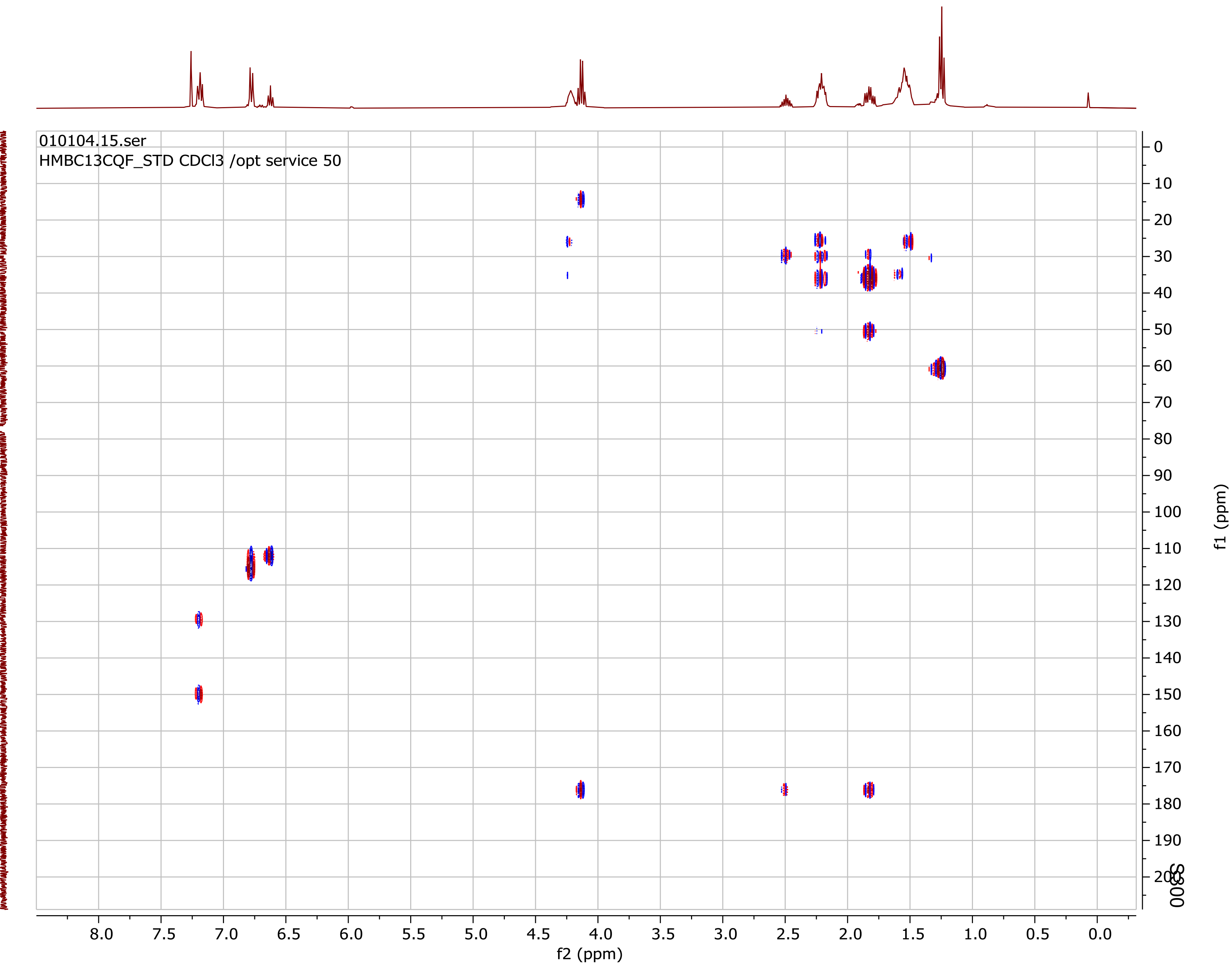

Ethyl 10-phenyl-10-azabicyclo[4.3.1]decane-8-carboxylate (**8nα**)

FT-IR, ATR-diamond

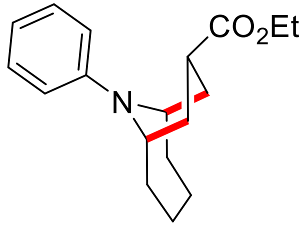

8n

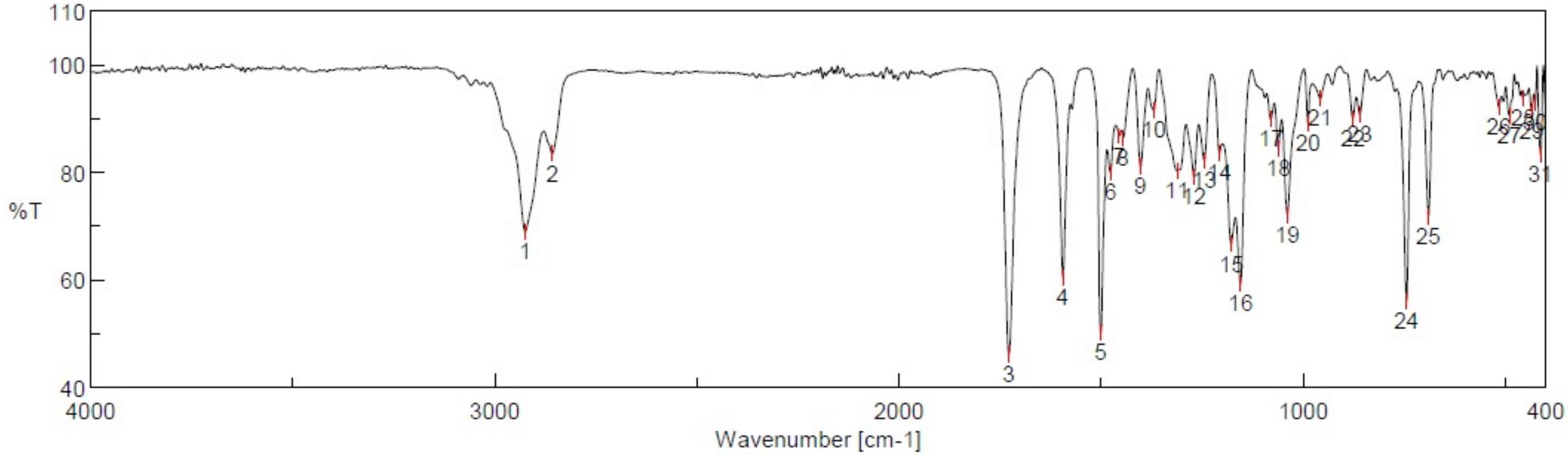

[Result of Peak Picking]

No.	Position	Intensity	No.	Position	Intensity	No.	Position	Intensity
1	2924.52	69	2	2857.99	83	3	1728.87	46
4	1594.84	61	5	1501.31	50	6	1477.21	80
7	1455.99	87	8	1445.39	86	9	1402.96	81
10	1370.18	92	11	1312.32	80	12	1270.86	79
13	1244.83	82	14	1207.22	84	15	1178.29	67
16	1155.15	59	17	1079.94	90	18	1061.62	85
19	1039.44	72	20	988.339	89	21	958.448	94
22	877.452	90	23	860.096	91	24	744.388	56
25	690.391	72	26	515.865	92	27	490.795	91
28	456.082	94	29	434.869	91	30	426.191	93
31	412.692	83						

Ethyl 9-(4-methoxycarbonylphenyl)-2-methyl-9-azabicyclo[3.3.1]nonane-3-carboxylate (**10**) **dr >20:1** ^{1}H-NMR (400 MHz, $CDCl_3$)

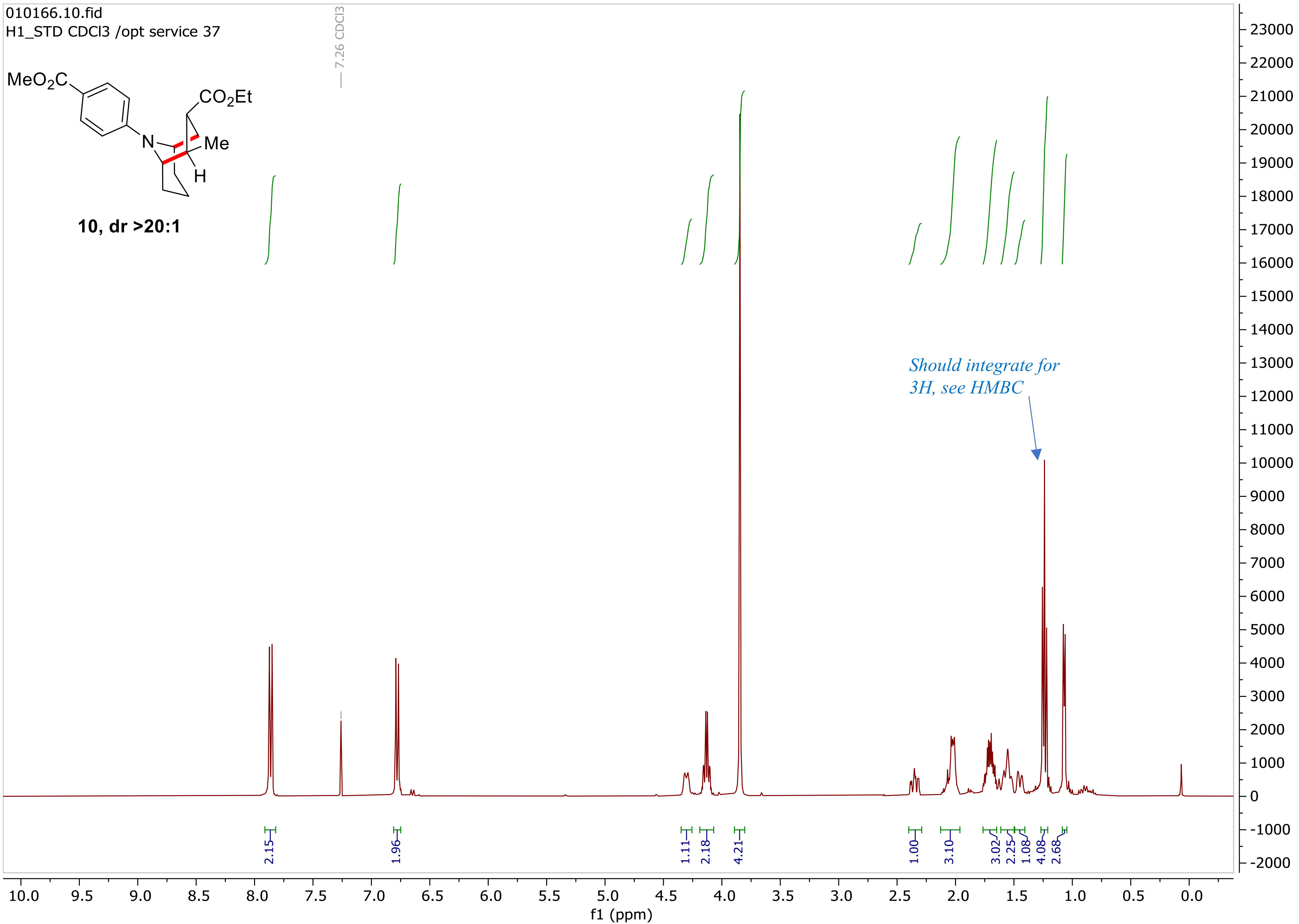

Ethyl 9-(4-methoxycarbonylphenyl)-2-methyl-9-azabicyclo[3.3.1]nonane-3-carboxylate (**10**) **dr >20:1** ^{1}H-NMR (400 MHz, $CDCl_3$)

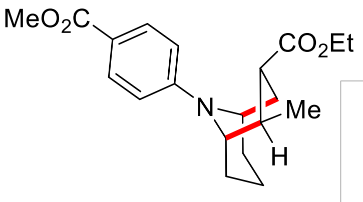

10, dr >20:1

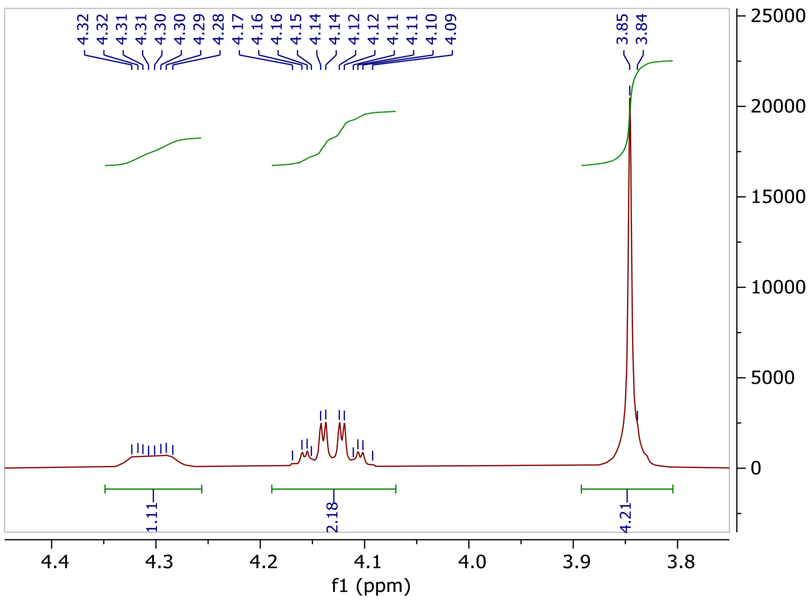

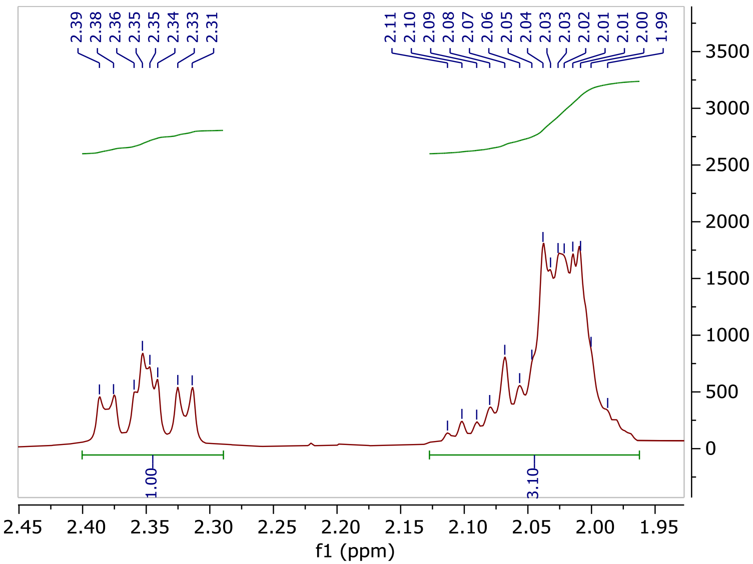

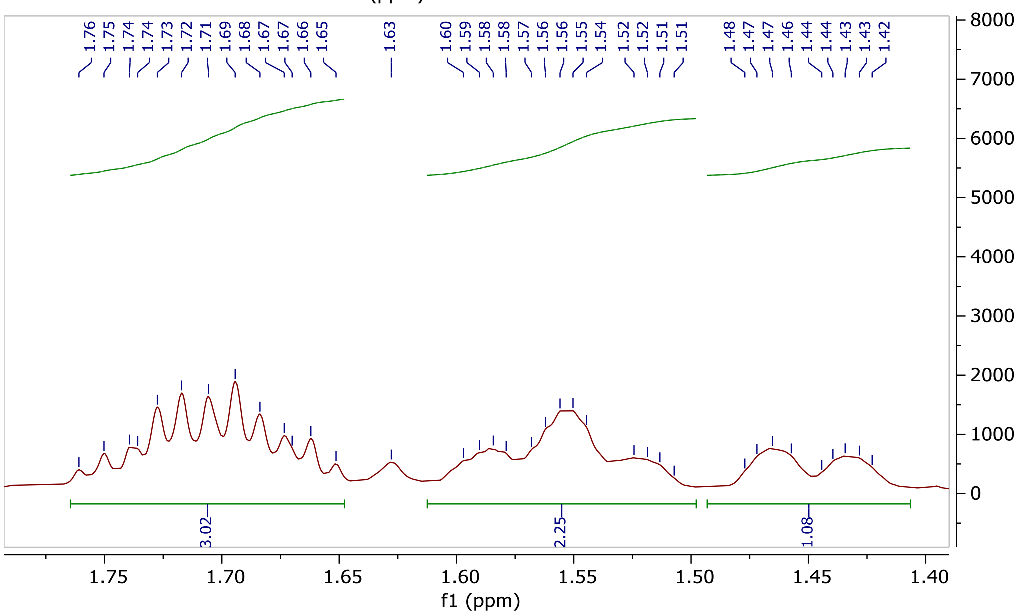

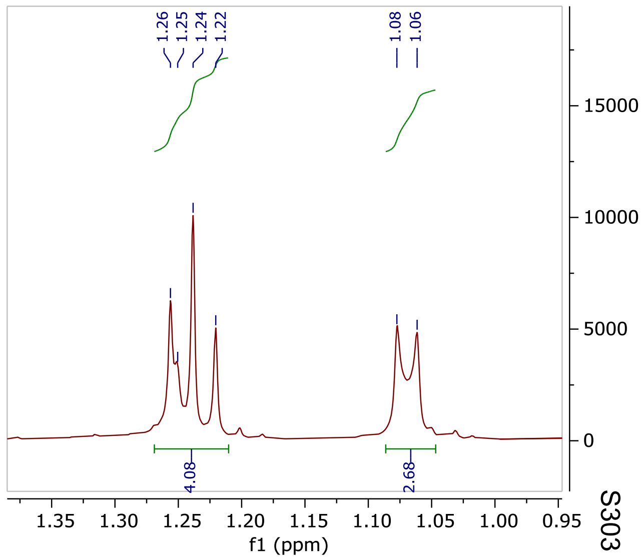

Ethyl 9-(4-methoxycarbonylphenyl)-2-methyl-9-azabicyclo[3.3.1]nonane-3-carboxylate (**10**) **dr >20:1** ^{13}C-NMR (75 MHz, $CDCl_3$)

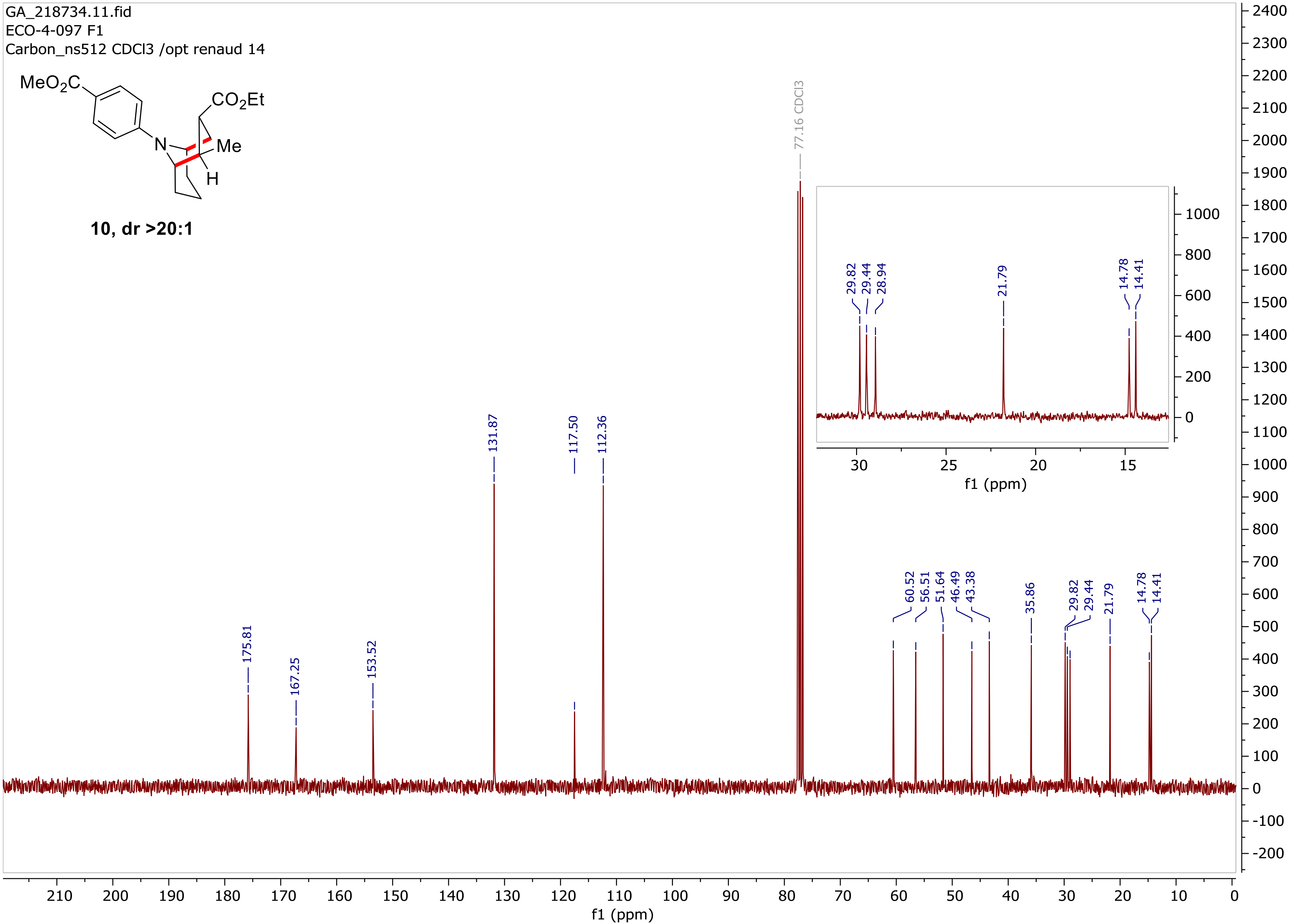

Ethyl 9-(4-methoxycarbonylphenyl)-2-methyl-9-azabicyclo[3.3.1]nonane-3-carboxylate (**10**) **dr >20:1** ^{13}C-NMR (75 MHz, $CDCl_3$)

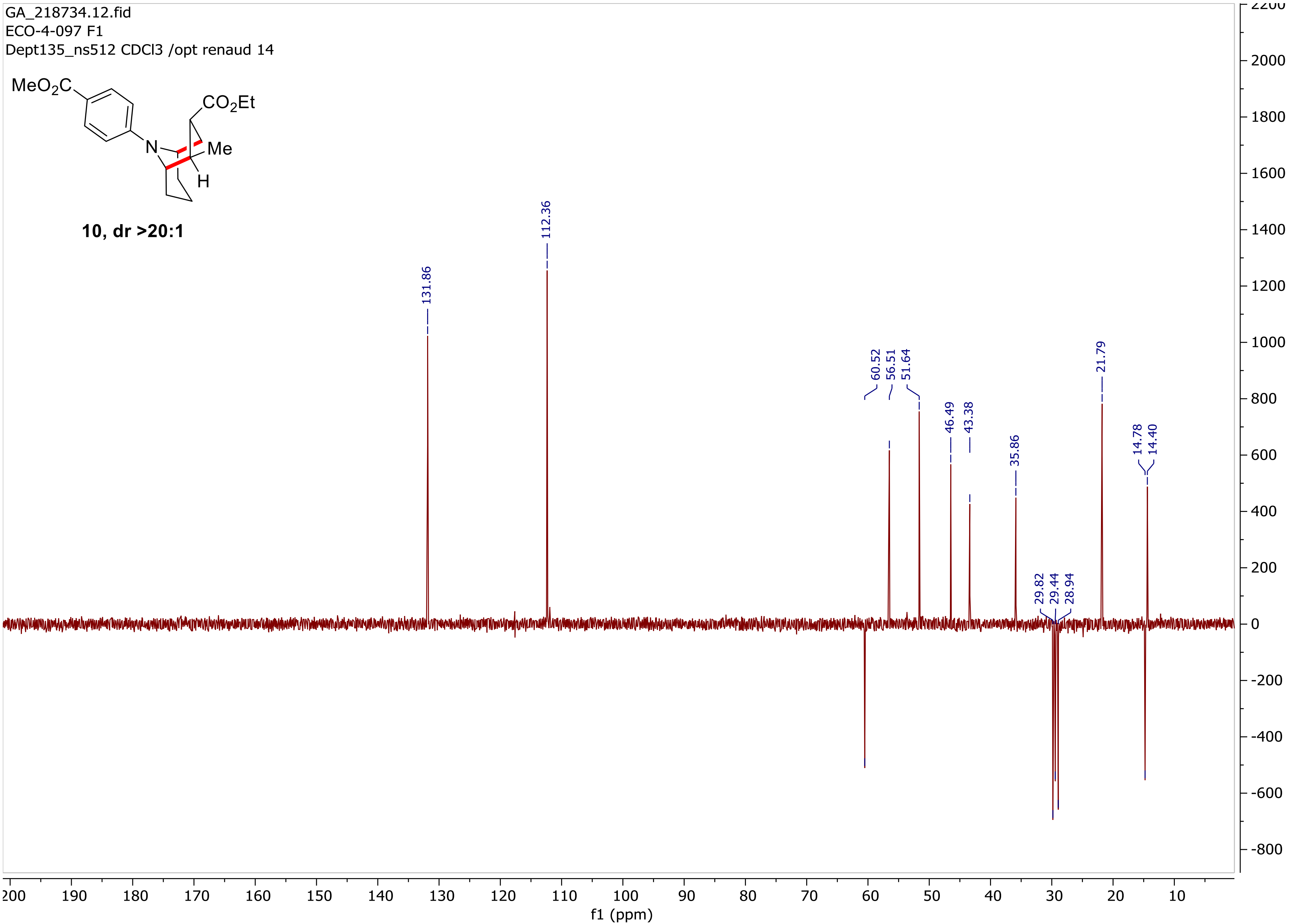

Ethyl 9-(4-methoxycarbonylphenyl)-2-methyl-9-azabicyclo[3.3.1]nonane-3-carboxylate (**10**) **dr >20:1** ^{1}H, ^{1}H-COSY NMR (400 MHz, $CDCl_3$)

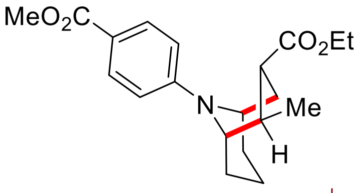

10, dr >20:1

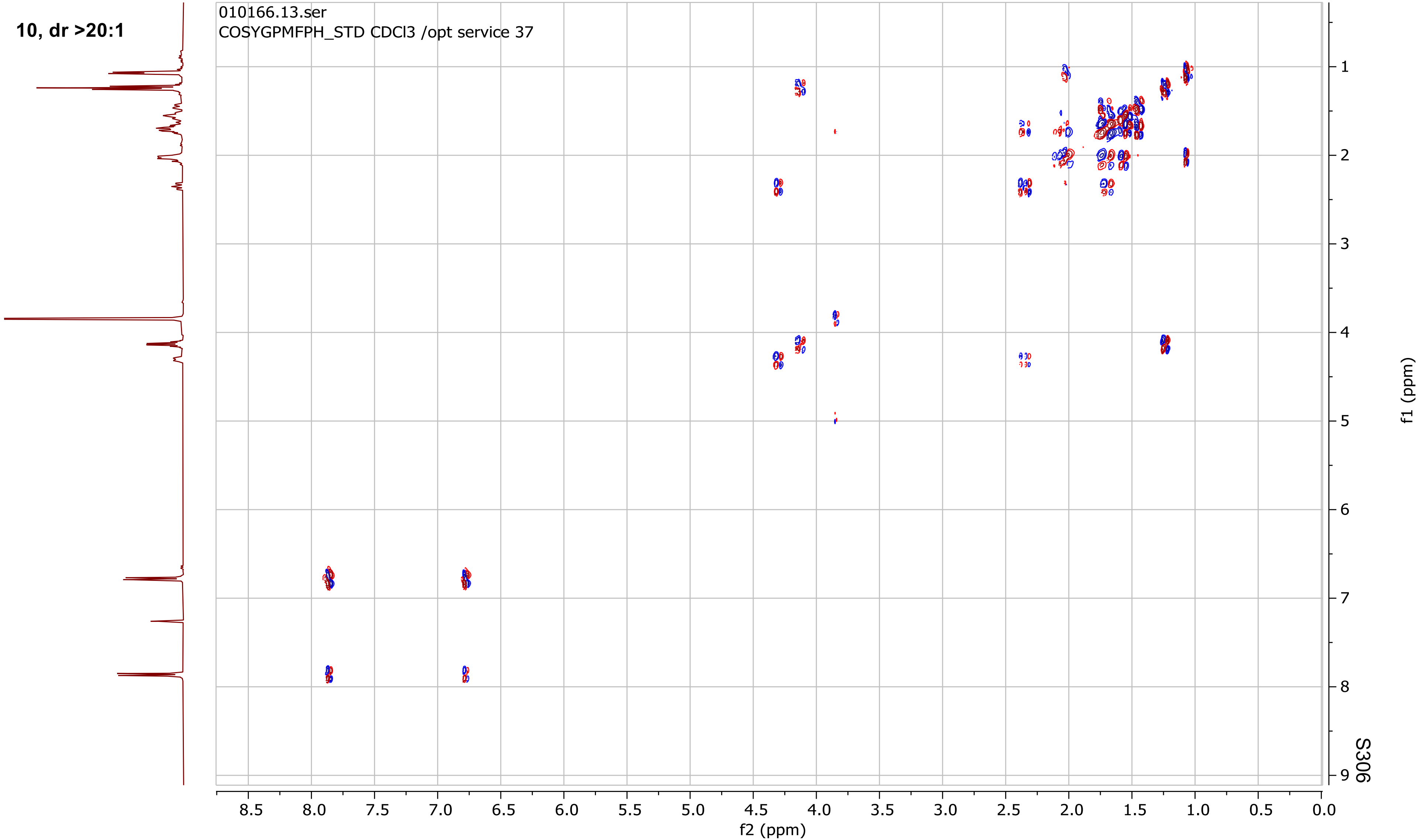

Ethyl 9-(4-methoxycarbonylphenyl)-2-methyl-9-azabicyclo[3.3.1]nonane-3-carboxylate (**10**) **dr >20:1** ^{1}H, ^{13}C-HSQC NMR (400 MHz, $CDCl_3$)

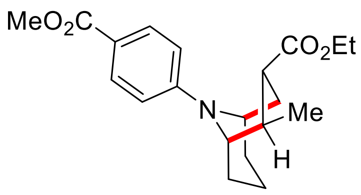

10, dr >20:1

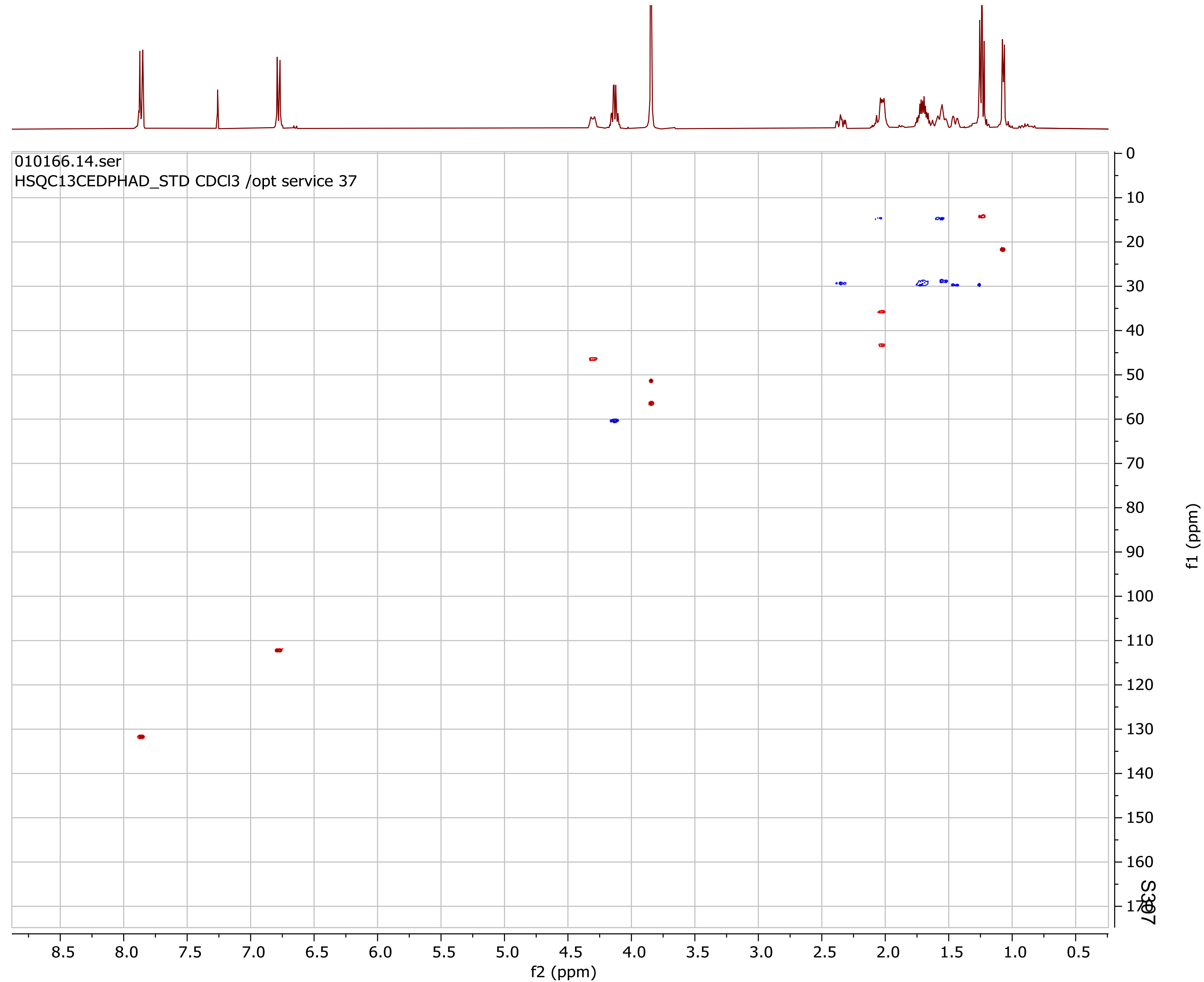

Ethyl 9-(4-methoxycarbonylphenyl)-2-methyl-9-azabicyclo[3.3.1]nonane-3-carboxylate (**10**) **dr >20:1** ^{1}H, ^{13}C-HSQC NMR (400 MHz, $CDCl_3$)

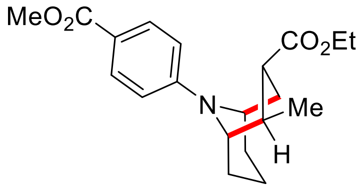

10, dr >20:1

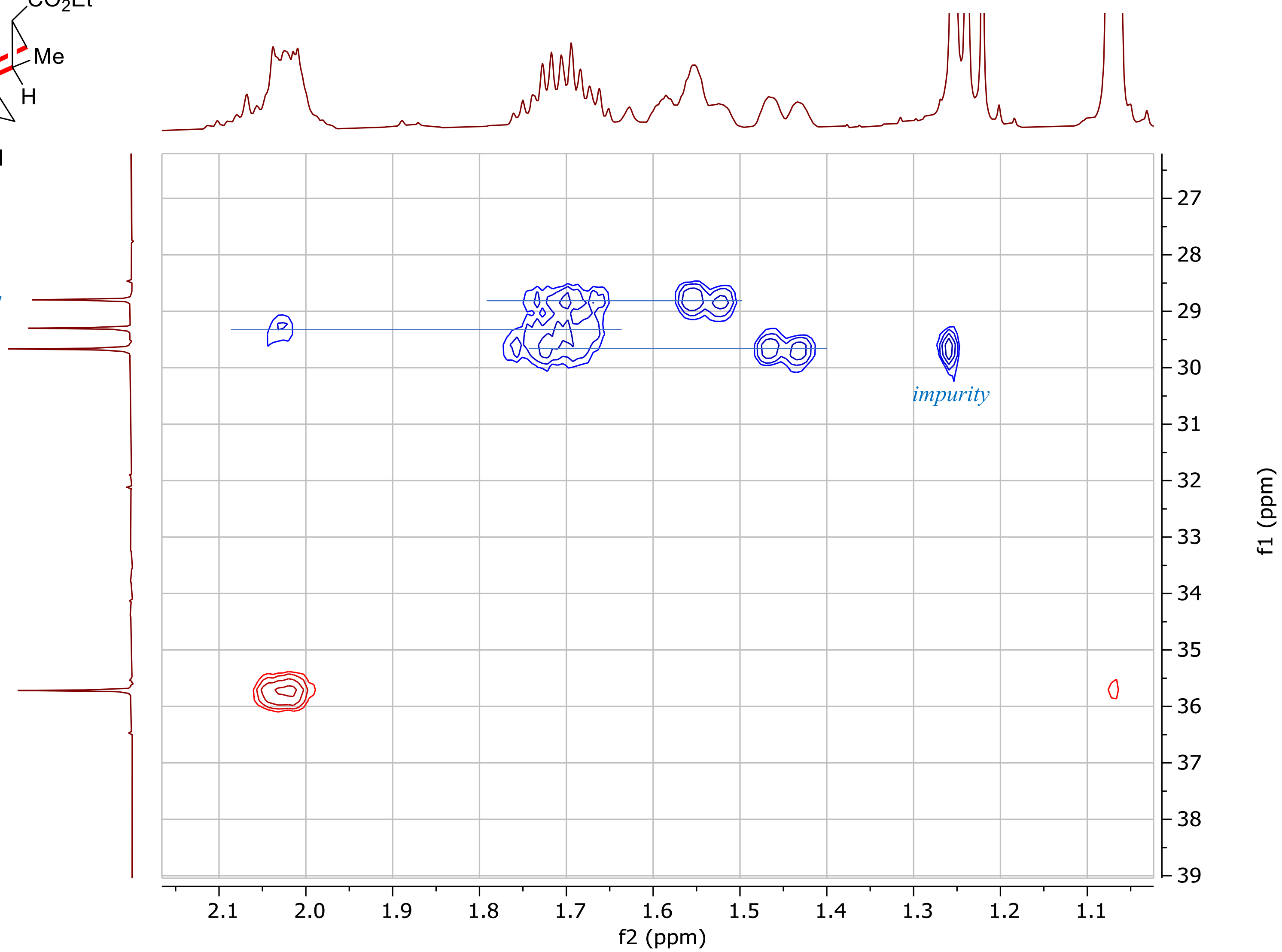

Ethyl 9-(4-methoxycarbonylphenyl)-2-methyl-9-azabicyclo[3.3.1]nonane-3-carboxylate (**10**) **dr >20:1** ^{1}H, ^{13}C-HMBC NMR (400 MHz, $CDCl_3$)

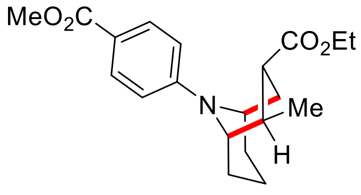

10, dr >20:1

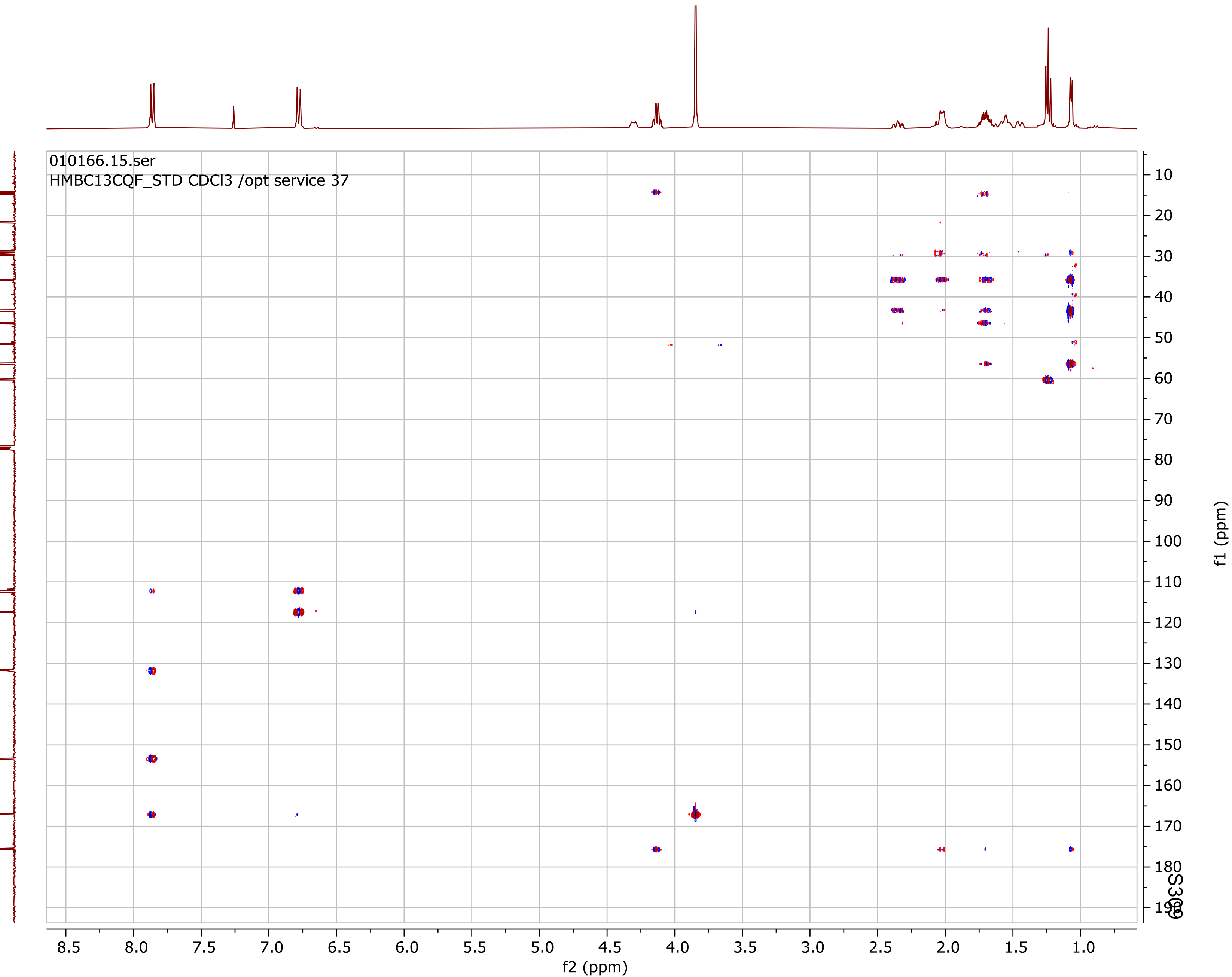

Ethyl 9-(4-methoxycarbonylphenyl)-2-methyl-9-azabicyclo[3.3.1]nonane-3-carboxylate (**10**) **dr >20:1** FT-IR, ATR-diamond

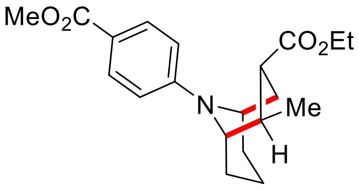

10, dr >20:1

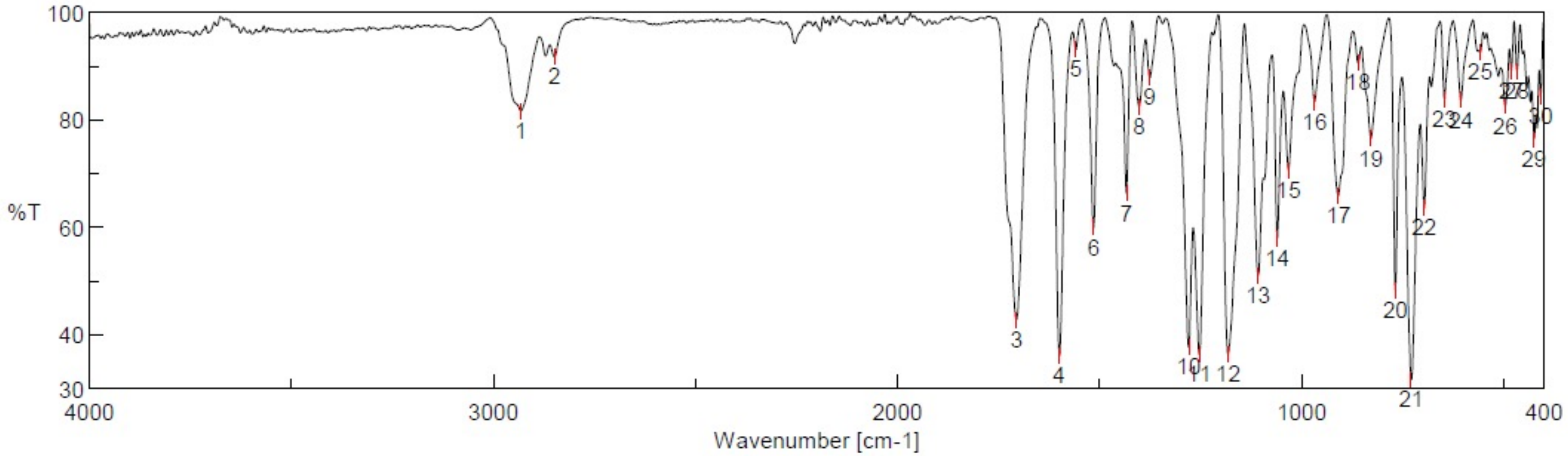

[Result of Peak Picking]

No.	Position	Intensity	No.	Position	Intensity	No.	Position	Intensity
1	2932.23	82	2	2849.31	92	3	1705.73	43
4	1599.66	36	5	1559.17	93	6	1514.81	60
7	1433.82	66	8	1402	82	9	1375.96	88
10	1279.54	38	11	1253.5	36	12	1182.15	36
13	1107.9	51	14	1060.66	58	15	1032.69	71
16	968.09	83	17	909.272	66	18	860.096	91
19	829.241	77	20	768.494	48	21	728.961	32
22	697.141	64	23	647.001	84	24	605.539	84
25	558.291	93	26	496.58	83	27	481.153	89
28	467.653	89	29	425.227	76	30	408.835	84

[4-[3-(hydroxymethyl)-2-methyl-9-azabicyclo[3.3.1]nonan-9-yl]phenyl]methanol (**11**) **dr >20:1**

^{1}H-NMR (400 MHz, $CDCl_3$)

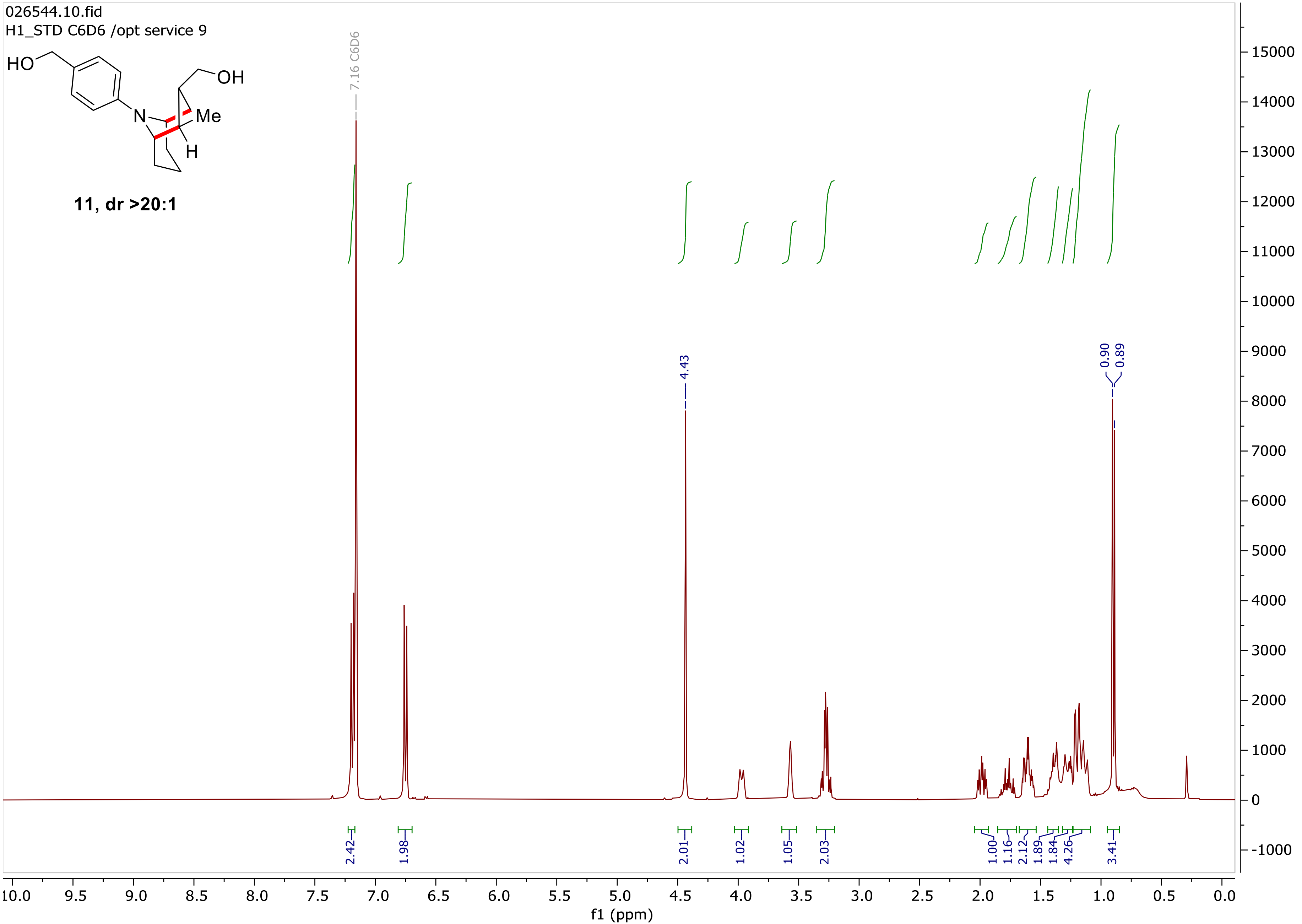

[4-[3-(hydroxymethyl)-2-methyl-9-azabicyclo[3.3.1]nonan-9-yl]phenyl]methanol (**11**) **dr >20:1**

^{1}H-NMR (400 MHz, $CDCl_3$)

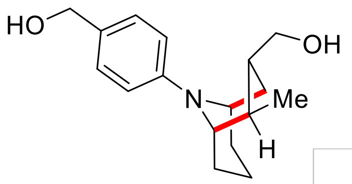

11, dr >20:1

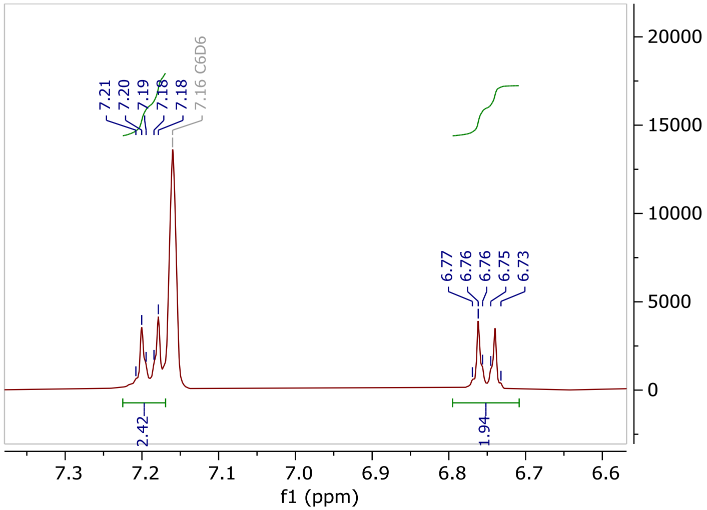

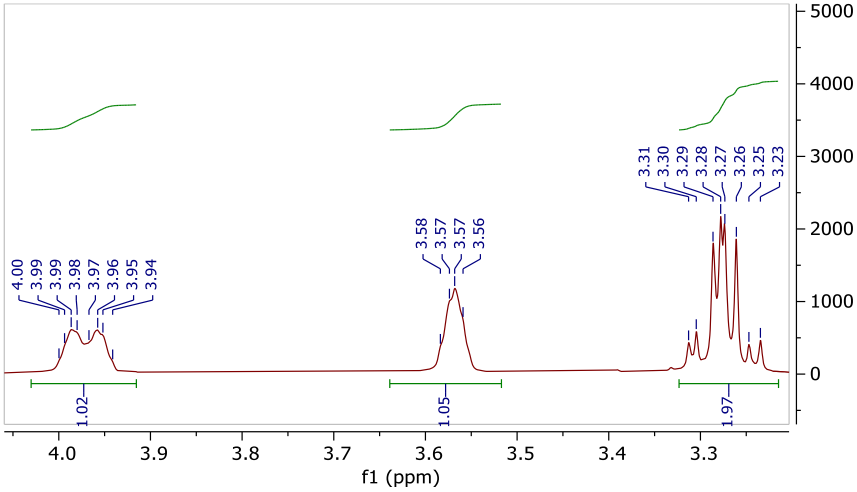

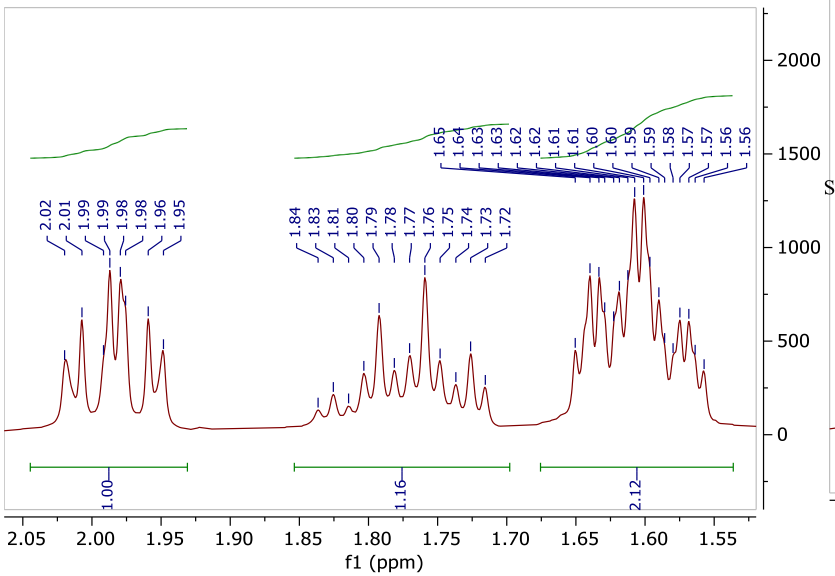

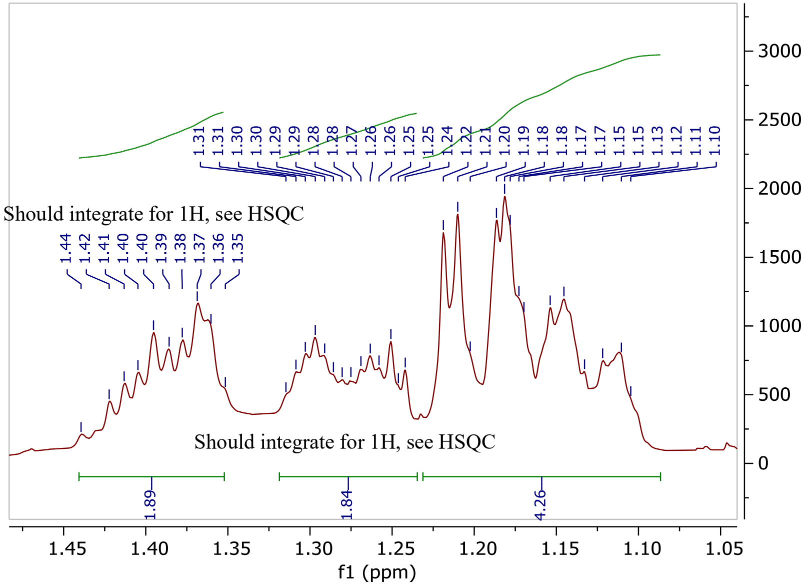

[4-[3-(hydroxymethyl)-2-methyl-9-azabicyclo[3.3.1]nonan-9-yl]phenyl]methanol (**11**) **dr >20:1**

^{13}C-NMR (101 MHz, $CDCl_3$)

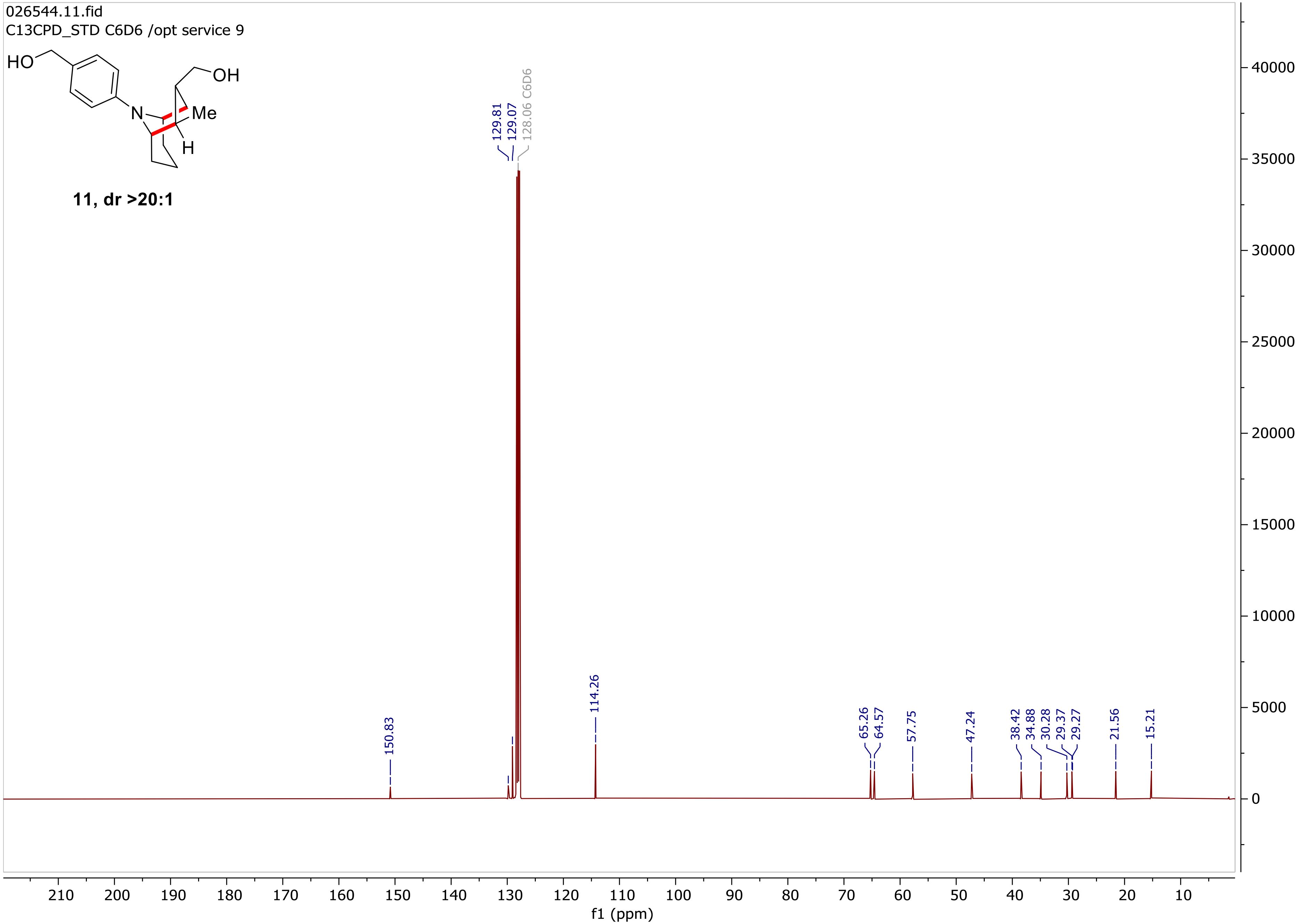

[4-[3-(hydroxymethyl)-2-methyl-9-azabicyclo[3.3.1]nonan-9-yl]phenyl]methanol (**11**) **dr >20:1**

^{13}C-NMR (101 MHz, $CDCl_3$)

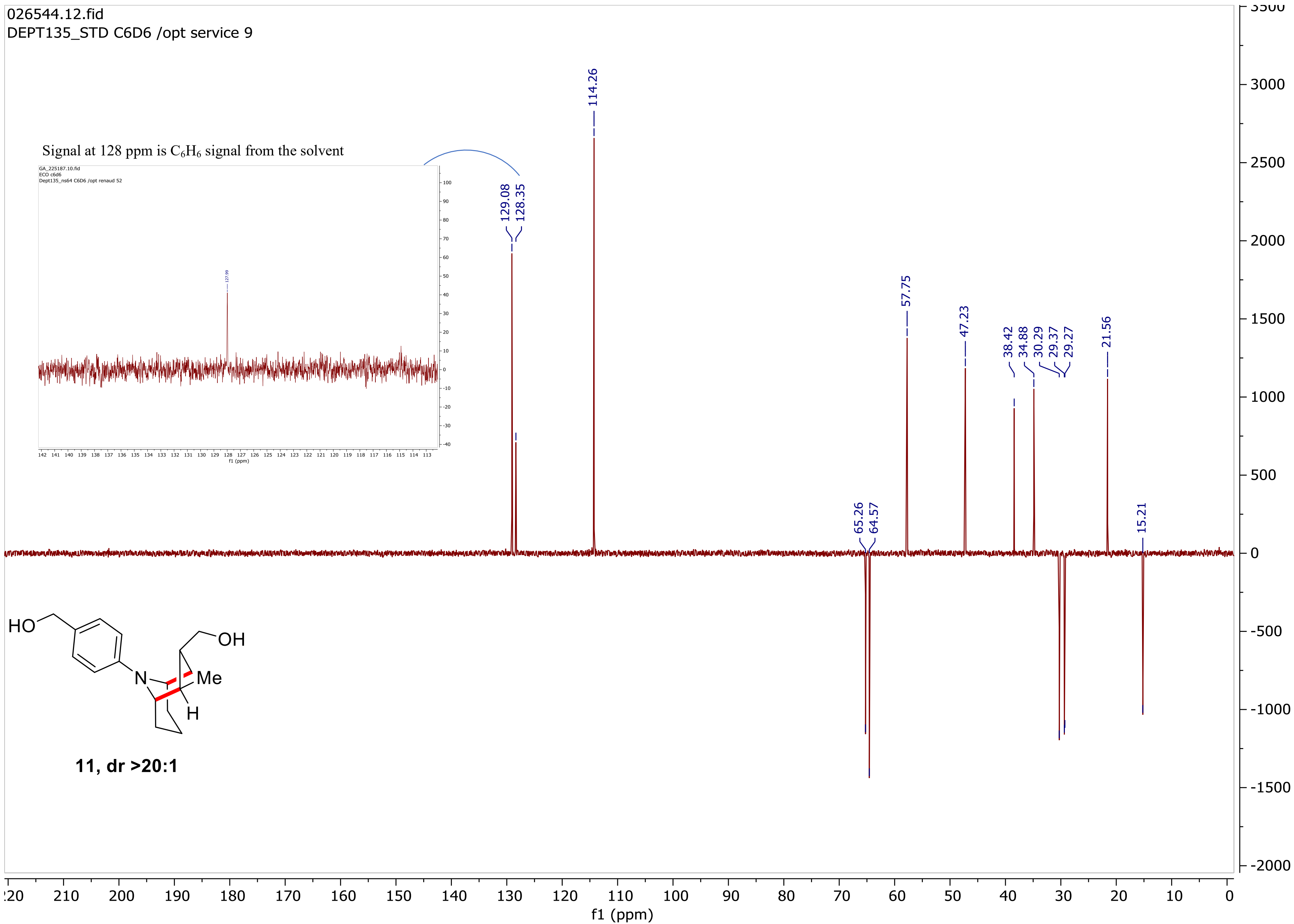

11, dr >20:1

[4-[3-(hydroxymethyl)-2-methyl-9-azabicyclo[3.3.1]nonan-9-yl]phenyl]methanol (**11**) **dr >20:1**

^{1}H, ^{1}H-COSY NMR (400 MHz, $CDCl_3$)

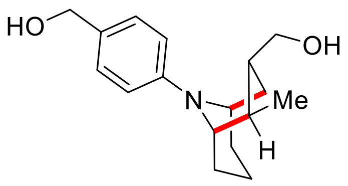

11, dr >20:1

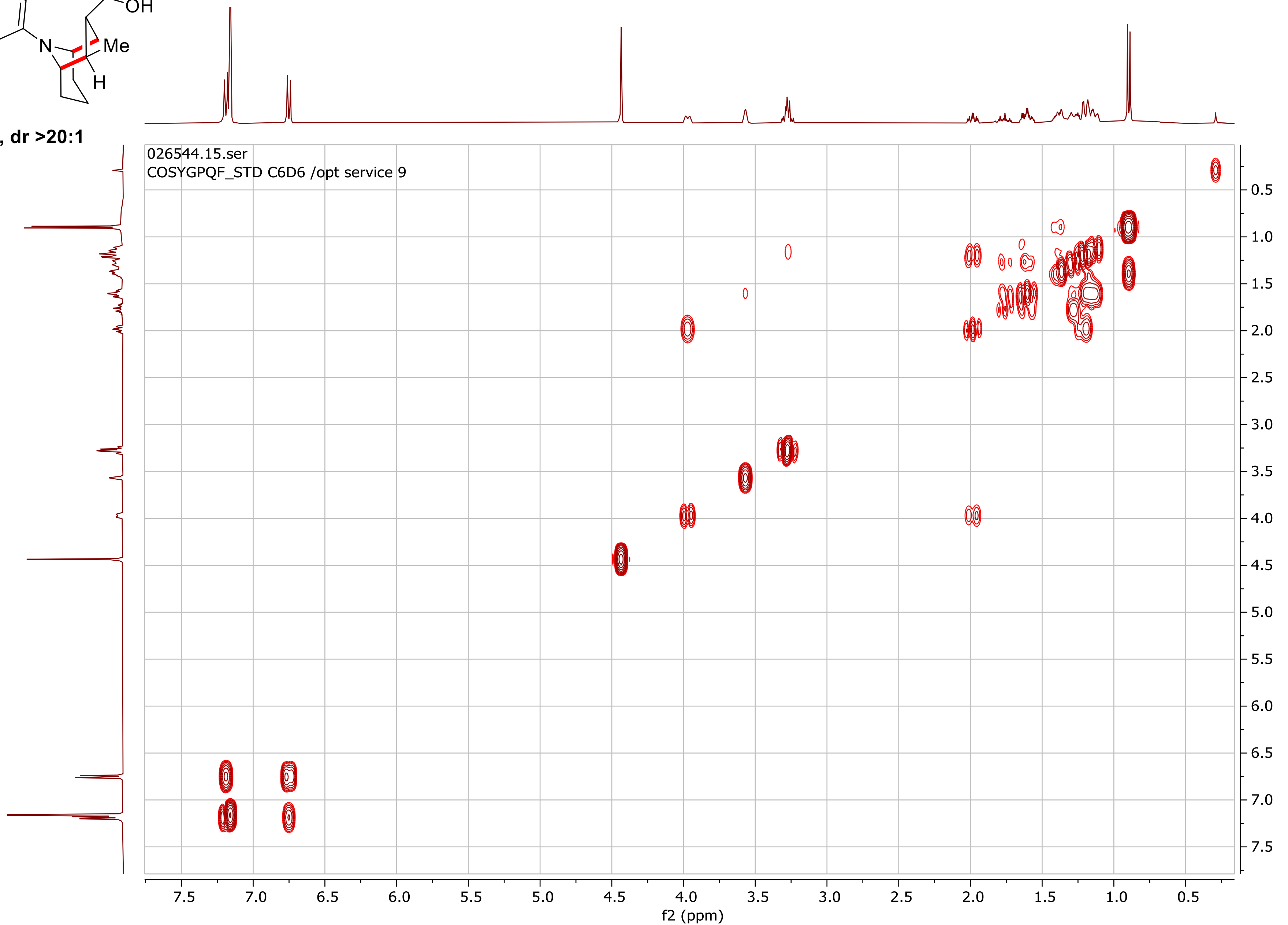

[4-[3-(hydroxymethyl)-2-methyl-9-azabicyclo[3.3.1]nonan-9-yl]phenyl]methanol (**11**) **dr >20:1**

^{1}H, ^{13}C-HSQC NMR (300 MHz, $CDCl_3$)

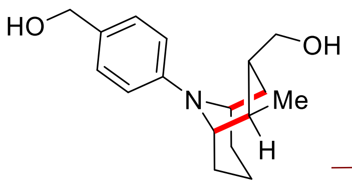

11, dr >20:1

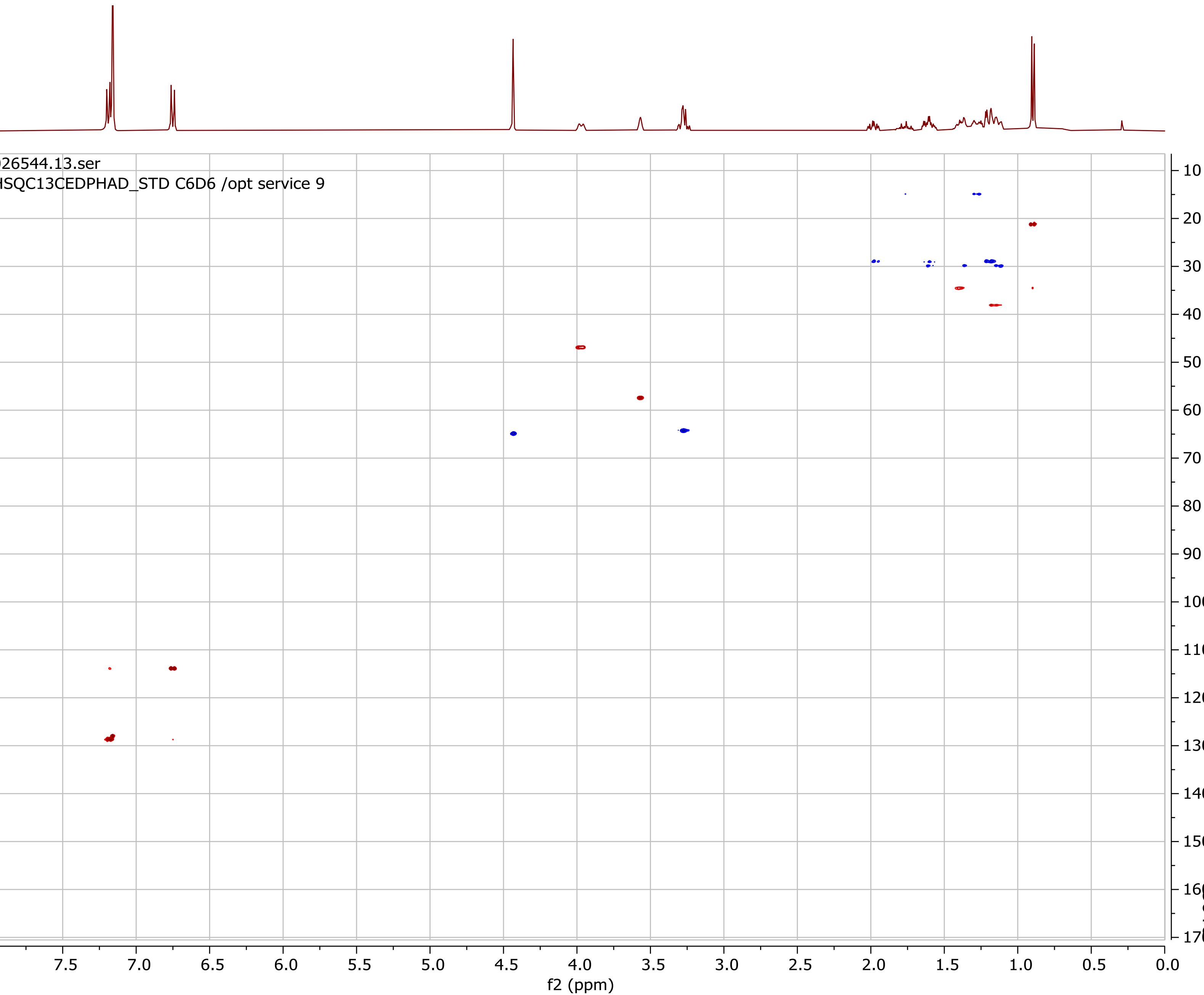

[4-[3-(hydroxymethyl)-2-methyl-9-azabicyclo[3.3.1]nonan-9-yl]phenyl]methanol (**11**) **dr >20:1**

^{1}H, ^{13}C-HMBC NMR (400 MHz, $CDCl_3$)

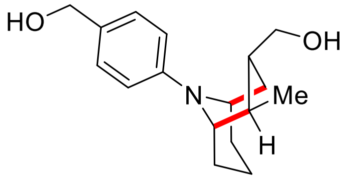

11, dr >20:1

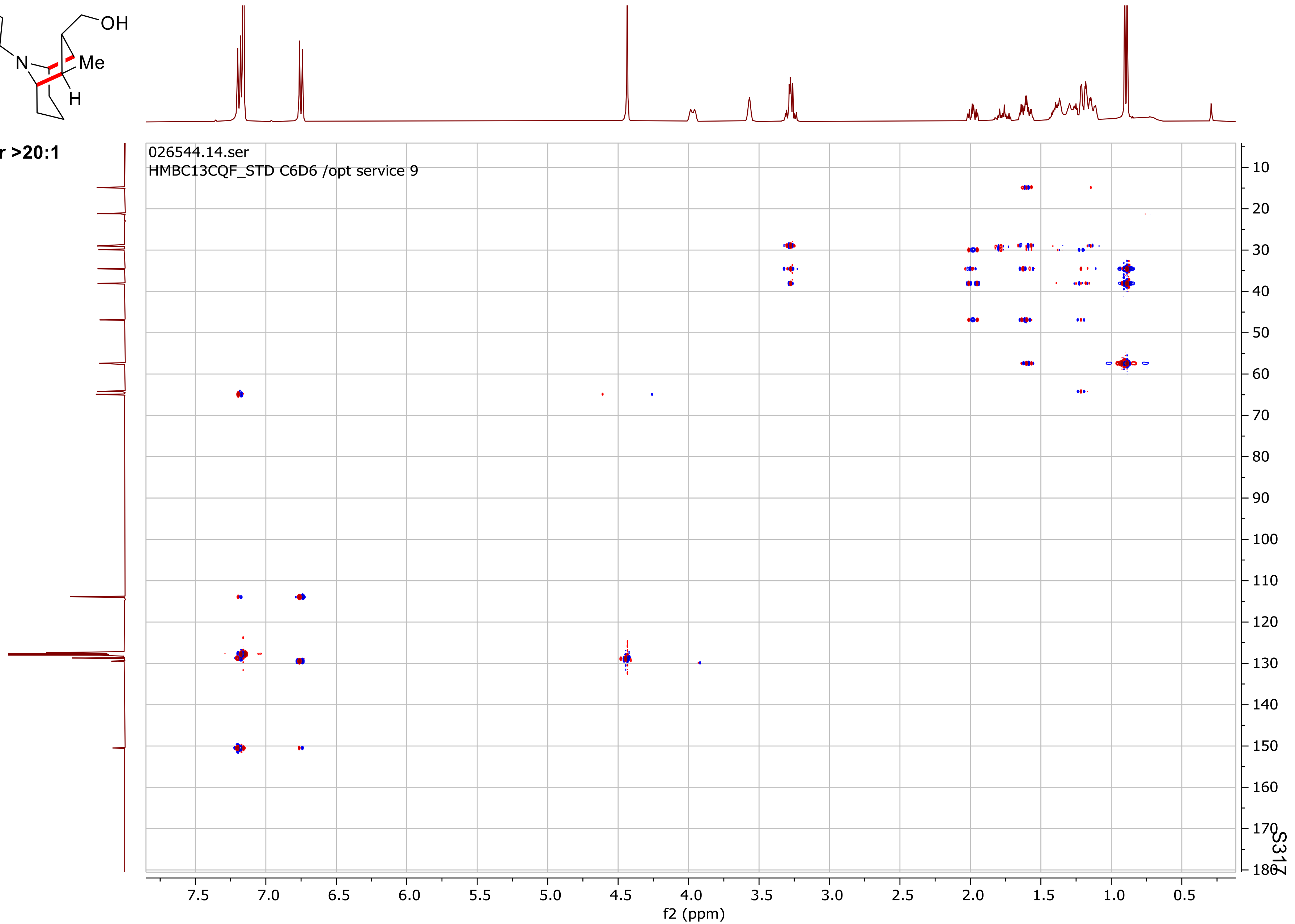

[4-[3-(hydroxymethyl)-2-methyl-9-azabicyclo[3.3.1]nonan-9-yl]phenyl]methanol (**11**) **dr >20:1**

FT-IR, ATR-diamond

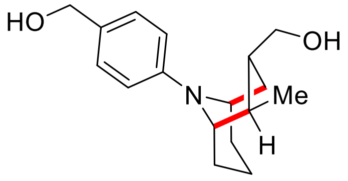

11, dr >20:1

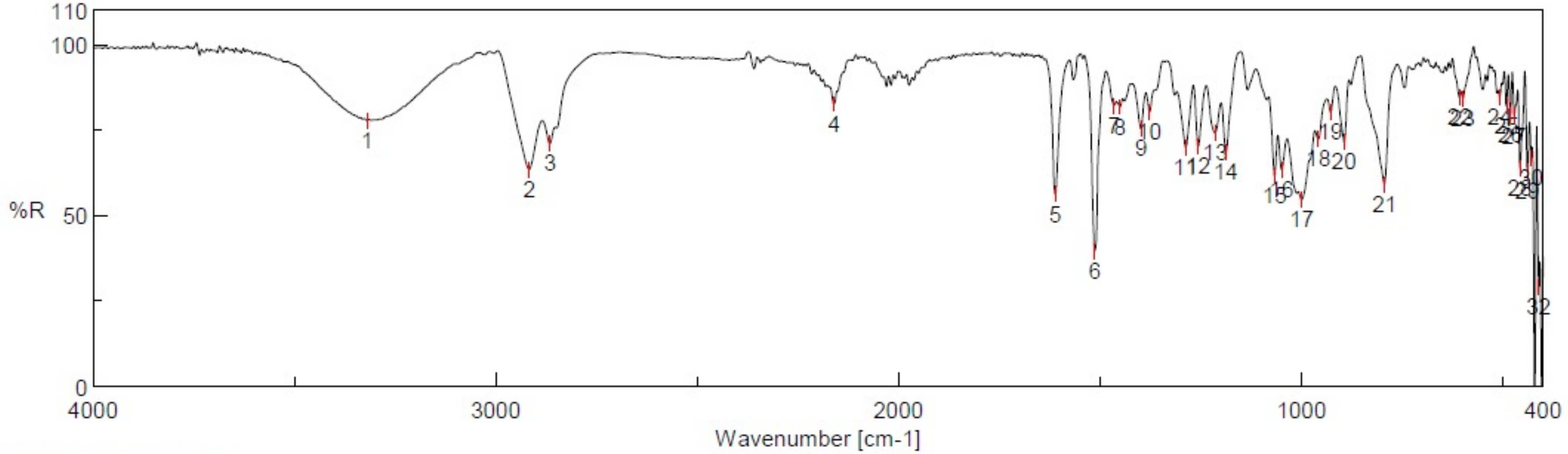

[Result of Peak Picking]

No.	Position	Intensity	No.	Position	Intensity	No.	Position	Intensity
1	3319.86	77.7156	2	2916.81	63.424	3	2865.7	70.9039
4	2159.88	82.6228	5	1611.23	56.1613	6	1512.88	39.5567
7	1465.63	82.14	8	1450.21	81.7142	9	1398.14	75.5495
10	1376.93	80.2783	11	1287.25	69.9316	12	1256.4	70.1338
13	1213.97	74.1082	14	1186.97	68.5396	15	1066.44	61.4718
16	1049.09	63.2568	17	998.946	54.6209	18	957.484	72.2618
19	927.593	80.0992	20	893.844	71.4322	21	793.564	59.136
22	607.467	84.5437	23	598.789	84.2035	24	507.187	84.5822
25	490.795	82.0058	26	481.153	79.3496	27	471.51	79.1521
28	457.047	63.714	29	438.726	62.6998	30	429.084	66.8997
31	420.406	0	32	410.763	29.1112	33	402.085	0

[4-[3-(hydroxymethyl)-2-methyl-9-azabicyclo[3.3.1]nonan-9-yl]phenyl]methanol (**11**)

X-ray

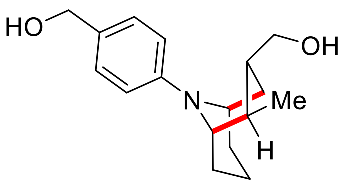

11

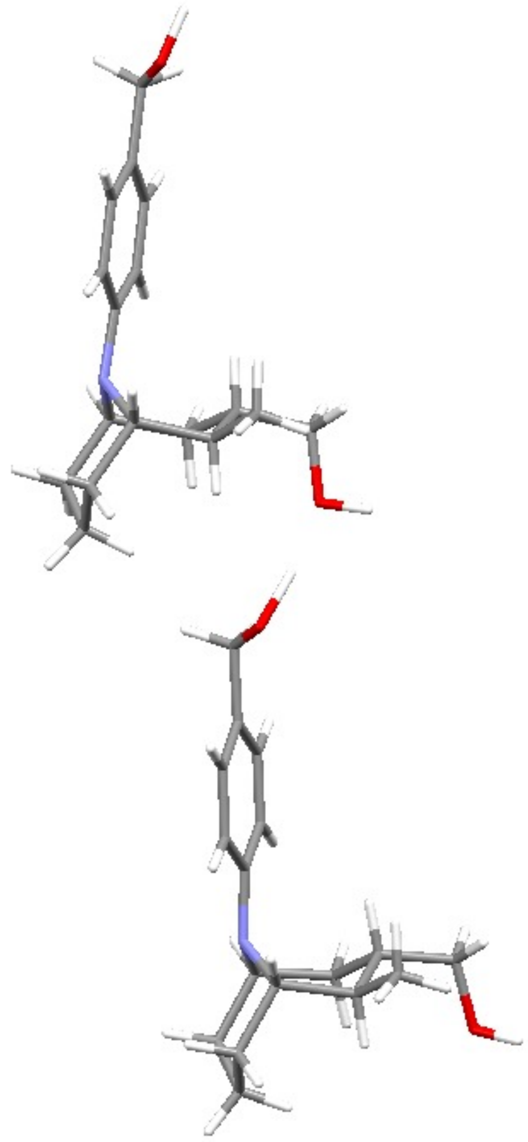

Synthesis of mono-allylated intermediates

Ethyl 2-[(1-phenylpyrrolidin-2-yl)methyl]prop-2-enoate (**2a**)

^{1}H-NMR (400 MHz, $CDCl_3$)

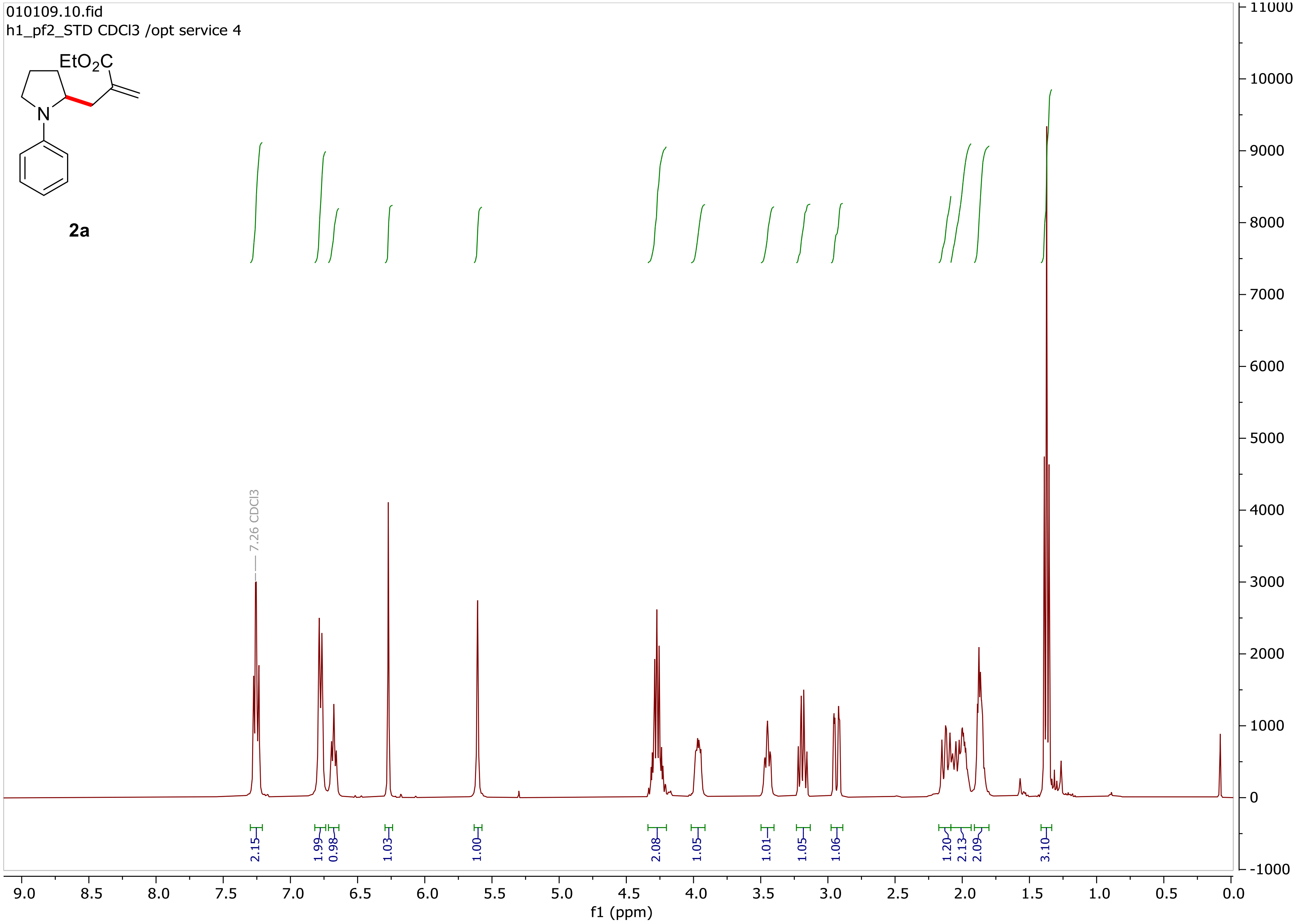

Ethyl 2-[(1-phenylpyrrolidin-2-yl)methyl]prop-2-enoate (**2a**)

^{1}H-NMR (400 MHz, $CDCl_3$)

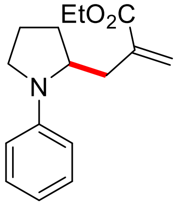

2a

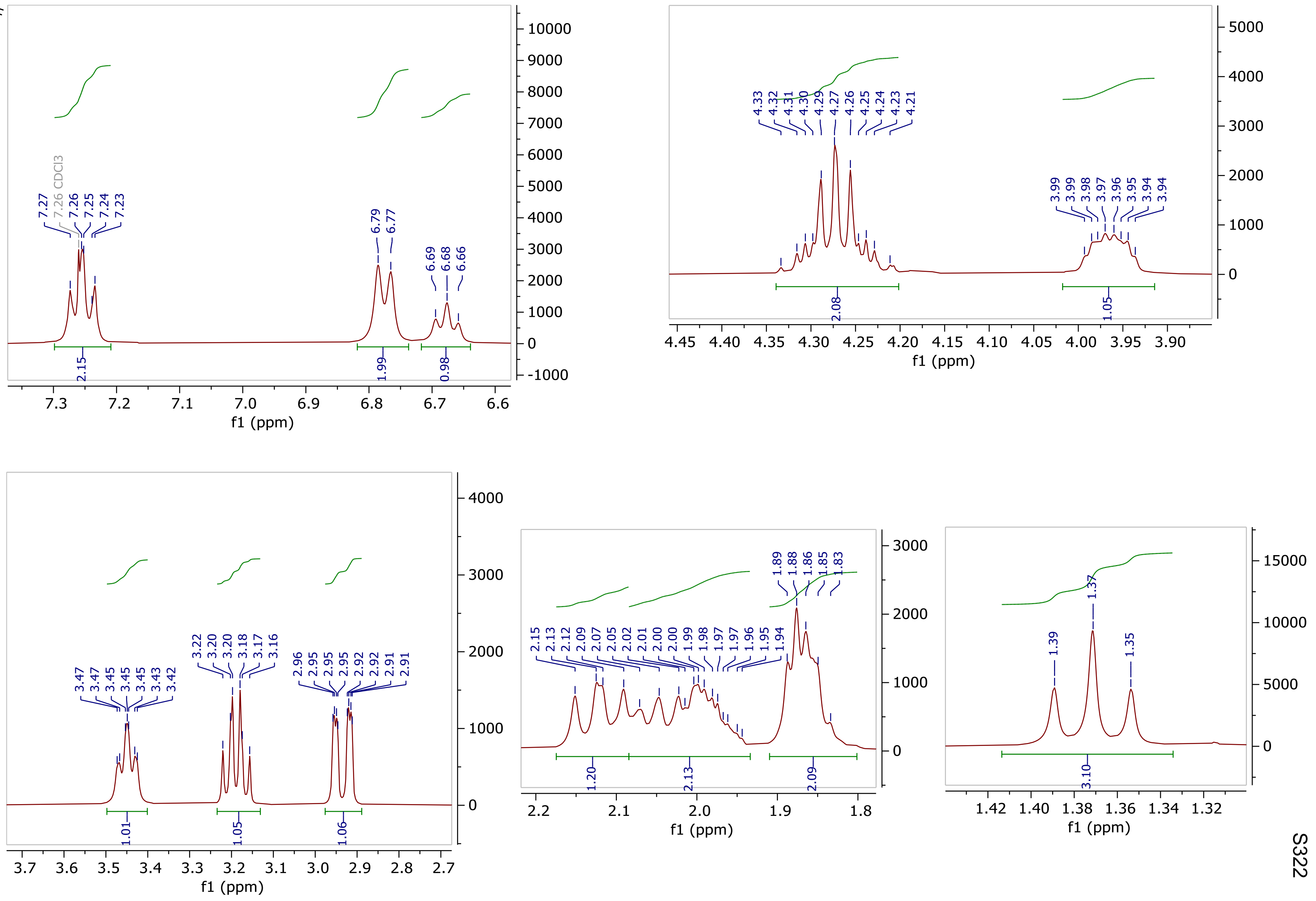

Ethyl 2-[(1-phenylpyrrolidin-2-yl)methyl]prop-2-enoate (**2a**)

^{13}C-NMR (101 MHz, $CDCl_3$)

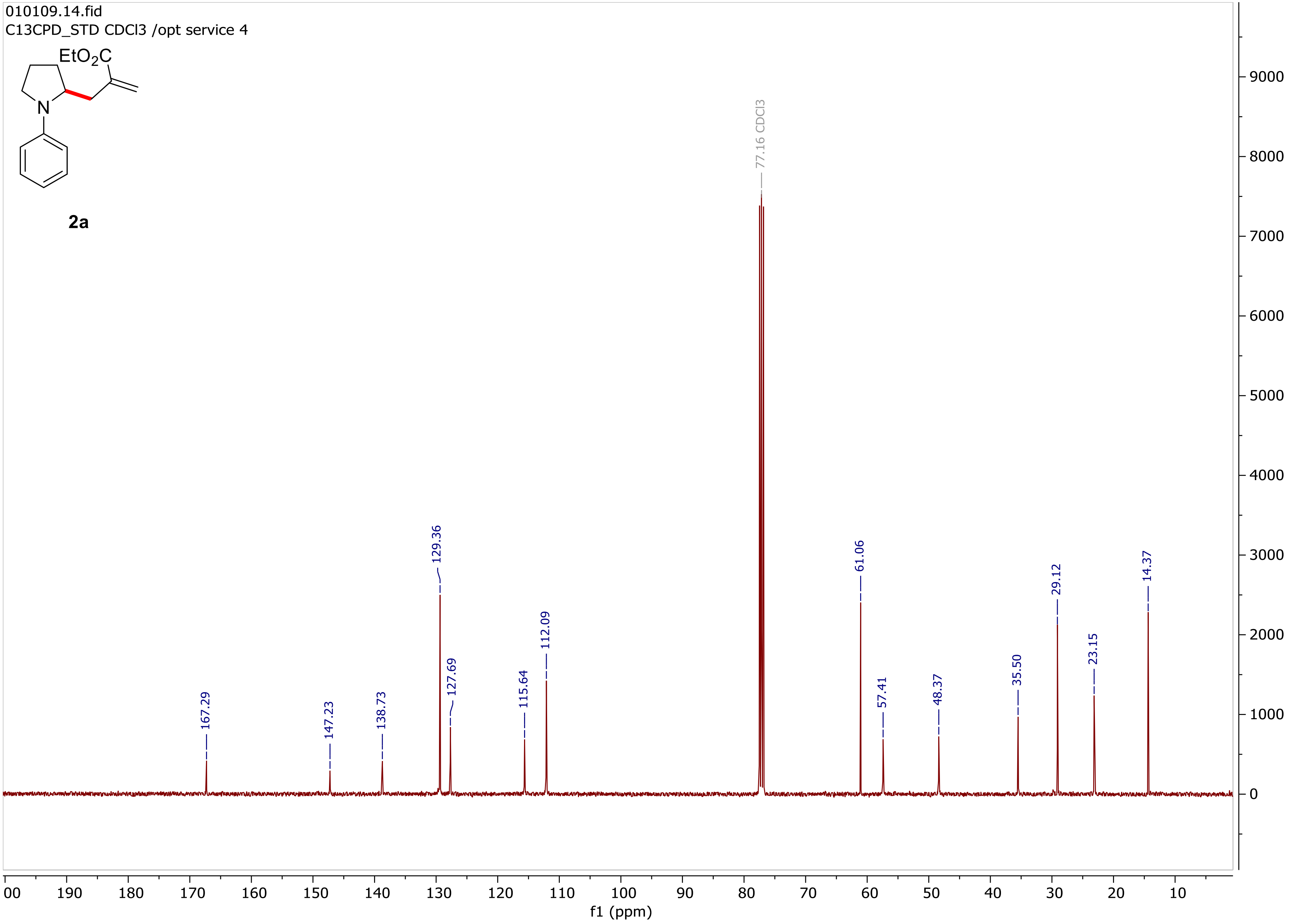

Ethyl 2-[(1-phenylpyrrolidin-2-yl)methyl]prop-2-enoate (**2a**)

^{13}C-NMR (101 MHz, $CDCl_3$)

010109.15.fid
DEPT135_STD CDCl3 /opt service 4

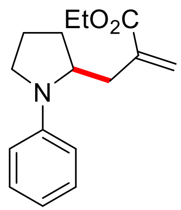

2a

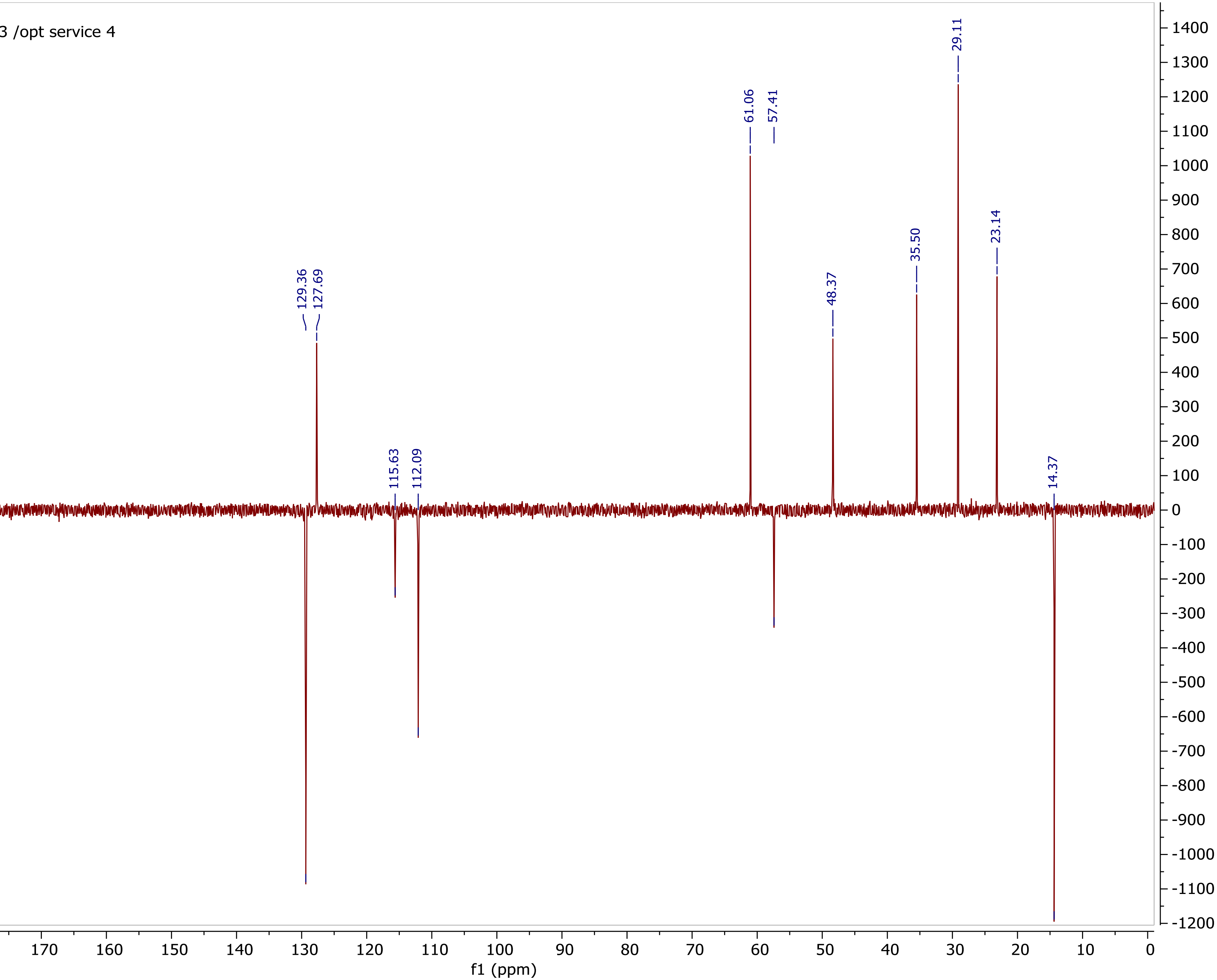

Ethyl 2-[(1-phenylpyrrolidin-2-yl)methyl]prop-2-enoate (**2a**)

^1H, ^1H-COSY NMR (400 MHz, $CDCl_3$)

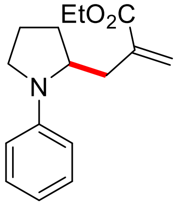

2a

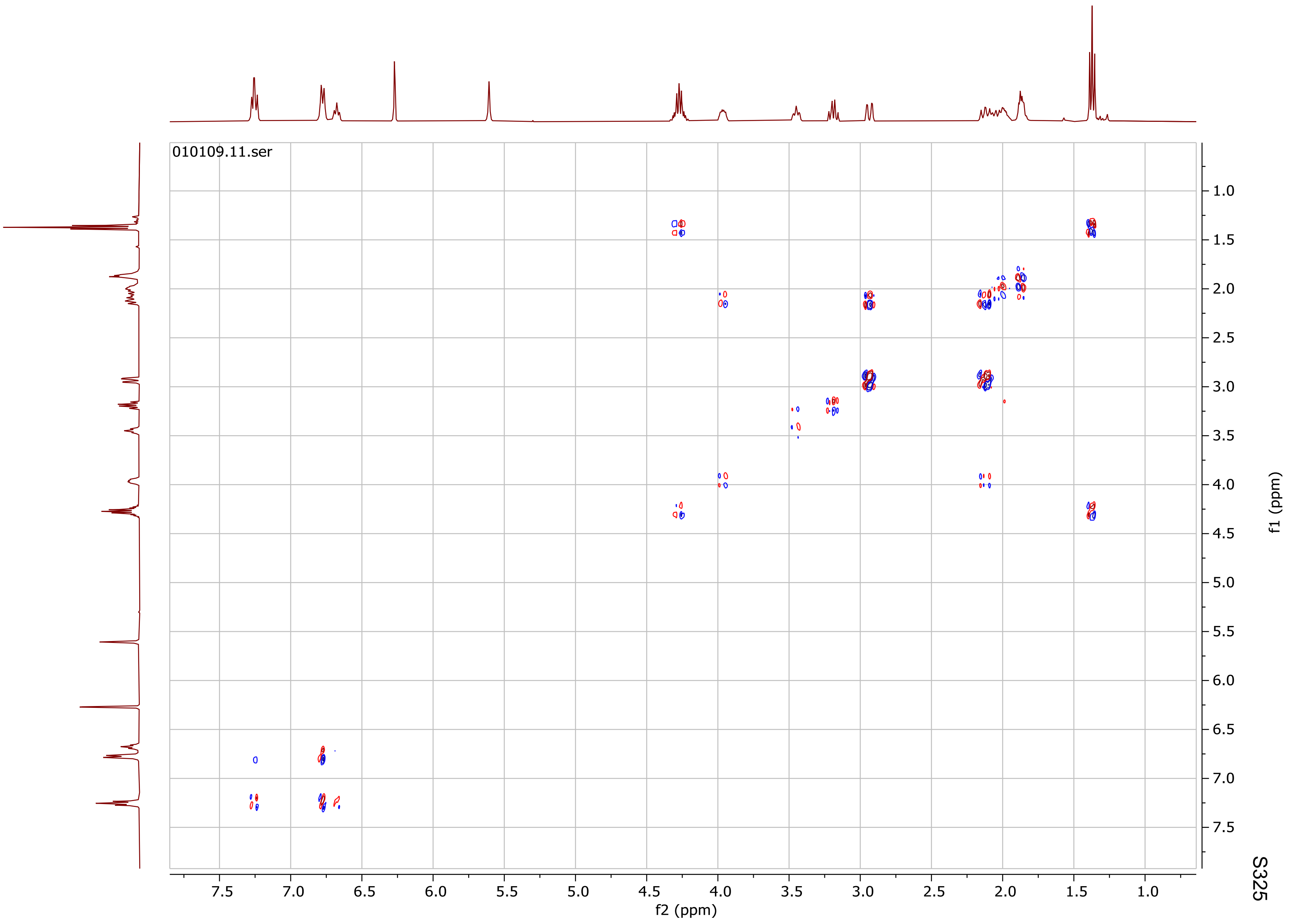

Ethyl 2-[(1-phenylpyrrolidin-2-yl)methyl]prop-2-enoate (**2a**)

^{1}H, ^{13}C-HSQC NMR (400 MHz, $CDCl_3$)

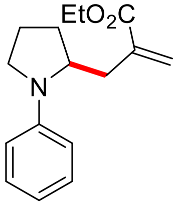

2a

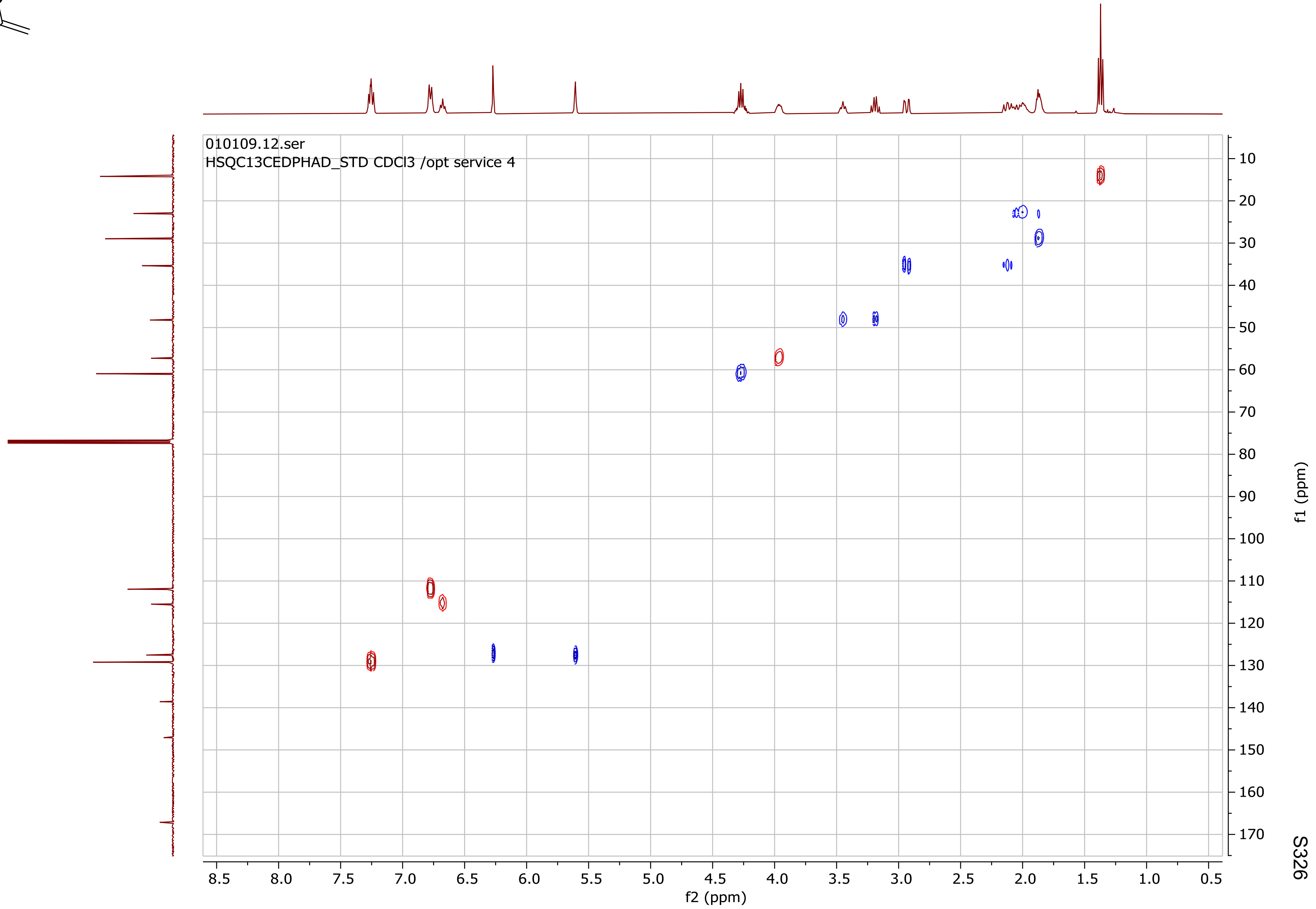

Ethyl 2-[(1-phenylpyrrolidin-2-yl)methyl]prop-2-enoate (**2a**)

^{1}H, ^{13}C-HMBC NMR (400 MHz, $CDCl_3$)

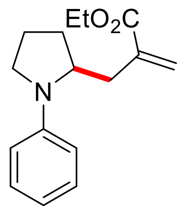

2a

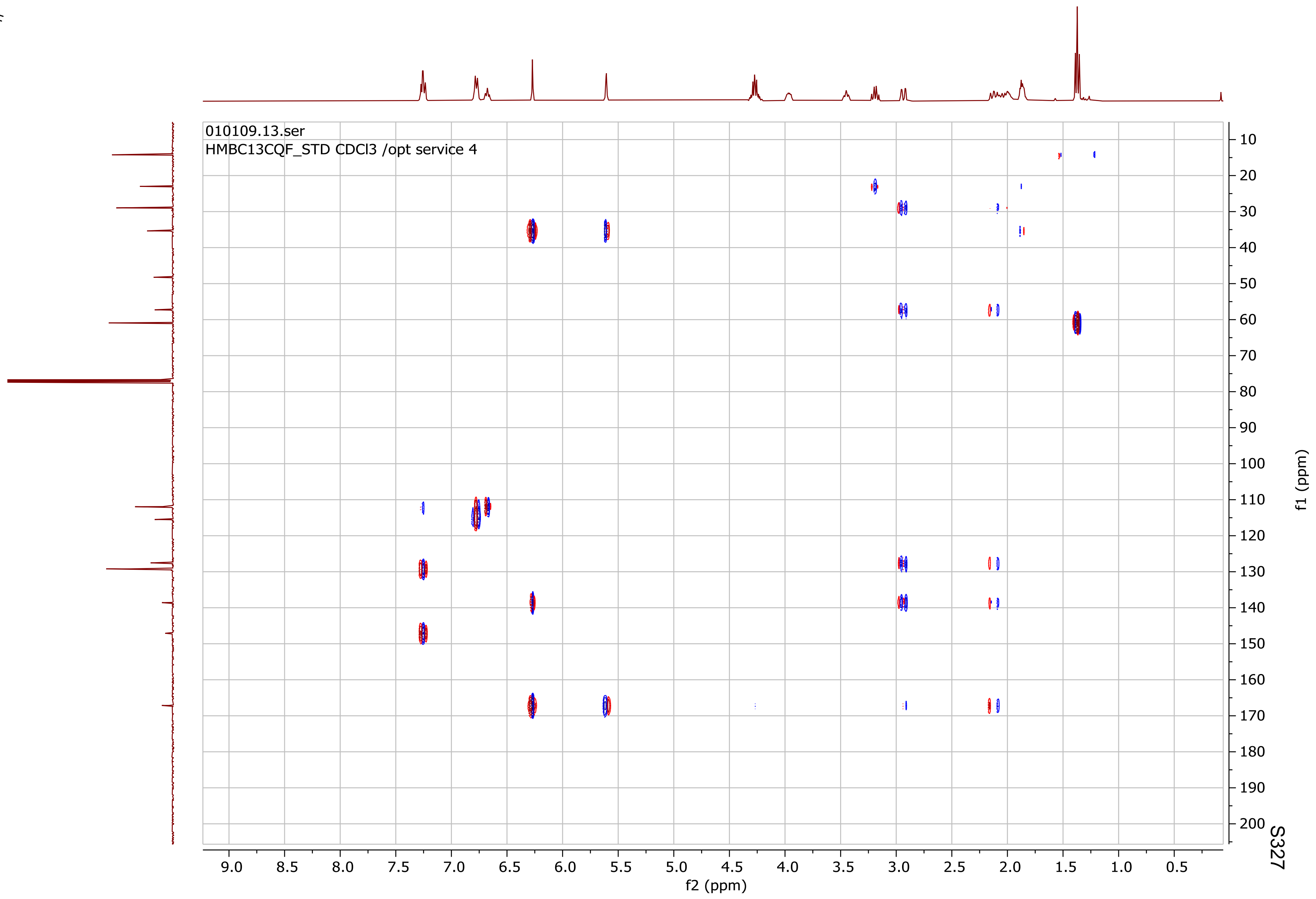

Ethyl 2-[(1-phenylpyrrolidin-2-yl)methyl]prop-2-enoate (**2a**)

FT-IR, ATR-diamond

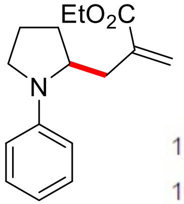

2a

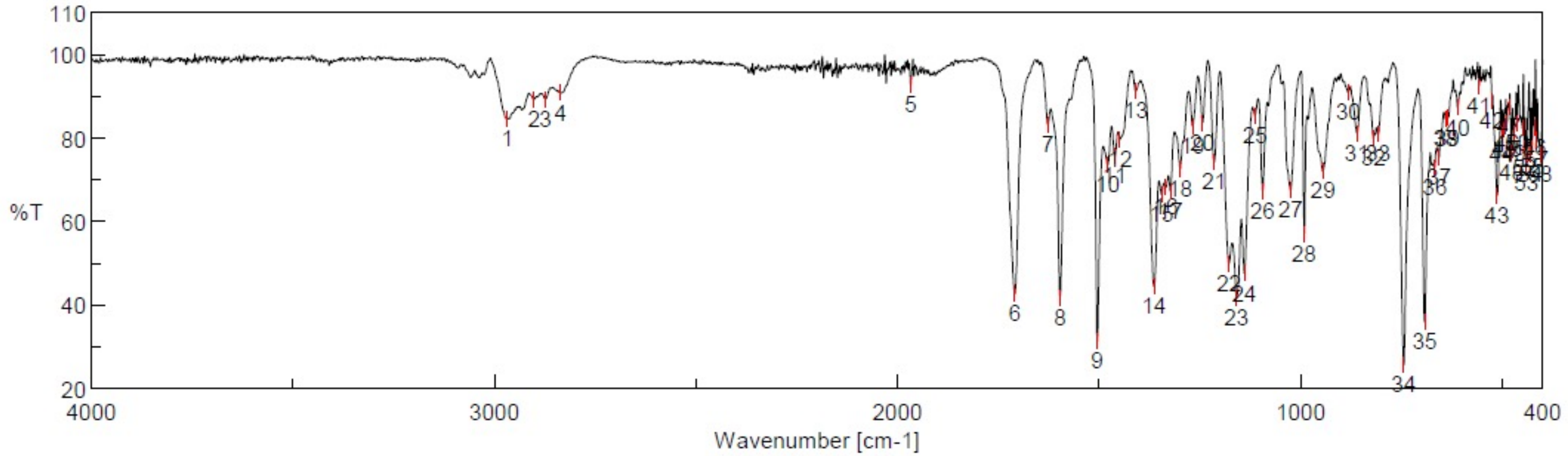

[Result of Peak Picking]

No.	Position	Intensity	No.	Position	Intensity	No.	Position	Intensity
1	2967.91	85	2	2901.86	89	3	2872.93	89
4	2835.33	91	5	1967.52	93	6	1709.1	43
7	1627.14	83	8	1596.77	42	9	1504.69	31
10	1478.65	74	11	1461.3	76	12	1448.28	80
13	1409.23	91	14	1363.91	44	15	1344.62	66
16	1334.5	68	17	1322.93	67	18	1298.82	73
19	1267.97	83	20	1243.38	84	21	1214.45	74
22	1177.81	50	23	1159.01	42	24	1139.72	48
25	1113.21	85	26	1094.4	67	27	1024.98	67
28	991.232	57	29	944.949	72	30	882.274	91
31	860.096	81	32	819.598	80	33	807.546	81
34	743.906	26	35	692.32	36	36	667.25	73
37	657.125	75	38	640.733	85	39	635.912	85
40	610.36	87	41	557.327	92	42	525.025	89
43	512.972	66	44	500.437	81	45	495.616	82
46	488.384	84	47	483.081	87	48	477.296	76
49	471.028	81	50	462.832	84	51	449.815	82

Ethyl 2-[(5-methyl-1-phenyl-pyrrolidin-2-yl)methyl]prop-2-enoate (**2f**) ***cis/trans*** **1:2.8**

^{1}H-NMR (300 MHz, $CDCl_3$)

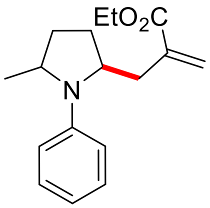

2f
cis/trans **1:2.8**

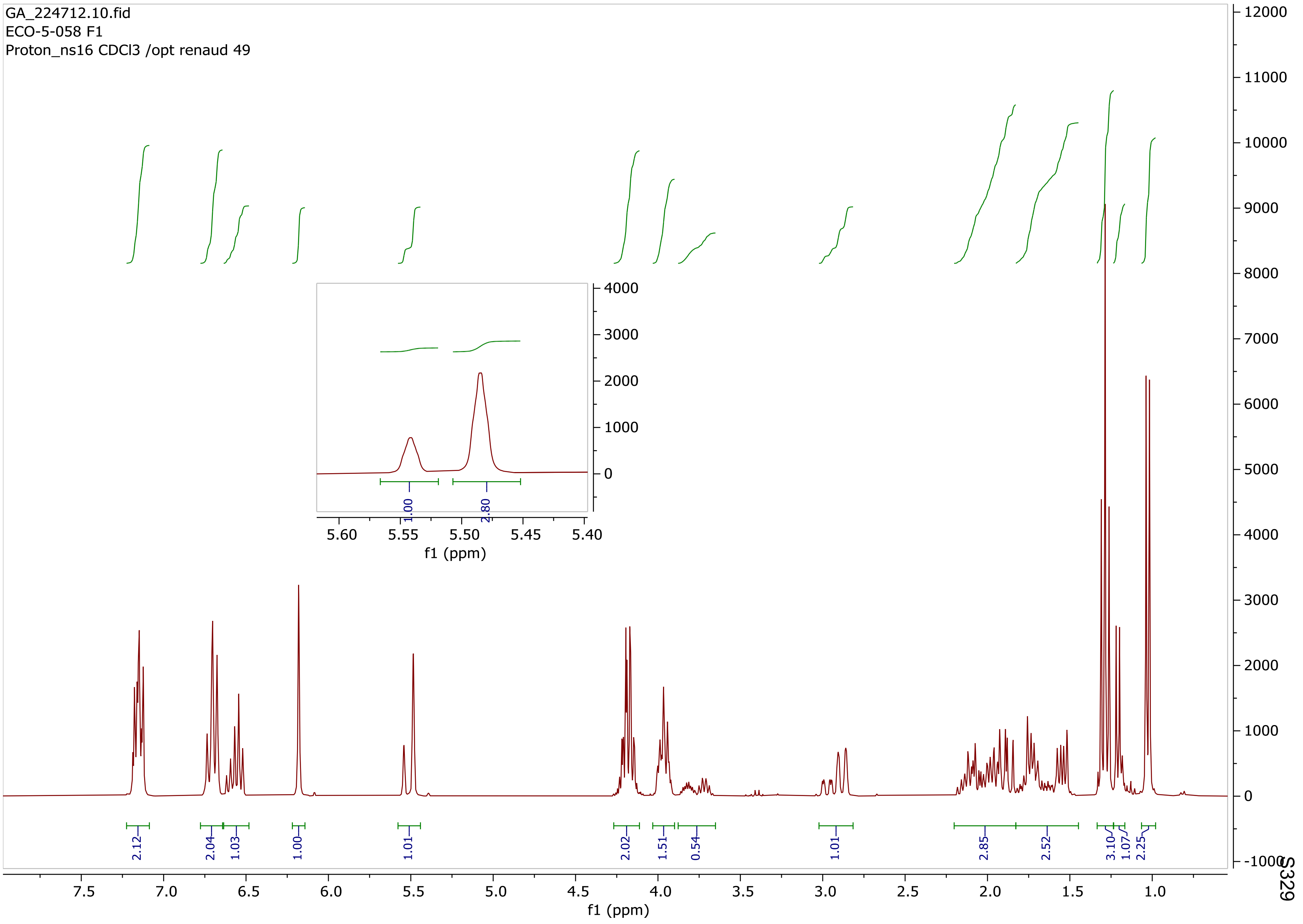

Ethyl 2-[(5-methyl-1-phenyl-pyrrolidin-2-yl)methyl]prop-2-enoate (***trans* 2f**)

^{1}H-NMR (400 MHz, $CDCl_3$)

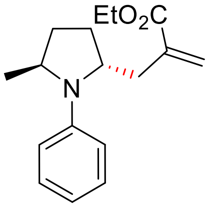

***trans* 2f**

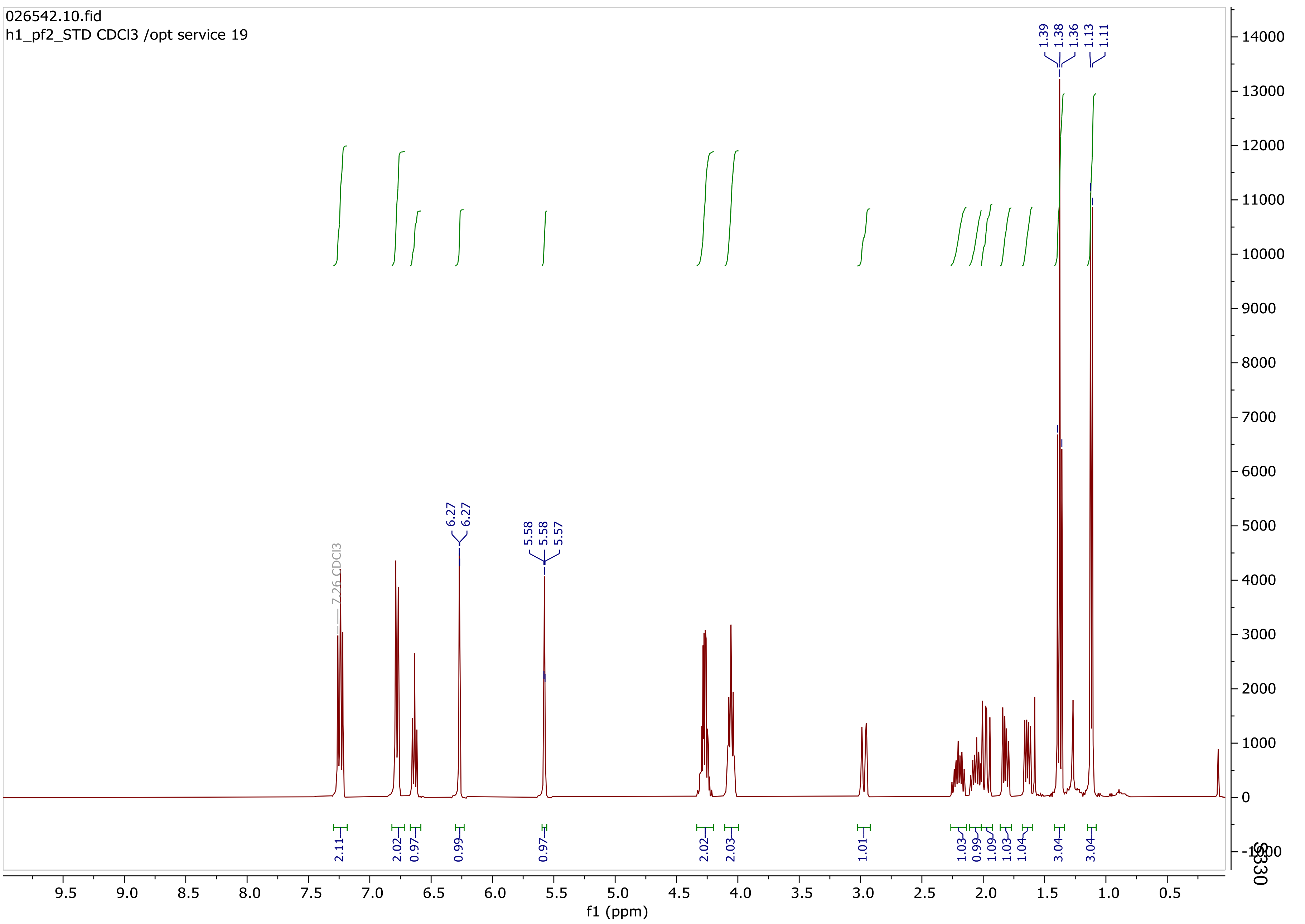

Ethyl 2-[(5-methyl-1-phenyl-pyrrolidin-2-yl)methyl]prop-2-enoate (***trans* 2f**)

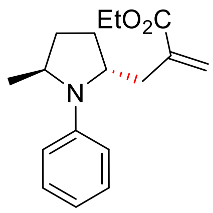

***trans* 2f**

^{1}H-NMR (400 MHz, $CDCl_3$)

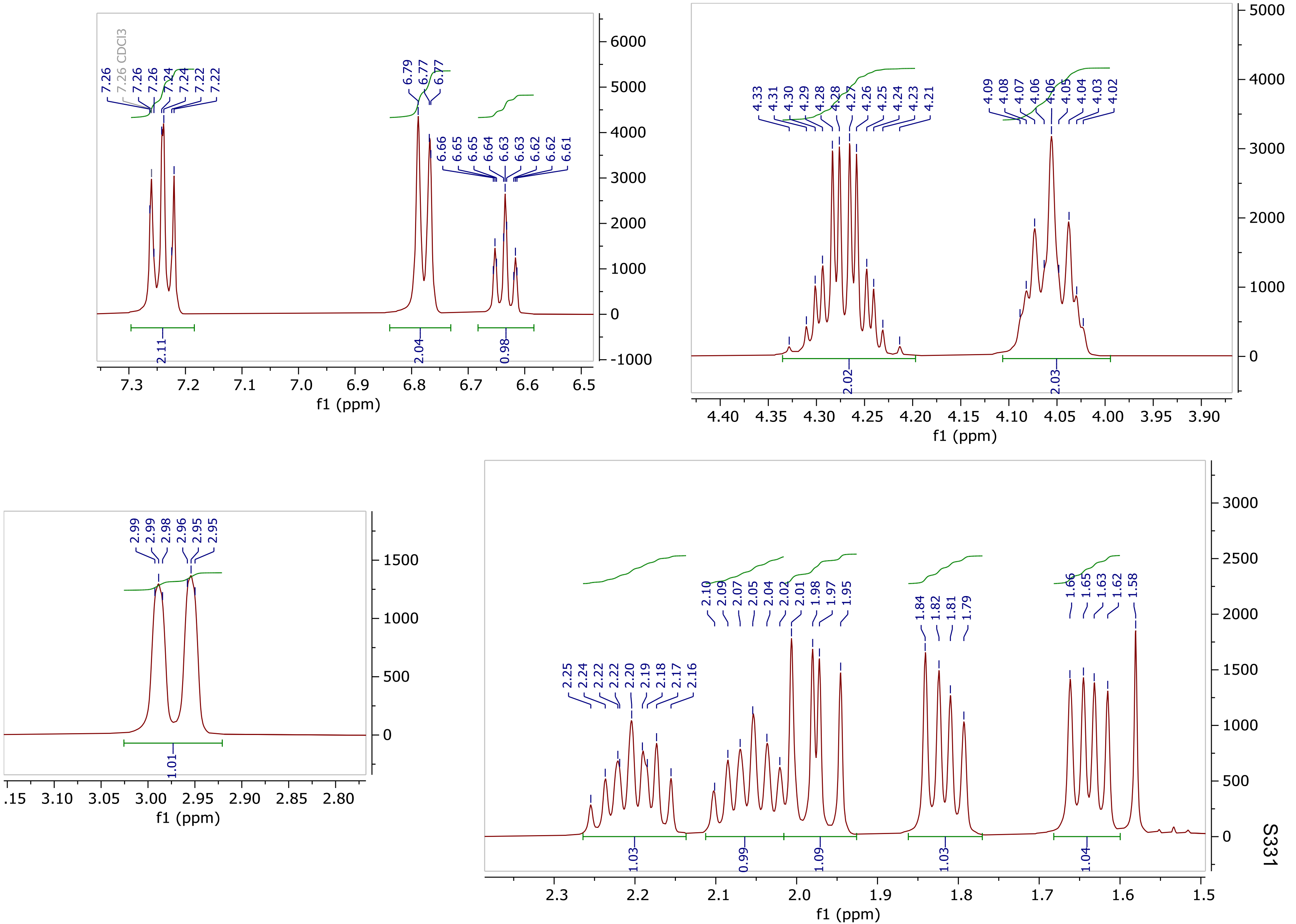

Ethyl 2-[(5-methyl-1-phenyl-pyrrolidin-2-yl)methyl]prop-2-enoate (***trans* 2f**)

^{13}C-NMR (101 MHz, $CDCl_3$)

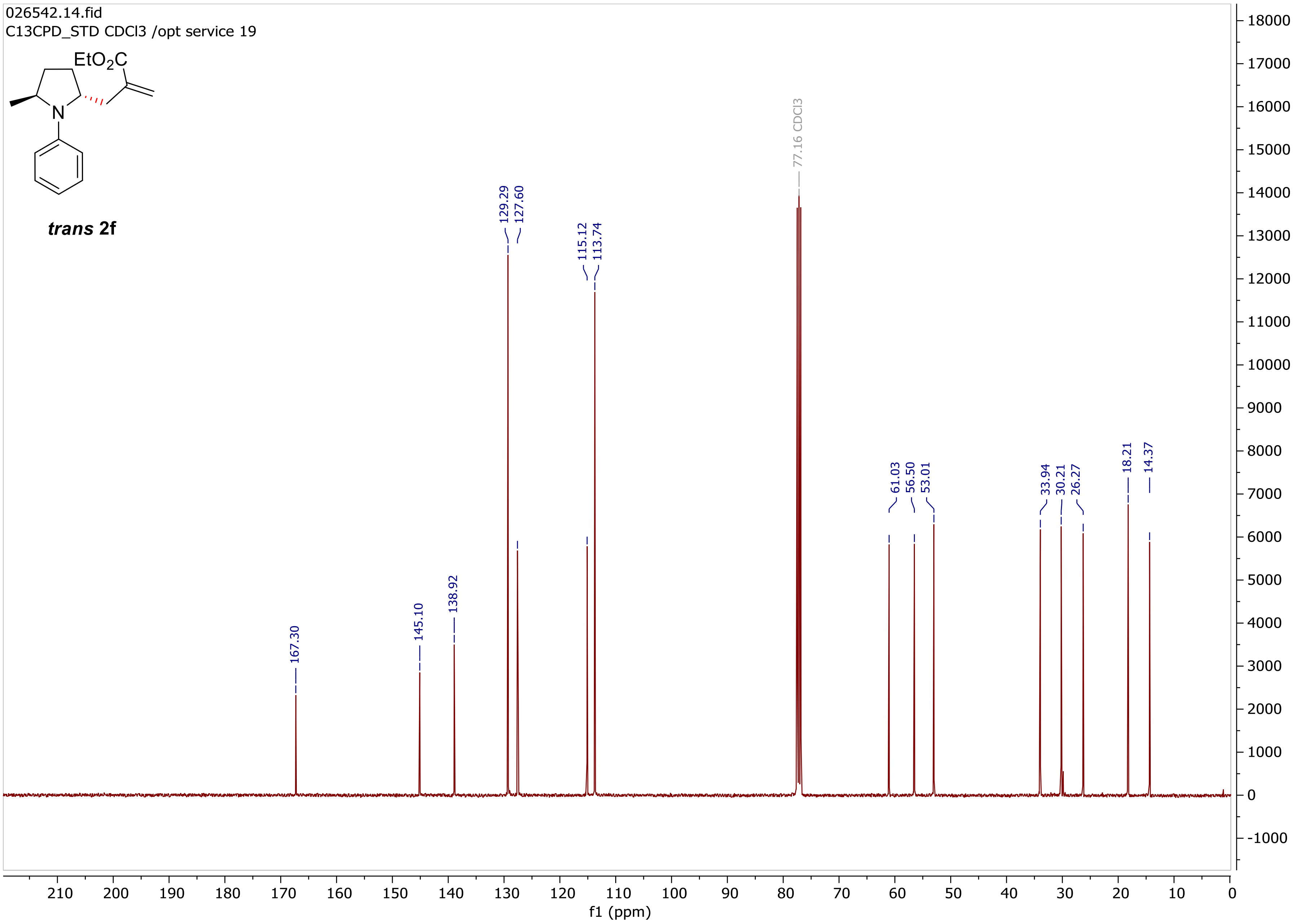

Ethyl 2-[(5-methyl-1-phenyl-pyrrolidin-2-yl)methyl]prop-2-enoate (***trans* 2f**)

^{13}C-NMR (101 MHz, $CDCl_3$)

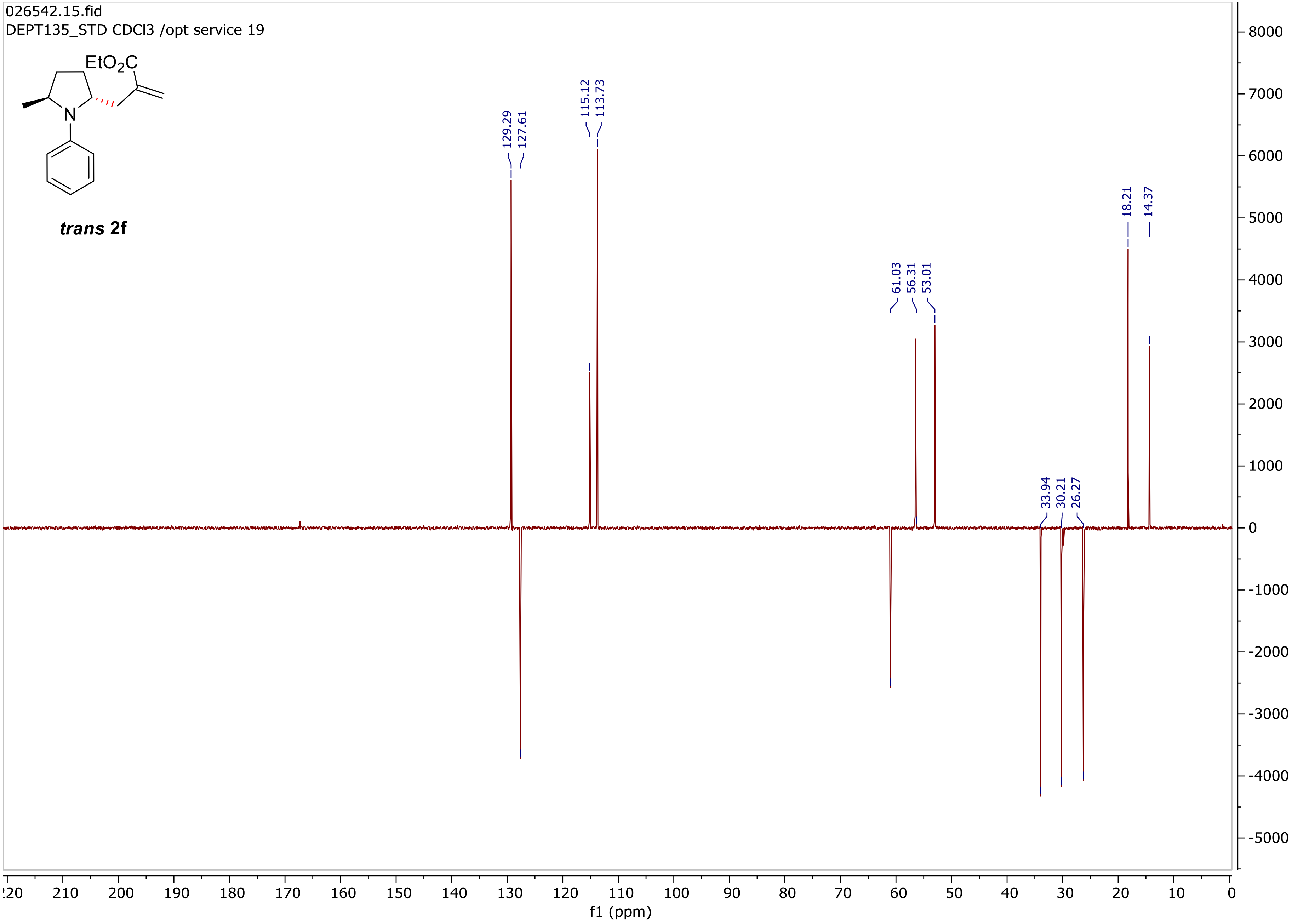

Ethyl 2-[(5-methyl-1-phenyl-pyrrolidin-2-yl)methyl]prop-2-enoate (***trans* 2f**)

^1H, ^1H-COSY NMR (400 MHz, $CDCl_3$)

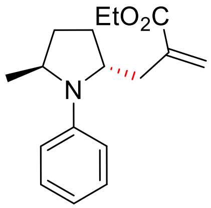

***trans* 2f**

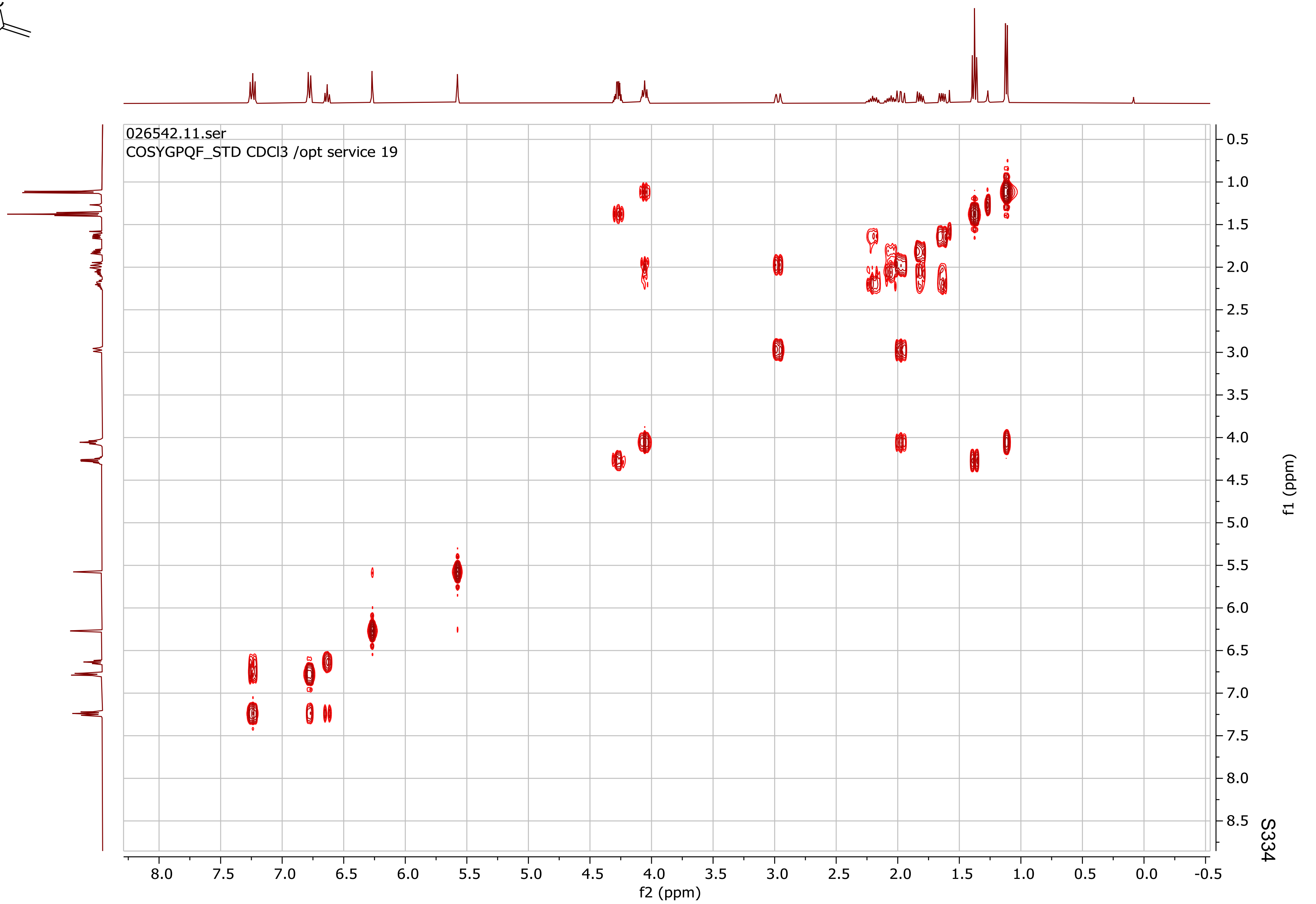

Ethyl 2-[(5-methyl-1-phenyl-pyrrolidin-2-yl)methyl]prop-2-enoate (***trans* 2f**)

^{1}H, ^{13}C-HSQC NMR (400 MHz, $CDCl_3$)

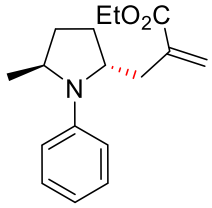

***trans* 2f**

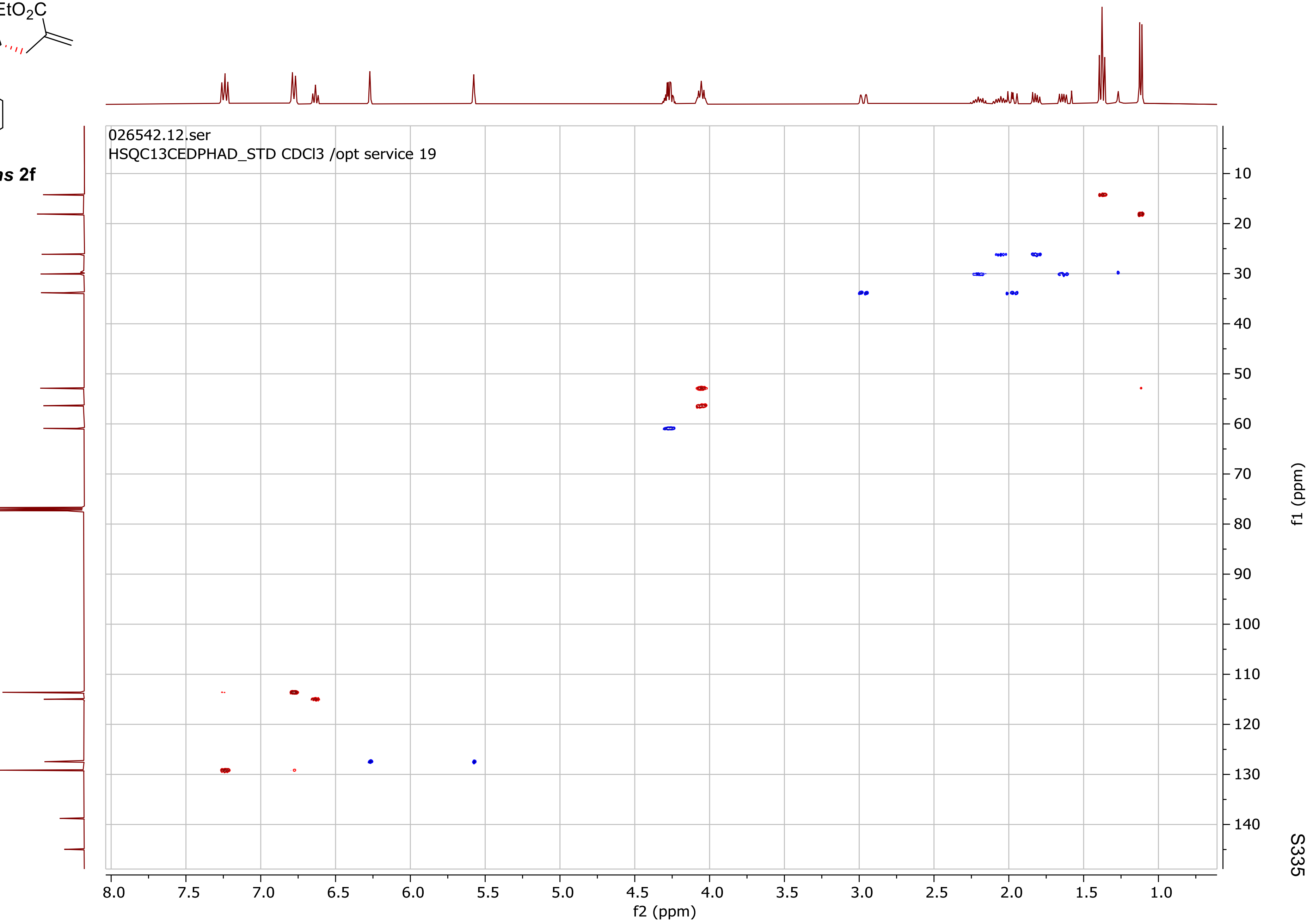

Ethyl 2-[(5-methyl-1-phenyl-pyrrolidin-2-yl)methyl]prop-2-enoate (***trans* 2f**)

^{1}H, ^{13}C-HMBC NMR (400 MHz, $CDCl_3$)

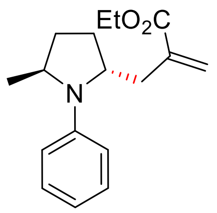

***trans* 2f**

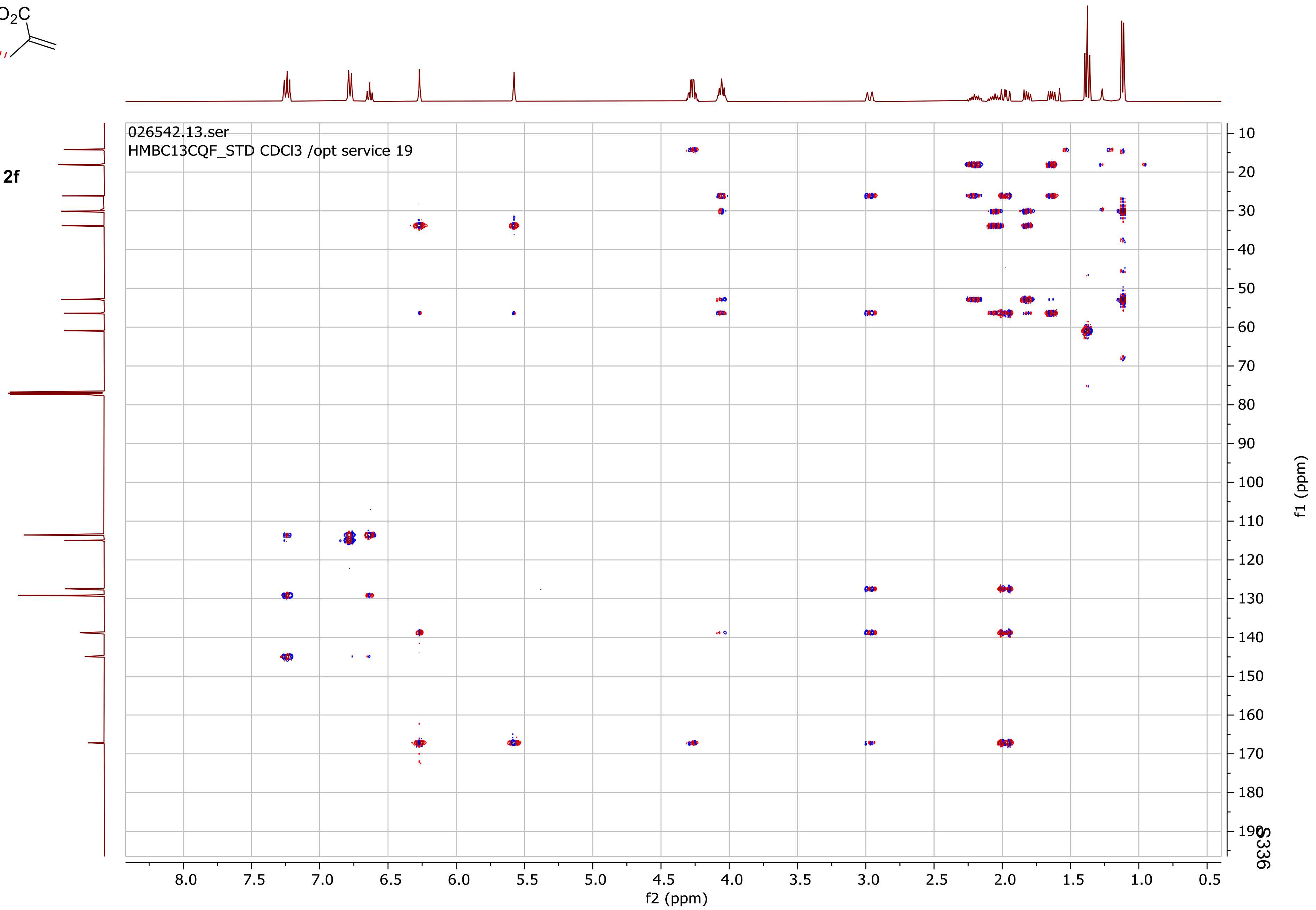

Ethyl 2-[(5-methyl-1-phenyl-pyrrolidin-2-yl)methyl]prop-2-enoate (***trans* 2f**)

FT-IR, ATR-diamond

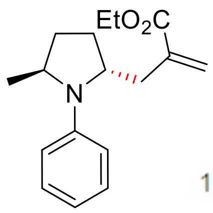

***trans* 2f**

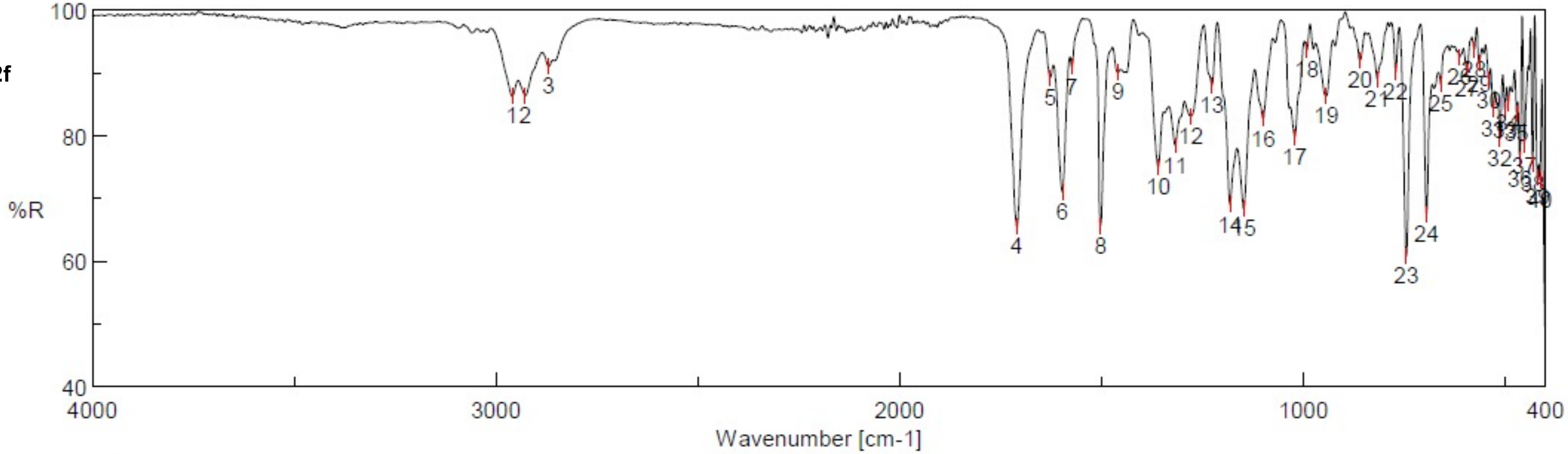

[Result of Peak Picking]

No.	Position	Intensity	No.	Position	Intensity	No.	Position	Intensity
1	2960.2	86.2774	2	2927.41	86.3788	3	2869.56	91.0113
4	1709.59	65.4651	5	1626.66	89.2188	6	1596.77	70.9945
7	1573.63	91.0002	8	1502.28	65.6236	9	1458.89	90.0558
10	1359.57	75.0471	11	1317.14	78.4321	12	1278.57	83.1245
13	1226.5	88.0306	14	1181.19	68.99	15	1147.44	68.3054
16	1099.23	82.6962	17	1021.12	79.7767	18	991.232	93.6375
19	943.02	86.3085	20	859.132	92.0179	21	816.706	89.0631
22	771.387	90.122	23	744.388	60.7531	24	694.248	67.437
25	659.536	88.2256	26	612.288	92.5142	27	594.932	90.6062
28	576.612	93.7655	29	564.077	91.4708	30	539.971	88.8468
31	529.364	84.2043	32	512.972	79.5594	33	500.437	84.0938
34	492.723	85.1987	35	471.51	83.5533	36	463.796	76.1812
37	452.225	78.4198	38	431.977	75.5349	39	419.442	73.3339
40	413.656	72.8658						

Ethyl 2-[(5-methyl-1-phenyl-pyrrolidin-2-yl)methyl]prop-2-enoat (***cis* 2f**)

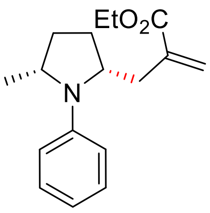

***cis* 2f**

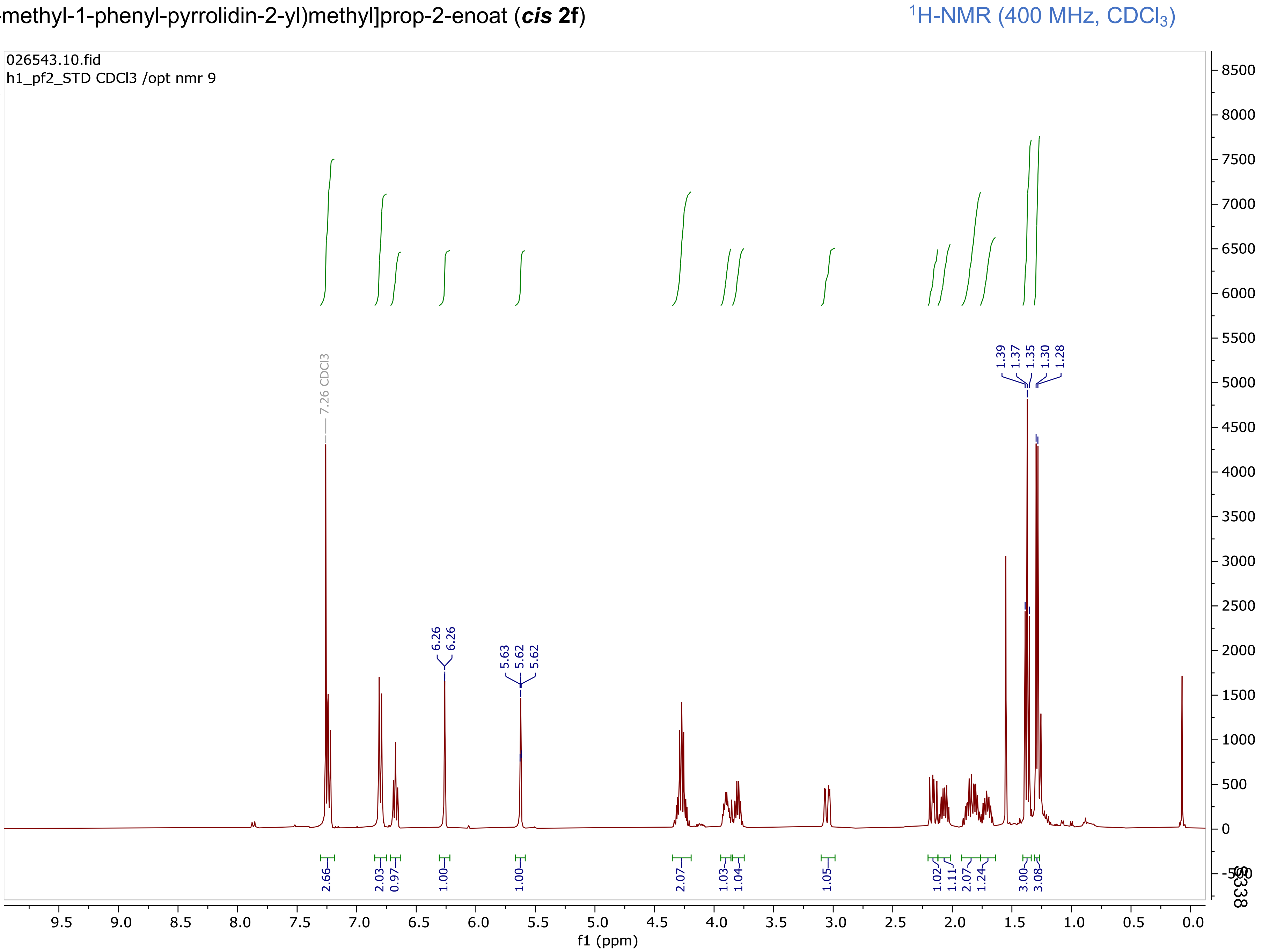

Ethyl 2-[(5-methyl-1-phenyl-pyrrolidin-2-yl)methyl]prop-2-enoate (*cis* 2f)

^{1}H-NMR (400 MHz, $CDCl_3$)

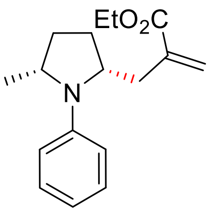

***cis* 2f**

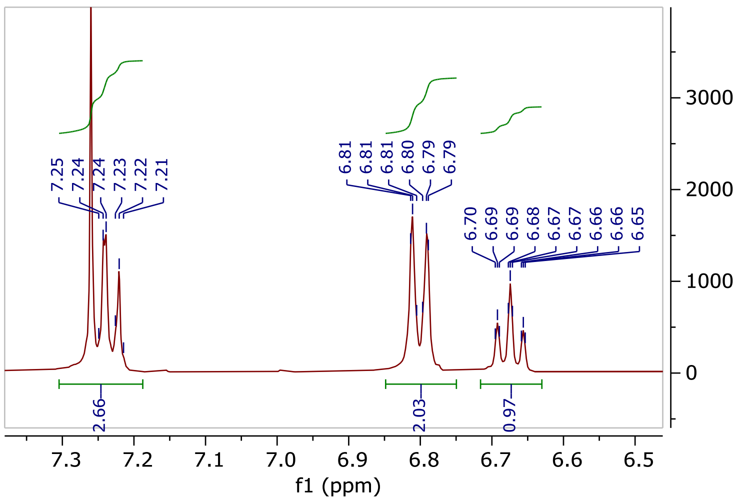

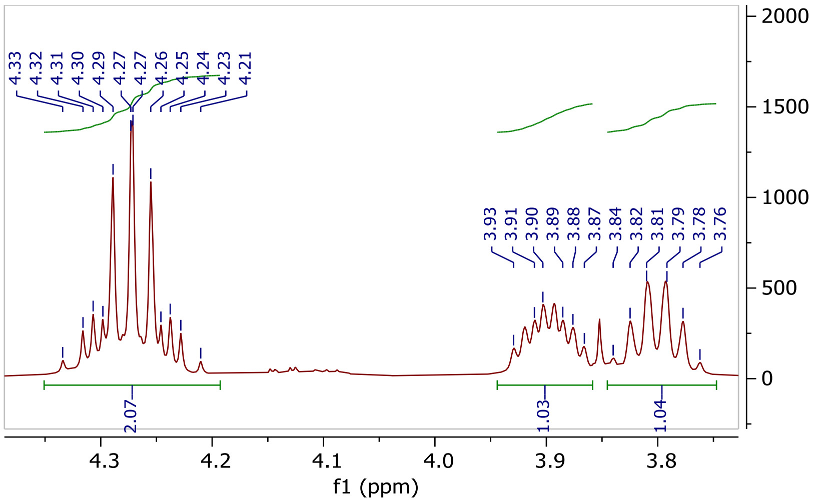

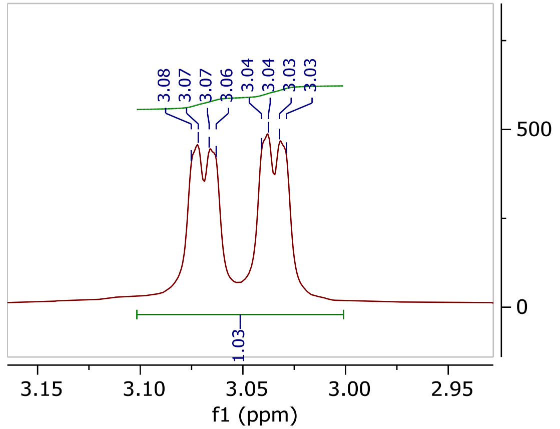

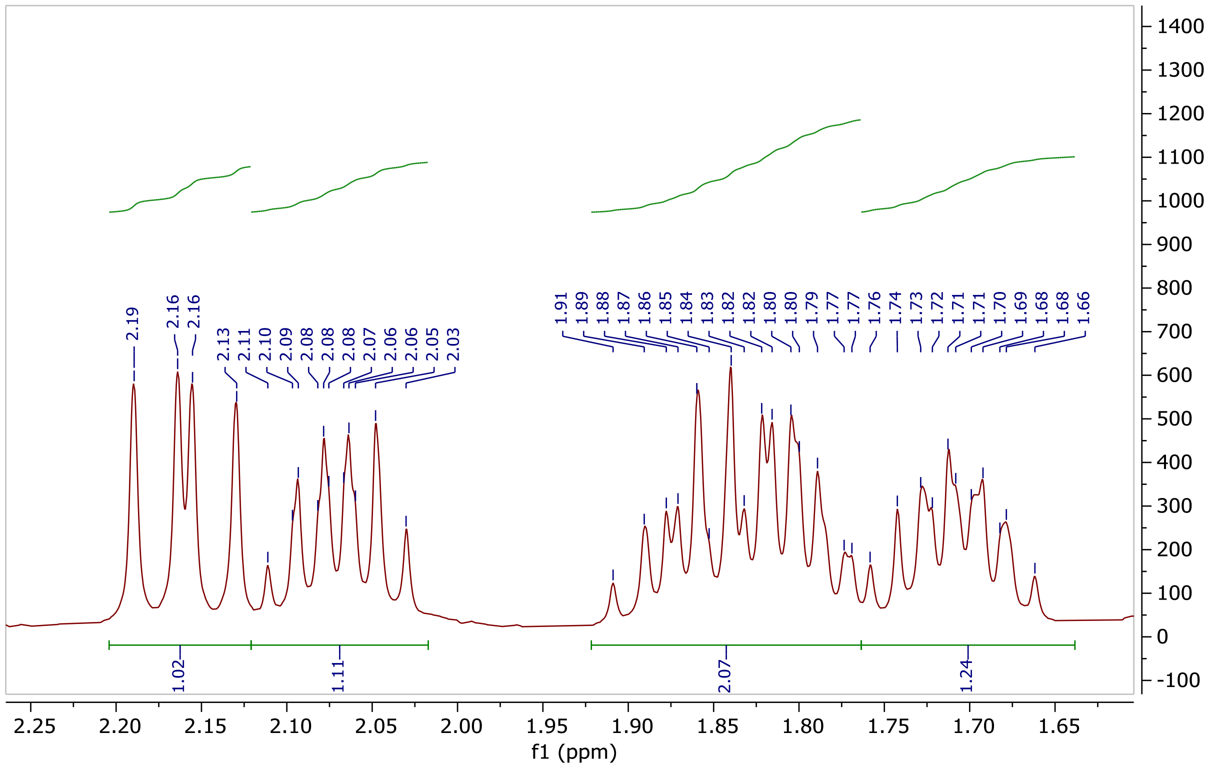

Ethyl 2-[(5-methyl-1-phenyl-pyrrolidin-2-yl)methyl]prop-2-enoate (***cis* 2f**)

^{13}C-NMR (101 MHz, $CDCl_3$)

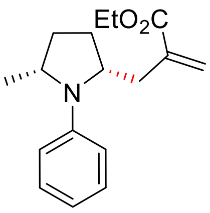

***cis* 2f**

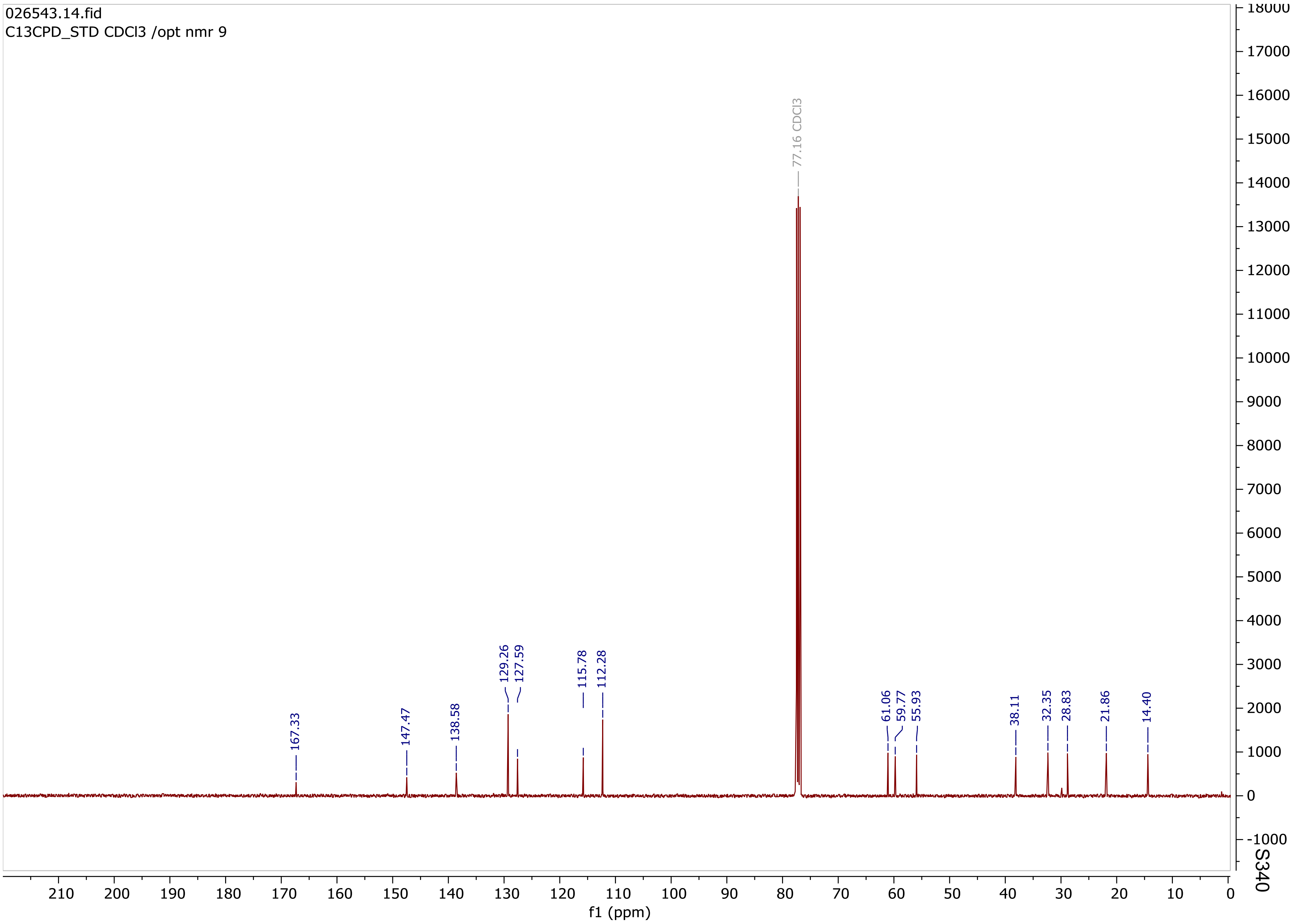

Ethyl 2-[(5-methyl-1-phenyl-pyrrolidin-2-yl)methyl]prop-2-enoate (***cis* 2f**)

^{13}C-NMR (101 MHz, $CDCl_3$)

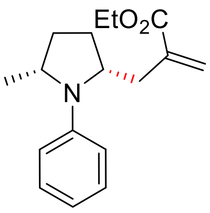

***cis* 2f**

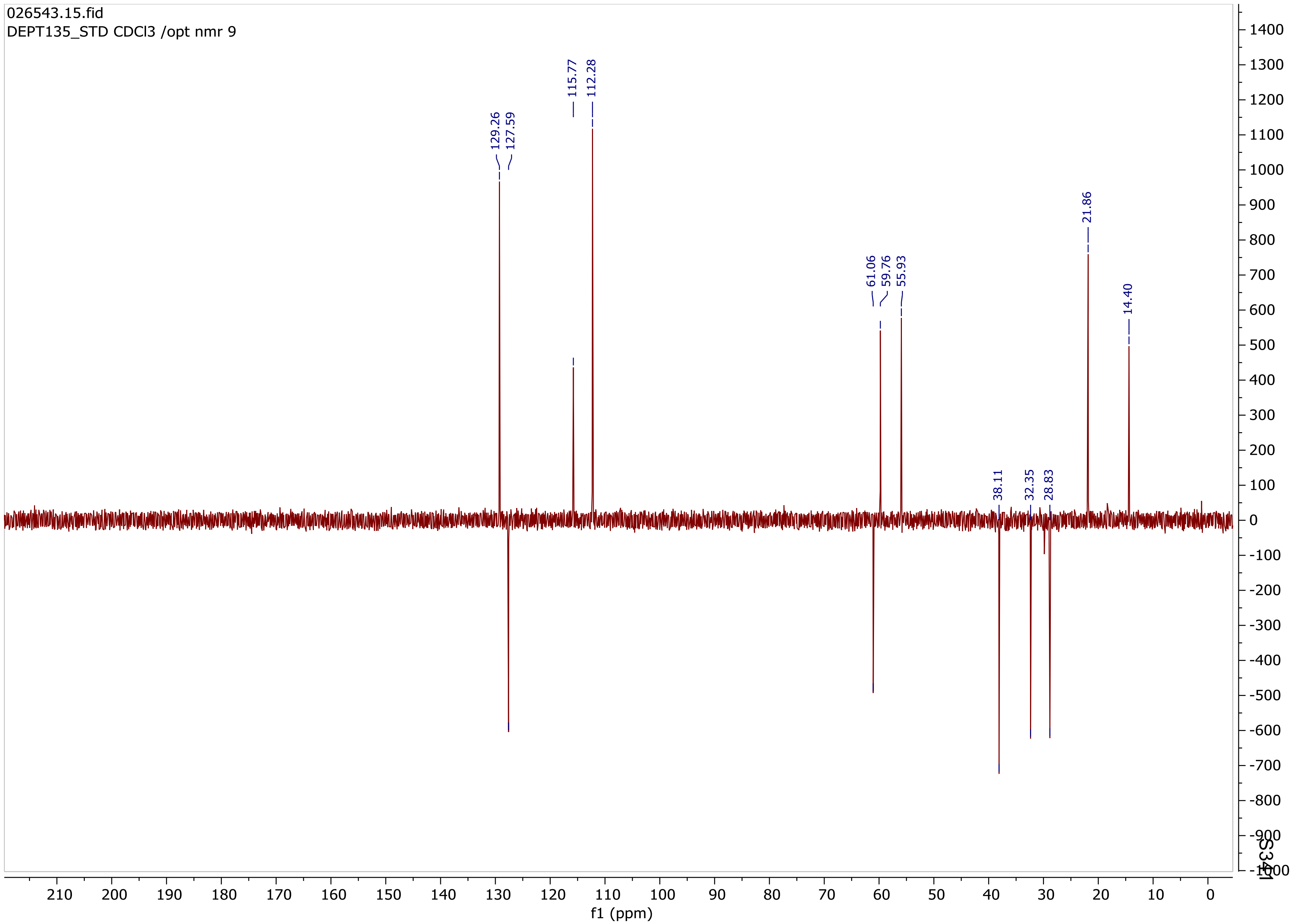

Ethyl 2-[(5-methyl-1-phenyl-pyrrolidin-2-yl)methyl]prop-2-enoate (***cis* 2f**)

^1H, ^1H-COSY NMR (400 MHz, $CDCl_3$)

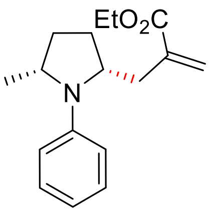

***cis* 2f**

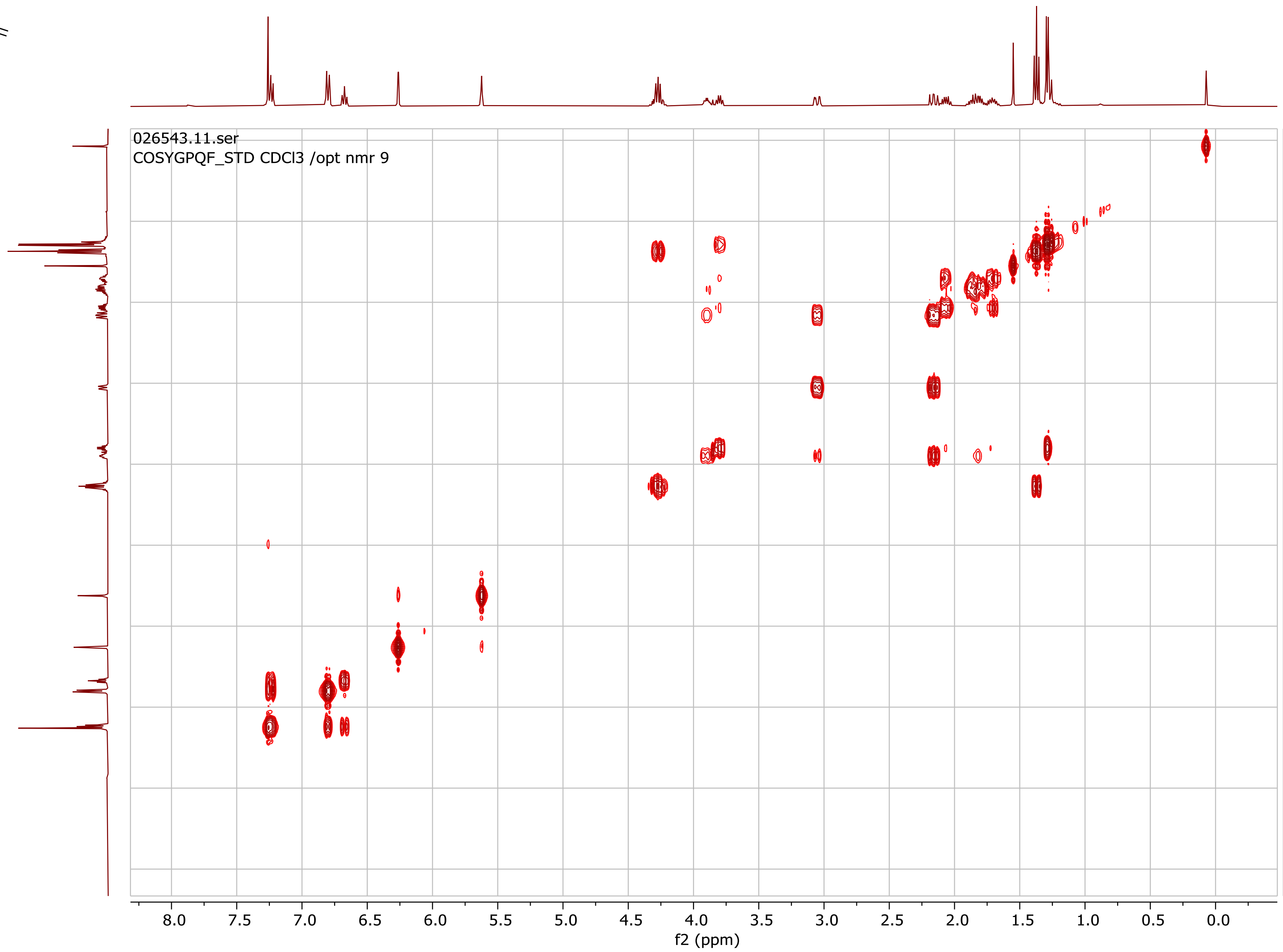

Ethyl 2-[(5-methyl-1-phenyl-pyrrolidin-2-yl)methyl]prop-2-enoate (***cis* 2f**)

^{1}H, ^{13}C-HSQC NMR (400 MHz, $CDCl_3$)

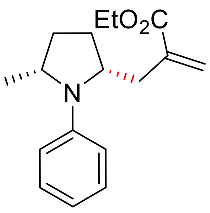

***cis* 2f**

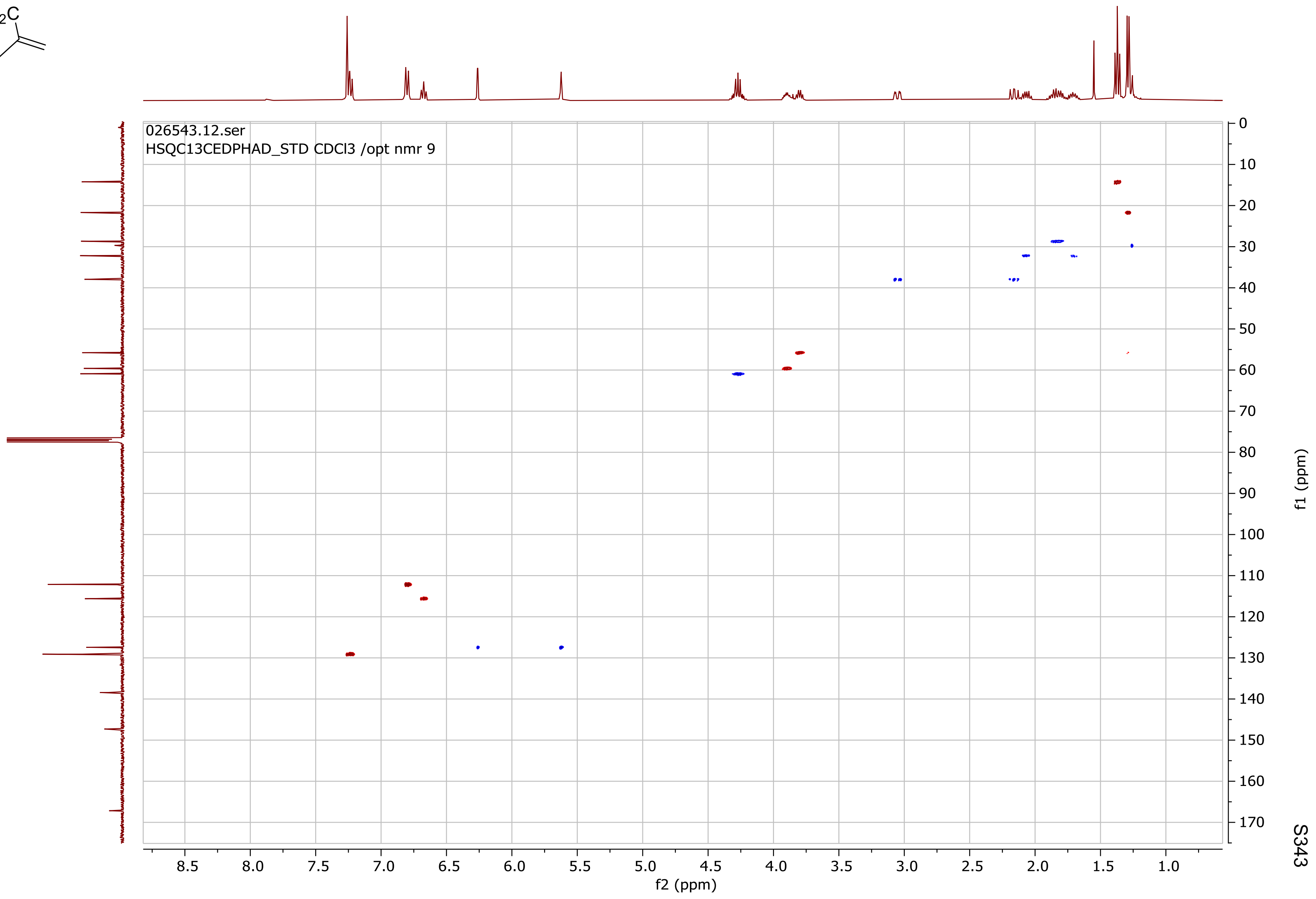

Ethyl 2-[(5-methyl-1-phenyl-pyrrolidin-2-yl)methyl]prop-2-enoate (***cis* 2f**)

^{1}H, ^{13}C-HMBC NMR (400 MHz, $CDCl_3$)

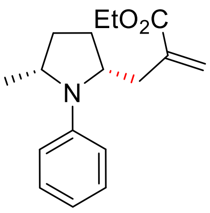

***cis* 2f**

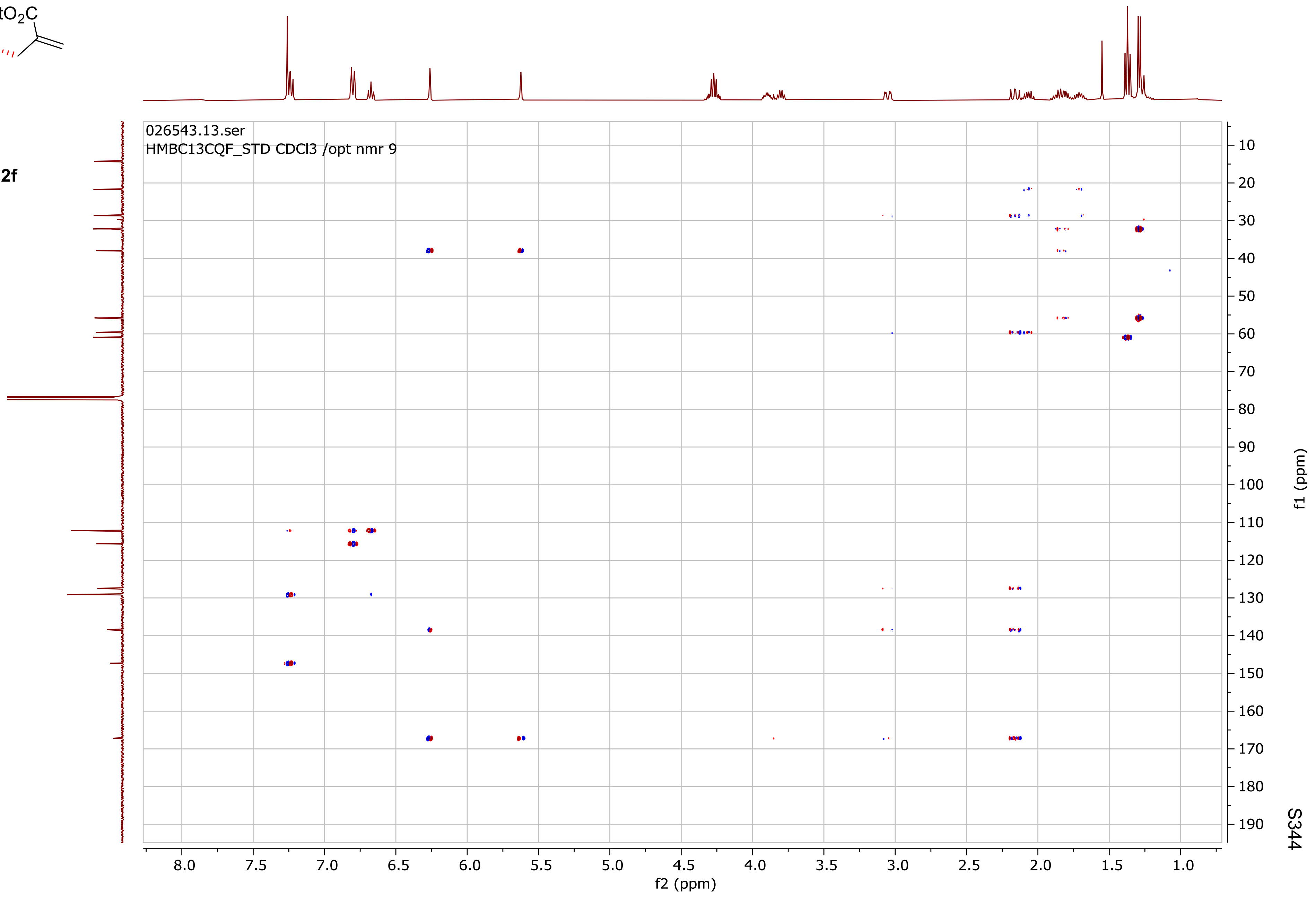

Ethyl 2-[(5-methyl-1-phenyl-pyrrolidin-2-yl)methyl]prop-2-enoate (***cis* 2f**)

FT-IR, ATR-diamond

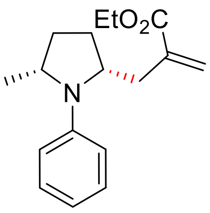

***cis* 2f**

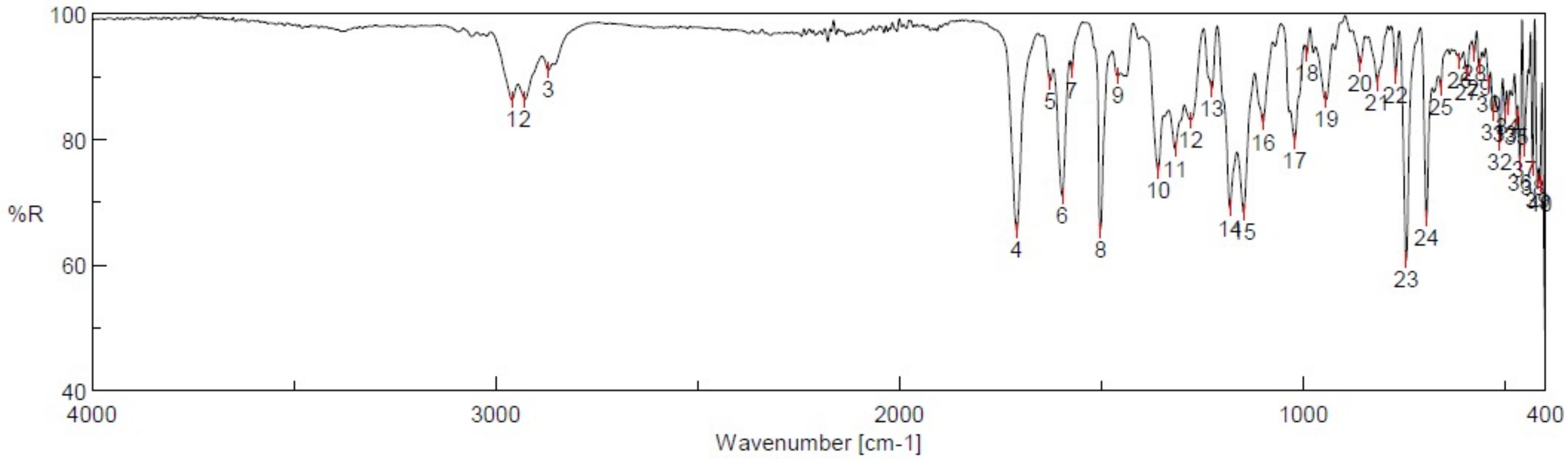

[Result of Peak Picking]

No.	Position	Intensity	No.	Position	Intensity	No.	Position	Intensity
1	2960.2	86.2774	2	2927.41	86.3788	3	2869.56	91.0113
4	1709.59	65.4651	5	1626.66	89.2188	6	1596.77	70.9945
7	1573.63	91.0002	8	1502.28	65.6236	9	1458.89	90.0558
10	1359.57	75.0471	11	1317.14	78.4321	12	1278.57	83.1245
13	1226.5	88.0306	14	1181.19	68.99	15	1147.44	68.3054
16	1099.23	82.6962	17	1021.12	79.7767	18	991.232	93.6375
19	943.02	86.3085	20	859.132	92.0179	21	816.706	89.0631
22	771.387	90.122	23	744.388	60.7531	24	694.248	67.437
25	659.536	88.2256	26	612.288	92.5142	27	594.932	90.6062
28	576.612	93.7655	29	564.077	91.4708	30	539.971	88.8468
31	529.364	84.2043	32	512.972	79.5594	33	500.437	84.0938
34	492.723	85.1987	35	471.51	83.5533	36	463.796	76.1812
37	452.225	78.4198	38	431.977	75.5349	39	419.442	73.3339
40	413.656	72.8658						

Ethyl 10-phenyl-10-azabicyclo[4.3.1]decane-8-carboxylate (**7n**)

^{1}H-NMR (300 MHz, $CDCl_3$)

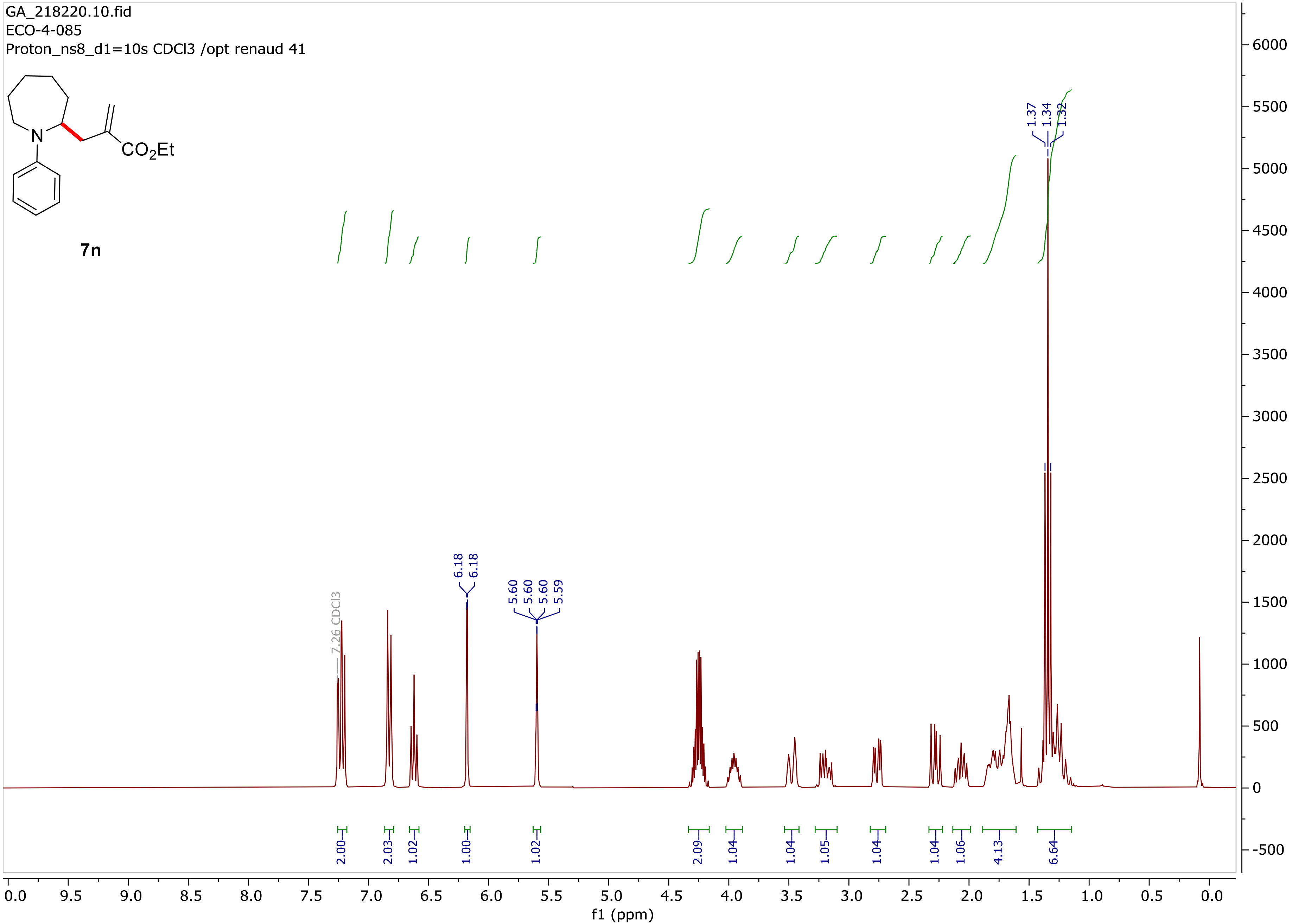

Ethyl 10-phenyl-10-azabicyclo[4.3.1]decane-8-carboxylate (**7n**)

^{1}H-NMR (300 MHz, $CDCl_3$)

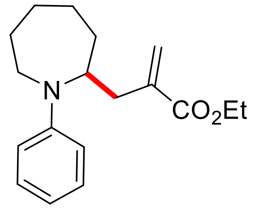

7n

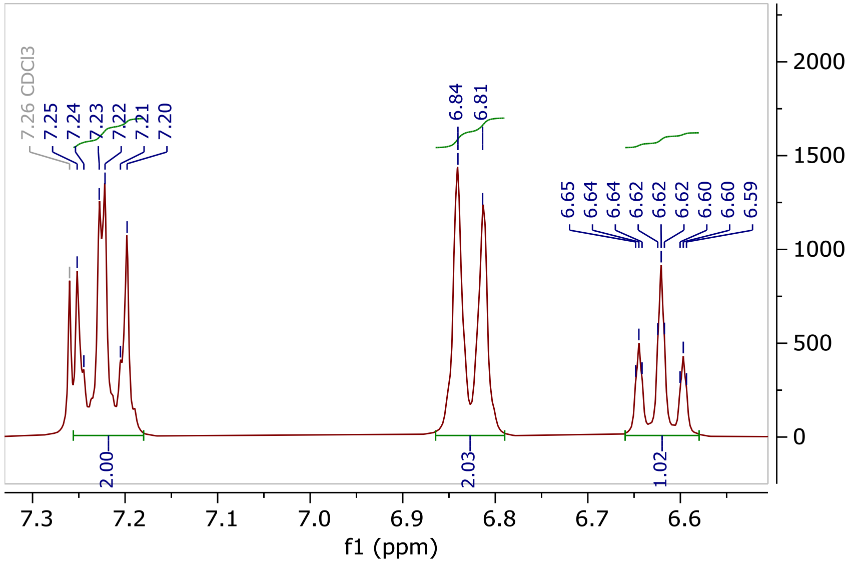

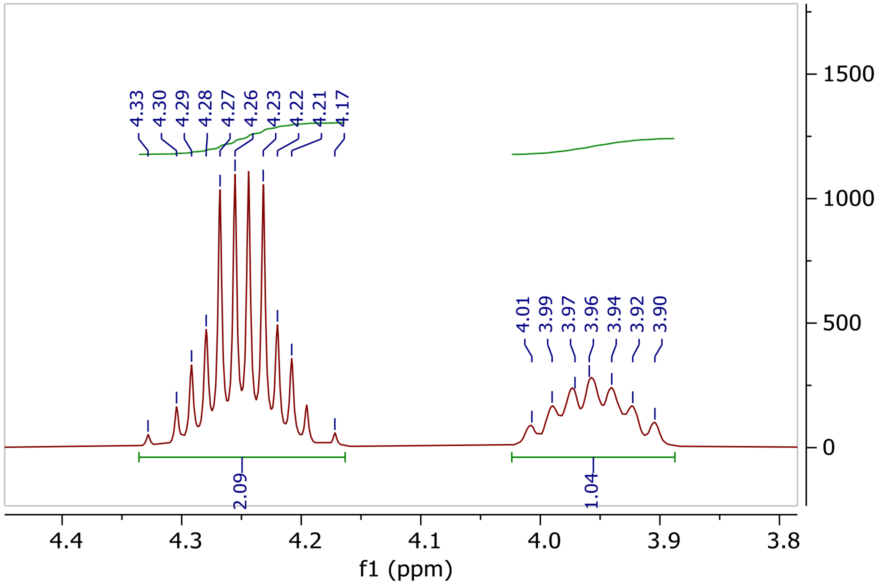

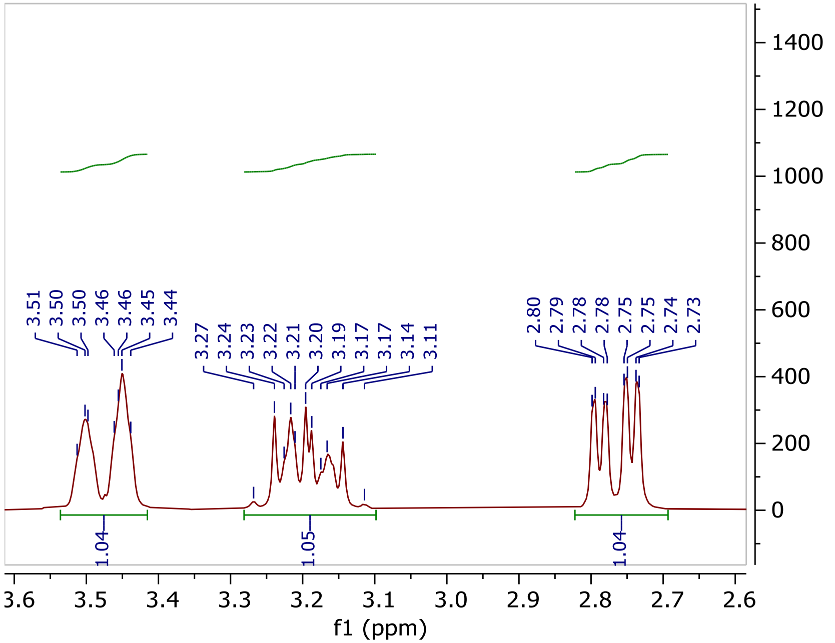

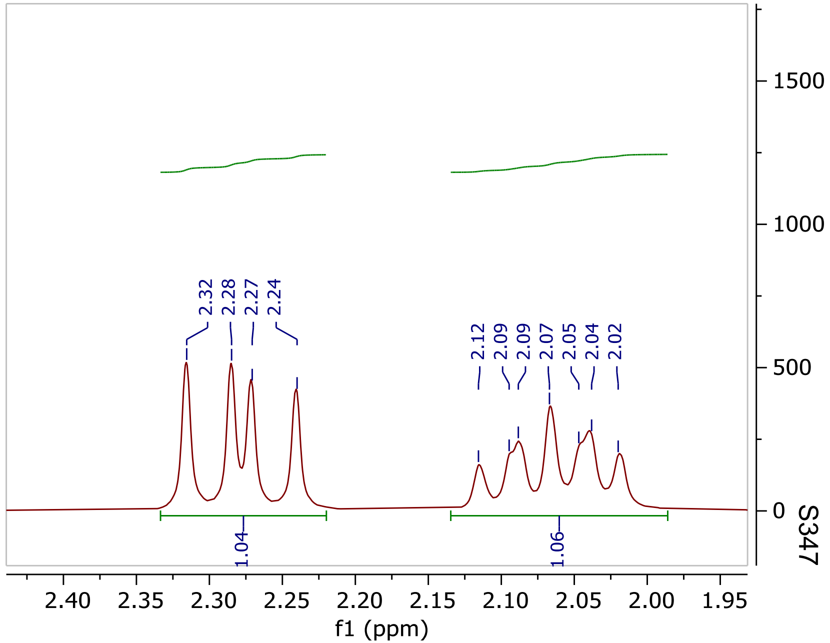

Ethyl 10-phenyl-10-azabicyclo[4.3.1]decane-8-carboxylate (**7n**)

^{1}H-NMR (300 MHz, $CDCl_3$)

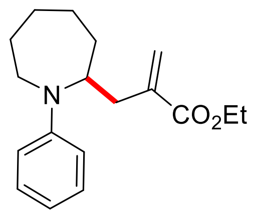

7n

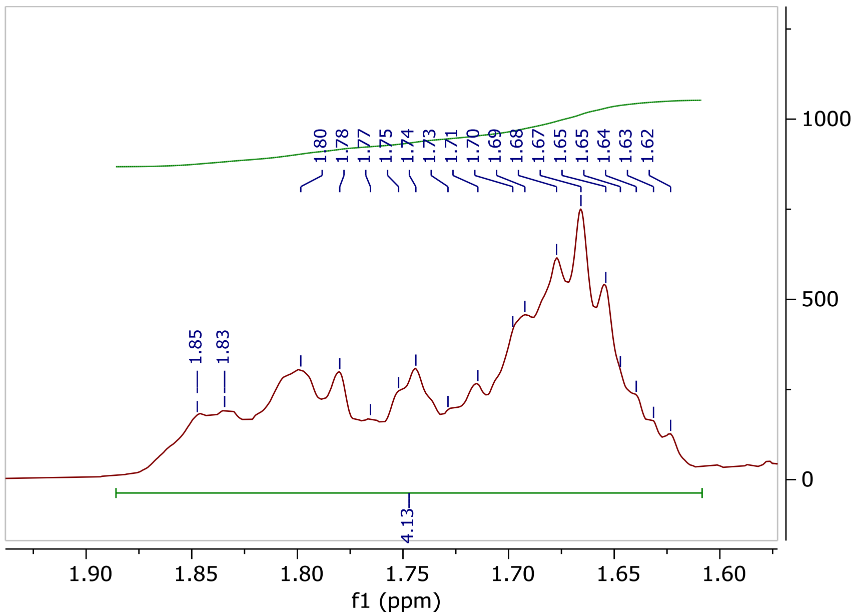

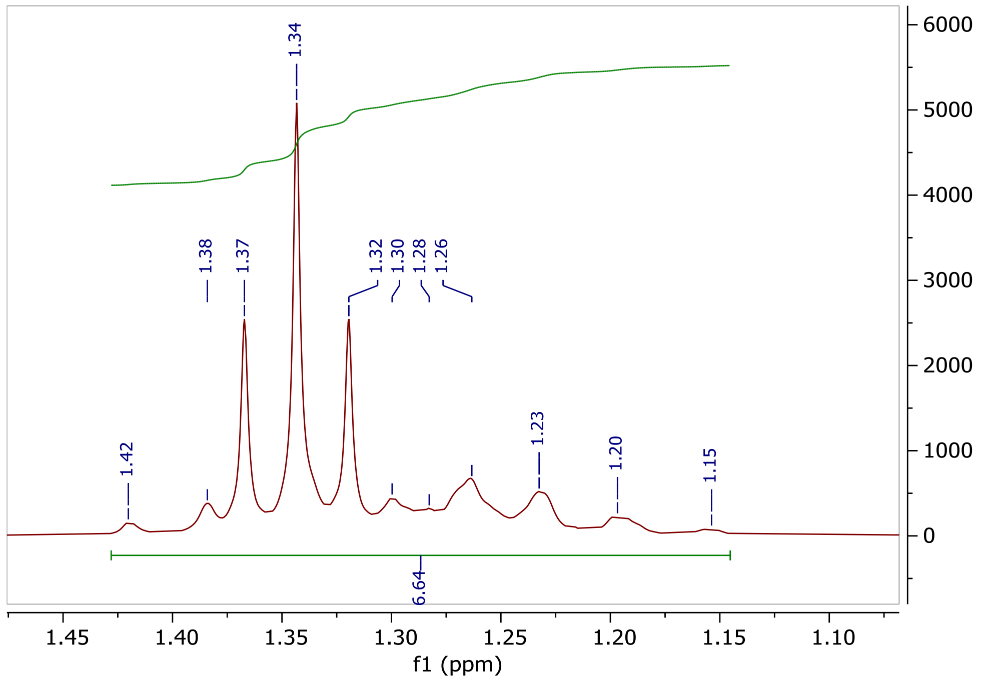

Ethyl 10-phenyl-10-azabicyclo[4.3.1]decane-8-carboxylate (**7n**)

^{13}C-NMR (101 MHz, $CDCl_3$)

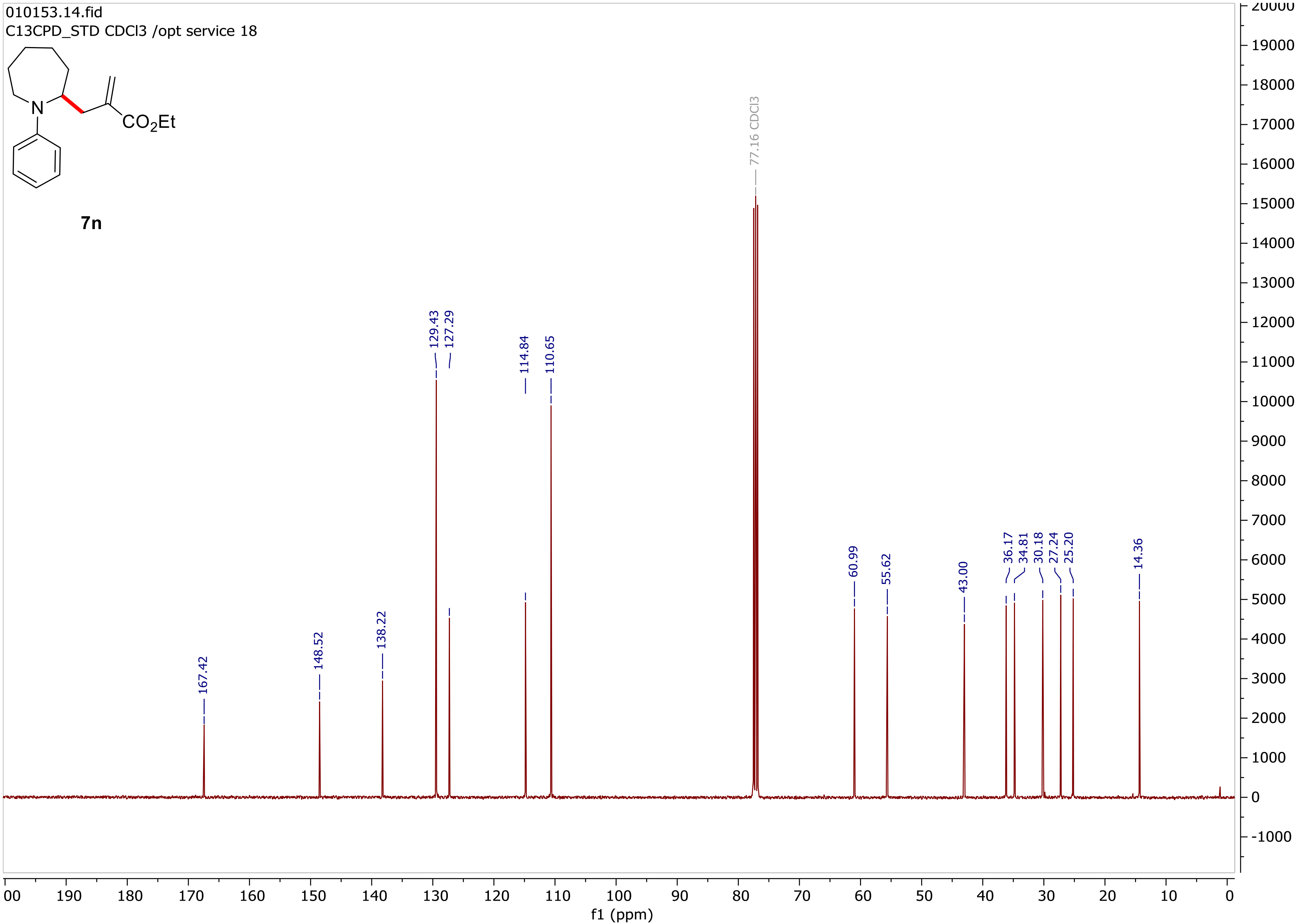

Ethyl 10-phenyl-10-azabicyclo[4.3.1]decane-8-carboxylate (**7n**)

^{13}C-NMR (101 MHz, $CDCl_3$)

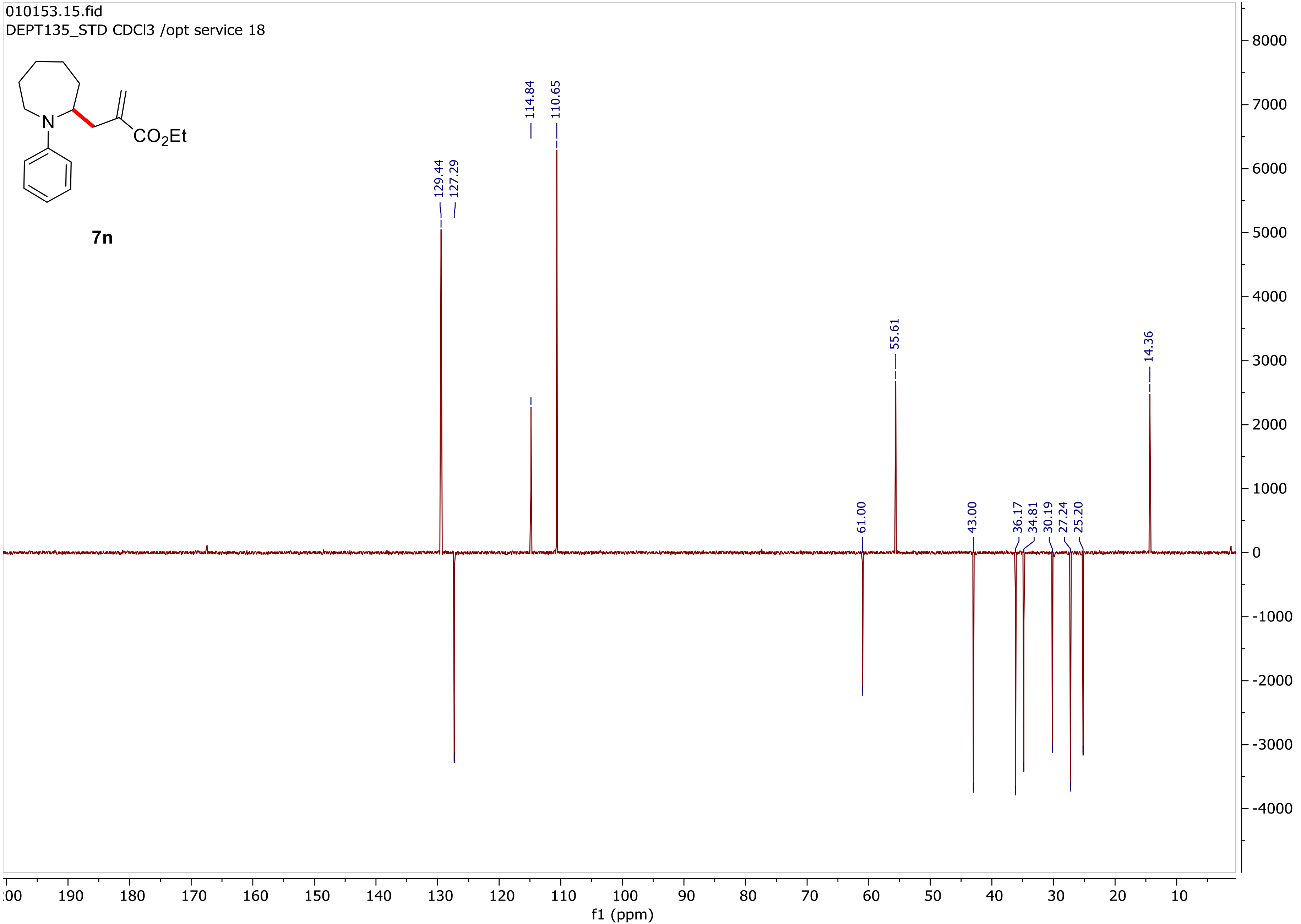

Ethyl 10-phenyl-10-azabicyclo[4.3.1]decane-8-carboxylate (**7n**)

^{1}H, ^{1}H-COSY NMR (400 MHz, $CDCl_3$)

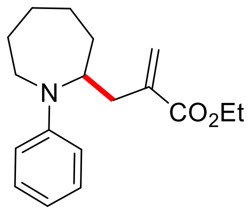

7n

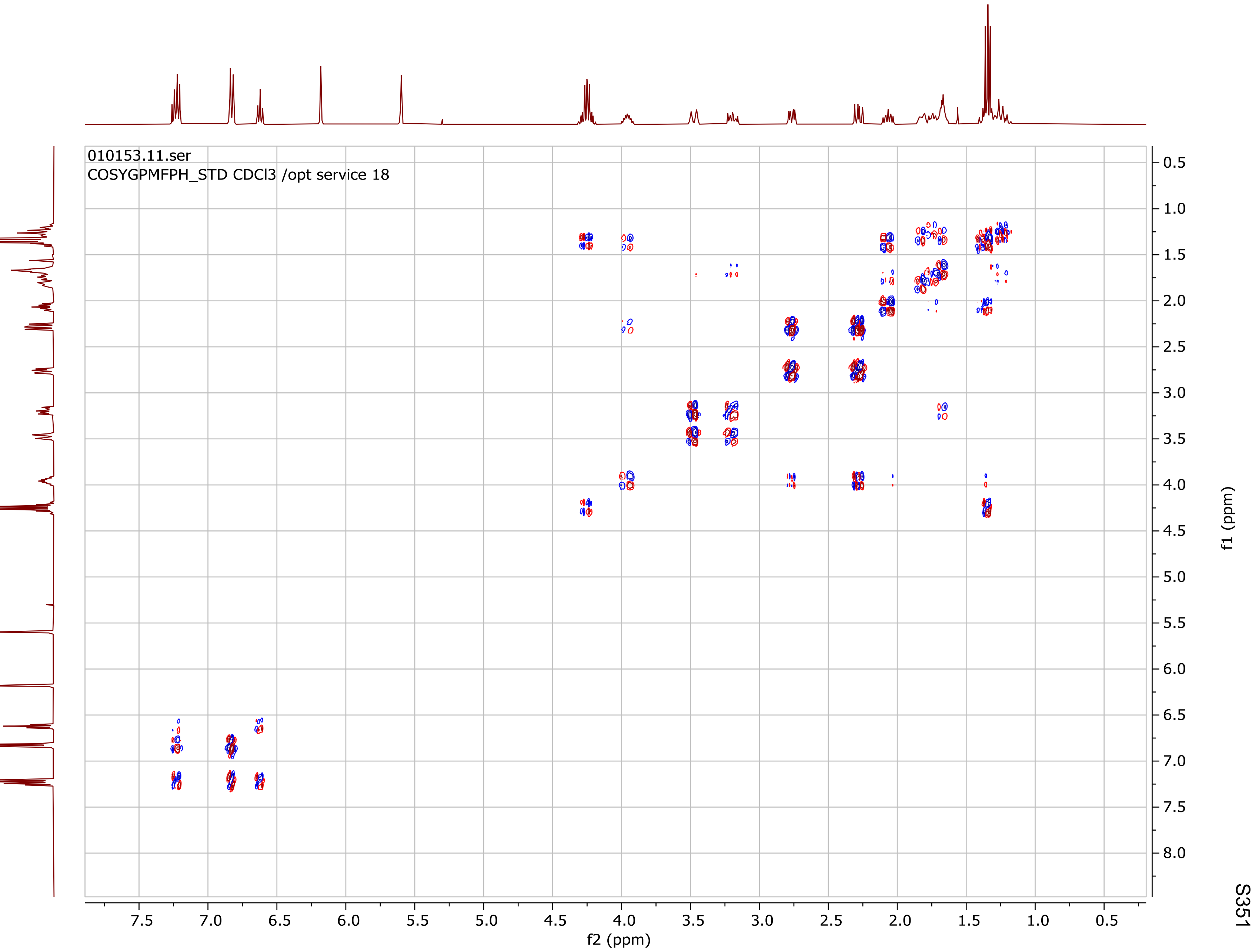

Ethyl 10-phenyl-10-azabicyclo[4.3.1]decane-8-carboxylate (**7n**)

^1H, ^{13}C-HSQC NMR (400 MHz, $CDCl_3$)

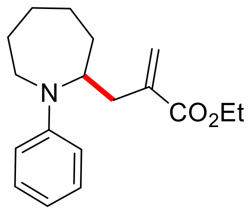

7n

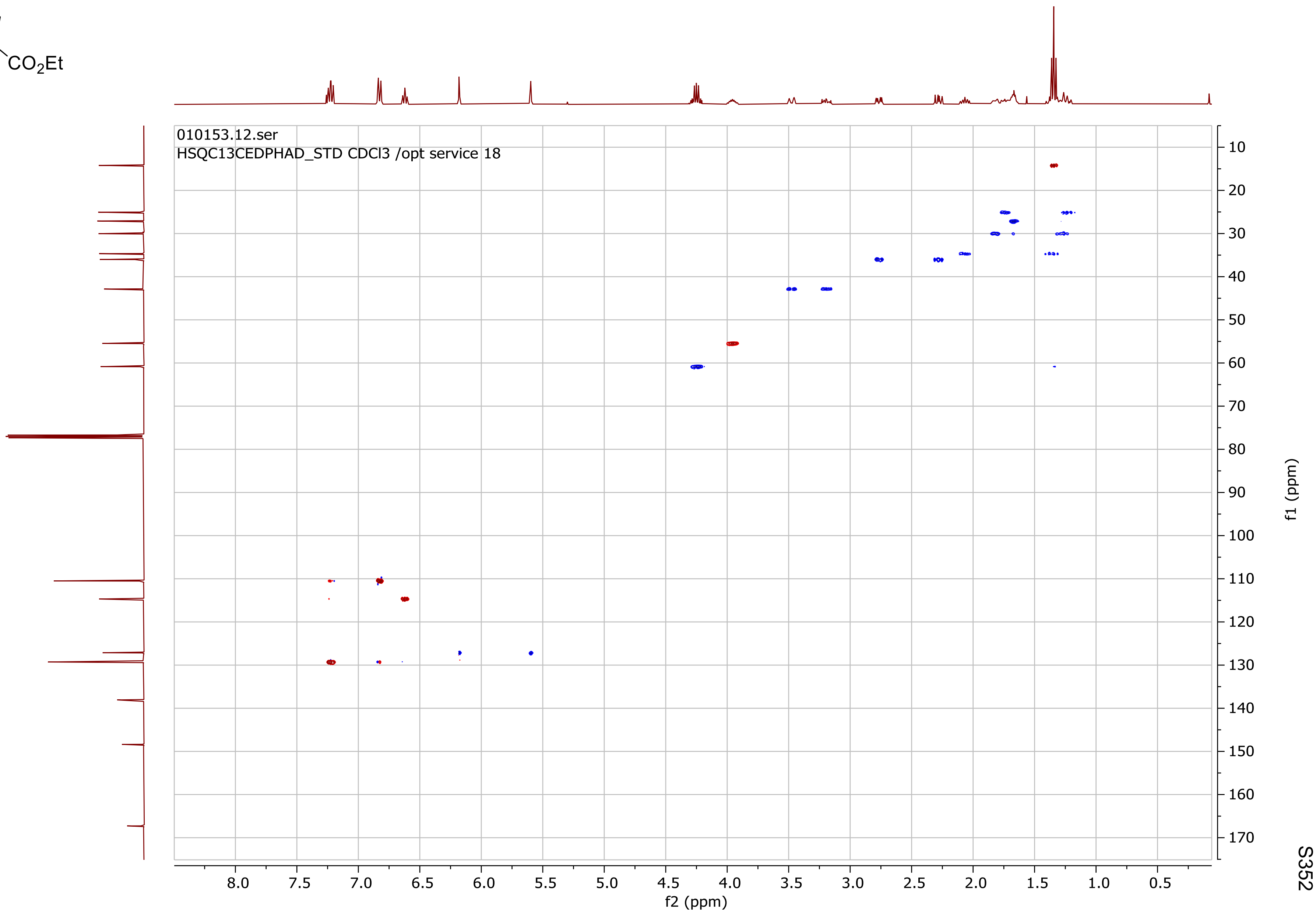

Ethyl 10-phenyl-10-azabicyclo[4.3.1]decane-8-carboxylate (**7n**)

^{1}H, ^{13}C-HMBC NMR (400 MHz, $CDCl_3$)

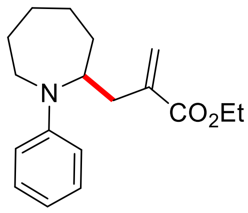

7n

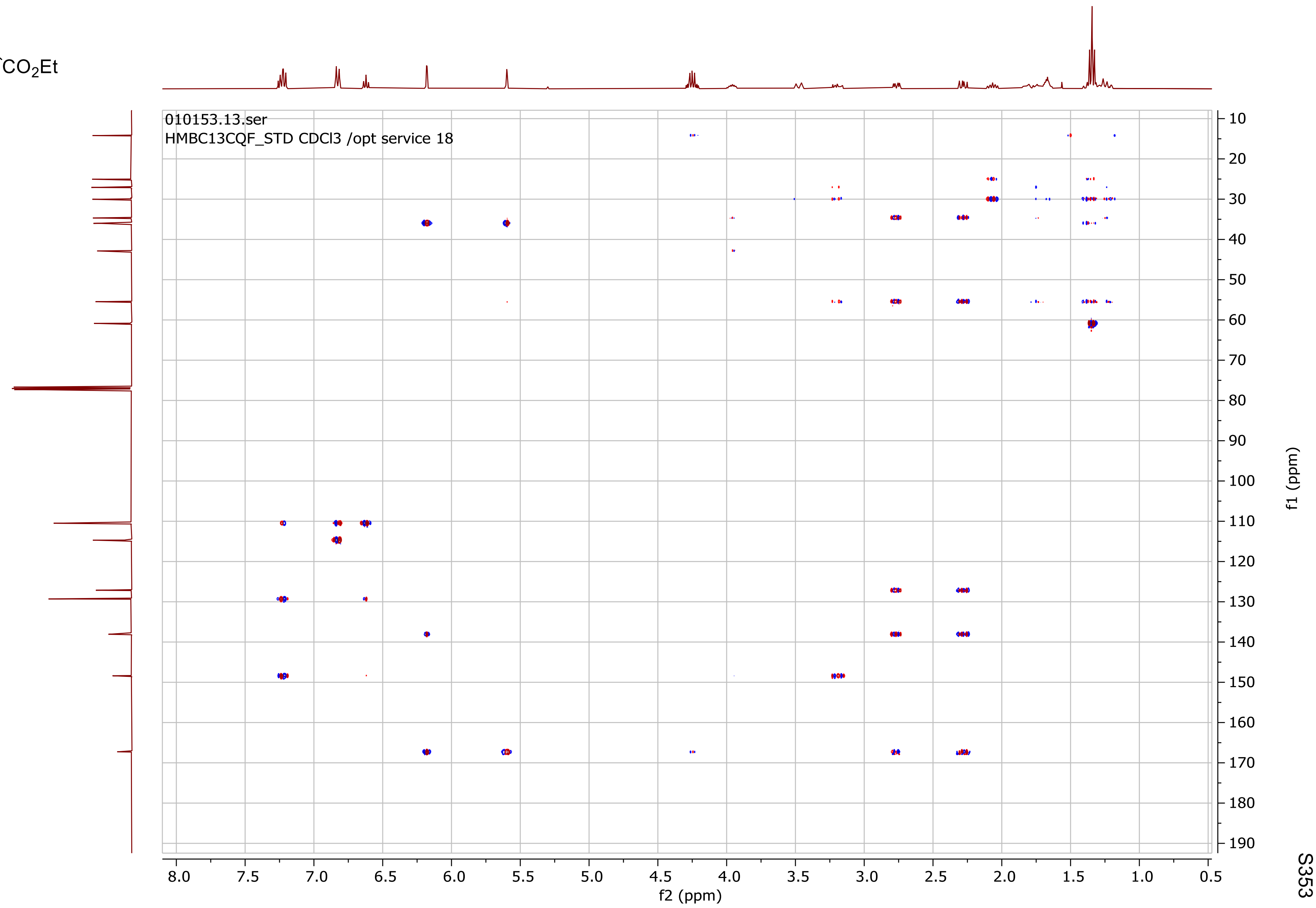

Ethyl 10-phenyl-10-azabicyclo[4.3.1]decane-8-carboxylate (**7n**)

FT-IR, ATR-diamond

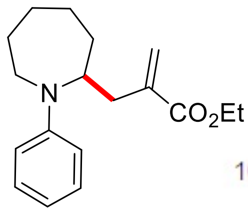

7n

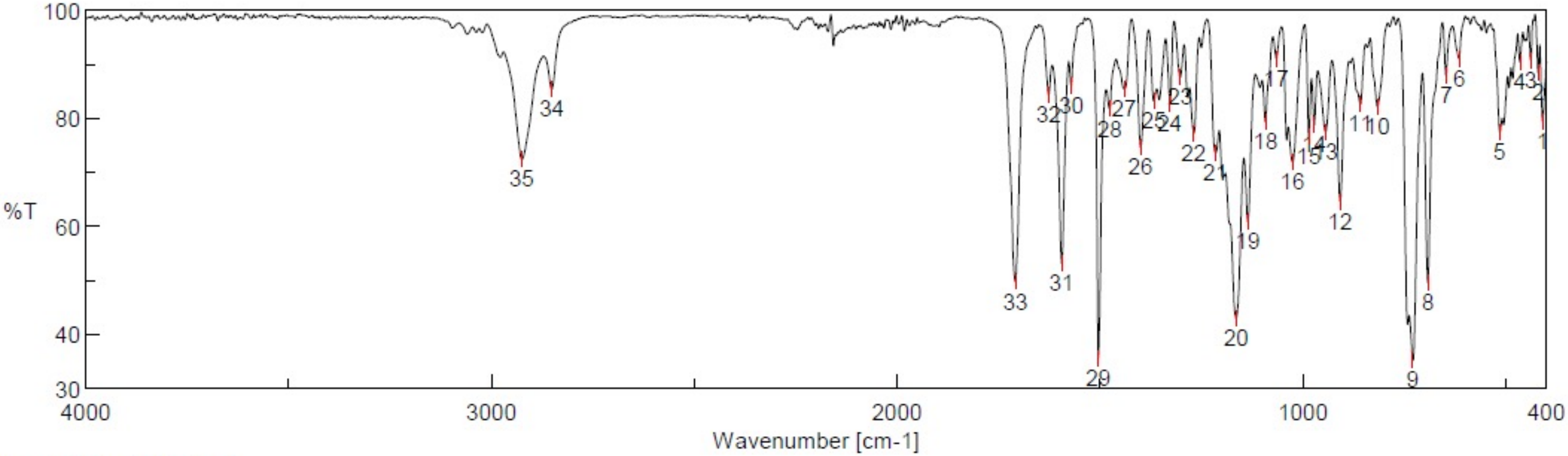

[Result of Peak Picking]

No.	Position	Intensity	No.	Position	Intensity	No.	Position	Intensity
1	408.835	79	2	420.406	88	3	438.726	91
4	464.761	90	5	514.901	77	6	615.181	91
7	647.001	88	8	692.32	50	9	728.961	35
10	816.706	82	11	858.168	83	12	908.308	65
13	943.985	78	14	971.947	79	15	985.447	77
16	1025.94	72	17	1065.48	91	18	1092.48	79
19	1135.87	61	20	1164.79	43	21	1214.93	74
22	1268.93	77	23	1302.68	88	24	1327.75	83
25	1367.28	83	26	1400.07	75	27	1440.56	85
28	1476.24	82	29	1504.2	36	30	1570.74	86
31	1594.84	53	32	1626.66	84	33	1708.62	50
34	2851.24	85	35	2925.48	72			

Methyl 4-[2-(2-ethoxycarbonylallyl)azepan-1-yl]benzoate (**7o**)

^{1}H-NMR (400 MHz, $CDCl_3$)

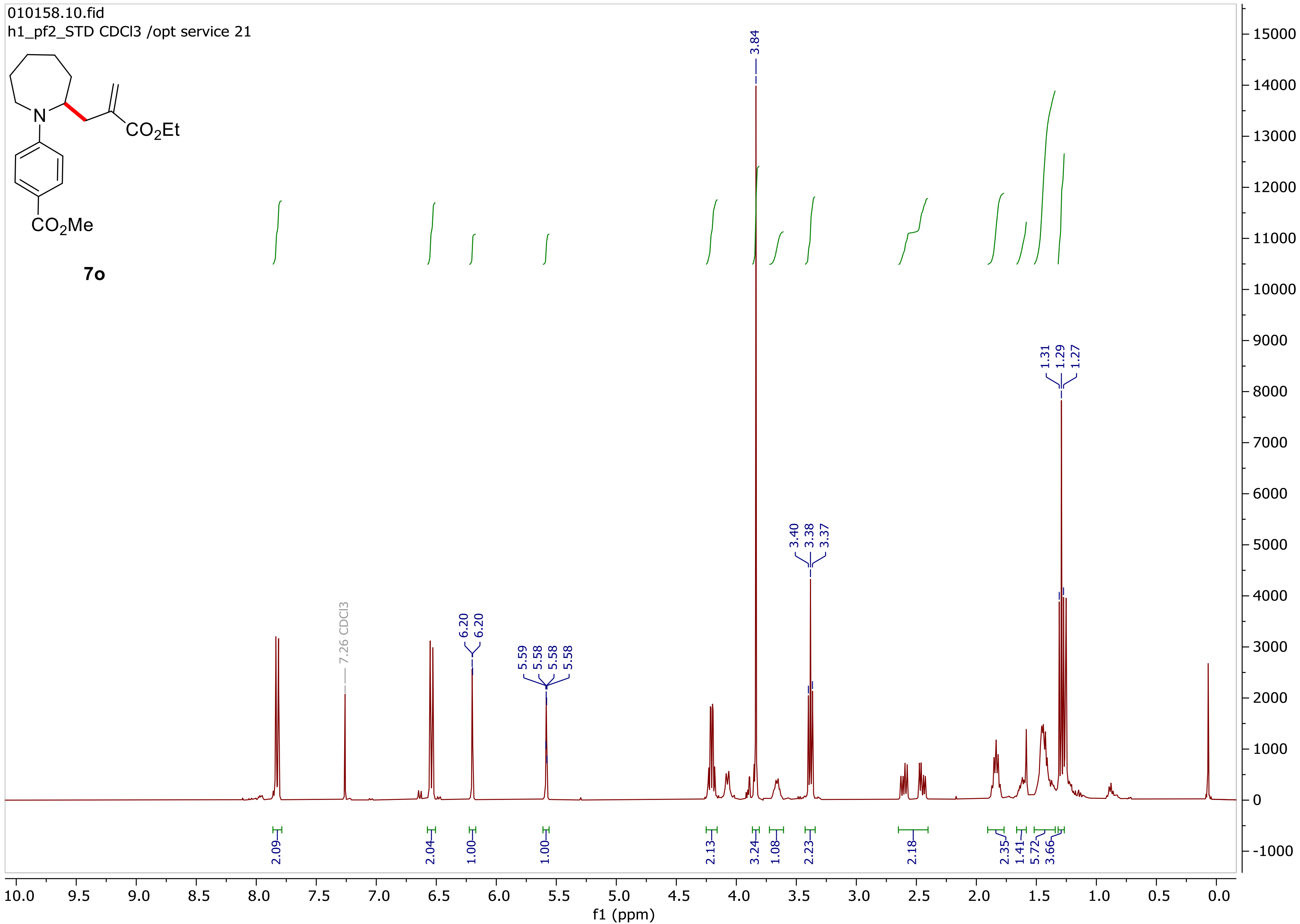

Methyl 4-[2-(2-ethoxycarbonylallyl)azepan-1-yl]benzoate (**7o**)

^{1}H-NMR (400 MHz, $CDCl_3$)

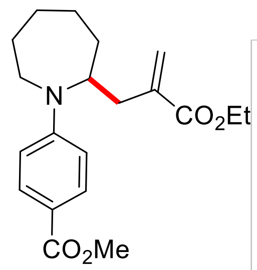

7o

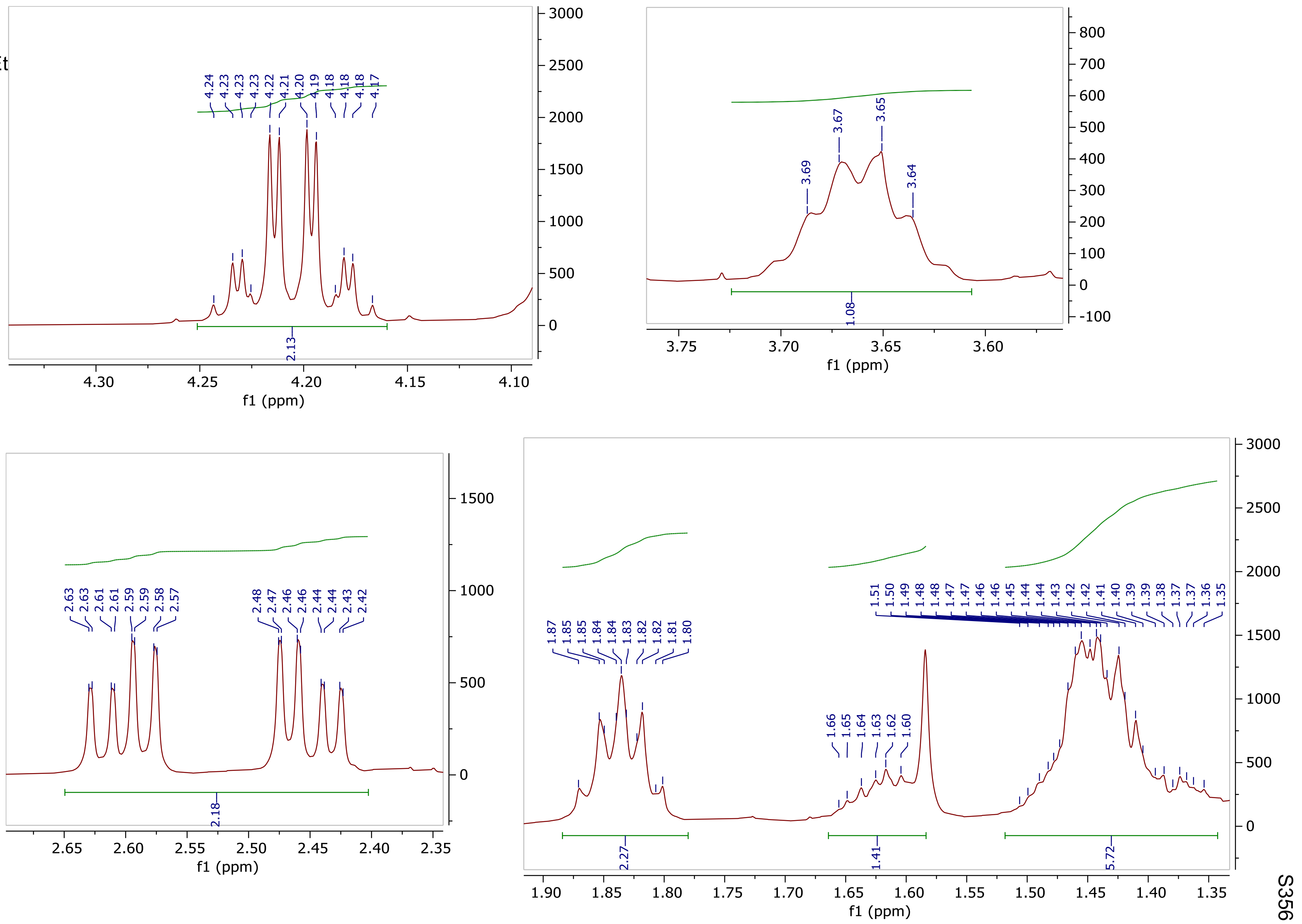

Methyl 4-[2-(2-ethoxycarbonylallyl)azepan-1-yl]benzoate (**7o**)

^{13}C-NMR (101 MHz, $CDCl_3$)

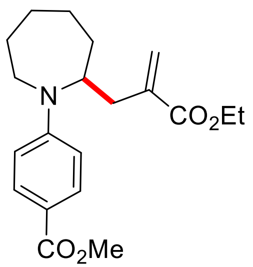

7o

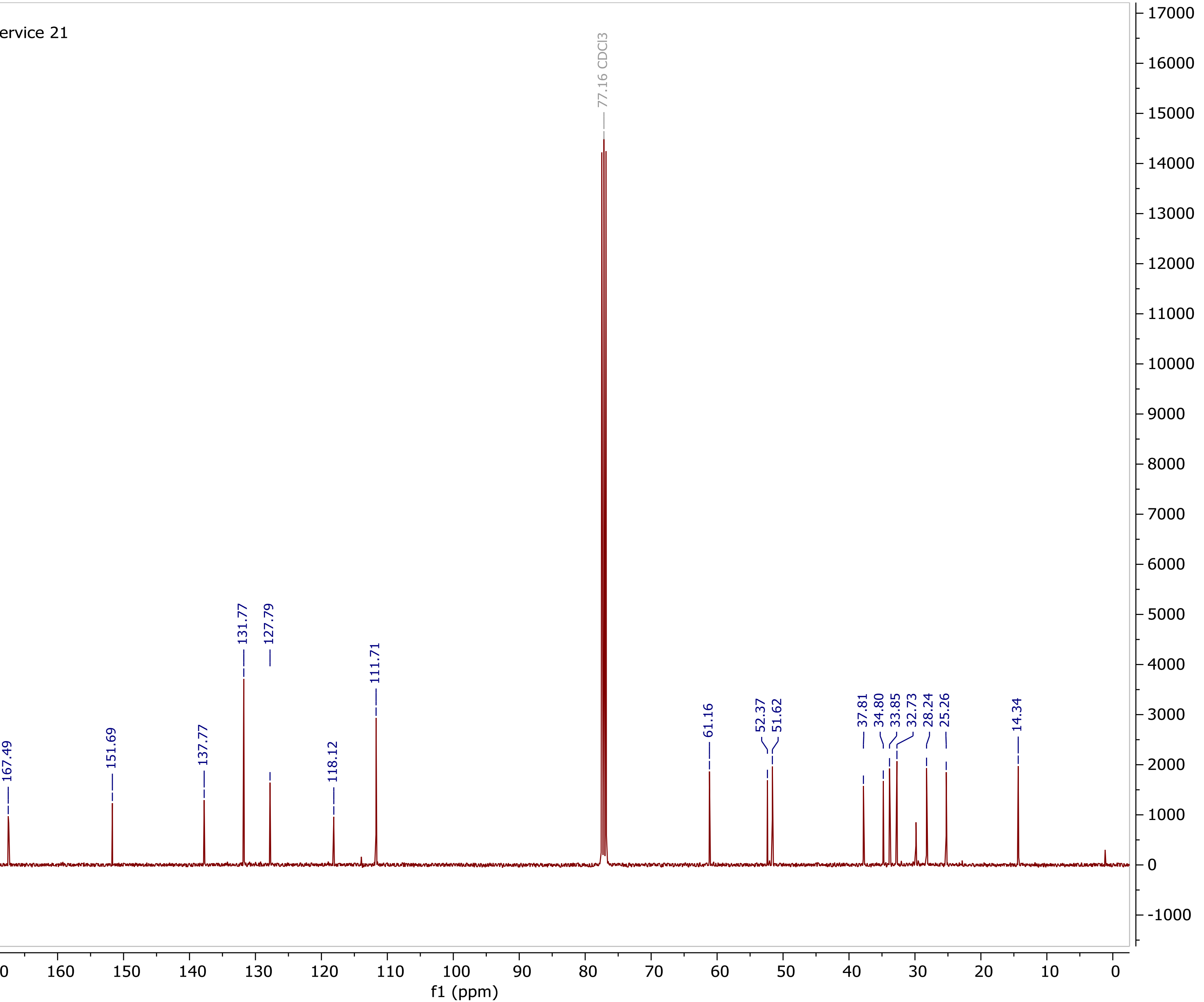

Methyl 4-[2-(2-ethoxycarbonylallyl)azepan-1-yl]benzoate (**7o**)

^{13}C-NMR (101 MHz, $CDCl_3$)

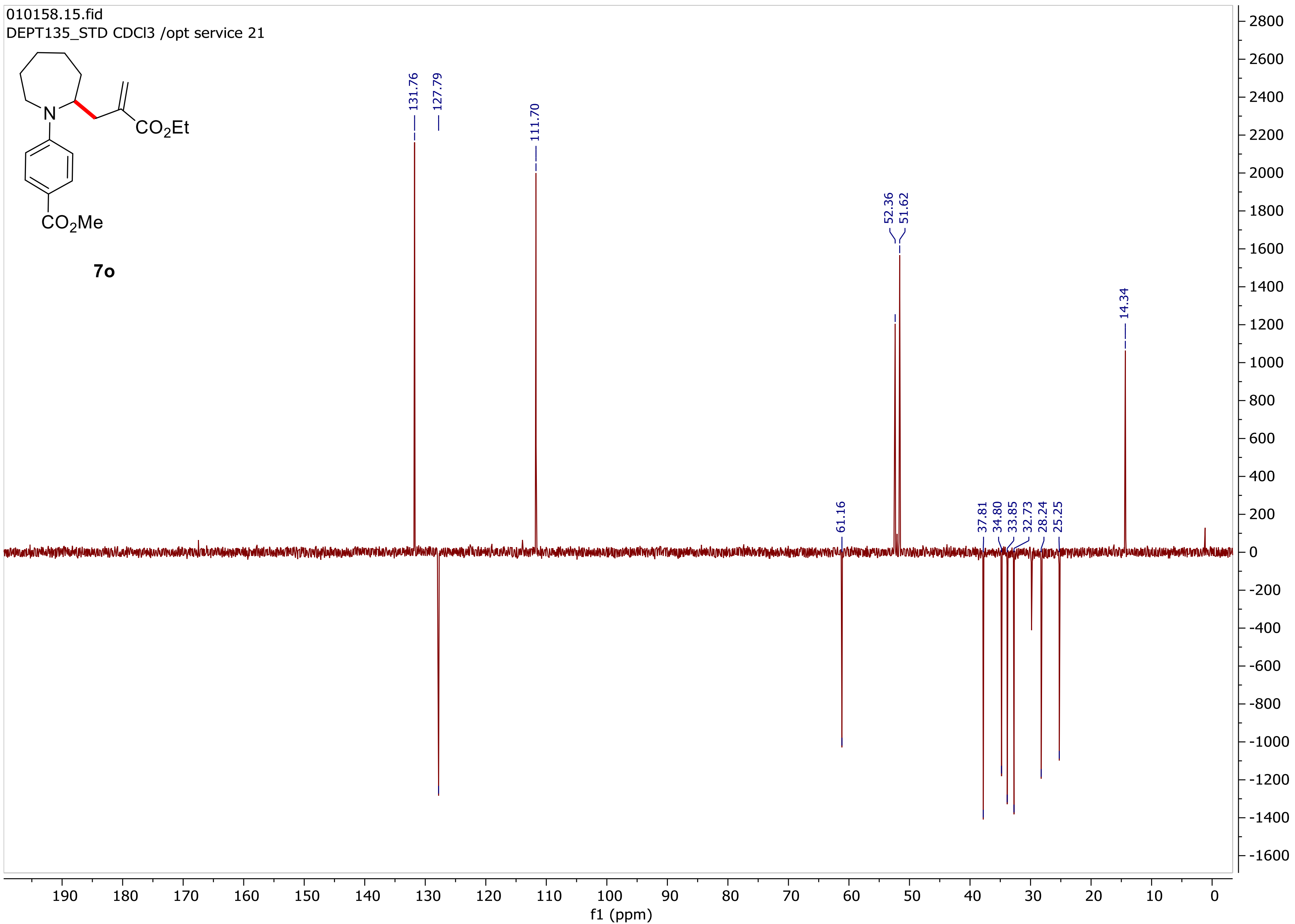

Methyl 4-[2-(2-ethoxycarbonylallyl)azepan-1-yl]benzoate (**7o**)

^{1}H, ^{1}H-COSY NMR (400 MHz, $CDCl_3$)

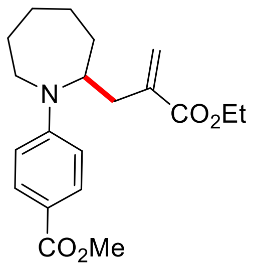

7o

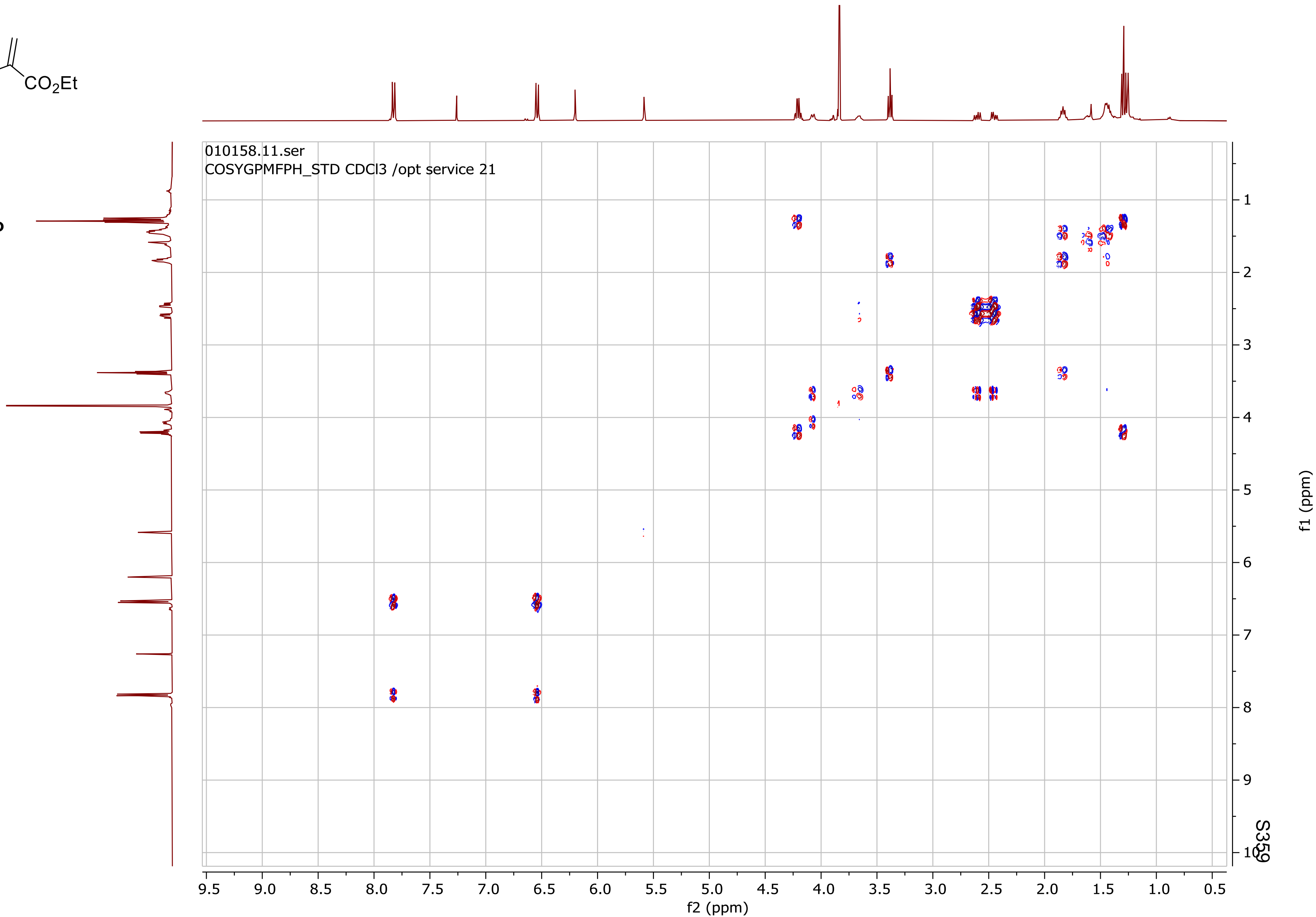

Methyl 4-[2-(2-ethoxycarbonylallyl)azepan-1-yl]benzoate (**7o**)

^{1}H, ^{13}C-HSQC NMR (400 MHz, $CDCl_3$)

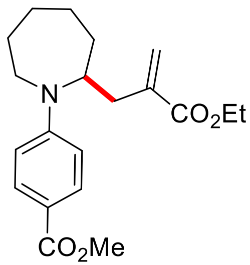

7o

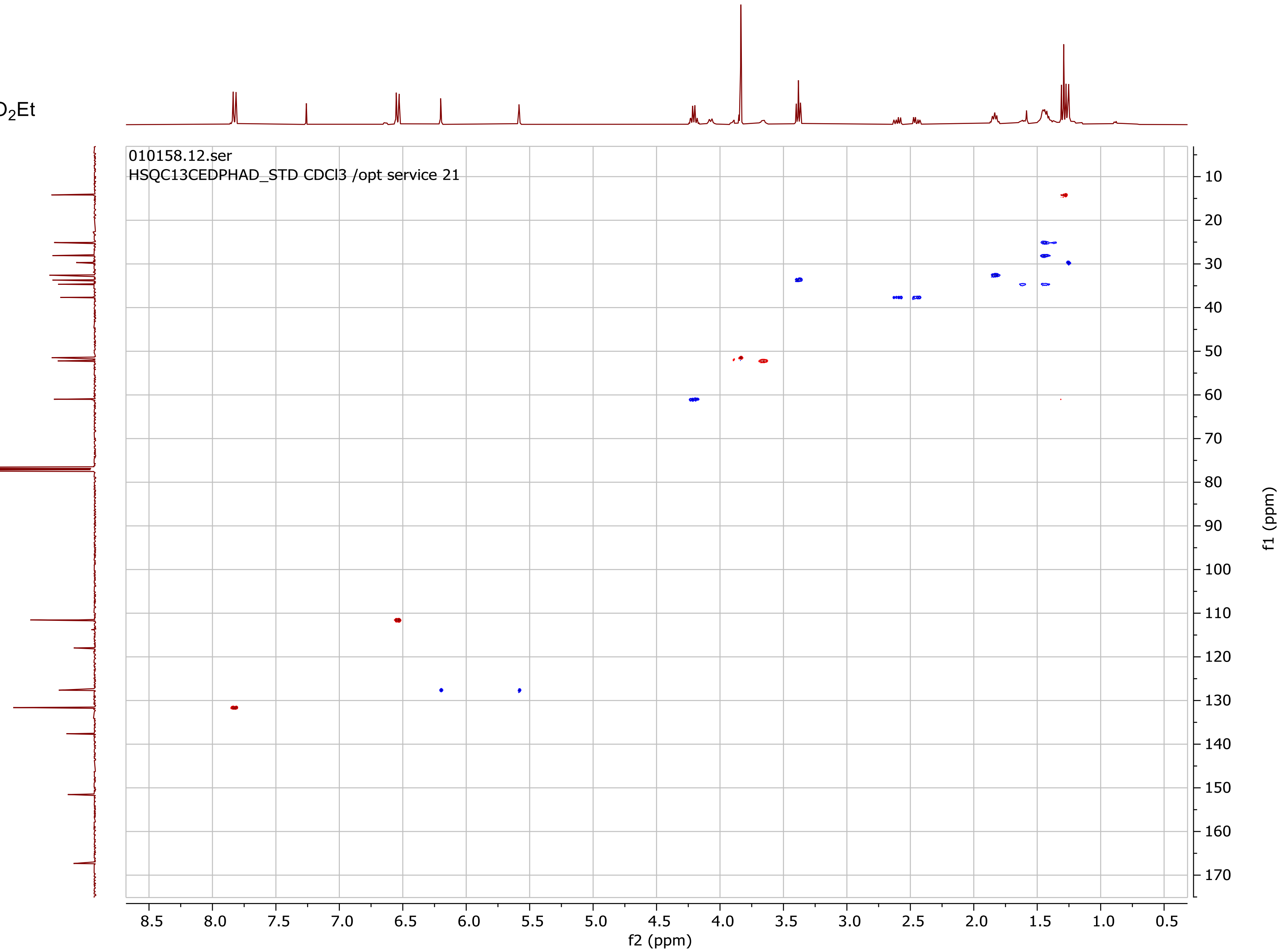

Methyl 4-[2-(2-ethoxycarbonylallyl)azepan-1-yl]benzoate (**7o**)

^1H, ^{13}C-HMBC NMR (400 MHz, $CDCl_3$)

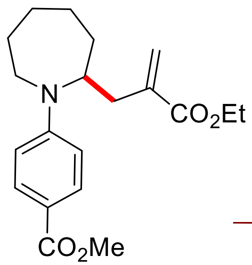

7o

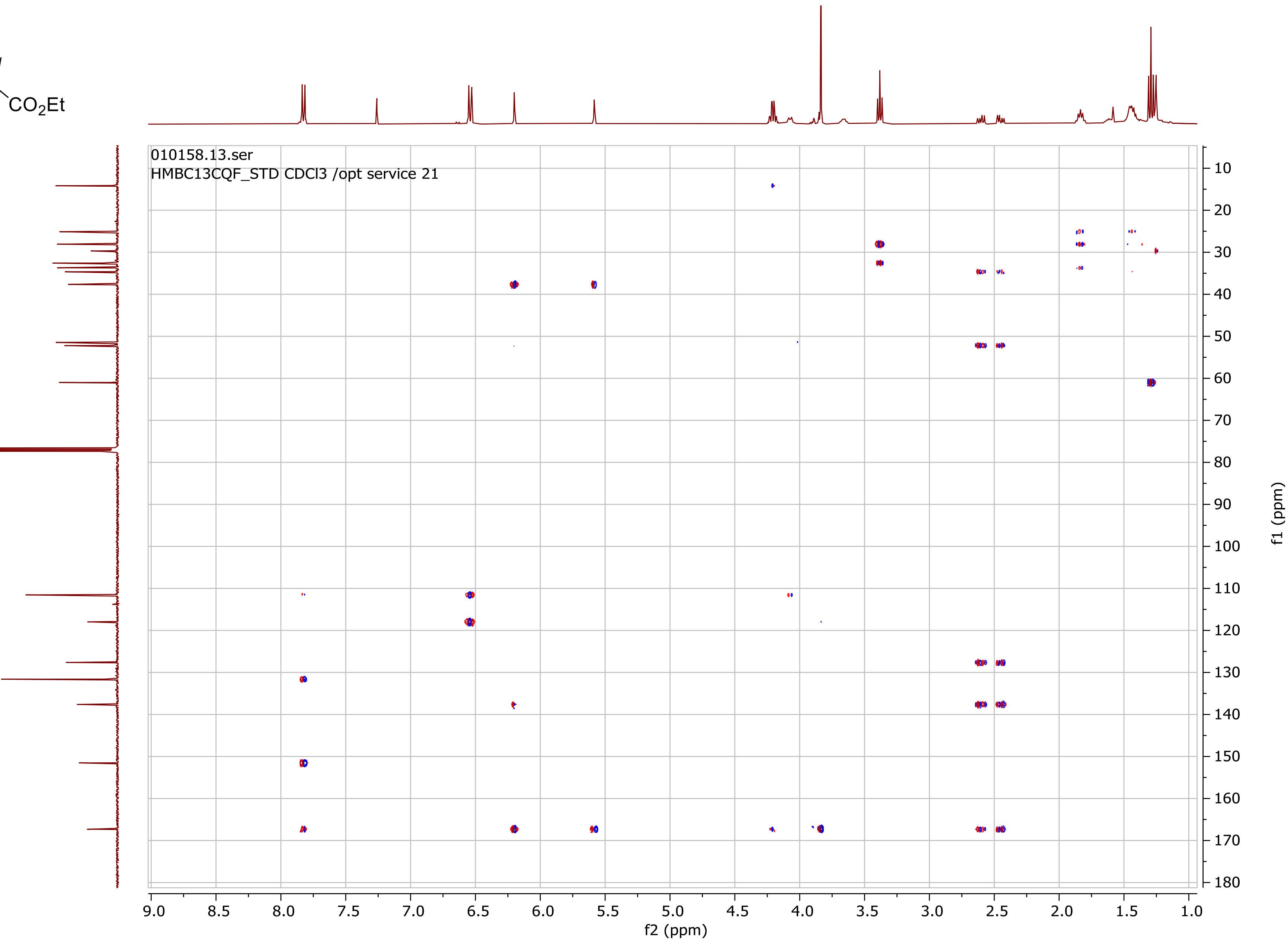

Methyl 4-[2-(2-ethoxycarbonylallyl)azepan-1-yl]benzoate (**7o**)

FT-IR, ATR-diamond

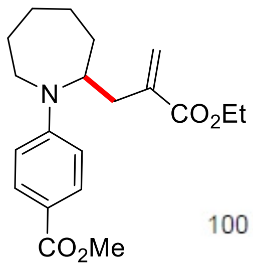

7o

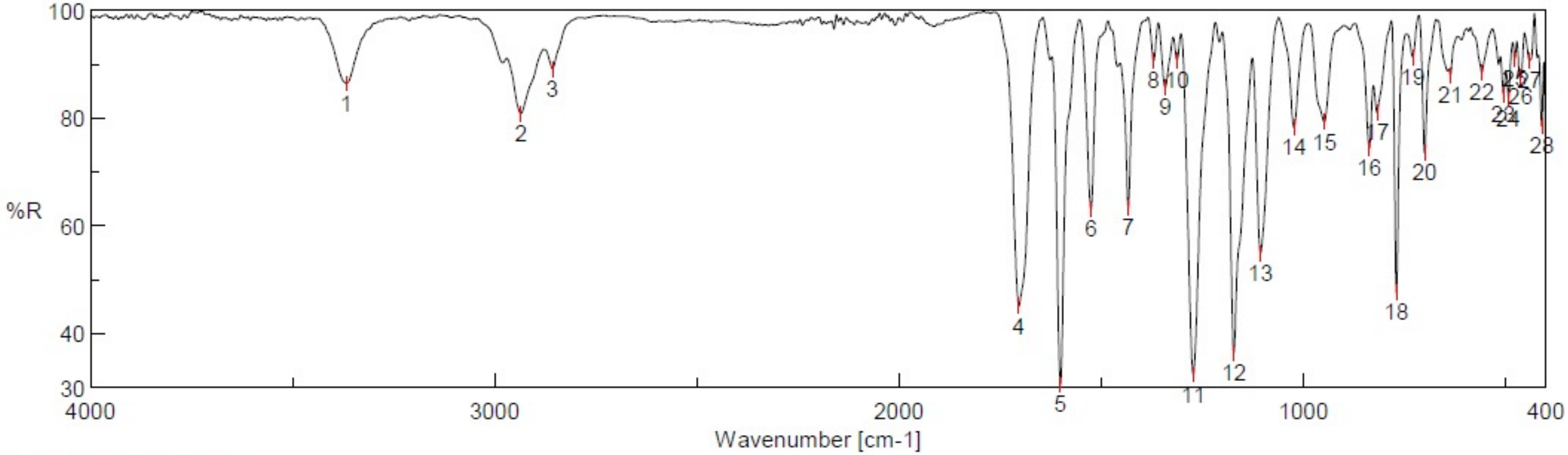

[Result of Peak Picking]

No.	Position	Intensity	No.	Position	Intensity	No.	Position	Intensity
1	3367.1	86.4056	2	2935.13	80.8216	3	2857.02	89.1497
4	1703.8	45.0529	5	1600.63	30.4485	6	1525.42	63.0252
7	1432.85	63.3347	8	1370.18	90.652	9	1341.25	85.6644
10	1312.32	90.8159	11	1271.82	32.3485	12	1171.54	36.3304
13	1105.98	54.9025	14	1023.05	78.22	15	947.842	79.2808
16	836.955	74.3547	17	817.67	80.9954	18	769.458	47.632
19	728.961	91.3178	20	700.034	73.5058	21	636.394	88.0698
22	558.291	88.4828	23	505.258	84.4259	24	492.723	83.5514
25	477.296	90.9915	26	462.832	87.5963	27	440.655	90.712
28	409.799	78.5606						

Methyl 4-[2-[(E)-2-ethoxycarbonylbut-2-enyl]-1-piperidyl]benzoate (**9**) *E*/*Z* 20:1

^{1}H-NMR (400 MHz, $CDCl_3$)

010160.10.fid
h1_pf2_STD CDCl3 /opt nmr 15

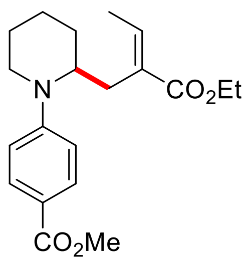

9, *E*/*Z* 20:1

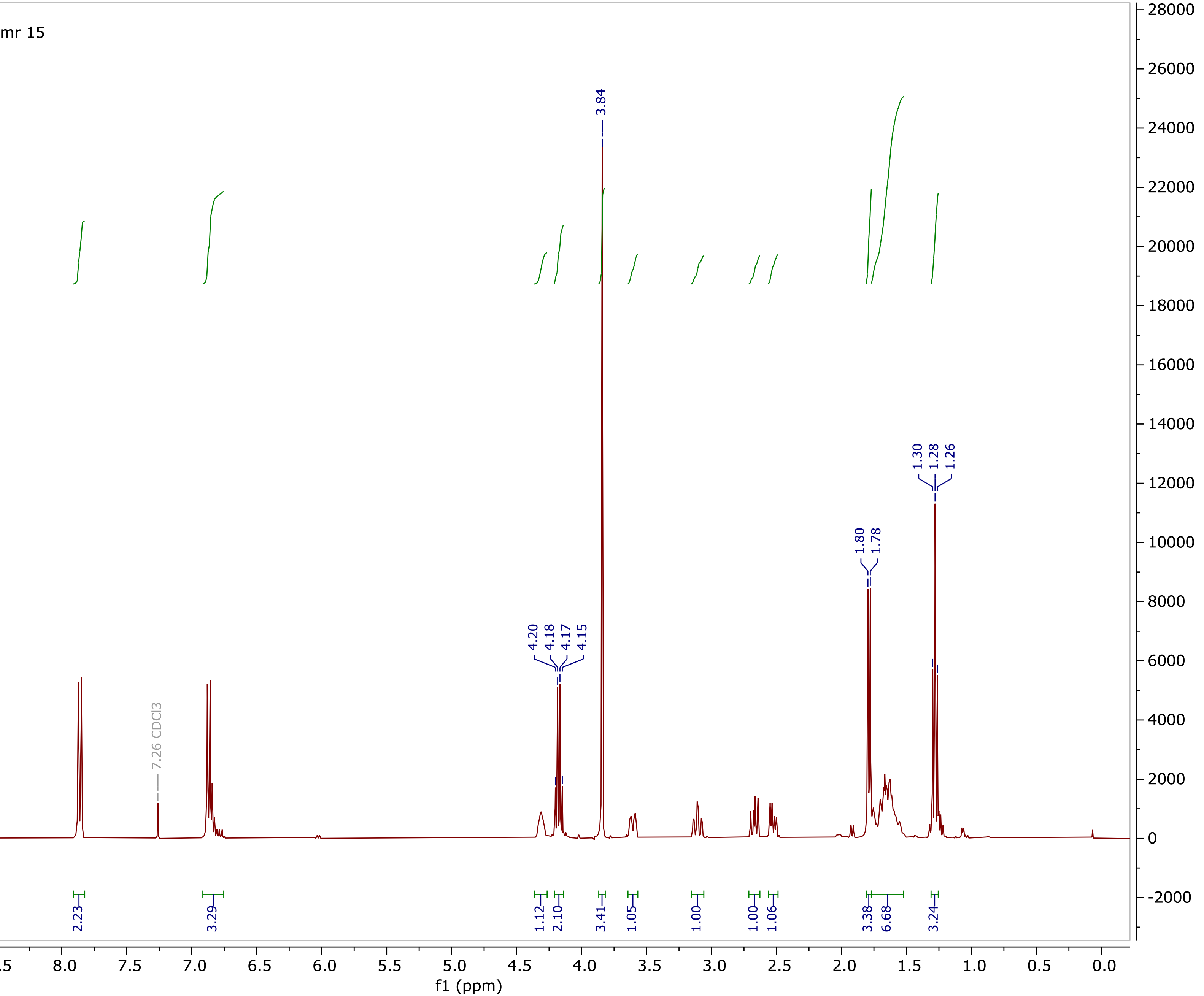

Methyl 4-[2-[(E)-2-ethoxycarbonylbut-2-enyl]-1-piperidyl]benzoate (**9**) *E*/*Z* 20:1

^{1}H-NMR (400 MHz, $CDCl_3$)

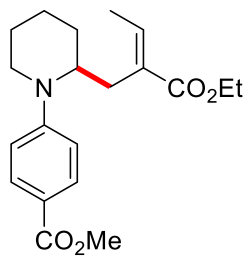

9, *E*/*Z* 20:1

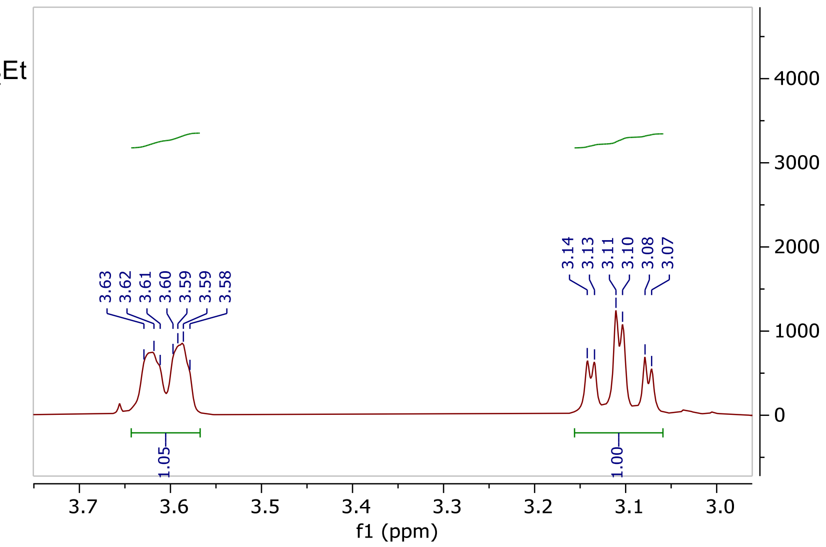

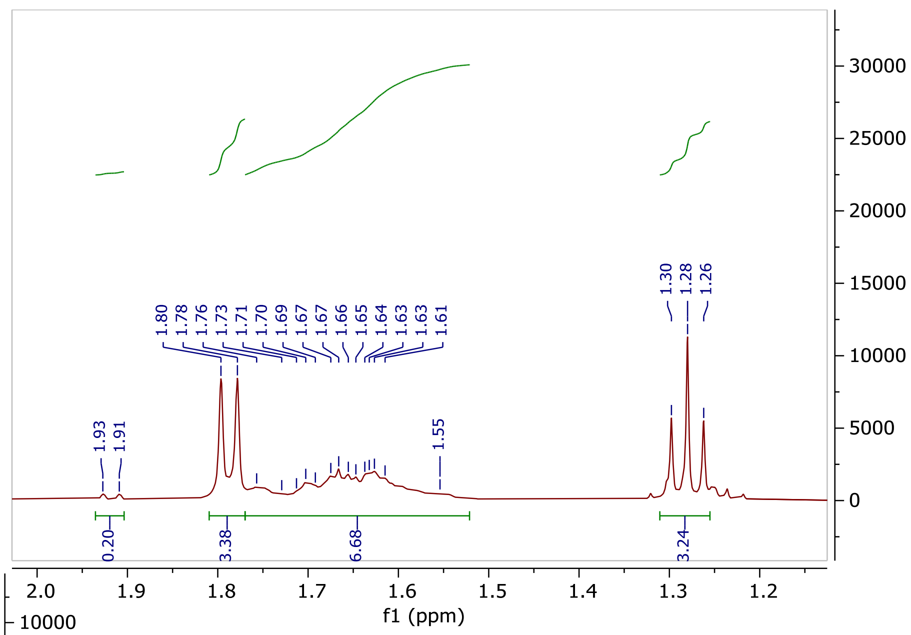

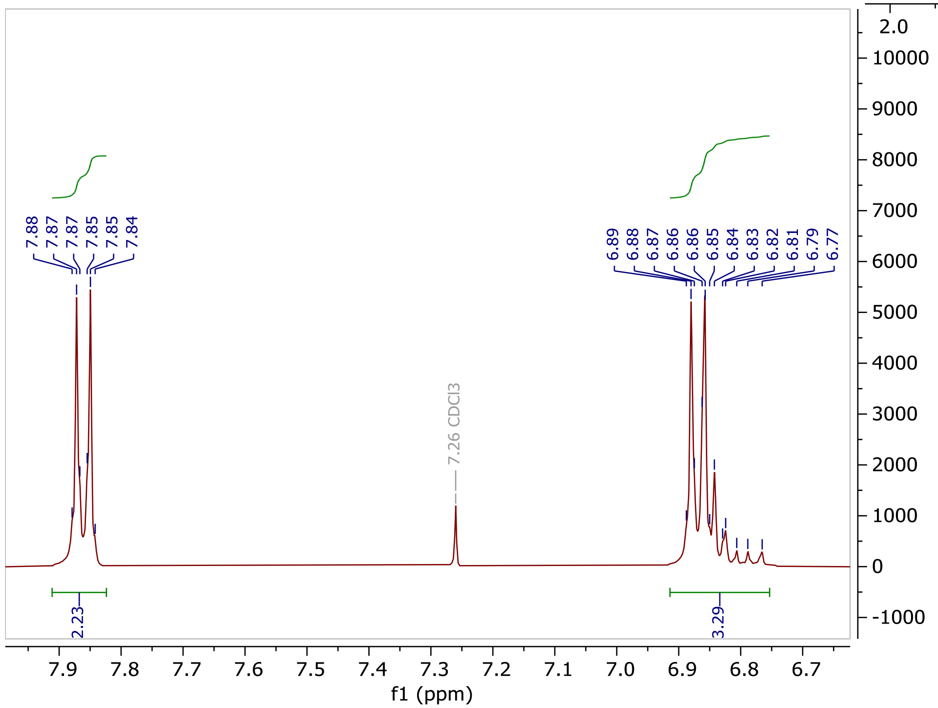

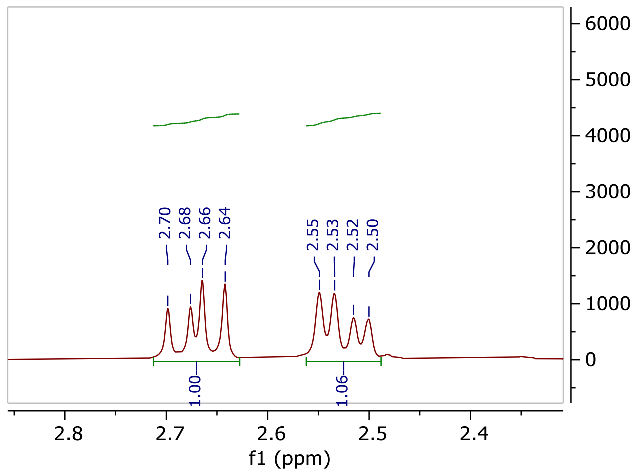

Methyl 4-[2-[(E)-2-ethoxycarbonylbut-2-enyl]-1-piperidyl]benzoate (**9**) *E*/*Z* 20:1

^{13}C-NMR (75 MHz, $CDCl_3$)

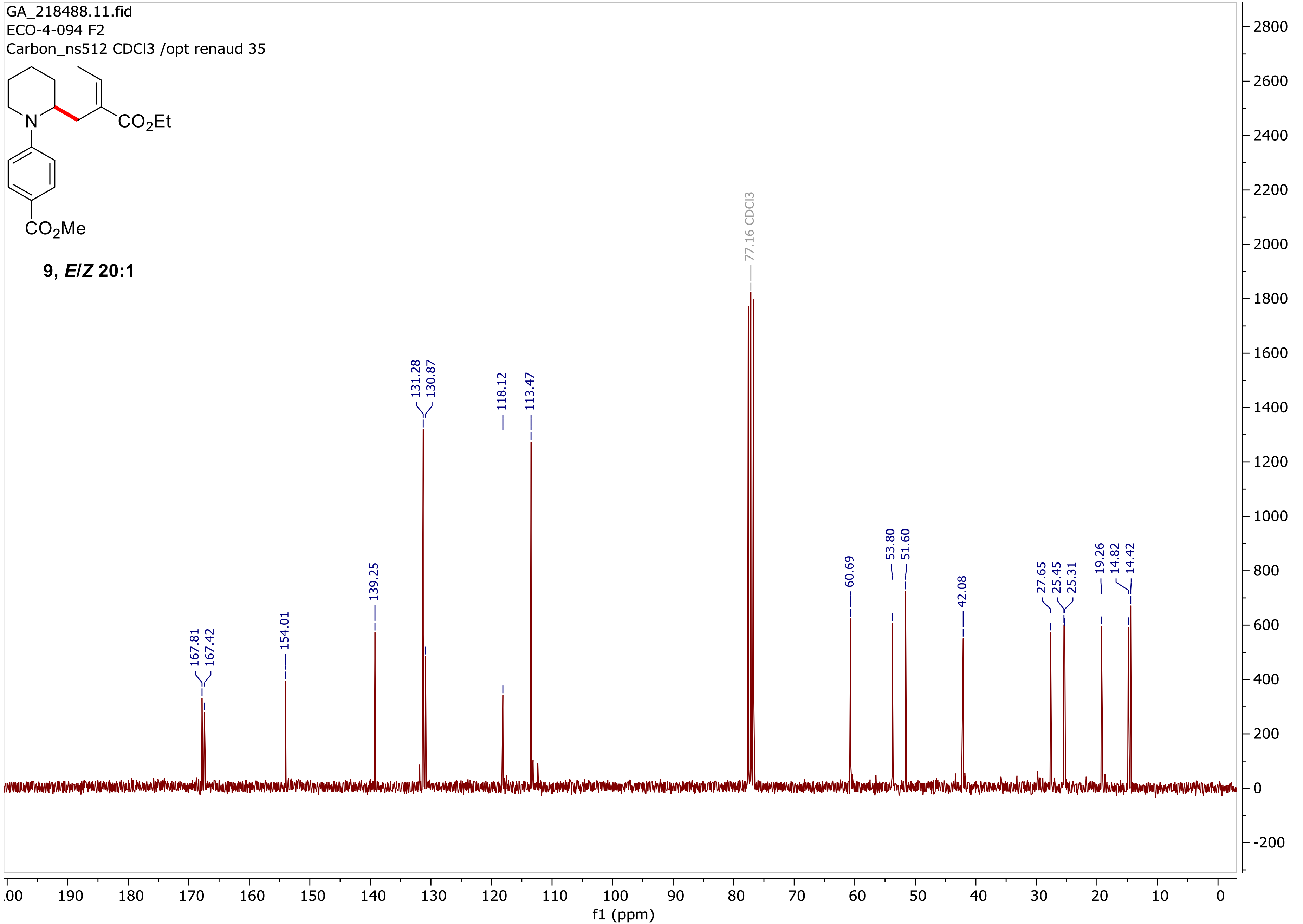

Methyl 4-[2-[(E)-2-ethoxycarbonylbut-2-enyl]-1-piperidyl]benzoate (**9**) *E*/*Z* 20:1

^{13}C-NMR (75 MHz, $CDCl_3$)

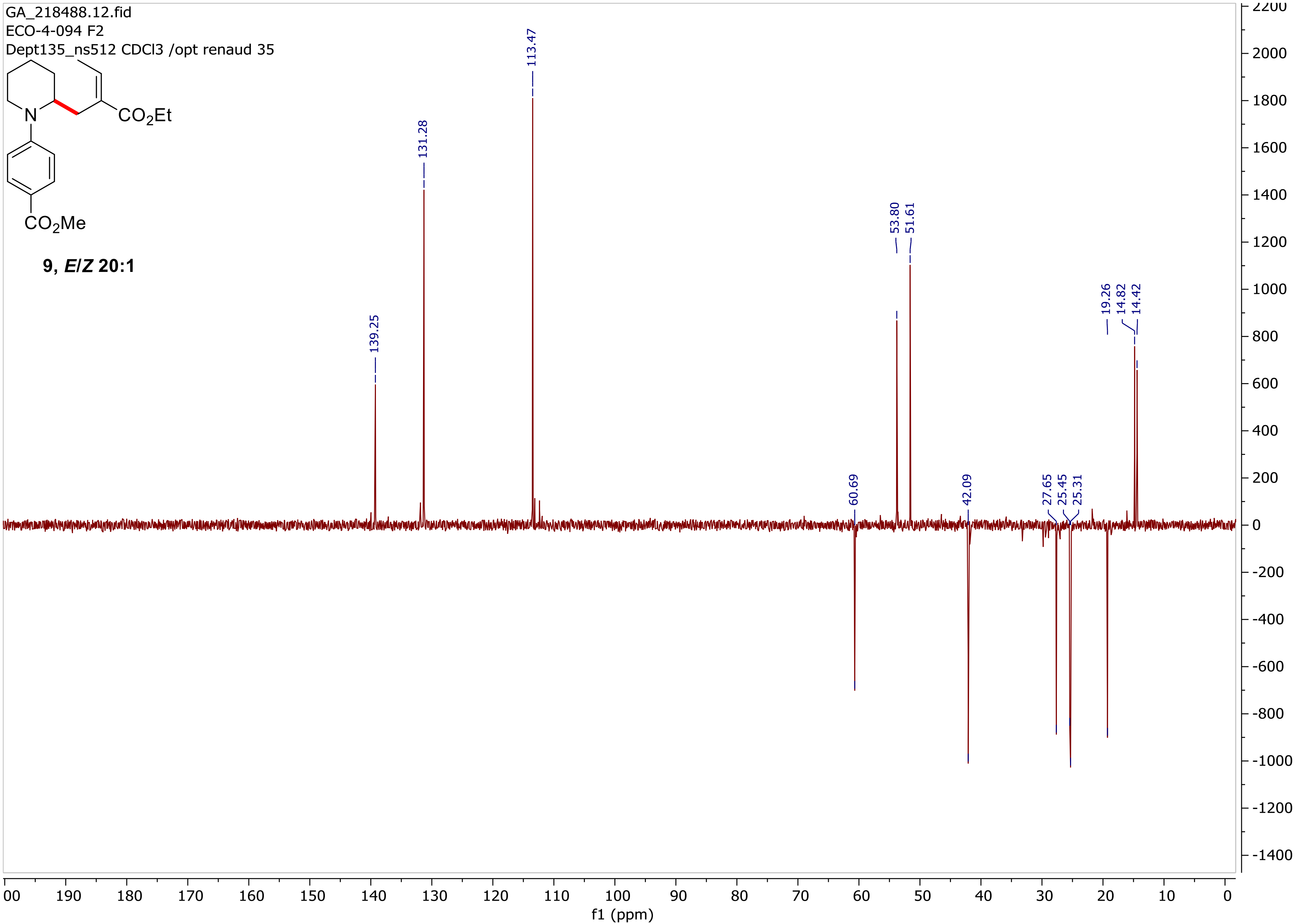

Methyl 4-[2-[(E)-2-ethoxycarbonylbut-2-enyl]-1-piperidyl]benzoate (**9**) *E*/*Z* 20:1

^{1}H, ^{1}H-COSY NMR (400 MHz, $CDCl_3$)

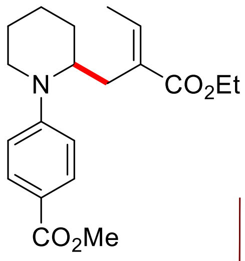

9, *E*/*Z* 20:1

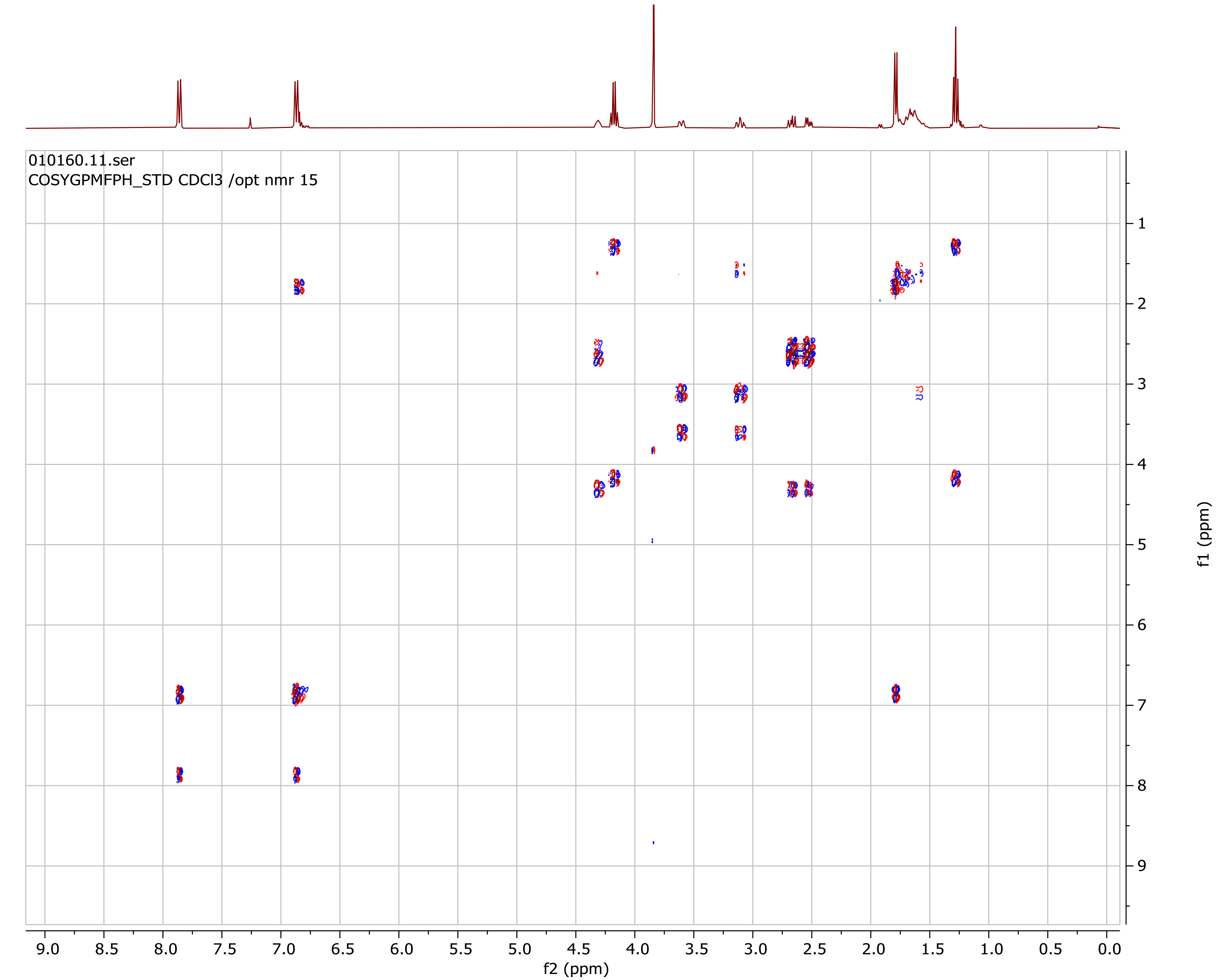

Methyl 4-[2-[(E)-2-ethoxycarbonylbut-2-enyl]-1-piperidyl]benzoate (**9**) *E*/*Z* 20:1

^{1}H, ^{13}C-HSQC NMR (400 MHz, $CDCl_3$)

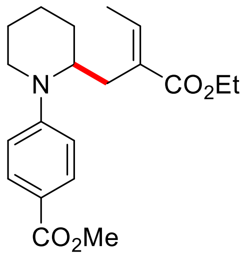

9, *E*/*Z* 20:1

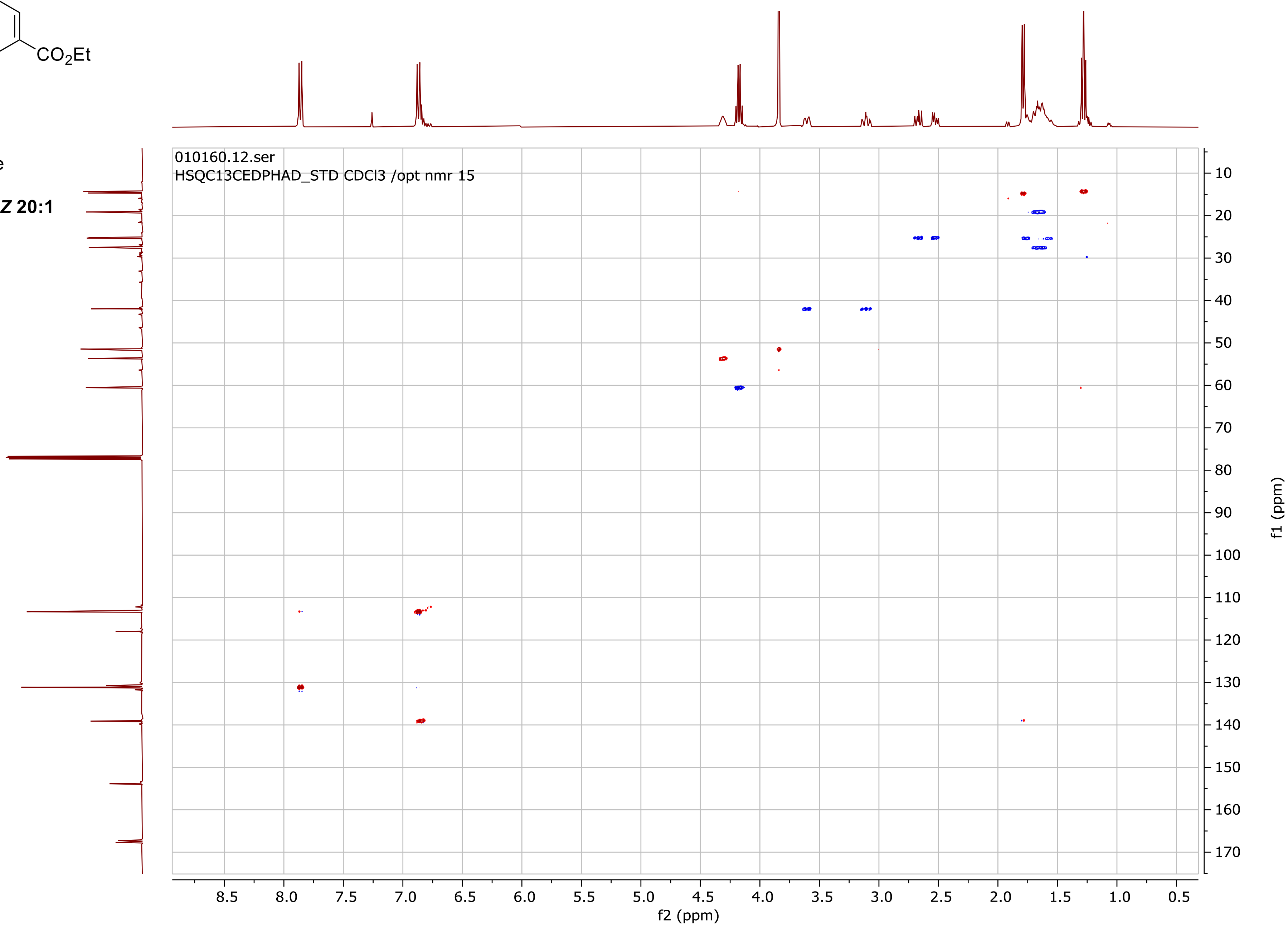

Methyl 4-[2-[(E)-2-ethoxycarbonylbut-2-enyl]-1-piperidyl]benzoate (**9**) *E*/*Z* 20:1

^1H, ^{13}C-HMBC NMR (400 MHz, $CDCl_3$)

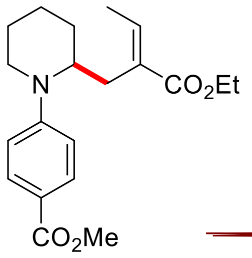

9, *E*/*Z* 20:1

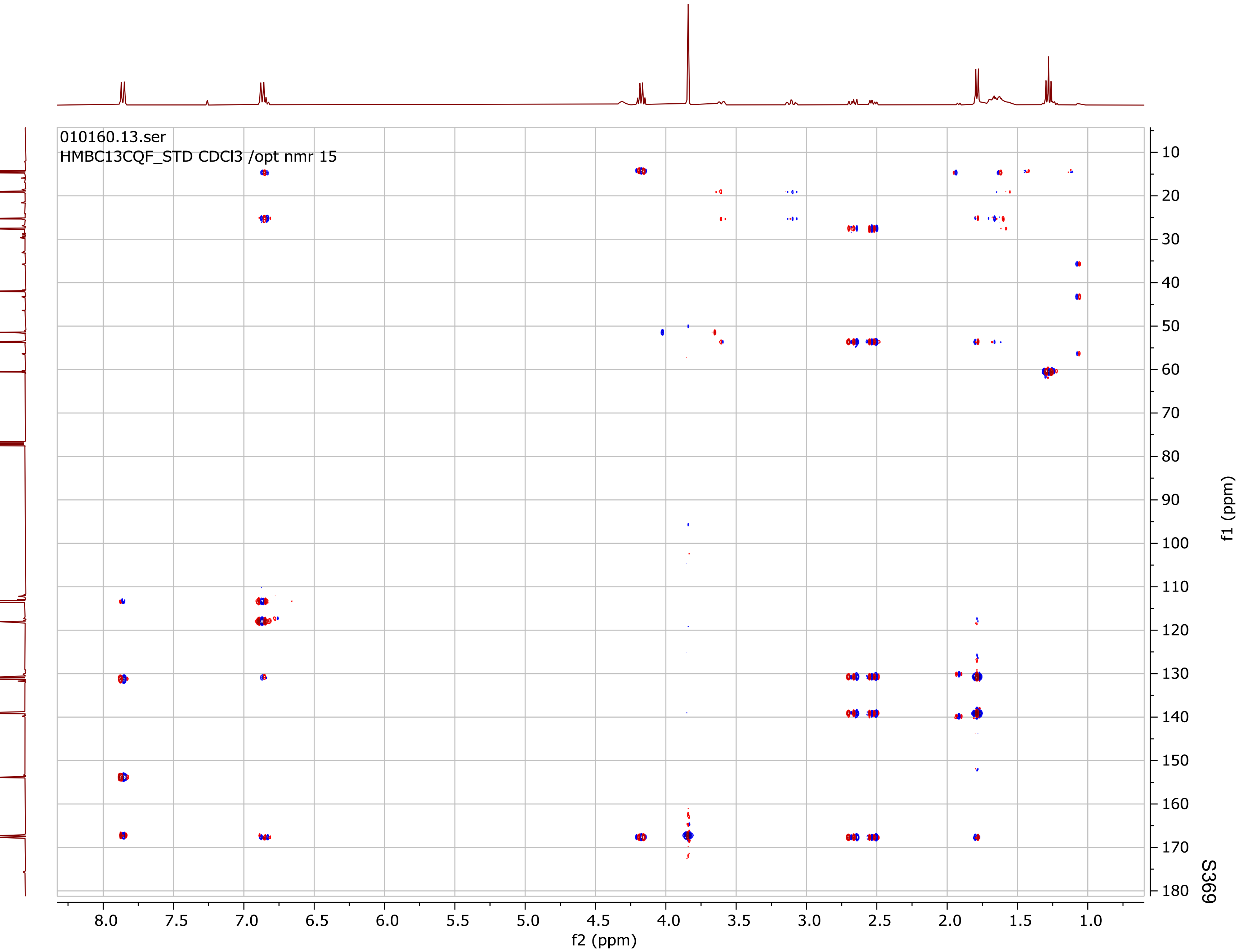

Methyl 4-[2-[(E)-2-ethoxycarbonylbut-2-enyl]-1-piperidyl]benzoate (**9**) *E*/*Z* 20:1

FT-IR, ATR-diamond

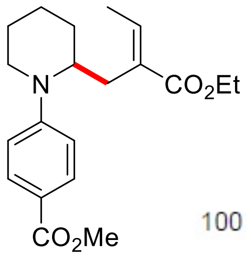

9, *E*/*Z* 20:1

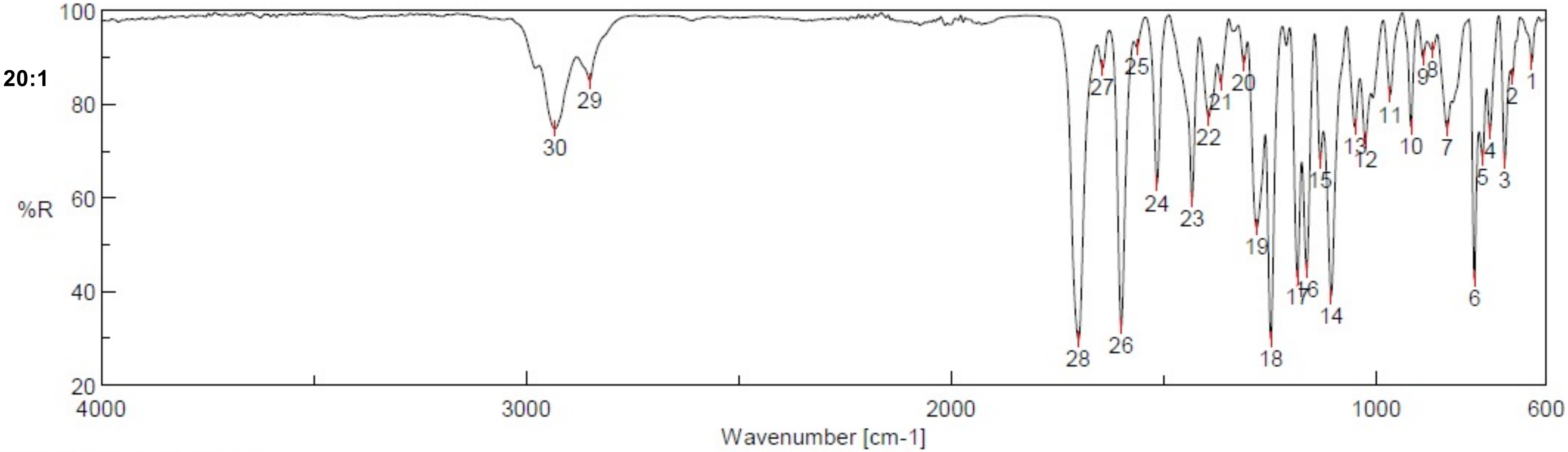

[Result of Peak Picking]

No.	Position	Intensity	No.	Position	Intensity	No.	Position	Intensity
1	634.466	89.0413	2	679.785	85.8353	3	697.141	67.8748
4	731.853	74.2253	5	750.174	68.7004	6	768.494	42.6058
7	833.098	74.9264	8	867.81	91.5371	9	889.987	89.8905
10	917.95	75.1871	11	967.126	81.9662	12	1025.94	72.2532
13	1050.05	75.0656	14	1105.98	39.014	15	1132.01	67.9259
16	1163.83	44.5958	17	1185.04	42.9615	18	1247.72	29.858
19	1281.47	53.6605	20	1311.36	88.782	21	1365.35	84.6149
22	1394.28	77.1796	23	1432.85	59.7563	24	1515.78	63.0618
25	1563.02	92.2047	26	1599.66	32.4707	27	1644.02	87.6606
28	1700.91	29.6715	29	2850.27	85.0547	30	2932.23	74.8021

Synthetic utility

Ethyl 9-phenyl-9-azabicyclo[3.3.1]nonane-3-carboxylate (**8aβ**)**_after epimerization α/β 1:8**

^{1}H-NMR (300 MHz, $CDCl_3$)

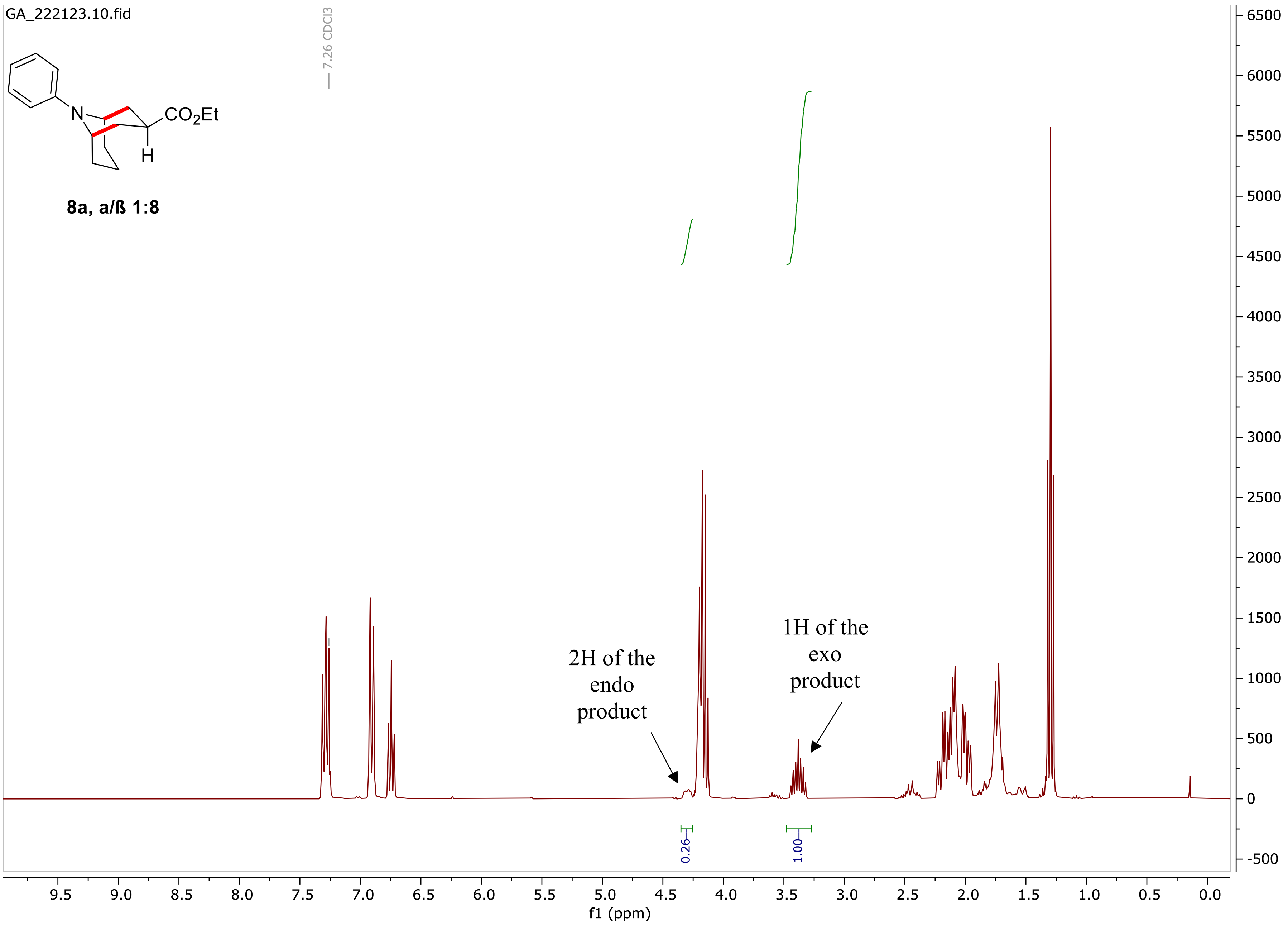

Ethyl 9-phenyl-9-azabicyclo[3.3.1]nonane-3-carboxylate (**8aβ**)

^{1}H-NMR (300 MHz, $CDCl_3$)

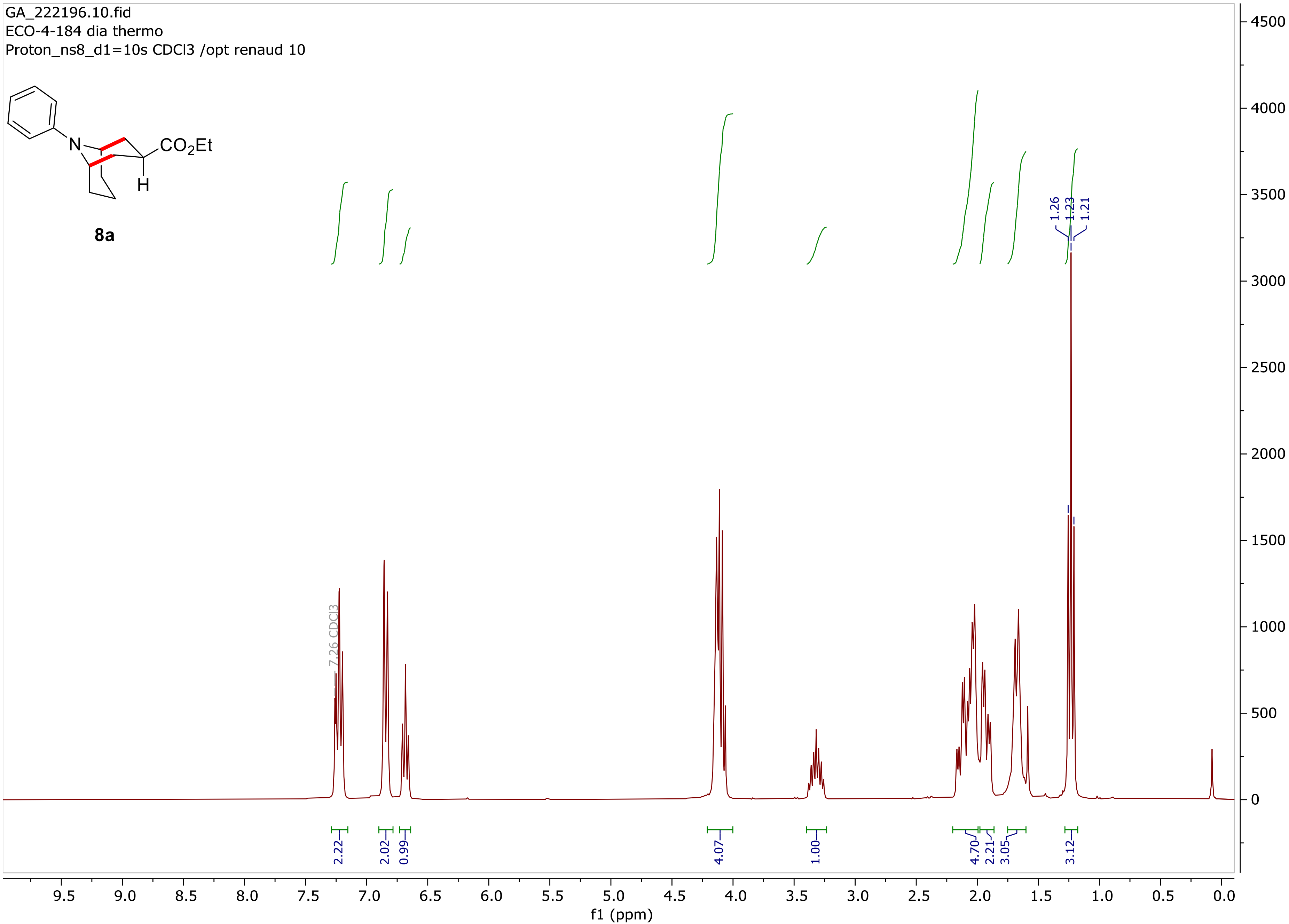

8a

Ethyl 9-phenyl-9-azabicyclo[3.3.1]nonane-3-carboxylate (**8aβ**)

^{1}H-NMR (300 MHz, $CDCl_3$)

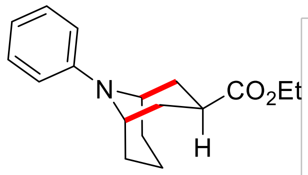

8a

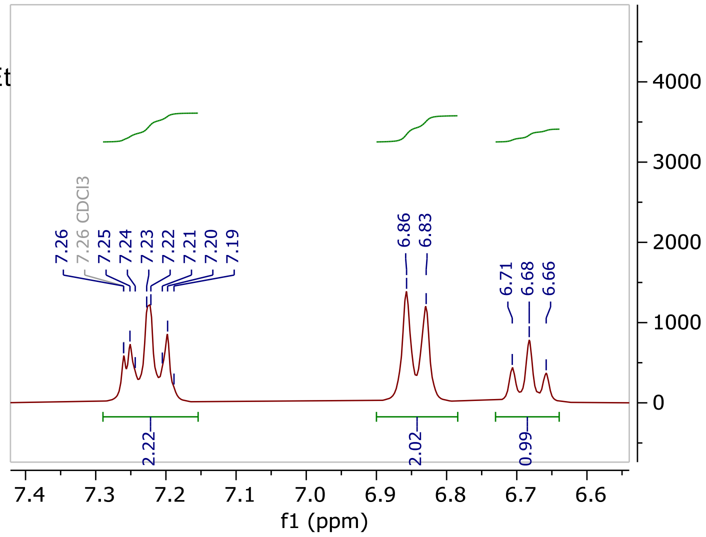

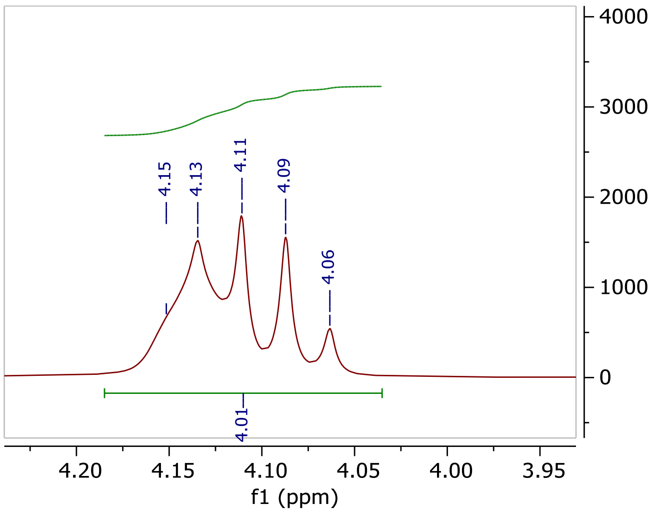

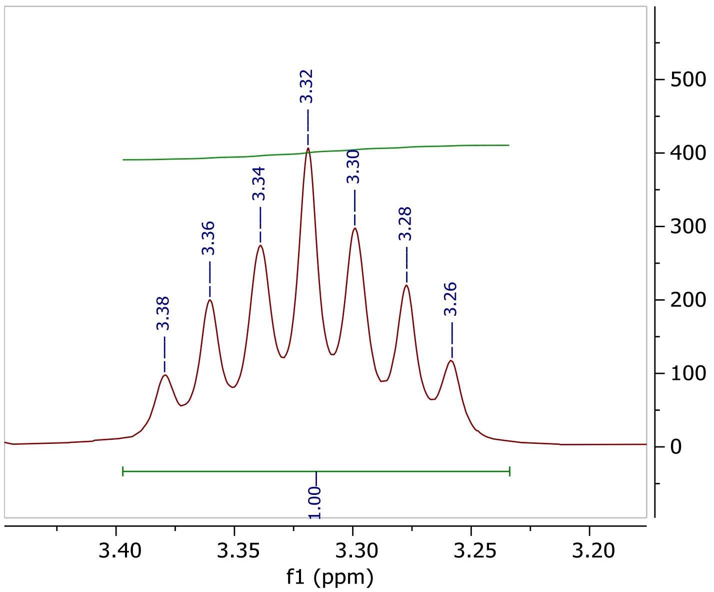

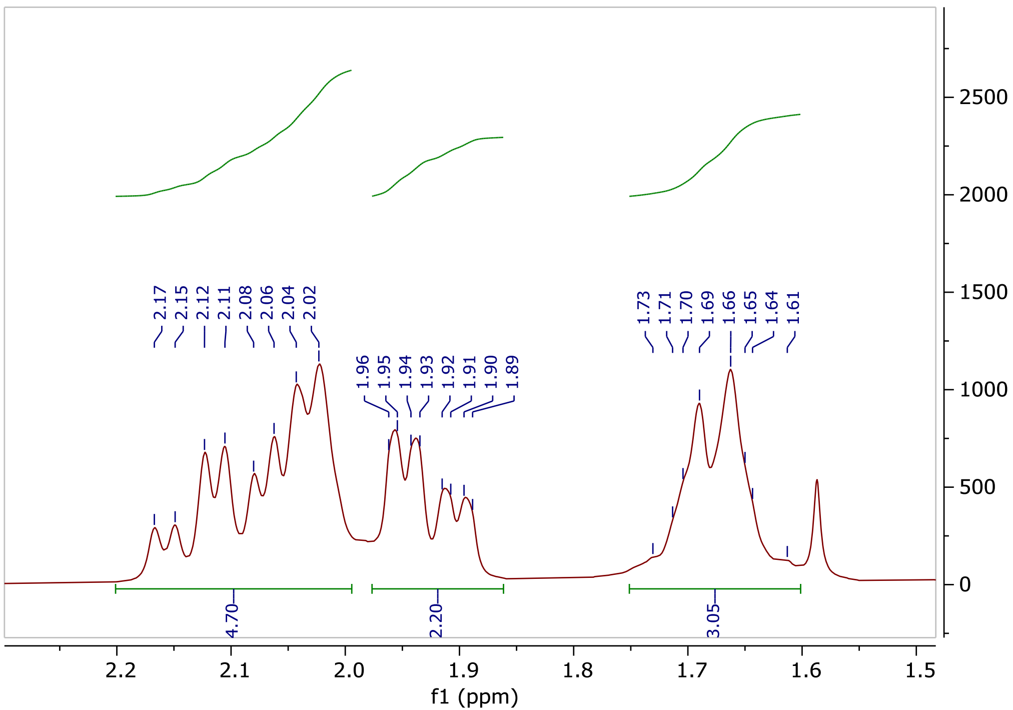

Ethyl 9-phenyl-9-azabicyclo[3.3.1]nonane-3-carboxylate (**8aβ**)

^{13}C-NMR (75 MHz, $CDCl_3$)

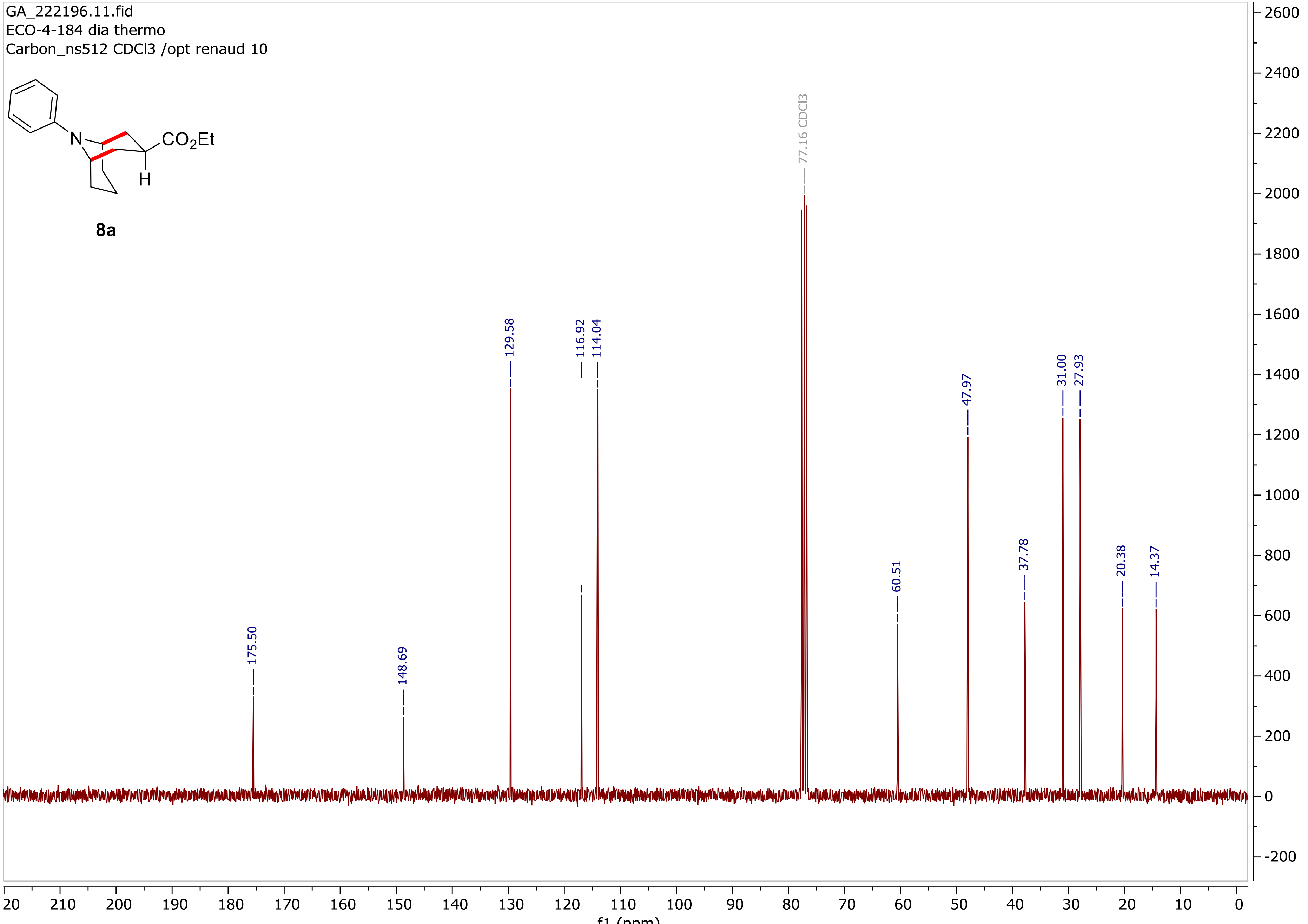

Ethyl 9-phenyl-9-azabicyclo[3.3.1]nonane-3-carboxylate (**8aβ**)

^{13}C-NMR (75 MHz, $CDCl_3$)

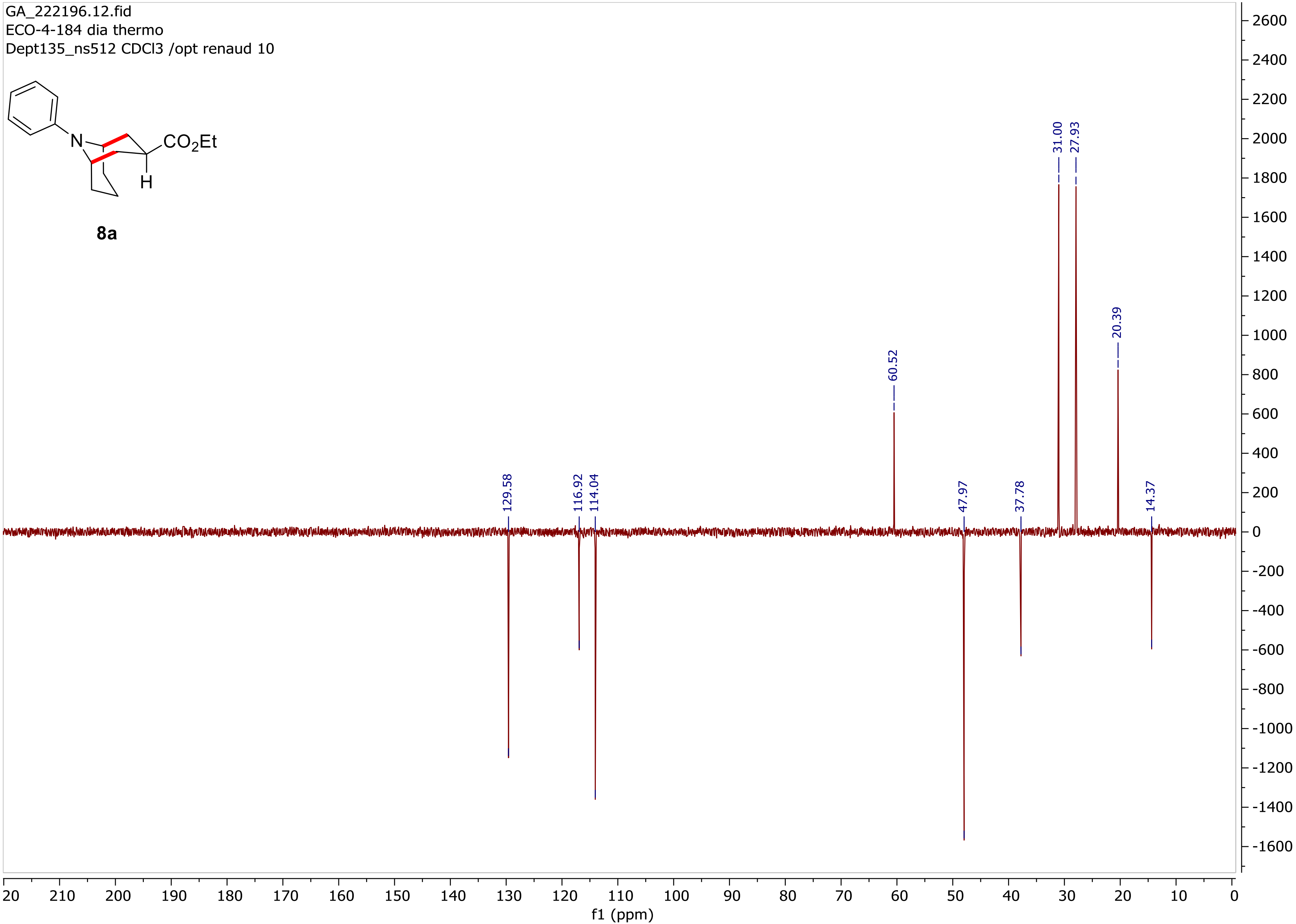

Ethyl 9-phenyl-9-azabicyclo[3.3.1]nonane-3-carboxylate (**8aβ**)

FT-IR, ATR-diamond

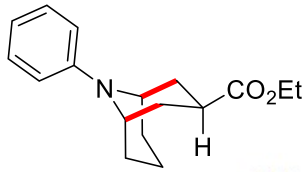

8a

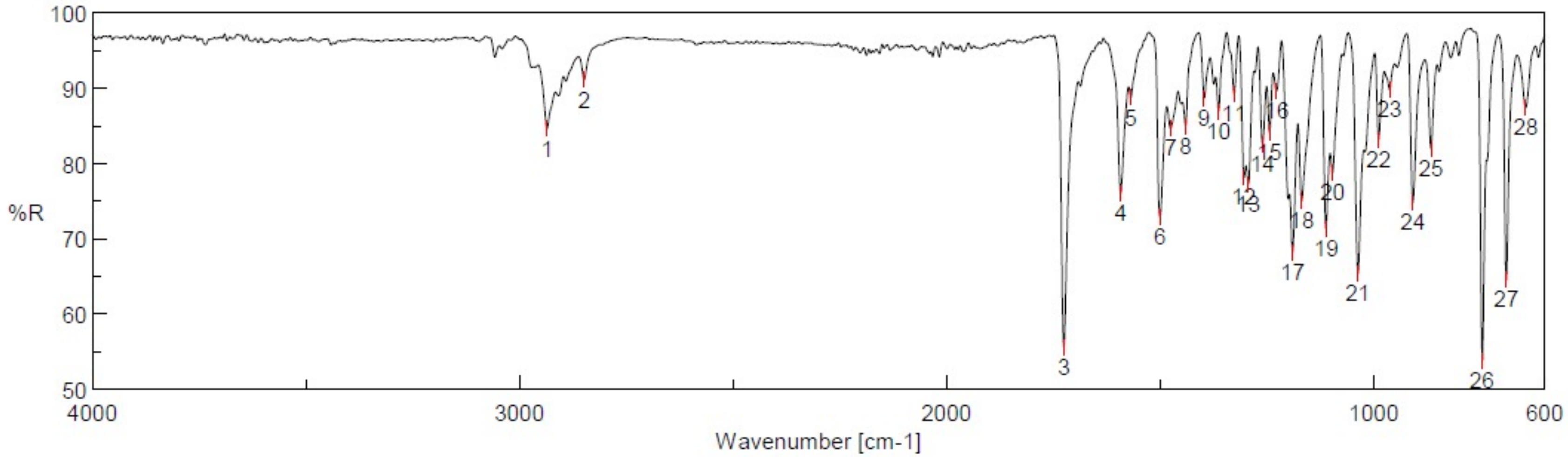

[Result of Peak Picking]

No.	Position	Intensity	No.	Position	Intensity	No.	Position	Intensity
1	2935.13	84.6093	2	2848.35	91.1081	3	1725.01	55.6013
4	1591.95	76.0434	5	1568.81	88.7333	6	1500.35	72.8549
7	1475.28	84.6326	8	1440.56	84.9306	9	1397.17	88.6919
10	1363.43	86.924	11	1326.79	89.2298	12	1303.64	78.0946
13	1294	77.2695	14	1260.25	82.5554	15	1243.86	84.0409
16	1227.47	89.5988	17	1189.86	68.1993	18	1168.65	74.9707
19	1111.76	71.3346	20	1096.33	78.7462	21	1036.55	65.3894
22	988.339	83.1901	23	963.269	89.8187	24	908.308	74.8481
25	865.882	81.9621	26	746.317	53.6914	27	689.427	64.5093
28	644.108	87.4125						

Ethyl 8-phenyl-8-azabicyclo[3.2.1]octane-3-carboxylate (**3aβ**)**_after epimerization α/β <1:20**

^{1}H-NMR (300 MHz, $CDCl_3$)

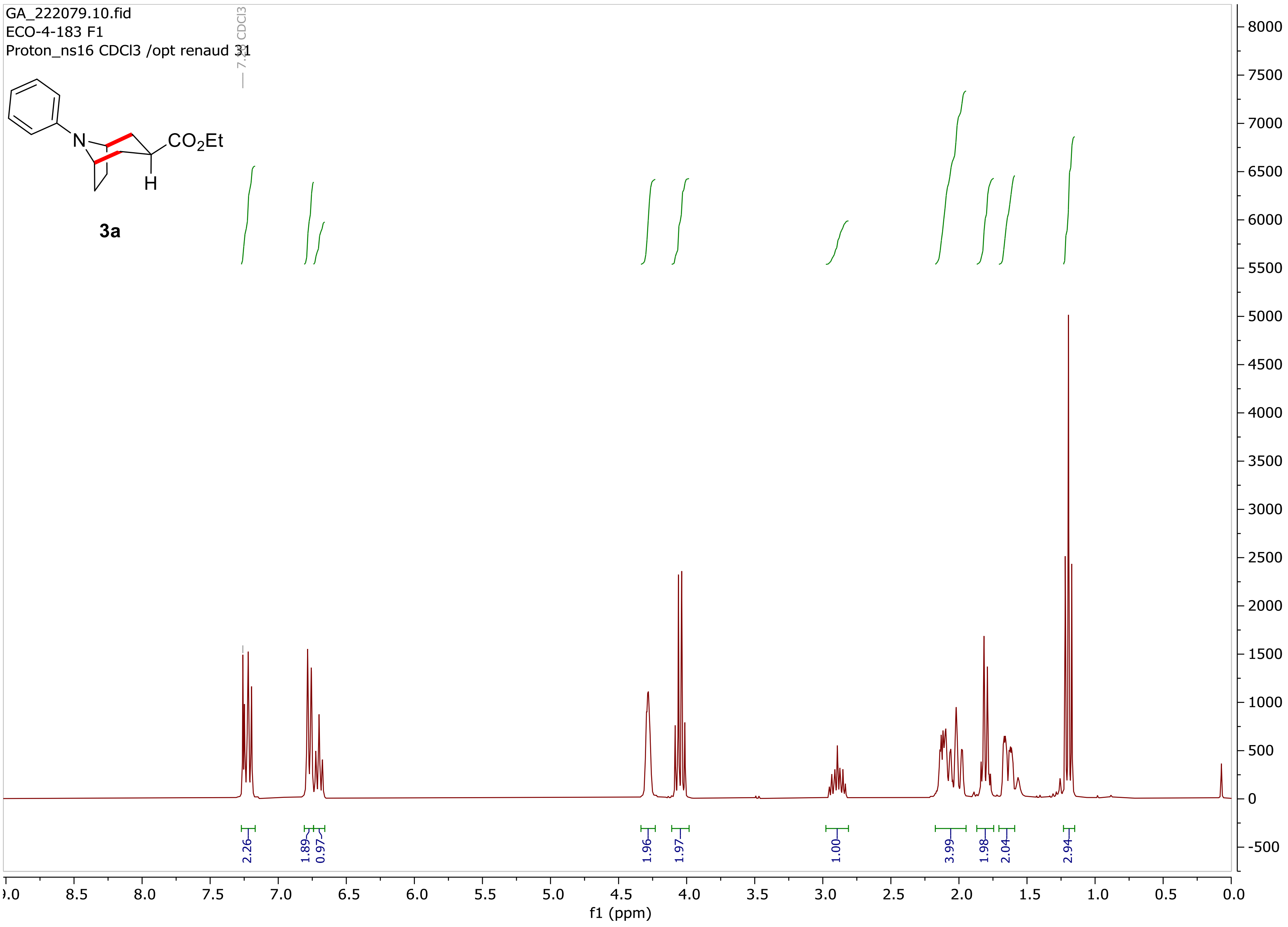

O8-benzyl O3-ethyl 8-azabicyclo[3.2.1]octane-3,8-dicarboxylate (**12**) **α/β 1:5**

^{1}H-NMR (300 MHz, $CDCl_3$)

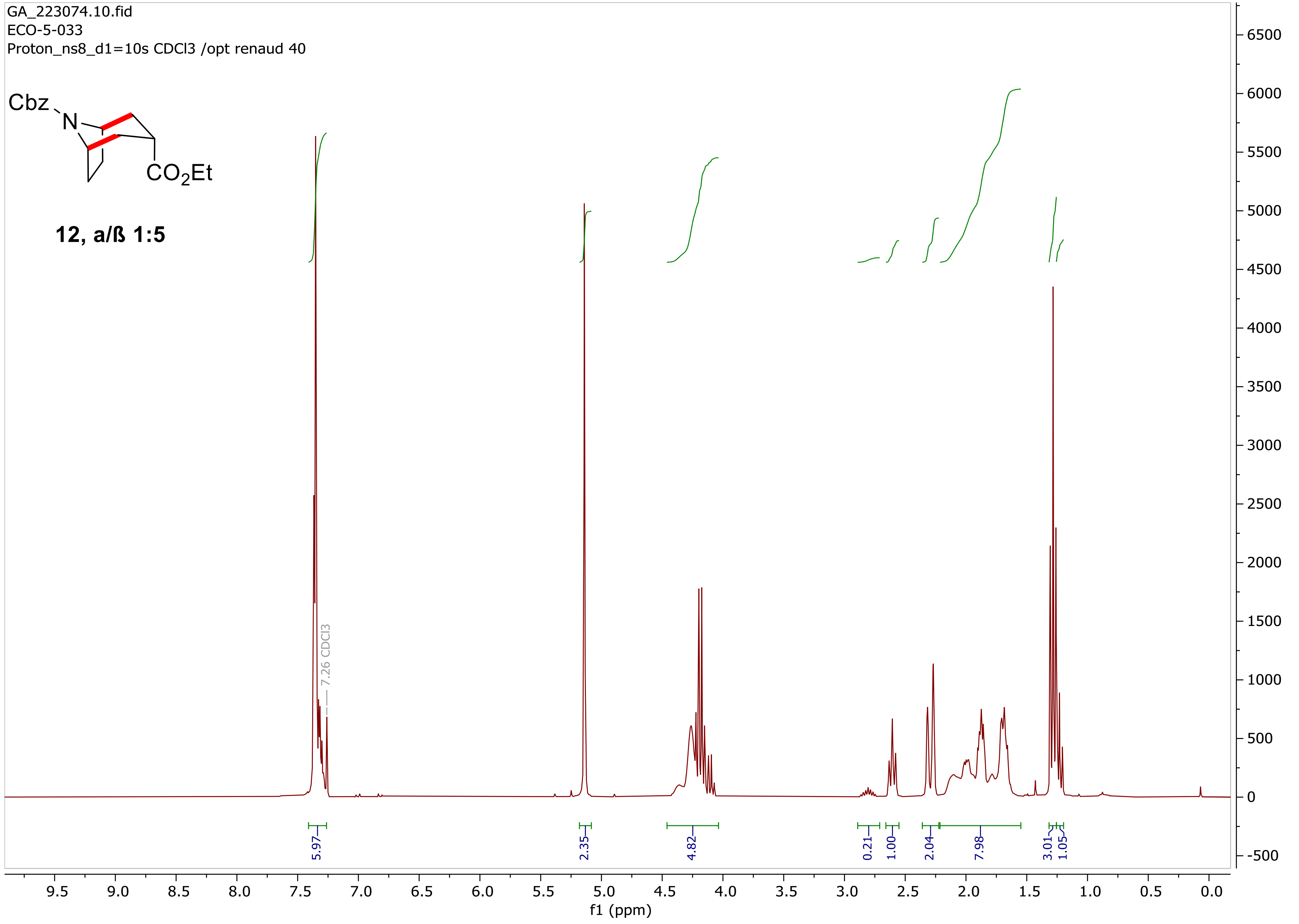

O8-benzyl O3-ethyl 8-azabicyclo[3.2.1]octane-3,8-dicarboxylate (**12**) **α/β 1:5**

^{1}H-NMR (300 MHz, $CDCl_3$)

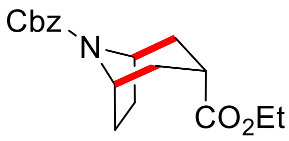

12, a/ß 1:5

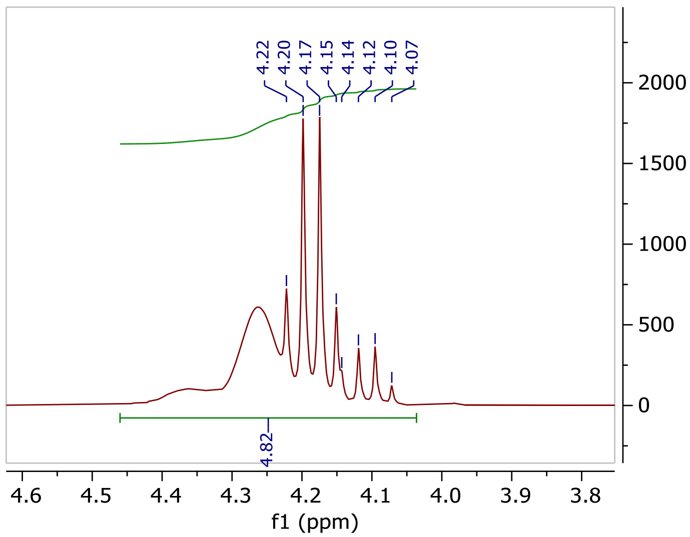

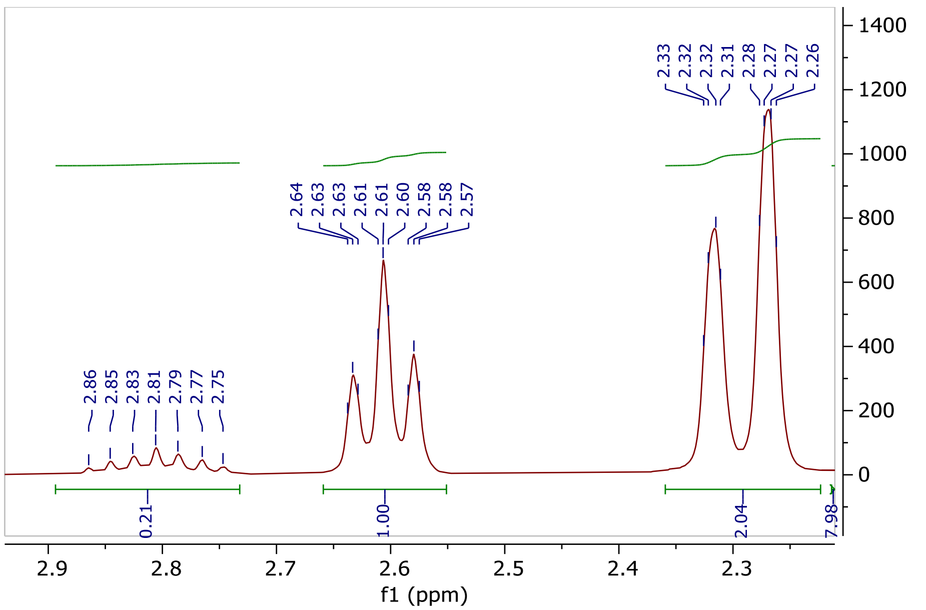

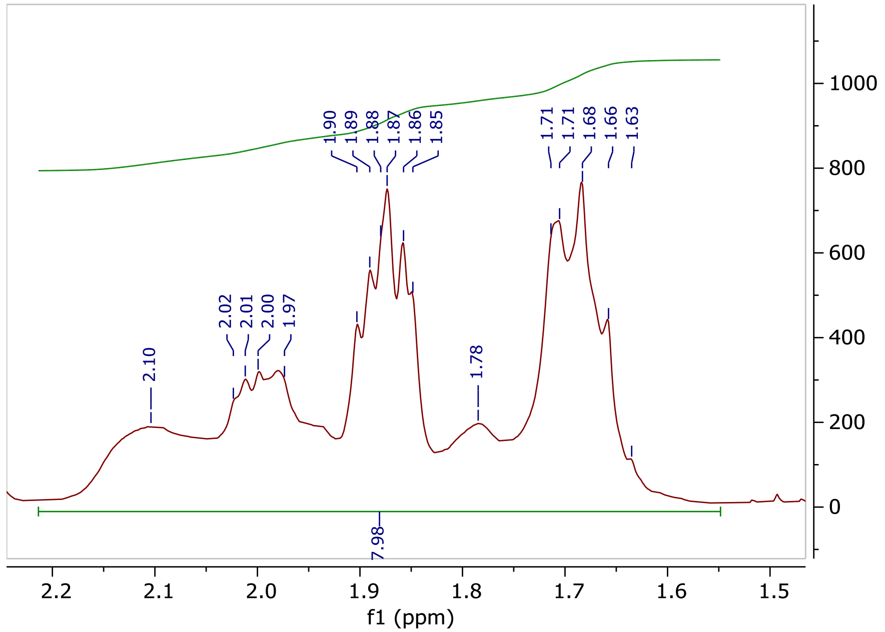

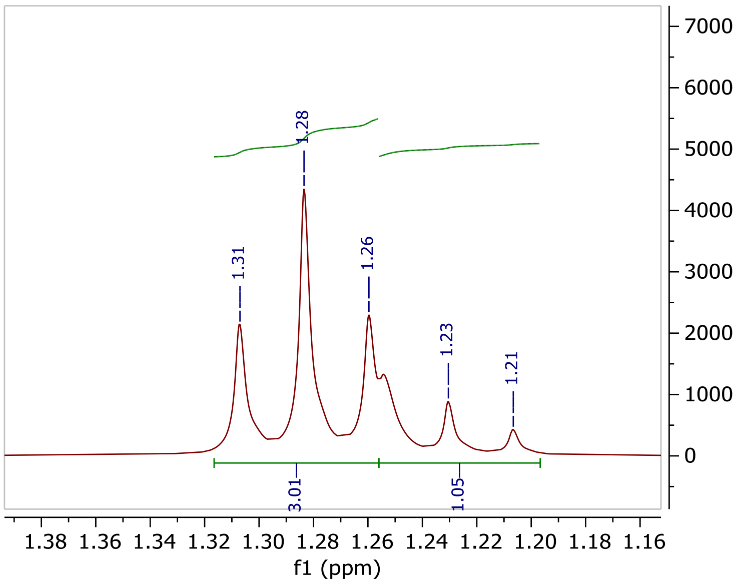

O8-benzyl O3-ethyl 8-azabicyclo[3.2.1]octane-3,8-dicarboxylate (**12**) **α/β 1:5**

^{13}C-NMR (75 MHz, $CDCl_3$)

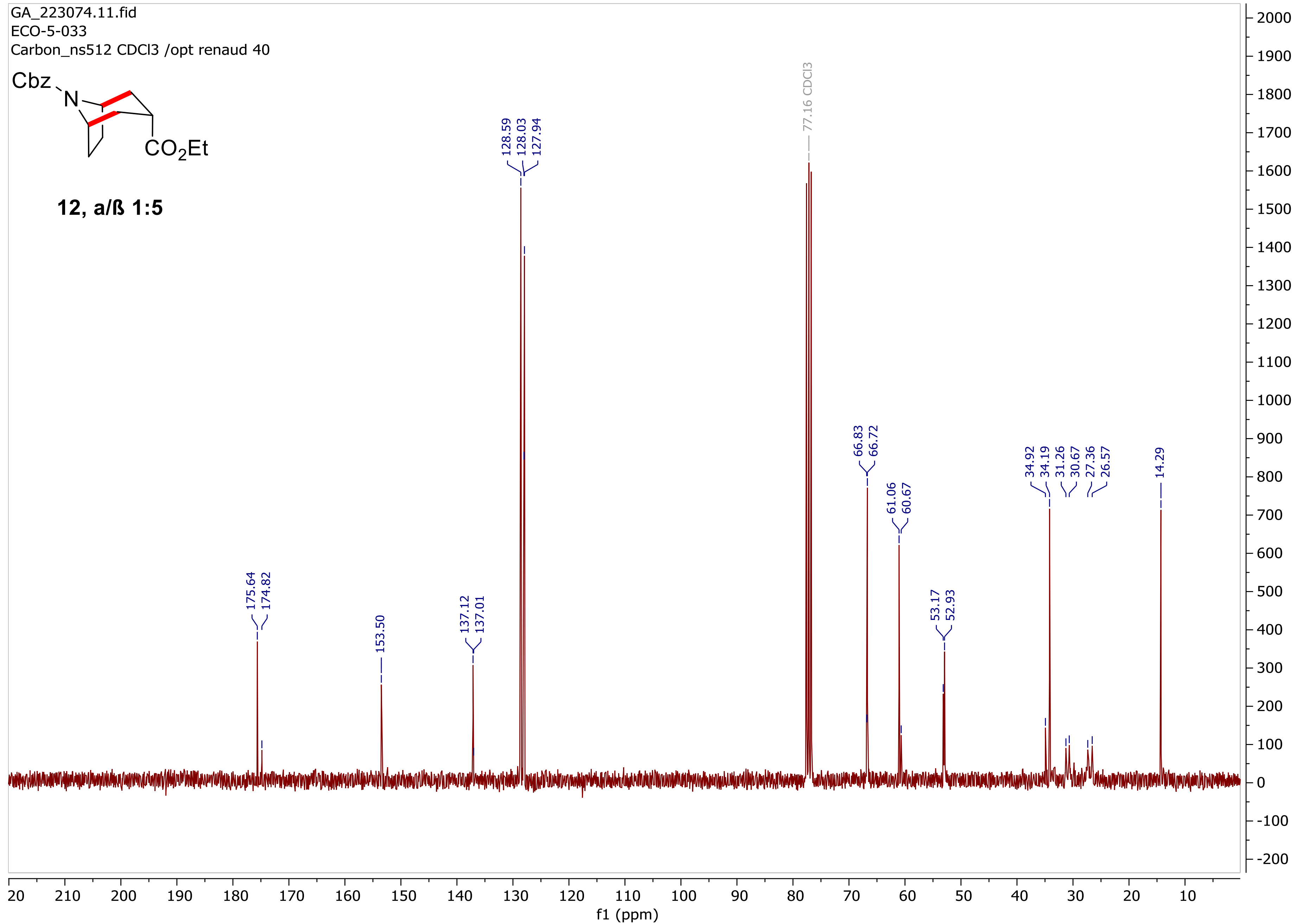

O8-benzyl O3-ethyl 8-azabicyclo[3.2.1]octane-3,8-dicarboxylate (**12**) **α/β 1:5**

^{13}C-NMR (75 MHz, $CDCl_3$)

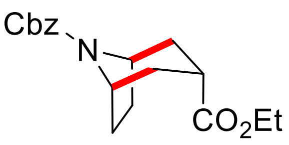

12, a/ß 1:5

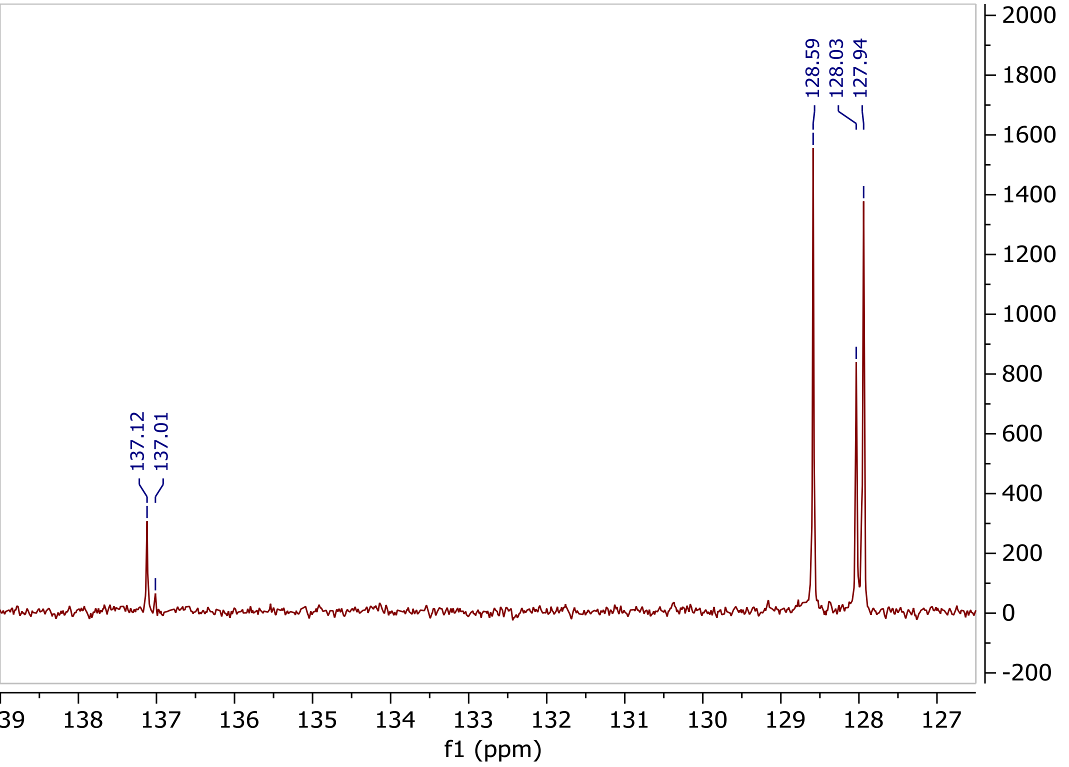

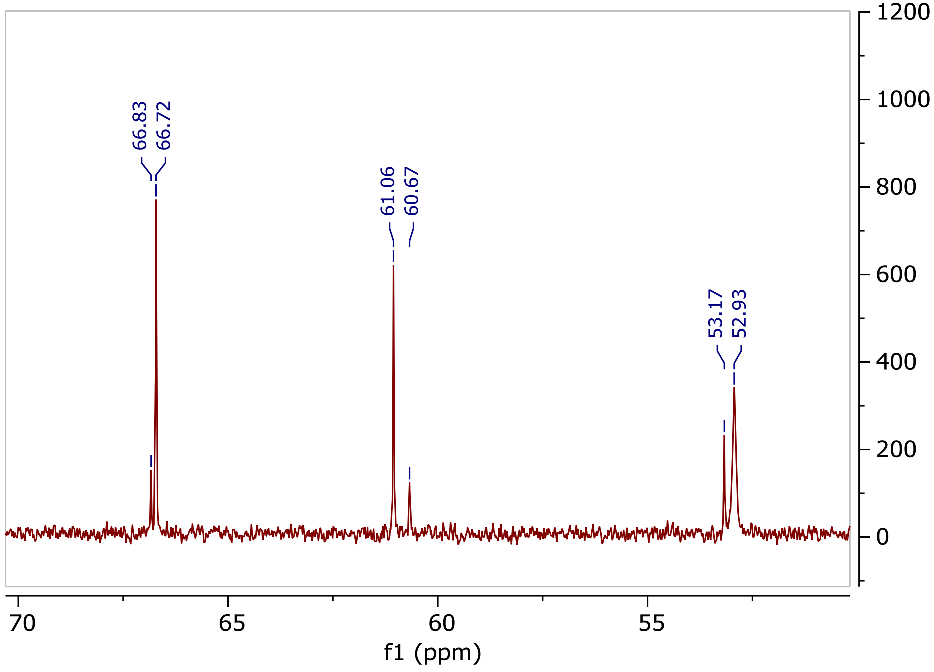

O8-benzyl O3-ethyl 8-azabicyclo[3.2.1]octane-3,8-dicarboxylate (**12**) **α/β 1:5**

^{13}C-NMR (75 MHz, $CDCl_3$)

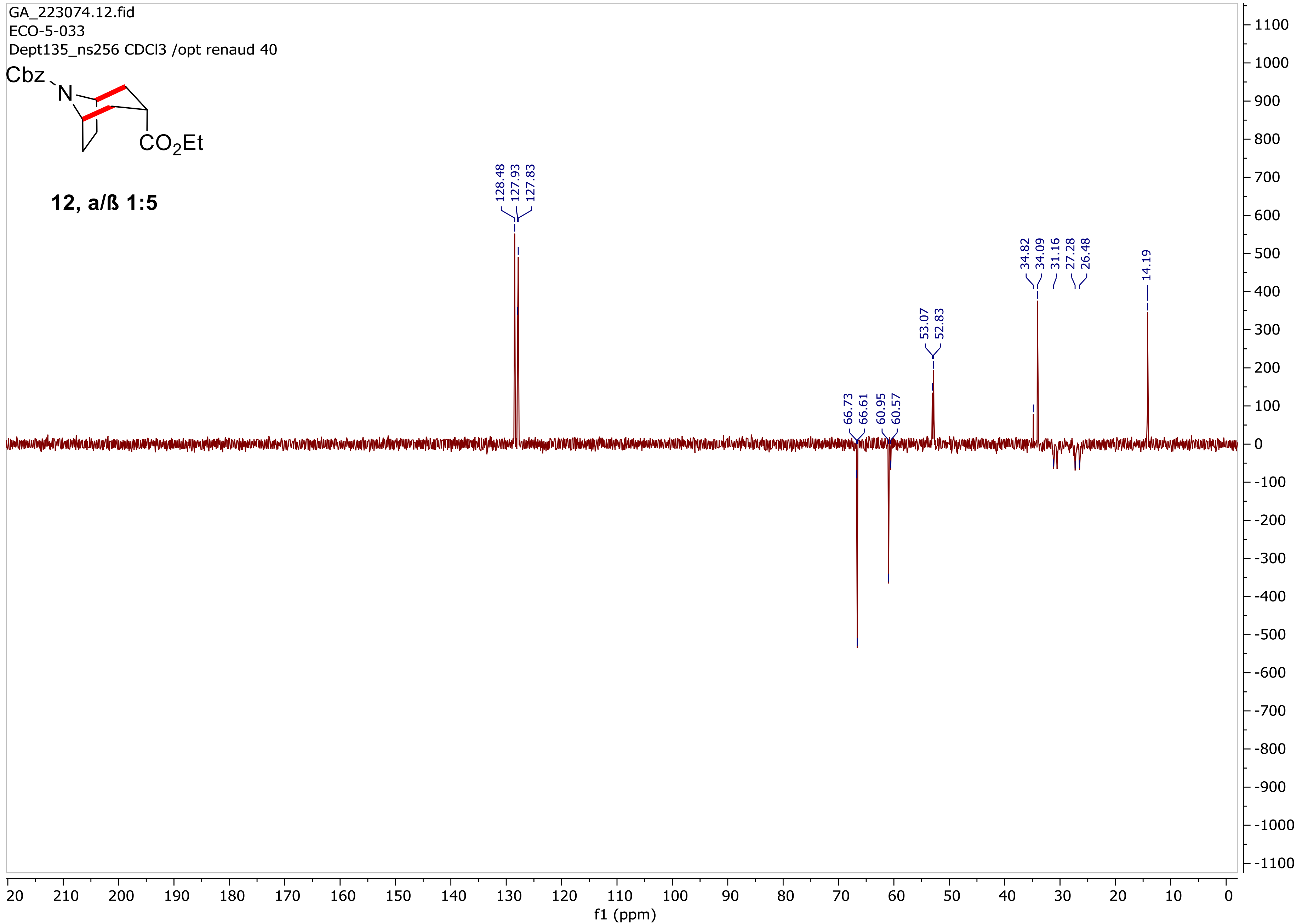

O8-benzyl O3-ethyl 8-azabicyclo[3.2.1]octane-3,8-dicarboxylate (**12**) **α/β 1:5**

^1H, ^1H-COSY NMR (400 MHz, $CDCl_3$)

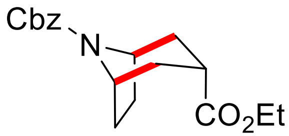

12, a/ß 1:5

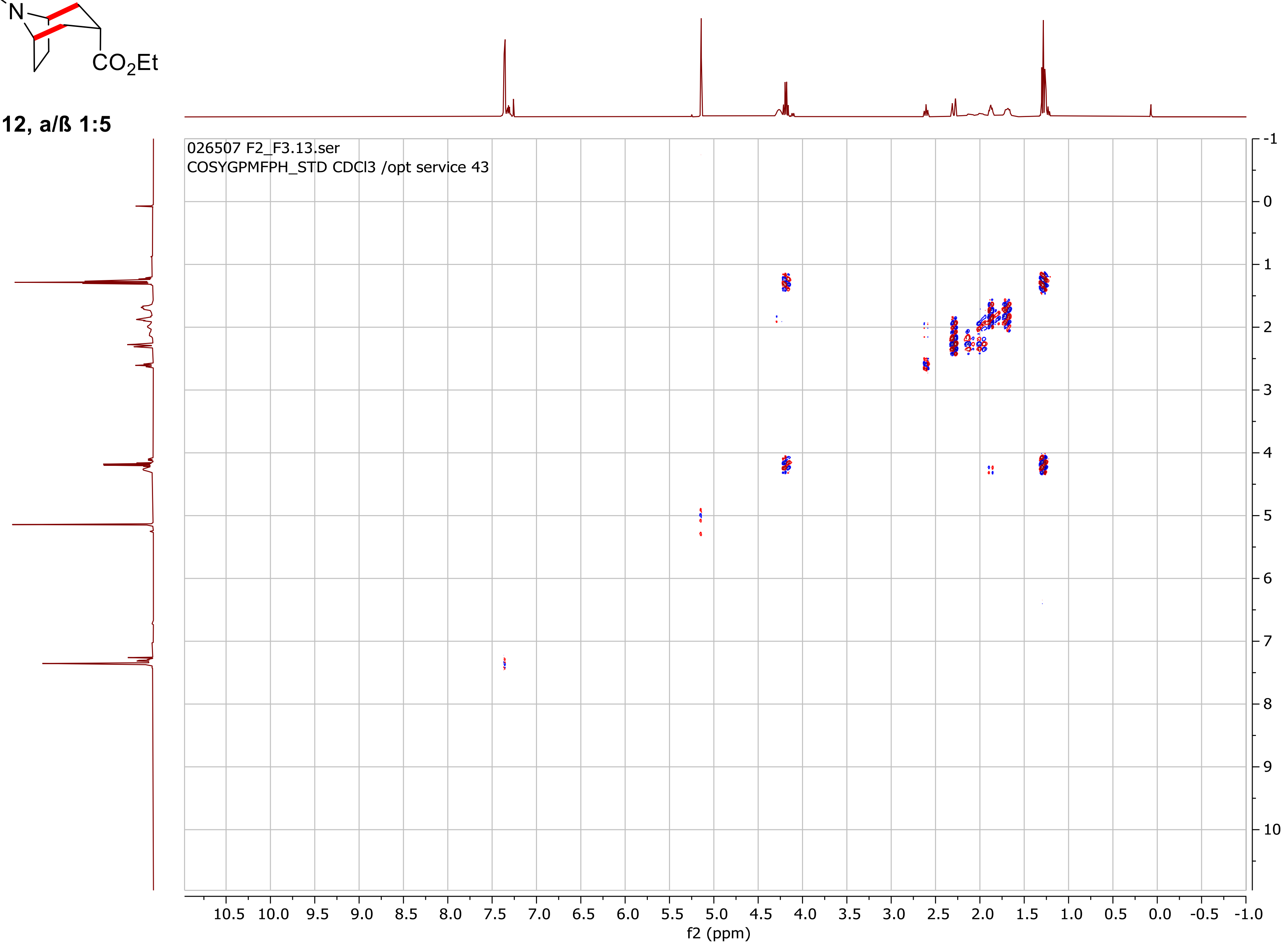

O8-benzyl O3-ethyl 8-azabicyclo[3.2.1]octane-3,8-dicarboxylate (**12**) **α/β 1:5**

^{1}H, ^{13}C-HSQC NMR (400 MHz, $CDCl_3$)

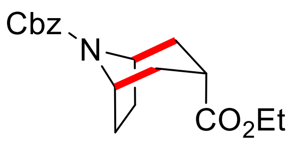

12, a/ß 1:5

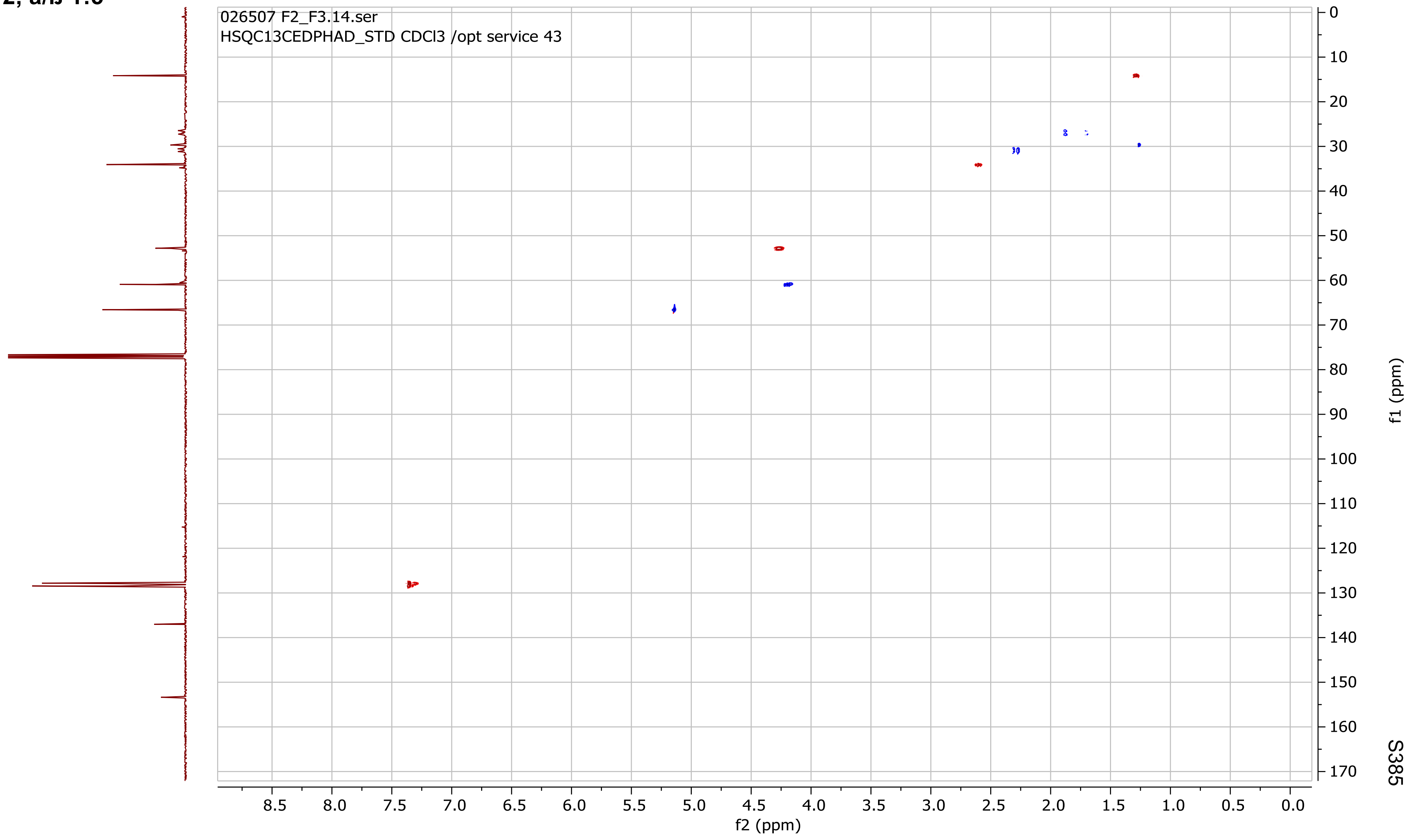

O8-benzyl O3-ethyl 8-azabicyclo[3.2.1]octane-3,8-dicarboxylate (**12**) **α/β 1:5**

^1H, ^{13}C-HSQC NMR (400 MHz, $CDCl_3$)

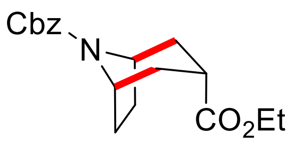

12, a/ß 1:5

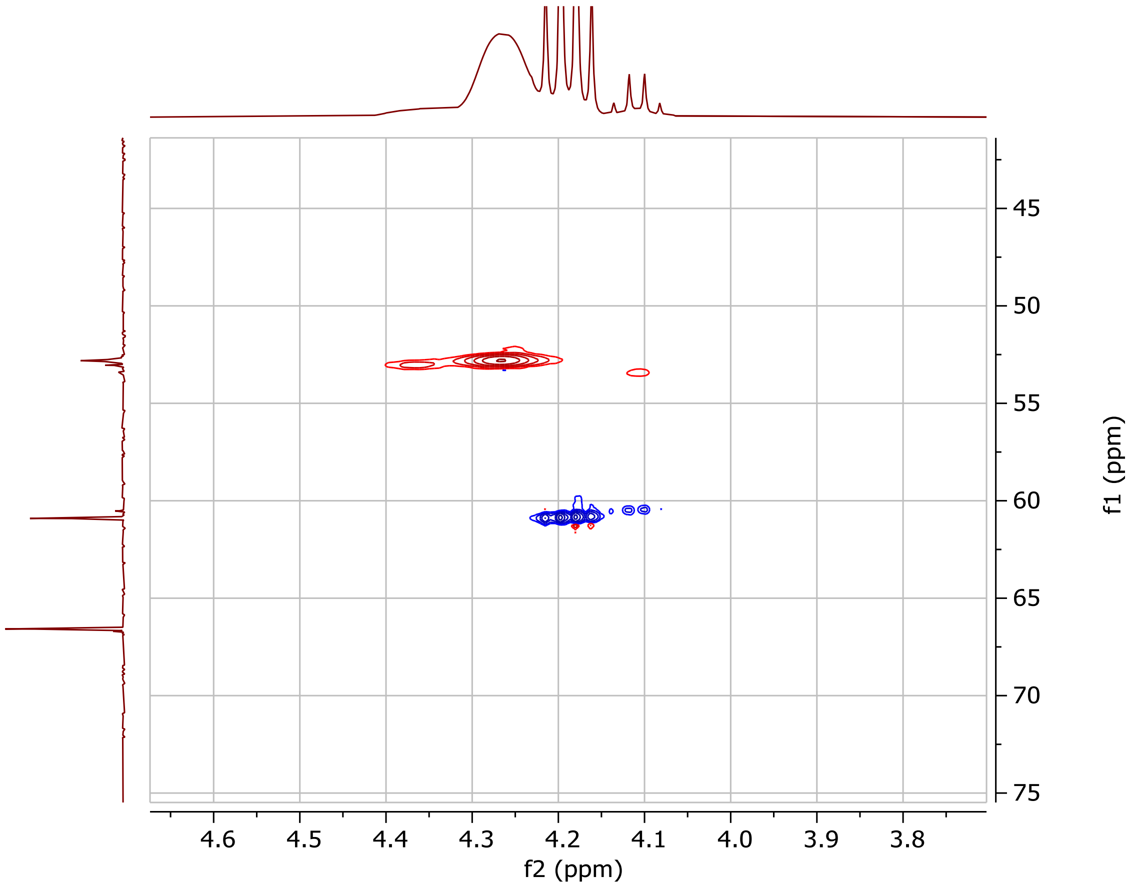

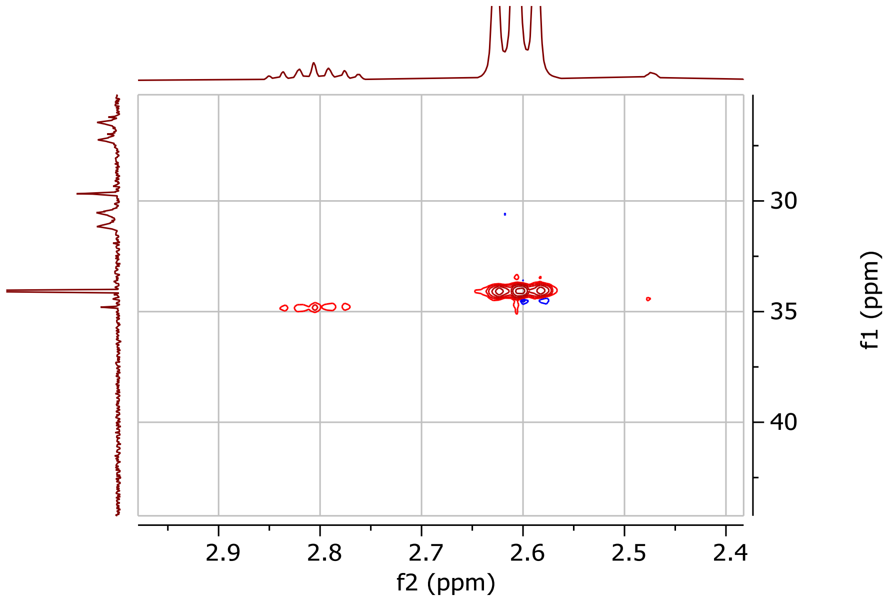

O8-benzyl O3-ethyl 8-azabicyclo[3.2.1]octane-3,8-dicarboxylate (**12**) **α/β 1:5**

^1H, ^{13}C-HMBC NMR (400 MHz, $CDCl_3$)

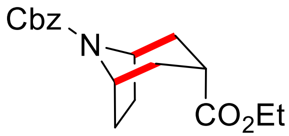

12, a/ß 1:5

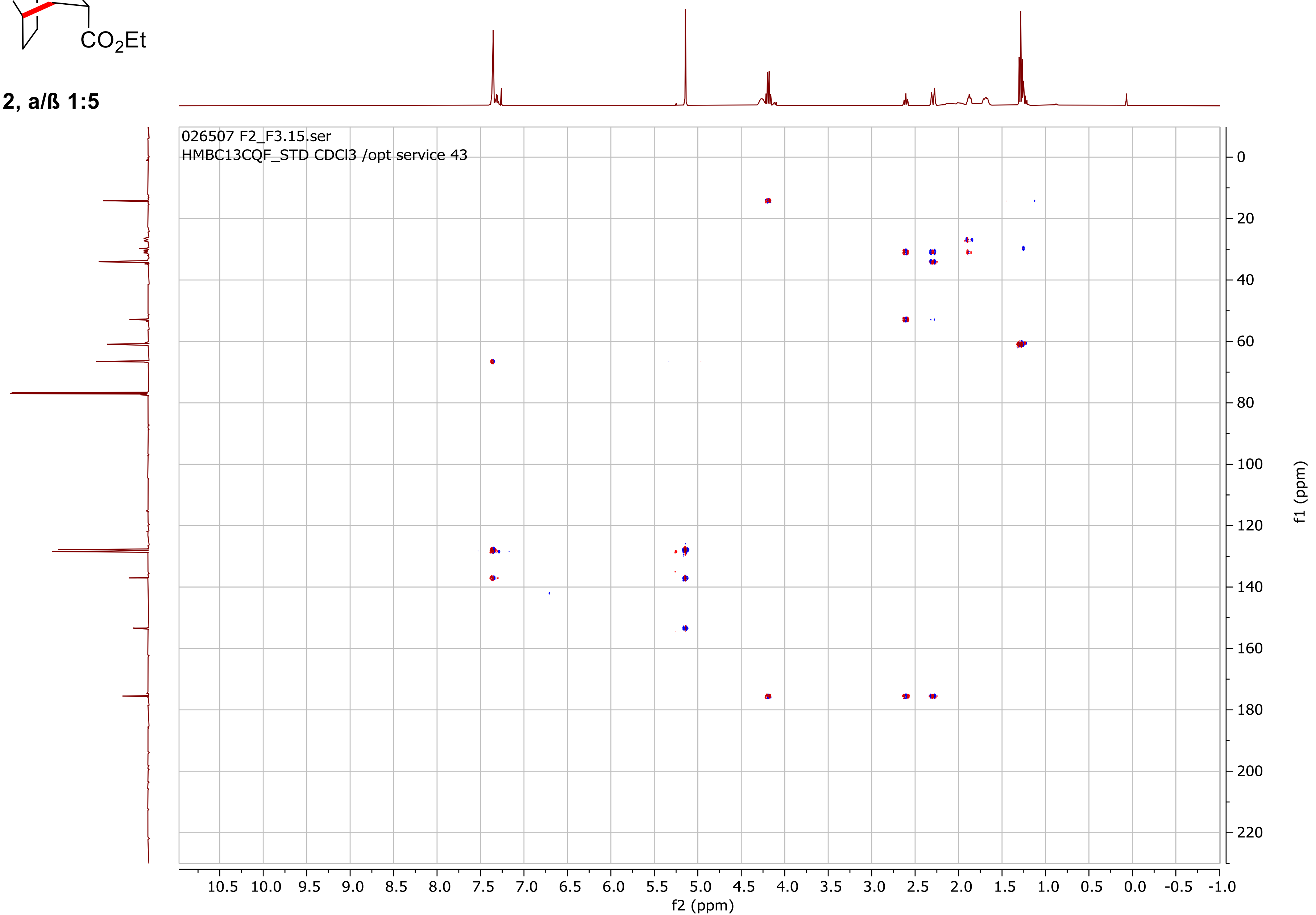

O8-benzyl O3-ethyl 8-azabicyclo[3.2.1]octane-3,8-dicarboxylate (**12**) **α/β 1:5**

FT-IR, ATR-diamond

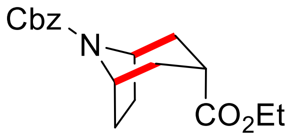

12, a/ß 1:5

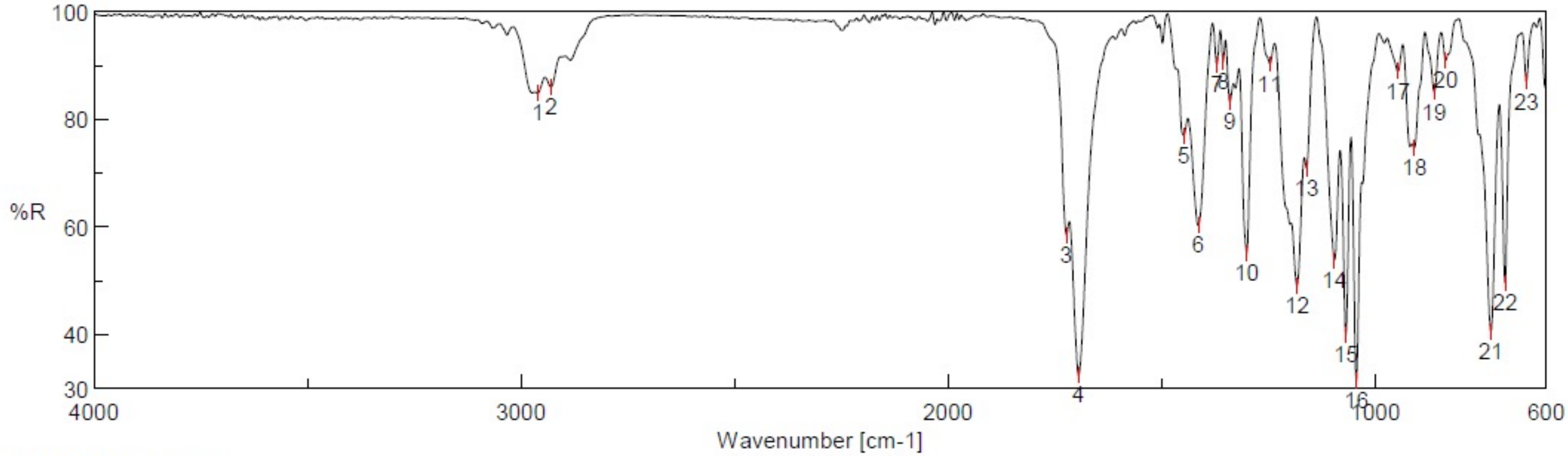

[Result of Peak Picking]

No.	Position	Intensity	No.	Position	Intensity	No.	Position	Intensity
1	2961.16	84.8157	2	2929.34	86.0179	3	1723.09	58.5253
4	1695.12	32.3882	5	1448.28	77.0077	6	1414.53	60.289
7	1371.14	90.1473	8	1355.71	90.7505	9	1340.28	83.1814
10	1300.75	55.1406	11	1246.75	90.2694	12	1183.11	49.0814
13	1160.94	70.9365	14	1095.37	53.8608	15	1068.37	39.8876
16	1044.26	31.2749	17	946.877	88.894	18	908.308	74.6922
19	862.025	85.05	20	835.026	90.9274	21	728.961	40.5364
22	696.177	49.5518	23	646.036	86.9769			

O8-benzyl O3-ethyl 8-azabicyclo[3.2.1]octane-3,8-dicarboxylate (**12**) **α/β 1:5**

^{1}H-NMR (400 MHz, $CDCl_3$. -45 °C)

026532.17.fid
T=-45C
H1_STD CDCl3 /opt service 42

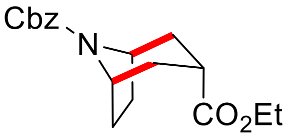

12, a/ß 1:5

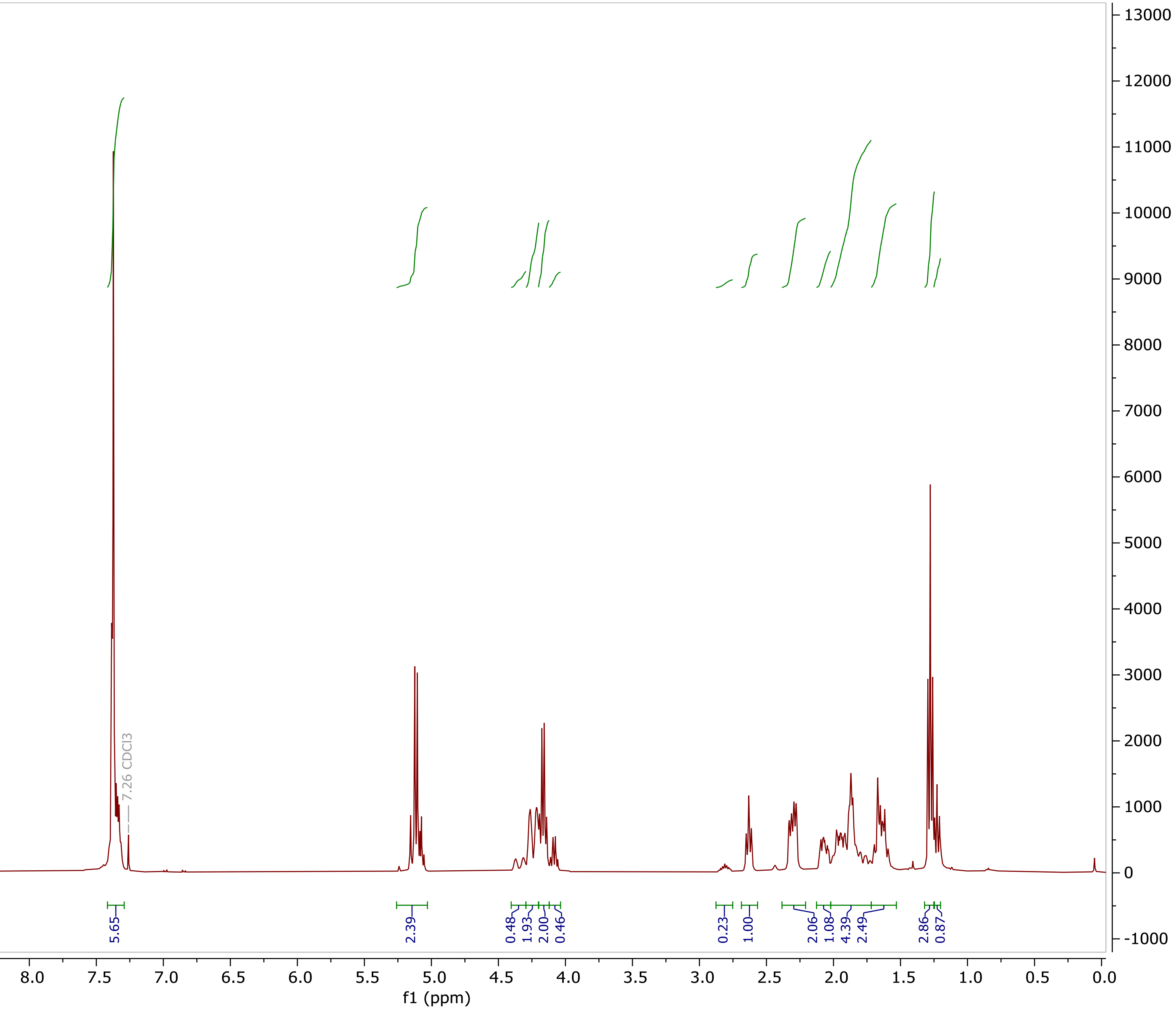

O8-benzyl O3-ethyl 8-azabicyclo[3.2.1]octane-3,8-dicarboxylate (**12**) **α/β 1:5**

^{1}H-NMR (400 MHz, $CDCl_3$. -45 °C)

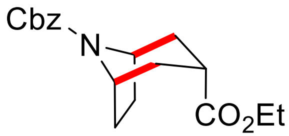

12, a/ß 1:5

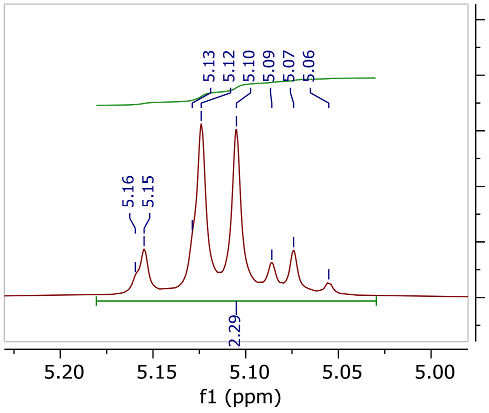

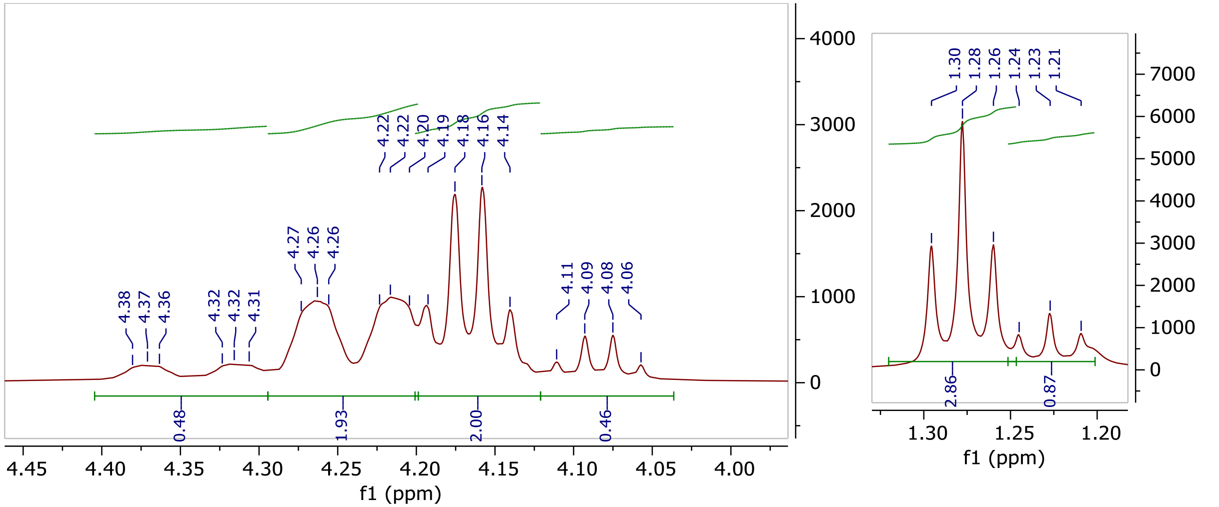

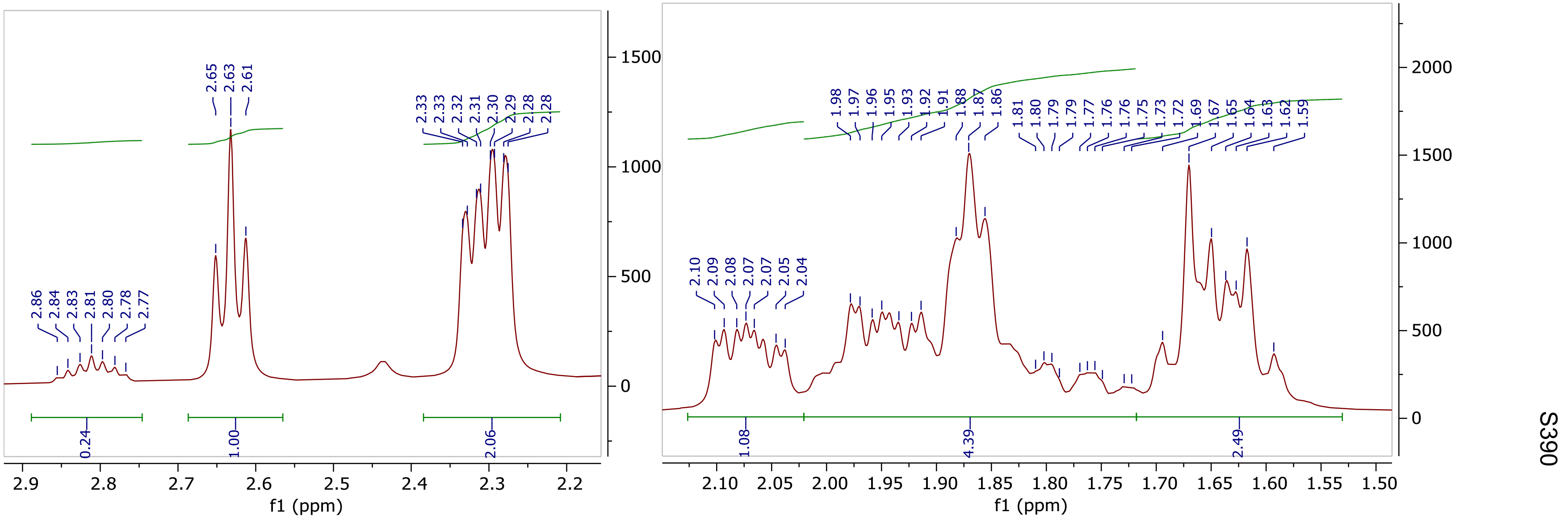

O8-benzyl O3-ethyl 8-azabicyclo[3.2.1]octane-3,8-dicarboxylate (**12**) **α/β 1:5**

^{13}C-NMR (101 MHz, $CDCl_3$. -45 °C)

026532.18.fid
T=-45C
C13CPD_STD CDCl3 /opt service 42

12, a/ß 1:5

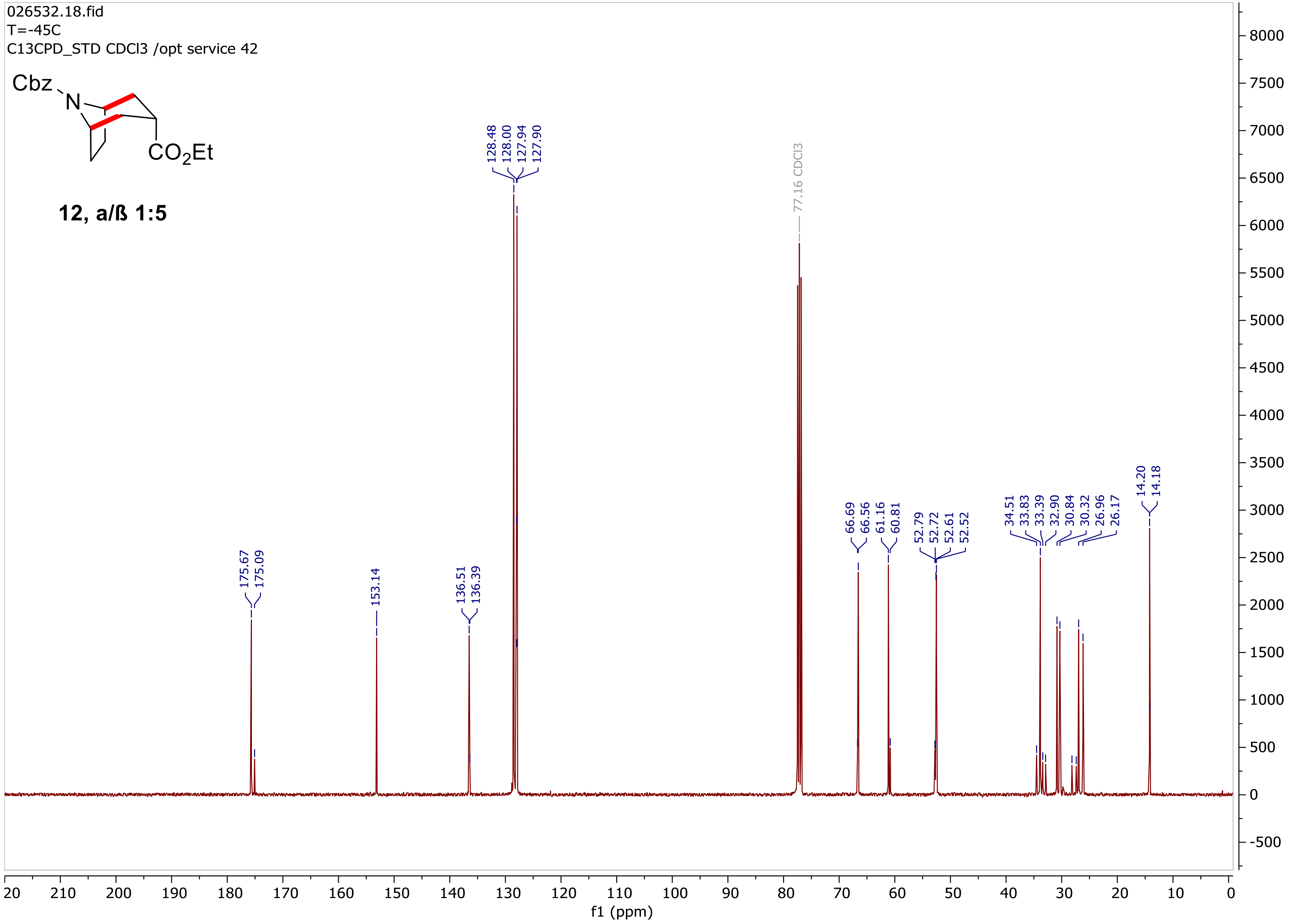

O8-benzyl O3-ethyl 8-azabicyclo[3.2.1]octane-3,8-dicarboxylate (**12**) **α/β 1:5**

^{13}C-NMR (101 MHz, $CDCl_3$. -45 °C)

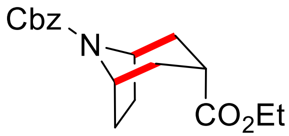

12, a/ß 1:5

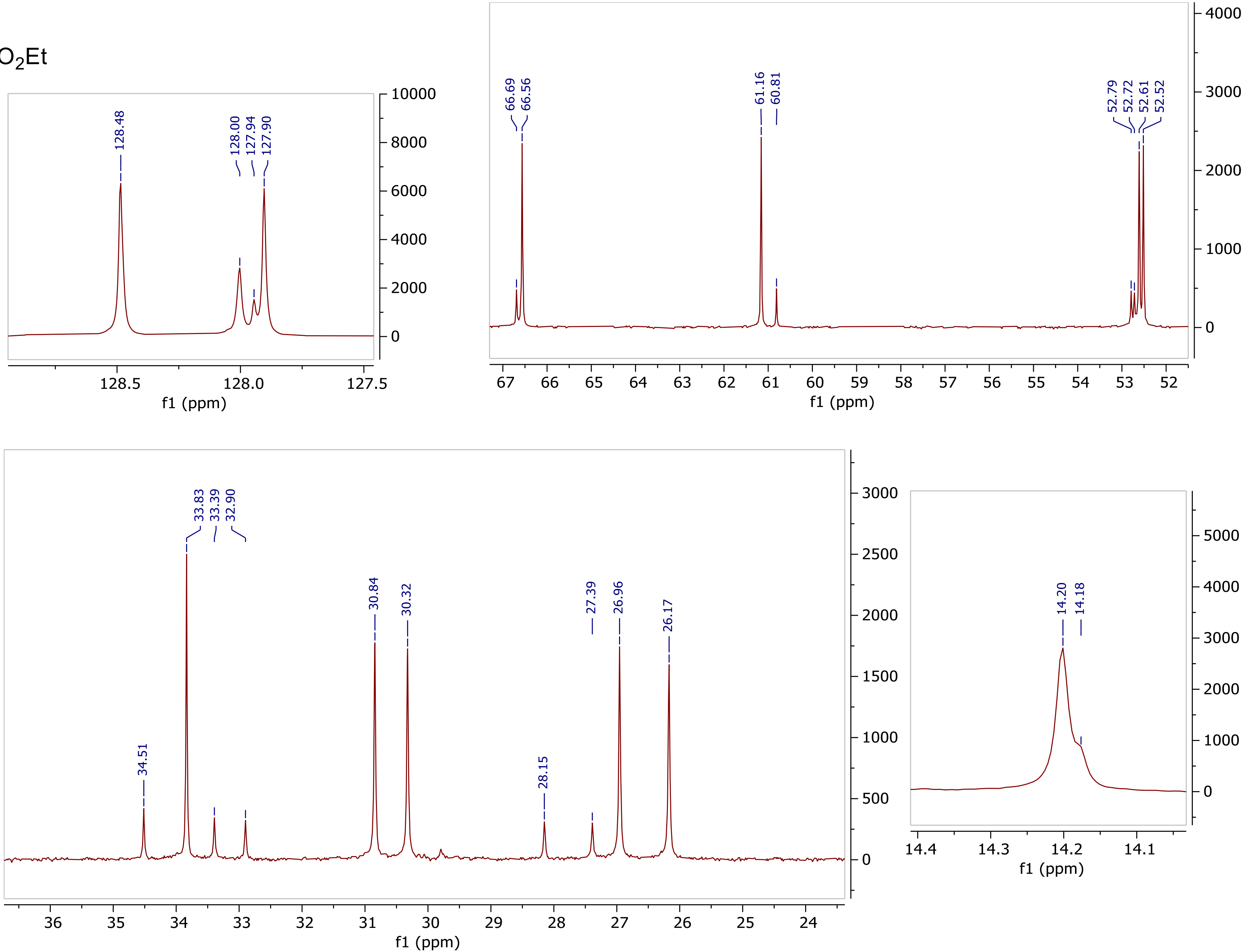

O8-benzyl O3-ethyl 8-azabicyclo[3.2.1]octane-3,8-dicarboxylate (**12**) **α/β 1:5**

^{13}C-NMR (101 MHz, $CDCl_3$. -45 °C)

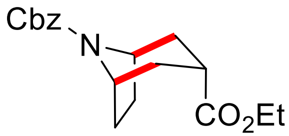

12, a/ß 1:5

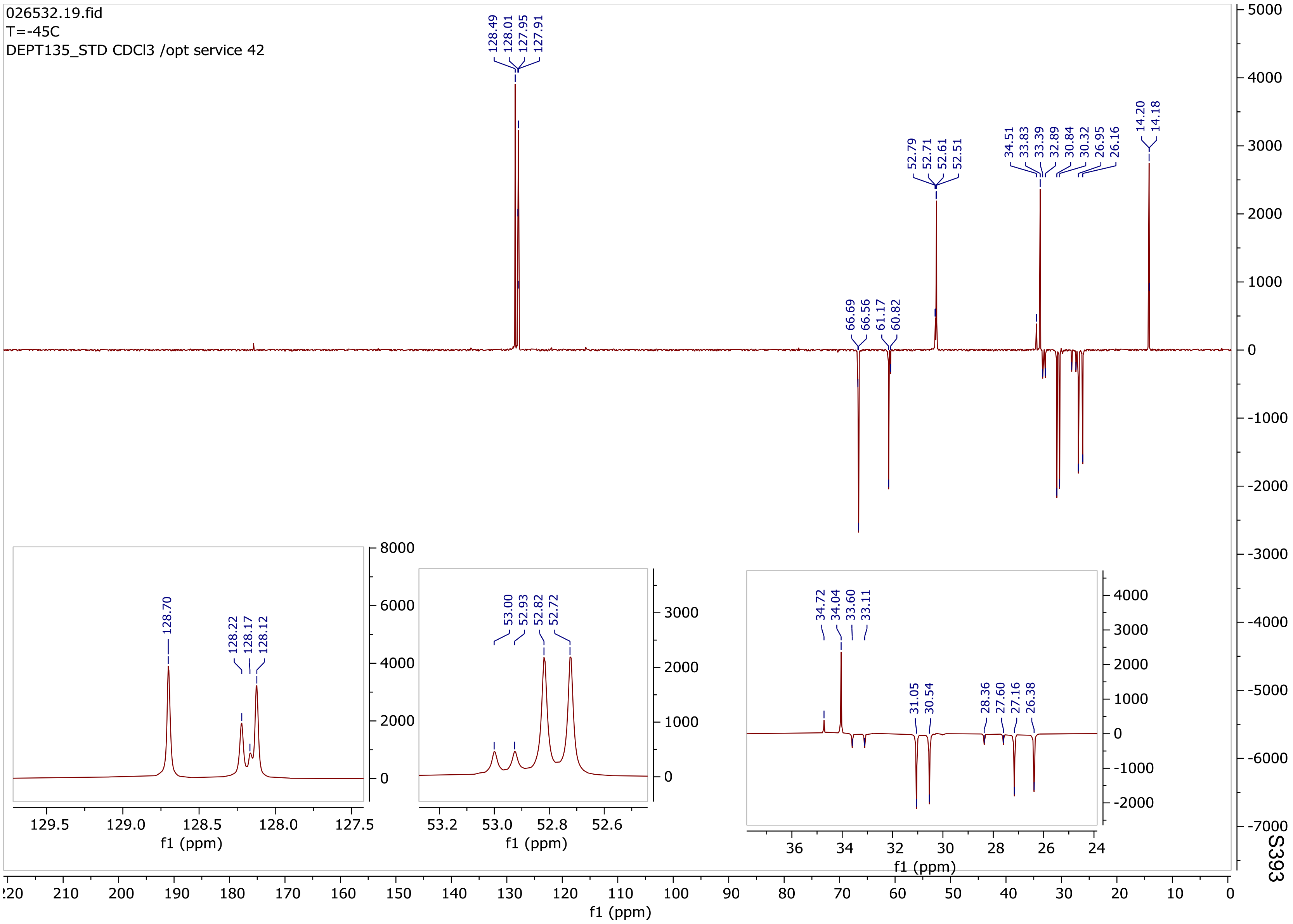

O8-benzyl O3-ethyl 8-azabicyclo[3.2.1]octane-3,8-dicarboxylate (**12**) **α/β 1:5**

^{1}H, ^{1}H-COSY NMR (101MHz, $CDCl_3$, -45 °C)

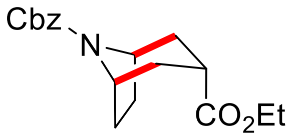

12, a/ß 1:5

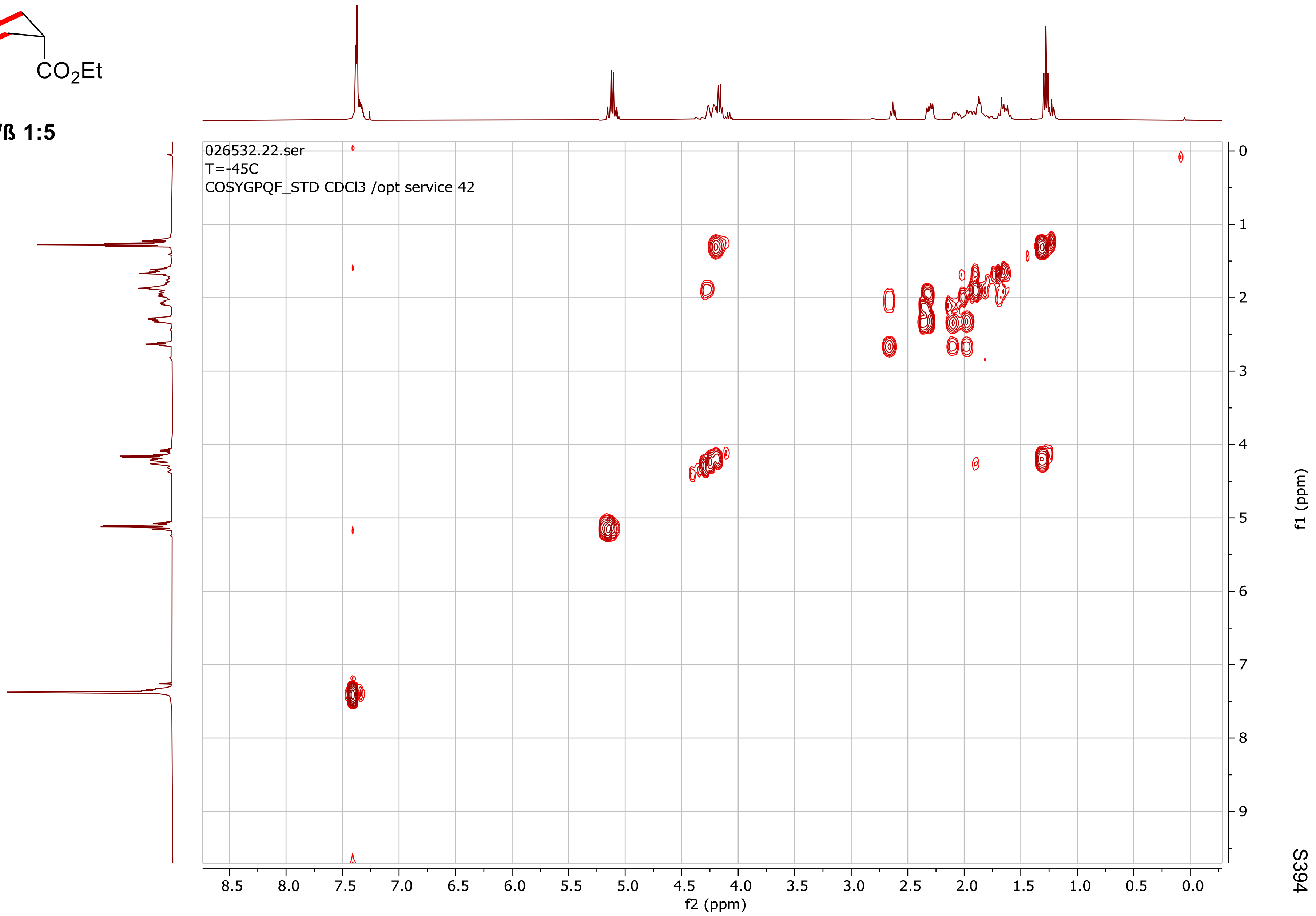

O8-benzyl O3-ethyl 8-azabicyclo[3.2.1]octane-3,8-dicarboxylate (**12**) **α/β 1:5**

^{1}H, ^{13}C-HSQC NMR (101MHz, $CDCl_3$, -45 °C)

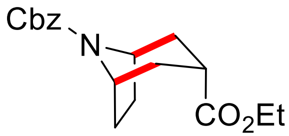

12, a/ß 1:5

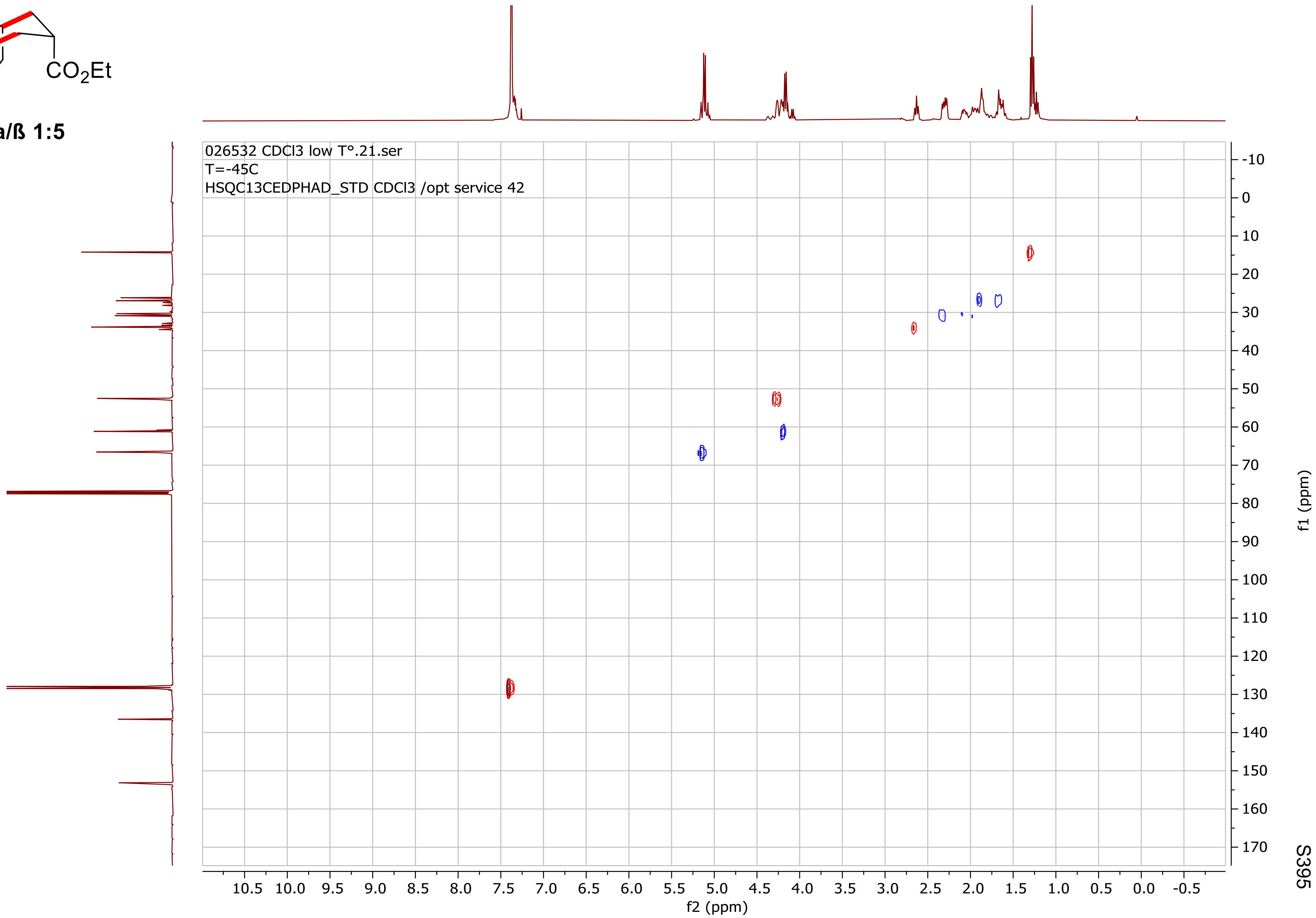

O8-benzyl O3-ethyl 8-azabicyclo[3.2.1]octane-3,8-dicarboxylate (**12**) **α/β 1:5**

^{1}H, ^{13C}-HSQC NMR (101MHz, $CDCl_3$, -45 °C)

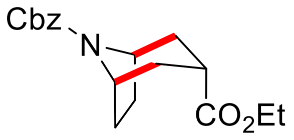

12, a/ß 1:5

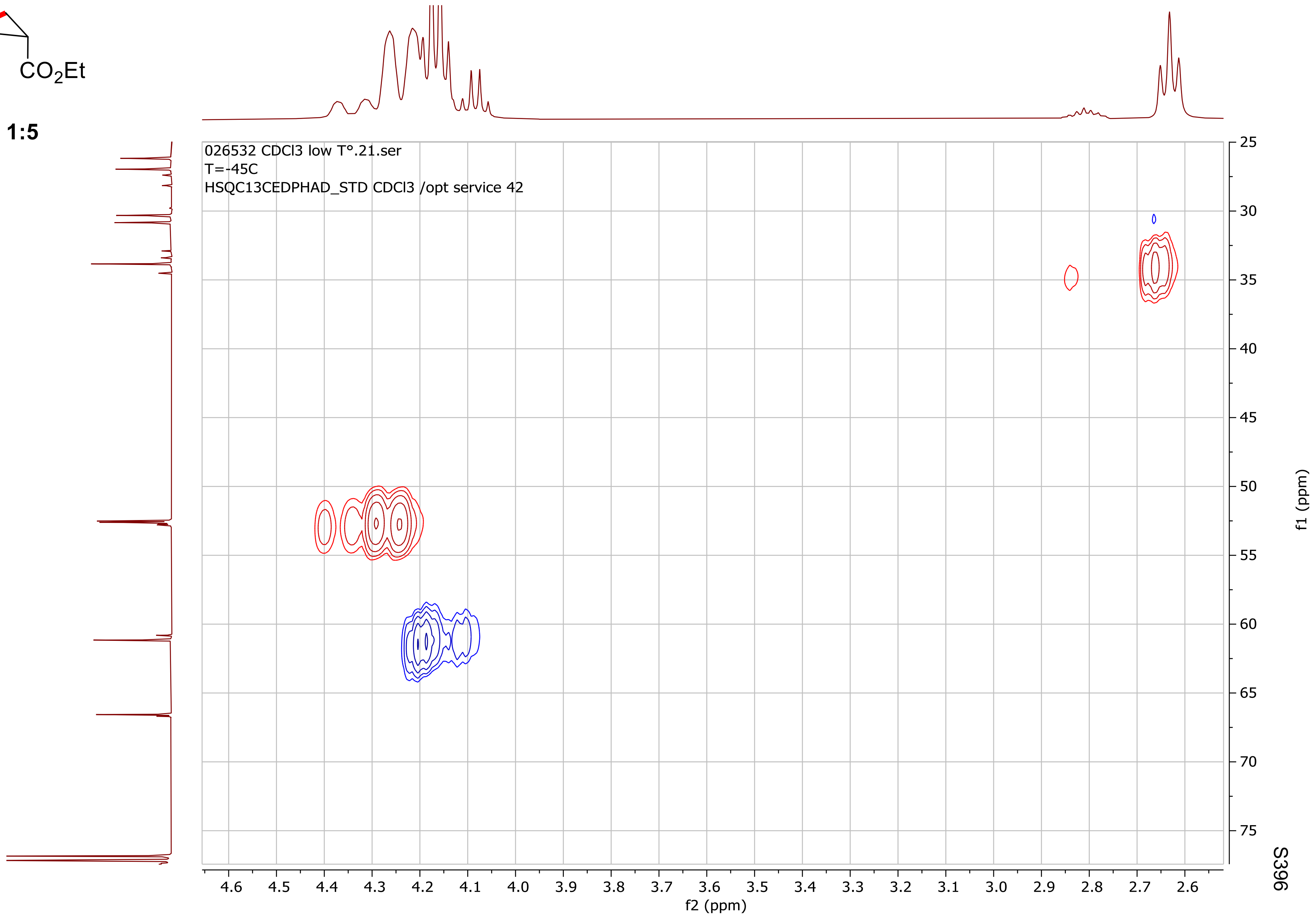

O9-benzyl O3-ethyl 9-azabicyclo[3.3.1]nonane-3,9-dicarboxylate (**13**) **α/β >20:1**

1H-NMR (400 MHz, CDCl3)

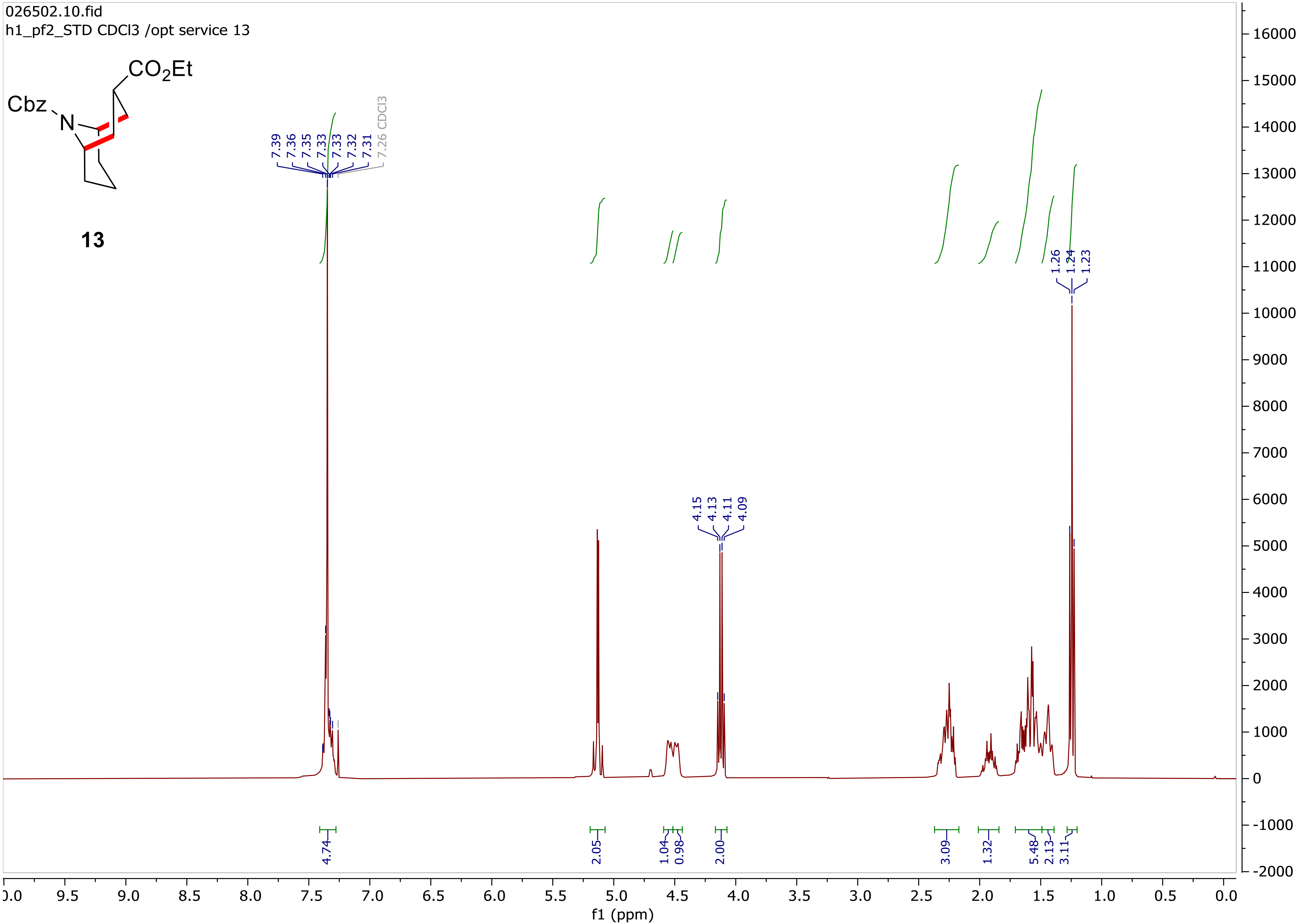

O9-benzyl O3-ethyl 9-azabicyclo[3.3.1]nonane-3,9-dicarboxylate (**13α**)

^{1}H-NMR (300 MHz, $CDCl_3$)

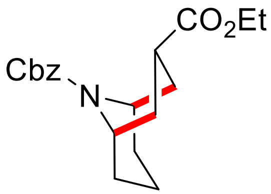

13

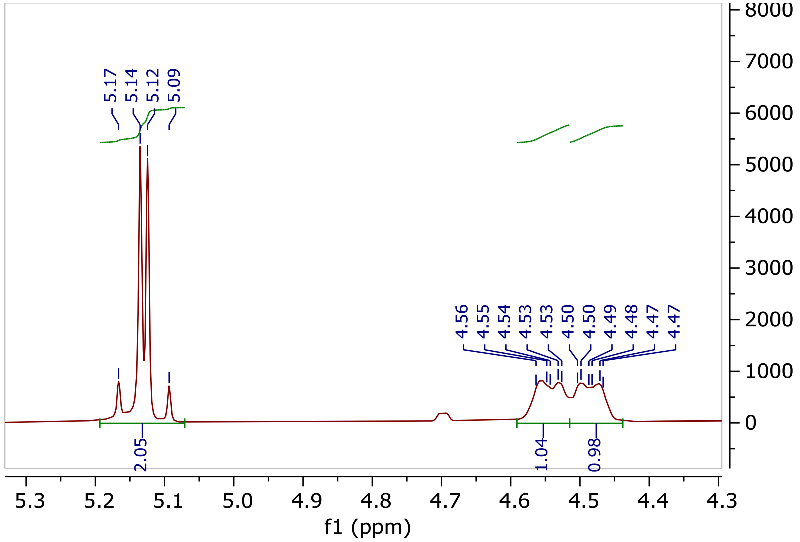

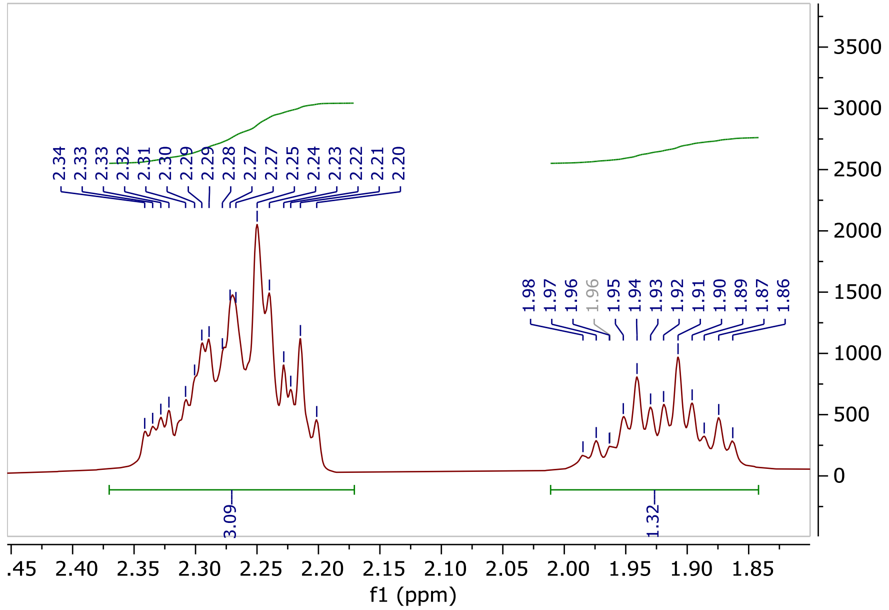

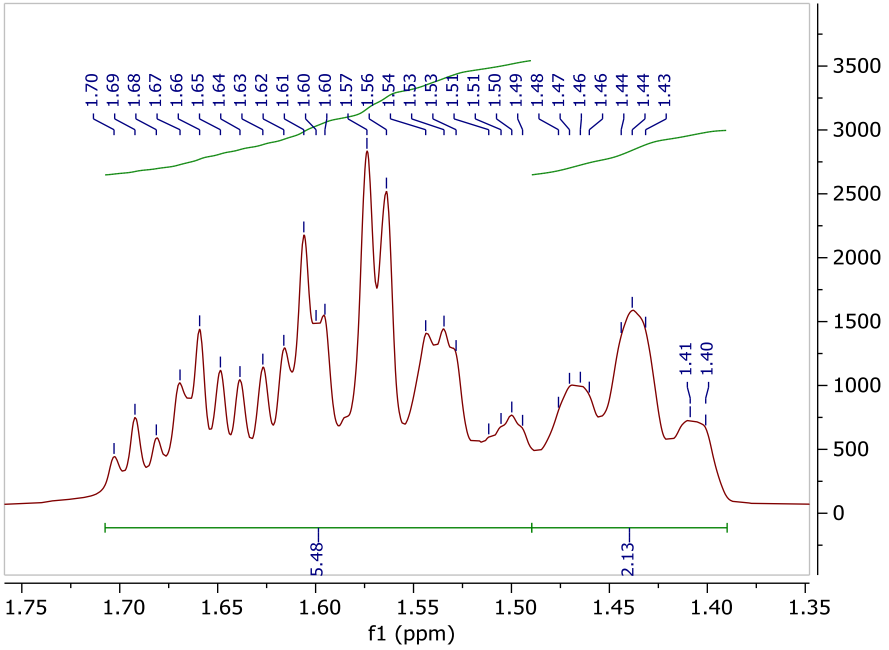

O9-benzyl O3-ethyl 9-azabicyclo[3.3.1]nonane-3,9-dicarboxylate (**13α**)

^{13}C-NMR (75 MHz, $CDCl_3$)

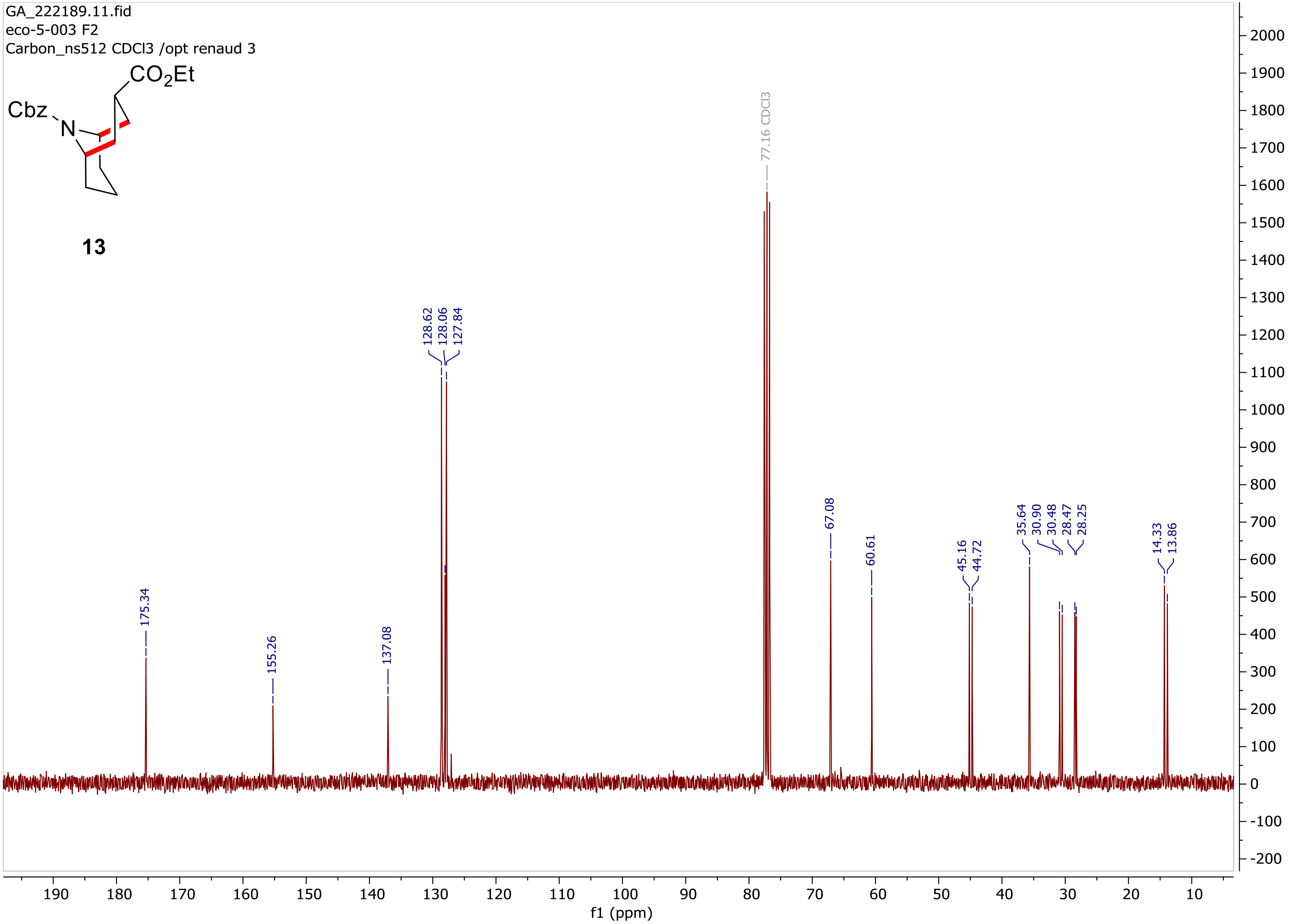

O9-benzyl O3-ethyl 9-azabicyclo[3.3.1]nonane-3,9-dicarboxylate (**13α**)

^{13}C-NMR (75 MHz, $CDCl_3$)

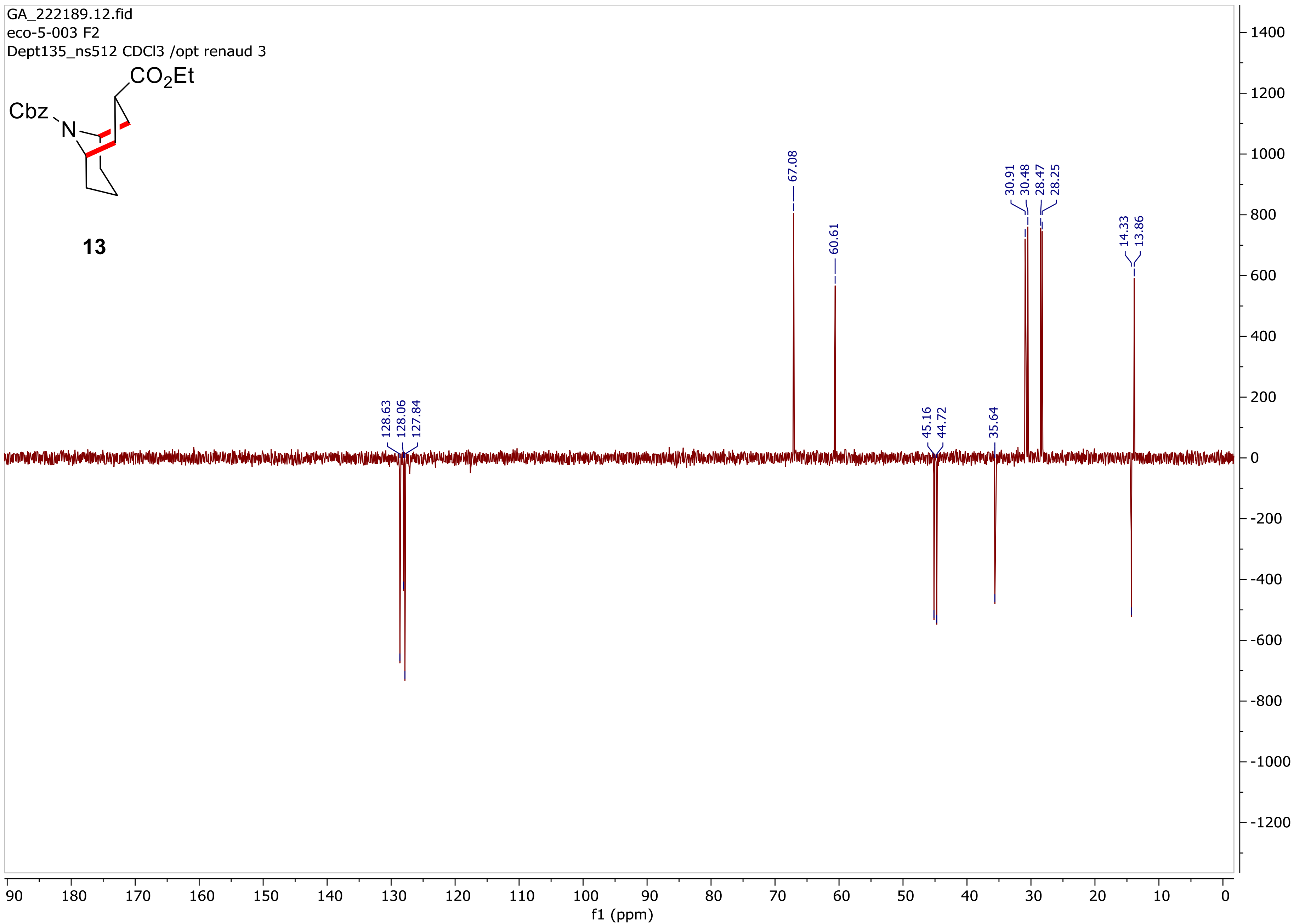

O9-benzyl O3-ethyl 9-azabicyclo[3.3.1]nonane-3,9-dicarboxylate (**13α**)

^1H, ^1H-COSY NMR (300 MHz, $CDCl_3$)

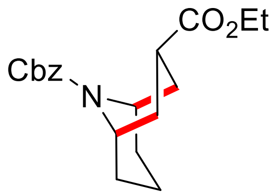

13

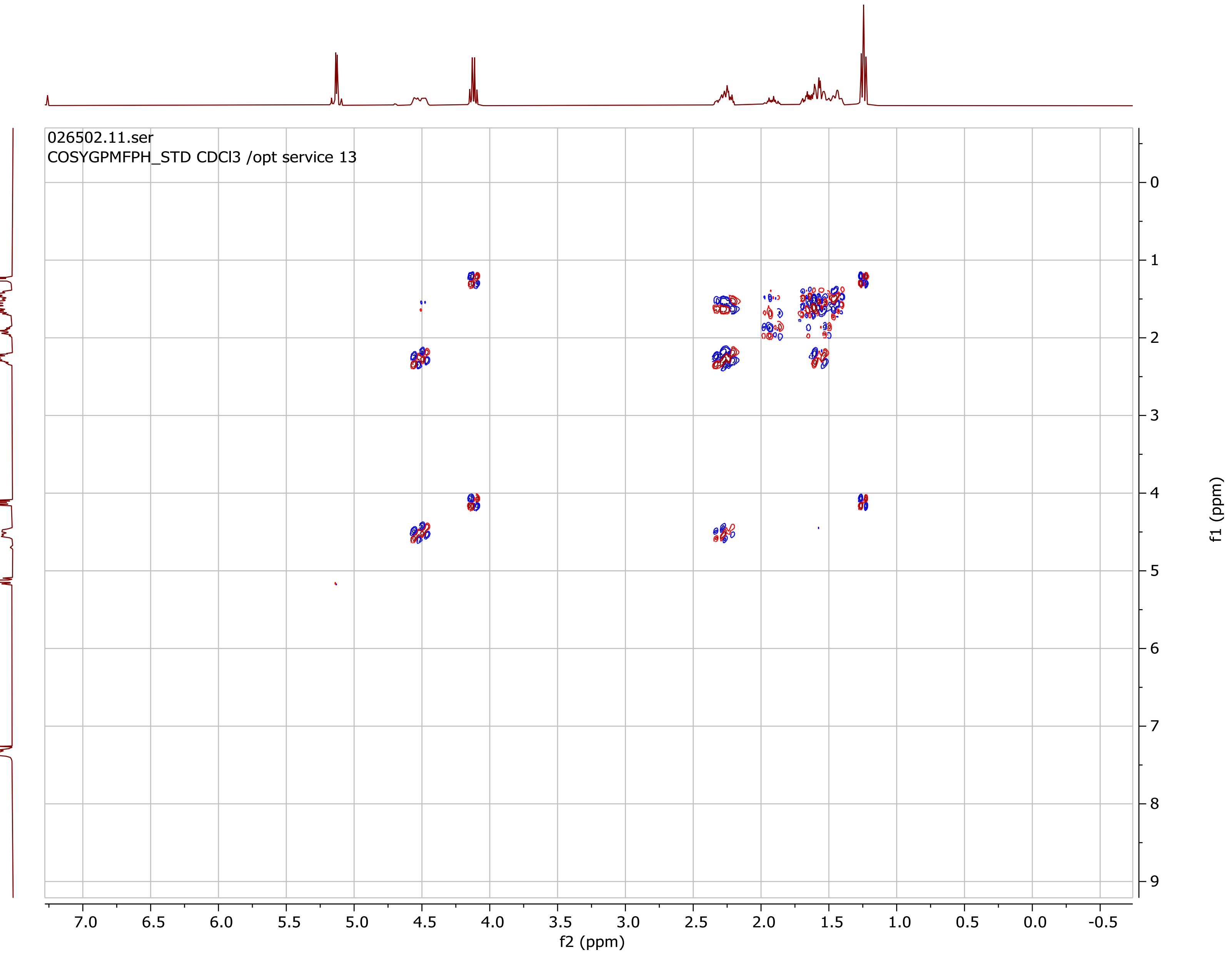

O9-benzyl O3-ethyl 9-azabicyclo[3.3.1]nonane-3,9-dicarboxylate (**13α**)

^1H, ^{13}C-HSQC NMR (300 MHz, $CDCl_3$)

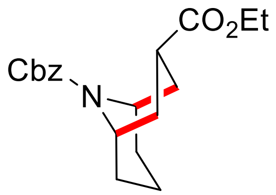

13

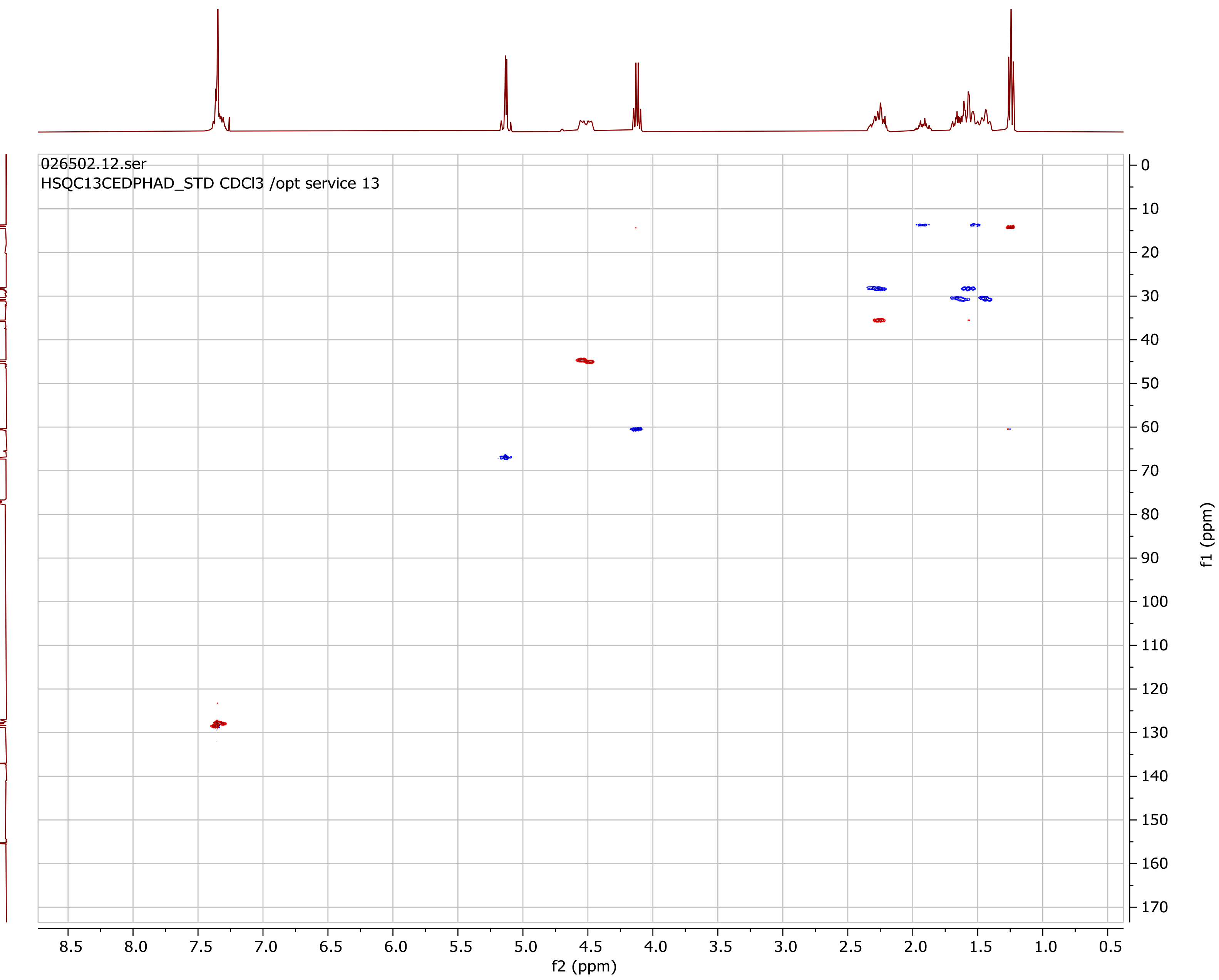

O9-benzyl O3-ethyl 9-azabicyclo[3.3.1]nonane-3,9-dicarboxylate (**13α**)

^1H, ^{13}C-HSQC NMR (300 MHz, $CDCl_3$)

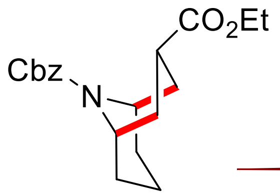

13

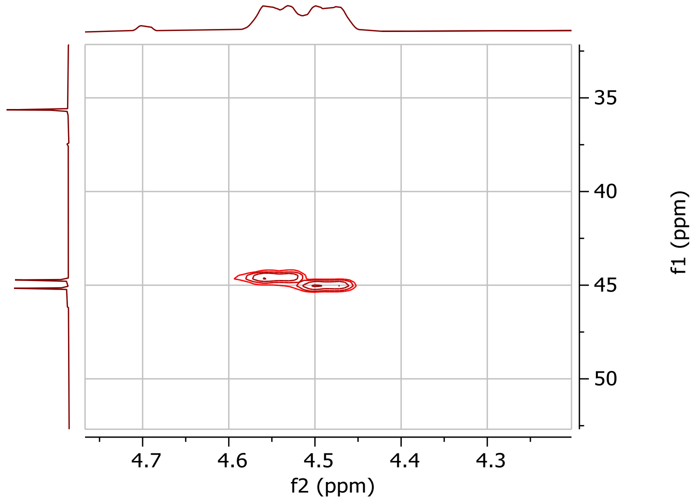

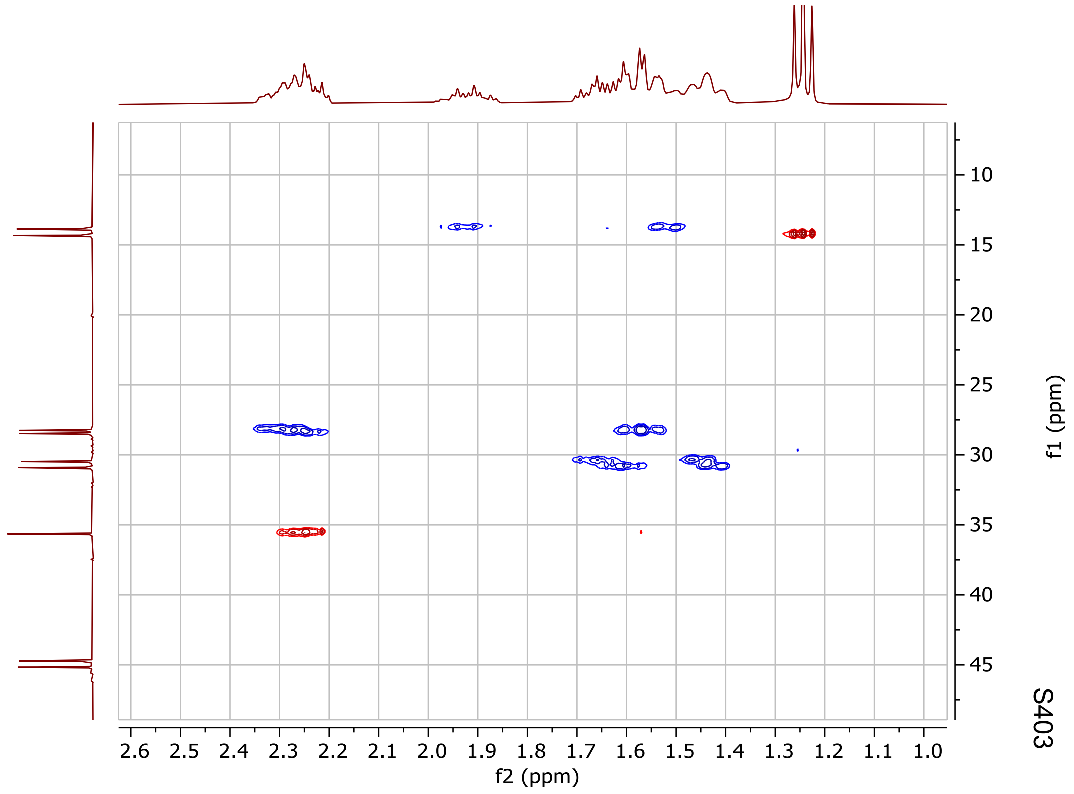

O9-benzyl O3-ethyl 9-azabicyclo[3.3.1]nonane-3,9-dicarboxylate (**13α**)

^{1}H, ^{13}C-HMBC NMR (300 MHz, $CDCl_3$)

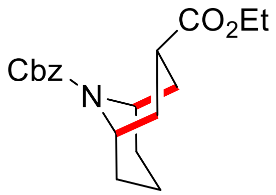

13

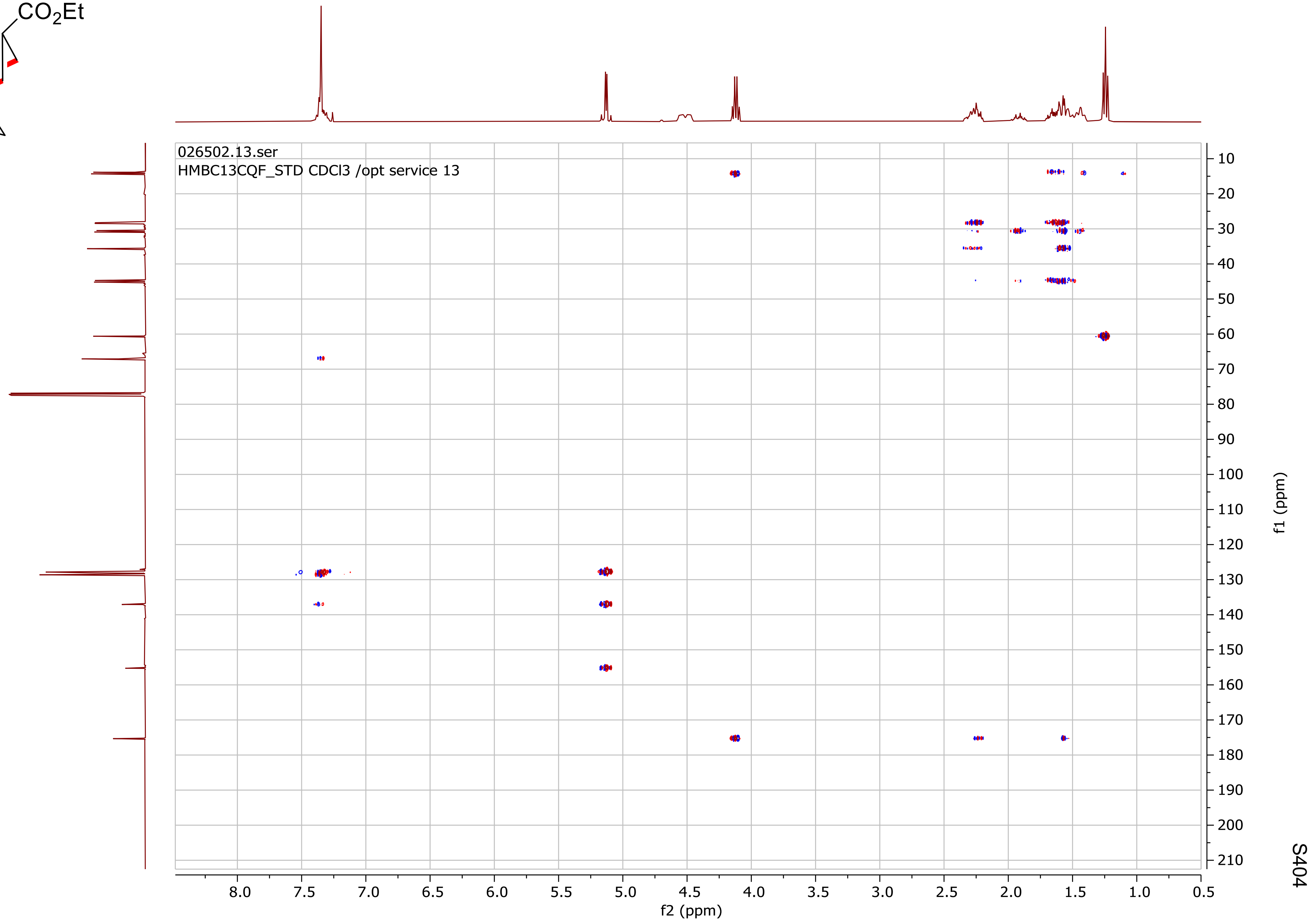

O9-benzyl O3-ethyl 9-azabicyclo[3.3.1]nonane-3,9-dicarboxylate (**13α**)

FT-IR, ATR-diamond

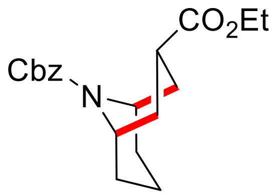

13

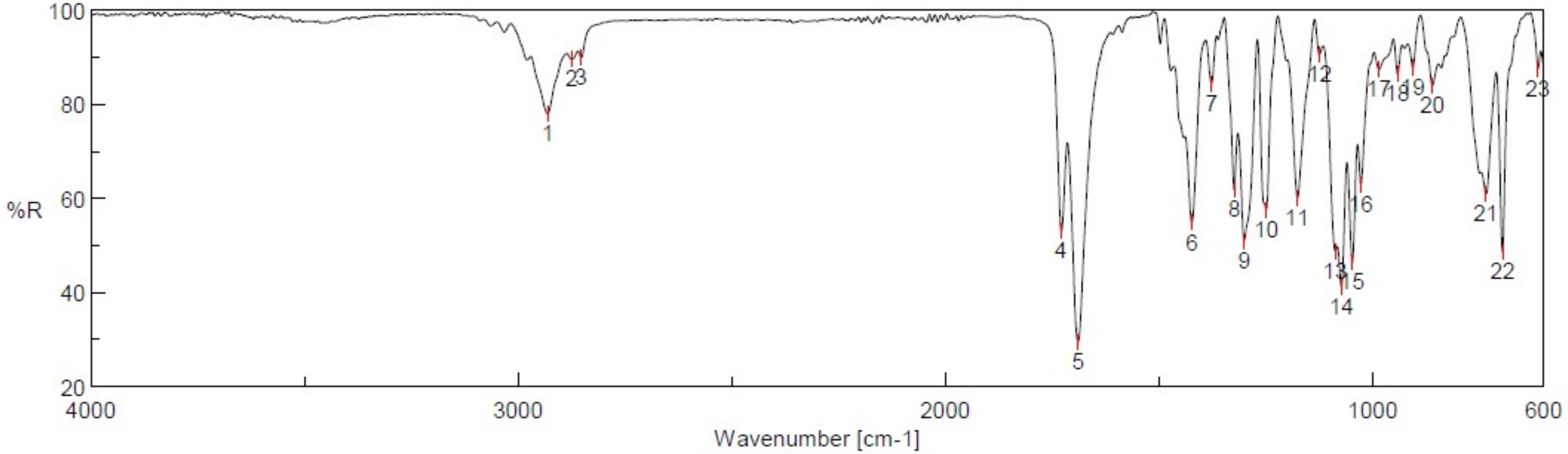

[Result of Peak Picking]

No.	Position	Intensity	No.	Position	Intensity	No.	Position	Intensity
1	2931.27	78.0341	2	2875.34	89.6515	3	2852.2	89.9511
4	1728.87	52.9883	5	1689.34	29.5505	6	1423.21	54.8637
7	1376.93	84.4241	8	1323.89	61.85	9	1300.75	50.9667
10	1248.68	57.5809	11	1176.36	60.081	12	1124.3	90.5408
13	1087.66	48.68	14	1073.19	41.1528	15	1048.12	46.3642
16	1027.87	62.9065	17	985.447	87.4459	18	942.056	86.5098
19	906.379	87.825	20	860.096	84.0056	21	734.746	60.9369
22	696.177	48.5987	23	613.252	87.6538			

Diethyl 9-phenyl-9-azabicyclo[3.3.1]nonane-3,7-dicarboxylate (**14**) **β,α/β,β 20:1**

1H-NMR (400 MHz, CDCl3)

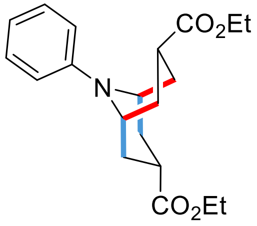

14
ß,a/ß,ß 20:1

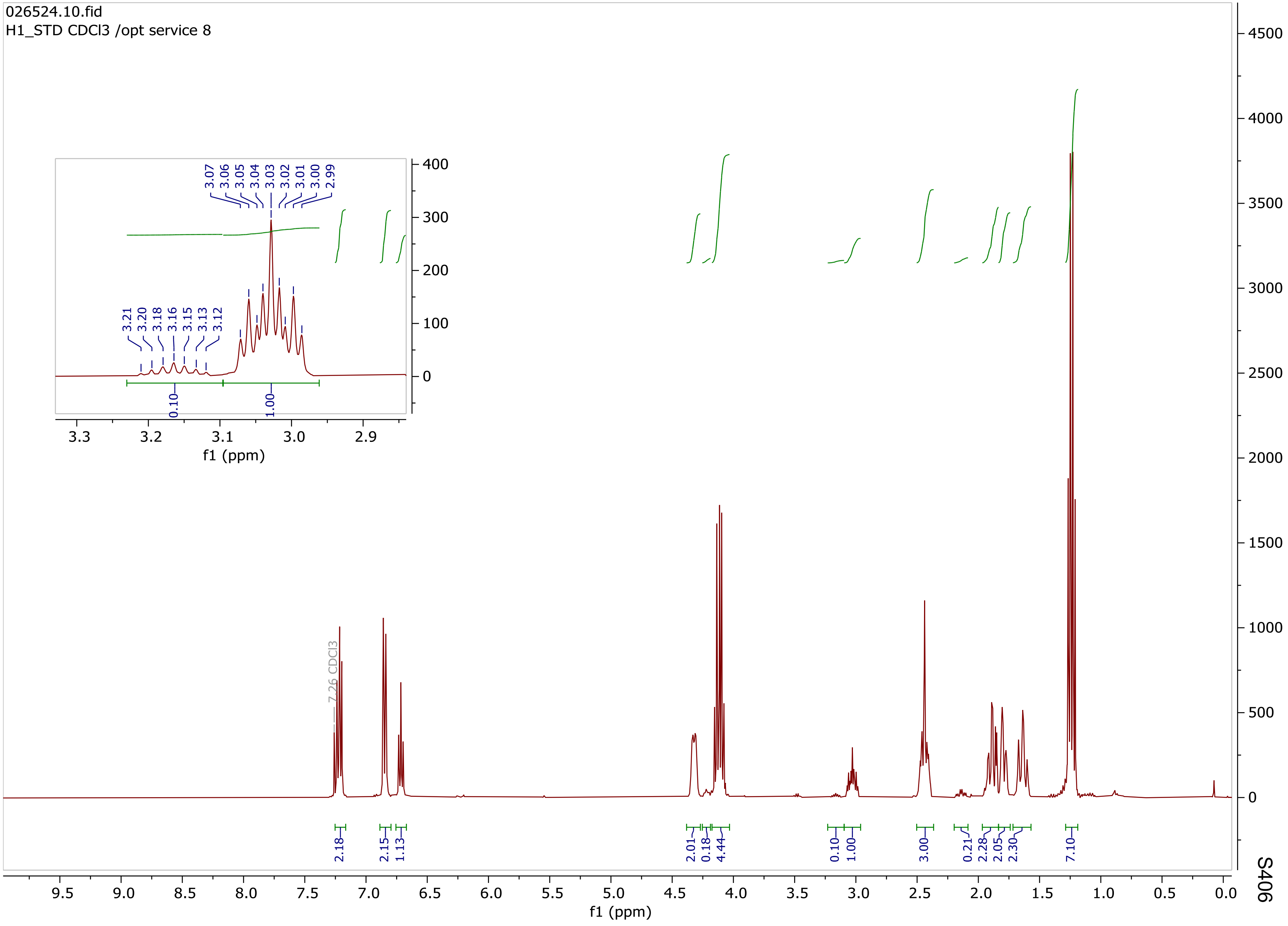

Diethyl 9-phenyl-9-azabicyclo[3.3.1]nonane-3,7-dicarboxylate (**14**) **β,α/β,β 20:1**

^{13}C-NMR (101 MHz, $CDCl_3$)

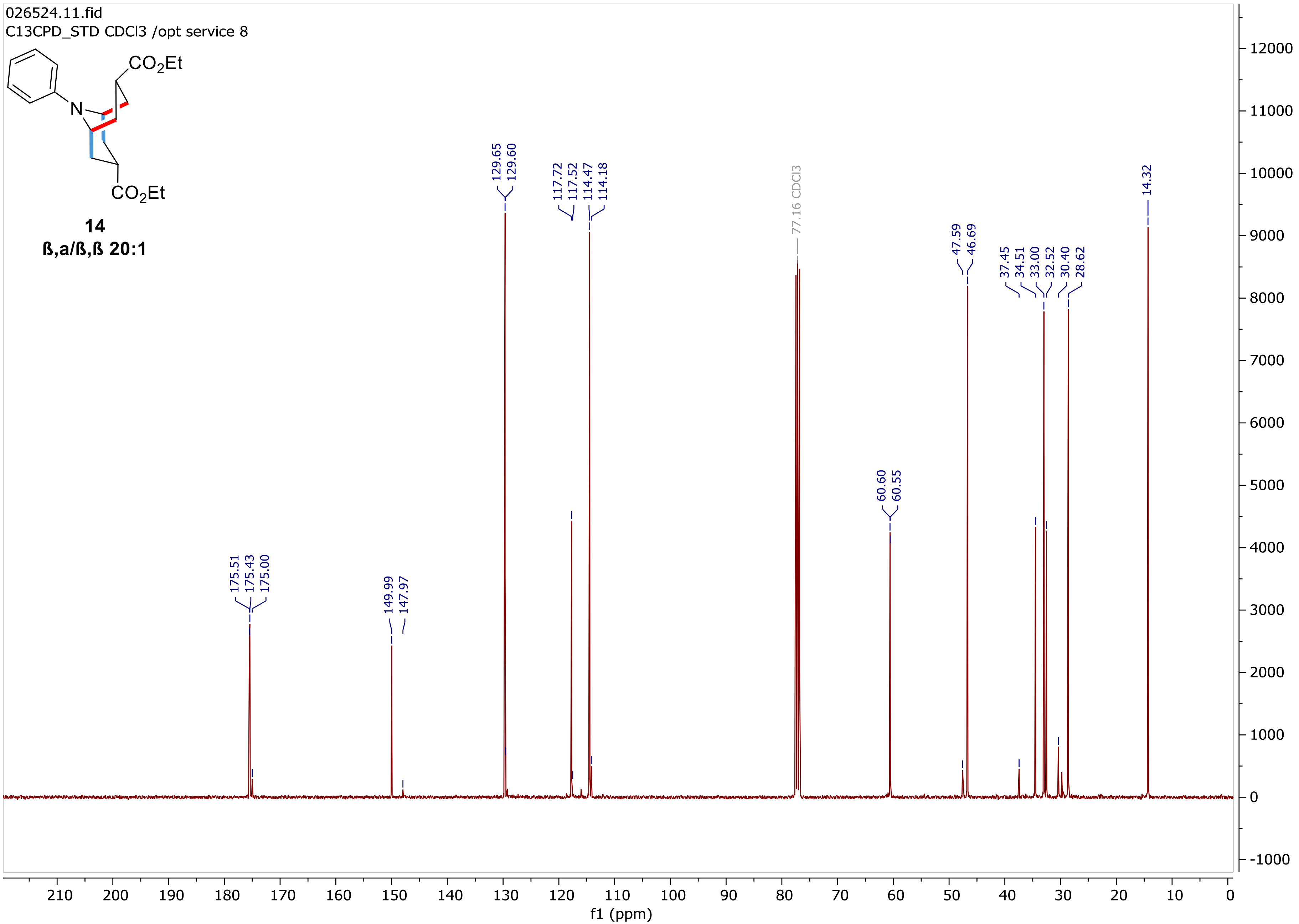

Diethyl 9-phenyl-9-azabicyclo[3.3.1]nonane-3,7-dicarboxylate (**14**) **β,α/β,β 20:1**

^{13}C-NMR (101 MHz, $CDCl_3$)

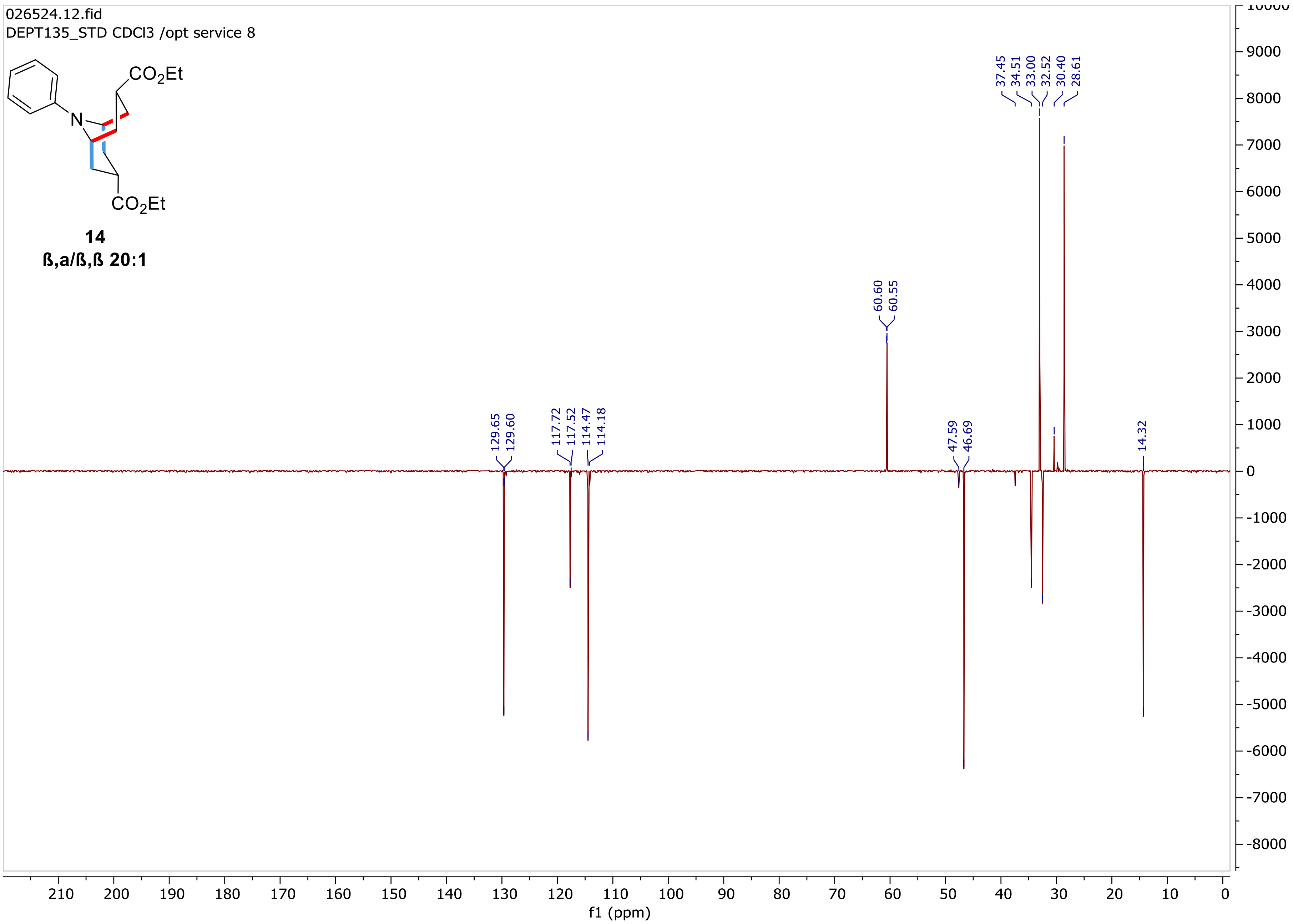

Diethyl 9-phenyl-9-azabicyclo[3.3.1]nonane-3,7-dicarboxylate (**14 β,α**)

1H-NMR (400 MHz, CDCl3)

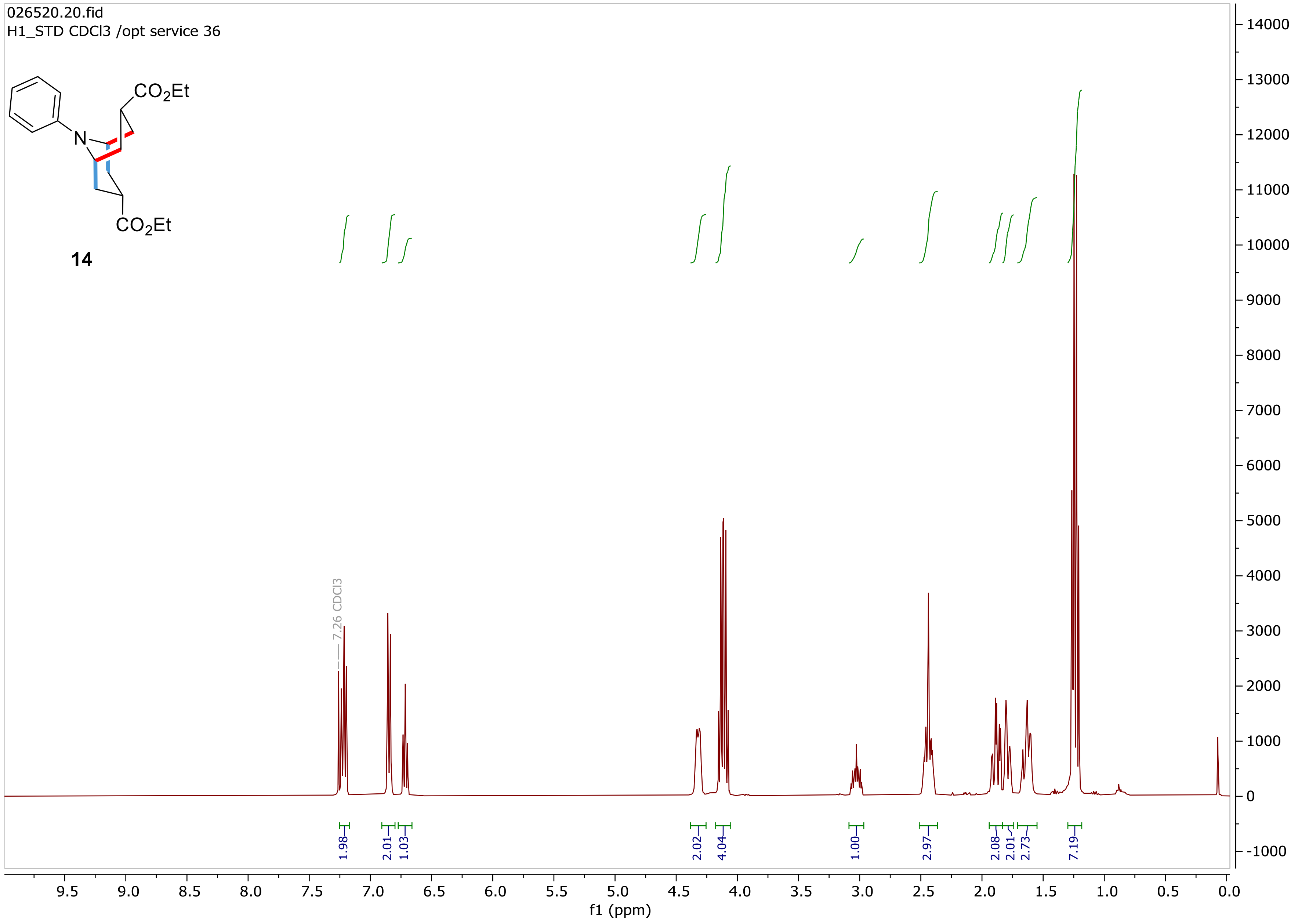

Diethyl 9-phenyl-9-azabicyclo[3.3.1]nonane-3,7-dicarboxylate (**14 β,α**)

^{1}H-NMR (400 MHz, $CDCl_3$)

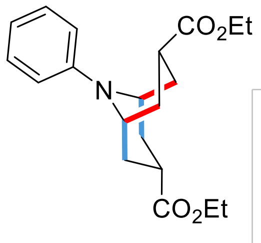

14

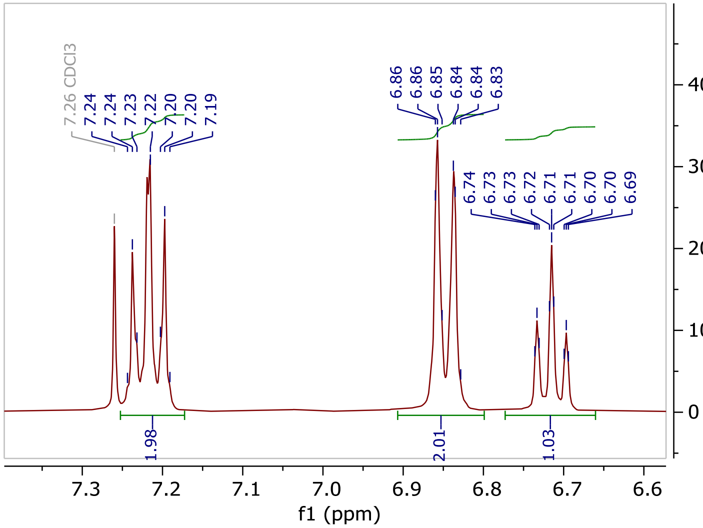

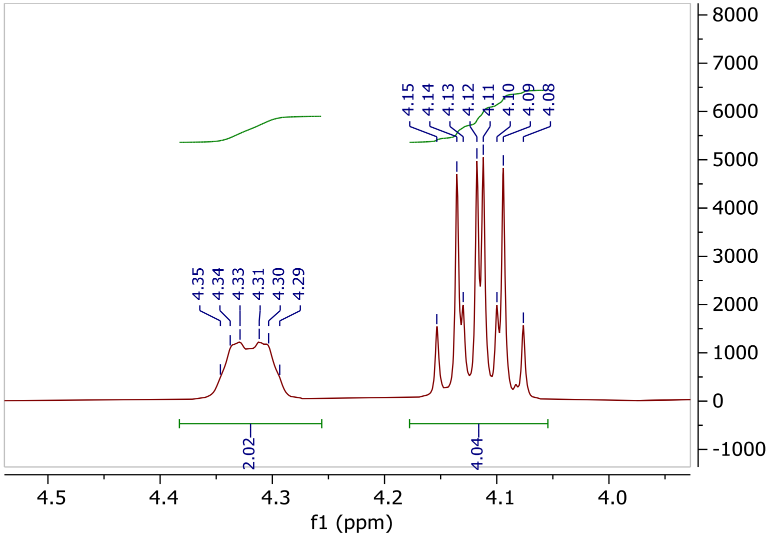

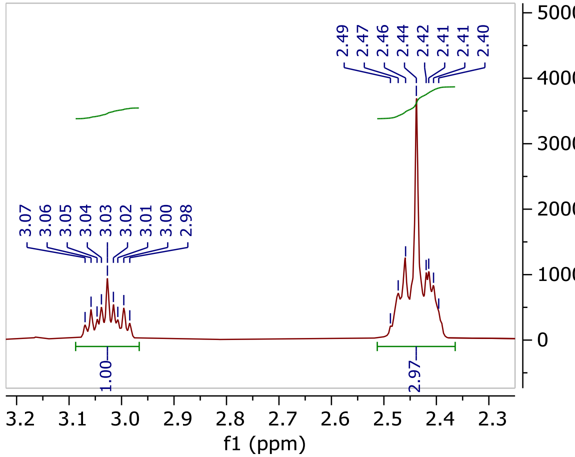

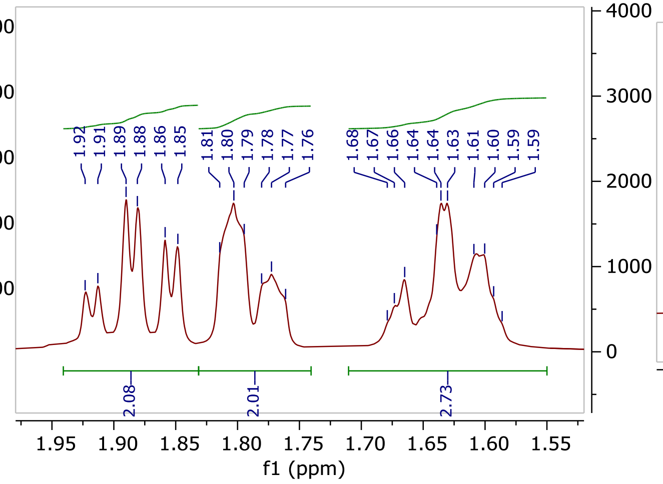

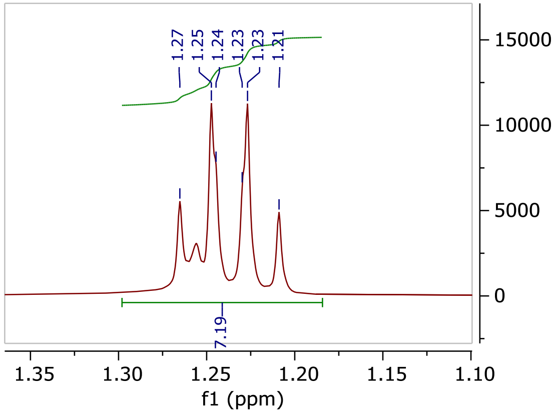

Diethyl 9-phenyl-9-azabicyclo[3.3.1]nonane-3,7-dicarboxylate (**14 β,α**)

^{13}C-NMR (101 MHz, $CDCl_3$)

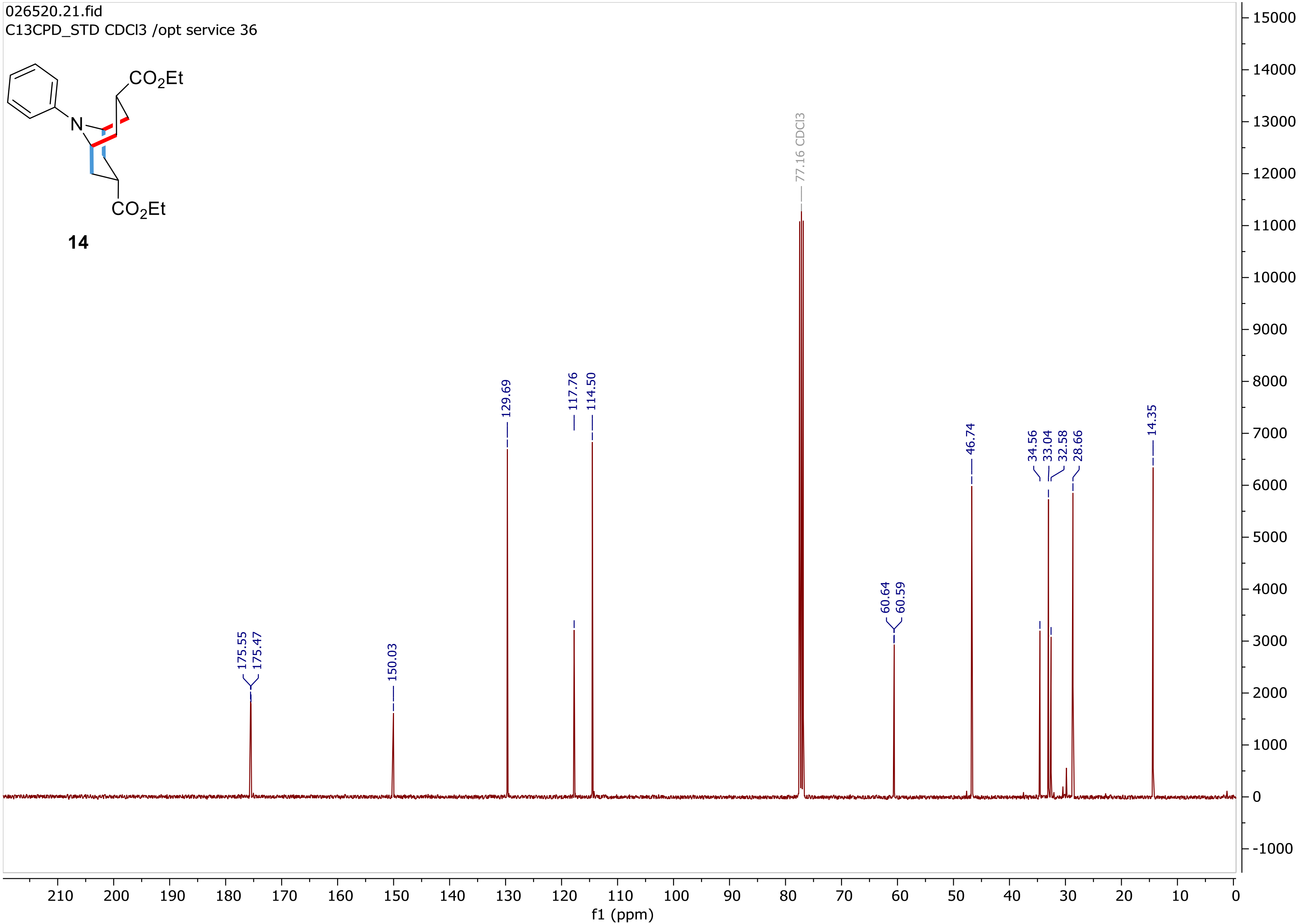

14

Diethyl 9-phenyl-9-azabicyclo[3.3.1]nonane-3,7-dicarboxylate (**14 β,α**)

^{13}C-NMR (101 MHz, $CDCl_3$)

026520.22.fid
DEPT135_STD CDCl3 /opt service 36

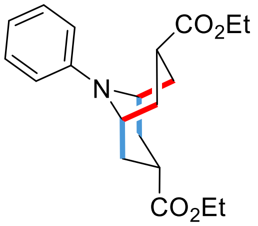

14

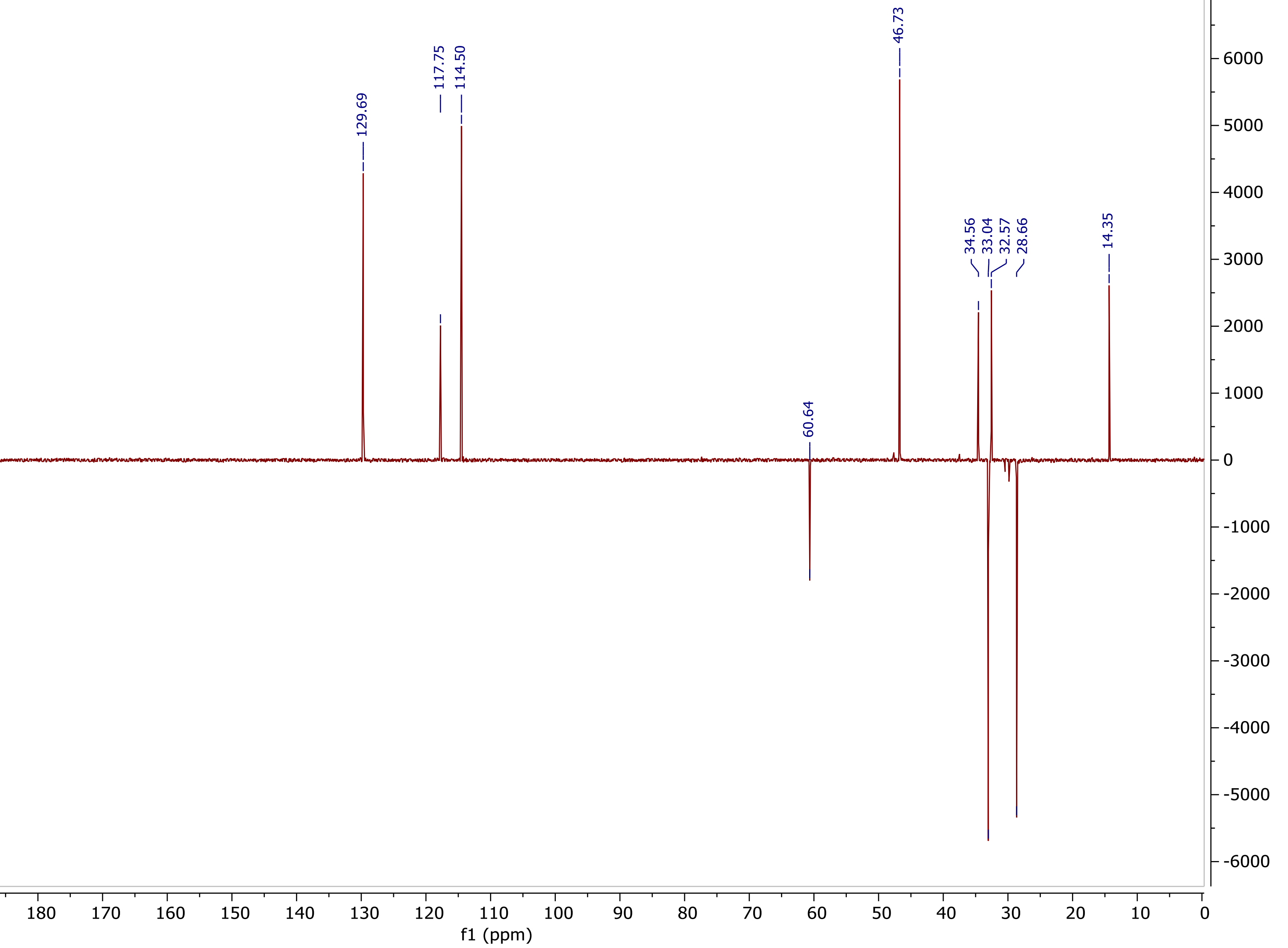

Diethyl 9-phenyl-9-azabicyclo[3.3.1]nonane-3,7-dicarboxylate (**14 β,α**)

^{1}H, ^{1}H-COSY NMR (400 MHz, $CDCl_3$)

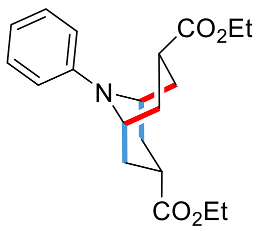

14

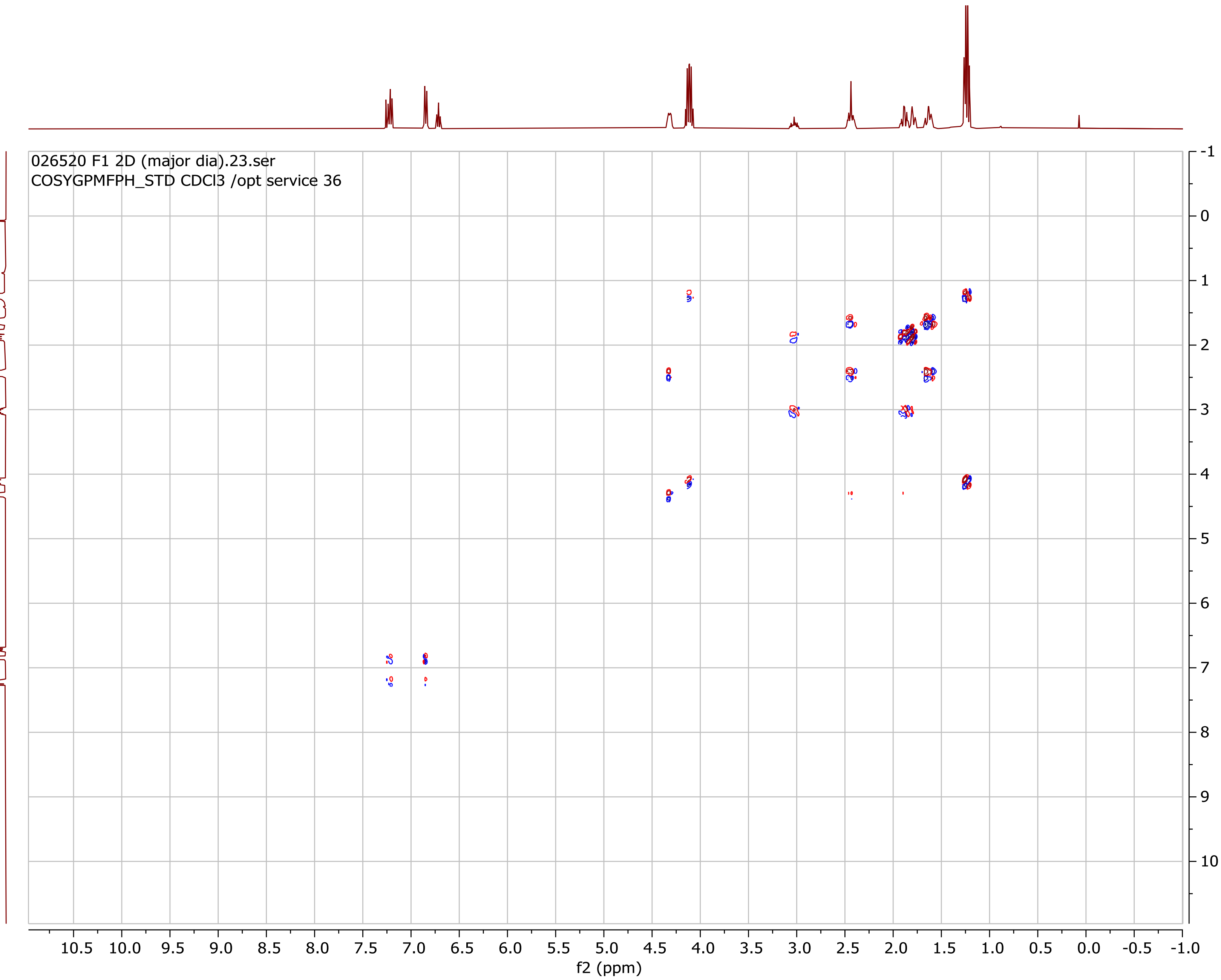

Diethyl 9-phenyl-9-azabicyclo[3.3.1]nonane-3,7-dicarboxylate (**14 β,α**)

^{1}H, ^{13}C-HSQC NMR (400 MHz, $CDCl_3$)

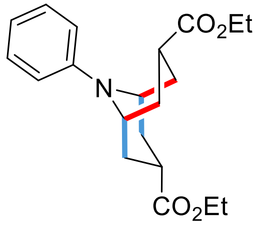

14

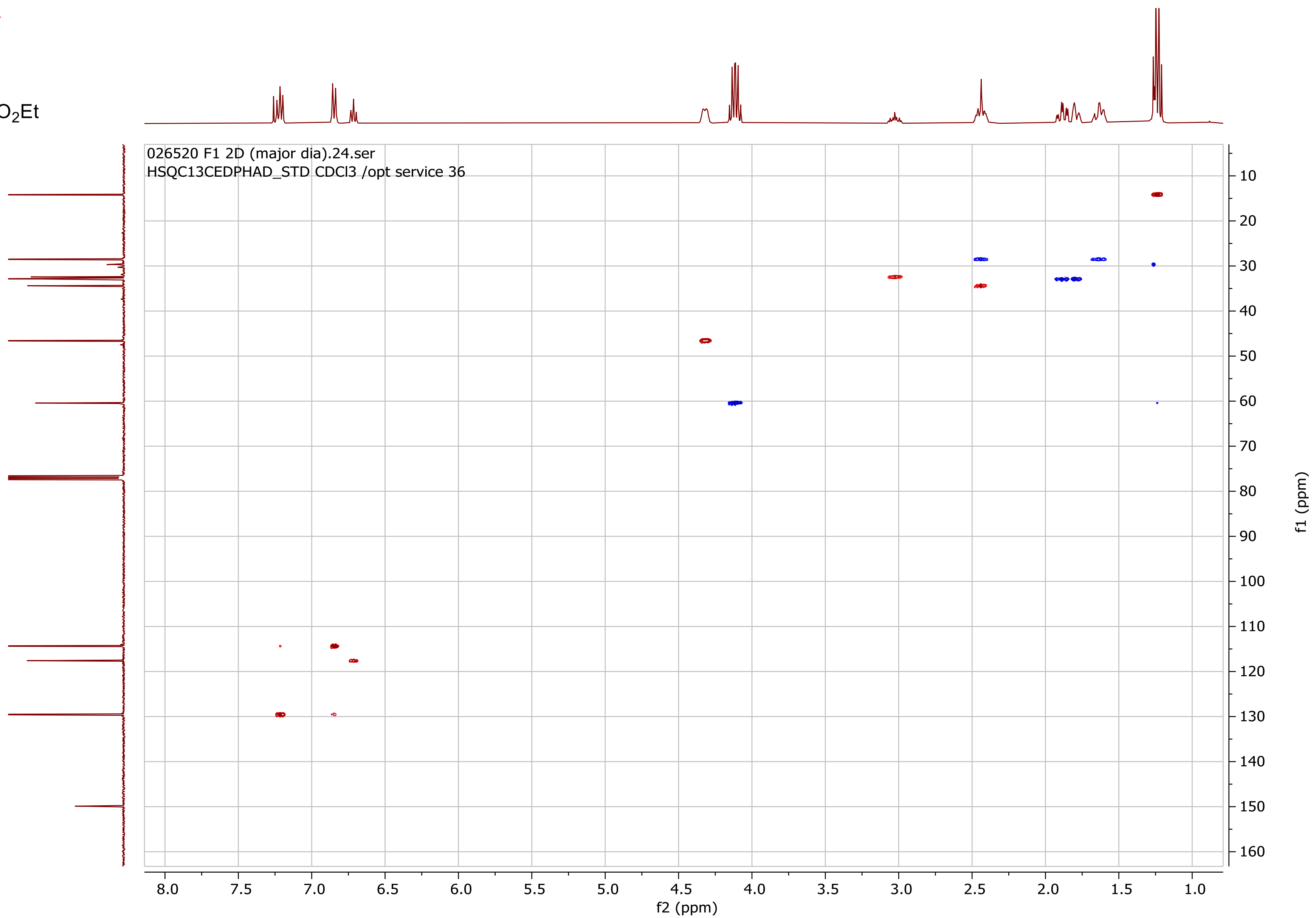

Diethyl 9-phenyl-9-azabicyclo[3.3.1]nonane-3,7-dicarboxylate (**14 β,α**)

^{1}H, ^{13}C-HMBC NMR (400 MHz, $CDCl_3$)

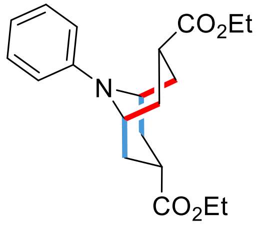

14

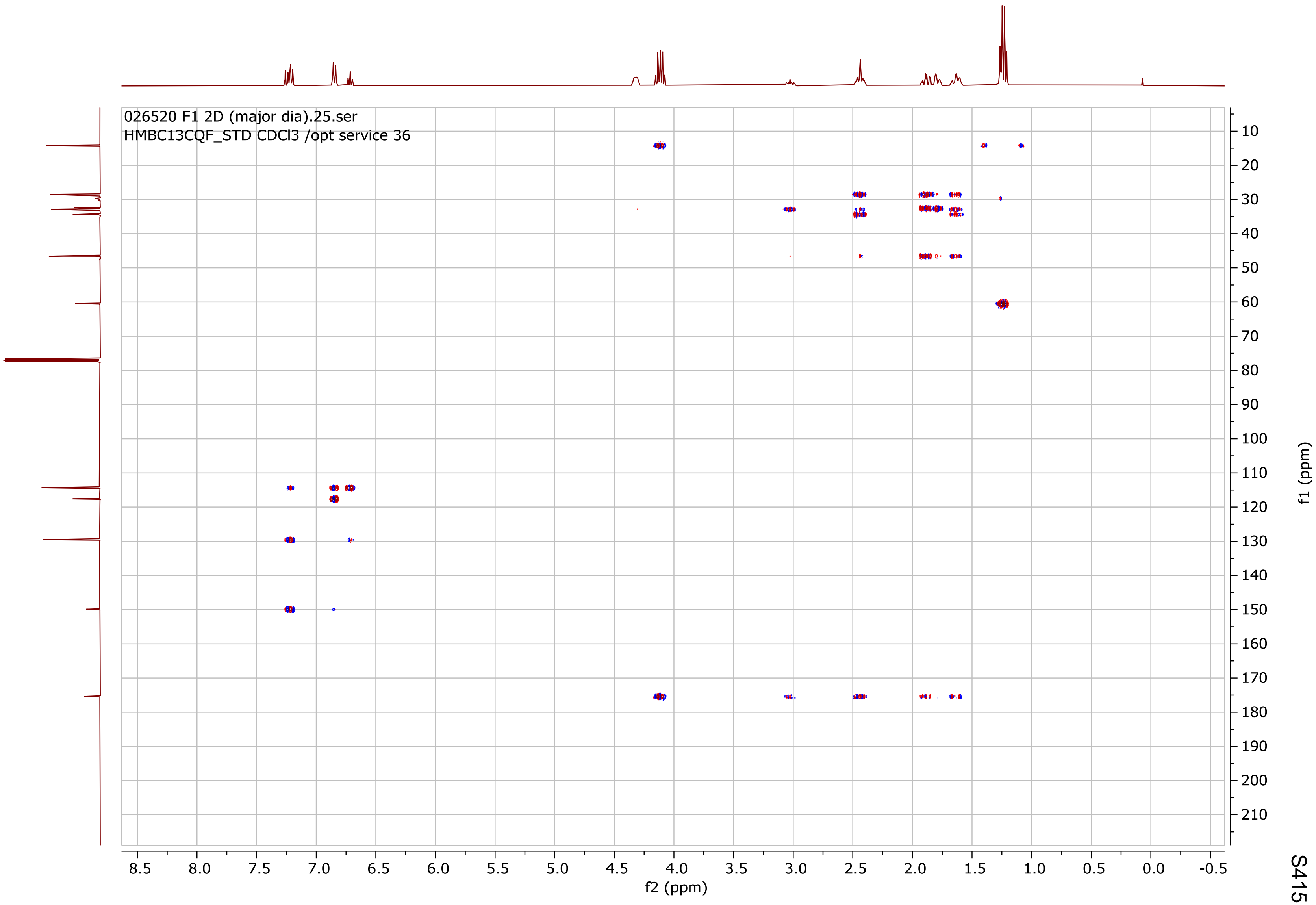

Diethyl 9-phenyl-9-azabicyclo[3.3.1]nonane-3,7-dicarboxylate (**14 β,α**)

FT-IR, ATR-diamond

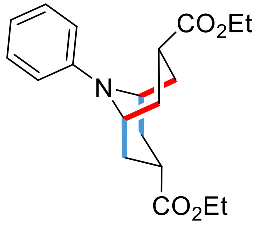

14

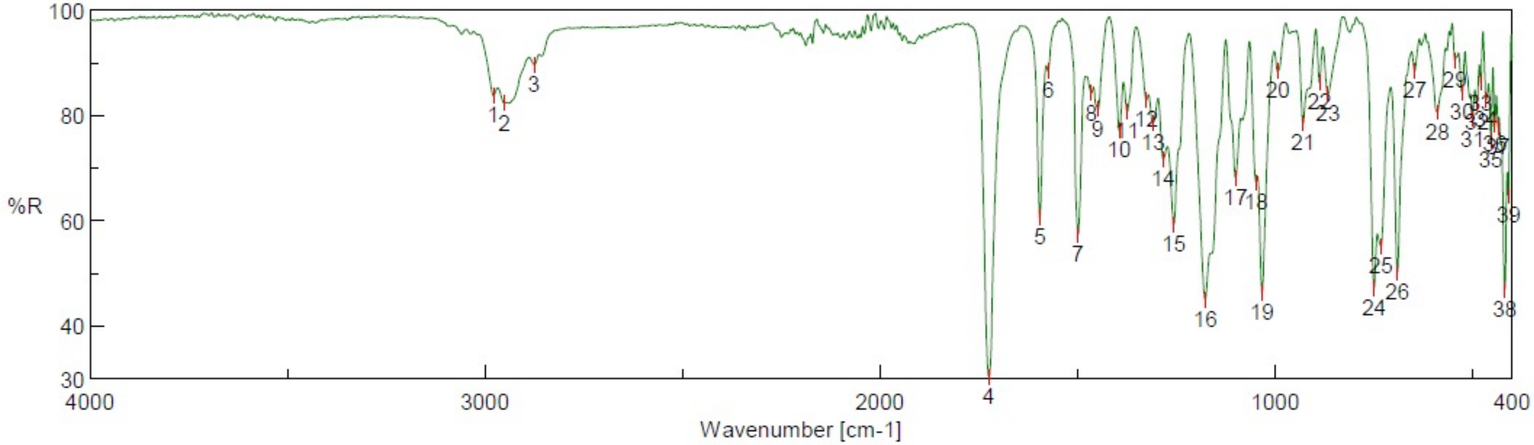

[Result of Peak Picking]

No.	Position	Intensity	No.	Position	Intensity	No.	Position	Intensity
1	2978.52	83.7555	2	2950.55	82.3253	3	2875.34	89.6703
4	1724.05	30.2681	5	1594.84	60.544	6	1574.59	88.4485
7	1498.42	57.3409	8	1464.67	84.2531	9	1448.28	81.166
10	1394.28	77.0638	11	1374.03	80.7228	12	1325.82	82.8983
13	1309.43	78.4807	14	1282.43	71.6367	15	1256.4	59.218
16	1176.36	45.0081	17	1100.19	68.1814	18	1048.12	67.3456
19	1032.69	46.233	20	993.16	88.4305	21	928.557	78.4818
22	887.095	86.247	23	865.882	84.097	24	749.209	47.1382
25	731.853	55.2	26	690.391	50.1878	27	646.036	88.4795
28	588.182	80.6133	29	544.792	90.4719	30	526.471	84.4312
31	499.473	79.2368	32	488.866	82.4712	33	479.224	86.1656
34	465.725	82.9286	35	453.19	75.2429	36	443.547	78.3539
37	434.869	77.8074	38	418.477	46.9417	39	408.835	64.8305

Caracterization of by-products

Ethyl 2-[[5-(2-ethoxycarbonylallyl)-1-phenyl-pyrrolidin-2-yl]methyl]prop-2-enoate **dr 1:4**

^{1}H-NMR (300 MHz, $CDCl_3$)

GA_206948.12.fid
ECO-2-129 F1
Proton_ns16 CDCl3 /opt service 26

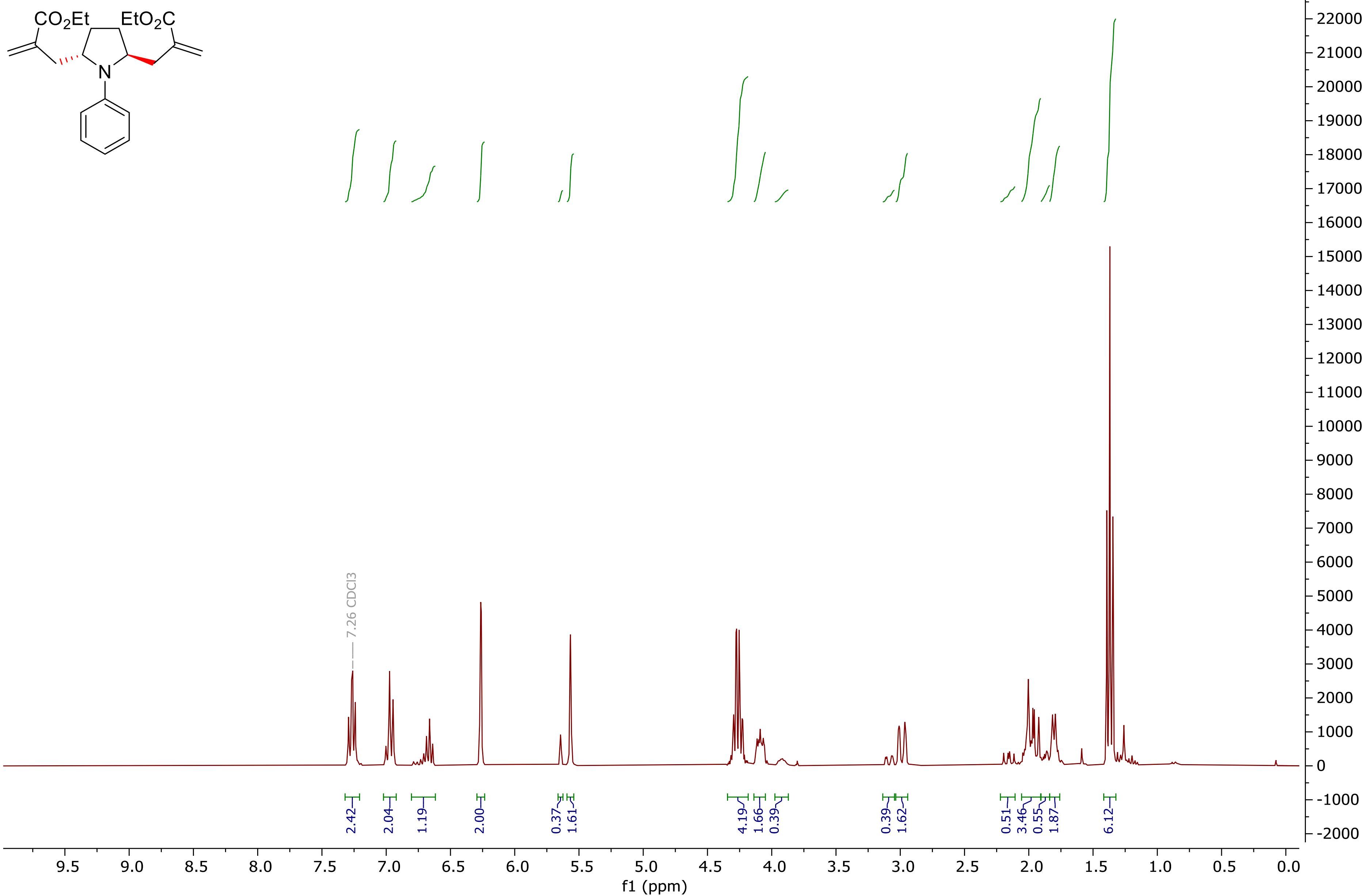

Ethyl 2-[[5-(2-ethoxycarbonylallyl)-1-phenyl-pyrrolidin-2-yl]methyl]prop-2-enoate

^{1}H-NMR (300 MHz, $CDCl_3$)

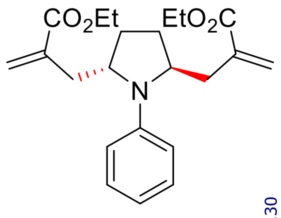

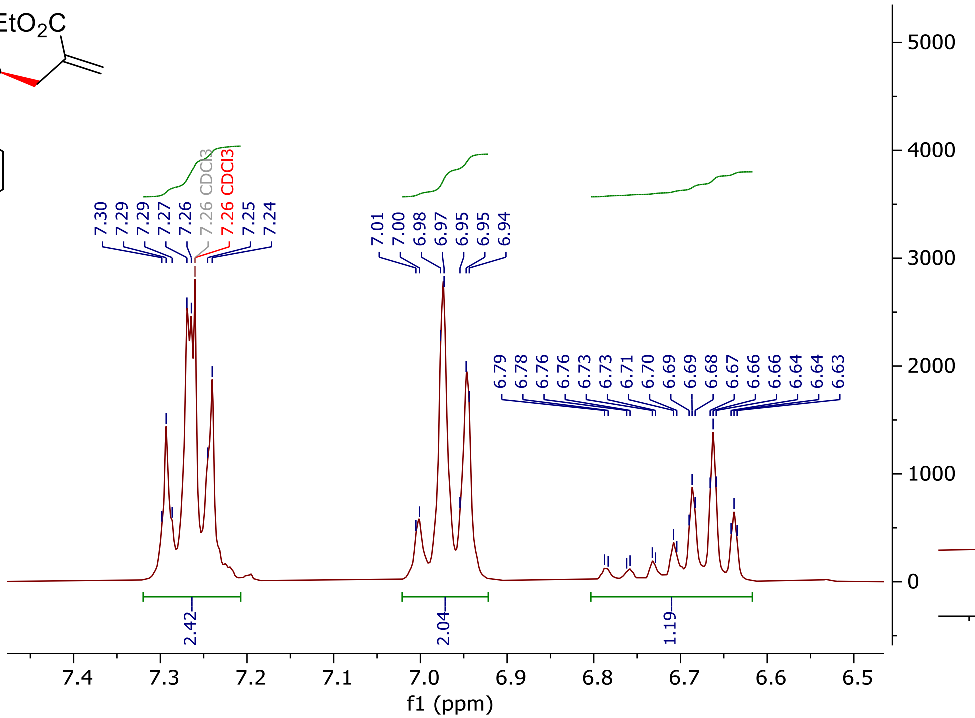

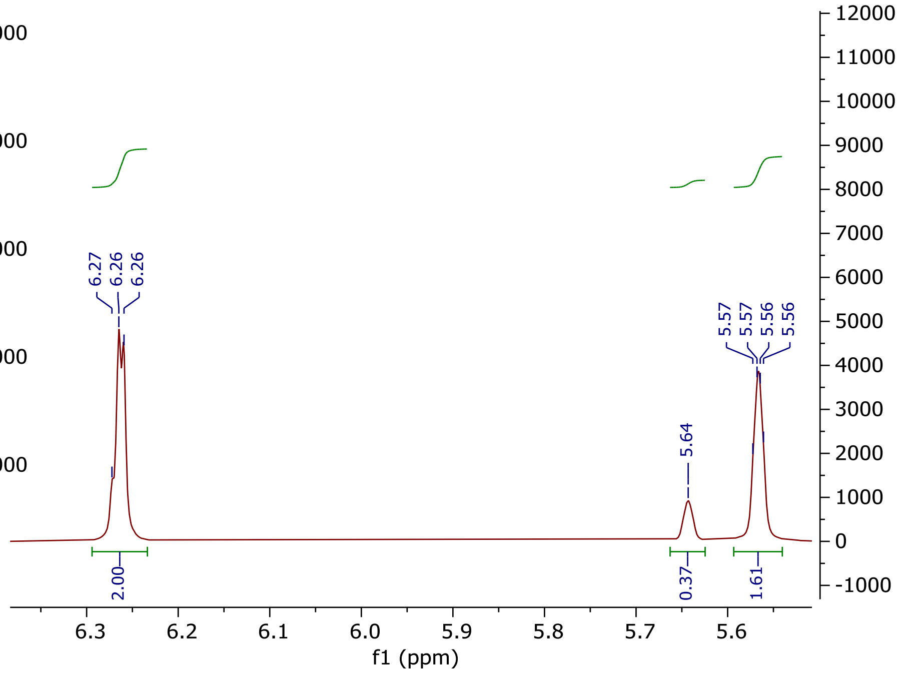

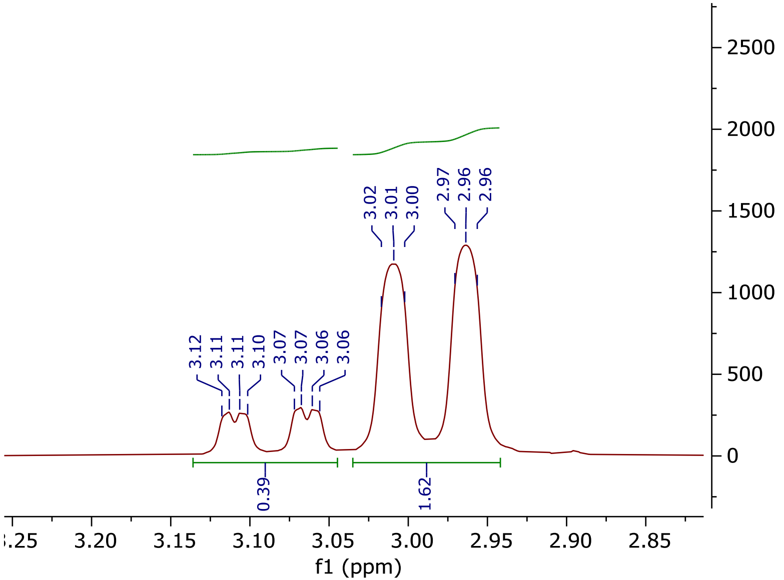

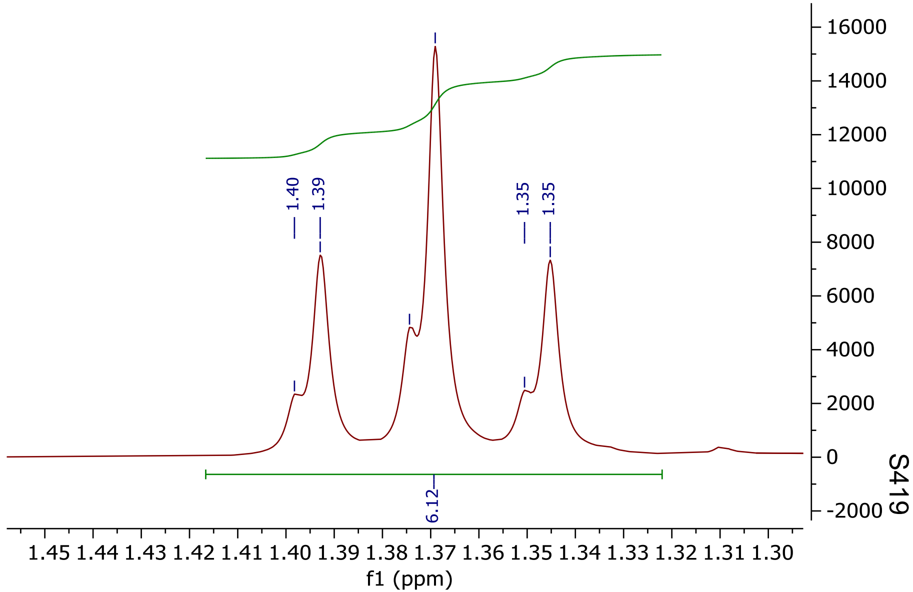

Ethyl 2-[[5-(2-ethoxycarbonylallyl)-1-phenyl-pyrrolidin-2-yl]methyl]prop-2-enoate

^{1}H-NMR (300 MHz, $CDCl_3$)

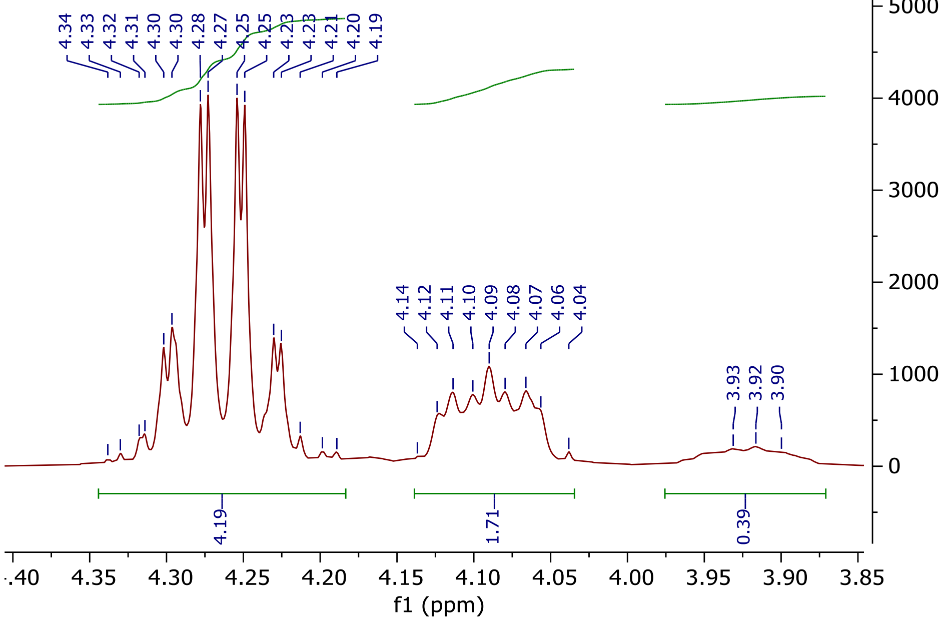

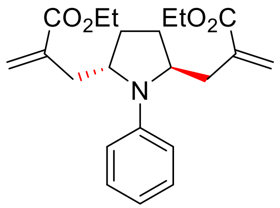

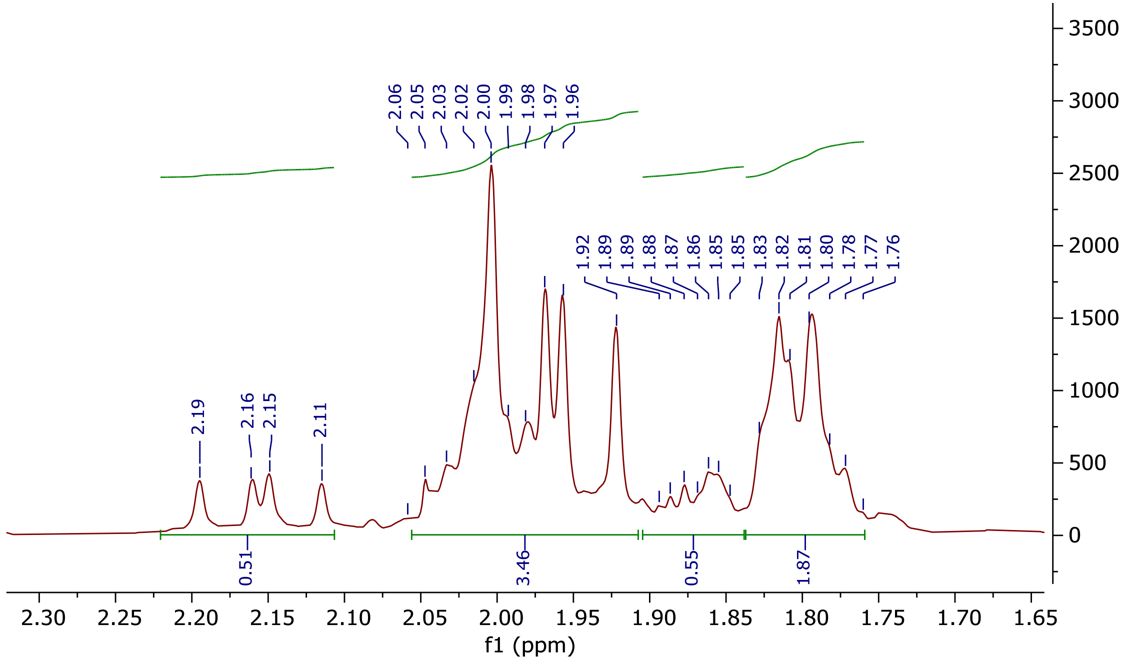

Ethyl 2-[[5-(2-ethoxycarbonylallyl)-1-phenyl-pyrrolidin-2-yl]methyl]prop-2-enoate

^{13}C-NMR (75 MHz, $CDCl_3$)

GA_206948.10.fid
ECO-2-129 F1
Carbon_ns512 CDCl3 /opt service 26

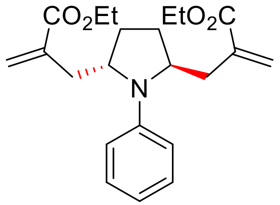

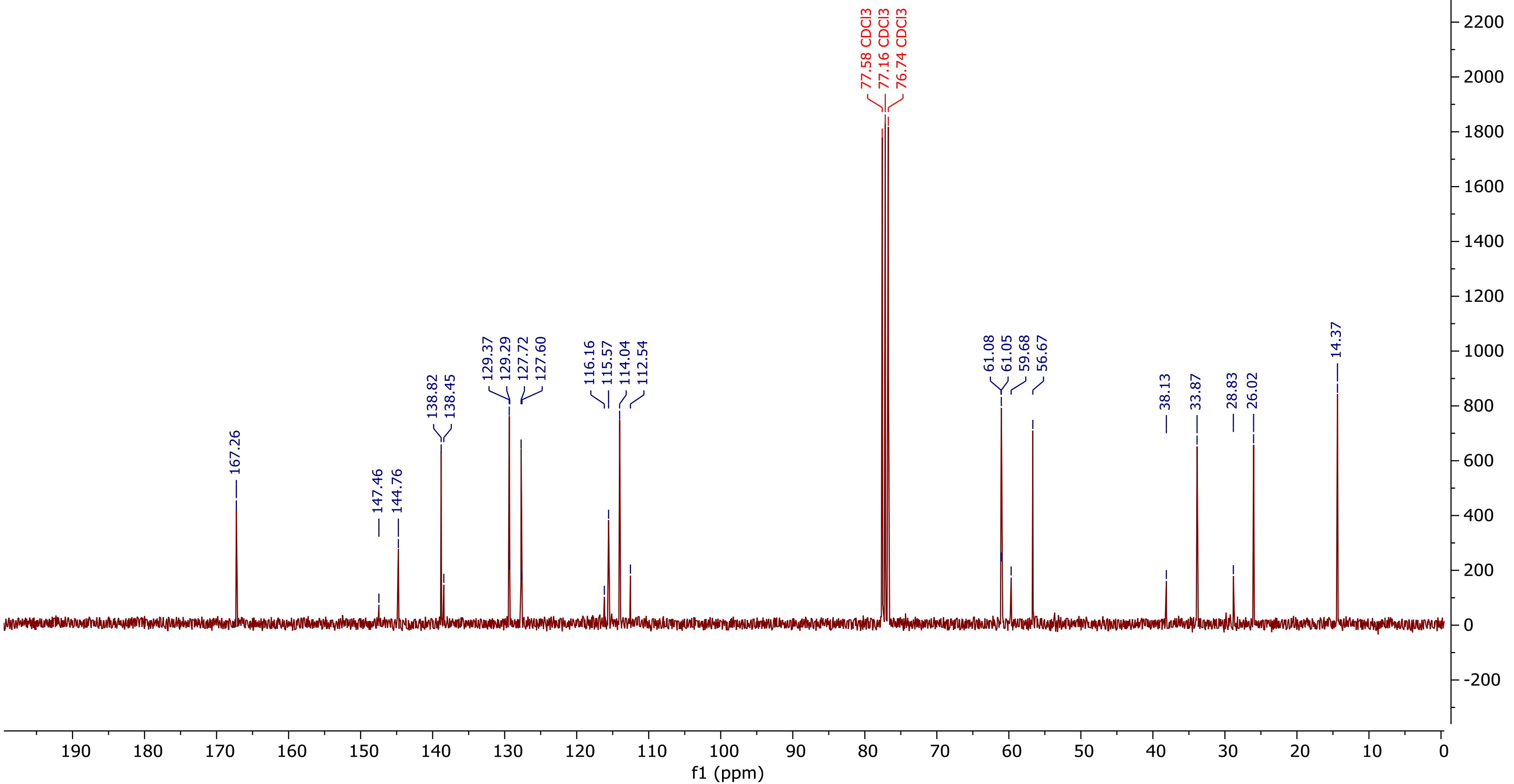

Ethyl 2-[[5-(2-ethoxycarbonylallyl)-1-phenyl-pyrrolidin-2-yl]methyl]prop-2-enoate

^{13}C-NMR (75 MHz, $CDCl_3$)

GA_206948.11.fid
ECO-2-129 F1
Dept135_ns512 CDCl3 /opt service 26

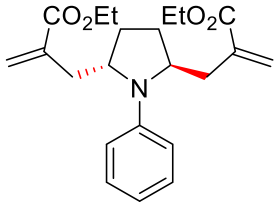

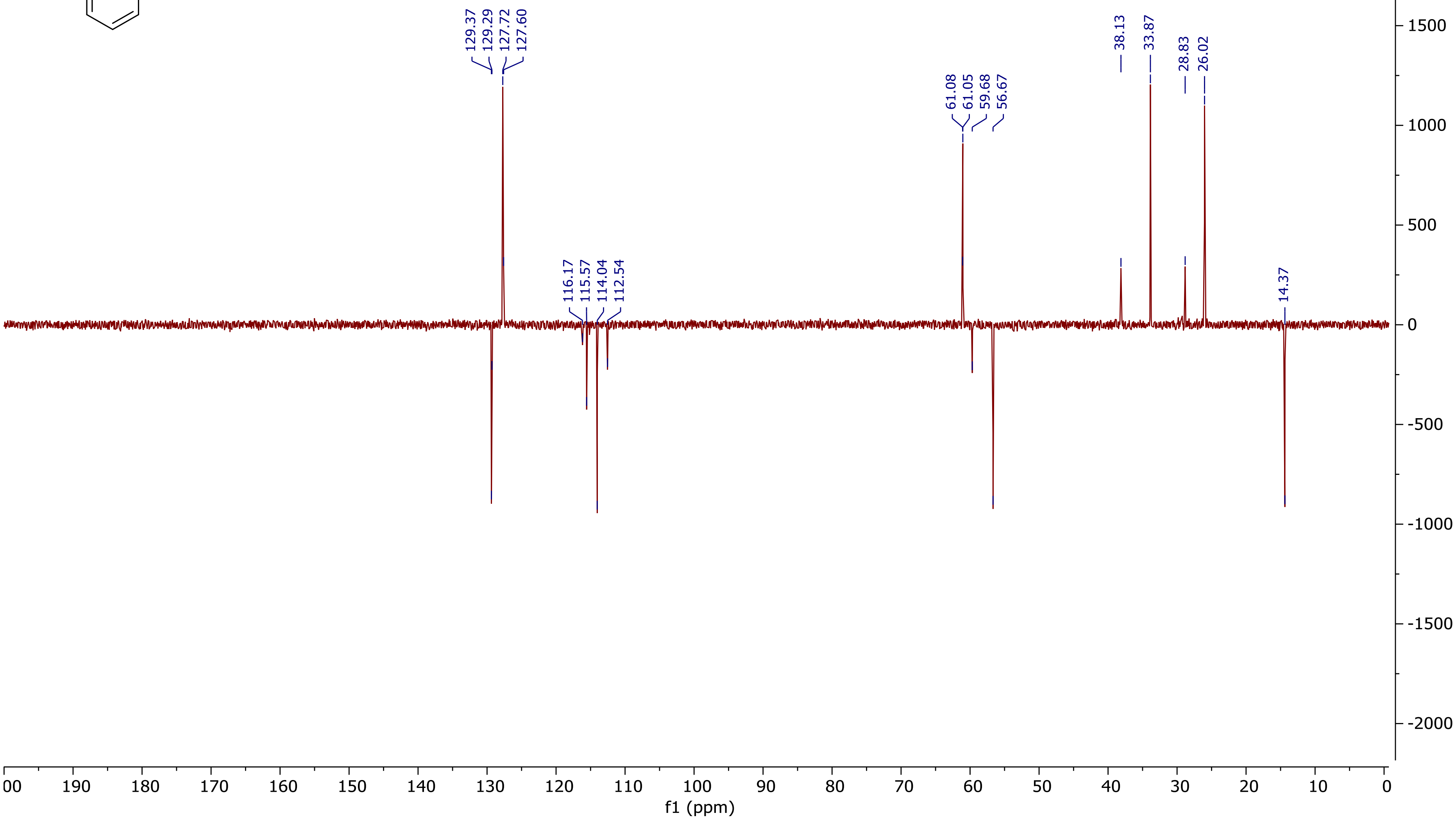

Ethyl 2-[[5-(2-ethoxycarbonylallyl)-1-phenyl-pyrrolidin-2-yl]methyl]prop-2-enoate

FT-IR, ATR-diamond

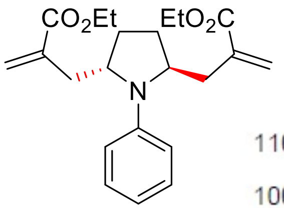

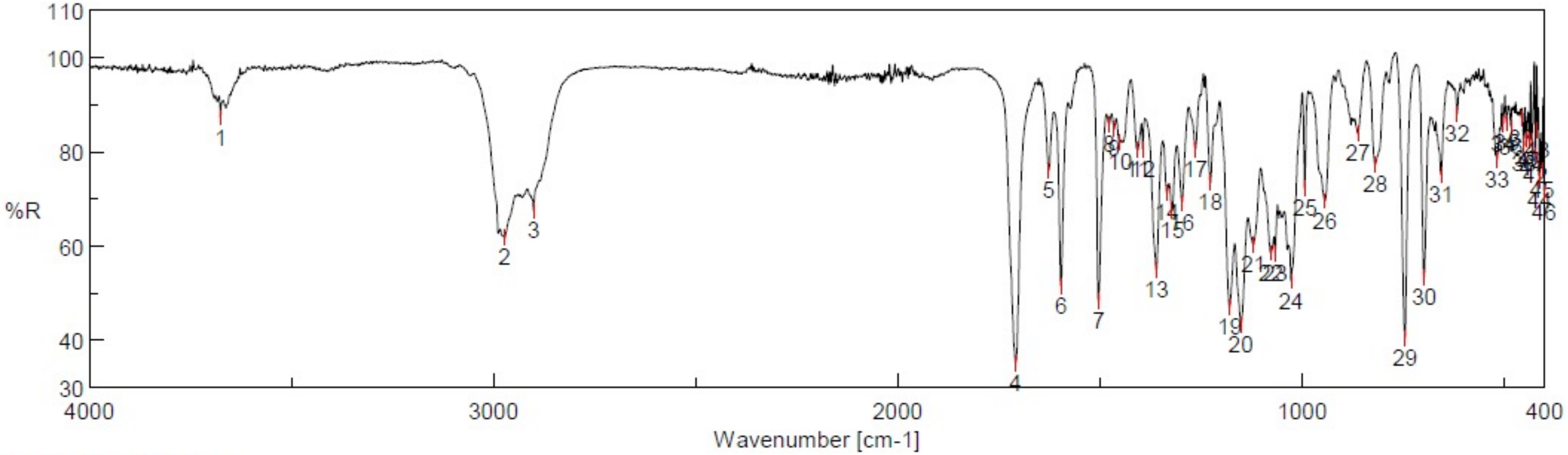

[Result of Peak Picking]

No.	Position	Intensity	No.	Position	Intensity	No.	Position	Intensity
1	3675.18	87.2342	2	2972.25	61.6931	3	2900.9	67.4783
4	1708.62	35.1464	5	1626.66	76.028	6	1596.29	51.523
7	1503.24	48.1832	8	1477.21	85.8452	9	1464.67	84.8021
10	1450.21	81.9103	11	1406.82	80.3598	12	1393.8	80.4589
13	1360.53	54.8847	14	1334.02	71.1752	15	1321	67.4202
16	1296.89	69.2167	17	1263.63	80.5624	18	1226.99	73.3917
19	1179.26	46.9501	20	1151.29	43.2276	21	1120.44	60.2365
22	1077.53	58.5475	23	1066.44	58.4538	24	1026.91	52.5566
25	993.16	72.182	26	943.502	69.5513	27	861.543	83.8624
28	819.116	77.3285	29	745.352	40.4883	30	698.105	53.4035
31	656.161	75.104	32	616.145	88.1016	33	517.311	78.3149
34	502.848	85.9074	35	493.688	86.1178	36	483.563	85.3153
37	457.047	87.0851	38	451.743	81.6932	39	444.994	82.6484
40	437.28	82.3264	41	431.977	80.9498	42	422.334	79.3215
43	417.513	84.6934	44	412.21	73.8676	45	406.424	76.1784
46	400.639	71.4294						

Ethyl 1-(2-ethoxycarbonylallyl)-8-phenyl-8-azabicyclo[3.2.1]octane-3-carboxylate_major dia

^{1}H-NMR (300 MHz, $CDCl_3$)

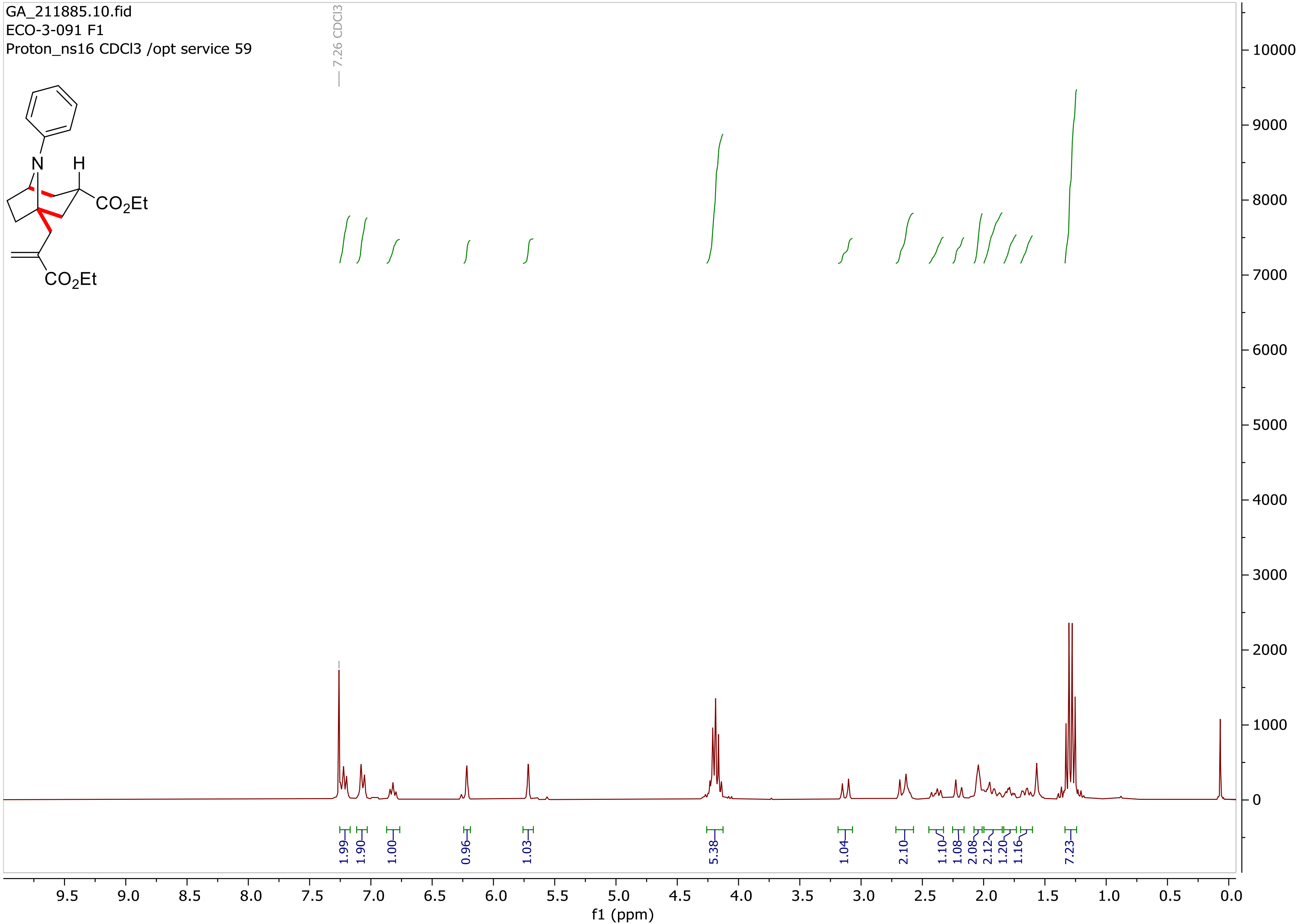

Ethyl 1-(2-ethoxycarbonylallyl)-8-phenyl-8-azabicyclo[3.2.1]octane-3-carboxylate_major dia

^{1}H-NMR (300 MHz, $CDCl_3$)

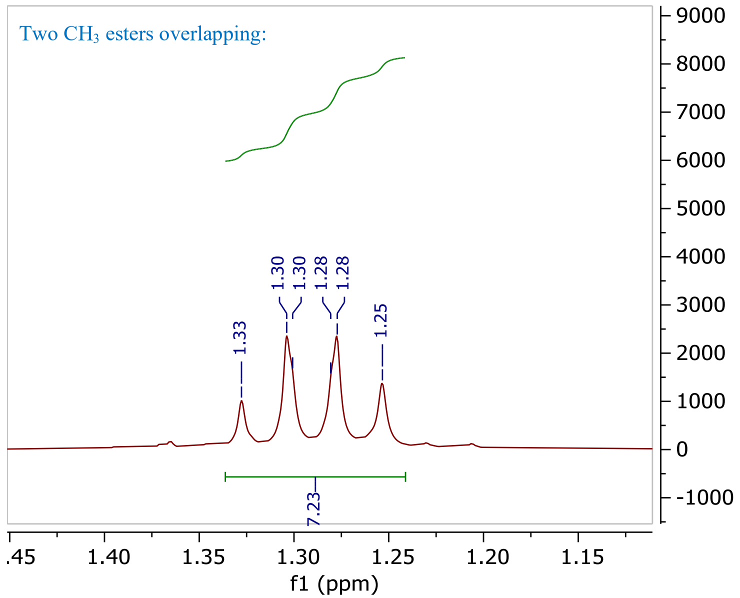

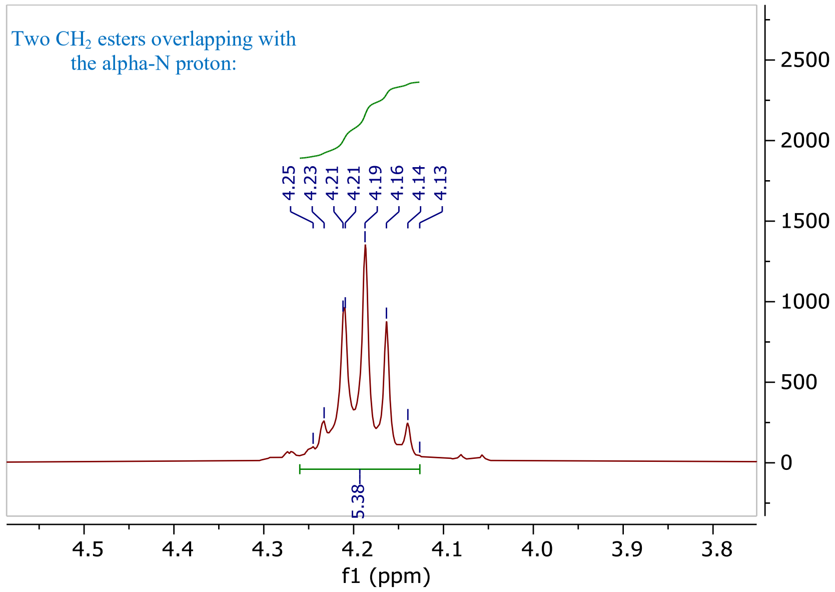

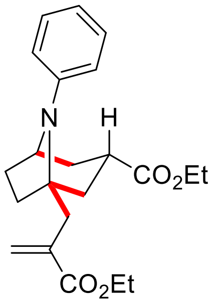

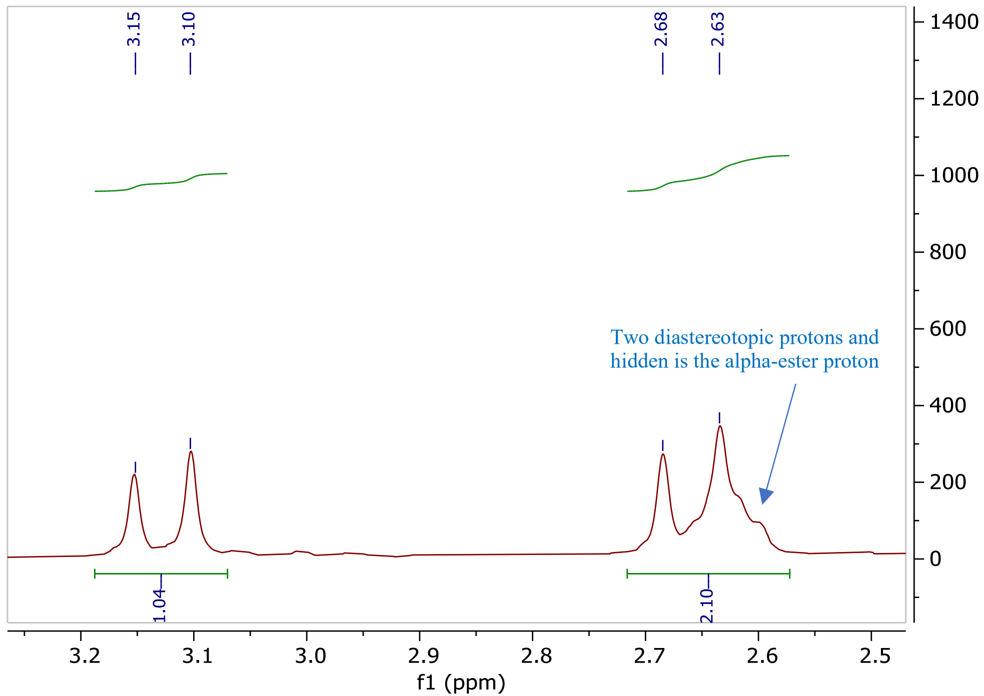

Ethyl 1-(2-ethoxycarbonylallyl)-8-phenyl-8-azabicyclo[3.2.1]octane-3-carboxylate_mix of **dia 1:4**

^{1}H-NMR (300 MHz, $CDCl_3$)

GA_211886.10.fid
ECO-3-091 F2
Proton_ns16 CDCl3 /opt service 60

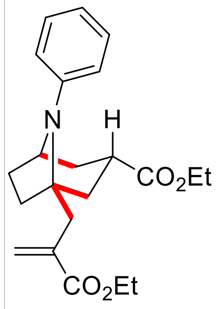

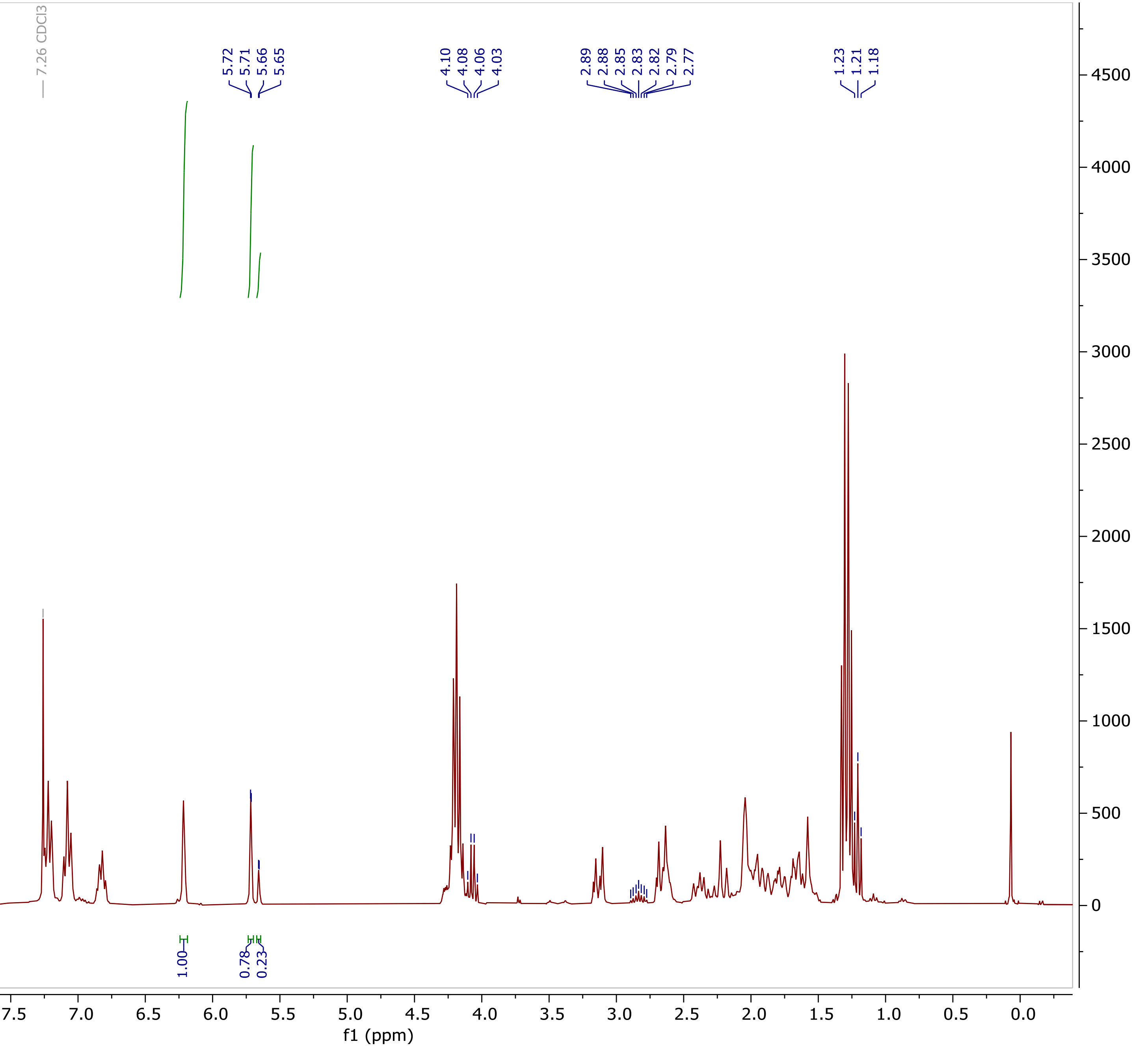

Ethyl 1-(2-ethoxycarbonylallyl)-8-phenyl-8-azabicyclo[3.2.1]octane-3-carboxylate_major dia

^{13}C-NMR (101 MHz, $CDCl_3$)

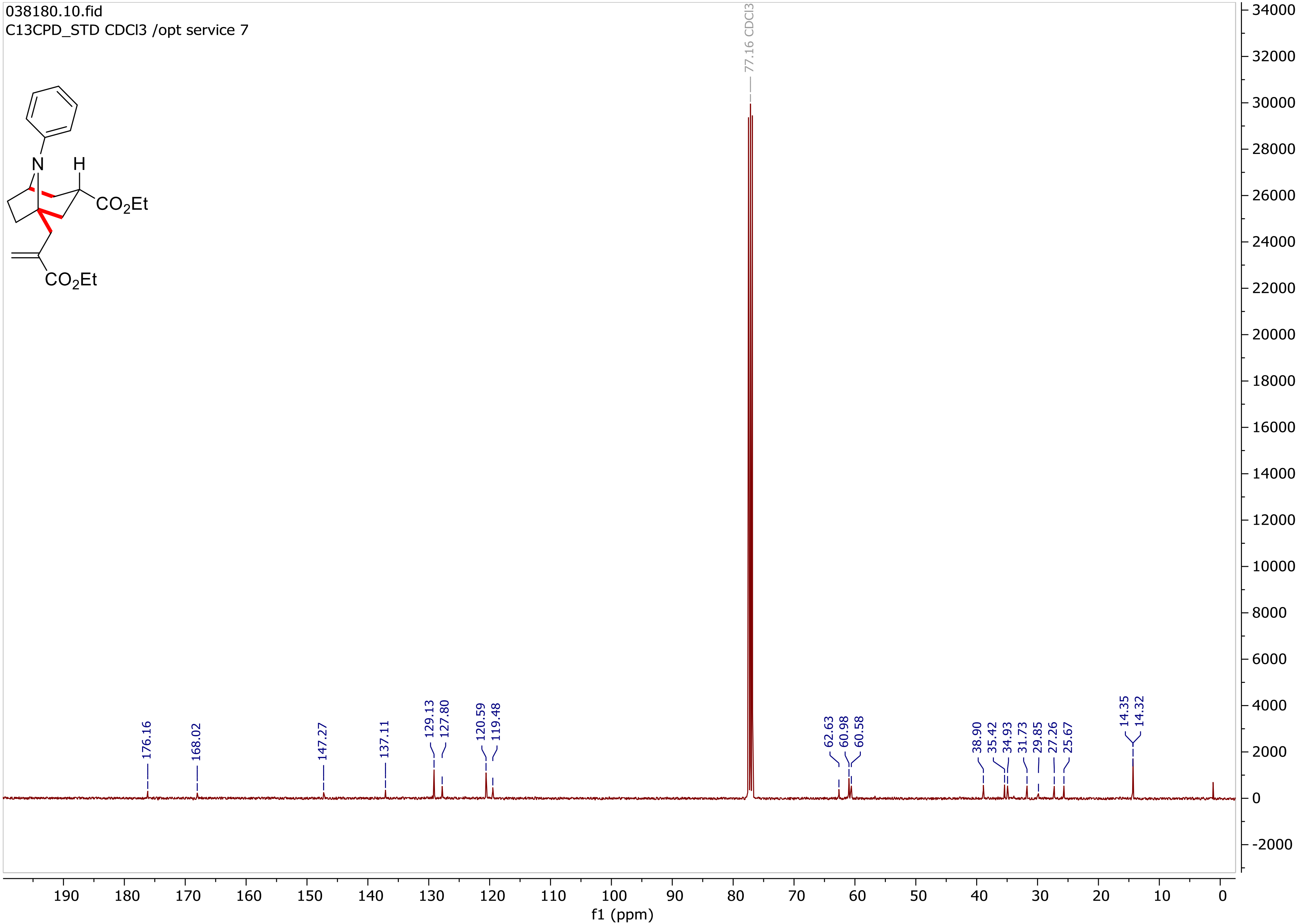

Ethyl 1-(2-ethoxycarbonylallyl)-8-phenyl-8-azabicyclo[3.2.1]octane-3-carboxylate_major dia

^{13}C-NMR (101 MHz, $CDCl_3$)

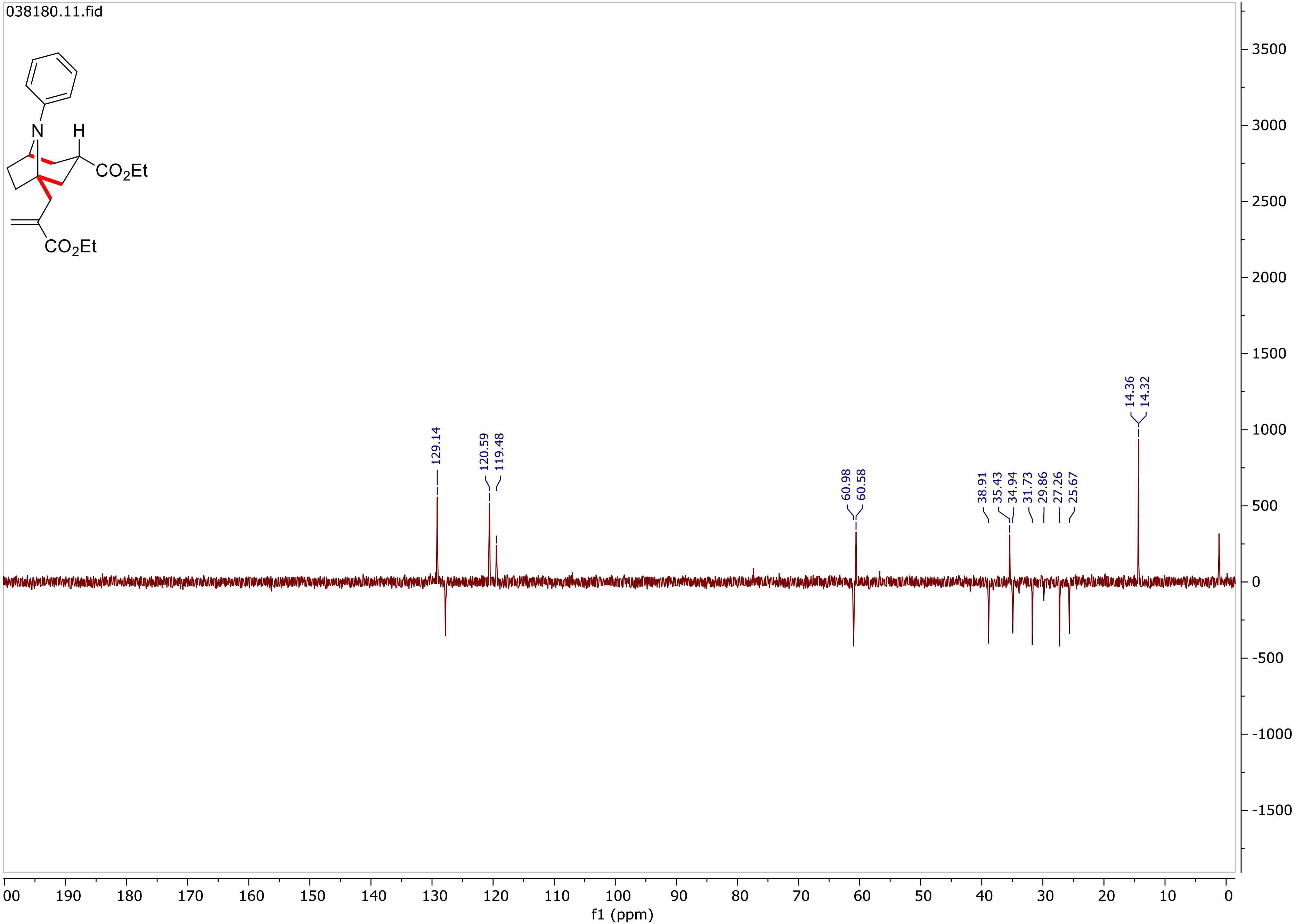

Ethyl 1-(2-ethoxycarbonylallyl)-8-phenyl-8-azabicyclo[3.2.1]octane-3-carboxylate_major dia

FT-IR, ATR-diamond

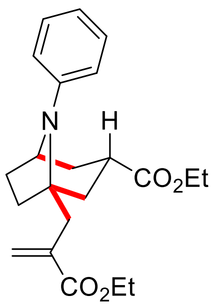

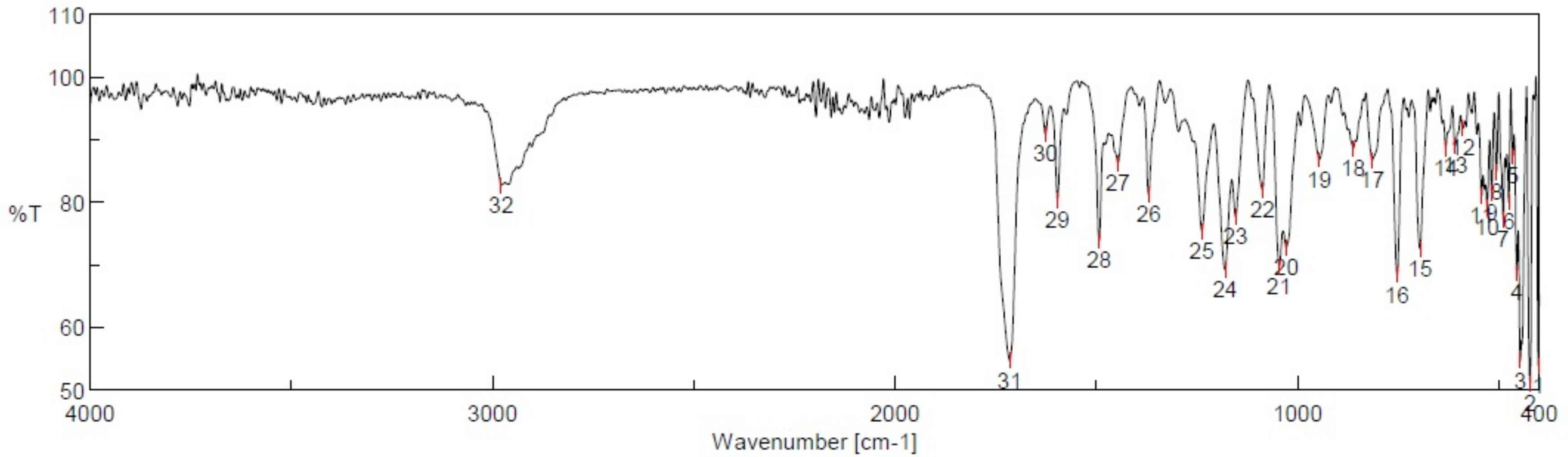

[Result of Peak Picking]

No.	Position	Intensity	No.	Position	Intensity	No.	Position	Intensity
1	402.085	54	2	422.334	51	3	446.44	55
4	456.082	69	5	465.725	87	6	475.367	80
7	489.831	77	8	506.223	85	9	517.793	82
10	529.364	79	11	542.863	81	12	590.111	92
13	608.431	89	14	632.537	89	15	696.177	73
16	753.066	68	17	814.777	87	18	862.025	89
19	945.913	87	20	1027.87	73	21	1046.19	70
22	1087.66	82	23	1154.19	78	24	1180.22	69
25	1238.08	75	26	1370.18	81	27	1446.35	86
28	1493.6	74	29	1596.77	80	30	1626.66	91
31	1715.37	55	32	2978.52	83			

Ethyl 3-(1-phenylpyrrolidin-2-yl)-2-[(1-phenylpyrrolidin-2-yl)methyl]propanoate

^{1}H-NMR (400 MHz, $CDCl_3$)

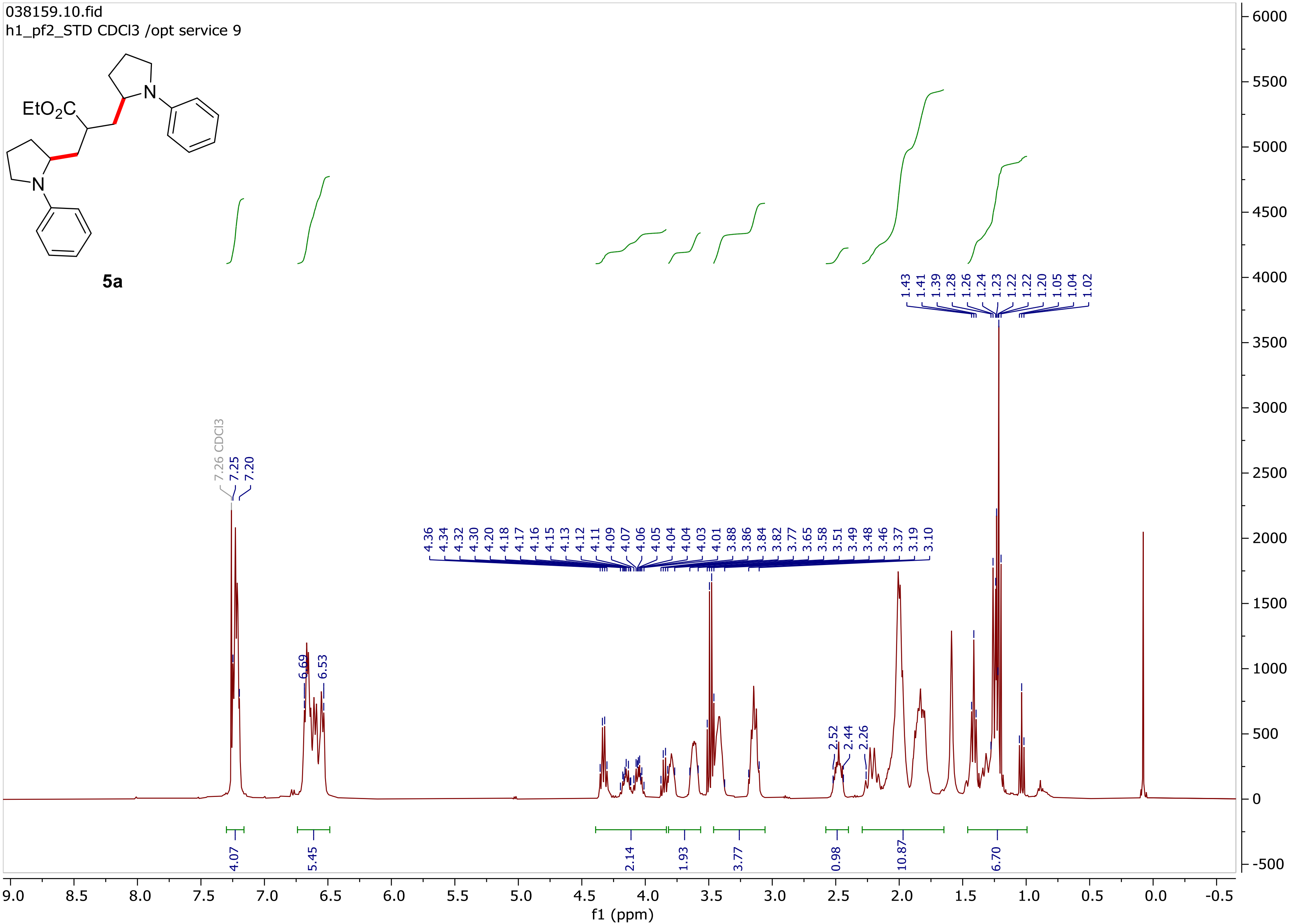

Ethyl 3-(1-phenylpyrrolidin-2-yl)-2-[(1-phenylpyrrolidin-2-yl)methyl]propanoate

^{13}C-NMR (101 MHz, $CDCl_3$)

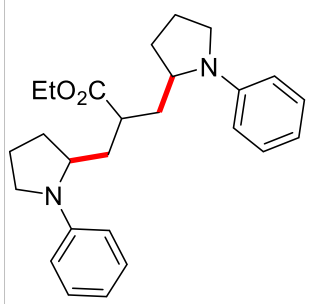

5a

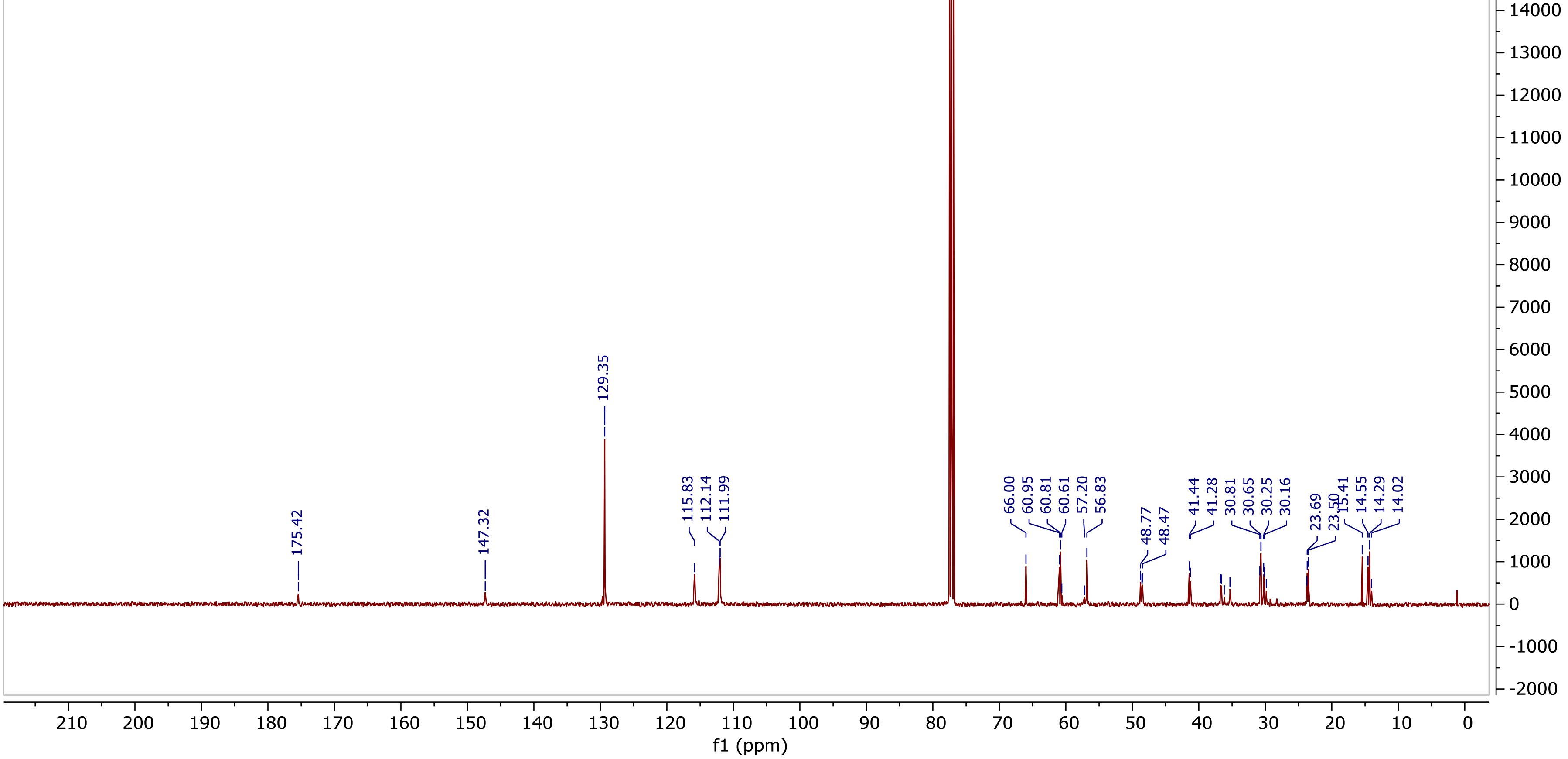

Ethyl 3-(1-phenylpyrrolidin-2-yl)-2-[(1-phenylpyrrolidin-2-yl)methyl]propanoate

^{13}C-NMR (101 MHz, $CDCl_3$)

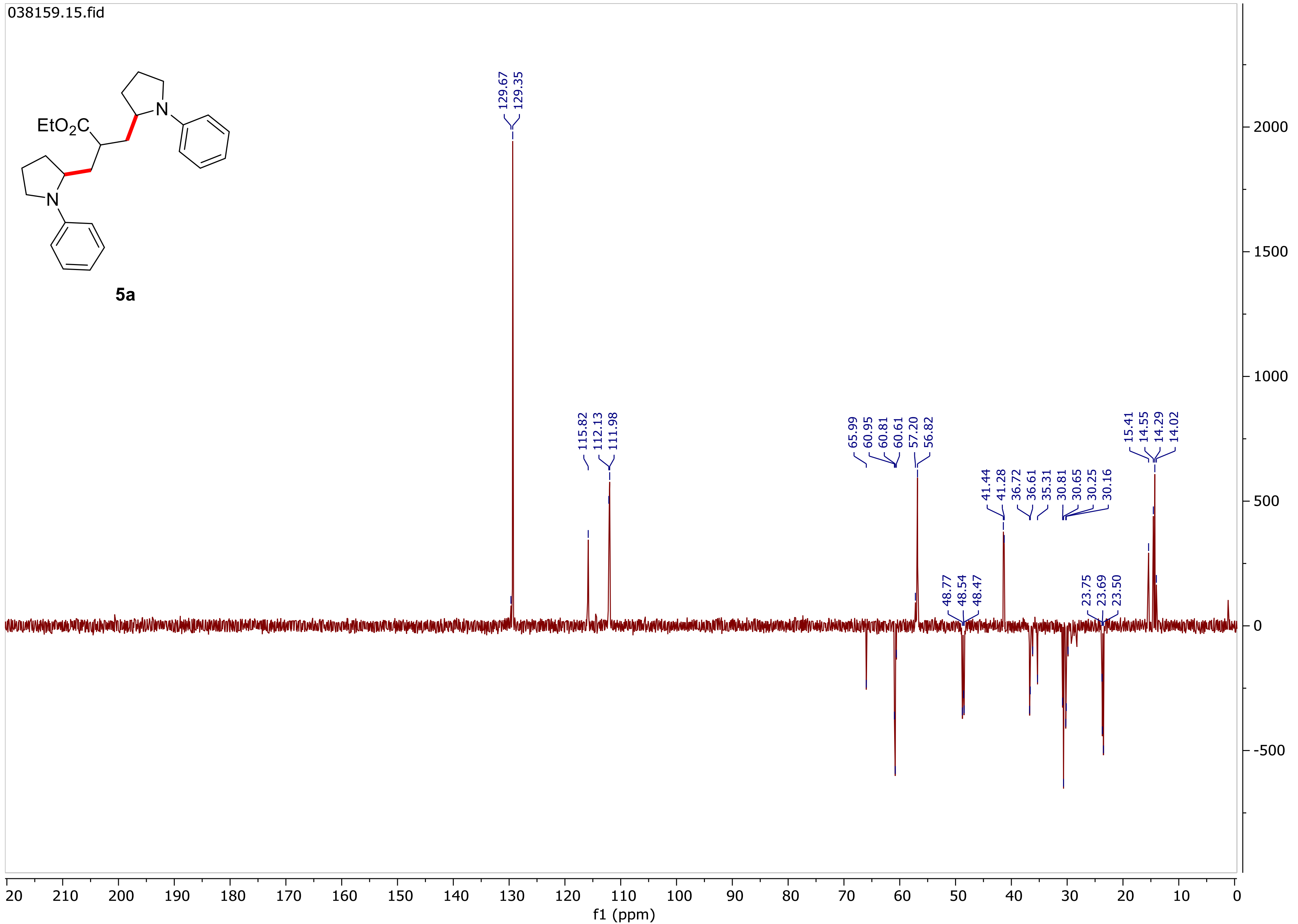

Ethyl 3-(1-phenylpyrrolidin-2-yl)-2-[(1-phenylpyrrolidin-2-yl)methyl]propanoate

^1H, ^1H-COSY NMR (400 MHz, $CDCl_3$)

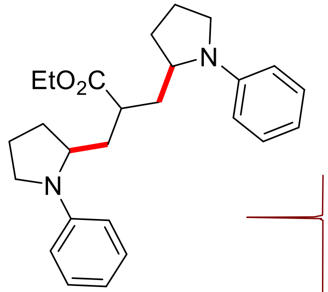

5a

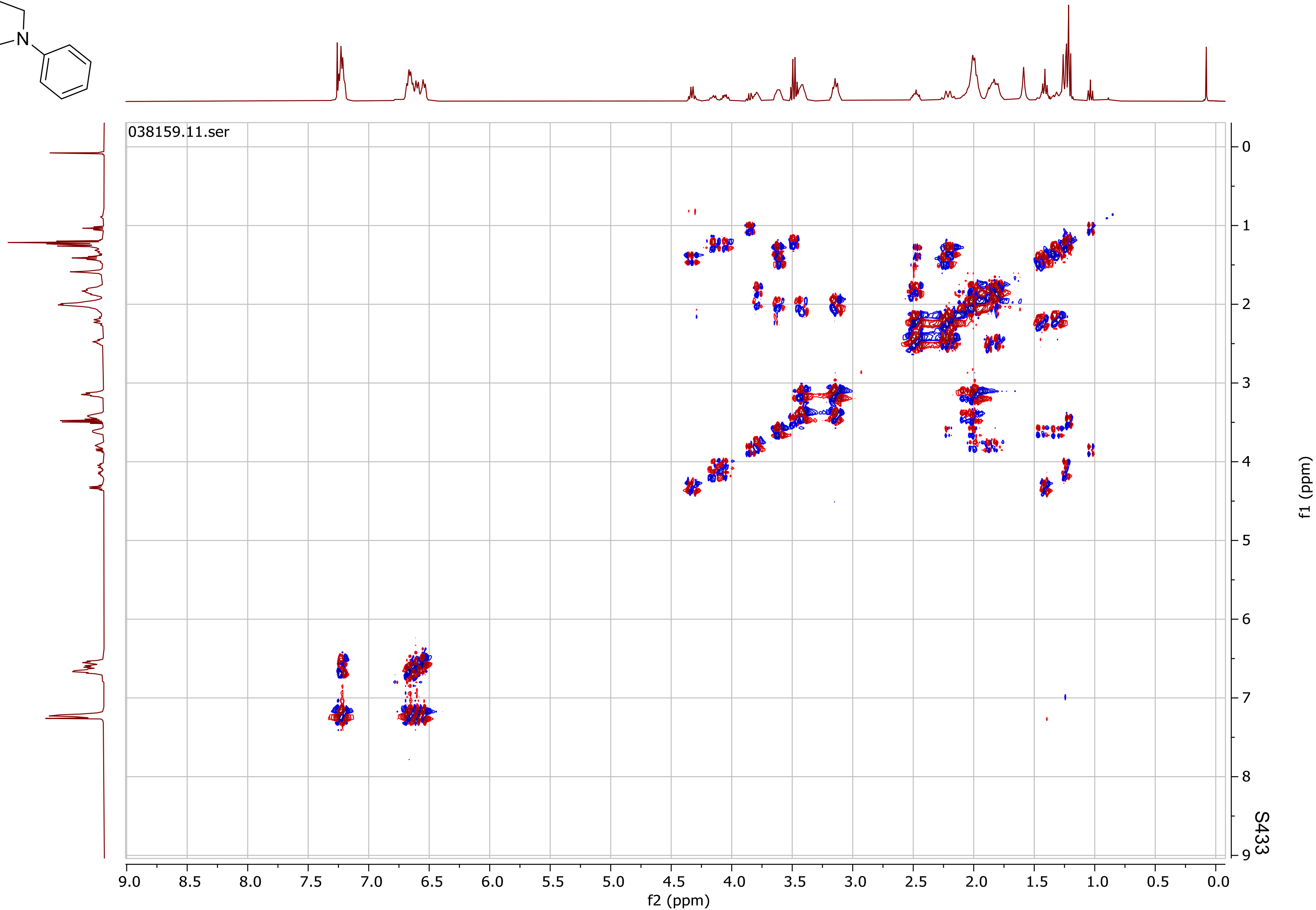

Ethyl 3-(1-phenylpyrrolidin-2-yl)-2-[(1-phenylpyrrolidin-2-yl)methyl]propanoate

^{1}H, ^{1}H-COSY NMR (400 MHz, $CDCl_3$)

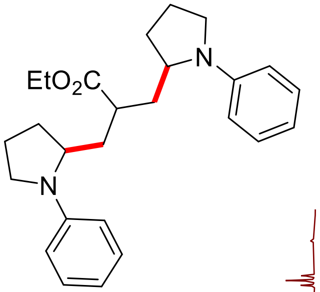

5a

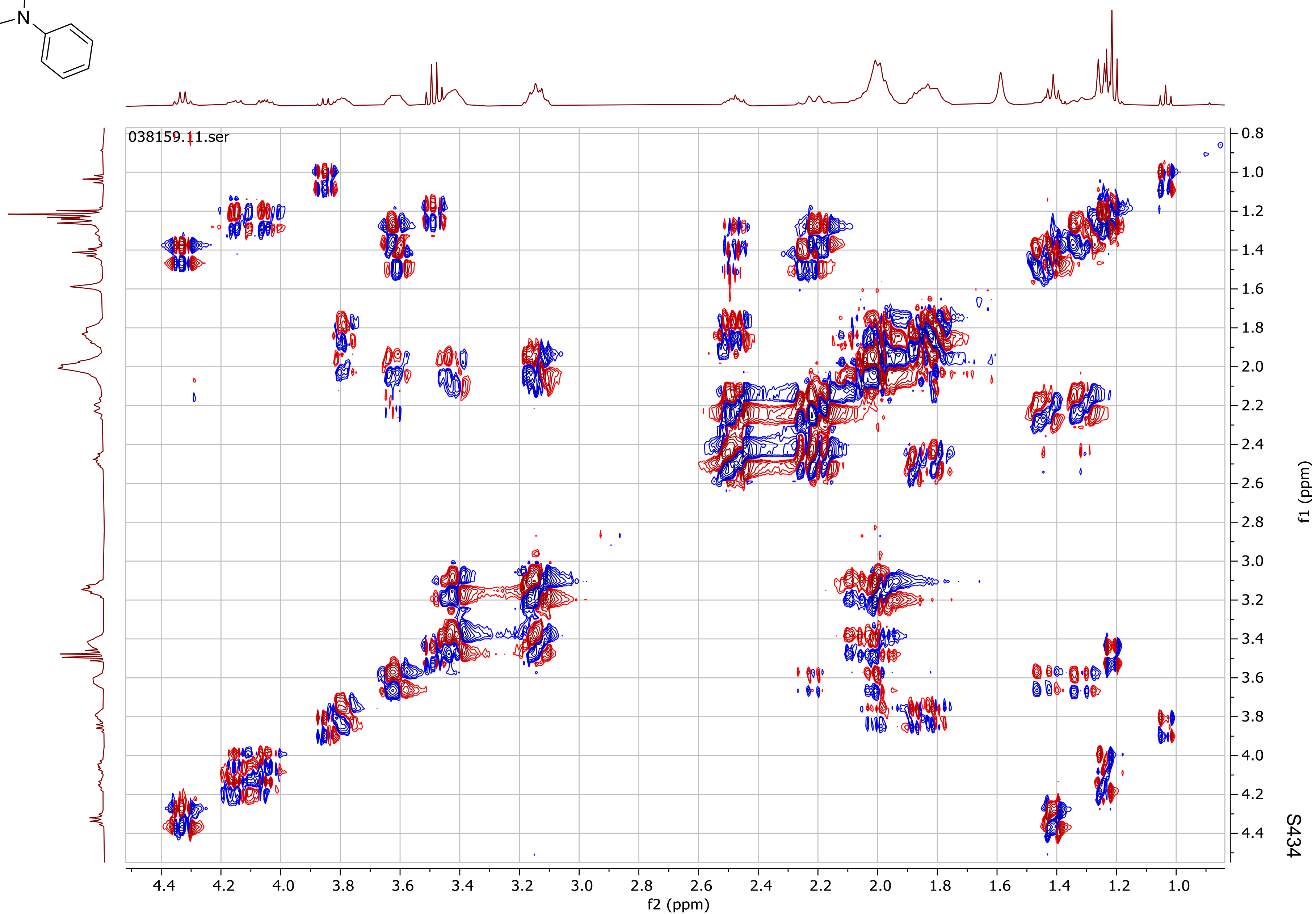

Ethyl 3-(1-phenylpyrrolidin-2-yl)-2-[(1-phenylpyrrolidin-2-yl)methyl]propanoate

^{1}H, ^{13}C-HSQC NMR (400 MHz, $CDCl_3$)

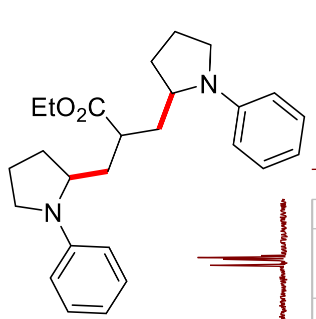

5a

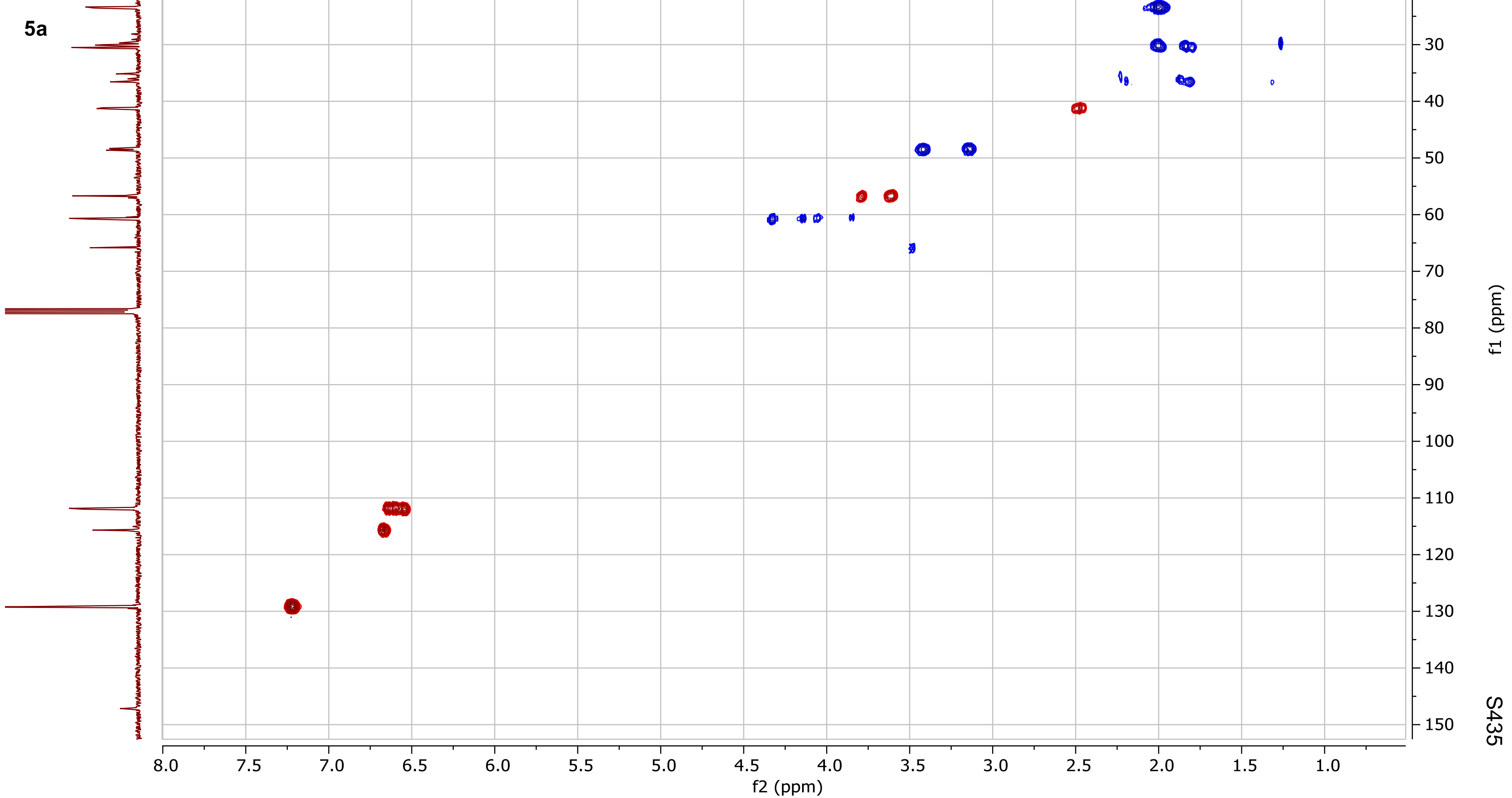

Ethyl 3-(1-phenylpyrrolidin-2-yl)-2-[(1-phenylpyrrolidin-2-yl)methyl]propanoate

^{1}H, ^{13}C-HSQC NMR (400 MHz, $CDCl_3$)

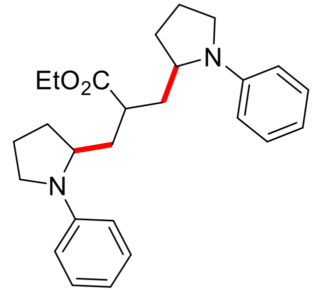

5a

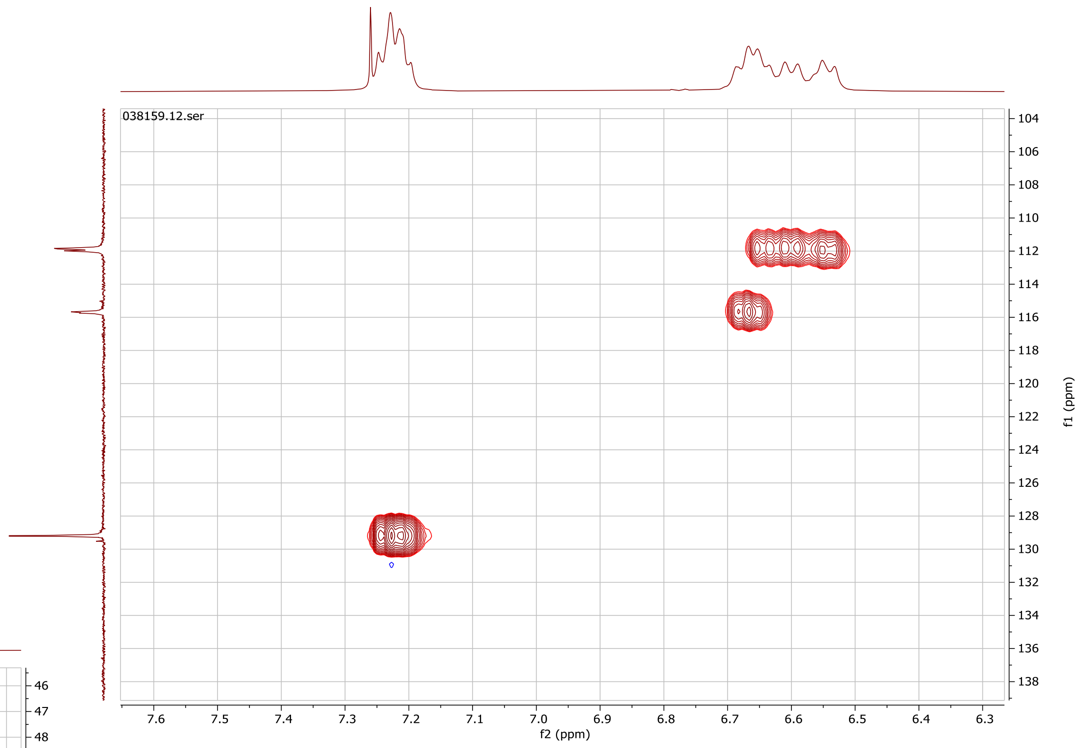

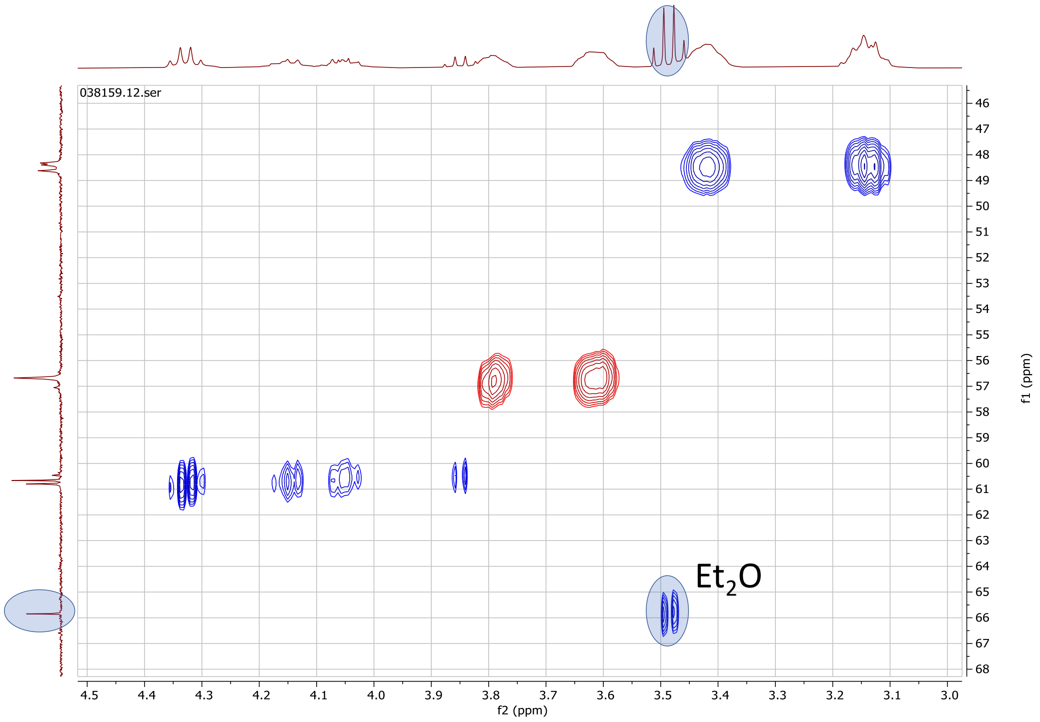

Ethyl 3-(1-phenylpyrrolidin-2-yl)-2-[(1-phenylpyrrolidin-2-yl)methyl]propanoate

^{1}H, ^{13}C-HSQC NMR (400 MHz, $CDCl_3$)

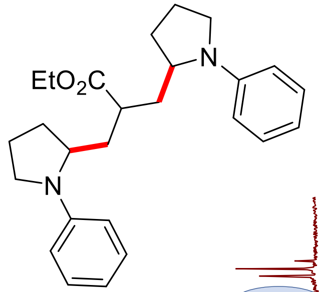

5a

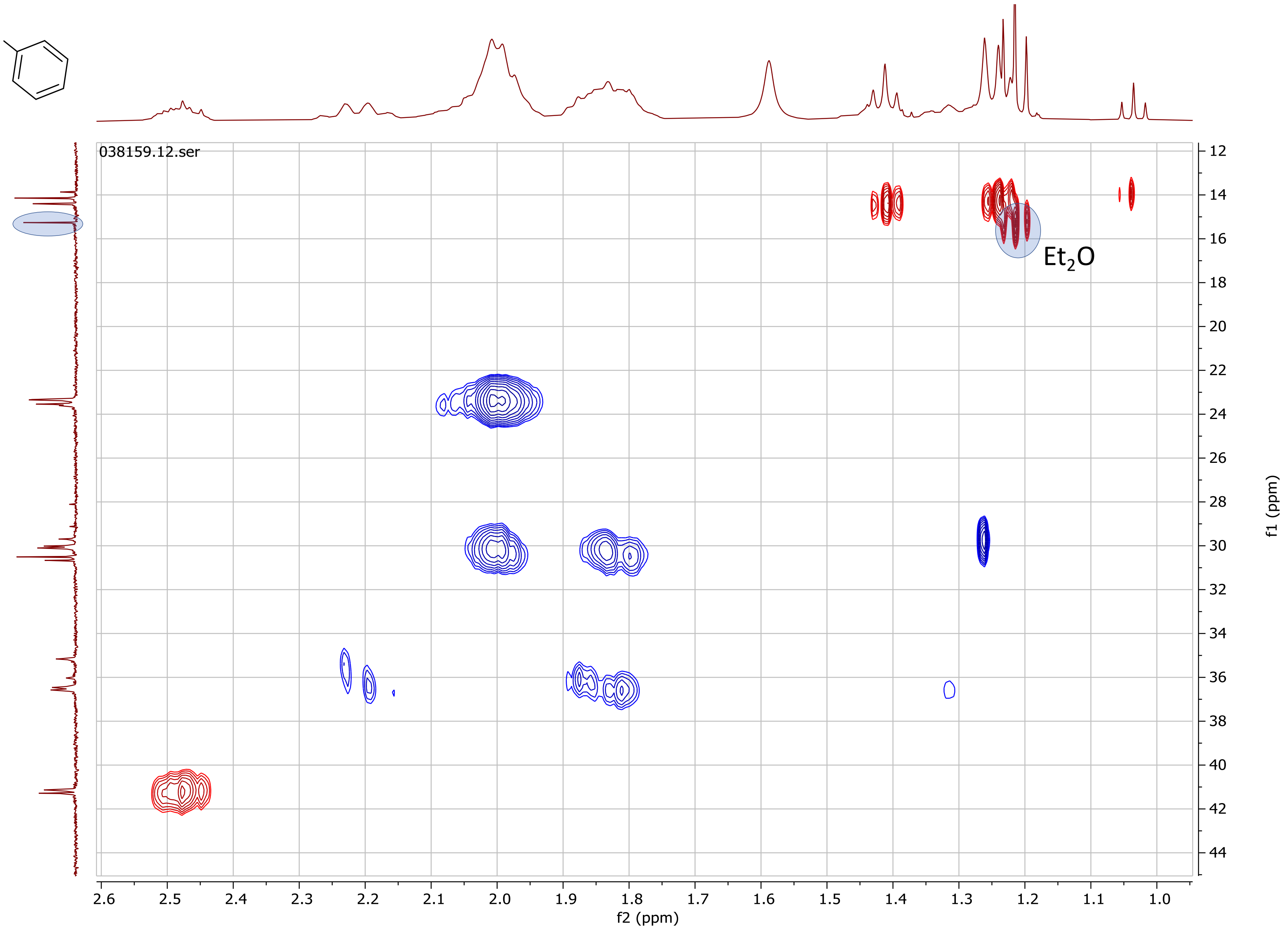

Ethyl 3-(1-phenylpyrrolidin-2-yl)-2-[(1-phenylpyrrolidin-2-yl)methyl]propanoate

^{1}H, ^{13}C-HMBC NMR (400 MHz, $CDCl_3$)

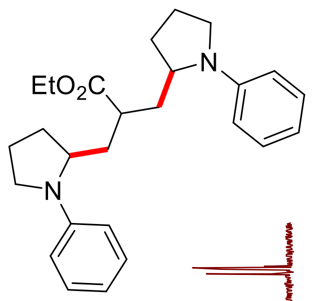

5a

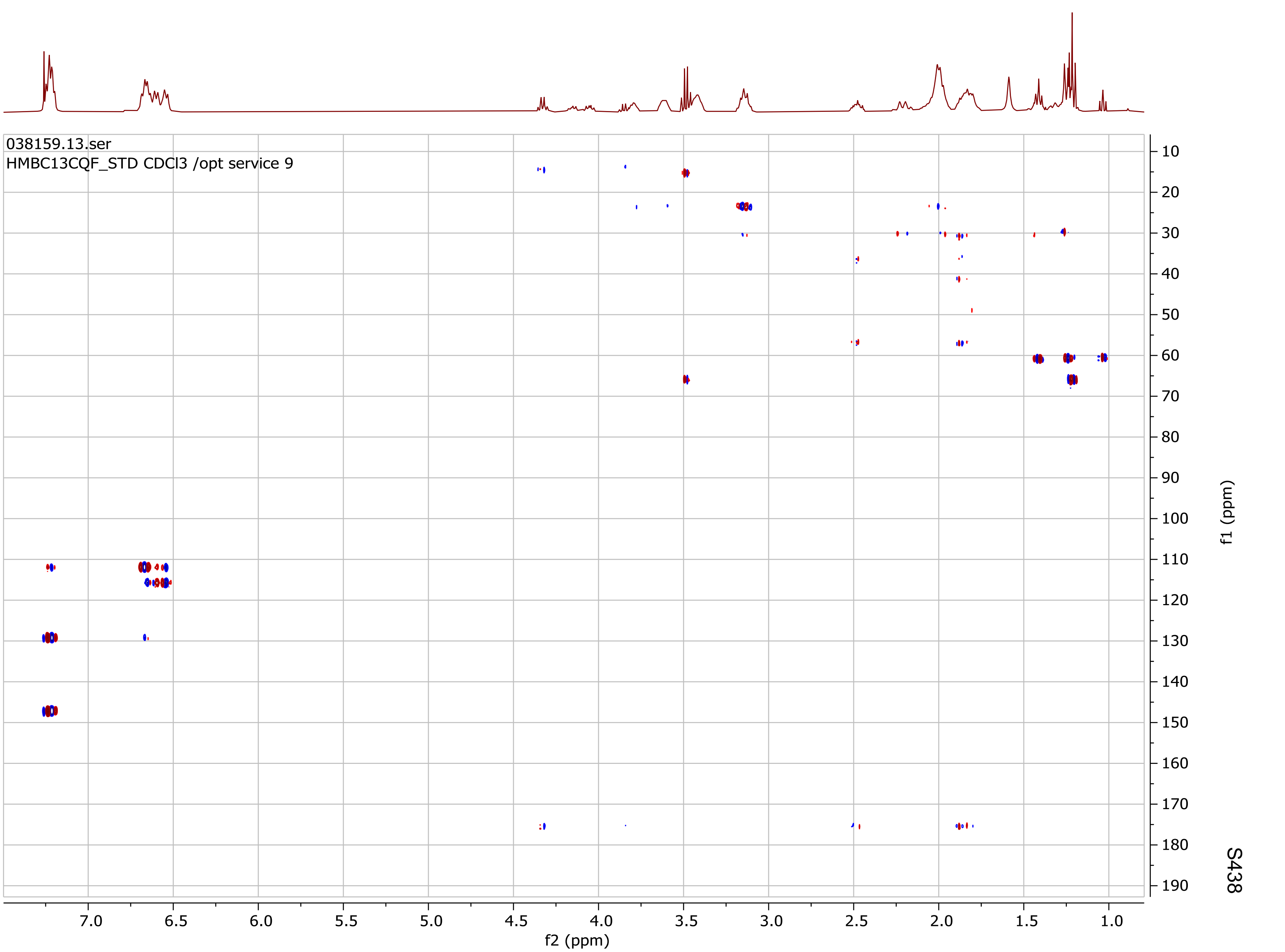

Ethyl 3-(1-phenylpyrrolidin-2-yl)-2-[(1-phenylpyrrolidin-2-yl)methyl]propanoate

^{1}H, ^{13}C-HMBC NMR (400 MHz, $CDCl_3$)

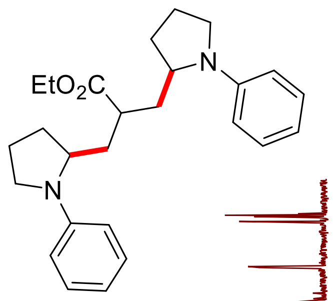

5a

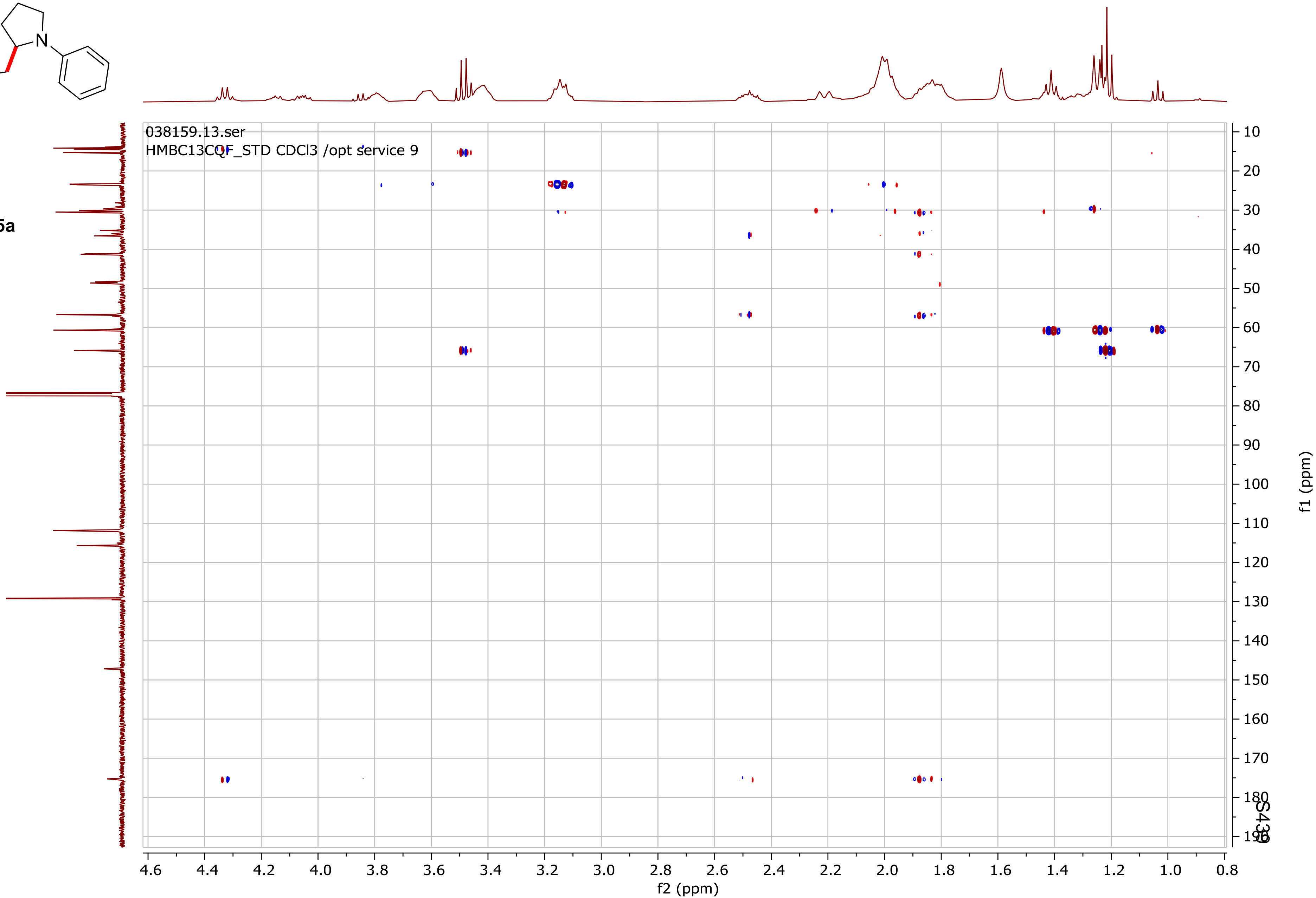

Ethyl 3-(1-phenylpyrrolidin-2-yl)-2-[(1-phenylpyrrolidin-2-yl)methyl]propanoate

^1H, ^{13}C-HMBC NMR (400 MHz, $CDCl_3$)

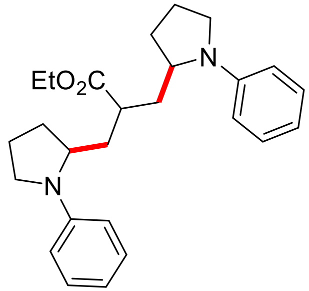

5a

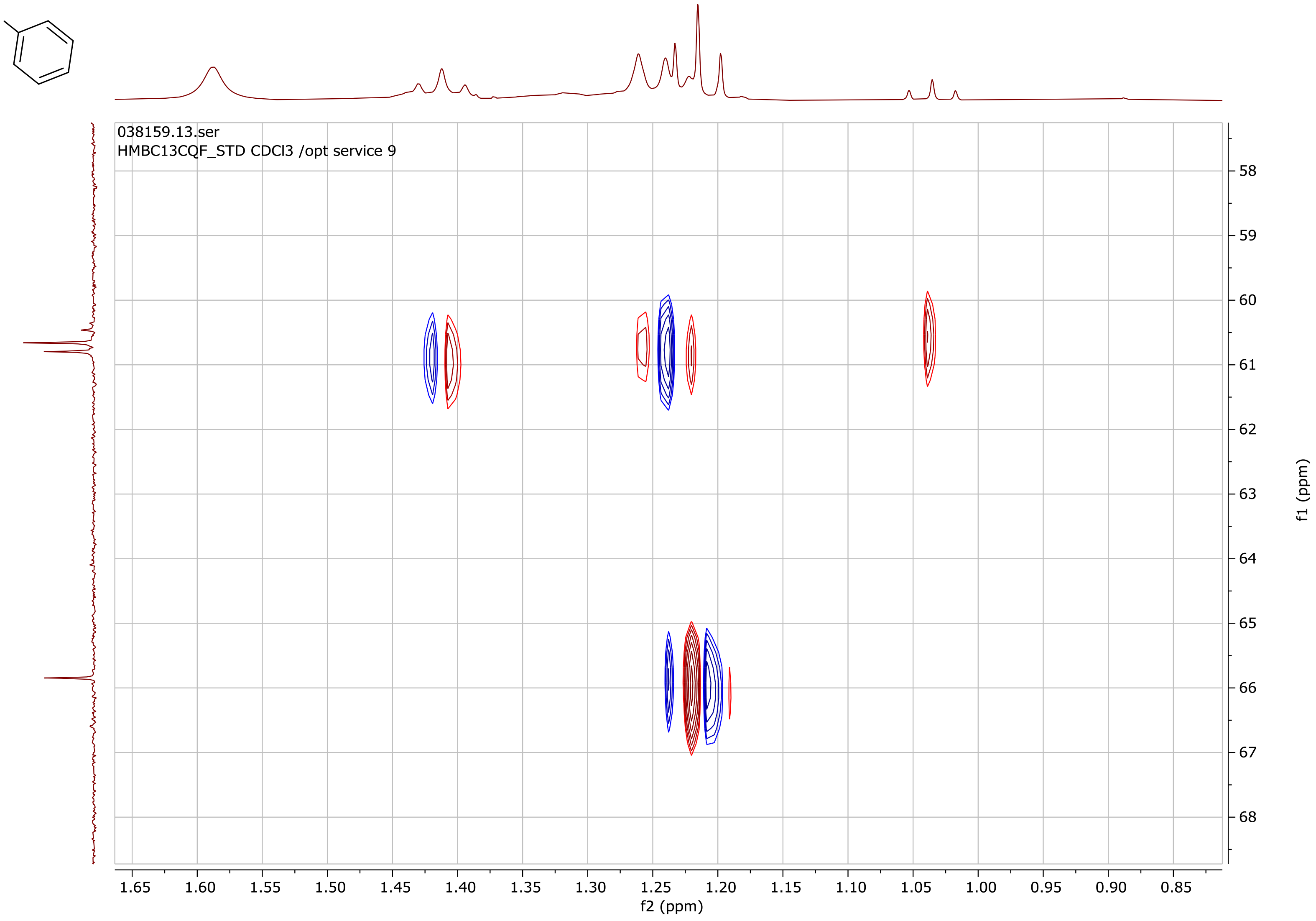

Ethyl 3-(1-phenylpyrrolidin-2-yl)-2-[(1-phenylpyrrolidin-2-yl)methyl]propanoate

FT-IR, ATR-diamond

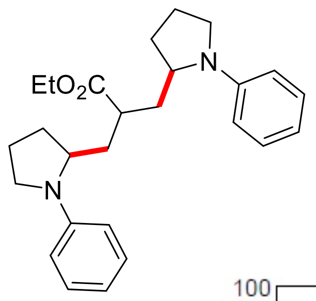

5a

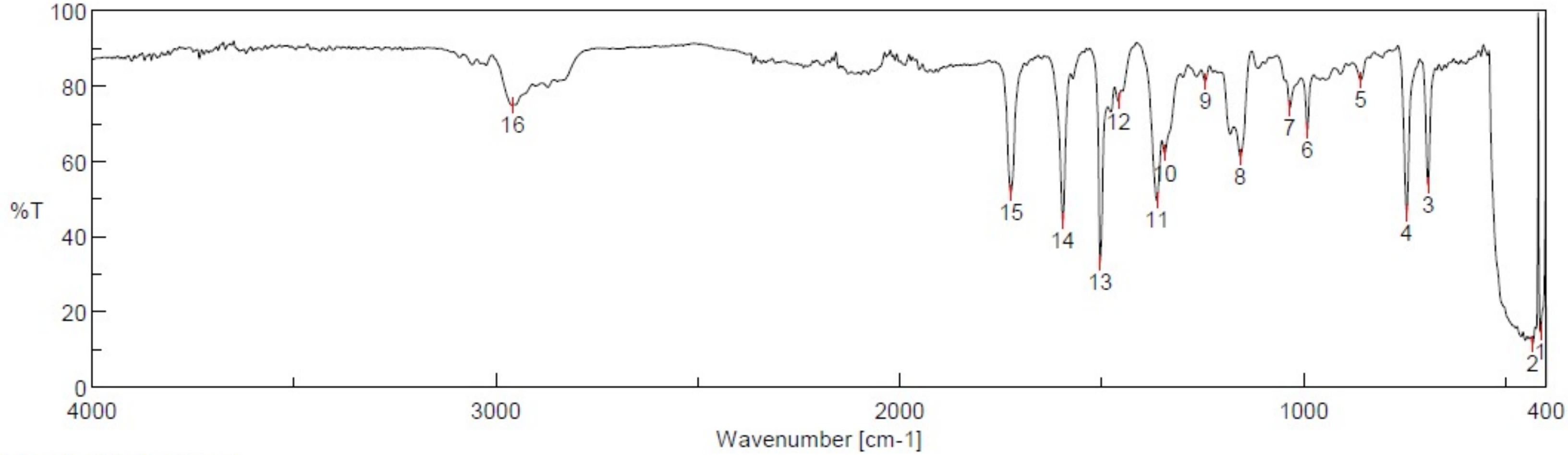

[Result of Peak Picking]

No.	Position	Intensity	No.	Position	Intensity	No.	Position	Intensity
1	413.656	15	2	432.941	12	3	692.32	54
4	745.352	46	5	859.132	82	6	991.232	68
7	1034.62	74	8	1157.08	61	9	1243.86	81
10	1344.14	62	11	1363.43	50	12	1458.89	76
13	1504.2	33	14	1596.77	44	15	1725.01	52
16	2959.23	75						

Methyl 4-[2,6-bis[(Z)-2-ethoxycarbonylbut-2-enyl]-1-piperidyl]benzoate

^{1}H-NMR (300 MHz, $CDCl_3$)

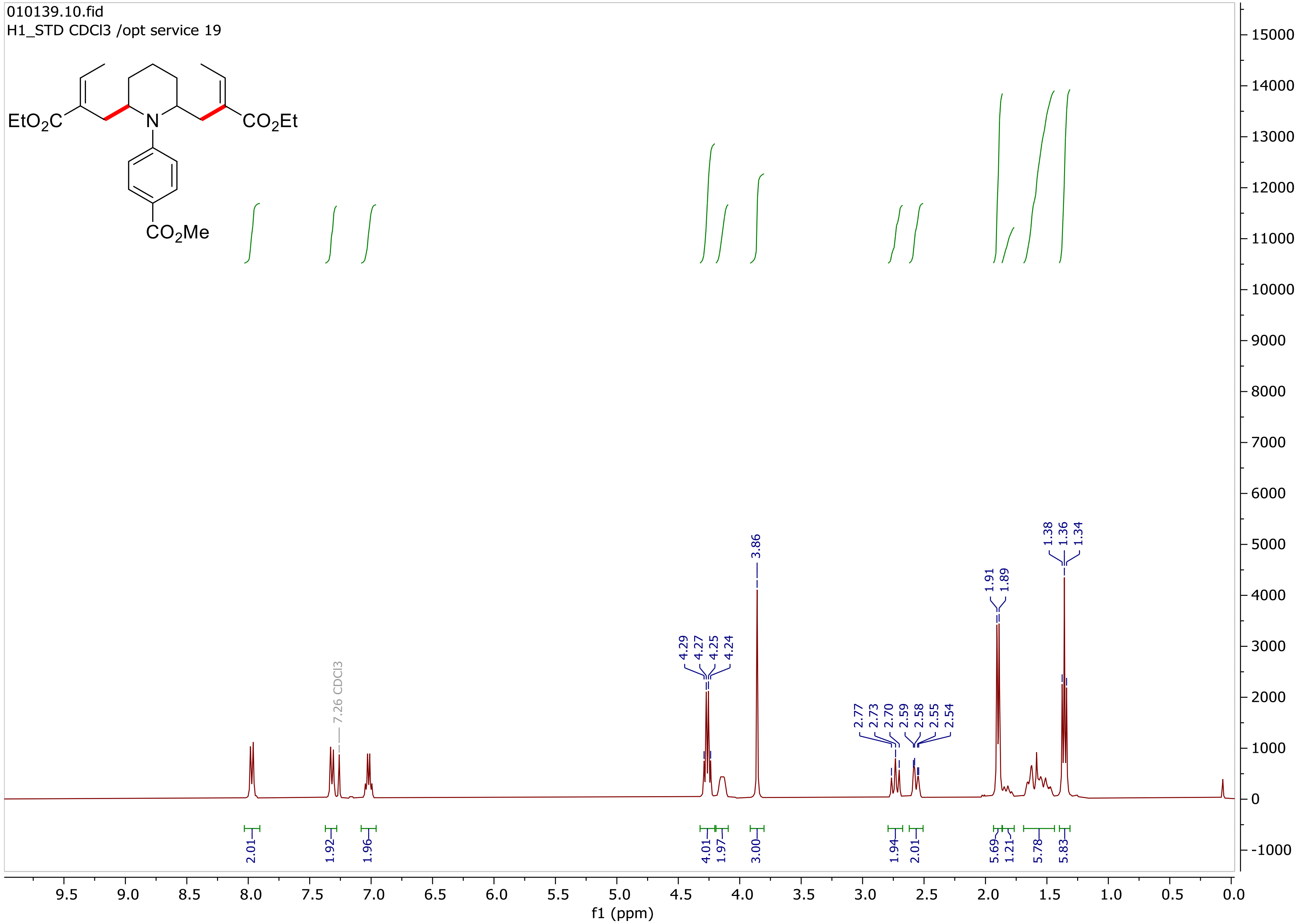

Methyl 4-[2,6-bis[(Z)-2-ethoxycarbonylbut-2-enyl]-1-piperidyl]benzoate

^{1}H-NMR (300 MHz, $CDCl_3$)

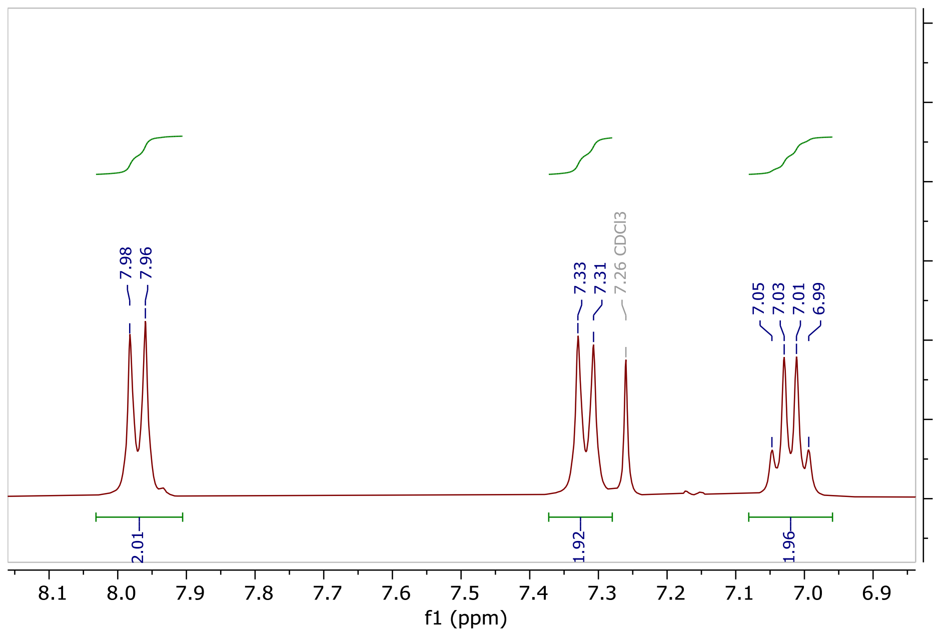

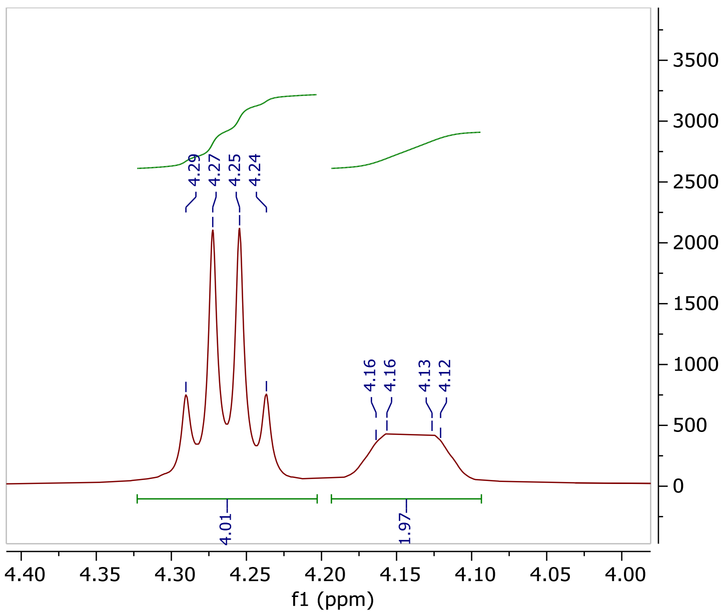

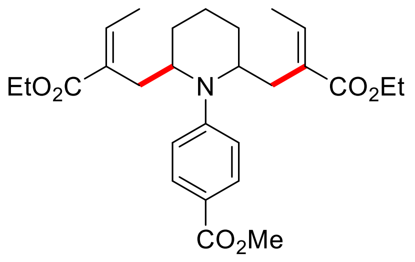

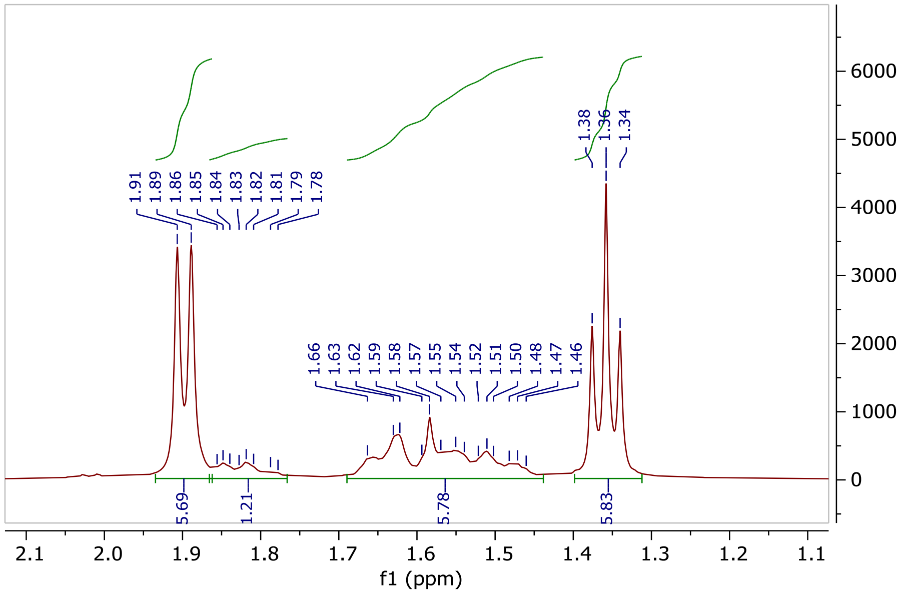

Methyl 4-[2,6-bis[(Z)-2-ethoxycarbonylbut-2-enyl]-1-piperidyl]benzoate

^{13}C-NMR (101 MHz, $CDCl_3$)

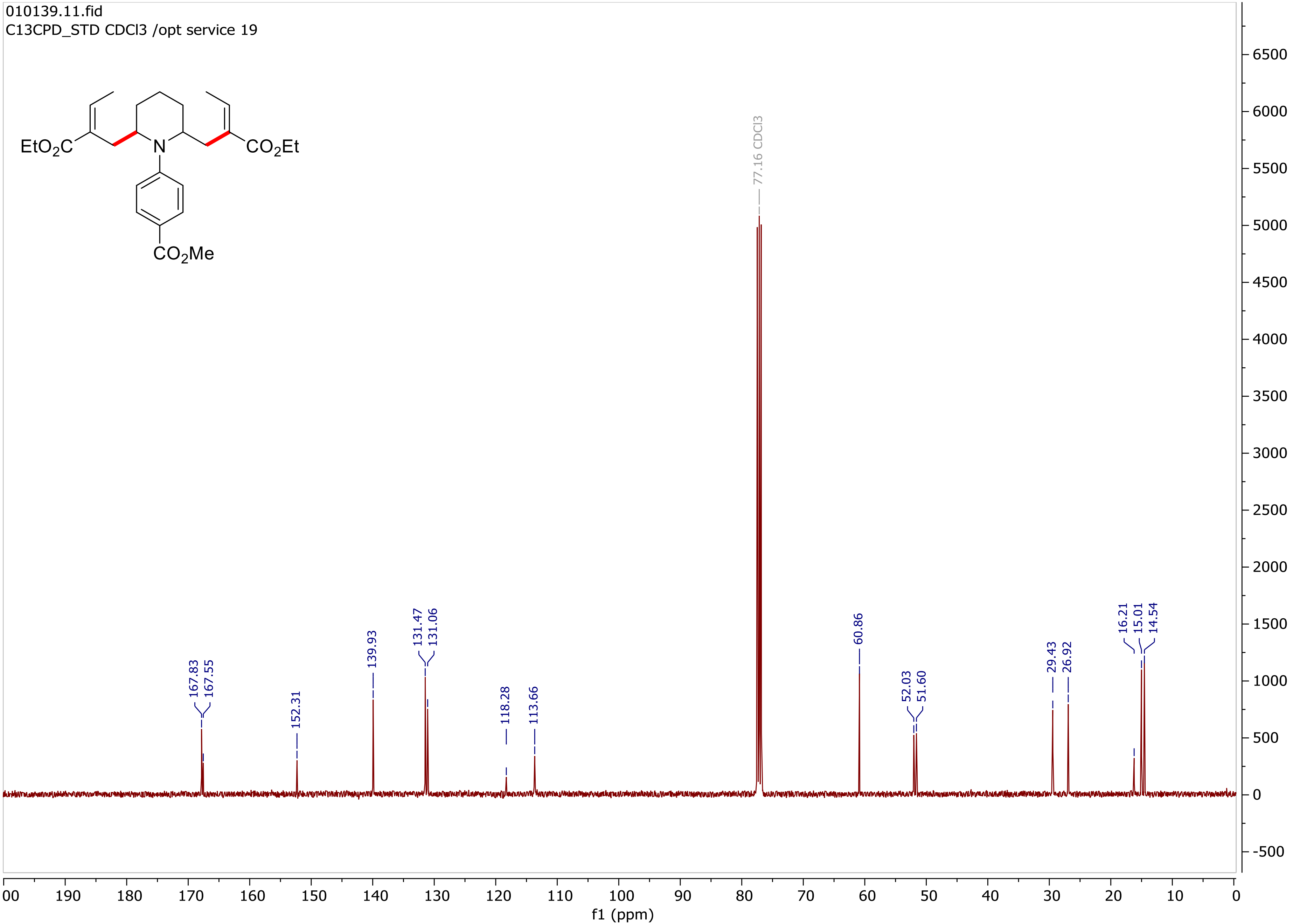

Methyl 4-[2,6-bis[(Z)-2-ethoxycarbonylbut-2-enyl]-1-piperidyl]benzoate

^{13}C-NMR (101 MHz, $CDCl_3$)

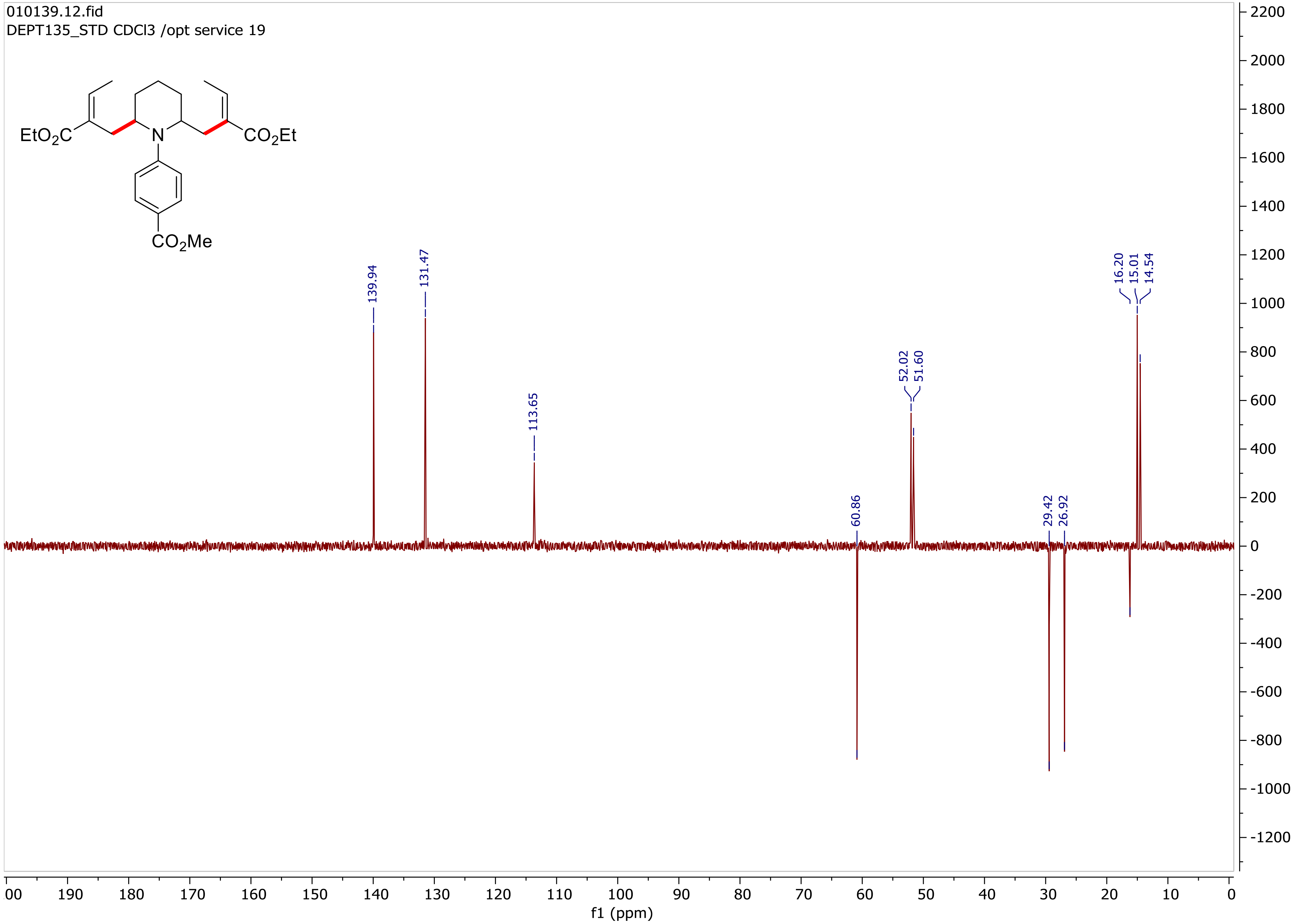

Methyl 4-[2,6-bis[(Z)-2-ethoxycarbonylbut-2-enyl]-1-piperidyl]benzoate

^1H, ^1H-COSY NMR (300 MHz, $CDCl_3$)

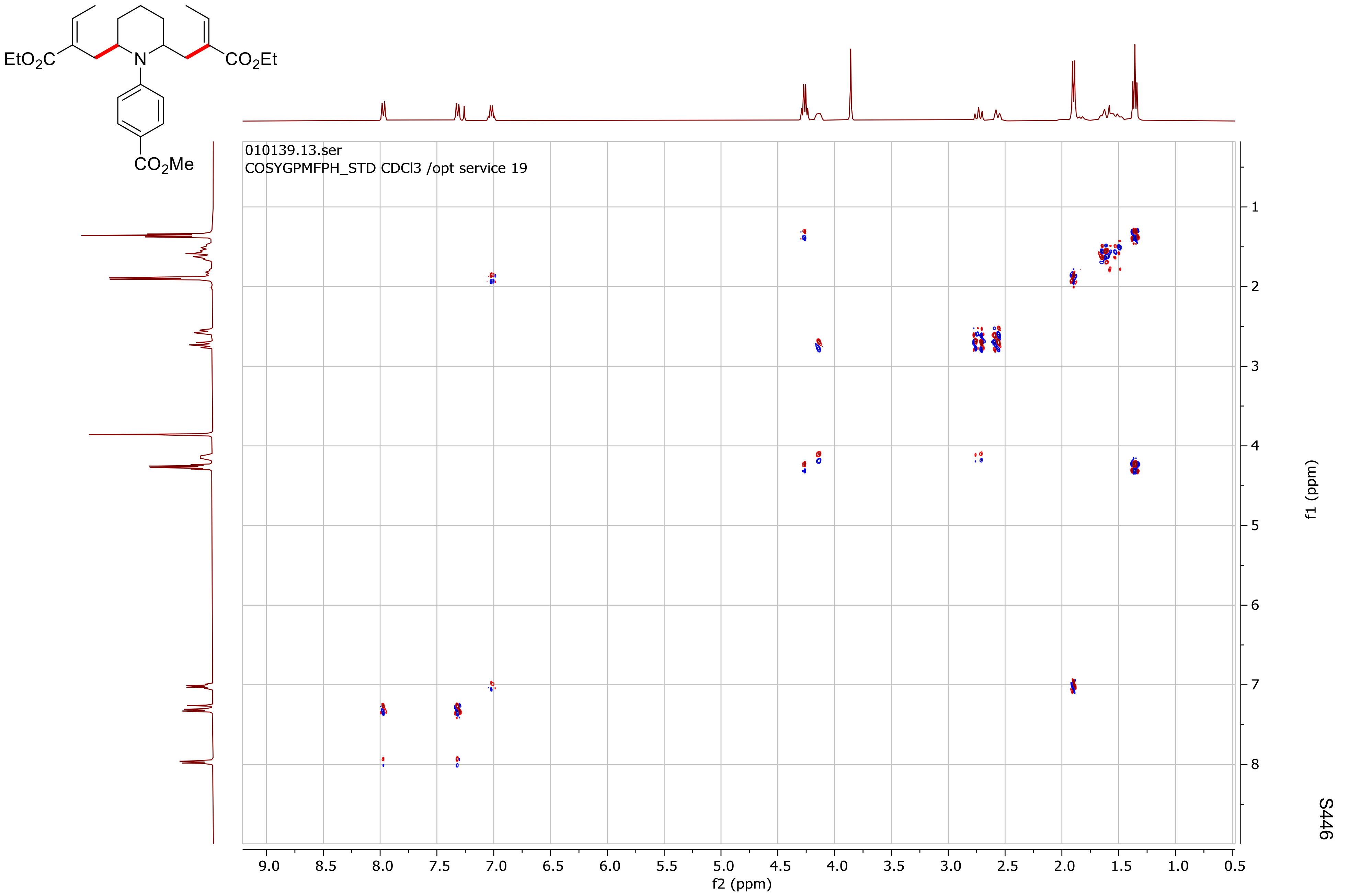

Methyl 4-[2,6-bis[(Z)-2-ethoxycarbonylbut-2-enyl]-1-piperidyl]benzoate

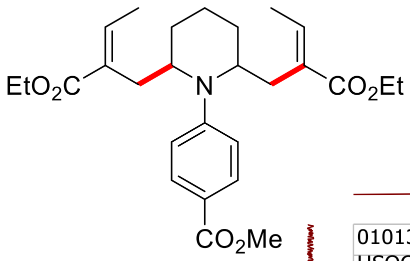

^{1}H, ^{13}C-HSQC NMR (300 MHz, $CDCl_3$)

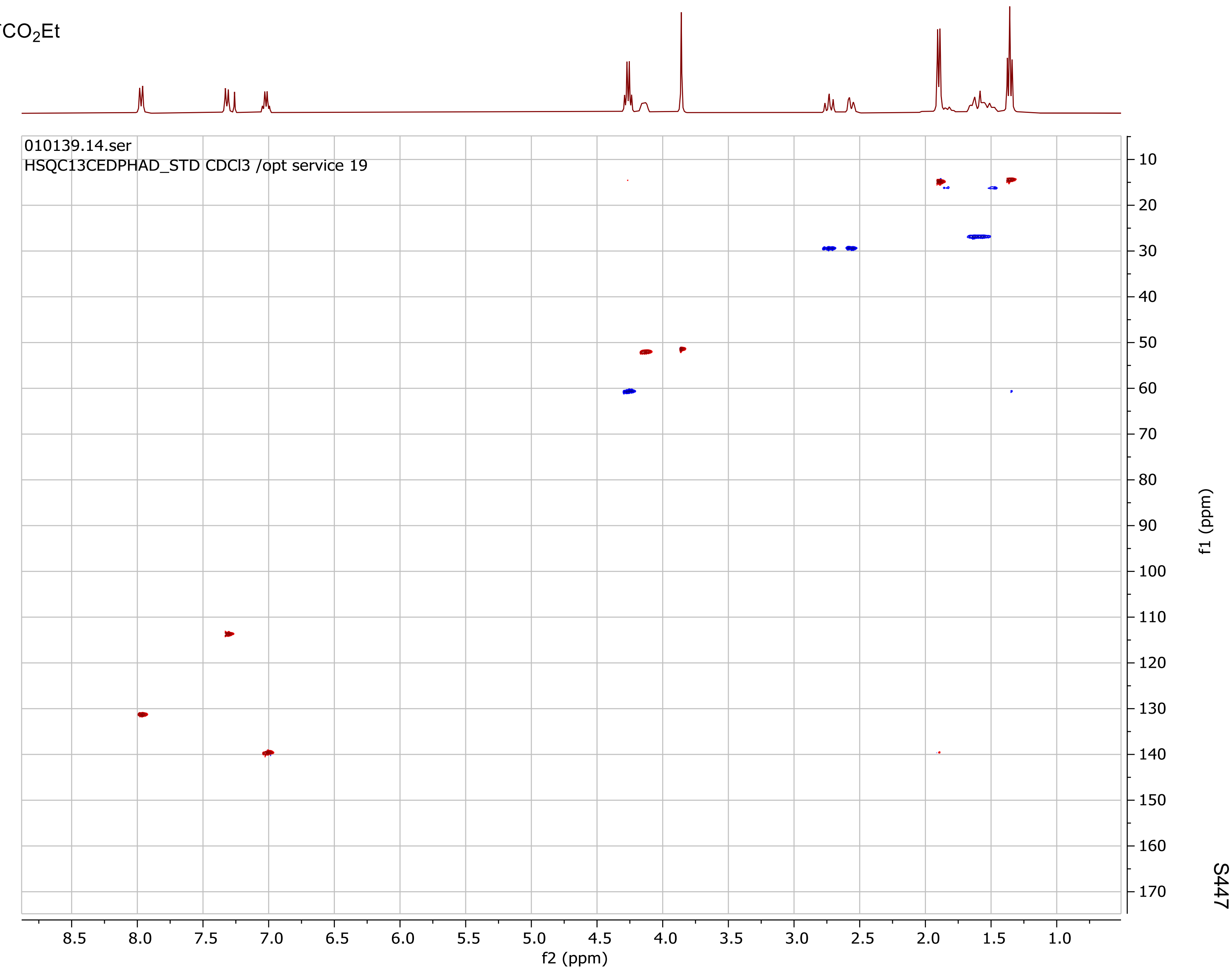

Methyl 4-[2,6-bis[(Z)-2-ethoxycarbonylbut-2-enyl]-1-piperidyl]benzoate

^{1}H, ^{13}C-HMBC NMR (300 MHz, $CDCl_3$)

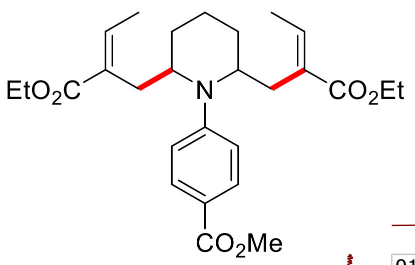

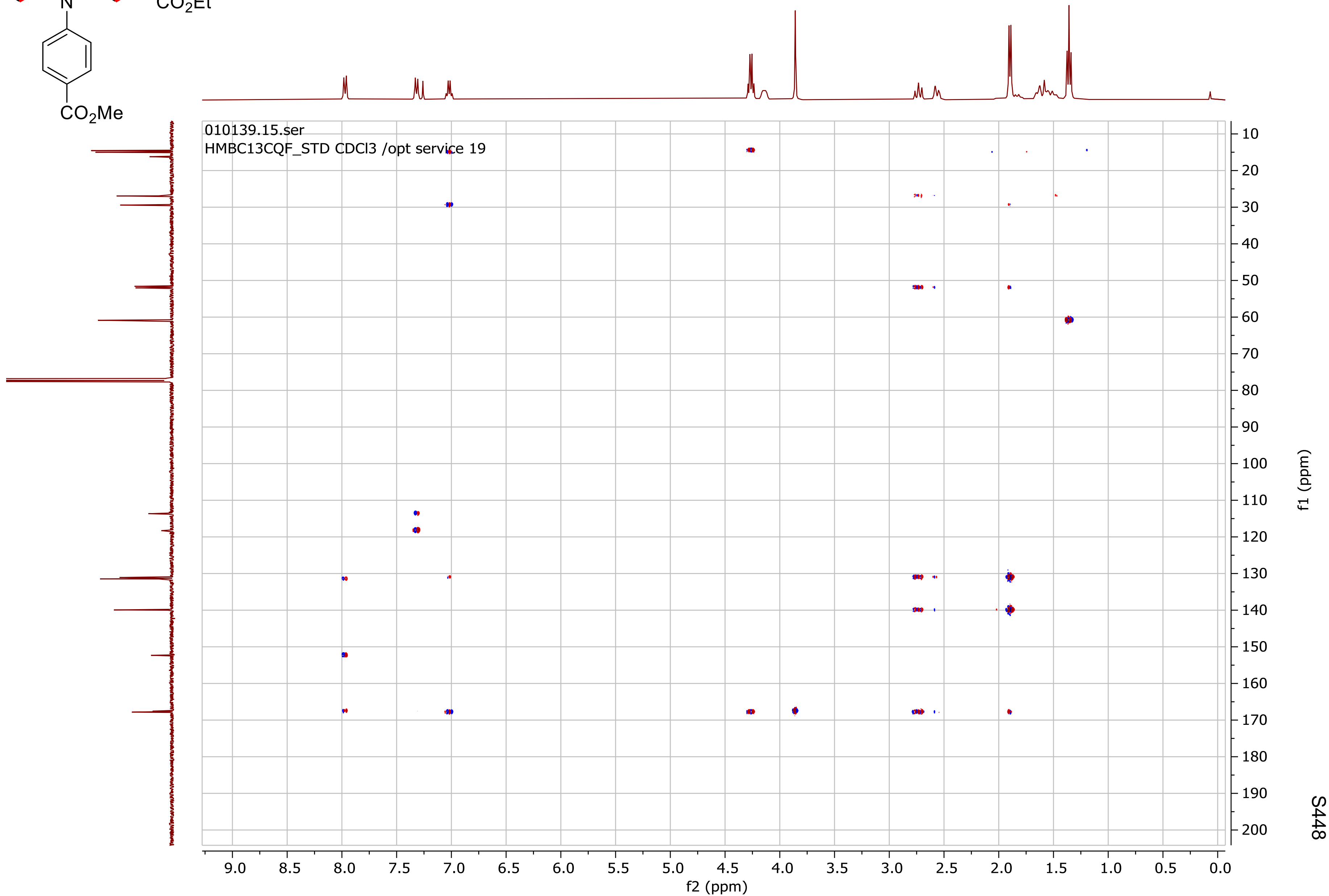

Methyl 4-[2,6-bis[(Z)-2-ethoxycarbonylbut-2-enyl]-1-piperidyl]benzoate

FT-IR, ATR-diamond

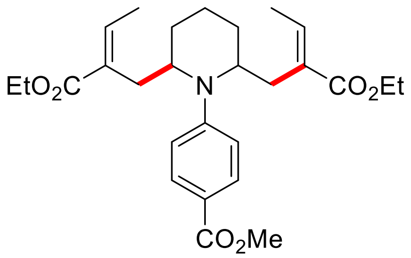

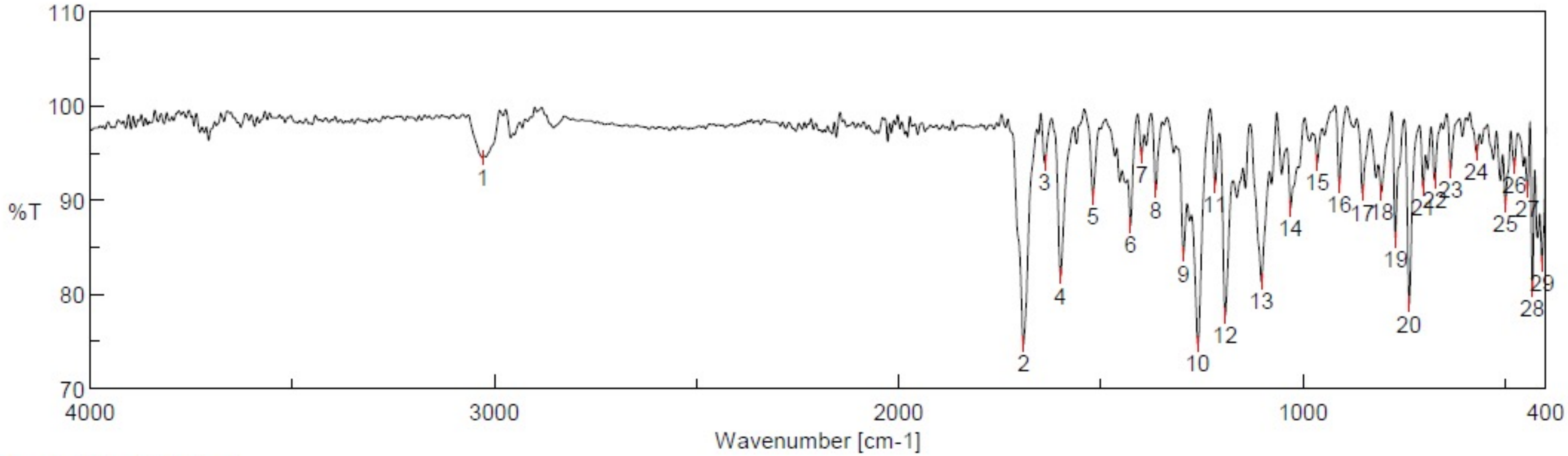

[Result of Peak Picking]

No.	Position	Intensity	No.	Position	Intensity	No.	Position	Intensity
1	3029.62	95	2	1691.27	75	3	1639.2	94
4	1599.66	82	5	1519.63	90	6	1427.07	87
7	1399.1	95	8	1364.39	91	9	1295.93	84
10	1260.25	75	11	1217.83	92	12	1192.76	78
13	1103.08	81	14	1030.77	89	15	965.198	94
16	910.236	92	17	853.347	91	18	806.099	91
19	771.387	86	20	737.639	79	21	702.926	91
22	673.999	92	23	634.466	93	24	570.826	95
25	499.473	89	26	477.296	94	27	444.512	91
28	432.941	81	29	408.835	83			
