# Supplementary material for: Tropane and related alkaloid skeletons via a radical [3+3]-annulation process
Source: Commun Chem. 2022 Apr 28;5:57. doi: 10.1038/s42004-022-00671-x (PMC9814087; doi:10.1038/s42004-022-00671-x)
Supplement: Supplementary file 2 — Description of Additional Supplementary Files [file 42004_2022_671_MOESM2_ESM.pdf]

Description of Additional Supplementary Files

File Name: Supplementary Data 1

Description: cif file 3a

File Name: Supplementary Data 2

Description: cif file 8

File Name: Supplementary Data 3

Description: cif file 9

File Name: Supplementary Data 4

Description: cif file 11

File Name: Supplementary Data 5

Description: DFT geometry-optimized coordinates
